# lobChIP: from cells to sequencing ready ChIP libraries in a single day

**DOI:** 10.1186/s13072-015-0017-5

**Published:** 2015-07-21

**Authors:** Ola Wallerman, Helena Nord, Madhusudhan Bysani, Lisa Borghini, Claes Wadelius

**Affiliations:** Science for Life Laboratory, Department of Immunology, Genetics and Pathology, BMC, Uppsala University, Box 815, 75108 Uppsala, Sweden; Department of Animal Breeding and Genetics, Swedish University of Agricultural Sciences, Uppsala, Sweden; Department of Clinical Sciences, CRC, Lund University Diabetes Center, Malmö, Sweden; Human Genetics, Genome Institute of Singapore, Singapore, Singapore

**Keywords:** ChIP-seq, Chromatin immunoprecipitation, Illumina, NGS, Library preparation

## Abstract

**Background:**

ChIP-seq is the method of choice for genome-wide studies of protein–DNA interactions. We describe a new method for ChIP-seq sample preparation, termed lobChIP, where the library reactions are performed on cross-linked ChIP fragments captured on beads.

**Results:**

The lobChIP method was found both to reduce time and cost and to simplify the processing of many samples in parallel. lobChIP has an early incorporation of barcoded sequencing adaptors that minimizes the risk of sample cross-contamination and can lead to reduced amount of adaptor dimers in the sequencing libraries, while allowing for direct decross-linking and amplification of the sample.

**Conclusions:**

With results for histone modifications and transcription factors, we show that lobChIP performs equal to or better than standard protocols and that it makes it possible to go from cells to sequencing ready libraries within a single day.

**Electronic supplementary material:**

The online version of this article (doi:10.1186/s13072-015-0017-5) contains supplementary material, which is available to authorized users.

## Background

The ability to decipher the regulatory information in the genome and epigenome is essential for understanding how transcription is controlled and how genetic variation affects disease states. Transcription factors (TF) bind DNA in a sequence-specific manner and can have either a repressive or activating function, and recent work shows that sequence variants affecting TF binding sites are the underlying mechanism of sequence-specific gene regulation [1] that in turn can lead to altered histone modification states. To get a better understanding of the complex regulatory networks in a cell, many transcription factors and histone modifications need to be analyzed together under different conditions. Since the introduction of massively parallel next-generation sequencers, chromatin immunoprecipitation followed by sequencing (ChIP-seq) has become the method of choice for genome-wide detection of regulatory elements [2]. In ChIP-seq, protein–DNA interactions are fixed with a cross-linking agent and after shearing; chromatin bound by a specific protein can be immunoprecipitated. The DNA is then purified and subjected to library construction reactions, where the fragment ends are made double stranded, phosphorylated and A-tailed before sequencer-specific adaptors are ligated to allow amplification and sequencing of the sample. While the first ChIP-seq experiments were limited by the sequencing capacity, the high throughputs of the current instruments allow many ChIP samples to be multiplexed and sequenced simultaneously in a single run. However, standard protocols for ChIP and library preparation have low throughput and can take up to 5 days to perform [3], thus library preparation can now be a limiting factor for ChIP-seq experiments. To overcome this, we developed a library-on-beads ChIP-seq protocol (lobChIP), where the library is made during the ChIP step before elution and decross-linking of the sample. We show here that the lobChIP protocol works well for both TFs and histone modifications, and facilitates the parallel handling of large sample numbers both manually and using automated pipetting robots.

## Results

### lobChIP permits a shorter ChIP-seq workflow which results in high concordance with public datasets

**Figure 1:**
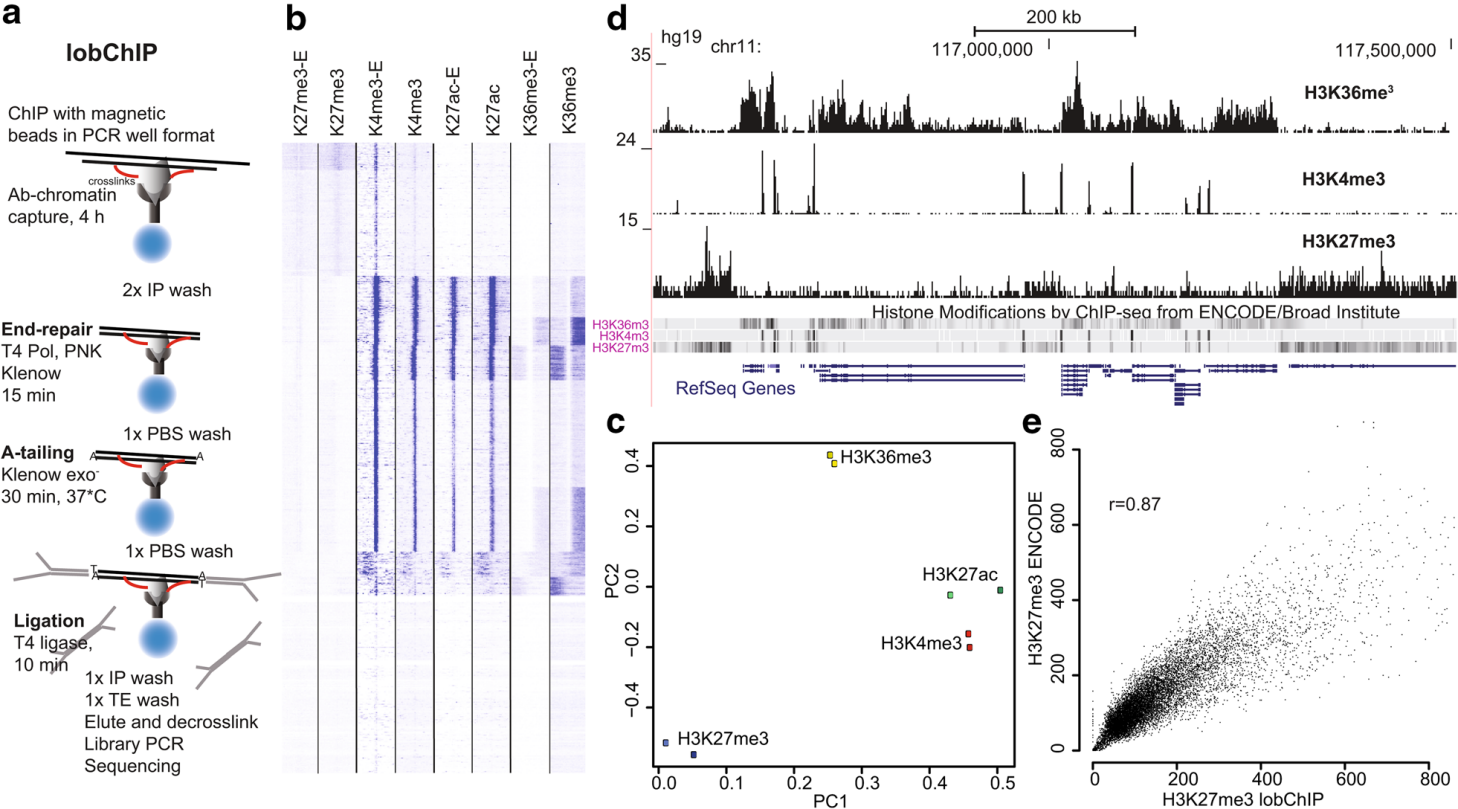
**a** Flowchart of the lobChIP procedure with timings used in the 1-day protocol. **b**–**e** Comparison of lobChIP to ENCODE data for histone modifications. **b** Clustering of read intensities at TSS for four different histone modifications from ENCODE (-E) and lobChIP. **c** PCA plot of the four different histone modifications, with ENCODE in *dark colors* and lobChIP in *light colors*. **d** Representative enrichment for H3K36me3, H3K4me3 and H3K27me3 over a 1 Mb window of chromosome 11. Corresponding ENCODE results are given as *dense tracks* above the RefSeq genes. **e** Scatter plot comparing lobChIP with ENCODE read counts for H3K27me3 in 40 kb windows centered at TSS.

We reasoned that when the desired end product of a ChIP experiment is a sequencing library, it would be advantageous to perform library reactions during the ChIP step rather than after DNA purification. This makes the protocol faster and easier to carry out, since the need for precipitations or column purifications between the reactions is removed. We performed ChIP using standard protocols until the IP washes and end repair, A-tailing and ligation reactions were done directly on cross-linked chromatin attached to magnetic beads (Figure 1a), with brief washes with PBS in between the reactions to remove enzymes. Besides reducing the risk of sample cross-contamination, the ligation of barcoded adapters before elution means that most adaptor dimers will be in solution and can easily be removed to allow amplification of the sample without a prior bead- or gel-based size selection. In comparison, standard ChIP-seq protocols followed by the Illumina TruSeq ChIP-seq library protocol takes 4–5 days even with recent modifications to remove several laborious spin-column steps (Additional file 1: Figure S1). The cost per reaction for lobChIP is lowered by more than tenfold compared to TruSeq and is also lower than other protocols using off-the-shelf reagents, since less purification reagents are needed.

To evaluate the lobChIP method and identify potential biases, we used a panel of antibodies targeting well-characterized histone modifications. We selected H3K4me3 and H3K27ac as markers for active genes and enhancers, H3K36me3 for actively transcribed regions and H3K27me3 as a mark for transcriptionally silent chromatin, and compared the results with public data. One concern with the lobChIP method could be that the cross-linked proteins might interfere with the library reactions in a non-random way throughout the genome; however, our results for this panel of histone modifications present in both heterochromatin and euchromatin are near identical to those obtained with standard methods in the ENCODE project [4] for the same cell line, as shown by read enrichment at transcription start sites (TSS) in Figure 1b and by principal component analysis of genome-wide read densities in Figure 1c. Representative lobChIP signals for H3K36me3, H3K4me3 and H3K27me3 are shown in Figure 1d, with the regions identified by ENCODE given as a dense track below for comparison. More variation at the base pair level is seen for the widespread modifications compared to the more punctate H3K4me3, but there is a good overall correlation between lobChIP and ENCODE when larger windows are considered as shown for H3K27me3 (Figure 1e, *r* = 0.88).

### Direct elution and amplification of lobChIP samples

**Figure 2:**
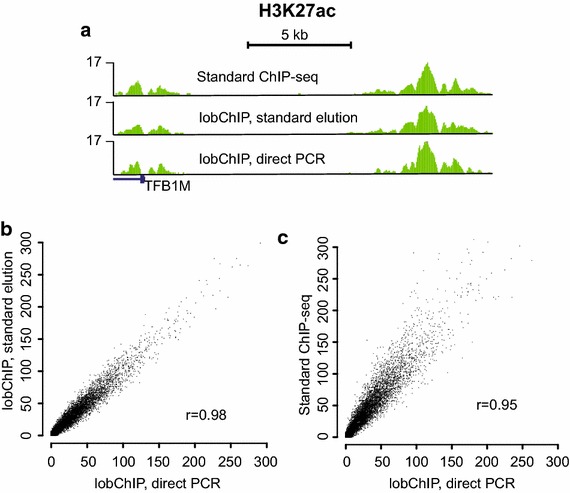
Comparison of direct elution and amplification to SDS elution followed by decross-linking and purification for H3K27ac. **a** Representative signals of enrichment for a TSS (*left*) and distal site (*right*). **b** Scatter plot of reads at TSS for the two different elutions and amplifications from the same lobChIP sample. **c** Comparison of enrichment at TSS for the directly eluted lobChIP sample and a sample done with standard ChIP-seq protocol. Read counts on *x-* and *y-axis* are normalized to sequencing depth.

In ChIP, formaldehyde cross-links are reversed for several hours at 65°C in the presence of salts, and although higher temperatures have been shown to be more efficient [5], this condition is used to minimize bias introduced by preferential denaturing of AT-rich sequences during de-cross-linking [6]. Given the complexity of ChIP DNA, denatured fragments may not re-anneal properly and could thus be depleted from the final library. In lobChIP, the ligation is done before reversing the cross-links; hence, decross-linking can be done at a higher temperature without affecting the base composition of the amplifiable fragments. We reasoned that this would also allow a direct amplification from the ChIP beads without prior elution of the sample. We first tested the ability to elute by heat only and found that, while most fragments were eluted after 15 min at 75°C in water, some additional fragments could be amplified from the remaining beads (Additional file 1: Figure S2). We therefore increased the decross-linking and elution temperature to 95°C, and added PCR reagents directly to the eluted sample and beads. We evaluated this method for H3K27Ac as follows. A lobChIP sample was split into two tubes after ligation and amplified either using the direct method or after standard SDS elution with proteinase K digestion. In parallel, a library was made using standard ChIP-seq protocols. The enrichment profiles for the two lobChIP samples amplified after different elutions were almost identical to the standard ChIP-seq sample both at TSS and distal sites, as exemplified in Figure 2a. The genome-wide enrichment at TSS was in good agreement for all samples, with the highest concordance seen between the two different elutions of the lobChIP sample (Figure 2b, c). This experiment was repeated using less chromatin and a shorter antibody incubation time, giving similar results but with lower overall enrichment (Additional file 1: Figure S3).

### Optimization of enrichment

Many histone modifications are highly abundant and can be regarded as easy ChIP-seq targets; despite this, the enrichment varies greatly in published datasets (Additional file 1: Figure S4). To test the effect of chromatin concentration on enrichment with lobChIP, we made a dilution series from 100 to 1 million cells, and found that while 1 million cells was sufficient to detect H3K27ac enrichment, there was a near linear correlation between enrichment and cell count even up to 100 million cells (Additional file 1: Figure S4). We further explored ways to reduce the overall time of the protocol with reduced IP and decross-linking times while retaining good enrichment and found that for H3K4me3 high enrichment could be obtained with a shorter protocol where both the time for antibody incubation and for decross-linking was reduced (“Methods”). This yielded an enrichment comparable to the best of the 175 tested ENCODE datasets, with high correlation to the best ENCODE replicate for HepG2 (Additional file 1: Figure S4), and while this represents a single experiment, it shows that with more work to optimize reaction conditions better results can be achieved even for common ChIP targets.

### Multiplex lobChIP in PCR strip format

**Figure 3:**
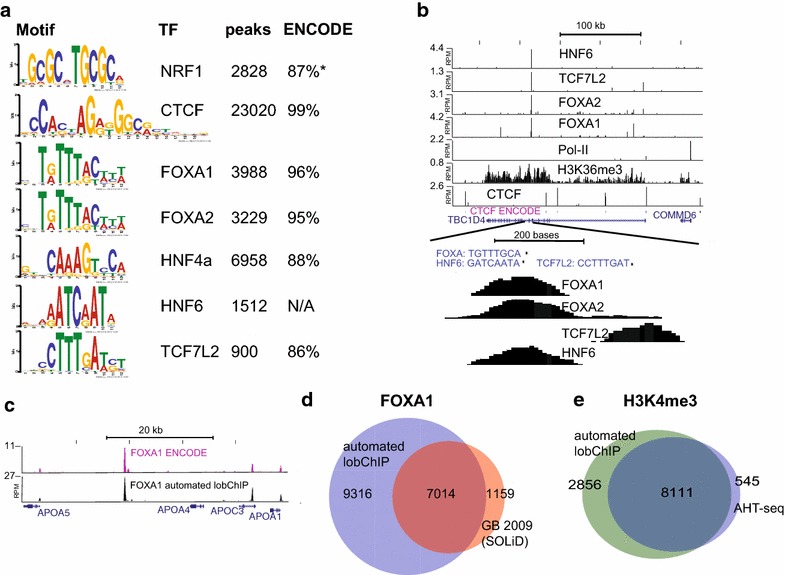
Results from manual and automated multiplexed lobChIP runs. **a** Motifs identified de novo for seven TFs from the manual 1-day lobChIP experiment, with the number of identified peaks and percentage of peaks overlapping (within 1 kb) with ENCODE peaks. (*Asterisk*) For NRF1 where more peaks were identified in our dataset, the percentage of ENCODE overlapping with our peaks is given. **b** Normalized signals (RPM) for H3K36me3, Pol II and TFs over the TBC1D4 gene with an enlarged region illustrating motif positions and peaks for FOXA1/2, TCF7L2 and HNF6 at distinct locations. **c** Automated lobChIP results for FOXA1 give an enrichment profile similar to the ENCODE sample, as exemplified here for the *APOA5*–*APOA1* region. **d** Venn diagram for FOXA1 overlaps for automated lobChIP (*blue*) and standard ChIP-seq protocols on the SOLiD instrument [8] (*red*). **e** Venn diagram of overlaps between genes with enrichment for H3K4me3 in the AHT-ChIP-seq study (*blue*) and in the automated lobChIP run (*green*).

One of the main objectives with the lobChIP protocol was to simplify the handling of multiple samples to allow more factors to be studied in parallel. We designed a multiplex experiment where the volumes of chromatin and washings were reduced to 150 µl, which allows the use of standard PCR strips and multichannel pipettes. We have previously studied the liver-specific TFs FOXA1, FOXA2 and HNF4a in HepG2 cells [7, 8], and to continue this work, we repeated these experiments and further included antibodies targeting TCF7L2, HNF6 and NRF1, since motifs for these TFs were found to be overrepresented in proximity to the previously studied factors [7, 9]. We also included antibodies for the more general factors, CTCF and Pol II, as well as for the histone modification H3K36me3 and a negative control (IgG). The protocol was adjusted, so that all steps from chromatin to amplified library could be carried out in a single day (“Methods”; Figure 1a), using a 4-h immunoprecipitation with chromatin from approximately 5 million cells per reaction. After washes and library reactions, each sample was divided into two to further compare the direct and standard elution methods described above by sequencing the two amplified libraries from each ChIP reaction on separate lanes (Additional file 1: Figure S5). We found that directly amplified samples had a slightly higher adaptor dimer contamination, but all samples had more than 90% aligned reads and a low level of PCR duplicates (Additional file 2). Peak finding and de novo motif prediction on combined reads were then used to validate that all lobChIP experiments for the sequence-specific TFs had been successful. The peak lists were further validated by comparison to ENCODE results. As can be expected, the ENCODE datasets which were produced from individual experiments using a larger number of cells and deeper sequencing had a larger number of peaks for all factors except NRF1, but there was a good agreement among the called peaks with overlaps as high as 86–99% for the smaller datasets (Figure 3a). Overall, this experiment shows that a single-day lobChIP protocol works well for both TFs and histone modifications, and further that potential tissue-specific regulatory elements can be identified using a combination of related TFs, as exemplified with the intragenic region of *TBC1D4* where binding sites for four liver-specific TFs were identified (Figure 3b).

### Automating the lobChIP protocol

A fully automated setup is advantageous to minimize variation in handling between samples in high-throughput experiments. We used the Tecan Freedom Evo pipetting robot for automatization of the lobChIP protocol. This robot has one liquid handling arm and one arm for lifting plates, but no plate heaters or shakers. A program was made to perform all steps in the lobChIP protocol from washing of IP beads to PCR setup (Additional file 3). Standard PCR plates were used, and beads were kept in solution by repeated pipetting during incubations. The plate was moved over the magnet to pull beads through the washing buffers. To make the protocol as easy as possible to set up without the need for manual intervention, all library reactions including A-tailing were done at room temperature. To test the automated protocol, we incubated chromatin overnight at +4°C with beads coupled to antibodies against the H3K4me3 and FOXA1 before transfer to the robot where all subsequent lobChIP steps were performed. This experiment gave a high enrichment and good overlap with public data for FOXA1 (Figure 3c, d), with more than twice as many peaks called as in our previously published FOXA1 study using standard ChIP-seq on the SOLiD platform [8]. We compared the H3K4me3 dataset to the best replicate from another automated protocol (AHT-ChIP-seq, [10]) and found a strong correlation of enrichment at TSS (Figure 3e, *r* = 0.71) even at a low read depth, with a large overlap among detected enriched genes (Figure 3f).

### Assessment of replicability and platform independence

**Figure 4:**
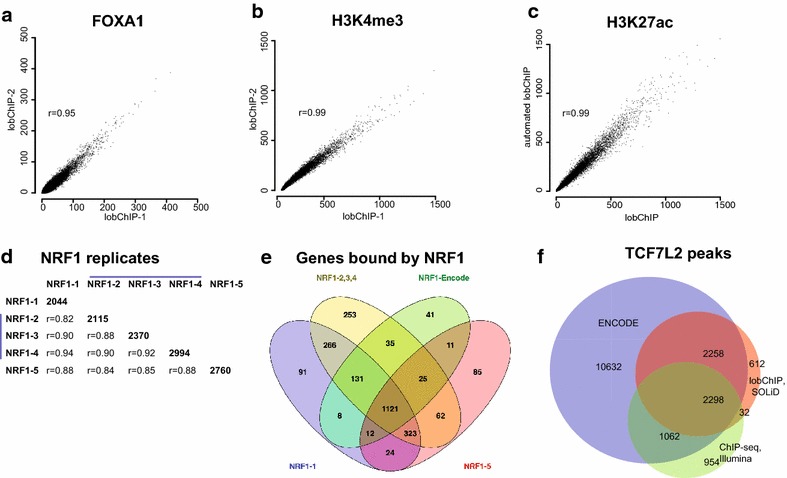
Relplicate analysis. **a** FOXA2 read counts at peak locations for two technical replicates. **b** Scatter plot comparing reads at TSS for two lobChIP technical replicates for H3K4me3. **c** H3K27ac results for automated and manual lobChIP at TSS. Read counts on *x-* and *y-axis* are normalized to sequencing depth. **d** Five replicates for NRF1, with replicate 2–4 (*blue bar*) from the same chromatin preparation. The number of peaks is given on the diagonal with correlation coefficients for enrichment below. **e** Four-way venn diagram for genes with NRF1 peaks, with merged results for the three NRF1 samples made from the same chromatin. **f** Overlap of TCF7L2 peaks identified in ENCODE, SOLiD lobChIP and standard Illumina ChIP-seq.

To assess the reproducibility of lobChIP experiments, we performed duplicate experiments for H3K4me3 and FOXA1 from the same batch of chromatin. This gave a high correlation of read enrichment (*r* = 0.99 and 0.95, respectively, Figure 4a, b). To compare the results from biological replicates, we performed a new automated lobChIP experiment for H3K27ac, which gave near identical enrichment to our best manual H3K27ac dataset (Figure 4c). We further tested the performance of lobChIP for transcription factors with five replicates for NRF1, coming from three different chromatin preparations. About 2–3,000 peaks were identified per sample, with high correlation of enrichment (Figure 4d). The vast majority of the genes identified as bound by NRF1 were found in more than one replicate, with as many as 1,121 common between all three chromatin reparations and the corresponding ENCODE dataset (Figure 4e). To test how well the protocol works with other library preparation and sequencing methods, we performed lobChIP for TCF7L2 for the SOLiD platform. We used the manufacturer’s reagents for library preparation, and for comparison a TCF7L2 sample was prepared using standard ChIP-seq on the Illumina platform. The results from the SOLiD platform showed high enrichment and good overlap with results from the Illumina platform: in total, 5,735 peaks were called, 84% were overlapping with ENCODE peaks and 45% were also present in our smaller Illumina dataset (Figure 4f).

## Discussion

Performing large-scale ChIP-seq experiments can be challenging due to the complexity of the protocols. Several attempts have been made to streamline the process either manually [11] or by automating the ChIP part or the full protocol using liquid handling robots [10, 12], and commercial instruments specific for the purpose have been developed [13]. Here, we have shown an alternative way of producing ChIP-seq libraries. While other general library preparation methods, such as the recently published TELP protocol [14], can be used for ChIP-seq, lobChIP is specific for ChIP-seq in that it takes advantage of the magnetic beads that are used to capture antibody–chromatin complexes, which can both reduce the sample preparation time and the hands-on time. Using a two-arm pipetting robot (Tecan), we show how the protocol can be automated by performing ChIP and library preparation in the 96-well format. We have also shown that lobChIP allows multiplexed experiments to be done using standard multi-channel pipettes with reduced hands-on time, since the purification steps normally done with spin columns or Ampure XP beads are removed. Throughout this manuscript we used IgG as a negative control, which can be handled in the same way as the lobChIP samples. Input DNA can also be used as a negative control; however, in that case, it would have to be processed separately since the library preparation for the input sample is done on naked DNA rather than on the chromatin.

When compared with publicly available datasets, we found no systematic bias from the change in reaction conditions. We further found that good results can be obtained when all reactions are done at room temperature, and that lobChIP allows a shorter decross-linking at a higher temperature prior to or within the PCR protocol. Reversal through boiling in a Chelex solution has been proven to work for ChIP-PCR [15], but to our knowledge it has not been used for ChIP-seq before since it will denature the sample. The early incorporation of barcoded sequencing adaptors also reduces the risk of sample cross-contamination in large-scale experiments. In summary, we found that lobChIP makes it possible to proceed from chromatin to sequencing-ready libraries within a single day. In our hands, this protocol has in many cases led to better results compared to runs using standard protocols, as shown by the comparisons of H3K4me3 and FOXA1, possibly due to the increased time on beads before elution of the samples compared to our standard protocol.

## Conclusions

The lobChIP protocol represents the advantages compared to standard procedures, especially when handling multiple samples. Compared to other high-throughput methods, the lobChIP protocol reduces the total and the hands-on time as well as the cost for the library preparation. Detailed protocols for manual and automated lobChIP, including a script for Tecan Freedom Evo, are given in the Additional file 3.

## Methods

### Cell culture and chromatin preparation

HepG2 cells were ordered from ATCC and cultured in RPMI 1640 medium supplemented with 10% non-inactivated FBS, l-glutamine and PEST (Sigma-Aldrich) at 37°C with 5% CO_2_. Cells were grown to confluence in T175-flasks and cross-linked with 0.37% formaldehyde in serum-free medium at room temperature for 10 min. Glycine was added to a final concentration of 0.125 M to stop the cross-linking and the cells were rinsed and scraped off in PBS before lysis in cell lysis buffer containing protease inhibitors. Nuclei were pelleted and lysed in RIPA buffer [1× phosphate-buffered saline (PBS), 1% NP-40, 0.5% Na-deoxycholate, 0.1% SDS, 0.004% Na-azide] before sonication with a Bioruptor (Diagenode, Liège, Belgium) for 3 × 15 min (30 s on/off at maximum amplitude) to shear the chromatin to fragments ranging between 100 and 300 bp. The cell count was estimated at 100 million per T175 flask.

### Manual lobChIP experiments

A detailed lobChIP protocol is given in Additional file 1: Supplementary methods. Protein G-coupled magnetic beads (Dynal) was used for all immunoprecipitations, with 40 μl beads per sample. Antibody at a concentration of 1 μg/10 μl beads was incubated at room temperature (RT) for 1 h in PBS containing 0.5% BSA, and unbound antibody was removed prior to IP. Beads were then incubated with chromatin in RIPA (1× PBS, NP-40 1%, Na-deoxycholate 0.5%, SDS 0.1%, sodium azide 0.004%) on a rotating platform in a cold room or RT as stated in the main text. Beads were washed either by pipetting or by alternating the position of the magnet to move beads through the washing buffer. For the manual multiplexed experiment, we used two washes with RIPA and one with IPWB2 (0.01 M Tris–HCl pH 8, 0.25 M LiCl, 0.001 M EDTA, 1% NP-40) prior to library construction. Beads were then dissolved in TE buffer (10 mM Tris–HCl pH 8, 1 mM EDTA) and transferred to new tubes to minimize the remaining inhibitory salts and SDS. Fermentas Fast end repair (15 min RT), Klenow exo^−^ (3 μl, 30 min at 37°C) and fast ligase (1 μl, 10 min RT) were used for library construction in 50 μl volumes, with a single PBS wash between each reaction. For the other experiments, End-IT (Epicenter) was used for end repair and NEB Quick ligase for adaptor ligation. Beads were further washed with IPWB2 and TE to remove adaptor dimers after ligation. For CTCF and HNF6, we used halved volumes of all reagents. For direct elution and decross-linking, a pre-incubation at 95°C (7–10 min) was used followed by vortexing and mixing with a PCR master mix (KAPA or Pfu). Libraries were amplified for 16–18 cycles. Library amplification was verified on a 2% agarose gel and pooling was done based on Qubit readings for the PCR products. Ampure XP (Agencourt) at a 1.1–1.3 × sample volume was used to purify pools and remove adaptor dimers after PCR. For samples with standard elution and decross-linking, an elution buffer compatible with proteinase K digestion and Ampure XP purification was used as described by Garber et al. [11]. For the H3K4me3 sample with reduced incubation times (Additional file 1: Figure S4), the amount of antibody-coupled beads was reduced to 15 µl to increase the ratio of chromatin to antibody, and a 40 min incubation at room temperature was used to pull down chromatin. Elution was done for 15 min at 55°C, followed by decross-linking for 15 min at 65°C.

### Sequencing, alignment and antibodies

All Illumina libraries except H3K4me3 were sequenced as single read (36 or 48 bp). H3K4me3 was sequenced as 100 bp paired end, but for the analysis only the first 36 bases were used to allow comparison to the shorter ENCODE reads. BWA aln v 0.7.5 [16] was used to align reads to hg19. For the SOLiD TCF7L2 sample, one library was made and used both for emulsion PCR for SOLiD 5500 XL sequencing and for direct sequencing on the 5500 Wildfire system and reads were aligned using the LifeScope software. A sample list with statistics of antibodies used and read is given in Additional file 2. All files have bene deposited to SRA (PRJNA283314).

### Peak analysis and motif discovery

SAMTOOLS [17] was used to remove reads with low alignment quality (<20) before peak calling with MACS [18] v 1.41, using a fixed fragment size of 160 bp and an IgG sample as negative control. The lists of summits were further filtered to remove false-positive peaks occurring in Satellite and rRNA repeats. De novo motif identification and comparison to established motifs was done using MEME-ChIP [19] (motif length 6–20 bases) on 100 bp fragments centered on the summits of the 500 highest peaks, and the strategy described in [7] for 8-mers was used to identify the motif locations in peaks as shown in Figure 4b. A gene was defined as bound by NRF1 if it had at least one NRF1 peak within 500 bp of a TSS. The four-way Venn diagram was made using the online Venny 2.0 tool (http://bioinfogp.cnb.csic.es/tools/venny/index.htm); two- and three-way venn diagrams were made using the R library venneuler. Pearson correlation coefficients were calculated using the “cor” function in R. Peak calls in BED format are given in Additional file 4.

### Automated lobChIP protocol

We automated the ChIP washing and library construction using a Tecan Freedom EVO^®^ robotic platform (Tecan). This model was equipped with a four-syringe LIHA (liquid handling arm) and a 96-well MCA. Chromatin corresponding to 15–20 million HepG2 cells was incubated overnight at +4°C with Dynal protein G beads coupled to antibody on a rotating platform. Six washes were performed in the 96-well plate using the pipetting robot, twice each with RIPA, IPWB2 and PBS. The supernatant was separated from the beads using a 96-well plate magnet (Invitrogen) for 2 min and 25 µl of end-repair master mix (1 µl T4 polymerase, 1 µl T4 PNK, 0.2 µl Klenow, 5 µl 10× T4 PNK buffer, 1 µl dNTP and 16.8 µl H_2_O) was added and mixed thoroughly together with the beads. The end-repair reaction was performed in room temperature for 30 min, with mixing by pipetting up and down five times every 5 min. After incubation, the plate was placed on the magnet to separate beads from supernatant and the beads were washed twice with PBS. The supernatant was removed and 25 µl of A-tail master mix (1.5 µl Klenow exo−, 5 µl Klenow buffer, 5 µl 1 mM dATP and 13.5 µl H_2_O) was added and mixed carefully with the beads. Incubation was performed for 30 min at room temperature with mixing every 5 min. Again, the beads were washed twice with 1× PBS. Adaptor ligation was then done for 15 min at room temperature by adding an adaptor and ligation master mix [0.5 µl NEB quick ligase, 12.5 µl 2× ligase buffer, 1 µl NEXTflex adaptor diluted 1:50 (BIOO Scientific), 11 µl H_2_O] to a volume of 25 µl. Thereafter, the beads were washed twice with IPWB2 and once with PBS. 50 µl of elution buffer and 4 µl of proteinase K were added and the plate was placed in a thermocycler at 65°C for at least 30 min. After elution and reverse cross-linking in the thermocycler, the plate was placed back in the magnet in the robot for 2 min and the supernatant was aspirated and transferred to new wells. An equal volume of Ampure XP beads was added, and beads together with liquid were thoroughly mixed by pipetting up and down 20 times. After incubating at room temperature for 5 min, the plate was placed on the magnet again and the supernatant was aspirated off and discarded. 70% freshly prepared ethanol was added to wash the Ampure XP beads twice. All traces of ethanol were removed and the beads let dry for 5 min. 30 µl of EB buffer was added to the beads to elute the DNA by pipetting up and down 20 times. After this step, the plate was placed onto the magnet and, after 5 min, 25 µl of supernatant was aspirated and dispensed in new wells. Finally, 25 µl of PCR master mix (2 µl primers, 0.625 µl dNTP, 3 µl Pfu buffer and 1 µl Pfu enzyme) was added and the library was amplified in a thermocycler (18 cycles). All washing steps done by the pipetting robot was performed by lifting the plate and putting it back and forth in the magnet and thereby letting the beads move from one side to the other through the liquid. All steps from removing liquid in the first step to the end when adding the PCR mix were done by the robot, with one interruption when incubating the plate at 65°C. The programming of the script was done in Freedom EVOware Standard 2.4 SP2 (Tecan).

### Comparison to public data

All ENCODE datasets were downloaded from UCSC. For peak locations, the uniform peak calls for combined replicates with the SPP peak caller were used (Additional file 2). seqMINER [20] was used to calculate read counts and produce heatmaps of read enrichment at TSS. Only TSS on the positive strand was used, and windows of 2 kb centered on TSS were merged to avoid duplicate counts. AHT-ChIP-seq reads were downloaded from GEO and aligned and processed as for the lobCHIP samples. Picard was used to subsample BAM files. Overlapping peaks were defined as those with a maximum distance of 1 kb between summits. Principal component analysis was done using the R function prcomp on read counts in 10 kb bins over the genome. Bins with less than ten reads or overlapping with satellite or rRNA repeats or ENCODE blacklisted regions were removed.

## Additional files

Additional file 1. Supplementary Figures 1–5 and a full lobChIP protocol. Additional file 2. Excel file with list of samples, read statistics and overlap between the datasets. Additional file 3. EVOware script for running lobChIP on the Tecan EVO pipetting robot. Additional file 4. A list of lobChIP peak calls used in the manuscript. For each peak the position and height of the summit as calculated by MACS is given, followed by the file name for the corresponding ChIP experiment.

## Abbreviations

Ac: acetylation
Me: methylation
ChIP: chromatin immunoprecipitation
Lob: library on beads
TSS: transcription start site
TF: transcription factor
PBS: phosphate-buffered saline
PCR: polymerase chain reaction
ENCODE: Encyclopedia of DNA Elements
DNA: deoxyribonucleic acid
SDS: sodium dodechyl sulfate
FOX: forkhead box
HNF: hepatocyte nuclear factor
TCF7L2: transcription factor 7-like 2 (T cell specific, HMG-box)
CTCF: CCCTC-binding factor
Pol: polymerase
NRF1: nuclear respiratory factor 1
IgG: immunoglobulin G
