# Supplementary material for: lobChIP: from cells to sequencing ready ChIP libraries in a single day
Source: Epigenetics Chromatin. 2015 Jul 21;8:25. doi: 10.1186/s13072-015-0017-5 (PMC4507313; doi:10.1186/s13072-015-0017-5)
Supplement: Additional file 1. — Supplementary Figures 1–5 and a full lobChIP protocol. [file 13072_2015_17_MOESM1_ESM.docx]

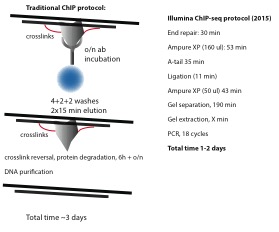


**Figure S1.** In traditional ChIP-seq the crosslinked protein-DNA complexes are eluted from the antibody, and crosslinks are reversed and DNA purified before library construction.


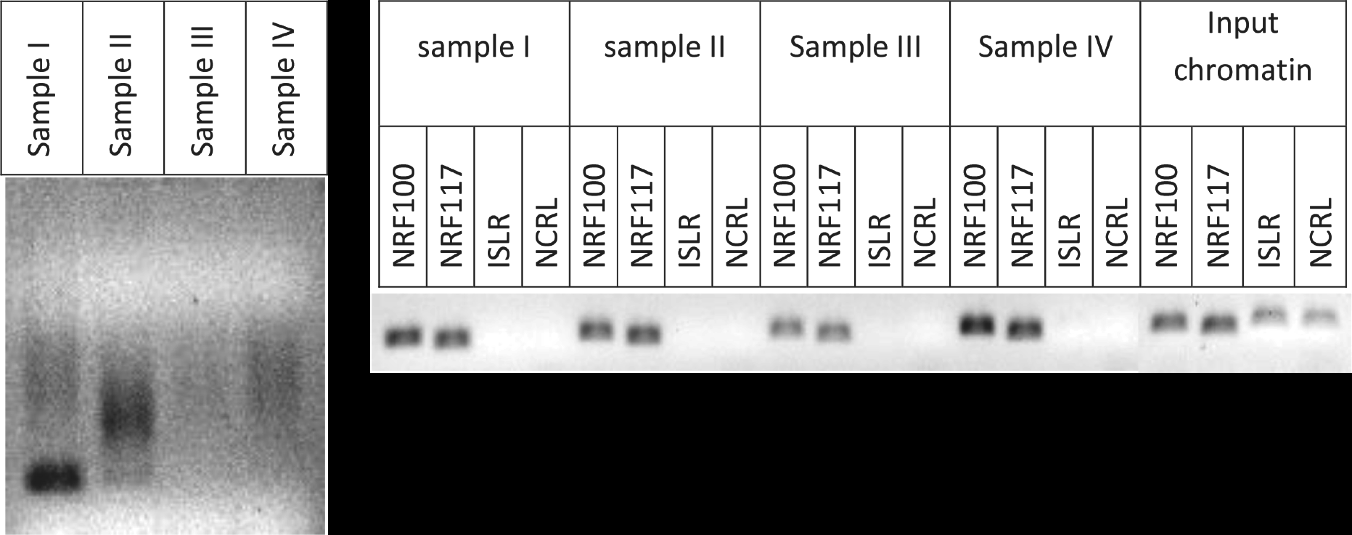


**Figure S2.** Amplification of a lobChIP experiment for NRF1 divided into four samples. Water (25 ul) was used to wash beads briefly after ligation and kept as sample I. For sample II beads were heated at 75°C for 15 minutes. Sample III was repeated as for sampe II, and the remaining beads were then transferred directly to a PCR as sample IV. Sample I shows that some ChIP fragments are lost in the washing, and that most adaptor dimers can be removed with a short wash. From sample IV it appears that mainly longer fragments are remaining on beads after heating in water. Semi-qPCR shows enrichment for two positive regions (NRF100 and NRF117) and no amplification in the negative region for all four samples.

**Figure S3**. Comparison of direct and standard elution for H3K27ac lobChIP samples. (A) Signal footprints over TSS. The tracks are from highest to lowest K27ac standard-1, K27ac direct-1, K27ac standard-2 and K27ac direct-2 where the two last samples were incubated for a shorter time (45 min) with less chromatin (150 ul vs 750 ul) compared to the top two tracks. (B) Comparison of enrichment for K27ac direct-2 (x-axis) och K27ac direct-1 (y-axis) shows high correlation despite the different reaction conditions.

**Figure S4.** (A) Comparison of enrichment at TSS for H3K27ac lobChIP experiments done in parallel with 100M, 20M, 4M and 1M cells. (B) Scatterplot of reads at TSS for the best lobChIP and the best ENCODE replicate for HepG2. (C) The fraction of reads at TSS differs between ENCODE groups. The most enriched lobChIP sample had had a higher fraction of reads at TSS compared to the ENCODE datasets.

**Figure S5**. Comparison of direct PCR to standard SDS-elution and de-crosslinking for lobChIP samples in a 8 Mb window. Only samples with at least four million reads each were used. All samples except the TCF7L2 are from the manual one-day experiment, TCF7L2 samples were done in parallel with K27ac for the first tests of the direct elution method.

**Supplementary Methods**

**Recommended protocol for lobChIP**

1. Grow or harvest the cells (~10 * 10^6^ cells/ChIP). Wash once with room temperature 1xPBS.

Suspension cells: collect the cells by centrifugation at 1200 rpm for 5-8 min.

Adherent cells: go to next step.

1. Resuspend the cells in 45 ml serum-free medium in cell culture flask/ Falcon tubes.
2. To cross-link, add 16% folmaldehyde to a final concentration of 0.37% and incubate at room temperature with gentle agitation (on rocking bed) for 10 min.
3. Prepare your IPs. Take 10 µl Protein-G beads (Dynal) / 1 µg antibody, wash the beads (by using a magnetic stand) twice by adding 300 µl 1xPBS + 0.05 % BSA. Add antibody and 200 µl 1xPBS + 0.1 % BSA. Pre-incubate on a rotating wheel at 4°C for at least 1 hour.
4. Stop the cross-linking reaction by adding 2M glycine to a final concentration of 0.125 M and incubate for 5 min at room temperature with gentle agitation. Put flask/tube on ice.
5. Only for adherent cells: Use a scraper to detach cells and collect them in a 50 ml Falcon tube. Keep the tube on ice or at 4°C for the following steps.
6. Centrifuge the cells at 1200 rpm for 6 min at 4°C.
7. Resuspend the pellet with 1.5 ml ice-cold 1x PBS and centrifuge the cells at 2000 rpm for 5 min at 4°C.
8. Add the following protease inhibitors:

40 µl/ml PIC (cOmplete Protease Inhibitor Cocktail, Roche)

5 µl/ml PMSF (100x) (add just before use)

to the cell lysis buffer (CLB). Gently resuspend the cell pellet in 450 µl of CLB by pipetting up and down. Incubate on ice for 10 min and centrifuge at 2500 rpm for 5 min at 4°C to collect the nuclei.

1. Remove the supernatant and resuspend nuclei in 1 ml RIPA with PIs added. Keep on ice for 10 min. Transfer to a 15 ml tube.
2. Sonicate the sample using Bioruptor. The settings for HepG2 cells are as follows:

On-time 30 sec

Off-time 30 sec

Total time 30 min

Output effect High

1. Transfer the sheared chromatin to 1.5 ml tube and spin at 13000 rpm for 5 min at 4°C.
2. Aliquot the supernatant to the tubes with beads and antibody. Incubate 45 min in room temperature or o.n. at 4°C.

Manual library construction with direct elution in PCR-tubes

*End-repair, A-tailing and adaptor ligation*

1. Put the 1.5 ml tubes from step 13 in the magnetic stand and wait until the solution is clear. Pipette off the liquid and discard. Resuspend the beads with 150 µl RIPA. Transfer to PCR-tubes and put the tubes in a magnetic stand suitable for PCR-plates. Discard the supernatant.
2. Wash twice with 150 µl of RIPA by alternating the position of the magnet to move beads through the washing buffer.
3. Wash once with 150 µl IPWB2.
4. Dissolve beads in 150 µl TE pH 8 and transfer to new tubes.
5. Fermentas Fast end repair (15 min RT) was used according to the manufacturer’s protocol.
6. Wash once with 150 µl of TE by alternating the position of the magnet.
7. Fermentas Klenow exo- (30 min RT) was used for A-tailing according to the manufacturer’s protocol.
8. Wash once with 150 µl of TE by alternating the position of the magnet.
9. Fermentas fast ligase (15 min RT) was used for adapter ligation according to the manufacturer’s protocol.
10. Wash beads once with IPWB2 and TE respectively.
11. For direct elution and de-crosslinking, pre-incubate the samples at 95°C (8-10 min) with 25 µl of ddH_2_O followed by vortexing and mixing with a PCR master mix (KAPA or Pfu).
12. Amplify 16-18 cycles (2 min 95°C, (30 sec 95°C, 30 sec 55°C, 1 min 72°C – 16-18 cy), 10 min 72°C).
13. Verify the library on a 2% agarose gel.
14. Pool samples based on Qubit quantifications for the PCR products.
15. Purify and remove adaptor dimers by adding 1.1-1.3 x Ampure XP beads and wash beads twice with 70% EtOH. Let beads air-dry and elute the library in ddH_2_O or EB buffer.

Automated library construction with standard elution in PCR-tubes

*End repair*

1. Wash twice each with 150 µl RIPA, IPWB2 and 1xPBS by moving the plate back and forth on a 96-well magnet to pull beads thought the washing solution.
2. Separate the supernatant from the beads for 2 min and discard.
3. Add 25 µl of End repair master mix (2.5 µl 10 x T4 PNK buffer, 16.8 µl ddH_2_O, 1 µl T4 Polymerase, 1 µl T4 PNK and 0.2 µl Klenow). Mix thoroughly by pipetting up and down.
4. Incubate 30 min in RT. Mix five times every five minute.
5. Put tubes on the magnet and wait for 2 minutes, discard the supernatant.
6. Wash twice with 1 x PBS, discard supernatant.

*A-tailing*

1. Add 25 µl of A-tail master mix (5 µl Klenow buffer, 5 µl 1mM dATP, 13.5 µl dH_2_O and 1.5 µl Klenow exo-). Mix thoroughly by pipetting up and down.
2. Incubate 30 min in RT. Mix five times every five minute.
3. Put tubes on the magnet and wait for 2 minutes, discard the supernatant.
4. Wash twice with 1 x PBS, discard supernatant.

*Adaptor ligation, elution and reverse crosslinking*

1. Add 24 µl of ligation master mix (12.5 µl 2x ligase buffer, 11 µl dH_2_O and 0.5 µl NEB quick ligase) and 1 µl of NEXTflex adaptor diluted 1:50 (BIOO Scientific).
2. Incubate 15 min in RT. Mix five times every five minute.
3. Put tubes on the magnet and wait for 2 minutes, discard the supernatant.
4. Wash twice with IPWB2 and once with 1 x PBS, discard supernatant.
5. Add 50 µl of elution buffer and 4 µl of Proteinase K
6. Place the tubes in a thermo cycler at 65°C for at least 30 min.
7. Put the tubes back in the magnet for 2 min.
8. Aspirate off supernatant and transfer to new wells.
9. Remove tubes from the magnet.
10. Add equal volume of Ampure XP beads and thoroughly mix by pipetting up and down 20 times.
11. Let the beads incubate for 5 min.
12. Put the tubes back in the magnet.
13. Discard the supernatant.
14. Wash twice with fresh 70% EtOH.
15. Remove all traces of EtOH and let air-dry for at least 5 min.
16. Remove the tubes from the magnet.
17. Add 30 µl of EB buffer and pipette up and down 20 times.
18. Put the tubes back to the magnet and wait for 5 min.

*Amplification*

1. Aspirate 20 µl of the supernatant and dispense in new wells.
2. Add 6.5 µl of PCR master mix (2 µl primers, 1 µl 1mM dNTP, 2.5 µl Pfu buffer and 1 µl Pfu enzyme).
3. Amplify 16-18 cycles (2 min 95°C, (30 sec 95°C, 30 sec 55°C, 1 min 72°C – 16-18 cy), 10 min 72°C).
4. Verify the library on a 2% agarose gel.
5. Pool samples based on Qubit quantifications for the PCR products.
6. Purify and remove adaptor dimers by adding 1.1-1.3 x Ampure XP beads and wash beads twice with 70% EtOH. Let beads air-dry and elute the library in ddH_2_O or EB buffer.

**Buffers**

RIPA: 1 x PBS, NP-40 1%, Na deoxycholate 0.5%, SDS 0.1%, Sodium azide 0.004%

IPWB2: 0.01 M Tris-HCl (pH 8), 0.25 M LiCl, 0.001 M EDTA, 1% NP-40

Elution buffer: 10 mM Tris-HCl Ph 8.0, 5 mM EDTA, 300 mM NaCl, 0.5 %SDS
