# Supplementary material for: lobChIP: from cells to sequencing ready ChIP libraries in a single day
Source: Epigenetics Chromatin. 2015 Jul 21;8:25. doi: 10.1186/s13072-015-0017-5 (PMC4507313; doi:10.1186/s13072-015-0017-5)
Supplement: Additional file 3. — EVOware script for running lobChIP on the Tecan EVO pipetting robot. [file 13072_2015_17_MOESM3_ESM.pdf]

Step	Command	Parameters
1	Set Variable	Number of samples<br>"How many samples?"<br>"How many samples?", 1 - 96
2	Wash Tips	1-4 3.0 + 4.0 ml
3	Group	Wash RIPA
4	Set Variable	SAMPLE_CNT = Number of samples
5	Begin Loop	24 times "Wash RIPA_1"
6	Get DiTis	1-4 DiTi aluminium 200 ul
7	Aspirate	1-4 100 µl Helena Water free dispense<br>"Magnet pos 1" (Col. 1, Rows 1-4) , 1 option
8	Drop DiTis	1-4 DiTi LiHa Waste
9	End Loop	"Wash RIPA_1"
10	Set Variable	SAMPLE_CNT = -1
11	Set Variable	SAMPLE_CNT = Number of samples
12	Begin Loop	24 times "Wash RIPA_1"
13	Get DiTis	1-4 DiTi aluminium 200 ul
14	Aspirate	1-4 150 µl Water free dispense<br>"RIPA" (Col. 1, Rows 3-6)
15	Dispense	1-4 150 µl Water Detect 500µL<br>"Magnet pos 1" (Col. 1, Rows 1-4) , 1 option
16	Drop DiTis	1-4 DiTi LiHa Waste
17	End Loop	"Wash RIPA_1"
18	Set Variable	SAMPLE_CNT = -1
19	Drop DiTis	1-4 DiTi LiHa Waste
20	Transfer Labware	Source: Grid '14,' Site '3'; Destination: Grid '14', Site '4'; Narrow (MCA96 1)
21	Transfer Labware	Source: Grid '14,' Site '4'; Destination: Grid '14', Site '3'; Narrow (MCA96 1)
22	Transfer Labware	Source: Grid '14,' Site '3'; Destination: Grid '14', Site '4'; Narrow (MCA96 1)
23	Transfer Labware	Source: Grid '14,' Site '4'; Destination: Grid '14', Site '3'; Narrow (MCA96 1)

24	Start Timer		2
25	Wait for Timer		Timer 2 : 60 sec
26	Set Variable		SAMPLE_CNT = Number of samples
27	Begin Loop		24 times "Wash RIPA_2"
28	Get DiTis	1-4	DiTi aluminium 200 ul
29	Aspirate	1-4	150 µl Helena Water free dispense<br>"Magnet pos 1" (Col. 1, Rows 1-4) , 1 option
30	Drop DiTis	1-4	DiTi LiHa Waste
31	End Loop		"Wash RIPA_2"
32	Set Variable		SAMPLE_CNT = -1
33	Set Variable		SAMPLE_CNT = Number of samples
34	Begin Loop		24 times "Wash RIPA_3"
35	Get DiTis	1-4	DiTi aluminium 200 ul
36	Aspirate	1-4	150 µl Water free dispense<br>"RIPA" (Col. 1, Rows 3-6)
37	Dispense	1-4	150 µl Water Detect 500µL<br>"Magnet pos 1" (Col. 1, Rows 1-4) , 1 option
38	Drop DiTis	1-4	DiTi LiHa Waste
39	End Loop		"Wash RIPA_3"
40	Set Variable		SAMPLE_CNT = -1
41	Transfer Labware		Source: Grid '14,' Site '3'; Destination: Grid '14', Site '4'; Narrow (MCA96 1)
42	Transfer Labware		Source: Grid '14,' Site '4'; Destination: Grid '14', Site '3'; Narrow (MCA96 1)
43	Transfer Labware		Source: Grid '14,' Site '3'; Destination: Grid '14', Site '4'; Narrow (MCA96 1)
44	Transfer Labware		Source: Grid '14,' Site '4'; Destination: Grid '14', Site '3'; Narrow (MCA96 1)
45	Start Timer		3
46	Wait for Timer		Timer 3 : 60 sec
47	Set Variable		SAMPLE_CNT = Number of samples

No.	Command	Tips	Parameters
48	Begin Loop		24 times "Wash RIPA_4"
49	Get DiTis	1-4	DiTi aluminium 200 ul
50	Aspirate	1-4	150 µl Helena Water free dispense<br>"Magnet pos 1" (Col. 1, Rows 1-4) , 1 option
51	Drop DiTis	1-4	DiTi LiHa Waste
52	End Loop		"Wash RIPA_4"
53	Set Variable		SAMPLE_CNT = -1
54	Group End		Wash RIPA
55	Group		Wash IPWB2
56	Set Variable		SAMPLE_CNT = Number of samples
57	Begin Loop		24 times "Wash IPWB2_1"
58	Get DiTis	1-4	DiTi aluminium 200 ul
59	Aspirate	1-4	150 µl Water free dispense<br>"IPWB2" (Col. 1, Rows 3-6)
60	Dispense	1-4	150 µl Water Detect 500µL<br>"Magnet pos 1" (Col. 1, Rows 1-4) , 1 option
61	Drop DiTis	1-4	DiTi LiHa Waste
62	End Loop		"Wash IPWB2_1"
63	Set Variable		SAMPLE_CNT = -1
64	Transfer Labware		Source: Grid '14,' Site '3'; Destination: Grid '14', Site '4'; Narrow (MCA96 1)
65	Transfer Labware		Source: Grid '14,' Site '4'; Destination: Grid '14', Site '3'; Narrow (MCA96 1)
66	Transfer Labware		Source: Grid '14,' Site '3'; Destination: Grid '14', Site '4'; Narrow (MCA96 1)
67	Transfer Labware		Source: Grid '14,' Site '4'; Destination: Grid '14', Site '3'; Narrow (MCA96 1)
68	Start Timer		4
69	Wait for Timer		Timer 4 : 60 sec
70	Set Variable		SAMPLE_CNT = Number of samples
71	Begin Loop		24 times "Wash IPWB2_2"

No.	Command	Tips	Details
72	Get DiTis	1-4	DiTi aluminium 200 ul
73	Aspirate	1-4	150 µl Helena Water free dispense<br>"Magnet pos 1" (Col. 1, Rows 1-4) , 1 option
74	Drop DiTis	1-4	DiTi LiHa Waste
75	End Loop		"Wash IPWB2_2"
76	Set Variable		SAMPLE_CNT = -1
77	Set Variable		SAMPLE_CNT = Number of samples
78	Begin Loop		24 times "Wash IPWB2_3"
79	Get DiTis	1-4	DiTi aluminium 200 ul
80	Aspirate	1-4	150 µl Water free dispense<br>"IPWB2" (Col. 1, Rows 3-6)
81	Dispense	1-4	150 µl Water Detect 500µL<br>"Magnet pos 1" (Col. 1, Rows 1-4) , 1 option
82	Drop DiTis	1-4	DiTi LiHa Waste
83	End Loop		"Wash IPWB2_3"
84	Set Variable		SAMPLE_CNT = -1
85	Transfer Labware		Source: Grid '14,' Site '3'; Destination: Grid '14', Site '4'; Narrow (MCA96 1)
86	Transfer Labware		Source: Grid '14,' Site '4'; Destination: Grid '14', Site '3'; Narrow (MCA96 1)
87	Transfer Labware		Source: Grid '14,' Site '3'; Destination: Grid '14', Site '4'; Narrow (MCA96 1)
88	Transfer Labware		Source: Grid '14,' Site '4'; Destination: Grid '14', Site '3'; Narrow (MCA96 1)
89	Start Timer		5
90	Wait for Timer		Timer 5 : 60 sec
91	Set Variable		SAMPLE_CNT = Number of samples
92	Begin Loop		24 times "Wash IPWB2_4"
93	Get DiTis	1-4	DiTi aluminium 200 ul
94	Aspirate	1-4	150 µl Helena Water free dispense<br>"Magnet pos 1" (Col. 1, Rows 1-4) , 1 option
95	Drop DiTis	1-4	DiTi LiHa Waste

96	End Loop	"Wash IPWB2_4"
97	Set Variable	SAMPLE_CNT = -1
98	Group End	Wash IPWB2
99	Group	Wash PBS
100	Set Variable	SAMPLE_CNT = Number of samples
101	Begin Loop	24 times "Wash PBS_1"
102	Get DiTis (1-4)	DiTi aluminium 200 ul
103	Aspirate (1-4)	150 µl Water free dispense "PBS" (Col. 1, Rows 3-6)
104	Dispense (1-4)	150 µl Water Detect 500µL "Magnet pos 1" (Col. 1, Rows 1-4) , 1 option
105	Drop DiTis (1-4)	DiTi LiHa Waste
106	End Loop	"Wash PBS_1"
107	Set Variable	SAMPLE_CNT = -1
108	Transfer Labware	Source: Grid '14,' Site '3'; Destination: Grid '14', Site '4'; Narrow (MCA96 1)
109	Transfer Labware	Source: Grid '14,' Site '4'; Destination: Grid '14', Site '3'; Narrow (MCA96 1)
110	Transfer Labware	Source: Grid '14,' Site '3'; Destination: Grid '14', Site '4'; Narrow (MCA96 1)
111	Transfer Labware	Source: Grid '14,' Site '4'; Destination: Grid '14', Site '3'; Narrow (MCA96 1)
112	Set Variable	SAMPLE_CNT = Number of samples
113	Begin Loop	24 times "Wash PBS_2"
114	Get DiTis (1-4)	DiTi aluminium 200 ul
115	Aspirate (1-4)	150 µl Water free dispense "Magnet pos 1" (Col. 1, Rows 1-4) , 1 option
116	Drop DiTis (1-4)	DiTi LiHa Waste
117	End Loop	"Wash PBS_2"
118	Set Variable	SAMPLE_CNT = -1
119	Set Variable	SAMPLE_CNT = Number of samples

Begin Loop 120		24 times "Wash PBS_3"
Get DiTis 121	1-4	DiTi aluminium 200 ul
Aspirate 122	1-4	150 µl Water free dispense<br>"PBS" (Col. 1, Rows 3-6)
Dispense 123	1-4	150 µl Water Detect 500µL<br>"Magnet pos 1" (Col. 1, Rows 1-4) , 1 option
Drop DiTis 124	1-4	DiTi LiHa Waste
End Loop 125		"Wash PBS_3"
Set Variable 126		SAMPLE_CNT = -1
Transfer Labware 127		Source: Grid '14,' Site '3'; Destination: Grid '14', Site '4'; Narrow (MCA96 1)
Transfer Labware 128		Source: Grid '14,' Site '4'; Destination: Grid '14', Site '3'; Narrow (MCA96 1)
Transfer Labware 129		Source: Grid '14,' Site '3'; Destination: Grid '14', Site '4'; Narrow (MCA96 1)
Transfer Labware 130		Source: Grid '14,' Site '4'; Destination: Grid '14', Site '3'; Narrow (MCA96 1)
Transfer Labware 131		Source: Grid '14,' Site '3'; Destination: Grid '14', Site '2'; Narrow (MCA96 1)
Group End 132		Wash PBS
Group 133		Transfer to new plate and discard PBS
Set Variable 134		SAMPLE_CNT = Number of samples
Begin Loop 135		24 times "New plate"
Get DiTis 136	1-4	DiTi aluminium 200 ul
Mix 137	1-4	1 x 150 µl Helena Water free dispense<br>"Working plate" (Col. 1, Rows 1-4) , 1 option
Aspirate 138	1-4	180 µl Water free dispense<br>"Working plate" (Col. 1, Rows 1-4) , 1 option
Dispense 139	1-4	180 µl Helena Water free dispense<br>"New plate" (Col. 1, Rows 1-4) , 1 option
Drop DiTis 140	1-4	DiTi LiHa Waste
End Loop 141		"New plate"
Set Variable 142		SAMPLE_CNT = -1
Transfer Labware 143		Source: Grid '14,' Site '1'; Destination: Grid '14', Site '3'; Narrow (MCA96 1)

144	Start Timer	6
145	Transfer Labware	Source: Grid '14,' Site '2'; Destination: Grid '32', Site '19'; Narrow (MCA96 1)
146	Wait for Timer	Timer 5 : 60 sec
147	User Prompt	"Put new plate on site 1<br><br>"<br>sound : once
148	Set Variable	SAMPLE_CNT = Number of samples
149	Begin Loop	24 times "To new plate"
150	Get DiTis	1-4 DiTi aluminium 200 ul
151	Aspirate	1-4 150 µl Helena Water free dispense "Magnet pos 1" (Col. 1, Rows 1-4)
152	Drop DiTis	1-4 DiTi LiHa Waste
153	End Loop	"To new plate"
154	Set Variable	SAMPLE_CNT = -1
155	Group End	Transfer to new plate and discard PBS
156	Group	End-repair
157	Transfer Labware	Source: Grid '14,' Site '3'; Destination: Grid '14', Site '2'; Narrow (MCA96 1)
158	Get DiTis	1-4 DiTi aluminium 200 ul
159	Set Variable	SAMPLE_CNT = Number of samples
160	Begin Loop	24 times "End repair"
161	Aspirate	1 25 µl Helena Water free dispense "Labware1" (Col. 1, Row 2)
162	Aspirate	2 25 µl Helena Water free dispense "Labware1" (Col. 1, Row 2)
163	Aspirate	3 25 µl Helena Water free dispense "Labware1" (Col. 1, Row 2)
164	Aspirate	4 25 µl Helena Water free dispense "Labware1" (Col. 1, Row 2)
165	Dispense	1-4 25 µl Helena Water free dispense "Working plate" (Col. 1, Rows 1-4) , 1 option
166	End Loop	"End repair"

167	Set Variable	SAMPLE_CNT = -1
168	Drop DiTis	1-4 DiTi LiHa Waste
169	Get DiTis	Grid 32; Site: 2 (DiTi 200ul Nested MCA96)<br>Fetch 8 rows and 12 columns
170	Mix	35 µl Helena Water free dispense<br>"Working plate" (Col. 1, Rows 1-8)
171	Begin Loop	6 times "4 first wells"
172	Start Timer	1
173	Wait for Timer	Timer 1 : 300 sec
174	Mix	35 µl Helena Water free dispense<br>"Working plate" (Col. 1, Rows 1-8)
175	End Loop	"4 first wells"
176	Drop DiTis	Grid 32; Site: 19 (DiTi Nested Waste)
177	Transfer Labware	Source: Grid '14,' Site '2'; Destination: Grid '14', Site '3'; Narrow<br>(MCA96 1)
178	Start Timer	2
179	Transfer Labware	Source: Grid '32,' Site '2'; Destination: Grid '32', Site '19'; Narrow<br>(MCA96 1)
180	Wait for Timer	Timer 2 : 30 sec
181	Group End	End-repair
182	Group	Wash after End-repair
183	Set Variable	SAMPLE_CNT = Number of samples
184	Begin Loop	24 times "wash End-repair"
185	Get DiTis	1-4 DiTi aluminium 200 ul
186	Aspirate	1-4 25 µl Helena Water free dispense<br>"Magnet pos 1" (Col. 1, Rows 1-4) , 1 option
187	Drop DiTis	1-4 DiTi LiHa Waste
188	End Loop	"wash End-repair"
189	Set Variable	SAMPLE_CNT = -1
190	Get DiTis	1-4 DiTi aluminium 200 ul

191	Set Variable	SAMPLE_CNT = Number of samples
192	Begin Loop	24 times "wash End-repair"
193	Aspirate	1-4 100 µl Water free dispense "PBS" (Col. 1, Rows 3-6)
194	Dispense	1-4 100 µl Helena Water free dispense "Magnet pos 1" (Col. 1, Rows 1-4) , 1 option
195	End Loop	"wash End-repair"
196	Set Variable	SAMPLE_CNT = -1
197	Drop DiTis	1-4 DiTi LiHa Waste
198	Move LIHA	1-4 Positioning with global Z-Travel "200 µl DiTi 1" (Col. 1, Rows 1-4)
199	Transfer Labware	Source: Grid '14,' Site '3'; Destination: Grid '14', Site '4'; Narrow (MCA96 1)
200	Transfer Labware	Source: Grid '14,' Site '4'; Destination: Grid '14', Site '3'; Narrow (MCA96 1)
201	Transfer Labware	Source: Grid '14,' Site '3'; Destination: Grid '14', Site '4'; Narrow (MCA96 1)
202	Transfer Labware	Source: Grid '14,' Site '4'; Destination: Grid '14', Site '3'; Narrow (MCA96 1)
203	Transfer Labware	Source: Grid '14,' Site '3'; Destination: Grid '14', Site '4'; Narrow (MCA96 1)
204	Transfer Labware	Source: Grid '14,' Site '4'; Destination: Grid '14', Site '3'; Narrow (MCA96 1)
205	Start Timer	3
206	Wait for Timer	Timer 3 : 30 sec
207	Set Variable	SAMPLE_CNT = Number of samples
208	Begin Loop	24 times "wash End-repair"
209	Get DiTis	1-4 DiTi aluminium 200 ul
210	Aspirate	1-4 105 µl Helena Water free dispense "Magnet pos 1" (Col. 1, Rows 1-4)
211	Drop DiTis	1-4 DiTi LiHa Waste
212	End Loop	"wash End-repair"
213	Set Variable	SAMPLE_CNT = -1
214	Get DiTis	1-4 DiTi aluminium 200 ul

215	Set Variable		SAMPLE_CNT = Number of samples
216	Begin Loop		24 times "wash End-repair"
217	Aspirate	1-4	100 µl Water free dispense<br>"PBS" (Col. 1, Rows 3-6)
218	Dispense	1-4	100 µl Helena Water free dispense<br>"Magnet pos 1" (Col. 1, Rows 1-4) , 1 option
219	End Loop		"wash End-repair"
220	Set Variable		SAMPLE_CNT = -1
221	Drop DiTis	1-4	DiTi LiHa Waste
222	Move LIHA	1-4	Positioning with global Z-Travel<br>"200 µl DiTi 1" (Col. 1, Rows 1-4)
223	Transfer Labware		Source: Grid '14,' Site '3'; Destination: Grid '14', Site '4'; Narrow<br>(MCA96 1)
224	Transfer Labware		Source: Grid '14,' Site '4'; Destination: Grid '14', Site '3'; Narrow<br>(MCA96 1)
225	Transfer Labware		Source: Grid '14,' Site '3'; Destination: Grid '14', Site '4'; Narrow<br>(MCA96 1)
226	Transfer Labware		Source: Grid '14,' Site '4'; Destination: Grid '14', Site '3'; Narrow<br>(MCA96 1)
227	Transfer Labware		Source: Grid '14,' Site '3'; Destination: Grid '14', Site '4'; Narrow<br>(MCA96 1)
228	Transfer Labware		Source: Grid '14,' Site '4'; Destination: Grid '14', Site '3'; Narrow<br>(MCA96 1)
229	Start Timer		4
230	Wait for Timer		Timer 4 : 30 sec
231	Set Variable		SAMPLE_CNT = Number of samples
232	Begin Loop		24 times "wash End-repair"
233	Get DiTis	1-4	DiTi aluminium 200 ul
234	Aspirate	1-4	105 µl Helena Water free dispense<br>"Magnet pos 1" (Col. 1, Rows 1-4)
235	Drop DiTis	1-4	DiTi LiHa Waste
236	End Loop		"wash End-repair"
237	Set Variable		SAMPLE_CNT = -1
238	Transfer Labware		Source: Grid '14,' Site '3'; Destination: Grid '14', Site '2'; Narrow<br>(MCA96 1)

No.	Command	Details
239	Group End	Wash after End-repair
240	Group	A-tailing
241	Get DiTis	1-4 DiTi aluminium 200 ul
242	Set Variable	SAMPLE_CNT = Number of samples
243	Begin Loop	4 times "4 first wells"
244	Aspirate	1 25 µl Helena Water free dispense "Labware1" (Col. 1, Row 3)
245	Aspirate	2 25 µl Helena Water free dispense "Labware1" (Col. 1, Row 3)
246	Aspirate	3 25 µl Helena Water free dispense "Labware1" (Col. 1, Row 3)
247	Aspirate	4 25 µl Helena Water free dispense "Labware1" (Col. 1, Row 3)
248	Dispense	1-4 25 µl Water Detect 500µL "Working plate" (Col. 1, Rows 1-4) , 1 option
249	End Loop	"4 first wells"
250	Set Variable	SAMPLE_CNT = -1
251	Drop DiTis	1-4 DiTi LiHa Waste
252	Get DiTis	Grid 32; Site: 3 (DiTi 200ul Nested MCA96) Fetch 8 rows and 12 columns
253	Mix	35 µl Helena Water free dispense "Working plate" (Col. 1, Rows 1-8)
254	Begin Loop	6 times "4 first wells"
255	Start Timer	5
256	Wait for Timer	Timer 5 : 300 sec
257	Mix	35 µl Helena Water free dispense "Working plate" (Col. 1, Rows 1-8)
258	End Loop	"4 first wells"
259	Drop DiTis	Grid 32; Site: 19 (DiTi Nested Waste)
260	Transfer Labware	Source: Grid '32,' Site '3'; Destination: Grid '32', Site '19'; Narrow (MCA96 1)
261	Transfer Labware	Source: Grid '14,' Site '2'; Destination: Grid '14', Site '3'; Narrow (MCA96 1)
262	Group End	A-tailing

263	Group	Wash after A-tailing
264	Set Variable	SAMPLE_CNT = Number of samples
265	Begin Loop	24 times "wash after a-tailing"
266	Get DiTis (1-4)	DiTi aluminium 200 ul
267	Aspirate (1-4)	30 µl Helena Water free dispense "Magnet pos 1" (Col. 1, Rows 1-4) , 1 option
268	Drop DiTis (1-4)	DiTi LiHa Waste
269	End Loop	"wash after a-tailing"
270	Set Variable	SAMPLE_CNT = -1
271	Get DiTis (1-4)	DiTi aluminium 200 ul
272	Set Variable	SAMPLE_CNT = Number of samples
273	Begin Loop	24 times "wash after a-tailing_1"
274	Aspirate (1-4)	100 µl Helena Water free dispense "PBS" (Col. 1, Rows 3-6)
275	Dispense (1-4)	100 µl Helena Water free dispense "Magnet pos 1" (Col. 1, Rows 1-4) , 1 option
276	End Loop	"wash after a-tailing_1"
277	Set Variable	SAMPLE_CNT = -1
278	Drop DiTis (1-4)	DiTi LiHa Waste
279	Transfer Labware	Source: Grid '14,' Site '3'; Destination: Grid '14', Site '4'; Narrow (MCA96 1)
280	Transfer Labware	Source: Grid '14,' Site '4'; Destination: Grid '14', Site '3'; Narrow (MCA96 1)
281	Transfer Labware	Source: Grid '14,' Site '3'; Destination: Grid '14', Site '4'; Narrow (MCA96 1)
282	Transfer Labware	Source: Grid '14,' Site '4'; Destination: Grid '14', Site '3'; Narrow (MCA96 1)
283	Transfer Labware	Source: Grid '14,' Site '3'; Destination: Grid '14', Site '4'; Narrow (MCA96 1)
284	Transfer Labware	Source: Grid '14,' Site '4'; Destination: Grid '14', Site '3'; Narrow (MCA96 1)
285	Set Variable	SAMPLE_CNT = Number of samples
286	Begin Loop	24 times "wash after a-tailing_2"

287 Get DiTis	1-4	DiTi aluminium 200 ul
288 Aspirate	1-4	100 µl Helena Water free dispense "Magnet pos 1" (Col. 1, Rows 1-4) , 1 option
289 Drop DiTis	1-4	DiTi LiHa Waste
290 End Loop		"wash after a-tailing_2"
291 Set Variable		SAMPLE_CNT = -1
292 Get DiTis	1-4	DiTi aluminium 200 ul
293 Set Variable		SAMPLE_CNT = Number of samples
294 Begin Loop		24 times "wash after a-tailing_3"
295 Aspirate	1-4	100 µl Helena Water free dispense "PBS" (Col. 1, Rows 3-6)
296 Dispense	1-4	100 µl Helena Water free dispense "Magnet pos 1" (Col. 1, Rows 1-4) , 1 option
297 End Loop		"wash after a-tailing_3"
298 Set Variable		SAMPLE_CNT = -1
299 Drop DiTis	1-4	DiTi LiHa Waste
300 Transfer Labware		Source: Grid '14,' Site '3'; Destination: Grid '14', Site '4'; Narrow (MCA96 1)
301 Transfer Labware		Source: Grid '14,' Site '4'; Destination: Grid '14', Site '3'; Narrow (MCA96 1)
302 Transfer Labware		Source: Grid '14,' Site '3'; Destination: Grid '14', Site '4'; Narrow (MCA96 1)
303 Transfer Labware		Source: Grid '14,' Site '4'; Destination: Grid '14', Site '3'; Narrow (MCA96 1)
304 Transfer Labware		Source: Grid '14,' Site '3'; Destination: Grid '14', Site '4'; Narrow (MCA96 1)
305 Transfer Labware		Source: Grid '14,' Site '4'; Destination: Grid '14', Site '3'; Narrow (MCA96 1)
306 Set Variable		SAMPLE_CNT = Number of samples
307 Begin Loop		24 times "wash after a-tailing_4"
308 Get DiTis	1-4	DiTi aluminium 200 ul
309 Aspirate	1-4	100 µl Helena Water free dispense "Magnet pos 1" (Col. 1, Rows 1-4) , 1 option
310 Drop DiTis	1-4	DiTi LiHa Waste

311	End Loop		"wash after a-tailing_4"
312	Set Variable		SAMPLE_CNT = -1
313	Transfer Labware		Source: Grid '14,' Site '3'; Destination: Grid '14', Site '2'; Narrow (MCA96 1)
314	Group End		Wash after A-tailing
315	Group		Adaptor ligation
316	Set Variable		SAMPLE_CNT = Number of samples
317	Begin Loop		24 times "adaptor ligation"
318	Get DiTis	1-4	DiTi aluminium 50ul
319	Aspirate	1-4	13 µl Helena Water free dispense "Adaptors" (Col. 1, Rows 1-4) , 1 option
320	Dispense	1-4	13 µl Water free dispense DiTi 50 "Working plate" (Col. 1, Rows 1-4) , 1 option
321	Drop DiTis	1-4	DiTi LiHa Waste
322	End Loop		"adaptor ligation"
323	Set Variable		SAMPLE_CNT = -1
324	Set Variable		SAMPLE_CNT = Number of samples
325	Begin Loop		24 times "adaptor ligation_1"
326	Get DiTis	1-4	DiTi aluminium 200 ul
327	Aspirate	1	13 µl Helena Water free dispense "Labware1" (Col. 1, Row 4)
328	Aspirate	2	13 µl Helena Water free dispense "Labware1" (Col. 1, Row 4)
329	Aspirate	3	13 µl Helena Water free dispense "Labware1" (Col. 1, Row 4)
330	Aspirate	4	13 µl Helena Water free dispense "Labware1" (Col. 1, Row 4)
331	Dispense	1-4	13 µl Water free dispense DiTi 50 "Working plate" (Col. 1, Rows 1-4) , 1 option
332	Mix	1-4	5 x 45 µl Water free dispense DiTi 50 "Working plate" (Col. 1, Rows 1-4) , 1 option
333	Drop DiTis	1-4	DiTi LiHa Waste
334	End Loop		"adaptor ligation_1"

No.	Command	Parameters
335	Set Variable	SAMPLE_CNT = -1
336	Get DiTis	Grid 32; Site: 4 (DiTi 200ul Nested MCA96)<br>Fetch 8 rows and 12 columns
337	Begin Loop	3 times "4 first wells"
338	Start Timer	6
339	Wait for Timer	Timer 6 : 300 sec
340	Mix	35 µl Helena Water free dispense<br>"Working plate" (Col. 1, Rows 1-8)
341	End Loop	"4 first wells"
342	Drop DiTis	Grid 32; Site: 19 (DiTi Nested Waste)
343	Transfer Labware	Source: Grid '32,' Site '4'; Destination: Grid '32', Site '19'; Narrow<br>(MCA96 1)
344	Transfer Labware	Source: Grid '14,' Site '2'; Destination: Grid '14', Site '3'; Narrow<br>(MCA96 1)
345	Start Timer	7
346	Wait for Timer	Timer 7 : 60 sec
347	Set Variable	SAMPLE_CNT = Number of samples
348	Begin Loop	24 times "adaptor ligation _1"
349	Get DiTis	1-4 DiTi aluminium 200 ul
350	Aspirate	1-4 26 µl Helena Water free dispense<br>"Magnet pos 1" (Col. 1, Rows 1-4) , 1 option
351	Drop DiTis	1-4 DiTi LiHa Waste
352	End Loop	"adaptor ligation _1"
353	Set Variable	SAMPLE_CNT = -1
354	Group End	Adaptor ligation
355	Group	Wash after adaptor ligering
356	Get DiTis	1-4 DiTi aluminium 200 ul
357	Set Variable	SAMPLE_CNT = Number of samples
358	Begin Loop	24 times "wash efter adaptor ligation"

359	Aspirate	1-4 — 100 µl Helena Water free dispense "IPWB2" (Col. 1, Rows 3-6)
360	Dispense	1-4 — 100 µl Helena Water free dispense "Magnet pos 1" (Col. 1, Rows 1-4) , 1 option
361	End Loop	"wash efter adaptor ligation"
362	Set Variable	SAMPLE_CNT = -1
363	Drop DiTis	1-4 — DiTi LiHa Waste
364	Transfer Labware	Source: Grid '14,' Site '3'; Destination: Grid '14', Site '4'; Narrow (MCA96 1)
365	Transfer Labware	Source: Grid '14,' Site '4'; Destination: Grid '14', Site '3'; Narrow (MCA96 1)
366	Transfer Labware	Source: Grid '14,' Site '3'; Destination: Grid '14', Site '4'; Narrow (MCA96 1)
367	Transfer Labware	Source: Grid '14,' Site '4'; Destination: Grid '14', Site '3'; Narrow (MCA96 1)
368	Start Timer	8
369	Wait for Timer	Timer 8 : 60 sec
370	Set Variable	SAMPLE_CNT = Number of samples
371	Begin Loop	24 times "wash after adaptor ligation_1"
372	Get DiTis	1-4 — DiTi aluminium 200 ul
373	Aspirate	1-4 — 100 µl Helena Water free dispense "Magnet pos 1" (Col. 1, Rows 1-4) , 1 option
374	Drop DiTis	1-4 — DiTi LiHa Waste
375	End Loop	"wash after adaptor ligation_1"
376	Set Variable	SAMPLE_CNT = -1
377	Get DiTis	1-4 — DiTi aluminium 200 ul
378	Set Variable	SAMPLE_CNT = Number of samples
379	Begin Loop	24 times "wash after adaptor ligation_2"
380	Aspirate	1-4 — 100 µl Helena Water free dispense "IPWB2" (Col. 1, Rows 3-6)
381	Dispense	1-4 — 100 µl Helena Water free dispense "Magnet pos 1" (Col. 1, Rows 1-4) , 1 option
382	End Loop	"wash after adaptor ligation_2"

383	Set Variable	SAMPLE_CNT = -1
384	Drop DiTis	1-4 DiTi LiHa Waste
385	Transfer Labware	Source: Grid '14,' Site '3'; Destination: Grid '14', Site '4'; Narrow (MCA96 1)
386	Transfer Labware	Source: Grid '14,' Site '4'; Destination: Grid '14', Site '3'; Narrow (MCA96 1)
387	Transfer Labware	Source: Grid '14,' Site '3'; Destination: Grid '14', Site '4'; Narrow (MCA96 1)
388	Transfer Labware	Source: Grid '14,' Site '4'; Destination: Grid '14', Site '3'; Narrow (MCA96 1)
389	Start Timer	9
390	Wait for Timer	Timer 9 : 60 sec
391	Set Variable	SAMPLE_CNT = Number of samples
392	Begin Loop	24 times "wash after adaptor ligation_3"
393	Get DiTis	1-4 DiTi aluminium 200 ul
394	Aspirate	1-4 100 µl Helena Water free dispense "Magnet pos 1" (Col. 1, Rows 1-4) , 1 option
395	Drop DiTis	1-4 DiTi LiHa Waste
396	End Loop	"wash after adaptor ligation_3"
397	Set Variable	SAMPLE_CNT = -1
398	Get DiTis	1-4 DiTi aluminium 200 ul
399	Set Variable	SAMPLE_CNT = Number of samples
400	Begin Loop	24 times "wash after adaptor ligation_4"
401	Aspirate	1-4 100 µl Helena Water free dispense "PBS" (Col. 1, Rows 3-6)
402	Dispense	1-4 100 µl Helena Water free dispense "Magnet pos 1" (Col. 1, Rows 1-4) , 1 option
403	End Loop	"wash after adaptor ligation_4"
404	Set Variable	SAMPLE_CNT = -1
405	Drop DiTis	1-4 DiTi LiHa Waste
406	Transfer Labware	Source: Grid '14,' Site '3'; Destination: Grid '14', Site '4'; Narrow (MCA96 1)

No.	Command	Tip	Parameters
407	Transfer Labware		Source: Grid '14,' Site '4'; Destination: Grid '14', Site '3'; Narrow (MCA96 1)
408	Transfer Labware		Source: Grid '14,' Site '3'; Destination: Grid '14', Site '4'; Narrow (MCA96 1)
409	Transfer Labware		Source: Grid '14,' Site '4'; Destination: Grid '14', Site '3'; Narrow (MCA96 1)
410	Start Timer		10
411	Wait for Timer		Timer 10 : 60 sec
412	Set Variable		SAMPLE_CNT = Number of samples
413	Begin Loop		24 times "wash after adaptor ligation_5"
414	Get DiTis	1-4	DiTi aluminium 200 ul
415	Aspirate	1-4	100 µl Helena Water free dispense "Magnet pos 1" (Col. 1, Rows 1-4) , 1 option
416	Drop DiTis	1-4	DiTi LiHa Waste
417	End Loop		"wash after adaptor ligation_5"
418	Set Variable		SAMPLE_CNT = -1
419	Group End		Wash after adaptor ligering
420	Group		Elution
421	Get DiTis	1-4	DiTi aluminium 200 ul
422	Set Variable		SAMPLE_CNT = Number of samples
423	Begin Loop		24 times "Elution"
424	Aspirate	1	50 µl Helena Water free dispense "Labware1" (Col. 1, Row 5)
425	Aspirate	2	50 µl Helena Water free dispense "Labware1" (Col. 1, Row 5)
426	Aspirate	3	50 µl Helena Water free dispense "Labware1" (Col. 1, Row 5)
427	Aspirate	4	50 µl Helena Water free dispense "Labware1" (Col. 1, Row 5)
428	Dispense	1-4	50 µl Helena Water free dispense "Magnet pos 1" (Col. 1, Rows 1-4) , 1 option
429	End Loop		"Elution"
430	Set Variable		SAMPLE_CNT = -1

431	Drop DiTis	DiTi LiHa Waste (1-4)
432	Transfer Labware	Source: Grid '14,' Site '3'; Destination: Grid '14', Site '2'; Narrow (MCA96 1)
433	User Prompt	"Add 4 µl of Proteinase K manually and put plate in the thermal cycler for reverse cross linking. " sound : once
434	Get DiTis	Grid 32; Site: 5 (DiTi 200ul Nested MCA96) Fetch 8 rows and 12 columns
435	Begin Loop	10 times "Mix loop 4 first wells"
436	Start Timer	11
437	Wait for Timer	Timer 11 : 180 sec
438	Mix	35 µl Helena Water free dispense "Working plate" (Col. 1, Rows 1-8)
439	End Loop	"Mix loop 4 first wells"
440	Drop DiTis	Grid 32; Site: 19 (DiTi Nested Waste)
441	Transfer Labware	Source: Grid '32,' Site '5'; Destination: Grid '32', Site '19'; Narrow (MCA96 1)
442	Transfer Labware	Source: Grid '14,' Site '2'; Destination: Grid '14', Site '3'; Narrow (MCA96 1)
443	User Prompt	"Put new plate on site 1 " sound : once
444	Start Timer	12
445	Wait for Timer	Timer 12 : 60 sec
446	Set Variable	SAMPLE_CNT = Number of samples
447	Begin Loop	10 times "Elution 1"
448	Get DiTis	DiTi aluminium 200 ul (1-4)
449	Aspirate	50 µl Helena Water free dispense "Magnet pos 1" (Col. 1, Rows 1-4) , 1 option (1-4)
450	Dispense	50 µl Helena Water free dispense "New plate" (Col. 1, Rows 1-4) , 1 option (1-4)
451	Drop DiTis	DiTi LiHa Waste (1-4)
452	End Loop	"Elution 1"
453	Set Variable	SAMPLE_CNT = -1

454	Group End	Elution
455	Group	Size selection + elution with EB
456	Get DiTis	1-4 DiTi aluminium 200 ul
457	Set Variable	SAMPLE_CNT = Number of samples
458	Begin Loop	24 times "size selection"
459	Mix	1 2 x 50 µl Helena Water free dispense "Labware1" (Col. 1, Row 6)
460	Aspirate	1 50 µl Helena Water free dispense "Labware1" (Col. 1, Row 6)
461	Mix	2 2 x 50 µl Helena Water free dispense "Labware1" (Col. 1, Row 6)
462	Aspirate	2 50 µl Helena Water free dispense "Labware1" (Col. 1, Row 6)
463	Mix	3 2 x 50 µl Helena Water free dispense "Labware1" (Col. 1, Row 6)
464	Aspirate	3 50 µl Helena Water free dispense "Labware1" (Col. 1, Row 6)
465	Mix	4 2 x 50 µl Helena Water free dispense "Labware1" (Col. 1, Row 6)
466	Aspirate	4 50 µl Helena Water free dispense "Labware1" (Col. 1, Row 6)
467	Dispense	1-4 50 µl Helena Water free dispense "New plate" (Col. 1, Rows 1-4) , 1 option
468	End Loop	"size selection"
469	Set Variable	SAMPLE_CNT = -1
470	Drop DiTis	1-4 DiTi LiHa Waste
471	Get DiTis	Grid 32; Site: 6 (DiTi 200ul Nested MCA96) Fetch 8 rows and 12 columns
472	Mix	50 µl Helena Water free dispense "New plate" (Col. 1, Rows 1-8)
473	Drop DiTis	Grid 32; Site: 19 (DiTi Nested Waste)
474	Start Timer	13
475	Transfer Labware	Source: Grid '32,' Site '6'; Destination: Grid '32', Site '19'; Narrow (MCA96 1)
476	Wait for Timer	Timer 13 : 300 sec
477	Transfer Labware	Source: Grid '14,' Site '3'; Destination: Grid '32', Site '19'; Narrow (MCA96 1)

478	Transfer Labware	Source: Grid '14,' Site '1'; Destination: Grid '14', Site '3'; Narrow (MCA96 1)
479	Start Timer	14
480	Wait for Timer	Timer 14 : 60 sec
481	Set Variable	SAMPLE_CNT = Number of samples
482	Begin Loop	24 times "size selection_1"
483	Get DiTis	1-4 DiTi aluminium 200 ul
484	Aspirate	1-4 50 µl Helena Water free dispense "Magnet pos 1" (Col. 1, Rows 1-4) , 1 option
485	Drop DiTis	1-4 DiTi LiHa Waste
486	End Loop	"size selection_1"
487	Set Variable	SAMPLE_CNT = -1
488	Get DiTis	1-4 DiTi aluminium 200 ul
489	Set Variable	SAMPLE_CNT = Number of samples
490	Begin Loop	24 times "size selection_wash"
491	Mix	1-4 5 x 150 µl Helena Water free dispense "70 percent EtOH" (Col. 1, Rows 3-6)
492	Aspirate	1-4 150 µl Helena Water free dispense "70 percent EtOH" (Col. 1, Rows 3-6)
493	Dispense	1-4 150 µl Helena Water free dispense "Magnet pos 1" (Col. 1, Rows 1-4) , 1 option
494	End Loop	"size selection_wash"
495	Set Variable	SAMPLE_CNT = -1
496	Drop DiTis	1-4 DiTi LiHa Waste
497	Transfer Labware	Source: Grid '14,' Site '3'; Destination: Grid '14', Site '4'; Narrow (MCA96 1)
498	Transfer Labware	Source: Grid '14,' Site '4'; Destination: Grid '14', Site '3'; Narrow (MCA96 1)
499	Transfer Labware	Source: Grid '14,' Site '3'; Destination: Grid '14', Site '4'; Narrow (MCA96 1)
500	Transfer Labware	Source: Grid '14,' Site '4'; Destination: Grid '14', Site '3'; Narrow (MCA96 1)
501	Transfer Labware	Source: Grid '14,' Site '3'; Destination: Grid '14', Site '4'; Narrow (MCA96 1)

502	Transfer Labware	Source: Grid '14,' Site '4'; Destination: Grid '14', Site '3'; Narrow (MCA96 1)
503	Set Variable	SAMPLE_CNT = Number of samples
504	Begin Loop	24 times "size selection_wash_2"
505	Get DiTis	1-4 DiTi aluminium 200 ul
506	Aspirate	1-4 150 µl Helena Water free dispense "Magnet pos 1" (Col. 1, Rows 1-4) , 1 option
507	Drop DiTis	1-4 DiTi LiHa Waste
508	End Loop	"size selection_wash_2"
509	Set Variable	SAMPLE_CNT = -1
510	Get DiTis	1-4 DiTi aluminium 200 ul
511	Mix	1-4 5 x 150 µl Helena Water free dispense "70 percent EtOH" (Col. 1, Rows 3-6)
512	Set Variable	SAMPLE_CNT = Number of samples
513	Begin Loop	24 times "size selection_wash_3"
514	Aspirate	1-4 150 µl Helena Water free dispense "70 percent EtOH" (Col. 1, Rows 3-6)
515	Dispense	1-4 150 µl Helena Water free dispense "Magnet pos 1" (Col. 1, Rows 1-4) , 1 option
516	End Loop	"size selection_wash_3"
517	Set Variable	SAMPLE_CNT = -1
518	Drop DiTis	1-4 DiTi LiHa Waste
519	Transfer Labware	Source: Grid '14,' Site '3'; Destination: Grid '14', Site '4'; Narrow (MCA96 1)
520	Transfer Labware	Source: Grid '14,' Site '4'; Destination: Grid '14', Site '3'; Narrow (MCA96 1)
521	Transfer Labware	Source: Grid '14,' Site '3'; Destination: Grid '14', Site '4'; Narrow (MCA96 1)
522	Transfer Labware	Source: Grid '14,' Site '4'; Destination: Grid '14', Site '3'; Narrow (MCA96 1)
523	Transfer Labware	Source: Grid '14,' Site '3'; Destination: Grid '14', Site '4'; Narrow (MCA96 1)
524	Transfer Labware	Source: Grid '14,' Site '4'; Destination: Grid '14', Site '3'; Narrow (MCA96 1)
525	Set Variable	SAMPLE_CNT = Number of samples

Step	Command	Tips	Details
526	Begin Loop		24 times "size selection_wash_4"
527	Get DiTis	1-4	DiTi aluminium 200 ul
528	Aspirate	1-4	150 µl Helena Water free dispense "Magnet pos 1" (Col. 1, Rows 1-4) , 1 option
529	Drop DiTis	1-4	DiTi LiHa Waste
530	End Loop		"size selection_wash_4"
531	Set Variable		SAMPLE_CNT = -1
532	Start Timer		15
533	Wait for Timer		Timer 15 : 300 sec
534	Get DiTis	1-4	DiTi aluminium 200 ul
535	Set Variable		SAMPLE_CNT = Number of samples
536	Begin Loop		24 times "EB"
537	Aspirate	1	30 µl Helena Water free dispense "Labware1" (Col. 1, Row 7)
538	Aspirate	2	30 µl Helena Water free dispense "Labware1" (Col. 1, Row 7)
539	Aspirate	3	30 µl Helena Water free dispense "Labware1" (Col. 1, Row 7)
540	Aspirate	4	30 µl Helena Water free dispense "Labware1" (Col. 1, Row 7)
541	Dispense	1-4	30 µl Helena Water free dispense "Magnet pos 1" (Col. 1, Rows 1-4) , 1 option
542	End Loop		"EB"
543	Set Variable		SAMPLE_CNT = -1
544	Drop DiTis	1-4	DiTi LiHa Waste
545	Transfer Labware		Source: Grid '14,' Site '3'; Destination: Grid '14', Site '2'; Narrow (MCA96 1)
546	Set Variable		SAMPLE_CNT = Number of samples
547	Begin Loop		24 times "EB_2"
548	Get DiTis	1-4	DiTi aluminium 200 ul
549	Mix	1-4	20 x 30 µl Helena Water free dispense "Working plate" (Col. 1, Rows 1-4) , 1 option

Drop DiTis 550	1-4	DiTi LiHa Waste
End Loop 551		"EB_2"
Set Variable 552		SAMPLE_CNT = -1
Start Timer 553		16
Wait for Timer 554		Timer 16 : 150 sec
Set Variable 555		SAMPLE_CNT = Number of samples
Begin Loop 556		24 times "EB_2"
Get DiTis 557	1-4	DiTi aluminium 200 ul
Mix 558	1-4	5 x 30 µl Helena Water free dispense "Working plate" (Col. 1, Rows 1-4) , 1 option
Drop DiTis 559	1-4	DiTi LiHa Waste
End Loop 560		"EB_2"
Set Variable 561		SAMPLE_CNT = -1
Start Timer 562		17
Wait for Timer 563		Timer 17 : 150 sec
Set Variable 564		SAMPLE_CNT = Number of samples
Begin Loop 565		24 times "EB_2_"
Get DiTis 566	1-4	DiTi aluminium 200 ul
Mix 567	1-4	5 x 30 µl Helena Water free dispense "Working plate" (Col. 1, Rows 1-4) , 1 option
Drop DiTis 568	1-4	DiTi LiHa Waste
End Loop 569		"EB_2_"
Set Variable 570		SAMPLE_CNT = -1
Transfer Labware 571		Source: Grid '14,' Site '2'; Destination: Grid '14', Site '3'; Narrow (MCA96 1)
Start Timer 572		17
Wait for Timer 573		Timer 17 : 60 sec

574	Group End		Size selection + elution with EB
575	Group		PCR
576	User Prompt		"Put new plate on site 1<br>"<br>sound : three times
577	Set Variable		SAMPLE_CNT = Number of samples
578	Begin Loop		24 times "PCR"
579	Get DiTis	1-4	DiTi aluminium 50ul
580	Aspirate	1-4	20 µl Helena Water free dispense<br>"Magnet pos 1" (Col. 1, Rows 1-4) , 1 option
581	Dispense	1-4	20 µl Helena Water free dispense<br>"New plate" (Col. 1, Rows 1-4) , 1 option
582	Drop DiTis	1-4	DiTi LiHa Waste
583	End Loop		"PCR"
584	Set Variable		SAMPLE_CNT = -1
585	Get DiTis	1-4	DiTi aluminium 200 ul
586	Set Variable		SAMPLE_CNT = Number of samples
587	Begin Loop		24 times "pcr"
588	Aspirate	1	6.6 µl Helena Water free dispense<br>"Labware1" (Col. 1, Row 8)
589	Aspirate	2	6.6 µl Helena Water free dispense<br>"Labware1" (Col. 1, Row 8)
590	Aspirate	3	6.6 µl Helena Water free dispense<br>"Labware1" (Col. 1, Row 8)
591	Aspirate	4	6.6 µl Helena Water free dispense<br>"Labware1" (Col. 1, Row 8)
592	Dispense	1-4	6.6 µl Water Detect 500µL<br>"New plate" (Col. 1, Rows 1-4) , 1 option
593	End Loop		"pcr"
594	Set Variable		SAMPLE_CNT = -1
595	Drop DiTis	1-4	DiTi LiHa Waste
596	Group End		PCR

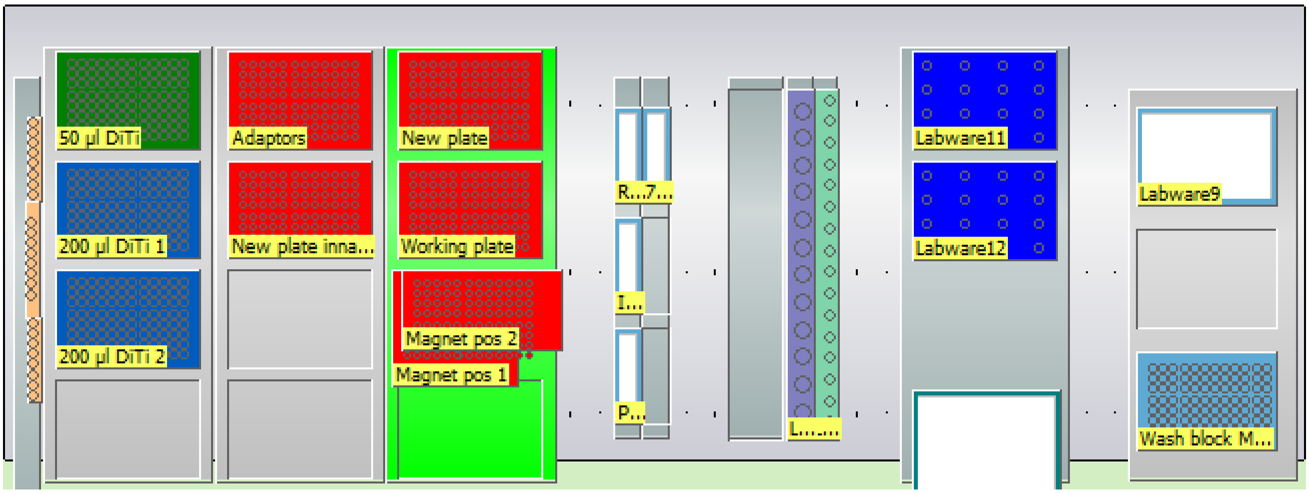
50 µl DiTi
200 µl DiTi 1
200 µl DiTi 2
Adaptors
New plate inna...
New plate
Working plate
Magnet pos 2
Magnet pos 1
R...7...
I...
P...
L...
Labware11
Labware12
Labware9
Wash block M...
