# Supplementary material for: lobChIP: from cells to sequencing ready ChIP libraries in a single day
Source: Epigenetics Chromatin. 2015 Jul 21;8:25. doi: 10.1186/s13072-015-0017-5 (PMC4507313; doi:10.1186/s13072-015-0017-5)
Supplement: Additional file 4. — A list of lobChIP peak calls used in the manuscript. For each peak the position and height of the summit as calculated by MACS is given, followed by the file name for the corresponding ChIP experiment. [file 13072_2015_17_MOESM4_ESM.docx]

```
Additional file 4. List of peaks used in the manuscript. This file has all the MACS peak calls that remained after filtering, with the file name added after each peak.

chr1	10083	10084	MACS_peak_1	9.00	TCF7L2_standard_ChIP_seq_CW34
chr1	825162	825163	MACS_peak_2	8.00	TCF7L2_standard_ChIP_seq_CW34
chr1	1060790	1060791	MACS_peak_3	27.00	TCF7L2_standard_ChIP_seq_CW34
chr1	1624327	1624328	MACS_peak_4	9.00	TCF7L2_standard_ChIP_seq_CW34
chr1	1677599	1677600	MACS_peak_5	28.00	TCF7L2_standard_ChIP_seq_CW34
chr1	1821782	1821783	MACS_peak_6	10.00	TCF7L2_standard_ChIP_seq_CW34
chr1	2542053	2542054	MACS_peak_7	7.00	TCF7L2_standard_ChIP_seq_CW34
chr1	3208953	3208954	MACS_peak_10	20.00	TCF7L2_standard_ChIP_seq_CW34
chr1	3209942	3209943	MACS_peak_11	14.00	TCF7L2_standard_ChIP_seq_CW34
chr1	3237280	3237281	MACS_peak_12	21.00	TCF7L2_standard_ChIP_seq_CW34
chr1	3251839	3251840	MACS_peak_13	8.00	TCF7L2_standard_ChIP_seq_CW34
chr1	4003050	4003051	MACS_peak_14	14.00	TCF7L2_standard_ChIP_seq_CW34
chr1	4327586	4327587	MACS_peak_15	40.00	TCF7L2_standard_ChIP_seq_CW34
chr1	4771068	4771069	MACS_peak_16	13.00	TCF7L2_standard_ChIP_seq_CW34
chr1	6015646	6015647	MACS_peak_17	8.00	TCF7L2_standard_ChIP_seq_CW34
chr1	6052203	6052204	MACS_peak_18	12.00	TCF7L2_standard_ChIP_seq_CW34
chr1	6064085	6064086	MACS_peak_19	8.00	TCF7L2_standard_ChIP_seq_CW34
chr1	6320226	6320227	MACS_peak_20	11.00	TCF7L2_standard_ChIP_seq_CW34
chr1	8250381	8250382	MACS_peak_21	9.00	TCF7L2_standard_ChIP_seq_CW34
chr1	8272142	8272143	MACS_peak_22	6.00	TCF7L2_standard_ChIP_seq_CW34
chr1	8319399	8319400	MACS_peak_23	16.00	TCF7L2_standard_ChIP_seq_CW34
chr1	8321046	8321047	MACS_peak_24	17.00	TCF7L2_standard_ChIP_seq_CW34
chr1	8612361	8612362	MACS_peak_25	5.00	TCF7L2_standard_ChIP_seq_CW34
chr1	8624267	8624268	MACS_peak_26	24.00	TCF7L2_standard_ChIP_seq_CW34
chr1	8631821	8631822	MACS_peak_27	11.00	TCF7L2_standard_ChIP_seq_CW34
chr1	8772305	8772306	MACS_peak_28	7.00	TCF7L2_standard_ChIP_seq_CW34
chr1	8802184	8802185	MACS_peak_29	19.00	TCF7L2_standard_ChIP_seq_CW34
chr1	9459840	9459841	MACS_peak_30	17.00	TCF7L2_standard_ChIP_seq_CW34
chr1	9610433	9610434	MACS_peak_31	9.00	TCF7L2_standard_ChIP_seq_CW34
chr1	9625266	9625267	MACS_peak_32	12.00	TCF7L2_standard_ChIP_seq_CW34
chr1	10490588	10490589	MACS_peak_33	9.00	TCF7L2_standard_ChIP_seq_CW34
chr1	10728438	10728439	MACS_peak_34	7.00	TCF7L2_standard_ChIP_seq_CW34
chr1	10764533	10764534	MACS_peak_35	14.00	TCF7L2_standard_ChIP_seq_CW34
chr1	10961009	10961010	MACS_peak_36	5.00	TCF7L2_standard_ChIP_seq_CW34
chr1	10997628	10997629	MACS_peak_37	84.00	TCF7L2_standard_ChIP_seq_CW34
chr1	11107323	11107324	MACS_peak_38	8.00	TCF7L2_standard_ChIP_seq_CW34
chr1	11539079	11539080	MACS_peak_39	18.00	TCF7L2_standard_ChIP_seq_CW34
chr1	12462466	12462467	MACS_peak_40	16.00	TCF7L2_standard_ChIP_seq_CW34
chr1	12714842	12714843	MACS_peak_41	13.00	TCF7L2_standard_ChIP_seq_CW34
chr1	13977946	13977947	MACS_peak_42	11.00	TCF7L2_standard_ChIP_seq_CW34
chr1	14048441	14048442	MACS_peak_43	5.00	TCF7L2_standard_ChIP_seq_CW34
chr1	15279332	15279333	MACS_peak_44	10.00	TCF7L2_standard_ChIP_seq_CW34
chr1	15466451	15466452	MACS_peak_45	14.00	TCF7L2_standard_ChIP_seq_CW34
chr1	15479083	15479084	MACS_peak_46	25.00	TCF7L2_standard_ChIP_seq_CW34
chr1	16126859	16126860	MACS_peak_47	15.00	TCF7L2_standard_ChIP_seq_CW34
chr1	16161401	16161402	MACS_peak_48	33.00	TCF7L2_standard_ChIP_seq_CW34
chr1	16330610	16330611	MACS_peak_49	24.00	TCF7L2_standard_ChIP_seq_CW34
chr1	16470589	16470590	MACS_peak_50	31.00	TCF7L2_standard_ChIP_seq_CW34
chr1	16693737	16693738	MACS_peak_51	10.00	TCF7L2_standard_ChIP_seq_CW34
chr1	17201901	17201902	MACS_peak_52	7.00	TCF7L2_standard_ChIP_seq_CW34
chr1	18825772	18825773	MACS_peak_53	6.00	TCF7L2_standard_ChIP_seq_CW34
chr1	18970215	18970216	MACS_peak_54	15.00	TCF7L2_standard_ChIP_seq_CW34
chr1	18971887	18971888	MACS_peak_55	13.00	TCF7L2_standard_ChIP_seq_CW34
chr1	19379114	19379115	MACS_peak_56	11.00	TCF7L2_standard_ChIP_seq_CW34
chr1	19736435	19736436	MACS_peak_57	11.00	TCF7L2_standard_ChIP_seq_CW34
chr1	19756939	19756940	MACS_peak_58	7.00	TCF7L2_standard_ChIP_seq_CW34
chr1	19946568	19946569	MACS_peak_59	8.00	TCF7L2_standard_ChIP_seq_CW34
chr1	20253240	20253241	MACS_peak_60	10.00	TCF7L2_standard_ChIP_seq_CW34
chr1	20301646	20301647	MACS_peak_61	13.00	TCF7L2_standard_ChIP_seq_CW34
chr1	20878801	20878802	MACS_peak_62	7.00	TCF7L2_standard_ChIP_seq_CW34
chr1	21864815	21864816	MACS_peak_63	10.00	TCF7L2_standard_ChIP_seq_CW34
chr1	22102746	22102747	MACS_peak_64	13.00	TCF7L2_standard_ChIP_seq_CW34
chr1	22249316	22249317	MACS_peak_65	38.00	TCF7L2_standard_ChIP_seq_CW34
chr1	22778637	22778638	MACS_peak_66	6.00	TCF7L2_standard_ChIP_seq_CW34
chr1	23072680	23072681	MACS_peak_67	12.00	TCF7L2_standard_ChIP_seq_CW34
chr1	23550084	23550085	MACS_peak_68	17.00	TCF7L2_standard_ChIP_seq_CW34
chr1	24136394	24136395	MACS_peak_69	25.00	TCF7L2_standard_ChIP_seq_CW34
chr1	24894650	24894651	MACS_peak_70	8.00	TCF7L2_standard_ChIP_seq_CW34
chr1	25453298	25453299	MACS_peak_71	9.00	TCF7L2_standard_ChIP_seq_CW34
chr1	25494196	25494197	MACS_peak_72	16.00	TCF7L2_standard_ChIP_seq_CW34
chr1	26017451	26017452	MACS_peak_73	6.00	TCF7L2_standard_ChIP_seq_CW34
chr1	26188131	26188132	MACS_peak_74	8.00	TCF7L2_standard_ChIP_seq_CW34
chr1	26202008	26202009	MACS_peak_75	17.00	TCF7L2_standard_ChIP_seq_CW34
chr1	26677458	26677459	MACS_peak_76	17.00	TCF7L2_standard_ChIP_seq_CW34
chr1	27022005	27022006	MACS_peak_77	7.00	TCF7L2_standard_ChIP_seq_CW34
chr1	27240920	27240921	MACS_peak_78	8.00	TCF7L2_standard_ChIP_seq_CW34
chr1	27330731	27330732	MACS_peak_79	9.00	TCF7L2_standard_ChIP_seq_CW34
chr1	27831327	27831328	MACS_peak_80	13.00	TCF7L2_standard_ChIP_seq_CW34
chr1	27933963	27933964	MACS_peak_81	39.00	TCF7L2_standard_ChIP_seq_CW34
chr1	28269978	28269979	MACS_peak_82	13.00	TCF7L2_standard_ChIP_seq_CW34
chr1	30852331	30852332	MACS_peak_83	10.00	TCF7L2_standard_ChIP_seq_CW34
chr1	31537103	31537104	MACS_peak_84	11.00	TCF7L2_standard_ChIP_seq_CW34
chr1	31922798	31922799	MACS_peak_85	13.00	TCF7L2_standard_ChIP_seq_CW34
chr1	32041586	32041587	MACS_peak_86	10.00	TCF7L2_standard_ChIP_seq_CW34
chr1	32573817	32573818	MACS_peak_87	6.00	TCF7L2_standard_ChIP_seq_CW34
chr1	32801411	32801412	MACS_peak_88	9.00	TCF7L2_standard_ChIP_seq_CW34
chr1	33226519	33226520	MACS_peak_89	8.00	TCF7L2_standard_ChIP_seq_CW34
chr1	35734509	35734510	MACS_peak_90	10.00	TCF7L2_standard_ChIP_seq_CW34
chr1	36952745	36952746	MACS_peak_91	8.00	TCF7L2_standard_ChIP_seq_CW34
chr1	38324866	38324867	MACS_peak_92	8.00	TCF7L2_standard_ChIP_seq_CW34
chr1	39284187	39284188	MACS_peak_93	7.00	TCF7L2_standard_ChIP_seq_CW34
chr1	39402811	39402812	MACS_peak_94	8.00	TCF7L2_standard_ChIP_seq_CW34
chr1	39434927	39434928	MACS_peak_95	31.00	TCF7L2_standard_ChIP_seq_CW34
chr1	39456991	39456992	MACS_peak_96	6.00	TCF7L2_standard_ChIP_seq_CW34
chr1	39570784	39570785	MACS_peak_97	6.00	TCF7L2_standard_ChIP_seq_CW34
chr1	39680057	39680058	MACS_peak_98	10.00	TCF7L2_standard_ChIP_seq_CW34
chr1	39853330	39853331	MACS_peak_99	11.00	TCF7L2_standard_ChIP_seq_CW34
chr1	40506454	40506455	MACS_peak_100	6.00	TCF7L2_standard_ChIP_seq_CW34
chr1	41705799	41705800	MACS_peak_101	13.00	TCF7L2_standard_ChIP_seq_CW34
chr1	43474639	43474640	MACS_peak_102	18.00	TCF7L2_standard_ChIP_seq_CW34
chr1	43501818	43501819	MACS_peak_103	20.00	TCF7L2_standard_ChIP_seq_CW34
chr1	44223198	44223199	MACS_peak_104	7.00	TCF7L2_standard_ChIP_seq_CW34
chr1	45147853	45147854	MACS_peak_105	7.00	TCF7L2_standard_ChIP_seq_CW34
chr1	45167048	45167049	MACS_peak_106	18.00	TCF7L2_standard_ChIP_seq_CW34
chr1	45293150	45293151	MACS_peak_107	15.00	TCF7L2_standard_ChIP_seq_CW34
chr1	45637843	45637844	MACS_peak_108	12.00	TCF7L2_standard_ChIP_seq_CW34
chr1	45769848	45769849	MACS_peak_109	28.00	TCF7L2_standard_ChIP_seq_CW34
chr1	46445080	46445081	MACS_peak_110	7.00	TCF7L2_standard_ChIP_seq_CW34
chr1	46598447	46598448	MACS_peak_111	8.00	TCF7L2_standard_ChIP_seq_CW34
chr1	46932295	46932296	MACS_peak_112	18.00	TCF7L2_standard_ChIP_seq_CW34
chr1	47799925	47799926	MACS_peak_113	7.00	TCF7L2_standard_ChIP_seq_CW34
chr1	47845419	47845420	MACS_peak_114	7.00	TCF7L2_standard_ChIP_seq_CW34
chr1	47943972	47943973	MACS_peak_115	9.00	TCF7L2_standard_ChIP_seq_CW34
chr1	48175181	48175182	MACS_peak_116	9.00	TCF7L2_standard_ChIP_seq_CW34
chr1	50993492	50993493	MACS_peak_117	10.00	TCF7L2_standard_ChIP_seq_CW34
chr1	51337401	51337402	MACS_peak_118	10.00	TCF7L2_standard_ChIP_seq_CW34
chr1	51443331	51443332	MACS_peak_119	6.00	TCF7L2_standard_ChIP_seq_CW34
chr1	52156525	52156526	MACS_peak_120	12.00	TCF7L2_standard_ChIP_seq_CW34
chr1	52503315	52503316	MACS_peak_121	12.00	TCF7L2_standard_ChIP_seq_CW34
chr1	52729239	52729240	MACS_peak_122	5.00	TCF7L2_standard_ChIP_seq_CW34
chr1	53704156	53704157	MACS_peak_123	9.00	TCF7L2_standard_ChIP_seq_CW34
chr1	54954086	54954087	MACS_peak_124	10.00	TCF7L2_standard_ChIP_seq_CW34
chr1	55007772	55007773	MACS_peak_125	6.00	TCF7L2_standard_ChIP_seq_CW34
chr1	55354286	55354287	MACS_peak_126	12.00	TCF7L2_standard_ChIP_seq_CW34
chr1	55894964	55894965	MACS_peak_127	24.00	TCF7L2_standard_ChIP_seq_CW34
chr1	55996656	55996657	MACS_peak_128	7.00	TCF7L2_standard_ChIP_seq_CW34
chr1	56655855	56655856	MACS_peak_129	25.00	TCF7L2_standard_ChIP_seq_CW34
chr1	56902820	56902821	MACS_peak_130	33.00	TCF7L2_standard_ChIP_seq_CW34
chr1	56914755	56914756	MACS_peak_131	9.00	TCF7L2_standard_ChIP_seq_CW34
chr1	56927248	56927249	MACS_peak_132	5.00	TCF7L2_standard_ChIP_seq_CW34
chr1	56960221	56960222	MACS_peak_133	21.00	TCF7L2_standard_ChIP_seq_CW34
chr1	57001908	57001909	MACS_peak_134	6.00	TCF7L2_standard_ChIP_seq_CW34
chr1	57154440	57154441	MACS_peak_135	8.00	TCF7L2_standard_ChIP_seq_CW34
chr1	57320502	57320503	MACS_peak_136	6.00	TCF7L2_standard_ChIP_seq_CW34
chr1	57385595	57385596	MACS_peak_137	17.00	TCF7L2_standard_ChIP_seq_CW34
chr1	57451809	57451810	MACS_peak_138	9.00	TCF7L2_standard_ChIP_seq_CW34
chr1	57746004	57746005	MACS_peak_139	40.00	TCF7L2_standard_ChIP_seq_CW34
chr1	57930606	57930607	MACS_peak_140	11.00	TCF7L2_standard_ChIP_seq_CW34
chr1	58555730	58555731	MACS_peak_141	34.00	TCF7L2_standard_ChIP_seq_CW34
chr1	59351199	59351200	MACS_peak_142	38.00	TCF7L2_standard_ChIP_seq_CW34
chr1	59518092	59518093	MACS_peak_143	32.00	TCF7L2_standard_ChIP_seq_CW34
chr1	59680594	59680595	MACS_peak_144	9.00	TCF7L2_standard_ChIP_seq_CW34
chr1	59812210	59812211	MACS_peak_145	8.00	TCF7L2_standard_ChIP_seq_CW34
chr1	59874453	59874454	MACS_peak_146	10.00	TCF7L2_standard_ChIP_seq_CW34
chr1	60022687	60022688	MACS_peak_147	36.00	TCF7L2_standard_ChIP_seq_CW34
chr1	60180088	60180089	MACS_peak_148	10.00	TCF7L2_standard_ChIP_seq_CW34
chr1	60196890	60196891	MACS_peak_149	9.00	TCF7L2_standard_ChIP_seq_CW34
chr1	61516433	61516434	MACS_peak_150	9.00	TCF7L2_standard_ChIP_seq_CW34
chr1	62208750	62208751	MACS_peak_151	8.00	TCF7L2_standard_ChIP_seq_CW34
chr1	62380700	62380701	MACS_peak_152	11.00	TCF7L2_standard_ChIP_seq_CW34
chr1	63358026	63358027	MACS_peak_153	11.00	TCF7L2_standard_ChIP_seq_CW34
chr1	63989013	63989014	MACS_peak_154	6.00	TCF7L2_standard_ChIP_seq_CW34
chr1	65061935	65061936	MACS_peak_155	7.00	TCF7L2_standard_ChIP_seq_CW34
chr1	65453342	65453343	MACS_peak_156	15.00	TCF7L2_standard_ChIP_seq_CW34
chr1	68000433	68000434	MACS_peak_157	28.00	TCF7L2_standard_ChIP_seq_CW34
chr1	68166565	68166566	MACS_peak_158	10.00	TCF7L2_standard_ChIP_seq_CW34
chr1	72948797	72948798	MACS_peak_159	9.00	TCF7L2_standard_ChIP_seq_CW34
chr1	77204133	77204134	MACS_peak_160	19.00	TCF7L2_standard_ChIP_seq_CW34
chr1	78726315	78726316	MACS_peak_161	5.00	TCF7L2_standard_ChIP_seq_CW34
chr1	78786974	78786975	MACS_peak_162	4.00	TCF7L2_standard_ChIP_seq_CW34
chr1	82936476	82936477	MACS_peak_163	6.00	TCF7L2_standard_ChIP_seq_CW34
chr1	85794949	85794950	MACS_peak_164	5.00	TCF7L2_standard_ChIP_seq_CW34
chr1	87240440	87240441	MACS_peak_165	49.00	TCF7L2_standard_ChIP_seq_CW34
chr1	87258860	87258861	MACS_peak_166	20.00	TCF7L2_standard_ChIP_seq_CW34
chr1	87289637	87289638	MACS_peak_167	14.00	TCF7L2_standard_ChIP_seq_CW34
chr1	88108316	88108317	MACS_peak_168	9.00	TCF7L2_standard_ChIP_seq_CW34
chr1	88641635	88641636	MACS_peak_169	15.00	TCF7L2_standard_ChIP_seq_CW34
chr1	90286829	90286830	MACS_peak_170	6.00	TCF7L2_standard_ChIP_seq_CW34
chr1	90461047	90461048	MACS_peak_171	8.00	TCF7L2_standard_ChIP_seq_CW34
chr1	91382432	91382433	MACS_peak_172	5.00	TCF7L2_standard_ChIP_seq_CW34
chr1	92292777	92292778	MACS_peak_174	24.00	TCF7L2_standard_ChIP_seq_CW34
chr1	93416792	93416793	MACS_peak_175	17.00	TCF7L2_standard_ChIP_seq_CW34
chr1	93544725	93544726	MACS_peak_176	20.00	TCF7L2_standard_ChIP_seq_CW34
chr1	93666481	93666482	MACS_peak_177	8.00	TCF7L2_standard_ChIP_seq_CW34
chr1	93694466	93694467	MACS_peak_178	8.00	TCF7L2_standard_ChIP_seq_CW34
chr1	93914250	93914251	MACS_peak_179	8.00	TCF7L2_standard_ChIP_seq_CW34
chr1	94311987	94311988	MACS_peak_180	7.00	TCF7L2_standard_ChIP_seq_CW34
chr1	94496914	94496915	MACS_peak_181	9.00	TCF7L2_standard_ChIP_seq_CW34
chr1	94891504	94891505	MACS_peak_182	8.00	TCF7L2_standard_ChIP_seq_CW34
chr1	95427382	95427383	MACS_peak_183	9.00	TCF7L2_standard_ChIP_seq_CW34
chr1	95479763	95479764	MACS_peak_184	8.00	TCF7L2_standard_ChIP_seq_CW34
chr1	95532791	95532792	MACS_peak_185	7.00	TCF7L2_standard_ChIP_seq_CW34
chr1	95587973	95587974	MACS_peak_186	7.00	TCF7L2_standard_ChIP_seq_CW34
chr1	95699495	95699496	MACS_peak_187	8.00	TCF7L2_standard_ChIP_seq_CW34
chr1	95850307	95850308	MACS_peak_188	6.00	TCF7L2_standard_ChIP_seq_CW34
chr1	97097713	97097714	MACS_peak_189	17.00	TCF7L2_standard_ChIP_seq_CW34
chr1	98924257	98924258	MACS_peak_190	9.00	TCF7L2_standard_ChIP_seq_CW34
chr1	100224699	100224700	MACS_peak_191	15.00	TCF7L2_standard_ChIP_seq_CW34
chr1	101469841	101469842	MACS_peak_192	13.00	TCF7L2_standard_ChIP_seq_CW34
chr1	101723741	101723742	MACS_peak_193	23.00	TCF7L2_standard_ChIP_seq_CW34
chr1	101775329	101775330	MACS_peak_194	7.00	TCF7L2_standard_ChIP_seq_CW34
chr1	106093359	106093360	MACS_peak_195	6.00	TCF7L2_standard_ChIP_seq_CW34
chr1	108069578	108069579	MACS_peak_196	23.00	TCF7L2_standard_ChIP_seq_CW34
chr1	108140101	108140102	MACS_peak_197	11.00	TCF7L2_standard_ChIP_seq_CW34
chr1	108223804	108223805	MACS_peak_198	11.00	TCF7L2_standard_ChIP_seq_CW34
chr1	109842135	109842136	MACS_peak_199	7.00	TCF7L2_standard_ChIP_seq_CW34
chr1	110491424	110491425	MACS_peak_200	6.00	TCF7L2_standard_ChIP_seq_CW34
chr1	110908794	110908795	MACS_peak_201	6.00	TCF7L2_standard_ChIP_seq_CW34
chr1	112102629	112102630	MACS_peak_202	17.00	TCF7L2_standard_ChIP_seq_CW34
chr1	112298536	112298537	MACS_peak_203	10.00	TCF7L2_standard_ChIP_seq_CW34
chr1	112519675	112519676	MACS_peak_204	11.00	TCF7L2_standard_ChIP_seq_CW34
chr1	113499945	113499946	MACS_peak_205	8.00	TCF7L2_standard_ChIP_seq_CW34
chr1	113547091	113547092	MACS_peak_206	11.00	TCF7L2_standard_ChIP_seq_CW34
chr1	114051451	114051452	MACS_peak_207	13.00	TCF7L2_standard_ChIP_seq_CW34
chr1	114472393	114472394	MACS_peak_208	7.00	TCF7L2_standard_ChIP_seq_CW34
chr1	115010266	115010267	MACS_peak_209	26.00	TCF7L2_standard_ChIP_seq_CW34
chr1	116254017	116254018	MACS_peak_210	13.00	TCF7L2_standard_ChIP_seq_CW34
chr1	117453298	117453299	MACS_peak_211	11.00	TCF7L2_standard_ChIP_seq_CW34
chr1	117459773	117459774	MACS_peak_212	7.00	TCF7L2_standard_ChIP_seq_CW34
chr1	118087549	118087550	MACS_peak_213	10.00	TCF7L2_standard_ChIP_seq_CW34
chr1	119116533	119116534	MACS_peak_214	6.00	TCF7L2_standard_ChIP_seq_CW34
chr1	119503424	119503425	MACS_peak_215	14.00	TCF7L2_standard_ChIP_seq_CW34
chr1	121138226	121138227	MACS_peak_216	7.00	TCF7L2_standard_ChIP_seq_CW34
chr1	121267336	121267337	MACS_peak_217	7.00	TCF7L2_standard_ChIP_seq_CW34
chr1	121328344	121328345	MACS_peak_218	10.00	TCF7L2_standard_ChIP_seq_CW34
chr1	142781917	142781918	MACS_peak_225	8.00	TCF7L2_standard_ChIP_seq_CW34
chr1	143251303	143251304	MACS_peak_226	6.00	TCF7L2_standard_ChIP_seq_CW34
chr1	143915381	143915382	MACS_peak_229	5.00	TCF7L2_standard_ChIP_seq_CW34
chr1	145209029	145209030	MACS_peak_230	10.00	TCF7L2_standard_ChIP_seq_CW34
chr1	145390961	145390962	MACS_peak_231	22.00	TCF7L2_standard_ChIP_seq_CW34
chr1	145743209	145743210	MACS_peak_232	9.00	TCF7L2_standard_ChIP_seq_CW34
chr1	147280295	147280296	MACS_peak_233	60.00	TCF7L2_standard_ChIP_seq_CW34
chr1	148009665	148009666	MACS_peak_234	8.00	TCF7L2_standard_ChIP_seq_CW34
chr1	148864532	148864533	MACS_peak_235	7.00	TCF7L2_standard_ChIP_seq_CW34
chr1	150039261	150039262	MACS_peak_236	5.00	TCF7L2_standard_ChIP_seq_CW34
chr1	150488080	150488081	MACS_peak_237	26.00	TCF7L2_standard_ChIP_seq_CW34
chr1	150848817	150848818	MACS_peak_238	9.00	TCF7L2_standard_ChIP_seq_CW34
chr1	150944134	150944135	MACS_peak_239	16.00	TCF7L2_standard_ChIP_seq_CW34
chr1	151042192	151042193	MACS_peak_240	7.00	TCF7L2_standard_ChIP_seq_CW34
chr1	151171579	151171580	MACS_peak_241	12.00	TCF7L2_standard_ChIP_seq_CW34
chr1	151238243	151238244	MACS_peak_242	8.00	TCF7L2_standard_ChIP_seq_CW34
chr1	153963131	153963132	MACS_peak_243	15.00	TCF7L2_standard_ChIP_seq_CW34
chr1	154245235	154245236	MACS_peak_244	11.00	TCF7L2_standard_ChIP_seq_CW34
chr1	154307026	154307027	MACS_peak_245	13.00	TCF7L2_standard_ChIP_seq_CW34
chr1	154444791	154444792	MACS_peak_246	7.00	TCF7L2_standard_ChIP_seq_CW34
chr1	155099801	155099802	MACS_peak_247	10.00	TCF7L2_standard_ChIP_seq_CW34
chr1	155221086	155221087	MACS_peak_248	9.00	TCF7L2_standard_ChIP_seq_CW34
chr1	155369848	155369849	MACS_peak_249	7.00	TCF7L2_standard_ChIP_seq_CW34
chr1	155532871	155532872	MACS_peak_250	9.00	TCF7L2_standard_ChIP_seq_CW34
chr1	155715388	155715389	MACS_peak_251	6.00	TCF7L2_standard_ChIP_seq_CW34
chr1	155990835	155990836	MACS_peak_252	17.00	TCF7L2_standard_ChIP_seq_CW34
chr1	156056210	156056211	MACS_peak_253	12.00	TCF7L2_standard_ChIP_seq_CW34
chr1	156154100	156154101	MACS_peak_254	8.00	TCF7L2_standard_ChIP_seq_CW34
chr1	156341856	156341857	MACS_peak_256	58.00	TCF7L2_standard_ChIP_seq_CW34
chr1	156676330	156676331	MACS_peak_257	7.00	TCF7L2_standard_ChIP_seq_CW34
chr1	156764464	156764465	MACS_peak_258	8.00	TCF7L2_standard_ChIP_seq_CW34
chr1	157210582	157210583	MACS_peak_259	13.00	TCF7L2_standard_ChIP_seq_CW34
chr1	160023628	160023629	MACS_peak_260	16.00	TCF7L2_standard_ChIP_seq_CW34
chr1	160948455	160948456	MACS_peak_261	7.00	TCF7L2_standard_ChIP_seq_CW34
chr1	161194395	161194396	MACS_peak_262	6.00	TCF7L2_standard_ChIP_seq_CW34
chr1	162061860	162061861	MACS_peak_263	8.00	TCF7L2_standard_ChIP_seq_CW34
chr1	162716636	162716637	MACS_peak_264	7.00	TCF7L2_standard_ChIP_seq_CW34
chr1	162863317	162863318	MACS_peak_265	43.00	TCF7L2_standard_ChIP_seq_CW34
chr1	165107272	165107273	MACS_peak_266	22.00	TCF7L2_standard_ChIP_seq_CW34
chr1	165455104	165455105	MACS_peak_267	9.00	TCF7L2_standard_ChIP_seq_CW34
chr1	165840840	165840841	MACS_peak_268	16.00	TCF7L2_standard_ChIP_seq_CW34
chr1	168430176	168430177	MACS_peak_269	6.00	TCF7L2_standard_ChIP_seq_CW34
chr1	168770887	168770888	MACS_peak_270	13.00	TCF7L2_standard_ChIP_seq_CW34
chr1	168803867	168803868	MACS_peak_271	7.00	TCF7L2_standard_ChIP_seq_CW34
chr1	169075437	169075438	MACS_peak_272	16.00	TCF7L2_standard_ChIP_seq_CW34
chr1	169515446	169515447	MACS_peak_273	23.00	TCF7L2_standard_ChIP_seq_CW34
chr1	169714504	169714505	MACS_peak_274	38.00	TCF7L2_standard_ChIP_seq_CW34
chr1	172439512	172439513	MACS_peak_275	8.00	TCF7L2_standard_ChIP_seq_CW34
chr1	174781967	174781968	MACS_peak_276	9.00	TCF7L2_standard_ChIP_seq_CW34
chr1	177616556	177616557	MACS_peak_277	6.00	TCF7L2_standard_ChIP_seq_CW34
chr1	178022245	178022246	MACS_peak_278	14.00	TCF7L2_standard_ChIP_seq_CW34
chr1	178177815	178177816	MACS_peak_279	9.00	TCF7L2_standard_ChIP_seq_CW34
chr1	178595059	178595060	MACS_peak_280	8.00	TCF7L2_standard_ChIP_seq_CW34
chr1	178694031	178694032	MACS_peak_281	7.00	TCF7L2_standard_ChIP_seq_CW34
chr1	178840396	178840397	MACS_peak_282	7.00	TCF7L2_standard_ChIP_seq_CW34
chr1	179015438	179015439	MACS_peak_283	9.00	TCF7L2_standard_ChIP_seq_CW34
chr1	180490932	180490933	MACS_peak_284	9.00	TCF7L2_standard_ChIP_seq_CW34
chr1	180784511	180784512	MACS_peak_285	9.00	TCF7L2_standard_ChIP_seq_CW34
chr1	180982477	180982478	MACS_peak_286	9.00	TCF7L2_standard_ChIP_seq_CW34
chr1	181074161	181074162	MACS_peak_287	10.00	TCF7L2_standard_ChIP_seq_CW34
chr1	182090053	182090054	MACS_peak_288	8.00	TCF7L2_standard_ChIP_seq_CW34
chr1	182271153	182271154	MACS_peak_289	9.00	TCF7L2_standard_ChIP_seq_CW34
chr1	183058412	183058413	MACS_peak_290	15.00	TCF7L2_standard_ChIP_seq_CW34
chr1	183749484	183749485	MACS_peak_291	18.00	TCF7L2_standard_ChIP_seq_CW34
chr1	185203518	185203519	MACS_peak_292	11.00	TCF7L2_standard_ChIP_seq_CW34
chr1	192977187	192977188	MACS_peak_293	7.00	TCF7L2_standard_ChIP_seq_CW34
chr1	197744416	197744417	MACS_peak_294	6.00	TCF7L2_standard_ChIP_seq_CW34
chr1	197851593	197851594	MACS_peak_295	8.00	TCF7L2_standard_ChIP_seq_CW34
chr1	197964534	197964535	MACS_peak_296	7.00	TCF7L2_standard_ChIP_seq_CW34
chr1	198503891	198503892	MACS_peak_297	13.00	TCF7L2_standard_ChIP_seq_CW34
chr1	200009894	200009895	MACS_peak_298	16.00	TCF7L2_standard_ChIP_seq_CW34
chr1	200373696	200373697	MACS_peak_299	7.00	TCF7L2_standard_ChIP_seq_CW34
chr1	201464775	201464776	MACS_peak_300	7.00	TCF7L2_standard_ChIP_seq_CW34
chr1	202012653	202012654	MACS_peak_301	11.00	TCF7L2_standard_ChIP_seq_CW34
chr1	202831934	202831935	MACS_peak_302	9.00	TCF7L2_standard_ChIP_seq_CW34
chr1	203180318	203180319	MACS_peak_303	10.00	TCF7L2_standard_ChIP_seq_CW34
chr1	204149421	204149422	MACS_peak_304	8.00	TCF7L2_standard_ChIP_seq_CW34
chr1	204462868	204462869	MACS_peak_305	14.00	TCF7L2_standard_ChIP_seq_CW34
chr1	204485605	204485606	MACS_peak_306	9.00	TCF7L2_standard_ChIP_seq_CW34
chr1	204616915	204616916	MACS_peak_307	11.00	TCF7L2_standard_ChIP_seq_CW34
chr1	205180479	205180480	MACS_peak_308	8.00	TCF7L2_standard_ChIP_seq_CW34
chr1	205270646	205270647	MACS_peak_309	10.00	TCF7L2_standard_ChIP_seq_CW34
chr1	205287910	205287911	MACS_peak_310	9.00	TCF7L2_standard_ChIP_seq_CW34
chr1	205454714	205454715	MACS_peak_311	15.00	TCF7L2_standard_ChIP_seq_CW34
chr1	205604717	205604718	MACS_peak_312	15.00	TCF7L2_standard_ChIP_seq_CW34
chr1	206136271	206136272	MACS_peak_313	12.00	TCF7L2_standard_ChIP_seq_CW34
chr1	206644439	206644440	MACS_peak_314	15.00	TCF7L2_standard_ChIP_seq_CW34
chr1	206873213	206873214	MACS_peak_315	8.00	TCF7L2_standard_ChIP_seq_CW34
chr1	207263574	207263575	MACS_peak_316	5.00	TCF7L2_standard_ChIP_seq_CW34
chr1	207414753	207414754	MACS_peak_317	17.00	TCF7L2_standard_ChIP_seq_CW34
chr1	207777256	207777257	MACS_peak_318	8.00	TCF7L2_standard_ChIP_seq_CW34
chr1	208308921	208308922	MACS_peak_319	5.00	TCF7L2_standard_ChIP_seq_CW34
chr1	208409724	208409725	MACS_peak_320	14.00	TCF7L2_standard_ChIP_seq_CW34
chr1	208417715	208417716	MACS_peak_321	8.00	TCF7L2_standard_ChIP_seq_CW34
chr1	208748030	208748031	MACS_peak_322	6.00	TCF7L2_standard_ChIP_seq_CW34
chr1	211432329	211432330	MACS_peak_323	21.00	TCF7L2_standard_ChIP_seq_CW34
chr1	211752963	211752964	MACS_peak_324	17.00	TCF7L2_standard_ChIP_seq_CW34
chr1	211816869	211816870	MACS_peak_325	19.00	TCF7L2_standard_ChIP_seq_CW34
chr1	211840740	211840741	MACS_peak_326	9.00	TCF7L2_standard_ChIP_seq_CW34
chr1	212003940	212003941	MACS_peak_327	7.00	TCF7L2_standard_ChIP_seq_CW34
chr1	212204575	212204576	MACS_peak_328	12.00	TCF7L2_standard_ChIP_seq_CW34
chr1	212458836	212458837	MACS_peak_329	10.00	TCF7L2_standard_ChIP_seq_CW34
chr1	213746474	213746475	MACS_peak_330	30.00	TCF7L2_standard_ChIP_seq_CW34
chr1	213907294	213907295	MACS_peak_331	7.00	TCF7L2_standard_ChIP_seq_CW34
chr1	214129952	214129953	MACS_peak_332	28.00	TCF7L2_standard_ChIP_seq_CW34
chr1	214201058	214201059	MACS_peak_333	11.00	TCF7L2_standard_ChIP_seq_CW34
chr1	214458248	214458249	MACS_peak_334	25.00	TCF7L2_standard_ChIP_seq_CW34
chr1	214633777	214633778	MACS_peak_335	12.00	TCF7L2_standard_ChIP_seq_CW34
chr1	214851953	214851954	MACS_peak_336	11.00	TCF7L2_standard_ChIP_seq_CW34
chr1	218458370	218458371	MACS_peak_337	9.00	TCF7L2_standard_ChIP_seq_CW34
chr1	219483946	219483947	MACS_peak_338	22.00	TCF7L2_standard_ChIP_seq_CW34
chr1	221509037	221509038	MACS_peak_339	6.00	TCF7L2_standard_ChIP_seq_CW34
chr1	222639726	222639727	MACS_peak_340	10.00	TCF7L2_standard_ChIP_seq_CW34
chr1	222817613	222817614	MACS_peak_341	13.00	TCF7L2_standard_ChIP_seq_CW34
chr1	223213643	223213644	MACS_peak_342	6.00	TCF7L2_standard_ChIP_seq_CW34
chr1	225133931	225133932	MACS_peak_343	24.00	TCF7L2_standard_ChIP_seq_CW34
chr1	225409553	225409554	MACS_peak_344	8.00	TCF7L2_standard_ChIP_seq_CW34
chr1	225416744	225416745	MACS_peak_345	36.00	TCF7L2_standard_ChIP_seq_CW34
chr1	225642058	225642059	MACS_peak_346	17.00	TCF7L2_standard_ChIP_seq_CW34
chr1	225859221	225859222	MACS_peak_347	8.00	TCF7L2_standard_ChIP_seq_CW34
chr1	225882104	225882105	MACS_peak_348	7.00	TCF7L2_standard_ChIP_seq_CW34
chr1	225955549	225955550	MACS_peak_349	16.00	TCF7L2_standard_ChIP_seq_CW34
chr1	226411331	226411332	MACS_peak_350	31.00	TCF7L2_standard_ChIP_seq_CW34
chr1	226667295	226667296	MACS_peak_351	19.00	TCF7L2_standard_ChIP_seq_CW34
chr1	226814416	226814417	MACS_peak_352	8.00	TCF7L2_standard_ChIP_seq_CW34
chr1	227751315	227751316	MACS_peak_353	7.00	TCF7L2_standard_ChIP_seq_CW34
chr1	228974500	228974501	MACS_peak_354	5.00	TCF7L2_standard_ChIP_seq_CW34
chr1	229167707	229167708	MACS_peak_355	9.00	TCF7L2_standard_ChIP_seq_CW34
chr1	229200323	229200324	MACS_peak_356	8.00	TCF7L2_standard_ChIP_seq_CW34
chr1	229434931	229434932	MACS_peak_357	8.00	TCF7L2_standard_ChIP_seq_CW34
chr1	230294681	230294682	MACS_peak_358	8.00	TCF7L2_standard_ChIP_seq_CW34
chr1	230775543	230775544	MACS_peak_359	5.00	TCF7L2_standard_ChIP_seq_CW34
chr1	230838164	230838165	MACS_peak_360	21.00	TCF7L2_standard_ChIP_seq_CW34
chr1	230864838	230864839	MACS_peak_361	28.00	TCF7L2_standard_ChIP_seq_CW34
chr1	231084730	231084731	MACS_peak_362	8.00	TCF7L2_standard_ChIP_seq_CW34
chr1	231355580	231355581	MACS_peak_363	31.00	TCF7L2_standard_ChIP_seq_CW34
chr1	231557323	231557324	MACS_peak_364	5.00	TCF7L2_standard_ChIP_seq_CW34
chr1	231762431	231762432	MACS_peak_365	12.00	TCF7L2_standard_ChIP_seq_CW34
chr1	231977845	231977846	MACS_peak_366	12.00	TCF7L2_standard_ChIP_seq_CW34
chr1	234449935	234449936	MACS_peak_367	12.00	TCF7L2_standard_ChIP_seq_CW34
chr1	234517903	234517904	MACS_peak_368	12.00	TCF7L2_standard_ChIP_seq_CW34
chr1	234659099	234659100	MACS_peak_369	10.00	TCF7L2_standard_ChIP_seq_CW34
chr1	234698273	234698274	MACS_peak_370	9.00	TCF7L2_standard_ChIP_seq_CW34
chr1	234735574	234735575	MACS_peak_371	14.00	TCF7L2_standard_ChIP_seq_CW34
chr1	234748304	234748305	MACS_peak_372	24.00	TCF7L2_standard_ChIP_seq_CW34
chr1	234779063	234779064	MACS_peak_373	14.00	TCF7L2_standard_ChIP_seq_CW34
chr1	234870829	234870830	MACS_peak_374	8.00	TCF7L2_standard_ChIP_seq_CW34
chr1	234899971	234899972	MACS_peak_375	11.00	TCF7L2_standard_ChIP_seq_CW34
chr1	234908247	234908248	MACS_peak_376	12.00	TCF7L2_standard_ChIP_seq_CW34
chr1	235012178	235012179	MACS_peak_377	9.00	TCF7L2_standard_ChIP_seq_CW34
chr1	235093445	235093446	MACS_peak_378	12.00	TCF7L2_standard_ChIP_seq_CW34
chr1	235099231	235099232	MACS_peak_379	11.00	TCF7L2_standard_ChIP_seq_CW34
chr1	235111089	235111090	MACS_peak_380	14.00	TCF7L2_standard_ChIP_seq_CW34
chr1	235459493	235459494	MACS_peak_381	7.00	TCF7L2_standard_ChIP_seq_CW34
chr1	235572924	235572925	MACS_peak_382	27.00	TCF7L2_standard_ChIP_seq_CW34
chr1	235667620	235667621	MACS_peak_383	6.00	TCF7L2_standard_ChIP_seq_CW34
chr1	235779412	235779413	MACS_peak_384	7.00	TCF7L2_standard_ChIP_seq_CW34
chr1	235987212	235987213	MACS_peak_385	17.00	TCF7L2_standard_ChIP_seq_CW34
chr1	236563073	236563074	MACS_peak_387	40.00	TCF7L2_standard_ChIP_seq_CW34
chr1	236644338	236644339	MACS_peak_388	7.00	TCF7L2_standard_ChIP_seq_CW34
chr1	236658077	236658078	MACS_peak_389	39.00	TCF7L2_standard_ChIP_seq_CW34
chr1	236958822	236958823	MACS_peak_390	6.00	TCF7L2_standard_ChIP_seq_CW34
chr1	237007928	237007929	MACS_peak_391	7.00	TCF7L2_standard_ChIP_seq_CW34
chr1	237068658	237068659	MACS_peak_392	9.00	TCF7L2_standard_ChIP_seq_CW34
chr1	237454660	237454661	MACS_peak_393	5.00	TCF7L2_standard_ChIP_seq_CW34
chr1	239979659	239979660	MACS_peak_394	7.00	TCF7L2_standard_ChIP_seq_CW34
chr1	243391065	243391066	MACS_peak_395	8.00	TCF7L2_standard_ChIP_seq_CW34
chr1	244065637	244065638	MACS_peak_396	19.00	TCF7L2_standard_ChIP_seq_CW34
chr1	244964767	244964768	MACS_peak_397	8.00	TCF7L2_standard_ChIP_seq_CW34
chr1	244997124	244997125	MACS_peak_398	20.00	TCF7L2_standard_ChIP_seq_CW34
chr1	245026315	245026316	MACS_peak_399	10.00	TCF7L2_standard_ChIP_seq_CW34
chr1	245320729	245320730	MACS_peak_400	11.00	TCF7L2_standard_ChIP_seq_CW34
chr1	245421762	245421763	MACS_peak_401	8.00	TCF7L2_standard_ChIP_seq_CW34
chr1	246879830	246879831	MACS_peak_402	9.00	TCF7L2_standard_ChIP_seq_CW34
chr10	1650049	1650050	MACS_peak_403	7.00	TCF7L2_standard_ChIP_seq_CW34
chr10	2122128	2122129	MACS_peak_404	5.00	TCF7L2_standard_ChIP_seq_CW34
chr10	3852511	3852512	MACS_peak_405	8.00	TCF7L2_standard_ChIP_seq_CW34
chr10	3932444	3932445	MACS_peak_406	9.00	TCF7L2_standard_ChIP_seq_CW34
chr10	4989588	4989589	MACS_peak_407	8.00	TCF7L2_standard_ChIP_seq_CW34
chr10	5189612	5189613	MACS_peak_408	8.00	TCF7L2_standard_ChIP_seq_CW34
chr10	5238165	5238166	MACS_peak_409	8.00	TCF7L2_standard_ChIP_seq_CW34
chr10	5285606	5285607	MACS_peak_410	12.00	TCF7L2_standard_ChIP_seq_CW34
chr10	5576292	5576293	MACS_peak_411	11.00	TCF7L2_standard_ChIP_seq_CW34
chr10	5590235	5590236	MACS_peak_412	11.00	TCF7L2_standard_ChIP_seq_CW34
chr10	5734787	5734788	MACS_peak_413	17.00	TCF7L2_standard_ChIP_seq_CW34
chr10	5873614	5873615	MACS_peak_414	14.00	TCF7L2_standard_ChIP_seq_CW34
chr10	7073676	7073677	MACS_peak_415	5.00	TCF7L2_standard_ChIP_seq_CW34
chr10	7482340	7482341	MACS_peak_416	5.00	TCF7L2_standard_ChIP_seq_CW34
chr10	7728597	7728598	MACS_peak_417	25.00	TCF7L2_standard_ChIP_seq_CW34
chr10	7749260	7749261	MACS_peak_418	12.00	TCF7L2_standard_ChIP_seq_CW34
chr10	7984994	7984995	MACS_peak_419	10.00	TCF7L2_standard_ChIP_seq_CW34
chr10	8092844	8092845	MACS_peak_420	10.00	TCF7L2_standard_ChIP_seq_CW34
chr10	11638075	11638076	MACS_peak_421	8.00	TCF7L2_standard_ChIP_seq_CW34
chr10	11645140	11645141	MACS_peak_422	8.00	TCF7L2_standard_ChIP_seq_CW34
chr10	11682424	11682425	MACS_peak_423	10.00	TCF7L2_standard_ChIP_seq_CW34
chr10	11741808	11741809	MACS_peak_424	9.00	TCF7L2_standard_ChIP_seq_CW34
chr10	11757033	11757034	MACS_peak_425	11.00	TCF7L2_standard_ChIP_seq_CW34
chr10	11874300	11874301	MACS_peak_426	10.00	TCF7L2_standard_ChIP_seq_CW34
chr10	11992548	11992549	MACS_peak_427	12.00	TCF7L2_standard_ChIP_seq_CW34
chr10	12279857	12279858	MACS_peak_428	13.00	TCF7L2_standard_ChIP_seq_CW34
chr10	13149792	13149793	MACS_peak_429	8.00	TCF7L2_standard_ChIP_seq_CW34
chr10	13542447	13542448	MACS_peak_430	6.00	TCF7L2_standard_ChIP_seq_CW34
chr10	14226993	14226994	MACS_peak_431	28.00	TCF7L2_standard_ChIP_seq_CW34
chr10	14316593	14316594	MACS_peak_432	7.00	TCF7L2_standard_ChIP_seq_CW34
chr10	14614152	14614153	MACS_peak_433	6.00	TCF7L2_standard_ChIP_seq_CW34
chr10	14921319	14921320	MACS_peak_434	13.00	TCF7L2_standard_ChIP_seq_CW34
chr10	15376390	15376391	MACS_peak_435	8.00	TCF7L2_standard_ChIP_seq_CW34
chr10	15386992	15386993	MACS_peak_436	9.00	TCF7L2_standard_ChIP_seq_CW34
chr10	15878962	15878963	MACS_peak_437	25.00	TCF7L2_standard_ChIP_seq_CW34
chr10	17693115	17693116	MACS_peak_438	19.00	TCF7L2_standard_ChIP_seq_CW34
chr10	21822754	21822755	MACS_peak_439	10.00	TCF7L2_standard_ChIP_seq_CW34
chr10	21824572	21824573	MACS_peak_440	11.00	TCF7L2_standard_ChIP_seq_CW34
chr10	21874006	21874007	MACS_peak_441	11.00	TCF7L2_standard_ChIP_seq_CW34
chr10	22013733	22013734	MACS_peak_442	10.00	TCF7L2_standard_ChIP_seq_CW34
chr10	22725848	22725849	MACS_peak_443	6.00	TCF7L2_standard_ChIP_seq_CW34
chr10	22972352	22972353	MACS_peak_444	9.00	TCF7L2_standard_ChIP_seq_CW34
chr10	29058389	29058390	MACS_peak_445	10.00	TCF7L2_standard_ChIP_seq_CW34
chr10	29164932	29164933	MACS_peak_446	7.00	TCF7L2_standard_ChIP_seq_CW34
chr10	29228988	29228989	MACS_peak_447	23.00	TCF7L2_standard_ChIP_seq_CW34
chr10	30831270	30831271	MACS_peak_448	12.00	TCF7L2_standard_ChIP_seq_CW34
chr10	30846887	30846888	MACS_peak_449	8.00	TCF7L2_standard_ChIP_seq_CW34
chr10	30901071	30901072	MACS_peak_450	20.00	TCF7L2_standard_ChIP_seq_CW34
chr10	31531984	31531985	MACS_peak_451	12.00	TCF7L2_standard_ChIP_seq_CW34
chr10	32230129	32230130	MACS_peak_452	7.00	TCF7L2_standard_ChIP_seq_CW34
chr10	32345206	32345207	MACS_peak_453	9.00	TCF7L2_standard_ChIP_seq_CW34
chr10	32409213	32409214	MACS_peak_454	10.00	TCF7L2_standard_ChIP_seq_CW34
chr10	33247100	33247101	MACS_peak_455	8.00	TCF7L2_standard_ChIP_seq_CW34
chr10	33797068	33797069	MACS_peak_456	23.00	TCF7L2_standard_ChIP_seq_CW34
chr10	34410375	34410376	MACS_peak_457	27.00	TCF7L2_standard_ChIP_seq_CW34
chr10	34502596	34502597	MACS_peak_458	32.00	TCF7L2_standard_ChIP_seq_CW34
chr10	38146615	38146616	MACS_peak_459	5.00	TCF7L2_standard_ChIP_seq_CW34
chr10	43913757	43913758	MACS_peak_472	20.00	TCF7L2_standard_ChIP_seq_CW34
chr10	44732406	44732407	MACS_peak_473	35.00	TCF7L2_standard_ChIP_seq_CW34
chr10	44754692	44754693	MACS_peak_474	7.00	TCF7L2_standard_ChIP_seq_CW34
chr10	45491512	45491513	MACS_peak_475	9.00	TCF7L2_standard_ChIP_seq_CW34
chr10	51489230	51489231	MACS_peak_476	5.00	TCF7L2_standard_ChIP_seq_CW34
chr10	52326081	52326082	MACS_peak_477	9.00	TCF7L2_standard_ChIP_seq_CW34
chr10	52332924	52332925	MACS_peak_478	6.00	TCF7L2_standard_ChIP_seq_CW34
chr10	52642576	52642577	MACS_peak_479	9.00	TCF7L2_standard_ChIP_seq_CW34
chr10	54073978	54073979	MACS_peak_480	28.00	TCF7L2_standard_ChIP_seq_CW34
chr10	54138157	54138158	MACS_peak_481	15.00	TCF7L2_standard_ChIP_seq_CW34
chr10	54212676	54212677	MACS_peak_482	31.00	TCF7L2_standard_ChIP_seq_CW34
chr10	54534511	54534512	MACS_peak_483	13.00	TCF7L2_standard_ChIP_seq_CW34
chr10	55750934	55750935	MACS_peak_484	5.00	TCF7L2_standard_ChIP_seq_CW34
chr10	57030671	57030672	MACS_peak_485	6.00	TCF7L2_standard_ChIP_seq_CW34
chr10	57853605	57853606	MACS_peak_486	19.00	TCF7L2_standard_ChIP_seq_CW34
chr10	58121097	58121098	MACS_peak_487	6.00	TCF7L2_standard_ChIP_seq_CW34
chr10	60029269	60029270	MACS_peak_488	6.00	TCF7L2_standard_ChIP_seq_CW34
chr10	60145011	60145012	MACS_peak_489	12.00	TCF7L2_standard_ChIP_seq_CW34
chr10	60228530	60228531	MACS_peak_490	23.00	TCF7L2_standard_ChIP_seq_CW34
chr10	60767271	60767272	MACS_peak_491	7.00	TCF7L2_standard_ChIP_seq_CW34
chr10	60817979	60817980	MACS_peak_492	30.00	TCF7L2_standard_ChIP_seq_CW34
chr10	60833491	60833492	MACS_peak_493	57.00	TCF7L2_standard_ChIP_seq_CW34
chr10	61198350	61198351	MACS_peak_494	6.00	TCF7L2_standard_ChIP_seq_CW34
chr10	61567934	61567935	MACS_peak_495	6.00	TCF7L2_standard_ChIP_seq_CW34
chr10	62761228	62761229	MACS_peak_496	7.00	TCF7L2_standard_ChIP_seq_CW34
chr10	62822948	62822949	MACS_peak_497	20.00	TCF7L2_standard_ChIP_seq_CW34
chr10	62826919	62826920	MACS_peak_498	27.00	TCF7L2_standard_ChIP_seq_CW34
chr10	62835495	62835496	MACS_peak_499	21.00	TCF7L2_standard_ChIP_seq_CW34
chr10	63855531	63855532	MACS_peak_500	14.00	TCF7L2_standard_ChIP_seq_CW34
chr10	64028465	64028466	MACS_peak_501	8.00	TCF7L2_standard_ChIP_seq_CW34
chr10	64544483	64544484	MACS_peak_502	14.00	TCF7L2_standard_ChIP_seq_CW34
chr10	65247977	65247978	MACS_peak_503	10.00	TCF7L2_standard_ChIP_seq_CW34
chr10	65835446	65835447	MACS_peak_504	21.00	TCF7L2_standard_ChIP_seq_CW34
chr10	67261121	67261122	MACS_peak_505	14.00	TCF7L2_standard_ChIP_seq_CW34
chr10	68043917	68043918	MACS_peak_506	9.00	TCF7L2_standard_ChIP_seq_CW34
chr10	69563573	69563574	MACS_peak_507	35.00	TCF7L2_standard_ChIP_seq_CW34
chr10	70481197	70481198	MACS_peak_508	6.00	TCF7L2_standard_ChIP_seq_CW34
chr10	70715652	70715653	MACS_peak_509	7.00	TCF7L2_standard_ChIP_seq_CW34
chr10	71178082	71178083	MACS_peak_510	13.00	TCF7L2_standard_ChIP_seq_CW34
chr10	71890953	71890954	MACS_peak_511	12.00	TCF7L2_standard_ChIP_seq_CW34
chr10	72202259	72202260	MACS_peak_512	24.00	TCF7L2_standard_ChIP_seq_CW34
chr10	72595978	72595979	MACS_peak_513	6.00	TCF7L2_standard_ChIP_seq_CW34
chr10	73790734	73790735	MACS_peak_514	9.00	TCF7L2_standard_ChIP_seq_CW34
chr10	73848383	73848384	MACS_peak_515	9.00	TCF7L2_standard_ChIP_seq_CW34
chr10	74079335	74079336	MACS_peak_516	16.00	TCF7L2_standard_ChIP_seq_CW34
chr10	74892242	74892243	MACS_peak_517	7.00	TCF7L2_standard_ChIP_seq_CW34
chr10	75571553	75571554	MACS_peak_518	12.00	TCF7L2_standard_ChIP_seq_CW34
chr10	75910632	75910633	MACS_peak_519	21.00	TCF7L2_standard_ChIP_seq_CW34
chr10	76582113	76582114	MACS_peak_520	9.00	TCF7L2_standard_ChIP_seq_CW34
chr10	76928193	76928194	MACS_peak_521	26.00	TCF7L2_standard_ChIP_seq_CW34
chr10	77054754	77054755	MACS_peak_522	20.00	TCF7L2_standard_ChIP_seq_CW34
chr10	77165038	77165039	MACS_peak_523	39.00	TCF7L2_standard_ChIP_seq_CW34
chr10	77190225	77190226	MACS_peak_524	42.00	TCF7L2_standard_ChIP_seq_CW34
chr10	77279208	77279209	MACS_peak_525	16.00	TCF7L2_standard_ChIP_seq_CW34
chr10	77515498	77515499	MACS_peak_526	23.00	TCF7L2_standard_ChIP_seq_CW34
chr10	77536696	77536697	MACS_peak_527	16.00	TCF7L2_standard_ChIP_seq_CW34
chr10	77811074	77811075	MACS_peak_528	65.00	TCF7L2_standard_ChIP_seq_CW34
chr10	78544055	78544056	MACS_peak_529	5.00	TCF7L2_standard_ChIP_seq_CW34
chr10	80218922	80218923	MACS_peak_530	29.00	TCF7L2_standard_ChIP_seq_CW34
chr10	80232038	80232039	MACS_peak_531	10.00	TCF7L2_standard_ChIP_seq_CW34
chr10	80280879	80280880	MACS_peak_532	7.00	TCF7L2_standard_ChIP_seq_CW34
chr10	80502961	80502962	MACS_peak_533	7.00	TCF7L2_standard_ChIP_seq_CW34
chr10	80819157	80819158	MACS_peak_534	10.00	TCF7L2_standard_ChIP_seq_CW34
chr10	80828643	80828644	MACS_peak_535	18.00	TCF7L2_standard_ChIP_seq_CW34
chr10	81095497	81095498	MACS_peak_536	11.00	TCF7L2_standard_ChIP_seq_CW34
chr10	86283385	86283386	MACS_peak_537	17.00	TCF7L2_standard_ChIP_seq_CW34
chr10	88160929	88160930	MACS_peak_538	6.00	TCF7L2_standard_ChIP_seq_CW34
chr10	88621287	88621288	MACS_peak_539	6.00	TCF7L2_standard_ChIP_seq_CW34
chr10	89260588	89260589	MACS_peak_540	19.00	TCF7L2_standard_ChIP_seq_CW34
chr10	89623370	89623371	MACS_peak_541	10.00	TCF7L2_standard_ChIP_seq_CW34
chr10	91574320	91574321	MACS_peak_542	22.00	TCF7L2_standard_ChIP_seq_CW34
chr10	92755788	92755789	MACS_peak_543	6.00	TCF7L2_standard_ChIP_seq_CW34
chr10	93316810	93316811	MACS_peak_544	7.00	TCF7L2_standard_ChIP_seq_CW34
chr10	93368653	93368654	MACS_peak_545	6.00	TCF7L2_standard_ChIP_seq_CW34
chr10	93466270	93466271	MACS_peak_546	22.00	TCF7L2_standard_ChIP_seq_CW34
chr10	93558337	93558338	MACS_peak_547	7.00	TCF7L2_standard_ChIP_seq_CW34
chr10	94000648	94000649	MACS_peak_548	9.00	TCF7L2_standard_ChIP_seq_CW34
chr10	94051043	94051044	MACS_peak_549	8.00	TCF7L2_standard_ChIP_seq_CW34
chr10	95297472	95297473	MACS_peak_550	6.00	TCF7L2_standard_ChIP_seq_CW34
chr10	96897960	96897961	MACS_peak_551	6.00	TCF7L2_standard_ChIP_seq_CW34
chr10	97002984	97002985	MACS_peak_552	8.00	TCF7L2_standard_ChIP_seq_CW34
chr10	97038063	97038064	MACS_peak_553	20.00	TCF7L2_standard_ChIP_seq_CW34
chr10	97107010	97107011	MACS_peak_554	24.00	TCF7L2_standard_ChIP_seq_CW34
chr10	97227157	97227158	MACS_peak_555	14.00	TCF7L2_standard_ChIP_seq_CW34
chr10	97240829	97240830	MACS_peak_556	11.00	TCF7L2_standard_ChIP_seq_CW34
chr10	97299614	97299615	MACS_peak_557	8.00	TCF7L2_standard_ChIP_seq_CW34
chr10	97324255	97324256	MACS_peak_558	8.00	TCF7L2_standard_ChIP_seq_CW34
chr10	98356611	98356612	MACS_peak_559	6.00	TCF7L2_standard_ChIP_seq_CW34
chr10	98437347	98437348	MACS_peak_560	7.00	TCF7L2_standard_ChIP_seq_CW34
chr10	98561386	98561387	MACS_peak_561	9.00	TCF7L2_standard_ChIP_seq_CW34
chr10	98602963	98602964	MACS_peak_562	19.00	TCF7L2_standard_ChIP_seq_CW34
chr10	98900435	98900436	MACS_peak_563	20.00	TCF7L2_standard_ChIP_seq_CW34
chr10	99094638	99094639	MACS_peak_564	9.00	TCF7L2_standard_ChIP_seq_CW34
chr10	99496785	99496786	MACS_peak_565	6.00	TCF7L2_standard_ChIP_seq_CW34
chr10	100030312	100030313	MACS_peak_566	9.00	TCF7L2_standard_ChIP_seq_CW34
chr10	101181280	101181281	MACS_peak_567	12.00	TCF7L2_standard_ChIP_seq_CW34
chr10	101190965	101190966	MACS_peak_568	7.00	TCF7L2_standard_ChIP_seq_CW34
chr10	101281283	101281284	MACS_peak_569	14.00	TCF7L2_standard_ChIP_seq_CW34
chr10	101380358	101380359	MACS_peak_570	32.00	TCF7L2_standard_ChIP_seq_CW34
chr10	101861585	101861586	MACS_peak_571	10.00	TCF7L2_standard_ChIP_seq_CW34
chr10	102635677	102635678	MACS_peak_572	14.00	TCF7L2_standard_ChIP_seq_CW34
chr10	102641203	102641204	MACS_peak_573	10.00	TCF7L2_standard_ChIP_seq_CW34
chr10	102731155	102731156	MACS_peak_574	7.00	TCF7L2_standard_ChIP_seq_CW34
chr10	102820965	102820966	MACS_peak_575	6.00	TCF7L2_standard_ChIP_seq_CW34
chr10	102872970	102872971	MACS_peak_576	33.00	TCF7L2_standard_ChIP_seq_CW34
chr10	103124665	103124666	MACS_peak_577	9.00	TCF7L2_standard_ChIP_seq_CW34
chr10	103538905	103538906	MACS_peak_578	12.00	TCF7L2_standard_ChIP_seq_CW34
chr10	104192320	104192321	MACS_peak_579	25.00	TCF7L2_standard_ChIP_seq_CW34
chr10	104402160	104402161	MACS_peak_580	32.00	TCF7L2_standard_ChIP_seq_CW34
chr10	104421077	104421078	MACS_peak_581	38.00	TCF7L2_standard_ChIP_seq_CW34
chr10	104598112	104598113	MACS_peak_582	8.00	TCF7L2_standard_ChIP_seq_CW34
chr10	104678137	104678138	MACS_peak_583	9.00	TCF7L2_standard_ChIP_seq_CW34
chr10	104748592	104748593	MACS_peak_584	8.00	TCF7L2_standard_ChIP_seq_CW34
chr10	105405354	105405355	MACS_peak_585	16.00	TCF7L2_standard_ChIP_seq_CW34
chr10	105554155	105554156	MACS_peak_586	17.00	TCF7L2_standard_ChIP_seq_CW34
chr10	105614175	105614176	MACS_peak_587	19.00	TCF7L2_standard_ChIP_seq_CW34
chr10	106048248	106048249	MACS_peak_588	12.00	TCF7L2_standard_ChIP_seq_CW34
chr10	106109129	106109130	MACS_peak_589	6.00	TCF7L2_standard_ChIP_seq_CW34
chr10	112329706	112329707	MACS_peak_590	8.00	TCF7L2_standard_ChIP_seq_CW34
chr10	113235645	113235646	MACS_peak_591	34.00	TCF7L2_standard_ChIP_seq_CW34
chr10	114021322	114021323	MACS_peak_592	71.00	TCF7L2_standard_ChIP_seq_CW34
chr10	114035764	114035765	MACS_peak_593	10.00	TCF7L2_standard_ChIP_seq_CW34
chr10	114047022	114047023	MACS_peak_594	8.00	TCF7L2_standard_ChIP_seq_CW34
chr10	114092443	114092444	MACS_peak_595	11.00	TCF7L2_standard_ChIP_seq_CW34
chr10	114133736	114133737	MACS_peak_596	9.00	TCF7L2_standard_ChIP_seq_CW34
chr10	114154458	114154459	MACS_peak_597	25.00	TCF7L2_standard_ChIP_seq_CW34
chr10	114250567	114250568	MACS_peak_598	15.00	TCF7L2_standard_ChIP_seq_CW34
chr10	114403767	114403768	MACS_peak_599	10.00	TCF7L2_standard_ChIP_seq_CW34
chr10	114571791	114571792	MACS_peak_600	8.00	TCF7L2_standard_ChIP_seq_CW34
chr10	114710091	114710092	MACS_peak_601	12.00	TCF7L2_standard_ChIP_seq_CW34
chr10	114767237	114767238	MACS_peak_602	47.00	TCF7L2_standard_ChIP_seq_CW34
chr10	116249491	116249492	MACS_peak_603	18.00	TCF7L2_standard_ChIP_seq_CW34
chr10	120183477	120183478	MACS_peak_604	44.00	TCF7L2_standard_ChIP_seq_CW34
chr10	120789587	120789588	MACS_peak_605	8.00	TCF7L2_standard_ChIP_seq_CW34
chr10	120880143	120880144	MACS_peak_606	7.00	TCF7L2_standard_ChIP_seq_CW34
chr10	121403355	121403356	MACS_peak_607	8.00	TCF7L2_standard_ChIP_seq_CW34
chr10	122823489	122823490	MACS_peak_608	8.00	TCF7L2_standard_ChIP_seq_CW34
chr10	123449791	123449792	MACS_peak_609	6.00	TCF7L2_standard_ChIP_seq_CW34
chr10	123558847	123558848	MACS_peak_610	5.00	TCF7L2_standard_ChIP_seq_CW34
chr10	124069987	124069988	MACS_peak_611	9.00	TCF7L2_standard_ChIP_seq_CW34
chr10	124134382	124134383	MACS_peak_612	8.00	TCF7L2_standard_ChIP_seq_CW34
chr10	124779699	124779700	MACS_peak_613	9.00	TCF7L2_standard_ChIP_seq_CW34
chr10	125764336	125764337	MACS_peak_614	10.00	TCF7L2_standard_ChIP_seq_CW34
chr10	125802977	125802978	MACS_peak_615	10.00	TCF7L2_standard_ChIP_seq_CW34
chr10	125853259	125853260	MACS_peak_616	11.00	TCF7L2_standard_ChIP_seq_CW34
chr10	127481256	127481257	MACS_peak_617	65.00	TCF7L2_standard_ChIP_seq_CW34
chr10	127600765	127600766	MACS_peak_618	12.00	TCF7L2_standard_ChIP_seq_CW34
chr10	128423736	128423737	MACS_peak_619	15.00	TCF7L2_standard_ChIP_seq_CW34
chr10	130262803	130262804	MACS_peak_620	16.00	TCF7L2_standard_ChIP_seq_CW34
chr10	134412707	134412708	MACS_peak_621	8.00	TCF7L2_standard_ChIP_seq_CW34
chr10	135160872	135160873	MACS_peak_622	8.00	TCF7L2_standard_ChIP_seq_CW34
chr11	354211	354212	MACS_peak_625	9.00	TCF7L2_standard_ChIP_seq_CW34
chr11	576297	576298	MACS_peak_626	9.00	TCF7L2_standard_ChIP_seq_CW34
chr11	705042	705043	MACS_peak_627	8.00	TCF7L2_standard_ChIP_seq_CW34
chr11	842677	842678	MACS_peak_628	12.00	TCF7L2_standard_ChIP_seq_CW34
chr11	925435	925436	MACS_peak_629	6.00	TCF7L2_standard_ChIP_seq_CW34
chr11	1092946	1092947	MACS_peak_630	11.00	TCF7L2_standard_ChIP_seq_CW34
chr11	1241260	1241261	MACS_peak_631	17.00	TCF7L2_standard_ChIP_seq_CW34
chr11	1712567	1712568	MACS_peak_632	14.00	TCF7L2_standard_ChIP_seq_CW34
chr11	1968945	1968946	MACS_peak_633	6.00	TCF7L2_standard_ChIP_seq_CW34
chr11	2036535	2036536	MACS_peak_634	14.00	TCF7L2_standard_ChIP_seq_CW34
chr11	2225327	2225328	MACS_peak_635	57.00	TCF7L2_standard_ChIP_seq_CW34
chr11	2232502	2232503	MACS_peak_636	33.00	TCF7L2_standard_ChIP_seq_CW34
chr11	2234774	2234775	MACS_peak_637	33.00	TCF7L2_standard_ChIP_seq_CW34
chr11	3981047	3981048	MACS_peak_638	8.00	TCF7L2_standard_ChIP_seq_CW34
chr11	6411732	6411733	MACS_peak_639	9.00	TCF7L2_standard_ChIP_seq_CW34
chr11	9482285	9482286	MACS_peak_640	10.00	TCF7L2_standard_ChIP_seq_CW34
chr11	9532472	9532473	MACS_peak_641	14.00	TCF7L2_standard_ChIP_seq_CW34
chr11	9954571	9954572	MACS_peak_642	27.00	TCF7L2_standard_ChIP_seq_CW34
chr11	10072265	10072266	MACS_peak_643	8.00	TCF7L2_standard_ChIP_seq_CW34
chr11	10445487	10445488	MACS_peak_644	9.00	TCF7L2_standard_ChIP_seq_CW34
chr11	10681341	10681342	MACS_peak_645	20.00	TCF7L2_standard_ChIP_seq_CW34
chr11	10783054	10783055	MACS_peak_646	15.00	TCF7L2_standard_ChIP_seq_CW34
chr11	12107444	12107445	MACS_peak_647	8.00	TCF7L2_standard_ChIP_seq_CW34
chr11	12170650	12170651	MACS_peak_648	13.00	TCF7L2_standard_ChIP_seq_CW34
chr11	12824357	12824358	MACS_peak_649	17.00	TCF7L2_standard_ChIP_seq_CW34
chr11	12867556	12867557	MACS_peak_650	9.00	TCF7L2_standard_ChIP_seq_CW34
chr11	13064493	13064494	MACS_peak_651	6.00	TCF7L2_standard_ChIP_seq_CW34
chr11	13218414	13218415	MACS_peak_652	4.00	TCF7L2_standard_ChIP_seq_CW34
chr11	13484682	13484683	MACS_peak_653	6.00	TCF7L2_standard_ChIP_seq_CW34
chr11	14586065	14586066	MACS_peak_654	4.00	TCF7L2_standard_ChIP_seq_CW34
chr11	14762877	14762878	MACS_peak_655	7.00	TCF7L2_standard_ChIP_seq_CW34
chr11	16791688	16791689	MACS_peak_656	9.00	TCF7L2_standard_ChIP_seq_CW34
chr11	19259519	19259520	MACS_peak_657	7.00	TCF7L2_standard_ChIP_seq_CW34
chr11	20052805	20052806	MACS_peak_658	11.00	TCF7L2_standard_ChIP_seq_CW34
chr11	21712766	21712767	MACS_peak_659	6.00	TCF7L2_standard_ChIP_seq_CW34
chr11	22656893	22656894	MACS_peak_660	14.00	TCF7L2_standard_ChIP_seq_CW34
chr11	25758866	25758867	MACS_peak_661	9.00	TCF7L2_standard_ChIP_seq_CW34
chr11	27352368	27352369	MACS_peak_662	14.00	TCF7L2_standard_ChIP_seq_CW34
chr11	34385052	34385053	MACS_peak_663	16.00	TCF7L2_standard_ChIP_seq_CW34
chr11	34385936	34385937	MACS_peak_664	10.00	TCF7L2_standard_ChIP_seq_CW34
chr11	34448858	34448859	MACS_peak_665	23.00	TCF7L2_standard_ChIP_seq_CW34
chr11	34517671	34517672	MACS_peak_666	9.00	TCF7L2_standard_ChIP_seq_CW34
chr11	34686704	34686705	MACS_peak_667	8.00	TCF7L2_standard_ChIP_seq_CW34
chr11	35381810	35381811	MACS_peak_668	43.00	TCF7L2_standard_ChIP_seq_CW34
chr11	36453375	36453376	MACS_peak_669	5.00	TCF7L2_standard_ChIP_seq_CW34
chr11	39756619	39756620	MACS_peak_670	5.00	TCF7L2_standard_ChIP_seq_CW34
chr11	42969164	42969165	MACS_peak_671	4.00	TCF7L2_standard_ChIP_seq_CW34
chr11	43701907	43701908	MACS_peak_672	8.00	TCF7L2_standard_ChIP_seq_CW34
chr11	44431040	44431041	MACS_peak_673	7.00	TCF7L2_standard_ChIP_seq_CW34
chr11	44545242	44545243	MACS_peak_674	11.00	TCF7L2_standard_ChIP_seq_CW34
chr11	45921545	45921546	MACS_peak_675	9.00	TCF7L2_standard_ChIP_seq_CW34
chr11	45943882	45943883	MACS_peak_676	15.00	TCF7L2_standard_ChIP_seq_CW34
chr11	46295656	46295657	MACS_peak_677	9.00	TCF7L2_standard_ChIP_seq_CW34
chr11	46575979	46575980	MACS_peak_678	47.00	TCF7L2_standard_ChIP_seq_CW34
chr11	46867761	46867762	MACS_peak_679	9.00	TCF7L2_standard_ChIP_seq_CW34
chr11	47138129	47138130	MACS_peak_680	6.00	TCF7L2_standard_ChIP_seq_CW34
chr11	47600570	47600571	MACS_peak_681	11.00	TCF7L2_standard_ChIP_seq_CW34
chr11	57320093	57320094	MACS_peak_691	28.00	TCF7L2_standard_ChIP_seq_CW34
chr11	57933244	57933245	MACS_peak_692	14.00	TCF7L2_standard_ChIP_seq_CW34
chr11	59578335	59578336	MACS_peak_693	10.00	TCF7L2_standard_ChIP_seq_CW34
chr11	60612470	60612471	MACS_peak_694	13.00	TCF7L2_standard_ChIP_seq_CW34
chr11	60929058	60929059	MACS_peak_695	5.00	TCF7L2_standard_ChIP_seq_CW34
chr11	61275894	61275895	MACS_peak_696	8.00	TCF7L2_standard_ChIP_seq_CW34
chr11	61537158	61537159	MACS_peak_697	9.00	TCF7L2_standard_ChIP_seq_CW34
chr11	61595327	61595328	MACS_peak_698	19.00	TCF7L2_standard_ChIP_seq_CW34
chr11	62027945	62027946	MACS_peak_699	20.00	TCF7L2_standard_ChIP_seq_CW34
chr11	62041407	62041408	MACS_peak_700	7.00	TCF7L2_standard_ChIP_seq_CW34
chr11	62235347	62235348	MACS_peak_701	9.00	TCF7L2_standard_ChIP_seq_CW34
chr11	62264635	62264636	MACS_peak_702	9.00	TCF7L2_standard_ChIP_seq_CW34
chr11	62389701	62389702	MACS_peak_703	6.00	TCF7L2_standard_ChIP_seq_CW34
chr11	62572910	62572911	MACS_peak_704	9.00	TCF7L2_standard_ChIP_seq_CW34
chr11	62609199	62609200	MACS_peak_705	12.00	TCF7L2_standard_ChIP_seq_CW34
chr11	62649048	62649049	MACS_peak_706	16.00	TCF7L2_standard_ChIP_seq_CW34
chr11	63017777	63017778	MACS_peak_707	23.00	TCF7L2_standard_ChIP_seq_CW34
chr11	63259351	63259352	MACS_peak_708	9.00	TCF7L2_standard_ChIP_seq_CW34
chr11	63738532	63738533	MACS_peak_709	8.00	TCF7L2_standard_ChIP_seq_CW34
chr11	63975708	63975709	MACS_peak_710	6.00	TCF7L2_standard_ChIP_seq_CW34
chr11	64334020	64334021	MACS_peak_711	30.00	TCF7L2_standard_ChIP_seq_CW34
chr11	64781860	64781861	MACS_peak_712	8.00	TCF7L2_standard_ChIP_seq_CW34
chr11	64902161	64902162	MACS_peak_713	13.00	TCF7L2_standard_ChIP_seq_CW34
chr11	64933577	64933578	MACS_peak_714	21.00	TCF7L2_standard_ChIP_seq_CW34
chr11	65151414	65151415	MACS_peak_715	12.00	TCF7L2_standard_ChIP_seq_CW34
chr11	65337501	65337502	MACS_peak_716	7.00	TCF7L2_standard_ChIP_seq_CW34
chr11	66675621	66675622	MACS_peak_717	15.00	TCF7L2_standard_ChIP_seq_CW34
chr11	67141089	67141090	MACS_peak_718	9.00	TCF7L2_standard_ChIP_seq_CW34
chr11	67334472	67334473	MACS_peak_719	5.00	TCF7L2_standard_ChIP_seq_CW34
chr11	67349248	67349249	MACS_peak_720	7.00	TCF7L2_standard_ChIP_seq_CW34
chr11	67390651	67390652	MACS_peak_721	8.00	TCF7L2_standard_ChIP_seq_CW34
chr11	67867084	67867085	MACS_peak_722	31.00	TCF7L2_standard_ChIP_seq_CW34
chr11	67981343	67981344	MACS_peak_723	10.00	TCF7L2_standard_ChIP_seq_CW34
chr11	68065813	68065814	MACS_peak_724	60.00	TCF7L2_standard_ChIP_seq_CW34
chr11	69383173	69383174	MACS_peak_725	33.00	TCF7L2_standard_ChIP_seq_CW34
chr11	69455938	69455939	MACS_peak_726	12.00	TCF7L2_standard_ChIP_seq_CW34
chr11	69472941	69472942	MACS_peak_727	33.00	TCF7L2_standard_ChIP_seq_CW34
chr11	69517281	69517282	MACS_peak_728	39.00	TCF7L2_standard_ChIP_seq_CW34
chr11	69816646	69816647	MACS_peak_729	18.00	TCF7L2_standard_ChIP_seq_CW34
chr11	70058545	70058546	MACS_peak_730	11.00	TCF7L2_standard_ChIP_seq_CW34
chr11	70484559	70484560	MACS_peak_731	14.00	TCF7L2_standard_ChIP_seq_CW34
chr11	70963991	70963992	MACS_peak_732	8.00	TCF7L2_standard_ChIP_seq_CW34
chr11	71984297	71984298	MACS_peak_733	33.00	TCF7L2_standard_ChIP_seq_CW34
chr11	72109290	72109291	MACS_peak_734	26.00	TCF7L2_standard_ChIP_seq_CW34
chr11	72460253	72460254	MACS_peak_735	9.00	TCF7L2_standard_ChIP_seq_CW34
chr11	72489151	72489152	MACS_peak_736	7.00	TCF7L2_standard_ChIP_seq_CW34
chr11	72525167	72525168	MACS_peak_737	7.00	TCF7L2_standard_ChIP_seq_CW34
chr11	73150851	73150852	MACS_peak_738	9.00	TCF7L2_standard_ChIP_seq_CW34
chr11	73588073	73588074	MACS_peak_739	29.00	TCF7L2_standard_ChIP_seq_CW34
chr11	74693105	74693106	MACS_peak_740	7.00	TCF7L2_standard_ChIP_seq_CW34
chr11	74724364	74724365	MACS_peak_741	8.00	TCF7L2_standard_ChIP_seq_CW34
chr11	74879774	74879775	MACS_peak_742	25.00	TCF7L2_standard_ChIP_seq_CW34
chr11	74915165	74915166	MACS_peak_743	11.00	TCF7L2_standard_ChIP_seq_CW34
chr11	75479674	75479675	MACS_peak_744	14.00	TCF7L2_standard_ChIP_seq_CW34
chr11	75921838	75921839	MACS_peak_745	34.00	TCF7L2_standard_ChIP_seq_CW34
chr11	76040312	76040313	MACS_peak_746	22.00	TCF7L2_standard_ChIP_seq_CW34
chr11	76663711	76663712	MACS_peak_747	10.00	TCF7L2_standard_ChIP_seq_CW34
chr11	76747691	76747692	MACS_peak_748	10.00	TCF7L2_standard_ChIP_seq_CW34
chr11	77300907	77300908	MACS_peak_749	17.00	TCF7L2_standard_ChIP_seq_CW34
chr11	77801857	77801858	MACS_peak_750	11.00	TCF7L2_standard_ChIP_seq_CW34
chr11	78312276	78312277	MACS_peak_751	81.00	TCF7L2_standard_ChIP_seq_CW34
chr11	78324451	78324452	MACS_peak_752	7.00	TCF7L2_standard_ChIP_seq_CW34
chr11	82700473	82700474	MACS_peak_753	21.00	TCF7L2_standard_ChIP_seq_CW34
chr11	82804118	82804119	MACS_peak_754	16.00	TCF7L2_standard_ChIP_seq_CW34
chr11	85214844	85214845	MACS_peak_756	6.00	TCF7L2_standard_ChIP_seq_CW34
chr11	85637929	85637930	MACS_peak_757	8.00	TCF7L2_standard_ChIP_seq_CW34
chr11	85916910	85916911	MACS_peak_758	14.00	TCF7L2_standard_ChIP_seq_CW34
chr11	86383401	86383402	MACS_peak_759	9.00	TCF7L2_standard_ChIP_seq_CW34
chr11	86417614	86417615	MACS_peak_760	15.00	TCF7L2_standard_ChIP_seq_CW34
chr11	86711607	86711608	MACS_peak_761	23.00	TCF7L2_standard_ChIP_seq_CW34
chr11	86716527	86716528	MACS_peak_762	13.00	TCF7L2_standard_ChIP_seq_CW34
chr11	86740765	86740766	MACS_peak_763	8.00	TCF7L2_standard_ChIP_seq_CW34
chr11	86788104	86788105	MACS_peak_764	16.00	TCF7L2_standard_ChIP_seq_CW34
chr11	86839666	86839667	MACS_peak_765	9.00	TCF7L2_standard_ChIP_seq_CW34
chr11	88166894	88166895	MACS_peak_766	8.00	TCF7L2_standard_ChIP_seq_CW34
chr11	90181585	90181586	MACS_peak_767	12.00	TCF7L2_standard_ChIP_seq_CW34
chr11	90470924	90470925	MACS_peak_768	11.00	TCF7L2_standard_ChIP_seq_CW34
chr11	93220545	93220546	MACS_peak_769	6.00	TCF7L2_standard_ChIP_seq_CW34
chr11	95365777	95365778	MACS_peak_770	7.00	TCF7L2_standard_ChIP_seq_CW34
chr11	95773848	95773849	MACS_peak_771	5.00	TCF7L2_standard_ChIP_seq_CW34
chr11	97005023	97005024	MACS_peak_772	5.00	TCF7L2_standard_ChIP_seq_CW34
chr11	98765062	98765063	MACS_peak_773	5.00	TCF7L2_standard_ChIP_seq_CW34
chr11	101812326	101812327	MACS_peak_774	5.00	TCF7L2_standard_ChIP_seq_CW34
chr11	101865121	101865122	MACS_peak_775	29.00	TCF7L2_standard_ChIP_seq_CW34
chr11	101956813	101956814	MACS_peak_776	12.00	TCF7L2_standard_ChIP_seq_CW34
chr11	101982411	101982412	MACS_peak_777	31.00	TCF7L2_standard_ChIP_seq_CW34
chr11	102057718	102057719	MACS_peak_778	24.00	TCF7L2_standard_ChIP_seq_CW34
chr11	102063596	102063597	MACS_peak_779	28.00	TCF7L2_standard_ChIP_seq_CW34
chr11	102150465	102150466	MACS_peak_780	12.00	TCF7L2_standard_ChIP_seq_CW34
chr11	102323500	102323501	MACS_peak_781	7.00	TCF7L2_standard_ChIP_seq_CW34
chr11	106320609	106320610	MACS_peak_782	17.00	TCF7L2_standard_ChIP_seq_CW34
chr11	106540077	106540078	MACS_peak_783	6.00	TCF7L2_standard_ChIP_seq_CW34
chr11	111231024	111231025	MACS_peak_784	11.00	TCF7L2_standard_ChIP_seq_CW34
chr11	111661014	111661015	MACS_peak_785	44.00	TCF7L2_standard_ChIP_seq_CW34
chr11	111671397	111671398	MACS_peak_786	15.00	TCF7L2_standard_ChIP_seq_CW34
chr11	111742181	111742182	MACS_peak_787	17.00	TCF7L2_standard_ChIP_seq_CW34
chr11	111750095	111750096	MACS_peak_788	10.00	TCF7L2_standard_ChIP_seq_CW34
chr11	111806121	111806122	MACS_peak_789	8.00	TCF7L2_standard_ChIP_seq_CW34
chr11	111888464	111888465	MACS_peak_790	26.00	TCF7L2_standard_ChIP_seq_CW34
chr11	113066649	113066650	MACS_peak_791	11.00	TCF7L2_standard_ChIP_seq_CW34
chr11	114128548	114128549	MACS_peak_792	6.00	TCF7L2_standard_ChIP_seq_CW34
chr11	114186817	114186818	MACS_peak_793	14.00	TCF7L2_standard_ChIP_seq_CW34
chr11	114310467	114310468	MACS_peak_794	7.00	TCF7L2_standard_ChIP_seq_CW34
chr11	116298792	116298793	MACS_peak_795	9.00	TCF7L2_standard_ChIP_seq_CW34
chr11	116450914	116450915	MACS_peak_796	18.00	TCF7L2_standard_ChIP_seq_CW34
chr11	116539821	116539822	MACS_peak_797	17.00	TCF7L2_standard_ChIP_seq_CW34
chr11	116663125	116663126	MACS_peak_798	9.00	TCF7L2_standard_ChIP_seq_CW34
chr11	116679168	116679169	MACS_peak_799	11.00	TCF7L2_standard_ChIP_seq_CW34
chr11	116948258	116948259	MACS_peak_800	10.00	TCF7L2_standard_ChIP_seq_CW34
chr11	117620134	117620135	MACS_peak_801	6.00	TCF7L2_standard_ChIP_seq_CW34
chr11	118083057	118083058	MACS_peak_802	25.00	TCF7L2_standard_ChIP_seq_CW34
chr11	118307027	118307028	MACS_peak_803	17.00	TCF7L2_standard_ChIP_seq_CW34
chr11	118544646	118544647	MACS_peak_804	9.00	TCF7L2_standard_ChIP_seq_CW34
chr11	118796045	118796046	MACS_peak_805	18.00	TCF7L2_standard_ChIP_seq_CW34
chr11	118977839	118977840	MACS_peak_806	10.00	TCF7L2_standard_ChIP_seq_CW34
chr11	119076695	119076696	MACS_peak_807	11.00	TCF7L2_standard_ChIP_seq_CW34
chr11	119497237	119497238	MACS_peak_808	15.00	TCF7L2_standard_ChIP_seq_CW34
chr11	120206843	120206844	MACS_peak_809	10.00	TCF7L2_standard_ChIP_seq_CW34
chr11	120403039	120403040	MACS_peak_810	16.00	TCF7L2_standard_ChIP_seq_CW34
chr11	120545480	120545481	MACS_peak_811	20.00	TCF7L2_standard_ChIP_seq_CW34
chr11	120581244	120581245	MACS_peak_812	18.00	TCF7L2_standard_ChIP_seq_CW34
chr11	121435918	121435919	MACS_peak_813	8.00	TCF7L2_standard_ChIP_seq_CW34
chr11	121799911	121799912	MACS_peak_814	29.00	TCF7L2_standard_ChIP_seq_CW34
chr11	122021520	122021521	MACS_peak_815	8.00	TCF7L2_standard_ChIP_seq_CW34
chr11	123547552	123547553	MACS_peak_816	7.00	TCF7L2_standard_ChIP_seq_CW34
chr11	124312972	124312973	MACS_peak_817	11.00	TCF7L2_standard_ChIP_seq_CW34
chr11	124990833	124990834	MACS_peak_818	5.00	TCF7L2_standard_ChIP_seq_CW34
chr11	126017905	126017906	MACS_peak_819	7.00	TCF7L2_standard_ChIP_seq_CW34
chr11	126063130	126063131	MACS_peak_820	21.00	TCF7L2_standard_ChIP_seq_CW34
chr11	128985723	128985724	MACS_peak_821	8.00	TCF7L2_standard_ChIP_seq_CW34
chr11	129613472	129613473	MACS_peak_822	7.00	TCF7L2_standard_ChIP_seq_CW34
chr11	129693806	129693807	MACS_peak_823	15.00	TCF7L2_standard_ChIP_seq_CW34
chr11	129765192	129765193	MACS_peak_824	5.00	TCF7L2_standard_ChIP_seq_CW34
chr11	129872293	129872294	MACS_peak_825	13.00	TCF7L2_standard_ChIP_seq_CW34
chr11	130022953	130022954	MACS_peak_826	9.00	TCF7L2_standard_ChIP_seq_CW34
chr11	130051648	130051649	MACS_peak_827	23.00	TCF7L2_standard_ChIP_seq_CW34
chr11	131295389	131295390	MACS_peak_828	18.00	TCF7L2_standard_ChIP_seq_CW34
chr11	131440787	131440788	MACS_peak_829	5.00	TCF7L2_standard_ChIP_seq_CW34
chr11	132344458	132344459	MACS_peak_830	5.00	TCF7L2_standard_ChIP_seq_CW34
chr12	319609	319610	MACS_peak_831	92.00	TCF7L2_standard_ChIP_seq_CW34
chr12	1987616	1987617	MACS_peak_832	6.00	TCF7L2_standard_ChIP_seq_CW34
chr12	2365131	2365132	MACS_peak_833	6.00	TCF7L2_standard_ChIP_seq_CW34
chr12	2921909	2921910	MACS_peak_834	5.00	TCF7L2_standard_ChIP_seq_CW34
chr12	4124787	4124788	MACS_peak_835	29.00	TCF7L2_standard_ChIP_seq_CW34
chr12	4161811	4161812	MACS_peak_836	6.00	TCF7L2_standard_ChIP_seq_CW34
chr12	4595456	4595457	MACS_peak_837	23.00	TCF7L2_standard_ChIP_seq_CW34
chr12	5677640	5677641	MACS_peak_838	8.00	TCF7L2_standard_ChIP_seq_CW34
chr12	6901786	6901787	MACS_peak_839	13.00	TCF7L2_standard_ChIP_seq_CW34
chr12	7023016	7023017	MACS_peak_840	12.00	TCF7L2_standard_ChIP_seq_CW34
chr12	8004343	8004344	MACS_peak_841	35.00	TCF7L2_standard_ChIP_seq_CW34
chr12	8043393	8043394	MACS_peak_842	12.00	TCF7L2_standard_ChIP_seq_CW34
chr12	8058392	8058393	MACS_peak_843	10.00	TCF7L2_standard_ChIP_seq_CW34
chr12	8135008	8135009	MACS_peak_844	10.00	TCF7L2_standard_ChIP_seq_CW34
chr12	9274833	9274834	MACS_peak_845	36.00	TCF7L2_standard_ChIP_seq_CW34
chr12	9284941	9284942	MACS_peak_846	8.00	TCF7L2_standard_ChIP_seq_CW34
chr12	12420072	12420073	MACS_peak_847	10.00	TCF7L2_standard_ChIP_seq_CW34
chr12	12592579	12592580	MACS_peak_848	7.00	TCF7L2_standard_ChIP_seq_CW34
chr12	12602743	12602744	MACS_peak_849	13.00	TCF7L2_standard_ChIP_seq_CW34
chr12	12699513	12699514	MACS_peak_850	8.00	TCF7L2_standard_ChIP_seq_CW34
chr12	14923602	14923603	MACS_peak_851	20.00	TCF7L2_standard_ChIP_seq_CW34
chr12	15769648	15769649	MACS_peak_852	11.00	TCF7L2_standard_ChIP_seq_CW34
chr12	15942304	15942305	MACS_peak_853	11.00	TCF7L2_standard_ChIP_seq_CW34
chr12	15973200	15973201	MACS_peak_854	19.00	TCF7L2_standard_ChIP_seq_CW34
chr12	18895437	18895438	MACS_peak_855	6.00	TCF7L2_standard_ChIP_seq_CW34
chr12	24070317	24070318	MACS_peak_857	5.00	TCF7L2_standard_ChIP_seq_CW34
chr12	24233488	24233489	MACS_peak_858	9.00	TCF7L2_standard_ChIP_seq_CW34
chr12	24907876	24907877	MACS_peak_859	11.00	TCF7L2_standard_ChIP_seq_CW34
chr12	26889923	26889924	MACS_peak_860	21.00	TCF7L2_standard_ChIP_seq_CW34
chr12	26898041	26898042	MACS_peak_861	20.00	TCF7L2_standard_ChIP_seq_CW34
chr12	26922255	26922256	MACS_peak_862	21.00	TCF7L2_standard_ChIP_seq_CW34
chr12	26968673	26968674	MACS_peak_863	10.00	TCF7L2_standard_ChIP_seq_CW34
chr12	26994211	26994212	MACS_peak_864	6.00	TCF7L2_standard_ChIP_seq_CW34
chr12	27702855	27702856	MACS_peak_865	14.00	TCF7L2_standard_ChIP_seq_CW34
chr12	28913425	28913426	MACS_peak_866	6.00	TCF7L2_standard_ChIP_seq_CW34
chr12	30661642	30661643	MACS_peak_867	35.00	TCF7L2_standard_ChIP_seq_CW34
chr12	30741602	30741603	MACS_peak_868	5.00	TCF7L2_standard_ChIP_seq_CW34
chr12	30752743	30752744	MACS_peak_869	16.00	TCF7L2_standard_ChIP_seq_CW34
chr12	30774898	30774899	MACS_peak_870	8.00	TCF7L2_standard_ChIP_seq_CW34
chr12	31227037	31227038	MACS_peak_871	5.00	TCF7L2_standard_ChIP_seq_CW34
chr12	31477258	31477259	MACS_peak_872	17.00	TCF7L2_standard_ChIP_seq_CW34
chr12	31902385	31902386	MACS_peak_873	14.00	TCF7L2_standard_ChIP_seq_CW34
chr12	31926210	31926211	MACS_peak_874	8.00	TCF7L2_standard_ChIP_seq_CW34
chr12	31998721	31998722	MACS_peak_875	14.00	TCF7L2_standard_ChIP_seq_CW34
chr12	32101986	32101987	MACS_peak_876	11.00	TCF7L2_standard_ChIP_seq_CW34
chr12	32112178	32112179	MACS_peak_877	35.00	TCF7L2_standard_ChIP_seq_CW34
chr12	32340295	32340296	MACS_peak_878	6.00	TCF7L2_standard_ChIP_seq_CW34
chr12	33046657	33046658	MACS_peak_879	11.00	TCF7L2_standard_ChIP_seq_CW34
chr12	38590847	38590848	MACS_peak_880	10.00	TCF7L2_standard_ChIP_seq_CW34
chr12	42375031	42375032	MACS_peak_881	6.00	TCF7L2_standard_ChIP_seq_CW34
chr12	42632131	42632132	MACS_peak_882	17.00	TCF7L2_standard_ChIP_seq_CW34
chr12	42877865	42877866	MACS_peak_883	9.00	TCF7L2_standard_ChIP_seq_CW34
chr12	44241916	44241917	MACS_peak_884	8.00	TCF7L2_standard_ChIP_seq_CW34
chr12	44389235	44389236	MACS_peak_885	6.00	TCF7L2_standard_ChIP_seq_CW34
chr12	45609812	45609813	MACS_peak_886	8.00	TCF7L2_standard_ChIP_seq_CW34
chr12	46124687	46124688	MACS_peak_887	9.00	TCF7L2_standard_ChIP_seq_CW34
chr12	46384985	46384986	MACS_peak_888	8.00	TCF7L2_standard_ChIP_seq_CW34
chr12	46580646	46580647	MACS_peak_889	7.00	TCF7L2_standard_ChIP_seq_CW34
chr12	46937491	46937492	MACS_peak_890	7.00	TCF7L2_standard_ChIP_seq_CW34
chr12	48417014	48417015	MACS_peak_891	9.00	TCF7L2_standard_ChIP_seq_CW34
chr12	48517101	48517102	MACS_peak_892	13.00	TCF7L2_standard_ChIP_seq_CW34
chr12	49304205	49304206	MACS_peak_893	7.00	TCF7L2_standard_ChIP_seq_CW34
chr12	49454559	49454560	MACS_peak_894	9.00	TCF7L2_standard_ChIP_seq_CW34
chr12	50101488	50101489	MACS_peak_895	6.00	TCF7L2_standard_ChIP_seq_CW34
chr12	50281906	50281907	MACS_peak_896	10.00	TCF7L2_standard_ChIP_seq_CW34
chr12	51664060	51664061	MACS_peak_897	7.00	TCF7L2_standard_ChIP_seq_CW34
chr12	52444923	52444924	MACS_peak_898	24.00	TCF7L2_standard_ChIP_seq_CW34
chr12	53278357	53278358	MACS_peak_899	13.00	TCF7L2_standard_ChIP_seq_CW34
chr12	53319937	53319938	MACS_peak_900	12.00	TCF7L2_standard_ChIP_seq_CW34
chr12	53363744	53363745	MACS_peak_901	11.00	TCF7L2_standard_ChIP_seq_CW34
chr12	53443695	53443696	MACS_peak_902	7.00	TCF7L2_standard_ChIP_seq_CW34
chr12	53497905	53497906	MACS_peak_903	8.00	TCF7L2_standard_ChIP_seq_CW34
chr12	53568711	53568712	MACS_peak_904	9.00	TCF7L2_standard_ChIP_seq_CW34
chr12	53719020	53719021	MACS_peak_905	34.00	TCF7L2_standard_ChIP_seq_CW34
chr12	53932117	53932118	MACS_peak_906	5.00	TCF7L2_standard_ChIP_seq_CW34
chr12	54219244	54219245	MACS_peak_907	12.00	TCF7L2_standard_ChIP_seq_CW34
chr12	54547565	54547566	MACS_peak_908	11.00	TCF7L2_standard_ChIP_seq_CW34
chr12	54695306	54695307	MACS_peak_909	8.00	TCF7L2_standard_ChIP_seq_CW34
chr12	55138600	55138601	MACS_peak_910	8.00	TCF7L2_standard_ChIP_seq_CW34
chr12	55893758	55893759	MACS_peak_911	10.00	TCF7L2_standard_ChIP_seq_CW34
chr12	56098802	56098803	MACS_peak_912	16.00	TCF7L2_standard_ChIP_seq_CW34
chr12	56695727	56695728	MACS_peak_913	36.00	TCF7L2_standard_ChIP_seq_CW34
chr12	57521876	57521877	MACS_peak_914	9.00	TCF7L2_standard_ChIP_seq_CW34
chr12	57532654	57532655	MACS_peak_915	13.00	TCF7L2_standard_ChIP_seq_CW34
chr12	57543910	57543911	MACS_peak_916	36.00	TCF7L2_standard_ChIP_seq_CW34
chr12	57890727	57890728	MACS_peak_917	8.00	TCF7L2_standard_ChIP_seq_CW34
chr12	57916292	57916293	MACS_peak_918	19.00	TCF7L2_standard_ChIP_seq_CW34
chr12	58685071	58685072	MACS_peak_919	8.00	TCF7L2_standard_ChIP_seq_CW34
chr12	63285867	63285868	MACS_peak_920	28.00	TCF7L2_standard_ChIP_seq_CW34
chr12	64256481	64256482	MACS_peak_921	7.00	TCF7L2_standard_ChIP_seq_CW34
chr12	65050384	65050385	MACS_peak_922	9.00	TCF7L2_standard_ChIP_seq_CW34
chr12	65493180	65493181	MACS_peak_923	6.00	TCF7L2_standard_ChIP_seq_CW34
chr12	65729303	65729304	MACS_peak_924	4.00	TCF7L2_standard_ChIP_seq_CW34
chr12	66136238	66136239	MACS_peak_925	14.00	TCF7L2_standard_ChIP_seq_CW34
chr12	66324055	66324056	MACS_peak_926	11.00	TCF7L2_standard_ChIP_seq_CW34
chr12	66331102	66331103	MACS_peak_927	7.00	TCF7L2_standard_ChIP_seq_CW34
chr12	66443077	66443078	MACS_peak_928	25.00	TCF7L2_standard_ChIP_seq_CW34
chr12	70132920	70132921	MACS_peak_929	8.00	TCF7L2_standard_ChIP_seq_CW34
chr12	71834773	71834774	MACS_peak_930	78.00	TCF7L2_standard_ChIP_seq_CW34
chr12	71886668	71886669	MACS_peak_931	7.00	TCF7L2_standard_ChIP_seq_CW34
chr12	71946982	71946983	MACS_peak_932	20.00	TCF7L2_standard_ChIP_seq_CW34
chr12	76029598	76029599	MACS_peak_933	10.00	TCF7L2_standard_ChIP_seq_CW34
chr12	76060312	76060313	MACS_peak_934	8.00	TCF7L2_standard_ChIP_seq_CW34
chr12	76106195	76106196	MACS_peak_935	7.00	TCF7L2_standard_ChIP_seq_CW34
chr12	76160604	76160605	MACS_peak_936	10.00	TCF7L2_standard_ChIP_seq_CW34
chr12	76338123	76338124	MACS_peak_937	7.00	TCF7L2_standard_ChIP_seq_CW34
chr12	76354039	76354040	MACS_peak_938	26.00	TCF7L2_standard_ChIP_seq_CW34
chr12	76384281	76384282	MACS_peak_939	24.00	TCF7L2_standard_ChIP_seq_CW34
chr12	76425401	76425402	MACS_peak_940	9.00	TCF7L2_standard_ChIP_seq_CW34
chr12	76477902	76477903	MACS_peak_941	10.00	TCF7L2_standard_ChIP_seq_CW34
chr12	77015560	77015561	MACS_peak_942	7.00	TCF7L2_standard_ChIP_seq_CW34
chr12	81480369	81480370	MACS_peak_943	10.00	TCF7L2_standard_ChIP_seq_CW34
chr12	81537126	81537127	MACS_peak_944	20.00	TCF7L2_standard_ChIP_seq_CW34
chr12	89320157	89320158	MACS_peak_945	6.00	TCF7L2_standard_ChIP_seq_CW34
chr12	89777163	89777164	MACS_peak_946	15.00	TCF7L2_standard_ChIP_seq_CW34
chr12	89936936	89936937	MACS_peak_947	10.00	TCF7L2_standard_ChIP_seq_CW34
chr12	93339651	93339652	MACS_peak_948	12.00	TCF7L2_standard_ChIP_seq_CW34
chr12	93414275	93414276	MACS_peak_949	29.00	TCF7L2_standard_ChIP_seq_CW34
chr12	93535174	93535175	MACS_peak_950	6.00	TCF7L2_standard_ChIP_seq_CW34
chr12	93638093	93638094	MACS_peak_951	8.00	TCF7L2_standard_ChIP_seq_CW34
chr12	93667878	93667879	MACS_peak_952	15.00	TCF7L2_standard_ChIP_seq_CW34
chr12	93920837	93920838	MACS_peak_953	10.00	TCF7L2_standard_ChIP_seq_CW34
chr12	93964689	93964690	MACS_peak_954	13.00	TCF7L2_standard_ChIP_seq_CW34
chr12	94472367	94472368	MACS_peak_955	13.00	TCF7L2_standard_ChIP_seq_CW34
chr12	94577587	94577588	MACS_peak_956	11.00	TCF7L2_standard_ChIP_seq_CW34
chr12	95734182	95734183	MACS_peak_957	11.00	TCF7L2_standard_ChIP_seq_CW34
chr12	96156073	96156074	MACS_peak_958	36.00	TCF7L2_standard_ChIP_seq_CW34
chr12	96336845	96336846	MACS_peak_959	11.00	TCF7L2_standard_ChIP_seq_CW34
chr12	96343767	96343768	MACS_peak_960	10.00	TCF7L2_standard_ChIP_seq_CW34
chr12	96370303	96370304	MACS_peak_961	11.00	TCF7L2_standard_ChIP_seq_CW34
chr12	96511480	96511481	MACS_peak_962	14.00	TCF7L2_standard_ChIP_seq_CW34
chr12	97064383	97064384	MACS_peak_963	5.00	TCF7L2_standard_ChIP_seq_CW34
chr12	97774413	97774414	MACS_peak_964	8.00	TCF7L2_standard_ChIP_seq_CW34
chr12	97962795	97962796	MACS_peak_965	8.00	TCF7L2_standard_ChIP_seq_CW34
chr12	98897810	98897811	MACS_peak_966	10.00	TCF7L2_standard_ChIP_seq_CW34
chr12	100073505	100073506	MACS_peak_967	6.00	TCF7L2_standard_ChIP_seq_CW34
chr12	100536228	100536229	MACS_peak_968	6.00	TCF7L2_standard_ChIP_seq_CW34
chr12	100710844	100710845	MACS_peak_969	8.00	TCF7L2_standard_ChIP_seq_CW34
chr12	100831279	100831280	MACS_peak_970	9.00	TCF7L2_standard_ChIP_seq_CW34
chr12	102001987	102001988	MACS_peak_971	27.00	TCF7L2_standard_ChIP_seq_CW34
chr12	102453405	102453406	MACS_peak_972	14.00	TCF7L2_standard_ChIP_seq_CW34
chr12	103060022	103060023	MACS_peak_973	6.00	TCF7L2_standard_ChIP_seq_CW34
chr12	103292917	103292918	MACS_peak_974	17.00	TCF7L2_standard_ChIP_seq_CW34
chr12	104162017	104162018	MACS_peak_975	5.00	TCF7L2_standard_ChIP_seq_CW34
chr12	104232488	104232489	MACS_peak_976	9.00	TCF7L2_standard_ChIP_seq_CW34
chr12	104350927	104350928	MACS_peak_977	5.00	TCF7L2_standard_ChIP_seq_CW34
chr12	106492604	106492605	MACS_peak_978	27.00	TCF7L2_standard_ChIP_seq_CW34
chr12	106751872	106751873	MACS_peak_979	5.00	TCF7L2_standard_ChIP_seq_CW34
chr12	107249216	107249217	MACS_peak_980	12.00	TCF7L2_standard_ChIP_seq_CW34
chr12	107746240	107746241	MACS_peak_981	28.00	TCF7L2_standard_ChIP_seq_CW34
chr12	108707752	108707753	MACS_peak_982	9.00	TCF7L2_standard_ChIP_seq_CW34
chr12	109058684	109058685	MACS_peak_983	9.00	TCF7L2_standard_ChIP_seq_CW34
chr12	109490395	109490396	MACS_peak_984	6.00	TCF7L2_standard_ChIP_seq_CW34
chr12	109715839	109715840	MACS_peak_985	8.00	TCF7L2_standard_ChIP_seq_CW34
chr12	110134038	110134039	MACS_peak_986	32.00	TCF7L2_standard_ChIP_seq_CW34
chr12	110144881	110144882	MACS_peak_987	9.00	TCF7L2_standard_ChIP_seq_CW34
chr12	110529361	110529362	MACS_peak_988	6.00	TCF7L2_standard_ChIP_seq_CW34
chr12	112054235	112054236	MACS_peak_989	21.00	TCF7L2_standard_ChIP_seq_CW34
chr12	112723729	112723730	MACS_peak_990	40.00	TCF7L2_standard_ChIP_seq_CW34
chr12	112955363	112955364	MACS_peak_991	6.00	TCF7L2_standard_ChIP_seq_CW34
chr12	113364430	113364431	MACS_peak_992	10.00	TCF7L2_standard_ChIP_seq_CW34
chr12	113708133	113708134	MACS_peak_993	9.00	TCF7L2_standard_ChIP_seq_CW34
chr12	114204014	114204015	MACS_peak_994	18.00	TCF7L2_standard_ChIP_seq_CW34
chr12	114246243	114246244	MACS_peak_995	8.00	TCF7L2_standard_ChIP_seq_CW34
chr12	114428616	114428617	MACS_peak_996	12.00	TCF7L2_standard_ChIP_seq_CW34
chr12	114475227	114475228	MACS_peak_997	8.00	TCF7L2_standard_ChIP_seq_CW34
chr12	115094158	115094159	MACS_peak_998	14.00	TCF7L2_standard_ChIP_seq_CW34
chr12	115176826	115176827	MACS_peak_999	48.00	TCF7L2_standard_ChIP_seq_CW34
chr12	115237622	115237623	MACS_peak_1000	56.00	TCF7L2_standard_ChIP_seq_CW34
chr12	115296329	115296330	MACS_peak_1001	7.00	TCF7L2_standard_ChIP_seq_CW34
chr12	115527374	115527375	MACS_peak_1002	23.00	TCF7L2_standard_ChIP_seq_CW34
chr12	115871248	115871249	MACS_peak_1003	8.00	TCF7L2_standard_ChIP_seq_CW34
chr12	117074862	117074863	MACS_peak_1004	16.00	TCF7L2_standard_ChIP_seq_CW34
chr12	117096730	117096731	MACS_peak_1005	7.00	TCF7L2_standard_ChIP_seq_CW34
chr12	117257295	117257296	MACS_peak_1006	8.00	TCF7L2_standard_ChIP_seq_CW34
chr12	117536573	117536574	MACS_peak_1007	16.00	TCF7L2_standard_ChIP_seq_CW34
chr12	118474332	118474333	MACS_peak_1008	7.00	TCF7L2_standard_ChIP_seq_CW34
chr12	119152516	119152517	MACS_peak_1009	41.00	TCF7L2_standard_ChIP_seq_CW34
chr12	119803393	119803394	MACS_peak_1010	7.00	TCF7L2_standard_ChIP_seq_CW34
chr12	120632633	120632634	MACS_peak_1011	6.00	TCF7L2_standard_ChIP_seq_CW34
chr12	120740390	120740391	MACS_peak_1012	7.00	TCF7L2_standard_ChIP_seq_CW34
chr12	121928680	121928681	MACS_peak_1013	26.00	TCF7L2_standard_ChIP_seq_CW34
chr12	121989076	121989077	MACS_peak_1014	38.00	TCF7L2_standard_ChIP_seq_CW34
chr12	122020732	122020733	MACS_peak_1015	7.00	TCF7L2_standard_ChIP_seq_CW34
chr12	122150910	122150911	MACS_peak_1016	4.00	TCF7L2_standard_ChIP_seq_CW34
chr12	122238123	122238124	MACS_peak_1017	22.00	TCF7L2_standard_ChIP_seq_CW34
chr12	122314388	122314389	MACS_peak_1018	43.00	TCF7L2_standard_ChIP_seq_CW34
chr12	123560256	123560257	MACS_peak_1019	7.00	TCF7L2_standard_ChIP_seq_CW34
chr12	123634411	123634412	MACS_peak_1020	7.00	TCF7L2_standard_ChIP_seq_CW34
chr12	123933173	123933174	MACS_peak_1021	18.00	TCF7L2_standard_ChIP_seq_CW34
chr12	124615377	124615378	MACS_peak_1022	12.00	TCF7L2_standard_ChIP_seq_CW34
chr12	124763294	124763295	MACS_peak_1023	7.00	TCF7L2_standard_ChIP_seq_CW34
chr12	125050515	125050516	MACS_peak_1024	12.00	TCF7L2_standard_ChIP_seq_CW34
chr12	125093835	125093836	MACS_peak_1025	71.00	TCF7L2_standard_ChIP_seq_CW34
chr12	125306374	125306375	MACS_peak_1026	9.00	TCF7L2_standard_ChIP_seq_CW34
chr12	125323142	125323143	MACS_peak_1027	7.00	TCF7L2_standard_ChIP_seq_CW34
chr12	125402182	125402183	MACS_peak_1028	10.00	TCF7L2_standard_ChIP_seq_CW34
chr12	127614456	127614457	MACS_peak_1029	13.00	TCF7L2_standard_ChIP_seq_CW34
chr12	130529855	130529856	MACS_peak_1031	16.00	TCF7L2_standard_ChIP_seq_CW34
chr12	131451309	131451310	MACS_peak_1032	8.00	TCF7L2_standard_ChIP_seq_CW34
chr12	132195419	132195420	MACS_peak_1033	7.00	TCF7L2_standard_ChIP_seq_CW34
chr12	133185501	133185502	MACS_peak_1034	13.00	TCF7L2_standard_ChIP_seq_CW34
chr12	133405856	133405857	MACS_peak_1035	14.00	TCF7L2_standard_ChIP_seq_CW34
chr12	133443111	133443112	MACS_peak_1036	9.00	TCF7L2_standard_ChIP_seq_CW34
chr12	133614185	133614186	MACS_peak_1037	12.00	TCF7L2_standard_ChIP_seq_CW34
chr13	20171188	20171189	MACS_peak_1038	33.00	TCF7L2_standard_ChIP_seq_CW34
chr13	21495856	21495857	MACS_peak_1039	11.00	TCF7L2_standard_ChIP_seq_CW34
chr13	21527817	21527818	MACS_peak_1040	32.00	TCF7L2_standard_ChIP_seq_CW34
chr13	21675488	21675489	MACS_peak_1041	9.00	TCF7L2_standard_ChIP_seq_CW34
chr13	23367858	23367859	MACS_peak_1042	15.00	TCF7L2_standard_ChIP_seq_CW34
chr13	23422911	23422912	MACS_peak_1043	6.00	TCF7L2_standard_ChIP_seq_CW34
chr13	24144784	24144785	MACS_peak_1044	18.00	TCF7L2_standard_ChIP_seq_CW34
chr13	24189919	24189920	MACS_peak_1045	10.00	TCF7L2_standard_ChIP_seq_CW34
chr13	24203792	24203793	MACS_peak_1046	19.00	TCF7L2_standard_ChIP_seq_CW34
chr13	24206518	24206519	MACS_peak_1047	13.00	TCF7L2_standard_ChIP_seq_CW34
chr13	24228798	24228799	MACS_peak_1048	9.00	TCF7L2_standard_ChIP_seq_CW34
chr13	25305620	25305621	MACS_peak_1049	6.00	TCF7L2_standard_ChIP_seq_CW34
chr13	27744680	27744681	MACS_peak_1050	7.00	TCF7L2_standard_ChIP_seq_CW34
chr13	27957834	27957835	MACS_peak_1051	8.00	TCF7L2_standard_ChIP_seq_CW34
chr13	28200194	28200195	MACS_peak_1052	6.00	TCF7L2_standard_ChIP_seq_CW34
chr13	29615686	29615687	MACS_peak_1053	6.00	TCF7L2_standard_ChIP_seq_CW34
chr13	30146118	30146119	MACS_peak_1054	12.00	TCF7L2_standard_ChIP_seq_CW34
chr13	30982722	30982723	MACS_peak_1055	13.00	TCF7L2_standard_ChIP_seq_CW34
chr13	31407124	31407125	MACS_peak_1056	10.00	TCF7L2_standard_ChIP_seq_CW34
chr13	32620803	32620804	MACS_peak_1057	15.00	TCF7L2_standard_ChIP_seq_CW34
chr13	32713587	32713588	MACS_peak_1058	7.00	TCF7L2_standard_ChIP_seq_CW34
chr13	33700340	33700341	MACS_peak_1059	13.00	TCF7L2_standard_ChIP_seq_CW34
chr13	33834728	33834729	MACS_peak_1060	30.00	TCF7L2_standard_ChIP_seq_CW34
chr13	33950789	33950790	MACS_peak_1061	7.00	TCF7L2_standard_ChIP_seq_CW34
chr13	34217136	34217137	MACS_peak_1062	19.00	TCF7L2_standard_ChIP_seq_CW34
chr13	34446232	34446233	MACS_peak_1063	9.00	TCF7L2_standard_ChIP_seq_CW34
chr13	34531782	34531783	MACS_peak_1064	6.00	TCF7L2_standard_ChIP_seq_CW34
chr13	34543420	34543421	MACS_peak_1065	12.00	TCF7L2_standard_ChIP_seq_CW34
chr13	34586125	34586126	MACS_peak_1066	12.00	TCF7L2_standard_ChIP_seq_CW34
chr13	34594144	34594145	MACS_peak_1067	30.00	TCF7L2_standard_ChIP_seq_CW34
chr13	39556614	39556615	MACS_peak_1068	8.00	TCF7L2_standard_ChIP_seq_CW34
chr13	41114756	41114757	MACS_peak_1069	8.00	TCF7L2_standard_ChIP_seq_CW34
chr13	41138180	41138181	MACS_peak_1070	8.00	TCF7L2_standard_ChIP_seq_CW34
chr13	41360744	41360745	MACS_peak_1071	7.00	TCF7L2_standard_ChIP_seq_CW34
chr13	41455104	41455105	MACS_peak_1072	8.00	TCF7L2_standard_ChIP_seq_CW34
chr13	41582725	41582726	MACS_peak_1073	8.00	TCF7L2_standard_ChIP_seq_CW34
chr13	41863919	41863920	MACS_peak_1074	12.00	TCF7L2_standard_ChIP_seq_CW34
chr13	42180995	42180996	MACS_peak_1075	14.00	TCF7L2_standard_ChIP_seq_CW34
chr13	43631953	43631954	MACS_peak_1076	17.00	TCF7L2_standard_ChIP_seq_CW34
chr13	46683831	46683832	MACS_peak_1077	12.00	TCF7L2_standard_ChIP_seq_CW34
chr13	48611754	48611755	MACS_peak_1078	8.00	TCF7L2_standard_ChIP_seq_CW34
chr13	49200360	49200361	MACS_peak_1079	10.00	TCF7L2_standard_ChIP_seq_CW34
chr13	49946235	49946236	MACS_peak_1080	13.00	TCF7L2_standard_ChIP_seq_CW34
chr13	50219878	50219879	MACS_peak_1081	6.00	TCF7L2_standard_ChIP_seq_CW34
chr13	50413349	50413350	MACS_peak_1082	29.00	TCF7L2_standard_ChIP_seq_CW34
chr13	50432921	50432922	MACS_peak_1083	8.00	TCF7L2_standard_ChIP_seq_CW34
chr13	50705503	50705504	MACS_peak_1084	8.00	TCF7L2_standard_ChIP_seq_CW34
chr13	51086856	51086857	MACS_peak_1085	8.00	TCF7L2_standard_ChIP_seq_CW34
chr13	51697162	51697163	MACS_peak_1086	8.00	TCF7L2_standard_ChIP_seq_CW34
chr13	52094153	52094154	MACS_peak_1087	7.00	TCF7L2_standard_ChIP_seq_CW34
chr13	52322289	52322290	MACS_peak_1088	5.00	TCF7L2_standard_ChIP_seq_CW34
chr13	52599732	52599733	MACS_peak_1089	12.00	TCF7L2_standard_ChIP_seq_CW34
chr13	52781719	52781720	MACS_peak_1090	9.00	TCF7L2_standard_ChIP_seq_CW34
chr13	52970093	52970094	MACS_peak_1091	6.00	TCF7L2_standard_ChIP_seq_CW34
chr13	61081197	61081198	MACS_peak_1092	9.00	TCF7L2_standard_ChIP_seq_CW34
chr13	65738858	65738859	MACS_peak_1093	5.00	TCF7L2_standard_ChIP_seq_CW34
chr13	68954923	68954924	MACS_peak_1094	11.00	TCF7L2_standard_ChIP_seq_CW34
chr13	71872918	71872919	MACS_peak_1095	9.00	TCF7L2_standard_ChIP_seq_CW34
chr13	72440880	72440881	MACS_peak_1096	10.00	TCF7L2_standard_ChIP_seq_CW34
chr13	73664709	73664710	MACS_peak_1097	19.00	TCF7L2_standard_ChIP_seq_CW34
chr13	74736464	74736465	MACS_peak_1098	15.00	TCF7L2_standard_ChIP_seq_CW34
chr13	75914475	75914476	MACS_peak_1099	11.00	TCF7L2_standard_ChIP_seq_CW34
chr13	76053071	76053072	MACS_peak_1100	15.00	TCF7L2_standard_ChIP_seq_CW34
chr13	76210550	76210551	MACS_peak_1101	18.00	TCF7L2_standard_ChIP_seq_CW34
chr13	76334754	76334755	MACS_peak_1102	8.00	TCF7L2_standard_ChIP_seq_CW34
chr13	78052647	78052648	MACS_peak_1103	10.00	TCF7L2_standard_ChIP_seq_CW34
chr13	80043874	80043875	MACS_peak_1104	6.00	TCF7L2_standard_ChIP_seq_CW34
chr13	91518970	91518971	MACS_peak_1105	6.00	TCF7L2_standard_ChIP_seq_CW34
chr13	91558333	91558334	MACS_peak_1106	7.00	TCF7L2_standard_ChIP_seq_CW34
chr13	93901678	93901679	MACS_peak_1107	6.00	TCF7L2_standard_ChIP_seq_CW34
chr13	94618719	94618720	MACS_peak_1108	16.00	TCF7L2_standard_ChIP_seq_CW34
chr13	94982221	94982222	MACS_peak_1109	10.00	TCF7L2_standard_ChIP_seq_CW34
chr13	97578296	97578297	MACS_peak_1110	9.00	TCF7L2_standard_ChIP_seq_CW34
chr13	97599339	97599340	MACS_peak_1111	10.00	TCF7L2_standard_ChIP_seq_CW34
chr13	97885957	97885958	MACS_peak_1112	9.00	TCF7L2_standard_ChIP_seq_CW34
chr13	98813777	98813778	MACS_peak_1113	17.00	TCF7L2_standard_ChIP_seq_CW34
chr13	100608886	100608887	MACS_peak_1114	9.00	TCF7L2_standard_ChIP_seq_CW34
chr13	100942744	100942745	MACS_peak_1115	6.00	TCF7L2_standard_ChIP_seq_CW34
chr13	100967045	100967046	MACS_peak_1116	27.00	TCF7L2_standard_ChIP_seq_CW34
chr13	101324246	101324247	MACS_peak_1117	7.00	TCF7L2_standard_ChIP_seq_CW34
chr13	101441946	101441947	MACS_peak_1118	6.00	TCF7L2_standard_ChIP_seq_CW34
chr13	110039768	110039769	MACS_peak_1119	6.00	TCF7L2_standard_ChIP_seq_CW34
chr13	110239015	110239016	MACS_peak_1120	8.00	TCF7L2_standard_ChIP_seq_CW34
chr13	110373420	110373421	MACS_peak_1121	8.00	TCF7L2_standard_ChIP_seq_CW34
chr13	111138111	111138112	MACS_peak_1122	29.00	TCF7L2_standard_ChIP_seq_CW34
chr13	111142892	111142893	MACS_peak_1123	23.00	TCF7L2_standard_ChIP_seq_CW34
chr13	113411621	113411622	MACS_peak_1124	17.00	TCF7L2_standard_ChIP_seq_CW34
chr13	113677194	113677195	MACS_peak_1125	9.00	TCF7L2_standard_ChIP_seq_CW34
chr13	113793546	113793547	MACS_peak_1126	7.00	TCF7L2_standard_ChIP_seq_CW34
chr13	114520139	114520140	MACS_peak_1127	29.00	TCF7L2_standard_ChIP_seq_CW34
chr13	115047917	115047918	MACS_peak_1128	11.00	TCF7L2_standard_ChIP_seq_CW34
chr14	19124345	19124346	MACS_peak_1129	8.00	TCF7L2_standard_ChIP_seq_CW34
chr14	21082007	21082008	MACS_peak_1130	9.00	TCF7L2_standard_ChIP_seq_CW34
chr14	21493107	21493108	MACS_peak_1131	42.00	TCF7L2_standard_ChIP_seq_CW34
chr14	21522715	21522716	MACS_peak_1132	35.00	TCF7L2_standard_ChIP_seq_CW34
chr14	21572070	21572071	MACS_peak_1133	15.00	TCF7L2_standard_ChIP_seq_CW34
chr14	23169503	23169504	MACS_peak_1134	12.00	TCF7L2_standard_ChIP_seq_CW34
chr14	23372494	23372495	MACS_peak_1135	42.00	TCF7L2_standard_ChIP_seq_CW34
chr14	23451242	23451243	MACS_peak_1136	15.00	TCF7L2_standard_ChIP_seq_CW34
chr14	23476320	23476321	MACS_peak_1137	10.00	TCF7L2_standard_ChIP_seq_CW34
chr14	23641024	23641025	MACS_peak_1138	14.00	TCF7L2_standard_ChIP_seq_CW34
chr14	23770663	23770664	MACS_peak_1139	28.00	TCF7L2_standard_ChIP_seq_CW34
chr14	24098684	24098685	MACS_peak_1140	10.00	TCF7L2_standard_ChIP_seq_CW34
chr14	24158971	24158972	MACS_peak_1141	12.00	TCF7L2_standard_ChIP_seq_CW34
chr14	24353311	24353312	MACS_peak_1142	19.00	TCF7L2_standard_ChIP_seq_CW34
chr14	24505454	24505455	MACS_peak_1143	20.00	TCF7L2_standard_ChIP_seq_CW34
chr14	24712173	24712174	MACS_peak_1144	12.00	TCF7L2_standard_ChIP_seq_CW34
chr14	24835475	24835476	MACS_peak_1145	12.00	TCF7L2_standard_ChIP_seq_CW34
chr14	25330811	25330812	MACS_peak_1146	10.00	TCF7L2_standard_ChIP_seq_CW34
chr14	25448597	25448598	MACS_peak_1147	18.00	TCF7L2_standard_ChIP_seq_CW34
chr14	28304260	28304261	MACS_peak_1148	8.00	TCF7L2_standard_ChIP_seq_CW34
chr14	29191293	29191294	MACS_peak_1149	9.00	TCF7L2_standard_ChIP_seq_CW34
chr14	29931847	29931848	MACS_peak_1150	7.00	TCF7L2_standard_ChIP_seq_CW34
chr14	30873193	30873194	MACS_peak_1151	13.00	TCF7L2_standard_ChIP_seq_CW34
chr14	30878769	30878770	MACS_peak_1152	23.00	TCF7L2_standard_ChIP_seq_CW34
chr14	30937787	30937788	MACS_peak_1153	10.00	TCF7L2_standard_ChIP_seq_CW34
chr14	31027914	31027915	MACS_peak_1154	10.00	TCF7L2_standard_ChIP_seq_CW34
chr14	31344054	31344055	MACS_peak_1155	7.00	TCF7L2_standard_ChIP_seq_CW34
chr14	31675056	31675057	MACS_peak_1156	38.00	TCF7L2_standard_ChIP_seq_CW34
chr14	31699128	31699129	MACS_peak_1157	24.00	TCF7L2_standard_ChIP_seq_CW34
chr14	31731330	31731331	MACS_peak_1158	14.00	TCF7L2_standard_ChIP_seq_CW34
chr14	31749802	31749803	MACS_peak_1159	13.00	TCF7L2_standard_ChIP_seq_CW34
chr14	31889584	31889585	MACS_peak_1160	11.00	TCF7L2_standard_ChIP_seq_CW34
chr14	32088260	32088261	MACS_peak_1161	17.00	TCF7L2_standard_ChIP_seq_CW34
chr14	32217635	32217636	MACS_peak_1162	11.00	TCF7L2_standard_ChIP_seq_CW34
chr14	32670948	32670949	MACS_peak_1163	11.00	TCF7L2_standard_ChIP_seq_CW34
chr14	32747415	32747416	MACS_peak_1164	12.00	TCF7L2_standard_ChIP_seq_CW34
chr14	33403890	33403891	MACS_peak_1165	18.00	TCF7L2_standard_ChIP_seq_CW34
chr14	34493634	34493635	MACS_peak_1166	14.00	TCF7L2_standard_ChIP_seq_CW34
chr14	34837661	34837662	MACS_peak_1167	18.00	TCF7L2_standard_ChIP_seq_CW34
chr14	35099066	35099067	MACS_peak_1168	10.00	TCF7L2_standard_ChIP_seq_CW34
chr14	35343555	35343556	MACS_peak_1169	17.00	TCF7L2_standard_ChIP_seq_CW34
chr14	35628626	35628627	MACS_peak_1170	11.00	TCF7L2_standard_ChIP_seq_CW34
chr14	35761512	35761513	MACS_peak_1171	31.00	TCF7L2_standard_ChIP_seq_CW34
chr14	35816778	35816779	MACS_peak_1172	70.00	TCF7L2_standard_ChIP_seq_CW34
chr14	35874257	35874258	MACS_peak_1173	16.00	TCF7L2_standard_ChIP_seq_CW34
chr14	36205166	36205167	MACS_peak_1174	8.00	TCF7L2_standard_ChIP_seq_CW34
chr14	36678076	36678077	MACS_peak_1175	86.00	TCF7L2_standard_ChIP_seq_CW34
chr14	36923971	36923972	MACS_peak_1176	17.00	TCF7L2_standard_ChIP_seq_CW34
chr14	37145613	37145614	MACS_peak_1177	9.00	TCF7L2_standard_ChIP_seq_CW34
chr14	37507415	37507416	MACS_peak_1178	13.00	TCF7L2_standard_ChIP_seq_CW34
chr14	37641209	37641210	MACS_peak_1179	7.00	TCF7L2_standard_ChIP_seq_CW34
chr14	37764913	37764914	MACS_peak_1180	10.00	TCF7L2_standard_ChIP_seq_CW34
chr14	37935949	37935950	MACS_peak_1181	14.00	TCF7L2_standard_ChIP_seq_CW34
chr14	38016300	38016301	MACS_peak_1182	14.00	TCF7L2_standard_ChIP_seq_CW34
chr14	38058237	38058238	MACS_peak_1183	12.00	TCF7L2_standard_ChIP_seq_CW34
chr14	38253632	38253633	MACS_peak_1184	12.00	TCF7L2_standard_ChIP_seq_CW34
chr14	38334482	38334483	MACS_peak_1185	76.00	TCF7L2_standard_ChIP_seq_CW34
chr14	39075156	39075157	MACS_peak_1186	8.00	TCF7L2_standard_ChIP_seq_CW34
chr14	39493491	39493492	MACS_peak_1187	13.00	TCF7L2_standard_ChIP_seq_CW34
chr14	39736487	39736488	MACS_peak_1188	12.00	TCF7L2_standard_ChIP_seq_CW34
chr14	39901466	39901467	MACS_peak_1189	18.00	TCF7L2_standard_ChIP_seq_CW34
chr14	42132585	42132586	MACS_peak_1190	11.00	TCF7L2_standard_ChIP_seq_CW34
chr14	43588656	43588657	MACS_peak_1191	9.00	TCF7L2_standard_ChIP_seq_CW34
chr14	43799471	43799472	MACS_peak_1192	9.00	TCF7L2_standard_ChIP_seq_CW34
chr14	44250021	44250022	MACS_peak_1193	19.00	TCF7L2_standard_ChIP_seq_CW34
chr14	50063153	50063154	MACS_peak_1194	12.00	TCF7L2_standard_ChIP_seq_CW34
chr14	50188564	50188565	MACS_peak_1195	5.00	TCF7L2_standard_ChIP_seq_CW34
chr14	51342294	51342295	MACS_peak_1196	18.00	TCF7L2_standard_ChIP_seq_CW34
chr14	53288615	53288616	MACS_peak_1197	9.00	TCF7L2_standard_ChIP_seq_CW34
chr14	53414944	53414945	MACS_peak_1198	19.00	TCF7L2_standard_ChIP_seq_CW34
chr14	53908619	53908620	MACS_peak_1199	21.00	TCF7L2_standard_ChIP_seq_CW34
chr14	54080619	54080620	MACS_peak_1200	17.00	TCF7L2_standard_ChIP_seq_CW34
chr14	54082164	54082165	MACS_peak_1201	15.00	TCF7L2_standard_ChIP_seq_CW34
chr14	54446951	54446952	MACS_peak_1202	11.00	TCF7L2_standard_ChIP_seq_CW34
chr14	54497864	54497865	MACS_peak_1203	8.00	TCF7L2_standard_ChIP_seq_CW34
chr14	54569067	54569068	MACS_peak_1204	27.00	TCF7L2_standard_ChIP_seq_CW34
chr14	54576060	54576061	MACS_peak_1205	7.00	TCF7L2_standard_ChIP_seq_CW34
chr14	54605488	54605489	MACS_peak_1206	15.00	TCF7L2_standard_ChIP_seq_CW34
chr14	54725337	54725338	MACS_peak_1207	6.00	TCF7L2_standard_ChIP_seq_CW34
chr14	54758848	54758849	MACS_peak_1208	16.00	TCF7L2_standard_ChIP_seq_CW34
chr14	55110376	55110377	MACS_peak_1209	14.00	TCF7L2_standard_ChIP_seq_CW34
chr14	55117620	55117621	MACS_peak_1210	9.00	TCF7L2_standard_ChIP_seq_CW34
chr14	55123065	55123066	MACS_peak_1211	31.00	TCF7L2_standard_ChIP_seq_CW34
chr14	57046428	57046429	MACS_peak_1212	9.00	TCF7L2_standard_ChIP_seq_CW34
chr14	59476750	59476751	MACS_peak_1213	6.00	TCF7L2_standard_ChIP_seq_CW34
chr14	59536318	59536319	MACS_peak_1214	17.00	TCF7L2_standard_ChIP_seq_CW34
chr14	59750247	59750248	MACS_peak_1215	9.00	TCF7L2_standard_ChIP_seq_CW34
chr14	59950803	59950804	MACS_peak_1216	8.00	TCF7L2_standard_ChIP_seq_CW34
chr14	61110861	61110862	MACS_peak_1217	9.00	TCF7L2_standard_ChIP_seq_CW34
chr14	61189646	61189647	MACS_peak_1218	8.00	TCF7L2_standard_ChIP_seq_CW34
chr14	61282357	61282358	MACS_peak_1219	9.00	TCF7L2_standard_ChIP_seq_CW34
chr14	61569578	61569579	MACS_peak_1220	7.00	TCF7L2_standard_ChIP_seq_CW34
chr14	62181866	62181867	MACS_peak_1221	16.00	TCF7L2_standard_ChIP_seq_CW34
chr14	65405313	65405314	MACS_peak_1222	25.00	TCF7L2_standard_ChIP_seq_CW34
chr14	65693916	65693917	MACS_peak_1223	16.00	TCF7L2_standard_ChIP_seq_CW34
chr14	67708372	67708373	MACS_peak_1224	5.00	TCF7L2_standard_ChIP_seq_CW34
chr14	68165758	68165759	MACS_peak_1225	5.00	TCF7L2_standard_ChIP_seq_CW34
chr14	68762190	68762191	MACS_peak_1226	17.00	TCF7L2_standard_ChIP_seq_CW34
chr14	68816798	68816799	MACS_peak_1227	15.00	TCF7L2_standard_ChIP_seq_CW34
chr14	68936264	68936265	MACS_peak_1228	11.00	TCF7L2_standard_ChIP_seq_CW34
chr14	69065843	69065844	MACS_peak_1229	8.00	TCF7L2_standard_ChIP_seq_CW34
chr14	69246874	69246875	MACS_peak_1230	11.00	TCF7L2_standard_ChIP_seq_CW34
chr14	70157044	70157045	MACS_peak_1231	13.00	TCF7L2_standard_ChIP_seq_CW34
chr14	70170536	70170537	MACS_peak_1232	10.00	TCF7L2_standard_ChIP_seq_CW34
chr14	70317156	70317157	MACS_peak_1233	15.00	TCF7L2_standard_ChIP_seq_CW34
chr14	70785226	70785227	MACS_peak_1234	10.00	TCF7L2_standard_ChIP_seq_CW34
chr14	71803646	71803647	MACS_peak_1235	6.00	TCF7L2_standard_ChIP_seq_CW34
chr14	73613697	73613698	MACS_peak_1236	15.00	TCF7L2_standard_ChIP_seq_CW34
chr14	74238810	74238811	MACS_peak_1237	9.00	TCF7L2_standard_ChIP_seq_CW34
chr14	74318626	74318627	MACS_peak_1238	6.00	TCF7L2_standard_ChIP_seq_CW34
chr14	75943394	75943395	MACS_peak_1239	10.00	TCF7L2_standard_ChIP_seq_CW34
chr14	76002615	76002616	MACS_peak_1240	9.00	TCF7L2_standard_ChIP_seq_CW34
chr14	76114846	76114847	MACS_peak_1241	12.00	TCF7L2_standard_ChIP_seq_CW34
chr14	76292612	76292613	MACS_peak_1242	19.00	TCF7L2_standard_ChIP_seq_CW34
chr14	76863454	76863455	MACS_peak_1243	13.00	TCF7L2_standard_ChIP_seq_CW34
chr14	77965411	77965412	MACS_peak_1244	57.00	TCF7L2_standard_ChIP_seq_CW34
chr14	78432449	78432450	MACS_peak_1245	5.00	TCF7L2_standard_ChIP_seq_CW34
chr14	80935155	80935156	MACS_peak_1246	107.00	TCF7L2_standard_ChIP_seq_CW34
chr14	81044123	81044124	MACS_peak_1247	8.00	TCF7L2_standard_ChIP_seq_CW34
chr14	81770142	81770143	MACS_peak_1248	7.00	TCF7L2_standard_ChIP_seq_CW34
chr14	83556518	83556519	MACS_peak_1249	6.00	TCF7L2_standard_ChIP_seq_CW34
chr14	87364448	87364449	MACS_peak_1250	6.00	TCF7L2_standard_ChIP_seq_CW34
chr14	88237654	88237655	MACS_peak_1251	21.00	TCF7L2_standard_ChIP_seq_CW34
chr14	88490829	88490830	MACS_peak_1252	10.00	TCF7L2_standard_ChIP_seq_CW34
chr14	88510824	88510825	MACS_peak_1253	7.00	TCF7L2_standard_ChIP_seq_CW34
chr14	89493770	89493771	MACS_peak_1254	119.00	TCF7L2_standard_ChIP_seq_CW34
chr14	89507153	89507154	MACS_peak_1255	29.00	TCF7L2_standard_ChIP_seq_CW34
chr14	89818030	89818031	MACS_peak_1256	33.00	TCF7L2_standard_ChIP_seq_CW34
chr14	89882677	89882678	MACS_peak_1257	8.00	TCF7L2_standard_ChIP_seq_CW34
chr14	90006544	90006545	MACS_peak_1258	10.00	TCF7L2_standard_ChIP_seq_CW34
chr14	92246988	92246989	MACS_peak_1259	6.00	TCF7L2_standard_ChIP_seq_CW34
chr14	93214886	93214887	MACS_peak_1260	7.00	TCF7L2_standard_ChIP_seq_CW34
chr14	93502417	93502418	MACS_peak_1261	8.00	TCF7L2_standard_ChIP_seq_CW34
chr14	94369164	94369165	MACS_peak_1262	8.00	TCF7L2_standard_ChIP_seq_CW34
chr14	94806934	94806935	MACS_peak_1263	6.00	TCF7L2_standard_ChIP_seq_CW34
chr14	94919062	94919063	MACS_peak_1264	14.00	TCF7L2_standard_ChIP_seq_CW34
chr14	94922462	94922463	MACS_peak_1265	10.00	TCF7L2_standard_ChIP_seq_CW34
chr14	95039086	95039087	MACS_peak_1266	7.00	TCF7L2_standard_ChIP_seq_CW34
chr14	95060498	95060499	MACS_peak_1267	11.00	TCF7L2_standard_ChIP_seq_CW34
chr14	95138665	95138666	MACS_peak_1268	10.00	TCF7L2_standard_ChIP_seq_CW34
chr14	95767534	95767535	MACS_peak_1269	19.00	TCF7L2_standard_ChIP_seq_CW34
chr14	96668478	96668479	MACS_peak_1270	7.00	TCF7L2_standard_ChIP_seq_CW34
chr14	99783009	99783010	MACS_peak_1271	11.00	TCF7L2_standard_ChIP_seq_CW34
chr14	100070805	100070806	MACS_peak_1272	9.00	TCF7L2_standard_ChIP_seq_CW34
chr14	101283757	101283758	MACS_peak_1273	24.00	TCF7L2_standard_ChIP_seq_CW34
chr14	101705668	101705669	MACS_peak_1274	15.00	TCF7L2_standard_ChIP_seq_CW34
chr14	101754984	101754985	MACS_peak_1275	24.00	TCF7L2_standard_ChIP_seq_CW34
chr14	101879278	101879279	MACS_peak_1276	13.00	TCF7L2_standard_ChIP_seq_CW34
chr14	102553725	102553726	MACS_peak_1277	8.00	TCF7L2_standard_ChIP_seq_CW34
chr14	102657465	102657466	MACS_peak_1278	25.00	TCF7L2_standard_ChIP_seq_CW34
chr14	102931374	102931375	MACS_peak_1279	11.00	TCF7L2_standard_ChIP_seq_CW34
chr14	102943774	102943775	MACS_peak_1280	16.00	TCF7L2_standard_ChIP_seq_CW34
chr14	103192742	103192743	MACS_peak_1281	7.00	TCF7L2_standard_ChIP_seq_CW34
chr14	104672899	104672900	MACS_peak_1282	9.00	TCF7L2_standard_ChIP_seq_CW34
chr14	105558084	105558085	MACS_peak_1283	24.00	TCF7L2_standard_ChIP_seq_CW34
chr14	107253744	107253745	MACS_peak_1284	13.00	TCF7L2_standard_ChIP_seq_CW34
chr15	22387567	22387568	MACS_peak_1285	6.00	TCF7L2_standard_ChIP_seq_CW34
chr15	23668479	23668480	MACS_peak_1286	8.00	TCF7L2_standard_ChIP_seq_CW34
chr15	23935872	23935873	MACS_peak_1287	10.00	TCF7L2_standard_ChIP_seq_CW34
chr15	23959594	23959595	MACS_peak_1288	10.00	TCF7L2_standard_ChIP_seq_CW34
chr15	26017700	26017701	MACS_peak_1289	8.00	TCF7L2_standard_ChIP_seq_CW34
chr15	28702299	28702300	MACS_peak_1290	6.00	TCF7L2_standard_ChIP_seq_CW34
chr15	28983140	28983141	MACS_peak_1291	9.00	TCF7L2_standard_ChIP_seq_CW34
chr15	30028957	30028958	MACS_peak_1292	8.00	TCF7L2_standard_ChIP_seq_CW34
chr15	31196334	31196335	MACS_peak_1293	11.00	TCF7L2_standard_ChIP_seq_CW34
chr15	32635148	32635149	MACS_peak_1294	11.00	TCF7L2_standard_ChIP_seq_CW34
chr15	32969460	32969461	MACS_peak_1295	6.00	TCF7L2_standard_ChIP_seq_CW34
chr15	33145236	33145237	MACS_peak_1296	11.00	TCF7L2_standard_ChIP_seq_CW34
chr15	34450772	34450773	MACS_peak_1297	12.00	TCF7L2_standard_ChIP_seq_CW34
chr15	34630149	34630150	MACS_peak_1298	10.00	TCF7L2_standard_ChIP_seq_CW34
chr15	34807202	34807203	MACS_peak_1299	14.00	TCF7L2_standard_ChIP_seq_CW34
chr15	35986308	35986309	MACS_peak_1300	5.00	TCF7L2_standard_ChIP_seq_CW34
chr15	36799150	36799151	MACS_peak_1301	21.00	TCF7L2_standard_ChIP_seq_CW34
chr15	37107144	37107145	MACS_peak_1302	23.00	TCF7L2_standard_ChIP_seq_CW34
chr15	38002022	38002023	MACS_peak_1303	10.00	TCF7L2_standard_ChIP_seq_CW34
chr15	39503839	39503840	MACS_peak_1304	11.00	TCF7L2_standard_ChIP_seq_CW34
chr15	39566180	39566181	MACS_peak_1305	16.00	TCF7L2_standard_ChIP_seq_CW34
chr15	39727155	39727156	MACS_peak_1306	8.00	TCF7L2_standard_ChIP_seq_CW34
chr15	40681468	40681469	MACS_peak_1307	5.00	TCF7L2_standard_ChIP_seq_CW34
chr15	41552251	41552252	MACS_peak_1308	7.00	TCF7L2_standard_ChIP_seq_CW34
chr15	41788321	41788322	MACS_peak_1309	8.00	TCF7L2_standard_ChIP_seq_CW34
chr15	42431482	42431483	MACS_peak_1310	7.00	TCF7L2_standard_ChIP_seq_CW34
chr15	44580668	44580669	MACS_peak_1311	10.00	TCF7L2_standard_ChIP_seq_CW34
chr15	45128454	45128455	MACS_peak_1312	39.00	TCF7L2_standard_ChIP_seq_CW34
chr15	45205495	45205496	MACS_peak_1313	15.00	TCF7L2_standard_ChIP_seq_CW34
chr15	45479240	45479241	MACS_peak_1314	8.00	TCF7L2_standard_ChIP_seq_CW34
chr15	45603134	45603135	MACS_peak_1315	8.00	TCF7L2_standard_ChIP_seq_CW34
chr15	45749444	45749445	MACS_peak_1316	5.00	TCF7L2_standard_ChIP_seq_CW34
chr15	49092641	49092642	MACS_peak_1317	7.00	TCF7L2_standard_ChIP_seq_CW34
chr15	50408871	50408872	MACS_peak_1318	11.00	TCF7L2_standard_ChIP_seq_CW34
chr15	51057322	51057323	MACS_peak_1319	16.00	TCF7L2_standard_ChIP_seq_CW34
chr15	51606009	51606010	MACS_peak_1320	12.00	TCF7L2_standard_ChIP_seq_CW34
chr15	52822106	52822107	MACS_peak_1321	6.00	TCF7L2_standard_ChIP_seq_CW34
chr15	52861303	52861304	MACS_peak_1322	16.00	TCF7L2_standard_ChIP_seq_CW34
chr15	53747668	53747669	MACS_peak_1323	10.00	TCF7L2_standard_ChIP_seq_CW34
chr15	55513572	55513573	MACS_peak_1324	5.00	TCF7L2_standard_ChIP_seq_CW34
chr15	55542784	55542785	MACS_peak_1325	10.00	TCF7L2_standard_ChIP_seq_CW34
chr15	55880152	55880153	MACS_peak_1326	38.00	TCF7L2_standard_ChIP_seq_CW34
chr15	56244957	56244958	MACS_peak_1327	13.00	TCF7L2_standard_ChIP_seq_CW34
chr15	57600008	57600009	MACS_peak_1328	15.00	TCF7L2_standard_ChIP_seq_CW34
chr15	57789448	57789449	MACS_peak_1329	8.00	TCF7L2_standard_ChIP_seq_CW34
chr15	58578278	58578279	MACS_peak_1330	10.00	TCF7L2_standard_ChIP_seq_CW34
chr15	59397119	59397120	MACS_peak_1331	5.00	TCF7L2_standard_ChIP_seq_CW34
chr15	59569157	59569158	MACS_peak_1332	8.00	TCF7L2_standard_ChIP_seq_CW34
chr15	59771256	59771257	MACS_peak_1333	5.00	TCF7L2_standard_ChIP_seq_CW34
chr15	59846436	59846437	MACS_peak_1334	14.00	TCF7L2_standard_ChIP_seq_CW34
chr15	60386070	60386071	MACS_peak_1335	9.00	TCF7L2_standard_ChIP_seq_CW34
chr15	60672046	60672047	MACS_peak_1336	6.00	TCF7L2_standard_ChIP_seq_CW34
chr15	62352775	62352776	MACS_peak_1337	25.00	TCF7L2_standard_ChIP_seq_CW34
chr15	63400205	63400206	MACS_peak_1338	8.00	TCF7L2_standard_ChIP_seq_CW34
chr15	63806952	63806953	MACS_peak_1339	13.00	TCF7L2_standard_ChIP_seq_CW34
chr15	64056501	64056502	MACS_peak_1340	7.00	TCF7L2_standard_ChIP_seq_CW34
chr15	64509551	64509552	MACS_peak_1341	11.00	TCF7L2_standard_ChIP_seq_CW34
chr15	65281801	65281802	MACS_peak_1342	6.00	TCF7L2_standard_ChIP_seq_CW34
chr15	65477964	65477965	MACS_peak_1343	14.00	TCF7L2_standard_ChIP_seq_CW34
chr15	65604878	65604879	MACS_peak_1344	28.00	TCF7L2_standard_ChIP_seq_CW34
chr15	65647548	65647549	MACS_peak_1345	6.00	TCF7L2_standard_ChIP_seq_CW34
chr15	65670525	65670526	MACS_peak_1346	23.00	TCF7L2_standard_ChIP_seq_CW34
chr15	66083903	66083904	MACS_peak_1347	14.00	TCF7L2_standard_ChIP_seq_CW34
chr15	66994598	66994599	MACS_peak_1348	12.00	TCF7L2_standard_ChIP_seq_CW34
chr15	67022192	67022193	MACS_peak_1349	10.00	TCF7L2_standard_ChIP_seq_CW34
chr15	67358008	67358009	MACS_peak_1350	11.00	TCF7L2_standard_ChIP_seq_CW34
chr15	67439540	67439541	MACS_peak_1351	14.00	TCF7L2_standard_ChIP_seq_CW34
chr15	68177082	68177083	MACS_peak_1352	14.00	TCF7L2_standard_ChIP_seq_CW34
chr15	68208009	68208010	MACS_peak_1353	8.00	TCF7L2_standard_ChIP_seq_CW34
chr15	68238725	68238726	MACS_peak_1354	11.00	TCF7L2_standard_ChIP_seq_CW34
chr15	69706689	69706690	MACS_peak_1355	5.00	TCF7L2_standard_ChIP_seq_CW34
chr15	69853841	69853842	MACS_peak_1356	14.00	TCF7L2_standard_ChIP_seq_CW34
chr15	69884428	69884429	MACS_peak_1357	61.00	TCF7L2_standard_ChIP_seq_CW34
chr15	69887097	69887098	MACS_peak_1358	26.00	TCF7L2_standard_ChIP_seq_CW34
chr15	69920364	69920365	MACS_peak_1359	8.00	TCF7L2_standard_ChIP_seq_CW34
chr15	70390972	70390973	MACS_peak_1360	33.00	TCF7L2_standard_ChIP_seq_CW34
chr15	70432995	70432996	MACS_peak_1361	78.00	TCF7L2_standard_ChIP_seq_CW34
chr15	70877481	70877482	MACS_peak_1362	25.00	TCF7L2_standard_ChIP_seq_CW34
chr15	70909676	70909677	MACS_peak_1363	28.00	TCF7L2_standard_ChIP_seq_CW34
chr15	71381260	71381261	MACS_peak_1364	7.00	TCF7L2_standard_ChIP_seq_CW34
chr15	71946546	71946547	MACS_peak_1365	8.00	TCF7L2_standard_ChIP_seq_CW34
chr15	72707885	72707886	MACS_peak_1366	7.00	TCF7L2_standard_ChIP_seq_CW34
chr15	74736191	74736192	MACS_peak_1367	25.00	TCF7L2_standard_ChIP_seq_CW34
chr15	74753605	74753606	MACS_peak_1368	11.00	TCF7L2_standard_ChIP_seq_CW34
chr15	74838672	74838673	MACS_peak_1369	10.00	TCF7L2_standard_ChIP_seq_CW34
chr15	75432640	75432641	MACS_peak_1370	13.00	TCF7L2_standard_ChIP_seq_CW34
chr15	76068669	76068670	MACS_peak_1371	7.00	TCF7L2_standard_ChIP_seq_CW34
chr15	77197761	77197762	MACS_peak_1372	8.00	TCF7L2_standard_ChIP_seq_CW34
chr15	77324531	77324532	MACS_peak_1373	28.00	TCF7L2_standard_ChIP_seq_CW34
chr15	78284207	78284208	MACS_peak_1374	6.00	TCF7L2_standard_ChIP_seq_CW34
chr15	78357720	78357721	MACS_peak_1375	18.00	TCF7L2_standard_ChIP_seq_CW34
chr15	79143507	79143508	MACS_peak_1376	6.00	TCF7L2_standard_ChIP_seq_CW34
chr15	79286485	79286486	MACS_peak_1377	41.00	TCF7L2_standard_ChIP_seq_CW34
chr15	80908559	80908560	MACS_peak_1378	6.00	TCF7L2_standard_ChIP_seq_CW34
chr15	81097477	81097478	MACS_peak_1379	19.00	TCF7L2_standard_ChIP_seq_CW34
chr15	83525419	83525420	MACS_peak_1380	19.00	TCF7L2_standard_ChIP_seq_CW34
chr15	83735510	83735511	MACS_peak_1381	6.00	TCF7L2_standard_ChIP_seq_CW34
chr15	84599735	84599736	MACS_peak_1382	9.00	TCF7L2_standard_ChIP_seq_CW34
chr15	85201713	85201714	MACS_peak_1383	6.00	TCF7L2_standard_ChIP_seq_CW34
chr15	86005508	86005509	MACS_peak_1384	6.00	TCF7L2_standard_ChIP_seq_CW34
chr15	89631520	89631521	MACS_peak_1385	7.00	TCF7L2_standard_ChIP_seq_CW34
chr15	89878438	89878439	MACS_peak_1386	7.00	TCF7L2_standard_ChIP_seq_CW34
chr15	90223704	90223705	MACS_peak_1387	9.00	TCF7L2_standard_ChIP_seq_CW34
chr15	90546653	90546654	MACS_peak_1388	11.00	TCF7L2_standard_ChIP_seq_CW34
chr15	90581996	90581997	MACS_peak_1389	28.00	TCF7L2_standard_ChIP_seq_CW34
chr15	91417571	91417572	MACS_peak_1390	9.00	TCF7L2_standard_ChIP_seq_CW34
chr15	93447503	93447504	MACS_peak_1391	15.00	TCF7L2_standard_ChIP_seq_CW34
chr15	93884586	93884587	MACS_peak_1392	11.00	TCF7L2_standard_ChIP_seq_CW34
chr15	94607723	94607724	MACS_peak_1393	6.00	TCF7L2_standard_ChIP_seq_CW34
chr15	95388025	95388026	MACS_peak_1394	11.00	TCF7L2_standard_ChIP_seq_CW34
chr15	96752870	96752871	MACS_peak_1395	6.00	TCF7L2_standard_ChIP_seq_CW34
chr15	99281106	99281107	MACS_peak_1396	20.00	TCF7L2_standard_ChIP_seq_CW34
chr15	99403376	99403377	MACS_peak_1397	5.00	TCF7L2_standard_ChIP_seq_CW34
chr15	99961565	99961566	MACS_peak_1398	9.00	TCF7L2_standard_ChIP_seq_CW34
chr15	100105975	100105976	MACS_peak_1399	9.00	TCF7L2_standard_ChIP_seq_CW34
chr15	100154653	100154654	MACS_peak_1400	11.00	TCF7L2_standard_ChIP_seq_CW34
chr15	100324672	100324673	MACS_peak_1401	8.00	TCF7L2_standard_ChIP_seq_CW34
chr15	100465570	100465571	MACS_peak_1402	6.00	TCF7L2_standard_ChIP_seq_CW34
chr15	101611173	101611174	MACS_peak_1403	14.00	TCF7L2_standard_ChIP_seq_CW34
chr15	101986897	101986898	MACS_peak_1404	9.00	TCF7L2_standard_ChIP_seq_CW34
chr15	102028803	102028804	MACS_peak_1405	21.00	TCF7L2_standard_ChIP_seq_CW34
chr16	1359107	1359108	MACS_peak_1406	6.00	TCF7L2_standard_ChIP_seq_CW34
chr16	2317932	2317933	MACS_peak_1407	9.00	TCF7L2_standard_ChIP_seq_CW34
chr16	3314265	3314266	MACS_peak_1408	9.00	TCF7L2_standard_ChIP_seq_CW34
chr16	4897114	4897115	MACS_peak_1409	9.00	TCF7L2_standard_ChIP_seq_CW34
chr16	5194519	5194520	MACS_peak_1410	19.00	TCF7L2_standard_ChIP_seq_CW34
chr16	8287710	8287711	MACS_peak_1411	12.00	TCF7L2_standard_ChIP_seq_CW34
chr16	9081455	9081456	MACS_peak_1412	34.00	TCF7L2_standard_ChIP_seq_CW34
chr16	10360025	10360026	MACS_peak_1413	67.00	TCF7L2_standard_ChIP_seq_CW34
chr16	10412836	10412837	MACS_peak_1414	18.00	TCF7L2_standard_ChIP_seq_CW34
chr16	10456430	10456431	MACS_peak_1415	11.00	TCF7L2_standard_ChIP_seq_CW34
chr16	10704903	10704904	MACS_peak_1416	14.00	TCF7L2_standard_ChIP_seq_CW34
chr16	10741924	10741925	MACS_peak_1417	61.00	TCF7L2_standard_ChIP_seq_CW34
chr16	11233409	11233410	MACS_peak_1418	10.00	TCF7L2_standard_ChIP_seq_CW34
chr16	11373518	11373519	MACS_peak_1419	8.00	TCF7L2_standard_ChIP_seq_CW34
chr16	11390526	11390527	MACS_peak_1420	10.00	TCF7L2_standard_ChIP_seq_CW34
chr16	11458344	11458345	MACS_peak_1421	28.00	TCF7L2_standard_ChIP_seq_CW34
chr16	11669755	11669756	MACS_peak_1422	18.00	TCF7L2_standard_ChIP_seq_CW34
chr16	12191629	12191630	MACS_peak_1423	16.00	TCF7L2_standard_ChIP_seq_CW34
chr16	12773088	12773089	MACS_peak_1424	15.00	TCF7L2_standard_ChIP_seq_CW34
chr16	12848076	12848077	MACS_peak_1425	14.00	TCF7L2_standard_ChIP_seq_CW34
chr16	12897743	12897744	MACS_peak_1426	33.00	TCF7L2_standard_ChIP_seq_CW34
chr16	13157604	13157605	MACS_peak_1427	7.00	TCF7L2_standard_ChIP_seq_CW34
chr16	13957070	13957071	MACS_peak_1428	15.00	TCF7L2_standard_ChIP_seq_CW34
chr16	14182126	14182127	MACS_peak_1429	10.00	TCF7L2_standard_ChIP_seq_CW34
chr16	14458604	14458605	MACS_peak_1430	14.00	TCF7L2_standard_ChIP_seq_CW34
chr16	14493524	14493525	MACS_peak_1431	38.00	TCF7L2_standard_ChIP_seq_CW34
chr16	15049012	15049013	MACS_peak_1432	5.00	TCF7L2_standard_ChIP_seq_CW34
chr16	15709601	15709602	MACS_peak_1433	14.00	TCF7L2_standard_ChIP_seq_CW34
chr16	15766908	15766909	MACS_peak_1434	12.00	TCF7L2_standard_ChIP_seq_CW34
chr16	16300354	16300355	MACS_peak_1435	20.00	TCF7L2_standard_ChIP_seq_CW34
chr16	18244532	18244533	MACS_peak_1436	9.00	TCF7L2_standard_ChIP_seq_CW34
chr16	18599517	18599518	MACS_peak_1437	20.00	TCF7L2_standard_ChIP_seq_CW34
chr16	18621662	18621663	MACS_peak_1438	9.00	TCF7L2_standard_ChIP_seq_CW34
chr16	19000565	19000566	MACS_peak_1439	27.00	TCF7L2_standard_ChIP_seq_CW34
chr16	19079541	19079542	MACS_peak_1440	8.00	TCF7L2_standard_ChIP_seq_CW34
chr16	19553545	19553546	MACS_peak_1441	17.00	TCF7L2_standard_ChIP_seq_CW34
chr16	19938250	19938251	MACS_peak_1442	26.00	TCF7L2_standard_ChIP_seq_CW34
chr16	19996977	19996978	MACS_peak_1443	7.00	TCF7L2_standard_ChIP_seq_CW34
chr16	20610390	20610391	MACS_peak_1444	7.00	TCF7L2_standard_ChIP_seq_CW34
chr16	20911929	20911930	MACS_peak_1445	10.00	TCF7L2_standard_ChIP_seq_CW34
chr16	21200289	21200290	MACS_peak_1446	9.00	TCF7L2_standard_ChIP_seq_CW34
chr16	21524111	21524112	MACS_peak_1447	89.00	TCF7L2_standard_ChIP_seq_CW34
chr16	21536828	21536829	MACS_peak_1448	69.00	TCF7L2_standard_ChIP_seq_CW34
chr16	21577378	21577379	MACS_peak_1449	19.00	TCF7L2_standard_ChIP_seq_CW34
chr16	21611065	21611066	MACS_peak_1450	10.00	TCF7L2_standard_ChIP_seq_CW34
chr16	21650232	21650233	MACS_peak_1451	12.00	TCF7L2_standard_ChIP_seq_CW34
chr16	21948777	21948778	MACS_peak_1452	14.00	TCF7L2_standard_ChIP_seq_CW34
chr16	22448576	22448577	MACS_peak_1453	7.00	TCF7L2_standard_ChIP_seq_CW34
chr16	23607740	23607741	MACS_peak_1454	9.00	TCF7L2_standard_ChIP_seq_CW34
chr16	23979225	23979226	MACS_peak_1455	10.00	TCF7L2_standard_ChIP_seq_CW34
chr16	24551251	24551252	MACS_peak_1456	9.00	TCF7L2_standard_ChIP_seq_CW34
chr16	25269458	25269459	MACS_peak_1457	9.00	TCF7L2_standard_ChIP_seq_CW34
chr16	26536743	26536744	MACS_peak_1458	6.00	TCF7L2_standard_ChIP_seq_CW34
chr16	27285370	27285371	MACS_peak_1459	33.00	TCF7L2_standard_ChIP_seq_CW34
chr16	28874940	28874941	MACS_peak_1460	6.00	TCF7L2_standard_ChIP_seq_CW34
chr16	28936924	28936925	MACS_peak_1461	27.00	TCF7L2_standard_ChIP_seq_CW34
chr16	29214042	29214043	MACS_peak_1462	12.00	TCF7L2_standard_ChIP_seq_CW34
chr16	29333146	29333147	MACS_peak_1463	8.00	TCF7L2_standard_ChIP_seq_CW34
chr16	29617408	29617409	MACS_peak_1464	52.00	TCF7L2_standard_ChIP_seq_CW34
chr16	29874713	29874714	MACS_peak_1465	9.00	TCF7L2_standard_ChIP_seq_CW34
chr16	30123715	30123716	MACS_peak_1466	14.00	TCF7L2_standard_ChIP_seq_CW34
chr16	30347446	30347447	MACS_peak_1467	13.00	TCF7L2_standard_ChIP_seq_CW34
chr16	30621676	30621677	MACS_peak_1468	14.00	TCF7L2_standard_ChIP_seq_CW34
chr16	30825715	30825716	MACS_peak_1469	8.00	TCF7L2_standard_ChIP_seq_CW34
chr16	31105991	31105992	MACS_peak_1470	6.00	TCF7L2_standard_ChIP_seq_CW34
chr16	31213406	31213407	MACS_peak_1471	8.00	TCF7L2_standard_ChIP_seq_CW34
chr16	31293799	31293800	MACS_peak_1472	25.00	TCF7L2_standard_ChIP_seq_CW34
chr16	31724740	31724741	MACS_peak_1473	13.00	TCF7L2_standard_ChIP_seq_CW34
chr16	31987509	31987510	MACS_peak_1474	8.00	TCF7L2_standard_ChIP_seq_CW34
chr16	32199523	32199524	MACS_peak_1475	8.00	TCF7L2_standard_ChIP_seq_CW34
chr16	32290032	32290033	MACS_peak_1476	6.00	TCF7L2_standard_ChIP_seq_CW34
chr16	33294291	33294292	MACS_peak_1479	10.00	TCF7L2_standard_ChIP_seq_CW34
chr16	33326021	33326022	MACS_peak_1480	7.00	TCF7L2_standard_ChIP_seq_CW34
chr16	46864867	46864868	MACS_peak_1496	9.00	TCF7L2_standard_ChIP_seq_CW34
chr16	46889577	46889578	MACS_peak_1497	14.00	TCF7L2_standard_ChIP_seq_CW34
chr16	46910682	46910683	MACS_peak_1498	13.00	TCF7L2_standard_ChIP_seq_CW34
chr16	47509787	47509788	MACS_peak_1499	9.00	TCF7L2_standard_ChIP_seq_CW34
chr16	47578619	47578620	MACS_peak_1500	9.00	TCF7L2_standard_ChIP_seq_CW34
chr16	47668227	47668228	MACS_peak_1501	13.00	TCF7L2_standard_ChIP_seq_CW34
chr16	48222548	48222549	MACS_peak_1502	10.00	TCF7L2_standard_ChIP_seq_CW34
chr16	48419657	48419658	MACS_peak_1503	10.00	TCF7L2_standard_ChIP_seq_CW34
chr16	48514135	48514136	MACS_peak_1504	12.00	TCF7L2_standard_ChIP_seq_CW34
chr16	48644272	48644273	MACS_peak_1505	15.00	TCF7L2_standard_ChIP_seq_CW34
chr16	50502045	50502046	MACS_peak_1506	134.00	TCF7L2_standard_ChIP_seq_CW34
chr16	50554623	50554624	MACS_peak_1507	66.00	TCF7L2_standard_ChIP_seq_CW34
chr16	50564737	50564738	MACS_peak_1508	9.00	TCF7L2_standard_ChIP_seq_CW34
chr16	50573310	50573311	MACS_peak_1509	49.00	TCF7L2_standard_ChIP_seq_CW34
chr16	50581574	50581575	MACS_peak_1510	106.00	TCF7L2_standard_ChIP_seq_CW34
chr16	50651258	50651259	MACS_peak_1511	15.00	TCF7L2_standard_ChIP_seq_CW34
chr16	51092658	51092659	MACS_peak_1512	17.00	TCF7L2_standard_ChIP_seq_CW34
chr16	51154796	51154797	MACS_peak_1513	37.00	TCF7L2_standard_ChIP_seq_CW34
chr16	52432569	52432570	MACS_peak_1514	11.00	TCF7L2_standard_ChIP_seq_CW34
chr16	52442619	52442620	MACS_peak_1515	43.00	TCF7L2_standard_ChIP_seq_CW34
chr16	52526850	52526851	MACS_peak_1516	14.00	TCF7L2_standard_ChIP_seq_CW34
chr16	52530552	52530553	MACS_peak_1517	22.00	TCF7L2_standard_ChIP_seq_CW34
chr16	54195709	54195710	MACS_peak_1518	15.00	TCF7L2_standard_ChIP_seq_CW34
chr16	54210355	54210356	MACS_peak_1519	34.00	TCF7L2_standard_ChIP_seq_CW34
chr16	54464620	54464621	MACS_peak_1520	12.00	TCF7L2_standard_ChIP_seq_CW34
chr16	54662931	54662932	MACS_peak_1521	110.00	TCF7L2_standard_ChIP_seq_CW34
chr16	55657129	55657130	MACS_peak_1522	36.00	TCF7L2_standard_ChIP_seq_CW34
chr16	55671162	55671163	MACS_peak_1523	15.00	TCF7L2_standard_ChIP_seq_CW34
chr16	55867227	55867228	MACS_peak_1524	20.00	TCF7L2_standard_ChIP_seq_CW34
chr16	56585988	56585989	MACS_peak_1525	12.00	TCF7L2_standard_ChIP_seq_CW34
chr16	56716381	56716382	MACS_peak_1526	12.00	TCF7L2_standard_ChIP_seq_CW34
chr16	56873070	56873071	MACS_peak_1527	9.00	TCF7L2_standard_ChIP_seq_CW34
chr16	57190399	57190400	MACS_peak_1528	13.00	TCF7L2_standard_ChIP_seq_CW34
chr16	57928814	57928815	MACS_peak_1529	18.00	TCF7L2_standard_ChIP_seq_CW34
chr16	58490351	58490352	MACS_peak_1530	10.00	TCF7L2_standard_ChIP_seq_CW34
chr16	64195107	64195108	MACS_peak_1531	10.00	TCF7L2_standard_ChIP_seq_CW34
chr16	65675670	65675671	MACS_peak_1532	8.00	TCF7L2_standard_ChIP_seq_CW34
chr16	66467909	66467910	MACS_peak_1533	27.00	TCF7L2_standard_ChIP_seq_CW34
chr16	66487298	66487299	MACS_peak_1534	19.00	TCF7L2_standard_ChIP_seq_CW34
chr16	66939277	66939278	MACS_peak_1535	31.00	TCF7L2_standard_ChIP_seq_CW34
chr16	67194620	67194621	MACS_peak_1536	12.00	TCF7L2_standard_ChIP_seq_CW34
chr16	67397448	67397449	MACS_peak_1537	13.00	TCF7L2_standard_ChIP_seq_CW34
chr16	67666519	67666520	MACS_peak_1538	10.00	TCF7L2_standard_ChIP_seq_CW34
chr16	68119590	68119591	MACS_peak_1539	8.00	TCF7L2_standard_ChIP_seq_CW34
chr16	68147144	68147145	MACS_peak_1540	34.00	TCF7L2_standard_ChIP_seq_CW34
chr16	68166156	68166157	MACS_peak_1541	10.00	TCF7L2_standard_ChIP_seq_CW34
chr16	68299494	68299495	MACS_peak_1542	10.00	TCF7L2_standard_ChIP_seq_CW34
chr16	68315795	68315796	MACS_peak_1543	38.00	TCF7L2_standard_ChIP_seq_CW34
chr16	68482427	68482428	MACS_peak_1544	57.00	TCF7L2_standard_ChIP_seq_CW34
chr16	68907751	68907752	MACS_peak_1545	18.00	TCF7L2_standard_ChIP_seq_CW34
chr16	69430607	69430608	MACS_peak_1546	20.00	TCF7L2_standard_ChIP_seq_CW34
chr16	69649624	69649625	MACS_peak_1547	14.00	TCF7L2_standard_ChIP_seq_CW34
chr16	69714419	69714420	MACS_peak_1548	19.00	TCF7L2_standard_ChIP_seq_CW34
chr16	69987094	69987095	MACS_peak_1549	8.00	TCF7L2_standard_ChIP_seq_CW34
chr16	70100101	70100102	MACS_peak_1550	7.00	TCF7L2_standard_ChIP_seq_CW34
chr16	70723608	70723609	MACS_peak_1551	29.00	TCF7L2_standard_ChIP_seq_CW34
chr16	70759300	70759301	MACS_peak_1552	13.00	TCF7L2_standard_ChIP_seq_CW34
chr16	70780194	70780195	MACS_peak_1553	7.00	TCF7L2_standard_ChIP_seq_CW34
chr16	71330720	71330721	MACS_peak_1554	8.00	TCF7L2_standard_ChIP_seq_CW34
chr16	71606621	71606622	MACS_peak_1555	8.00	TCF7L2_standard_ChIP_seq_CW34
chr16	71628119	71628120	MACS_peak_1556	10.00	TCF7L2_standard_ChIP_seq_CW34
chr16	71929299	71929300	MACS_peak_1557	48.00	TCF7L2_standard_ChIP_seq_CW34
chr16	72258337	72258338	MACS_peak_1558	10.00	TCF7L2_standard_ChIP_seq_CW34
chr16	73082457	73082458	MACS_peak_1559	21.00	TCF7L2_standard_ChIP_seq_CW34
chr16	73498701	73498702	MACS_peak_1560	7.00	TCF7L2_standard_ChIP_seq_CW34
chr16	75018725	75018726	MACS_peak_1561	8.00	TCF7L2_standard_ChIP_seq_CW34
chr16	75163874	75163875	MACS_peak_1562	10.00	TCF7L2_standard_ChIP_seq_CW34
chr16	75272213	75272214	MACS_peak_1563	7.00	TCF7L2_standard_ChIP_seq_CW34
chr16	76668974	76668975	MACS_peak_1564	6.00	TCF7L2_standard_ChIP_seq_CW34
chr16	77756662	77756663	MACS_peak_1565	8.00	TCF7L2_standard_ChIP_seq_CW34
chr16	77875044	77875045	MACS_peak_1566	7.00	TCF7L2_standard_ChIP_seq_CW34
chr16	78331149	78331150	MACS_peak_1567	27.00	TCF7L2_standard_ChIP_seq_CW34
chr16	78519630	78519631	MACS_peak_1568	24.00	TCF7L2_standard_ChIP_seq_CW34
chr16	78631054	78631055	MACS_peak_1569	26.00	TCF7L2_standard_ChIP_seq_CW34
chr16	78768696	78768697	MACS_peak_1570	13.00	TCF7L2_standard_ChIP_seq_CW34
chr16	79635487	79635488	MACS_peak_1571	11.00	TCF7L2_standard_ChIP_seq_CW34
chr16	79804512	79804513	MACS_peak_1572	6.00	TCF7L2_standard_ChIP_seq_CW34
chr16	80917288	80917289	MACS_peak_1573	9.00	TCF7L2_standard_ChIP_seq_CW34
chr16	80948400	80948401	MACS_peak_1574	10.00	TCF7L2_standard_ChIP_seq_CW34
chr16	81040716	81040717	MACS_peak_1575	17.00	TCF7L2_standard_ChIP_seq_CW34
chr16	81156821	81156822	MACS_peak_1576	25.00	TCF7L2_standard_ChIP_seq_CW34
chr16	81485975	81485976	MACS_peak_1577	37.00	TCF7L2_standard_ChIP_seq_CW34
chr16	81491528	81491529	MACS_peak_1578	28.00	TCF7L2_standard_ChIP_seq_CW34
chr16	82116589	82116590	MACS_peak_1579	14.00	TCF7L2_standard_ChIP_seq_CW34
chr16	83021243	83021244	MACS_peak_1580	8.00	TCF7L2_standard_ChIP_seq_CW34
chr16	84172962	84172963	MACS_peak_1581	24.00	TCF7L2_standard_ChIP_seq_CW34
chr16	84569261	84569262	MACS_peak_1582	9.00	TCF7L2_standard_ChIP_seq_CW34
chr16	85325390	85325391	MACS_peak_1583	12.00	TCF7L2_standard_ChIP_seq_CW34
chr16	85341419	85341420	MACS_peak_1584	37.00	TCF7L2_standard_ChIP_seq_CW34
chr16	85350131	85350132	MACS_peak_1585	9.00	TCF7L2_standard_ChIP_seq_CW34
chr16	85560162	85560163	MACS_peak_1586	11.00	TCF7L2_standard_ChIP_seq_CW34
chr16	85569144	85569145	MACS_peak_1587	75.00	TCF7L2_standard_ChIP_seq_CW34
chr16	85587013	85587014	MACS_peak_1588	17.00	TCF7L2_standard_ChIP_seq_CW34
chr16	87389525	87389526	MACS_peak_1589	7.00	TCF7L2_standard_ChIP_seq_CW34
chr16	87416060	87416061	MACS_peak_1590	58.00	TCF7L2_standard_ChIP_seq_CW34
chr16	87562557	87562558	MACS_peak_1591	16.00	TCF7L2_standard_ChIP_seq_CW34
chr16	87895490	87895491	MACS_peak_1592	44.00	TCF7L2_standard_ChIP_seq_CW34
chr16	87970320	87970321	MACS_peak_1593	13.00	TCF7L2_standard_ChIP_seq_CW34
chr16	88202297	88202298	MACS_peak_1594	7.00	TCF7L2_standard_ChIP_seq_CW34
chr16	88522075	88522076	MACS_peak_1595	28.00	TCF7L2_standard_ChIP_seq_CW34
chr16	88554555	88554556	MACS_peak_1596	11.00	TCF7L2_standard_ChIP_seq_CW34
chr16	89264436	89264437	MACS_peak_1597	14.00	TCF7L2_standard_ChIP_seq_CW34
chr16	89461796	89461797	MACS_peak_1598	41.00	TCF7L2_standard_ChIP_seq_CW34
chr16	89691313	89691314	MACS_peak_1599	14.00	TCF7L2_standard_ChIP_seq_CW34
chr16	89984464	89984465	MACS_peak_1600	42.00	TCF7L2_standard_ChIP_seq_CW34
chr16	90061968	90061969	MACS_peak_1601	6.00	TCF7L2_standard_ChIP_seq_CW34
chr16	90235808	90235809	MACS_peak_1602	7.00	TCF7L2_standard_ChIP_seq_CW34
chr17	833759	833760	MACS_peak_1603	10.00	TCF7L2_standard_ChIP_seq_CW34
chr17	1171824	1171825	MACS_peak_1604	21.00	TCF7L2_standard_ChIP_seq_CW34
chr17	1588235	1588236	MACS_peak_1605	7.00	TCF7L2_standard_ChIP_seq_CW34
chr17	1970129	1970130	MACS_peak_1606	18.00	TCF7L2_standard_ChIP_seq_CW34
chr17	2061453	2061454	MACS_peak_1607	7.00	TCF7L2_standard_ChIP_seq_CW34
chr17	2078090	2078091	MACS_peak_1608	9.00	TCF7L2_standard_ChIP_seq_CW34
chr17	2415380	2415381	MACS_peak_1609	9.00	TCF7L2_standard_ChIP_seq_CW34
chr17	2771347	2771348	MACS_peak_1610	15.00	TCF7L2_standard_ChIP_seq_CW34
chr17	4213940	4213941	MACS_peak_1611	10.00	TCF7L2_standard_ChIP_seq_CW34
chr17	5342298	5342299	MACS_peak_1612	6.00	TCF7L2_standard_ChIP_seq_CW34
chr17	5667092	5667093	MACS_peak_1613	22.00	TCF7L2_standard_ChIP_seq_CW34
chr17	5685665	5685666	MACS_peak_1614	9.00	TCF7L2_standard_ChIP_seq_CW34
chr17	6603567	6603568	MACS_peak_1615	19.00	TCF7L2_standard_ChIP_seq_CW34
chr17	6950525	6950526	MACS_peak_1616	18.00	TCF7L2_standard_ChIP_seq_CW34
chr17	7083018	7083019	MACS_peak_1617	32.00	TCF7L2_standard_ChIP_seq_CW34
chr17	7185164	7185165	MACS_peak_1618	16.00	TCF7L2_standard_ChIP_seq_CW34
chr17	7589707	7589708	MACS_peak_1619	8.00	TCF7L2_standard_ChIP_seq_CW34
chr17	7959960	7959961	MACS_peak_1620	23.00	TCF7L2_standard_ChIP_seq_CW34
chr17	8177344	8177345	MACS_peak_1621	15.00	TCF7L2_standard_ChIP_seq_CW34
chr17	8323807	8323808	MACS_peak_1622	10.00	TCF7L2_standard_ChIP_seq_CW34
chr17	8537040	8537041	MACS_peak_1623	20.00	TCF7L2_standard_ChIP_seq_CW34
chr17	9001673	9001674	MACS_peak_1624	6.00	TCF7L2_standard_ChIP_seq_CW34
chr17	9054641	9054642	MACS_peak_1625	10.00	TCF7L2_standard_ChIP_seq_CW34
chr17	10519911	10519912	MACS_peak_1626	9.00	TCF7L2_standard_ChIP_seq_CW34
chr17	10647957	10647958	MACS_peak_1627	9.00	TCF7L2_standard_ChIP_seq_CW34
chr17	14206089	14206090	MACS_peak_1628	12.00	TCF7L2_standard_ChIP_seq_CW34
chr17	14276561	14276562	MACS_peak_1629	19.00	TCF7L2_standard_ChIP_seq_CW34
chr17	14641789	14641790	MACS_peak_1630	7.00	TCF7L2_standard_ChIP_seq_CW34
chr17	15597882	15597883	MACS_peak_1631	7.00	TCF7L2_standard_ChIP_seq_CW34
chr17	16256187	16256188	MACS_peak_1632	6.00	TCF7L2_standard_ChIP_seq_CW34
chr17	16284265	16284266	MACS_peak_1633	9.00	TCF7L2_standard_ChIP_seq_CW34
chr17	16497187	16497188	MACS_peak_1634	11.00	TCF7L2_standard_ChIP_seq_CW34
chr17	16955302	16955303	MACS_peak_1635	14.00	TCF7L2_standard_ChIP_seq_CW34
chr17	17140600	17140601	MACS_peak_1636	10.00	TCF7L2_standard_ChIP_seq_CW34
chr17	17349692	17349693	MACS_peak_1637	12.00	TCF7L2_standard_ChIP_seq_CW34
chr17	17437406	17437407	MACS_peak_1638	30.00	TCF7L2_standard_ChIP_seq_CW34
chr17	17495001	17495002	MACS_peak_1639	11.00	TCF7L2_standard_ChIP_seq_CW34
chr17	17686622	17686623	MACS_peak_1640	8.00	TCF7L2_standard_ChIP_seq_CW34
chr17	17804110	17804111	MACS_peak_1641	18.00	TCF7L2_standard_ChIP_seq_CW34
chr17	19563811	19563812	MACS_peak_1642	22.00	TCF7L2_standard_ChIP_seq_CW34
chr17	19575615	19575616	MACS_peak_1643	32.00	TCF7L2_standard_ChIP_seq_CW34
chr17	19651833	19651834	MACS_peak_1644	16.00	TCF7L2_standard_ChIP_seq_CW34
chr17	21156834	21156835	MACS_peak_1645	8.00	TCF7L2_standard_ChIP_seq_CW34
chr17	26125003	26125004	MACS_peak_1652	9.00	TCF7L2_standard_ChIP_seq_CW34
chr17	26697629	26697630	MACS_peak_1653	7.00	TCF7L2_standard_ChIP_seq_CW34
chr17	26926121	26926122	MACS_peak_1654	5.00	TCF7L2_standard_ChIP_seq_CW34
chr17	27403176	27403177	MACS_peak_1655	6.00	TCF7L2_standard_ChIP_seq_CW34
chr17	27503853	27503854	MACS_peak_1656	7.00	TCF7L2_standard_ChIP_seq_CW34
chr17	28553083	28553084	MACS_peak_1657	9.00	TCF7L2_standard_ChIP_seq_CW34
chr17	28671547	28671548	MACS_peak_1658	10.00	TCF7L2_standard_ChIP_seq_CW34
chr17	29035975	29035976	MACS_peak_1659	10.00	TCF7L2_standard_ChIP_seq_CW34
chr17	29243260	29243261	MACS_peak_1660	18.00	TCF7L2_standard_ChIP_seq_CW34
chr17	30813474	30813475	MACS_peak_1661	23.00	TCF7L2_standard_ChIP_seq_CW34
chr17	31255385	31255386	MACS_peak_1663	8.00	TCF7L2_standard_ChIP_seq_CW34
chr17	32925657	32925658	MACS_peak_1664	16.00	TCF7L2_standard_ChIP_seq_CW34
chr17	33390696	33390697	MACS_peak_1665	6.00	TCF7L2_standard_ChIP_seq_CW34
chr17	34040145	34040146	MACS_peak_1667	13.00	TCF7L2_standard_ChIP_seq_CW34
chr17	34300657	34300658	MACS_peak_1668	18.00	TCF7L2_standard_ChIP_seq_CW34
chr17	36349999	36350000	MACS_peak_1669	8.00	TCF7L2_standard_ChIP_seq_CW34
chr17	36571979	36571980	MACS_peak_1670	14.00	TCF7L2_standard_ChIP_seq_CW34
chr17	36777175	36777176	MACS_peak_1671	29.00	TCF7L2_standard_ChIP_seq_CW34
chr17	36860240	36860241	MACS_peak_1672	120.00	TCF7L2_standard_ChIP_seq_CW34
chr17	36887220	36887221	MACS_peak_1673	8.00	TCF7L2_standard_ChIP_seq_CW34
chr17	36956240	36956241	MACS_peak_1674	9.00	TCF7L2_standard_ChIP_seq_CW34
chr17	37607660	37607661	MACS_peak_1675	33.00	TCF7L2_standard_ChIP_seq_CW34
chr17	37617583	37617584	MACS_peak_1676	8.00	TCF7L2_standard_ChIP_seq_CW34
chr17	37893150	37893151	MACS_peak_1677	46.00	TCF7L2_standard_ChIP_seq_CW34
chr17	37910296	37910297	MACS_peak_1678	22.00	TCF7L2_standard_ChIP_seq_CW34
chr17	38020674	38020675	MACS_peak_1679	7.00	TCF7L2_standard_ChIP_seq_CW34
chr17	38257261	38257262	MACS_peak_1680	43.00	TCF7L2_standard_ChIP_seq_CW34
chr17	38296720	38296721	MACS_peak_1681	9.00	TCF7L2_standard_ChIP_seq_CW34
chr17	38329839	38329840	MACS_peak_1682	13.00	TCF7L2_standard_ChIP_seq_CW34
chr17	38705563	38705564	MACS_peak_1683	11.00	TCF7L2_standard_ChIP_seq_CW34
chr17	38804153	38804154	MACS_peak_1684	8.00	TCF7L2_standard_ChIP_seq_CW34
chr17	39113280	39113281	MACS_peak_1685	36.00	TCF7L2_standard_ChIP_seq_CW34
chr17	39165627	39165628	MACS_peak_1686	30.00	TCF7L2_standard_ChIP_seq_CW34
chr17	39215660	39215661	MACS_peak_1687	22.00	TCF7L2_standard_ChIP_seq_CW34
chr17	39560459	39560460	MACS_peak_1688	13.00	TCF7L2_standard_ChIP_seq_CW34
chr17	39968969	39968970	MACS_peak_1689	10.00	TCF7L2_standard_ChIP_seq_CW34
chr17	40398793	40398794	MACS_peak_1690	7.00	TCF7L2_standard_ChIP_seq_CW34
chr17	40636529	40636530	MACS_peak_1691	21.00	TCF7L2_standard_ChIP_seq_CW34
chr17	40786172	40786173	MACS_peak_1692	8.00	TCF7L2_standard_ChIP_seq_CW34
chr17	41221028	41221029	MACS_peak_1693	22.00	TCF7L2_standard_ChIP_seq_CW34
chr17	41286869	41286870	MACS_peak_1694	17.00	TCF7L2_standard_ChIP_seq_CW34
chr17	41400541	41400542	MACS_peak_1696	43.00	TCF7L2_standard_ChIP_seq_CW34
chr17	41527073	41527074	MACS_peak_1698	7.00	TCF7L2_standard_ChIP_seq_CW34
chr17	41547295	41547296	MACS_peak_1699	12.00	TCF7L2_standard_ChIP_seq_CW34
chr17	41557571	41557572	MACS_peak_1700	11.00	TCF7L2_standard_ChIP_seq_CW34
chr17	41608382	41608383	MACS_peak_1701	32.00	TCF7L2_standard_ChIP_seq_CW34
chr17	41623026	41623027	MACS_peak_1702	36.00	TCF7L2_standard_ChIP_seq_CW34
chr17	41649926	41649927	MACS_peak_1703	12.00	TCF7L2_standard_ChIP_seq_CW34
chr17	41879384	41879385	MACS_peak_1704	15.00	TCF7L2_standard_ChIP_seq_CW34
chr17	42004575	42004576	MACS_peak_1705	9.00	TCF7L2_standard_ChIP_seq_CW34
chr17	42061372	42061373	MACS_peak_1706	10.00	TCF7L2_standard_ChIP_seq_CW34
chr17	43210145	43210146	MACS_peak_1707	10.00	TCF7L2_standard_ChIP_seq_CW34
chr17	43238858	43238859	MACS_peak_1708	11.00	TCF7L2_standard_ChIP_seq_CW34
chr17	43279445	43279446	MACS_peak_1709	19.00	TCF7L2_standard_ChIP_seq_CW34
chr17	43393984	43393985	MACS_peak_1710	7.00	TCF7L2_standard_ChIP_seq_CW34
chr17	43507153	43507154	MACS_peak_1711	8.00	TCF7L2_standard_ChIP_seq_CW34
chr17	45213172	45213173	MACS_peak_1712	7.00	TCF7L2_standard_ChIP_seq_CW34
chr17	45234333	45234334	MACS_peak_1713	22.00	TCF7L2_standard_ChIP_seq_CW34
chr17	45973824	45973825	MACS_peak_1714	5.00	TCF7L2_standard_ChIP_seq_CW34
chr17	46019318	46019319	MACS_peak_1715	10.00	TCF7L2_standard_ChIP_seq_CW34
chr17	46908098	46908099	MACS_peak_1716	9.00	TCF7L2_standard_ChIP_seq_CW34
chr17	47048790	47048791	MACS_peak_1717	23.00	TCF7L2_standard_ChIP_seq_CW34
chr17	47184313	47184314	MACS_peak_1718	8.00	TCF7L2_standard_ChIP_seq_CW34
chr17	47308247	47308248	MACS_peak_1719	51.00	TCF7L2_standard_ChIP_seq_CW34
chr17	47929077	47929078	MACS_peak_1720	17.00	TCF7L2_standard_ChIP_seq_CW34
chr17	48210721	48210722	MACS_peak_1721	11.00	TCF7L2_standard_ChIP_seq_CW34
chr17	48506456	48506457	MACS_peak_1722	13.00	TCF7L2_standard_ChIP_seq_CW34
chr17	48968297	48968298	MACS_peak_1723	15.00	TCF7L2_standard_ChIP_seq_CW34
chr17	52002027	52002028	MACS_peak_1724	57.00	TCF7L2_standard_ChIP_seq_CW34
chr17	52977805	52977806	MACS_peak_1725	11.00	TCF7L2_standard_ChIP_seq_CW34
chr17	53146461	53146462	MACS_peak_1726	8.00	TCF7L2_standard_ChIP_seq_CW34
chr17	53363404	53363405	MACS_peak_1727	28.00	TCF7L2_standard_ChIP_seq_CW34
chr17	53364467	53364468	MACS_peak_1728	29.00	TCF7L2_standard_ChIP_seq_CW34
chr17	53383807	53383808	MACS_peak_1729	45.00	TCF7L2_standard_ChIP_seq_CW34
chr17	53485535	53485536	MACS_peak_1730	8.00	TCF7L2_standard_ChIP_seq_CW34
chr17	55163233	55163234	MACS_peak_1731	8.00	TCF7L2_standard_ChIP_seq_CW34
chr17	55593864	55593865	MACS_peak_1732	10.00	TCF7L2_standard_ChIP_seq_CW34
chr17	55595081	55595082	MACS_peak_1733	13.00	TCF7L2_standard_ChIP_seq_CW34
chr17	55638938	55638939	MACS_peak_1734	50.00	TCF7L2_standard_ChIP_seq_CW34
chr17	55655389	55655390	MACS_peak_1735	12.00	TCF7L2_standard_ChIP_seq_CW34
chr17	55775857	55775858	MACS_peak_1736	23.00	TCF7L2_standard_ChIP_seq_CW34
chr17	55938591	55938592	MACS_peak_1737	31.00	TCF7L2_standard_ChIP_seq_CW34
chr17	55957374	55957375	MACS_peak_1738	8.00	TCF7L2_standard_ChIP_seq_CW34
chr17	56071058	56071059	MACS_peak_1739	12.00	TCF7L2_standard_ChIP_seq_CW34
chr17	56084823	56084824	MACS_peak_1740	18.00	TCF7L2_standard_ChIP_seq_CW34
chr17	56429850	56429851	MACS_peak_1741	16.00	TCF7L2_standard_ChIP_seq_CW34
chr17	56468947	56468948	MACS_peak_1742	58.00	TCF7L2_standard_ChIP_seq_CW34
chr17	56906884	56906885	MACS_peak_1743	11.00	TCF7L2_standard_ChIP_seq_CW34
chr17	57232425	57232426	MACS_peak_1744	12.00	TCF7L2_standard_ChIP_seq_CW34
chr17	57542711	57542712	MACS_peak_1745	11.00	TCF7L2_standard_ChIP_seq_CW34
chr17	57697193	57697194	MACS_peak_1746	7.00	TCF7L2_standard_ChIP_seq_CW34
chr17	57910462	57910463	MACS_peak_1747	11.00	TCF7L2_standard_ChIP_seq_CW34
chr17	58433579	58433580	MACS_peak_1748	12.00	TCF7L2_standard_ChIP_seq_CW34
chr17	58807444	58807445	MACS_peak_1749	11.00	TCF7L2_standard_ChIP_seq_CW34
chr17	59413592	59413593	MACS_peak_1750	47.00	TCF7L2_standard_ChIP_seq_CW34
chr17	59444516	59444517	MACS_peak_1751	15.00	TCF7L2_standard_ChIP_seq_CW34
chr17	59532535	59532536	MACS_peak_1752	17.00	TCF7L2_standard_ChIP_seq_CW34
chr17	59538358	59538359	MACS_peak_1753	24.00	TCF7L2_standard_ChIP_seq_CW34
chr17	59941218	59941219	MACS_peak_1754	7.00	TCF7L2_standard_ChIP_seq_CW34
chr17	60618140	60618141	MACS_peak_1755	8.00	TCF7L2_standard_ChIP_seq_CW34
chr17	61608679	61608680	MACS_peak_1756	21.00	TCF7L2_standard_ChIP_seq_CW34
chr17	61678278	61678279	MACS_peak_1757	7.00	TCF7L2_standard_ChIP_seq_CW34
chr17	62501714	62501715	MACS_peak_1758	9.00	TCF7L2_standard_ChIP_seq_CW34
chr17	62658286	62658287	MACS_peak_1759	22.00	TCF7L2_standard_ChIP_seq_CW34
chr17	62770419	62770420	MACS_peak_1760	8.00	TCF7L2_standard_ChIP_seq_CW34
chr17	63052398	63052399	MACS_peak_1761	15.00	TCF7L2_standard_ChIP_seq_CW34
chr17	63435423	63435424	MACS_peak_1762	7.00	TCF7L2_standard_ChIP_seq_CW34
chr17	63512281	63512282	MACS_peak_1763	23.00	TCF7L2_standard_ChIP_seq_CW34
chr17	63556864	63556865	MACS_peak_1764	208.00	TCF7L2_standard_ChIP_seq_CW34
chr17	63605598	63605599	MACS_peak_1765	9.00	TCF7L2_standard_ChIP_seq_CW34
chr17	63653171	63653172	MACS_peak_1766	22.00	TCF7L2_standard_ChIP_seq_CW34
chr17	63686863	63686864	MACS_peak_1767	14.00	TCF7L2_standard_ChIP_seq_CW34
chr17	63746935	63746936	MACS_peak_1768	20.00	TCF7L2_standard_ChIP_seq_CW34
chr17	64231622	64231623	MACS_peak_1769	15.00	TCF7L2_standard_ChIP_seq_CW34
chr17	64281370	64281371	MACS_peak_1770	7.00	TCF7L2_standard_ChIP_seq_CW34
chr17	64411709	64411710	MACS_peak_1771	30.00	TCF7L2_standard_ChIP_seq_CW34
chr17	64456140	64456141	MACS_peak_1772	12.00	TCF7L2_standard_ChIP_seq_CW34
chr17	64773432	64773433	MACS_peak_1773	7.00	TCF7L2_standard_ChIP_seq_CW34
chr17	66374373	66374374	MACS_peak_1774	13.00	TCF7L2_standard_ChIP_seq_CW34
chr17	66380156	66380157	MACS_peak_1775	81.00	TCF7L2_standard_ChIP_seq_CW34
chr17	66470685	66470686	MACS_peak_1776	12.00	TCF7L2_standard_ChIP_seq_CW34
chr17	66481610	66481611	MACS_peak_1777	15.00	TCF7L2_standard_ChIP_seq_CW34
chr17	66785528	66785529	MACS_peak_1778	13.00	TCF7L2_standard_ChIP_seq_CW34
chr17	67043801	67043802	MACS_peak_1779	19.00	TCF7L2_standard_ChIP_seq_CW34
chr17	67069135	67069136	MACS_peak_1780	62.00	TCF7L2_standard_ChIP_seq_CW34
chr17	67265093	67265094	MACS_peak_1781	8.00	TCF7L2_standard_ChIP_seq_CW34
chr17	70089119	70089120	MACS_peak_1782	16.00	TCF7L2_standard_ChIP_seq_CW34
chr17	70335605	70335606	MACS_peak_1783	8.00	TCF7L2_standard_ChIP_seq_CW34
chr17	70383065	70383066	MACS_peak_1784	7.00	TCF7L2_standard_ChIP_seq_CW34
chr17	70396053	70396054	MACS_peak_1785	39.00	TCF7L2_standard_ChIP_seq_CW34
chr17	70409482	70409483	MACS_peak_1786	80.00	TCF7L2_standard_ChIP_seq_CW34
chr17	70443651	70443652	MACS_peak_1787	14.00	TCF7L2_standard_ChIP_seq_CW34
chr17	70515318	70515319	MACS_peak_1788	13.00	TCF7L2_standard_ChIP_seq_CW34
chr17	70576917	70576918	MACS_peak_1789	16.00	TCF7L2_standard_ChIP_seq_CW34
chr17	70968665	70968666	MACS_peak_1790	12.00	TCF7L2_standard_ChIP_seq_CW34
chr17	71183643	71183644	MACS_peak_1791	33.00	TCF7L2_standard_ChIP_seq_CW34
chr17	72415018	72415019	MACS_peak_1792	23.00	TCF7L2_standard_ChIP_seq_CW34
chr17	72439190	72439191	MACS_peak_1793	27.00	TCF7L2_standard_ChIP_seq_CW34
chr17	73196172	73196173	MACS_peak_1794	18.00	TCF7L2_standard_ChIP_seq_CW34
chr17	73379357	73379358	MACS_peak_1795	12.00	TCF7L2_standard_ChIP_seq_CW34
chr17	73573006	73573007	MACS_peak_1796	13.00	TCF7L2_standard_ChIP_seq_CW34
chr17	73693941	73693942	MACS_peak_1797	13.00	TCF7L2_standard_ChIP_seq_CW34
chr17	73843332	73843333	MACS_peak_1798	18.00	TCF7L2_standard_ChIP_seq_CW34
chr17	74115851	74115852	MACS_peak_1799	20.00	TCF7L2_standard_ChIP_seq_CW34
chr17	74128124	74128125	MACS_peak_1800	9.00	TCF7L2_standard_ChIP_seq_CW34
chr17	74134658	74134659	MACS_peak_1801	8.00	TCF7L2_standard_ChIP_seq_CW34
chr17	74479769	74479770	MACS_peak_1802	12.00	TCF7L2_standard_ChIP_seq_CW34
chr17	74495551	74495552	MACS_peak_1803	35.00	TCF7L2_standard_ChIP_seq_CW34
chr17	74722624	74722625	MACS_peak_1804	12.00	TCF7L2_standard_ChIP_seq_CW34
chr17	74758900	74758901	MACS_peak_1805	48.00	TCF7L2_standard_ChIP_seq_CW34
chr17	74911210	74911211	MACS_peak_1806	76.00	TCF7L2_standard_ChIP_seq_CW34
chr17	74998457	74998458	MACS_peak_1807	12.00	TCF7L2_standard_ChIP_seq_CW34
chr17	75296571	75296572	MACS_peak_1808	13.00	TCF7L2_standard_ChIP_seq_CW34
chr17	75373488	75373489	MACS_peak_1809	10.00	TCF7L2_standard_ChIP_seq_CW34
chr17	75447539	75447540	MACS_peak_1810	26.00	TCF7L2_standard_ChIP_seq_CW34
chr17	75508287	75508288	MACS_peak_1811	12.00	TCF7L2_standard_ChIP_seq_CW34
chr17	76417737	76417738	MACS_peak_1812	9.00	TCF7L2_standard_ChIP_seq_CW34
chr17	76646481	76646482	MACS_peak_1813	29.00	TCF7L2_standard_ChIP_seq_CW34
chr17	76757511	76757512	MACS_peak_1814	6.00	TCF7L2_standard_ChIP_seq_CW34
chr17	76943805	76943806	MACS_peak_1815	9.00	TCF7L2_standard_ChIP_seq_CW34
chr17	76958670	76958671	MACS_peak_1816	9.00	TCF7L2_standard_ChIP_seq_CW34
chr17	77900146	77900147	MACS_peak_1817	21.00	TCF7L2_standard_ChIP_seq_CW34
chr17	77962059	77962060	MACS_peak_1818	33.00	TCF7L2_standard_ChIP_seq_CW34
chr17	78647407	78647408	MACS_peak_1819	25.00	TCF7L2_standard_ChIP_seq_CW34
chr17	79360826	79360827	MACS_peak_1820	9.00	TCF7L2_standard_ChIP_seq_CW34
chr17	79367793	79367794	MACS_peak_1821	68.00	TCF7L2_standard_ChIP_seq_CW34
chr17	79371312	79371313	MACS_peak_1822	56.00	TCF7L2_standard_ChIP_seq_CW34
chr17	79679459	79679460	MACS_peak_1823	24.00	TCF7L2_standard_ChIP_seq_CW34
chr17	79919163	79919164	MACS_peak_1824	52.00	TCF7L2_standard_ChIP_seq_CW34
chr17	79925992	79925993	MACS_peak_1825	64.00	TCF7L2_standard_ChIP_seq_CW34
chr17	79950299	79950300	MACS_peak_1826	58.00	TCF7L2_standard_ChIP_seq_CW34
chr17	79953043	79953044	MACS_peak_1827	28.00	TCF7L2_standard_ChIP_seq_CW34
chr17	79958789	79958790	MACS_peak_1828	96.00	TCF7L2_standard_ChIP_seq_CW34
chr17	79973801	79973802	MACS_peak_1829	14.00	TCF7L2_standard_ChIP_seq_CW34
chr17	79995751	79995752	MACS_peak_1830	9.00	TCF7L2_standard_ChIP_seq_CW34
chr17	80186412	80186413	MACS_peak_1831	10.00	TCF7L2_standard_ChIP_seq_CW34
chr17	80279429	80279430	MACS_peak_1832	30.00	TCF7L2_standard_ChIP_seq_CW34
chr17	80294510	80294511	MACS_peak_1833	16.00	TCF7L2_standard_ChIP_seq_CW34
chr17	80416606	80416607	MACS_peak_1834	31.00	TCF7L2_standard_ChIP_seq_CW34
chr17	80526458	80526459	MACS_peak_1835	7.00	TCF7L2_standard_ChIP_seq_CW34
chr17	80606056	80606057	MACS_peak_1836	8.00	TCF7L2_standard_ChIP_seq_CW34
chr17	80710099	80710100	MACS_peak_1837	8.00	TCF7L2_standard_ChIP_seq_CW34
chr18	572679	572680	MACS_peak_1839	11.00	TCF7L2_standard_ChIP_seq_CW34
chr18	657848	657849	MACS_peak_1840	5.00	TCF7L2_standard_ChIP_seq_CW34
chr18	812533	812534	MACS_peak_1841	6.00	TCF7L2_standard_ChIP_seq_CW34
chr18	2571230	2571231	MACS_peak_1842	7.00	TCF7L2_standard_ChIP_seq_CW34
chr18	2906201	2906202	MACS_peak_1843	17.00	TCF7L2_standard_ChIP_seq_CW34
chr18	2986508	2986509	MACS_peak_1844	12.00	TCF7L2_standard_ChIP_seq_CW34
chr18	3052237	3052238	MACS_peak_1845	22.00	TCF7L2_standard_ChIP_seq_CW34
chr18	3218994	3218995	MACS_peak_1846	8.00	TCF7L2_standard_ChIP_seq_CW34
chr18	3233983	3233984	MACS_peak_1847	22.00	TCF7L2_standard_ChIP_seq_CW34
chr18	3448476	3448477	MACS_peak_1848	10.00	TCF7L2_standard_ChIP_seq_CW34
chr18	3618355	3618356	MACS_peak_1849	36.00	TCF7L2_standard_ChIP_seq_CW34
chr18	3665880	3665881	MACS_peak_1850	15.00	TCF7L2_standard_ChIP_seq_CW34
chr18	5310586	5310587	MACS_peak_1851	7.00	TCF7L2_standard_ChIP_seq_CW34
chr18	6835563	6835564	MACS_peak_1852	10.00	TCF7L2_standard_ChIP_seq_CW34
chr18	7646391	7646392	MACS_peak_1853	17.00	TCF7L2_standard_ChIP_seq_CW34
chr18	8609209	8609210	MACS_peak_1854	13.00	TCF7L2_standard_ChIP_seq_CW34
chr18	9094895	9094896	MACS_peak_1855	11.00	TCF7L2_standard_ChIP_seq_CW34
chr18	10454533	10454534	MACS_peak_1856	11.00	TCF7L2_standard_ChIP_seq_CW34
chr18	10464251	10464252	MACS_peak_1857	11.00	TCF7L2_standard_ChIP_seq_CW34
chr18	11500865	11500866	MACS_peak_1858	14.00	TCF7L2_standard_ChIP_seq_CW34
chr18	12008509	12008510	MACS_peak_1859	29.00	TCF7L2_standard_ChIP_seq_CW34
chr18	12730336	12730337	MACS_peak_1860	20.00	TCF7L2_standard_ChIP_seq_CW34
chr18	13216584	13216585	MACS_peak_1861	8.00	TCF7L2_standard_ChIP_seq_CW34
chr18	13418481	13418482	MACS_peak_1862	6.00	TCF7L2_standard_ChIP_seq_CW34
chr18	13542273	13542274	MACS_peak_1863	8.00	TCF7L2_standard_ChIP_seq_CW34
chr18	19266596	19266597	MACS_peak_1866	12.00	TCF7L2_standard_ChIP_seq_CW34
chr18	20121379	20121380	MACS_peak_1867	7.00	TCF7L2_standard_ChIP_seq_CW34
chr18	21656324	21656325	MACS_peak_1868	18.00	TCF7L2_standard_ChIP_seq_CW34
chr18	22657104	22657105	MACS_peak_1869	8.00	TCF7L2_standard_ChIP_seq_CW34
chr18	23408151	23408152	MACS_peak_1870	6.00	TCF7L2_standard_ChIP_seq_CW34
chr18	23670705	23670706	MACS_peak_1871	10.00	TCF7L2_standard_ChIP_seq_CW34
chr18	24641654	24641655	MACS_peak_1872	25.00	TCF7L2_standard_ChIP_seq_CW34
chr18	28822058	28822059	MACS_peak_1873	14.00	TCF7L2_standard_ChIP_seq_CW34
chr18	29092368	29092369	MACS_peak_1874	27.00	TCF7L2_standard_ChIP_seq_CW34
chr18	30383769	30383770	MACS_peak_1875	20.00	TCF7L2_standard_ChIP_seq_CW34
chr18	30384709	30384710	MACS_peak_1876	11.00	TCF7L2_standard_ChIP_seq_CW34
chr18	34834866	34834867	MACS_peak_1877	5.00	TCF7L2_standard_ChIP_seq_CW34
chr18	36924589	36924590	MACS_peak_1878	11.00	TCF7L2_standard_ChIP_seq_CW34
chr18	37200884	37200885	MACS_peak_1879	8.00	TCF7L2_standard_ChIP_seq_CW34
chr18	40973475	40973476	MACS_peak_1880	7.00	TCF7L2_standard_ChIP_seq_CW34
chr18	43547254	43547255	MACS_peak_1881	6.00	TCF7L2_standard_ChIP_seq_CW34
chr18	43632321	43632322	MACS_peak_1882	20.00	TCF7L2_standard_ChIP_seq_CW34
chr18	44622359	44622360	MACS_peak_1883	10.00	TCF7L2_standard_ChIP_seq_CW34
chr18	44676323	44676324	MACS_peak_1884	5.00	TCF7L2_standard_ChIP_seq_CW34
chr18	45101587	45101588	MACS_peak_1885	8.00	TCF7L2_standard_ChIP_seq_CW34
chr18	46065352	46065353	MACS_peak_1886	15.00	TCF7L2_standard_ChIP_seq_CW34
chr18	46420104	46420105	MACS_peak_1887	25.00	TCF7L2_standard_ChIP_seq_CW34
chr18	46474971	46474972	MACS_peak_1888	19.00	TCF7L2_standard_ChIP_seq_CW34
chr18	47358611	47358612	MACS_peak_1889	8.00	TCF7L2_standard_ChIP_seq_CW34
chr18	47814418	47814419	MACS_peak_1890	7.00	TCF7L2_standard_ChIP_seq_CW34
chr18	47951719	47951720	MACS_peak_1891	10.00	TCF7L2_standard_ChIP_seq_CW34
chr18	48141343	48141344	MACS_peak_1892	11.00	TCF7L2_standard_ChIP_seq_CW34
chr18	54157945	54157946	MACS_peak_1893	6.00	TCF7L2_standard_ChIP_seq_CW34
chr18	55594834	55594835	MACS_peak_1894	7.00	TCF7L2_standard_ChIP_seq_CW34
chr18	55681023	55681024	MACS_peak_1895	15.00	TCF7L2_standard_ChIP_seq_CW34
chr18	56075156	56075157	MACS_peak_1896	21.00	TCF7L2_standard_ChIP_seq_CW34
chr18	56081415	56081416	MACS_peak_1897	20.00	TCF7L2_standard_ChIP_seq_CW34
chr18	56217317	56217318	MACS_peak_1898	11.00	TCF7L2_standard_ChIP_seq_CW34
chr18	56272803	56272804	MACS_peak_1899	17.00	TCF7L2_standard_ChIP_seq_CW34
chr18	57026026	57026027	MACS_peak_1900	12.00	TCF7L2_standard_ChIP_seq_CW34
chr18	57120998	57120999	MACS_peak_1901	10.00	TCF7L2_standard_ChIP_seq_CW34
chr18	60119701	60119702	MACS_peak_1902	10.00	TCF7L2_standard_ChIP_seq_CW34
chr18	60346304	60346305	MACS_peak_1903	8.00	TCF7L2_standard_ChIP_seq_CW34
chr18	63190634	63190635	MACS_peak_1904	6.00	TCF7L2_standard_ChIP_seq_CW34
chr18	66554827	66554828	MACS_peak_1905	8.00	TCF7L2_standard_ChIP_seq_CW34
chr18	67025955	67025956	MACS_peak_1906	12.00	TCF7L2_standard_ChIP_seq_CW34
chr18	67420322	67420323	MACS_peak_1907	13.00	TCF7L2_standard_ChIP_seq_CW34
chr18	67794695	67794696	MACS_peak_1908	8.00	TCF7L2_standard_ChIP_seq_CW34
chr18	67872999	67873000	MACS_peak_1909	6.00	TCF7L2_standard_ChIP_seq_CW34
chr18	68003256	68003257	MACS_peak_1910	25.00	TCF7L2_standard_ChIP_seq_CW34
chr18	68044695	68044696	MACS_peak_1911	10.00	TCF7L2_standard_ChIP_seq_CW34
chr18	68098571	68098572	MACS_peak_1912	14.00	TCF7L2_standard_ChIP_seq_CW34
chr18	71959736	71959737	MACS_peak_1913	33.00	TCF7L2_standard_ChIP_seq_CW34
chr18	72209367	72209368	MACS_peak_1914	8.00	TCF7L2_standard_ChIP_seq_CW34
chr18	73003034	73003035	MACS_peak_1915	9.00	TCF7L2_standard_ChIP_seq_CW34
chr18	73544094	73544095	MACS_peak_1916	13.00	TCF7L2_standard_ChIP_seq_CW34
chr18	74312261	74312262	MACS_peak_1917	4.00	TCF7L2_standard_ChIP_seq_CW34
chr18	76774620	76774621	MACS_peak_1918	7.00	TCF7L2_standard_ChIP_seq_CW34
chr18	77694823	77694824	MACS_peak_1919	10.00	TCF7L2_standard_ChIP_seq_CW34
chr19	323817	323818	MACS_peak_1920	5.00	TCF7L2_standard_ChIP_seq_CW34
chr19	462103	462104	MACS_peak_1921	28.00	TCF7L2_standard_ChIP_seq_CW34
chr19	926860	926861	MACS_peak_1922	9.00	TCF7L2_standard_ChIP_seq_CW34
chr19	952808	952809	MACS_peak_1923	31.00	TCF7L2_standard_ChIP_seq_CW34
chr19	1028873	1028874	MACS_peak_1924	13.00	TCF7L2_standard_ChIP_seq_CW34
chr19	1075544	1075545	MACS_peak_1925	26.00	TCF7L2_standard_ChIP_seq_CW34
chr19	1250068	1250069	MACS_peak_1926	11.00	TCF7L2_standard_ChIP_seq_CW34
chr19	1266447	1266448	MACS_peak_1927	16.00	TCF7L2_standard_ChIP_seq_CW34
chr19	1325329	1325330	MACS_peak_1928	14.00	TCF7L2_standard_ChIP_seq_CW34
chr19	1651681	1651682	MACS_peak_1929	19.00	TCF7L2_standard_ChIP_seq_CW34
chr19	1755468	1755469	MACS_peak_1930	10.00	TCF7L2_standard_ChIP_seq_CW34
chr19	2047599	2047600	MACS_peak_1931	13.00	TCF7L2_standard_ChIP_seq_CW34
chr19	2060010	2060011	MACS_peak_1932	8.00	TCF7L2_standard_ChIP_seq_CW34
chr19	2169012	2169013	MACS_peak_1933	9.00	TCF7L2_standard_ChIP_seq_CW34
chr19	2237090	2237091	MACS_peak_1934	7.00	TCF7L2_standard_ChIP_seq_CW34
chr19	2309122	2309123	MACS_peak_1935	13.00	TCF7L2_standard_ChIP_seq_CW34
chr19	2463249	2463250	MACS_peak_1936	7.00	TCF7L2_standard_ChIP_seq_CW34
chr19	2599640	2599641	MACS_peak_1937	12.00	TCF7L2_standard_ChIP_seq_CW34
chr19	3186132	3186133	MACS_peak_1938	8.00	TCF7L2_standard_ChIP_seq_CW34
chr19	3300649	3300650	MACS_peak_1939	6.00	TCF7L2_standard_ChIP_seq_CW34
chr19	3414091	3414092	MACS_peak_1940	19.00	TCF7L2_standard_ChIP_seq_CW34
chr19	3500760	3500761	MACS_peak_1941	9.00	TCF7L2_standard_ChIP_seq_CW34
chr19	3507688	3507689	MACS_peak_1942	8.00	TCF7L2_standard_ChIP_seq_CW34
chr19	5880701	5880702	MACS_peak_1943	13.00	TCF7L2_standard_ChIP_seq_CW34
chr19	5975032	5975033	MACS_peak_1944	8.00	TCF7L2_standard_ChIP_seq_CW34
chr19	6246671	6246672	MACS_peak_1945	11.00	TCF7L2_standard_ChIP_seq_CW34
chr19	6406137	6406138	MACS_peak_1946	7.00	TCF7L2_standard_ChIP_seq_CW34
chr19	6604034	6604035	MACS_peak_1947	8.00	TCF7L2_standard_ChIP_seq_CW34
chr19	6720958	6720959	MACS_peak_1948	8.00	TCF7L2_standard_ChIP_seq_CW34
chr19	6737422	6737423	MACS_peak_1949	18.00	TCF7L2_standard_ChIP_seq_CW34
chr19	7717068	7717069	MACS_peak_1950	60.00	TCF7L2_standard_ChIP_seq_CW34
chr19	8323145	8323146	MACS_peak_1951	8.00	TCF7L2_standard_ChIP_seq_CW34
chr19	8439248	8439249	MACS_peak_1952	15.00	TCF7L2_standard_ChIP_seq_CW34
chr19	8741060	8741061	MACS_peak_1953	8.00	TCF7L2_standard_ChIP_seq_CW34
chr19	10197486	10197487	MACS_peak_1954	10.00	TCF7L2_standard_ChIP_seq_CW34
chr19	10305777	10305778	MACS_peak_1955	9.00	TCF7L2_standard_ChIP_seq_CW34
chr19	10443832	10443833	MACS_peak_1956	11.00	TCF7L2_standard_ChIP_seq_CW34
chr19	11071294	11071295	MACS_peak_1957	7.00	TCF7L2_standard_ChIP_seq_CW34
chr19	11849732	11849733	MACS_peak_1958	8.00	TCF7L2_standard_ChIP_seq_CW34
chr19	11877881	11877882	MACS_peak_1959	19.00	TCF7L2_standard_ChIP_seq_CW34
chr19	12436756	12436757	MACS_peak_1960	8.00	TCF7L2_standard_ChIP_seq_CW34
chr19	12444435	12444436	MACS_peak_1961	17.00	TCF7L2_standard_ChIP_seq_CW34
chr19	12512181	12512182	MACS_peak_1962	12.00	TCF7L2_standard_ChIP_seq_CW34
chr19	12552173	12552174	MACS_peak_1963	8.00	TCF7L2_standard_ChIP_seq_CW34
chr19	12911949	12911950	MACS_peak_1964	15.00	TCF7L2_standard_ChIP_seq_CW34
chr19	13137534	13137535	MACS_peak_1965	21.00	TCF7L2_standard_ChIP_seq_CW34
chr19	13703694	13703695	MACS_peak_1966	7.00	TCF7L2_standard_ChIP_seq_CW34
chr19	13957534	13957535	MACS_peak_1967	15.00	TCF7L2_standard_ChIP_seq_CW34
chr19	14010468	14010469	MACS_peak_1968	8.00	TCF7L2_standard_ChIP_seq_CW34
chr19	14630907	14630908	MACS_peak_1969	11.00	TCF7L2_standard_ChIP_seq_CW34
chr19	14727537	14727538	MACS_peak_1970	6.00	TCF7L2_standard_ChIP_seq_CW34
chr19	15408316	15408317	MACS_peak_1971	38.00	TCF7L2_standard_ChIP_seq_CW34
chr19	16208887	16208888	MACS_peak_1972	7.00	TCF7L2_standard_ChIP_seq_CW34
chr19	17186125	17186126	MACS_peak_1973	8.00	TCF7L2_standard_ChIP_seq_CW34
chr19	17358845	17358846	MACS_peak_1974	19.00	TCF7L2_standard_ChIP_seq_CW34
chr19	17448016	17448017	MACS_peak_1975	7.00	TCF7L2_standard_ChIP_seq_CW34
chr19	18491012	18491013	MACS_peak_1976	10.00	TCF7L2_standard_ChIP_seq_CW34
chr19	18625853	18625854	MACS_peak_1977	28.00	TCF7L2_standard_ChIP_seq_CW34
chr19	18722616	18722617	MACS_peak_1978	18.00	TCF7L2_standard_ChIP_seq_CW34
chr19	18943002	18943003	MACS_peak_1979	8.00	TCF7L2_standard_ChIP_seq_CW34
chr19	21393034	21393035	MACS_peak_1980	19.00	TCF7L2_standard_ChIP_seq_CW34
chr19	29539929	29539930	MACS_peak_1984	7.00	TCF7L2_standard_ChIP_seq_CW34
chr19	30336500	30336501	MACS_peak_1985	6.00	TCF7L2_standard_ChIP_seq_CW34
chr19	31141049	31141050	MACS_peak_1986	9.00	TCF7L2_standard_ChIP_seq_CW34
chr19	31155714	31155715	MACS_peak_1987	8.00	TCF7L2_standard_ChIP_seq_CW34
chr19	31850709	31850710	MACS_peak_1988	8.00	TCF7L2_standard_ChIP_seq_CW34
chr19	32827460	32827461	MACS_peak_1989	17.00	TCF7L2_standard_ChIP_seq_CW34
chr19	32854195	32854196	MACS_peak_1990	7.00	TCF7L2_standard_ChIP_seq_CW34
chr19	33193161	33193162	MACS_peak_1991	7.00	TCF7L2_standard_ChIP_seq_CW34
chr19	33591926	33591927	MACS_peak_1992	23.00	TCF7L2_standard_ChIP_seq_CW34
chr19	33784464	33784465	MACS_peak_1993	19.00	TCF7L2_standard_ChIP_seq_CW34
chr19	34889156	34889157	MACS_peak_1994	7.00	TCF7L2_standard_ChIP_seq_CW34
chr19	34972556	34972557	MACS_peak_1995	8.00	TCF7L2_standard_ChIP_seq_CW34
chr19	35164675	35164676	MACS_peak_1996	10.00	TCF7L2_standard_ChIP_seq_CW34
chr19	35456699	35456700	MACS_peak_1997	6.00	TCF7L2_standard_ChIP_seq_CW34
chr19	35497375	35497376	MACS_peak_1998	24.00	TCF7L2_standard_ChIP_seq_CW34
chr19	35739974	35739975	MACS_peak_1999	6.00	TCF7L2_standard_ChIP_seq_CW34
chr19	36249431	36249432	MACS_peak_2001	11.00	TCF7L2_standard_ChIP_seq_CW34
chr19	36464791	36464792	MACS_peak_2002	6.00	TCF7L2_standard_ChIP_seq_CW34
chr19	36869983	36869984	MACS_peak_2003	6.00	TCF7L2_standard_ChIP_seq_CW34
chr19	37329186	37329187	MACS_peak_2004	8.00	TCF7L2_standard_ChIP_seq_CW34
chr19	38741554	38741555	MACS_peak_2006	9.00	TCF7L2_standard_ChIP_seq_CW34
chr19	39340420	39340421	MACS_peak_2007	7.00	TCF7L2_standard_ChIP_seq_CW34
chr19	39355659	39355660	MACS_peak_2008	7.00	TCF7L2_standard_ChIP_seq_CW34
chr19	40377932	40377933	MACS_peak_2009	9.00	TCF7L2_standard_ChIP_seq_CW34
chr19	40561938	40561939	MACS_peak_2010	11.00	TCF7L2_standard_ChIP_seq_CW34
chr19	40625504	40625505	MACS_peak_2011	11.00	TCF7L2_standard_ChIP_seq_CW34
chr19	40791612	40791613	MACS_peak_2012	15.00	TCF7L2_standard_ChIP_seq_CW34
chr19	41338006	41338007	MACS_peak_2013	7.00	TCF7L2_standard_ChIP_seq_CW34
chr19	41427376	41427377	MACS_peak_2014	13.00	TCF7L2_standard_ChIP_seq_CW34
chr19	41682210	41682211	MACS_peak_2015	16.00	TCF7L2_standard_ChIP_seq_CW34
chr19	42408744	42408745	MACS_peak_2016	7.00	TCF7L2_standard_ChIP_seq_CW34
chr19	42721375	42721376	MACS_peak_2017	9.00	TCF7L2_standard_ChIP_seq_CW34
chr19	44258745	44258746	MACS_peak_2018	7.00	TCF7L2_standard_ChIP_seq_CW34
chr19	45116879	45116880	MACS_peak_2020	24.00	TCF7L2_standard_ChIP_seq_CW34
chr19	45427650	45427651	MACS_peak_2021	13.00	TCF7L2_standard_ChIP_seq_CW34
chr19	45439307	45439308	MACS_peak_2022	9.00	TCF7L2_standard_ChIP_seq_CW34
chr19	45776435	45776436	MACS_peak_2023	63.00	TCF7L2_standard_ChIP_seq_CW34
chr19	46143238	46143239	MACS_peak_2024	7.00	TCF7L2_standard_ChIP_seq_CW34
chr19	46386255	46386256	MACS_peak_2025	15.00	TCF7L2_standard_ChIP_seq_CW34
chr19	47447039	47447040	MACS_peak_2026	9.00	TCF7L2_standard_ChIP_seq_CW34
chr19	47615917	47615918	MACS_peak_2027	7.00	TCF7L2_standard_ChIP_seq_CW34
chr19	47693758	47693759	MACS_peak_2028	10.00	TCF7L2_standard_ChIP_seq_CW34
chr19	48127513	48127514	MACS_peak_2029	17.00	TCF7L2_standard_ChIP_seq_CW34
chr19	48281792	48281793	MACS_peak_2030	9.00	TCF7L2_standard_ChIP_seq_CW34
chr19	48453608	48453609	MACS_peak_2031	8.00	TCF7L2_standard_ChIP_seq_CW34
chr19	48798919	48798920	MACS_peak_2032	16.00	TCF7L2_standard_ChIP_seq_CW34
chr19	48858009	48858010	MACS_peak_2033	27.00	TCF7L2_standard_ChIP_seq_CW34
chr19	49061768	49061769	MACS_peak_2034	47.00	TCF7L2_standard_ChIP_seq_CW34
chr19	49319638	49319639	MACS_peak_2035	6.00	TCF7L2_standard_ChIP_seq_CW34
chr19	49467166	49467167	MACS_peak_2036	8.00	TCF7L2_standard_ChIP_seq_CW34
chr19	49540283	49540284	MACS_peak_2037	15.00	TCF7L2_standard_ChIP_seq_CW34
chr19	49653728	49653729	MACS_peak_2038	10.00	TCF7L2_standard_ChIP_seq_CW34
chr19	50354360	50354361	MACS_peak_2039	9.00	TCF7L2_standard_ChIP_seq_CW34
chr19	50637155	50637156	MACS_peak_2040	7.00	TCF7L2_standard_ChIP_seq_CW34
chr19	51858246	51858247	MACS_peak_2041	9.00	TCF7L2_standard_ChIP_seq_CW34
chr19	52212931	52212932	MACS_peak_2042	6.00	TCF7L2_standard_ChIP_seq_CW34
chr19	52308238	52308239	MACS_peak_2043	14.00	TCF7L2_standard_ChIP_seq_CW34
chr19	52511728	52511729	MACS_peak_2044	13.00	TCF7L2_standard_ChIP_seq_CW34
chr19	53192416	53192417	MACS_peak_2045	8.00	TCF7L2_standard_ChIP_seq_CW34
chr19	54034936	54034937	MACS_peak_2046	9.00	TCF7L2_standard_ChIP_seq_CW34
chr19	54123206	54123207	MACS_peak_2047	38.00	TCF7L2_standard_ChIP_seq_CW34
chr19	54288628	54288629	MACS_peak_2048	19.00	TCF7L2_standard_ChIP_seq_CW34
chr19	54367838	54367839	MACS_peak_2049	27.00	TCF7L2_standard_ChIP_seq_CW34
chr19	55718265	55718266	MACS_peak_2050	11.00	TCF7L2_standard_ChIP_seq_CW34
chr19	56064830	56064831	MACS_peak_2051	9.00	TCF7L2_standard_ChIP_seq_CW34
chr19	56631970	56631971	MACS_peak_2052	8.00	TCF7L2_standard_ChIP_seq_CW34
chr19	57446358	57446359	MACS_peak_2053	11.00	TCF7L2_standard_ChIP_seq_CW34
chr19	57486353	57486354	MACS_peak_2054	15.00	TCF7L2_standard_ChIP_seq_CW34
chr19	58838357	58838358	MACS_peak_2055	7.00	TCF7L2_standard_ChIP_seq_CW34
chr19	58892106	58892107	MACS_peak_2056	13.00	TCF7L2_standard_ChIP_seq_CW34
chr2	108847	108848	MACS_peak_2058	9.00	TCF7L2_standard_ChIP_seq_CW34
chr2	176055	176056	MACS_peak_2059	34.00	TCF7L2_standard_ChIP_seq_CW34
chr2	249819	249820	MACS_peak_2060	9.00	TCF7L2_standard_ChIP_seq_CW34
chr2	260805	260806	MACS_peak_2061	14.00	TCF7L2_standard_ChIP_seq_CW34
chr2	2540268	2540269	MACS_peak_2062	7.00	TCF7L2_standard_ChIP_seq_CW34
chr2	2905914	2905915	MACS_peak_2063	9.00	TCF7L2_standard_ChIP_seq_CW34
chr2	3668620	3668621	MACS_peak_2064	7.00	TCF7L2_standard_ChIP_seq_CW34
chr2	5843210	5843211	MACS_peak_2065	8.00	TCF7L2_standard_ChIP_seq_CW34
chr2	6480368	6480369	MACS_peak_2066	8.00	TCF7L2_standard_ChIP_seq_CW34
chr2	8243394	8243395	MACS_peak_2067	8.00	TCF7L2_standard_ChIP_seq_CW34
chr2	8266147	8266148	MACS_peak_2068	24.00	TCF7L2_standard_ChIP_seq_CW34
chr2	8305812	8305813	MACS_peak_2069	9.00	TCF7L2_standard_ChIP_seq_CW34
chr2	8753147	8753148	MACS_peak_2070	25.00	TCF7L2_standard_ChIP_seq_CW34
chr2	8818501	8818502	MACS_peak_2071	13.00	TCF7L2_standard_ChIP_seq_CW34
chr2	9771068	9771069	MACS_peak_2072	10.00	TCF7L2_standard_ChIP_seq_CW34
chr2	11485134	11485135	MACS_peak_2073	17.00	TCF7L2_standard_ChIP_seq_CW34
chr2	11497711	11497712	MACS_peak_2074	128.00	TCF7L2_standard_ChIP_seq_CW34
chr2	11639544	11639545	MACS_peak_2075	22.00	TCF7L2_standard_ChIP_seq_CW34
chr2	11646155	11646156	MACS_peak_2076	7.00	TCF7L2_standard_ChIP_seq_CW34
chr2	11673524	11673525	MACS_peak_2077	16.00	TCF7L2_standard_ChIP_seq_CW34
chr2	11680110	11680111	MACS_peak_2078	27.00	TCF7L2_standard_ChIP_seq_CW34
chr2	11959555	11959556	MACS_peak_2079	17.00	TCF7L2_standard_ChIP_seq_CW34
chr2	11970288	11970289	MACS_peak_2080	9.00	TCF7L2_standard_ChIP_seq_CW34
chr2	12596760	12596761	MACS_peak_2081	27.00	TCF7L2_standard_ChIP_seq_CW34
chr2	12654968	12654969	MACS_peak_2082	11.00	TCF7L2_standard_ChIP_seq_CW34
chr2	12918594	12918595	MACS_peak_2083	62.00	TCF7L2_standard_ChIP_seq_CW34
chr2	13405547	13405548	MACS_peak_2084	7.00	TCF7L2_standard_ChIP_seq_CW34
chr2	14417587	14417588	MACS_peak_2085	5.00	TCF7L2_standard_ChIP_seq_CW34
chr2	15906650	15906651	MACS_peak_2086	16.00	TCF7L2_standard_ChIP_seq_CW34
chr2	15916886	15916887	MACS_peak_2087	10.00	TCF7L2_standard_ChIP_seq_CW34
chr2	15922381	15922382	MACS_peak_2088	13.00	TCF7L2_standard_ChIP_seq_CW34
chr2	16268291	16268292	MACS_peak_2089	11.00	TCF7L2_standard_ChIP_seq_CW34
chr2	16511975	16511976	MACS_peak_2090	20.00	TCF7L2_standard_ChIP_seq_CW34
chr2	16607199	16607200	MACS_peak_2091	39.00	TCF7L2_standard_ChIP_seq_CW34
chr2	17775332	17775333	MACS_peak_2092	18.00	TCF7L2_standard_ChIP_seq_CW34
chr2	18630606	18630607	MACS_peak_2093	7.00	TCF7L2_standard_ChIP_seq_CW34
chr2	18981874	18981875	MACS_peak_2094	48.00	TCF7L2_standard_ChIP_seq_CW34
chr2	20372479	20372480	MACS_peak_2095	23.00	TCF7L2_standard_ChIP_seq_CW34
chr2	20396097	20396098	MACS_peak_2096	9.00	TCF7L2_standard_ChIP_seq_CW34
chr2	20550750	20550751	MACS_peak_2097	12.00	TCF7L2_standard_ChIP_seq_CW34
chr2	20650214	20650215	MACS_peak_2098	14.00	TCF7L2_standard_ChIP_seq_CW34
chr2	20779216	20779217	MACS_peak_2099	12.00	TCF7L2_standard_ChIP_seq_CW34
chr2	20785360	20785361	MACS_peak_2100	10.00	TCF7L2_standard_ChIP_seq_CW34
chr2	20793290	20793291	MACS_peak_2101	41.00	TCF7L2_standard_ChIP_seq_CW34
chr2	20913569	20913570	MACS_peak_2102	11.00	TCF7L2_standard_ChIP_seq_CW34
chr2	21279462	21279463	MACS_peak_2103	57.00	TCF7L2_standard_ChIP_seq_CW34
chr2	25212645	25212646	MACS_peak_2104	7.00	TCF7L2_standard_ChIP_seq_CW34
chr2	25265299	25265300	MACS_peak_2105	10.00	TCF7L2_standard_ChIP_seq_CW34
chr2	26031649	26031650	MACS_peak_2106	40.00	TCF7L2_standard_ChIP_seq_CW34
chr2	26281611	26281612	MACS_peak_2107	7.00	TCF7L2_standard_ChIP_seq_CW34
chr2	27400693	27400694	MACS_peak_2108	43.00	TCF7L2_standard_ChIP_seq_CW34
chr2	27564018	27564019	MACS_peak_2109	6.00	TCF7L2_standard_ChIP_seq_CW34
chr2	27592973	27592974	MACS_peak_2110	99.00	TCF7L2_standard_ChIP_seq_CW34
chr2	27652080	27652081	MACS_peak_2111	38.00	TCF7L2_standard_ChIP_seq_CW34
chr2	27975747	27975748	MACS_peak_2112	11.00	TCF7L2_standard_ChIP_seq_CW34
chr2	28280390	28280391	MACS_peak_2113	6.00	TCF7L2_standard_ChIP_seq_CW34
chr2	28619479	28619480	MACS_peak_2114	16.00	TCF7L2_standard_ChIP_seq_CW34
chr2	28841235	28841236	MACS_peak_2115	12.00	TCF7L2_standard_ChIP_seq_CW34
chr2	29117541	29117542	MACS_peak_2116	6.00	TCF7L2_standard_ChIP_seq_CW34
chr2	31961814	31961815	MACS_peak_2117	8.00	TCF7L2_standard_ChIP_seq_CW34
chr2	31982830	31982831	MACS_peak_2118	6.00	TCF7L2_standard_ChIP_seq_CW34
chr2	32582561	32582562	MACS_peak_2119	14.00	TCF7L2_standard_ChIP_seq_CW34
chr2	33621698	33621699	MACS_peak_2120	10.00	TCF7L2_standard_ChIP_seq_CW34
chr2	35566919	35566920	MACS_peak_2121	6.00	TCF7L2_standard_ChIP_seq_CW34
chr2	36729346	36729347	MACS_peak_2122	78.00	TCF7L2_standard_ChIP_seq_CW34
chr2	37194798	37194799	MACS_peak_2123	11.00	TCF7L2_standard_ChIP_seq_CW34
chr2	37215357	37215358	MACS_peak_2124	7.00	TCF7L2_standard_ChIP_seq_CW34
chr2	37571834	37571835	MACS_peak_2125	6.00	TCF7L2_standard_ChIP_seq_CW34
chr2	38146054	38146055	MACS_peak_2126	42.00	TCF7L2_standard_ChIP_seq_CW34
chr2	38152341	38152342	MACS_peak_2127	11.00	TCF7L2_standard_ChIP_seq_CW34
chr2	38572633	38572634	MACS_peak_2128	8.00	TCF7L2_standard_ChIP_seq_CW34
chr2	38660254	38660255	MACS_peak_2129	11.00	TCF7L2_standard_ChIP_seq_CW34
chr2	38830312	38830313	MACS_peak_2130	36.00	TCF7L2_standard_ChIP_seq_CW34
chr2	39208848	39208849	MACS_peak_2131	26.00	TCF7L2_standard_ChIP_seq_CW34
chr2	39695233	39695234	MACS_peak_2132	13.00	TCF7L2_standard_ChIP_seq_CW34
chr2	39731490	39731491	MACS_peak_2133	9.00	TCF7L2_standard_ChIP_seq_CW34
chr2	39777998	39777999	MACS_peak_2134	10.00	TCF7L2_standard_ChIP_seq_CW34
chr2	42075347	42075348	MACS_peak_2135	15.00	TCF7L2_standard_ChIP_seq_CW34
chr2	42344095	42344096	MACS_peak_2136	14.00	TCF7L2_standard_ChIP_seq_CW34
chr2	42360355	42360356	MACS_peak_2137	24.00	TCF7L2_standard_ChIP_seq_CW34
chr2	42363151	42363152	MACS_peak_2138	27.00	TCF7L2_standard_ChIP_seq_CW34
chr2	42424219	42424220	MACS_peak_2139	10.00	TCF7L2_standard_ChIP_seq_CW34
chr2	43169590	43169591	MACS_peak_2140	7.00	TCF7L2_standard_ChIP_seq_CW34
chr2	43390153	43390154	MACS_peak_2141	19.00	TCF7L2_standard_ChIP_seq_CW34
chr2	43422175	43422176	MACS_peak_2142	21.00	TCF7L2_standard_ChIP_seq_CW34
chr2	43560113	43560114	MACS_peak_2143	32.00	TCF7L2_standard_ChIP_seq_CW34
chr2	43690466	43690467	MACS_peak_2144	15.00	TCF7L2_standard_ChIP_seq_CW34
chr2	43696316	43696317	MACS_peak_2145	9.00	TCF7L2_standard_ChIP_seq_CW34
chr2	44069839	44069840	MACS_peak_2146	37.00	TCF7L2_standard_ChIP_seq_CW34
chr2	46219553	46219554	MACS_peak_2147	11.00	TCF7L2_standard_ChIP_seq_CW34
chr2	46400939	46400940	MACS_peak_2148	39.00	TCF7L2_standard_ChIP_seq_CW34
chr2	46525420	46525421	MACS_peak_2149	25.00	TCF7L2_standard_ChIP_seq_CW34
chr2	46622048	46622049	MACS_peak_2150	10.00	TCF7L2_standard_ChIP_seq_CW34
chr2	47194491	47194492	MACS_peak_2151	14.00	TCF7L2_standard_ChIP_seq_CW34
chr2	47537347	47537348	MACS_peak_2152	9.00	TCF7L2_standard_ChIP_seq_CW34
chr2	48010416	48010417	MACS_peak_2153	8.00	TCF7L2_standard_ChIP_seq_CW34
chr2	48339032	48339033	MACS_peak_2154	6.00	TCF7L2_standard_ChIP_seq_CW34
chr2	48449166	48449167	MACS_peak_2155	17.00	TCF7L2_standard_ChIP_seq_CW34
chr2	53833219	53833220	MACS_peak_2156	8.00	TCF7L2_standard_ChIP_seq_CW34
chr2	54054132	54054133	MACS_peak_2157	8.00	TCF7L2_standard_ChIP_seq_CW34
chr2	54271321	54271322	MACS_peak_2158	11.00	TCF7L2_standard_ChIP_seq_CW34
chr2	54342792	54342793	MACS_peak_2159	8.00	TCF7L2_standard_ChIP_seq_CW34
chr2	54434997	54434998	MACS_peak_2160	42.00	TCF7L2_standard_ChIP_seq_CW34
chr2	54461544	54461545	MACS_peak_2161	25.00	TCF7L2_standard_ChIP_seq_CW34
chr2	54817291	54817292	MACS_peak_2162	7.00	TCF7L2_standard_ChIP_seq_CW34
chr2	54847224	54847225	MACS_peak_2163	11.00	TCF7L2_standard_ChIP_seq_CW34
chr2	54897108	54897109	MACS_peak_2164	15.00	TCF7L2_standard_ChIP_seq_CW34
chr2	55085665	55085666	MACS_peak_2165	34.00	TCF7L2_standard_ChIP_seq_CW34
chr2	55647107	55647108	MACS_peak_2166	44.00	TCF7L2_standard_ChIP_seq_CW34
chr2	58273831	58273832	MACS_peak_2167	10.00	TCF7L2_standard_ChIP_seq_CW34
chr2	58397882	58397883	MACS_peak_2168	29.00	TCF7L2_standard_ChIP_seq_CW34
chr2	58431978	58431979	MACS_peak_2169	6.00	TCF7L2_standard_ChIP_seq_CW34
chr2	59643148	59643149	MACS_peak_2170	6.00	TCF7L2_standard_ChIP_seq_CW34
chr2	60321929	60321930	MACS_peak_2171	6.00	TCF7L2_standard_ChIP_seq_CW34
chr2	62797209	62797210	MACS_peak_2172	7.00	TCF7L2_standard_ChIP_seq_CW34
chr2	62805278	62805279	MACS_peak_2173	10.00	TCF7L2_standard_ChIP_seq_CW34
chr2	62869938	62869939	MACS_peak_2174	12.00	TCF7L2_standard_ChIP_seq_CW34
chr2	63420212	63420213	MACS_peak_2175	59.00	TCF7L2_standard_ChIP_seq_CW34
chr2	63815983	63815984	MACS_peak_2176	7.00	TCF7L2_standard_ChIP_seq_CW34
chr2	64055181	64055182	MACS_peak_2177	14.00	TCF7L2_standard_ChIP_seq_CW34
chr2	64211617	64211618	MACS_peak_2178	18.00	TCF7L2_standard_ChIP_seq_CW34
chr2	64218732	64218733	MACS_peak_2179	23.00	TCF7L2_standard_ChIP_seq_CW34
chr2	64957765	64957766	MACS_peak_2180	8.00	TCF7L2_standard_ChIP_seq_CW34
chr2	65150110	65150111	MACS_peak_2181	62.00	TCF7L2_standard_ChIP_seq_CW34
chr2	65283929	65283930	MACS_peak_2182	8.00	TCF7L2_standard_ChIP_seq_CW34
chr2	65372322	65372323	MACS_peak_2183	8.00	TCF7L2_standard_ChIP_seq_CW34
chr2	65623592	65623593	MACS_peak_2184	10.00	TCF7L2_standard_ChIP_seq_CW34
chr2	65663831	65663832	MACS_peak_2185	14.00	TCF7L2_standard_ChIP_seq_CW34
chr2	67936612	67936613	MACS_peak_2186	7.00	TCF7L2_standard_ChIP_seq_CW34
chr2	68530407	68530408	MACS_peak_2187	11.00	TCF7L2_standard_ChIP_seq_CW34
chr2	69405390	69405391	MACS_peak_2188	13.00	TCF7L2_standard_ChIP_seq_CW34
chr2	69614703	69614704	MACS_peak_2189	6.00	TCF7L2_standard_ChIP_seq_CW34
chr2	69914273	69914274	MACS_peak_2190	10.00	TCF7L2_standard_ChIP_seq_CW34
chr2	70008844	70008845	MACS_peak_2191	19.00	TCF7L2_standard_ChIP_seq_CW34
chr2	70315258	70315259	MACS_peak_2192	31.00	TCF7L2_standard_ChIP_seq_CW34
chr2	70521134	70521135	MACS_peak_2193	9.00	TCF7L2_standard_ChIP_seq_CW34
chr2	70659428	70659429	MACS_peak_2194	6.00	TCF7L2_standard_ChIP_seq_CW34
chr2	70792365	70792366	MACS_peak_2195	11.00	TCF7L2_standard_ChIP_seq_CW34
chr2	72110239	72110240	MACS_peak_2196	9.00	TCF7L2_standard_ChIP_seq_CW34
chr2	72259581	72259582	MACS_peak_2197	9.00	TCF7L2_standard_ChIP_seq_CW34
chr2	73298421	73298422	MACS_peak_2198	7.00	TCF7L2_standard_ChIP_seq_CW34
chr2	74704294	74704295	MACS_peak_2199	7.00	TCF7L2_standard_ChIP_seq_CW34
chr2	74716339	74716340	MACS_peak_2200	39.00	TCF7L2_standard_ChIP_seq_CW34
chr2	74992061	74992062	MACS_peak_2201	26.00	TCF7L2_standard_ChIP_seq_CW34
chr2	75067626	75067627	MACS_peak_2202	16.00	TCF7L2_standard_ChIP_seq_CW34
chr2	75083588	75083589	MACS_peak_2203	11.00	TCF7L2_standard_ChIP_seq_CW34
chr2	75167401	75167402	MACS_peak_2204	16.00	TCF7L2_standard_ChIP_seq_CW34
chr2	75708663	75708664	MACS_peak_2205	13.00	TCF7L2_standard_ChIP_seq_CW34
chr2	75748836	75748837	MACS_peak_2206	18.00	TCF7L2_standard_ChIP_seq_CW34
chr2	75749834	75749835	MACS_peak_2207	25.00	TCF7L2_standard_ChIP_seq_CW34
chr2	75798358	75798359	MACS_peak_2208	11.00	TCF7L2_standard_ChIP_seq_CW34
chr2	75874137	75874138	MACS_peak_2209	5.00	TCF7L2_standard_ChIP_seq_CW34
chr2	76399497	76399498	MACS_peak_2210	12.00	TCF7L2_standard_ChIP_seq_CW34
chr2	77391356	77391357	MACS_peak_2211	11.00	TCF7L2_standard_ChIP_seq_CW34
chr2	78058388	78058389	MACS_peak_2212	9.00	TCF7L2_standard_ChIP_seq_CW34
chr2	78389564	78389565	MACS_peak_2213	57.00	TCF7L2_standard_ChIP_seq_CW34
chr2	78400407	78400408	MACS_peak_2214	5.00	TCF7L2_standard_ChIP_seq_CW34
chr2	78488219	78488220	MACS_peak_2215	15.00	TCF7L2_standard_ChIP_seq_CW34
chr2	78644022	78644023	MACS_peak_2216	9.00	TCF7L2_standard_ChIP_seq_CW34
chr2	79194525	79194526	MACS_peak_2217	11.00	TCF7L2_standard_ChIP_seq_CW34
chr2	83264542	83264543	MACS_peak_2218	6.00	TCF7L2_standard_ChIP_seq_CW34
chr2	83298644	83298645	MACS_peak_2219	10.00	TCF7L2_standard_ChIP_seq_CW34
chr2	84527584	84527585	MACS_peak_2220	148.00	TCF7L2_standard_ChIP_seq_CW34
chr2	84840395	84840396	MACS_peak_2221	40.00	TCF7L2_standard_ChIP_seq_CW34
chr2	85552173	85552174	MACS_peak_2222	11.00	TCF7L2_standard_ChIP_seq_CW34
chr2	85592881	85592882	MACS_peak_2223	10.00	TCF7L2_standard_ChIP_seq_CW34
chr2	85682945	85682946	MACS_peak_2224	14.00	TCF7L2_standard_ChIP_seq_CW34
chr2	85982003	85982004	MACS_peak_2225	16.00	TCF7L2_standard_ChIP_seq_CW34
chr2	86333192	86333193	MACS_peak_2226	9.00	TCF7L2_standard_ChIP_seq_CW34
chr2	86783767	86783768	MACS_peak_2227	17.00	TCF7L2_standard_ChIP_seq_CW34
chr2	86790304	86790305	MACS_peak_2228	10.00	TCF7L2_standard_ChIP_seq_CW34
chr2	86947395	86947396	MACS_peak_2229	8.00	TCF7L2_standard_ChIP_seq_CW34
chr2	87112748	87112749	MACS_peak_2230	7.00	TCF7L2_standard_ChIP_seq_CW34
chr2	87303644	87303645	MACS_peak_2231	8.00	TCF7L2_standard_ChIP_seq_CW34
chr2	87529225	87529226	MACS_peak_2232	8.00	TCF7L2_standard_ChIP_seq_CW34
chr2	87623919	87623920	MACS_peak_2233	10.00	TCF7L2_standard_ChIP_seq_CW34
chr2	87642073	87642074	MACS_peak_2234	10.00	TCF7L2_standard_ChIP_seq_CW34
chr2	88428109	88428110	MACS_peak_2235	31.00	TCF7L2_standard_ChIP_seq_CW34
chr2	88509956	88509957	MACS_peak_2236	10.00	TCF7L2_standard_ChIP_seq_CW34
chr2	88512069	88512070	MACS_peak_2237	16.00	TCF7L2_standard_ChIP_seq_CW34
chr2	88704530	88704531	MACS_peak_2238	6.00	TCF7L2_standard_ChIP_seq_CW34
chr2	91662860	91662861	MACS_peak_2243	5.00	TCF7L2_standard_ChIP_seq_CW34
chr2	91670253	91670254	MACS_peak_2244	40.00	TCF7L2_standard_ChIP_seq_CW34
chr2	91845062	91845063	MACS_peak_2245	10.00	TCF7L2_standard_ChIP_seq_CW34
chr2	91887698	91887699	MACS_peak_2246	7.00	TCF7L2_standard_ChIP_seq_CW34
chr2	97202674	97202675	MACS_peak_2254	9.00	TCF7L2_standard_ChIP_seq_CW34
chr2	97733452	97733453	MACS_peak_2255	5.00	TCF7L2_standard_ChIP_seq_CW34
chr2	98466652	98466653	MACS_peak_2256	6.00	TCF7L2_standard_ChIP_seq_CW34
chr2	99348238	99348239	MACS_peak_2257	8.00	TCF7L2_standard_ChIP_seq_CW34
chr2	99412638	99412639	MACS_peak_2258	17.00	TCF7L2_standard_ChIP_seq_CW34
chr2	99895460	99895461	MACS_peak_2259	26.00	TCF7L2_standard_ChIP_seq_CW34
chr2	100643679	100643680	MACS_peak_2260	6.00	TCF7L2_standard_ChIP_seq_CW34
chr2	100684347	100684348	MACS_peak_2261	6.00	TCF7L2_standard_ChIP_seq_CW34
chr2	101396748	101396749	MACS_peak_2262	9.00	TCF7L2_standard_ChIP_seq_CW34
chr2	101436956	101436957	MACS_peak_2263	8.00	TCF7L2_standard_ChIP_seq_CW34
chr2	101487578	101487579	MACS_peak_2264	8.00	TCF7L2_standard_ChIP_seq_CW34
chr2	101681932	101681933	MACS_peak_2265	13.00	TCF7L2_standard_ChIP_seq_CW34
chr2	101727148	101727149	MACS_peak_2266	11.00	TCF7L2_standard_ChIP_seq_CW34
chr2	101950078	101950079	MACS_peak_2267	13.00	TCF7L2_standard_ChIP_seq_CW34
chr2	102172801	102172802	MACS_peak_2268	21.00	TCF7L2_standard_ChIP_seq_CW34
chr2	102183379	102183380	MACS_peak_2269	131.00	TCF7L2_standard_ChIP_seq_CW34
chr2	102348030	102348031	MACS_peak_2270	15.00	TCF7L2_standard_ChIP_seq_CW34
chr2	102388507	102388508	MACS_peak_2271	11.00	TCF7L2_standard_ChIP_seq_CW34
chr2	102647434	102647435	MACS_peak_2272	20.00	TCF7L2_standard_ChIP_seq_CW34
chr2	102674536	102674537	MACS_peak_2273	58.00	TCF7L2_standard_ChIP_seq_CW34
chr2	102772644	102772645	MACS_peak_2274	53.00	TCF7L2_standard_ChIP_seq_CW34
chr2	103352357	103352358	MACS_peak_2275	9.00	TCF7L2_standard_ChIP_seq_CW34
chr2	106011504	106011505	MACS_peak_2276	14.00	TCF7L2_standard_ChIP_seq_CW34
chr2	106551191	106551192	MACS_peak_2277	22.00	TCF7L2_standard_ChIP_seq_CW34
chr2	106900048	106900049	MACS_peak_2278	30.00	TCF7L2_standard_ChIP_seq_CW34
chr2	107714663	107714664	MACS_peak_2279	8.00	TCF7L2_standard_ChIP_seq_CW34
chr2	109149643	109149644	MACS_peak_2280	12.00	TCF7L2_standard_ChIP_seq_CW34
chr2	110402408	110402409	MACS_peak_2281	18.00	TCF7L2_standard_ChIP_seq_CW34
chr2	110435015	110435016	MACS_peak_2282	19.00	TCF7L2_standard_ChIP_seq_CW34
chr2	110903946	110903947	MACS_peak_2283	12.00	TCF7L2_standard_ChIP_seq_CW34
chr2	110970123	110970124	MACS_peak_2284	8.00	TCF7L2_standard_ChIP_seq_CW34
chr2	111526352	111526353	MACS_peak_2285	18.00	TCF7L2_standard_ChIP_seq_CW34
chr2	111718763	111718764	MACS_peak_2286	8.00	TCF7L2_standard_ChIP_seq_CW34
chr2	112253936	112253937	MACS_peak_2287	8.00	TCF7L2_standard_ChIP_seq_CW34
chr2	112317068	112317069	MACS_peak_2288	9.00	TCF7L2_standard_ChIP_seq_CW34
chr2	112396285	112396286	MACS_peak_2289	8.00	TCF7L2_standard_ChIP_seq_CW34
chr2	112596799	112596800	MACS_peak_2290	9.00	TCF7L2_standard_ChIP_seq_CW34
chr2	112625623	112625624	MACS_peak_2291	7.00	TCF7L2_standard_ChIP_seq_CW34
chr2	112745061	112745062	MACS_peak_2292	26.00	TCF7L2_standard_ChIP_seq_CW34
chr2	112927403	112927404	MACS_peak_2293	27.00	TCF7L2_standard_ChIP_seq_CW34
chr2	113232759	113232760	MACS_peak_2294	8.00	TCF7L2_standard_ChIP_seq_CW34
chr2	113575799	113575800	MACS_peak_2295	19.00	TCF7L2_standard_ChIP_seq_CW34
chr2	113884647	113884648	MACS_peak_2296	10.00	TCF7L2_standard_ChIP_seq_CW34
chr2	114040504	114040505	MACS_peak_2297	8.00	TCF7L2_standard_ChIP_seq_CW34
chr2	114341291	114341292	MACS_peak_2298	7.00	TCF7L2_standard_ChIP_seq_CW34
chr2	114584391	114584392	MACS_peak_2299	11.00	TCF7L2_standard_ChIP_seq_CW34
chr2	114735895	114735896	MACS_peak_2300	10.00	TCF7L2_standard_ChIP_seq_CW34
chr2	114737996	114737997	MACS_peak_2301	15.00	TCF7L2_standard_ChIP_seq_CW34
chr2	114938701	114938702	MACS_peak_2302	5.00	TCF7L2_standard_ChIP_seq_CW34
chr2	115482357	115482358	MACS_peak_2303	7.00	TCF7L2_standard_ChIP_seq_CW34
chr2	120348036	120348037	MACS_peak_2304	7.00	TCF7L2_standard_ChIP_seq_CW34
chr2	120436105	120436106	MACS_peak_2305	83.00	TCF7L2_standard_ChIP_seq_CW34
chr2	121223802	121223803	MACS_peak_2306	93.00	TCF7L2_standard_ChIP_seq_CW34
chr2	121693482	121693483	MACS_peak_2307	26.00	TCF7L2_standard_ChIP_seq_CW34
chr2	121794147	121794148	MACS_peak_2308	10.00	TCF7L2_standard_ChIP_seq_CW34
chr2	122288153	122288154	MACS_peak_2309	7.00	TCF7L2_standard_ChIP_seq_CW34
chr2	122406955	122406956	MACS_peak_2310	10.00	TCF7L2_standard_ChIP_seq_CW34
chr2	122776699	122776700	MACS_peak_2311	6.00	TCF7L2_standard_ChIP_seq_CW34
chr2	123369015	123369016	MACS_peak_2312	8.00	TCF7L2_standard_ChIP_seq_CW34
chr2	123641214	123641215	MACS_peak_2313	7.00	TCF7L2_standard_ChIP_seq_CW34
chr2	128268904	128268905	MACS_peak_2314	10.00	TCF7L2_standard_ChIP_seq_CW34
chr2	128542825	128542826	MACS_peak_2315	7.00	TCF7L2_standard_ChIP_seq_CW34
chr2	128617077	128617078	MACS_peak_2316	15.00	TCF7L2_standard_ChIP_seq_CW34
chr2	128927897	128927898	MACS_peak_2317	28.00	TCF7L2_standard_ChIP_seq_CW34
chr2	128936404	128936405	MACS_peak_2318	13.00	TCF7L2_standard_ChIP_seq_CW34
chr2	129067313	129067314	MACS_peak_2319	15.00	TCF7L2_standard_ChIP_seq_CW34
chr2	131555314	131555315	MACS_peak_2320	13.00	TCF7L2_standard_ChIP_seq_CW34
chr2	131622567	131622568	MACS_peak_2321	6.00	TCF7L2_standard_ChIP_seq_CW34
chr2	132767494	132767495	MACS_peak_2322	8.00	TCF7L2_standard_ChIP_seq_CW34
chr2	133025801	133025802	MACS_peak_2324	68.00	TCF7L2_standard_ChIP_seq_CW34
chr2	135169946	135169947	MACS_peak_2325	8.00	TCF7L2_standard_ChIP_seq_CW34
chr2	137806213	137806214	MACS_peak_2326	10.00	TCF7L2_standard_ChIP_seq_CW34
chr2	138721880	138721881	MACS_peak_2327	8.00	TCF7L2_standard_ChIP_seq_CW34
chr2	138868234	138868235	MACS_peak_2328	12.00	TCF7L2_standard_ChIP_seq_CW34
chr2	138942234	138942235	MACS_peak_2329	13.00	TCF7L2_standard_ChIP_seq_CW34
chr2	139036419	139036420	MACS_peak_2330	7.00	TCF7L2_standard_ChIP_seq_CW34
chr2	139248959	139248960	MACS_peak_2331	22.00	TCF7L2_standard_ChIP_seq_CW34
chr2	140208739	140208740	MACS_peak_2332	5.00	TCF7L2_standard_ChIP_seq_CW34
chr2	143098308	143098309	MACS_peak_2333	6.00	TCF7L2_standard_ChIP_seq_CW34
chr2	143344395	143344396	MACS_peak_2334	6.00	TCF7L2_standard_ChIP_seq_CW34
chr2	143571870	143571871	MACS_peak_2335	15.00	TCF7L2_standard_ChIP_seq_CW34
chr2	143621235	143621236	MACS_peak_2336	9.00	TCF7L2_standard_ChIP_seq_CW34
chr2	146518928	146518929	MACS_peak_2337	8.00	TCF7L2_standard_ChIP_seq_CW34
chr2	146558248	146558249	MACS_peak_2338	19.00	TCF7L2_standard_ChIP_seq_CW34
chr2	146972247	146972248	MACS_peak_2339	8.00	TCF7L2_standard_ChIP_seq_CW34
chr2	149241191	149241192	MACS_peak_2340	8.00	TCF7L2_standard_ChIP_seq_CW34
chr2	149476969	149476970	MACS_peak_2341	15.00	TCF7L2_standard_ChIP_seq_CW34
chr2	152832124	152832125	MACS_peak_2342	12.00	TCF7L2_standard_ChIP_seq_CW34
chr2	152852488	152852489	MACS_peak_2343	25.00	TCF7L2_standard_ChIP_seq_CW34
chr2	153192104	153192105	MACS_peak_2344	10.00	TCF7L2_standard_ChIP_seq_CW34
chr2	153201274	153201275	MACS_peak_2345	45.00	TCF7L2_standard_ChIP_seq_CW34
chr2	153216175	153216176	MACS_peak_2346	8.00	TCF7L2_standard_ChIP_seq_CW34
chr2	153575003	153575004	MACS_peak_2347	8.00	TCF7L2_standard_ChIP_seq_CW34
chr2	157217573	157217574	MACS_peak_2348	5.00	TCF7L2_standard_ChIP_seq_CW34
chr2	158485211	158485212	MACS_peak_2349	58.00	TCF7L2_standard_ChIP_seq_CW34
chr2	159805958	159805959	MACS_peak_2350	17.00	TCF7L2_standard_ChIP_seq_CW34
chr2	160472494	160472495	MACS_peak_2351	12.00	TCF7L2_standard_ChIP_seq_CW34
chr2	160763131	160763132	MACS_peak_2352	19.00	TCF7L2_standard_ChIP_seq_CW34
chr2	161290927	161290928	MACS_peak_2353	9.00	TCF7L2_standard_ChIP_seq_CW34
chr2	161349244	161349245	MACS_peak_2354	27.00	TCF7L2_standard_ChIP_seq_CW34
chr2	161972419	161972420	MACS_peak_2355	14.00	TCF7L2_standard_ChIP_seq_CW34
chr2	162023725	162023726	MACS_peak_2356	10.00	TCF7L2_standard_ChIP_seq_CW34
chr2	162165345	162165346	MACS_peak_2359	10.00	TCF7L2_standard_ChIP_seq_CW34
chr2	162701554	162701555	MACS_peak_2360	16.00	TCF7L2_standard_ChIP_seq_CW34
chr2	162884420	162884421	MACS_peak_2361	15.00	TCF7L2_standard_ChIP_seq_CW34
chr2	162930696	162930697	MACS_peak_2362	13.00	TCF7L2_standard_ChIP_seq_CW34
chr2	163439132	163439133	MACS_peak_2363	18.00	TCF7L2_standard_ChIP_seq_CW34
chr2	164331092	164331093	MACS_peak_2364	39.00	TCF7L2_standard_ChIP_seq_CW34
chr2	165599457	165599458	MACS_peak_2365	8.00	TCF7L2_standard_ChIP_seq_CW34
chr2	165667475	165667476	MACS_peak_2366	38.00	TCF7L2_standard_ChIP_seq_CW34
chr2	165744104	165744105	MACS_peak_2367	17.00	TCF7L2_standard_ChIP_seq_CW34
chr2	165791686	165791687	MACS_peak_2368	40.00	TCF7L2_standard_ChIP_seq_CW34
chr2	166991930	166991931	MACS_peak_2369	22.00	TCF7L2_standard_ChIP_seq_CW34
chr2	167011339	167011340	MACS_peak_2370	8.00	TCF7L2_standard_ChIP_seq_CW34
chr2	167350324	167350325	MACS_peak_2371	7.00	TCF7L2_standard_ChIP_seq_CW34
chr2	168367100	168367101	MACS_peak_2372	8.00	TCF7L2_standard_ChIP_seq_CW34
chr2	168651252	168651253	MACS_peak_2373	15.00	TCF7L2_standard_ChIP_seq_CW34
chr2	169023641	169023642	MACS_peak_2374	11.00	TCF7L2_standard_ChIP_seq_CW34
chr2	169047304	169047305	MACS_peak_2375	11.00	TCF7L2_standard_ChIP_seq_CW34
chr2	171571794	171571795	MACS_peak_2376	103.00	TCF7L2_standard_ChIP_seq_CW34
chr2	171959589	171959590	MACS_peak_2377	9.00	TCF7L2_standard_ChIP_seq_CW34
chr2	172023210	172023211	MACS_peak_2378	7.00	TCF7L2_standard_ChIP_seq_CW34
chr2	172544272	172544273	MACS_peak_2379	6.00	TCF7L2_standard_ChIP_seq_CW34
chr2	172585918	172585919	MACS_peak_2380	10.00	TCF7L2_standard_ChIP_seq_CW34
chr2	172615493	172615494	MACS_peak_2381	21.00	TCF7L2_standard_ChIP_seq_CW34
chr2	172917259	172917260	MACS_peak_2382	14.00	TCF7L2_standard_ChIP_seq_CW34
chr2	173099892	173099893	MACS_peak_2383	9.00	TCF7L2_standard_ChIP_seq_CW34
chr2	173194923	173194924	MACS_peak_2384	14.00	TCF7L2_standard_ChIP_seq_CW34
chr2	173206015	173206016	MACS_peak_2385	9.00	TCF7L2_standard_ChIP_seq_CW34
chr2	174875761	174875762	MACS_peak_2386	9.00	TCF7L2_standard_ChIP_seq_CW34
chr2	175199670	175199671	MACS_peak_2387	38.00	TCF7L2_standard_ChIP_seq_CW34
chr2	175205170	175205171	MACS_peak_2388	45.00	TCF7L2_standard_ChIP_seq_CW34
chr2	176032944	176032945	MACS_peak_2389	10.00	TCF7L2_standard_ChIP_seq_CW34
chr2	176046216	176046217	MACS_peak_2390	6.00	TCF7L2_standard_ChIP_seq_CW34
chr2	177469630	177469631	MACS_peak_2391	5.00	TCF7L2_standard_ChIP_seq_CW34
chr2	178077858	178077859	MACS_peak_2392	16.00	TCF7L2_standard_ChIP_seq_CW34
chr2	178129852	178129853	MACS_peak_2393	50.00	TCF7L2_standard_ChIP_seq_CW34
chr2	178585991	178585992	MACS_peak_2394	10.00	TCF7L2_standard_ChIP_seq_CW34
chr2	179058383	179058384	MACS_peak_2395	10.00	TCF7L2_standard_ChIP_seq_CW34
chr2	179315707	179315708	MACS_peak_2396	8.00	TCF7L2_standard_ChIP_seq_CW34
chr2	179345127	179345128	MACS_peak_2397	9.00	TCF7L2_standard_ChIP_seq_CW34
chr2	180104218	180104219	MACS_peak_2398	6.00	TCF7L2_standard_ChIP_seq_CW34
chr2	180360391	180360392	MACS_peak_2399	5.00	TCF7L2_standard_ChIP_seq_CW34
chr2	180408298	180408299	MACS_peak_2400	36.00	TCF7L2_standard_ChIP_seq_CW34
chr2	180415091	180415092	MACS_peak_2401	9.00	TCF7L2_standard_ChIP_seq_CW34
chr2	180422877	180422878	MACS_peak_2402	12.00	TCF7L2_standard_ChIP_seq_CW34
chr2	180496701	180496702	MACS_peak_2403	21.00	TCF7L2_standard_ChIP_seq_CW34
chr2	182170485	182170486	MACS_peak_2404	16.00	TCF7L2_standard_ChIP_seq_CW34
chr2	182757057	182757058	MACS_peak_2405	10.00	TCF7L2_standard_ChIP_seq_CW34
chr2	183382523	183382524	MACS_peak_2406	6.00	TCF7L2_standard_ChIP_seq_CW34
chr2	185234251	185234252	MACS_peak_2407	12.00	TCF7L2_standard_ChIP_seq_CW34
chr2	186129374	186129375	MACS_peak_2408	6.00	TCF7L2_standard_ChIP_seq_CW34
chr2	186853726	186853727	MACS_peak_2409	9.00	TCF7L2_standard_ChIP_seq_CW34
chr2	187247775	187247776	MACS_peak_2410	7.00	TCF7L2_standard_ChIP_seq_CW34
chr2	188374528	188374529	MACS_peak_2411	28.00	TCF7L2_standard_ChIP_seq_CW34
chr2	188382653	188382654	MACS_peak_2412	24.00	TCF7L2_standard_ChIP_seq_CW34
chr2	188707505	188707506	MACS_peak_2413	7.00	TCF7L2_standard_ChIP_seq_CW34
chr2	189533210	189533211	MACS_peak_2414	5.00	TCF7L2_standard_ChIP_seq_CW34
chr2	189914910	189914911	MACS_peak_2415	42.00	TCF7L2_standard_ChIP_seq_CW34
chr2	190527621	190527622	MACS_peak_2416	8.00	TCF7L2_standard_ChIP_seq_CW34
chr2	191208730	191208731	MACS_peak_2417	6.00	TCF7L2_standard_ChIP_seq_CW34
chr2	191217810	191217811	MACS_peak_2418	9.00	TCF7L2_standard_ChIP_seq_CW34
chr2	191513259	191513260	MACS_peak_2419	8.00	TCF7L2_standard_ChIP_seq_CW34
chr2	191674255	191674256	MACS_peak_2420	11.00	TCF7L2_standard_ChIP_seq_CW34
chr2	192110862	192110863	MACS_peak_2421	32.00	TCF7L2_standard_ChIP_seq_CW34
chr2	192304373	192304374	MACS_peak_2422	25.00	TCF7L2_standard_ChIP_seq_CW34
chr2	192731814	192731815	MACS_peak_2423	21.00	TCF7L2_standard_ChIP_seq_CW34
chr2	192747258	192747259	MACS_peak_2424	15.00	TCF7L2_standard_ChIP_seq_CW34
chr2	192782195	192782196	MACS_peak_2425	12.00	TCF7L2_standard_ChIP_seq_CW34
chr2	194293301	194293302	MACS_peak_2426	20.00	TCF7L2_standard_ChIP_seq_CW34
chr2	194680963	194680964	MACS_peak_2427	8.00	TCF7L2_standard_ChIP_seq_CW34
chr2	198364680	198364681	MACS_peak_2428	13.00	TCF7L2_standard_ChIP_seq_CW34
chr2	198380213	198380214	MACS_peak_2429	12.00	TCF7L2_standard_ChIP_seq_CW34
chr2	198478455	198478456	MACS_peak_2430	18.00	TCF7L2_standard_ChIP_seq_CW34
chr2	200820330	200820331	MACS_peak_2431	8.00	TCF7L2_standard_ChIP_seq_CW34
chr2	201167957	201167958	MACS_peak_2432	21.00	TCF7L2_standard_ChIP_seq_CW34
chr2	201183840	201183841	MACS_peak_2433	8.00	TCF7L2_standard_ChIP_seq_CW34
chr2	201567600	201567601	MACS_peak_2434	31.00	TCF7L2_standard_ChIP_seq_CW34
chr2	201613002	201613003	MACS_peak_2435	11.00	TCF7L2_standard_ChIP_seq_CW34
chr2	201676973	201676974	MACS_peak_2436	9.00	TCF7L2_standard_ChIP_seq_CW34
chr2	201729583	201729584	MACS_peak_2437	17.00	TCF7L2_standard_ChIP_seq_CW34
chr2	201922408	201922409	MACS_peak_2438	10.00	TCF7L2_standard_ChIP_seq_CW34
chr2	202069812	202069813	MACS_peak_2439	14.00	TCF7L2_standard_ChIP_seq_CW34
chr2	202445381	202445382	MACS_peak_2440	10.00	TCF7L2_standard_ChIP_seq_CW34
chr2	203662158	203662159	MACS_peak_2441	7.00	TCF7L2_standard_ChIP_seq_CW34
chr2	203840001	203840002	MACS_peak_2442	5.00	TCF7L2_standard_ChIP_seq_CW34
chr2	204648744	204648745	MACS_peak_2443	36.00	TCF7L2_standard_ChIP_seq_CW34
chr2	204684607	204684608	MACS_peak_2444	10.00	TCF7L2_standard_ChIP_seq_CW34
chr2	205410772	205410773	MACS_peak_2445	6.00	TCF7L2_standard_ChIP_seq_CW34
chr2	205535052	205535053	MACS_peak_2446	12.00	TCF7L2_standard_ChIP_seq_CW34
chr2	206085512	206085513	MACS_peak_2447	9.00	TCF7L2_standard_ChIP_seq_CW34
chr2	206296572	206296573	MACS_peak_2448	6.00	TCF7L2_standard_ChIP_seq_CW34
chr2	206583803	206583804	MACS_peak_2449	12.00	TCF7L2_standard_ChIP_seq_CW34
chr2	206950523	206950524	MACS_peak_2450	17.00	TCF7L2_standard_ChIP_seq_CW34
chr2	208031386	208031387	MACS_peak_2451	9.00	TCF7L2_standard_ChIP_seq_CW34
chr2	208533688	208533689	MACS_peak_2452	6.00	TCF7L2_standard_ChIP_seq_CW34
chr2	208600107	208600108	MACS_peak_2453	10.00	TCF7L2_standard_ChIP_seq_CW34
chr2	208735134	208735135	MACS_peak_2454	24.00	TCF7L2_standard_ChIP_seq_CW34
chr2	211035315	211035316	MACS_peak_2455	8.00	TCF7L2_standard_ChIP_seq_CW34
chr2	211170883	211170884	MACS_peak_2456	5.00	TCF7L2_standard_ChIP_seq_CW34
chr2	211406338	211406339	MACS_peak_2457	13.00	TCF7L2_standard_ChIP_seq_CW34
chr2	211413857	211413858	MACS_peak_2458	22.00	TCF7L2_standard_ChIP_seq_CW34
chr2	213224420	213224421	MACS_peak_2459	9.00	TCF7L2_standard_ChIP_seq_CW34
chr2	214103799	214103800	MACS_peak_2460	8.00	TCF7L2_standard_ChIP_seq_CW34
chr2	214149290	214149291	MACS_peak_2461	9.00	TCF7L2_standard_ChIP_seq_CW34
chr2	216300347	216300348	MACS_peak_2462	8.00	TCF7L2_standard_ChIP_seq_CW34
chr2	216344724	216344725	MACS_peak_2463	8.00	TCF7L2_standard_ChIP_seq_CW34
chr2	216482071	216482072	MACS_peak_2464	21.00	TCF7L2_standard_ChIP_seq_CW34
chr2	216546027	216546028	MACS_peak_2465	13.00	TCF7L2_standard_ChIP_seq_CW34
chr2	216658840	216658841	MACS_peak_2466	11.00	TCF7L2_standard_ChIP_seq_CW34
chr2	216943609	216943610	MACS_peak_2467	13.00	TCF7L2_standard_ChIP_seq_CW34
chr2	217388117	217388118	MACS_peak_2468	28.00	TCF7L2_standard_ChIP_seq_CW34
chr2	217480473	217480474	MACS_peak_2469	8.00	TCF7L2_standard_ChIP_seq_CW34
chr2	219219894	219219895	MACS_peak_2470	13.00	TCF7L2_standard_ChIP_seq_CW34
chr2	219254607	219254608	MACS_peak_2471	22.00	TCF7L2_standard_ChIP_seq_CW34
chr2	219524370	219524371	MACS_peak_2472	16.00	TCF7L2_standard_ChIP_seq_CW34
chr2	219620672	219620673	MACS_peak_2473	12.00	TCF7L2_standard_ChIP_seq_CW34
chr2	219671235	219671236	MACS_peak_2474	9.00	TCF7L2_standard_ChIP_seq_CW34
chr2	219773932	219773933	MACS_peak_2475	6.00	TCF7L2_standard_ChIP_seq_CW34
chr2	219946054	219946055	MACS_peak_2476	46.00	TCF7L2_standard_ChIP_seq_CW34
chr2	223405770	223405771	MACS_peak_2477	6.00	TCF7L2_standard_ChIP_seq_CW34
chr2	223661522	223661523	MACS_peak_2478	45.00	TCF7L2_standard_ChIP_seq_CW34
chr2	223670968	223670969	MACS_peak_2479	20.00	TCF7L2_standard_ChIP_seq_CW34
chr2	225010780	225010781	MACS_peak_2480	12.00	TCF7L2_standard_ChIP_seq_CW34
chr2	225137201	225137202	MACS_peak_2481	9.00	TCF7L2_standard_ChIP_seq_CW34
chr2	226925173	226925174	MACS_peak_2482	6.00	TCF7L2_standard_ChIP_seq_CW34
chr2	226983632	226983633	MACS_peak_2483	14.00	TCF7L2_standard_ChIP_seq_CW34
chr2	227024836	227024837	MACS_peak_2484	16.00	TCF7L2_standard_ChIP_seq_CW34
chr2	227321035	227321036	MACS_peak_2485	37.00	TCF7L2_standard_ChIP_seq_CW34
chr2	227465118	227465119	MACS_peak_2486	7.00	TCF7L2_standard_ChIP_seq_CW34
chr2	227483729	227483730	MACS_peak_2487	37.00	TCF7L2_standard_ChIP_seq_CW34
chr2	227625661	227625662	MACS_peak_2488	15.00	TCF7L2_standard_ChIP_seq_CW34
chr2	227903432	227903433	MACS_peak_2489	13.00	TCF7L2_standard_ChIP_seq_CW34
chr2	228293794	228293795	MACS_peak_2490	11.00	TCF7L2_standard_ChIP_seq_CW34
chr2	228319463	228319464	MACS_peak_2491	13.00	TCF7L2_standard_ChIP_seq_CW34
chr2	228509095	228509096	MACS_peak_2492	29.00	TCF7L2_standard_ChIP_seq_CW34
chr2	228678517	228678518	MACS_peak_2493	22.00	TCF7L2_standard_ChIP_seq_CW34
chr2	230786713	230786714	MACS_peak_2495	27.00	TCF7L2_standard_ChIP_seq_CW34
chr2	231220889	231220890	MACS_peak_2496	13.00	TCF7L2_standard_ChIP_seq_CW34
chr2	231988389	231988390	MACS_peak_2497	7.00	TCF7L2_standard_ChIP_seq_CW34
chr2	232546087	232546088	MACS_peak_2498	11.00	TCF7L2_standard_ChIP_seq_CW34
chr2	232560349	232560350	MACS_peak_2499	11.00	TCF7L2_standard_ChIP_seq_CW34
chr2	232646155	232646156	MACS_peak_2500	7.00	TCF7L2_standard_ChIP_seq_CW34
chr2	232826384	232826385	MACS_peak_2501	8.00	TCF7L2_standard_ChIP_seq_CW34
chr2	233489862	233489863	MACS_peak_2502	24.00	TCF7L2_standard_ChIP_seq_CW34
chr2	233722105	233722106	MACS_peak_2503	8.00	TCF7L2_standard_ChIP_seq_CW34
chr2	233928348	233928349	MACS_peak_2504	23.00	TCF7L2_standard_ChIP_seq_CW34
chr2	233950176	233950177	MACS_peak_2505	7.00	TCF7L2_standard_ChIP_seq_CW34
chr2	234297042	234297043	MACS_peak_2506	9.00	TCF7L2_standard_ChIP_seq_CW34
chr2	234654274	234654275	MACS_peak_2507	34.00	TCF7L2_standard_ChIP_seq_CW34
chr2	234665583	234665584	MACS_peak_2508	60.00	TCF7L2_standard_ChIP_seq_CW34
chr2	234783306	234783307	MACS_peak_2509	6.00	TCF7L2_standard_ChIP_seq_CW34
chr2	234890647	234890648	MACS_peak_2510	31.00	TCF7L2_standard_ChIP_seq_CW34
chr2	235371429	235371430	MACS_peak_2511	12.00	TCF7L2_standard_ChIP_seq_CW34
chr2	235597915	235597916	MACS_peak_2512	12.00	TCF7L2_standard_ChIP_seq_CW34
chr2	235776636	235776637	MACS_peak_2513	31.00	TCF7L2_standard_ChIP_seq_CW34
chr2	235789358	235789359	MACS_peak_2514	31.00	TCF7L2_standard_ChIP_seq_CW34
chr2	235914416	235914417	MACS_peak_2515	24.00	TCF7L2_standard_ChIP_seq_CW34
chr2	236174180	236174181	MACS_peak_2516	12.00	TCF7L2_standard_ChIP_seq_CW34
chr2	236414067	236414068	MACS_peak_2517	9.00	TCF7L2_standard_ChIP_seq_CW34
chr2	236451024	236451025	MACS_peak_2518	31.00	TCF7L2_standard_ChIP_seq_CW34
chr2	236917626	236917627	MACS_peak_2519	13.00	TCF7L2_standard_ChIP_seq_CW34
chr2	237026746	237026747	MACS_peak_2520	19.00	TCF7L2_standard_ChIP_seq_CW34
chr2	237082093	237082094	MACS_peak_2521	14.00	TCF7L2_standard_ChIP_seq_CW34
chr2	237433354	237433355	MACS_peak_2522	6.00	TCF7L2_standard_ChIP_seq_CW34
chr2	238206223	238206224	MACS_peak_2523	8.00	TCF7L2_standard_ChIP_seq_CW34
chr2	238523002	238523003	MACS_peak_2524	12.00	TCF7L2_standard_ChIP_seq_CW34
chr2	238769861	238769862	MACS_peak_2525	8.00	TCF7L2_standard_ChIP_seq_CW34
chr2	238875482	238875483	MACS_peak_2526	9.00	TCF7L2_standard_ChIP_seq_CW34
chr2	238969509	238969510	MACS_peak_2527	10.00	TCF7L2_standard_ChIP_seq_CW34
chr2	239426931	239426932	MACS_peak_2528	15.00	TCF7L2_standard_ChIP_seq_CW34
chr2	239432104	239432105	MACS_peak_2529	16.00	TCF7L2_standard_ChIP_seq_CW34
chr2	239773064	239773065	MACS_peak_2530	21.00	TCF7L2_standard_ChIP_seq_CW34
chr2	240367147	240367148	MACS_peak_2531	10.00	TCF7L2_standard_ChIP_seq_CW34
chr2	240496550	240496551	MACS_peak_2532	17.00	TCF7L2_standard_ChIP_seq_CW34
chr2	241195569	241195570	MACS_peak_2533	41.00	TCF7L2_standard_ChIP_seq_CW34
chr2	241835600	241835601	MACS_peak_2534	8.00	TCF7L2_standard_ChIP_seq_CW34
chr2	241900249	241900250	MACS_peak_2535	22.00	TCF7L2_standard_ChIP_seq_CW34
chr2	242088723	242088724	MACS_peak_2536	9.00	TCF7L2_standard_ChIP_seq_CW34
chr2	242164293	242164294	MACS_peak_2537	11.00	TCF7L2_standard_ChIP_seq_CW34
chr2	242372441	242372442	MACS_peak_2538	11.00	TCF7L2_standard_ChIP_seq_CW34
chr2	242455402	242455403	MACS_peak_2539	9.00	TCF7L2_standard_ChIP_seq_CW34
chr2	242467640	242467641	MACS_peak_2540	14.00	TCF7L2_standard_ChIP_seq_CW34
chr20	308030	308031	MACS_peak_2541	14.00	TCF7L2_standard_ChIP_seq_CW34
chr20	352651	352652	MACS_peak_2542	17.00	TCF7L2_standard_ChIP_seq_CW34
chr20	361514	361515	MACS_peak_2543	9.00	TCF7L2_standard_ChIP_seq_CW34
chr20	623234	623235	MACS_peak_2544	20.00	TCF7L2_standard_ChIP_seq_CW34
chr20	634022	634023	MACS_peak_2545	15.00	TCF7L2_standard_ChIP_seq_CW34
chr20	656192	656193	MACS_peak_2546	13.00	TCF7L2_standard_ChIP_seq_CW34
chr20	814449	814450	MACS_peak_2547	11.00	TCF7L2_standard_ChIP_seq_CW34
chr20	829716	829717	MACS_peak_2548	8.00	TCF7L2_standard_ChIP_seq_CW34
chr20	1927992	1927993	MACS_peak_2549	17.00	TCF7L2_standard_ChIP_seq_CW34
chr20	2083155	2083156	MACS_peak_2550	15.00	TCF7L2_standard_ChIP_seq_CW34
chr20	2483847	2483848	MACS_peak_2551	13.00	TCF7L2_standard_ChIP_seq_CW34
chr20	2633188	2633189	MACS_peak_2552	11.00	TCF7L2_standard_ChIP_seq_CW34
chr20	3869625	3869626	MACS_peak_2553	11.00	TCF7L2_standard_ChIP_seq_CW34
chr20	4203210	4203211	MACS_peak_2554	18.00	TCF7L2_standard_ChIP_seq_CW34
chr20	4667728	4667729	MACS_peak_2555	17.00	TCF7L2_standard_ChIP_seq_CW34
chr20	4917824	4917825	MACS_peak_2556	23.00	TCF7L2_standard_ChIP_seq_CW34
chr20	4948074	4948075	MACS_peak_2557	14.00	TCF7L2_standard_ChIP_seq_CW34
chr20	4954385	4954386	MACS_peak_2558	49.00	TCF7L2_standard_ChIP_seq_CW34
chr20	5100413	5100414	MACS_peak_2559	8.00	TCF7L2_standard_ChIP_seq_CW34
chr20	5133706	5133707	MACS_peak_2560	13.00	TCF7L2_standard_ChIP_seq_CW34
chr20	5278203	5278204	MACS_peak_2561	10.00	TCF7L2_standard_ChIP_seq_CW34
chr20	5494994	5494995	MACS_peak_2562	17.00	TCF7L2_standard_ChIP_seq_CW34
chr20	5705418	5705419	MACS_peak_2563	9.00	TCF7L2_standard_ChIP_seq_CW34
chr20	5763250	5763251	MACS_peak_2564	18.00	TCF7L2_standard_ChIP_seq_CW34
chr20	5785631	5785632	MACS_peak_2565	7.00	TCF7L2_standard_ChIP_seq_CW34
chr20	5812363	5812364	MACS_peak_2566	20.00	TCF7L2_standard_ChIP_seq_CW34
chr20	5988413	5988414	MACS_peak_2567	21.00	TCF7L2_standard_ChIP_seq_CW34
chr20	7128017	7128018	MACS_peak_2568	12.00	TCF7L2_standard_ChIP_seq_CW34
chr20	8000554	8000555	MACS_peak_2569	16.00	TCF7L2_standard_ChIP_seq_CW34
chr20	8110335	8110336	MACS_peak_2570	7.00	TCF7L2_standard_ChIP_seq_CW34
chr20	8113203	8113204	MACS_peak_2571	10.00	TCF7L2_standard_ChIP_seq_CW34
chr20	8125494	8125495	MACS_peak_2572	9.00	TCF7L2_standard_ChIP_seq_CW34
chr20	8231457	8231458	MACS_peak_2573	20.00	TCF7L2_standard_ChIP_seq_CW34
chr20	10244532	10244533	MACS_peak_2574	9.00	TCF7L2_standard_ChIP_seq_CW34
chr20	10351261	10351262	MACS_peak_2575	40.00	TCF7L2_standard_ChIP_seq_CW34
chr20	10444423	10444424	MACS_peak_2576	8.00	TCF7L2_standard_ChIP_seq_CW34
chr20	10521539	10521540	MACS_peak_2577	22.00	TCF7L2_standard_ChIP_seq_CW34
chr20	10546467	10546468	MACS_peak_2578	23.00	TCF7L2_standard_ChIP_seq_CW34
chr20	11006470	11006471	MACS_peak_2579	7.00	TCF7L2_standard_ChIP_seq_CW34
chr20	11963419	11963420	MACS_peak_2580	13.00	TCF7L2_standard_ChIP_seq_CW34
chr20	12950129	12950130	MACS_peak_2581	8.00	TCF7L2_standard_ChIP_seq_CW34
chr20	13075822	13075823	MACS_peak_2582	14.00	TCF7L2_standard_ChIP_seq_CW34
chr20	13102694	13102695	MACS_peak_2583	35.00	TCF7L2_standard_ChIP_seq_CW34
chr20	13224669	13224670	MACS_peak_2584	9.00	TCF7L2_standard_ChIP_seq_CW34
chr20	13303464	13303465	MACS_peak_2585	11.00	TCF7L2_standard_ChIP_seq_CW34
chr20	13319921	13319922	MACS_peak_2586	14.00	TCF7L2_standard_ChIP_seq_CW34
chr20	13399465	13399466	MACS_peak_2587	14.00	TCF7L2_standard_ChIP_seq_CW34
chr20	14693329	14693330	MACS_peak_2588	5.00	TCF7L2_standard_ChIP_seq_CW34
chr20	14966068	14966069	MACS_peak_2589	8.00	TCF7L2_standard_ChIP_seq_CW34
chr20	15041380	15041381	MACS_peak_2590	40.00	TCF7L2_standard_ChIP_seq_CW34
chr20	15785890	15785891	MACS_peak_2591	8.00	TCF7L2_standard_ChIP_seq_CW34
chr20	17123432	17123433	MACS_peak_2592	8.00	TCF7L2_standard_ChIP_seq_CW34
chr20	17550987	17550988	MACS_peak_2593	9.00	TCF7L2_standard_ChIP_seq_CW34
chr20	17702869	17702870	MACS_peak_2594	98.00	TCF7L2_standard_ChIP_seq_CW34
chr20	17752172	17752173	MACS_peak_2595	49.00	TCF7L2_standard_ChIP_seq_CW34
chr20	17839791	17839792	MACS_peak_2596	42.00	TCF7L2_standard_ChIP_seq_CW34
chr20	18477485	18477486	MACS_peak_2597	11.00	TCF7L2_standard_ChIP_seq_CW34
chr20	19739422	19739423	MACS_peak_2598	12.00	TCF7L2_standard_ChIP_seq_CW34
chr20	19765509	19765510	MACS_peak_2599	7.00	TCF7L2_standard_ChIP_seq_CW34
chr20	19899217	19899218	MACS_peak_2600	51.00	TCF7L2_standard_ChIP_seq_CW34
chr20	20998540	20998541	MACS_peak_2601	15.00	TCF7L2_standard_ChIP_seq_CW34
chr20	21058688	21058689	MACS_peak_2602	25.00	TCF7L2_standard_ChIP_seq_CW34
chr20	21115788	21115789	MACS_peak_2603	28.00	TCF7L2_standard_ChIP_seq_CW34
chr20	22471686	22471687	MACS_peak_2604	9.00	TCF7L2_standard_ChIP_seq_CW34
chr20	22492824	22492825	MACS_peak_2605	12.00	TCF7L2_standard_ChIP_seq_CW34
chr20	22566139	22566140	MACS_peak_2606	12.00	TCF7L2_standard_ChIP_seq_CW34
chr20	23251311	23251312	MACS_peak_2607	10.00	TCF7L2_standard_ChIP_seq_CW34
chr20	23342616	23342617	MACS_peak_2608	11.00	TCF7L2_standard_ChIP_seq_CW34
chr20	23566658	23566659	MACS_peak_2609	18.00	TCF7L2_standard_ChIP_seq_CW34
chr20	23589352	23589353	MACS_peak_2610	11.00	TCF7L2_standard_ChIP_seq_CW34
chr20	24968143	24968144	MACS_peak_2611	14.00	TCF7L2_standard_ChIP_seq_CW34
chr20	25165188	25165189	MACS_peak_2612	23.00	TCF7L2_standard_ChIP_seq_CW34
chr20	25359199	25359200	MACS_peak_2613	52.00	TCF7L2_standard_ChIP_seq_CW34
chr20	25566165	25566166	MACS_peak_2614	10.00	TCF7L2_standard_ChIP_seq_CW34
chr20	26188862	26188863	MACS_peak_2615	47.00	TCF7L2_standard_ChIP_seq_CW34
chr20	26190074	26190075	MACS_peak_2616	34.00	TCF7L2_standard_ChIP_seq_CW34
chr20	29651132	29651133	MACS_peak_2617	13.00	TCF7L2_standard_ChIP_seq_CW34
chr20	30196486	30196487	MACS_peak_2618	83.00	TCF7L2_standard_ChIP_seq_CW34
chr20	30252275	30252276	MACS_peak_2619	15.00	TCF7L2_standard_ChIP_seq_CW34
chr20	30292604	30292605	MACS_peak_2620	38.00	TCF7L2_standard_ChIP_seq_CW34
chr20	30294665	30294666	MACS_peak_2621	22.00	TCF7L2_standard_ChIP_seq_CW34
chr20	30300599	30300600	MACS_peak_2622	15.00	TCF7L2_standard_ChIP_seq_CW34
chr20	30307789	30307790	MACS_peak_2623	24.00	TCF7L2_standard_ChIP_seq_CW34
chr20	30795485	30795486	MACS_peak_2624	6.00	TCF7L2_standard_ChIP_seq_CW34
chr20	30930205	30930206	MACS_peak_2625	12.00	TCF7L2_standard_ChIP_seq_CW34
chr20	31273695	31273696	MACS_peak_2626	82.00	TCF7L2_standard_ChIP_seq_CW34
chr20	31839310	31839311	MACS_peak_2627	7.00	TCF7L2_standard_ChIP_seq_CW34
chr20	32290115	32290116	MACS_peak_2628	16.00	TCF7L2_standard_ChIP_seq_CW34
chr20	32592718	32592719	MACS_peak_2629	42.00	TCF7L2_standard_ChIP_seq_CW34
chr20	32699514	32699515	MACS_peak_2630	6.00	TCF7L2_standard_ChIP_seq_CW34
chr20	32969230	32969231	MACS_peak_2631	6.00	TCF7L2_standard_ChIP_seq_CW34
chr20	33209698	33209699	MACS_peak_2632	12.00	TCF7L2_standard_ChIP_seq_CW34
chr20	33292282	33292283	MACS_peak_2633	10.00	TCF7L2_standard_ChIP_seq_CW34
chr20	33680604	33680605	MACS_peak_2634	8.00	TCF7L2_standard_ChIP_seq_CW34
chr20	35579916	35579917	MACS_peak_2635	17.00	TCF7L2_standard_ChIP_seq_CW34
chr20	35583436	35583437	MACS_peak_2636	23.00	TCF7L2_standard_ChIP_seq_CW34
chr20	35807606	35807607	MACS_peak_2637	14.00	TCF7L2_standard_ChIP_seq_CW34
chr20	35968969	35968970	MACS_peak_2638	28.00	TCF7L2_standard_ChIP_seq_CW34
chr20	36019678	36019679	MACS_peak_2639	12.00	TCF7L2_standard_ChIP_seq_CW34
chr20	36027396	36027397	MACS_peak_2640	17.00	TCF7L2_standard_ChIP_seq_CW34
chr20	36133863	36133864	MACS_peak_2641	17.00	TCF7L2_standard_ChIP_seq_CW34
chr20	36662059	36662060	MACS_peak_2642	7.00	TCF7L2_standard_ChIP_seq_CW34
chr20	36711470	36711471	MACS_peak_2643	33.00	TCF7L2_standard_ChIP_seq_CW34
chr20	36763346	36763347	MACS_peak_2644	10.00	TCF7L2_standard_ChIP_seq_CW34
chr20	37296387	37296388	MACS_peak_2645	20.00	TCF7L2_standard_ChIP_seq_CW34
chr20	39317877	39317878	MACS_peak_2646	14.00	TCF7L2_standard_ChIP_seq_CW34
chr20	39657622	39657623	MACS_peak_2647	8.00	TCF7L2_standard_ChIP_seq_CW34
chr20	39765923	39765924	MACS_peak_2648	8.00	TCF7L2_standard_ChIP_seq_CW34
chr20	40322195	40322196	MACS_peak_2649	17.00	TCF7L2_standard_ChIP_seq_CW34
chr20	42186698	42186699	MACS_peak_2650	11.00	TCF7L2_standard_ChIP_seq_CW34
chr20	42903030	42903031	MACS_peak_2651	14.00	TCF7L2_standard_ChIP_seq_CW34
chr20	42980943	42980944	MACS_peak_2652	19.00	TCF7L2_standard_ChIP_seq_CW34
chr20	43023241	43023242	MACS_peak_2653	82.00	TCF7L2_standard_ChIP_seq_CW34
chr20	43040041	43040042	MACS_peak_2654	15.00	TCF7L2_standard_ChIP_seq_CW34
chr20	43090235	43090236	MACS_peak_2655	10.00	TCF7L2_standard_ChIP_seq_CW34
chr20	44353887	44353888	MACS_peak_2656	14.00	TCF7L2_standard_ChIP_seq_CW34
chr20	44458525	44458526	MACS_peak_2657	29.00	TCF7L2_standard_ChIP_seq_CW34
chr20	44600165	44600166	MACS_peak_2658	10.00	TCF7L2_standard_ChIP_seq_CW34
chr20	45213398	45213399	MACS_peak_2659	12.00	TCF7L2_standard_ChIP_seq_CW34
chr20	45295793	45295794	MACS_peak_2660	114.00	TCF7L2_standard_ChIP_seq_CW34
chr20	45303138	45303139	MACS_peak_2661	22.00	TCF7L2_standard_ChIP_seq_CW34
chr20	45317613	45317614	MACS_peak_2662	7.00	TCF7L2_standard_ChIP_seq_CW34
chr20	45338166	45338167	MACS_peak_2663	7.00	TCF7L2_standard_ChIP_seq_CW34
chr20	45829573	45829574	MACS_peak_2664	12.00	TCF7L2_standard_ChIP_seq_CW34
chr20	45862340	45862341	MACS_peak_2665	16.00	TCF7L2_standard_ChIP_seq_CW34
chr20	45988038	45988039	MACS_peak_2666	10.00	TCF7L2_standard_ChIP_seq_CW34
chr20	46046743	46046744	MACS_peak_2667	11.00	TCF7L2_standard_ChIP_seq_CW34
chr20	46058127	46058128	MACS_peak_2668	6.00	TCF7L2_standard_ChIP_seq_CW34
chr20	46084210	46084211	MACS_peak_2669	30.00	TCF7L2_standard_ChIP_seq_CW34
chr20	46185294	46185295	MACS_peak_2670	13.00	TCF7L2_standard_ChIP_seq_CW34
chr20	46755209	46755210	MACS_peak_2672	27.00	TCF7L2_standard_ChIP_seq_CW34
chr20	47001846	47001847	MACS_peak_2673	32.00	TCF7L2_standard_ChIP_seq_CW34
chr20	47023423	47023424	MACS_peak_2674	13.00	TCF7L2_standard_ChIP_seq_CW34
chr20	47802220	47802221	MACS_peak_2676	17.00	TCF7L2_standard_ChIP_seq_CW34
chr20	47896096	47896097	MACS_peak_2677	10.00	TCF7L2_standard_ChIP_seq_CW34
chr20	48388996	48388997	MACS_peak_2678	13.00	TCF7L2_standard_ChIP_seq_CW34
chr20	48782404	48782405	MACS_peak_2679	8.00	TCF7L2_standard_ChIP_seq_CW34
chr20	49006071	49006072	MACS_peak_2680	80.00	TCF7L2_standard_ChIP_seq_CW34
chr20	49015815	49015816	MACS_peak_2681	47.00	TCF7L2_standard_ChIP_seq_CW34
chr20	49022527	49022528	MACS_peak_2682	19.00	TCF7L2_standard_ChIP_seq_CW34
chr20	49348274	49348275	MACS_peak_2683	8.00	TCF7L2_standard_ChIP_seq_CW34
chr20	49575837	49575838	MACS_peak_2684	6.00	TCF7L2_standard_ChIP_seq_CW34
chr20	50045262	50045263	MACS_peak_2685	21.00	TCF7L2_standard_ChIP_seq_CW34
chr20	50245747	50245748	MACS_peak_2686	11.00	TCF7L2_standard_ChIP_seq_CW34
chr20	50377724	50377725	MACS_peak_2687	31.00	TCF7L2_standard_ChIP_seq_CW34
chr20	50808287	50808288	MACS_peak_2688	9.00	TCF7L2_standard_ChIP_seq_CW34
chr20	51050167	51050168	MACS_peak_2689	7.00	TCF7L2_standard_ChIP_seq_CW34
chr20	51541415	51541416	MACS_peak_2690	42.00	TCF7L2_standard_ChIP_seq_CW34
chr20	51698631	51698632	MACS_peak_2691	14.00	TCF7L2_standard_ChIP_seq_CW34
chr20	52168777	52168778	MACS_peak_2692	39.00	TCF7L2_standard_ChIP_seq_CW34
chr20	52196214	52196215	MACS_peak_2693	40.00	TCF7L2_standard_ChIP_seq_CW34
chr20	52294364	52294365	MACS_peak_2694	13.00	TCF7L2_standard_ChIP_seq_CW34
chr20	52417586	52417587	MACS_peak_2695	58.00	TCF7L2_standard_ChIP_seq_CW34
chr20	52559693	52559694	MACS_peak_2696	9.00	TCF7L2_standard_ChIP_seq_CW34
chr20	52740529	52740530	MACS_peak_2697	23.00	TCF7L2_standard_ChIP_seq_CW34
chr20	52749513	52749514	MACS_peak_2698	60.00	TCF7L2_standard_ChIP_seq_CW34
chr20	52824297	52824298	MACS_peak_2699	58.00	TCF7L2_standard_ChIP_seq_CW34
chr20	55363532	55363533	MACS_peak_2700	14.00	TCF7L2_standard_ChIP_seq_CW34
chr20	55839417	55839418	MACS_peak_2701	11.00	TCF7L2_standard_ChIP_seq_CW34
chr20	56970852	56970853	MACS_peak_2702	9.00	TCF7L2_standard_ChIP_seq_CW34
chr20	57556137	57556138	MACS_peak_2703	24.00	TCF7L2_standard_ChIP_seq_CW34
chr20	57591411	57591412	MACS_peak_2704	29.00	TCF7L2_standard_ChIP_seq_CW34
chr20	57623028	57623029	MACS_peak_2705	10.00	TCF7L2_standard_ChIP_seq_CW34
chr20	58113572	58113573	MACS_peak_2706	7.00	TCF7L2_standard_ChIP_seq_CW34
chr20	60347032	60347033	MACS_peak_2707	15.00	TCF7L2_standard_ChIP_seq_CW34
chr20	61005464	61005465	MACS_peak_2708	26.00	TCF7L2_standard_ChIP_seq_CW34
chr20	61271271	61271272	MACS_peak_2709	18.00	TCF7L2_standard_ChIP_seq_CW34
chr20	61436310	61436311	MACS_peak_2710	14.00	TCF7L2_standard_ChIP_seq_CW34
chr20	61448679	61448680	MACS_peak_2711	16.00	TCF7L2_standard_ChIP_seq_CW34
chr20	61892942	61892943	MACS_peak_2712	14.00	TCF7L2_standard_ChIP_seq_CW34
chr20	61904312	61904313	MACS_peak_2713	9.00	TCF7L2_standard_ChIP_seq_CW34
chr20	61971023	61971024	MACS_peak_2714	11.00	TCF7L2_standard_ChIP_seq_CW34
chr20	62485817	62485818	MACS_peak_2715	23.00	TCF7L2_standard_ChIP_seq_CW34
chr20	62667522	62667523	MACS_peak_2716	25.00	TCF7L2_standard_ChIP_seq_CW34
chr21	9723350	9723351	MACS_peak_2719	9.00	TCF7L2_standard_ChIP_seq_CW34
chr21	10139902	10139903	MACS_peak_2722	8.00	TCF7L2_standard_ChIP_seq_CW34
chr21	10162599	10162600	MACS_peak_2723	4.00	TCF7L2_standard_ChIP_seq_CW34
chr21	10204535	10204536	MACS_peak_2725	6.00	TCF7L2_standard_ChIP_seq_CW34
chr21	11031969	11031970	MACS_peak_2729	9.00	TCF7L2_standard_ChIP_seq_CW34
chr21	11145464	11145465	MACS_peak_2730	13.00	TCF7L2_standard_ChIP_seq_CW34
chr21	14742982	14742983	MACS_peak_2732	6.00	TCF7L2_standard_ChIP_seq_CW34
chr21	16250282	16250283	MACS_peak_2733	8.00	TCF7L2_standard_ChIP_seq_CW34
chr21	16315567	16315568	MACS_peak_2734	12.00	TCF7L2_standard_ChIP_seq_CW34
chr21	16512969	16512970	MACS_peak_2735	11.00	TCF7L2_standard_ChIP_seq_CW34
chr21	16584079	16584080	MACS_peak_2736	20.00	TCF7L2_standard_ChIP_seq_CW34
chr21	16813663	16813664	MACS_peak_2737	9.00	TCF7L2_standard_ChIP_seq_CW34
chr21	16816721	16816722	MACS_peak_2738	10.00	TCF7L2_standard_ChIP_seq_CW34
chr21	18741117	18741118	MACS_peak_2739	6.00	TCF7L2_standard_ChIP_seq_CW34
chr21	18746825	18746826	MACS_peak_2740	12.00	TCF7L2_standard_ChIP_seq_CW34
chr21	18873894	18873895	MACS_peak_2741	59.00	TCF7L2_standard_ChIP_seq_CW34
chr21	18883865	18883866	MACS_peak_2742	9.00	TCF7L2_standard_ChIP_seq_CW34
chr21	18914916	18914917	MACS_peak_2743	14.00	TCF7L2_standard_ChIP_seq_CW34
chr21	19191779	19191780	MACS_peak_2744	13.00	TCF7L2_standard_ChIP_seq_CW34
chr21	24257828	24257829	MACS_peak_2745	7.00	TCF7L2_standard_ChIP_seq_CW34
chr21	30612399	30612400	MACS_peak_2746	7.00	TCF7L2_standard_ChIP_seq_CW34
chr21	30671522	30671523	MACS_peak_2747	7.00	TCF7L2_standard_ChIP_seq_CW34
chr21	33302010	33302011	MACS_peak_2748	32.00	TCF7L2_standard_ChIP_seq_CW34
chr21	33703310	33703311	MACS_peak_2749	19.00	TCF7L2_standard_ChIP_seq_CW34
chr21	33805527	33805528	MACS_peak_2750	8.00	TCF7L2_standard_ChIP_seq_CW34
chr21	34144382	34144383	MACS_peak_2751	8.00	TCF7L2_standard_ChIP_seq_CW34
chr21	34785334	34785335	MACS_peak_2752	7.00	TCF7L2_standard_ChIP_seq_CW34
chr21	35060217	35060218	MACS_peak_2753	13.00	TCF7L2_standard_ChIP_seq_CW34
chr21	35245606	35245607	MACS_peak_2754	19.00	TCF7L2_standard_ChIP_seq_CW34
chr21	35831995	35831996	MACS_peak_2755	8.00	TCF7L2_standard_ChIP_seq_CW34
chr21	36136743	36136744	MACS_peak_2756	7.00	TCF7L2_standard_ChIP_seq_CW34
chr21	36280997	36280998	MACS_peak_2757	17.00	TCF7L2_standard_ChIP_seq_CW34
chr21	36423742	36423743	MACS_peak_2758	9.00	TCF7L2_standard_ChIP_seq_CW34
chr21	37298087	37298088	MACS_peak_2759	6.00	TCF7L2_standard_ChIP_seq_CW34
chr21	37438067	37438068	MACS_peak_2760	5.00	TCF7L2_standard_ChIP_seq_CW34
chr21	40362543	40362544	MACS_peak_2761	16.00	TCF7L2_standard_ChIP_seq_CW34
chr21	40406272	40406273	MACS_peak_2762	8.00	TCF7L2_standard_ChIP_seq_CW34
chr21	40555301	40555302	MACS_peak_2763	6.00	TCF7L2_standard_ChIP_seq_CW34
chr21	40685620	40685621	MACS_peak_2764	7.00	TCF7L2_standard_ChIP_seq_CW34
chr21	42694676	42694677	MACS_peak_2765	38.00	TCF7L2_standard_ChIP_seq_CW34
chr21	43806581	43806582	MACS_peak_2766	20.00	TCF7L2_standard_ChIP_seq_CW34
chr21	44153586	44153587	MACS_peak_2767	7.00	TCF7L2_standard_ChIP_seq_CW34
chr21	44394141	44394142	MACS_peak_2768	6.00	TCF7L2_standard_ChIP_seq_CW34
chr21	44824017	44824018	MACS_peak_2769	37.00	TCF7L2_standard_ChIP_seq_CW34
chr21	44860994	44860995	MACS_peak_2770	30.00	TCF7L2_standard_ChIP_seq_CW34
chr21	44885139	44885140	MACS_peak_2771	8.00	TCF7L2_standard_ChIP_seq_CW34
chr21	44897968	44897969	MACS_peak_2772	8.00	TCF7L2_standard_ChIP_seq_CW34
chr21	45117794	45117795	MACS_peak_2773	11.00	TCF7L2_standard_ChIP_seq_CW34
chr21	45359190	45359191	MACS_peak_2774	8.00	TCF7L2_standard_ChIP_seq_CW34
chr21	45935041	45935042	MACS_peak_2775	13.00	TCF7L2_standard_ChIP_seq_CW34
chr21	46221706	46221707	MACS_peak_2776	8.00	TCF7L2_standard_ChIP_seq_CW34
chr21	46403068	46403069	MACS_peak_2777	17.00	TCF7L2_standard_ChIP_seq_CW34
chr21	46441702	46441703	MACS_peak_2778	11.00	TCF7L2_standard_ChIP_seq_CW34
chr21	46785793	46785794	MACS_peak_2779	19.00	TCF7L2_standard_ChIP_seq_CW34
chr21	46825903	46825904	MACS_peak_2780	26.00	TCF7L2_standard_ChIP_seq_CW34
chr21	46869006	46869007	MACS_peak_2781	33.00	TCF7L2_standard_ChIP_seq_CW34
chr21	46889712	46889713	MACS_peak_2782	11.00	TCF7L2_standard_ChIP_seq_CW34
chr22	18264223	18264224	MACS_peak_2783	10.00	TCF7L2_standard_ChIP_seq_CW34
chr22	18484534	18484535	MACS_peak_2784	6.00	TCF7L2_standard_ChIP_seq_CW34
chr22	18718146	18718147	MACS_peak_2785	35.00	TCF7L2_standard_ChIP_seq_CW34
chr22	18982343	18982344	MACS_peak_2787	11.00	TCF7L2_standard_ChIP_seq_CW34
chr22	19466769	19466770	MACS_peak_2788	8.00	TCF7L2_standard_ChIP_seq_CW34
chr22	19718661	19718662	MACS_peak_2789	5.00	TCF7L2_standard_ChIP_seq_CW34
chr22	19925232	19925233	MACS_peak_2790	12.00	TCF7L2_standard_ChIP_seq_CW34
chr22	19939854	19939855	MACS_peak_2791	6.00	TCF7L2_standard_ChIP_seq_CW34
chr22	21128313	21128314	MACS_peak_2792	10.00	TCF7L2_standard_ChIP_seq_CW34
chr22	21304214	21304215	MACS_peak_2793	11.00	TCF7L2_standard_ChIP_seq_CW34
chr22	21576100	21576101	MACS_peak_2794	8.00	TCF7L2_standard_ChIP_seq_CW34
chr22	22874649	22874650	MACS_peak_2795	8.00	TCF7L2_standard_ChIP_seq_CW34
chr22	23863936	23863937	MACS_peak_2796	25.00	TCF7L2_standard_ChIP_seq_CW34
chr22	23908500	23908501	MACS_peak_2797	9.00	TCF7L2_standard_ChIP_seq_CW34
chr22	24018026	24018027	MACS_peak_2798	10.00	TCF7L2_standard_ChIP_seq_CW34
chr22	24059426	24059427	MACS_peak_2799	7.00	TCF7L2_standard_ChIP_seq_CW34
chr22	24190690	24190691	MACS_peak_2800	9.00	TCF7L2_standard_ChIP_seq_CW34
chr22	24405761	24405762	MACS_peak_2801	22.00	TCF7L2_standard_ChIP_seq_CW34
chr22	24604129	24604130	MACS_peak_2802	9.00	TCF7L2_standard_ChIP_seq_CW34
chr22	24901525	24901526	MACS_peak_2803	19.00	TCF7L2_standard_ChIP_seq_CW34
chr22	24975048	24975049	MACS_peak_2804	22.00	TCF7L2_standard_ChIP_seq_CW34
chr22	25565836	25565837	MACS_peak_2805	9.00	TCF7L2_standard_ChIP_seq_CW34
chr22	25792108	25792109	MACS_peak_2806	11.00	TCF7L2_standard_ChIP_seq_CW34
chr22	26064646	26064647	MACS_peak_2807	17.00	TCF7L2_standard_ChIP_seq_CW34
chr22	28066263	28066264	MACS_peak_2808	18.00	TCF7L2_standard_ChIP_seq_CW34
chr22	28733574	28733575	MACS_peak_2809	7.00	TCF7L2_standard_ChIP_seq_CW34
chr22	29108486	29108487	MACS_peak_2810	10.00	TCF7L2_standard_ChIP_seq_CW34
chr22	29156118	29156119	MACS_peak_2811	19.00	TCF7L2_standard_ChIP_seq_CW34
chr22	29261059	29261060	MACS_peak_2812	9.00	TCF7L2_standard_ChIP_seq_CW34
chr22	29279615	29279616	MACS_peak_2813	52.00	TCF7L2_standard_ChIP_seq_CW34
chr22	29308456	29308457	MACS_peak_2814	14.00	TCF7L2_standard_ChIP_seq_CW34
chr22	29317043	29317044	MACS_peak_2815	31.00	TCF7L2_standard_ChIP_seq_CW34
chr22	29319993	29319994	MACS_peak_2816	94.00	TCF7L2_standard_ChIP_seq_CW34
chr22	29373720	29373721	MACS_peak_2817	11.00	TCF7L2_standard_ChIP_seq_CW34
chr22	29378102	29378103	MACS_peak_2818	14.00	TCF7L2_standard_ChIP_seq_CW34
chr22	29400769	29400770	MACS_peak_2819	31.00	TCF7L2_standard_ChIP_seq_CW34
chr22	29426872	29426873	MACS_peak_2820	49.00	TCF7L2_standard_ChIP_seq_CW34
chr22	29467637	29467638	MACS_peak_2821	10.00	TCF7L2_standard_ChIP_seq_CW34
chr22	29492868	29492869	MACS_peak_2822	12.00	TCF7L2_standard_ChIP_seq_CW34
chr22	30166000	30166001	MACS_peak_2823	15.00	TCF7L2_standard_ChIP_seq_CW34
chr22	30901199	30901200	MACS_peak_2824	7.00	TCF7L2_standard_ChIP_seq_CW34
chr22	31475265	31475266	MACS_peak_2825	8.00	TCF7L2_standard_ChIP_seq_CW34
chr22	31617970	31617971	MACS_peak_2826	10.00	TCF7L2_standard_ChIP_seq_CW34
chr22	32050509	32050510	MACS_peak_2827	6.00	TCF7L2_standard_ChIP_seq_CW34
chr22	32808129	32808130	MACS_peak_2828	10.00	TCF7L2_standard_ChIP_seq_CW34
chr22	32821089	32821090	MACS_peak_2829	10.00	TCF7L2_standard_ChIP_seq_CW34
chr22	32908808	32908809	MACS_peak_2830	13.00	TCF7L2_standard_ChIP_seq_CW34
chr22	32980219	32980220	MACS_peak_2831	12.00	TCF7L2_standard_ChIP_seq_CW34
chr22	33449478	33449479	MACS_peak_2832	7.00	TCF7L2_standard_ChIP_seq_CW34
chr22	33935905	33935906	MACS_peak_2833	9.00	TCF7L2_standard_ChIP_seq_CW34
chr22	33974110	33974111	MACS_peak_2834	13.00	TCF7L2_standard_ChIP_seq_CW34
chr22	35653703	35653704	MACS_peak_2835	9.00	TCF7L2_standard_ChIP_seq_CW34
chr22	35724685	35724686	MACS_peak_2836	10.00	TCF7L2_standard_ChIP_seq_CW34
chr22	35768773	35768774	MACS_peak_2837	9.00	TCF7L2_standard_ChIP_seq_CW34
chr22	36806545	36806546	MACS_peak_2838	5.00	TCF7L2_standard_ChIP_seq_CW34
chr22	36815523	36815524	MACS_peak_2839	24.00	TCF7L2_standard_ChIP_seq_CW34
chr22	36831786	36831787	MACS_peak_2840	14.00	TCF7L2_standard_ChIP_seq_CW34
chr22	36901514	36901515	MACS_peak_2841	28.00	TCF7L2_standard_ChIP_seq_CW34
chr22	37429103	37429104	MACS_peak_2842	31.00	TCF7L2_standard_ChIP_seq_CW34
chr22	38131302	38131303	MACS_peak_2843	10.00	TCF7L2_standard_ChIP_seq_CW34
chr22	38199054	38199055	MACS_peak_2844	16.00	TCF7L2_standard_ChIP_seq_CW34
chr22	38429053	38429054	MACS_peak_2845	9.00	TCF7L2_standard_ChIP_seq_CW34
chr22	39052554	39052555	MACS_peak_2846	7.00	TCF7L2_standard_ChIP_seq_CW34
chr22	39077701	39077702	MACS_peak_2847	6.00	TCF7L2_standard_ChIP_seq_CW34
chr22	40894714	40894715	MACS_peak_2848	9.00	TCF7L2_standard_ChIP_seq_CW34
chr22	42700094	42700095	MACS_peak_2849	22.00	TCF7L2_standard_ChIP_seq_CW34
chr22	42916795	42916796	MACS_peak_2850	13.00	TCF7L2_standard_ChIP_seq_CW34
chr22	43139658	43139659	MACS_peak_2851	13.00	TCF7L2_standard_ChIP_seq_CW34
chr22	43310005	43310006	MACS_peak_2852	31.00	TCF7L2_standard_ChIP_seq_CW34
chr22	43409988	43409989	MACS_peak_2853	11.00	TCF7L2_standard_ChIP_seq_CW34
chr22	43500285	43500286	MACS_peak_2854	13.00	TCF7L2_standard_ChIP_seq_CW34
chr22	43659420	43659421	MACS_peak_2855	20.00	TCF7L2_standard_ChIP_seq_CW34
chr22	43774480	43774481	MACS_peak_2856	8.00	TCF7L2_standard_ChIP_seq_CW34
chr22	44573407	44573408	MACS_peak_2857	7.00	TCF7L2_standard_ChIP_seq_CW34
chr22	45559587	45559588	MACS_peak_2858	11.00	TCF7L2_standard_ChIP_seq_CW34
chr22	45669542	45669543	MACS_peak_2859	6.00	TCF7L2_standard_ChIP_seq_CW34
chr22	45892702	45892703	MACS_peak_2860	12.00	TCF7L2_standard_ChIP_seq_CW34
chr22	45898406	45898407	MACS_peak_2861	12.00	TCF7L2_standard_ChIP_seq_CW34
chr22	45949919	45949920	MACS_peak_2862	11.00	TCF7L2_standard_ChIP_seq_CW34
chr22	46138924	46138925	MACS_peak_2863	26.00	TCF7L2_standard_ChIP_seq_CW34
chr22	46457438	46457439	MACS_peak_2864	10.00	TCF7L2_standard_ChIP_seq_CW34
chr22	46502048	46502049	MACS_peak_2865	7.00	TCF7L2_standard_ChIP_seq_CW34
chr22	46663665	46663666	MACS_peak_2866	10.00	TCF7L2_standard_ChIP_seq_CW34
chr22	46774072	46774073	MACS_peak_2867	24.00	TCF7L2_standard_ChIP_seq_CW34
chr22	47331811	47331812	MACS_peak_2868	9.00	TCF7L2_standard_ChIP_seq_CW34
chr22	49314619	49314620	MACS_peak_2869	8.00	TCF7L2_standard_ChIP_seq_CW34
chr22	49342000	49342001	MACS_peak_2870	7.00	TCF7L2_standard_ChIP_seq_CW34
chr22	50352957	50352958	MACS_peak_2871	17.00	TCF7L2_standard_ChIP_seq_CW34
chr22	51082260	51082261	MACS_peak_2872	9.00	TCF7L2_standard_ChIP_seq_CW34
chr3	306655	306656	MACS_peak_2873	8.00	TCF7L2_standard_ChIP_seq_CW34
chr3	1111182	1111183	MACS_peak_2874	5.00	TCF7L2_standard_ChIP_seq_CW34
chr3	5020328	5020329	MACS_peak_2875	9.00	TCF7L2_standard_ChIP_seq_CW34
chr3	5028652	5028653	MACS_peak_2876	46.00	TCF7L2_standard_ChIP_seq_CW34
chr3	5341016	5341017	MACS_peak_2877	7.00	TCF7L2_standard_ChIP_seq_CW34
chr3	8528759	8528760	MACS_peak_2878	14.00	TCF7L2_standard_ChIP_seq_CW34
chr3	9876805	9876806	MACS_peak_2879	12.00	TCF7L2_standard_ChIP_seq_CW34
chr3	9942766	9942767	MACS_peak_2880	24.00	TCF7L2_standard_ChIP_seq_CW34
chr3	9974867	9974868	MACS_peak_2881	6.00	TCF7L2_standard_ChIP_seq_CW34
chr3	10854051	10854052	MACS_peak_2882	11.00	TCF7L2_standard_ChIP_seq_CW34
chr3	11635243	11635244	MACS_peak_2883	16.00	TCF7L2_standard_ChIP_seq_CW34
chr3	11660635	11660636	MACS_peak_2884	36.00	TCF7L2_standard_ChIP_seq_CW34
chr3	11684371	11684372	MACS_peak_2885	5.00	TCF7L2_standard_ChIP_seq_CW34
chr3	12450704	12450705	MACS_peak_2886	9.00	TCF7L2_standard_ChIP_seq_CW34
chr3	13304280	13304281	MACS_peak_2887	8.00	TCF7L2_standard_ChIP_seq_CW34
chr3	14290975	14290976	MACS_peak_2888	9.00	TCF7L2_standard_ChIP_seq_CW34
chr3	14719858	14719859	MACS_peak_2889	18.00	TCF7L2_standard_ChIP_seq_CW34
chr3	15212843	15212844	MACS_peak_2890	33.00	TCF7L2_standard_ChIP_seq_CW34
chr3	15661553	15661554	MACS_peak_2891	9.00	TCF7L2_standard_ChIP_seq_CW34
chr3	19188749	19188750	MACS_peak_2892	9.00	TCF7L2_standard_ChIP_seq_CW34
chr3	24493384	24493385	MACS_peak_2893	12.00	TCF7L2_standard_ChIP_seq_CW34
chr3	24561354	24561355	MACS_peak_2894	11.00	TCF7L2_standard_ChIP_seq_CW34
chr3	24564800	24564801	MACS_peak_2895	12.00	TCF7L2_standard_ChIP_seq_CW34
chr3	25706254	25706255	MACS_peak_2896	9.00	TCF7L2_standard_ChIP_seq_CW34
chr3	27402416	27402417	MACS_peak_2897	13.00	TCF7L2_standard_ChIP_seq_CW34
chr3	27418047	27418048	MACS_peak_2898	14.00	TCF7L2_standard_ChIP_seq_CW34
chr3	27664803	27664804	MACS_peak_2899	8.00	TCF7L2_standard_ChIP_seq_CW34
chr3	29391707	29391708	MACS_peak_2900	7.00	TCF7L2_standard_ChIP_seq_CW34
chr3	30269521	30269522	MACS_peak_2901	8.00	TCF7L2_standard_ChIP_seq_CW34
chr3	30638930	30638931	MACS_peak_2902	17.00	TCF7L2_standard_ChIP_seq_CW34
chr3	31330304	31330305	MACS_peak_2903	17.00	TCF7L2_standard_ChIP_seq_CW34
chr3	31388268	31388269	MACS_peak_2904	8.00	TCF7L2_standard_ChIP_seq_CW34
chr3	31418750	31418751	MACS_peak_2905	88.00	TCF7L2_standard_ChIP_seq_CW34
chr3	32087026	32087027	MACS_peak_2906	9.00	TCF7L2_standard_ChIP_seq_CW34
chr3	32179581	32179582	MACS_peak_2907	11.00	TCF7L2_standard_ChIP_seq_CW34
chr3	32318167	32318168	MACS_peak_2908	6.00	TCF7L2_standard_ChIP_seq_CW34
chr3	32858082	32858083	MACS_peak_2909	10.00	TCF7L2_standard_ChIP_seq_CW34
chr3	32881798	32881799	MACS_peak_2910	17.00	TCF7L2_standard_ChIP_seq_CW34
chr3	33143238	33143239	MACS_peak_2911	9.00	TCF7L2_standard_ChIP_seq_CW34
chr3	33155805	33155806	MACS_peak_2912	10.00	TCF7L2_standard_ChIP_seq_CW34
chr3	33759230	33759231	MACS_peak_2913	11.00	TCF7L2_standard_ChIP_seq_CW34
chr3	33890754	33890755	MACS_peak_2914	7.00	TCF7L2_standard_ChIP_seq_CW34
chr3	37034300	37034301	MACS_peak_2915	6.00	TCF7L2_standard_ChIP_seq_CW34
chr3	37947587	37947588	MACS_peak_2916	10.00	TCF7L2_standard_ChIP_seq_CW34
chr3	38175351	38175352	MACS_peak_2917	8.00	TCF7L2_standard_ChIP_seq_CW34
chr3	40129129	40129130	MACS_peak_2918	8.00	TCF7L2_standard_ChIP_seq_CW34
chr3	40885012	40885013	MACS_peak_2919	11.00	TCF7L2_standard_ChIP_seq_CW34
chr3	40913147	40913148	MACS_peak_2920	8.00	TCF7L2_standard_ChIP_seq_CW34
chr3	41289192	41289193	MACS_peak_2921	7.00	TCF7L2_standard_ChIP_seq_CW34
chr3	41307564	41307565	MACS_peak_2922	9.00	TCF7L2_standard_ChIP_seq_CW34
chr3	41331980	41331981	MACS_peak_2923	7.00	TCF7L2_standard_ChIP_seq_CW34
chr3	41849657	41849658	MACS_peak_2924	6.00	TCF7L2_standard_ChIP_seq_CW34
chr3	42056660	42056661	MACS_peak_2925	9.00	TCF7L2_standard_ChIP_seq_CW34
chr3	42172006	42172007	MACS_peak_2926	38.00	TCF7L2_standard_ChIP_seq_CW34
chr3	42463974	42463975	MACS_peak_2927	17.00	TCF7L2_standard_ChIP_seq_CW34
chr3	42642032	42642033	MACS_peak_2928	9.00	TCF7L2_standard_ChIP_seq_CW34
chr3	42864490	42864491	MACS_peak_2929	9.00	TCF7L2_standard_ChIP_seq_CW34
chr3	42888258	42888259	MACS_peak_2930	7.00	TCF7L2_standard_ChIP_seq_CW34
chr3	42895360	42895361	MACS_peak_2931	19.00	TCF7L2_standard_ChIP_seq_CW34
chr3	44481171	44481172	MACS_peak_2932	7.00	TCF7L2_standard_ChIP_seq_CW34
chr3	44552729	44552730	MACS_peak_2933	6.00	TCF7L2_standard_ChIP_seq_CW34
chr3	45680685	45680686	MACS_peak_2934	8.00	TCF7L2_standard_ChIP_seq_CW34
chr3	46620641	46620642	MACS_peak_2935	22.00	TCF7L2_standard_ChIP_seq_CW34
chr3	47421261	47421262	MACS_peak_2936	9.00	TCF7L2_standard_ChIP_seq_CW34
chr3	47466387	47466388	MACS_peak_2937	8.00	TCF7L2_standard_ChIP_seq_CW34
chr3	47823032	47823033	MACS_peak_2938	20.00	TCF7L2_standard_ChIP_seq_CW34
chr3	48470850	48470851	MACS_peak_2939	14.00	TCF7L2_standard_ChIP_seq_CW34
chr3	49977520	49977521	MACS_peak_2940	14.00	TCF7L2_standard_ChIP_seq_CW34
chr3	50209252	50209253	MACS_peak_2941	119.00	TCF7L2_standard_ChIP_seq_CW34
chr3	50297894	50297895	MACS_peak_2942	53.00	TCF7L2_standard_ChIP_seq_CW34
chr3	50461125	50461126	MACS_peak_2943	10.00	TCF7L2_standard_ChIP_seq_CW34
chr3	50649431	50649432	MACS_peak_2944	12.00	TCF7L2_standard_ChIP_seq_CW34
chr3	51132451	51132452	MACS_peak_2945	9.00	TCF7L2_standard_ChIP_seq_CW34
chr3	51422369	51422370	MACS_peak_2946	7.00	TCF7L2_standard_ChIP_seq_CW34
chr3	52738671	52738672	MACS_peak_2947	23.00	TCF7L2_standard_ChIP_seq_CW34
chr3	53512631	53512632	MACS_peak_2948	12.00	TCF7L2_standard_ChIP_seq_CW34
chr3	54477906	54477907	MACS_peak_2949	17.00	TCF7L2_standard_ChIP_seq_CW34
chr3	56782943	56782944	MACS_peak_2950	7.00	TCF7L2_standard_ChIP_seq_CW34
chr3	56858111	56858112	MACS_peak_2951	6.00	TCF7L2_standard_ChIP_seq_CW34
chr3	56994007	56994008	MACS_peak_2952	7.00	TCF7L2_standard_ChIP_seq_CW34
chr3	57023917	57023918	MACS_peak_2953	18.00	TCF7L2_standard_ChIP_seq_CW34
chr3	57199043	57199044	MACS_peak_2954	10.00	TCF7L2_standard_ChIP_seq_CW34
chr3	57436768	57436769	MACS_peak_2955	14.00	TCF7L2_standard_ChIP_seq_CW34
chr3	57715122	57715123	MACS_peak_2956	6.00	TCF7L2_standard_ChIP_seq_CW34
chr3	57813100	57813101	MACS_peak_2957	9.00	TCF7L2_standard_ChIP_seq_CW34
chr3	58161374	58161375	MACS_peak_2958	11.00	TCF7L2_standard_ChIP_seq_CW34
chr3	58179238	58179239	MACS_peak_2959	23.00	TCF7L2_standard_ChIP_seq_CW34
chr3	60827237	60827238	MACS_peak_2960	11.00	TCF7L2_standard_ChIP_seq_CW34
chr3	61547562	61547563	MACS_peak_2961	6.00	TCF7L2_standard_ChIP_seq_CW34
chr3	62757185	62757186	MACS_peak_2962	6.00	TCF7L2_standard_ChIP_seq_CW34
chr3	64234160	64234161	MACS_peak_2963	7.00	TCF7L2_standard_ChIP_seq_CW34
chr3	64491334	64491335	MACS_peak_2964	8.00	TCF7L2_standard_ChIP_seq_CW34
chr3	64519428	64519429	MACS_peak_2965	9.00	TCF7L2_standard_ChIP_seq_CW34
chr3	64613708	64613709	MACS_peak_2966	15.00	TCF7L2_standard_ChIP_seq_CW34
chr3	66531616	66531617	MACS_peak_2967	38.00	TCF7L2_standard_ChIP_seq_CW34
chr3	68995607	68995608	MACS_peak_2968	8.00	TCF7L2_standard_ChIP_seq_CW34
chr3	69788664	69788665	MACS_peak_2969	6.00	TCF7L2_standard_ChIP_seq_CW34
chr3	70501026	70501027	MACS_peak_2970	5.00	TCF7L2_standard_ChIP_seq_CW34
chr3	71109809	71109810	MACS_peak_2971	24.00	TCF7L2_standard_ChIP_seq_CW34
chr3	71257656	71257657	MACS_peak_2972	20.00	TCF7L2_standard_ChIP_seq_CW34
chr3	71465099	71465100	MACS_peak_2973	18.00	TCF7L2_standard_ChIP_seq_CW34
chr3	71699170	71699171	MACS_peak_2974	6.00	TCF7L2_standard_ChIP_seq_CW34
chr3	73002798	73002799	MACS_peak_2975	22.00	TCF7L2_standard_ChIP_seq_CW34
chr3	73016941	73016942	MACS_peak_2976	9.00	TCF7L2_standard_ChIP_seq_CW34
chr3	73046431	73046432	MACS_peak_2977	8.00	TCF7L2_standard_ChIP_seq_CW34
chr3	73101068	73101069	MACS_peak_2978	21.00	TCF7L2_standard_ChIP_seq_CW34
chr3	73620696	73620697	MACS_peak_2979	8.00	TCF7L2_standard_ChIP_seq_CW34
chr3	75736805	75736806	MACS_peak_2981	11.00	TCF7L2_standard_ChIP_seq_CW34
chr3	76735244	76735245	MACS_peak_2982	22.00	TCF7L2_standard_ChIP_seq_CW34
chr3	78556705	78556706	MACS_peak_2983	16.00	TCF7L2_standard_ChIP_seq_CW34
chr3	78893643	78893644	MACS_peak_2984	14.00	TCF7L2_standard_ChIP_seq_CW34
chr3	78899006	78899007	MACS_peak_2985	10.00	TCF7L2_standard_ChIP_seq_CW34
chr3	78921639	78921640	MACS_peak_2986	8.00	TCF7L2_standard_ChIP_seq_CW34
chr3	79021434	79021435	MACS_peak_2987	34.00	TCF7L2_standard_ChIP_seq_CW34
chr3	80667653	80667654	MACS_peak_2988	10.00	TCF7L2_standard_ChIP_seq_CW34
chr3	81349684	81349685	MACS_peak_2989	85.00	TCF7L2_standard_ChIP_seq_CW34
chr3	82510770	82510771	MACS_peak_2990	8.00	TCF7L2_standard_ChIP_seq_CW34
chr3	82682624	82682625	MACS_peak_2991	8.00	TCF7L2_standard_ChIP_seq_CW34
chr3	85775669	85775670	MACS_peak_2992	7.00	TCF7L2_standard_ChIP_seq_CW34
chr3	88065071	88065072	MACS_peak_2993	7.00	TCF7L2_standard_ChIP_seq_CW34
chr3	97540921	97540922	MACS_peak_2994	5.00	TCF7L2_standard_ChIP_seq_CW34
chr3	98451827	98451828	MACS_peak_2995	7.00	TCF7L2_standard_ChIP_seq_CW34
chr3	98505450	98505451	MACS_peak_2996	8.00	TCF7L2_standard_ChIP_seq_CW34
chr3	99650111	99650112	MACS_peak_2997	9.00	TCF7L2_standard_ChIP_seq_CW34
chr3	99795134	99795135	MACS_peak_2998	23.00	TCF7L2_standard_ChIP_seq_CW34
chr3	100417443	100417444	MACS_peak_2999	6.00	TCF7L2_standard_ChIP_seq_CW34
chr3	101292965	101292966	MACS_peak_3000	15.00	TCF7L2_standard_ChIP_seq_CW34
chr3	105106168	105106169	MACS_peak_3001	17.00	TCF7L2_standard_ChIP_seq_CW34
chr3	105222126	105222127	MACS_peak_3002	12.00	TCF7L2_standard_ChIP_seq_CW34
chr3	107403723	107403724	MACS_peak_3003	10.00	TCF7L2_standard_ChIP_seq_CW34
chr3	110492462	110492463	MACS_peak_3004	8.00	TCF7L2_standard_ChIP_seq_CW34
chr3	110657763	110657764	MACS_peak_3005	9.00	TCF7L2_standard_ChIP_seq_CW34
chr3	111578361	111578362	MACS_peak_3006	10.00	TCF7L2_standard_ChIP_seq_CW34
chr3	113464796	113464797	MACS_peak_3007	10.00	TCF7L2_standard_ChIP_seq_CW34
chr3	113541145	113541146	MACS_peak_3008	7.00	TCF7L2_standard_ChIP_seq_CW34
chr3	115377367	115377368	MACS_peak_3009	6.00	TCF7L2_standard_ChIP_seq_CW34
chr3	115425568	115425569	MACS_peak_3010	7.00	TCF7L2_standard_ChIP_seq_CW34
chr3	115472117	115472118	MACS_peak_3011	9.00	TCF7L2_standard_ChIP_seq_CW34
chr3	118259510	118259511	MACS_peak_3012	9.00	TCF7L2_standard_ChIP_seq_CW34
chr3	119012844	119012845	MACS_peak_3013	11.00	TCF7L2_standard_ChIP_seq_CW34
chr3	119182585	119182586	MACS_peak_3014	5.00	TCF7L2_standard_ChIP_seq_CW34
chr3	119514184	119514185	MACS_peak_3015	8.00	TCF7L2_standard_ChIP_seq_CW34
chr3	120247243	120247244	MACS_peak_3016	5.00	TCF7L2_standard_ChIP_seq_CW34
chr3	122111106	122111107	MACS_peak_3017	7.00	TCF7L2_standard_ChIP_seq_CW34
chr3	122370632	122370633	MACS_peak_3018	17.00	TCF7L2_standard_ChIP_seq_CW34
chr3	123303846	123303847	MACS_peak_3019	7.00	TCF7L2_standard_ChIP_seq_CW34
chr3	123324762	123324763	MACS_peak_3020	13.00	TCF7L2_standard_ChIP_seq_CW34
chr3	123538076	123538077	MACS_peak_3021	9.00	TCF7L2_standard_ChIP_seq_CW34
chr3	124409383	124409384	MACS_peak_3022	29.00	TCF7L2_standard_ChIP_seq_CW34
chr3	124553771	124553772	MACS_peak_3023	29.00	TCF7L2_standard_ChIP_seq_CW34
chr3	124605496	124605497	MACS_peak_3024	6.00	TCF7L2_standard_ChIP_seq_CW34
chr3	124782053	124782054	MACS_peak_3025	15.00	TCF7L2_standard_ChIP_seq_CW34
chr3	125315848	125315849	MACS_peak_3026	19.00	TCF7L2_standard_ChIP_seq_CW34
chr3	125785672	125785673	MACS_peak_3027	21.00	TCF7L2_standard_ChIP_seq_CW34
chr3	125979994	125979995	MACS_peak_3028	34.00	TCF7L2_standard_ChIP_seq_CW34
chr3	126701725	126701726	MACS_peak_3029	10.00	TCF7L2_standard_ChIP_seq_CW34
chr3	126832557	126832558	MACS_peak_3030	9.00	TCF7L2_standard_ChIP_seq_CW34
chr3	127729235	127729236	MACS_peak_3031	11.00	TCF7L2_standard_ChIP_seq_CW34
chr3	129107835	129107836	MACS_peak_3032	7.00	TCF7L2_standard_ChIP_seq_CW34
chr3	129312937	129312938	MACS_peak_3033	7.00	TCF7L2_standard_ChIP_seq_CW34
chr3	129375684	129375685	MACS_peak_3034	10.00	TCF7L2_standard_ChIP_seq_CW34
chr3	129690418	129690419	MACS_peak_3035	18.00	TCF7L2_standard_ChIP_seq_CW34
chr3	130421948	130421949	MACS_peak_3036	5.00	TCF7L2_standard_ChIP_seq_CW34
chr3	133452311	133452312	MACS_peak_3037	9.00	TCF7L2_standard_ChIP_seq_CW34
chr3	134093363	134093364	MACS_peak_3038	52.00	TCF7L2_standard_ChIP_seq_CW34
chr3	134204984	134204985	MACS_peak_3039	9.00	TCF7L2_standard_ChIP_seq_CW34
chr3	135222915	135222916	MACS_peak_3040	6.00	TCF7L2_standard_ChIP_seq_CW34
chr3	138281928	138281929	MACS_peak_3041	19.00	TCF7L2_standard_ChIP_seq_CW34
chr3	140418914	140418915	MACS_peak_3042	8.00	TCF7L2_standard_ChIP_seq_CW34
chr3	140430323	140430324	MACS_peak_3043	30.00	TCF7L2_standard_ChIP_seq_CW34
chr3	140676188	140676189	MACS_peak_3044	9.00	TCF7L2_standard_ChIP_seq_CW34
chr3	141051483	141051484	MACS_peak_3045	17.00	TCF7L2_standard_ChIP_seq_CW34
chr3	141087240	141087241	MACS_peak_3046	20.00	TCF7L2_standard_ChIP_seq_CW34
chr3	141150407	141150408	MACS_peak_3047	12.00	TCF7L2_standard_ChIP_seq_CW34
chr3	141201370	141201371	MACS_peak_3048	11.00	TCF7L2_standard_ChIP_seq_CW34
chr3	141668951	141668952	MACS_peak_3049	10.00	TCF7L2_standard_ChIP_seq_CW34
chr3	141830460	141830461	MACS_peak_3050	7.00	TCF7L2_standard_ChIP_seq_CW34
chr3	142297649	142297650	MACS_peak_3051	9.00	TCF7L2_standard_ChIP_seq_CW34
chr3	146344672	146344673	MACS_peak_3052	11.00	TCF7L2_standard_ChIP_seq_CW34
chr3	146355828	146355829	MACS_peak_3053	16.00	TCF7L2_standard_ChIP_seq_CW34
chr3	148318998	148318999	MACS_peak_3054	6.00	TCF7L2_standard_ChIP_seq_CW34
chr3	148755819	148755820	MACS_peak_3055	7.00	TCF7L2_standard_ChIP_seq_CW34
chr3	148954031	148954032	MACS_peak_3056	19.00	TCF7L2_standard_ChIP_seq_CW34
chr3	149087166	149087167	MACS_peak_3057	9.00	TCF7L2_standard_ChIP_seq_CW34
chr3	149104866	149104867	MACS_peak_3058	21.00	TCF7L2_standard_ChIP_seq_CW34
chr3	149132749	149132750	MACS_peak_3059	6.00	TCF7L2_standard_ChIP_seq_CW34
chr3	149359679	149359680	MACS_peak_3060	13.00	TCF7L2_standard_ChIP_seq_CW34
chr3	150455256	150455257	MACS_peak_3061	31.00	TCF7L2_standard_ChIP_seq_CW34
chr3	150916354	150916355	MACS_peak_3062	37.00	TCF7L2_standard_ChIP_seq_CW34
chr3	151002020	151002021	MACS_peak_3063	8.00	TCF7L2_standard_ChIP_seq_CW34
chr3	151356804	151356805	MACS_peak_3064	6.00	TCF7L2_standard_ChIP_seq_CW34
chr3	151534082	151534083	MACS_peak_3065	7.00	TCF7L2_standard_ChIP_seq_CW34
chr3	151535439	151535440	MACS_peak_3066	8.00	TCF7L2_standard_ChIP_seq_CW34
chr3	153068348	153068349	MACS_peak_3067	10.00	TCF7L2_standard_ChIP_seq_CW34
chr3	156848069	156848070	MACS_peak_3068	7.00	TCF7L2_standard_ChIP_seq_CW34
chr3	157815890	157815891	MACS_peak_3069	5.00	TCF7L2_standard_ChIP_seq_CW34
chr3	158169338	158169339	MACS_peak_3070	11.00	TCF7L2_standard_ChIP_seq_CW34
chr3	158487236	158487237	MACS_peak_3071	5.00	TCF7L2_standard_ChIP_seq_CW34
chr3	161138723	161138724	MACS_peak_3072	6.00	TCF7L2_standard_ChIP_seq_CW34
chr3	165853918	165853919	MACS_peak_3073	7.00	TCF7L2_standard_ChIP_seq_CW34
chr3	166547835	166547836	MACS_peak_3074	21.00	TCF7L2_standard_ChIP_seq_CW34
chr3	169939913	169939914	MACS_peak_3075	6.00	TCF7L2_standard_ChIP_seq_CW34
chr3	170690890	170690891	MACS_peak_3076	39.00	TCF7L2_standard_ChIP_seq_CW34
chr3	170719257	170719258	MACS_peak_3077	18.00	TCF7L2_standard_ChIP_seq_CW34
chr3	170731776	170731777	MACS_peak_3078	12.00	TCF7L2_standard_ChIP_seq_CW34
chr3	170763405	170763406	MACS_peak_3079	6.00	TCF7L2_standard_ChIP_seq_CW34
chr3	171527666	171527667	MACS_peak_3080	9.00	TCF7L2_standard_ChIP_seq_CW34
chr3	171559140	171559141	MACS_peak_3081	16.00	TCF7L2_standard_ChIP_seq_CW34
chr3	171601188	171601189	MACS_peak_3082	19.00	TCF7L2_standard_ChIP_seq_CW34
chr3	171619976	171619977	MACS_peak_3083	6.00	TCF7L2_standard_ChIP_seq_CW34
chr3	171709134	171709135	MACS_peak_3084	13.00	TCF7L2_standard_ChIP_seq_CW34
chr3	171724196	171724197	MACS_peak_3085	14.00	TCF7L2_standard_ChIP_seq_CW34
chr3	171801930	171801931	MACS_peak_3086	26.00	TCF7L2_standard_ChIP_seq_CW34
chr3	171819028	171819029	MACS_peak_3087	10.00	TCF7L2_standard_ChIP_seq_CW34
chr3	171848337	171848338	MACS_peak_3088	9.00	TCF7L2_standard_ChIP_seq_CW34
chr3	174097305	174097306	MACS_peak_3089	6.00	TCF7L2_standard_ChIP_seq_CW34
chr3	174159118	174159119	MACS_peak_3090	6.00	TCF7L2_standard_ChIP_seq_CW34
chr3	177534785	177534786	MACS_peak_3091	7.00	TCF7L2_standard_ChIP_seq_CW34
chr3	179040717	179040718	MACS_peak_3092	8.00	TCF7L2_standard_ChIP_seq_CW34
chr3	180772383	180772384	MACS_peak_3093	8.00	TCF7L2_standard_ChIP_seq_CW34
chr3	181446553	181446554	MACS_peak_3094	10.00	TCF7L2_standard_ChIP_seq_CW34
chr3	181930678	181930679	MACS_peak_3095	9.00	TCF7L2_standard_ChIP_seq_CW34
chr3	182880362	182880363	MACS_peak_3096	24.00	TCF7L2_standard_ChIP_seq_CW34
chr3	183009646	183009647	MACS_peak_3097	7.00	TCF7L2_standard_ChIP_seq_CW34
chr3	183180811	183180812	MACS_peak_3098	5.00	TCF7L2_standard_ChIP_seq_CW34
chr3	183353688	183353689	MACS_peak_3099	8.00	TCF7L2_standard_ChIP_seq_CW34
chr3	183706184	183706185	MACS_peak_3100	14.00	TCF7L2_standard_ChIP_seq_CW34
chr3	183873500	183873501	MACS_peak_3101	6.00	TCF7L2_standard_ChIP_seq_CW34
chr3	184133111	184133112	MACS_peak_3102	8.00	TCF7L2_standard_ChIP_seq_CW34
chr3	185684597	185684598	MACS_peak_3103	19.00	TCF7L2_standard_ChIP_seq_CW34
chr3	185691127	185691128	MACS_peak_3104	8.00	TCF7L2_standard_ChIP_seq_CW34
chr3	186072161	186072162	MACS_peak_3105	9.00	TCF7L2_standard_ChIP_seq_CW34
chr3	186330736	186330737	MACS_peak_3106	37.00	TCF7L2_standard_ChIP_seq_CW34
chr3	186625052	186625053	MACS_peak_3107	42.00	TCF7L2_standard_ChIP_seq_CW34
chr3	187437568	187437569	MACS_peak_3108	16.00	TCF7L2_standard_ChIP_seq_CW34
chr3	187754384	187754385	MACS_peak_3109	11.00	TCF7L2_standard_ChIP_seq_CW34
chr3	187841077	187841078	MACS_peak_3110	8.00	TCF7L2_standard_ChIP_seq_CW34
chr3	187889005	187889006	MACS_peak_3111	8.00	TCF7L2_standard_ChIP_seq_CW34
chr3	187982542	187982543	MACS_peak_3112	5.00	TCF7L2_standard_ChIP_seq_CW34
chr3	188464199	188464200	MACS_peak_3113	26.00	TCF7L2_standard_ChIP_seq_CW34
chr3	190025217	190025218	MACS_peak_3114	12.00	TCF7L2_standard_ChIP_seq_CW34
chr3	191874378	191874379	MACS_peak_3115	14.00	TCF7L2_standard_ChIP_seq_CW34
chr3	193430166	193430167	MACS_peak_3116	12.00	TCF7L2_standard_ChIP_seq_CW34
chr3	193852307	193852308	MACS_peak_3117	9.00	TCF7L2_standard_ChIP_seq_CW34
chr3	194168106	194168107	MACS_peak_3118	9.00	TCF7L2_standard_ChIP_seq_CW34
chr3	194716230	194716231	MACS_peak_3119	18.00	TCF7L2_standard_ChIP_seq_CW34
chr3	194886691	194886692	MACS_peak_3120	10.00	TCF7L2_standard_ChIP_seq_CW34
chr3	194980663	194980664	MACS_peak_3121	21.00	TCF7L2_standard_ChIP_seq_CW34
chr3	195277806	195277807	MACS_peak_3122	9.00	TCF7L2_standard_ChIP_seq_CW34
chr3	195717085	195717086	MACS_peak_3123	25.00	TCF7L2_standard_ChIP_seq_CW34
chr3	196074378	196074379	MACS_peak_3124	11.00	TCF7L2_standard_ChIP_seq_CW34
chr3	196439206	196439207	MACS_peak_3125	7.00	TCF7L2_standard_ChIP_seq_CW34
chr3	197043019	197043020	MACS_peak_3127	10.00	TCF7L2_standard_ChIP_seq_CW34
chr3	197091710	197091711	MACS_peak_3128	8.00	TCF7L2_standard_ChIP_seq_CW34
chr3	197317610	197317611	MACS_peak_3129	5.00	TCF7L2_standard_ChIP_seq_CW34
chr3	197547736	197547737	MACS_peak_3130	6.00	TCF7L2_standard_ChIP_seq_CW34
chr4	322788	322789	MACS_peak_3131	7.00	TCF7L2_standard_ChIP_seq_CW34
chr4	1714175	1714176	MACS_peak_3132	9.00	TCF7L2_standard_ChIP_seq_CW34
chr4	1768509	1768510	MACS_peak_3133	8.00	TCF7L2_standard_ChIP_seq_CW34
chr4	1787189	1787190	MACS_peak_3134	9.00	TCF7L2_standard_ChIP_seq_CW34
chr4	2001222	2001223	MACS_peak_3135	6.00	TCF7L2_standard_ChIP_seq_CW34
chr4	2010919	2010920	MACS_peak_3136	8.00	TCF7L2_standard_ChIP_seq_CW34
chr4	2431878	2431879	MACS_peak_3137	10.00	TCF7L2_standard_ChIP_seq_CW34
chr4	3209431	3209432	MACS_peak_3138	6.00	TCF7L2_standard_ChIP_seq_CW34
chr4	3673772	3673773	MACS_peak_3139	7.00	TCF7L2_standard_ChIP_seq_CW34
chr4	3805679	3805680	MACS_peak_3140	6.00	TCF7L2_standard_ChIP_seq_CW34
chr4	3838795	3838796	MACS_peak_3141	120.00	TCF7L2_standard_ChIP_seq_CW34
chr4	4261350	4261351	MACS_peak_3142	17.00	TCF7L2_standard_ChIP_seq_CW34
chr4	4474323	4474324	MACS_peak_3143	10.00	TCF7L2_standard_ChIP_seq_CW34
chr4	5014289	5014290	MACS_peak_3144	8.00	TCF7L2_standard_ChIP_seq_CW34
chr4	6339503	6339504	MACS_peak_3145	12.00	TCF7L2_standard_ChIP_seq_CW34
chr4	6976328	6976329	MACS_peak_3146	10.00	TCF7L2_standard_ChIP_seq_CW34
chr4	7454301	7454302	MACS_peak_3147	7.00	TCF7L2_standard_ChIP_seq_CW34
chr4	7640947	7640948	MACS_peak_3148	25.00	TCF7L2_standard_ChIP_seq_CW34
chr4	7751430	7751431	MACS_peak_3149	11.00	TCF7L2_standard_ChIP_seq_CW34
chr4	7767613	7767614	MACS_peak_3150	16.00	TCF7L2_standard_ChIP_seq_CW34
chr4	9240421	9240422	MACS_peak_3151	13.00	TCF7L2_standard_ChIP_seq_CW34
chr4	10019022	10019023	MACS_peak_3152	11.00	TCF7L2_standard_ChIP_seq_CW34
chr4	12463448	12463449	MACS_peak_3153	22.00	TCF7L2_standard_ChIP_seq_CW34
chr4	12949953	12949954	MACS_peak_3154	6.00	TCF7L2_standard_ChIP_seq_CW34
chr4	13598962	13598963	MACS_peak_3155	9.00	TCF7L2_standard_ChIP_seq_CW34
chr4	15151774	15151775	MACS_peak_3156	6.00	TCF7L2_standard_ChIP_seq_CW34
chr4	17558353	17558354	MACS_peak_3157	14.00	TCF7L2_standard_ChIP_seq_CW34
chr4	22336081	22336082	MACS_peak_3158	8.00	TCF7L2_standard_ChIP_seq_CW34
chr4	22458179	22458180	MACS_peak_3159	8.00	TCF7L2_standard_ChIP_seq_CW34
chr4	26466236	26466237	MACS_peak_3160	8.00	TCF7L2_standard_ChIP_seq_CW34
chr4	37953956	37953957	MACS_peak_3161	6.00	TCF7L2_standard_ChIP_seq_CW34
chr4	38039466	38039467	MACS_peak_3162	6.00	TCF7L2_standard_ChIP_seq_CW34
chr4	38079668	38079669	MACS_peak_3163	15.00	TCF7L2_standard_ChIP_seq_CW34
chr4	38134978	38134979	MACS_peak_3164	30.00	TCF7L2_standard_ChIP_seq_CW34
chr4	38162666	38162667	MACS_peak_3165	9.00	TCF7L2_standard_ChIP_seq_CW34
chr4	38426407	38426408	MACS_peak_3166	9.00	TCF7L2_standard_ChIP_seq_CW34
chr4	39560095	39560096	MACS_peak_3167	12.00	TCF7L2_standard_ChIP_seq_CW34
chr4	40059480	40059481	MACS_peak_3168	8.00	TCF7L2_standard_ChIP_seq_CW34
chr4	41079129	41079130	MACS_peak_3169	7.00	TCF7L2_standard_ChIP_seq_CW34
chr4	41109811	41109812	MACS_peak_3170	5.00	TCF7L2_standard_ChIP_seq_CW34
chr4	41166512	41166513	MACS_peak_3171	11.00	TCF7L2_standard_ChIP_seq_CW34
chr4	41937247	41937248	MACS_peak_3172	12.00	TCF7L2_standard_ChIP_seq_CW34
chr4	42304640	42304641	MACS_peak_3173	12.00	TCF7L2_standard_ChIP_seq_CW34
chr4	43362474	43362475	MACS_peak_3174	8.00	TCF7L2_standard_ChIP_seq_CW34
chr4	47421488	47421489	MACS_peak_3175	9.00	TCF7L2_standard_ChIP_seq_CW34
chr4	47789079	47789080	MACS_peak_3176	12.00	TCF7L2_standard_ChIP_seq_CW34
chr4	52886539	52886540	MACS_peak_3192	7.00	TCF7L2_standard_ChIP_seq_CW34
chr4	53236001	53236002	MACS_peak_3193	11.00	TCF7L2_standard_ChIP_seq_CW34
chr4	53801664	53801665	MACS_peak_3194	6.00	TCF7L2_standard_ChIP_seq_CW34
chr4	53886214	53886215	MACS_peak_3195	11.00	TCF7L2_standard_ChIP_seq_CW34
chr4	53988615	53988616	MACS_peak_3196	8.00	TCF7L2_standard_ChIP_seq_CW34
chr4	54054574	54054575	MACS_peak_3197	25.00	TCF7L2_standard_ChIP_seq_CW34
chr4	54243883	54243884	MACS_peak_3198	8.00	TCF7L2_standard_ChIP_seq_CW34
chr4	54448492	54448493	MACS_peak_3199	6.00	TCF7L2_standard_ChIP_seq_CW34
chr4	56212693	56212694	MACS_peak_3200	37.00	TCF7L2_standard_ChIP_seq_CW34
chr4	56262577	56262578	MACS_peak_3201	19.00	TCF7L2_standard_ChIP_seq_CW34
chr4	56839132	56839133	MACS_peak_3202	6.00	TCF7L2_standard_ChIP_seq_CW34
chr4	59800878	59800879	MACS_peak_3203	6.00	TCF7L2_standard_ChIP_seq_CW34
chr4	66864743	66864744	MACS_peak_3204	8.00	TCF7L2_standard_ChIP_seq_CW34
chr4	69215586	69215587	MACS_peak_3206	7.00	TCF7L2_standard_ChIP_seq_CW34
chr4	69522327	69522328	MACS_peak_3207	5.00	TCF7L2_standard_ChIP_seq_CW34
chr4	69598755	69598756	MACS_peak_3208	8.00	TCF7L2_standard_ChIP_seq_CW34
chr4	69637759	69637760	MACS_peak_3209	16.00	TCF7L2_standard_ChIP_seq_CW34
chr4	69681559	69681560	MACS_peak_3210	8.00	TCF7L2_standard_ChIP_seq_CW34
chr4	69736638	69736639	MACS_peak_3211	21.00	TCF7L2_standard_ChIP_seq_CW34
chr4	69886275	69886276	MACS_peak_3212	7.00	TCF7L2_standard_ChIP_seq_CW34
chr4	69968595	69968596	MACS_peak_3213	7.00	TCF7L2_standard_ChIP_seq_CW34
chr4	70647699	70647700	MACS_peak_3214	6.00	TCF7L2_standard_ChIP_seq_CW34
chr4	70698584	70698585	MACS_peak_3215	7.00	TCF7L2_standard_ChIP_seq_CW34
chr4	71048379	71048380	MACS_peak_3216	27.00	TCF7L2_standard_ChIP_seq_CW34
chr4	73412270	73412271	MACS_peak_3217	6.00	TCF7L2_standard_ChIP_seq_CW34
chr4	74871376	74871377	MACS_peak_3218	18.00	TCF7L2_standard_ChIP_seq_CW34
chr4	74965604	74965605	MACS_peak_3219	8.00	TCF7L2_standard_ChIP_seq_CW34
chr4	75067996	75067997	MACS_peak_3220	25.00	TCF7L2_standard_ChIP_seq_CW34
chr4	75344971	75344972	MACS_peak_3221	14.00	TCF7L2_standard_ChIP_seq_CW34
chr4	75579537	75579538	MACS_peak_3222	8.00	TCF7L2_standard_ChIP_seq_CW34
chr4	77777541	77777542	MACS_peak_3223	6.00	TCF7L2_standard_ChIP_seq_CW34
chr4	78936231	78936232	MACS_peak_3224	24.00	TCF7L2_standard_ChIP_seq_CW34
chr4	79583303	79583304	MACS_peak_3225	9.00	TCF7L2_standard_ChIP_seq_CW34
chr4	83453161	83453162	MACS_peak_3226	59.00	TCF7L2_standard_ChIP_seq_CW34
chr4	84772625	84772626	MACS_peak_3227	10.00	TCF7L2_standard_ChIP_seq_CW34
chr4	86396379	86396380	MACS_peak_3228	9.00	TCF7L2_standard_ChIP_seq_CW34
chr4	87915632	87915633	MACS_peak_3229	8.00	TCF7L2_standard_ChIP_seq_CW34
chr4	87928190	87928191	MACS_peak_3230	9.00	TCF7L2_standard_ChIP_seq_CW34
chr4	88199640	88199641	MACS_peak_3231	6.00	TCF7L2_standard_ChIP_seq_CW34
chr4	88305911	88305912	MACS_peak_3232	10.00	TCF7L2_standard_ChIP_seq_CW34
chr4	88312691	88312692	MACS_peak_3233	16.00	TCF7L2_standard_ChIP_seq_CW34
chr4	88586696	88586697	MACS_peak_3234	7.00	TCF7L2_standard_ChIP_seq_CW34
chr4	88787598	88787599	MACS_peak_3235	14.00	TCF7L2_standard_ChIP_seq_CW34
chr4	95136286	95136287	MACS_peak_3236	8.00	TCF7L2_standard_ChIP_seq_CW34
chr4	96141888	96141889	MACS_peak_3237	12.00	TCF7L2_standard_ChIP_seq_CW34
chr4	99181641	99181642	MACS_peak_3238	14.00	TCF7L2_standard_ChIP_seq_CW34
chr4	99850716	99850717	MACS_peak_3239	9.00	TCF7L2_standard_ChIP_seq_CW34
chr4	100078835	100078836	MACS_peak_3240	77.00	TCF7L2_standard_ChIP_seq_CW34
chr4	103363514	103363515	MACS_peak_3241	12.00	TCF7L2_standard_ChIP_seq_CW34
chr4	103435751	103435752	MACS_peak_3242	5.00	TCF7L2_standard_ChIP_seq_CW34
chr4	104308918	104308919	MACS_peak_3243	17.00	TCF7L2_standard_ChIP_seq_CW34
chr4	106266678	106266679	MACS_peak_3244	23.00	TCF7L2_standard_ChIP_seq_CW34
chr4	106958207	106958208	MACS_peak_3245	22.00	TCF7L2_standard_ChIP_seq_CW34
chr4	107179669	107179670	MACS_peak_3246	33.00	TCF7L2_standard_ChIP_seq_CW34
chr4	108136573	108136574	MACS_peak_3247	19.00	TCF7L2_standard_ChIP_seq_CW34
chr4	109094494	109094495	MACS_peak_3248	20.00	TCF7L2_standard_ChIP_seq_CW34
chr4	113129684	113129685	MACS_peak_3249	17.00	TCF7L2_standard_ChIP_seq_CW34
chr4	119253973	119253974	MACS_peak_3250	5.00	TCF7L2_standard_ChIP_seq_CW34
chr4	120002323	120002324	MACS_peak_3251	7.00	TCF7L2_standard_ChIP_seq_CW34
chr4	120221741	120221742	MACS_peak_3252	10.00	TCF7L2_standard_ChIP_seq_CW34
chr4	120292632	120292633	MACS_peak_3253	11.00	TCF7L2_standard_ChIP_seq_CW34
chr4	121993460	121993461	MACS_peak_3254	11.00	TCF7L2_standard_ChIP_seq_CW34
chr4	123897431	123897432	MACS_peak_3255	11.00	TCF7L2_standard_ChIP_seq_CW34
chr4	124270715	124270716	MACS_peak_3256	7.00	TCF7L2_standard_ChIP_seq_CW34
chr4	124400100	124400101	MACS_peak_3257	44.00	TCF7L2_standard_ChIP_seq_CW34
chr4	124426963	124426964	MACS_peak_3258	9.00	TCF7L2_standard_ChIP_seq_CW34
chr4	124476749	124476750	MACS_peak_3259	7.00	TCF7L2_standard_ChIP_seq_CW34
chr4	124778569	124778570	MACS_peak_3260	11.00	TCF7L2_standard_ChIP_seq_CW34
chr4	125451934	125451935	MACS_peak_3261	22.00	TCF7L2_standard_ChIP_seq_CW34
chr4	129271194	129271195	MACS_peak_3262	19.00	TCF7L2_standard_ChIP_seq_CW34
chr4	129350573	129350574	MACS_peak_3263	12.00	TCF7L2_standard_ChIP_seq_CW34
chr4	129783270	129783271	MACS_peak_3264	8.00	TCF7L2_standard_ChIP_seq_CW34
chr4	134918145	134918146	MACS_peak_3265	9.00	TCF7L2_standard_ChIP_seq_CW34
chr4	139289277	139289278	MACS_peak_3266	11.00	TCF7L2_standard_ChIP_seq_CW34
chr4	140581842	140581843	MACS_peak_3267	8.00	TCF7L2_standard_ChIP_seq_CW34
chr4	140660530	140660531	MACS_peak_3268	16.00	TCF7L2_standard_ChIP_seq_CW34
chr4	140682503	140682504	MACS_peak_3269	8.00	TCF7L2_standard_ChIP_seq_CW34
chr4	141536799	141536800	MACS_peak_3270	9.00	TCF7L2_standard_ChIP_seq_CW34
chr4	141562420	141562421	MACS_peak_3271	14.00	TCF7L2_standard_ChIP_seq_CW34
chr4	142477101	142477102	MACS_peak_3272	6.00	TCF7L2_standard_ChIP_seq_CW34
chr4	146403179	146403180	MACS_peak_3273	7.00	TCF7L2_standard_ChIP_seq_CW34
chr4	146860017	146860018	MACS_peak_3274	43.00	TCF7L2_standard_ChIP_seq_CW34
chr4	148079844	148079845	MACS_peak_3275	5.00	TCF7L2_standard_ChIP_seq_CW34
chr4	149297601	149297602	MACS_peak_3276	8.00	TCF7L2_standard_ChIP_seq_CW34
chr4	151002230	151002231	MACS_peak_3277	7.00	TCF7L2_standard_ChIP_seq_CW34
chr4	152188366	152188367	MACS_peak_3278	7.00	TCF7L2_standard_ChIP_seq_CW34
chr4	152245738	152245739	MACS_peak_3279	14.00	TCF7L2_standard_ChIP_seq_CW34
chr4	153407458	153407459	MACS_peak_3280	6.00	TCF7L2_standard_ChIP_seq_CW34
chr4	154130779	154130780	MACS_peak_3281	9.00	TCF7L2_standard_ChIP_seq_CW34
chr4	154463509	154463510	MACS_peak_3282	5.00	TCF7L2_standard_ChIP_seq_CW34
chr4	155519345	155519346	MACS_peak_3283	12.00	TCF7L2_standard_ChIP_seq_CW34
chr4	155539030	155539031	MACS_peak_3284	26.00	TCF7L2_standard_ChIP_seq_CW34
chr4	157863300	157863301	MACS_peak_3285	13.00	TCF7L2_standard_ChIP_seq_CW34
chr4	158916160	158916161	MACS_peak_3286	46.00	TCF7L2_standard_ChIP_seq_CW34
chr4	159164440	159164441	MACS_peak_3287	7.00	TCF7L2_standard_ChIP_seq_CW34
chr4	159644599	159644600	MACS_peak_3288	15.00	TCF7L2_standard_ChIP_seq_CW34
chr4	159821697	159821698	MACS_peak_3289	10.00	TCF7L2_standard_ChIP_seq_CW34
chr4	161350669	161350670	MACS_peak_3290	6.00	TCF7L2_standard_ChIP_seq_CW34
chr4	166033672	166033673	MACS_peak_3291	28.00	TCF7L2_standard_ChIP_seq_CW34
chr4	166177677	166177678	MACS_peak_3292	17.00	TCF7L2_standard_ChIP_seq_CW34
chr4	166300220	166300221	MACS_peak_3293	7.00	TCF7L2_standard_ChIP_seq_CW34
chr4	169858773	169858774	MACS_peak_3294	8.00	TCF7L2_standard_ChIP_seq_CW34
chr4	170305498	170305499	MACS_peak_3295	7.00	TCF7L2_standard_ChIP_seq_CW34
chr4	170467782	170467783	MACS_peak_3296	7.00	TCF7L2_standard_ChIP_seq_CW34
chr4	173647381	173647382	MACS_peak_3297	6.00	TCF7L2_standard_ChIP_seq_CW34
chr4	175205015	175205016	MACS_peak_3298	16.00	TCF7L2_standard_ChIP_seq_CW34
chr4	177540854	177540855	MACS_peak_3299	5.00	TCF7L2_standard_ChIP_seq_CW34
chr4	180962911	180962912	MACS_peak_3300	10.00	TCF7L2_standard_ChIP_seq_CW34
chr4	183369773	183369774	MACS_peak_3301	8.00	TCF7L2_standard_ChIP_seq_CW34
chr4	183720751	183720752	MACS_peak_3302	5.00	TCF7L2_standard_ChIP_seq_CW34
chr4	184001918	184001919	MACS_peak_3303	9.00	TCF7L2_standard_ChIP_seq_CW34
chr4	184019373	184019374	MACS_peak_3304	6.00	TCF7L2_standard_ChIP_seq_CW34
chr4	184365476	184365477	MACS_peak_3305	14.00	TCF7L2_standard_ChIP_seq_CW34
chr4	184540073	184540074	MACS_peak_3306	8.00	TCF7L2_standard_ChIP_seq_CW34
chr4	184645183	184645184	MACS_peak_3307	14.00	TCF7L2_standard_ChIP_seq_CW34
chr4	185025797	185025798	MACS_peak_3308	10.00	TCF7L2_standard_ChIP_seq_CW34
chr4	185395943	185395944	MACS_peak_3309	8.00	TCF7L2_standard_ChIP_seq_CW34
chr4	185547282	185547283	MACS_peak_3310	14.00	TCF7L2_standard_ChIP_seq_CW34
chr4	185760055	185760056	MACS_peak_3311	17.00	TCF7L2_standard_ChIP_seq_CW34
chr4	186175758	186175759	MACS_peak_3312	7.00	TCF7L2_standard_ChIP_seq_CW34
chr4	186254433	186254434	MACS_peak_3313	9.00	TCF7L2_standard_ChIP_seq_CW34
chr4	186347166	186347167	MACS_peak_3314	8.00	TCF7L2_standard_ChIP_seq_CW34
chr4	186456346	186456347	MACS_peak_3315	8.00	TCF7L2_standard_ChIP_seq_CW34
chr4	186513045	186513046	MACS_peak_3316	5.00	TCF7L2_standard_ChIP_seq_CW34
chr4	186695576	186695577	MACS_peak_3317	11.00	TCF7L2_standard_ChIP_seq_CW34
chr4	186732331	186732332	MACS_peak_3318	8.00	TCF7L2_standard_ChIP_seq_CW34
chr4	187565424	187565425	MACS_peak_3319	7.00	TCF7L2_standard_ChIP_seq_CW34
chr4	187606328	187606329	MACS_peak_3320	17.00	TCF7L2_standard_ChIP_seq_CW34
chr4	189136373	189136374	MACS_peak_3321	6.00	TCF7L2_standard_ChIP_seq_CW34
chr4	190845803	190845804	MACS_peak_3322	7.00	TCF7L2_standard_ChIP_seq_CW34
chr5	11613	11614	MACS_peak_3323	19.00	TCF7L2_standard_ChIP_seq_CW34
chr5	1333628	1333629	MACS_peak_3324	8.00	TCF7L2_standard_ChIP_seq_CW34
chr5	1540085	1540086	MACS_peak_3325	5.00	TCF7L2_standard_ChIP_seq_CW34
chr5	1555655	1555656	MACS_peak_3326	11.00	TCF7L2_standard_ChIP_seq_CW34
chr5	1564738	1564739	MACS_peak_3327	13.00	TCF7L2_standard_ChIP_seq_CW34
chr5	5132501	5132502	MACS_peak_3328	8.00	TCF7L2_standard_ChIP_seq_CW34
chr5	6496143	6496144	MACS_peak_3329	7.00	TCF7L2_standard_ChIP_seq_CW34
chr5	10157572	10157573	MACS_peak_3330	9.00	TCF7L2_standard_ChIP_seq_CW34
chr5	10350754	10350755	MACS_peak_3331	6.00	TCF7L2_standard_ChIP_seq_CW34
chr5	10760361	10760362	MACS_peak_3332	7.00	TCF7L2_standard_ChIP_seq_CW34
chr5	11596974	11596975	MACS_peak_3333	7.00	TCF7L2_standard_ChIP_seq_CW34
chr5	14144613	14144614	MACS_peak_3334	7.00	TCF7L2_standard_ChIP_seq_CW34
chr5	14214062	14214063	MACS_peak_3335	13.00	TCF7L2_standard_ChIP_seq_CW34
chr5	14753483	14753484	MACS_peak_3336	11.00	TCF7L2_standard_ChIP_seq_CW34
chr5	14842456	14842457	MACS_peak_3337	18.00	TCF7L2_standard_ChIP_seq_CW34
chr5	14915616	14915617	MACS_peak_3338	7.00	TCF7L2_standard_ChIP_seq_CW34
chr5	15388230	15388231	MACS_peak_3339	12.00	TCF7L2_standard_ChIP_seq_CW34
chr5	16758303	16758304	MACS_peak_3340	5.00	TCF7L2_standard_ChIP_seq_CW34
chr5	19881137	19881138	MACS_peak_3341	12.00	TCF7L2_standard_ChIP_seq_CW34
chr5	21533989	21533990	MACS_peak_3342	7.00	TCF7L2_standard_ChIP_seq_CW34
chr5	29759367	29759368	MACS_peak_3344	8.00	TCF7L2_standard_ChIP_seq_CW34
chr5	30505301	30505302	MACS_peak_3345	9.00	TCF7L2_standard_ChIP_seq_CW34
chr5	30605957	30605958	MACS_peak_3346	18.00	TCF7L2_standard_ChIP_seq_CW34
chr5	32201377	32201378	MACS_peak_3347	17.00	TCF7L2_standard_ChIP_seq_CW34
chr5	32268278	32268279	MACS_peak_3348	9.00	TCF7L2_standard_ChIP_seq_CW34
chr5	32343342	32343343	MACS_peak_3349	10.00	TCF7L2_standard_ChIP_seq_CW34
chr5	32527221	32527222	MACS_peak_3350	12.00	TCF7L2_standard_ChIP_seq_CW34
chr5	32531911	32531912	MACS_peak_3351	8.00	TCF7L2_standard_ChIP_seq_CW34
chr5	32556870	32556871	MACS_peak_3352	16.00	TCF7L2_standard_ChIP_seq_CW34
chr5	34036063	34036064	MACS_peak_3353	18.00	TCF7L2_standard_ChIP_seq_CW34
chr5	35116241	35116242	MACS_peak_3354	12.00	TCF7L2_standard_ChIP_seq_CW34
chr5	36180991	36180992	MACS_peak_3355	14.00	TCF7L2_standard_ChIP_seq_CW34
chr5	36239583	36239584	MACS_peak_3356	16.00	TCF7L2_standard_ChIP_seq_CW34
chr5	36403424	36403425	MACS_peak_3357	6.00	TCF7L2_standard_ChIP_seq_CW34
chr5	37002410	37002411	MACS_peak_3358	10.00	TCF7L2_standard_ChIP_seq_CW34
chr5	37090630	37090631	MACS_peak_3359	11.00	TCF7L2_standard_ChIP_seq_CW34
chr5	37349486	37349487	MACS_peak_3360	10.00	TCF7L2_standard_ChIP_seq_CW34
chr5	37371170	37371171	MACS_peak_3361	12.00	TCF7L2_standard_ChIP_seq_CW34
chr5	37559304	37559305	MACS_peak_3362	6.00	TCF7L2_standard_ChIP_seq_CW34
chr5	37654830	37654831	MACS_peak_3363	25.00	TCF7L2_standard_ChIP_seq_CW34
chr5	37718302	37718303	MACS_peak_3364	9.00	TCF7L2_standard_ChIP_seq_CW34
chr5	38149384	38149385	MACS_peak_3365	20.00	TCF7L2_standard_ChIP_seq_CW34
chr5	38165216	38165217	MACS_peak_3366	14.00	TCF7L2_standard_ChIP_seq_CW34
chr5	38257562	38257563	MACS_peak_3367	7.00	TCF7L2_standard_ChIP_seq_CW34
chr5	39103006	39103007	MACS_peak_3368	7.00	TCF7L2_standard_ChIP_seq_CW34
chr5	39218032	39218033	MACS_peak_3369	11.00	TCF7L2_standard_ChIP_seq_CW34
chr5	39401026	39401027	MACS_peak_3370	6.00	TCF7L2_standard_ChIP_seq_CW34
chr5	39437743	39437744	MACS_peak_3371	8.00	TCF7L2_standard_ChIP_seq_CW34
chr5	39527382	39527383	MACS_peak_3372	10.00	TCF7L2_standard_ChIP_seq_CW34
chr5	40827155	40827156	MACS_peak_3373	6.00	TCF7L2_standard_ChIP_seq_CW34
chr5	40991621	40991622	MACS_peak_3374	8.00	TCF7L2_standard_ChIP_seq_CW34
chr5	41638927	41638928	MACS_peak_3375	15.00	TCF7L2_standard_ChIP_seq_CW34
chr5	42812150	42812151	MACS_peak_3376	7.00	TCF7L2_standard_ChIP_seq_CW34
chr5	43432690	43432691	MACS_peak_3377	12.00	TCF7L2_standard_ChIP_seq_CW34
chr5	43997923	43997924	MACS_peak_3378	10.00	TCF7L2_standard_ChIP_seq_CW34
chr5	44002876	44002877	MACS_peak_3379	11.00	TCF7L2_standard_ChIP_seq_CW34
chr5	52270223	52270224	MACS_peak_3385	7.00	TCF7L2_standard_ChIP_seq_CW34
chr5	52749918	52749919	MACS_peak_3386	11.00	TCF7L2_standard_ChIP_seq_CW34
chr5	52773069	52773070	MACS_peak_3387	12.00	TCF7L2_standard_ChIP_seq_CW34
chr5	54872229	54872230	MACS_peak_3388	11.00	TCF7L2_standard_ChIP_seq_CW34
chr5	55230204	55230205	MACS_peak_3389	38.00	TCF7L2_standard_ChIP_seq_CW34
chr5	55412511	55412512	MACS_peak_3390	16.00	TCF7L2_standard_ChIP_seq_CW34
chr5	56112763	56112764	MACS_peak_3391	19.00	TCF7L2_standard_ChIP_seq_CW34
chr5	56276037	56276038	MACS_peak_3392	8.00	TCF7L2_standard_ChIP_seq_CW34
chr5	57014165	57014166	MACS_peak_3393	7.00	TCF7L2_standard_ChIP_seq_CW34
chr5	58335621	58335622	MACS_peak_3394	18.00	TCF7L2_standard_ChIP_seq_CW34
chr5	58359245	58359246	MACS_peak_3395	7.00	TCF7L2_standard_ChIP_seq_CW34
chr5	58389419	58389420	MACS_peak_3396	11.00	TCF7L2_standard_ChIP_seq_CW34
chr5	59502258	59502259	MACS_peak_3397	15.00	TCF7L2_standard_ChIP_seq_CW34
chr5	59743443	59743444	MACS_peak_3398	10.00	TCF7L2_standard_ChIP_seq_CW34
chr5	59769714	59769715	MACS_peak_3399	12.00	TCF7L2_standard_ChIP_seq_CW34
chr5	59838035	59838036	MACS_peak_3400	12.00	TCF7L2_standard_ChIP_seq_CW34
chr5	59968877	59968878	MACS_peak_3401	17.00	TCF7L2_standard_ChIP_seq_CW34
chr5	61601739	61601740	MACS_peak_3402	10.00	TCF7L2_standard_ChIP_seq_CW34
chr5	61618448	61618449	MACS_peak_3403	11.00	TCF7L2_standard_ChIP_seq_CW34
chr5	61953784	61953785	MACS_peak_3404	12.00	TCF7L2_standard_ChIP_seq_CW34
chr5	62233918	62233919	MACS_peak_3405	8.00	TCF7L2_standard_ChIP_seq_CW34
chr5	64064660	64064661	MACS_peak_3406	6.00	TCF7L2_standard_ChIP_seq_CW34
chr5	66300173	66300174	MACS_peak_3407	11.00	TCF7L2_standard_ChIP_seq_CW34
chr5	67066722	67066723	MACS_peak_3408	8.00	TCF7L2_standard_ChIP_seq_CW34
chr5	67267329	67267330	MACS_peak_3409	11.00	TCF7L2_standard_ChIP_seq_CW34
chr5	72840634	72840635	MACS_peak_3411	11.00	TCF7L2_standard_ChIP_seq_CW34
chr5	72919927	72919928	MACS_peak_3412	42.00	TCF7L2_standard_ChIP_seq_CW34
chr5	73059245	73059246	MACS_peak_3413	12.00	TCF7L2_standard_ChIP_seq_CW34
chr5	73128714	73128715	MACS_peak_3414	5.00	TCF7L2_standard_ChIP_seq_CW34
chr5	73242245	73242246	MACS_peak_3415	9.00	TCF7L2_standard_ChIP_seq_CW34
chr5	73796475	73796476	MACS_peak_3416	8.00	TCF7L2_standard_ChIP_seq_CW34
chr5	73877086	73877087	MACS_peak_3417	25.00	TCF7L2_standard_ChIP_seq_CW34
chr5	74172257	74172258	MACS_peak_3418	15.00	TCF7L2_standard_ChIP_seq_CW34
chr5	74670424	74670425	MACS_peak_3419	11.00	TCF7L2_standard_ChIP_seq_CW34
chr5	74819272	74819273	MACS_peak_3420	9.00	TCF7L2_standard_ChIP_seq_CW34
chr5	76682343	76682344	MACS_peak_3421	11.00	TCF7L2_standard_ChIP_seq_CW34
chr5	77842721	77842722	MACS_peak_3422	12.00	TCF7L2_standard_ChIP_seq_CW34
chr5	78281753	78281754	MACS_peak_3423	37.00	TCF7L2_standard_ChIP_seq_CW34
chr5	78326729	78326730	MACS_peak_3424	25.00	TCF7L2_standard_ChIP_seq_CW34
chr5	79286769	79286770	MACS_peak_3425	14.00	TCF7L2_standard_ChIP_seq_CW34
chr5	79477699	79477700	MACS_peak_3426	11.00	TCF7L2_standard_ChIP_seq_CW34
chr5	81652881	81652882	MACS_peak_3427	7.00	TCF7L2_standard_ChIP_seq_CW34
chr5	81683323	81683324	MACS_peak_3428	42.00	TCF7L2_standard_ChIP_seq_CW34
chr5	82273069	82273070	MACS_peak_3429	14.00	TCF7L2_standard_ChIP_seq_CW34
chr5	82420521	82420522	MACS_peak_3430	8.00	TCF7L2_standard_ChIP_seq_CW34
chr5	82737804	82737805	MACS_peak_3431	6.00	TCF7L2_standard_ChIP_seq_CW34
chr5	85126797	85126798	MACS_peak_3432	5.00	TCF7L2_standard_ChIP_seq_CW34
chr5	90207179	90207180	MACS_peak_3433	11.00	TCF7L2_standard_ChIP_seq_CW34
chr5	90303241	90303242	MACS_peak_3434	7.00	TCF7L2_standard_ChIP_seq_CW34
chr5	90576670	90576671	MACS_peak_3435	10.00	TCF7L2_standard_ChIP_seq_CW34
chr5	92263142	92263143	MACS_peak_3436	13.00	TCF7L2_standard_ChIP_seq_CW34
chr5	92281014	92281015	MACS_peak_3437	6.00	TCF7L2_standard_ChIP_seq_CW34
chr5	92582269	92582270	MACS_peak_3438	74.00	TCF7L2_standard_ChIP_seq_CW34
chr5	94139158	94139159	MACS_peak_3439	5.00	TCF7L2_standard_ChIP_seq_CW34
chr5	95116553	95116554	MACS_peak_3440	8.00	TCF7L2_standard_ChIP_seq_CW34
chr5	95148053	95148054	MACS_peak_3441	8.00	TCF7L2_standard_ChIP_seq_CW34
chr5	95306003	95306004	MACS_peak_3442	8.00	TCF7L2_standard_ChIP_seq_CW34
chr5	95322961	95322962	MACS_peak_3443	16.00	TCF7L2_standard_ChIP_seq_CW34
chr5	95453863	95453864	MACS_peak_3444	10.00	TCF7L2_standard_ChIP_seq_CW34
chr5	95523317	95523318	MACS_peak_3445	9.00	TCF7L2_standard_ChIP_seq_CW34
chr5	95633803	95633804	MACS_peak_3446	7.00	TCF7L2_standard_ChIP_seq_CW34
chr5	95642937	95642938	MACS_peak_3447	20.00	TCF7L2_standard_ChIP_seq_CW34
chr5	98115443	98115444	MACS_peak_3448	11.00	TCF7L2_standard_ChIP_seq_CW34
chr5	100457567	100457568	MACS_peak_3449	7.00	TCF7L2_standard_ChIP_seq_CW34
chr5	101552705	101552706	MACS_peak_3450	11.00	TCF7L2_standard_ChIP_seq_CW34
chr5	104188095	104188096	MACS_peak_3451	8.00	TCF7L2_standard_ChIP_seq_CW34
chr5	108874541	108874542	MACS_peak_3452	7.00	TCF7L2_standard_ChIP_seq_CW34
chr5	111078508	111078509	MACS_peak_3453	10.00	TCF7L2_standard_ChIP_seq_CW34
chr5	111093725	111093726	MACS_peak_3454	6.00	TCF7L2_standard_ChIP_seq_CW34
chr5	111496450	111496451	MACS_peak_3455	10.00	TCF7L2_standard_ChIP_seq_CW34
chr5	112296894	112296895	MACS_peak_3456	5.00	TCF7L2_standard_ChIP_seq_CW34
chr5	113271341	113271342	MACS_peak_3457	6.00	TCF7L2_standard_ChIP_seq_CW34
chr5	114577829	114577830	MACS_peak_3458	9.00	TCF7L2_standard_ChIP_seq_CW34
chr5	114880192	114880193	MACS_peak_3459	5.00	TCF7L2_standard_ChIP_seq_CW34
chr5	114970528	114970529	MACS_peak_3460	10.00	TCF7L2_standard_ChIP_seq_CW34
chr5	115180454	115180455	MACS_peak_3461	9.00	TCF7L2_standard_ChIP_seq_CW34
chr5	115860834	115860835	MACS_peak_3462	7.00	TCF7L2_standard_ChIP_seq_CW34
chr5	115868758	115868759	MACS_peak_3463	16.00	TCF7L2_standard_ChIP_seq_CW34
chr5	116198970	116198971	MACS_peak_3464	9.00	TCF7L2_standard_ChIP_seq_CW34
chr5	118788622	118788623	MACS_peak_3465	20.00	TCF7L2_standard_ChIP_seq_CW34
chr5	123625610	123625611	MACS_peak_3466	21.00	TCF7L2_standard_ChIP_seq_CW34
chr5	123935606	123935607	MACS_peak_3467	13.00	TCF7L2_standard_ChIP_seq_CW34
chr5	123997394	123997395	MACS_peak_3468	9.00	TCF7L2_standard_ChIP_seq_CW34
chr5	124353664	124353665	MACS_peak_3469	10.00	TCF7L2_standard_ChIP_seq_CW34
chr5	126366232	126366233	MACS_peak_3470	8.00	TCF7L2_standard_ChIP_seq_CW34
chr5	126433284	126433285	MACS_peak_3471	8.00	TCF7L2_standard_ChIP_seq_CW34
chr5	130415096	130415097	MACS_peak_3472	8.00	TCF7L2_standard_ChIP_seq_CW34
chr5	130500333	130500334	MACS_peak_3473	5.00	TCF7L2_standard_ChIP_seq_CW34
chr5	130682076	130682077	MACS_peak_3474	7.00	TCF7L2_standard_ChIP_seq_CW34
chr5	131132698	131132699	MACS_peak_3475	16.00	TCF7L2_standard_ChIP_seq_CW34
chr5	131332653	131332654	MACS_peak_3476	34.00	TCF7L2_standard_ChIP_seq_CW34
chr5	131339212	131339213	MACS_peak_3477	10.00	TCF7L2_standard_ChIP_seq_CW34
chr5	131593129	131593130	MACS_peak_3478	10.00	TCF7L2_standard_ChIP_seq_CW34
chr5	132166315	132166316	MACS_peak_3479	11.00	TCF7L2_standard_ChIP_seq_CW34
chr5	132175861	132175862	MACS_peak_3480	36.00	TCF7L2_standard_ChIP_seq_CW34
chr5	132387391	132387392	MACS_peak_3481	10.00	TCF7L2_standard_ChIP_seq_CW34
chr5	133354587	133354588	MACS_peak_3482	7.00	TCF7L2_standard_ChIP_seq_CW34
chr5	133406995	133406996	MACS_peak_3483	74.00	TCF7L2_standard_ChIP_seq_CW34
chr5	133450397	133450398	MACS_peak_3484	25.00	TCF7L2_standard_ChIP_seq_CW34
chr5	133635761	133635762	MACS_peak_3485	6.00	TCF7L2_standard_ChIP_seq_CW34
chr5	133707096	133707097	MACS_peak_3486	8.00	TCF7L2_standard_ChIP_seq_CW34
chr5	133751767	133751768	MACS_peak_3487	10.00	TCF7L2_standard_ChIP_seq_CW34
chr5	134181852	134181853	MACS_peak_3488	6.00	TCF7L2_standard_ChIP_seq_CW34
chr5	134300545	134300546	MACS_peak_3489	11.00	TCF7L2_standard_ChIP_seq_CW34
chr5	134599133	134599134	MACS_peak_3490	32.00	TCF7L2_standard_ChIP_seq_CW34
chr5	134607627	134607628	MACS_peak_3491	6.00	TCF7L2_standard_ChIP_seq_CW34
chr5	134640302	134640303	MACS_peak_3492	8.00	TCF7L2_standard_ChIP_seq_CW34
chr5	134770616	134770617	MACS_peak_3493	10.00	TCF7L2_standard_ChIP_seq_CW34
chr5	134818279	134818280	MACS_peak_3494	16.00	TCF7L2_standard_ChIP_seq_CW34
chr5	134824679	134824680	MACS_peak_3495	20.00	TCF7L2_standard_ChIP_seq_CW34
chr5	137431389	137431390	MACS_peak_3496	13.00	TCF7L2_standard_ChIP_seq_CW34
chr5	137673597	137673598	MACS_peak_3497	9.00	TCF7L2_standard_ChIP_seq_CW34
chr5	138066825	138066826	MACS_peak_3498	8.00	TCF7L2_standard_ChIP_seq_CW34
chr5	138941105	138941106	MACS_peak_3499	7.00	TCF7L2_standard_ChIP_seq_CW34
chr5	139030062	139030063	MACS_peak_3500	38.00	TCF7L2_standard_ChIP_seq_CW34
chr5	139126154	139126155	MACS_peak_3501	9.00	TCF7L2_standard_ChIP_seq_CW34
chr5	139135978	139135979	MACS_peak_3502	16.00	TCF7L2_standard_ChIP_seq_CW34
chr5	139743012	139743013	MACS_peak_3503	11.00	TCF7L2_standard_ChIP_seq_CW34
chr5	142302149	142302150	MACS_peak_3504	16.00	TCF7L2_standard_ChIP_seq_CW34
chr5	142926916	142926917	MACS_peak_3505	5.00	TCF7L2_standard_ChIP_seq_CW34
chr5	142934956	142934957	MACS_peak_3506	16.00	TCF7L2_standard_ChIP_seq_CW34
chr5	142975875	142975876	MACS_peak_3507	12.00	TCF7L2_standard_ChIP_seq_CW34
chr5	144857746	144857747	MACS_peak_3508	8.00	TCF7L2_standard_ChIP_seq_CW34
chr5	145593278	145593279	MACS_peak_3509	7.00	TCF7L2_standard_ChIP_seq_CW34
chr5	146550467	146550468	MACS_peak_3510	17.00	TCF7L2_standard_ChIP_seq_CW34
chr5	147215038	147215039	MACS_peak_3511	23.00	TCF7L2_standard_ChIP_seq_CW34
chr5	148931371	148931372	MACS_peak_3512	12.00	TCF7L2_standard_ChIP_seq_CW34
chr5	149517012	149517013	MACS_peak_3513	25.00	TCF7L2_standard_ChIP_seq_CW34
chr5	149687610	149687611	MACS_peak_3514	27.00	TCF7L2_standard_ChIP_seq_CW34
chr5	149792319	149792320	MACS_peak_3515	12.00	TCF7L2_standard_ChIP_seq_CW34
chr5	149902224	149902225	MACS_peak_3516	10.00	TCF7L2_standard_ChIP_seq_CW34
chr5	149922758	149922759	MACS_peak_3517	11.00	TCF7L2_standard_ChIP_seq_CW34
chr5	150384286	150384287	MACS_peak_3518	8.00	TCF7L2_standard_ChIP_seq_CW34
chr5	150614581	150614582	MACS_peak_3519	8.00	TCF7L2_standard_ChIP_seq_CW34
chr5	150972604	150972605	MACS_peak_3520	6.00	TCF7L2_standard_ChIP_seq_CW34
chr5	150997722	150997723	MACS_peak_3521	14.00	TCF7L2_standard_ChIP_seq_CW34
chr5	154026890	154026891	MACS_peak_3522	8.00	TCF7L2_standard_ChIP_seq_CW34
chr5	154222847	154222848	MACS_peak_3523	8.00	TCF7L2_standard_ChIP_seq_CW34
chr5	154388409	154388410	MACS_peak_3524	7.00	TCF7L2_standard_ChIP_seq_CW34
chr5	157946807	157946808	MACS_peak_3525	11.00	TCF7L2_standard_ChIP_seq_CW34
chr5	158100347	158100348	MACS_peak_3526	5.00	TCF7L2_standard_ChIP_seq_CW34
chr5	160973730	160973731	MACS_peak_3527	20.00	TCF7L2_standard_ChIP_seq_CW34
chr5	165055042	165055043	MACS_peak_3528	10.00	TCF7L2_standard_ChIP_seq_CW34
chr5	168023329	168023330	MACS_peak_3529	12.00	TCF7L2_standard_ChIP_seq_CW34
chr5	168052050	168052051	MACS_peak_3530	22.00	TCF7L2_standard_ChIP_seq_CW34
chr5	168510605	168510606	MACS_peak_3531	12.00	TCF7L2_standard_ChIP_seq_CW34
chr5	170846496	170846497	MACS_peak_3532	17.00	TCF7L2_standard_ChIP_seq_CW34
chr5	171051026	171051027	MACS_peak_3533	11.00	TCF7L2_standard_ChIP_seq_CW34
chr5	171591505	171591506	MACS_peak_3534	9.00	TCF7L2_standard_ChIP_seq_CW34
chr5	172087232	172087233	MACS_peak_3535	15.00	TCF7L2_standard_ChIP_seq_CW34
chr5	172286510	172286511	MACS_peak_3536	22.00	TCF7L2_standard_ChIP_seq_CW34
chr5	172314136	172314137	MACS_peak_3537	10.00	TCF7L2_standard_ChIP_seq_CW34
chr5	172321866	172321867	MACS_peak_3538	15.00	TCF7L2_standard_ChIP_seq_CW34
chr5	173043236	173043237	MACS_peak_3539	7.00	TCF7L2_standard_ChIP_seq_CW34
chr5	173216180	173216181	MACS_peak_3540	6.00	TCF7L2_standard_ChIP_seq_CW34
chr5	174895184	174895185	MACS_peak_3541	20.00	TCF7L2_standard_ChIP_seq_CW34
chr5	175084237	175084238	MACS_peak_3542	7.00	TCF7L2_standard_ChIP_seq_CW34
chr5	175224201	175224202	MACS_peak_3543	8.00	TCF7L2_standard_ChIP_seq_CW34
chr5	175281757	175281758	MACS_peak_3544	15.00	TCF7L2_standard_ChIP_seq_CW34
chr5	175288605	175288606	MACS_peak_3545	38.00	TCF7L2_standard_ChIP_seq_CW34
chr5	175964206	175964207	MACS_peak_3546	10.00	TCF7L2_standard_ChIP_seq_CW34
chr5	177557868	177557869	MACS_peak_3547	11.00	TCF7L2_standard_ChIP_seq_CW34
chr5	178057622	178057623	MACS_peak_3548	32.00	TCF7L2_standard_ChIP_seq_CW34
chr5	180127710	180127711	MACS_peak_3549	5.00	TCF7L2_standard_ChIP_seq_CW34
chr5	180429181	180429182	MACS_peak_3550	8.00	TCF7L2_standard_ChIP_seq_CW34
chr6	1055462	1055463	MACS_peak_3551	129.00	TCF7L2_standard_ChIP_seq_CW34
chr6	1228928	1228929	MACS_peak_3552	26.00	TCF7L2_standard_ChIP_seq_CW34
chr6	1264401	1264402	MACS_peak_3553	38.00	TCF7L2_standard_ChIP_seq_CW34
chr6	1268767	1268768	MACS_peak_3554	38.00	TCF7L2_standard_ChIP_seq_CW34
chr6	1317858	1317859	MACS_peak_3555	16.00	TCF7L2_standard_ChIP_seq_CW34
chr6	1335853	1335854	MACS_peak_3556	8.00	TCF7L2_standard_ChIP_seq_CW34
chr6	2226076	2226077	MACS_peak_3557	11.00	TCF7L2_standard_ChIP_seq_CW34
chr6	2515423	2515424	MACS_peak_3558	52.00	TCF7L2_standard_ChIP_seq_CW34
chr6	2765812	2765813	MACS_peak_3559	7.00	TCF7L2_standard_ChIP_seq_CW34
chr6	2841891	2841892	MACS_peak_3560	11.00	TCF7L2_standard_ChIP_seq_CW34
chr6	2876982	2876983	MACS_peak_3561	37.00	TCF7L2_standard_ChIP_seq_CW34
chr6	2994193	2994194	MACS_peak_3562	11.00	TCF7L2_standard_ChIP_seq_CW34
chr6	3403659	3403660	MACS_peak_3563	10.00	TCF7L2_standard_ChIP_seq_CW34
chr6	3410219	3410220	MACS_peak_3564	52.00	TCF7L2_standard_ChIP_seq_CW34
chr6	3504751	3504752	MACS_peak_3565	27.00	TCF7L2_standard_ChIP_seq_CW34
chr6	3750934	3750935	MACS_peak_3566	24.00	TCF7L2_standard_ChIP_seq_CW34
chr6	4018914	4018915	MACS_peak_3567	9.00	TCF7L2_standard_ChIP_seq_CW34
chr6	5068521	5068522	MACS_peak_3568	8.00	TCF7L2_standard_ChIP_seq_CW34
chr6	5115571	5115572	MACS_peak_3569	30.00	TCF7L2_standard_ChIP_seq_CW34
chr6	5713917	5713918	MACS_peak_3570	9.00	TCF7L2_standard_ChIP_seq_CW34
chr6	7029433	7029434	MACS_peak_3571	10.00	TCF7L2_standard_ChIP_seq_CW34
chr6	7464127	7464128	MACS_peak_3572	10.00	TCF7L2_standard_ChIP_seq_CW34
chr6	7543147	7543148	MACS_peak_3573	9.00	TCF7L2_standard_ChIP_seq_CW34
chr6	7548929	7548930	MACS_peak_3574	18.00	TCF7L2_standard_ChIP_seq_CW34
chr6	7562017	7562018	MACS_peak_3575	12.00	TCF7L2_standard_ChIP_seq_CW34
chr6	7698602	7698603	MACS_peak_3576	11.00	TCF7L2_standard_ChIP_seq_CW34
chr6	7975468	7975469	MACS_peak_3577	7.00	TCF7L2_standard_ChIP_seq_CW34
chr6	8138020	8138021	MACS_peak_3578	22.00	TCF7L2_standard_ChIP_seq_CW34
chr6	8959283	8959284	MACS_peak_3579	9.00	TCF7L2_standard_ChIP_seq_CW34
chr6	9801567	9801568	MACS_peak_3580	5.00	TCF7L2_standard_ChIP_seq_CW34
chr6	10505985	10505986	MACS_peak_3581	63.00	TCF7L2_standard_ChIP_seq_CW34
chr6	10957964	10957965	MACS_peak_3582	14.00	TCF7L2_standard_ChIP_seq_CW34
chr6	11012731	11012732	MACS_peak_3583	10.00	TCF7L2_standard_ChIP_seq_CW34
chr6	11044484	11044485	MACS_peak_3584	6.00	TCF7L2_standard_ChIP_seq_CW34
chr6	11093911	11093912	MACS_peak_3585	13.00	TCF7L2_standard_ChIP_seq_CW34
chr6	11324504	11324505	MACS_peak_3586	15.00	TCF7L2_standard_ChIP_seq_CW34
chr6	11368005	11368006	MACS_peak_3587	13.00	TCF7L2_standard_ChIP_seq_CW34
chr6	11392867	11392868	MACS_peak_3588	20.00	TCF7L2_standard_ChIP_seq_CW34
chr6	11399325	11399326	MACS_peak_3589	8.00	TCF7L2_standard_ChIP_seq_CW34
chr6	11435243	11435244	MACS_peak_3590	10.00	TCF7L2_standard_ChIP_seq_CW34
chr6	11454569	11454570	MACS_peak_3591	9.00	TCF7L2_standard_ChIP_seq_CW34
chr6	11463383	11463384	MACS_peak_3592	12.00	TCF7L2_standard_ChIP_seq_CW34
chr6	11537812	11537813	MACS_peak_3593	7.00	TCF7L2_standard_ChIP_seq_CW34
chr6	12001479	12001480	MACS_peak_3594	11.00	TCF7L2_standard_ChIP_seq_CW34
chr6	12008947	12008948	MACS_peak_3595	52.00	TCF7L2_standard_ChIP_seq_CW34
chr6	12348958	12348959	MACS_peak_3596	13.00	TCF7L2_standard_ChIP_seq_CW34
chr6	13181845	13181846	MACS_peak_3597	8.00	TCF7L2_standard_ChIP_seq_CW34
chr6	13290454	13290455	MACS_peak_3598	10.00	TCF7L2_standard_ChIP_seq_CW34
chr6	13408121	13408122	MACS_peak_3599	13.00	TCF7L2_standard_ChIP_seq_CW34
chr6	13414014	13414015	MACS_peak_3600	16.00	TCF7L2_standard_ChIP_seq_CW34
chr6	13605256	13605257	MACS_peak_3601	29.00	TCF7L2_standard_ChIP_seq_CW34
chr6	13829286	13829287	MACS_peak_3602	21.00	TCF7L2_standard_ChIP_seq_CW34
chr6	13989080	13989081	MACS_peak_3603	10.00	TCF7L2_standard_ChIP_seq_CW34
chr6	14622554	14622555	MACS_peak_3604	27.00	TCF7L2_standard_ChIP_seq_CW34
chr6	15526177	15526178	MACS_peak_3605	10.00	TCF7L2_standard_ChIP_seq_CW34
chr6	15764224	15764225	MACS_peak_3606	15.00	TCF7L2_standard_ChIP_seq_CW34
chr6	16691154	16691155	MACS_peak_3607	8.00	TCF7L2_standard_ChIP_seq_CW34
chr6	16729525	16729526	MACS_peak_3608	11.00	TCF7L2_standard_ChIP_seq_CW34
chr6	16808415	16808416	MACS_peak_3609	14.00	TCF7L2_standard_ChIP_seq_CW34
chr6	16822296	16822297	MACS_peak_3610	22.00	TCF7L2_standard_ChIP_seq_CW34
chr6	17355220	17355221	MACS_peak_3611	12.00	TCF7L2_standard_ChIP_seq_CW34
chr6	17930229	17930230	MACS_peak_3612	8.00	TCF7L2_standard_ChIP_seq_CW34
chr6	17987050	17987051	MACS_peak_3613	7.00	TCF7L2_standard_ChIP_seq_CW34
chr6	20594688	20594689	MACS_peak_3614	27.00	TCF7L2_standard_ChIP_seq_CW34
chr6	20631363	20631364	MACS_peak_3615	26.00	TCF7L2_standard_ChIP_seq_CW34
chr6	21004224	21004225	MACS_peak_3616	35.00	TCF7L2_standard_ChIP_seq_CW34
chr6	21381970	21381971	MACS_peak_3617	15.00	TCF7L2_standard_ChIP_seq_CW34
chr6	23965203	23965204	MACS_peak_3618	6.00	TCF7L2_standard_ChIP_seq_CW34
chr6	24146077	24146078	MACS_peak_3619	32.00	TCF7L2_standard_ChIP_seq_CW34
chr6	24148466	24148467	MACS_peak_3620	40.00	TCF7L2_standard_ChIP_seq_CW34
chr6	24313807	24313808	MACS_peak_3621	10.00	TCF7L2_standard_ChIP_seq_CW34
chr6	24330868	24330869	MACS_peak_3622	18.00	TCF7L2_standard_ChIP_seq_CW34
chr6	24341494	24341495	MACS_peak_3623	12.00	TCF7L2_standard_ChIP_seq_CW34
chr6	24385922	24385923	MACS_peak_3624	20.00	TCF7L2_standard_ChIP_seq_CW34
chr6	24403402	24403403	MACS_peak_3625	6.00	TCF7L2_standard_ChIP_seq_CW34
chr6	24667199	24667200	MACS_peak_3626	8.00	TCF7L2_standard_ChIP_seq_CW34
chr6	24721134	24721135	MACS_peak_3627	8.00	TCF7L2_standard_ChIP_seq_CW34
chr6	25209859	25209860	MACS_peak_3628	9.00	TCF7L2_standard_ChIP_seq_CW34
chr6	25270974	25270975	MACS_peak_3629	13.00	TCF7L2_standard_ChIP_seq_CW34
chr6	25910757	25910758	MACS_peak_3630	8.00	TCF7L2_standard_ChIP_seq_CW34
chr6	25929389	25929390	MACS_peak_3631	27.00	TCF7L2_standard_ChIP_seq_CW34
chr6	26475514	26475515	MACS_peak_3632	15.00	TCF7L2_standard_ChIP_seq_CW34
chr6	27760764	27760765	MACS_peak_3633	7.00	TCF7L2_standard_ChIP_seq_CW34
chr6	28186530	28186531	MACS_peak_3634	8.00	TCF7L2_standard_ChIP_seq_CW34
chr6	28193073	28193074	MACS_peak_3635	12.00	TCF7L2_standard_ChIP_seq_CW34
chr6	28367603	28367604	MACS_peak_3636	10.00	TCF7L2_standard_ChIP_seq_CW34
chr6	28831420	28831421	MACS_peak_3637	7.00	TCF7L2_standard_ChIP_seq_CW34
chr6	29933927	29933928	MACS_peak_3638	27.00	TCF7L2_standard_ChIP_seq_CW34
chr6	30054372	30054373	MACS_peak_3639	7.00	TCF7L2_standard_ChIP_seq_CW34
chr6	30457701	30457702	MACS_peak_3640	7.00	TCF7L2_standard_ChIP_seq_CW34
chr6	30737467	30737468	MACS_peak_3641	7.00	TCF7L2_standard_ChIP_seq_CW34
chr6	30790658	30790659	MACS_peak_3642	10.00	TCF7L2_standard_ChIP_seq_CW34
chr6	31634026	31634027	MACS_peak_3643	12.00	TCF7L2_standard_ChIP_seq_CW34
chr6	31682145	31682146	MACS_peak_3644	9.00	TCF7L2_standard_ChIP_seq_CW34
chr6	31914227	31914228	MACS_peak_3645	19.00	TCF7L2_standard_ChIP_seq_CW34
chr6	33166448	33166449	MACS_peak_3646	11.00	TCF7L2_standard_ChIP_seq_CW34
chr6	33581303	33581304	MACS_peak_3647	32.00	TCF7L2_standard_ChIP_seq_CW34
chr6	33756920	33756921	MACS_peak_3648	8.00	TCF7L2_standard_ChIP_seq_CW34
chr6	33956234	33956235	MACS_peak_3649	12.00	TCF7L2_standard_ChIP_seq_CW34
chr6	34216819	34216820	MACS_peak_3650	26.00	TCF7L2_standard_ChIP_seq_CW34
chr6	34359669	34359670	MACS_peak_3651	14.00	TCF7L2_standard_ChIP_seq_CW34
chr6	34641587	34641588	MACS_peak_3652	18.00	TCF7L2_standard_ChIP_seq_CW34
chr6	34725036	34725037	MACS_peak_3653	9.00	TCF7L2_standard_ChIP_seq_CW34
chr6	35227525	35227526	MACS_peak_3654	10.00	TCF7L2_standard_ChIP_seq_CW34
chr6	35336411	35336412	MACS_peak_3655	11.00	TCF7L2_standard_ChIP_seq_CW34
chr6	35700038	35700039	MACS_peak_3656	48.00	TCF7L2_standard_ChIP_seq_CW34
chr6	36651601	36651602	MACS_peak_3657	19.00	TCF7L2_standard_ChIP_seq_CW34
chr6	36954238	36954239	MACS_peak_3658	14.00	TCF7L2_standard_ChIP_seq_CW34
chr6	37073143	37073144	MACS_peak_3659	40.00	TCF7L2_standard_ChIP_seq_CW34
chr6	37094061	37094062	MACS_peak_3660	19.00	TCF7L2_standard_ChIP_seq_CW34
chr6	37137501	37137502	MACS_peak_3661	44.00	TCF7L2_standard_ChIP_seq_CW34
chr6	37787010	37787011	MACS_peak_3662	9.00	TCF7L2_standard_ChIP_seq_CW34
chr6	38013763	38013764	MACS_peak_3663	11.00	TCF7L2_standard_ChIP_seq_CW34
chr6	40867571	40867572	MACS_peak_3664	26.00	TCF7L2_standard_ChIP_seq_CW34
chr6	41702452	41702453	MACS_peak_3665	9.00	TCF7L2_standard_ChIP_seq_CW34
chr6	41709519	41709520	MACS_peak_3666	11.00	TCF7L2_standard_ChIP_seq_CW34
chr6	41715248	41715249	MACS_peak_3667	14.00	TCF7L2_standard_ChIP_seq_CW34
chr6	42018651	42018652	MACS_peak_3668	9.00	TCF7L2_standard_ChIP_seq_CW34
chr6	43058030	43058031	MACS_peak_3669	53.00	TCF7L2_standard_ChIP_seq_CW34
chr6	43238035	43238036	MACS_peak_3670	13.00	TCF7L2_standard_ChIP_seq_CW34
chr6	43603565	43603566	MACS_peak_3671	12.00	TCF7L2_standard_ChIP_seq_CW34
chr6	43737188	43737189	MACS_peak_3672	8.00	TCF7L2_standard_ChIP_seq_CW34
chr6	43880554	43880555	MACS_peak_3673	9.00	TCF7L2_standard_ChIP_seq_CW34
chr6	43895078	43895079	MACS_peak_3674	8.00	TCF7L2_standard_ChIP_seq_CW34
chr6	43902906	43902907	MACS_peak_3675	10.00	TCF7L2_standard_ChIP_seq_CW34
chr6	43970089	43970090	MACS_peak_3676	16.00	TCF7L2_standard_ChIP_seq_CW34
chr6	44041091	44041092	MACS_peak_3677	8.00	TCF7L2_standard_ChIP_seq_CW34
chr6	44187123	44187124	MACS_peak_3678	10.00	TCF7L2_standard_ChIP_seq_CW34
chr6	44355496	44355497	MACS_peak_3679	5.00	TCF7L2_standard_ChIP_seq_CW34
chr6	45413794	45413795	MACS_peak_3680	8.00	TCF7L2_standard_ChIP_seq_CW34
chr6	46545281	46545282	MACS_peak_3681	6.00	TCF7L2_standard_ChIP_seq_CW34
chr6	46757868	46757869	MACS_peak_3682	9.00	TCF7L2_standard_ChIP_seq_CW34
chr6	47041084	47041085	MACS_peak_3683	13.00	TCF7L2_standard_ChIP_seq_CW34
chr6	47044698	47044699	MACS_peak_3684	19.00	TCF7L2_standard_ChIP_seq_CW34
chr6	47277728	47277729	MACS_peak_3685	11.00	TCF7L2_standard_ChIP_seq_CW34
chr6	47428428	47428429	MACS_peak_3686	8.00	TCF7L2_standard_ChIP_seq_CW34
chr6	48181296	48181297	MACS_peak_3687	8.00	TCF7L2_standard_ChIP_seq_CW34
chr6	49490746	49490747	MACS_peak_3688	10.00	TCF7L2_standard_ChIP_seq_CW34
chr6	49734625	49734626	MACS_peak_3689	7.00	TCF7L2_standard_ChIP_seq_CW34
chr6	51946182	51946183	MACS_peak_3690	7.00	TCF7L2_standard_ChIP_seq_CW34
chr6	52190784	52190785	MACS_peak_3691	27.00	TCF7L2_standard_ChIP_seq_CW34
chr6	52590728	52590729	MACS_peak_3692	17.00	TCF7L2_standard_ChIP_seq_CW34
chr6	52666258	52666259	MACS_peak_3693	7.00	TCF7L2_standard_ChIP_seq_CW34
chr6	52919521	52919522	MACS_peak_3694	12.00	TCF7L2_standard_ChIP_seq_CW34
chr6	52999733	52999734	MACS_peak_3695	32.00	TCF7L2_standard_ChIP_seq_CW34
chr6	53213323	53213324	MACS_peak_3696	6.00	TCF7L2_standard_ChIP_seq_CW34
chr6	53480659	53480660	MACS_peak_3697	15.00	TCF7L2_standard_ChIP_seq_CW34
chr6	53719803	53719804	MACS_peak_3698	9.00	TCF7L2_standard_ChIP_seq_CW34
chr6	53962714	53962715	MACS_peak_3699	11.00	TCF7L2_standard_ChIP_seq_CW34
chr6	54069797	54069798	MACS_peak_3700	9.00	TCF7L2_standard_ChIP_seq_CW34
chr6	56264688	56264689	MACS_peak_3701	13.00	TCF7L2_standard_ChIP_seq_CW34
chr6	56507322	56507323	MACS_peak_3702	10.00	TCF7L2_standard_ChIP_seq_CW34
chr6	56954888	56954889	MACS_peak_3703	8.00	TCF7L2_standard_ChIP_seq_CW34
chr6	57006821	57006822	MACS_peak_3704	9.00	TCF7L2_standard_ChIP_seq_CW34
chr6	57687178	57687179	MACS_peak_3705	8.00	TCF7L2_standard_ChIP_seq_CW34
chr6	57929437	57929438	MACS_peak_3706	8.00	TCF7L2_standard_ChIP_seq_CW34
chr6	63943987	63943988	MACS_peak_3708	8.00	TCF7L2_standard_ChIP_seq_CW34
chr6	64237754	64237755	MACS_peak_3709	11.00	TCF7L2_standard_ChIP_seq_CW34
chr6	64516476	64516477	MACS_peak_3710	9.00	TCF7L2_standard_ChIP_seq_CW34
chr6	64756776	64756777	MACS_peak_3711	16.00	TCF7L2_standard_ChIP_seq_CW34
chr6	65011644	65011645	MACS_peak_3712	15.00	TCF7L2_standard_ChIP_seq_CW34
chr6	71123743	71123744	MACS_peak_3713	6.00	TCF7L2_standard_ChIP_seq_CW34
chr6	72061510	72061511	MACS_peak_3714	6.00	TCF7L2_standard_ChIP_seq_CW34
chr6	74295412	74295413	MACS_peak_3715	11.00	TCF7L2_standard_ChIP_seq_CW34
chr6	76234285	76234286	MACS_peak_3716	9.00	TCF7L2_standard_ChIP_seq_CW34
chr6	78544530	78544531	MACS_peak_3717	11.00	TCF7L2_standard_ChIP_seq_CW34
chr6	79577470	79577471	MACS_peak_3718	9.00	TCF7L2_standard_ChIP_seq_CW34
chr6	79787983	79787984	MACS_peak_3719	5.00	TCF7L2_standard_ChIP_seq_CW34
chr6	80341028	80341029	MACS_peak_3720	18.00	TCF7L2_standard_ChIP_seq_CW34
chr6	82573000	82573001	MACS_peak_3721	10.00	TCF7L2_standard_ChIP_seq_CW34
chr6	84569179	84569180	MACS_peak_3722	8.00	TCF7L2_standard_ChIP_seq_CW34
chr6	84805144	84805145	MACS_peak_3723	11.00	TCF7L2_standard_ChIP_seq_CW34
chr6	86115066	86115067	MACS_peak_3724	7.00	TCF7L2_standard_ChIP_seq_CW34
chr6	86303568	86303569	MACS_peak_3725	6.00	TCF7L2_standard_ChIP_seq_CW34
chr6	88381661	88381662	MACS_peak_3726	36.00	TCF7L2_standard_ChIP_seq_CW34
chr6	88495099	88495100	MACS_peak_3727	11.00	TCF7L2_standard_ChIP_seq_CW34
chr6	88508691	88508692	MACS_peak_3728	52.00	TCF7L2_standard_ChIP_seq_CW34
chr6	89539556	89539557	MACS_peak_3729	9.00	TCF7L2_standard_ChIP_seq_CW34
chr6	90218896	90218897	MACS_peak_3730	7.00	TCF7L2_standard_ChIP_seq_CW34
chr6	91296694	91296695	MACS_peak_3731	8.00	TCF7L2_standard_ChIP_seq_CW34
chr6	96253134	96253135	MACS_peak_3732	37.00	TCF7L2_standard_ChIP_seq_CW34
chr6	96502073	96502074	MACS_peak_3733	6.00	TCF7L2_standard_ChIP_seq_CW34
chr6	101396893	101396894	MACS_peak_3734	9.00	TCF7L2_standard_ChIP_seq_CW34
chr6	105381568	105381569	MACS_peak_3735	7.00	TCF7L2_standard_ChIP_seq_CW34
chr6	105388926	105388927	MACS_peak_3736	6.00	TCF7L2_standard_ChIP_seq_CW34
chr6	105514264	105514265	MACS_peak_3737	8.00	TCF7L2_standard_ChIP_seq_CW34
chr6	106709801	106709802	MACS_peak_3738	19.00	TCF7L2_standard_ChIP_seq_CW34
chr6	106911225	106911226	MACS_peak_3739	13.00	TCF7L2_standard_ChIP_seq_CW34
chr6	107070391	107070392	MACS_peak_3740	8.00	TCF7L2_standard_ChIP_seq_CW34
chr6	108181582	108181583	MACS_peak_3741	14.00	TCF7L2_standard_ChIP_seq_CW34
chr6	108274016	108274017	MACS_peak_3742	13.00	TCF7L2_standard_ChIP_seq_CW34
chr6	108323120	108323121	MACS_peak_3743	8.00	TCF7L2_standard_ChIP_seq_CW34
chr6	108517869	108517870	MACS_peak_3744	7.00	TCF7L2_standard_ChIP_seq_CW34
chr6	109225225	109225226	MACS_peak_3745	19.00	TCF7L2_standard_ChIP_seq_CW34
chr6	109371733	109371734	MACS_peak_3746	11.00	TCF7L2_standard_ChIP_seq_CW34
chr6	111426991	111426992	MACS_peak_3747	9.00	TCF7L2_standard_ChIP_seq_CW34
chr6	111804982	111804983	MACS_peak_3748	8.00	TCF7L2_standard_ChIP_seq_CW34
chr6	113673576	113673577	MACS_peak_3749	29.00	TCF7L2_standard_ChIP_seq_CW34
chr6	113964073	113964074	MACS_peak_3750	35.00	TCF7L2_standard_ChIP_seq_CW34
chr6	114466237	114466238	MACS_peak_3751	5.00	TCF7L2_standard_ChIP_seq_CW34
chr6	114489044	114489045	MACS_peak_3752	16.00	TCF7L2_standard_ChIP_seq_CW34
chr6	116258872	116258873	MACS_peak_3753	14.00	TCF7L2_standard_ChIP_seq_CW34
chr6	117245559	117245560	MACS_peak_3754	10.00	TCF7L2_standard_ChIP_seq_CW34
chr6	117763192	117763193	MACS_peak_3755	18.00	TCF7L2_standard_ChIP_seq_CW34
chr6	119117856	119117857	MACS_peak_3756	8.00	TCF7L2_standard_ChIP_seq_CW34
chr6	119491753	119491754	MACS_peak_3757	31.00	TCF7L2_standard_ChIP_seq_CW34
chr6	119558631	119558632	MACS_peak_3758	27.00	TCF7L2_standard_ChIP_seq_CW34
chr6	119582553	119582554	MACS_peak_3759	33.00	TCF7L2_standard_ChIP_seq_CW34
chr6	119669652	119669653	MACS_peak_3760	20.00	TCF7L2_standard_ChIP_seq_CW34
chr6	121915390	121915391	MACS_peak_3761	7.00	TCF7L2_standard_ChIP_seq_CW34
chr6	121974920	121974921	MACS_peak_3762	6.00	TCF7L2_standard_ChIP_seq_CW34
chr6	123265878	123265879	MACS_peak_3763	6.00	TCF7L2_standard_ChIP_seq_CW34
chr6	123378165	123378166	MACS_peak_3764	35.00	TCF7L2_standard_ChIP_seq_CW34
chr6	126277964	126277965	MACS_peak_3765	77.00	TCF7L2_standard_ChIP_seq_CW34
chr6	127344255	127344256	MACS_peak_3766	9.00	TCF7L2_standard_ChIP_seq_CW34
chr6	127877048	127877049	MACS_peak_3767	7.00	TCF7L2_standard_ChIP_seq_CW34
chr6	128953451	128953452	MACS_peak_3768	8.00	TCF7L2_standard_ChIP_seq_CW34
chr6	130017099	130017100	MACS_peak_3769	20.00	TCF7L2_standard_ChIP_seq_CW34
chr6	131136592	131136593	MACS_peak_3770	9.00	TCF7L2_standard_ChIP_seq_CW34
chr6	131174860	131174861	MACS_peak_3771	7.00	TCF7L2_standard_ChIP_seq_CW34
chr6	131184795	131184796	MACS_peak_3772	13.00	TCF7L2_standard_ChIP_seq_CW34
chr6	131360340	131360341	MACS_peak_3773	8.00	TCF7L2_standard_ChIP_seq_CW34
chr6	131860749	131860750	MACS_peak_3774	8.00	TCF7L2_standard_ChIP_seq_CW34
chr6	132090942	132090943	MACS_peak_3775	15.00	TCF7L2_standard_ChIP_seq_CW34
chr6	132157882	132157883	MACS_peak_3776	10.00	TCF7L2_standard_ChIP_seq_CW34
chr6	132315335	132315336	MACS_peak_3777	7.00	TCF7L2_standard_ChIP_seq_CW34
chr6	132855855	132855856	MACS_peak_3778	12.00	TCF7L2_standard_ChIP_seq_CW34
chr6	133056026	133056027	MACS_peak_3779	13.00	TCF7L2_standard_ChIP_seq_CW34
chr6	133355969	133355970	MACS_peak_3780	27.00	TCF7L2_standard_ChIP_seq_CW34
chr6	133372543	133372544	MACS_peak_3781	5.00	TCF7L2_standard_ChIP_seq_CW34
chr6	133789821	133789822	MACS_peak_3782	6.00	TCF7L2_standard_ChIP_seq_CW34
chr6	134519382	134519383	MACS_peak_3783	16.00	TCF7L2_standard_ChIP_seq_CW34
chr6	134521204	134521205	MACS_peak_3784	20.00	TCF7L2_standard_ChIP_seq_CW34
chr6	134851188	134851189	MACS_peak_3785	15.00	TCF7L2_standard_ChIP_seq_CW34
chr6	137209141	137209142	MACS_peak_3786	11.00	TCF7L2_standard_ChIP_seq_CW34
chr6	137470081	137470082	MACS_peak_3787	8.00	TCF7L2_standard_ChIP_seq_CW34
chr6	137563016	137563017	MACS_peak_3788	14.00	TCF7L2_standard_ChIP_seq_CW34
chr6	137636346	137636347	MACS_peak_3789	9.00	TCF7L2_standard_ChIP_seq_CW34
chr6	137675354	137675355	MACS_peak_3790	33.00	TCF7L2_standard_ChIP_seq_CW34
chr6	137711747	137711748	MACS_peak_3791	7.00	TCF7L2_standard_ChIP_seq_CW34
chr6	138028895	138028896	MACS_peak_3792	28.00	TCF7L2_standard_ChIP_seq_CW34
chr6	138053338	138053339	MACS_peak_3793	32.00	TCF7L2_standard_ChIP_seq_CW34
chr6	138116224	138116225	MACS_peak_3794	22.00	TCF7L2_standard_ChIP_seq_CW34
chr6	138703383	138703384	MACS_peak_3795	7.00	TCF7L2_standard_ChIP_seq_CW34
chr6	138725296	138725297	MACS_peak_3796	9.00	TCF7L2_standard_ChIP_seq_CW34
chr6	138825373	138825374	MACS_peak_3797	6.00	TCF7L2_standard_ChIP_seq_CW34
chr6	138864983	138864984	MACS_peak_3798	18.00	TCF7L2_standard_ChIP_seq_CW34
chr6	141347878	141347879	MACS_peak_3799	9.00	TCF7L2_standard_ChIP_seq_CW34
chr6	142629187	142629188	MACS_peak_3800	20.00	TCF7L2_standard_ChIP_seq_CW34
chr6	142652416	142652417	MACS_peak_3801	18.00	TCF7L2_standard_ChIP_seq_CW34
chr6	143167197	143167198	MACS_peak_3802	33.00	TCF7L2_standard_ChIP_seq_CW34
chr6	143189240	143189241	MACS_peak_3803	8.00	TCF7L2_standard_ChIP_seq_CW34
chr6	143247975	143247976	MACS_peak_3804	11.00	TCF7L2_standard_ChIP_seq_CW34
chr6	143999251	143999252	MACS_peak_3805	11.00	TCF7L2_standard_ChIP_seq_CW34
chr6	144066986	144066987	MACS_peak_3806	11.00	TCF7L2_standard_ChIP_seq_CW34
chr6	144121275	144121276	MACS_peak_3807	9.00	TCF7L2_standard_ChIP_seq_CW34
chr6	144176337	144176338	MACS_peak_3808	11.00	TCF7L2_standard_ChIP_seq_CW34
chr6	144294333	144294334	MACS_peak_3809	10.00	TCF7L2_standard_ChIP_seq_CW34
chr6	144624138	144624139	MACS_peak_3810	12.00	TCF7L2_standard_ChIP_seq_CW34
chr6	144661965	144661966	MACS_peak_3811	13.00	TCF7L2_standard_ChIP_seq_CW34
chr6	144664813	144664814	MACS_peak_3812	10.00	TCF7L2_standard_ChIP_seq_CW34
chr6	145435774	145435775	MACS_peak_3813	8.00	TCF7L2_standard_ChIP_seq_CW34
chr6	145588388	145588389	MACS_peak_3814	12.00	TCF7L2_standard_ChIP_seq_CW34
chr6	145616376	145616377	MACS_peak_3815	7.00	TCF7L2_standard_ChIP_seq_CW34
chr6	146056328	146056329	MACS_peak_3816	8.00	TCF7L2_standard_ChIP_seq_CW34
chr6	147208759	147208760	MACS_peak_3817	7.00	TCF7L2_standard_ChIP_seq_CW34
chr6	147306418	147306419	MACS_peak_3818	27.00	TCF7L2_standard_ChIP_seq_CW34
chr6	149300779	149300780	MACS_peak_3819	26.00	TCF7L2_standard_ChIP_seq_CW34
chr6	149396113	149396114	MACS_peak_3820	9.00	TCF7L2_standard_ChIP_seq_CW34
chr6	149498395	149498396	MACS_peak_3821	9.00	TCF7L2_standard_ChIP_seq_CW34
chr6	151417523	151417524	MACS_peak_3822	16.00	TCF7L2_standard_ChIP_seq_CW34
chr6	151441377	151441378	MACS_peak_3823	17.00	TCF7L2_standard_ChIP_seq_CW34
chr6	151644241	151644242	MACS_peak_3824	14.00	TCF7L2_standard_ChIP_seq_CW34
chr6	151823097	151823098	MACS_peak_3825	12.00	TCF7L2_standard_ChIP_seq_CW34
chr6	152283735	152283736	MACS_peak_3826	10.00	TCF7L2_standard_ChIP_seq_CW34
chr6	152457439	152457440	MACS_peak_3827	17.00	TCF7L2_standard_ChIP_seq_CW34
chr6	152623115	152623116	MACS_peak_3828	10.00	TCF7L2_standard_ChIP_seq_CW34
chr6	155650026	155650027	MACS_peak_3829	78.00	TCF7L2_standard_ChIP_seq_CW34
chr6	155720554	155720555	MACS_peak_3830	27.00	TCF7L2_standard_ChIP_seq_CW34
chr6	155765651	155765652	MACS_peak_3831	10.00	TCF7L2_standard_ChIP_seq_CW34
chr6	155808115	155808116	MACS_peak_3832	48.00	TCF7L2_standard_ChIP_seq_CW34
chr6	156582142	156582143	MACS_peak_3833	8.00	TCF7L2_standard_ChIP_seq_CW34
chr6	157041093	157041094	MACS_peak_3834	11.00	TCF7L2_standard_ChIP_seq_CW34
chr6	157074089	157074090	MACS_peak_3835	10.00	TCF7L2_standard_ChIP_seq_CW34
chr6	157099888	157099889	MACS_peak_3836	11.00	TCF7L2_standard_ChIP_seq_CW34
chr6	158148738	158148739	MACS_peak_3837	10.00	TCF7L2_standard_ChIP_seq_CW34
chr6	158402642	158402643	MACS_peak_3838	9.00	TCF7L2_standard_ChIP_seq_CW34
chr6	158414657	158414658	MACS_peak_3839	13.00	TCF7L2_standard_ChIP_seq_CW34
chr6	158433122	158433123	MACS_peak_3840	7.00	TCF7L2_standard_ChIP_seq_CW34
chr6	158663641	158663642	MACS_peak_3841	6.00	TCF7L2_standard_ChIP_seq_CW34
chr6	158717501	158717502	MACS_peak_3842	10.00	TCF7L2_standard_ChIP_seq_CW34
chr6	158724502	158724503	MACS_peak_3843	9.00	TCF7L2_standard_ChIP_seq_CW34
chr6	158743893	158743894	MACS_peak_3844	11.00	TCF7L2_standard_ChIP_seq_CW34
chr6	160114571	160114572	MACS_peak_3845	12.00	TCF7L2_standard_ChIP_seq_CW34
chr6	160418937	160418938	MACS_peak_3846	8.00	TCF7L2_standard_ChIP_seq_CW34
chr6	160535730	160535731	MACS_peak_3847	7.00	TCF7L2_standard_ChIP_seq_CW34
chr6	160770276	160770277	MACS_peak_3848	12.00	TCF7L2_standard_ChIP_seq_CW34
chr6	162671452	162671453	MACS_peak_3849	17.00	TCF7L2_standard_ChIP_seq_CW34
chr6	167655209	167655210	MACS_peak_3850	8.00	TCF7L2_standard_ChIP_seq_CW34
chr6	167755829	167755830	MACS_peak_3851	27.00	TCF7L2_standard_ChIP_seq_CW34
chr6	168069116	168069117	MACS_peak_3852	6.00	TCF7L2_standard_ChIP_seq_CW34
chr6	168134862	168134863	MACS_peak_3853	20.00	TCF7L2_standard_ChIP_seq_CW34
chr6	168136326	168136327	MACS_peak_3854	36.00	TCF7L2_standard_ChIP_seq_CW34
chr6	168595177	168595178	MACS_peak_3855	9.00	TCF7L2_standard_ChIP_seq_CW34
chr6	168702523	168702524	MACS_peak_3856	38.00	TCF7L2_standard_ChIP_seq_CW34
chr6	168727263	168727264	MACS_peak_3857	17.00	TCF7L2_standard_ChIP_seq_CW34
chr6	170151880	170151881	MACS_peak_3858	11.00	TCF7L2_standard_ChIP_seq_CW34
chr6	170707982	170707983	MACS_peak_3859	12.00	TCF7L2_standard_ChIP_seq_CW34
chr6	170709274	170709275	MACS_peak_3860	12.00	TCF7L2_standard_ChIP_seq_CW34
chr7	413242	413243	MACS_peak_3861	11.00	TCF7L2_standard_ChIP_seq_CW34
chr7	658295	658296	MACS_peak_3862	10.00	TCF7L2_standard_ChIP_seq_CW34
chr7	1196979	1196980	MACS_peak_3863	61.00	TCF7L2_standard_ChIP_seq_CW34
chr7	1753651	1753652	MACS_peak_3864	14.00	TCF7L2_standard_ChIP_seq_CW34
chr7	1843486	1843487	MACS_peak_3865	29.00	TCF7L2_standard_ChIP_seq_CW34
chr7	1915656	1915657	MACS_peak_3866	10.00	TCF7L2_standard_ChIP_seq_CW34
chr7	2067435	2067436	MACS_peak_3867	7.00	TCF7L2_standard_ChIP_seq_CW34
chr7	2394407	2394408	MACS_peak_3868	10.00	TCF7L2_standard_ChIP_seq_CW34
chr7	2594969	2594970	MACS_peak_3869	8.00	TCF7L2_standard_ChIP_seq_CW34
chr7	2844188	2844189	MACS_peak_3870	11.00	TCF7L2_standard_ChIP_seq_CW34
chr7	3340647	3340648	MACS_peak_3871	11.00	TCF7L2_standard_ChIP_seq_CW34
chr7	3635314	3635315	MACS_peak_3872	8.00	TCF7L2_standard_ChIP_seq_CW34
chr7	3954260	3954261	MACS_peak_3873	12.00	TCF7L2_standard_ChIP_seq_CW34
chr7	4365532	4365533	MACS_peak_3874	8.00	TCF7L2_standard_ChIP_seq_CW34
chr7	4671509	4671510	MACS_peak_3875	9.00	TCF7L2_standard_ChIP_seq_CW34
chr7	5624794	5624795	MACS_peak_3876	11.00	TCF7L2_standard_ChIP_seq_CW34
chr7	5633877	5633878	MACS_peak_3877	19.00	TCF7L2_standard_ChIP_seq_CW34
chr7	6117048	6117049	MACS_peak_3878	18.00	TCF7L2_standard_ChIP_seq_CW34
chr7	7013373	7013374	MACS_peak_3879	6.00	TCF7L2_standard_ChIP_seq_CW34
chr7	7158575	7158576	MACS_peak_3880	20.00	TCF7L2_standard_ChIP_seq_CW34
chr7	7216937	7216938	MACS_peak_3881	26.00	TCF7L2_standard_ChIP_seq_CW34
chr7	7239438	7239439	MACS_peak_3882	12.00	TCF7L2_standard_ChIP_seq_CW34
chr7	7539741	7539742	MACS_peak_3883	6.00	TCF7L2_standard_ChIP_seq_CW34
chr7	7699736	7699737	MACS_peak_3884	8.00	TCF7L2_standard_ChIP_seq_CW34
chr7	10711227	10711228	MACS_peak_3885	6.00	TCF7L2_standard_ChIP_seq_CW34
chr7	12680075	12680076	MACS_peak_3886	20.00	TCF7L2_standard_ChIP_seq_CW34
chr7	13057201	13057202	MACS_peak_3887	36.00	TCF7L2_standard_ChIP_seq_CW34
chr7	16685618	16685619	MACS_peak_3888	9.00	TCF7L2_standard_ChIP_seq_CW34
chr7	16998639	16998640	MACS_peak_3889	14.00	TCF7L2_standard_ChIP_seq_CW34
chr7	17029768	17029769	MACS_peak_3890	28.00	TCF7L2_standard_ChIP_seq_CW34
chr7	17056154	17056155	MACS_peak_3891	8.00	TCF7L2_standard_ChIP_seq_CW34
chr7	17139247	17139248	MACS_peak_3892	16.00	TCF7L2_standard_ChIP_seq_CW34
chr7	17170129	17170130	MACS_peak_3893	14.00	TCF7L2_standard_ChIP_seq_CW34
chr7	17182850	17182851	MACS_peak_3894	7.00	TCF7L2_standard_ChIP_seq_CW34
chr7	17224629	17224630	MACS_peak_3895	11.00	TCF7L2_standard_ChIP_seq_CW34
chr7	17545946	17545947	MACS_peak_3896	8.00	TCF7L2_standard_ChIP_seq_CW34
chr7	20826558	20826559	MACS_peak_3897	35.00	TCF7L2_standard_ChIP_seq_CW34
chr7	22473744	22473745	MACS_peak_3898	11.00	TCF7L2_standard_ChIP_seq_CW34
chr7	23374366	23374367	MACS_peak_3899	13.00	TCF7L2_standard_ChIP_seq_CW34
chr7	23396917	23396918	MACS_peak_3900	8.00	TCF7L2_standard_ChIP_seq_CW34
chr7	24941088	24941089	MACS_peak_3901	10.00	TCF7L2_standard_ChIP_seq_CW34
chr7	25992434	25992435	MACS_peak_3902	11.00	TCF7L2_standard_ChIP_seq_CW34
chr7	26041445	26041446	MACS_peak_3903	25.00	TCF7L2_standard_ChIP_seq_CW34
chr7	26989057	26989058	MACS_peak_3904	23.00	TCF7L2_standard_ChIP_seq_CW34
chr7	27161971	27161972	MACS_peak_3905	8.00	TCF7L2_standard_ChIP_seq_CW34
chr7	29180712	29180713	MACS_peak_3906	14.00	TCF7L2_standard_ChIP_seq_CW34
chr7	29230147	29230148	MACS_peak_3907	20.00	TCF7L2_standard_ChIP_seq_CW34
chr7	29235308	29235309	MACS_peak_3908	7.00	TCF7L2_standard_ChIP_seq_CW34
chr7	29268336	29268337	MACS_peak_3909	7.00	TCF7L2_standard_ChIP_seq_CW34
chr7	29523085	29523086	MACS_peak_3910	7.00	TCF7L2_standard_ChIP_seq_CW34
chr7	29724709	29724710	MACS_peak_3911	7.00	TCF7L2_standard_ChIP_seq_CW34
chr7	30068002	30068003	MACS_peak_3912	8.00	TCF7L2_standard_ChIP_seq_CW34
chr7	30294984	30294985	MACS_peak_3913	17.00	TCF7L2_standard_ChIP_seq_CW34
chr7	32708107	32708108	MACS_peak_3914	18.00	TCF7L2_standard_ChIP_seq_CW34
chr7	32931125	32931126	MACS_peak_3915	14.00	TCF7L2_standard_ChIP_seq_CW34
chr7	32997173	32997174	MACS_peak_3916	6.00	TCF7L2_standard_ChIP_seq_CW34
chr7	33255680	33255681	MACS_peak_3917	25.00	TCF7L2_standard_ChIP_seq_CW34
chr7	34075785	34075786	MACS_peak_3918	14.00	TCF7L2_standard_ChIP_seq_CW34
chr7	34465773	34465774	MACS_peak_3919	6.00	TCF7L2_standard_ChIP_seq_CW34
chr7	34617771	34617772	MACS_peak_3920	15.00	TCF7L2_standard_ChIP_seq_CW34
chr7	35841191	35841192	MACS_peak_3921	7.00	TCF7L2_standard_ChIP_seq_CW34
chr7	36204908	36204909	MACS_peak_3922	7.00	TCF7L2_standard_ChIP_seq_CW34
chr7	37011254	37011255	MACS_peak_3923	35.00	TCF7L2_standard_ChIP_seq_CW34
chr7	37960610	37960611	MACS_peak_3924	10.00	TCF7L2_standard_ChIP_seq_CW34
chr7	37982869	37982870	MACS_peak_3925	33.00	TCF7L2_standard_ChIP_seq_CW34
chr7	40443449	40443450	MACS_peak_3926	10.00	TCF7L2_standard_ChIP_seq_CW34
chr7	40509916	40509917	MACS_peak_3927	10.00	TCF7L2_standard_ChIP_seq_CW34
chr7	40772936	40772937	MACS_peak_3928	8.00	TCF7L2_standard_ChIP_seq_CW34
chr7	40795236	40795237	MACS_peak_3929	8.00	TCF7L2_standard_ChIP_seq_CW34
chr7	41356197	41356198	MACS_peak_3930	16.00	TCF7L2_standard_ChIP_seq_CW34
chr7	41547653	41547654	MACS_peak_3931	11.00	TCF7L2_standard_ChIP_seq_CW34
chr7	43022295	43022296	MACS_peak_3932	8.00	TCF7L2_standard_ChIP_seq_CW34
chr7	43526796	43526797	MACS_peak_3933	9.00	TCF7L2_standard_ChIP_seq_CW34
chr7	43623021	43623022	MACS_peak_3934	6.00	TCF7L2_standard_ChIP_seq_CW34
chr7	43821898	43821899	MACS_peak_3935	8.00	TCF7L2_standard_ChIP_seq_CW34
chr7	44646639	44646640	MACS_peak_3936	8.00	TCF7L2_standard_ChIP_seq_CW34
chr7	44925181	44925182	MACS_peak_3937	7.00	TCF7L2_standard_ChIP_seq_CW34
chr7	45374732	45374733	MACS_peak_3938	9.00	TCF7L2_standard_ChIP_seq_CW34
chr7	45921717	45921718	MACS_peak_3939	48.00	TCF7L2_standard_ChIP_seq_CW34
chr7	47332661	47332662	MACS_peak_3940	18.00	TCF7L2_standard_ChIP_seq_CW34
chr7	47510795	47510796	MACS_peak_3941	18.00	TCF7L2_standard_ChIP_seq_CW34
chr7	47616095	47616096	MACS_peak_3942	13.00	TCF7L2_standard_ChIP_seq_CW34
chr7	47803339	47803340	MACS_peak_3943	11.00	TCF7L2_standard_ChIP_seq_CW34
chr7	50682964	50682965	MACS_peak_3944	6.00	TCF7L2_standard_ChIP_seq_CW34
chr7	63349394	63349395	MACS_peak_3952	5.00	TCF7L2_standard_ChIP_seq_CW34
chr7	64460063	64460064	MACS_peak_3953	25.00	TCF7L2_standard_ChIP_seq_CW34
chr7	64910182	64910183	MACS_peak_3954	8.00	TCF7L2_standard_ChIP_seq_CW34
chr7	65591241	65591242	MACS_peak_3955	11.00	TCF7L2_standard_ChIP_seq_CW34
chr7	72149059	72149060	MACS_peak_3956	6.00	TCF7L2_standard_ChIP_seq_CW34
chr7	73037638	73037639	MACS_peak_3957	13.00	TCF7L2_standard_ChIP_seq_CW34
chr7	73868064	73868065	MACS_peak_3958	7.00	TCF7L2_standard_ChIP_seq_CW34
chr7	74024840	74024841	MACS_peak_3959	38.00	TCF7L2_standard_ChIP_seq_CW34
chr7	74072544	74072545	MACS_peak_3960	8.00	TCF7L2_standard_ChIP_seq_CW34
chr7	75544402	75544403	MACS_peak_3961	8.00	TCF7L2_standard_ChIP_seq_CW34
chr7	75549925	75549926	MACS_peak_3962	13.00	TCF7L2_standard_ChIP_seq_CW34
chr7	75810258	75810259	MACS_peak_3963	7.00	TCF7L2_standard_ChIP_seq_CW34
chr7	75878845	75878846	MACS_peak_3964	12.00	TCF7L2_standard_ChIP_seq_CW34
chr7	77038965	77038966	MACS_peak_3965	8.00	TCF7L2_standard_ChIP_seq_CW34
chr7	79839870	79839871	MACS_peak_3966	18.00	TCF7L2_standard_ChIP_seq_CW34
chr7	80272325	80272326	MACS_peak_3967	8.00	TCF7L2_standard_ChIP_seq_CW34
chr7	85221447	85221448	MACS_peak_3968	8.00	TCF7L2_standard_ChIP_seq_CW34
chr7	87127088	87127089	MACS_peak_3969	10.00	TCF7L2_standard_ChIP_seq_CW34
chr7	87178021	87178022	MACS_peak_3970	28.00	TCF7L2_standard_ChIP_seq_CW34
chr7	89809801	89809802	MACS_peak_3971	8.00	TCF7L2_standard_ChIP_seq_CW34
chr7	89839988	89839989	MACS_peak_3972	10.00	TCF7L2_standard_ChIP_seq_CW34
chr7	90388668	90388669	MACS_peak_3973	11.00	TCF7L2_standard_ChIP_seq_CW34
chr7	92072224	92072225	MACS_peak_3974	7.00	TCF7L2_standard_ChIP_seq_CW34
chr7	92307790	92307791	MACS_peak_3975	35.00	TCF7L2_standard_ChIP_seq_CW34
chr7	92325111	92325112	MACS_peak_3976	37.00	TCF7L2_standard_ChIP_seq_CW34
chr7	92464967	92464968	MACS_peak_3977	33.00	TCF7L2_standard_ChIP_seq_CW34
chr7	93764141	93764142	MACS_peak_3978	7.00	TCF7L2_standard_ChIP_seq_CW34
chr7	94191403	94191404	MACS_peak_3979	11.00	TCF7L2_standard_ChIP_seq_CW34
chr7	94253396	94253397	MACS_peak_3980	7.00	TCF7L2_standard_ChIP_seq_CW34
chr7	94272718	94272719	MACS_peak_3981	12.00	TCF7L2_standard_ChIP_seq_CW34
chr7	94610269	94610270	MACS_peak_3982	11.00	TCF7L2_standard_ChIP_seq_CW34
chr7	95069918	95069919	MACS_peak_3983	25.00	TCF7L2_standard_ChIP_seq_CW34
chr7	95099954	95099955	MACS_peak_3984	7.00	TCF7L2_standard_ChIP_seq_CW34
chr7	95932668	95932669	MACS_peak_3985	16.00	TCF7L2_standard_ChIP_seq_CW34
chr7	96045032	96045033	MACS_peak_3986	16.00	TCF7L2_standard_ChIP_seq_CW34
chr7	96328958	96328959	MACS_peak_3987	14.00	TCF7L2_standard_ChIP_seq_CW34
chr7	96430325	96430326	MACS_peak_3988	9.00	TCF7L2_standard_ChIP_seq_CW34
chr7	96463135	96463136	MACS_peak_3989	10.00	TCF7L2_standard_ChIP_seq_CW34
chr7	96600179	96600180	MACS_peak_3990	33.00	TCF7L2_standard_ChIP_seq_CW34
chr7	96646281	96646282	MACS_peak_3991	25.00	TCF7L2_standard_ChIP_seq_CW34
chr7	98047013	98047014	MACS_peak_3992	24.00	TCF7L2_standard_ChIP_seq_CW34
chr7	98768560	98768561	MACS_peak_3993	12.00	TCF7L2_standard_ChIP_seq_CW34
chr7	100033523	100033524	MACS_peak_3994	8.00	TCF7L2_standard_ChIP_seq_CW34
chr7	100091388	100091389	MACS_peak_3995	9.00	TCF7L2_standard_ChIP_seq_CW34
chr7	100137858	100137859	MACS_peak_3996	9.00	TCF7L2_standard_ChIP_seq_CW34
chr7	100747584	100747585	MACS_peak_3997	8.00	TCF7L2_standard_ChIP_seq_CW34
chr7	101228359	101228360	MACS_peak_3998	7.00	TCF7L2_standard_ChIP_seq_CW34
chr7	101288336	101288337	MACS_peak_3999	7.00	TCF7L2_standard_ChIP_seq_CW34
chr7	101337011	101337012	MACS_peak_4000	7.00	TCF7L2_standard_ChIP_seq_CW34
chr7	101765130	101765131	MACS_peak_4001	6.00	TCF7L2_standard_ChIP_seq_CW34
chr7	102747069	102747070	MACS_peak_4002	6.00	TCF7L2_standard_ChIP_seq_CW34
chr7	102932499	102932500	MACS_peak_4003	7.00	TCF7L2_standard_ChIP_seq_CW34
chr7	103029358	103029359	MACS_peak_4004	18.00	TCF7L2_standard_ChIP_seq_CW34
chr7	103301192	103301193	MACS_peak_4005	11.00	TCF7L2_standard_ChIP_seq_CW34
chr7	103677482	103677483	MACS_peak_4006	16.00	TCF7L2_standard_ChIP_seq_CW34
chr7	103705594	103705595	MACS_peak_4007	23.00	TCF7L2_standard_ChIP_seq_CW34
chr7	104584185	104584186	MACS_peak_4008	7.00	TCF7L2_standard_ChIP_seq_CW34
chr7	105321207	105321208	MACS_peak_4009	8.00	TCF7L2_standard_ChIP_seq_CW34
chr7	105752506	105752507	MACS_peak_4010	9.00	TCF7L2_standard_ChIP_seq_CW34
chr7	107420203	107420204	MACS_peak_4011	16.00	TCF7L2_standard_ChIP_seq_CW34
chr7	107589592	107589593	MACS_peak_4012	10.00	TCF7L2_standard_ChIP_seq_CW34
chr7	107696172	107696173	MACS_peak_4013	14.00	TCF7L2_standard_ChIP_seq_CW34
chr7	109717568	109717569	MACS_peak_4014	6.00	TCF7L2_standard_ChIP_seq_CW34
chr7	110832829	110832830	MACS_peak_4015	8.00	TCF7L2_standard_ChIP_seq_CW34
chr7	115813739	115813740	MACS_peak_4016	8.00	TCF7L2_standard_ChIP_seq_CW34
chr7	116450938	116450939	MACS_peak_4017	6.00	TCF7L2_standard_ChIP_seq_CW34
chr7	117578313	117578314	MACS_peak_4018	6.00	TCF7L2_standard_ChIP_seq_CW34
chr7	122392794	122392795	MACS_peak_4019	9.00	TCF7L2_standard_ChIP_seq_CW34
chr7	127938316	127938317	MACS_peak_4020	8.00	TCF7L2_standard_ChIP_seq_CW34
chr7	127950746	127950747	MACS_peak_4021	10.00	TCF7L2_standard_ChIP_seq_CW34
chr7	128510169	128510170	MACS_peak_4022	13.00	TCF7L2_standard_ChIP_seq_CW34
chr7	129426787	129426788	MACS_peak_4023	17.00	TCF7L2_standard_ChIP_seq_CW34
chr7	129588946	129588947	MACS_peak_4024	7.00	TCF7L2_standard_ChIP_seq_CW34
chr7	131903765	131903766	MACS_peak_4025	21.00	TCF7L2_standard_ChIP_seq_CW34
chr7	133012957	133012958	MACS_peak_4026	29.00	TCF7L2_standard_ChIP_seq_CW34
chr7	133500674	133500675	MACS_peak_4027	6.00	TCF7L2_standard_ChIP_seq_CW34
chr7	134001511	134001512	MACS_peak_4028	8.00	TCF7L2_standard_ChIP_seq_CW34
chr7	134586173	134586174	MACS_peak_4029	5.00	TCF7L2_standard_ChIP_seq_CW34
chr7	137380907	137380908	MACS_peak_4030	13.00	TCF7L2_standard_ChIP_seq_CW34
chr7	137828985	137828986	MACS_peak_4031	8.00	TCF7L2_standard_ChIP_seq_CW34
chr7	137942590	137942591	MACS_peak_4032	14.00	TCF7L2_standard_ChIP_seq_CW34
chr7	138037397	138037398	MACS_peak_4033	25.00	TCF7L2_standard_ChIP_seq_CW34
chr7	138073800	138073801	MACS_peak_4034	18.00	TCF7L2_standard_ChIP_seq_CW34
chr7	138081329	138081330	MACS_peak_4035	61.00	TCF7L2_standard_ChIP_seq_CW34
chr7	138720673	138720674	MACS_peak_4036	6.00	TCF7L2_standard_ChIP_seq_CW34
chr7	139749181	139749182	MACS_peak_4037	7.00	TCF7L2_standard_ChIP_seq_CW34
chr7	139875753	139875754	MACS_peak_4038	8.00	TCF7L2_standard_ChIP_seq_CW34
chr7	139929496	139929497	MACS_peak_4039	73.00	TCF7L2_standard_ChIP_seq_CW34
chr7	140244539	140244540	MACS_peak_4040	15.00	TCF7L2_standard_ChIP_seq_CW34
chr7	140676020	140676021	MACS_peak_4041	28.00	TCF7L2_standard_ChIP_seq_CW34
chr7	141251203	141251204	MACS_peak_4042	7.00	TCF7L2_standard_ChIP_seq_CW34
chr7	142740108	142740109	MACS_peak_4043	10.00	TCF7L2_standard_ChIP_seq_CW34
chr7	143579400	143579401	MACS_peak_4044	8.00	TCF7L2_standard_ChIP_seq_CW34
chr7	143604202	143604203	MACS_peak_4045	42.00	TCF7L2_standard_ChIP_seq_CW34
chr7	145406711	145406712	MACS_peak_4046	5.00	TCF7L2_standard_ChIP_seq_CW34
chr7	148125835	148125836	MACS_peak_4047	62.00	TCF7L2_standard_ChIP_seq_CW34
chr7	148160101	148160102	MACS_peak_4048	8.00	TCF7L2_standard_ChIP_seq_CW34
chr7	148580985	148580986	MACS_peak_4049	7.00	TCF7L2_standard_ChIP_seq_CW34
chr7	148725931	148725932	MACS_peak_4050	11.00	TCF7L2_standard_ChIP_seq_CW34
chr7	149109559	149109560	MACS_peak_4051	13.00	TCF7L2_standard_ChIP_seq_CW34
chr7	149389827	149389828	MACS_peak_4052	8.00	TCF7L2_standard_ChIP_seq_CW34
chr7	149437917	149437918	MACS_peak_4053	6.00	TCF7L2_standard_ChIP_seq_CW34
chr7	150065510	150065511	MACS_peak_4054	37.00	TCF7L2_standard_ChIP_seq_CW34
chr7	150076261	150076262	MACS_peak_4055	26.00	TCF7L2_standard_ChIP_seq_CW34
chr7	150755307	150755308	MACS_peak_4056	28.00	TCF7L2_standard_ChIP_seq_CW34
chr7	151778469	151778470	MACS_peak_4057	6.00	TCF7L2_standard_ChIP_seq_CW34
chr7	154794989	154794990	MACS_peak_4058	12.00	TCF7L2_standard_ChIP_seq_CW34
chr7	154950521	154950522	MACS_peak_4059	6.00	TCF7L2_standard_ChIP_seq_CW34
chr7	155475333	155475334	MACS_peak_4060	8.00	TCF7L2_standard_ChIP_seq_CW34
chr7	156407739	156407740	MACS_peak_4061	14.00	TCF7L2_standard_ChIP_seq_CW34
chr7	156459519	156459520	MACS_peak_4062	26.00	TCF7L2_standard_ChIP_seq_CW34
chr7	156781461	156781462	MACS_peak_4063	16.00	TCF7L2_standard_ChIP_seq_CW34
chr7	156792537	156792538	MACS_peak_4064	8.00	TCF7L2_standard_ChIP_seq_CW34
chr7	156804509	156804510	MACS_peak_4065	11.00	TCF7L2_standard_ChIP_seq_CW34
chr7	157109498	157109499	MACS_peak_4066	8.00	TCF7L2_standard_ChIP_seq_CW34
chr7	157819982	157819983	MACS_peak_4067	7.00	TCF7L2_standard_ChIP_seq_CW34
chr7	158317088	158317089	MACS_peak_4068	6.00	TCF7L2_standard_ChIP_seq_CW34
chr8	980333	980334	MACS_peak_4069	25.00	TCF7L2_standard_ChIP_seq_CW34
chr8	3845441	3845442	MACS_peak_4070	23.00	TCF7L2_standard_ChIP_seq_CW34
chr8	6321554	6321555	MACS_peak_4071	6.00	TCF7L2_standard_ChIP_seq_CW34
chr8	8145996	8145997	MACS_peak_4072	29.00	TCF7L2_standard_ChIP_seq_CW34
chr8	8178236	8178237	MACS_peak_4073	7.00	TCF7L2_standard_ChIP_seq_CW34
chr8	8287047	8287048	MACS_peak_4074	36.00	TCF7L2_standard_ChIP_seq_CW34
chr8	8663825	8663826	MACS_peak_4075	20.00	TCF7L2_standard_ChIP_seq_CW34
chr8	8953461	8953462	MACS_peak_4076	6.00	TCF7L2_standard_ChIP_seq_CW34
chr8	8960352	8960353	MACS_peak_4077	10.00	TCF7L2_standard_ChIP_seq_CW34
chr8	9212290	9212291	MACS_peak_4078	9.00	TCF7L2_standard_ChIP_seq_CW34
chr8	9308957	9308958	MACS_peak_4079	13.00	TCF7L2_standard_ChIP_seq_CW34
chr8	9638963	9638964	MACS_peak_4080	21.00	TCF7L2_standard_ChIP_seq_CW34
chr8	9651374	9651375	MACS_peak_4081	26.00	TCF7L2_standard_ChIP_seq_CW34
chr8	9676061	9676062	MACS_peak_4082	38.00	TCF7L2_standard_ChIP_seq_CW34
chr8	10657398	10657399	MACS_peak_4083	6.00	TCF7L2_standard_ChIP_seq_CW34
chr8	10873702	10873703	MACS_peak_4084	6.00	TCF7L2_standard_ChIP_seq_CW34
chr8	11711645	11711646	MACS_peak_4085	11.00	TCF7L2_standard_ChIP_seq_CW34
chr8	11792303	11792304	MACS_peak_4086	10.00	TCF7L2_standard_ChIP_seq_CW34
chr8	12954207	12954208	MACS_peak_4087	14.00	TCF7L2_standard_ChIP_seq_CW34
chr8	12989467	12989468	MACS_peak_4088	8.00	TCF7L2_standard_ChIP_seq_CW34
chr8	13306902	13306903	MACS_peak_4089	11.00	TCF7L2_standard_ChIP_seq_CW34
chr8	13844992	13844993	MACS_peak_4090	7.00	TCF7L2_standard_ChIP_seq_CW34
chr8	16909203	16909204	MACS_peak_4091	11.00	TCF7L2_standard_ChIP_seq_CW34
chr8	17160952	17160953	MACS_peak_4092	8.00	TCF7L2_standard_ChIP_seq_CW34
chr8	17186722	17186723	MACS_peak_4093	7.00	TCF7L2_standard_ChIP_seq_CW34
chr8	17208803	17208804	MACS_peak_4094	10.00	TCF7L2_standard_ChIP_seq_CW34
chr8	17472393	17472394	MACS_peak_4095	13.00	TCF7L2_standard_ChIP_seq_CW34
chr8	17611447	17611448	MACS_peak_4096	9.00	TCF7L2_standard_ChIP_seq_CW34
chr8	17658771	17658772	MACS_peak_4097	7.00	TCF7L2_standard_ChIP_seq_CW34
chr8	17753742	17753743	MACS_peak_4098	8.00	TCF7L2_standard_ChIP_seq_CW34
chr8	17764858	17764859	MACS_peak_4099	23.00	TCF7L2_standard_ChIP_seq_CW34
chr8	17773535	17773536	MACS_peak_4100	20.00	TCF7L2_standard_ChIP_seq_CW34
chr8	18871930	18871931	MACS_peak_4101	54.00	TCF7L2_standard_ChIP_seq_CW34
chr8	19729157	19729158	MACS_peak_4102	7.00	TCF7L2_standard_ChIP_seq_CW34
chr8	21350028	21350029	MACS_peak_4103	12.00	TCF7L2_standard_ChIP_seq_CW34
chr8	21924021	21924022	MACS_peak_4104	9.00	TCF7L2_standard_ChIP_seq_CW34
chr8	22102324	22102325	MACS_peak_4105	7.00	TCF7L2_standard_ChIP_seq_CW34
chr8	22416226	22416227	MACS_peak_4106	14.00	TCF7L2_standard_ChIP_seq_CW34
chr8	22500009	22500010	MACS_peak_4107	11.00	TCF7L2_standard_ChIP_seq_CW34
chr8	22940979	22940980	MACS_peak_4108	24.00	TCF7L2_standard_ChIP_seq_CW34
chr8	25250588	25250589	MACS_peak_4109	8.00	TCF7L2_standard_ChIP_seq_CW34
chr8	27317003	27317004	MACS_peak_4110	23.00	TCF7L2_standard_ChIP_seq_CW34
chr8	28551421	28551422	MACS_peak_4111	6.00	TCF7L2_standard_ChIP_seq_CW34
chr8	29206345	29206346	MACS_peak_4112	9.00	TCF7L2_standard_ChIP_seq_CW34
chr8	29355962	29355963	MACS_peak_4113	12.00	TCF7L2_standard_ChIP_seq_CW34
chr8	29940378	29940379	MACS_peak_4114	7.00	TCF7L2_standard_ChIP_seq_CW34
chr8	30242711	30242712	MACS_peak_4115	5.00	TCF7L2_standard_ChIP_seq_CW34
chr8	30378696	30378697	MACS_peak_4116	13.00	TCF7L2_standard_ChIP_seq_CW34
chr8	31964687	31964688	MACS_peak_4117	7.00	TCF7L2_standard_ChIP_seq_CW34
chr8	32892640	32892641	MACS_peak_4118	5.00	TCF7L2_standard_ChIP_seq_CW34
chr8	32949455	32949456	MACS_peak_4119	12.00	TCF7L2_standard_ChIP_seq_CW34
chr8	33135527	33135528	MACS_peak_4120	5.00	TCF7L2_standard_ChIP_seq_CW34
chr8	37265961	37265962	MACS_peak_4121	12.00	TCF7L2_standard_ChIP_seq_CW34
chr8	37456237	37456238	MACS_peak_4122	18.00	TCF7L2_standard_ChIP_seq_CW34
chr8	37482971	37482972	MACS_peak_4123	8.00	TCF7L2_standard_ChIP_seq_CW34
chr8	37533022	37533023	MACS_peak_4124	23.00	TCF7L2_standard_ChIP_seq_CW34
chr8	37553254	37553255	MACS_peak_4125	68.00	TCF7L2_standard_ChIP_seq_CW34
chr8	37857655	37857656	MACS_peak_4126	8.00	TCF7L2_standard_ChIP_seq_CW34
chr8	38033944	38033945	MACS_peak_4127	6.00	TCF7L2_standard_ChIP_seq_CW34
chr8	38415348	38415349	MACS_peak_4128	23.00	TCF7L2_standard_ChIP_seq_CW34
chr8	38803201	38803202	MACS_peak_4129	7.00	TCF7L2_standard_ChIP_seq_CW34
chr8	39424590	39424591	MACS_peak_4130	7.00	TCF7L2_standard_ChIP_seq_CW34
chr8	39901494	39901495	MACS_peak_4131	7.00	TCF7L2_standard_ChIP_seq_CW34
chr8	39912318	39912319	MACS_peak_4132	27.00	TCF7L2_standard_ChIP_seq_CW34
chr8	40020114	40020115	MACS_peak_4133	9.00	TCF7L2_standard_ChIP_seq_CW34
chr8	41009362	41009363	MACS_peak_4134	8.00	TCF7L2_standard_ChIP_seq_CW34
chr8	41435732	41435733	MACS_peak_4135	8.00	TCF7L2_standard_ChIP_seq_CW34
chr8	41787209	41787210	MACS_peak_4136	9.00	TCF7L2_standard_ChIP_seq_CW34
chr8	42224406	42224407	MACS_peak_4137	14.00	TCF7L2_standard_ChIP_seq_CW34
chr8	42310617	42310618	MACS_peak_4138	7.00	TCF7L2_standard_ChIP_seq_CW34
chr8	42996773	42996774	MACS_peak_4139	7.00	TCF7L2_standard_ChIP_seq_CW34
chr8	48260312	48260313	MACS_peak_4144	7.00	TCF7L2_standard_ChIP_seq_CW34
chr8	48670953	48670954	MACS_peak_4145	10.00	TCF7L2_standard_ChIP_seq_CW34
chr8	48895889	48895890	MACS_peak_4146	6.00	TCF7L2_standard_ChIP_seq_CW34
chr8	52608485	52608486	MACS_peak_4147	5.00	TCF7L2_standard_ChIP_seq_CW34
chr8	53303412	53303413	MACS_peak_4148	12.00	TCF7L2_standard_ChIP_seq_CW34
chr8	56685495	56685496	MACS_peak_4149	17.00	TCF7L2_standard_ChIP_seq_CW34
chr8	58463140	58463141	MACS_peak_4150	8.00	TCF7L2_standard_ChIP_seq_CW34
chr8	58477003	58477004	MACS_peak_4151	9.00	TCF7L2_standard_ChIP_seq_CW34
chr8	58721289	58721290	MACS_peak_4152	12.00	TCF7L2_standard_ChIP_seq_CW34
chr8	59008610	59008611	MACS_peak_4153	28.00	TCF7L2_standard_ChIP_seq_CW34
chr8	59085185	59085186	MACS_peak_4154	9.00	TCF7L2_standard_ChIP_seq_CW34
chr8	59571853	59571854	MACS_peak_4155	9.00	TCF7L2_standard_ChIP_seq_CW34
chr8	61564453	61564454	MACS_peak_4156	6.00	TCF7L2_standard_ChIP_seq_CW34
chr8	61705633	61705634	MACS_peak_4157	18.00	TCF7L2_standard_ChIP_seq_CW34
chr8	62626815	62626816	MACS_peak_4158	7.00	TCF7L2_standard_ChIP_seq_CW34
chr8	63951955	63951956	MACS_peak_4159	12.00	TCF7L2_standard_ChIP_seq_CW34
chr8	63952523	63952524	MACS_peak_4160	15.00	TCF7L2_standard_ChIP_seq_CW34
chr8	63965021	63965022	MACS_peak_4161	21.00	TCF7L2_standard_ChIP_seq_CW34
chr8	64242740	64242741	MACS_peak_4162	5.00	TCF7L2_standard_ChIP_seq_CW34
chr8	65642772	65642773	MACS_peak_4163	7.00	TCF7L2_standard_ChIP_seq_CW34
chr8	67454541	67454542	MACS_peak_4164	7.00	TCF7L2_standard_ChIP_seq_CW34
chr8	67804970	67804971	MACS_peak_4165	6.00	TCF7L2_standard_ChIP_seq_CW34
chr8	68649907	68649908	MACS_peak_4166	11.00	TCF7L2_standard_ChIP_seq_CW34
chr8	70491176	70491177	MACS_peak_4167	7.00	TCF7L2_standard_ChIP_seq_CW34
chr8	76888753	76888754	MACS_peak_4169	5.00	TCF7L2_standard_ChIP_seq_CW34
chr8	81522837	81522838	MACS_peak_4170	5.00	TCF7L2_standard_ChIP_seq_CW34
chr8	81718190	81718191	MACS_peak_4171	7.00	TCF7L2_standard_ChIP_seq_CW34
chr8	81733235	81733236	MACS_peak_4172	6.00	TCF7L2_standard_ChIP_seq_CW34
chr8	81877767	81877768	MACS_peak_4173	8.00	TCF7L2_standard_ChIP_seq_CW34
chr8	81942180	81942181	MACS_peak_4174	9.00	TCF7L2_standard_ChIP_seq_CW34
chr8	82518408	82518409	MACS_peak_4175	6.00	TCF7L2_standard_ChIP_seq_CW34
chr8	87223091	87223092	MACS_peak_4176	16.00	TCF7L2_standard_ChIP_seq_CW34
chr8	90914480	90914481	MACS_peak_4177	5.00	TCF7L2_standard_ChIP_seq_CW34
chr8	91013577	91013578	MACS_peak_4178	7.00	TCF7L2_standard_ChIP_seq_CW34
chr8	94678684	94678685	MACS_peak_4179	8.00	TCF7L2_standard_ChIP_seq_CW34
chr8	94962992	94962993	MACS_peak_4180	9.00	TCF7L2_standard_ChIP_seq_CW34
chr8	95972116	95972117	MACS_peak_4181	10.00	TCF7L2_standard_ChIP_seq_CW34
chr8	96087284	96087285	MACS_peak_4182	7.00	TCF7L2_standard_ChIP_seq_CW34
chr8	97273976	97273977	MACS_peak_4183	8.00	TCF7L2_standard_ChIP_seq_CW34
chr8	97536300	97536301	MACS_peak_4184	10.00	TCF7L2_standard_ChIP_seq_CW34
chr8	97602045	97602046	MACS_peak_4185	10.00	TCF7L2_standard_ChIP_seq_CW34
chr8	98175811	98175812	MACS_peak_4186	32.00	TCF7L2_standard_ChIP_seq_CW34
chr8	98244677	98244678	MACS_peak_4187	8.00	TCF7L2_standard_ChIP_seq_CW34
chr8	98375825	98375826	MACS_peak_4188	8.00	TCF7L2_standard_ChIP_seq_CW34
chr8	99610483	99610484	MACS_peak_4189	10.00	TCF7L2_standard_ChIP_seq_CW34
chr8	99848062	99848063	MACS_peak_4190	7.00	TCF7L2_standard_ChIP_seq_CW34
chr8	99926794	99926795	MACS_peak_4191	10.00	TCF7L2_standard_ChIP_seq_CW34
chr8	101347101	101347102	MACS_peak_4192	8.00	TCF7L2_standard_ChIP_seq_CW34
chr8	101572041	101572042	MACS_peak_4193	17.00	TCF7L2_standard_ChIP_seq_CW34
chr8	103428940	103428941	MACS_peak_4194	11.00	TCF7L2_standard_ChIP_seq_CW34
chr8	103504423	103504424	MACS_peak_4195	27.00	TCF7L2_standard_ChIP_seq_CW34
chr8	103818855	103818856	MACS_peak_4196	10.00	TCF7L2_standard_ChIP_seq_CW34
chr8	107637024	107637025	MACS_peak_4197	25.00	TCF7L2_standard_ChIP_seq_CW34
chr8	117845685	117845686	MACS_peak_4198	7.00	TCF7L2_standard_ChIP_seq_CW34
chr8	119561184	119561185	MACS_peak_4199	12.00	TCF7L2_standard_ChIP_seq_CW34
chr8	120064499	120064500	MACS_peak_4200	6.00	TCF7L2_standard_ChIP_seq_CW34
chr8	120667580	120667581	MACS_peak_4201	8.00	TCF7L2_standard_ChIP_seq_CW34
chr8	121760375	121760376	MACS_peak_4202	10.00	TCF7L2_standard_ChIP_seq_CW34
chr8	121787600	121787601	MACS_peak_4203	20.00	TCF7L2_standard_ChIP_seq_CW34
chr8	122361254	122361255	MACS_peak_4204	33.00	TCF7L2_standard_ChIP_seq_CW34
chr8	123741021	123741022	MACS_peak_4205	8.00	TCF7L2_standard_ChIP_seq_CW34
chr8	124217570	124217571	MACS_peak_4206	9.00	TCF7L2_standard_ChIP_seq_CW34
chr8	124287214	124287215	MACS_peak_4207	8.00	TCF7L2_standard_ChIP_seq_CW34
chr8	124387007	124387008	MACS_peak_4208	5.00	TCF7L2_standard_ChIP_seq_CW34
chr8	124417929	124417930	MACS_peak_4209	5.00	TCF7L2_standard_ChIP_seq_CW34
chr8	125384918	125384919	MACS_peak_4210	8.00	TCF7L2_standard_ChIP_seq_CW34
chr8	125643714	125643715	MACS_peak_4211	6.00	TCF7L2_standard_ChIP_seq_CW34
chr8	126245297	126245298	MACS_peak_4212	9.00	TCF7L2_standard_ChIP_seq_CW34
chr8	126514088	126514089	MACS_peak_4213	48.00	TCF7L2_standard_ChIP_seq_CW34
chr8	126525432	126525433	MACS_peak_4214	10.00	TCF7L2_standard_ChIP_seq_CW34
chr8	128231340	128231341	MACS_peak_4215	29.00	TCF7L2_standard_ChIP_seq_CW34
chr8	128309950	128309951	MACS_peak_4216	10.00	TCF7L2_standard_ChIP_seq_CW34
chr8	128413371	128413372	MACS_peak_4217	11.00	TCF7L2_standard_ChIP_seq_CW34
chr8	128614250	128614251	MACS_peak_4218	6.00	TCF7L2_standard_ChIP_seq_CW34
chr8	129129985	129129986	MACS_peak_4219	16.00	TCF7L2_standard_ChIP_seq_CW34
chr8	129197553	129197554	MACS_peak_4220	35.00	TCF7L2_standard_ChIP_seq_CW34
chr8	129370428	129370429	MACS_peak_4221	32.00	TCF7L2_standard_ChIP_seq_CW34
chr8	129460508	129460509	MACS_peak_4222	14.00	TCF7L2_standard_ChIP_seq_CW34
chr8	129567418	129567419	MACS_peak_4223	44.00	TCF7L2_standard_ChIP_seq_CW34
chr8	129568908	129568909	MACS_peak_4224	30.00	TCF7L2_standard_ChIP_seq_CW34
chr8	129597575	129597576	MACS_peak_4225	19.00	TCF7L2_standard_ChIP_seq_CW34
chr8	129615000	129615001	MACS_peak_4226	14.00	TCF7L2_standard_ChIP_seq_CW34
chr8	129665715	129665716	MACS_peak_4227	8.00	TCF7L2_standard_ChIP_seq_CW34
chr8	131342434	131342435	MACS_peak_4228	9.00	TCF7L2_standard_ChIP_seq_CW34
chr8	131439159	131439160	MACS_peak_4229	14.00	TCF7L2_standard_ChIP_seq_CW34
chr8	134502137	134502138	MACS_peak_4230	9.00	TCF7L2_standard_ChIP_seq_CW34
chr8	135766570	135766571	MACS_peak_4231	7.00	TCF7L2_standard_ChIP_seq_CW34
chr8	135807470	135807471	MACS_peak_4232	9.00	TCF7L2_standard_ChIP_seq_CW34
chr8	135844509	135844510	MACS_peak_4233	8.00	TCF7L2_standard_ChIP_seq_CW34
chr8	137042094	137042095	MACS_peak_4234	11.00	TCF7L2_standard_ChIP_seq_CW34
chr8	139272410	139272411	MACS_peak_4235	6.00	TCF7L2_standard_ChIP_seq_CW34
chr8	140131128	140131129	MACS_peak_4236	8.00	TCF7L2_standard_ChIP_seq_CW34
chr8	140399449	140399450	MACS_peak_4237	18.00	TCF7L2_standard_ChIP_seq_CW34
chr8	140874767	140874768	MACS_peak_4238	24.00	TCF7L2_standard_ChIP_seq_CW34
chr8	140878350	140878351	MACS_peak_4239	9.00	TCF7L2_standard_ChIP_seq_CW34
chr8	141059225	141059226	MACS_peak_4240	12.00	TCF7L2_standard_ChIP_seq_CW34
chr8	141063974	141063975	MACS_peak_4241	11.00	TCF7L2_standard_ChIP_seq_CW34
chr8	141101794	141101795	MACS_peak_4242	12.00	TCF7L2_standard_ChIP_seq_CW34
chr8	141636498	141636499	MACS_peak_4243	34.00	TCF7L2_standard_ChIP_seq_CW34
chr8	141637509	141637510	MACS_peak_4244	16.00	TCF7L2_standard_ChIP_seq_CW34
chr8	142238769	142238770	MACS_peak_4245	19.00	TCF7L2_standard_ChIP_seq_CW34
chr8	142409431	142409432	MACS_peak_4246	53.00	TCF7L2_standard_ChIP_seq_CW34
chr8	142427814	142427815	MACS_peak_4247	8.00	TCF7L2_standard_ChIP_seq_CW34
chr8	144392505	144392506	MACS_peak_4248	8.00	TCF7L2_standard_ChIP_seq_CW34
chr8	144591355	144591356	MACS_peak_4249	32.00	TCF7L2_standard_ChIP_seq_CW34
chr8	144679520	144679521	MACS_peak_4250	23.00	TCF7L2_standard_ChIP_seq_CW34
chr8	144816248	144816249	MACS_peak_4251	11.00	TCF7L2_standard_ChIP_seq_CW34
chr8	144940667	144940668	MACS_peak_4252	7.00	TCF7L2_standard_ChIP_seq_CW34
chr9	674466	674467	MACS_peak_4253	10.00	TCF7L2_standard_ChIP_seq_CW34
chr9	682799	682800	MACS_peak_4254	13.00	TCF7L2_standard_ChIP_seq_CW34
chr9	755911	755912	MACS_peak_4255	8.00	TCF7L2_standard_ChIP_seq_CW34
chr9	4023451	4023452	MACS_peak_4256	5.00	TCF7L2_standard_ChIP_seq_CW34
chr9	4662934	4662935	MACS_peak_4257	11.00	TCF7L2_standard_ChIP_seq_CW34
chr9	4740726	4740727	MACS_peak_4258	29.00	TCF7L2_standard_ChIP_seq_CW34
chr9	5721853	5721854	MACS_peak_4259	11.00	TCF7L2_standard_ChIP_seq_CW34
chr9	6590943	6590944	MACS_peak_4260	52.00	TCF7L2_standard_ChIP_seq_CW34
chr9	6898080	6898081	MACS_peak_4261	39.00	TCF7L2_standard_ChIP_seq_CW34
chr9	6955516	6955517	MACS_peak_4262	14.00	TCF7L2_standard_ChIP_seq_CW34
chr9	14518370	14518371	MACS_peak_4263	6.00	TCF7L2_standard_ChIP_seq_CW34
chr9	14869227	14869228	MACS_peak_4264	9.00	TCF7L2_standard_ChIP_seq_CW34
chr9	14969282	14969283	MACS_peak_4265	9.00	TCF7L2_standard_ChIP_seq_CW34
chr9	15080199	15080200	MACS_peak_4266	10.00	TCF7L2_standard_ChIP_seq_CW34
chr9	15100308	15100309	MACS_peak_4267	9.00	TCF7L2_standard_ChIP_seq_CW34
chr9	15280465	15280466	MACS_peak_4268	6.00	TCF7L2_standard_ChIP_seq_CW34
chr9	15422956	15422957	MACS_peak_4269	5.00	TCF7L2_standard_ChIP_seq_CW34
chr9	19050933	19050934	MACS_peak_4270	6.00	TCF7L2_standard_ChIP_seq_CW34
chr9	19161906	19161907	MACS_peak_4271	11.00	TCF7L2_standard_ChIP_seq_CW34
chr9	19202459	19202460	MACS_peak_4272	20.00	TCF7L2_standard_ChIP_seq_CW34
chr9	19626002	19626003	MACS_peak_4273	7.00	TCF7L2_standard_ChIP_seq_CW34
chr9	19675974	19675975	MACS_peak_4274	5.00	TCF7L2_standard_ChIP_seq_CW34
chr9	20835900	20835901	MACS_peak_4275	13.00	TCF7L2_standard_ChIP_seq_CW34
chr9	21814098	21814099	MACS_peak_4276	6.00	TCF7L2_standard_ChIP_seq_CW34
chr9	21963026	21963027	MACS_peak_4277	7.00	TCF7L2_standard_ChIP_seq_CW34
chr9	27101107	27101108	MACS_peak_4278	7.00	TCF7L2_standard_ChIP_seq_CW34
chr9	27566297	27566298	MACS_peak_4279	9.00	TCF7L2_standard_ChIP_seq_CW34
chr9	28213166	28213167	MACS_peak_4280	7.00	TCF7L2_standard_ChIP_seq_CW34
chr9	32130909	32130910	MACS_peak_4281	20.00	TCF7L2_standard_ChIP_seq_CW34
chr9	32136962	32136963	MACS_peak_4282	8.00	TCF7L2_standard_ChIP_seq_CW34
chr9	33415703	33415704	MACS_peak_4283	31.00	TCF7L2_standard_ChIP_seq_CW34
chr9	33818012	33818013	MACS_peak_4284	8.00	TCF7L2_standard_ChIP_seq_CW34
chr9	34206701	34206702	MACS_peak_4285	9.00	TCF7L2_standard_ChIP_seq_CW34
chr9	35490049	35490050	MACS_peak_4286	9.00	TCF7L2_standard_ChIP_seq_CW34
chr9	36400762	36400763	MACS_peak_4287	6.00	TCF7L2_standard_ChIP_seq_CW34
chr9	36420193	36420194	MACS_peak_4288	16.00	TCF7L2_standard_ChIP_seq_CW34
chr9	37800591	37800592	MACS_peak_4289	6.00	TCF7L2_standard_ChIP_seq_CW34
chr9	37908800	37908801	MACS_peak_4290	6.00	TCF7L2_standard_ChIP_seq_CW34
chr9	38048019	38048020	MACS_peak_4291	11.00	TCF7L2_standard_ChIP_seq_CW34
chr9	38063131	38063132	MACS_peak_4292	13.00	TCF7L2_standard_ChIP_seq_CW34
chr9	38555132	38555133	MACS_peak_4293	7.00	TCF7L2_standard_ChIP_seq_CW34
chr9	40798196	40798197	MACS_peak_4294	6.00	TCF7L2_standard_ChIP_seq_CW34
chr9	44763881	44763882	MACS_peak_4296	5.00	TCF7L2_standard_ChIP_seq_CW34
chr9	45362670	45362671	MACS_peak_4300	8.00	TCF7L2_standard_ChIP_seq_CW34
chr9	66454991	66454992	MACS_peak_4302	11.00	TCF7L2_standard_ChIP_seq_CW34
chr9	68405086	68405087	MACS_peak_4307	15.00	TCF7L2_standard_ChIP_seq_CW34
chr9	68484627	68484628	MACS_peak_4308	9.00	TCF7L2_standard_ChIP_seq_CW34
chr9	69073641	69073642	MACS_peak_4309	8.00	TCF7L2_standard_ChIP_seq_CW34
chr9	69825864	69825865	MACS_peak_4310	11.00	TCF7L2_standard_ChIP_seq_CW34
chr9	71788941	71788942	MACS_peak_4311	10.00	TCF7L2_standard_ChIP_seq_CW34
chr9	71793533	71793534	MACS_peak_4312	9.00	TCF7L2_standard_ChIP_seq_CW34
chr9	71805276	71805277	MACS_peak_4313	19.00	TCF7L2_standard_ChIP_seq_CW34
chr9	73034363	73034364	MACS_peak_4314	11.00	TCF7L2_standard_ChIP_seq_CW34
chr9	73711490	73711491	MACS_peak_4315	8.00	TCF7L2_standard_ChIP_seq_CW34
chr9	75549374	75549375	MACS_peak_4316	7.00	TCF7L2_standard_ChIP_seq_CW34
chr9	76640598	76640599	MACS_peak_4317	8.00	TCF7L2_standard_ChIP_seq_CW34
chr9	77646198	77646199	MACS_peak_4318	8.00	TCF7L2_standard_ChIP_seq_CW34
chr9	79038862	79038863	MACS_peak_4319	12.00	TCF7L2_standard_ChIP_seq_CW34
chr9	79071256	79071257	MACS_peak_4320	20.00	TCF7L2_standard_ChIP_seq_CW34
chr9	80127937	80127938	MACS_peak_4322	12.00	TCF7L2_standard_ChIP_seq_CW34
chr9	81014186	81014187	MACS_peak_4323	13.00	TCF7L2_standard_ChIP_seq_CW34
chr9	81022061	81022062	MACS_peak_4324	10.00	TCF7L2_standard_ChIP_seq_CW34
chr9	82104710	82104711	MACS_peak_4325	8.00	TCF7L2_standard_ChIP_seq_CW34
chr9	84150635	84150636	MACS_peak_4326	9.00	TCF7L2_standard_ChIP_seq_CW34
chr9	84202789	84202790	MACS_peak_4327	8.00	TCF7L2_standard_ChIP_seq_CW34
chr9	84272007	84272008	MACS_peak_4328	14.00	TCF7L2_standard_ChIP_seq_CW34
chr9	84301974	84301975	MACS_peak_4329	13.00	TCF7L2_standard_ChIP_seq_CW34
chr9	85175504	85175505	MACS_peak_4330	14.00	TCF7L2_standard_ChIP_seq_CW34
chr9	85216045	85216046	MACS_peak_4331	10.00	TCF7L2_standard_ChIP_seq_CW34
chr9	86044346	86044347	MACS_peak_4332	17.00	TCF7L2_standard_ChIP_seq_CW34
chr9	86235917	86235918	MACS_peak_4333	22.00	TCF7L2_standard_ChIP_seq_CW34
chr9	88951137	88951138	MACS_peak_4334	18.00	TCF7L2_standard_ChIP_seq_CW34
chr9	89020260	89020261	MACS_peak_4335	17.00	TCF7L2_standard_ChIP_seq_CW34
chr9	90184212	90184213	MACS_peak_4336	8.00	TCF7L2_standard_ChIP_seq_CW34
chr9	90559065	90559066	MACS_peak_4337	6.00	TCF7L2_standard_ChIP_seq_CW34
chr9	91321351	91321352	MACS_peak_4338	10.00	TCF7L2_standard_ChIP_seq_CW34
chr9	92171640	92171641	MACS_peak_4339	8.00	TCF7L2_standard_ChIP_seq_CW34
chr9	92263390	92263391	MACS_peak_4340	31.00	TCF7L2_standard_ChIP_seq_CW34
chr9	92686446	92686447	MACS_peak_4341	10.00	TCF7L2_standard_ChIP_seq_CW34
chr9	93960164	93960165	MACS_peak_4342	27.00	TCF7L2_standard_ChIP_seq_CW34
chr9	95088027	95088028	MACS_peak_4343	8.00	TCF7L2_standard_ChIP_seq_CW34
chr9	95379291	95379292	MACS_peak_4344	8.00	TCF7L2_standard_ChIP_seq_CW34
chr9	95882406	95882407	MACS_peak_4345	8.00	TCF7L2_standard_ChIP_seq_CW34
chr9	97590081	97590082	MACS_peak_4346	24.00	TCF7L2_standard_ChIP_seq_CW34
chr9	97691138	97691139	MACS_peak_4347	8.00	TCF7L2_standard_ChIP_seq_CW34
chr9	97733893	97733894	MACS_peak_4348	8.00	TCF7L2_standard_ChIP_seq_CW34
chr9	97794167	97794168	MACS_peak_4349	9.00	TCF7L2_standard_ChIP_seq_CW34
chr9	97810589	97810590	MACS_peak_4350	6.00	TCF7L2_standard_ChIP_seq_CW34
chr9	98487053	98487054	MACS_peak_4351	12.00	TCF7L2_standard_ChIP_seq_CW34
chr9	98912151	98912152	MACS_peak_4352	6.00	TCF7L2_standard_ChIP_seq_CW34
chr9	99146400	99146401	MACS_peak_4353	8.00	TCF7L2_standard_ChIP_seq_CW34
chr9	99253936	99253937	MACS_peak_4354	31.00	TCF7L2_standard_ChIP_seq_CW34
chr9	99329191	99329192	MACS_peak_4355	30.00	TCF7L2_standard_ChIP_seq_CW34
chr9	100157422	100157423	MACS_peak_4356	17.00	TCF7L2_standard_ChIP_seq_CW34
chr9	100167253	100167254	MACS_peak_4357	11.00	TCF7L2_standard_ChIP_seq_CW34
chr9	100263599	100263600	MACS_peak_4358	7.00	TCF7L2_standard_ChIP_seq_CW34
chr9	101104869	101104870	MACS_peak_4359	5.00	TCF7L2_standard_ChIP_seq_CW34
chr9	103791379	103791380	MACS_peak_4360	7.00	TCF7L2_standard_ChIP_seq_CW34
chr9	104077865	104077866	MACS_peak_4361	9.00	TCF7L2_standard_ChIP_seq_CW34
chr9	104089871	104089872	MACS_peak_4362	16.00	TCF7L2_standard_ChIP_seq_CW34
chr9	104195792	104195793	MACS_peak_4363	8.00	TCF7L2_standard_ChIP_seq_CW34
chr9	104296288	104296289	MACS_peak_4364	9.00	TCF7L2_standard_ChIP_seq_CW34
chr9	107393136	107393137	MACS_peak_4365	6.00	TCF7L2_standard_ChIP_seq_CW34
chr9	107674961	107674962	MACS_peak_4366	22.00	TCF7L2_standard_ChIP_seq_CW34
chr9	107827016	107827017	MACS_peak_4367	11.00	TCF7L2_standard_ChIP_seq_CW34
chr9	110309442	110309443	MACS_peak_4368	7.00	TCF7L2_standard_ChIP_seq_CW34
chr9	111207834	111207835	MACS_peak_4369	37.00	TCF7L2_standard_ChIP_seq_CW34
chr9	111648319	111648320	MACS_peak_4370	16.00	TCF7L2_standard_ChIP_seq_CW34
chr9	111656795	111656796	MACS_peak_4371	14.00	TCF7L2_standard_ChIP_seq_CW34
chr9	111844535	111844536	MACS_peak_4372	13.00	TCF7L2_standard_ChIP_seq_CW34
chr9	112956785	112956786	MACS_peak_4373	13.00	TCF7L2_standard_ChIP_seq_CW34
chr9	114881965	114881966	MACS_peak_4374	25.00	TCF7L2_standard_ChIP_seq_CW34
chr9	115258812	115258813	MACS_peak_4375	7.00	TCF7L2_standard_ChIP_seq_CW34
chr9	115603670	115603671	MACS_peak_4376	9.00	TCF7L2_standard_ChIP_seq_CW34
chr9	116089741	116089742	MACS_peak_4377	8.00	TCF7L2_standard_ChIP_seq_CW34
chr9	116267342	116267343	MACS_peak_4378	15.00	TCF7L2_standard_ChIP_seq_CW34
chr9	116916360	116916361	MACS_peak_4379	67.00	TCF7L2_standard_ChIP_seq_CW34
chr9	117647303	117647304	MACS_peak_4380	14.00	TCF7L2_standard_ChIP_seq_CW34
chr9	118257726	118257727	MACS_peak_4381	11.00	TCF7L2_standard_ChIP_seq_CW34
chr9	118706885	118706886	MACS_peak_4382	15.00	TCF7L2_standard_ChIP_seq_CW34
chr9	123497968	123497969	MACS_peak_4383	8.00	TCF7L2_standard_ChIP_seq_CW34
chr9	123837027	123837028	MACS_peak_4384	7.00	TCF7L2_standard_ChIP_seq_CW34
chr9	123893622	123893623	MACS_peak_4385	6.00	TCF7L2_standard_ChIP_seq_CW34
chr9	124186360	124186361	MACS_peak_4386	6.00	TCF7L2_standard_ChIP_seq_CW34
chr9	124366752	124366753	MACS_peak_4387	7.00	TCF7L2_standard_ChIP_seq_CW34
chr9	125109387	125109388	MACS_peak_4388	8.00	TCF7L2_standard_ChIP_seq_CW34
chr9	126227784	126227785	MACS_peak_4389	10.00	TCF7L2_standard_ChIP_seq_CW34
chr9	126360385	126360386	MACS_peak_4390	7.00	TCF7L2_standard_ChIP_seq_CW34
chr9	127148641	127148642	MACS_peak_4391	9.00	TCF7L2_standard_ChIP_seq_CW34
chr9	127703438	127703439	MACS_peak_4392	7.00	TCF7L2_standard_ChIP_seq_CW34
chr9	129279587	129279588	MACS_peak_4393	11.00	TCF7L2_standard_ChIP_seq_CW34
chr9	129289995	129289996	MACS_peak_4394	16.00	TCF7L2_standard_ChIP_seq_CW34
chr9	129445376	129445377	MACS_peak_4395	16.00	TCF7L2_standard_ChIP_seq_CW34
chr9	130150876	130150877	MACS_peak_4396	13.00	TCF7L2_standard_ChIP_seq_CW34
chr9	130153028	130153029	MACS_peak_4397	23.00	TCF7L2_standard_ChIP_seq_CW34
chr9	130369944	130369945	MACS_peak_4398	19.00	TCF7L2_standard_ChIP_seq_CW34
chr9	130713119	130713120	MACS_peak_4399	17.00	TCF7L2_standard_ChIP_seq_CW34
chr9	130820493	130820494	MACS_peak_4400	8.00	TCF7L2_standard_ChIP_seq_CW34
chr9	131008252	131008253	MACS_peak_4401	14.00	TCF7L2_standard_ChIP_seq_CW34
chr9	131600856	131600857	MACS_peak_4402	11.00	TCF7L2_standard_ChIP_seq_CW34
chr9	132319918	132319919	MACS_peak_4403	37.00	TCF7L2_standard_ChIP_seq_CW34
chr9	132331048	132331049	MACS_peak_4404	33.00	TCF7L2_standard_ChIP_seq_CW34
chr9	132359798	132359799	MACS_peak_4405	146.00	TCF7L2_standard_ChIP_seq_CW34
chr9	132417179	132417180	MACS_peak_4406	9.00	TCF7L2_standard_ChIP_seq_CW34
chr9	133320881	133320882	MACS_peak_4407	8.00	TCF7L2_standard_ChIP_seq_CW34
chr9	133476771	133476772	MACS_peak_4408	9.00	TCF7L2_standard_ChIP_seq_CW34
chr9	135349910	135349911	MACS_peak_4409	10.00	TCF7L2_standard_ChIP_seq_CW34
chr9	136242723	136242724	MACS_peak_4410	5.00	TCF7L2_standard_ChIP_seq_CW34
chr9	136399837	136399838	MACS_peak_4411	13.00	TCF7L2_standard_ChIP_seq_CW34
chr9	136423429	136423430	MACS_peak_4412	11.00	TCF7L2_standard_ChIP_seq_CW34
chr9	137225454	137225455	MACS_peak_4413	13.00	TCF7L2_standard_ChIP_seq_CW34
chr9	137977918	137977919	MACS_peak_4414	9.00	TCF7L2_standard_ChIP_seq_CW34
chr9	139294458	139294459	MACS_peak_4415	8.00	TCF7L2_standard_ChIP_seq_CW34
chr9	139886899	139886900	MACS_peak_4416	8.00	TCF7L2_standard_ChIP_seq_CW34
chr9	140345011	140345012	MACS_peak_4417	27.00	TCF7L2_standard_ChIP_seq_CW34
chr9	140684025	140684026	MACS_peak_4418	11.00	TCF7L2_standard_ChIP_seq_CW34
chrX	7136821	7136822	MACS_peak_4419	12.00	TCF7L2_standard_ChIP_seq_CW34
chrX	9451519	9451520	MACS_peak_4420	8.00	TCF7L2_standard_ChIP_seq_CW34
chrX	9677155	9677156	MACS_peak_4421	7.00	TCF7L2_standard_ChIP_seq_CW34
chrX	12862112	12862113	MACS_peak_4422	10.00	TCF7L2_standard_ChIP_seq_CW34
chrX	15620336	15620337	MACS_peak_4423	10.00	TCF7L2_standard_ChIP_seq_CW34
chrX	15762593	15762594	MACS_peak_4424	11.00	TCF7L2_standard_ChIP_seq_CW34
chrX	20338991	20338992	MACS_peak_4425	18.00	TCF7L2_standard_ChIP_seq_CW34
chrX	20498258	20498259	MACS_peak_4426	6.00	TCF7L2_standard_ChIP_seq_CW34
chrX	22046377	22046378	MACS_peak_4427	13.00	TCF7L2_standard_ChIP_seq_CW34
chrX	23860072	23860073	MACS_peak_4428	10.00	TCF7L2_standard_ChIP_seq_CW34
chrX	25554667	25554668	MACS_peak_4429	19.00	TCF7L2_standard_ChIP_seq_CW34
chrX	30646116	30646117	MACS_peak_4430	8.00	TCF7L2_standard_ChIP_seq_CW34
chrX	30661661	30661662	MACS_peak_4431	14.00	TCF7L2_standard_ChIP_seq_CW34
chrX	31010782	31010783	MACS_peak_4432	6.00	TCF7L2_standard_ChIP_seq_CW34
chrX	31239611	31239612	MACS_peak_4433	8.00	TCF7L2_standard_ChIP_seq_CW34
chrX	37818665	37818666	MACS_peak_4434	15.00	TCF7L2_standard_ChIP_seq_CW34
chrX	38662963	38662964	MACS_peak_4435	5.00	TCF7L2_standard_ChIP_seq_CW34
chrX	39631459	39631460	MACS_peak_4436	9.00	TCF7L2_standard_ChIP_seq_CW34
chrX	39862858	39862859	MACS_peak_4437	23.00	TCF7L2_standard_ChIP_seq_CW34
chrX	40014057	40014058	MACS_peak_4438	10.00	TCF7L2_standard_ChIP_seq_CW34
chrX	41730182	41730183	MACS_peak_4439	7.00	TCF7L2_standard_ChIP_seq_CW34
chrX	43741839	43741840	MACS_peak_4440	9.00	TCF7L2_standard_ChIP_seq_CW34
chrX	45754906	45754907	MACS_peak_4441	6.00	TCF7L2_standard_ChIP_seq_CW34
chrX	46180071	46180072	MACS_peak_4442	7.00	TCF7L2_standard_ChIP_seq_CW34
chrX	46187447	46187448	MACS_peak_4443	8.00	TCF7L2_standard_ChIP_seq_CW34
chrX	46938295	46938296	MACS_peak_4444	13.00	TCF7L2_standard_ChIP_seq_CW34
chrX	47004389	47004390	MACS_peak_4445	7.00	TCF7L2_standard_ChIP_seq_CW34
chrX	47095290	47095291	MACS_peak_4446	6.00	TCF7L2_standard_ChIP_seq_CW34
chrX	47518729	47518730	MACS_peak_4447	10.00	TCF7L2_standard_ChIP_seq_CW34
chrX	49577010	49577011	MACS_peak_4448	17.00	TCF7L2_standard_ChIP_seq_CW34
chrX	49581257	49581258	MACS_peak_4449	7.00	TCF7L2_standard_ChIP_seq_CW34
chrX	54440345	54440346	MACS_peak_4450	7.00	TCF7L2_standard_ChIP_seq_CW34
chrX	54835356	54835357	MACS_peak_4451	6.00	TCF7L2_standard_ChIP_seq_CW34
chrX	68123146	68123147	MACS_peak_4454	6.00	TCF7L2_standard_ChIP_seq_CW34
chrX	68415407	68415408	MACS_peak_4455	7.00	TCF7L2_standard_ChIP_seq_CW34
chrX	69440379	69440380	MACS_peak_4456	12.00	TCF7L2_standard_ChIP_seq_CW34
chrX	70573017	70573018	MACS_peak_4457	11.00	TCF7L2_standard_ChIP_seq_CW34
chrX	70613121	70613122	MACS_peak_4458	39.00	TCF7L2_standard_ChIP_seq_CW34
chrX	76308350	76308351	MACS_peak_4459	8.00	TCF7L2_standard_ChIP_seq_CW34
chrX	76975096	76975097	MACS_peak_4460	7.00	TCF7L2_standard_ChIP_seq_CW34
chrX	87695126	87695127	MACS_peak_4461	6.00	TCF7L2_standard_ChIP_seq_CW34
chrX	87815950	87815951	MACS_peak_4462	18.00	TCF7L2_standard_ChIP_seq_CW34
chrX	95939684	95939685	MACS_peak_4463	9.00	TCF7L2_standard_ChIP_seq_CW34
chrX	105936578	105936579	MACS_peak_4464	6.00	TCF7L2_standard_ChIP_seq_CW34
chrX	106012701	106012702	MACS_peak_4465	7.00	TCF7L2_standard_ChIP_seq_CW34
chrX	106362047	106362048	MACS_peak_4466	6.00	TCF7L2_standard_ChIP_seq_CW34
chrX	108983375	108983376	MACS_peak_4468	17.00	TCF7L2_standard_ChIP_seq_CW34
chrX	109066332	109066333	MACS_peak_4469	10.00	TCF7L2_standard_ChIP_seq_CW34
chrX	109074122	109074123	MACS_peak_4470	11.00	TCF7L2_standard_ChIP_seq_CW34
chrX	109115512	109115513	MACS_peak_4471	6.00	TCF7L2_standard_ChIP_seq_CW34
chrX	117957754	117957755	MACS_peak_4472	7.00	TCF7L2_standard_ChIP_seq_CW34
chrX	119420754	119420755	MACS_peak_4473	13.00	TCF7L2_standard_ChIP_seq_CW34
chrX	119620199	119620200	MACS_peak_4474	9.00	TCF7L2_standard_ChIP_seq_CW34
chrX	120379842	120379843	MACS_peak_4475	8.00	TCF7L2_standard_ChIP_seq_CW34
chrX	123596963	123596964	MACS_peak_4476	5.00	TCF7L2_standard_ChIP_seq_CW34
chrX	128278849	128278850	MACS_peak_4477	7.00	TCF7L2_standard_ChIP_seq_CW34
chrX	128659223	128659224	MACS_peak_4478	8.00	TCF7L2_standard_ChIP_seq_CW34
chrX	128911362	128911363	MACS_peak_4479	10.00	TCF7L2_standard_ChIP_seq_CW34
chrX	131465207	131465208	MACS_peak_4480	48.00	TCF7L2_standard_ChIP_seq_CW34
chrX	131623630	131623631	MACS_peak_4481	5.00	TCF7L2_standard_ChIP_seq_CW34
chrX	131724651	131724652	MACS_peak_4482	31.00	TCF7L2_standard_ChIP_seq_CW34
chrX	132422061	132422062	MACS_peak_4483	8.00	TCF7L2_standard_ChIP_seq_CW34
chrX	133128855	133128856	MACS_peak_4484	13.00	TCF7L2_standard_ChIP_seq_CW34
chrX	133156887	133156888	MACS_peak_4485	15.00	TCF7L2_standard_ChIP_seq_CW34
chrX	133158072	133158073	MACS_peak_4486	10.00	TCF7L2_standard_ChIP_seq_CW34
chrX	134457895	134457896	MACS_peak_4487	14.00	TCF7L2_standard_ChIP_seq_CW34
chrX	136520468	136520469	MACS_peak_4488	16.00	TCF7L2_standard_ChIP_seq_CW34
chrX	140376929	140376930	MACS_peak_4489	5.00	TCF7L2_standard_ChIP_seq_CW34
chrX	146966742	146966743	MACS_peak_4490	25.00	TCF7L2_standard_ChIP_seq_CW34
chrX	150716973	150716974	MACS_peak_4491	7.00	TCF7L2_standard_ChIP_seq_CW34
chrX	153016090	153016091	MACS_peak_4492	8.00	TCF7L2_standard_ChIP_seq_CW34
chrX	153626440	153626441	MACS_peak_4493	9.00	TCF7L2_standard_ChIP_seq_CW34
chrX	154028206	154028207	MACS_peak_4494	6.00	TCF7L2_standard_ChIP_seq_CW34
chrX	154255128	154255129	MACS_peak_4495	8.00	TCF7L2_standard_ChIP_seq_CW34
chrY	7148558	7148559	MACS_peak_4496	13.00	TCF7L2_standard_ChIP_seq_CW34
chrY	7283533	7283534	MACS_peak_4497	14.00	TCF7L2_standard_ChIP_seq_CW34
chrY	10025231	10025232	MACS_peak_4498	42.00	TCF7L2_standard_ChIP_seq_CW34
chrY	10037811	10037812	MACS_peak_4499	31.00	TCF7L2_standard_ChIP_seq_CW34
chrY	21730029	21730030	MACS_peak_4512	12.00	TCF7L2_standard_ChIP_seq_CW34
chr1	1060296	1060297	MACS_peak_1	8.00	lobChIP_FOXA1_robot
chr1	1356693	1356694	MACS_peak_2	5.00	lobChIP_FOXA1_robot
chr1	1714582	1714583	MACS_peak_3	7.00	lobChIP_FOXA1_robot
chr1	2066280	2066281	MACS_peak_4	5.00	lobChIP_FOXA1_robot
chr1	2121695	2121696	MACS_peak_5	4.00	lobChIP_FOXA1_robot
chr1	3155121	3155122	MACS_peak_6	5.00	lobChIP_FOXA1_robot
chr1	3210102	3210103	MACS_peak_7	5.00	lobChIP_FOXA1_robot
chr1	3252179	3252180	MACS_peak_8	4.00	lobChIP_FOXA1_robot
chr1	3360591	3360592	MACS_peak_9	4.00	lobChIP_FOXA1_robot
chr1	3541257	3541258	MACS_peak_10	5.00	lobChIP_FOXA1_robot
chr1	4003075	4003076	MACS_peak_11	13.00	lobChIP_FOXA1_robot
chr1	4326094	4326095	MACS_peak_12	9.00	lobChIP_FOXA1_robot
chr1	4327594	4327595	MACS_peak_13	5.00	lobChIP_FOXA1_robot
chr1	5083613	5083614	MACS_peak_14	4.00	lobChIP_FOXA1_robot
chr1	5990891	5990892	MACS_peak_15	5.00	lobChIP_FOXA1_robot
chr1	6009313	6009314	MACS_peak_16	9.00	lobChIP_FOXA1_robot
chr1	6015742	6015743	MACS_peak_17	7.00	lobChIP_FOXA1_robot
chr1	6300385	6300386	MACS_peak_18	9.00	lobChIP_FOXA1_robot
chr1	6642563	6642564	MACS_peak_19	7.00	lobChIP_FOXA1_robot
chr1	6970099	6970100	MACS_peak_20	7.00	lobChIP_FOXA1_robot
chr1	7707446	7707447	MACS_peak_21	7.00	lobChIP_FOXA1_robot
chr1	8066577	8066578	MACS_peak_22	4.00	lobChIP_FOXA1_robot
chr1	8087624	8087625	MACS_peak_23	11.00	lobChIP_FOXA1_robot
chr1	8091438	8091439	MACS_peak_24	4.00	lobChIP_FOXA1_robot
chr1	8100269	8100270	MACS_peak_25	3.00	lobChIP_FOXA1_robot
chr1	8100854	8100855	MACS_peak_26	6.00	lobChIP_FOXA1_robot
chr1	8121524	8121525	MACS_peak_27	11.00	lobChIP_FOXA1_robot
chr1	8144433	8144434	MACS_peak_28	19.00	lobChIP_FOXA1_robot
chr1	8155108	8155109	MACS_peak_29	14.00	lobChIP_FOXA1_robot
chr1	8177182	8177183	MACS_peak_30	9.00	lobChIP_FOXA1_robot
chr1	8181130	8181131	MACS_peak_31	8.00	lobChIP_FOXA1_robot
chr1	8214829	8214830	MACS_peak_32	5.00	lobChIP_FOXA1_robot
chr1	8215651	8215652	MACS_peak_33	8.00	lobChIP_FOXA1_robot
chr1	8242634	8242635	MACS_peak_34	14.00	lobChIP_FOXA1_robot
chr1	8250247	8250248	MACS_peak_35	8.00	lobChIP_FOXA1_robot
chr1	8254813	8254814	MACS_peak_36	5.00	lobChIP_FOXA1_robot
chr1	8260499	8260500	MACS_peak_37	12.00	lobChIP_FOXA1_robot
chr1	8272348	8272349	MACS_peak_38	10.00	lobChIP_FOXA1_robot
chr1	8301811	8301812	MACS_peak_39	4.00	lobChIP_FOXA1_robot
chr1	8319389	8319390	MACS_peak_40	41.00	lobChIP_FOXA1_robot
chr1	8372364	8372365	MACS_peak_41	6.00	lobChIP_FOXA1_robot
chr1	8611949	8611950	MACS_peak_42	5.00	lobChIP_FOXA1_robot
chr1	8624273	8624274	MACS_peak_43	4.00	lobChIP_FOXA1_robot
chr1	8625007	8625008	MACS_peak_44	5.00	lobChIP_FOXA1_robot
chr1	8631761	8631762	MACS_peak_45	14.00	lobChIP_FOXA1_robot
chr1	8686348	8686349	MACS_peak_46	3.00	lobChIP_FOXA1_robot
chr1	8704118	8704119	MACS_peak_47	7.00	lobChIP_FOXA1_robot
chr1	8724109	8724110	MACS_peak_48	5.00	lobChIP_FOXA1_robot
chr1	8728546	8728547	MACS_peak_49	4.00	lobChIP_FOXA1_robot
chr1	8730480	8730481	MACS_peak_50	6.00	lobChIP_FOXA1_robot
chr1	8741610	8741611	MACS_peak_51	9.00	lobChIP_FOXA1_robot
chr1	8762445	8762446	MACS_peak_52	8.00	lobChIP_FOXA1_robot
chr1	8772335	8772336	MACS_peak_53	10.00	lobChIP_FOXA1_robot
chr1	8800329	8800330	MACS_peak_54	8.00	lobChIP_FOXA1_robot
chr1	8802251	8802252	MACS_peak_55	106.00	lobChIP_FOXA1_robot
chr1	8806241	8806242	MACS_peak_56	4.00	lobChIP_FOXA1_robot
chr1	8940320	8940321	MACS_peak_57	5.00	lobChIP_FOXA1_robot
chr1	8950415	8950416	MACS_peak_58	6.00	lobChIP_FOXA1_robot
chr1	9061958	9061959	MACS_peak_59	4.00	lobChIP_FOXA1_robot
chr1	9173338	9173339	MACS_peak_60	9.00	lobChIP_FOXA1_robot
chr1	9254322	9254323	MACS_peak_61	10.00	lobChIP_FOXA1_robot
chr1	9495346	9495347	MACS_peak_62	30.00	lobChIP_FOXA1_robot
chr1	9496533	9496534	MACS_peak_63	6.00	lobChIP_FOXA1_robot
chr1	9498643	9498644	MACS_peak_64	5.00	lobChIP_FOXA1_robot
chr1	9499640	9499641	MACS_peak_65	4.00	lobChIP_FOXA1_robot
chr1	9598128	9598129	MACS_peak_66	5.00	lobChIP_FOXA1_robot
chr1	9610452	9610453	MACS_peak_67	26.00	lobChIP_FOXA1_robot
chr1	9614054	9614055	MACS_peak_68	14.00	lobChIP_FOXA1_robot
chr1	9974560	9974561	MACS_peak_69	17.00	lobChIP_FOXA1_robot
chr1	9989674	9989675	MACS_peak_70	10.00	lobChIP_FOXA1_robot
chr1	10116531	10116532	MACS_peak_71	11.00	lobChIP_FOXA1_robot
chr1	10229705	10229706	MACS_peak_72	7.00	lobChIP_FOXA1_robot
chr1	10231305	10231306	MACS_peak_73	4.00	lobChIP_FOXA1_robot
chr1	10550002	10550003	MACS_peak_74	16.00	lobChIP_FOXA1_robot
chr1	10659818	10659819	MACS_peak_75	7.00	lobChIP_FOXA1_robot
chr1	11327889	11327890	MACS_peak_76	5.00	lobChIP_FOXA1_robot
chr1	11329936	11329937	MACS_peak_77	5.00	lobChIP_FOXA1_robot
chr1	11903043	11903044	MACS_peak_78	8.00	lobChIP_FOXA1_robot
chr1	11968433	11968434	MACS_peak_79	5.00	lobChIP_FOXA1_robot
chr1	11969808	11969809	MACS_peak_80	9.00	lobChIP_FOXA1_robot
chr1	12416230	12416231	MACS_peak_81	6.00	lobChIP_FOXA1_robot
chr1	12440550	12440551	MACS_peak_82	6.00	lobChIP_FOXA1_robot
chr1	12441432	12441433	MACS_peak_83	44.00	lobChIP_FOXA1_robot
chr1	12462481	12462482	MACS_peak_84	44.00	lobChIP_FOXA1_robot
chr1	12465784	12465785	MACS_peak_85	4.00	lobChIP_FOXA1_robot
chr1	12513701	12513702	MACS_peak_86	5.00	lobChIP_FOXA1_robot
chr1	12536318	12536319	MACS_peak_87	3.00	lobChIP_FOXA1_robot
chr1	12585576	12585577	MACS_peak_88	4.00	lobChIP_FOXA1_robot
chr1	12588530	12588531	MACS_peak_89	31.00	lobChIP_FOXA1_robot
chr1	12589598	12589599	MACS_peak_90	18.00	lobChIP_FOXA1_robot
chr1	12609944	12609945	MACS_peak_91	4.00	lobChIP_FOXA1_robot
chr1	12648395	12648396	MACS_peak_92	5.00	lobChIP_FOXA1_robot
chr1	12664551	12664552	MACS_peak_93	12.00	lobChIP_FOXA1_robot
chr1	12668729	12668730	MACS_peak_94	6.00	lobChIP_FOXA1_robot
chr1	12674603	12674604	MACS_peak_95	9.00	lobChIP_FOXA1_robot
chr1	12676102	12676103	MACS_peak_96	41.00	lobChIP_FOXA1_robot
chr1	12714835	12714836	MACS_peak_97	8.00	lobChIP_FOXA1_robot
chr1	14862336	14862337	MACS_peak_98	4.00	lobChIP_FOXA1_robot
chr1	15348005	15348006	MACS_peak_99	4.00	lobChIP_FOXA1_robot
chr1	15472050	15472051	MACS_peak_100	10.00	lobChIP_FOXA1_robot
chr1	15524477	15524478	MACS_peak_101	5.00	lobChIP_FOXA1_robot
chr1	15611592	15611593	MACS_peak_102	12.00	lobChIP_FOXA1_robot
chr1	15680586	15680587	MACS_peak_103	6.00	lobChIP_FOXA1_robot
chr1	15684519	15684520	MACS_peak_104	6.00	lobChIP_FOXA1_robot
chr1	15932385	15932386	MACS_peak_105	4.00	lobChIP_FOXA1_robot
chr1	15945408	15945409	MACS_peak_106	5.00	lobChIP_FOXA1_robot
chr1	15946948	15946949	MACS_peak_107	5.00	lobChIP_FOXA1_robot
chr1	16107299	16107300	MACS_peak_108	5.00	lobChIP_FOXA1_robot
chr1	16158600	16158601	MACS_peak_109	11.00	lobChIP_FOXA1_robot
chr1	16330599	16330600	MACS_peak_110	13.00	lobChIP_FOXA1_robot
chr1	16447717	16447718	MACS_peak_111	7.00	lobChIP_FOXA1_robot
chr1	16490830	16490831	MACS_peak_112	5.00	lobChIP_FOXA1_robot
chr1	16505403	16505404	MACS_peak_113	4.00	lobChIP_FOXA1_robot
chr1	16661328	16661329	MACS_peak_114	3.00	lobChIP_FOXA1_robot
chr1	16934918	16934919	MACS_peak_115	12.00	lobChIP_FOXA1_robot
chr1	17908165	17908166	MACS_peak_116	14.00	lobChIP_FOXA1_robot
chr1	17918756	17918757	MACS_peak_117	10.00	lobChIP_FOXA1_robot
chr1	18967578	18967579	MACS_peak_118	5.00	lobChIP_FOXA1_robot
chr1	18973373	18973374	MACS_peak_119	5.00	lobChIP_FOXA1_robot
chr1	19315460	19315461	MACS_peak_120	8.00	lobChIP_FOXA1_robot
chr1	19379098	19379099	MACS_peak_121	5.00	lobChIP_FOXA1_robot
chr1	19413984	19413985	MACS_peak_122	6.00	lobChIP_FOXA1_robot
chr1	19465713	19465714	MACS_peak_123	5.00	lobChIP_FOXA1_robot
chr1	19736450	19736451	MACS_peak_124	24.00	lobChIP_FOXA1_robot
chr1	19739311	19739312	MACS_peak_125	5.00	lobChIP_FOXA1_robot
chr1	19751508	19751509	MACS_peak_126	3.00	lobChIP_FOXA1_robot
chr1	19753862	19753863	MACS_peak_127	7.00	lobChIP_FOXA1_robot
chr1	19763200	19763201	MACS_peak_128	3.00	lobChIP_FOXA1_robot
chr1	19772503	19772504	MACS_peak_129	7.00	lobChIP_FOXA1_robot
chr1	19841183	19841184	MACS_peak_130	4.00	lobChIP_FOXA1_robot
chr1	19874459	19874460	MACS_peak_131	13.00	lobChIP_FOXA1_robot
chr1	19946484	19946485	MACS_peak_132	15.00	lobChIP_FOXA1_robot
chr1	20253360	20253361	MACS_peak_133	5.00	lobChIP_FOXA1_robot
chr1	20273126	20273127	MACS_peak_134	10.00	lobChIP_FOXA1_robot
chr1	20301606	20301607	MACS_peak_135	6.00	lobChIP_FOXA1_robot
chr1	20307019	20307020	MACS_peak_136	6.00	lobChIP_FOXA1_robot
chr1	20314407	20314408	MACS_peak_137	27.00	lobChIP_FOXA1_robot
chr1	20577043	20577044	MACS_peak_138	5.00	lobChIP_FOXA1_robot
chr1	20809280	20809281	MACS_peak_139	3.00	lobChIP_FOXA1_robot
chr1	21111417	21111418	MACS_peak_140	4.00	lobChIP_FOXA1_robot
chr1	21223500	21223501	MACS_peak_141	6.00	lobChIP_FOXA1_robot
chr1	21348936	21348937	MACS_peak_142	4.00	lobChIP_FOXA1_robot
chr1	21452944	21452945	MACS_peak_143	4.00	lobChIP_FOXA1_robot
chr1	21578557	21578558	MACS_peak_144	13.00	lobChIP_FOXA1_robot
chr1	21603118	21603119	MACS_peak_145	4.00	lobChIP_FOXA1_robot
chr1	21665315	21665316	MACS_peak_146	7.00	lobChIP_FOXA1_robot
chr1	21950521	21950522	MACS_peak_147	5.00	lobChIP_FOXA1_robot
chr1	21993548	21993549	MACS_peak_148	8.00	lobChIP_FOXA1_robot
chr1	22032271	22032272	MACS_peak_149	6.00	lobChIP_FOXA1_robot
chr1	22102735	22102736	MACS_peak_150	27.00	lobChIP_FOXA1_robot
chr1	22257758	22257759	MACS_peak_151	7.00	lobChIP_FOXA1_robot
chr1	22262143	22262144	MACS_peak_152	4.00	lobChIP_FOXA1_robot
chr1	22299664	22299665	MACS_peak_153	8.00	lobChIP_FOXA1_robot
chr1	22324556	22324557	MACS_peak_154	11.00	lobChIP_FOXA1_robot
chr1	22675059	22675060	MACS_peak_155	33.00	lobChIP_FOXA1_robot
chr1	22739390	22739391	MACS_peak_156	4.00	lobChIP_FOXA1_robot
chr1	22740862	22740863	MACS_peak_157	38.00	lobChIP_FOXA1_robot
chr1	22778435	22778436	MACS_peak_158	5.00	lobChIP_FOXA1_robot
chr1	23366760	23366761	MACS_peak_159	6.00	lobChIP_FOXA1_robot
chr1	23441801	23441802	MACS_peak_160	19.00	lobChIP_FOXA1_robot
chr1	23475777	23475778	MACS_peak_161	4.00	lobChIP_FOXA1_robot
chr1	23514471	23514472	MACS_peak_162	13.00	lobChIP_FOXA1_robot
chr1	23550064	23550065	MACS_peak_163	6.00	lobChIP_FOXA1_robot
chr1	23618137	23618138	MACS_peak_164	4.00	lobChIP_FOXA1_robot
chr1	23704014	23704015	MACS_peak_165	4.00	lobChIP_FOXA1_robot
chr1	23724558	23724559	MACS_peak_166	4.00	lobChIP_FOXA1_robot
chr1	23785921	23785922	MACS_peak_167	7.00	lobChIP_FOXA1_robot
chr1	23824532	23824533	MACS_peak_168	4.00	lobChIP_FOXA1_robot
chr1	23888889	23888890	MACS_peak_169	10.00	lobChIP_FOXA1_robot
chr1	23890533	23890534	MACS_peak_170	5.00	lobChIP_FOXA1_robot
chr1	23903852	23903853	MACS_peak_171	7.00	lobChIP_FOXA1_robot
chr1	23918132	23918133	MACS_peak_172	10.00	lobChIP_FOXA1_robot
chr1	24054056	24054057	MACS_peak_173	6.00	lobChIP_FOXA1_robot
chr1	24279722	24279723	MACS_peak_174	5.00	lobChIP_FOXA1_robot
chr1	24336074	24336075	MACS_peak_175	4.00	lobChIP_FOXA1_robot
chr1	24345425	24345426	MACS_peak_176	4.00	lobChIP_FOXA1_robot
chr1	24348119	24348120	MACS_peak_177	10.00	lobChIP_FOXA1_robot
chr1	24445000	24445001	MACS_peak_178	10.00	lobChIP_FOXA1_robot
chr1	24867693	24867694	MACS_peak_179	29.00	lobChIP_FOXA1_robot
chr1	24875809	24875810	MACS_peak_180	4.00	lobChIP_FOXA1_robot
chr1	24885739	24885740	MACS_peak_181	11.00	lobChIP_FOXA1_robot
chr1	24894674	24894675	MACS_peak_182	7.00	lobChIP_FOXA1_robot
chr1	25032412	25032413	MACS_peak_183	13.00	lobChIP_FOXA1_robot
chr1	25094050	25094051	MACS_peak_184	8.00	lobChIP_FOXA1_robot
chr1	25237749	25237750	MACS_peak_185	3.00	lobChIP_FOXA1_robot
chr1	25453360	25453361	MACS_peak_186	4.00	lobChIP_FOXA1_robot
chr1	25494599	25494600	MACS_peak_187	3.00	lobChIP_FOXA1_robot
chr1	25495159	25495160	MACS_peak_188	36.00	lobChIP_FOXA1_robot
chr1	25500995	25500996	MACS_peak_189	9.00	lobChIP_FOXA1_robot
chr1	25532188	25532189	MACS_peak_190	5.00	lobChIP_FOXA1_robot
chr1	25758793	25758794	MACS_peak_191	5.00	lobChIP_FOXA1_robot
chr1	26197621	26197622	MACS_peak_192	5.00	lobChIP_FOXA1_robot
chr1	26324046	26324047	MACS_peak_193	4.00	lobChIP_FOXA1_robot
chr1	26677425	26677426	MACS_peak_194	7.00	lobChIP_FOXA1_robot
chr1	27017411	27017412	MACS_peak_195	4.00	lobChIP_FOXA1_robot
chr1	27110816	27110817	MACS_peak_196	3.00	lobChIP_FOXA1_robot
chr1	27158392	27158393	MACS_peak_197	11.00	lobChIP_FOXA1_robot
chr1	27240900	27240901	MACS_peak_198	26.00	lobChIP_FOXA1_robot
chr1	27288553	27288554	MACS_peak_199	3.00	lobChIP_FOXA1_robot
chr1	27319544	27319545	MACS_peak_200	3.00	lobChIP_FOXA1_robot
chr1	27490054	27490055	MACS_peak_201	3.00	lobChIP_FOXA1_robot
chr1	27518648	27518649	MACS_peak_202	4.00	lobChIP_FOXA1_robot
chr1	27869062	27869063	MACS_peak_203	3.00	lobChIP_FOXA1_robot
chr1	27911788	27911789	MACS_peak_204	5.00	lobChIP_FOXA1_robot
chr1	28143818	28143819	MACS_peak_205	5.00	lobChIP_FOXA1_robot
chr1	28306357	28306358	MACS_peak_206	8.00	lobChIP_FOXA1_robot
chr1	28479829	28479830	MACS_peak_207	5.00	lobChIP_FOXA1_robot
chr1	28496785	28496786	MACS_peak_208	7.00	lobChIP_FOXA1_robot
chr1	28573084	28573085	MACS_peak_209	4.00	lobChIP_FOXA1_robot
chr1	28622631	28622632	MACS_peak_210	9.00	lobChIP_FOXA1_robot
chr1	28672927	28672928	MACS_peak_211	6.00	lobChIP_FOXA1_robot
chr1	28834811	28834812	MACS_peak_212	5.00	lobChIP_FOXA1_robot
chr1	28845490	28845491	MACS_peak_213	6.00	lobChIP_FOXA1_robot
chr1	28933612	28933613	MACS_peak_214	3.00	lobChIP_FOXA1_robot
chr1	28961039	28961040	MACS_peak_215	30.00	lobChIP_FOXA1_robot
chr1	28975211	28975212	MACS_peak_216	5.00	lobChIP_FOXA1_robot
chr1	29253568	29253569	MACS_peak_217	6.00	lobChIP_FOXA1_robot
chr1	29293046	29293047	MACS_peak_218	3.00	lobChIP_FOXA1_robot
chr1	29310075	29310076	MACS_peak_219	5.00	lobChIP_FOXA1_robot
chr1	29432636	29432637	MACS_peak_220	8.00	lobChIP_FOXA1_robot
chr1	29472641	29472642	MACS_peak_221	4.00	lobChIP_FOXA1_robot
chr1	29512849	29512850	MACS_peak_222	4.00	lobChIP_FOXA1_robot
chr1	30702600	30702601	MACS_peak_223	3.00	lobChIP_FOXA1_robot
chr1	31457080	31457081	MACS_peak_224	3.00	lobChIP_FOXA1_robot
chr1	31472620	31472621	MACS_peak_225	4.00	lobChIP_FOXA1_robot
chr1	31513081	31513082	MACS_peak_226	4.00	lobChIP_FOXA1_robot
chr1	31537018	31537019	MACS_peak_227	29.00	lobChIP_FOXA1_robot
chr1	31642861	31642862	MACS_peak_228	5.00	lobChIP_FOXA1_robot
chr1	31920219	31920220	MACS_peak_229	6.00	lobChIP_FOXA1_robot
chr1	31922751	31922752	MACS_peak_230	10.00	lobChIP_FOXA1_robot
chr1	31974993	31974994	MACS_peak_231	4.00	lobChIP_FOXA1_robot
chr1	31977714	31977715	MACS_peak_232	5.00	lobChIP_FOXA1_robot
chr1	32038431	32038432	MACS_peak_233	4.00	lobChIP_FOXA1_robot
chr1	32346727	32346728	MACS_peak_234	4.00	lobChIP_FOXA1_robot
chr1	32644146	32644147	MACS_peak_235	5.00	lobChIP_FOXA1_robot
chr1	32775751	32775752	MACS_peak_236	8.00	lobChIP_FOXA1_robot
chr1	32777495	32777496	MACS_peak_237	4.00	lobChIP_FOXA1_robot
chr1	32787510	32787511	MACS_peak_238	40.00	lobChIP_FOXA1_robot
chr1	33722607	33722608	MACS_peak_239	8.00	lobChIP_FOXA1_robot
chr1	33724640	33724641	MACS_peak_240	10.00	lobChIP_FOXA1_robot
chr1	33808061	33808062	MACS_peak_241	20.00	lobChIP_FOXA1_robot
chr1	33813004	33813005	MACS_peak_242	12.00	lobChIP_FOXA1_robot
chr1	34535491	34535492	MACS_peak_243	3.00	lobChIP_FOXA1_robot
chr1	35048125	35048126	MACS_peak_244	7.00	lobChIP_FOXA1_robot
chr1	35458259	35458260	MACS_peak_245	4.00	lobChIP_FOXA1_robot
chr1	35576899	35576900	MACS_peak_246	8.00	lobChIP_FOXA1_robot
chr1	35648523	35648524	MACS_peak_247	4.00	lobChIP_FOXA1_robot
chr1	36021940	36021941	MACS_peak_248	11.00	lobChIP_FOXA1_robot
chr1	36275130	36275131	MACS_peak_249	6.00	lobChIP_FOXA1_robot
chr1	36705595	36705596	MACS_peak_250	5.00	lobChIP_FOXA1_robot
chr1	36714406	36714407	MACS_peak_251	9.00	lobChIP_FOXA1_robot
chr1	36735129	36735130	MACS_peak_252	46.00	lobChIP_FOXA1_robot
chr1	36845875	36845876	MACS_peak_253	5.00	lobChIP_FOXA1_robot
chr1	36952785	36952786	MACS_peak_254	15.00	lobChIP_FOXA1_robot
chr1	38000289	38000290	MACS_peak_255	12.00	lobChIP_FOXA1_robot
chr1	38245790	38245791	MACS_peak_256	5.00	lobChIP_FOXA1_robot
chr1	38328205	38328206	MACS_peak_257	5.00	lobChIP_FOXA1_robot
chr1	38380686	38380687	MACS_peak_258	8.00	lobChIP_FOXA1_robot
chr1	38731998	38731999	MACS_peak_259	9.00	lobChIP_FOXA1_robot
chr1	39404563	39404564	MACS_peak_260	5.00	lobChIP_FOXA1_robot
chr1	39434929	39434930	MACS_peak_261	44.00	lobChIP_FOXA1_robot
chr1	39451750	39451751	MACS_peak_262	8.00	lobChIP_FOXA1_robot
chr1	39452447	39452448	MACS_peak_263	13.00	lobChIP_FOXA1_robot
chr1	39458592	39458593	MACS_peak_264	6.00	lobChIP_FOXA1_robot
chr1	39482351	39482352	MACS_peak_265	11.00	lobChIP_FOXA1_robot
chr1	39558155	39558156	MACS_peak_266	7.00	lobChIP_FOXA1_robot
chr1	39572508	39572509	MACS_peak_267	4.00	lobChIP_FOXA1_robot
chr1	39583062	39583063	MACS_peak_268	9.00	lobChIP_FOXA1_robot
chr1	39624106	39624107	MACS_peak_269	6.00	lobChIP_FOXA1_robot
chr1	39644680	39644681	MACS_peak_270	5.00	lobChIP_FOXA1_robot
chr1	39680057	39680058	MACS_peak_271	12.00	lobChIP_FOXA1_robot
chr1	39857941	39857942	MACS_peak_272	5.00	lobChIP_FOXA1_robot
chr1	40265863	40265864	MACS_peak_273	5.00	lobChIP_FOXA1_robot
chr1	40524476	40524477	MACS_peak_274	5.00	lobChIP_FOXA1_robot
chr1	40713591	40713592	MACS_peak_275	5.00	lobChIP_FOXA1_robot
chr1	40849936	40849937	MACS_peak_276	11.00	lobChIP_FOXA1_robot
chr1	40855928	40855929	MACS_peak_277	8.00	lobChIP_FOXA1_robot
chr1	40887936	40887937	MACS_peak_278	4.00	lobChIP_FOXA1_robot
chr1	41033708	41033709	MACS_peak_279	3.00	lobChIP_FOXA1_robot
chr1	41158753	41158754	MACS_peak_280	6.00	lobChIP_FOXA1_robot
chr1	41178178	41178179	MACS_peak_281	26.00	lobChIP_FOXA1_robot
chr1	41181555	41181556	MACS_peak_282	11.00	lobChIP_FOXA1_robot
chr1	41224350	41224351	MACS_peak_283	5.00	lobChIP_FOXA1_robot
chr1	41290659	41290660	MACS_peak_284	4.00	lobChIP_FOXA1_robot
chr1	41334168	41334169	MACS_peak_285	7.00	lobChIP_FOXA1_robot
chr1	41385257	41385258	MACS_peak_286	7.00	lobChIP_FOXA1_robot
chr1	41616763	41616764	MACS_peak_287	5.00	lobChIP_FOXA1_robot
chr1	41704264	41704265	MACS_peak_288	5.00	lobChIP_FOXA1_robot
chr1	41705877	41705878	MACS_peak_289	36.00	lobChIP_FOXA1_robot
chr1	42631619	42631620	MACS_peak_290	5.00	lobChIP_FOXA1_robot
chr1	42680431	42680432	MACS_peak_291	7.00	lobChIP_FOXA1_robot
chr1	42781301	42781302	MACS_peak_292	4.00	lobChIP_FOXA1_robot
chr1	42790869	42790870	MACS_peak_293	11.00	lobChIP_FOXA1_robot
chr1	42801735	42801736	MACS_peak_294	3.00	lobChIP_FOXA1_robot
chr1	42805970	42805971	MACS_peak_295	11.00	lobChIP_FOXA1_robot
chr1	43086168	43086169	MACS_peak_296	8.00	lobChIP_FOXA1_robot
chr1	43427559	43427560	MACS_peak_297	5.00	lobChIP_FOXA1_robot
chr1	43446382	43446383	MACS_peak_298	8.00	lobChIP_FOXA1_robot
chr1	43499966	43499967	MACS_peak_299	3.00	lobChIP_FOXA1_robot
chr1	43501859	43501860	MACS_peak_300	7.00	lobChIP_FOXA1_robot
chr1	43505208	43505209	MACS_peak_301	31.00	lobChIP_FOXA1_robot
chr1	43712743	43712744	MACS_peak_302	6.00	lobChIP_FOXA1_robot
chr1	43748229	43748230	MACS_peak_303	5.00	lobChIP_FOXA1_robot
chr1	43779889	43779890	MACS_peak_304	4.00	lobChIP_FOXA1_robot
chr1	43806616	43806617	MACS_peak_305	6.00	lobChIP_FOXA1_robot
chr1	44014203	44014204	MACS_peak_306	6.00	lobChIP_FOXA1_robot
chr1	44098108	44098109	MACS_peak_307	7.00	lobChIP_FOXA1_robot
chr1	44223349	44223350	MACS_peak_308	6.00	lobChIP_FOXA1_robot
chr1	44453062	44453063	MACS_peak_309	8.00	lobChIP_FOXA1_robot
chr1	44475534	44475535	MACS_peak_310	10.00	lobChIP_FOXA1_robot
chr1	44749396	44749397	MACS_peak_311	24.00	lobChIP_FOXA1_robot
chr1	44867245	44867246	MACS_peak_312	3.00	lobChIP_FOXA1_robot
chr1	45147963	45147964	MACS_peak_313	16.00	lobChIP_FOXA1_robot
chr1	45166405	45166406	MACS_peak_314	6.00	lobChIP_FOXA1_robot
chr1	45166985	45166986	MACS_peak_315	24.00	lobChIP_FOXA1_robot
chr1	45187168	45187169	MACS_peak_316	7.00	lobChIP_FOXA1_robot
chr1	45412598	45412599	MACS_peak_317	10.00	lobChIP_FOXA1_robot
chr1	45415094	45415095	MACS_peak_318	7.00	lobChIP_FOXA1_robot
chr1	45600235	45600236	MACS_peak_319	16.00	lobChIP_FOXA1_robot
chr1	45637898	45637899	MACS_peak_320	85.00	lobChIP_FOXA1_robot
chr1	45732139	45732140	MACS_peak_321	7.00	lobChIP_FOXA1_robot
chr1	45763847	45763848	MACS_peak_322	10.00	lobChIP_FOXA1_robot
chr1	45967925	45967926	MACS_peak_323	10.00	lobChIP_FOXA1_robot
chr1	45992038	45992039	MACS_peak_324	8.00	lobChIP_FOXA1_robot
chr1	46124005	46124006	MACS_peak_325	9.00	lobChIP_FOXA1_robot
chr1	46337983	46337984	MACS_peak_326	15.00	lobChIP_FOXA1_robot
chr1	46444921	46444922	MACS_peak_327	5.00	lobChIP_FOXA1_robot
chr1	46648985	46648986	MACS_peak_328	6.00	lobChIP_FOXA1_robot
chr1	47719949	47719950	MACS_peak_329	4.00	lobChIP_FOXA1_robot
chr1	47844038	47844039	MACS_peak_330	17.00	lobChIP_FOXA1_robot
chr1	47845419	47845420	MACS_peak_331	12.00	lobChIP_FOXA1_robot
chr1	47856687	47856688	MACS_peak_332	7.00	lobChIP_FOXA1_robot
chr1	47943942	47943943	MACS_peak_333	43.00	lobChIP_FOXA1_robot
chr1	48332832	48332833	MACS_peak_334	7.00	lobChIP_FOXA1_robot
chr1	48333470	48333471	MACS_peak_335	5.00	lobChIP_FOXA1_robot
chr1	48359357	48359358	MACS_peak_336	5.00	lobChIP_FOXA1_robot
chr1	48379186	48379187	MACS_peak_337	6.00	lobChIP_FOXA1_robot
chr1	48688428	48688429	MACS_peak_338	5.00	lobChIP_FOXA1_robot
chr1	48775340	48775341	MACS_peak_339	4.00	lobChIP_FOXA1_robot
chr1	50099458	50099459	MACS_peak_340	5.00	lobChIP_FOXA1_robot
chr1	51110596	51110597	MACS_peak_341	4.00	lobChIP_FOXA1_robot
chr1	51636206	51636207	MACS_peak_342	5.00	lobChIP_FOXA1_robot
chr1	52090690	52090691	MACS_peak_343	4.00	lobChIP_FOXA1_robot
chr1	52156539	52156540	MACS_peak_344	35.00	lobChIP_FOXA1_robot
chr1	52434433	52434434	MACS_peak_345	6.00	lobChIP_FOXA1_robot
chr1	52443057	52443058	MACS_peak_346	6.00	lobChIP_FOXA1_robot
chr1	52503353	52503354	MACS_peak_347	8.00	lobChIP_FOXA1_robot
chr1	52545949	52545950	MACS_peak_348	8.00	lobChIP_FOXA1_robot
chr1	52564024	52564025	MACS_peak_349	7.00	lobChIP_FOXA1_robot
chr1	52655978	52655979	MACS_peak_350	7.00	lobChIP_FOXA1_robot
chr1	52707180	52707181	MACS_peak_351	4.00	lobChIP_FOXA1_robot
chr1	52729327	52729328	MACS_peak_352	4.00	lobChIP_FOXA1_robot
chr1	52747428	52747429	MACS_peak_353	12.00	lobChIP_FOXA1_robot
chr1	52938945	52938946	MACS_peak_354	5.00	lobChIP_FOXA1_robot
chr1	53573471	53573472	MACS_peak_355	42.00	lobChIP_FOXA1_robot
chr1	53581759	53581760	MACS_peak_356	5.00	lobChIP_FOXA1_robot
chr1	53611694	53611695	MACS_peak_357	8.00	lobChIP_FOXA1_robot
chr1	53649310	53649311	MACS_peak_358	16.00	lobChIP_FOXA1_robot
chr1	54151077	54151078	MACS_peak_359	6.00	lobChIP_FOXA1_robot
chr1	54375700	54375701	MACS_peak_360	4.00	lobChIP_FOXA1_robot
chr1	54422585	54422586	MACS_peak_361	14.00	lobChIP_FOXA1_robot
chr1	54423224	54423225	MACS_peak_362	3.00	lobChIP_FOXA1_robot
chr1	54433972	54433973	MACS_peak_363	4.00	lobChIP_FOXA1_robot
chr1	54482221	54482222	MACS_peak_364	5.00	lobChIP_FOXA1_robot
chr1	54536388	54536389	MACS_peak_365	4.00	lobChIP_FOXA1_robot
chr1	54849214	54849215	MACS_peak_366	4.00	lobChIP_FOXA1_robot
chr1	54873476	54873477	MACS_peak_367	7.00	lobChIP_FOXA1_robot
chr1	54890905	54890906	MACS_peak_368	5.00	lobChIP_FOXA1_robot
chr1	55326806	55326807	MACS_peak_369	4.00	lobChIP_FOXA1_robot
chr1	55332987	55332988	MACS_peak_370	4.00	lobChIP_FOXA1_robot
chr1	55335071	55335072	MACS_peak_371	11.00	lobChIP_FOXA1_robot
chr1	55348354	55348355	MACS_peak_372	6.00	lobChIP_FOXA1_robot
chr1	55350008	55350009	MACS_peak_373	5.00	lobChIP_FOXA1_robot
chr1	55354263	55354264	MACS_peak_374	54.00	lobChIP_FOXA1_robot
chr1	55374493	55374494	MACS_peak_375	5.00	lobChIP_FOXA1_robot
chr1	55594193	55594194	MACS_peak_376	5.00	lobChIP_FOXA1_robot
chr1	55677344	55677345	MACS_peak_377	10.00	lobChIP_FOXA1_robot
chr1	55894992	55894993	MACS_peak_378	19.00	lobChIP_FOXA1_robot
chr1	55913314	55913315	MACS_peak_379	5.00	lobChIP_FOXA1_robot
chr1	56000592	56000593	MACS_peak_380	6.00	lobChIP_FOXA1_robot
chr1	56638493	56638494	MACS_peak_381	3.00	lobChIP_FOXA1_robot
chr1	56655980	56655981	MACS_peak_382	9.00	lobChIP_FOXA1_robot
chr1	56763770	56763771	MACS_peak_383	14.00	lobChIP_FOXA1_robot
chr1	56797618	56797619	MACS_peak_384	11.00	lobChIP_FOXA1_robot
chr1	56880593	56880594	MACS_peak_385	24.00	lobChIP_FOXA1_robot
chr1	56927109	56927110	MACS_peak_386	4.00	lobChIP_FOXA1_robot
chr1	56948273	56948274	MACS_peak_387	5.00	lobChIP_FOXA1_robot
chr1	56960256	56960257	MACS_peak_388	18.00	lobChIP_FOXA1_robot
chr1	56974379	56974380	MACS_peak_389	6.00	lobChIP_FOXA1_robot
chr1	57001907	57001908	MACS_peak_390	12.00	lobChIP_FOXA1_robot
chr1	57005242	57005243	MACS_peak_391	4.00	lobChIP_FOXA1_robot
chr1	57032116	57032117	MACS_peak_392	4.00	lobChIP_FOXA1_robot
chr1	57035451	57035452	MACS_peak_393	17.00	lobChIP_FOXA1_robot
chr1	57044112	57044113	MACS_peak_394	3.00	lobChIP_FOXA1_robot
chr1	57105115	57105116	MACS_peak_395	19.00	lobChIP_FOXA1_robot
chr1	57109589	57109590	MACS_peak_396	5.00	lobChIP_FOXA1_robot
chr1	57113024	57113025	MACS_peak_397	5.00	lobChIP_FOXA1_robot
chr1	57154374	57154375	MACS_peak_398	10.00	lobChIP_FOXA1_robot
chr1	57220972	57220973	MACS_peak_399	6.00	lobChIP_FOXA1_robot
chr1	57253993	57253994	MACS_peak_400	5.00	lobChIP_FOXA1_robot
chr1	57287256	57287257	MACS_peak_401	6.00	lobChIP_FOXA1_robot
chr1	57298267	57298268	MACS_peak_402	4.00	lobChIP_FOXA1_robot
chr1	57320346	57320347	MACS_peak_403	11.00	lobChIP_FOXA1_robot
chr1	57356101	57356102	MACS_peak_404	6.00	lobChIP_FOXA1_robot
chr1	57376384	57376385	MACS_peak_405	5.00	lobChIP_FOXA1_robot
chr1	57385486	57385487	MACS_peak_406	11.00	lobChIP_FOXA1_robot
chr1	57388146	57388147	MACS_peak_407	6.00	lobChIP_FOXA1_robot
chr1	57431742	57431743	MACS_peak_408	6.00	lobChIP_FOXA1_robot
chr1	57433701	57433702	MACS_peak_409	9.00	lobChIP_FOXA1_robot
chr1	57451668	57451669	MACS_peak_410	5.00	lobChIP_FOXA1_robot
chr1	57526367	57526368	MACS_peak_411	7.00	lobChIP_FOXA1_robot
chr1	57527237	57527238	MACS_peak_412	4.00	lobChIP_FOXA1_robot
chr1	57745995	57745996	MACS_peak_413	33.00	lobChIP_FOXA1_robot
chr1	57747084	57747085	MACS_peak_414	4.00	lobChIP_FOXA1_robot
chr1	57930558	57930559	MACS_peak_415	33.00	lobChIP_FOXA1_robot
chr1	58555806	58555807	MACS_peak_416	6.00	lobChIP_FOXA1_robot
chr1	59196910	59196911	MACS_peak_417	6.00	lobChIP_FOXA1_robot
chr1	59218744	59218745	MACS_peak_418	4.00	lobChIP_FOXA1_robot
chr1	59280968	59280969	MACS_peak_419	7.00	lobChIP_FOXA1_robot
chr1	59351165	59351166	MACS_peak_420	55.00	lobChIP_FOXA1_robot
chr1	59370118	59370119	MACS_peak_421	3.00	lobChIP_FOXA1_robot
chr1	59518123	59518124	MACS_peak_422	34.00	lobChIP_FOXA1_robot
chr1	59537202	59537203	MACS_peak_423	4.00	lobChIP_FOXA1_robot
chr1	59649841	59649842	MACS_peak_424	5.00	lobChIP_FOXA1_robot
chr1	59680596	59680597	MACS_peak_425	17.00	lobChIP_FOXA1_robot
chr1	59812428	59812429	MACS_peak_426	6.00	lobChIP_FOXA1_robot
chr1	59874386	59874387	MACS_peak_427	4.00	lobChIP_FOXA1_robot
chr1	59892375	59892376	MACS_peak_428	5.00	lobChIP_FOXA1_robot
chr1	59965963	59965964	MACS_peak_429	4.00	lobChIP_FOXA1_robot
chr1	59977917	59977918	MACS_peak_430	10.00	lobChIP_FOXA1_robot
chr1	60015709	60015710	MACS_peak_431	10.00	lobChIP_FOXA1_robot
chr1	60022672	60022673	MACS_peak_432	6.00	lobChIP_FOXA1_robot
chr1	60109427	60109428	MACS_peak_433	6.00	lobChIP_FOXA1_robot
chr1	60123769	60123770	MACS_peak_434	8.00	lobChIP_FOXA1_robot
chr1	60135684	60135685	MACS_peak_435	3.00	lobChIP_FOXA1_robot
chr1	60140545	60140546	MACS_peak_436	5.00	lobChIP_FOXA1_robot
chr1	60204077	60204078	MACS_peak_437	6.00	lobChIP_FOXA1_robot
chr1	60322554	60322555	MACS_peak_438	11.00	lobChIP_FOXA1_robot
chr1	60402350	60402351	MACS_peak_439	9.00	lobChIP_FOXA1_robot
chr1	61406555	61406556	MACS_peak_440	4.00	lobChIP_FOXA1_robot
chr1	61521194	61521195	MACS_peak_441	4.00	lobChIP_FOXA1_robot
chr1	61555232	61555233	MACS_peak_442	20.00	lobChIP_FOXA1_robot
chr1	61575121	61575122	MACS_peak_443	4.00	lobChIP_FOXA1_robot
chr1	61580399	61580400	MACS_peak_444	19.00	lobChIP_FOXA1_robot
chr1	61584968	61584969	MACS_peak_445	4.00	lobChIP_FOXA1_robot
chr1	61586727	61586728	MACS_peak_446	5.00	lobChIP_FOXA1_robot
chr1	61588248	61588249	MACS_peak_447	8.00	lobChIP_FOXA1_robot
chr1	61598898	61598899	MACS_peak_448	8.00	lobChIP_FOXA1_robot
chr1	61623588	61623589	MACS_peak_449	5.00	lobChIP_FOXA1_robot
chr1	61649978	61649979	MACS_peak_450	4.00	lobChIP_FOXA1_robot
chr1	61765334	61765335	MACS_peak_451	13.00	lobChIP_FOXA1_robot
chr1	61767735	61767736	MACS_peak_452	3.00	lobChIP_FOXA1_robot
chr1	61788653	61788654	MACS_peak_453	4.00	lobChIP_FOXA1_robot
chr1	61803767	61803768	MACS_peak_454	8.00	lobChIP_FOXA1_robot
chr1	61804746	61804747	MACS_peak_455	10.00	lobChIP_FOXA1_robot
chr1	62218686	62218687	MACS_peak_456	4.00	lobChIP_FOXA1_robot
chr1	62407648	62407649	MACS_peak_457	7.00	lobChIP_FOXA1_robot
chr1	62416268	62416269	MACS_peak_458	4.00	lobChIP_FOXA1_robot
chr1	62707523	62707524	MACS_peak_459	26.00	lobChIP_FOXA1_robot
chr1	62852637	62852638	MACS_peak_460	7.00	lobChIP_FOXA1_robot
chr1	62989691	62989692	MACS_peak_461	7.00	lobChIP_FOXA1_robot
chr1	63010398	63010399	MACS_peak_462	4.00	lobChIP_FOXA1_robot
chr1	63057290	63057291	MACS_peak_463	6.00	lobChIP_FOXA1_robot
chr1	63087706	63087707	MACS_peak_464	12.00	lobChIP_FOXA1_robot
chr1	63138091	63138092	MACS_peak_465	4.00	lobChIP_FOXA1_robot
chr1	63156931	63156932	MACS_peak_466	3.00	lobChIP_FOXA1_robot
chr1	63199940	63199941	MACS_peak_467	10.00	lobChIP_FOXA1_robot
chr1	63358046	63358047	MACS_peak_468	4.00	lobChIP_FOXA1_robot
chr1	64000287	64000288	MACS_peak_469	4.00	lobChIP_FOXA1_robot
chr1	64107085	64107086	MACS_peak_470	13.00	lobChIP_FOXA1_robot
chr1	64140029	64140030	MACS_peak_471	5.00	lobChIP_FOXA1_robot
chr1	64140401	64140402	MACS_peak_472	5.00	lobChIP_FOXA1_robot
chr1	64158787	64158788	MACS_peak_473	5.00	lobChIP_FOXA1_robot
chr1	65061953	65061954	MACS_peak_474	28.00	lobChIP_FOXA1_robot
chr1	65364023	65364024	MACS_peak_475	5.00	lobChIP_FOXA1_robot
chr1	65372408	65372409	MACS_peak_476	4.00	lobChIP_FOXA1_robot
chr1	65376629	65376630	MACS_peak_477	7.00	lobChIP_FOXA1_robot
chr1	65381808	65381809	MACS_peak_478	15.00	lobChIP_FOXA1_robot
chr1	65453459	65453460	MACS_peak_479	7.00	lobChIP_FOXA1_robot
chr1	65516661	65516662	MACS_peak_480	4.00	lobChIP_FOXA1_robot
chr1	65573957	65573958	MACS_peak_481	4.00	lobChIP_FOXA1_robot
chr1	65624635	65624636	MACS_peak_482	27.00	lobChIP_FOXA1_robot
chr1	65644124	65644125	MACS_peak_483	6.00	lobChIP_FOXA1_robot
chr1	65933407	65933408	MACS_peak_484	7.00	lobChIP_FOXA1_robot
chr1	65937295	65937296	MACS_peak_485	6.00	lobChIP_FOXA1_robot
chr1	65950142	65950143	MACS_peak_486	15.00	lobChIP_FOXA1_robot
chr1	66153717	66153718	MACS_peak_487	8.00	lobChIP_FOXA1_robot
chr1	66613024	66613025	MACS_peak_488	4.00	lobChIP_FOXA1_robot
chr1	67009215	67009216	MACS_peak_489	7.00	lobChIP_FOXA1_robot
chr1	67170929	67170930	MACS_peak_490	5.00	lobChIP_FOXA1_robot
chr1	67446658	67446659	MACS_peak_491	5.00	lobChIP_FOXA1_robot
chr1	67518551	67518552	MACS_peak_492	4.00	lobChIP_FOXA1_robot
chr1	67526598	67526599	MACS_peak_493	3.00	lobChIP_FOXA1_robot
chr1	67833340	67833341	MACS_peak_494	5.00	lobChIP_FOXA1_robot
chr1	67979648	67979649	MACS_peak_495	4.00	lobChIP_FOXA1_robot
chr1	67998222	67998223	MACS_peak_496	3.00	lobChIP_FOXA1_robot
chr1	67999125	67999126	MACS_peak_497	7.00	lobChIP_FOXA1_robot
chr1	68000445	68000446	MACS_peak_498	36.00	lobChIP_FOXA1_robot
chr1	68044526	68044527	MACS_peak_499	4.00	lobChIP_FOXA1_robot
chr1	68129907	68129908	MACS_peak_500	12.00	lobChIP_FOXA1_robot
chr1	68163606	68163607	MACS_peak_501	4.00	lobChIP_FOXA1_robot
chr1	68166515	68166516	MACS_peak_502	5.00	lobChIP_FOXA1_robot
chr1	68204077	68204078	MACS_peak_503	3.00	lobChIP_FOXA1_robot
chr1	70672030	70672031	MACS_peak_504	4.00	lobChIP_FOXA1_robot
chr1	72448852	72448853	MACS_peak_505	4.00	lobChIP_FOXA1_robot
chr1	72948818	72948819	MACS_peak_506	13.00	lobChIP_FOXA1_robot
chr1	74822385	74822386	MACS_peak_507	7.00	lobChIP_FOXA1_robot
chr1	75155683	75155684	MACS_peak_508	22.00	lobChIP_FOXA1_robot
chr1	75276122	75276123	MACS_peak_509	6.00	lobChIP_FOXA1_robot
chr1	76201879	76201880	MACS_peak_510	5.00	lobChIP_FOXA1_robot
chr1	76525492	76525493	MACS_peak_511	5.00	lobChIP_FOXA1_robot
chr1	77762716	77762717	MACS_peak_512	5.00	lobChIP_FOXA1_robot
chr1	78123150	78123151	MACS_peak_513	4.00	lobChIP_FOXA1_robot
chr1	78472389	78472390	MACS_peak_514	7.00	lobChIP_FOXA1_robot
chr1	81348330	81348331	MACS_peak_515	10.00	lobChIP_FOXA1_robot
chr1	82314679	82314680	MACS_peak_516	16.00	lobChIP_FOXA1_robot
chr1	82930563	82930564	MACS_peak_517	5.00	lobChIP_FOXA1_robot
chr1	85186357	85186358	MACS_peak_518	10.00	lobChIP_FOXA1_robot
chr1	85204667	85204668	MACS_peak_519	6.00	lobChIP_FOXA1_robot
chr1	85663909	85663910	MACS_peak_520	3.00	lobChIP_FOXA1_robot
chr1	85668458	85668459	MACS_peak_521	4.00	lobChIP_FOXA1_robot
chr1	85794936	85794937	MACS_peak_522	5.00	lobChIP_FOXA1_robot
chr1	85956145	85956146	MACS_peak_523	5.00	lobChIP_FOXA1_robot
chr1	85968015	85968016	MACS_peak_524	5.00	lobChIP_FOXA1_robot
chr1	86073208	86073209	MACS_peak_525	3.00	lobChIP_FOXA1_robot
chr1	86095474	86095475	MACS_peak_526	6.00	lobChIP_FOXA1_robot
chr1	86146913	86146914	MACS_peak_527	6.00	lobChIP_FOXA1_robot
chr1	86175307	86175308	MACS_peak_528	4.00	lobChIP_FOXA1_robot
chr1	87224436	87224437	MACS_peak_529	4.00	lobChIP_FOXA1_robot
chr1	87240433	87240434	MACS_peak_530	40.00	lobChIP_FOXA1_robot
chr1	87248385	87248386	MACS_peak_531	7.00	lobChIP_FOXA1_robot
chr1	87254180	87254181	MACS_peak_532	9.00	lobChIP_FOXA1_robot
chr1	87258898	87258899	MACS_peak_533	31.00	lobChIP_FOXA1_robot
chr1	87289629	87289630	MACS_peak_534	49.00	lobChIP_FOXA1_robot
chr1	87326334	87326335	MACS_peak_535	4.00	lobChIP_FOXA1_robot
chr1	87434028	87434029	MACS_peak_536	5.00	lobChIP_FOXA1_robot
chr1	87459033	87459034	MACS_peak_537	16.00	lobChIP_FOXA1_robot
chr1	87461326	87461327	MACS_peak_538	6.00	lobChIP_FOXA1_robot
chr1	87491100	87491101	MACS_peak_539	8.00	lobChIP_FOXA1_robot
chr1	88252785	88252786	MACS_peak_540	10.00	lobChIP_FOXA1_robot
chr1	88261863	88261864	MACS_peak_541	10.00	lobChIP_FOXA1_robot
chr1	88641666	88641667	MACS_peak_542	13.00	lobChIP_FOXA1_robot
chr1	89279287	89279288	MACS_peak_543	6.00	lobChIP_FOXA1_robot
chr1	89377517	89377518	MACS_peak_544	4.00	lobChIP_FOXA1_robot
chr1	89386763	89386764	MACS_peak_545	10.00	lobChIP_FOXA1_robot
chr1	89511251	89511252	MACS_peak_546	6.00	lobChIP_FOXA1_robot
chr1	89822814	89822815	MACS_peak_547	12.00	lobChIP_FOXA1_robot
chr1	90276518	90276519	MACS_peak_548	13.00	lobChIP_FOXA1_robot
chr1	90292280	90292281	MACS_peak_549	12.00	lobChIP_FOXA1_robot
chr1	90302688	90302689	MACS_peak_550	4.00	lobChIP_FOXA1_robot
chr1	90336861	90336862	MACS_peak_551	12.00	lobChIP_FOXA1_robot
chr1	90364452	90364453	MACS_peak_552	5.00	lobChIP_FOXA1_robot
chr1	90397820	90397821	MACS_peak_553	3.00	lobChIP_FOXA1_robot
chr1	90408666	90408667	MACS_peak_554	11.00	lobChIP_FOXA1_robot
chr1	90711769	90711770	MACS_peak_555	3.00	lobChIP_FOXA1_robot
chr1	91214503	91214504	MACS_peak_556	11.00	lobChIP_FOXA1_robot
chr1	91425328	91425329	MACS_peak_557	7.00	lobChIP_FOXA1_robot
chr1	91481923	91481924	MACS_peak_558	14.00	lobChIP_FOXA1_robot
chr1	91492013	91492014	MACS_peak_559	5.00	lobChIP_FOXA1_robot
chr1	91558278	91558279	MACS_peak_560	7.00	lobChIP_FOXA1_robot
chr1	91963662	91963663	MACS_peak_562	5.00	lobChIP_FOXA1_robot
chr1	92256141	92256142	MACS_peak_563	4.00	lobChIP_FOXA1_robot
chr1	92293276	92293277	MACS_peak_564	11.00	lobChIP_FOXA1_robot
chr1	92297265	92297266	MACS_peak_565	10.00	lobChIP_FOXA1_robot
chr1	92311103	92311104	MACS_peak_566	5.00	lobChIP_FOXA1_robot
chr1	92504468	92504469	MACS_peak_567	5.00	lobChIP_FOXA1_robot
chr1	92554571	92554572	MACS_peak_568	5.00	lobChIP_FOXA1_robot
chr1	92907702	92907703	MACS_peak_569	14.00	lobChIP_FOXA1_robot
chr1	92971027	92971028	MACS_peak_570	4.00	lobChIP_FOXA1_robot
chr1	93289498	93289499	MACS_peak_571	5.00	lobChIP_FOXA1_robot
chr1	93352540	93352541	MACS_peak_572	18.00	lobChIP_FOXA1_robot
chr1	93411459	93411460	MACS_peak_573	7.00	lobChIP_FOXA1_robot
chr1	93416832	93416833	MACS_peak_574	8.00	lobChIP_FOXA1_robot
chr1	93508025	93508026	MACS_peak_575	4.00	lobChIP_FOXA1_robot
chr1	93580013	93580014	MACS_peak_576	5.00	lobChIP_FOXA1_robot
chr1	93595285	93595286	MACS_peak_577	12.00	lobChIP_FOXA1_robot
chr1	93666504	93666505	MACS_peak_578	9.00	lobChIP_FOXA1_robot
chr1	93694410	93694411	MACS_peak_579	10.00	lobChIP_FOXA1_robot
chr1	93741238	93741239	MACS_peak_580	4.00	lobChIP_FOXA1_robot
chr1	94051003	94051004	MACS_peak_581	14.00	lobChIP_FOXA1_robot
chr1	94083913	94083914	MACS_peak_582	6.00	lobChIP_FOXA1_robot
chr1	94118024	94118025	MACS_peak_583	3.00	lobChIP_FOXA1_robot
chr1	94169383	94169384	MACS_peak_584	5.00	lobChIP_FOXA1_robot
chr1	94224749	94224750	MACS_peak_585	5.00	lobChIP_FOXA1_robot
chr1	94267053	94267054	MACS_peak_586	3.00	lobChIP_FOXA1_robot
chr1	94445663	94445664	MACS_peak_587	4.00	lobChIP_FOXA1_robot
chr1	94534679	94534680	MACS_peak_588	5.00	lobChIP_FOXA1_robot
chr1	94544891	94544892	MACS_peak_589	5.00	lobChIP_FOXA1_robot
chr1	94582072	94582073	MACS_peak_590	6.00	lobChIP_FOXA1_robot
chr1	94591655	94591656	MACS_peak_591	7.00	lobChIP_FOXA1_robot
chr1	94700356	94700357	MACS_peak_592	8.00	lobChIP_FOXA1_robot
chr1	94774956	94774957	MACS_peak_593	4.00	lobChIP_FOXA1_robot
chr1	94776250	94776251	MACS_peak_594	5.00	lobChIP_FOXA1_robot
chr1	94781938	94781939	MACS_peak_595	3.00	lobChIP_FOXA1_robot
chr1	94820906	94820907	MACS_peak_596	4.00	lobChIP_FOXA1_robot
chr1	94821490	94821491	MACS_peak_597	5.00	lobChIP_FOXA1_robot
chr1	94849770	94849771	MACS_peak_598	9.00	lobChIP_FOXA1_robot
chr1	94891505	94891506	MACS_peak_599	27.00	lobChIP_FOXA1_robot
chr1	95028834	95028835	MACS_peak_600	3.00	lobChIP_FOXA1_robot
chr1	95163584	95163585	MACS_peak_601	12.00	lobChIP_FOXA1_robot
chr1	95164718	95164719	MACS_peak_602	11.00	lobChIP_FOXA1_robot
chr1	95202296	95202297	MACS_peak_603	8.00	lobChIP_FOXA1_robot
chr1	95258642	95258643	MACS_peak_604	4.00	lobChIP_FOXA1_robot
chr1	95263774	95263775	MACS_peak_605	6.00	lobChIP_FOXA1_robot
chr1	95273822	95273823	MACS_peak_606	6.00	lobChIP_FOXA1_robot
chr1	95427378	95427379	MACS_peak_607	5.00	lobChIP_FOXA1_robot
chr1	95428257	95428258	MACS_peak_608	3.00	lobChIP_FOXA1_robot
chr1	95479762	95479763	MACS_peak_609	27.00	lobChIP_FOXA1_robot
chr1	95552791	95552792	MACS_peak_610	5.00	lobChIP_FOXA1_robot
chr1	95573117	95573118	MACS_peak_611	4.00	lobChIP_FOXA1_robot
chr1	95588010	95588011	MACS_peak_612	16.00	lobChIP_FOXA1_robot
chr1	95707439	95707440	MACS_peak_613	5.00	lobChIP_FOXA1_robot
chr1	95752203	95752204	MACS_peak_614	4.00	lobChIP_FOXA1_robot
chr1	95836368	95836369	MACS_peak_615	6.00	lobChIP_FOXA1_robot
chr1	97097708	97097709	MACS_peak_616	22.00	lobChIP_FOXA1_robot
chr1	97737912	97737913	MACS_peak_617	5.00	lobChIP_FOXA1_robot
chr1	98552465	98552466	MACS_peak_618	4.00	lobChIP_FOXA1_robot
chr1	99953498	99953499	MACS_peak_619	4.00	lobChIP_FOXA1_robot
chr1	100020491	100020492	MACS_peak_620	3.00	lobChIP_FOXA1_robot
chr1	100024986	100024987	MACS_peak_621	5.00	lobChIP_FOXA1_robot
chr1	100031444	100031445	MACS_peak_622	4.00	lobChIP_FOXA1_robot
chr1	100032335	100032336	MACS_peak_623	17.00	lobChIP_FOXA1_robot
chr1	100054941	100054942	MACS_peak_624	14.00	lobChIP_FOXA1_robot
chr1	100065332	100065333	MACS_peak_625	6.00	lobChIP_FOXA1_robot
chr1	100101012	100101013	MACS_peak_626	7.00	lobChIP_FOXA1_robot
chr1	100204680	100204681	MACS_peak_627	5.00	lobChIP_FOXA1_robot
chr1	100220478	100220479	MACS_peak_628	11.00	lobChIP_FOXA1_robot
chr1	100224824	100224825	MACS_peak_629	3.00	lobChIP_FOXA1_robot
chr1	100225483	100225484	MACS_peak_630	17.00	lobChIP_FOXA1_robot
chr1	100227653	100227654	MACS_peak_631	6.00	lobChIP_FOXA1_robot
chr1	100878675	100878676	MACS_peak_632	6.00	lobChIP_FOXA1_robot
chr1	100915568	100915569	MACS_peak_633	4.00	lobChIP_FOXA1_robot
chr1	100946133	100946134	MACS_peak_634	5.00	lobChIP_FOXA1_robot
chr1	100951526	100951527	MACS_peak_635	22.00	lobChIP_FOXA1_robot
chr1	100960219	100960220	MACS_peak_636	3.00	lobChIP_FOXA1_robot
chr1	100981538	100981539	MACS_peak_637	7.00	lobChIP_FOXA1_robot
chr1	101050783	101050784	MACS_peak_638	6.00	lobChIP_FOXA1_robot
chr1	101197061	101197062	MACS_peak_639	9.00	lobChIP_FOXA1_robot
chr1	101469812	101469813	MACS_peak_640	22.00	lobChIP_FOXA1_robot
chr1	101602367	101602368	MACS_peak_641	6.00	lobChIP_FOXA1_robot
chr1	101603184	101603185	MACS_peak_642	4.00	lobChIP_FOXA1_robot
chr1	101754301	101754302	MACS_peak_643	7.00	lobChIP_FOXA1_robot
chr1	102018669	102018670	MACS_peak_644	4.00	lobChIP_FOXA1_robot
chr1	105413030	105413031	MACS_peak_645	6.00	lobChIP_FOXA1_robot
chr1	106175731	106175732	MACS_peak_646	5.00	lobChIP_FOXA1_robot
chr1	108069438	108069439	MACS_peak_647	25.00	lobChIP_FOXA1_robot
chr1	108140210	108140211	MACS_peak_648	8.00	lobChIP_FOXA1_robot
chr1	108223954	108223955	MACS_peak_649	31.00	lobChIP_FOXA1_robot
chr1	108225963	108225964	MACS_peak_650	7.00	lobChIP_FOXA1_robot
chr1	108231064	108231065	MACS_peak_651	5.00	lobChIP_FOXA1_robot
chr1	109127093	109127094	MACS_peak_652	4.00	lobChIP_FOXA1_robot
chr1	109265226	109265227	MACS_peak_653	5.00	lobChIP_FOXA1_robot
chr1	109507272	109507273	MACS_peak_654	6.00	lobChIP_FOXA1_robot
chr1	109633306	109633307	MACS_peak_655	6.00	lobChIP_FOXA1_robot
chr1	109659161	109659162	MACS_peak_656	8.00	lobChIP_FOXA1_robot
chr1	109724140	109724141	MACS_peak_657	20.00	lobChIP_FOXA1_robot
chr1	109817551	109817552	MACS_peak_658	8.00	lobChIP_FOXA1_robot
chr1	109842075	109842076	MACS_peak_659	24.00	lobChIP_FOXA1_robot
chr1	109935834	109935835	MACS_peak_660	13.00	lobChIP_FOXA1_robot
chr1	109983976	109983977	MACS_peak_661	12.00	lobChIP_FOXA1_robot
chr1	110425452	110425453	MACS_peak_662	4.00	lobChIP_FOXA1_robot
chr1	110491077	110491078	MACS_peak_663	8.00	lobChIP_FOXA1_robot
chr1	110503288	110503289	MACS_peak_664	32.00	lobChIP_FOXA1_robot
chr1	110577069	110577070	MACS_peak_665	4.00	lobChIP_FOXA1_robot
chr1	110863966	110863967	MACS_peak_666	6.00	lobChIP_FOXA1_robot
chr1	111323867	111323868	MACS_peak_667	6.00	lobChIP_FOXA1_robot
chr1	111588020	111588021	MACS_peak_668	6.00	lobChIP_FOXA1_robot
chr1	111652994	111652995	MACS_peak_669	9.00	lobChIP_FOXA1_robot
chr1	111771215	111771216	MACS_peak_670	5.00	lobChIP_FOXA1_robot
chr1	111960552	111960553	MACS_peak_671	14.00	lobChIP_FOXA1_robot
chr1	111989756	111989757	MACS_peak_672	7.00	lobChIP_FOXA1_robot
chr1	112078133	112078134	MACS_peak_673	3.00	lobChIP_FOXA1_robot
chr1	112094570	112094571	MACS_peak_674	5.00	lobChIP_FOXA1_robot
chr1	112102607	112102608	MACS_peak_675	5.00	lobChIP_FOXA1_robot
chr1	112182712	112182713	MACS_peak_676	6.00	lobChIP_FOXA1_robot
chr1	112519006	112519007	MACS_peak_677	3.00	lobChIP_FOXA1_robot
chr1	112519681	112519682	MACS_peak_678	15.00	lobChIP_FOXA1_robot
chr1	112531179	112531180	MACS_peak_679	4.00	lobChIP_FOXA1_robot
chr1	112909564	112909565	MACS_peak_680	3.00	lobChIP_FOXA1_robot
chr1	112937322	112937323	MACS_peak_681	4.00	lobChIP_FOXA1_robot
chr1	112939903	112939904	MACS_peak_682	4.00	lobChIP_FOXA1_robot
chr1	113003398	113003399	MACS_peak_683	15.00	lobChIP_FOXA1_robot
chr1	113178078	113178079	MACS_peak_684	4.00	lobChIP_FOXA1_robot
chr1	113547179	113547180	MACS_peak_685	6.00	lobChIP_FOXA1_robot
chr1	113689417	113689418	MACS_peak_686	4.00	lobChIP_FOXA1_robot
chr1	113760523	113760524	MACS_peak_687	5.00	lobChIP_FOXA1_robot
chr1	113863113	113863114	MACS_peak_688	3.00	lobChIP_FOXA1_robot
chr1	113931436	113931437	MACS_peak_689	14.00	lobChIP_FOXA1_robot
chr1	113937187	113937188	MACS_peak_690	6.00	lobChIP_FOXA1_robot
chr1	113999766	113999767	MACS_peak_691	7.00	lobChIP_FOXA1_robot
chr1	114050888	114050889	MACS_peak_692	7.00	lobChIP_FOXA1_robot
chr1	114060150	114060151	MACS_peak_693	11.00	lobChIP_FOXA1_robot
chr1	114217452	114217453	MACS_peak_694	6.00	lobChIP_FOXA1_robot
chr1	114347523	114347524	MACS_peak_695	6.00	lobChIP_FOXA1_robot
chr1	114471011	114471012	MACS_peak_696	4.00	lobChIP_FOXA1_robot
chr1	114471672	114471673	MACS_peak_697	6.00	lobChIP_FOXA1_robot
chr1	114996302	114996303	MACS_peak_698	4.00	lobChIP_FOXA1_robot
chr1	115010224	115010225	MACS_peak_699	34.00	lobChIP_FOXA1_robot
chr1	115124254	115124255	MACS_peak_700	4.00	lobChIP_FOXA1_robot
chr1	115211650	115211651	MACS_peak_701	4.00	lobChIP_FOXA1_robot
chr1	115300862	115300863	MACS_peak_702	4.00	lobChIP_FOXA1_robot
chr1	115579971	115579972	MACS_peak_703	7.00	lobChIP_FOXA1_robot
chr1	116109499	116109500	MACS_peak_704	5.00	lobChIP_FOXA1_robot
chr1	116254085	116254086	MACS_peak_705	7.00	lobChIP_FOXA1_robot
chr1	116665832	116665833	MACS_peak_706	7.00	lobChIP_FOXA1_robot
chr1	116695777	116695778	MACS_peak_707	3.00	lobChIP_FOXA1_robot
chr1	116835803	116835804	MACS_peak_708	10.00	lobChIP_FOXA1_robot
chr1	116855096	116855097	MACS_peak_709	3.00	lobChIP_FOXA1_robot
chr1	116866099	116866100	MACS_peak_710	4.00	lobChIP_FOXA1_robot
chr1	116963217	116963218	MACS_peak_711	7.00	lobChIP_FOXA1_robot
chr1	117008764	117008765	MACS_peak_712	18.00	lobChIP_FOXA1_robot
chr1	117018043	117018044	MACS_peak_713	5.00	lobChIP_FOXA1_robot
chr1	117032249	117032250	MACS_peak_714	11.00	lobChIP_FOXA1_robot
chr1	117396430	117396431	MACS_peak_715	5.00	lobChIP_FOXA1_robot
chr1	118297269	118297270	MACS_peak_716	5.00	lobChIP_FOXA1_robot
chr1	118364574	118364575	MACS_peak_717	8.00	lobChIP_FOXA1_robot
chr1	119503436	119503437	MACS_peak_718	8.00	lobChIP_FOXA1_robot
chr1	119506426	119506427	MACS_peak_719	7.00	lobChIP_FOXA1_robot
chr1	119879805	119879806	MACS_peak_720	5.00	lobChIP_FOXA1_robot
chr1	119884205	119884206	MACS_peak_721	29.00	lobChIP_FOXA1_robot
chr1	119904004	119904005	MACS_peak_722	4.00	lobChIP_FOXA1_robot
chr1	120309212	120309213	MACS_peak_723	19.00	lobChIP_FOXA1_robot
chr1	120359195	120359196	MACS_peak_724	4.00	lobChIP_FOXA1_robot
chr1	120575232	120575233	MACS_peak_725	6.00	lobChIP_FOXA1_robot
chr1	121264061	121264062	MACS_peak_726	12.00	lobChIP_FOXA1_robot
chr1	121280903	121280904	MACS_peak_727	5.00	lobChIP_FOXA1_robot
chr1	121328476	121328477	MACS_peak_728	20.00	lobChIP_FOXA1_robot
chr1	144009994	144009995	MACS_peak_730	6.00	lobChIP_FOXA1_robot
chr1	144532153	144532154	MACS_peak_731	4.00	lobChIP_FOXA1_robot
chr1	144534171	144534172	MACS_peak_732	4.00	lobChIP_FOXA1_robot
chr1	144924134	144924135	MACS_peak_733	3.00	lobChIP_FOXA1_robot
chr1	144937346	144937347	MACS_peak_734	12.00	lobChIP_FOXA1_robot
chr1	144999018	144999019	MACS_peak_735	4.00	lobChIP_FOXA1_robot
chr1	145246354	145246355	MACS_peak_736	10.00	lobChIP_FOXA1_robot
chr1	145384658	145384659	MACS_peak_737	7.00	lobChIP_FOXA1_robot
chr1	145391008	145391009	MACS_peak_738	6.00	lobChIP_FOXA1_robot
chr1	145391837	145391838	MACS_peak_739	4.00	lobChIP_FOXA1_robot
chr1	145422129	145422130	MACS_peak_740	7.00	lobChIP_FOXA1_robot
chr1	145542696	145542697	MACS_peak_741	10.00	lobChIP_FOXA1_robot
chr1	145723761	145723762	MACS_peak_742	9.00	lobChIP_FOXA1_robot
chr1	146640405	146640406	MACS_peak_743	5.00	lobChIP_FOXA1_robot
chr1	146640906	146640907	MACS_peak_744	5.00	lobChIP_FOXA1_robot
chr1	146687244	146687245	MACS_peak_745	15.00	lobChIP_FOXA1_robot
chr1	146709944	146709945	MACS_peak_746	15.00	lobChIP_FOXA1_robot
chr1	147084375	147084376	MACS_peak_747	9.00	lobChIP_FOXA1_robot
chr1	147097265	147097266	MACS_peak_748	17.00	lobChIP_FOXA1_robot
chr1	147148713	147148714	MACS_peak_749	3.00	lobChIP_FOXA1_robot
chr1	147189217	147189218	MACS_peak_750	5.00	lobChIP_FOXA1_robot
chr1	149223806	149223807	MACS_peak_751	4.00	lobChIP_FOXA1_robot
chr1	149980833	149980834	MACS_peak_752	20.00	lobChIP_FOXA1_robot
chr1	150229522	150229523	MACS_peak_753	4.00	lobChIP_FOXA1_robot
chr1	150359080	150359081	MACS_peak_754	5.00	lobChIP_FOXA1_robot
chr1	150378275	150378276	MACS_peak_755	5.00	lobChIP_FOXA1_robot
chr1	150383106	150383107	MACS_peak_756	5.00	lobChIP_FOXA1_robot
chr1	150446473	150446474	MACS_peak_757	8.00	lobChIP_FOXA1_robot
chr1	150540772	150540773	MACS_peak_758	11.00	lobChIP_FOXA1_robot
chr1	150548485	150548486	MACS_peak_759	9.00	lobChIP_FOXA1_robot
chr1	150584042	150584043	MACS_peak_760	6.00	lobChIP_FOXA1_robot
chr1	150847251	150847252	MACS_peak_761	4.00	lobChIP_FOXA1_robot
chr1	150944069	150944070	MACS_peak_762	11.00	lobChIP_FOXA1_robot
chr1	150985656	150985657	MACS_peak_763	6.00	lobChIP_FOXA1_robot
chr1	151044321	151044322	MACS_peak_764	5.00	lobChIP_FOXA1_robot
chr1	151254846	151254847	MACS_peak_765	6.00	lobChIP_FOXA1_robot
chr1	151345284	151345285	MACS_peak_766	5.00	lobChIP_FOXA1_robot
chr1	151486591	151486592	MACS_peak_767	4.00	lobChIP_FOXA1_robot
chr1	151521533	151521534	MACS_peak_768	5.00	lobChIP_FOXA1_robot
chr1	151569198	151569199	MACS_peak_769	6.00	lobChIP_FOXA1_robot
chr1	151948075	151948076	MACS_peak_770	8.00	lobChIP_FOXA1_robot
chr1	152714409	152714410	MACS_peak_771	3.00	lobChIP_FOXA1_robot
chr1	153603711	153603712	MACS_peak_772	5.00	lobChIP_FOXA1_robot
chr1	153715006	153715007	MACS_peak_773	7.00	lobChIP_FOXA1_robot
chr1	153741490	153741491	MACS_peak_774	3.00	lobChIP_FOXA1_robot
chr1	153926510	153926511	MACS_peak_775	7.00	lobChIP_FOXA1_robot
chr1	154151767	154151768	MACS_peak_776	5.00	lobChIP_FOXA1_robot
chr1	154210045	154210046	MACS_peak_777	6.00	lobChIP_FOXA1_robot
chr1	154324039	154324040	MACS_peak_778	8.00	lobChIP_FOXA1_robot
chr1	154381123	154381124	MACS_peak_779	8.00	lobChIP_FOXA1_robot
chr1	154390970	154390971	MACS_peak_780	15.00	lobChIP_FOXA1_robot
chr1	154392263	154392264	MACS_peak_781	10.00	lobChIP_FOXA1_robot
chr1	154442426	154442427	MACS_peak_782	6.00	lobChIP_FOXA1_robot
chr1	155099223	155099224	MACS_peak_783	5.00	lobChIP_FOXA1_robot
chr1	155099851	155099852	MACS_peak_784	21.00	lobChIP_FOXA1_robot
chr1	155428125	155428126	MACS_peak_785	8.00	lobChIP_FOXA1_robot
chr1	155641766	155641767	MACS_peak_786	4.00	lobChIP_FOXA1_robot
chr1	155668366	155668367	MACS_peak_787	4.00	lobChIP_FOXA1_robot
chr1	155914709	155914710	MACS_peak_788	22.00	lobChIP_FOXA1_robot
chr1	156056156	156056157	MACS_peak_789	20.00	lobChIP_FOXA1_robot
chr1	156154238	156154239	MACS_peak_790	5.00	lobChIP_FOXA1_robot
chr1	156161282	156161283	MACS_peak_791	3.00	lobChIP_FOXA1_robot
chr1	156195322	156195323	MACS_peak_792	7.00	lobChIP_FOXA1_robot
chr1	156341805	156341806	MACS_peak_793	5.00	lobChIP_FOXA1_robot
chr1	156441467	156441468	MACS_peak_794	10.00	lobChIP_FOXA1_robot
chr1	156765869	156765870	MACS_peak_795	5.00	lobChIP_FOXA1_robot
chr1	157206061	157206062	MACS_peak_796	4.00	lobChIP_FOXA1_robot
chr1	157210519	157210520	MACS_peak_797	15.00	lobChIP_FOXA1_robot
chr1	159787524	159787525	MACS_peak_798	4.00	lobChIP_FOXA1_robot
chr1	159940921	159940922	MACS_peak_799	8.00	lobChIP_FOXA1_robot
chr1	160023628	160023629	MACS_peak_800	5.00	lobChIP_FOXA1_robot
chr1	160094650	160094651	MACS_peak_801	4.00	lobChIP_FOXA1_robot
chr1	160118123	160118124	MACS_peak_802	5.00	lobChIP_FOXA1_robot
chr1	160204736	160204737	MACS_peak_803	4.00	lobChIP_FOXA1_robot
chr1	161188512	161188513	MACS_peak_804	8.00	lobChIP_FOXA1_robot
chr1	161193428	161193429	MACS_peak_805	6.00	lobChIP_FOXA1_robot
chr1	161194220	161194221	MACS_peak_806	8.00	lobChIP_FOXA1_robot
chr1	161225927	161225928	MACS_peak_807	6.00	lobChIP_FOXA1_robot
chr1	161368072	161368073	MACS_peak_808	15.00	lobChIP_FOXA1_robot
chr1	161672136	161672137	MACS_peak_809	5.00	lobChIP_FOXA1_robot
chr1	161736011	161736012	MACS_peak_810	17.00	lobChIP_FOXA1_robot
chr1	161883756	161883757	MACS_peak_811	8.00	lobChIP_FOXA1_robot
chr1	162057667	162057668	MACS_peak_812	3.00	lobChIP_FOXA1_robot
chr1	162062006	162062007	MACS_peak_813	12.00	lobChIP_FOXA1_robot
chr1	162122278	162122279	MACS_peak_814	5.00	lobChIP_FOXA1_robot
chr1	162183629	162183630	MACS_peak_815	6.00	lobChIP_FOXA1_robot
chr1	162205174	162205175	MACS_peak_816	4.00	lobChIP_FOXA1_robot
chr1	162217338	162217339	MACS_peak_817	6.00	lobChIP_FOXA1_robot
chr1	162327638	162327639	MACS_peak_818	5.00	lobChIP_FOXA1_robot
chr1	162530483	162530484	MACS_peak_819	17.00	lobChIP_FOXA1_robot
chr1	162566618	162566619	MACS_peak_820	4.00	lobChIP_FOXA1_robot
chr1	162696936	162696937	MACS_peak_821	8.00	lobChIP_FOXA1_robot
chr1	162844011	162844012	MACS_peak_822	12.00	lobChIP_FOXA1_robot
chr1	165495006	165495007	MACS_peak_823	3.00	lobChIP_FOXA1_robot
chr1	165499080	165499081	MACS_peak_824	3.00	lobChIP_FOXA1_robot
chr1	165840852	165840853	MACS_peak_825	4.00	lobChIP_FOXA1_robot
chr1	165880196	165880197	MACS_peak_826	5.00	lobChIP_FOXA1_robot
chr1	167786644	167786645	MACS_peak_827	28.00	lobChIP_FOXA1_robot
chr1	168115975	168115976	MACS_peak_828	5.00	lobChIP_FOXA1_robot
chr1	168280020	168280021	MACS_peak_829	5.00	lobChIP_FOXA1_robot
chr1	168435253	168435254	MACS_peak_830	3.00	lobChIP_FOXA1_robot
chr1	168465039	168465040	MACS_peak_831	13.00	lobChIP_FOXA1_robot
chr1	168467001	168467002	MACS_peak_832	5.00	lobChIP_FOXA1_robot
chr1	168521097	168521098	MACS_peak_833	18.00	lobChIP_FOXA1_robot
chr1	168524905	168524906	MACS_peak_834	6.00	lobChIP_FOXA1_robot
chr1	168526780	168526781	MACS_peak_835	12.00	lobChIP_FOXA1_robot
chr1	168538860	168538861	MACS_peak_836	6.00	lobChIP_FOXA1_robot
chr1	168658600	168658601	MACS_peak_837	13.00	lobChIP_FOXA1_robot
chr1	168662621	168662622	MACS_peak_838	4.00	lobChIP_FOXA1_robot
chr1	168770795	168770796	MACS_peak_839	15.00	lobChIP_FOXA1_robot
chr1	168831062	168831063	MACS_peak_840	9.00	lobChIP_FOXA1_robot
chr1	168889861	168889862	MACS_peak_841	4.00	lobChIP_FOXA1_robot
chr1	169079307	169079308	MACS_peak_842	12.00	lobChIP_FOXA1_robot
chr1	169085608	169085609	MACS_peak_843	7.00	lobChIP_FOXA1_robot
chr1	169220273	169220274	MACS_peak_844	14.00	lobChIP_FOXA1_robot
chr1	169454428	169454429	MACS_peak_845	5.00	lobChIP_FOXA1_robot
chr1	169476871	169476872	MACS_peak_846	6.00	lobChIP_FOXA1_robot
chr1	169535202	169535203	MACS_peak_847	3.00	lobChIP_FOXA1_robot
chr1	169539538	169539539	MACS_peak_848	21.00	lobChIP_FOXA1_robot
chr1	169541617	169541618	MACS_peak_849	7.00	lobChIP_FOXA1_robot
chr1	169557771	169557772	MACS_peak_850	8.00	lobChIP_FOXA1_robot
chr1	169714584	169714585	MACS_peak_851	9.00	lobChIP_FOXA1_robot
chr1	171557824	171557825	MACS_peak_852	3.00	lobChIP_FOXA1_robot
chr1	172238381	172238382	MACS_peak_853	8.00	lobChIP_FOXA1_robot
chr1	172329856	172329857	MACS_peak_854	13.00	lobChIP_FOXA1_robot
chr1	172368750	172368751	MACS_peak_855	6.00	lobChIP_FOXA1_robot
chr1	172418417	172418418	MACS_peak_856	6.00	lobChIP_FOXA1_robot
chr1	172419135	172419136	MACS_peak_857	5.00	lobChIP_FOXA1_robot
chr1	172420428	172420429	MACS_peak_858	10.00	lobChIP_FOXA1_robot
chr1	172439509	172439510	MACS_peak_859	15.00	lobChIP_FOXA1_robot
chr1	172526618	172526619	MACS_peak_860	7.00	lobChIP_FOXA1_robot
chr1	172593747	172593748	MACS_peak_861	5.00	lobChIP_FOXA1_robot
chr1	172612236	172612237	MACS_peak_862	5.00	lobChIP_FOXA1_robot
chr1	172938873	172938874	MACS_peak_863	3.00	lobChIP_FOXA1_robot
chr1	172945242	172945243	MACS_peak_864	3.00	lobChIP_FOXA1_robot
chr1	172945869	172945870	MACS_peak_865	5.00	lobChIP_FOXA1_robot
chr1	173097177	173097178	MACS_peak_866	13.00	lobChIP_FOXA1_robot
chr1	173182325	173182326	MACS_peak_867	8.00	lobChIP_FOXA1_robot
chr1	173443447	173443448	MACS_peak_868	4.00	lobChIP_FOXA1_robot
chr1	173444360	173444361	MACS_peak_869	4.00	lobChIP_FOXA1_robot
chr1	174236574	174236575	MACS_peak_870	29.00	lobChIP_FOXA1_robot
chr1	174635541	174635542	MACS_peak_871	14.00	lobChIP_FOXA1_robot
chr1	174781972	174781973	MACS_peak_872	28.00	lobChIP_FOXA1_robot
chr1	174912193	174912194	MACS_peak_873	4.00	lobChIP_FOXA1_robot
chr1	174923172	174923173	MACS_peak_874	3.00	lobChIP_FOXA1_robot
chr1	174996084	174996085	MACS_peak_875	5.00	lobChIP_FOXA1_robot
chr1	175009674	175009675	MACS_peak_876	4.00	lobChIP_FOXA1_robot
chr1	176042824	176042825	MACS_peak_877	5.00	lobChIP_FOXA1_robot
chr1	176119734	176119735	MACS_peak_878	10.00	lobChIP_FOXA1_robot
chr1	177541063	177541064	MACS_peak_879	14.00	lobChIP_FOXA1_robot
chr1	177841812	177841813	MACS_peak_880	16.00	lobChIP_FOXA1_robot
chr1	178019361	178019362	MACS_peak_881	5.00	lobChIP_FOXA1_robot
chr1	178022312	178022313	MACS_peak_882	10.00	lobChIP_FOXA1_robot
chr1	178208065	178208066	MACS_peak_883	7.00	lobChIP_FOXA1_robot
chr1	178328865	178328866	MACS_peak_884	3.00	lobChIP_FOXA1_robot
chr1	178396368	178396369	MACS_peak_885	4.00	lobChIP_FOXA1_robot
chr1	178590205	178590206	MACS_peak_886	5.00	lobChIP_FOXA1_robot
chr1	178651797	178651798	MACS_peak_887	5.00	lobChIP_FOXA1_robot
chr1	178722219	178722220	MACS_peak_888	4.00	lobChIP_FOXA1_robot
chr1	178816972	178816973	MACS_peak_889	5.00	lobChIP_FOXA1_robot
chr1	178840474	178840475	MACS_peak_890	3.00	lobChIP_FOXA1_robot
chr1	178840932	178840933	MACS_peak_891	5.00	lobChIP_FOXA1_robot
chr1	178886237	178886238	MACS_peak_892	10.00	lobChIP_FOXA1_robot
chr1	178888252	178888253	MACS_peak_893	3.00	lobChIP_FOXA1_robot
chr1	178888947	178888948	MACS_peak_894	24.00	lobChIP_FOXA1_robot
chr1	179015350	179015351	MACS_peak_895	7.00	lobChIP_FOXA1_robot
chr1	179074137	179074138	MACS_peak_896	5.00	lobChIP_FOXA1_robot
chr1	179121207	179121208	MACS_peak_897	8.00	lobChIP_FOXA1_robot
chr1	179312107	179312108	MACS_peak_898	8.00	lobChIP_FOXA1_robot
chr1	179353356	179353357	MACS_peak_899	6.00	lobChIP_FOXA1_robot
chr1	179842720	179842721	MACS_peak_900	5.00	lobChIP_FOXA1_robot
chr1	180126919	180126920	MACS_peak_901	13.00	lobChIP_FOXA1_robot
chr1	180280051	180280052	MACS_peak_902	3.00	lobChIP_FOXA1_robot
chr1	180555885	180555886	MACS_peak_903	3.00	lobChIP_FOXA1_robot
chr1	180784546	180784547	MACS_peak_904	14.00	lobChIP_FOXA1_robot
chr1	180796555	180796556	MACS_peak_905	11.00	lobChIP_FOXA1_robot
chr1	181012212	181012213	MACS_peak_906	10.00	lobChIP_FOXA1_robot
chr1	181062225	181062226	MACS_peak_907	9.00	lobChIP_FOXA1_robot
chr1	181081808	181081809	MACS_peak_908	7.00	lobChIP_FOXA1_robot
chr1	181587873	181587874	MACS_peak_909	4.00	lobChIP_FOXA1_robot
chr1	182041230	182041231	MACS_peak_910	5.00	lobChIP_FOXA1_robot
chr1	182090129	182090130	MACS_peak_911	11.00	lobChIP_FOXA1_robot
chr1	182150308	182150309	MACS_peak_912	4.00	lobChIP_FOXA1_robot
chr1	182155612	182155613	MACS_peak_913	5.00	lobChIP_FOXA1_robot
chr1	182249009	182249010	MACS_peak_914	3.00	lobChIP_FOXA1_robot
chr1	182258104	182258105	MACS_peak_915	5.00	lobChIP_FOXA1_robot
chr1	182261126	182261127	MACS_peak_916	39.00	lobChIP_FOXA1_robot
chr1	182271020	182271021	MACS_peak_917	8.00	lobChIP_FOXA1_robot
chr1	182278882	182278883	MACS_peak_918	3.00	lobChIP_FOXA1_robot
chr1	182354329	182354330	MACS_peak_919	5.00	lobChIP_FOXA1_robot
chr1	182366101	182366102	MACS_peak_920	6.00	lobChIP_FOXA1_robot
chr1	182427143	182427144	MACS_peak_921	5.00	lobChIP_FOXA1_robot
chr1	182999387	182999388	MACS_peak_922	6.00	lobChIP_FOXA1_robot
chr1	183039357	183039358	MACS_peak_923	4.00	lobChIP_FOXA1_robot
chr1	183058309	183058310	MACS_peak_924	11.00	lobChIP_FOXA1_robot
chr1	183067529	183067530	MACS_peak_925	5.00	lobChIP_FOXA1_robot
chr1	183098712	183098713	MACS_peak_926	5.00	lobChIP_FOXA1_robot
chr1	183108893	183108894	MACS_peak_927	14.00	lobChIP_FOXA1_robot
chr1	183601527	183601528	MACS_peak_928	3.00	lobChIP_FOXA1_robot
chr1	183609550	183609551	MACS_peak_929	19.00	lobChIP_FOXA1_robot
chr1	183645961	183645962	MACS_peak_930	5.00	lobChIP_FOXA1_robot
chr1	183668895	183668896	MACS_peak_931	33.00	lobChIP_FOXA1_robot
chr1	183749489	183749490	MACS_peak_932	9.00	lobChIP_FOXA1_robot
chr1	183811704	183811705	MACS_peak_933	9.00	lobChIP_FOXA1_robot
chr1	183962482	183962483	MACS_peak_934	7.00	lobChIP_FOXA1_robot
chr1	184006997	184006998	MACS_peak_935	4.00	lobChIP_FOXA1_robot
chr1	184728437	184728438	MACS_peak_936	5.00	lobChIP_FOXA1_robot
chr1	184874925	184874926	MACS_peak_937	8.00	lobChIP_FOXA1_robot
chr1	184944480	184944481	MACS_peak_938	4.00	lobChIP_FOXA1_robot
chr1	185284811	185284812	MACS_peak_939	5.00	lobChIP_FOXA1_robot
chr1	185296408	185296409	MACS_peak_940	4.00	lobChIP_FOXA1_robot
chr1	185319371	185319372	MACS_peak_941	7.00	lobChIP_FOXA1_robot
chr1	185555109	185555110	MACS_peak_942	13.00	lobChIP_FOXA1_robot
chr1	185615586	185615587	MACS_peak_943	6.00	lobChIP_FOXA1_robot
chr1	185643533	185643534	MACS_peak_944	17.00	lobChIP_FOXA1_robot
chr1	187460766	187460767	MACS_peak_945	6.00	lobChIP_FOXA1_robot
chr1	187955882	187955883	MACS_peak_946	4.00	lobChIP_FOXA1_robot
chr1	192500445	192500446	MACS_peak_947	5.00	lobChIP_FOXA1_robot
chr1	192670364	192670365	MACS_peak_948	5.00	lobChIP_FOXA1_robot
chr1	192671505	192671506	MACS_peak_949	14.00	lobChIP_FOXA1_robot
chr1	192977057	192977058	MACS_peak_950	37.00	lobChIP_FOXA1_robot
chr1	192981510	192981511	MACS_peak_951	4.00	lobChIP_FOXA1_robot
chr1	193005670	193005671	MACS_peak_952	8.00	lobChIP_FOXA1_robot
chr1	193081381	193081382	MACS_peak_953	6.00	lobChIP_FOXA1_robot
chr1	193125883	193125884	MACS_peak_954	4.00	lobChIP_FOXA1_robot
chr1	193324383	193324384	MACS_peak_955	3.00	lobChIP_FOXA1_robot
chr1	195801625	195801626	MACS_peak_956	4.00	lobChIP_FOXA1_robot
chr1	195977165	195977166	MACS_peak_957	4.00	lobChIP_FOXA1_robot
chr1	196047623	196047624	MACS_peak_958	3.00	lobChIP_FOXA1_robot
chr1	196370217	196370218	MACS_peak_959	5.00	lobChIP_FOXA1_robot
chr1	196407769	196407770	MACS_peak_960	10.00	lobChIP_FOXA1_robot
chr1	196993729	196993730	MACS_peak_961	5.00	lobChIP_FOXA1_robot
chr1	197139762	197139763	MACS_peak_962	4.00	lobChIP_FOXA1_robot
chr1	197143582	197143583	MACS_peak_963	5.00	lobChIP_FOXA1_robot
chr1	197590045	197590046	MACS_peak_964	6.00	lobChIP_FOXA1_robot
chr1	197807244	197807245	MACS_peak_965	6.00	lobChIP_FOXA1_robot
chr1	197962867	197962868	MACS_peak_966	4.00	lobChIP_FOXA1_robot
chr1	197964310	197964311	MACS_peak_967	4.00	lobChIP_FOXA1_robot
chr1	198055369	198055370	MACS_peak_968	4.00	lobChIP_FOXA1_robot
chr1	198461883	198461884	MACS_peak_969	6.00	lobChIP_FOXA1_robot
chr1	198503880	198503881	MACS_peak_970	6.00	lobChIP_FOXA1_robot
chr1	200229922	200229923	MACS_peak_971	3.00	lobChIP_FOXA1_robot
chr1	200244738	200244739	MACS_peak_972	8.00	lobChIP_FOXA1_robot
chr1	200265538	200265539	MACS_peak_973	6.00	lobChIP_FOXA1_robot
chr1	200283773	200283774	MACS_peak_974	4.00	lobChIP_FOXA1_robot
chr1	200305732	200305733	MACS_peak_975	5.00	lobChIP_FOXA1_robot
chr1	200307570	200307571	MACS_peak_976	5.00	lobChIP_FOXA1_robot
chr1	200341331	200341332	MACS_peak_977	64.00	lobChIP_FOXA1_robot
chr1	200343106	200343107	MACS_peak_978	10.00	lobChIP_FOXA1_robot
chr1	200380231	200380232	MACS_peak_979	6.00	lobChIP_FOXA1_robot
chr1	200385405	200385406	MACS_peak_980	5.00	lobChIP_FOXA1_robot
chr1	200712014	200712015	MACS_peak_981	7.00	lobChIP_FOXA1_robot
chr1	200786342	200786343	MACS_peak_982	4.00	lobChIP_FOXA1_robot
chr1	201096064	201096065	MACS_peak_983	5.00	lobChIP_FOXA1_robot
chr1	201139737	201139738	MACS_peak_984	6.00	lobChIP_FOXA1_robot
chr1	201811815	201811816	MACS_peak_985	4.00	lobChIP_FOXA1_robot
chr1	201975295	201975296	MACS_peak_986	7.00	lobChIP_FOXA1_robot
chr1	201987975	201987976	MACS_peak_987	8.00	lobChIP_FOXA1_robot
chr1	202016348	202016349	MACS_peak_988	6.00	lobChIP_FOXA1_robot
chr1	202027641	202027642	MACS_peak_989	4.00	lobChIP_FOXA1_robot
chr1	202045546	202045547	MACS_peak_990	5.00	lobChIP_FOXA1_robot
chr1	202054147	202054148	MACS_peak_991	4.00	lobChIP_FOXA1_robot
chr1	202055408	202055409	MACS_peak_992	4.00	lobChIP_FOXA1_robot
chr1	202083183	202083184	MACS_peak_993	4.00	lobChIP_FOXA1_robot
chr1	202506505	202506506	MACS_peak_994	9.00	lobChIP_FOXA1_robot
chr1	203158072	203158073	MACS_peak_995	5.00	lobChIP_FOXA1_robot
chr1	203180307	203180308	MACS_peak_996	12.00	lobChIP_FOXA1_robot
chr1	203298029	203298030	MACS_peak_997	4.00	lobChIP_FOXA1_robot
chr1	203831168	203831169	MACS_peak_998	5.00	lobChIP_FOXA1_robot
chr1	204047616	204047617	MACS_peak_999	4.00	lobChIP_FOXA1_robot
chr1	204071354	204071355	MACS_peak_1000	8.00	lobChIP_FOXA1_robot
chr1	204139203	204139204	MACS_peak_1001	4.00	lobChIP_FOXA1_robot
chr1	204140939	204140940	MACS_peak_1002	13.00	lobChIP_FOXA1_robot
chr1	204183526	204183527	MACS_peak_1003	4.00	lobChIP_FOXA1_robot
chr1	204223402	204223403	MACS_peak_1004	3.00	lobChIP_FOXA1_robot
chr1	204264964	204264965	MACS_peak_1005	3.00	lobChIP_FOXA1_robot
chr1	204333207	204333208	MACS_peak_1006	6.00	lobChIP_FOXA1_robot
chr1	204352481	204352482	MACS_peak_1007	3.00	lobChIP_FOXA1_robot
chr1	204367765	204367766	MACS_peak_1008	13.00	lobChIP_FOXA1_robot
chr1	204373895	204373896	MACS_peak_1009	3.00	lobChIP_FOXA1_robot
chr1	204478695	204478696	MACS_peak_1010	4.00	lobChIP_FOXA1_robot
chr1	204893607	204893608	MACS_peak_1011	4.00	lobChIP_FOXA1_robot
chr1	205287929	205287930	MACS_peak_1012	7.00	lobChIP_FOXA1_robot
chr1	205461533	205461534	MACS_peak_1013	8.00	lobChIP_FOXA1_robot
chr1	205604897	205604898	MACS_peak_1014	5.00	lobChIP_FOXA1_robot
chr1	205612335	205612336	MACS_peak_1015	5.00	lobChIP_FOXA1_robot
chr1	205645326	205645327	MACS_peak_1016	5.00	lobChIP_FOXA1_robot
chr1	205672353	205672354	MACS_peak_1017	14.00	lobChIP_FOXA1_robot
chr1	205678900	205678901	MACS_peak_1018	4.00	lobChIP_FOXA1_robot
chr1	205679771	205679772	MACS_peak_1019	5.00	lobChIP_FOXA1_robot
chr1	205695839	205695840	MACS_peak_1020	4.00	lobChIP_FOXA1_robot
chr1	205709126	205709127	MACS_peak_1021	6.00	lobChIP_FOXA1_robot
chr1	205788597	205788598	MACS_peak_1022	3.00	lobChIP_FOXA1_robot
chr1	205914372	205914373	MACS_peak_1023	4.00	lobChIP_FOXA1_robot
chr1	206321580	206321581	MACS_peak_1024	4.00	lobChIP_FOXA1_robot
chr1	206873176	206873177	MACS_peak_1025	5.00	lobChIP_FOXA1_robot
chr1	206883627	206883628	MACS_peak_1026	5.00	lobChIP_FOXA1_robot
chr1	207195044	207195045	MACS_peak_1027	5.00	lobChIP_FOXA1_robot
chr1	207199060	207199061	MACS_peak_1028	8.00	lobChIP_FOXA1_robot
chr1	207208166	207208167	MACS_peak_1029	3.00	lobChIP_FOXA1_robot
chr1	207262504	207262505	MACS_peak_1030	15.00	lobChIP_FOXA1_robot
chr1	207277505	207277506	MACS_peak_1031	11.00	lobChIP_FOXA1_robot
chr1	207414677	207414678	MACS_peak_1032	14.00	lobChIP_FOXA1_robot
chr1	207777323	207777324	MACS_peak_1033	8.00	lobChIP_FOXA1_robot
chr1	207999938	207999939	MACS_peak_1034	6.00	lobChIP_FOXA1_robot
chr1	208293510	208293511	MACS_peak_1035	5.00	lobChIP_FOXA1_robot
chr1	208376925	208376926	MACS_peak_1036	7.00	lobChIP_FOXA1_robot
chr1	208409699	208409700	MACS_peak_1037	5.00	lobChIP_FOXA1_robot
chr1	210545060	210545061	MACS_peak_1038	3.00	lobChIP_FOXA1_robot
chr1	210547415	210547416	MACS_peak_1039	16.00	lobChIP_FOXA1_robot
chr1	210558760	210558761	MACS_peak_1040	6.00	lobChIP_FOXA1_robot
chr1	211693084	211693085	MACS_peak_1041	7.00	lobChIP_FOXA1_robot
chr1	211694918	211694919	MACS_peak_1042	4.00	lobChIP_FOXA1_robot
chr1	211776838	211776839	MACS_peak_1043	10.00	lobChIP_FOXA1_robot
chr1	211778100	211778101	MACS_peak_1044	15.00	lobChIP_FOXA1_robot
chr1	211817305	211817306	MACS_peak_1045	5.00	lobChIP_FOXA1_robot
chr1	211840789	211840790	MACS_peak_1046	28.00	lobChIP_FOXA1_robot
chr1	212059638	212059639	MACS_peak_1047	4.00	lobChIP_FOXA1_robot
chr1	212068933	212068934	MACS_peak_1048	5.00	lobChIP_FOXA1_robot
chr1	212091361	212091362	MACS_peak_1049	7.00	lobChIP_FOXA1_robot
chr1	212402064	212402065	MACS_peak_1050	9.00	lobChIP_FOXA1_robot
chr1	212428510	212428511	MACS_peak_1051	6.00	lobChIP_FOXA1_robot
chr1	212431585	212431586	MACS_peak_1052	7.00	lobChIP_FOXA1_robot
chr1	212456446	212456447	MACS_peak_1053	3.00	lobChIP_FOXA1_robot
chr1	212464947	212464948	MACS_peak_1054	6.00	lobChIP_FOXA1_robot
chr1	212483673	212483674	MACS_peak_1055	3.00	lobChIP_FOXA1_robot
chr1	212672224	212672225	MACS_peak_1056	6.00	lobChIP_FOXA1_robot
chr1	212965447	212965448	MACS_peak_1057	3.00	lobChIP_FOXA1_robot
chr1	212968266	212968267	MACS_peak_1058	3.00	lobChIP_FOXA1_robot
chr1	213001239	213001240	MACS_peak_1059	8.00	lobChIP_FOXA1_robot
chr1	213311246	213311247	MACS_peak_1060	4.00	lobChIP_FOXA1_robot
chr1	213545733	213545734	MACS_peak_1061	21.00	lobChIP_FOXA1_robot
chr1	213742902	213742903	MACS_peak_1062	7.00	lobChIP_FOXA1_robot
chr1	213746506	213746507	MACS_peak_1063	8.00	lobChIP_FOXA1_robot
chr1	213824217	213824218	MACS_peak_1064	5.00	lobChIP_FOXA1_robot
chr1	213907302	213907303	MACS_peak_1065	7.00	lobChIP_FOXA1_robot
chr1	213933408	213933409	MACS_peak_1066	8.00	lobChIP_FOXA1_robot
chr1	213967293	213967294	MACS_peak_1067	9.00	lobChIP_FOXA1_robot
chr1	214129927	214129928	MACS_peak_1068	28.00	lobChIP_FOXA1_robot
chr1	214131965	214131966	MACS_peak_1069	4.00	lobChIP_FOXA1_robot
chr1	214201040	214201041	MACS_peak_1070	19.00	lobChIP_FOXA1_robot
chr1	214202457	214202458	MACS_peak_1071	18.00	lobChIP_FOXA1_robot
chr1	214212785	214212786	MACS_peak_1072	9.00	lobChIP_FOXA1_robot
chr1	214218028	214218029	MACS_peak_1073	9.00	lobChIP_FOXA1_robot
chr1	214224473	214224474	MACS_peak_1074	29.00	lobChIP_FOXA1_robot
chr1	214242116	214242117	MACS_peak_1075	7.00	lobChIP_FOXA1_robot
chr1	214458332	214458333	MACS_peak_1076	23.00	lobChIP_FOXA1_robot
chr1	214490004	214490005	MACS_peak_1077	4.00	lobChIP_FOXA1_robot
chr1	214588188	214588189	MACS_peak_1078	17.00	lobChIP_FOXA1_robot
chr1	214614335	214614336	MACS_peak_1079	6.00	lobChIP_FOXA1_robot
chr1	214633897	214633898	MACS_peak_1080	7.00	lobChIP_FOXA1_robot
chr1	214680406	214680407	MACS_peak_1081	11.00	lobChIP_FOXA1_robot
chr1	214689931	214689932	MACS_peak_1082	12.00	lobChIP_FOXA1_robot
chr1	214696940	214696941	MACS_peak_1083	3.00	lobChIP_FOXA1_robot
chr1	214851973	214851974	MACS_peak_1084	9.00	lobChIP_FOXA1_robot
chr1	215006863	215006864	MACS_peak_1085	20.00	lobChIP_FOXA1_robot
chr1	216395115	216395116	MACS_peak_1086	4.00	lobChIP_FOXA1_robot
chr1	216397778	216397779	MACS_peak_1087	6.00	lobChIP_FOXA1_robot
chr1	216611529	216611530	MACS_peak_1088	3.00	lobChIP_FOXA1_robot
chr1	217397865	217397866	MACS_peak_1089	5.00	lobChIP_FOXA1_robot
chr1	218337485	218337486	MACS_peak_1090	4.00	lobChIP_FOXA1_robot
chr1	218540156	218540157	MACS_peak_1091	6.00	lobChIP_FOXA1_robot
chr1	218545672	218545673	MACS_peak_1092	3.00	lobChIP_FOXA1_robot
chr1	218575707	218575708	MACS_peak_1093	7.00	lobChIP_FOXA1_robot
chr1	219344621	219344622	MACS_peak_1094	7.00	lobChIP_FOXA1_robot
chr1	219483853	219483854	MACS_peak_1095	14.00	lobChIP_FOXA1_robot
chr1	219504238	219504239	MACS_peak_1096	6.00	lobChIP_FOXA1_robot
chr1	219504851	219504852	MACS_peak_1097	4.00	lobChIP_FOXA1_robot
chr1	219587823	219587824	MACS_peak_1098	11.00	lobChIP_FOXA1_robot
chr1	220167016	220167017	MACS_peak_1099	4.00	lobChIP_FOXA1_robot
chr1	220908069	220908070	MACS_peak_1100	12.00	lobChIP_FOXA1_robot
chr1	220940978	220940979	MACS_peak_1101	4.00	lobChIP_FOXA1_robot
chr1	220941603	220941604	MACS_peak_1102	3.00	lobChIP_FOXA1_robot
chr1	221092137	221092138	MACS_peak_1103	9.00	lobChIP_FOXA1_robot
chr1	221508862	221508863	MACS_peak_1104	6.00	lobChIP_FOXA1_robot
chr1	221888969	221888970	MACS_peak_1105	11.00	lobChIP_FOXA1_robot
chr1	221890795	221890796	MACS_peak_1106	5.00	lobChIP_FOXA1_robot
chr1	221892004	221892005	MACS_peak_1107	13.00	lobChIP_FOXA1_robot
chr1	222235518	222235519	MACS_peak_1108	4.00	lobChIP_FOXA1_robot
chr1	222639597	222639598	MACS_peak_1109	4.00	lobChIP_FOXA1_robot
chr1	222719574	222719575	MACS_peak_1110	4.00	lobChIP_FOXA1_robot
chr1	223060151	223060152	MACS_peak_1111	6.00	lobChIP_FOXA1_robot
chr1	223219602	223219603	MACS_peak_1112	5.00	lobChIP_FOXA1_robot
chr1	223842009	223842010	MACS_peak_1113	5.00	lobChIP_FOXA1_robot
chr1	223843052	223843053	MACS_peak_1114	5.00	lobChIP_FOXA1_robot
chr1	223894375	223894376	MACS_peak_1115	7.00	lobChIP_FOXA1_robot
chr1	223898432	223898433	MACS_peak_1116	20.00	lobChIP_FOXA1_robot
chr1	223907341	223907342	MACS_peak_1117	5.00	lobChIP_FOXA1_robot
chr1	223908887	223908888	MACS_peak_1118	6.00	lobChIP_FOXA1_robot
chr1	223953103	223953104	MACS_peak_1119	4.00	lobChIP_FOXA1_robot
chr1	224002884	224002885	MACS_peak_1120	4.00	lobChIP_FOXA1_robot
chr1	224074449	224074450	MACS_peak_1121	9.00	lobChIP_FOXA1_robot
chr1	224294837	224294838	MACS_peak_1122	5.00	lobChIP_FOXA1_robot
chr1	224302540	224302541	MACS_peak_1123	3.00	lobChIP_FOXA1_robot
chr1	224399129	224399130	MACS_peak_1124	4.00	lobChIP_FOXA1_robot
chr1	224626863	224626864	MACS_peak_1125	6.00	lobChIP_FOXA1_robot
chr1	224970728	224970729	MACS_peak_1126	4.00	lobChIP_FOXA1_robot
chr1	225133871	225133872	MACS_peak_1127	4.00	lobChIP_FOXA1_robot
chr1	225169750	225169751	MACS_peak_1128	10.00	lobChIP_FOXA1_robot
chr1	225396921	225396922	MACS_peak_1129	4.00	lobChIP_FOXA1_robot
chr1	225416777	225416778	MACS_peak_1130	14.00	lobChIP_FOXA1_robot
chr1	225594534	225594535	MACS_peak_1131	3.00	lobChIP_FOXA1_robot
chr1	225600571	225600572	MACS_peak_1132	5.00	lobChIP_FOXA1_robot
chr1	225642137	225642138	MACS_peak_1133	5.00	lobChIP_FOXA1_robot
chr1	225791350	225791351	MACS_peak_1134	8.00	lobChIP_FOXA1_robot
chr1	225859312	225859313	MACS_peak_1135	5.00	lobChIP_FOXA1_robot
chr1	225882042	225882043	MACS_peak_1136	13.00	lobChIP_FOXA1_robot
chr1	225888127	225888128	MACS_peak_1137	21.00	lobChIP_FOXA1_robot
chr1	225955591	225955592	MACS_peak_1138	4.00	lobChIP_FOXA1_robot
chr1	225956553	225956554	MACS_peak_1139	4.00	lobChIP_FOXA1_robot
chr1	225960061	225960062	MACS_peak_1140	5.00	lobChIP_FOXA1_robot
chr1	226001332	226001333	MACS_peak_1141	10.00	lobChIP_FOXA1_robot
chr1	226008771	226008772	MACS_peak_1142	17.00	lobChIP_FOXA1_robot
chr1	226066195	226066196	MACS_peak_1143	4.00	lobChIP_FOXA1_robot
chr1	226411048	226411049	MACS_peak_1144	4.00	lobChIP_FOXA1_robot
chr1	226416353	226416354	MACS_peak_1145	3.00	lobChIP_FOXA1_robot
chr1	226667297	226667298	MACS_peak_1146	44.00	lobChIP_FOXA1_robot
chr1	227133134	227133135	MACS_peak_1147	5.00	lobChIP_FOXA1_robot
chr1	227431373	227431374	MACS_peak_1148	5.00	lobChIP_FOXA1_robot
chr1	227516102	227516103	MACS_peak_1149	3.00	lobChIP_FOXA1_robot
chr1	227614889	227614890	MACS_peak_1150	5.00	lobChIP_FOXA1_robot
chr1	228800278	228800279	MACS_peak_1151	6.00	lobChIP_FOXA1_robot
chr1	228872338	228872339	MACS_peak_1152	4.00	lobChIP_FOXA1_robot
chr1	228972434	228972435	MACS_peak_1153	5.00	lobChIP_FOXA1_robot
chr1	228974459	228974460	MACS_peak_1154	7.00	lobChIP_FOXA1_robot
chr1	228997051	228997052	MACS_peak_1155	5.00	lobChIP_FOXA1_robot
chr1	229021062	229021063	MACS_peak_1156	4.00	lobChIP_FOXA1_robot
chr1	229071048	229071049	MACS_peak_1157	5.00	lobChIP_FOXA1_robot
chr1	229139430	229139431	MACS_peak_1158	5.00	lobChIP_FOXA1_robot
chr1	229200181	229200182	MACS_peak_1159	12.00	lobChIP_FOXA1_robot
chr1	229235027	229235028	MACS_peak_1160	10.00	lobChIP_FOXA1_robot
chr1	229267463	229267464	MACS_peak_1161	5.00	lobChIP_FOXA1_robot
chr1	229284132	229284133	MACS_peak_1162	11.00	lobChIP_FOXA1_robot
chr1	229357372	229357373	MACS_peak_1163	15.00	lobChIP_FOXA1_robot
chr1	229378896	229378897	MACS_peak_1164	3.00	lobChIP_FOXA1_robot
chr1	229389026	229389027	MACS_peak_1165	5.00	lobChIP_FOXA1_robot
chr1	229409711	229409712	MACS_peak_1166	15.00	lobChIP_FOXA1_robot
chr1	229435127	229435128	MACS_peak_1167	11.00	lobChIP_FOXA1_robot
chr1	229546425	229546426	MACS_peak_1168	3.00	lobChIP_FOXA1_robot
chr1	229645523	229645524	MACS_peak_1169	12.00	lobChIP_FOXA1_robot
chr1	230289801	230289802	MACS_peak_1170	4.00	lobChIP_FOXA1_robot
chr1	230294741	230294742	MACS_peak_1171	15.00	lobChIP_FOXA1_robot
chr1	230410955	230410956	MACS_peak_1172	4.00	lobChIP_FOXA1_robot
chr1	230775685	230775686	MACS_peak_1173	5.00	lobChIP_FOXA1_robot
chr1	230816571	230816572	MACS_peak_1174	20.00	lobChIP_FOXA1_robot
chr1	230838965	230838966	MACS_peak_1175	5.00	lobChIP_FOXA1_robot
chr1	230844511	230844512	MACS_peak_1176	3.00	lobChIP_FOXA1_robot
chr1	230861240	230861241	MACS_peak_1177	14.00	lobChIP_FOXA1_robot
chr1	230864800	230864801	MACS_peak_1178	13.00	lobChIP_FOXA1_robot
chr1	230880359	230880360	MACS_peak_1179	7.00	lobChIP_FOXA1_robot
chr1	231069141	231069142	MACS_peak_1180	4.00	lobChIP_FOXA1_robot
chr1	231084818	231084819	MACS_peak_1181	3.00	lobChIP_FOXA1_robot
chr1	231100852	231100853	MACS_peak_1182	6.00	lobChIP_FOXA1_robot
chr1	231113618	231113619	MACS_peak_1183	3.00	lobChIP_FOXA1_robot
chr1	231128296	231128297	MACS_peak_1184	4.00	lobChIP_FOXA1_robot
chr1	231330919	231330920	MACS_peak_1185	5.00	lobChIP_FOXA1_robot
chr1	231355588	231355589	MACS_peak_1186	4.00	lobChIP_FOXA1_robot
chr1	231368992	231368993	MACS_peak_1187	4.00	lobChIP_FOXA1_robot
chr1	231369780	231369781	MACS_peak_1188	10.00	lobChIP_FOXA1_robot
chr1	231372028	231372029	MACS_peak_1189	5.00	lobChIP_FOXA1_robot
chr1	231374162	231374163	MACS_peak_1190	4.00	lobChIP_FOXA1_robot
chr1	231384212	231384213	MACS_peak_1191	3.00	lobChIP_FOXA1_robot
chr1	231558603	231558604	MACS_peak_1192	3.00	lobChIP_FOXA1_robot
chr1	231559642	231559643	MACS_peak_1193	31.00	lobChIP_FOXA1_robot
chr1	231623750	231623751	MACS_peak_1194	4.00	lobChIP_FOXA1_robot
chr1	231658013	231658014	MACS_peak_1195	4.00	lobChIP_FOXA1_robot
chr1	231713018	231713019	MACS_peak_1196	4.00	lobChIP_FOXA1_robot
chr1	231764037	231764038	MACS_peak_1197	4.00	lobChIP_FOXA1_robot
chr1	231794283	231794284	MACS_peak_1198	3.00	lobChIP_FOXA1_robot
chr1	231810591	231810592	MACS_peak_1199	3.00	lobChIP_FOXA1_robot
chr1	231814035	231814036	MACS_peak_1200	5.00	lobChIP_FOXA1_robot
chr1	231974302	231974303	MACS_peak_1201	8.00	lobChIP_FOXA1_robot
chr1	232070701	232070702	MACS_peak_1202	5.00	lobChIP_FOXA1_robot
chr1	232300264	232300265	MACS_peak_1203	19.00	lobChIP_FOXA1_robot
chr1	232422770	232422771	MACS_peak_1204	3.00	lobChIP_FOXA1_robot
chr1	233390216	233390217	MACS_peak_1205	4.00	lobChIP_FOXA1_robot
chr1	234463766	234463767	MACS_peak_1206	4.00	lobChIP_FOXA1_robot
chr1	234491760	234491761	MACS_peak_1207	5.00	lobChIP_FOXA1_robot
chr1	234507316	234507317	MACS_peak_1208	6.00	lobChIP_FOXA1_robot
chr1	234518105	234518106	MACS_peak_1209	4.00	lobChIP_FOXA1_robot
chr1	234594339	234594340	MACS_peak_1210	5.00	lobChIP_FOXA1_robot
chr1	234680552	234680553	MACS_peak_1211	4.00	lobChIP_FOXA1_robot
chr1	234698079	234698080	MACS_peak_1212	5.00	lobChIP_FOXA1_robot
chr1	234736452	234736453	MACS_peak_1213	7.00	lobChIP_FOXA1_robot
chr1	234738630	234738631	MACS_peak_1214	6.00	lobChIP_FOXA1_robot
chr1	234748416	234748417	MACS_peak_1215	23.00	lobChIP_FOXA1_robot
chr1	234749451	234749452	MACS_peak_1216	4.00	lobChIP_FOXA1_robot
chr1	234755679	234755680	MACS_peak_1217	9.00	lobChIP_FOXA1_robot
chr1	234756941	234756942	MACS_peak_1218	9.00	lobChIP_FOXA1_robot
chr1	234764236	234764237	MACS_peak_1219	5.00	lobChIP_FOXA1_robot
chr1	234767630	234767631	MACS_peak_1220	6.00	lobChIP_FOXA1_robot
chr1	234775322	234775323	MACS_peak_1221	4.00	lobChIP_FOXA1_robot
chr1	234779092	234779093	MACS_peak_1222	36.00	lobChIP_FOXA1_robot
chr1	234820133	234820134	MACS_peak_1223	4.00	lobChIP_FOXA1_robot
chr1	234852279	234852280	MACS_peak_1224	20.00	lobChIP_FOXA1_robot
chr1	234857351	234857352	MACS_peak_1225	10.00	lobChIP_FOXA1_robot
chr1	234860134	234860135	MACS_peak_1226	14.00	lobChIP_FOXA1_robot
chr1	234887136	234887137	MACS_peak_1227	5.00	lobChIP_FOXA1_robot
chr1	234904033	234904034	MACS_peak_1228	6.00	lobChIP_FOXA1_robot
chr1	234904941	234904942	MACS_peak_1229	5.00	lobChIP_FOXA1_robot
chr1	234908167	234908168	MACS_peak_1230	5.00	lobChIP_FOXA1_robot
chr1	235011678	235011679	MACS_peak_1231	3.00	lobChIP_FOXA1_robot
chr1	235012254	235012255	MACS_peak_1232	9.00	lobChIP_FOXA1_robot
chr1	235027400	235027401	MACS_peak_1233	5.00	lobChIP_FOXA1_robot
chr1	235093315	235093316	MACS_peak_1234	8.00	lobChIP_FOXA1_robot
chr1	235096617	235096618	MACS_peak_1235	23.00	lobChIP_FOXA1_robot
chr1	235099391	235099392	MACS_peak_1236	19.00	lobChIP_FOXA1_robot
chr1	235100203	235100204	MACS_peak_1237	5.00	lobChIP_FOXA1_robot
chr1	235111013	235111014	MACS_peak_1238	28.00	lobChIP_FOXA1_robot
chr1	235112162	235112163	MACS_peak_1239	6.00	lobChIP_FOXA1_robot
chr1	235114680	235114681	MACS_peak_1240	10.00	lobChIP_FOXA1_robot
chr1	235293229	235293230	MACS_peak_1241	8.00	lobChIP_FOXA1_robot
chr1	235324915	235324916	MACS_peak_1242	6.00	lobChIP_FOXA1_robot
chr1	235459468	235459469	MACS_peak_1243	10.00	lobChIP_FOXA1_robot
chr1	235572143	235572144	MACS_peak_1244	4.00	lobChIP_FOXA1_robot
chr1	235572900	235572901	MACS_peak_1245	13.00	lobChIP_FOXA1_robot
chr1	235575354	235575355	MACS_peak_1246	5.00	lobChIP_FOXA1_robot
chr1	235794138	235794139	MACS_peak_1247	14.00	lobChIP_FOXA1_robot
chr1	235989615	235989616	MACS_peak_1248	4.00	lobChIP_FOXA1_robot
chr1	236056546	236056547	MACS_peak_1249	8.00	lobChIP_FOXA1_robot
chr1	236203198	236203199	MACS_peak_1250	16.00	lobChIP_FOXA1_robot
chr1	236238021	236238022	MACS_peak_1251	4.00	lobChIP_FOXA1_robot
chr1	236423058	236423059	MACS_peak_1252	9.00	lobChIP_FOXA1_robot
chr1	236470978	236470979	MACS_peak_1253	5.00	lobChIP_FOXA1_robot
chr1	236563018	236563019	MACS_peak_1254	25.00	lobChIP_FOXA1_robot
chr1	236654024	236654025	MACS_peak_1255	7.00	lobChIP_FOXA1_robot
chr1	236678594	236678595	MACS_peak_1256	18.00	lobChIP_FOXA1_robot
chr1	236680754	236680755	MACS_peak_1257	13.00	lobChIP_FOXA1_robot
chr1	237007956	237007957	MACS_peak_1258	4.00	lobChIP_FOXA1_robot
chr1	237008486	237008487	MACS_peak_1259	8.00	lobChIP_FOXA1_robot
chr1	237009378	237009379	MACS_peak_1260	3.00	lobChIP_FOXA1_robot
chr1	237025285	237025286	MACS_peak_1261	6.00	lobChIP_FOXA1_robot
chr1	237026123	237026124	MACS_peak_1262	6.00	lobChIP_FOXA1_robot
chr1	237068640	237068641	MACS_peak_1263	4.00	lobChIP_FOXA1_robot
chr1	237130080	237130081	MACS_peak_1264	5.00	lobChIP_FOXA1_robot
chr1	241835863	241835864	MACS_peak_1265	6.00	lobChIP_FOXA1_robot
chr1	242012181	242012182	MACS_peak_1266	4.00	lobChIP_FOXA1_robot
chr1	242374728	242374729	MACS_peak_1267	8.00	lobChIP_FOXA1_robot
chr1	242905830	242905831	MACS_peak_1268	5.00	lobChIP_FOXA1_robot
chr1	243669457	243669458	MACS_peak_1269	4.00	lobChIP_FOXA1_robot
chr1	244065640	244065641	MACS_peak_1270	39.00	lobChIP_FOXA1_robot
chr1	244304864	244304865	MACS_peak_1271	4.00	lobChIP_FOXA1_robot
chr1	244436314	244436315	MACS_peak_1272	5.00	lobChIP_FOXA1_robot
chr1	244491044	244491045	MACS_peak_1273	17.00	lobChIP_FOXA1_robot
chr1	244997158	244997159	MACS_peak_1274	6.00	lobChIP_FOXA1_robot
chr1	245208943	245208944	MACS_peak_1275	12.00	lobChIP_FOXA1_robot
chr1	245224237	245224238	MACS_peak_1276	6.00	lobChIP_FOXA1_robot
chr1	245320679	245320680	MACS_peak_1277	8.00	lobChIP_FOXA1_robot
chr1	245421841	245421842	MACS_peak_1278	7.00	lobChIP_FOXA1_robot
chr1	245974468	245974469	MACS_peak_1279	12.00	lobChIP_FOXA1_robot
chr1	246329973	246329974	MACS_peak_1280	5.00	lobChIP_FOXA1_robot
chr1	246425158	246425159	MACS_peak_1281	5.00	lobChIP_FOXA1_robot
chr1	246445190	246445191	MACS_peak_1282	5.00	lobChIP_FOXA1_robot
chr1	246746898	246746899	MACS_peak_1283	15.00	lobChIP_FOXA1_robot
chr1	246751131	246751132	MACS_peak_1284	4.00	lobChIP_FOXA1_robot
chr1	246772161	246772162	MACS_peak_1285	6.00	lobChIP_FOXA1_robot
chr1	246859800	246859801	MACS_peak_1286	6.00	lobChIP_FOXA1_robot
chr1	246873334	246873335	MACS_peak_1287	24.00	lobChIP_FOXA1_robot
chr1	246889961	246889962	MACS_peak_1288	7.00	lobChIP_FOXA1_robot
chr1	247070332	247070333	MACS_peak_1289	7.00	lobChIP_FOXA1_robot
chr1	247242180	247242181	MACS_peak_1290	3.00	lobChIP_FOXA1_robot
chr10	179608	179609	MACS_peak_1291	9.00	lobChIP_FOXA1_robot
chr10	406666	406667	MACS_peak_1292	4.00	lobChIP_FOXA1_robot
chr10	434522	434523	MACS_peak_1293	5.00	lobChIP_FOXA1_robot
chr10	560122	560123	MACS_peak_1294	4.00	lobChIP_FOXA1_robot
chr10	1016478	1016479	MACS_peak_1295	20.00	lobChIP_FOXA1_robot
chr10	1020128	1020129	MACS_peak_1296	7.00	lobChIP_FOXA1_robot
chr10	1249095	1249096	MACS_peak_1297	8.00	lobChIP_FOXA1_robot
chr10	1474569	1474570	MACS_peak_1298	7.00	lobChIP_FOXA1_robot
chr10	2626331	2626332	MACS_peak_1299	6.00	lobChIP_FOXA1_robot
chr10	3137802	3137803	MACS_peak_1300	5.00	lobChIP_FOXA1_robot
chr10	3235247	3235248	MACS_peak_1301	4.00	lobChIP_FOXA1_robot
chr10	3408019	3408020	MACS_peak_1302	15.00	lobChIP_FOXA1_robot
chr10	3745049	3745050	MACS_peak_1303	5.00	lobChIP_FOXA1_robot
chr10	3782812	3782813	MACS_peak_1304	6.00	lobChIP_FOXA1_robot
chr10	3790445	3790446	MACS_peak_1305	9.00	lobChIP_FOXA1_robot
chr10	3826249	3826250	MACS_peak_1306	9.00	lobChIP_FOXA1_robot
chr10	3829041	3829042	MACS_peak_1307	4.00	lobChIP_FOXA1_robot
chr10	3852648	3852649	MACS_peak_1308	7.00	lobChIP_FOXA1_robot
chr10	3867616	3867617	MACS_peak_1309	6.00	lobChIP_FOXA1_robot
chr10	3873359	3873360	MACS_peak_1310	15.00	lobChIP_FOXA1_robot
chr10	3881355	3881356	MACS_peak_1311	3.00	lobChIP_FOXA1_robot
chr10	3893960	3893961	MACS_peak_1312	9.00	lobChIP_FOXA1_robot
chr10	3916048	3916049	MACS_peak_1313	4.00	lobChIP_FOXA1_robot
chr10	3921365	3921366	MACS_peak_1314	7.00	lobChIP_FOXA1_robot
chr10	3932430	3932431	MACS_peak_1315	8.00	lobChIP_FOXA1_robot
chr10	3978059	3978060	MACS_peak_1316	3.00	lobChIP_FOXA1_robot
chr10	4501997	4501998	MACS_peak_1317	4.00	lobChIP_FOXA1_robot
chr10	4550654	4550655	MACS_peak_1318	5.00	lobChIP_FOXA1_robot
chr10	4552498	4552499	MACS_peak_1319	5.00	lobChIP_FOXA1_robot
chr10	4768337	4768338	MACS_peak_1320	6.00	lobChIP_FOXA1_robot
chr10	4932150	4932151	MACS_peak_1321	5.00	lobChIP_FOXA1_robot
chr10	4989472	4989473	MACS_peak_1322	13.00	lobChIP_FOXA1_robot
chr10	5051513	5051514	MACS_peak_1323	12.00	lobChIP_FOXA1_robot
chr10	5090316	5090317	MACS_peak_1324	4.00	lobChIP_FOXA1_robot
chr10	5113524	5113525	MACS_peak_1325	3.00	lobChIP_FOXA1_robot
chr10	5114089	5114090	MACS_peak_1326	5.00	lobChIP_FOXA1_robot
chr10	5122202	5122203	MACS_peak_1327	7.00	lobChIP_FOXA1_robot
chr10	5132361	5132362	MACS_peak_1328	4.00	lobChIP_FOXA1_robot
chr10	5189654	5189655	MACS_peak_1329	31.00	lobChIP_FOXA1_robot
chr10	5190625	5190626	MACS_peak_1330	16.00	lobChIP_FOXA1_robot
chr10	5238096	5238097	MACS_peak_1331	8.00	lobChIP_FOXA1_robot
chr10	5261768	5261769	MACS_peak_1332	4.00	lobChIP_FOXA1_robot
chr10	5276363	5276364	MACS_peak_1333	3.00	lobChIP_FOXA1_robot
chr10	5285663	5285664	MACS_peak_1334	23.00	lobChIP_FOXA1_robot
chr10	5432736	5432737	MACS_peak_1335	4.00	lobChIP_FOXA1_robot
chr10	5535824	5535825	MACS_peak_1336	16.00	lobChIP_FOXA1_robot
chr10	5558255	5558256	MACS_peak_1337	19.00	lobChIP_FOXA1_robot
chr10	5560210	5560211	MACS_peak_1338	6.00	lobChIP_FOXA1_robot
chr10	5570556	5570557	MACS_peak_1339	6.00	lobChIP_FOXA1_robot
chr10	5590073	5590074	MACS_peak_1340	5.00	lobChIP_FOXA1_robot
chr10	5590857	5590858	MACS_peak_1341	3.00	lobChIP_FOXA1_robot
chr10	5596445	5596446	MACS_peak_1342	5.00	lobChIP_FOXA1_robot
chr10	5609674	5609675	MACS_peak_1343	5.00	lobChIP_FOXA1_robot
chr10	5613540	5613541	MACS_peak_1344	4.00	lobChIP_FOXA1_robot
chr10	5623250	5623251	MACS_peak_1345	5.00	lobChIP_FOXA1_robot
chr10	5631536	5631537	MACS_peak_1346	5.00	lobChIP_FOXA1_robot
chr10	5734769	5734770	MACS_peak_1347	6.00	lobChIP_FOXA1_robot
chr10	5804677	5804678	MACS_peak_1348	16.00	lobChIP_FOXA1_robot
chr10	5859770	5859771	MACS_peak_1349	6.00	lobChIP_FOXA1_robot
chr10	5903524	5903525	MACS_peak_1350	5.00	lobChIP_FOXA1_robot
chr10	6011047	6011048	MACS_peak_1351	5.00	lobChIP_FOXA1_robot
chr10	6128380	6128381	MACS_peak_1352	3.00	lobChIP_FOXA1_robot
chr10	6313871	6313872	MACS_peak_1353	3.00	lobChIP_FOXA1_robot
chr10	6321567	6321568	MACS_peak_1354	3.00	lobChIP_FOXA1_robot
chr10	6407545	6407546	MACS_peak_1355	5.00	lobChIP_FOXA1_robot
chr10	6487870	6487871	MACS_peak_1356	6.00	lobChIP_FOXA1_robot
chr10	6706937	6706938	MACS_peak_1357	4.00	lobChIP_FOXA1_robot
chr10	6879390	6879391	MACS_peak_1358	3.00	lobChIP_FOXA1_robot
chr10	7555563	7555564	MACS_peak_1359	3.00	lobChIP_FOXA1_robot
chr10	7582762	7582763	MACS_peak_1360	8.00	lobChIP_FOXA1_robot
chr10	7583787	7583788	MACS_peak_1361	3.00	lobChIP_FOXA1_robot
chr10	7584367	7584368	MACS_peak_1362	9.00	lobChIP_FOXA1_robot
chr10	7728606	7728607	MACS_peak_1363	13.00	lobChIP_FOXA1_robot
chr10	7738770	7738771	MACS_peak_1364	17.00	lobChIP_FOXA1_robot
chr10	7742788	7742789	MACS_peak_1365	24.00	lobChIP_FOXA1_robot
chr10	7745247	7745248	MACS_peak_1366	11.00	lobChIP_FOXA1_robot
chr10	7749269	7749270	MACS_peak_1367	22.00	lobChIP_FOXA1_robot
chr10	7837834	7837835	MACS_peak_1368	12.00	lobChIP_FOXA1_robot
chr10	7974425	7974426	MACS_peak_1369	6.00	lobChIP_FOXA1_robot
chr10	8092755	8092756	MACS_peak_1370	16.00	lobChIP_FOXA1_robot
chr10	8658385	8658386	MACS_peak_1371	4.00	lobChIP_FOXA1_robot
chr10	9373943	9373944	MACS_peak_1372	4.00	lobChIP_FOXA1_robot
chr10	10235388	10235389	MACS_peak_1373	6.00	lobChIP_FOXA1_robot
chr10	11565063	11565064	MACS_peak_1374	7.00	lobChIP_FOXA1_robot
chr10	11587760	11587761	MACS_peak_1375	4.00	lobChIP_FOXA1_robot
chr10	11602669	11602670	MACS_peak_1376	7.00	lobChIP_FOXA1_robot
chr10	11610178	11610179	MACS_peak_1377	4.00	lobChIP_FOXA1_robot
chr10	11645089	11645090	MACS_peak_1378	21.00	lobChIP_FOXA1_robot
chr10	11682374	11682375	MACS_peak_1379	20.00	lobChIP_FOXA1_robot
chr10	11727887	11727888	MACS_peak_1380	4.00	lobChIP_FOXA1_robot
chr10	11739804	11739805	MACS_peak_1381	4.00	lobChIP_FOXA1_robot
chr10	11758139	11758140	MACS_peak_1382	15.00	lobChIP_FOXA1_robot
chr10	11799638	11799639	MACS_peak_1383	7.00	lobChIP_FOXA1_robot
chr10	11866397	11866398	MACS_peak_1384	4.00	lobChIP_FOXA1_robot
chr10	11873876	11873877	MACS_peak_1385	12.00	lobChIP_FOXA1_robot
chr10	11917917	11917918	MACS_peak_1386	6.00	lobChIP_FOXA1_robot
chr10	11992436	11992437	MACS_peak_1387	8.00	lobChIP_FOXA1_robot
chr10	12028762	12028763	MACS_peak_1388	12.00	lobChIP_FOXA1_robot
chr10	12110669	12110670	MACS_peak_1389	7.00	lobChIP_FOXA1_robot
chr10	12279837	12279838	MACS_peak_1390	13.00	lobChIP_FOXA1_robot
chr10	12304840	12304841	MACS_peak_1391	9.00	lobChIP_FOXA1_robot
chr10	12543472	12543473	MACS_peak_1392	4.00	lobChIP_FOXA1_robot
chr10	12647828	12647829	MACS_peak_1393	7.00	lobChIP_FOXA1_robot
chr10	12673957	12673958	MACS_peak_1394	7.00	lobChIP_FOXA1_robot
chr10	12762598	12762599	MACS_peak_1395	10.00	lobChIP_FOXA1_robot
chr10	13082385	13082386	MACS_peak_1396	6.00	lobChIP_FOXA1_robot
chr10	13149794	13149795	MACS_peak_1397	17.00	lobChIP_FOXA1_robot
chr10	13325432	13325433	MACS_peak_1398	5.00	lobChIP_FOXA1_robot
chr10	13344120	13344121	MACS_peak_1399	6.00	lobChIP_FOXA1_robot
chr10	13456938	13456939	MACS_peak_1400	6.00	lobChIP_FOXA1_robot
chr10	13542294	13542295	MACS_peak_1401	7.00	lobChIP_FOXA1_robot
chr10	14226968	14226969	MACS_peak_1402	6.00	lobChIP_FOXA1_robot
chr10	14302366	14302367	MACS_peak_1403	5.00	lobChIP_FOXA1_robot
chr10	14592602	14592603	MACS_peak_1404	6.00	lobChIP_FOXA1_robot
chr10	14614205	14614206	MACS_peak_1405	8.00	lobChIP_FOXA1_robot
chr10	14620596	14620597	MACS_peak_1406	8.00	lobChIP_FOXA1_robot
chr10	14630354	14630355	MACS_peak_1407	4.00	lobChIP_FOXA1_robot
chr10	14654094	14654095	MACS_peak_1408	3.00	lobChIP_FOXA1_robot
chr10	15126698	15126699	MACS_peak_1409	10.00	lobChIP_FOXA1_robot
chr10	15179068	15179069	MACS_peak_1410	5.00	lobChIP_FOXA1_robot
chr10	15363056	15363057	MACS_peak_1411	4.00	lobChIP_FOXA1_robot
chr10	15376486	15376487	MACS_peak_1412	10.00	lobChIP_FOXA1_robot
chr10	15387060	15387061	MACS_peak_1413	10.00	lobChIP_FOXA1_robot
chr10	15395311	15395312	MACS_peak_1414	4.00	lobChIP_FOXA1_robot
chr10	15736514	15736515	MACS_peak_1415	6.00	lobChIP_FOXA1_robot
chr10	16556389	16556390	MACS_peak_1416	4.00	lobChIP_FOXA1_robot
chr10	17476590	17476591	MACS_peak_1417	3.00	lobChIP_FOXA1_robot
chr10	17710997	17710998	MACS_peak_1418	13.00	lobChIP_FOXA1_robot
chr10	17715572	17715573	MACS_peak_1419	5.00	lobChIP_FOXA1_robot
chr10	18899648	18899649	MACS_peak_1420	22.00	lobChIP_FOXA1_robot
chr10	18904669	18904670	MACS_peak_1421	3.00	lobChIP_FOXA1_robot
chr10	18926574	18926575	MACS_peak_1422	11.00	lobChIP_FOXA1_robot
chr10	19390467	19390468	MACS_peak_1423	4.00	lobChIP_FOXA1_robot
chr10	19658058	19658059	MACS_peak_1424	3.00	lobChIP_FOXA1_robot
chr10	19766926	19766927	MACS_peak_1425	3.00	lobChIP_FOXA1_robot
chr10	19780785	19780786	MACS_peak_1426	5.00	lobChIP_FOXA1_robot
chr10	19781708	19781709	MACS_peak_1427	4.00	lobChIP_FOXA1_robot
chr10	20048243	20048244	MACS_peak_1428	4.00	lobChIP_FOXA1_robot
chr10	20208096	20208097	MACS_peak_1429	9.00	lobChIP_FOXA1_robot
chr10	20377400	20377401	MACS_peak_1430	8.00	lobChIP_FOXA1_robot
chr10	21255483	21255484	MACS_peak_1431	3.00	lobChIP_FOXA1_robot
chr10	21275198	21275199	MACS_peak_1432	4.00	lobChIP_FOXA1_robot
chr10	21374685	21374686	MACS_peak_1433	4.00	lobChIP_FOXA1_robot
chr10	21439440	21439441	MACS_peak_1434	4.00	lobChIP_FOXA1_robot
chr10	21461536	21461537	MACS_peak_1435	9.00	lobChIP_FOXA1_robot
chr10	21626099	21626100	MACS_peak_1436	3.00	lobChIP_FOXA1_robot
chr10	21661439	21661440	MACS_peak_1437	5.00	lobChIP_FOXA1_robot
chr10	21794984	21794985	MACS_peak_1438	12.00	lobChIP_FOXA1_robot
chr10	21800408	21800409	MACS_peak_1439	6.00	lobChIP_FOXA1_robot
chr10	21806884	21806885	MACS_peak_1440	3.00	lobChIP_FOXA1_robot
chr10	21814382	21814383	MACS_peak_1441	4.00	lobChIP_FOXA1_robot
chr10	21856222	21856223	MACS_peak_1442	18.00	lobChIP_FOXA1_robot
chr10	21859932	21859933	MACS_peak_1443	5.00	lobChIP_FOXA1_robot
chr10	22013707	22013708	MACS_peak_1444	4.00	lobChIP_FOXA1_robot
chr10	22016880	22016881	MACS_peak_1445	9.00	lobChIP_FOXA1_robot
chr10	22216045	22216046	MACS_peak_1446	4.00	lobChIP_FOXA1_robot
chr10	22375709	22375710	MACS_peak_1447	4.00	lobChIP_FOXA1_robot
chr10	22605991	22605992	MACS_peak_1448	11.00	lobChIP_FOXA1_robot
chr10	22824075	22824076	MACS_peak_1449	7.00	lobChIP_FOXA1_robot
chr10	22895502	22895503	MACS_peak_1450	5.00	lobChIP_FOXA1_robot
chr10	22907319	22907320	MACS_peak_1451	5.00	lobChIP_FOXA1_robot
chr10	22913356	22913357	MACS_peak_1452	25.00	lobChIP_FOXA1_robot
chr10	22920769	22920770	MACS_peak_1453	5.00	lobChIP_FOXA1_robot
chr10	22970753	22970754	MACS_peak_1454	5.00	lobChIP_FOXA1_robot
chr10	22971515	22971516	MACS_peak_1455	6.00	lobChIP_FOXA1_robot
chr10	22972371	22972372	MACS_peak_1456	19.00	lobChIP_FOXA1_robot
chr10	23049149	23049150	MACS_peak_1457	5.00	lobChIP_FOXA1_robot
chr10	23248405	23248406	MACS_peak_1458	8.00	lobChIP_FOXA1_robot
chr10	23475745	23475746	MACS_peak_1459	11.00	lobChIP_FOXA1_robot
chr10	23542887	23542888	MACS_peak_1460	4.00	lobChIP_FOXA1_robot
chr10	24276696	24276697	MACS_peak_1461	4.00	lobChIP_FOXA1_robot
chr10	24497253	24497254	MACS_peak_1462	4.00	lobChIP_FOXA1_robot
chr10	24526946	24526947	MACS_peak_1463	5.00	lobChIP_FOXA1_robot
chr10	24557127	24557128	MACS_peak_1464	4.00	lobChIP_FOXA1_robot
chr10	24607595	24607596	MACS_peak_1465	4.00	lobChIP_FOXA1_robot
chr10	24731304	24731305	MACS_peak_1466	5.00	lobChIP_FOXA1_robot
chr10	24755024	24755025	MACS_peak_1467	6.00	lobChIP_FOXA1_robot
chr10	24830438	24830439	MACS_peak_1468	13.00	lobChIP_FOXA1_robot
chr10	24842442	24842443	MACS_peak_1469	5.00	lobChIP_FOXA1_robot
chr10	24932126	24932127	MACS_peak_1470	7.00	lobChIP_FOXA1_robot
chr10	24962367	24962368	MACS_peak_1471	3.00	lobChIP_FOXA1_robot
chr10	24970235	24970236	MACS_peak_1472	27.00	lobChIP_FOXA1_robot
chr10	25009309	25009310	MACS_peak_1473	4.00	lobChIP_FOXA1_robot
chr10	27023218	27023219	MACS_peak_1474	4.00	lobChIP_FOXA1_robot
chr10	27372211	27372212	MACS_peak_1475	7.00	lobChIP_FOXA1_robot
chr10	27555135	27555136	MACS_peak_1476	7.00	lobChIP_FOXA1_robot
chr10	27974020	27974021	MACS_peak_1477	4.00	lobChIP_FOXA1_robot
chr10	28734302	28734303	MACS_peak_1478	5.00	lobChIP_FOXA1_robot
chr10	28858817	28858818	MACS_peak_1479	14.00	lobChIP_FOXA1_robot
chr10	28971400	28971401	MACS_peak_1480	4.00	lobChIP_FOXA1_robot
chr10	29003500	29003501	MACS_peak_1481	5.00	lobChIP_FOXA1_robot
chr10	29058387	29058388	MACS_peak_1482	8.00	lobChIP_FOXA1_robot
chr10	29063139	29063140	MACS_peak_1483	12.00	lobChIP_FOXA1_robot
chr10	29086889	29086890	MACS_peak_1484	5.00	lobChIP_FOXA1_robot
chr10	29095546	29095547	MACS_peak_1485	5.00	lobChIP_FOXA1_robot
chr10	29128101	29128102	MACS_peak_1486	7.00	lobChIP_FOXA1_robot
chr10	29170828	29170829	MACS_peak_1487	3.00	lobChIP_FOXA1_robot
chr10	29173552	29173553	MACS_peak_1488	20.00	lobChIP_FOXA1_robot
chr10	29229028	29229029	MACS_peak_1489	77.00	lobChIP_FOXA1_robot
chr10	29419357	29419358	MACS_peak_1490	5.00	lobChIP_FOXA1_robot
chr10	29512788	29512789	MACS_peak_1491	5.00	lobChIP_FOXA1_robot
chr10	29607302	29607303	MACS_peak_1492	14.00	lobChIP_FOXA1_robot
chr10	29616589	29616590	MACS_peak_1493	30.00	lobChIP_FOXA1_robot
chr10	29681844	29681845	MACS_peak_1494	6.00	lobChIP_FOXA1_robot
chr10	29785568	29785569	MACS_peak_1495	5.00	lobChIP_FOXA1_robot
chr10	29824452	29824453	MACS_peak_1496	5.00	lobChIP_FOXA1_robot
chr10	29907756	29907757	MACS_peak_1497	5.00	lobChIP_FOXA1_robot
chr10	29908350	29908351	MACS_peak_1498	16.00	lobChIP_FOXA1_robot
chr10	29917008	29917009	MACS_peak_1499	5.00	lobChIP_FOXA1_robot
chr10	30598492	30598493	MACS_peak_1500	4.00	lobChIP_FOXA1_robot
chr10	30727787	30727788	MACS_peak_1501	7.00	lobChIP_FOXA1_robot
chr10	30811790	30811791	MACS_peak_1502	4.00	lobChIP_FOXA1_robot
chr10	30817007	30817008	MACS_peak_1503	9.00	lobChIP_FOXA1_robot
chr10	30827195	30827196	MACS_peak_1504	7.00	lobChIP_FOXA1_robot
chr10	30831360	30831361	MACS_peak_1505	16.00	lobChIP_FOXA1_robot
chr10	30843251	30843252	MACS_peak_1506	3.00	lobChIP_FOXA1_robot
chr10	30847474	30847475	MACS_peak_1507	4.00	lobChIP_FOXA1_robot
chr10	30891899	30891900	MACS_peak_1508	8.00	lobChIP_FOXA1_robot
chr10	30894953	30894954	MACS_peak_1509	26.00	lobChIP_FOXA1_robot
chr10	31307905	31307906	MACS_peak_1510	5.00	lobChIP_FOXA1_robot
chr10	31371045	31371046	MACS_peak_1511	5.00	lobChIP_FOXA1_robot
chr10	32154076	32154077	MACS_peak_1512	5.00	lobChIP_FOXA1_robot
chr10	32226173	32226174	MACS_peak_1513	6.00	lobChIP_FOXA1_robot
chr10	32230176	32230177	MACS_peak_1514	6.00	lobChIP_FOXA1_robot
chr10	32232074	32232075	MACS_peak_1515	6.00	lobChIP_FOXA1_robot
chr10	32233934	32233935	MACS_peak_1516	13.00	lobChIP_FOXA1_robot
chr10	32348625	32348626	MACS_peak_1517	11.00	lobChIP_FOXA1_robot
chr10	32384250	32384251	MACS_peak_1518	12.00	lobChIP_FOXA1_robot
chr10	32394211	32394212	MACS_peak_1519	7.00	lobChIP_FOXA1_robot
chr10	32394951	32394952	MACS_peak_1520	22.00	lobChIP_FOXA1_robot
chr10	32409221	32409222	MACS_peak_1521	7.00	lobChIP_FOXA1_robot
chr10	33084722	33084723	MACS_peak_1522	9.00	lobChIP_FOXA1_robot
chr10	33241462	33241463	MACS_peak_1523	4.00	lobChIP_FOXA1_robot
chr10	33294890	33294891	MACS_peak_1524	3.00	lobChIP_FOXA1_robot
chr10	33445133	33445134	MACS_peak_1525	87.00	lobChIP_FOXA1_robot
chr10	33446235	33446236	MACS_peak_1526	4.00	lobChIP_FOXA1_robot
chr10	33452458	33452459	MACS_peak_1527	4.00	lobChIP_FOXA1_robot
chr10	33485017	33485018	MACS_peak_1528	16.00	lobChIP_FOXA1_robot
chr10	33487736	33487737	MACS_peak_1529	14.00	lobChIP_FOXA1_robot
chr10	33526840	33526841	MACS_peak_1530	5.00	lobChIP_FOXA1_robot
chr10	33553349	33553350	MACS_peak_1531	13.00	lobChIP_FOXA1_robot
chr10	33576257	33576258	MACS_peak_1532	7.00	lobChIP_FOXA1_robot
chr10	33595858	33595859	MACS_peak_1533	7.00	lobChIP_FOXA1_robot
chr10	33616002	33616003	MACS_peak_1534	6.00	lobChIP_FOXA1_robot
chr10	33617700	33617701	MACS_peak_1535	3.00	lobChIP_FOXA1_robot
chr10	33620040	33620041	MACS_peak_1536	5.00	lobChIP_FOXA1_robot
chr10	33797065	33797066	MACS_peak_1537	15.00	lobChIP_FOXA1_robot
chr10	33798340	33798341	MACS_peak_1538	13.00	lobChIP_FOXA1_robot
chr10	34118411	34118412	MACS_peak_1539	3.00	lobChIP_FOXA1_robot
chr10	34275633	34275634	MACS_peak_1540	7.00	lobChIP_FOXA1_robot
chr10	34386802	34386803	MACS_peak_1541	10.00	lobChIP_FOXA1_robot
chr10	34405372	34405373	MACS_peak_1542	5.00	lobChIP_FOXA1_robot
chr10	34410508	34410509	MACS_peak_1543	11.00	lobChIP_FOXA1_robot
chr10	34453077	34453078	MACS_peak_1544	4.00	lobChIP_FOXA1_robot
chr10	34474136	34474137	MACS_peak_1545	8.00	lobChIP_FOXA1_robot
chr10	34502593	34502594	MACS_peak_1546	15.00	lobChIP_FOXA1_robot
chr10	34505572	34505573	MACS_peak_1547	10.00	lobChIP_FOXA1_robot
chr10	34520648	34520649	MACS_peak_1548	4.00	lobChIP_FOXA1_robot
chr10	34543795	34543796	MACS_peak_1549	3.00	lobChIP_FOXA1_robot
chr10	34634387	34634388	MACS_peak_1550	15.00	lobChIP_FOXA1_robot
chr10	34694882	34694883	MACS_peak_1551	5.00	lobChIP_FOXA1_robot
chr10	34712112	34712113	MACS_peak_1552	4.00	lobChIP_FOXA1_robot
chr10	34819970	34819971	MACS_peak_1553	8.00	lobChIP_FOXA1_robot
chr10	34926835	34926836	MACS_peak_1554	7.00	lobChIP_FOXA1_robot
chr10	35000611	35000612	MACS_peak_1555	6.00	lobChIP_FOXA1_robot
chr10	35002980	35002981	MACS_peak_1556	15.00	lobChIP_FOXA1_robot
chr10	35020890	35020891	MACS_peak_1557	4.00	lobChIP_FOXA1_robot
chr10	35026097	35026098	MACS_peak_1558	40.00	lobChIP_FOXA1_robot
chr10	35507482	35507483	MACS_peak_1559	4.00	lobChIP_FOXA1_robot
chr10	35509762	35509763	MACS_peak_1560	5.00	lobChIP_FOXA1_robot
chr10	35653935	35653936	MACS_peak_1561	7.00	lobChIP_FOXA1_robot
chr10	35676700	35676701	MACS_peak_1562	4.00	lobChIP_FOXA1_robot
chr10	35736678	35736679	MACS_peak_1563	5.00	lobChIP_FOXA1_robot
chr10	35901890	35901891	MACS_peak_1564	4.00	lobChIP_FOXA1_robot
chr10	35974529	35974530	MACS_peak_1565	9.00	lobChIP_FOXA1_robot
chr10	36222567	36222568	MACS_peak_1566	6.00	lobChIP_FOXA1_robot
chr10	36987324	36987325	MACS_peak_1567	4.00	lobChIP_FOXA1_robot
chr10	37178007	37178008	MACS_peak_1568	5.00	lobChIP_FOXA1_robot
chr10	37912606	37912607	MACS_peak_1569	5.00	lobChIP_FOXA1_robot
chr10	38078682	38078683	MACS_peak_1570	8.00	lobChIP_FOXA1_robot
chr10	42379909	42379910	MACS_peak_1574	44.00	lobChIP_FOXA1_robot
chr10	43039510	43039511	MACS_peak_1579	3.00	lobChIP_FOXA1_robot
chr10	43636048	43636049	MACS_peak_1580	8.00	lobChIP_FOXA1_robot
chr10	43891669	43891670	MACS_peak_1581	4.00	lobChIP_FOXA1_robot
chr10	43957527	43957528	MACS_peak_1582	4.00	lobChIP_FOXA1_robot
chr10	44359534	44359535	MACS_peak_1583	11.00	lobChIP_FOXA1_robot
chr10	44691188	44691189	MACS_peak_1584	19.00	lobChIP_FOXA1_robot
chr10	44732489	44732490	MACS_peak_1585	29.00	lobChIP_FOXA1_robot
chr10	44749412	44749413	MACS_peak_1586	3.00	lobChIP_FOXA1_robot
chr10	44754657	44754658	MACS_peak_1587	7.00	lobChIP_FOXA1_robot
chr10	44820709	44820710	MACS_peak_1588	4.00	lobChIP_FOXA1_robot
chr10	44988367	44988368	MACS_peak_1589	5.00	lobChIP_FOXA1_robot
chr10	45241498	45241499	MACS_peak_1590	6.00	lobChIP_FOXA1_robot
chr10	45435651	45435652	MACS_peak_1591	4.00	lobChIP_FOXA1_robot
chr10	45473699	45473700	MACS_peak_1592	4.00	lobChIP_FOXA1_robot
chr10	45474402	45474403	MACS_peak_1593	6.00	lobChIP_FOXA1_robot
chr10	45476426	45476427	MACS_peak_1594	9.00	lobChIP_FOXA1_robot
chr10	45485831	45485832	MACS_peak_1595	6.00	lobChIP_FOXA1_robot
chr10	45491527	45491528	MACS_peak_1596	12.00	lobChIP_FOXA1_robot
chr10	45501048	45501049	MACS_peak_1597	4.00	lobChIP_FOXA1_robot
chr10	45525024	45525025	MACS_peak_1598	4.00	lobChIP_FOXA1_robot
chr10	45965609	45965610	MACS_peak_1599	3.00	lobChIP_FOXA1_robot
chr10	45966249	45966250	MACS_peak_1600	13.00	lobChIP_FOXA1_robot
chr10	45998128	45998129	MACS_peak_1601	16.00	lobChIP_FOXA1_robot
chr10	46054128	46054129	MACS_peak_1602	5.00	lobChIP_FOXA1_robot
chr10	46071706	46071707	MACS_peak_1603	4.00	lobChIP_FOXA1_robot
chr10	46094945	46094946	MACS_peak_1604	4.00	lobChIP_FOXA1_robot
chr10	47102045	47102046	MACS_peak_1605	4.00	lobChIP_FOXA1_robot
chr10	49503777	49503778	MACS_peak_1606	5.00	lobChIP_FOXA1_robot
chr10	49571859	49571860	MACS_peak_1607	6.00	lobChIP_FOXA1_robot
chr10	49625064	49625065	MACS_peak_1608	8.00	lobChIP_FOXA1_robot
chr10	51053306	51053307	MACS_peak_1609	5.00	lobChIP_FOXA1_robot
chr10	51543232	51543233	MACS_peak_1610	45.00	lobChIP_FOXA1_robot
chr10	51559728	51559729	MACS_peak_1611	4.00	lobChIP_FOXA1_robot
chr10	51563675	51563676	MACS_peak_1612	27.00	lobChIP_FOXA1_robot
chr10	51566652	51566653	MACS_peak_1613	4.00	lobChIP_FOXA1_robot
chr10	51574733	51574734	MACS_peak_1614	10.00	lobChIP_FOXA1_robot
chr10	51593219	51593220	MACS_peak_1615	21.00	lobChIP_FOXA1_robot
chr10	52152448	52152449	MACS_peak_1616	4.00	lobChIP_FOXA1_robot
chr10	52155319	52155320	MACS_peak_1617	11.00	lobChIP_FOXA1_robot
chr10	52261790	52261791	MACS_peak_1618	6.00	lobChIP_FOXA1_robot
chr10	52270988	52270989	MACS_peak_1619	4.00	lobChIP_FOXA1_robot
chr10	52326143	52326144	MACS_peak_1620	10.00	lobChIP_FOXA1_robot
chr10	52339888	52339889	MACS_peak_1621	7.00	lobChIP_FOXA1_robot
chr10	52364670	52364671	MACS_peak_1622	5.00	lobChIP_FOXA1_robot
chr10	52365771	52365772	MACS_peak_1623	11.00	lobChIP_FOXA1_robot
chr10	52366496	52366497	MACS_peak_1624	6.00	lobChIP_FOXA1_robot
chr10	52568332	52568333	MACS_peak_1625	8.00	lobChIP_FOXA1_robot
chr10	52642504	52642505	MACS_peak_1626	17.00	lobChIP_FOXA1_robot
chr10	52650953	52650954	MACS_peak_1627	5.00	lobChIP_FOXA1_robot
chr10	53972827	53972828	MACS_peak_1628	6.00	lobChIP_FOXA1_robot
chr10	53981942	53981943	MACS_peak_1629	4.00	lobChIP_FOXA1_robot
chr10	54073947	54073948	MACS_peak_1630	11.00	lobChIP_FOXA1_robot
chr10	54092864	54092865	MACS_peak_1631	5.00	lobChIP_FOXA1_robot
chr10	54094559	54094560	MACS_peak_1632	5.00	lobChIP_FOXA1_robot
chr10	54113759	54113760	MACS_peak_1633	6.00	lobChIP_FOXA1_robot
chr10	54136692	54136693	MACS_peak_1634	11.00	lobChIP_FOXA1_robot
chr10	54138130	54138131	MACS_peak_1635	15.00	lobChIP_FOXA1_robot
chr10	54212584	54212585	MACS_peak_1636	30.00	lobChIP_FOXA1_robot
chr10	54397505	54397506	MACS_peak_1637	12.00	lobChIP_FOXA1_robot
chr10	54528194	54528195	MACS_peak_1638	10.00	lobChIP_FOXA1_robot
chr10	54531500	54531501	MACS_peak_1639	8.00	lobChIP_FOXA1_robot
chr10	54534487	54534488	MACS_peak_1640	17.00	lobChIP_FOXA1_robot
chr10	54536051	54536052	MACS_peak_1641	4.00	lobChIP_FOXA1_robot
chr10	54540044	54540045	MACS_peak_1642	4.00	lobChIP_FOXA1_robot
chr10	54643858	54643859	MACS_peak_1643	14.00	lobChIP_FOXA1_robot
chr10	54766994	54766995	MACS_peak_1644	15.00	lobChIP_FOXA1_robot
chr10	54770368	54770369	MACS_peak_1645	3.00	lobChIP_FOXA1_robot
chr10	55361128	55361129	MACS_peak_1646	14.00	lobChIP_FOXA1_robot
chr10	55646574	55646575	MACS_peak_1647	5.00	lobChIP_FOXA1_robot
chr10	59997390	59997391	MACS_peak_1648	5.00	lobChIP_FOXA1_robot
chr10	59998937	59998938	MACS_peak_1649	4.00	lobChIP_FOXA1_robot
chr10	60065760	60065761	MACS_peak_1650	3.00	lobChIP_FOXA1_robot
chr10	60082443	60082444	MACS_peak_1651	5.00	lobChIP_FOXA1_robot
chr10	60108459	60108460	MACS_peak_1652	4.00	lobChIP_FOXA1_robot
chr10	60110635	60110636	MACS_peak_1653	5.00	lobChIP_FOXA1_robot
chr10	60212951	60212952	MACS_peak_1654	3.00	lobChIP_FOXA1_robot
chr10	60218234	60218235	MACS_peak_1655	6.00	lobChIP_FOXA1_robot
chr10	60227835	60227836	MACS_peak_1656	3.00	lobChIP_FOXA1_robot
chr10	60228538	60228539	MACS_peak_1657	117.00	lobChIP_FOXA1_robot
chr10	60229338	60229339	MACS_peak_1658	4.00	lobChIP_FOXA1_robot
chr10	60315531	60315532	MACS_peak_1659	7.00	lobChIP_FOXA1_robot
chr10	60316392	60316393	MACS_peak_1660	5.00	lobChIP_FOXA1_robot
chr10	60401158	60401159	MACS_peak_1661	4.00	lobChIP_FOXA1_robot
chr10	60422253	60422254	MACS_peak_1662	6.00	lobChIP_FOXA1_robot
chr10	60577457	60577458	MACS_peak_1663	4.00	lobChIP_FOXA1_robot
chr10	60578832	60578833	MACS_peak_1664	5.00	lobChIP_FOXA1_robot
chr10	60711021	60711022	MACS_peak_1665	5.00	lobChIP_FOXA1_robot
chr10	60754098	60754099	MACS_peak_1666	8.00	lobChIP_FOXA1_robot
chr10	60766634	60766635	MACS_peak_1667	19.00	lobChIP_FOXA1_robot
chr10	60797469	60797470	MACS_peak_1668	10.00	lobChIP_FOXA1_robot
chr10	60818001	60818002	MACS_peak_1669	38.00	lobChIP_FOXA1_robot
chr10	60833531	60833532	MACS_peak_1670	67.00	lobChIP_FOXA1_robot
chr10	60867195	60867196	MACS_peak_1671	4.00	lobChIP_FOXA1_robot
chr10	60868796	60868797	MACS_peak_1672	4.00	lobChIP_FOXA1_robot
chr10	60939678	60939679	MACS_peak_1673	6.00	lobChIP_FOXA1_robot
chr10	60976137	60976138	MACS_peak_1674	8.00	lobChIP_FOXA1_robot
chr10	61082896	61082897	MACS_peak_1675	10.00	lobChIP_FOXA1_robot
chr10	61437444	61437445	MACS_peak_1676	5.00	lobChIP_FOXA1_robot
chr10	61462382	61462383	MACS_peak_1677	5.00	lobChIP_FOXA1_robot
chr10	61521228	61521229	MACS_peak_1678	4.00	lobChIP_FOXA1_robot
chr10	61655626	61655627	MACS_peak_1679	4.00	lobChIP_FOXA1_robot
chr10	61731919	61731920	MACS_peak_1680	9.00	lobChIP_FOXA1_robot
chr10	62042822	62042823	MACS_peak_1681	27.00	lobChIP_FOXA1_robot
chr10	62182685	62182686	MACS_peak_1682	7.00	lobChIP_FOXA1_robot
chr10	62561399	62561400	MACS_peak_1683	4.00	lobChIP_FOXA1_robot
chr10	62702527	62702528	MACS_peak_1684	8.00	lobChIP_FOXA1_robot
chr10	62712858	62712859	MACS_peak_1685	5.00	lobChIP_FOXA1_robot
chr10	62716156	62716157	MACS_peak_1686	20.00	lobChIP_FOXA1_robot
chr10	62724672	62724673	MACS_peak_1687	3.00	lobChIP_FOXA1_robot
chr10	62737682	62737683	MACS_peak_1688	5.00	lobChIP_FOXA1_robot
chr10	62822960	62822961	MACS_peak_1689	43.00	lobChIP_FOXA1_robot
chr10	62824942	62824943	MACS_peak_1690	20.00	lobChIP_FOXA1_robot
chr10	62826876	62826877	MACS_peak_1691	45.00	lobChIP_FOXA1_robot
chr10	62831446	62831447	MACS_peak_1692	4.00	lobChIP_FOXA1_robot
chr10	62835437	62835438	MACS_peak_1693	39.00	lobChIP_FOXA1_robot
chr10	63593649	63593650	MACS_peak_1694	4.00	lobChIP_FOXA1_robot
chr10	63597145	63597146	MACS_peak_1695	17.00	lobChIP_FOXA1_robot
chr10	63599846	63599847	MACS_peak_1696	4.00	lobChIP_FOXA1_robot
chr10	63678033	63678034	MACS_peak_1697	7.00	lobChIP_FOXA1_robot
chr10	63710747	63710748	MACS_peak_1698	4.00	lobChIP_FOXA1_robot
chr10	63712263	63712264	MACS_peak_1699	9.00	lobChIP_FOXA1_robot
chr10	63724420	63724421	MACS_peak_1700	33.00	lobChIP_FOXA1_robot
chr10	63727136	63727137	MACS_peak_1701	8.00	lobChIP_FOXA1_robot
chr10	63788067	63788068	MACS_peak_1702	6.00	lobChIP_FOXA1_robot
chr10	63797852	63797853	MACS_peak_1703	13.00	lobChIP_FOXA1_robot
chr10	63805629	63805630	MACS_peak_1704	10.00	lobChIP_FOXA1_robot
chr10	63809288	63809289	MACS_peak_1705	3.00	lobChIP_FOXA1_robot
chr10	63842275	63842276	MACS_peak_1706	7.00	lobChIP_FOXA1_robot
chr10	63853748	63853749	MACS_peak_1707	12.00	lobChIP_FOXA1_robot
chr10	63855562	63855563	MACS_peak_1708	19.00	lobChIP_FOXA1_robot
chr10	63856448	63856449	MACS_peak_1709	4.00	lobChIP_FOXA1_robot
chr10	63856900	63856901	MACS_peak_1710	6.00	lobChIP_FOXA1_robot
chr10	63943905	63943906	MACS_peak_1711	38.00	lobChIP_FOXA1_robot
chr10	64251552	64251553	MACS_peak_1712	7.00	lobChIP_FOXA1_robot
chr10	65026565	65026566	MACS_peak_1713	4.00	lobChIP_FOXA1_robot
chr10	65028114	65028115	MACS_peak_1714	8.00	lobChIP_FOXA1_robot
chr10	65229293	65229294	MACS_peak_1715	4.00	lobChIP_FOXA1_robot
chr10	65238073	65238074	MACS_peak_1716	4.00	lobChIP_FOXA1_robot
chr10	65247922	65247923	MACS_peak_1717	8.00	lobChIP_FOXA1_robot
chr10	65265508	65265509	MACS_peak_1718	5.00	lobChIP_FOXA1_robot
chr10	65266491	65266492	MACS_peak_1719	7.00	lobChIP_FOXA1_robot
chr10	65278661	65278662	MACS_peak_1720	4.00	lobChIP_FOXA1_robot
chr10	65280674	65280675	MACS_peak_1721	6.00	lobChIP_FOXA1_robot
chr10	65291591	65291592	MACS_peak_1722	3.00	lobChIP_FOXA1_robot
chr10	65804130	65804131	MACS_peak_1723	14.00	lobChIP_FOXA1_robot
chr10	65835491	65835492	MACS_peak_1724	5.00	lobChIP_FOXA1_robot
chr10	66660652	66660653	MACS_peak_1725	3.00	lobChIP_FOXA1_robot
chr10	66793537	66793538	MACS_peak_1726	8.00	lobChIP_FOXA1_robot
chr10	67261133	67261134	MACS_peak_1727	6.00	lobChIP_FOXA1_robot
chr10	68043836	68043837	MACS_peak_1728	7.00	lobChIP_FOXA1_robot
chr10	68261273	68261274	MACS_peak_1729	5.00	lobChIP_FOXA1_robot
chr10	68332654	68332655	MACS_peak_1730	4.00	lobChIP_FOXA1_robot
chr10	68691392	68691393	MACS_peak_1731	8.00	lobChIP_FOXA1_robot
chr10	68874930	68874931	MACS_peak_1732	10.00	lobChIP_FOXA1_robot
chr10	69001377	69001378	MACS_peak_1733	6.00	lobChIP_FOXA1_robot
chr10	69487253	69487254	MACS_peak_1734	4.00	lobChIP_FOXA1_robot
chr10	69553227	69553228	MACS_peak_1735	5.00	lobChIP_FOXA1_robot
chr10	69563530	69563531	MACS_peak_1736	46.00	lobChIP_FOXA1_robot
chr10	69586365	69586366	MACS_peak_1737	4.00	lobChIP_FOXA1_robot
chr10	69807268	69807269	MACS_peak_1738	4.00	lobChIP_FOXA1_robot
chr10	70286603	70286604	MACS_peak_1739	4.00	lobChIP_FOXA1_robot
chr10	70362360	70362361	MACS_peak_1740	11.00	lobChIP_FOXA1_robot
chr10	70636446	70636447	MACS_peak_1741	21.00	lobChIP_FOXA1_robot
chr10	70638358	70638359	MACS_peak_1742	5.00	lobChIP_FOXA1_robot
chr10	70817281	70817282	MACS_peak_1743	3.00	lobChIP_FOXA1_robot
chr10	71075002	71075003	MACS_peak_1744	4.00	lobChIP_FOXA1_robot
chr10	71152628	71152629	MACS_peak_1745	4.00	lobChIP_FOXA1_robot
chr10	71185795	71185796	MACS_peak_1746	4.00	lobChIP_FOXA1_robot
chr10	71206497	71206498	MACS_peak_1747	9.00	lobChIP_FOXA1_robot
chr10	71455873	71455874	MACS_peak_1748	4.00	lobChIP_FOXA1_robot
chr10	71846024	71846025	MACS_peak_1749	25.00	lobChIP_FOXA1_robot
chr10	71992434	71992435	MACS_peak_1750	3.00	lobChIP_FOXA1_robot
chr10	72086085	72086086	MACS_peak_1751	8.00	lobChIP_FOXA1_robot
chr10	72112596	72112597	MACS_peak_1752	24.00	lobChIP_FOXA1_robot
chr10	72202261	72202262	MACS_peak_1753	9.00	lobChIP_FOXA1_robot
chr10	72561360	72561361	MACS_peak_1754	3.00	lobChIP_FOXA1_robot
chr10	72564386	72564387	MACS_peak_1755	6.00	lobChIP_FOXA1_robot
chr10	72603729	72603730	MACS_peak_1756	7.00	lobChIP_FOXA1_robot
chr10	72609635	72609636	MACS_peak_1757	7.00	lobChIP_FOXA1_robot
chr10	72612097	72612098	MACS_peak_1758	11.00	lobChIP_FOXA1_robot
chr10	73052549	73052550	MACS_peak_1759	4.00	lobChIP_FOXA1_robot
chr10	73094328	73094329	MACS_peak_1760	7.00	lobChIP_FOXA1_robot
chr10	73100015	73100016	MACS_peak_1761	15.00	lobChIP_FOXA1_robot
chr10	73103198	73103199	MACS_peak_1762	9.00	lobChIP_FOXA1_robot
chr10	73107969	73107970	MACS_peak_1763	7.00	lobChIP_FOXA1_robot
chr10	73135872	73135873	MACS_peak_1764	4.00	lobChIP_FOXA1_robot
chr10	73526715	73526716	MACS_peak_1765	4.00	lobChIP_FOXA1_robot
chr10	73640376	73640377	MACS_peak_1766	5.00	lobChIP_FOXA1_robot
chr10	73687919	73687920	MACS_peak_1767	8.00	lobChIP_FOXA1_robot
chr10	73736714	73736715	MACS_peak_1768	8.00	lobChIP_FOXA1_robot
chr10	73790643	73790644	MACS_peak_1769	9.00	lobChIP_FOXA1_robot
chr10	73798355	73798356	MACS_peak_1770	14.00	lobChIP_FOXA1_robot
chr10	73857807	73857808	MACS_peak_1771	29.00	lobChIP_FOXA1_robot
chr10	74036803	74036804	MACS_peak_1772	12.00	lobChIP_FOXA1_robot
chr10	74283757	74283758	MACS_peak_1773	13.00	lobChIP_FOXA1_robot
chr10	74285643	74285644	MACS_peak_1774	5.00	lobChIP_FOXA1_robot
chr10	74398792	74398793	MACS_peak_1775	7.00	lobChIP_FOXA1_robot
chr10	74439760	74439761	MACS_peak_1776	9.00	lobChIP_FOXA1_robot
chr10	74448073	74448074	MACS_peak_1777	4.00	lobChIP_FOXA1_robot
chr10	74500414	74500415	MACS_peak_1778	3.00	lobChIP_FOXA1_robot
chr10	74560405	74560406	MACS_peak_1779	5.00	lobChIP_FOXA1_robot
chr10	74712773	74712774	MACS_peak_1780	6.00	lobChIP_FOXA1_robot
chr10	74879310	74879311	MACS_peak_1781	18.00	lobChIP_FOXA1_robot
chr10	74892256	74892257	MACS_peak_1782	22.00	lobChIP_FOXA1_robot
chr10	75178492	75178493	MACS_peak_1783	8.00	lobChIP_FOXA1_robot
chr10	75346571	75346572	MACS_peak_1784	8.00	lobChIP_FOXA1_robot
chr10	75351836	75351837	MACS_peak_1785	12.00	lobChIP_FOXA1_robot
chr10	75490410	75490411	MACS_peak_1786	5.00	lobChIP_FOXA1_robot
chr10	75973653	75973654	MACS_peak_1787	3.00	lobChIP_FOXA1_robot
chr10	76289765	76289766	MACS_peak_1788	5.00	lobChIP_FOXA1_robot
chr10	76308605	76308606	MACS_peak_1789	11.00	lobChIP_FOXA1_robot
chr10	76347082	76347083	MACS_peak_1790	4.00	lobChIP_FOXA1_robot
chr10	76443738	76443739	MACS_peak_1791	10.00	lobChIP_FOXA1_robot
chr10	76516205	76516206	MACS_peak_1792	6.00	lobChIP_FOXA1_robot
chr10	76582112	76582113	MACS_peak_1793	9.00	lobChIP_FOXA1_robot
chr10	76583661	76583662	MACS_peak_1794	5.00	lobChIP_FOXA1_robot
chr10	76635868	76635869	MACS_peak_1795	6.00	lobChIP_FOXA1_robot
chr10	76728297	76728298	MACS_peak_1796	5.00	lobChIP_FOXA1_robot
chr10	76781735	76781736	MACS_peak_1797	5.00	lobChIP_FOXA1_robot
chr10	76928190	76928191	MACS_peak_1798	28.00	lobChIP_FOXA1_robot
chr10	76946588	76946589	MACS_peak_1799	5.00	lobChIP_FOXA1_robot
chr10	76949495	76949496	MACS_peak_1800	9.00	lobChIP_FOXA1_robot
chr10	76951987	76951988	MACS_peak_1801	7.00	lobChIP_FOXA1_robot
chr10	77162344	77162345	MACS_peak_1802	4.00	lobChIP_FOXA1_robot
chr10	77165049	77165050	MACS_peak_1803	17.00	lobChIP_FOXA1_robot
chr10	77168608	77168609	MACS_peak_1804	3.00	lobChIP_FOXA1_robot
chr10	77190295	77190296	MACS_peak_1805	20.00	lobChIP_FOXA1_robot
chr10	77325334	77325335	MACS_peak_1806	6.00	lobChIP_FOXA1_robot
chr10	77360259	77360260	MACS_peak_1807	6.00	lobChIP_FOXA1_robot
chr10	77493246	77493247	MACS_peak_1808	48.00	lobChIP_FOXA1_robot
chr10	77503571	77503572	MACS_peak_1809	4.00	lobChIP_FOXA1_robot
chr10	77506308	77506309	MACS_peak_1810	8.00	lobChIP_FOXA1_robot
chr10	77523411	77523412	MACS_peak_1811	5.00	lobChIP_FOXA1_robot
chr10	77533446	77533447	MACS_peak_1812	13.00	lobChIP_FOXA1_robot
chr10	77536810	77536811	MACS_peak_1813	24.00	lobChIP_FOXA1_robot
chr10	77548642	77548643	MACS_peak_1814	7.00	lobChIP_FOXA1_robot
chr10	77710211	77710212	MACS_peak_1815	4.00	lobChIP_FOXA1_robot
chr10	77710952	77710953	MACS_peak_1816	4.00	lobChIP_FOXA1_robot
chr10	77784721	77784722	MACS_peak_1817	17.00	lobChIP_FOXA1_robot
chr10	77786096	77786097	MACS_peak_1818	5.00	lobChIP_FOXA1_robot
chr10	77793442	77793443	MACS_peak_1819	7.00	lobChIP_FOXA1_robot
chr10	77811269	77811270	MACS_peak_1820	28.00	lobChIP_FOXA1_robot
chr10	77874475	77874476	MACS_peak_1821	4.00	lobChIP_FOXA1_robot
chr10	77910119	77910120	MACS_peak_1822	5.00	lobChIP_FOXA1_robot
chr10	77910890	77910891	MACS_peak_1823	10.00	lobChIP_FOXA1_robot
chr10	78057646	78057647	MACS_peak_1824	4.00	lobChIP_FOXA1_robot
chr10	78062777	78062778	MACS_peak_1825	3.00	lobChIP_FOXA1_robot
chr10	78871785	78871786	MACS_peak_1826	4.00	lobChIP_FOXA1_robot
chr10	79093885	79093886	MACS_peak_1827	10.00	lobChIP_FOXA1_robot
chr10	79471430	79471431	MACS_peak_1828	4.00	lobChIP_FOXA1_robot
chr10	79609024	79609025	MACS_peak_1829	31.00	lobChIP_FOXA1_robot
chr10	79647635	79647636	MACS_peak_1830	4.00	lobChIP_FOXA1_robot
chr10	79671601	79671602	MACS_peak_1831	11.00	lobChIP_FOXA1_robot
chr10	80218951	80218952	MACS_peak_1832	78.00	lobChIP_FOXA1_robot
chr10	80280811	80280812	MACS_peak_1833	5.00	lobChIP_FOXA1_robot
chr10	80283481	80283482	MACS_peak_1834	4.00	lobChIP_FOXA1_robot
chr10	80288730	80288731	MACS_peak_1835	28.00	lobChIP_FOXA1_robot
chr10	80315160	80315161	MACS_peak_1836	3.00	lobChIP_FOXA1_robot
chr10	80349574	80349575	MACS_peak_1837	10.00	lobChIP_FOXA1_robot
chr10	80502697	80502698	MACS_peak_1838	7.00	lobChIP_FOXA1_robot
chr10	80613789	80613790	MACS_peak_1839	6.00	lobChIP_FOXA1_robot
chr10	80654777	80654778	MACS_peak_1840	5.00	lobChIP_FOXA1_robot
chr10	80733403	80733404	MACS_peak_1841	5.00	lobChIP_FOXA1_robot
chr10	80734101	80734102	MACS_peak_1842	4.00	lobChIP_FOXA1_robot
chr10	80819190	80819191	MACS_peak_1843	17.00	lobChIP_FOXA1_robot
chr10	80853872	80853873	MACS_peak_1844	6.00	lobChIP_FOXA1_robot
chr10	80887152	80887153	MACS_peak_1845	4.00	lobChIP_FOXA1_robot
chr10	80917493	80917494	MACS_peak_1846	5.00	lobChIP_FOXA1_robot
chr10	81140245	81140246	MACS_peak_1847	6.00	lobChIP_FOXA1_robot
chr10	81842261	81842262	MACS_peak_1848	4.00	lobChIP_FOXA1_robot
chr10	81870852	81870853	MACS_peak_1849	4.00	lobChIP_FOXA1_robot
chr10	81966480	81966481	MACS_peak_1850	4.00	lobChIP_FOXA1_robot
chr10	81982627	81982628	MACS_peak_1851	6.00	lobChIP_FOXA1_robot
chr10	82032817	82032818	MACS_peak_1852	5.00	lobChIP_FOXA1_robot
chr10	82050809	82050810	MACS_peak_1853	9.00	lobChIP_FOXA1_robot
chr10	82079499	82079500	MACS_peak_1854	3.00	lobChIP_FOXA1_robot
chr10	82174133	82174134	MACS_peak_1855	5.00	lobChIP_FOXA1_robot
chr10	82251560	82251561	MACS_peak_1856	7.00	lobChIP_FOXA1_robot
chr10	84351386	84351387	MACS_peak_1857	5.00	lobChIP_FOXA1_robot
chr10	84573632	84573633	MACS_peak_1858	3.00	lobChIP_FOXA1_robot
chr10	86148118	86148119	MACS_peak_1859	6.00	lobChIP_FOXA1_robot
chr10	86183201	86183202	MACS_peak_1860	5.00	lobChIP_FOXA1_robot
chr10	86257870	86257871	MACS_peak_1861	8.00	lobChIP_FOXA1_robot
chr10	86636072	86636073	MACS_peak_1862	10.00	lobChIP_FOXA1_robot
chr10	88170480	88170481	MACS_peak_1863	8.00	lobChIP_FOXA1_robot
chr10	88179111	88179112	MACS_peak_1864	3.00	lobChIP_FOXA1_robot
chr10	88198396	88198397	MACS_peak_1865	4.00	lobChIP_FOXA1_robot
chr10	88200976	88200977	MACS_peak_1866	12.00	lobChIP_FOXA1_robot
chr10	88534974	88534975	MACS_peak_1867	14.00	lobChIP_FOXA1_robot
chr10	88621381	88621382	MACS_peak_1868	6.00	lobChIP_FOXA1_robot
chr10	88622638	88622639	MACS_peak_1869	23.00	lobChIP_FOXA1_robot
chr10	88631727	88631728	MACS_peak_1870	4.00	lobChIP_FOXA1_robot
chr10	88852696	88852697	MACS_peak_1871	8.00	lobChIP_FOXA1_robot
chr10	88931626	88931627	MACS_peak_1872	8.00	lobChIP_FOXA1_robot
chr10	89260619	89260620	MACS_peak_1873	26.00	lobChIP_FOXA1_robot
chr10	89378807	89378808	MACS_peak_1874	22.00	lobChIP_FOXA1_robot
chr10	89602209	89602210	MACS_peak_1875	4.00	lobChIP_FOXA1_robot
chr10	90945616	90945617	MACS_peak_1876	19.00	lobChIP_FOXA1_robot
chr10	91109729	91109730	MACS_peak_1877	3.00	lobChIP_FOXA1_robot
chr10	91133290	91133291	MACS_peak_1878	16.00	lobChIP_FOXA1_robot
chr10	91134201	91134202	MACS_peak_1879	4.00	lobChIP_FOXA1_robot
chr10	91235533	91235534	MACS_peak_1880	4.00	lobChIP_FOXA1_robot
chr10	91403623	91403624	MACS_peak_1881	6.00	lobChIP_FOXA1_robot
chr10	91405758	91405759	MACS_peak_1882	5.00	lobChIP_FOXA1_robot
chr10	91574290	91574291	MACS_peak_1883	12.00	lobChIP_FOXA1_robot
chr10	92177016	92177017	MACS_peak_1884	3.00	lobChIP_FOXA1_robot
chr10	92402018	92402019	MACS_peak_1885	5.00	lobChIP_FOXA1_robot
chr10	92671809	92671810	MACS_peak_1886	3.00	lobChIP_FOXA1_robot
chr10	92681117	92681118	MACS_peak_1887	14.00	lobChIP_FOXA1_robot
chr10	92691376	92691377	MACS_peak_1888	10.00	lobChIP_FOXA1_robot
chr10	92731763	92731764	MACS_peak_1889	5.00	lobChIP_FOXA1_robot
chr10	92979917	92979918	MACS_peak_1890	16.00	lobChIP_FOXA1_robot
chr10	93253109	93253110	MACS_peak_1891	9.00	lobChIP_FOXA1_robot
chr10	93316692	93316693	MACS_peak_1892	22.00	lobChIP_FOXA1_robot
chr10	93412470	93412471	MACS_peak_1893	4.00	lobChIP_FOXA1_robot
chr10	93563426	93563427	MACS_peak_1894	7.00	lobChIP_FOXA1_robot
chr10	93564053	93564054	MACS_peak_1895	6.00	lobChIP_FOXA1_robot
chr10	93651223	93651224	MACS_peak_1896	4.00	lobChIP_FOXA1_robot
chr10	93842862	93842863	MACS_peak_1897	8.00	lobChIP_FOXA1_robot
chr10	93871507	93871508	MACS_peak_1898	11.00	lobChIP_FOXA1_robot
chr10	93874106	93874107	MACS_peak_1899	8.00	lobChIP_FOXA1_robot
chr10	93882010	93882011	MACS_peak_1900	9.00	lobChIP_FOXA1_robot
chr10	93882972	93882973	MACS_peak_1901	13.00	lobChIP_FOXA1_robot
chr10	93884649	93884650	MACS_peak_1902	3.00	lobChIP_FOXA1_robot
chr10	94050213	94050214	MACS_peak_1903	6.00	lobChIP_FOXA1_robot
chr10	94340847	94340848	MACS_peak_1904	5.00	lobChIP_FOXA1_robot
chr10	94351442	94351443	MACS_peak_1905	4.00	lobChIP_FOXA1_robot
chr10	94417007	94417008	MACS_peak_1906	4.00	lobChIP_FOXA1_robot
chr10	94453162	94453163	MACS_peak_1907	6.00	lobChIP_FOXA1_robot
chr10	94457575	94457576	MACS_peak_1908	24.00	lobChIP_FOXA1_robot
chr10	94472038	94472039	MACS_peak_1909	8.00	lobChIP_FOXA1_robot
chr10	94516185	94516186	MACS_peak_1910	22.00	lobChIP_FOXA1_robot
chr10	94632925	94632926	MACS_peak_1911	3.00	lobChIP_FOXA1_robot
chr10	94652364	94652365	MACS_peak_1912	5.00	lobChIP_FOXA1_robot
chr10	94725181	94725182	MACS_peak_1913	6.00	lobChIP_FOXA1_robot
chr10	94866549	94866550	MACS_peak_1914	25.00	lobChIP_FOXA1_robot
chr10	95014169	95014170	MACS_peak_1915	9.00	lobChIP_FOXA1_robot
chr10	95060894	95060895	MACS_peak_1916	4.00	lobChIP_FOXA1_robot
chr10	95205542	95205543	MACS_peak_1917	26.00	lobChIP_FOXA1_robot
chr10	95338983	95338984	MACS_peak_1918	5.00	lobChIP_FOXA1_robot
chr10	95361513	95361514	MACS_peak_1919	4.00	lobChIP_FOXA1_robot
chr10	95430975	95430976	MACS_peak_1920	10.00	lobChIP_FOXA1_robot
chr10	95431927	95431928	MACS_peak_1921	10.00	lobChIP_FOXA1_robot
chr10	95883408	95883409	MACS_peak_1922	5.00	lobChIP_FOXA1_robot
chr10	96075764	96075765	MACS_peak_1923	6.00	lobChIP_FOXA1_robot
chr10	96222099	96222100	MACS_peak_1924	13.00	lobChIP_FOXA1_robot
chr10	96440441	96440442	MACS_peak_1925	5.00	lobChIP_FOXA1_robot
chr10	96662551	96662552	MACS_peak_1926	10.00	lobChIP_FOXA1_robot
chr10	96676313	96676314	MACS_peak_1927	4.00	lobChIP_FOXA1_robot
chr10	96795315	96795316	MACS_peak_1928	3.00	lobChIP_FOXA1_robot
chr10	96897995	96897996	MACS_peak_1929	6.00	lobChIP_FOXA1_robot
chr10	96938755	96938756	MACS_peak_1930	10.00	lobChIP_FOXA1_robot
chr10	97038001	97038002	MACS_peak_1931	32.00	lobChIP_FOXA1_robot
chr10	97039304	97039305	MACS_peak_1932	5.00	lobChIP_FOXA1_robot
chr10	97096003	97096004	MACS_peak_1933	3.00	lobChIP_FOXA1_robot
chr10	97107024	97107025	MACS_peak_1934	5.00	lobChIP_FOXA1_robot
chr10	97110195	97110196	MACS_peak_1935	8.00	lobChIP_FOXA1_robot
chr10	97148468	97148469	MACS_peak_1936	8.00	lobChIP_FOXA1_robot
chr10	97212105	97212106	MACS_peak_1937	5.00	lobChIP_FOXA1_robot
chr10	97217297	97217298	MACS_peak_1938	8.00	lobChIP_FOXA1_robot
chr10	97217870	97217871	MACS_peak_1939	12.00	lobChIP_FOXA1_robot
chr10	97227179	97227180	MACS_peak_1940	9.00	lobChIP_FOXA1_robot
chr10	97234518	97234519	MACS_peak_1941	4.00	lobChIP_FOXA1_robot
chr10	97235802	97235803	MACS_peak_1942	19.00	lobChIP_FOXA1_robot
chr10	97240776	97240777	MACS_peak_1943	5.00	lobChIP_FOXA1_robot
chr10	97241513	97241514	MACS_peak_1944	6.00	lobChIP_FOXA1_robot
chr10	97259671	97259672	MACS_peak_1945	4.00	lobChIP_FOXA1_robot
chr10	97261545	97261546	MACS_peak_1946	9.00	lobChIP_FOXA1_robot
chr10	97273150	97273151	MACS_peak_1947	5.00	lobChIP_FOXA1_robot
chr10	97299532	97299533	MACS_peak_1948	7.00	lobChIP_FOXA1_robot
chr10	97302370	97302371	MACS_peak_1949	8.00	lobChIP_FOXA1_robot
chr10	97303805	97303806	MACS_peak_1950	4.00	lobChIP_FOXA1_robot
chr10	97312552	97312553	MACS_peak_1951	3.00	lobChIP_FOXA1_robot
chr10	97313136	97313137	MACS_peak_1952	5.00	lobChIP_FOXA1_robot
chr10	97395123	97395124	MACS_peak_1953	13.00	lobChIP_FOXA1_robot
chr10	97400899	97400900	MACS_peak_1954	7.00	lobChIP_FOXA1_robot
chr10	97578612	97578613	MACS_peak_1955	9.00	lobChIP_FOXA1_robot
chr10	97596712	97596713	MACS_peak_1956	4.00	lobChIP_FOXA1_robot
chr10	97756445	97756446	MACS_peak_1957	3.00	lobChIP_FOXA1_robot
chr10	97853151	97853152	MACS_peak_1958	4.00	lobChIP_FOXA1_robot
chr10	97853904	97853905	MACS_peak_1959	5.00	lobChIP_FOXA1_robot
chr10	97884943	97884944	MACS_peak_1960	5.00	lobChIP_FOXA1_robot
chr10	98356620	98356621	MACS_peak_1961	21.00	lobChIP_FOXA1_robot
chr10	98430838	98430839	MACS_peak_1962	25.00	lobChIP_FOXA1_robot
chr10	98433638	98433639	MACS_peak_1963	9.00	lobChIP_FOXA1_robot
chr10	98437324	98437325	MACS_peak_1964	10.00	lobChIP_FOXA1_robot
chr10	98558487	98558488	MACS_peak_1965	10.00	lobChIP_FOXA1_robot
chr10	98600853	98600854	MACS_peak_1966	6.00	lobChIP_FOXA1_robot
chr10	98602862	98602863	MACS_peak_1967	9.00	lobChIP_FOXA1_robot
chr10	98624095	98624096	MACS_peak_1968	58.00	lobChIP_FOXA1_robot
chr10	98731342	98731343	MACS_peak_1969	6.00	lobChIP_FOXA1_robot
chr10	98786589	98786590	MACS_peak_1970	3.00	lobChIP_FOXA1_robot
chr10	98900310	98900311	MACS_peak_1971	23.00	lobChIP_FOXA1_robot
chr10	99068272	99068273	MACS_peak_1972	3.00	lobChIP_FOXA1_robot
chr10	99087788	99087789	MACS_peak_1973	26.00	lobChIP_FOXA1_robot
chr10	99088718	99088719	MACS_peak_1974	16.00	lobChIP_FOXA1_robot
chr10	99097793	99097794	MACS_peak_1975	5.00	lobChIP_FOXA1_robot
chr10	99161080	99161081	MACS_peak_1976	4.00	lobChIP_FOXA1_robot
chr10	99194036	99194037	MACS_peak_1977	3.00	lobChIP_FOXA1_robot
chr10	99424202	99424203	MACS_peak_1978	4.00	lobChIP_FOXA1_robot
chr10	99444926	99444927	MACS_peak_1979	8.00	lobChIP_FOXA1_robot
chr10	99621945	99621946	MACS_peak_1980	13.00	lobChIP_FOXA1_robot
chr10	100030292	100030293	MACS_peak_1981	11.00	lobChIP_FOXA1_robot
chr10	100444265	100444266	MACS_peak_1982	4.00	lobChIP_FOXA1_robot
chr10	100631885	100631886	MACS_peak_1983	4.00	lobChIP_FOXA1_robot
chr10	101149714	101149715	MACS_peak_1984	6.00	lobChIP_FOXA1_robot
chr10	101155812	101155813	MACS_peak_1985	16.00	lobChIP_FOXA1_robot
chr10	101181223	101181224	MACS_peak_1986	16.00	lobChIP_FOXA1_robot
chr10	101407430	101407431	MACS_peak_1987	6.00	lobChIP_FOXA1_robot
chr10	101514591	101514592	MACS_peak_1988	11.00	lobChIP_FOXA1_robot
chr10	101541939	101541940	MACS_peak_1989	5.00	lobChIP_FOXA1_robot
chr10	101574137	101574138	MACS_peak_1990	3.00	lobChIP_FOXA1_robot
chr10	101609427	101609428	MACS_peak_1991	9.00	lobChIP_FOXA1_robot
chr10	101687670	101687671	MACS_peak_1992	10.00	lobChIP_FOXA1_robot
chr10	101768657	101768658	MACS_peak_1993	9.00	lobChIP_FOXA1_robot
chr10	101780122	101780123	MACS_peak_1994	14.00	lobChIP_FOXA1_robot
chr10	101832434	101832435	MACS_peak_1995	3.00	lobChIP_FOXA1_robot
chr10	101841670	101841671	MACS_peak_1996	6.00	lobChIP_FOXA1_robot
chr10	101845590	101845591	MACS_peak_1997	32.00	lobChIP_FOXA1_robot
chr10	101861504	101861505	MACS_peak_1998	22.00	lobChIP_FOXA1_robot
chr10	101877873	101877874	MACS_peak_1999	4.00	lobChIP_FOXA1_robot
chr10	101924024	101924025	MACS_peak_2000	6.00	lobChIP_FOXA1_robot
chr10	101927789	101927790	MACS_peak_2001	5.00	lobChIP_FOXA1_robot
chr10	101943399	101943400	MACS_peak_2002	9.00	lobChIP_FOXA1_robot
chr10	102101852	102101853	MACS_peak_2003	9.00	lobChIP_FOXA1_robot
chr10	102152300	102152301	MACS_peak_2004	8.00	lobChIP_FOXA1_robot
chr10	102155613	102155614	MACS_peak_2005	8.00	lobChIP_FOXA1_robot
chr10	102635673	102635674	MACS_peak_2006	22.00	lobChIP_FOXA1_robot
chr10	102641202	102641203	MACS_peak_2007	18.00	lobChIP_FOXA1_robot
chr10	102656958	102656959	MACS_peak_2008	4.00	lobChIP_FOXA1_robot
chr10	102667165	102667166	MACS_peak_2009	4.00	lobChIP_FOXA1_robot
chr10	102731040	102731041	MACS_peak_2010	22.00	lobChIP_FOXA1_robot
chr10	102872977	102872978	MACS_peak_2011	8.00	lobChIP_FOXA1_robot
chr10	103124731	103124732	MACS_peak_2012	5.00	lobChIP_FOXA1_robot
chr10	103227103	103227104	MACS_peak_2013	5.00	lobChIP_FOXA1_robot
chr10	103755644	103755645	MACS_peak_2014	4.00	lobChIP_FOXA1_robot
chr10	103802099	103802100	MACS_peak_2015	4.00	lobChIP_FOXA1_robot
chr10	104357269	104357270	MACS_peak_2016	5.00	lobChIP_FOXA1_robot
chr10	104359552	104359553	MACS_peak_2017	8.00	lobChIP_FOXA1_robot
chr10	104470500	104470501	MACS_peak_2018	6.00	lobChIP_FOXA1_robot
chr10	104529242	104529243	MACS_peak_2019	12.00	lobChIP_FOXA1_robot
chr10	104574833	104574834	MACS_peak_2020	5.00	lobChIP_FOXA1_robot
chr10	104610043	104610044	MACS_peak_2021	7.00	lobChIP_FOXA1_robot
chr10	104615483	104615484	MACS_peak_2022	4.00	lobChIP_FOXA1_robot
chr10	104636594	104636595	MACS_peak_2023	5.00	lobChIP_FOXA1_robot
chr10	104723192	104723193	MACS_peak_2024	4.00	lobChIP_FOXA1_robot
chr10	104748899	104748900	MACS_peak_2025	15.00	lobChIP_FOXA1_robot
chr10	104785628	104785629	MACS_peak_2026	8.00	lobChIP_FOXA1_robot
chr10	104813683	104813684	MACS_peak_2027	7.00	lobChIP_FOXA1_robot
chr10	104819181	104819182	MACS_peak_2028	5.00	lobChIP_FOXA1_robot
chr10	104881292	104881293	MACS_peak_2029	8.00	lobChIP_FOXA1_robot
chr10	104914864	104914865	MACS_peak_2030	7.00	lobChIP_FOXA1_robot
chr10	104934451	104934452	MACS_peak_2031	4.00	lobChIP_FOXA1_robot
chr10	104991135	104991136	MACS_peak_2032	11.00	lobChIP_FOXA1_robot
chr10	105264319	105264320	MACS_peak_2033	6.00	lobChIP_FOXA1_robot
chr10	105336813	105336814	MACS_peak_2034	11.00	lobChIP_FOXA1_robot
chr10	105358729	105358730	MACS_peak_2035	6.00	lobChIP_FOXA1_robot
chr10	105418441	105418442	MACS_peak_2036	4.00	lobChIP_FOXA1_robot
chr10	105554145	105554146	MACS_peak_2037	54.00	lobChIP_FOXA1_robot
chr10	105578764	105578765	MACS_peak_2038	4.00	lobChIP_FOXA1_robot
chr10	105614231	105614232	MACS_peak_2039	4.00	lobChIP_FOXA1_robot
chr10	106013112	106013113	MACS_peak_2040	21.00	lobChIP_FOXA1_robot
chr10	106015588	106015589	MACS_peak_2041	3.00	lobChIP_FOXA1_robot
chr10	106016206	106016207	MACS_peak_2042	10.00	lobChIP_FOXA1_robot
chr10	106024635	106024636	MACS_peak_2043	7.00	lobChIP_FOXA1_robot
chr10	106037786	106037787	MACS_peak_2044	5.00	lobChIP_FOXA1_robot
chr10	106048364	106048365	MACS_peak_2045	31.00	lobChIP_FOXA1_robot
chr10	108212883	108212884	MACS_peak_2046	3.00	lobChIP_FOXA1_robot
chr10	109595235	109595236	MACS_peak_2047	3.00	lobChIP_FOXA1_robot
chr10	110076633	110076634	MACS_peak_2048	3.00	lobChIP_FOXA1_robot
chr10	110481920	110481921	MACS_peak_2049	5.00	lobChIP_FOXA1_robot
chr10	111654812	111654813	MACS_peak_2050	5.00	lobChIP_FOXA1_robot
chr10	111725395	111725396	MACS_peak_2051	13.00	lobChIP_FOXA1_robot
chr10	111731649	111731650	MACS_peak_2052	10.00	lobChIP_FOXA1_robot
chr10	111734679	111734680	MACS_peak_2053	6.00	lobChIP_FOXA1_robot
chr10	111736505	111736506	MACS_peak_2054	5.00	lobChIP_FOXA1_robot
chr10	111737467	111737468	MACS_peak_2055	5.00	lobChIP_FOXA1_robot
chr10	111836921	111836922	MACS_peak_2056	7.00	lobChIP_FOXA1_robot
chr10	112282631	112282632	MACS_peak_2057	4.00	lobChIP_FOXA1_robot
chr10	112329718	112329719	MACS_peak_2058	13.00	lobChIP_FOXA1_robot
chr10	112534837	112534838	MACS_peak_2059	6.00	lobChIP_FOXA1_robot
chr10	112605633	112605634	MACS_peak_2060	7.00	lobChIP_FOXA1_robot
chr10	112636723	112636724	MACS_peak_2061	5.00	lobChIP_FOXA1_robot
chr10	112684080	112684081	MACS_peak_2062	4.00	lobChIP_FOXA1_robot
chr10	113235660	113235661	MACS_peak_2063	5.00	lobChIP_FOXA1_robot
chr10	113339684	113339685	MACS_peak_2064	4.00	lobChIP_FOXA1_robot
chr10	113610659	113610660	MACS_peak_2065	3.00	lobChIP_FOXA1_robot
chr10	113937330	113937331	MACS_peak_2066	4.00	lobChIP_FOXA1_robot
chr10	113985647	113985648	MACS_peak_2067	8.00	lobChIP_FOXA1_robot
chr10	113987484	113987485	MACS_peak_2068	10.00	lobChIP_FOXA1_robot
chr10	113991085	113991086	MACS_peak_2069	6.00	lobChIP_FOXA1_robot
chr10	114018302	114018303	MACS_peak_2070	7.00	lobChIP_FOXA1_robot
chr10	114021433	114021434	MACS_peak_2071	14.00	lobChIP_FOXA1_robot
chr10	114035699	114035700	MACS_peak_2072	7.00	lobChIP_FOXA1_robot
chr10	114040377	114040378	MACS_peak_2073	3.00	lobChIP_FOXA1_robot
chr10	114046954	114046955	MACS_peak_2074	11.00	lobChIP_FOXA1_robot
chr10	114062383	114062384	MACS_peak_2075	3.00	lobChIP_FOXA1_robot
chr10	114064453	114064454	MACS_peak_2076	18.00	lobChIP_FOXA1_robot
chr10	114125107	114125108	MACS_peak_2077	6.00	lobChIP_FOXA1_robot
chr10	114133760	114133761	MACS_peak_2078	12.00	lobChIP_FOXA1_robot
chr10	114149309	114149310	MACS_peak_2079	7.00	lobChIP_FOXA1_robot
chr10	114151380	114151381	MACS_peak_2080	8.00	lobChIP_FOXA1_robot
chr10	114154516	114154517	MACS_peak_2081	26.00	lobChIP_FOXA1_robot
chr10	114250476	114250477	MACS_peak_2082	15.00	lobChIP_FOXA1_robot
chr10	114322046	114322047	MACS_peak_2083	6.00	lobChIP_FOXA1_robot
chr10	114323326	114323327	MACS_peak_2084	32.00	lobChIP_FOXA1_robot
chr10	114324319	114324320	MACS_peak_2085	13.00	lobChIP_FOXA1_robot
chr10	114334776	114334777	MACS_peak_2086	6.00	lobChIP_FOXA1_robot
chr10	114391641	114391642	MACS_peak_2087	13.00	lobChIP_FOXA1_robot
chr10	114398166	114398167	MACS_peak_2088	4.00	lobChIP_FOXA1_robot
chr10	114403510	114403511	MACS_peak_2089	25.00	lobChIP_FOXA1_robot
chr10	114418959	114418960	MACS_peak_2090	26.00	lobChIP_FOXA1_robot
chr10	114438613	114438614	MACS_peak_2091	9.00	lobChIP_FOXA1_robot
chr10	114455320	114455321	MACS_peak_2092	11.00	lobChIP_FOXA1_robot
chr10	114476956	114476957	MACS_peak_2093	15.00	lobChIP_FOXA1_robot
chr10	114488311	114488312	MACS_peak_2094	14.00	lobChIP_FOXA1_robot
chr10	114499574	114499575	MACS_peak_2095	32.00	lobChIP_FOXA1_robot
chr10	114502109	114502110	MACS_peak_2096	11.00	lobChIP_FOXA1_robot
chr10	114510610	114510611	MACS_peak_2097	5.00	lobChIP_FOXA1_robot
chr10	114571798	114571799	MACS_peak_2098	47.00	lobChIP_FOXA1_robot
chr10	114575752	114575753	MACS_peak_2099	4.00	lobChIP_FOXA1_robot
chr10	114602592	114602593	MACS_peak_2100	3.00	lobChIP_FOXA1_robot
chr10	114658878	114658879	MACS_peak_2101	4.00	lobChIP_FOXA1_robot
chr10	114709990	114709991	MACS_peak_2102	4.00	lobChIP_FOXA1_robot
chr10	114729591	114729592	MACS_peak_2103	5.00	lobChIP_FOXA1_robot
chr10	114779639	114779640	MACS_peak_2104	5.00	lobChIP_FOXA1_robot
chr10	114795653	114795654	MACS_peak_2105	10.00	lobChIP_FOXA1_robot
chr10	114820098	114820099	MACS_peak_2106	4.00	lobChIP_FOXA1_robot
chr10	114833797	114833798	MACS_peak_2107	6.00	lobChIP_FOXA1_robot
chr10	114846280	114846281	MACS_peak_2108	7.00	lobChIP_FOXA1_robot
chr10	114847315	114847316	MACS_peak_2109	10.00	lobChIP_FOXA1_robot
chr10	114853582	114853583	MACS_peak_2110	22.00	lobChIP_FOXA1_robot
chr10	115509561	115509562	MACS_peak_2111	4.00	lobChIP_FOXA1_robot
chr10	116249485	116249486	MACS_peak_2112	8.00	lobChIP_FOXA1_robot
chr10	116383700	116383701	MACS_peak_2113	8.00	lobChIP_FOXA1_robot
chr10	116444650	116444651	MACS_peak_2114	6.00	lobChIP_FOXA1_robot
chr10	116582787	116582788	MACS_peak_2115	4.00	lobChIP_FOXA1_robot
chr10	116789445	116789446	MACS_peak_2116	4.00	lobChIP_FOXA1_robot
chr10	117022047	117022048	MACS_peak_2117	4.00	lobChIP_FOXA1_robot
chr10	117204996	117204997	MACS_peak_2118	9.00	lobChIP_FOXA1_robot
chr10	117326561	117326562	MACS_peak_2119	13.00	lobChIP_FOXA1_robot
chr10	117816544	117816545	MACS_peak_2120	5.00	lobChIP_FOXA1_robot
chr10	118598574	118598575	MACS_peak_2121	6.00	lobChIP_FOXA1_robot
chr10	118707445	118707446	MACS_peak_2122	8.00	lobChIP_FOXA1_robot
chr10	118763003	118763004	MACS_peak_2123	4.00	lobChIP_FOXA1_robot
chr10	119120941	119120942	MACS_peak_2124	25.00	lobChIP_FOXA1_robot
chr10	119125319	119125320	MACS_peak_2125	5.00	lobChIP_FOXA1_robot
chr10	119164571	119164572	MACS_peak_2126	5.00	lobChIP_FOXA1_robot
chr10	119967356	119967357	MACS_peak_2127	5.00	lobChIP_FOXA1_robot
chr10	120070534	120070535	MACS_peak_2128	4.00	lobChIP_FOXA1_robot
chr10	120873497	120873498	MACS_peak_2129	16.00	lobChIP_FOXA1_robot
chr10	120880126	120880127	MACS_peak_2130	7.00	lobChIP_FOXA1_robot
chr10	120937355	120937356	MACS_peak_2131	9.00	lobChIP_FOXA1_robot
chr10	120957678	120957679	MACS_peak_2132	4.00	lobChIP_FOXA1_robot
chr10	120966145	120966146	MACS_peak_2133	4.00	lobChIP_FOXA1_robot
chr10	121003189	121003190	MACS_peak_2134	4.00	lobChIP_FOXA1_robot
chr10	121013404	121013405	MACS_peak_2135	6.00	lobChIP_FOXA1_robot
chr10	121076776	121076777	MACS_peak_2136	5.00	lobChIP_FOXA1_robot
chr10	121169622	121169623	MACS_peak_2137	9.00	lobChIP_FOXA1_robot
chr10	121414229	121414230	MACS_peak_2138	4.00	lobChIP_FOXA1_robot
chr10	121503242	121503243	MACS_peak_2139	4.00	lobChIP_FOXA1_robot
chr10	121521815	121521816	MACS_peak_2140	5.00	lobChIP_FOXA1_robot
chr10	121544359	121544360	MACS_peak_2141	5.00	lobChIP_FOXA1_robot
chr10	121655894	121655895	MACS_peak_2142	10.00	lobChIP_FOXA1_robot
chr10	121926425	121926426	MACS_peak_2143	4.00	lobChIP_FOXA1_robot
chr10	122012819	122012820	MACS_peak_2144	5.00	lobChIP_FOXA1_robot
chr10	122799111	122799112	MACS_peak_2145	5.00	lobChIP_FOXA1_robot
chr10	122823444	122823445	MACS_peak_2146	8.00	lobChIP_FOXA1_robot
chr10	122937611	122937612	MACS_peak_2147	6.00	lobChIP_FOXA1_robot
chr10	123128070	123128071	MACS_peak_2148	4.00	lobChIP_FOXA1_robot
chr10	123283602	123283603	MACS_peak_2149	16.00	lobChIP_FOXA1_robot
chr10	123303498	123303499	MACS_peak_2150	3.00	lobChIP_FOXA1_robot
chr10	123313123	123313124	MACS_peak_2151	5.00	lobChIP_FOXA1_robot
chr10	123365254	123365255	MACS_peak_2152	4.00	lobChIP_FOXA1_robot
chr10	123532783	123532784	MACS_peak_2153	4.00	lobChIP_FOXA1_robot
chr10	123534091	123534092	MACS_peak_2154	6.00	lobChIP_FOXA1_robot
chr10	123603053	123603054	MACS_peak_2155	11.00	lobChIP_FOXA1_robot
chr10	123614595	123614596	MACS_peak_2156	10.00	lobChIP_FOXA1_robot
chr10	123690254	123690255	MACS_peak_2157	4.00	lobChIP_FOXA1_robot
chr10	123774446	123774447	MACS_peak_2158	3.00	lobChIP_FOXA1_robot
chr10	123804382	123804383	MACS_peak_2159	6.00	lobChIP_FOXA1_robot
chr10	123814328	123814329	MACS_peak_2160	4.00	lobChIP_FOXA1_robot
chr10	123822920	123822921	MACS_peak_2161	22.00	lobChIP_FOXA1_robot
chr10	124060715	124060716	MACS_peak_2162	5.00	lobChIP_FOXA1_robot
chr10	124063414	124063415	MACS_peak_2163	4.00	lobChIP_FOXA1_robot
chr10	124065393	124065394	MACS_peak_2164	4.00	lobChIP_FOXA1_robot
chr10	124070354	124070355	MACS_peak_2165	7.00	lobChIP_FOXA1_robot
chr10	124075865	124075866	MACS_peak_2166	3.00	lobChIP_FOXA1_robot
chr10	124091316	124091317	MACS_peak_2167	6.00	lobChIP_FOXA1_robot
chr10	124135713	124135714	MACS_peak_2168	4.00	lobChIP_FOXA1_robot
chr10	124717300	124717301	MACS_peak_2169	10.00	lobChIP_FOXA1_robot
chr10	124779714	124779715	MACS_peak_2170	58.00	lobChIP_FOXA1_robot
chr10	124916137	124916138	MACS_peak_2171	5.00	lobChIP_FOXA1_robot
chr10	124963459	124963460	MACS_peak_2172	5.00	lobChIP_FOXA1_robot
chr10	125764469	125764470	MACS_peak_2173	5.00	lobChIP_FOXA1_robot
chr10	125795676	125795677	MACS_peak_2174	8.00	lobChIP_FOXA1_robot
chr10	125802995	125802996	MACS_peak_2175	6.00	lobChIP_FOXA1_robot
chr10	125808301	125808302	MACS_peak_2176	13.00	lobChIP_FOXA1_robot
chr10	126105462	126105463	MACS_peak_2177	13.00	lobChIP_FOXA1_robot
chr10	126114362	126114363	MACS_peak_2178	7.00	lobChIP_FOXA1_robot
chr10	126159433	126159434	MACS_peak_2179	19.00	lobChIP_FOXA1_robot
chr10	126197656	126197657	MACS_peak_2180	6.00	lobChIP_FOXA1_robot
chr10	126413147	126413148	MACS_peak_2181	4.00	lobChIP_FOXA1_robot
chr10	126433458	126433459	MACS_peak_2182	7.00	lobChIP_FOXA1_robot
chr10	127481307	127481308	MACS_peak_2183	15.00	lobChIP_FOXA1_robot
chr10	127529696	127529697	MACS_peak_2184	4.00	lobChIP_FOXA1_robot
chr10	127578206	127578207	MACS_peak_2185	8.00	lobChIP_FOXA1_robot
chr10	127600755	127600756	MACS_peak_2186	35.00	lobChIP_FOXA1_robot
chr10	128139963	128139964	MACS_peak_2187	4.00	lobChIP_FOXA1_robot
chr10	128547334	128547335	MACS_peak_2188	4.00	lobChIP_FOXA1_robot
chr10	128932092	128932093	MACS_peak_2189	12.00	lobChIP_FOXA1_robot
chr10	129026254	129026255	MACS_peak_2190	4.00	lobChIP_FOXA1_robot
chr10	129077789	129077790	MACS_peak_2191	17.00	lobChIP_FOXA1_robot
chr10	129099541	129099542	MACS_peak_2192	6.00	lobChIP_FOXA1_robot
chr10	129937323	129937324	MACS_peak_2193	5.00	lobChIP_FOXA1_robot
chr10	130958640	130958641	MACS_peak_2194	4.00	lobChIP_FOXA1_robot
chr10	131260672	131260673	MACS_peak_2195	24.00	lobChIP_FOXA1_robot
chr10	131266302	131266303	MACS_peak_2196	7.00	lobChIP_FOXA1_robot
chr10	131754390	131754391	MACS_peak_2197	6.00	lobChIP_FOXA1_robot
chr10	133473779	133473780	MACS_peak_2198	4.00	lobChIP_FOXA1_robot
chr10	133794302	133794303	MACS_peak_2199	4.00	lobChIP_FOXA1_robot
chr10	133811416	133811417	MACS_peak_2200	7.00	lobChIP_FOXA1_robot
chr10	134404804	134404805	MACS_peak_2201	8.00	lobChIP_FOXA1_robot
chr10	134499237	134499238	MACS_peak_2202	4.00	lobChIP_FOXA1_robot
chr10	135160698	135160699	MACS_peak_2203	4.00	lobChIP_FOXA1_robot
chr10	135325608	135325609	MACS_peak_2204	9.00	lobChIP_FOXA1_robot
chr11	655398	655399	MACS_peak_2205	7.00	lobChIP_FOXA1_robot
chr11	844354	844355	MACS_peak_2206	4.00	lobChIP_FOXA1_robot
chr11	1068330	1068331	MACS_peak_2207	4.00	lobChIP_FOXA1_robot
chr11	1241252	1241253	MACS_peak_2208	32.00	lobChIP_FOXA1_robot
chr11	1325854	1325855	MACS_peak_2209	9.00	lobChIP_FOXA1_robot
chr11	1354761	1354762	MACS_peak_2210	4.00	lobChIP_FOXA1_robot
chr11	1682924	1682925	MACS_peak_2211	8.00	lobChIP_FOXA1_robot
chr11	1695373	1695374	MACS_peak_2212	14.00	lobChIP_FOXA1_robot
chr11	1697379	1697380	MACS_peak_2213	10.00	lobChIP_FOXA1_robot
chr11	1703630	1703631	MACS_peak_2214	5.00	lobChIP_FOXA1_robot
chr11	1711262	1711263	MACS_peak_2215	12.00	lobChIP_FOXA1_robot
chr11	1712622	1712623	MACS_peak_2216	10.00	lobChIP_FOXA1_robot
chr11	2004472	2004473	MACS_peak_2217	5.00	lobChIP_FOXA1_robot
chr11	2009479	2009480	MACS_peak_2218	11.00	lobChIP_FOXA1_robot
chr11	2011256	2011257	MACS_peak_2219	7.00	lobChIP_FOXA1_robot
chr11	2232501	2232502	MACS_peak_2220	16.00	lobChIP_FOXA1_robot
chr11	2234907	2234908	MACS_peak_2221	5.00	lobChIP_FOXA1_robot
chr11	2431502	2431503	MACS_peak_2222	8.00	lobChIP_FOXA1_robot
chr11	2584998	2584999	MACS_peak_2223	6.00	lobChIP_FOXA1_robot
chr11	2634299	2634300	MACS_peak_2224	4.00	lobChIP_FOXA1_robot
chr11	2716845	2716846	MACS_peak_2225	6.00	lobChIP_FOXA1_robot
chr11	3754007	3754008	MACS_peak_2226	4.00	lobChIP_FOXA1_robot
chr11	3898830	3898831	MACS_peak_2227	4.00	lobChIP_FOXA1_robot
chr11	3914461	3914462	MACS_peak_2228	3.00	lobChIP_FOXA1_robot
chr11	3926736	3926737	MACS_peak_2229	4.00	lobChIP_FOXA1_robot
chr11	3954689	3954690	MACS_peak_2230	16.00	lobChIP_FOXA1_robot
chr11	3963886	3963887	MACS_peak_2231	14.00	lobChIP_FOXA1_robot
chr11	3981057	3981058	MACS_peak_2232	4.00	lobChIP_FOXA1_robot
chr11	4156273	4156274	MACS_peak_2233	4.00	lobChIP_FOXA1_robot
chr11	5226742	5226743	MACS_peak_2234	3.00	lobChIP_FOXA1_robot
chr11	5642605	5642606	MACS_peak_2235	10.00	lobChIP_FOXA1_robot
chr11	6088947	6088948	MACS_peak_2236	3.00	lobChIP_FOXA1_robot
chr11	6455395	6455396	MACS_peak_2237	8.00	lobChIP_FOXA1_robot
chr11	6462315	6462316	MACS_peak_2238	18.00	lobChIP_FOXA1_robot
chr11	6633922	6633923	MACS_peak_2239	4.00	lobChIP_FOXA1_robot
chr11	7532580	7532581	MACS_peak_2240	3.00	lobChIP_FOXA1_robot
chr11	7593678	7593679	MACS_peak_2241	10.00	lobChIP_FOXA1_robot
chr11	7594695	7594696	MACS_peak_2242	5.00	lobChIP_FOXA1_robot
chr11	7625071	7625072	MACS_peak_2243	4.00	lobChIP_FOXA1_robot
chr11	7633801	7633802	MACS_peak_2244	5.00	lobChIP_FOXA1_robot
chr11	7634772	7634773	MACS_peak_2245	10.00	lobChIP_FOXA1_robot
chr11	7891298	7891299	MACS_peak_2246	5.00	lobChIP_FOXA1_robot
chr11	8720163	8720164	MACS_peak_2247	3.00	lobChIP_FOXA1_robot
chr11	8816599	8816600	MACS_peak_2248	4.00	lobChIP_FOXA1_robot
chr11	9105779	9105780	MACS_peak_2249	4.00	lobChIP_FOXA1_robot
chr11	9365641	9365642	MACS_peak_2250	3.00	lobChIP_FOXA1_robot
chr11	9532501	9532502	MACS_peak_2251	72.00	lobChIP_FOXA1_robot
chr11	9534370	9534371	MACS_peak_2252	4.00	lobChIP_FOXA1_robot
chr11	9587666	9587667	MACS_peak_2253	4.00	lobChIP_FOXA1_robot
chr11	9590933	9590934	MACS_peak_2254	13.00	lobChIP_FOXA1_robot
chr11	9594411	9594412	MACS_peak_2255	10.00	lobChIP_FOXA1_robot
chr11	9634733	9634734	MACS_peak_2256	9.00	lobChIP_FOXA1_robot
chr11	9758077	9758078	MACS_peak_2257	7.00	lobChIP_FOXA1_robot
chr11	9879109	9879110	MACS_peak_2258	3.00	lobChIP_FOXA1_robot
chr11	9928142	9928143	MACS_peak_2259	4.00	lobChIP_FOXA1_robot
chr11	9952603	9952604	MACS_peak_2260	7.00	lobChIP_FOXA1_robot
chr11	10042987	10042988	MACS_peak_2261	4.00	lobChIP_FOXA1_robot
chr11	10068238	10068239	MACS_peak_2262	4.00	lobChIP_FOXA1_robot
chr11	10072228	10072229	MACS_peak_2263	6.00	lobChIP_FOXA1_robot
chr11	10127116	10127117	MACS_peak_2264	5.00	lobChIP_FOXA1_robot
chr11	10307073	10307074	MACS_peak_2265	13.00	lobChIP_FOXA1_robot
chr11	10348229	10348230	MACS_peak_2266	13.00	lobChIP_FOXA1_robot
chr11	10373441	10373442	MACS_peak_2267	5.00	lobChIP_FOXA1_robot
chr11	10681373	10681374	MACS_peak_2268	6.00	lobChIP_FOXA1_robot
chr11	10915393	10915394	MACS_peak_2269	5.00	lobChIP_FOXA1_robot
chr11	10949266	10949267	MACS_peak_2270	4.00	lobChIP_FOXA1_robot
chr11	12014206	12014207	MACS_peak_2271	20.00	lobChIP_FOXA1_robot
chr11	12051595	12051596	MACS_peak_2272	6.00	lobChIP_FOXA1_robot
chr11	12093286	12093287	MACS_peak_2273	4.00	lobChIP_FOXA1_robot
chr11	12107576	12107577	MACS_peak_2274	3.00	lobChIP_FOXA1_robot
chr11	12170540	12170541	MACS_peak_2275	6.00	lobChIP_FOXA1_robot
chr11	12240662	12240663	MACS_peak_2276	7.00	lobChIP_FOXA1_robot
chr11	12621204	12621205	MACS_peak_2277	6.00	lobChIP_FOXA1_robot
chr11	12691756	12691757	MACS_peak_2278	6.00	lobChIP_FOXA1_robot
chr11	12728882	12728883	MACS_peak_2279	6.00	lobChIP_FOXA1_robot
chr11	12824342	12824343	MACS_peak_2280	6.00	lobChIP_FOXA1_robot
chr11	12867683	12867684	MACS_peak_2281	9.00	lobChIP_FOXA1_robot
chr11	13029699	13029700	MACS_peak_2282	13.00	lobChIP_FOXA1_robot
chr11	13033168	13033169	MACS_peak_2283	10.00	lobChIP_FOXA1_robot
chr11	13034902	13034903	MACS_peak_2284	4.00	lobChIP_FOXA1_robot
chr11	13112065	13112066	MACS_peak_2285	3.00	lobChIP_FOXA1_robot
chr11	13162082	13162083	MACS_peak_2286	10.00	lobChIP_FOXA1_robot
chr11	13204828	13204829	MACS_peak_2287	6.00	lobChIP_FOXA1_robot
chr11	13408319	13408320	MACS_peak_2288	4.00	lobChIP_FOXA1_robot
chr11	14542353	14542354	MACS_peak_2289	5.00	lobChIP_FOXA1_robot
chr11	14588270	14588271	MACS_peak_2290	11.00	lobChIP_FOXA1_robot
chr11	14600296	14600297	MACS_peak_2291	4.00	lobChIP_FOXA1_robot
chr11	14605075	14605076	MACS_peak_2292	4.00	lobChIP_FOXA1_robot
chr11	14634292	14634293	MACS_peak_2293	5.00	lobChIP_FOXA1_robot
chr11	14641773	14641774	MACS_peak_2294	23.00	lobChIP_FOXA1_robot
chr11	14679361	14679362	MACS_peak_2295	3.00	lobChIP_FOXA1_robot
chr11	14697797	14697798	MACS_peak_2296	6.00	lobChIP_FOXA1_robot
chr11	14702457	14702458	MACS_peak_2297	3.00	lobChIP_FOXA1_robot
chr11	14709900	14709901	MACS_peak_2298	4.00	lobChIP_FOXA1_robot
chr11	14762897	14762898	MACS_peak_2299	7.00	lobChIP_FOXA1_robot
chr11	14787738	14787739	MACS_peak_2300	3.00	lobChIP_FOXA1_robot
chr11	14839527	14839528	MACS_peak_2301	7.00	lobChIP_FOXA1_robot
chr11	14908341	14908342	MACS_peak_2302	5.00	lobChIP_FOXA1_robot
chr11	15640193	15640194	MACS_peak_2303	5.00	lobChIP_FOXA1_robot
chr11	16023864	16023865	MACS_peak_2304	4.00	lobChIP_FOXA1_robot
chr11	16136598	16136599	MACS_peak_2305	13.00	lobChIP_FOXA1_robot
chr11	16176523	16176524	MACS_peak_2306	4.00	lobChIP_FOXA1_robot
chr11	16206303	16206304	MACS_peak_2307	13.00	lobChIP_FOXA1_robot
chr11	16215959	16215960	MACS_peak_2308	5.00	lobChIP_FOXA1_robot
chr11	16378111	16378112	MACS_peak_2309	3.00	lobChIP_FOXA1_robot
chr11	17003500	17003501	MACS_peak_2310	4.00	lobChIP_FOXA1_robot
chr11	17026516	17026517	MACS_peak_2311	8.00	lobChIP_FOXA1_robot
chr11	17070218	17070219	MACS_peak_2312	6.00	lobChIP_FOXA1_robot
chr11	17162716	17162717	MACS_peak_2314	4.00	lobChIP_FOXA1_robot
chr11	17219292	17219293	MACS_peak_2315	3.00	lobChIP_FOXA1_robot
chr11	17461201	17461202	MACS_peak_2316	24.00	lobChIP_FOXA1_robot
chr11	17791723	17791724	MACS_peak_2317	25.00	lobChIP_FOXA1_robot
chr11	18008032	18008033	MACS_peak_2318	7.00	lobChIP_FOXA1_robot
chr11	18008754	18008755	MACS_peak_2319	5.00	lobChIP_FOXA1_robot
chr11	18022532	18022533	MACS_peak_2320	5.00	lobChIP_FOXA1_robot
chr11	18045513	18045514	MACS_peak_2321	5.00	lobChIP_FOXA1_robot
chr11	18140323	18140324	MACS_peak_2322	9.00	lobChIP_FOXA1_robot
chr11	18245666	18245667	MACS_peak_2323	14.00	lobChIP_FOXA1_robot
chr11	18258742	18258743	MACS_peak_2324	10.00	lobChIP_FOXA1_robot
chr11	18429101	18429102	MACS_peak_2325	5.00	lobChIP_FOXA1_robot
chr11	18600782	18600783	MACS_peak_2326	9.00	lobChIP_FOXA1_robot
chr11	19529954	19529955	MACS_peak_2327	6.00	lobChIP_FOXA1_robot
chr11	19929002	19929003	MACS_peak_2328	12.00	lobChIP_FOXA1_robot
chr11	19932624	19932625	MACS_peak_2329	5.00	lobChIP_FOXA1_robot
chr11	19933892	19933893	MACS_peak_2330	16.00	lobChIP_FOXA1_robot
chr11	19973972	19973973	MACS_peak_2331	18.00	lobChIP_FOXA1_robot
chr11	20000522	20000523	MACS_peak_2332	11.00	lobChIP_FOXA1_robot
chr11	20022195	20022196	MACS_peak_2333	5.00	lobChIP_FOXA1_robot
chr11	20024499	20024500	MACS_peak_2334	4.00	lobChIP_FOXA1_robot
chr11	20033044	20033045	MACS_peak_2335	9.00	lobChIP_FOXA1_robot
chr11	20033495	20033496	MACS_peak_2336	3.00	lobChIP_FOXA1_robot
chr11	20044039	20044040	MACS_peak_2337	4.00	lobChIP_FOXA1_robot
chr11	20052785	20052786	MACS_peak_2338	13.00	lobChIP_FOXA1_robot
chr11	20057906	20057907	MACS_peak_2339	9.00	lobChIP_FOXA1_robot
chr11	20106871	20106872	MACS_peak_2340	5.00	lobChIP_FOXA1_robot
chr11	20111829	20111830	MACS_peak_2341	5.00	lobChIP_FOXA1_robot
chr11	20377430	20377431	MACS_peak_2342	5.00	lobChIP_FOXA1_robot
chr11	20499149	20499150	MACS_peak_2343	6.00	lobChIP_FOXA1_robot
chr11	20540027	20540028	MACS_peak_2344	5.00	lobChIP_FOXA1_robot
chr11	22658695	22658696	MACS_peak_2345	6.00	lobChIP_FOXA1_robot
chr11	22696220	22696221	MACS_peak_2346	20.00	lobChIP_FOXA1_robot
chr11	22702317	22702318	MACS_peak_2347	5.00	lobChIP_FOXA1_robot
chr11	22724692	22724693	MACS_peak_2348	5.00	lobChIP_FOXA1_robot
chr11	22752022	22752023	MACS_peak_2349	7.00	lobChIP_FOXA1_robot
chr11	22814083	22814084	MACS_peak_2350	6.00	lobChIP_FOXA1_robot
chr11	22949528	22949529	MACS_peak_2351	4.00	lobChIP_FOXA1_robot
chr11	25456996	25456997	MACS_peak_2352	6.00	lobChIP_FOXA1_robot
chr11	25758813	25758814	MACS_peak_2353	10.00	lobChIP_FOXA1_robot
chr11	26165730	26165731	MACS_peak_2354	6.00	lobChIP_FOXA1_robot
chr11	26209408	26209409	MACS_peak_2355	8.00	lobChIP_FOXA1_robot
chr11	27076847	27076848	MACS_peak_2356	6.00	lobChIP_FOXA1_robot
chr11	27223637	27223638	MACS_peak_2357	6.00	lobChIP_FOXA1_robot
chr11	27227030	27227031	MACS_peak_2358	5.00	lobChIP_FOXA1_robot
chr11	27318238	27318239	MACS_peak_2359	5.00	lobChIP_FOXA1_robot
chr11	27333867	27333868	MACS_peak_2360	6.00	lobChIP_FOXA1_robot
chr11	27343248	27343249	MACS_peak_2361	4.00	lobChIP_FOXA1_robot
chr11	27352348	27352349	MACS_peak_2362	16.00	lobChIP_FOXA1_robot
chr11	27396034	27396035	MACS_peak_2363	25.00	lobChIP_FOXA1_robot
chr11	27417272	27417273	MACS_peak_2364	6.00	lobChIP_FOXA1_robot
chr11	27423401	27423402	MACS_peak_2365	25.00	lobChIP_FOXA1_robot
chr11	27477877	27477878	MACS_peak_2366	5.00	lobChIP_FOXA1_robot
chr11	27478646	27478647	MACS_peak_2367	12.00	lobChIP_FOXA1_robot
chr11	27481871	27481872	MACS_peak_2368	4.00	lobChIP_FOXA1_robot
chr11	27489239	27489240	MACS_peak_2369	5.00	lobChIP_FOXA1_robot
chr11	27497369	27497370	MACS_peak_2370	14.00	lobChIP_FOXA1_robot
chr11	27895330	27895331	MACS_peak_2371	4.00	lobChIP_FOXA1_robot
chr11	28037198	28037199	MACS_peak_2372	3.00	lobChIP_FOXA1_robot
chr11	29303814	29303815	MACS_peak_2373	4.00	lobChIP_FOXA1_robot
chr11	29342989	29342990	MACS_peak_2374	10.00	lobChIP_FOXA1_robot
chr11	30510382	30510383	MACS_peak_2375	28.00	lobChIP_FOXA1_robot
chr11	31081292	31081293	MACS_peak_2376	8.00	lobChIP_FOXA1_robot
chr11	31529100	31529101	MACS_peak_2377	4.00	lobChIP_FOXA1_robot
chr11	31676774	31676775	MACS_peak_2378	4.00	lobChIP_FOXA1_robot
chr11	31703710	31703711	MACS_peak_2379	8.00	lobChIP_FOXA1_robot
chr11	31706125	31706126	MACS_peak_2380	24.00	lobChIP_FOXA1_robot
chr11	31833665	31833666	MACS_peak_2381	8.00	lobChIP_FOXA1_robot
chr11	32125132	32125133	MACS_peak_2382	33.00	lobChIP_FOXA1_robot
chr11	32602818	32602819	MACS_peak_2383	3.00	lobChIP_FOXA1_robot
chr11	32861291	32861292	MACS_peak_2384	6.00	lobChIP_FOXA1_robot
chr11	32913211	32913212	MACS_peak_2385	4.00	lobChIP_FOXA1_robot
chr11	33089073	33089074	MACS_peak_2386	3.00	lobChIP_FOXA1_robot
chr11	33155857	33155858	MACS_peak_2387	4.00	lobChIP_FOXA1_robot
chr11	33204270	33204271	MACS_peak_2388	6.00	lobChIP_FOXA1_robot
chr11	33288323	33288324	MACS_peak_2389	4.00	lobChIP_FOXA1_robot
chr11	33653389	33653390	MACS_peak_2390	4.00	lobChIP_FOXA1_robot
chr11	33655275	33655276	MACS_peak_2391	4.00	lobChIP_FOXA1_robot
chr11	33721273	33721274	MACS_peak_2392	5.00	lobChIP_FOXA1_robot
chr11	33853831	33853832	MACS_peak_2393	4.00	lobChIP_FOXA1_robot
chr11	34235640	34235641	MACS_peak_2394	6.00	lobChIP_FOXA1_robot
chr11	34240473	34240474	MACS_peak_2395	5.00	lobChIP_FOXA1_robot
chr11	34354235	34354236	MACS_peak_2396	8.00	lobChIP_FOXA1_robot
chr11	34367005	34367006	MACS_peak_2397	9.00	lobChIP_FOXA1_robot
chr11	34385047	34385048	MACS_peak_2398	13.00	lobChIP_FOXA1_robot
chr11	34385848	34385849	MACS_peak_2399	13.00	lobChIP_FOXA1_robot
chr11	34437018	34437019	MACS_peak_2400	3.00	lobChIP_FOXA1_robot
chr11	34448800	34448801	MACS_peak_2401	5.00	lobChIP_FOXA1_robot
chr11	34527619	34527620	MACS_peak_2402	5.00	lobChIP_FOXA1_robot
chr11	34662044	34662045	MACS_peak_2403	18.00	lobChIP_FOXA1_robot
chr11	34676250	34676251	MACS_peak_2404	4.00	lobChIP_FOXA1_robot
chr11	34676689	34676690	MACS_peak_2405	4.00	lobChIP_FOXA1_robot
chr11	34677897	34677898	MACS_peak_2406	6.00	lobChIP_FOXA1_robot
chr11	34686836	34686837	MACS_peak_2407	35.00	lobChIP_FOXA1_robot
chr11	34687806	34687807	MACS_peak_2408	5.00	lobChIP_FOXA1_robot
chr11	34689874	34689875	MACS_peak_2409	39.00	lobChIP_FOXA1_robot
chr11	34702681	34702682	MACS_peak_2410	6.00	lobChIP_FOXA1_robot
chr11	34805763	34805764	MACS_peak_2411	12.00	lobChIP_FOXA1_robot
chr11	34908956	34908957	MACS_peak_2412	9.00	lobChIP_FOXA1_robot
chr11	34951585	34951586	MACS_peak_2413	30.00	lobChIP_FOXA1_robot
chr11	35031407	35031408	MACS_peak_2414	5.00	lobChIP_FOXA1_robot
chr11	35194983	35194984	MACS_peak_2415	4.00	lobChIP_FOXA1_robot
chr11	35368372	35368373	MACS_peak_2416	3.00	lobChIP_FOXA1_robot
chr11	35381041	35381042	MACS_peak_2417	6.00	lobChIP_FOXA1_robot
chr11	35381807	35381808	MACS_peak_2418	16.00	lobChIP_FOXA1_robot
chr11	35412775	35412776	MACS_peak_2419	5.00	lobChIP_FOXA1_robot
chr11	35430687	35430688	MACS_peak_2420	4.00	lobChIP_FOXA1_robot
chr11	35683853	35683854	MACS_peak_2421	16.00	lobChIP_FOXA1_robot
chr11	35772038	35772039	MACS_peak_2422	5.00	lobChIP_FOXA1_robot
chr11	36158812	36158813	MACS_peak_2423	3.00	lobChIP_FOXA1_robot
chr11	37897133	37897134	MACS_peak_2424	5.00	lobChIP_FOXA1_robot
chr11	38112818	38112819	MACS_peak_2425	4.00	lobChIP_FOXA1_robot
chr11	38190988	38190989	MACS_peak_2426	17.00	lobChIP_FOXA1_robot
chr11	38669738	38669739	MACS_peak_2427	3.00	lobChIP_FOXA1_robot
chr11	39671670	39671671	MACS_peak_2428	4.00	lobChIP_FOXA1_robot
chr11	40350947	40350948	MACS_peak_2429	4.00	lobChIP_FOXA1_robot
chr11	41211916	41211917	MACS_peak_2430	5.00	lobChIP_FOXA1_robot
chr11	41516326	41516327	MACS_peak_2431	3.00	lobChIP_FOXA1_robot
chr11	42227230	42227231	MACS_peak_2432	5.00	lobChIP_FOXA1_robot
chr11	42516420	42516421	MACS_peak_2433	8.00	lobChIP_FOXA1_robot
chr11	43192039	43192040	MACS_peak_2434	6.00	lobChIP_FOXA1_robot
chr11	44277049	44277050	MACS_peak_2435	4.00	lobChIP_FOXA1_robot
chr11	45292545	45292546	MACS_peak_2436	3.00	lobChIP_FOXA1_robot
chr11	45413026	45413027	MACS_peak_2437	5.00	lobChIP_FOXA1_robot
chr11	45416048	45416049	MACS_peak_2438	8.00	lobChIP_FOXA1_robot
chr11	45870319	45870320	MACS_peak_2439	5.00	lobChIP_FOXA1_robot
chr11	46148831	46148832	MACS_peak_2440	5.00	lobChIP_FOXA1_robot
chr11	46218403	46218404	MACS_peak_2441	4.00	lobChIP_FOXA1_robot
chr11	46274190	46274191	MACS_peak_2442	8.00	lobChIP_FOXA1_robot
chr11	46295589	46295590	MACS_peak_2443	21.00	lobChIP_FOXA1_robot
chr11	46526516	46526517	MACS_peak_2444	4.00	lobChIP_FOXA1_robot
chr11	46576040	46576041	MACS_peak_2445	56.00	lobChIP_FOXA1_robot
chr11	46618885	46618886	MACS_peak_2446	6.00	lobChIP_FOXA1_robot
chr11	46662507	46662508	MACS_peak_2447	7.00	lobChIP_FOXA1_robot
chr11	46722136	46722137	MACS_peak_2448	11.00	lobChIP_FOXA1_robot
chr11	46739909	46739910	MACS_peak_2449	14.00	lobChIP_FOXA1_robot
chr11	46969026	46969027	MACS_peak_2450	4.00	lobChIP_FOXA1_robot
chr11	47006691	47006692	MACS_peak_2451	14.00	lobChIP_FOXA1_robot
chr11	47007215	47007216	MACS_peak_2452	5.00	lobChIP_FOXA1_robot
chr11	47055818	47055819	MACS_peak_2453	6.00	lobChIP_FOXA1_robot
chr11	47057481	47057482	MACS_peak_2454	4.00	lobChIP_FOXA1_robot
chr11	47064060	47064061	MACS_peak_2455	4.00	lobChIP_FOXA1_robot
chr11	47088599	47088600	MACS_peak_2456	3.00	lobChIP_FOXA1_robot
chr11	47121212	47121213	MACS_peak_2457	14.00	lobChIP_FOXA1_robot
chr11	47140695	47140696	MACS_peak_2458	5.00	lobChIP_FOXA1_robot
chr11	47206572	47206573	MACS_peak_2459	6.00	lobChIP_FOXA1_robot
chr11	47211057	47211058	MACS_peak_2460	9.00	lobChIP_FOXA1_robot
chr11	47238165	47238166	MACS_peak_2461	6.00	lobChIP_FOXA1_robot
chr11	47270917	47270918	MACS_peak_2462	6.00	lobChIP_FOXA1_robot
chr11	47293090	47293091	MACS_peak_2463	4.00	lobChIP_FOXA1_robot
chr11	47457332	47457333	MACS_peak_2464	8.00	lobChIP_FOXA1_robot
chr11	47509829	47509830	MACS_peak_2465	4.00	lobChIP_FOXA1_robot
chr11	47538906	47538907	MACS_peak_2466	5.00	lobChIP_FOXA1_robot
chr11	47745749	47745750	MACS_peak_2467	6.00	lobChIP_FOXA1_robot
chr11	47747849	47747850	MACS_peak_2468	5.00	lobChIP_FOXA1_robot
chr11	47748556	47748557	MACS_peak_2469	5.00	lobChIP_FOXA1_robot
chr11	56884268	56884269	MACS_peak_2476	4.00	lobChIP_FOXA1_robot
chr11	57074216	57074217	MACS_peak_2477	9.00	lobChIP_FOXA1_robot
chr11	57078307	57078308	MACS_peak_2478	6.00	lobChIP_FOXA1_robot
chr11	57127962	57127963	MACS_peak_2479	5.00	lobChIP_FOXA1_robot
chr11	57167563	57167564	MACS_peak_2480	13.00	lobChIP_FOXA1_robot
chr11	57209537	57209538	MACS_peak_2481	16.00	lobChIP_FOXA1_robot
chr11	57221209	57221210	MACS_peak_2482	15.00	lobChIP_FOXA1_robot
chr11	57265447	57265448	MACS_peak_2483	7.00	lobChIP_FOXA1_robot
chr11	57270509	57270510	MACS_peak_2484	5.00	lobChIP_FOXA1_robot
chr11	57320170	57320171	MACS_peak_2485	6.00	lobChIP_FOXA1_robot
chr11	57525869	57525870	MACS_peak_2486	9.00	lobChIP_FOXA1_robot
chr11	57545690	57545691	MACS_peak_2487	11.00	lobChIP_FOXA1_robot
chr11	57568203	57568204	MACS_peak_2488	26.00	lobChIP_FOXA1_robot
chr11	57703658	57703659	MACS_peak_2489	6.00	lobChIP_FOXA1_robot
chr11	57739516	57739517	MACS_peak_2490	22.00	lobChIP_FOXA1_robot
chr11	57746335	57746336	MACS_peak_2491	15.00	lobChIP_FOXA1_robot
chr11	57828938	57828939	MACS_peak_2492	3.00	lobChIP_FOXA1_robot
chr11	57933036	57933037	MACS_peak_2493	3.00	lobChIP_FOXA1_robot
chr11	58375751	58375752	MACS_peak_2494	4.00	lobChIP_FOXA1_robot
chr11	58529913	58529914	MACS_peak_2495	19.00	lobChIP_FOXA1_robot
chr11	58648267	58648268	MACS_peak_2496	9.00	lobChIP_FOXA1_robot
chr11	58693965	58693966	MACS_peak_2497	21.00	lobChIP_FOXA1_robot
chr11	59328665	59328666	MACS_peak_2498	4.00	lobChIP_FOXA1_robot
chr11	59544144	59544145	MACS_peak_2499	10.00	lobChIP_FOXA1_robot
chr11	59554644	59554645	MACS_peak_2500	6.00	lobChIP_FOXA1_robot
chr11	59578382	59578383	MACS_peak_2501	4.00	lobChIP_FOXA1_robot
chr11	59866294	59866295	MACS_peak_2502	9.00	lobChIP_FOXA1_robot
chr11	59937028	59937029	MACS_peak_2503	5.00	lobChIP_FOXA1_robot
chr11	60138410	60138411	MACS_peak_2504	8.00	lobChIP_FOXA1_robot
chr11	60612410	60612411	MACS_peak_2505	15.00	lobChIP_FOXA1_robot
chr11	60623686	60623687	MACS_peak_2506	5.00	lobChIP_FOXA1_robot
chr11	60648649	60648650	MACS_peak_2507	13.00	lobChIP_FOXA1_robot
chr11	61066365	61066366	MACS_peak_2508	3.00	lobChIP_FOXA1_robot
chr11	61369229	61369230	MACS_peak_2509	4.00	lobChIP_FOXA1_robot
chr11	61377001	61377002	MACS_peak_2510	11.00	lobChIP_FOXA1_robot
chr11	61449903	61449904	MACS_peak_2511	7.00	lobChIP_FOXA1_robot
chr11	61691198	61691199	MACS_peak_2512	10.00	lobChIP_FOXA1_robot
chr11	61740601	61740602	MACS_peak_2513	3.00	lobChIP_FOXA1_robot
chr11	61762649	61762650	MACS_peak_2514	4.00	lobChIP_FOXA1_robot
chr11	61944509	61944510	MACS_peak_2515	5.00	lobChIP_FOXA1_robot
chr11	62027930	62027931	MACS_peak_2516	8.00	lobChIP_FOXA1_robot
chr11	62108287	62108288	MACS_peak_2517	5.00	lobChIP_FOXA1_robot
chr11	62193096	62193097	MACS_peak_2518	3.00	lobChIP_FOXA1_robot
chr11	62206188	62206189	MACS_peak_2519	5.00	lobChIP_FOXA1_robot
chr11	62235443	62235444	MACS_peak_2520	12.00	lobChIP_FOXA1_robot
chr11	62255392	62255393	MACS_peak_2521	6.00	lobChIP_FOXA1_robot
chr11	62271847	62271848	MACS_peak_2522	6.00	lobChIP_FOXA1_robot
chr11	62282597	62282598	MACS_peak_2523	6.00	lobChIP_FOXA1_robot
chr11	62316544	62316545	MACS_peak_2524	12.00	lobChIP_FOXA1_robot
chr11	62355398	62355399	MACS_peak_2525	16.00	lobChIP_FOXA1_robot
chr11	62408383	62408384	MACS_peak_2526	6.00	lobChIP_FOXA1_robot
chr11	62420962	62420963	MACS_peak_2527	5.00	lobChIP_FOXA1_robot
chr11	62599184	62599185	MACS_peak_2528	5.00	lobChIP_FOXA1_robot
chr11	62609252	62609253	MACS_peak_2529	7.00	lobChIP_FOXA1_robot
chr11	62620093	62620094	MACS_peak_2530	5.00	lobChIP_FOXA1_robot
chr11	63006918	63006919	MACS_peak_2531	16.00	lobChIP_FOXA1_robot
chr11	63008355	63008356	MACS_peak_2532	15.00	lobChIP_FOXA1_robot
chr11	63015395	63015396	MACS_peak_2533	9.00	lobChIP_FOXA1_robot
chr11	63017783	63017784	MACS_peak_2534	13.00	lobChIP_FOXA1_robot
chr11	63259389	63259390	MACS_peak_2535	5.00	lobChIP_FOXA1_robot
chr11	63629184	63629185	MACS_peak_2536	5.00	lobChIP_FOXA1_robot
chr11	63630551	63630552	MACS_peak_2537	5.00	lobChIP_FOXA1_robot
chr11	64005231	64005232	MACS_peak_2538	5.00	lobChIP_FOXA1_robot
chr11	64334161	64334162	MACS_peak_2539	9.00	lobChIP_FOXA1_robot
chr11	64683863	64683864	MACS_peak_2540	6.00	lobChIP_FOXA1_robot
chr11	64863947	64863948	MACS_peak_2541	7.00	lobChIP_FOXA1_robot
chr11	64933079	64933080	MACS_peak_2542	5.00	lobChIP_FOXA1_robot
chr11	64933509	64933510	MACS_peak_2543	4.00	lobChIP_FOXA1_robot
chr11	65042883	65042884	MACS_peak_2544	6.00	lobChIP_FOXA1_robot
chr11	65073568	65073569	MACS_peak_2545	23.00	lobChIP_FOXA1_robot
chr11	65125571	65125572	MACS_peak_2546	4.00	lobChIP_FOXA1_robot
chr11	65138874	65138875	MACS_peak_2547	10.00	lobChIP_FOXA1_robot
chr11	65169414	65169415	MACS_peak_2548	6.00	lobChIP_FOXA1_robot
chr11	65186414	65186415	MACS_peak_2549	5.00	lobChIP_FOXA1_robot
chr11	65187574	65187575	MACS_peak_2550	5.00	lobChIP_FOXA1_robot
chr11	65259408	65259409	MACS_peak_2551	4.00	lobChIP_FOXA1_robot
chr11	65431663	65431664	MACS_peak_2552	5.00	lobChIP_FOXA1_robot
chr11	65904534	65904535	MACS_peak_2553	10.00	lobChIP_FOXA1_robot
chr11	65963481	65963482	MACS_peak_2554	4.00	lobChIP_FOXA1_robot
chr11	66118877	66118878	MACS_peak_2555	30.00	lobChIP_FOXA1_robot
chr11	66147584	66147585	MACS_peak_2556	5.00	lobChIP_FOXA1_robot
chr11	66669615	66669616	MACS_peak_2557	19.00	lobChIP_FOXA1_robot
chr11	66704054	66704055	MACS_peak_2558	5.00	lobChIP_FOXA1_robot
chr11	66738432	66738433	MACS_peak_2559	4.00	lobChIP_FOXA1_robot
chr11	66761226	66761227	MACS_peak_2560	3.00	lobChIP_FOXA1_robot
chr11	66764525	66764526	MACS_peak_2561	5.00	lobChIP_FOXA1_robot
chr11	67025652	67025653	MACS_peak_2562	4.00	lobChIP_FOXA1_robot
chr11	67184480	67184481	MACS_peak_2563	5.00	lobChIP_FOXA1_robot
chr11	67275639	67275640	MACS_peak_2564	5.00	lobChIP_FOXA1_robot
chr11	67346695	67346696	MACS_peak_2565	20.00	lobChIP_FOXA1_robot
chr11	67384835	67384836	MACS_peak_2566	5.00	lobChIP_FOXA1_robot
chr11	67769736	67769737	MACS_peak_2567	8.00	lobChIP_FOXA1_robot
chr11	67780931	67780932	MACS_peak_2568	5.00	lobChIP_FOXA1_robot
chr11	67867037	67867038	MACS_peak_2569	20.00	lobChIP_FOXA1_robot
chr11	67905501	67905502	MACS_peak_2570	11.00	lobChIP_FOXA1_robot
chr11	67914678	67914679	MACS_peak_2571	20.00	lobChIP_FOXA1_robot
chr11	68038758	68038759	MACS_peak_2572	10.00	lobChIP_FOXA1_robot
chr11	68065795	68065796	MACS_peak_2573	10.00	lobChIP_FOXA1_robot
chr11	68081458	68081459	MACS_peak_2574	6.00	lobChIP_FOXA1_robot
chr11	68084526	68084527	MACS_peak_2575	5.00	lobChIP_FOXA1_robot
chr11	68099238	68099239	MACS_peak_2576	8.00	lobChIP_FOXA1_robot
chr11	68148034	68148035	MACS_peak_2577	15.00	lobChIP_FOXA1_robot
chr11	68174572	68174573	MACS_peak_2578	6.00	lobChIP_FOXA1_robot
chr11	68223780	68223781	MACS_peak_2579	4.00	lobChIP_FOXA1_robot
chr11	68235909	68235910	MACS_peak_2580	9.00	lobChIP_FOXA1_robot
chr11	68236837	68236838	MACS_peak_2581	6.00	lobChIP_FOXA1_robot
chr11	68300425	68300426	MACS_peak_2582	4.00	lobChIP_FOXA1_robot
chr11	68613070	68613071	MACS_peak_2583	15.00	lobChIP_FOXA1_robot
chr11	68625195	68625196	MACS_peak_2584	26.00	lobChIP_FOXA1_robot
chr11	68890382	68890383	MACS_peak_2585	7.00	lobChIP_FOXA1_robot
chr11	68924917	68924918	MACS_peak_2586	8.00	lobChIP_FOXA1_robot
chr11	68942110	68942111	MACS_peak_2587	4.00	lobChIP_FOXA1_robot
chr11	69312293	69312294	MACS_peak_2588	6.00	lobChIP_FOXA1_robot
chr11	69383183	69383184	MACS_peak_2589	12.00	lobChIP_FOXA1_robot
chr11	69469791	69469792	MACS_peak_2590	19.00	lobChIP_FOXA1_robot
chr11	69482926	69482927	MACS_peak_2591	4.00	lobChIP_FOXA1_robot
chr11	69503416	69503417	MACS_peak_2592	4.00	lobChIP_FOXA1_robot
chr11	69504651	69504652	MACS_peak_2593	4.00	lobChIP_FOXA1_robot
chr11	69816630	69816631	MACS_peak_2594	45.00	lobChIP_FOXA1_robot
chr11	70058560	70058561	MACS_peak_2595	8.00	lobChIP_FOXA1_robot
chr11	70545571	70545572	MACS_peak_2596	7.00	lobChIP_FOXA1_robot
chr11	70952379	70952380	MACS_peak_2597	5.00	lobChIP_FOXA1_robot
chr11	70964654	70964655	MACS_peak_2598	20.00	lobChIP_FOXA1_robot
chr11	71018158	71018159	MACS_peak_2599	14.00	lobChIP_FOXA1_robot
chr11	71161608	71161609	MACS_peak_2600	5.00	lobChIP_FOXA1_robot
chr11	71274939	71274940	MACS_peak_2601	4.00	lobChIP_FOXA1_robot
chr11	71658755	71658756	MACS_peak_2602	6.00	lobChIP_FOXA1_robot
chr11	71910443	71910444	MACS_peak_2603	4.00	lobChIP_FOXA1_robot
chr11	71980446	71980447	MACS_peak_2604	3.00	lobChIP_FOXA1_robot
chr11	72109248	72109249	MACS_peak_2605	31.00	lobChIP_FOXA1_robot
chr11	72125776	72125777	MACS_peak_2606	7.00	lobChIP_FOXA1_robot
chr11	72129239	72129240	MACS_peak_2607	3.00	lobChIP_FOXA1_robot
chr11	72142209	72142210	MACS_peak_2608	8.00	lobChIP_FOXA1_robot
chr11	72477570	72477571	MACS_peak_2609	12.00	lobChIP_FOXA1_robot
chr11	72489054	72489055	MACS_peak_2610	4.00	lobChIP_FOXA1_robot
chr11	72490144	72490145	MACS_peak_2611	11.00	lobChIP_FOXA1_robot
chr11	72493347	72493348	MACS_peak_2612	5.00	lobChIP_FOXA1_robot
chr11	72497648	72497649	MACS_peak_2613	8.00	lobChIP_FOXA1_robot
chr11	72518499	72518500	MACS_peak_2614	4.00	lobChIP_FOXA1_robot
chr11	72758938	72758939	MACS_peak_2615	5.00	lobChIP_FOXA1_robot
chr11	72770272	72770273	MACS_peak_2616	10.00	lobChIP_FOXA1_robot
chr11	72784017	72784018	MACS_peak_2617	8.00	lobChIP_FOXA1_robot
chr11	72846767	72846768	MACS_peak_2618	5.00	lobChIP_FOXA1_robot
chr11	72851063	72851064	MACS_peak_2619	3.00	lobChIP_FOXA1_robot
chr11	72851573	72851574	MACS_peak_2620	5.00	lobChIP_FOXA1_robot
chr11	72854856	72854857	MACS_peak_2621	5.00	lobChIP_FOXA1_robot
chr11	72874903	72874904	MACS_peak_2622	3.00	lobChIP_FOXA1_robot
chr11	72884545	72884546	MACS_peak_2623	8.00	lobChIP_FOXA1_robot
chr11	72886871	72886872	MACS_peak_2624	7.00	lobChIP_FOXA1_robot
chr11	72901136	72901137	MACS_peak_2625	8.00	lobChIP_FOXA1_robot
chr11	72983236	72983237	MACS_peak_2626	7.00	lobChIP_FOXA1_robot
chr11	73150844	73150845	MACS_peak_2627	5.00	lobChIP_FOXA1_robot
chr11	73340195	73340196	MACS_peak_2628	13.00	lobChIP_FOXA1_robot
chr11	73359928	73359929	MACS_peak_2629	5.00	lobChIP_FOXA1_robot
chr11	73436257	73436258	MACS_peak_2630	3.00	lobChIP_FOXA1_robot
chr11	73459540	73459541	MACS_peak_2631	9.00	lobChIP_FOXA1_robot
chr11	73698064	73698065	MACS_peak_2632	6.00	lobChIP_FOXA1_robot
chr11	73729755	73729756	MACS_peak_2633	4.00	lobChIP_FOXA1_robot
chr11	73777908	73777909	MACS_peak_2634	6.00	lobChIP_FOXA1_robot
chr11	73780337	73780338	MACS_peak_2635	3.00	lobChIP_FOXA1_robot
chr11	73849693	73849694	MACS_peak_2636	6.00	lobChIP_FOXA1_robot
chr11	74176685	74176686	MACS_peak_2637	8.00	lobChIP_FOXA1_robot
chr11	74304631	74304632	MACS_peak_2638	8.00	lobChIP_FOXA1_robot
chr11	74445047	74445048	MACS_peak_2639	12.00	lobChIP_FOXA1_robot
chr11	74451047	74451048	MACS_peak_2640	8.00	lobChIP_FOXA1_robot
chr11	74561111	74561112	MACS_peak_2641	8.00	lobChIP_FOXA1_robot
chr11	74662265	74662266	MACS_peak_2642	3.00	lobChIP_FOXA1_robot
chr11	74724378	74724379	MACS_peak_2643	5.00	lobChIP_FOXA1_robot
chr11	74867119	74867120	MACS_peak_2644	6.00	lobChIP_FOXA1_robot
chr11	74879835	74879836	MACS_peak_2645	8.00	lobChIP_FOXA1_robot
chr11	74915289	74915290	MACS_peak_2646	3.00	lobChIP_FOXA1_robot
chr11	75034906	75034907	MACS_peak_2647	20.00	lobChIP_FOXA1_robot
chr11	75054115	75054116	MACS_peak_2648	7.00	lobChIP_FOXA1_robot
chr11	75083526	75083527	MACS_peak_2649	14.00	lobChIP_FOXA1_robot
chr11	75110549	75110550	MACS_peak_2650	4.00	lobChIP_FOXA1_robot
chr11	75133436	75133437	MACS_peak_2651	16.00	lobChIP_FOXA1_robot
chr11	75429919	75429920	MACS_peak_2652	4.00	lobChIP_FOXA1_robot
chr11	75474085	75474086	MACS_peak_2653	5.00	lobChIP_FOXA1_robot
chr11	75494132	75494133	MACS_peak_2654	14.00	lobChIP_FOXA1_robot
chr11	75495273	75495274	MACS_peak_2655	19.00	lobChIP_FOXA1_robot
chr11	75788768	75788769	MACS_peak_2656	4.00	lobChIP_FOXA1_robot
chr11	75860314	75860315	MACS_peak_2657	6.00	lobChIP_FOXA1_robot
chr11	76040496	76040497	MACS_peak_2658	4.00	lobChIP_FOXA1_robot
chr11	76235223	76235224	MACS_peak_2659	16.00	lobChIP_FOXA1_robot
chr11	76238053	76238054	MACS_peak_2660	22.00	lobChIP_FOXA1_robot
chr11	76483591	76483592	MACS_peak_2661	6.00	lobChIP_FOXA1_robot
chr11	76492420	76492421	MACS_peak_2662	4.00	lobChIP_FOXA1_robot
chr11	76504657	76504658	MACS_peak_2663	8.00	lobChIP_FOXA1_robot
chr11	76510588	76510589	MACS_peak_2664	3.00	lobChIP_FOXA1_robot
chr11	76513057	76513058	MACS_peak_2665	20.00	lobChIP_FOXA1_robot
chr11	76627980	76627981	MACS_peak_2666	20.00	lobChIP_FOXA1_robot
chr11	76649004	76649005	MACS_peak_2667	4.00	lobChIP_FOXA1_robot
chr11	76650362	76650363	MACS_peak_2668	24.00	lobChIP_FOXA1_robot
chr11	76674235	76674236	MACS_peak_2669	17.00	lobChIP_FOXA1_robot
chr11	76731378	76731379	MACS_peak_2670	11.00	lobChIP_FOXA1_robot
chr11	76747568	76747569	MACS_peak_2671	30.00	lobChIP_FOXA1_robot
chr11	76772389	76772390	MACS_peak_2672	30.00	lobChIP_FOXA1_robot
chr11	76842474	76842475	MACS_peak_2673	5.00	lobChIP_FOXA1_robot
chr11	76954460	76954461	MACS_peak_2674	6.00	lobChIP_FOXA1_robot
chr11	77170608	77170609	MACS_peak_2675	10.00	lobChIP_FOXA1_robot
chr11	77342286	77342287	MACS_peak_2676	4.00	lobChIP_FOXA1_robot
chr11	77381338	77381339	MACS_peak_2677	7.00	lobChIP_FOXA1_robot
chr11	77440309	77440310	MACS_peak_2678	8.00	lobChIP_FOXA1_robot
chr11	77491131	77491132	MACS_peak_2679	7.00	lobChIP_FOXA1_robot
chr11	77495872	77495873	MACS_peak_2680	3.00	lobChIP_FOXA1_robot
chr11	77625542	77625543	MACS_peak_2681	4.00	lobChIP_FOXA1_robot
chr11	77744292	77744293	MACS_peak_2682	5.00	lobChIP_FOXA1_robot
chr11	77747618	77747619	MACS_peak_2683	6.00	lobChIP_FOXA1_robot
chr11	77797838	77797839	MACS_peak_2684	3.00	lobChIP_FOXA1_robot
chr11	77801893	77801894	MACS_peak_2685	8.00	lobChIP_FOXA1_robot
chr11	77861993	77861994	MACS_peak_2686	5.00	lobChIP_FOXA1_robot
chr11	77867694	77867695	MACS_peak_2687	9.00	lobChIP_FOXA1_robot
chr11	77897126	77897127	MACS_peak_2688	8.00	lobChIP_FOXA1_robot
chr11	77913948	77913949	MACS_peak_2689	3.00	lobChIP_FOXA1_robot
chr11	77992761	77992762	MACS_peak_2690	3.00	lobChIP_FOXA1_robot
chr11	78001402	78001403	MACS_peak_2691	15.00	lobChIP_FOXA1_robot
chr11	78010546	78010547	MACS_peak_2692	3.00	lobChIP_FOXA1_robot
chr11	78185570	78185571	MACS_peak_2693	4.00	lobChIP_FOXA1_robot
chr11	78251481	78251482	MACS_peak_2694	3.00	lobChIP_FOXA1_robot
chr11	78272322	78272323	MACS_peak_2695	12.00	lobChIP_FOXA1_robot
chr11	78287681	78287682	MACS_peak_2696	4.00	lobChIP_FOXA1_robot
chr11	78312286	78312287	MACS_peak_2697	20.00	lobChIP_FOXA1_robot
chr11	78317896	78317897	MACS_peak_2698	5.00	lobChIP_FOXA1_robot
chr11	78324441	78324442	MACS_peak_2699	8.00	lobChIP_FOXA1_robot
chr11	78832206	78832207	MACS_peak_2700	4.00	lobChIP_FOXA1_robot
chr11	80186776	80186777	MACS_peak_2701	3.00	lobChIP_FOXA1_robot
chr11	80306262	80306263	MACS_peak_2702	5.00	lobChIP_FOXA1_robot
chr11	80693989	80693990	MACS_peak_2703	4.00	lobChIP_FOXA1_robot
chr11	81700310	81700311	MACS_peak_2704	4.00	lobChIP_FOXA1_robot
chr11	82529561	82529562	MACS_peak_2705	4.00	lobChIP_FOXA1_robot
chr11	82667262	82667263	MACS_peak_2706	5.00	lobChIP_FOXA1_robot
chr11	82700137	82700138	MACS_peak_2707	5.00	lobChIP_FOXA1_robot
chr11	82701199	82701200	MACS_peak_2708	5.00	lobChIP_FOXA1_robot
chr11	82719145	82719146	MACS_peak_2709	6.00	lobChIP_FOXA1_robot
chr11	82736593	82736594	MACS_peak_2710	4.00	lobChIP_FOXA1_robot
chr11	82804209	82804210	MACS_peak_2711	12.00	lobChIP_FOXA1_robot
chr11	82827254	82827255	MACS_peak_2712	3.00	lobChIP_FOXA1_robot
chr11	82964027	82964028	MACS_peak_2713	4.00	lobChIP_FOXA1_robot
chr11	82995693	82995694	MACS_peak_2714	9.00	lobChIP_FOXA1_robot
chr11	83232255	83232256	MACS_peak_2715	5.00	lobChIP_FOXA1_robot
chr11	83359875	83359876	MACS_peak_2716	10.00	lobChIP_FOXA1_robot
chr11	83422176	83422177	MACS_peak_2717	4.00	lobChIP_FOXA1_robot
chr11	83557002	83557003	MACS_peak_2718	4.00	lobChIP_FOXA1_robot
chr11	83564111	83564112	MACS_peak_2719	6.00	lobChIP_FOXA1_robot
chr11	83962217	83962218	MACS_peak_2720	4.00	lobChIP_FOXA1_robot
chr11	85340135	85340136	MACS_peak_2722	5.00	lobChIP_FOXA1_robot
chr11	85346036	85346037	MACS_peak_2723	5.00	lobChIP_FOXA1_robot
chr11	85630659	85630660	MACS_peak_2724	11.00	lobChIP_FOXA1_robot
chr11	85634691	85634692	MACS_peak_2725	5.00	lobChIP_FOXA1_robot
chr11	85642280	85642281	MACS_peak_2726	21.00	lobChIP_FOXA1_robot
chr11	85681912	85681913	MACS_peak_2727	8.00	lobChIP_FOXA1_robot
chr11	85740862	85740863	MACS_peak_2728	3.00	lobChIP_FOXA1_robot
chr11	85777185	85777186	MACS_peak_2729	4.00	lobChIP_FOXA1_robot
chr11	85835409	85835410	MACS_peak_2730	5.00	lobChIP_FOXA1_robot
chr11	85845863	85845864	MACS_peak_2731	4.00	lobChIP_FOXA1_robot
chr11	85871744	85871745	MACS_peak_2732	5.00	lobChIP_FOXA1_robot
chr11	85917032	85917033	MACS_peak_2733	16.00	lobChIP_FOXA1_robot
chr11	85921120	85921121	MACS_peak_2734	49.00	lobChIP_FOXA1_robot
chr11	86019058	86019059	MACS_peak_2735	7.00	lobChIP_FOXA1_robot
chr11	86210851	86210852	MACS_peak_2736	6.00	lobChIP_FOXA1_robot
chr11	86235398	86235399	MACS_peak_2737	7.00	lobChIP_FOXA1_robot
chr11	86385241	86385242	MACS_peak_2738	7.00	lobChIP_FOXA1_robot
chr11	86417623	86417624	MACS_peak_2739	32.00	lobChIP_FOXA1_robot
chr11	86453034	86453035	MACS_peak_2740	15.00	lobChIP_FOXA1_robot
chr11	86651314	86651315	MACS_peak_2741	6.00	lobChIP_FOXA1_robot
chr11	86652559	86652560	MACS_peak_2742	19.00	lobChIP_FOXA1_robot
chr11	86663782	86663783	MACS_peak_2743	3.00	lobChIP_FOXA1_robot
chr11	86666968	86666969	MACS_peak_2744	4.00	lobChIP_FOXA1_robot
chr11	86711299	86711300	MACS_peak_2745	13.00	lobChIP_FOXA1_robot
chr11	86716485	86716486	MACS_peak_2746	78.00	lobChIP_FOXA1_robot
chr11	86723468	86723469	MACS_peak_2747	4.00	lobChIP_FOXA1_robot
chr11	86740285	86740286	MACS_peak_2748	16.00	lobChIP_FOXA1_robot
chr11	86747974	86747975	MACS_peak_2749	3.00	lobChIP_FOXA1_robot
chr11	86755105	86755106	MACS_peak_2750	4.00	lobChIP_FOXA1_robot
chr11	86788101	86788102	MACS_peak_2751	26.00	lobChIP_FOXA1_robot
chr11	86832129	86832130	MACS_peak_2752	8.00	lobChIP_FOXA1_robot
chr11	86838186	86838187	MACS_peak_2753	4.00	lobChIP_FOXA1_robot
chr11	86839680	86839681	MACS_peak_2754	18.00	lobChIP_FOXA1_robot
chr11	86865606	86865607	MACS_peak_2755	5.00	lobChIP_FOXA1_robot
chr11	86991269	86991270	MACS_peak_2756	4.00	lobChIP_FOXA1_robot
chr11	87000060	87000061	MACS_peak_2757	3.00	lobChIP_FOXA1_robot
chr11	87687357	87687358	MACS_peak_2758	3.00	lobChIP_FOXA1_robot
chr11	87688348	87688349	MACS_peak_2759	3.00	lobChIP_FOXA1_robot
chr11	88134998	88134999	MACS_peak_2760	4.00	lobChIP_FOXA1_robot
chr11	89982927	89982928	MACS_peak_2761	6.00	lobChIP_FOXA1_robot
chr11	90181461	90181462	MACS_peak_2762	5.00	lobChIP_FOXA1_robot
chr11	90456003	90456004	MACS_peak_2763	6.00	lobChIP_FOXA1_robot
chr11	90471002	90471003	MACS_peak_2764	5.00	lobChIP_FOXA1_robot
chr11	91088000	91088001	MACS_peak_2765	4.00	lobChIP_FOXA1_robot
chr11	91128382	91128383	MACS_peak_2766	6.00	lobChIP_FOXA1_robot
chr11	91130209	91130210	MACS_peak_2767	4.00	lobChIP_FOXA1_robot
chr11	91874393	91874394	MACS_peak_2768	11.00	lobChIP_FOXA1_robot
chr11	92228353	92228354	MACS_peak_2769	8.00	lobChIP_FOXA1_robot
chr11	92903430	92903431	MACS_peak_2770	3.00	lobChIP_FOXA1_robot
chr11	93220479	93220480	MACS_peak_2771	11.00	lobChIP_FOXA1_robot
chr11	93226119	93226120	MACS_peak_2772	8.00	lobChIP_FOXA1_robot
chr11	93259501	93259502	MACS_peak_2773	21.00	lobChIP_FOXA1_robot
chr11	93263620	93263621	MACS_peak_2774	4.00	lobChIP_FOXA1_robot
chr11	93275132	93275133	MACS_peak_2775	3.00	lobChIP_FOXA1_robot
chr11	93303974	93303975	MACS_peak_2776	4.00	lobChIP_FOXA1_robot
chr11	93347150	93347151	MACS_peak_2777	5.00	lobChIP_FOXA1_robot
chr11	93475252	93475253	MACS_peak_2778	4.00	lobChIP_FOXA1_robot
chr11	93478630	93478631	MACS_peak_2779	7.00	lobChIP_FOXA1_robot
chr11	93480434	93480435	MACS_peak_2780	4.00	lobChIP_FOXA1_robot
chr11	93526848	93526849	MACS_peak_2781	4.00	lobChIP_FOXA1_robot
chr11	93550860	93550861	MACS_peak_2782	4.00	lobChIP_FOXA1_robot
chr11	93555070	93555071	MACS_peak_2783	4.00	lobChIP_FOXA1_robot
chr11	93863689	93863690	MACS_peak_2784	4.00	lobChIP_FOXA1_robot
chr11	93878972	93878973	MACS_peak_2785	4.00	lobChIP_FOXA1_robot
chr11	93886654	93886655	MACS_peak_2786	4.00	lobChIP_FOXA1_robot
chr11	93934409	93934410	MACS_peak_2787	5.00	lobChIP_FOXA1_robot
chr11	94182780	94182781	MACS_peak_2788	11.00	lobChIP_FOXA1_robot
chr11	94442060	94442061	MACS_peak_2789	3.00	lobChIP_FOXA1_robot
chr11	95098063	95098064	MACS_peak_2790	13.00	lobChIP_FOXA1_robot
chr11	95221304	95221305	MACS_peak_2791	8.00	lobChIP_FOXA1_robot
chr11	95637352	95637353	MACS_peak_2792	5.00	lobChIP_FOXA1_robot
chr11	95670776	95670777	MACS_peak_2793	7.00	lobChIP_FOXA1_robot
chr11	95745473	95745474	MACS_peak_2794	3.00	lobChIP_FOXA1_robot
chr11	95747566	95747567	MACS_peak_2795	14.00	lobChIP_FOXA1_robot
chr11	95749683	95749684	MACS_peak_2796	8.00	lobChIP_FOXA1_robot
chr11	95832108	95832109	MACS_peak_2797	4.00	lobChIP_FOXA1_robot
chr11	95865696	95865697	MACS_peak_2798	7.00	lobChIP_FOXA1_robot
chr11	95878535	95878536	MACS_peak_2799	5.00	lobChIP_FOXA1_robot
chr11	95941021	95941022	MACS_peak_2800	4.00	lobChIP_FOXA1_robot
chr11	95953649	95953650	MACS_peak_2801	8.00	lobChIP_FOXA1_robot
chr11	95979972	95979973	MACS_peak_2802	5.00	lobChIP_FOXA1_robot
chr11	96062638	96062639	MACS_peak_2803	4.00	lobChIP_FOXA1_robot
chr11	96065724	96065725	MACS_peak_2804	9.00	lobChIP_FOXA1_robot
chr11	96070431	96070432	MACS_peak_2805	6.00	lobChIP_FOXA1_robot
chr11	96117582	96117583	MACS_peak_2806	4.00	lobChIP_FOXA1_robot
chr11	101058721	101058722	MACS_peak_2807	4.00	lobChIP_FOXA1_robot
chr11	101735589	101735590	MACS_peak_2808	6.00	lobChIP_FOXA1_robot
chr11	101783683	101783684	MACS_peak_2809	4.00	lobChIP_FOXA1_robot
chr11	101845108	101845109	MACS_peak_2810	7.00	lobChIP_FOXA1_robot
chr11	101845624	101845625	MACS_peak_2811	5.00	lobChIP_FOXA1_robot
chr11	101865109	101865110	MACS_peak_2812	15.00	lobChIP_FOXA1_robot
chr11	101877808	101877809	MACS_peak_2813	11.00	lobChIP_FOXA1_robot
chr11	101956744	101956745	MACS_peak_2814	10.00	lobChIP_FOXA1_robot
chr11	101982813	101982814	MACS_peak_2815	8.00	lobChIP_FOXA1_robot
chr11	101986484	101986485	MACS_peak_2816	5.00	lobChIP_FOXA1_robot
chr11	101987409	101987410	MACS_peak_2817	9.00	lobChIP_FOXA1_robot
chr11	101989831	101989832	MACS_peak_2818	6.00	lobChIP_FOXA1_robot
chr11	101990958	101990959	MACS_peak_2819	16.00	lobChIP_FOXA1_robot
chr11	102018929	102018930	MACS_peak_2820	6.00	lobChIP_FOXA1_robot
chr11	102028102	102028103	MACS_peak_2821	6.00	lobChIP_FOXA1_robot
chr11	102049163	102049164	MACS_peak_2822	5.00	lobChIP_FOXA1_robot
chr11	102057817	102057818	MACS_peak_2823	14.00	lobChIP_FOXA1_robot
chr11	102061014	102061015	MACS_peak_2824	10.00	lobChIP_FOXA1_robot
chr11	102062864	102062865	MACS_peak_2825	5.00	lobChIP_FOXA1_robot
chr11	102063589	102063590	MACS_peak_2826	18.00	lobChIP_FOXA1_robot
chr11	102072684	102072685	MACS_peak_2827	5.00	lobChIP_FOXA1_robot
chr11	102113051	102113052	MACS_peak_2828	11.00	lobChIP_FOXA1_robot
chr11	102143486	102143487	MACS_peak_2829	4.00	lobChIP_FOXA1_robot
chr11	102150358	102150359	MACS_peak_2830	12.00	lobChIP_FOXA1_robot
chr11	102186915	102186916	MACS_peak_2831	3.00	lobChIP_FOXA1_robot
chr11	102219981	102219982	MACS_peak_2832	5.00	lobChIP_FOXA1_robot
chr11	102238188	102238189	MACS_peak_2833	6.00	lobChIP_FOXA1_robot
chr11	102317647	102317648	MACS_peak_2834	4.00	lobChIP_FOXA1_robot
chr11	102322442	102322443	MACS_peak_2835	25.00	lobChIP_FOXA1_robot
chr11	102331910	102331911	MACS_peak_2836	5.00	lobChIP_FOXA1_robot
chr11	102333539	102333540	MACS_peak_2837	4.00	lobChIP_FOXA1_robot
chr11	102347582	102347583	MACS_peak_2838	4.00	lobChIP_FOXA1_robot
chr11	102355943	102355944	MACS_peak_2839	3.00	lobChIP_FOXA1_robot
chr11	102365080	102365081	MACS_peak_2840	5.00	lobChIP_FOXA1_robot
chr11	102424760	102424761	MACS_peak_2841	4.00	lobChIP_FOXA1_robot
chr11	102470823	102470824	MACS_peak_2842	18.00	lobChIP_FOXA1_robot
chr11	102486013	102486014	MACS_peak_2843	8.00	lobChIP_FOXA1_robot
chr11	103903951	103903952	MACS_peak_2844	7.00	lobChIP_FOXA1_robot
chr11	103955486	103955487	MACS_peak_2845	4.00	lobChIP_FOXA1_robot
chr11	104539913	104539914	MACS_peak_2846	10.00	lobChIP_FOXA1_robot
chr11	104814479	104814480	MACS_peak_2847	5.00	lobChIP_FOXA1_robot
chr11	104827462	104827463	MACS_peak_2848	6.00	lobChIP_FOXA1_robot
chr11	104866025	104866026	MACS_peak_2849	5.00	lobChIP_FOXA1_robot
chr11	104918223	104918224	MACS_peak_2850	13.00	lobChIP_FOXA1_robot
chr11	104922465	104922466	MACS_peak_2851	7.00	lobChIP_FOXA1_robot
chr11	105015726	105015727	MACS_peak_2852	3.00	lobChIP_FOXA1_robot
chr11	105073856	105073857	MACS_peak_2853	10.00	lobChIP_FOXA1_robot
chr11	105077502	105077503	MACS_peak_2854	5.00	lobChIP_FOXA1_robot
chr11	105958723	105958724	MACS_peak_2855	6.00	lobChIP_FOXA1_robot
chr11	106079654	106079655	MACS_peak_2856	6.00	lobChIP_FOXA1_robot
chr11	106320626	106320627	MACS_peak_2857	4.00	lobChIP_FOXA1_robot
chr11	106326996	106326997	MACS_peak_2858	6.00	lobChIP_FOXA1_robot
chr11	106327395	106327396	MACS_peak_2859	4.00	lobChIP_FOXA1_robot
chr11	106333064	106333065	MACS_peak_2860	9.00	lobChIP_FOXA1_robot
chr11	106337737	106337738	MACS_peak_2861	7.00	lobChIP_FOXA1_robot
chr11	106338541	106338542	MACS_peak_2862	4.00	lobChIP_FOXA1_robot
chr11	106665653	106665654	MACS_peak_2863	4.00	lobChIP_FOXA1_robot
chr11	107067704	107067705	MACS_peak_2864	5.00	lobChIP_FOXA1_robot
chr11	107279576	107279577	MACS_peak_2865	20.00	lobChIP_FOXA1_robot
chr11	107298498	107298499	MACS_peak_2866	5.00	lobChIP_FOXA1_robot
chr11	107435798	107435799	MACS_peak_2867	3.00	lobChIP_FOXA1_robot
chr11	107560373	107560374	MACS_peak_2868	3.00	lobChIP_FOXA1_robot
chr11	107730940	107730941	MACS_peak_2869	5.00	lobChIP_FOXA1_robot
chr11	107965069	107965070	MACS_peak_2870	3.00	lobChIP_FOXA1_robot
chr11	108160373	108160374	MACS_peak_2871	4.00	lobChIP_FOXA1_robot
chr11	108186979	108186980	MACS_peak_2872	4.00	lobChIP_FOXA1_robot
chr11	108464654	108464655	MACS_peak_2873	12.00	lobChIP_FOXA1_robot
chr11	108668424	108668425	MACS_peak_2874	5.00	lobChIP_FOXA1_robot
chr11	108759255	108759256	MACS_peak_2875	5.00	lobChIP_FOXA1_robot
chr11	108847153	108847154	MACS_peak_2876	4.00	lobChIP_FOXA1_robot
chr11	108980027	108980028	MACS_peak_2877	9.00	lobChIP_FOXA1_robot
chr11	110155111	110155112	MACS_peak_2878	5.00	lobChIP_FOXA1_robot
chr11	110161547	110161548	MACS_peak_2879	5.00	lobChIP_FOXA1_robot
chr11	110169198	110169199	MACS_peak_2880	20.00	lobChIP_FOXA1_robot
chr11	110176568	110176569	MACS_peak_2881	9.00	lobChIP_FOXA1_robot
chr11	110247877	110247878	MACS_peak_2882	6.00	lobChIP_FOXA1_robot
chr11	111180421	111180422	MACS_peak_2883	22.00	lobChIP_FOXA1_robot
chr11	111205570	111205571	MACS_peak_2884	23.00	lobChIP_FOXA1_robot
chr11	111211025	111211026	MACS_peak_2885	4.00	lobChIP_FOXA1_robot
chr11	111216645	111216646	MACS_peak_2886	4.00	lobChIP_FOXA1_robot
chr11	111222344	111222345	MACS_peak_2887	5.00	lobChIP_FOXA1_robot
chr11	111226778	111226779	MACS_peak_2888	5.00	lobChIP_FOXA1_robot
chr11	111231091	111231092	MACS_peak_2889	20.00	lobChIP_FOXA1_robot
chr11	111235061	111235062	MACS_peak_2890	3.00	lobChIP_FOXA1_robot
chr11	111236748	111236749	MACS_peak_2891	9.00	lobChIP_FOXA1_robot
chr11	111504139	111504140	MACS_peak_2892	22.00	lobChIP_FOXA1_robot
chr11	111509016	111509017	MACS_peak_2893	5.00	lobChIP_FOXA1_robot
chr11	111559455	111559456	MACS_peak_2894	4.00	lobChIP_FOXA1_robot
chr11	111597299	111597300	MACS_peak_2895	10.00	lobChIP_FOXA1_robot
chr11	111627477	111627478	MACS_peak_2896	15.00	lobChIP_FOXA1_robot
chr11	111649176	111649177	MACS_peak_2897	4.00	lobChIP_FOXA1_robot
chr11	111661094	111661095	MACS_peak_2898	17.00	lobChIP_FOXA1_robot
chr11	111671461	111671462	MACS_peak_2899	43.00	lobChIP_FOXA1_robot
chr11	111689514	111689515	MACS_peak_2900	4.00	lobChIP_FOXA1_robot
chr11	111694582	111694583	MACS_peak_2901	4.00	lobChIP_FOXA1_robot
chr11	111711218	111711219	MACS_peak_2902	17.00	lobChIP_FOXA1_robot
chr11	111806177	111806178	MACS_peak_2903	5.00	lobChIP_FOXA1_robot
chr11	111854285	111854286	MACS_peak_2904	4.00	lobChIP_FOXA1_robot
chr11	111859726	111859727	MACS_peak_2905	18.00	lobChIP_FOXA1_robot
chr11	111888450	111888451	MACS_peak_2906	19.00	lobChIP_FOXA1_robot
chr11	112028114	112028115	MACS_peak_2907	6.00	lobChIP_FOXA1_robot
chr11	112087332	112087333	MACS_peak_2908	6.00	lobChIP_FOXA1_robot
chr11	112112426	112112427	MACS_peak_2909	4.00	lobChIP_FOXA1_robot
chr11	113066656	113066657	MACS_peak_2910	8.00	lobChIP_FOXA1_robot
chr11	113487025	113487026	MACS_peak_2911	4.00	lobChIP_FOXA1_robot
chr11	113594728	113594729	MACS_peak_2912	5.00	lobChIP_FOXA1_robot
chr11	114080839	114080840	MACS_peak_2913	4.00	lobChIP_FOXA1_robot
chr11	114186817	114186818	MACS_peak_2914	44.00	lobChIP_FOXA1_robot
chr11	114473501	114473502	MACS_peak_2915	4.00	lobChIP_FOXA1_robot
chr11	115040666	115040667	MACS_peak_2916	10.00	lobChIP_FOXA1_robot
chr11	115118867	115118868	MACS_peak_2917	5.00	lobChIP_FOXA1_robot
chr11	115120084	115120085	MACS_peak_2918	9.00	lobChIP_FOXA1_robot
chr11	115151350	115151351	MACS_peak_2919	6.00	lobChIP_FOXA1_robot
chr11	115204808	115204809	MACS_peak_2920	6.00	lobChIP_FOXA1_robot
chr11	115228019	115228020	MACS_peak_2921	4.00	lobChIP_FOXA1_robot
chr11	115248729	115248730	MACS_peak_2922	5.00	lobChIP_FOXA1_robot
chr11	115280550	115280551	MACS_peak_2923	26.00	lobChIP_FOXA1_robot
chr11	115323284	115323285	MACS_peak_2924	13.00	lobChIP_FOXA1_robot
chr11	115348256	115348257	MACS_peak_2925	3.00	lobChIP_FOXA1_robot
chr11	115360047	115360048	MACS_peak_2926	5.00	lobChIP_FOXA1_robot
chr11	115364903	115364904	MACS_peak_2927	8.00	lobChIP_FOXA1_robot
chr11	115373518	115373519	MACS_peak_2928	5.00	lobChIP_FOXA1_robot
chr11	115389760	115389761	MACS_peak_2929	6.00	lobChIP_FOXA1_robot
chr11	115394847	115394848	MACS_peak_2930	4.00	lobChIP_FOXA1_robot
chr11	115419779	115419780	MACS_peak_2931	6.00	lobChIP_FOXA1_robot
chr11	115834965	115834966	MACS_peak_2932	12.00	lobChIP_FOXA1_robot
chr11	116165680	116165681	MACS_peak_2933	10.00	lobChIP_FOXA1_robot
chr11	116210208	116210209	MACS_peak_2934	7.00	lobChIP_FOXA1_robot
chr11	116247410	116247411	MACS_peak_2935	5.00	lobChIP_FOXA1_robot
chr11	116268651	116268652	MACS_peak_2936	4.00	lobChIP_FOXA1_robot
chr11	116283430	116283431	MACS_peak_2937	20.00	lobChIP_FOXA1_robot
chr11	116298133	116298134	MACS_peak_2938	3.00	lobChIP_FOXA1_robot
chr11	116298869	116298870	MACS_peak_2939	4.00	lobChIP_FOXA1_robot
chr11	116434345	116434346	MACS_peak_2940	10.00	lobChIP_FOXA1_robot
chr11	116538259	116538260	MACS_peak_2941	12.00	lobChIP_FOXA1_robot
chr11	116663138	116663139	MACS_peak_2942	7.00	lobChIP_FOXA1_robot
chr11	116679126	116679127	MACS_peak_2943	50.00	lobChIP_FOXA1_robot
chr11	116680453	116680454	MACS_peak_2944	4.00	lobChIP_FOXA1_robot
chr11	116697710	116697711	MACS_peak_2945	3.00	lobChIP_FOXA1_robot
chr11	116703166	116703167	MACS_peak_2946	30.00	lobChIP_FOXA1_robot
chr11	116708515	116708516	MACS_peak_2947	16.00	lobChIP_FOXA1_robot
chr11	116781590	116781591	MACS_peak_2948	6.00	lobChIP_FOXA1_robot
chr11	116861104	116861105	MACS_peak_2949	4.00	lobChIP_FOXA1_robot
chr11	116892053	116892054	MACS_peak_2950	6.00	lobChIP_FOXA1_robot
chr11	116916938	116916939	MACS_peak_2951	7.00	lobChIP_FOXA1_robot
chr11	116942234	116942235	MACS_peak_2952	8.00	lobChIP_FOXA1_robot
chr11	116943371	116943372	MACS_peak_2953	8.00	lobChIP_FOXA1_robot
chr11	116948286	116948287	MACS_peak_2954	57.00	lobChIP_FOXA1_robot
chr11	116964093	116964094	MACS_peak_2955	7.00	lobChIP_FOXA1_robot
chr11	116966717	116966718	MACS_peak_2956	29.00	lobChIP_FOXA1_robot
chr11	117189902	117189903	MACS_peak_2957	4.00	lobChIP_FOXA1_robot
chr11	117574198	117574199	MACS_peak_2958	11.00	lobChIP_FOXA1_robot
chr11	117588701	117588702	MACS_peak_2959	13.00	lobChIP_FOXA1_robot
chr11	117620135	117620136	MACS_peak_2960	5.00	lobChIP_FOXA1_robot
chr11	117961497	117961498	MACS_peak_2961	5.00	lobChIP_FOXA1_robot
chr11	118066078	118066079	MACS_peak_2962	8.00	lobChIP_FOXA1_robot
chr11	118073577	118073578	MACS_peak_2963	9.00	lobChIP_FOXA1_robot
chr11	118082961	118082962	MACS_peak_2964	10.00	lobChIP_FOXA1_robot
chr11	118117983	118117984	MACS_peak_2965	6.00	lobChIP_FOXA1_robot
chr11	118122907	118122908	MACS_peak_2966	4.00	lobChIP_FOXA1_robot
chr11	118148333	118148334	MACS_peak_2967	4.00	lobChIP_FOXA1_robot
chr11	118185875	118185876	MACS_peak_2968	50.00	lobChIP_FOXA1_robot
chr11	118544609	118544610	MACS_peak_2969	21.00	lobChIP_FOXA1_robot
chr11	118799247	118799248	MACS_peak_2970	6.00	lobChIP_FOXA1_robot
chr11	118891962	118891963	MACS_peak_2971	6.00	lobChIP_FOXA1_robot
chr11	118971139	118971140	MACS_peak_2972	6.00	lobChIP_FOXA1_robot
chr11	118979696	118979697	MACS_peak_2973	7.00	lobChIP_FOXA1_robot
chr11	119205115	119205116	MACS_peak_2974	6.00	lobChIP_FOXA1_robot
chr11	119343115	119343116	MACS_peak_2975	6.00	lobChIP_FOXA1_robot
chr11	119352104	119352105	MACS_peak_2976	3.00	lobChIP_FOXA1_robot
chr11	120078978	120078979	MACS_peak_2977	5.00	lobChIP_FOXA1_robot
chr11	120205731	120205732	MACS_peak_2978	5.00	lobChIP_FOXA1_robot
chr11	120208389	120208390	MACS_peak_2979	4.00	lobChIP_FOXA1_robot
chr11	120259206	120259207	MACS_peak_2980	15.00	lobChIP_FOXA1_robot
chr11	120545360	120545361	MACS_peak_2981	4.00	lobChIP_FOXA1_robot
chr11	120679513	120679514	MACS_peak_2982	4.00	lobChIP_FOXA1_robot
chr11	120689118	120689119	MACS_peak_2983	3.00	lobChIP_FOXA1_robot
chr11	120710127	120710128	MACS_peak_2984	6.00	lobChIP_FOXA1_robot
chr11	120728298	120728299	MACS_peak_2985	3.00	lobChIP_FOXA1_robot
chr11	120906618	120906619	MACS_peak_2986	5.00	lobChIP_FOXA1_robot
chr11	120909735	120909736	MACS_peak_2987	8.00	lobChIP_FOXA1_robot
chr11	120938280	120938281	MACS_peak_2988	22.00	lobChIP_FOXA1_robot
chr11	121163094	121163095	MACS_peak_2989	4.00	lobChIP_FOXA1_robot
chr11	121298396	121298397	MACS_peak_2990	4.00	lobChIP_FOXA1_robot
chr11	121322119	121322120	MACS_peak_2991	5.00	lobChIP_FOXA1_robot
chr11	121324417	121324418	MACS_peak_2992	5.00	lobChIP_FOXA1_robot
chr11	121325019	121325020	MACS_peak_2993	4.00	lobChIP_FOXA1_robot
chr11	121330444	121330445	MACS_peak_2994	24.00	lobChIP_FOXA1_robot
chr11	121331821	121331822	MACS_peak_2995	7.00	lobChIP_FOXA1_robot
chr11	121347218	121347219	MACS_peak_2996	16.00	lobChIP_FOXA1_robot
chr11	121424803	121424804	MACS_peak_2997	5.00	lobChIP_FOXA1_robot
chr11	121435878	121435879	MACS_peak_2998	11.00	lobChIP_FOXA1_robot
chr11	121525659	121525660	MACS_peak_2999	7.00	lobChIP_FOXA1_robot
chr11	121527322	121527323	MACS_peak_3000	8.00	lobChIP_FOXA1_robot
chr11	121582395	121582396	MACS_peak_3001	7.00	lobChIP_FOXA1_robot
chr11	121802943	121802944	MACS_peak_3002	9.00	lobChIP_FOXA1_robot
chr11	122010422	122010423	MACS_peak_3003	8.00	lobChIP_FOXA1_robot
chr11	122021519	122021520	MACS_peak_3004	17.00	lobChIP_FOXA1_robot
chr11	122174379	122174380	MACS_peak_3005	3.00	lobChIP_FOXA1_robot
chr11	122451916	122451917	MACS_peak_3006	4.00	lobChIP_FOXA1_robot
chr11	122513309	122513310	MACS_peak_3007	11.00	lobChIP_FOXA1_robot
chr11	122517518	122517519	MACS_peak_3008	5.00	lobChIP_FOXA1_robot
chr11	122577177	122577178	MACS_peak_3009	6.00	lobChIP_FOXA1_robot
chr11	122714154	122714155	MACS_peak_3010	4.00	lobChIP_FOXA1_robot
chr11	123458952	123458953	MACS_peak_3011	5.00	lobChIP_FOXA1_robot
chr11	124312857	124312858	MACS_peak_3012	5.00	lobChIP_FOXA1_robot
chr11	124479022	124479023	MACS_peak_3013	3.00	lobChIP_FOXA1_robot
chr11	124636337	124636338	MACS_peak_3014	5.00	lobChIP_FOXA1_robot
chr11	124812758	124812759	MACS_peak_3015	5.00	lobChIP_FOXA1_robot
chr11	124928607	124928608	MACS_peak_3016	8.00	lobChIP_FOXA1_robot
chr11	125838547	125838548	MACS_peak_3017	4.00	lobChIP_FOXA1_robot
chr11	125854090	125854091	MACS_peak_3018	5.00	lobChIP_FOXA1_robot
chr11	125857397	125857398	MACS_peak_3019	13.00	lobChIP_FOXA1_robot
chr11	125877357	125877358	MACS_peak_3020	7.00	lobChIP_FOXA1_robot
chr11	125959208	125959209	MACS_peak_3021	9.00	lobChIP_FOXA1_robot
chr11	125985979	125985980	MACS_peak_3022	9.00	lobChIP_FOXA1_robot
chr11	126017822	126017823	MACS_peak_3023	14.00	lobChIP_FOXA1_robot
chr11	126023116	126023117	MACS_peak_3024	7.00	lobChIP_FOXA1_robot
chr11	126033527	126033528	MACS_peak_3025	7.00	lobChIP_FOXA1_robot
chr11	127590087	127590088	MACS_peak_3026	3.00	lobChIP_FOXA1_robot
chr11	128204577	128204578	MACS_peak_3027	3.00	lobChIP_FOXA1_robot
chr11	128623496	128623497	MACS_peak_3028	3.00	lobChIP_FOXA1_robot
chr11	128680986	128680987	MACS_peak_3029	4.00	lobChIP_FOXA1_robot
chr11	128985771	128985772	MACS_peak_3030	20.00	lobChIP_FOXA1_robot
chr11	129072737	129072738	MACS_peak_3031	3.00	lobChIP_FOXA1_robot
chr11	129118730	129118731	MACS_peak_3032	9.00	lobChIP_FOXA1_robot
chr11	129156800	129156801	MACS_peak_3033	6.00	lobChIP_FOXA1_robot
chr11	129194618	129194619	MACS_peak_3034	25.00	lobChIP_FOXA1_robot
chr11	129297524	129297525	MACS_peak_3035	5.00	lobChIP_FOXA1_robot
chr11	129330790	129330791	MACS_peak_3036	8.00	lobChIP_FOXA1_robot
chr11	129375683	129375684	MACS_peak_3037	4.00	lobChIP_FOXA1_robot
chr11	129661724	129661725	MACS_peak_3038	10.00	lobChIP_FOXA1_robot
chr11	129670281	129670282	MACS_peak_3039	7.00	lobChIP_FOXA1_robot
chr11	129670755	129670756	MACS_peak_3040	11.00	lobChIP_FOXA1_robot
chr11	129687747	129687748	MACS_peak_3041	5.00	lobChIP_FOXA1_robot
chr11	129698542	129698543	MACS_peak_3042	12.00	lobChIP_FOXA1_robot
chr11	129723959	129723960	MACS_peak_3043	9.00	lobChIP_FOXA1_robot
chr11	129833467	129833468	MACS_peak_3044	4.00	lobChIP_FOXA1_robot
chr11	129865269	129865270	MACS_peak_3045	5.00	lobChIP_FOXA1_robot
chr11	129869424	129869425	MACS_peak_3046	19.00	lobChIP_FOXA1_robot
chr11	129893548	129893549	MACS_peak_3047	3.00	lobChIP_FOXA1_robot
chr11	129912123	129912124	MACS_peak_3048	5.00	lobChIP_FOXA1_robot
chr11	129920240	129920241	MACS_peak_3049	7.00	lobChIP_FOXA1_robot
chr11	129959476	129959477	MACS_peak_3050	8.00	lobChIP_FOXA1_robot
chr11	129994290	129994291	MACS_peak_3051	4.00	lobChIP_FOXA1_robot
chr11	130014091	130014092	MACS_peak_3052	10.00	lobChIP_FOXA1_robot
chr11	130051668	130051669	MACS_peak_3053	9.00	lobChIP_FOXA1_robot
chr11	130130060	130130061	MACS_peak_3054	3.00	lobChIP_FOXA1_robot
chr11	130132504	130132505	MACS_peak_3055	4.00	lobChIP_FOXA1_robot
chr11	130260212	130260213	MACS_peak_3056	4.00	lobChIP_FOXA1_robot
chr11	130870287	130870288	MACS_peak_3057	4.00	lobChIP_FOXA1_robot
chr11	130969191	130969192	MACS_peak_3058	16.00	lobChIP_FOXA1_robot
chr11	134132071	134132072	MACS_peak_3059	12.00	lobChIP_FOXA1_robot
chr11	134142353	134142354	MACS_peak_3060	4.00	lobChIP_FOXA1_robot
chr11	134613030	134613031	MACS_peak_3061	11.00	lobChIP_FOXA1_robot
chr12	319706	319707	MACS_peak_3062	19.00	lobChIP_FOXA1_robot
chr12	524639	524640	MACS_peak_3063	3.00	lobChIP_FOXA1_robot
chr12	684096	684097	MACS_peak_3064	8.00	lobChIP_FOXA1_robot
chr12	694856	694857	MACS_peak_3065	11.00	lobChIP_FOXA1_robot
chr12	726930	726931	MACS_peak_3066	3.00	lobChIP_FOXA1_robot
chr12	741469	741470	MACS_peak_3067	11.00	lobChIP_FOXA1_robot
chr12	782294	782295	MACS_peak_3068	8.00	lobChIP_FOXA1_robot
chr12	859677	859678	MACS_peak_3069	5.00	lobChIP_FOXA1_robot
chr12	1163954	1163955	MACS_peak_3070	11.00	lobChIP_FOXA1_robot
chr12	1476061	1476062	MACS_peak_3071	4.00	lobChIP_FOXA1_robot
chr12	1776050	1776051	MACS_peak_3072	5.00	lobChIP_FOXA1_robot
chr12	1815611	1815612	MACS_peak_3073	5.00	lobChIP_FOXA1_robot
chr12	1842283	1842284	MACS_peak_3074	7.00	lobChIP_FOXA1_robot
chr12	1854336	1854337	MACS_peak_3075	16.00	lobChIP_FOXA1_robot
chr12	1868213	1868214	MACS_peak_3076	5.00	lobChIP_FOXA1_robot
chr12	1869746	1869747	MACS_peak_3077	4.00	lobChIP_FOXA1_robot
chr12	1875452	1875453	MACS_peak_3078	4.00	lobChIP_FOXA1_robot
chr12	1877085	1877086	MACS_peak_3079	7.00	lobChIP_FOXA1_robot
chr12	1891150	1891151	MACS_peak_3080	5.00	lobChIP_FOXA1_robot
chr12	1947157	1947158	MACS_peak_3081	11.00	lobChIP_FOXA1_robot
chr12	2103175	2103176	MACS_peak_3082	16.00	lobChIP_FOXA1_robot
chr12	2353481	2353482	MACS_peak_3083	3.00	lobChIP_FOXA1_robot
chr12	2357333	2357334	MACS_peak_3084	10.00	lobChIP_FOXA1_robot
chr12	2894445	2894446	MACS_peak_3085	6.00	lobChIP_FOXA1_robot
chr12	2959190	2959191	MACS_peak_3086	7.00	lobChIP_FOXA1_robot
chr12	2986854	2986855	MACS_peak_3087	6.00	lobChIP_FOXA1_robot
chr12	3653913	3653914	MACS_peak_3088	4.00	lobChIP_FOXA1_robot
chr12	4108730	4108731	MACS_peak_3089	5.00	lobChIP_FOXA1_robot
chr12	4121028	4121029	MACS_peak_3090	12.00	lobChIP_FOXA1_robot
chr12	4124864	4124865	MACS_peak_3091	7.00	lobChIP_FOXA1_robot
chr12	4166590	4166591	MACS_peak_3092	10.00	lobChIP_FOXA1_robot
chr12	4595410	4595411	MACS_peak_3093	21.00	lobChIP_FOXA1_robot
chr12	4615763	4615764	MACS_peak_3094	9.00	lobChIP_FOXA1_robot
chr12	4648098	4648099	MACS_peak_3095	5.00	lobChIP_FOXA1_robot
chr12	4651823	4651824	MACS_peak_3096	4.00	lobChIP_FOXA1_robot
chr12	5677741	5677742	MACS_peak_3097	7.00	lobChIP_FOXA1_robot
chr12	5695561	5695562	MACS_peak_3098	4.00	lobChIP_FOXA1_robot
chr12	6344910	6344911	MACS_peak_3099	5.00	lobChIP_FOXA1_robot
chr12	6389332	6389333	MACS_peak_3100	6.00	lobChIP_FOXA1_robot
chr12	6403820	6403821	MACS_peak_3101	5.00	lobChIP_FOXA1_robot
chr12	6480432	6480433	MACS_peak_3102	5.00	lobChIP_FOXA1_robot
chr12	6481842	6481843	MACS_peak_3103	3.00	lobChIP_FOXA1_robot
chr12	6529803	6529804	MACS_peak_3104	4.00	lobChIP_FOXA1_robot
chr12	6589590	6589591	MACS_peak_3105	3.00	lobChIP_FOXA1_robot
chr12	6885130	6885131	MACS_peak_3106	18.00	lobChIP_FOXA1_robot
chr12	7123295	7123296	MACS_peak_3107	4.00	lobChIP_FOXA1_robot
chr12	7514923	7514924	MACS_peak_3108	4.00	lobChIP_FOXA1_robot
chr12	7951400	7951401	MACS_peak_3109	4.00	lobChIP_FOXA1_robot
chr12	8004414	8004415	MACS_peak_3110	20.00	lobChIP_FOXA1_robot
chr12	8031962	8031963	MACS_peak_3111	5.00	lobChIP_FOXA1_robot
chr12	8043562	8043563	MACS_peak_3112	3.00	lobChIP_FOXA1_robot
chr12	8058360	8058361	MACS_peak_3113	16.00	lobChIP_FOXA1_robot
chr12	8059893	8059894	MACS_peak_3114	13.00	lobChIP_FOXA1_robot
chr12	8088729	8088730	MACS_peak_3115	5.00	lobChIP_FOXA1_robot
chr12	8135080	8135081	MACS_peak_3116	10.00	lobChIP_FOXA1_robot
chr12	9266719	9266720	MACS_peak_3117	29.00	lobChIP_FOXA1_robot
chr12	9268557	9268558	MACS_peak_3118	9.00	lobChIP_FOXA1_robot
chr12	9274852	9274853	MACS_peak_3119	60.00	lobChIP_FOXA1_robot
chr12	9284850	9284851	MACS_peak_3120	32.00	lobChIP_FOXA1_robot
chr12	9288253	9288254	MACS_peak_3121	5.00	lobChIP_FOXA1_robot
chr12	9288650	9288651	MACS_peak_3122	29.00	lobChIP_FOXA1_robot
chr12	9495768	9495769	MACS_peak_3123	3.00	lobChIP_FOXA1_robot
chr12	9624818	9624819	MACS_peak_3124	4.00	lobChIP_FOXA1_robot
chr12	9803770	9803771	MACS_peak_3125	5.00	lobChIP_FOXA1_robot
chr12	10122216	10122217	MACS_peak_3126	4.00	lobChIP_FOXA1_robot
chr12	10419424	10419425	MACS_peak_3127	4.00	lobChIP_FOXA1_robot
chr12	10548473	10548474	MACS_peak_3128	8.00	lobChIP_FOXA1_robot
chr12	10592409	10592410	MACS_peak_3129	4.00	lobChIP_FOXA1_robot
chr12	11044751	11044752	MACS_peak_3130	30.00	lobChIP_FOXA1_robot
chr12	11071415	11071416	MACS_peak_3131	3.00	lobChIP_FOXA1_robot
chr12	11168569	11168570	MACS_peak_3132	6.00	lobChIP_FOXA1_robot
chr12	11224130	11224131	MACS_peak_3133	11.00	lobChIP_FOXA1_robot
chr12	11940184	11940185	MACS_peak_3134	4.00	lobChIP_FOXA1_robot
chr12	11967225	11967226	MACS_peak_3135	4.00	lobChIP_FOXA1_robot
chr12	11993647	11993648	MACS_peak_3136	3.00	lobChIP_FOXA1_robot
chr12	12019569	12019570	MACS_peak_3137	4.00	lobChIP_FOXA1_robot
chr12	12035008	12035009	MACS_peak_3138	4.00	lobChIP_FOXA1_robot
chr12	12038864	12038865	MACS_peak_3139	12.00	lobChIP_FOXA1_robot
chr12	12248088	12248089	MACS_peak_3140	4.00	lobChIP_FOXA1_robot
chr12	12369383	12369384	MACS_peak_3141	8.00	lobChIP_FOXA1_robot
chr12	12394054	12394055	MACS_peak_3142	3.00	lobChIP_FOXA1_robot
chr12	12464575	12464576	MACS_peak_3143	12.00	lobChIP_FOXA1_robot
chr12	12467084	12467085	MACS_peak_3144	3.00	lobChIP_FOXA1_robot
chr12	12504358	12504359	MACS_peak_3145	18.00	lobChIP_FOXA1_robot
chr12	12534342	12534343	MACS_peak_3146	8.00	lobChIP_FOXA1_robot
chr12	12545106	12545107	MACS_peak_3147	6.00	lobChIP_FOXA1_robot
chr12	12551401	12551402	MACS_peak_3148	23.00	lobChIP_FOXA1_robot
chr12	12556917	12556918	MACS_peak_3149	28.00	lobChIP_FOXA1_robot
chr12	12588024	12588025	MACS_peak_3150	8.00	lobChIP_FOXA1_robot
chr12	12592661	12592662	MACS_peak_3151	6.00	lobChIP_FOXA1_robot
chr12	12602662	12602663	MACS_peak_3152	16.00	lobChIP_FOXA1_robot
chr12	12642224	12642225	MACS_peak_3153	10.00	lobChIP_FOXA1_robot
chr12	12684076	12684077	MACS_peak_3154	4.00	lobChIP_FOXA1_robot
chr12	12699470	12699471	MACS_peak_3155	22.00	lobChIP_FOXA1_robot
chr12	12713996	12713997	MACS_peak_3156	10.00	lobChIP_FOXA1_robot
chr12	12857707	12857708	MACS_peak_3157	7.00	lobChIP_FOXA1_robot
chr12	12874765	12874766	MACS_peak_3158	4.00	lobChIP_FOXA1_robot
chr12	12875470	12875471	MACS_peak_3159	4.00	lobChIP_FOXA1_robot
chr12	12932723	12932724	MACS_peak_3160	7.00	lobChIP_FOXA1_robot
chr12	13025205	13025206	MACS_peak_3161	4.00	lobChIP_FOXA1_robot
chr12	13036545	13036546	MACS_peak_3162	4.00	lobChIP_FOXA1_robot
chr12	13100176	13100177	MACS_peak_3163	7.00	lobChIP_FOXA1_robot
chr12	14145670	14145671	MACS_peak_3164	6.00	lobChIP_FOXA1_robot
chr12	14537937	14537938	MACS_peak_3165	7.00	lobChIP_FOXA1_robot
chr12	14930393	14930394	MACS_peak_3166	5.00	lobChIP_FOXA1_robot
chr12	15680686	15680687	MACS_peak_3167	5.00	lobChIP_FOXA1_robot
chr12	15746032	15746033	MACS_peak_3168	8.00	lobChIP_FOXA1_robot
chr12	15769703	15769704	MACS_peak_3169	23.00	lobChIP_FOXA1_robot
chr12	15865870	15865871	MACS_peak_3170	6.00	lobChIP_FOXA1_robot
chr12	15933472	15933473	MACS_peak_3171	6.00	lobChIP_FOXA1_robot
chr12	15973160	15973161	MACS_peak_3172	23.00	lobChIP_FOXA1_robot
chr12	15976605	15976606	MACS_peak_3173	11.00	lobChIP_FOXA1_robot
chr12	16046520	16046521	MACS_peak_3174	5.00	lobChIP_FOXA1_robot
chr12	16283577	16283578	MACS_peak_3175	6.00	lobChIP_FOXA1_robot
chr12	16503887	16503888	MACS_peak_3176	6.00	lobChIP_FOXA1_robot
chr12	18395834	18395835	MACS_peak_3177	7.00	lobChIP_FOXA1_robot
chr12	19219553	19219554	MACS_peak_3178	28.00	lobChIP_FOXA1_robot
chr12	19250066	19250067	MACS_peak_3179	5.00	lobChIP_FOXA1_robot
chr12	20328960	20328961	MACS_peak_3180	5.00	lobChIP_FOXA1_robot
chr12	20778713	20778714	MACS_peak_3182	4.00	lobChIP_FOXA1_robot
chr12	20781832	20781833	MACS_peak_3183	4.00	lobChIP_FOXA1_robot
chr12	20961683	20961684	MACS_peak_3184	6.00	lobChIP_FOXA1_robot
chr12	21504135	21504136	MACS_peak_3185	18.00	lobChIP_FOXA1_robot
chr12	21716022	21716023	MACS_peak_3186	10.00	lobChIP_FOXA1_robot
chr12	22751771	22751772	MACS_peak_3187	4.00	lobChIP_FOXA1_robot
chr12	23533845	23533846	MACS_peak_3188	4.00	lobChIP_FOXA1_robot
chr12	23910534	23910535	MACS_peak_3189	8.00	lobChIP_FOXA1_robot
chr12	24000883	24000884	MACS_peak_3190	4.00	lobChIP_FOXA1_robot
chr12	24094967	24094968	MACS_peak_3191	12.00	lobChIP_FOXA1_robot
chr12	24178290	24178291	MACS_peak_3192	15.00	lobChIP_FOXA1_robot
chr12	24191496	24191497	MACS_peak_3193	16.00	lobChIP_FOXA1_robot
chr12	24202307	24202308	MACS_peak_3194	5.00	lobChIP_FOXA1_robot
chr12	24223010	24223011	MACS_peak_3195	14.00	lobChIP_FOXA1_robot
chr12	24231316	24231317	MACS_peak_3196	7.00	lobChIP_FOXA1_robot
chr12	24233644	24233645	MACS_peak_3197	15.00	lobChIP_FOXA1_robot
chr12	24514011	24514012	MACS_peak_3198	8.00	lobChIP_FOXA1_robot
chr12	24704526	24704527	MACS_peak_3199	4.00	lobChIP_FOXA1_robot
chr12	24802758	24802759	MACS_peak_3200	7.00	lobChIP_FOXA1_robot
chr12	24907809	24907810	MACS_peak_3201	38.00	lobChIP_FOXA1_robot
chr12	24913311	24913312	MACS_peak_3202	6.00	lobChIP_FOXA1_robot
chr12	25409106	25409107	MACS_peak_3203	10.00	lobChIP_FOXA1_robot
chr12	25429646	25429647	MACS_peak_3204	5.00	lobChIP_FOXA1_robot
chr12	25539222	25539223	MACS_peak_3205	4.00	lobChIP_FOXA1_robot
chr12	26182391	26182392	MACS_peak_3206	7.00	lobChIP_FOXA1_robot
chr12	26392800	26392801	MACS_peak_3207	3.00	lobChIP_FOXA1_robot
chr12	26427855	26427856	MACS_peak_3208	10.00	lobChIP_FOXA1_robot
chr12	26582173	26582174	MACS_peak_3209	18.00	lobChIP_FOXA1_robot
chr12	26793227	26793228	MACS_peak_3210	5.00	lobChIP_FOXA1_robot
chr12	26834181	26834182	MACS_peak_3211	7.00	lobChIP_FOXA1_robot
chr12	26889878	26889879	MACS_peak_3212	42.00	lobChIP_FOXA1_robot
chr12	26898089	26898090	MACS_peak_3213	10.00	lobChIP_FOXA1_robot
chr12	26922315	26922316	MACS_peak_3214	120.00	lobChIP_FOXA1_robot
chr12	26968587	26968588	MACS_peak_3215	9.00	lobChIP_FOXA1_robot
chr12	26972006	26972007	MACS_peak_3216	3.00	lobChIP_FOXA1_robot
chr12	27395312	27395313	MACS_peak_3217	4.00	lobChIP_FOXA1_robot
chr12	27702740	27702741	MACS_peak_3218	4.00	lobChIP_FOXA1_robot
chr12	27792323	27792324	MACS_peak_3219	5.00	lobChIP_FOXA1_robot
chr12	27863243	27863244	MACS_peak_3220	5.00	lobChIP_FOXA1_robot
chr12	27971742	27971743	MACS_peak_3221	3.00	lobChIP_FOXA1_robot
chr12	28051179	28051180	MACS_peak_3222	8.00	lobChIP_FOXA1_robot
chr12	28052504	28052505	MACS_peak_3223	5.00	lobChIP_FOXA1_robot
chr12	28174826	28174827	MACS_peak_3224	9.00	lobChIP_FOXA1_robot
chr12	28355827	28355828	MACS_peak_3225	5.00	lobChIP_FOXA1_robot
chr12	29961833	29961834	MACS_peak_3226	3.00	lobChIP_FOXA1_robot
chr12	30661758	30661759	MACS_peak_3227	25.00	lobChIP_FOXA1_robot
chr12	30752675	30752676	MACS_peak_3228	36.00	lobChIP_FOXA1_robot
chr12	30766530	30766531	MACS_peak_3229	7.00	lobChIP_FOXA1_robot
chr12	30775028	30775029	MACS_peak_3230	6.00	lobChIP_FOXA1_robot
chr12	30779985	30779986	MACS_peak_3231	4.00	lobChIP_FOXA1_robot
chr12	30885998	30885999	MACS_peak_3232	5.00	lobChIP_FOXA1_robot
chr12	30916268	30916269	MACS_peak_3233	16.00	lobChIP_FOXA1_robot
chr12	31508555	31508556	MACS_peak_3234	10.00	lobChIP_FOXA1_robot
chr12	31677814	31677815	MACS_peak_3235	3.00	lobChIP_FOXA1_robot
chr12	31679272	31679273	MACS_peak_3236	5.00	lobChIP_FOXA1_robot
chr12	31742921	31742922	MACS_peak_3237	3.00	lobChIP_FOXA1_robot
chr12	31812554	31812555	MACS_peak_3238	4.00	lobChIP_FOXA1_robot
chr12	31868346	31868347	MACS_peak_3239	9.00	lobChIP_FOXA1_robot
chr12	31902427	31902428	MACS_peak_3240	33.00	lobChIP_FOXA1_robot
chr12	31926164	31926165	MACS_peak_3241	15.00	lobChIP_FOXA1_robot
chr12	31998734	31998735	MACS_peak_3242	19.00	lobChIP_FOXA1_robot
chr12	32007288	32007289	MACS_peak_3243	11.00	lobChIP_FOXA1_robot
chr12	32123450	32123451	MACS_peak_3244	6.00	lobChIP_FOXA1_robot
chr12	32340189	32340190	MACS_peak_3245	24.00	lobChIP_FOXA1_robot
chr12	32599433	32599434	MACS_peak_3246	4.00	lobChIP_FOXA1_robot
chr12	32614457	32614458	MACS_peak_3247	11.00	lobChIP_FOXA1_robot
chr12	32687128	32687129	MACS_peak_3248	9.00	lobChIP_FOXA1_robot
chr12	33046100	33046101	MACS_peak_3249	4.00	lobChIP_FOXA1_robot
chr12	33046596	33046597	MACS_peak_3250	6.00	lobChIP_FOXA1_robot
chr12	33054812	33054813	MACS_peak_3251	3.00	lobChIP_FOXA1_robot
chr12	33478526	33478527	MACS_peak_3252	4.00	lobChIP_FOXA1_robot
chr12	38518503	38518504	MACS_peak_3256	9.00	lobChIP_FOXA1_robot
chr12	39280206	39280207	MACS_peak_3257	5.00	lobChIP_FOXA1_robot
chr12	39286090	39286091	MACS_peak_3258	27.00	lobChIP_FOXA1_robot
chr12	39783057	39783058	MACS_peak_3259	5.00	lobChIP_FOXA1_robot
chr12	39821407	39821408	MACS_peak_3260	8.00	lobChIP_FOXA1_robot
chr12	41145961	41145962	MACS_peak_3261	5.00	lobChIP_FOXA1_robot
chr12	41450872	41450873	MACS_peak_3262	3.00	lobChIP_FOXA1_robot
chr12	41680494	41680495	MACS_peak_3263	6.00	lobChIP_FOXA1_robot
chr12	42789270	42789271	MACS_peak_3264	4.00	lobChIP_FOXA1_robot
chr12	44189818	44189819	MACS_peak_3265	4.00	lobChIP_FOXA1_robot
chr12	44374837	44374838	MACS_peak_3266	4.00	lobChIP_FOXA1_robot
chr12	44389193	44389194	MACS_peak_3267	7.00	lobChIP_FOXA1_robot
chr12	44553146	44553147	MACS_peak_3268	5.00	lobChIP_FOXA1_robot
chr12	44838958	44838959	MACS_peak_3269	7.00	lobChIP_FOXA1_robot
chr12	44919769	44919770	MACS_peak_3270	8.00	lobChIP_FOXA1_robot
chr12	45586095	45586096	MACS_peak_3271	3.00	lobChIP_FOXA1_robot
chr12	45654129	45654130	MACS_peak_3272	4.00	lobChIP_FOXA1_robot
chr12	45655346	45655347	MACS_peak_3273	3.00	lobChIP_FOXA1_robot
chr12	45990268	45990269	MACS_peak_3274	4.00	lobChIP_FOXA1_robot
chr12	46752104	46752105	MACS_peak_3275	5.00	lobChIP_FOXA1_robot
chr12	46759878	46759879	MACS_peak_3276	10.00	lobChIP_FOXA1_robot
chr12	46763802	46763803	MACS_peak_3277	7.00	lobChIP_FOXA1_robot
chr12	46780496	46780497	MACS_peak_3278	7.00	lobChIP_FOXA1_robot
chr12	46824144	46824145	MACS_peak_3279	6.00	lobChIP_FOXA1_robot
chr12	46850974	46850975	MACS_peak_3280	9.00	lobChIP_FOXA1_robot
chr12	46867559	46867560	MACS_peak_3281	18.00	lobChIP_FOXA1_robot
chr12	46877737	46877738	MACS_peak_3282	7.00	lobChIP_FOXA1_robot
chr12	46886457	46886458	MACS_peak_3283	71.00	lobChIP_FOXA1_robot
chr12	46887849	46887850	MACS_peak_3284	30.00	lobChIP_FOXA1_robot
chr12	46917816	46917817	MACS_peak_3285	4.00	lobChIP_FOXA1_robot
chr12	46970973	46970974	MACS_peak_3286	5.00	lobChIP_FOXA1_robot
chr12	46973558	46973559	MACS_peak_3287	7.00	lobChIP_FOXA1_robot
chr12	46979773	46979774	MACS_peak_3288	7.00	lobChIP_FOXA1_robot
chr12	47058988	47058989	MACS_peak_3289	6.00	lobChIP_FOXA1_robot
chr12	47169793	47169794	MACS_peak_3290	5.00	lobChIP_FOXA1_robot
chr12	47208214	47208215	MACS_peak_3291	16.00	lobChIP_FOXA1_robot
chr12	47328746	47328747	MACS_peak_3292	5.00	lobChIP_FOXA1_robot
chr12	47481998	47481999	MACS_peak_3293	4.00	lobChIP_FOXA1_robot
chr12	47615494	47615495	MACS_peak_3294	5.00	lobChIP_FOXA1_robot
chr12	48046837	48046838	MACS_peak_3295	7.00	lobChIP_FOXA1_robot
chr12	48136094	48136095	MACS_peak_3296	3.00	lobChIP_FOXA1_robot
chr12	48162405	48162406	MACS_peak_3297	5.00	lobChIP_FOXA1_robot
chr12	48449729	48449730	MACS_peak_3298	7.00	lobChIP_FOXA1_robot
chr12	48450168	48450169	MACS_peak_3299	6.00	lobChIP_FOXA1_robot
chr12	48517213	48517214	MACS_peak_3300	6.00	lobChIP_FOXA1_robot
chr12	49260898	49260899	MACS_peak_3301	10.00	lobChIP_FOXA1_robot
chr12	49277552	49277553	MACS_peak_3302	5.00	lobChIP_FOXA1_robot
chr12	49304115	49304116	MACS_peak_3303	30.00	lobChIP_FOXA1_robot
chr12	49661446	49661447	MACS_peak_3304	4.00	lobChIP_FOXA1_robot
chr12	49795322	49795323	MACS_peak_3305	4.00	lobChIP_FOXA1_robot
chr12	49815311	49815312	MACS_peak_3306	7.00	lobChIP_FOXA1_robot
chr12	49845352	49845353	MACS_peak_3307	4.00	lobChIP_FOXA1_robot
chr12	50136245	50136246	MACS_peak_3308	5.00	lobChIP_FOXA1_robot
chr12	50635228	50635229	MACS_peak_3309	64.00	lobChIP_FOXA1_robot
chr12	50636182	50636183	MACS_peak_3310	4.00	lobChIP_FOXA1_robot
chr12	50643492	50643493	MACS_peak_3311	4.00	lobChIP_FOXA1_robot
chr12	50653534	50653535	MACS_peak_3312	10.00	lobChIP_FOXA1_robot
chr12	50665482	50665483	MACS_peak_3313	4.00	lobChIP_FOXA1_robot
chr12	50720615	50720616	MACS_peak_3314	8.00	lobChIP_FOXA1_robot
chr12	50781411	50781412	MACS_peak_3315	5.00	lobChIP_FOXA1_robot
chr12	50790437	50790438	MACS_peak_3316	16.00	lobChIP_FOXA1_robot
chr12	50903313	50903314	MACS_peak_3317	5.00	lobChIP_FOXA1_robot
chr12	50920268	50920269	MACS_peak_3318	7.00	lobChIP_FOXA1_robot
chr12	50921034	50921035	MACS_peak_3319	29.00	lobChIP_FOXA1_robot
chr12	50932543	50932544	MACS_peak_3320	4.00	lobChIP_FOXA1_robot
chr12	50942826	50942827	MACS_peak_3321	8.00	lobChIP_FOXA1_robot
chr12	50950181	50950182	MACS_peak_3322	5.00	lobChIP_FOXA1_robot
chr12	51053368	51053369	MACS_peak_3323	4.00	lobChIP_FOXA1_robot
chr12	51180892	51180893	MACS_peak_3324	5.00	lobChIP_FOXA1_robot
chr12	51329059	51329060	MACS_peak_3325	8.00	lobChIP_FOXA1_robot
chr12	51374485	51374486	MACS_peak_3326	4.00	lobChIP_FOXA1_robot
chr12	51418817	51418818	MACS_peak_3327	4.00	lobChIP_FOXA1_robot
chr12	51419468	51419469	MACS_peak_3328	5.00	lobChIP_FOXA1_robot
chr12	51555090	51555091	MACS_peak_3329	4.00	lobChIP_FOXA1_robot
chr12	51592555	51592556	MACS_peak_3330	5.00	lobChIP_FOXA1_robot
chr12	51824102	51824103	MACS_peak_3331	5.00	lobChIP_FOXA1_robot
chr12	51864040	51864041	MACS_peak_3332	8.00	lobChIP_FOXA1_robot
chr12	51918769	51918770	MACS_peak_3333	5.00	lobChIP_FOXA1_robot
chr12	52513280	52513281	MACS_peak_3334	7.00	lobChIP_FOXA1_robot
chr12	52641364	52641365	MACS_peak_3335	6.00	lobChIP_FOXA1_robot
chr12	53278461	53278462	MACS_peak_3336	5.00	lobChIP_FOXA1_robot
chr12	53289237	53289238	MACS_peak_3337	7.00	lobChIP_FOXA1_robot
chr12	53320015	53320016	MACS_peak_3338	5.00	lobChIP_FOXA1_robot
chr12	53343721	53343722	MACS_peak_3339	10.00	lobChIP_FOXA1_robot
chr12	53363721	53363722	MACS_peak_3340	6.00	lobChIP_FOXA1_robot
chr12	53375249	53375250	MACS_peak_3341	4.00	lobChIP_FOXA1_robot
chr12	53386803	53386804	MACS_peak_3342	4.00	lobChIP_FOXA1_robot
chr12	53445119	53445120	MACS_peak_3343	5.00	lobChIP_FOXA1_robot
chr12	53455420	53455421	MACS_peak_3344	7.00	lobChIP_FOXA1_robot
chr12	53568788	53568789	MACS_peak_3345	6.00	lobChIP_FOXA1_robot
chr12	53932071	53932072	MACS_peak_3346	14.00	lobChIP_FOXA1_robot
chr12	54052977	54052978	MACS_peak_3347	7.00	lobChIP_FOXA1_robot
chr12	54219281	54219282	MACS_peak_3348	19.00	lobChIP_FOXA1_robot
chr12	54529539	54529540	MACS_peak_3349	8.00	lobChIP_FOXA1_robot
chr12	54530794	54530795	MACS_peak_3350	8.00	lobChIP_FOXA1_robot
chr12	54547587	54547588	MACS_peak_3351	8.00	lobChIP_FOXA1_robot
chr12	55138603	55138604	MACS_peak_3352	5.00	lobChIP_FOXA1_robot
chr12	55286069	55286070	MACS_peak_3353	25.00	lobChIP_FOXA1_robot
chr12	55893767	55893768	MACS_peak_3354	20.00	lobChIP_FOXA1_robot
chr12	56098814	56098815	MACS_peak_3355	11.00	lobChIP_FOXA1_robot
chr12	56323939	56323940	MACS_peak_3356	6.00	lobChIP_FOXA1_robot
chr12	56475826	56475827	MACS_peak_3357	5.00	lobChIP_FOXA1_robot
chr12	56695769	56695770	MACS_peak_3358	8.00	lobChIP_FOXA1_robot
chr12	56756652	56756653	MACS_peak_3359	9.00	lobChIP_FOXA1_robot
chr12	56802837	56802838	MACS_peak_3360	5.00	lobChIP_FOXA1_robot
chr12	57153333	57153334	MACS_peak_3361	16.00	lobChIP_FOXA1_robot
chr12	57162441	57162442	MACS_peak_3362	4.00	lobChIP_FOXA1_robot
chr12	57335632	57335633	MACS_peak_3363	9.00	lobChIP_FOXA1_robot
chr12	57395450	57395451	MACS_peak_3364	7.00	lobChIP_FOXA1_robot
chr12	57495824	57495825	MACS_peak_3365	8.00	lobChIP_FOXA1_robot
chr12	57524898	57524899	MACS_peak_3366	6.00	lobChIP_FOXA1_robot
chr12	57543862	57543863	MACS_peak_3367	22.00	lobChIP_FOXA1_robot
chr12	57548823	57548824	MACS_peak_3368	6.00	lobChIP_FOXA1_robot
chr12	57762486	57762487	MACS_peak_3369	6.00	lobChIP_FOXA1_robot
chr12	57845423	57845424	MACS_peak_3370	6.00	lobChIP_FOXA1_robot
chr12	57970098	57970099	MACS_peak_3371	6.00	lobChIP_FOXA1_robot
chr12	57973729	57973730	MACS_peak_3372	4.00	lobChIP_FOXA1_robot
chr12	58087036	58087037	MACS_peak_3373	7.00	lobChIP_FOXA1_robot
chr12	58091742	58091743	MACS_peak_3374	4.00	lobChIP_FOXA1_robot
chr12	58296429	58296430	MACS_peak_3375	6.00	lobChIP_FOXA1_robot
chr12	58619263	58619264	MACS_peak_3376	5.00	lobChIP_FOXA1_robot
chr12	59160008	59160009	MACS_peak_3377	12.00	lobChIP_FOXA1_robot
chr12	59306793	59306794	MACS_peak_3378	5.00	lobChIP_FOXA1_robot
chr12	60118244	60118245	MACS_peak_3379	7.00	lobChIP_FOXA1_robot
chr12	60562388	60562389	MACS_peak_3380	9.00	lobChIP_FOXA1_robot
chr12	60660544	60660545	MACS_peak_3381	8.00	lobChIP_FOXA1_robot
chr12	60845472	60845473	MACS_peak_3382	7.00	lobChIP_FOXA1_robot
chr12	62400108	62400109	MACS_peak_3383	7.00	lobChIP_FOXA1_robot
chr12	62762450	62762451	MACS_peak_3384	5.00	lobChIP_FOXA1_robot
chr12	63154766	63154767	MACS_peak_3385	17.00	lobChIP_FOXA1_robot
chr12	63286110	63286111	MACS_peak_3386	8.00	lobChIP_FOXA1_robot
chr12	63319663	63319664	MACS_peak_3387	5.00	lobChIP_FOXA1_robot
chr12	63351065	63351066	MACS_peak_3388	7.00	lobChIP_FOXA1_robot
chr12	63360804	63360805	MACS_peak_3389	22.00	lobChIP_FOXA1_robot
chr12	64256301	64256302	MACS_peak_3390	7.00	lobChIP_FOXA1_robot
chr12	64312022	64312023	MACS_peak_3391	4.00	lobChIP_FOXA1_robot
chr12	64338423	64338424	MACS_peak_3392	8.00	lobChIP_FOXA1_robot
chr12	64984157	64984158	MACS_peak_3393	3.00	lobChIP_FOXA1_robot
chr12	65019134	65019135	MACS_peak_3394	5.00	lobChIP_FOXA1_robot
chr12	65028001	65028002	MACS_peak_3395	4.00	lobChIP_FOXA1_robot
chr12	65050387	65050388	MACS_peak_3396	14.00	lobChIP_FOXA1_robot
chr12	65059927	65059928	MACS_peak_3397	5.00	lobChIP_FOXA1_robot
chr12	65138922	65138923	MACS_peak_3398	5.00	lobChIP_FOXA1_robot
chr12	65507558	65507559	MACS_peak_3399	11.00	lobChIP_FOXA1_robot
chr12	65721602	65721603	MACS_peak_3400	11.00	lobChIP_FOXA1_robot
chr12	66025559	66025560	MACS_peak_3401	5.00	lobChIP_FOXA1_robot
chr12	66202612	66202613	MACS_peak_3402	6.00	lobChIP_FOXA1_robot
chr12	66232533	66232534	MACS_peak_3403	10.00	lobChIP_FOXA1_robot
chr12	66232946	66232947	MACS_peak_3404	4.00	lobChIP_FOXA1_robot
chr12	66283115	66283116	MACS_peak_3405	5.00	lobChIP_FOXA1_robot
chr12	66289702	66289703	MACS_peak_3406	5.00	lobChIP_FOXA1_robot
chr12	66323991	66323992	MACS_peak_3407	16.00	lobChIP_FOXA1_robot
chr12	66331248	66331249	MACS_peak_3408	3.00	lobChIP_FOXA1_robot
chr12	66355053	66355054	MACS_peak_3409	7.00	lobChIP_FOXA1_robot
chr12	66443050	66443051	MACS_peak_3410	39.00	lobChIP_FOXA1_robot
chr12	66468408	66468409	MACS_peak_3411	3.00	lobChIP_FOXA1_robot
chr12	67680530	67680531	MACS_peak_3412	3.00	lobChIP_FOXA1_robot
chr12	68693563	68693564	MACS_peak_3413	4.00	lobChIP_FOXA1_robot
chr12	68738089	68738090	MACS_peak_3414	8.00	lobChIP_FOXA1_robot
chr12	69181330	69181331	MACS_peak_3415	8.00	lobChIP_FOXA1_robot
chr12	69183058	69183059	MACS_peak_3416	7.00	lobChIP_FOXA1_robot
chr12	69201638	69201639	MACS_peak_3417	6.00	lobChIP_FOXA1_robot
chr12	69446687	69446688	MACS_peak_3418	5.00	lobChIP_FOXA1_robot
chr12	69719601	69719602	MACS_peak_3419	5.00	lobChIP_FOXA1_robot
chr12	69726791	69726792	MACS_peak_3420	5.00	lobChIP_FOXA1_robot
chr12	69732269	69732270	MACS_peak_3421	5.00	lobChIP_FOXA1_robot
chr12	69741979	69741980	MACS_peak_3422	4.00	lobChIP_FOXA1_robot
chr12	69810428	69810429	MACS_peak_3423	4.00	lobChIP_FOXA1_robot
chr12	69811808	69811809	MACS_peak_3424	11.00	lobChIP_FOXA1_robot
chr12	70319318	70319319	MACS_peak_3425	8.00	lobChIP_FOXA1_robot
chr12	71549267	71549268	MACS_peak_3426	8.00	lobChIP_FOXA1_robot
chr12	71552031	71552032	MACS_peak_3427	4.00	lobChIP_FOXA1_robot
chr12	71552577	71552578	MACS_peak_3428	13.00	lobChIP_FOXA1_robot
chr12	71553290	71553291	MACS_peak_3429	4.00	lobChIP_FOXA1_robot
chr12	71557122	71557123	MACS_peak_3430	22.00	lobChIP_FOXA1_robot
chr12	71805569	71805570	MACS_peak_3431	9.00	lobChIP_FOXA1_robot
chr12	71832937	71832938	MACS_peak_3432	25.00	lobChIP_FOXA1_robot
chr12	71834759	71834760	MACS_peak_3433	10.00	lobChIP_FOXA1_robot
chr12	71866403	71866404	MACS_peak_3434	4.00	lobChIP_FOXA1_robot
chr12	71886619	71886620	MACS_peak_3435	6.00	lobChIP_FOXA1_robot
chr12	71930818	71930819	MACS_peak_3436	21.00	lobChIP_FOXA1_robot
chr12	71947132	71947133	MACS_peak_3437	4.00	lobChIP_FOXA1_robot
chr12	72137058	72137059	MACS_peak_3438	3.00	lobChIP_FOXA1_robot
chr12	72234400	72234401	MACS_peak_3439	4.00	lobChIP_FOXA1_robot
chr12	72341527	72341528	MACS_peak_3440	6.00	lobChIP_FOXA1_robot
chr12	72348893	72348894	MACS_peak_3441	9.00	lobChIP_FOXA1_robot
chr12	73777761	73777762	MACS_peak_3442	6.00	lobChIP_FOXA1_robot
chr12	74163248	74163249	MACS_peak_3443	23.00	lobChIP_FOXA1_robot
chr12	74305154	74305155	MACS_peak_3444	5.00	lobChIP_FOXA1_robot
chr12	75009272	75009273	MACS_peak_3445	6.00	lobChIP_FOXA1_robot
chr12	75307837	75307838	MACS_peak_3446	8.00	lobChIP_FOXA1_robot
chr12	75310318	75310319	MACS_peak_3447	4.00	lobChIP_FOXA1_robot
chr12	75357704	75357705	MACS_peak_3448	5.00	lobChIP_FOXA1_robot
chr12	75972525	75972526	MACS_peak_3449	5.00	lobChIP_FOXA1_robot
chr12	76022915	76022916	MACS_peak_3450	5.00	lobChIP_FOXA1_robot
chr12	76029572	76029573	MACS_peak_3451	8.00	lobChIP_FOXA1_robot
chr12	76060333	76060334	MACS_peak_3452	41.00	lobChIP_FOXA1_robot
chr12	76102710	76102711	MACS_peak_3453	7.00	lobChIP_FOXA1_robot
chr12	76106218	76106219	MACS_peak_3454	4.00	lobChIP_FOXA1_robot
chr12	76145767	76145768	MACS_peak_3455	5.00	lobChIP_FOXA1_robot
chr12	76149067	76149068	MACS_peak_3456	3.00	lobChIP_FOXA1_robot
chr12	76151089	76151090	MACS_peak_3457	4.00	lobChIP_FOXA1_robot
chr12	76160651	76160652	MACS_peak_3458	15.00	lobChIP_FOXA1_robot
chr12	76192502	76192503	MACS_peak_3459	19.00	lobChIP_FOXA1_robot
chr12	76238650	76238651	MACS_peak_3460	4.00	lobChIP_FOXA1_robot
chr12	76329906	76329907	MACS_peak_3461	10.00	lobChIP_FOXA1_robot
chr12	76338067	76338068	MACS_peak_3462	63.00	lobChIP_FOXA1_robot
chr12	76372211	76372212	MACS_peak_3463	5.00	lobChIP_FOXA1_robot
chr12	76380361	76380362	MACS_peak_3464	5.00	lobChIP_FOXA1_robot
chr12	76384337	76384338	MACS_peak_3465	47.00	lobChIP_FOXA1_robot
chr12	76419379	76419380	MACS_peak_3466	5.00	lobChIP_FOXA1_robot
chr12	76425946	76425947	MACS_peak_3467	4.00	lobChIP_FOXA1_robot
chr12	76426454	76426455	MACS_peak_3468	5.00	lobChIP_FOXA1_robot
chr12	76436030	76436031	MACS_peak_3469	4.00	lobChIP_FOXA1_robot
chr12	77015557	77015558	MACS_peak_3470	17.00	lobChIP_FOXA1_robot
chr12	78687376	78687377	MACS_peak_3471	6.00	lobChIP_FOXA1_robot
chr12	79809726	79809727	MACS_peak_3472	3.00	lobChIP_FOXA1_robot
chr12	79815902	79815903	MACS_peak_3473	4.00	lobChIP_FOXA1_robot
chr12	79818240	79818241	MACS_peak_3474	7.00	lobChIP_FOXA1_robot
chr12	79924235	79924236	MACS_peak_3475	18.00	lobChIP_FOXA1_robot
chr12	79943609	79943610	MACS_peak_3476	22.00	lobChIP_FOXA1_robot
chr12	79949038	79949039	MACS_peak_3477	4.00	lobChIP_FOXA1_robot
chr12	79954036	79954037	MACS_peak_3478	9.00	lobChIP_FOXA1_robot
chr12	79960508	79960509	MACS_peak_3479	5.00	lobChIP_FOXA1_robot
chr12	80037790	80037791	MACS_peak_3480	24.00	lobChIP_FOXA1_robot
chr12	80056188	80056189	MACS_peak_3481	6.00	lobChIP_FOXA1_robot
chr12	80079011	80079012	MACS_peak_3482	5.00	lobChIP_FOXA1_robot
chr12	80082794	80082795	MACS_peak_3483	5.00	lobChIP_FOXA1_robot
chr12	80166256	80166257	MACS_peak_3484	4.00	lobChIP_FOXA1_robot
chr12	80283035	80283036	MACS_peak_3485	4.00	lobChIP_FOXA1_robot
chr12	80294204	80294205	MACS_peak_3486	4.00	lobChIP_FOXA1_robot
chr12	81318412	81318413	MACS_peak_3487	5.00	lobChIP_FOXA1_robot
chr12	81320038	81320039	MACS_peak_3488	12.00	lobChIP_FOXA1_robot
chr12	81332403	81332404	MACS_peak_3489	9.00	lobChIP_FOXA1_robot
chr12	81472539	81472540	MACS_peak_3490	5.00	lobChIP_FOXA1_robot
chr12	81473626	81473627	MACS_peak_3491	4.00	lobChIP_FOXA1_robot
chr12	81478884	81478885	MACS_peak_3492	3.00	lobChIP_FOXA1_robot
chr12	81479791	81479792	MACS_peak_3493	3.00	lobChIP_FOXA1_robot
chr12	81480426	81480427	MACS_peak_3494	5.00	lobChIP_FOXA1_robot
chr12	81515005	81515006	MACS_peak_3495	4.00	lobChIP_FOXA1_robot
chr12	81535266	81535267	MACS_peak_3496	5.00	lobChIP_FOXA1_robot
chr12	81537120	81537121	MACS_peak_3497	26.00	lobChIP_FOXA1_robot
chr12	81540427	81540428	MACS_peak_3498	11.00	lobChIP_FOXA1_robot
chr12	81656505	81656506	MACS_peak_3499	4.00	lobChIP_FOXA1_robot
chr12	82322204	82322205	MACS_peak_3500	5.00	lobChIP_FOXA1_robot
chr12	86215748	86215749	MACS_peak_3501	9.00	lobChIP_FOXA1_robot
chr12	88806347	88806348	MACS_peak_3502	3.00	lobChIP_FOXA1_robot
chr12	88852296	88852297	MACS_peak_3503	4.00	lobChIP_FOXA1_robot
chr12	88947777	88947778	MACS_peak_3504	4.00	lobChIP_FOXA1_robot
chr12	88948465	88948466	MACS_peak_3505	4.00	lobChIP_FOXA1_robot
chr12	88956154	88956155	MACS_peak_3506	8.00	lobChIP_FOXA1_robot
chr12	89200139	89200140	MACS_peak_3507	5.00	lobChIP_FOXA1_robot
chr12	89298705	89298706	MACS_peak_3508	7.00	lobChIP_FOXA1_robot
chr12	89320161	89320162	MACS_peak_3509	42.00	lobChIP_FOXA1_robot
chr12	89325529	89325530	MACS_peak_3510	3.00	lobChIP_FOXA1_robot
chr12	89352146	89352147	MACS_peak_3511	12.00	lobChIP_FOXA1_robot
chr12	89586314	89586315	MACS_peak_3512	6.00	lobChIP_FOXA1_robot
chr12	89738004	89738005	MACS_peak_3513	6.00	lobChIP_FOXA1_robot
chr12	89746453	89746454	MACS_peak_3514	15.00	lobChIP_FOXA1_robot
chr12	89747804	89747805	MACS_peak_3515	5.00	lobChIP_FOXA1_robot
chr12	89773140	89773141	MACS_peak_3516	4.00	lobChIP_FOXA1_robot
chr12	89777115	89777116	MACS_peak_3517	19.00	lobChIP_FOXA1_robot
chr12	89784768	89784769	MACS_peak_3518	7.00	lobChIP_FOXA1_robot
chr12	89785284	89785285	MACS_peak_3519	5.00	lobChIP_FOXA1_robot
chr12	89785716	89785717	MACS_peak_3520	3.00	lobChIP_FOXA1_robot
chr12	89915944	89915945	MACS_peak_3521	5.00	lobChIP_FOXA1_robot
chr12	90019530	90019531	MACS_peak_3522	4.00	lobChIP_FOXA1_robot
chr12	90026756	90026757	MACS_peak_3523	65.00	lobChIP_FOXA1_robot
chr12	90891921	90891922	MACS_peak_3524	7.00	lobChIP_FOXA1_robot
chr12	91245816	91245817	MACS_peak_3525	5.00	lobChIP_FOXA1_robot
chr12	91253517	91253518	MACS_peak_3526	9.00	lobChIP_FOXA1_robot
chr12	92458929	92458930	MACS_peak_3527	6.00	lobChIP_FOXA1_robot
chr12	92465038	92465039	MACS_peak_3528	14.00	lobChIP_FOXA1_robot
chr12	92589482	92589483	MACS_peak_3529	3.00	lobChIP_FOXA1_robot
chr12	92897604	92897605	MACS_peak_3530	10.00	lobChIP_FOXA1_robot
chr12	93096697	93096698	MACS_peak_3531	5.00	lobChIP_FOXA1_robot
chr12	93339826	93339827	MACS_peak_3532	20.00	lobChIP_FOXA1_robot
chr12	93341741	93341742	MACS_peak_3533	4.00	lobChIP_FOXA1_robot
chr12	93367511	93367512	MACS_peak_3534	6.00	lobChIP_FOXA1_robot
chr12	93382617	93382618	MACS_peak_3535	5.00	lobChIP_FOXA1_robot
chr12	93399773	93399774	MACS_peak_3536	6.00	lobChIP_FOXA1_robot
chr12	93401508	93401509	MACS_peak_3537	6.00	lobChIP_FOXA1_robot
chr12	93410042	93410043	MACS_peak_3538	19.00	lobChIP_FOXA1_robot
chr12	93414275	93414276	MACS_peak_3539	22.00	lobChIP_FOXA1_robot
chr12	93433854	93433855	MACS_peak_3540	5.00	lobChIP_FOXA1_robot
chr12	93481309	93481310	MACS_peak_3541	46.00	lobChIP_FOXA1_robot
chr12	93496766	93496767	MACS_peak_3542	5.00	lobChIP_FOXA1_robot
chr12	93521664	93521665	MACS_peak_3543	5.00	lobChIP_FOXA1_robot
chr12	93529229	93529230	MACS_peak_3544	16.00	lobChIP_FOXA1_robot
chr12	93668051	93668052	MACS_peak_3545	13.00	lobChIP_FOXA1_robot
chr12	93686907	93686908	MACS_peak_3546	7.00	lobChIP_FOXA1_robot
chr12	93708703	93708704	MACS_peak_3547	6.00	lobChIP_FOXA1_robot
chr12	93712744	93712745	MACS_peak_3548	4.00	lobChIP_FOXA1_robot
chr12	93833946	93833947	MACS_peak_3549	7.00	lobChIP_FOXA1_robot
chr12	93935765	93935766	MACS_peak_3550	3.00	lobChIP_FOXA1_robot
chr12	93957786	93957787	MACS_peak_3551	15.00	lobChIP_FOXA1_robot
chr12	94071587	94071588	MACS_peak_3552	3.00	lobChIP_FOXA1_robot
chr12	94092448	94092449	MACS_peak_3553	4.00	lobChIP_FOXA1_robot
chr12	94093156	94093157	MACS_peak_3554	5.00	lobChIP_FOXA1_robot
chr12	94136369	94136370	MACS_peak_3555	5.00	lobChIP_FOXA1_robot
chr12	94145036	94145037	MACS_peak_3556	6.00	lobChIP_FOXA1_robot
chr12	94153293	94153294	MACS_peak_3557	3.00	lobChIP_FOXA1_robot
chr12	94153565	94153566	MACS_peak_3558	4.00	lobChIP_FOXA1_robot
chr12	94451708	94451709	MACS_peak_3559	3.00	lobChIP_FOXA1_robot
chr12	94474666	94474667	MACS_peak_3560	6.00	lobChIP_FOXA1_robot
chr12	94545457	94545458	MACS_peak_3561	4.00	lobChIP_FOXA1_robot
chr12	94546704	94546705	MACS_peak_3562	24.00	lobChIP_FOXA1_robot
chr12	94577712	94577713	MACS_peak_3563	7.00	lobChIP_FOXA1_robot
chr12	94594071	94594072	MACS_peak_3564	7.00	lobChIP_FOXA1_robot
chr12	94624343	94624344	MACS_peak_3565	12.00	lobChIP_FOXA1_robot
chr12	94699536	94699537	MACS_peak_3566	6.00	lobChIP_FOXA1_robot
chr12	94929423	94929424	MACS_peak_3567	7.00	lobChIP_FOXA1_robot
chr12	95324235	95324236	MACS_peak_3568	14.00	lobChIP_FOXA1_robot
chr12	95557793	95557794	MACS_peak_3569	4.00	lobChIP_FOXA1_robot
chr12	95712116	95712117	MACS_peak_3570	48.00	lobChIP_FOXA1_robot
chr12	95734105	95734106	MACS_peak_3571	14.00	lobChIP_FOXA1_robot
chr12	96028878	96028879	MACS_peak_3572	6.00	lobChIP_FOXA1_robot
chr12	96131905	96131906	MACS_peak_3573	6.00	lobChIP_FOXA1_robot
chr12	96156066	96156067	MACS_peak_3574	20.00	lobChIP_FOXA1_robot
chr12	96160338	96160339	MACS_peak_3575	8.00	lobChIP_FOXA1_robot
chr12	96196616	96196617	MACS_peak_3576	5.00	lobChIP_FOXA1_robot
chr12	96334715	96334716	MACS_peak_3577	4.00	lobChIP_FOXA1_robot
chr12	96343664	96343665	MACS_peak_3578	20.00	lobChIP_FOXA1_robot
chr12	96360178	96360179	MACS_peak_3579	23.00	lobChIP_FOXA1_robot
chr12	96368294	96368295	MACS_peak_3580	19.00	lobChIP_FOXA1_robot
chr12	96390173	96390174	MACS_peak_3581	13.00	lobChIP_FOXA1_robot
chr12	96442353	96442354	MACS_peak_3582	13.00	lobChIP_FOXA1_robot
chr12	96506581	96506582	MACS_peak_3583	4.00	lobChIP_FOXA1_robot
chr12	96657340	96657341	MACS_peak_3584	11.00	lobChIP_FOXA1_robot
chr12	96728438	96728439	MACS_peak_3585	5.00	lobChIP_FOXA1_robot
chr12	96795221	96795222	MACS_peak_3586	3.00	lobChIP_FOXA1_robot
chr12	97219519	97219520	MACS_peak_3587	4.00	lobChIP_FOXA1_robot
chr12	97296060	97296061	MACS_peak_3588	5.00	lobChIP_FOXA1_robot
chr12	97961807	97961808	MACS_peak_3589	21.00	lobChIP_FOXA1_robot
chr12	97962646	97962647	MACS_peak_3590	28.00	lobChIP_FOXA1_robot
chr12	97989305	97989306	MACS_peak_3591	4.00	lobChIP_FOXA1_robot
chr12	99047852	99047853	MACS_peak_3592	5.00	lobChIP_FOXA1_robot
chr12	99131074	99131075	MACS_peak_3593	8.00	lobChIP_FOXA1_robot
chr12	99606186	99606187	MACS_peak_3594	5.00	lobChIP_FOXA1_robot
chr12	100353498	100353499	MACS_peak_3595	4.00	lobChIP_FOXA1_robot
chr12	100535781	100535782	MACS_peak_3596	22.00	lobChIP_FOXA1_robot
chr12	100578590	100578591	MACS_peak_3597	6.00	lobChIP_FOXA1_robot
chr12	100607523	100607524	MACS_peak_3598	6.00	lobChIP_FOXA1_robot
chr12	100831299	100831300	MACS_peak_3599	6.00	lobChIP_FOXA1_robot
chr12	100858504	100858505	MACS_peak_3600	9.00	lobChIP_FOXA1_robot
chr12	100868473	100868474	MACS_peak_3601	5.00	lobChIP_FOXA1_robot
chr12	100869875	100869876	MACS_peak_3602	4.00	lobChIP_FOXA1_robot
chr12	100949742	100949743	MACS_peak_3603	5.00	lobChIP_FOXA1_robot
chr12	101993372	101993373	MACS_peak_3604	6.00	lobChIP_FOXA1_robot
chr12	101994064	101994065	MACS_peak_3605	12.00	lobChIP_FOXA1_robot
chr12	102002317	102002318	MACS_peak_3606	55.00	lobChIP_FOXA1_robot
chr12	102010164	102010165	MACS_peak_3607	5.00	lobChIP_FOXA1_robot
chr12	102012090	102012091	MACS_peak_3608	13.00	lobChIP_FOXA1_robot
chr12	102104311	102104312	MACS_peak_3609	5.00	lobChIP_FOXA1_robot
chr12	102162152	102162153	MACS_peak_3610	5.00	lobChIP_FOXA1_robot
chr12	102285379	102285380	MACS_peak_3611	7.00	lobChIP_FOXA1_robot
chr12	102303230	102303231	MACS_peak_3612	21.00	lobChIP_FOXA1_robot
chr12	102448260	102448261	MACS_peak_3613	7.00	lobChIP_FOXA1_robot
chr12	102453372	102453373	MACS_peak_3614	21.00	lobChIP_FOXA1_robot
chr12	103292873	103292874	MACS_peak_3615	30.00	lobChIP_FOXA1_robot
chr12	103314251	103314252	MACS_peak_3616	31.00	lobChIP_FOXA1_robot
chr12	103389270	103389271	MACS_peak_3617	3.00	lobChIP_FOXA1_robot
chr12	104066627	104066628	MACS_peak_3618	5.00	lobChIP_FOXA1_robot
chr12	104198931	104198932	MACS_peak_3619	4.00	lobChIP_FOXA1_robot
chr12	104232480	104232481	MACS_peak_3620	25.00	lobChIP_FOXA1_robot
chr12	104242063	104242064	MACS_peak_3621	6.00	lobChIP_FOXA1_robot
chr12	104249188	104249189	MACS_peak_3622	8.00	lobChIP_FOXA1_robot
chr12	104264245	104264246	MACS_peak_3623	3.00	lobChIP_FOXA1_robot
chr12	104525501	104525502	MACS_peak_3624	6.00	lobChIP_FOXA1_robot
chr12	105290177	105290178	MACS_peak_3625	10.00	lobChIP_FOXA1_robot
chr12	105559597	105559598	MACS_peak_3626	4.00	lobChIP_FOXA1_robot
chr12	105675635	105675636	MACS_peak_3627	6.00	lobChIP_FOXA1_robot
chr12	105711960	105711961	MACS_peak_3628	10.00	lobChIP_FOXA1_robot
chr12	106492636	106492637	MACS_peak_3629	11.00	lobChIP_FOXA1_robot
chr12	106514452	106514453	MACS_peak_3630	14.00	lobChIP_FOXA1_robot
chr12	106525521	106525522	MACS_peak_3631	7.00	lobChIP_FOXA1_robot
chr12	106668783	106668784	MACS_peak_3632	4.00	lobChIP_FOXA1_robot
chr12	106675027	106675028	MACS_peak_3633	13.00	lobChIP_FOXA1_robot
chr12	106676544	106676545	MACS_peak_3634	28.00	lobChIP_FOXA1_robot
chr12	106678530	106678531	MACS_peak_3635	8.00	lobChIP_FOXA1_robot
chr12	106682345	106682346	MACS_peak_3636	9.00	lobChIP_FOXA1_robot
chr12	106695603	106695604	MACS_peak_3637	4.00	lobChIP_FOXA1_robot
chr12	106752287	106752288	MACS_peak_3638	3.00	lobChIP_FOXA1_robot
chr12	106753602	106753603	MACS_peak_3639	6.00	lobChIP_FOXA1_robot
chr12	107038873	107038874	MACS_peak_3640	6.00	lobChIP_FOXA1_robot
chr12	107249080	107249081	MACS_peak_3641	17.00	lobChIP_FOXA1_robot
chr12	107368484	107368485	MACS_peak_3642	9.00	lobChIP_FOXA1_robot
chr12	107370166	107370167	MACS_peak_3643	6.00	lobChIP_FOXA1_robot
chr12	107746228	107746229	MACS_peak_3644	15.00	lobChIP_FOXA1_robot
chr12	107749517	107749518	MACS_peak_3645	3.00	lobChIP_FOXA1_robot
chr12	107758884	107758885	MACS_peak_3646	5.00	lobChIP_FOXA1_robot
chr12	107774147	107774148	MACS_peak_3647	8.00	lobChIP_FOXA1_robot
chr12	107900464	107900465	MACS_peak_3648	9.00	lobChIP_FOXA1_robot
chr12	107909047	107909048	MACS_peak_3649	7.00	lobChIP_FOXA1_robot
chr12	107918133	107918134	MACS_peak_3650	8.00	lobChIP_FOXA1_robot
chr12	108025579	108025580	MACS_peak_3651	3.00	lobChIP_FOXA1_robot
chr12	108707431	108707432	MACS_peak_3652	6.00	lobChIP_FOXA1_robot
chr12	109077877	109077878	MACS_peak_3653	10.00	lobChIP_FOXA1_robot
chr12	109085553	109085554	MACS_peak_3654	3.00	lobChIP_FOXA1_robot
chr12	109229803	109229804	MACS_peak_3655	6.00	lobChIP_FOXA1_robot
chr12	109635398	109635399	MACS_peak_3656	4.00	lobChIP_FOXA1_robot
chr12	109636146	109636147	MACS_peak_3657	5.00	lobChIP_FOXA1_robot
chr12	109715800	109715801	MACS_peak_3658	6.00	lobChIP_FOXA1_robot
chr12	110024902	110024903	MACS_peak_3659	9.00	lobChIP_FOXA1_robot
chr12	110026435	110026436	MACS_peak_3660	5.00	lobChIP_FOXA1_robot
chr12	110134155	110134156	MACS_peak_3661	4.00	lobChIP_FOXA1_robot
chr12	110256922	110256923	MACS_peak_3662	5.00	lobChIP_FOXA1_robot
chr12	110302295	110302296	MACS_peak_3663	9.00	lobChIP_FOXA1_robot
chr12	110386949	110386950	MACS_peak_3664	4.00	lobChIP_FOXA1_robot
chr12	110477103	110477104	MACS_peak_3665	13.00	lobChIP_FOXA1_robot
chr12	110498199	110498200	MACS_peak_3666	5.00	lobChIP_FOXA1_robot
chr12	110517581	110517582	MACS_peak_3667	5.00	lobChIP_FOXA1_robot
chr12	110529159	110529160	MACS_peak_3668	5.00	lobChIP_FOXA1_robot
chr12	110546855	110546856	MACS_peak_3669	5.00	lobChIP_FOXA1_robot
chr12	110547169	110547170	MACS_peak_3670	4.00	lobChIP_FOXA1_robot
chr12	110549405	110549406	MACS_peak_3671	11.00	lobChIP_FOXA1_robot
chr12	110556387	110556388	MACS_peak_3672	5.00	lobChIP_FOXA1_robot
chr12	110671893	110671894	MACS_peak_3673	3.00	lobChIP_FOXA1_robot
chr12	110790957	110790958	MACS_peak_3674	3.00	lobChIP_FOXA1_robot
chr12	111013279	111013280	MACS_peak_3675	4.00	lobChIP_FOXA1_robot
chr12	111021518	111021519	MACS_peak_3676	4.00	lobChIP_FOXA1_robot
chr12	111169414	111169415	MACS_peak_3677	8.00	lobChIP_FOXA1_robot
chr12	111841349	111841350	MACS_peak_3678	4.00	lobChIP_FOXA1_robot
chr12	112016375	112016376	MACS_peak_3679	9.00	lobChIP_FOXA1_robot
chr12	112034979	112034980	MACS_peak_3680	4.00	lobChIP_FOXA1_robot
chr12	112211541	112211542	MACS_peak_3681	7.00	lobChIP_FOXA1_robot
chr12	112723772	112723773	MACS_peak_3682	45.00	lobChIP_FOXA1_robot
chr12	112809084	112809085	MACS_peak_3683	3.00	lobChIP_FOXA1_robot
chr12	112904128	112904129	MACS_peak_3684	13.00	lobChIP_FOXA1_robot
chr12	113661078	113661079	MACS_peak_3685	9.00	lobChIP_FOXA1_robot
chr12	114204180	114204181	MACS_peak_3686	4.00	lobChIP_FOXA1_robot
chr12	115014420	115014421	MACS_peak_3687	6.00	lobChIP_FOXA1_robot
chr12	115046474	115046475	MACS_peak_3688	6.00	lobChIP_FOXA1_robot
chr12	115049479	115049480	MACS_peak_3689	21.00	lobChIP_FOXA1_robot
chr12	115094112	115094113	MACS_peak_3690	41.00	lobChIP_FOXA1_robot
chr12	115095070	115095071	MACS_peak_3691	11.00	lobChIP_FOXA1_robot
chr12	115095965	115095966	MACS_peak_3692	9.00	lobChIP_FOXA1_robot
chr12	115122158	115122159	MACS_peak_3693	7.00	lobChIP_FOXA1_robot
chr12	115237582	115237583	MACS_peak_3694	11.00	lobChIP_FOXA1_robot
chr12	115247408	115247409	MACS_peak_3695	3.00	lobChIP_FOXA1_robot
chr12	115251170	115251171	MACS_peak_3696	14.00	lobChIP_FOXA1_robot
chr12	115516251	115516252	MACS_peak_3697	5.00	lobChIP_FOXA1_robot
chr12	115527371	115527372	MACS_peak_3698	4.00	lobChIP_FOXA1_robot
chr12	115750703	115750704	MACS_peak_3699	3.00	lobChIP_FOXA1_robot
chr12	115859784	115859785	MACS_peak_3700	38.00	lobChIP_FOXA1_robot
chr12	115861501	115861502	MACS_peak_3701	31.00	lobChIP_FOXA1_robot
chr12	115864701	115864702	MACS_peak_3702	15.00	lobChIP_FOXA1_robot
chr12	115871195	115871196	MACS_peak_3703	4.00	lobChIP_FOXA1_robot
chr12	115945940	115945941	MACS_peak_3704	7.00	lobChIP_FOXA1_robot
chr12	116086465	116086466	MACS_peak_3705	6.00	lobChIP_FOXA1_robot
chr12	116201926	116201927	MACS_peak_3706	4.00	lobChIP_FOXA1_robot
chr12	116311630	116311631	MACS_peak_3707	4.00	lobChIP_FOXA1_robot
chr12	116571064	116571065	MACS_peak_3708	6.00	lobChIP_FOXA1_robot
chr12	116621890	116621891	MACS_peak_3709	3.00	lobChIP_FOXA1_robot
chr12	116714328	116714329	MACS_peak_3710	4.00	lobChIP_FOXA1_robot
chr12	116814320	116814321	MACS_peak_3711	6.00	lobChIP_FOXA1_robot
chr12	116967053	116967054	MACS_peak_3712	19.00	lobChIP_FOXA1_robot
chr12	117074923	117074924	MACS_peak_3713	56.00	lobChIP_FOXA1_robot
chr12	117172350	117172351	MACS_peak_3714	17.00	lobChIP_FOXA1_robot
chr12	117174280	117174281	MACS_peak_3715	7.00	lobChIP_FOXA1_robot
chr12	117486160	117486161	MACS_peak_3716	7.00	lobChIP_FOXA1_robot
chr12	117581164	117581165	MACS_peak_3717	7.00	lobChIP_FOXA1_robot
chr12	117956791	117956792	MACS_peak_3718	8.00	lobChIP_FOXA1_robot
chr12	118494311	118494312	MACS_peak_3719	9.00	lobChIP_FOXA1_robot
chr12	118501894	118501895	MACS_peak_3720	6.00	lobChIP_FOXA1_robot
chr12	118562460	118562461	MACS_peak_3721	15.00	lobChIP_FOXA1_robot
chr12	118576695	118576696	MACS_peak_3722	5.00	lobChIP_FOXA1_robot
chr12	119041623	119041624	MACS_peak_3723	9.00	lobChIP_FOXA1_robot
chr12	119152497	119152498	MACS_peak_3724	36.00	lobChIP_FOXA1_robot
chr12	120108094	120108095	MACS_peak_3725	8.00	lobChIP_FOXA1_robot
chr12	120121897	120121898	MACS_peak_3726	6.00	lobChIP_FOXA1_robot
chr12	120154548	120154549	MACS_peak_3727	6.00	lobChIP_FOXA1_robot
chr12	120245508	120245509	MACS_peak_3728	5.00	lobChIP_FOXA1_robot
chr12	120378914	120378915	MACS_peak_3729	7.00	lobChIP_FOXA1_robot
chr12	120479290	120479291	MACS_peak_3730	9.00	lobChIP_FOXA1_robot
chr12	120481690	120481691	MACS_peak_3731	4.00	lobChIP_FOXA1_robot
chr12	120493240	120493241	MACS_peak_3732	4.00	lobChIP_FOXA1_robot
chr12	120665687	120665688	MACS_peak_3733	7.00	lobChIP_FOXA1_robot
chr12	120729637	120729638	MACS_peak_3734	6.00	lobChIP_FOXA1_robot
chr12	120730631	120730632	MACS_peak_3735	6.00	lobChIP_FOXA1_robot
chr12	120755652	120755653	MACS_peak_3736	10.00	lobChIP_FOXA1_robot
chr12	120983680	120983681	MACS_peak_3737	4.00	lobChIP_FOXA1_robot
chr12	121263333	121263334	MACS_peak_3738	14.00	lobChIP_FOXA1_robot
chr12	121266921	121266922	MACS_peak_3739	3.00	lobChIP_FOXA1_robot
chr12	121365961	121365962	MACS_peak_3740	4.00	lobChIP_FOXA1_robot
chr12	121416360	121416361	MACS_peak_3741	6.00	lobChIP_FOXA1_robot
chr12	121418783	121418784	MACS_peak_3742	7.00	lobChIP_FOXA1_robot
chr12	121419594	121419595	MACS_peak_3743	5.00	lobChIP_FOXA1_robot
chr12	121464083	121464084	MACS_peak_3744	4.00	lobChIP_FOXA1_robot
chr12	121554789	121554790	MACS_peak_3745	6.00	lobChIP_FOXA1_robot
chr12	121564354	121564355	MACS_peak_3746	5.00	lobChIP_FOXA1_robot
chr12	121928715	121928716	MACS_peak_3747	5.00	lobChIP_FOXA1_robot
chr12	121989052	121989053	MACS_peak_3748	40.00	lobChIP_FOXA1_robot
chr12	122036777	122036778	MACS_peak_3749	4.00	lobChIP_FOXA1_robot
chr12	122298263	122298264	MACS_peak_3750	5.00	lobChIP_FOXA1_robot
chr12	122853184	122853185	MACS_peak_3751	11.00	lobChIP_FOXA1_robot
chr12	122884607	122884608	MACS_peak_3752	5.00	lobChIP_FOXA1_robot
chr12	122885220	122885221	MACS_peak_3753	7.00	lobChIP_FOXA1_robot
chr12	122916025	122916026	MACS_peak_3754	9.00	lobChIP_FOXA1_robot
chr12	122956111	122956112	MACS_peak_3755	12.00	lobChIP_FOXA1_robot
chr12	122973803	122973804	MACS_peak_3756	16.00	lobChIP_FOXA1_robot
chr12	123012637	123012638	MACS_peak_3757	5.00	lobChIP_FOXA1_robot
chr12	123163867	123163868	MACS_peak_3758	6.00	lobChIP_FOXA1_robot
chr12	123201457	123201458	MACS_peak_3759	4.00	lobChIP_FOXA1_robot
chr12	123383609	123383610	MACS_peak_3760	11.00	lobChIP_FOXA1_robot
chr12	123754780	123754781	MACS_peak_3761	9.00	lobChIP_FOXA1_robot
chr12	123930675	123930676	MACS_peak_3762	5.00	lobChIP_FOXA1_robot
chr12	123933214	123933215	MACS_peak_3763	13.00	lobChIP_FOXA1_robot
chr12	123942566	123942567	MACS_peak_3764	5.00	lobChIP_FOXA1_robot
chr12	123957211	123957212	MACS_peak_3765	5.00	lobChIP_FOXA1_robot
chr12	124351882	124351883	MACS_peak_3766	6.00	lobChIP_FOXA1_robot
chr12	124615429	124615430	MACS_peak_3767	22.00	lobChIP_FOXA1_robot
chr12	124763266	124763267	MACS_peak_3768	6.00	lobChIP_FOXA1_robot
chr12	125391430	125391431	MACS_peak_3769	10.00	lobChIP_FOXA1_robot
chr12	125401298	125401299	MACS_peak_3770	4.00	lobChIP_FOXA1_robot
chr12	126466335	126466336	MACS_peak_3771	7.00	lobChIP_FOXA1_robot
chr12	126706746	126706747	MACS_peak_3772	4.00	lobChIP_FOXA1_robot
chr12	129391149	129391150	MACS_peak_3774	4.00	lobChIP_FOXA1_robot
chr12	130528407	130528408	MACS_peak_3775	10.00	lobChIP_FOXA1_robot
chr12	131261310	131261311	MACS_peak_3776	5.00	lobChIP_FOXA1_robot
chr12	131347956	131347957	MACS_peak_3777	4.00	lobChIP_FOXA1_robot
chr12	131497069	131497070	MACS_peak_3778	7.00	lobChIP_FOXA1_robot
chr12	132049502	132049503	MACS_peak_3779	5.00	lobChIP_FOXA1_robot
chr12	132380995	132380996	MACS_peak_3780	4.00	lobChIP_FOXA1_robot
chr12	133047927	133047928	MACS_peak_3781	5.00	lobChIP_FOXA1_robot
chr12	133067969	133067970	MACS_peak_3782	4.00	lobChIP_FOXA1_robot
chr12	133277260	133277261	MACS_peak_3783	4.00	lobChIP_FOXA1_robot
chr13	20171237	20171238	MACS_peak_3784	23.00	lobChIP_FOXA1_robot
chr13	20178625	20178626	MACS_peak_3785	17.00	lobChIP_FOXA1_robot
chr13	20690582	20690583	MACS_peak_3786	7.00	lobChIP_FOXA1_robot
chr13	20737756	20737757	MACS_peak_3787	7.00	lobChIP_FOXA1_robot
chr13	20837673	20837674	MACS_peak_3788	5.00	lobChIP_FOXA1_robot
chr13	20862342	20862343	MACS_peak_3789	11.00	lobChIP_FOXA1_robot
chr13	21069705	21069706	MACS_peak_3790	5.00	lobChIP_FOXA1_robot
chr13	21080018	21080019	MACS_peak_3791	7.00	lobChIP_FOXA1_robot
chr13	21108628	21108629	MACS_peak_3792	16.00	lobChIP_FOXA1_robot
chr13	21289655	21289656	MACS_peak_3793	5.00	lobChIP_FOXA1_robot
chr13	21495882	21495883	MACS_peak_3794	6.00	lobChIP_FOXA1_robot
chr13	21514527	21514528	MACS_peak_3795	6.00	lobChIP_FOXA1_robot
chr13	21524772	21524773	MACS_peak_3796	4.00	lobChIP_FOXA1_robot
chr13	21527751	21527752	MACS_peak_3797	24.00	lobChIP_FOXA1_robot
chr13	21535283	21535284	MACS_peak_3798	3.00	lobChIP_FOXA1_robot
chr13	21588876	21588877	MACS_peak_3799	7.00	lobChIP_FOXA1_robot
chr13	21675418	21675419	MACS_peak_3800	6.00	lobChIP_FOXA1_robot
chr13	21856518	21856519	MACS_peak_3801	5.00	lobChIP_FOXA1_robot
chr13	21977286	21977287	MACS_peak_3802	7.00	lobChIP_FOXA1_robot
chr13	22101061	22101062	MACS_peak_3803	20.00	lobChIP_FOXA1_robot
chr13	22237619	22237620	MACS_peak_3804	5.00	lobChIP_FOXA1_robot
chr13	22271176	22271177	MACS_peak_3805	15.00	lobChIP_FOXA1_robot
chr13	22412115	22412116	MACS_peak_3806	6.00	lobChIP_FOXA1_robot
chr13	22993128	22993129	MACS_peak_3807	4.00	lobChIP_FOXA1_robot
chr13	23363092	23363093	MACS_peak_3808	4.00	lobChIP_FOXA1_robot
chr13	23368019	23368020	MACS_peak_3809	6.00	lobChIP_FOXA1_robot
chr13	24115520	24115521	MACS_peak_3810	4.00	lobChIP_FOXA1_robot
chr13	24126459	24126460	MACS_peak_3811	21.00	lobChIP_FOXA1_robot
chr13	24133424	24133425	MACS_peak_3812	6.00	lobChIP_FOXA1_robot
chr13	24144749	24144750	MACS_peak_3813	9.00	lobChIP_FOXA1_robot
chr13	24173802	24173803	MACS_peak_3814	7.00	lobChIP_FOXA1_robot
chr13	24203801	24203802	MACS_peak_3815	23.00	lobChIP_FOXA1_robot
chr13	24204978	24204979	MACS_peak_3816	19.00	lobChIP_FOXA1_robot
chr13	24228812	24228813	MACS_peak_3817	5.00	lobChIP_FOXA1_robot
chr13	24231033	24231034	MACS_peak_3818	5.00	lobChIP_FOXA1_robot
chr13	24293371	24293372	MACS_peak_3819	8.00	lobChIP_FOXA1_robot
chr13	24296937	24296938	MACS_peak_3820	4.00	lobChIP_FOXA1_robot
chr13	24309653	24309654	MACS_peak_3821	6.00	lobChIP_FOXA1_robot
chr13	24318036	24318037	MACS_peak_3822	6.00	lobChIP_FOXA1_robot
chr13	24327703	24327704	MACS_peak_3823	6.00	lobChIP_FOXA1_robot
chr13	24329400	24329401	MACS_peak_3824	6.00	lobChIP_FOXA1_robot
chr13	24353327	24353328	MACS_peak_3825	6.00	lobChIP_FOXA1_robot
chr13	24370860	24370861	MACS_peak_3826	11.00	lobChIP_FOXA1_robot
chr13	24544076	24544077	MACS_peak_3827	6.00	lobChIP_FOXA1_robot
chr13	24723108	24723109	MACS_peak_3828	3.00	lobChIP_FOXA1_robot
chr13	24727151	24727152	MACS_peak_3829	4.00	lobChIP_FOXA1_robot
chr13	24757014	24757015	MACS_peak_3830	6.00	lobChIP_FOXA1_robot
chr13	24758717	24758718	MACS_peak_3831	5.00	lobChIP_FOXA1_robot
chr13	25733340	25733341	MACS_peak_3832	5.00	lobChIP_FOXA1_robot
chr13	25869578	25869579	MACS_peak_3833	3.00	lobChIP_FOXA1_robot
chr13	26829961	26829962	MACS_peak_3834	4.00	lobChIP_FOXA1_robot
chr13	26970593	26970594	MACS_peak_3835	3.00	lobChIP_FOXA1_robot
chr13	27159401	27159402	MACS_peak_3836	4.00	lobChIP_FOXA1_robot
chr13	27536192	27536193	MACS_peak_3837	5.00	lobChIP_FOXA1_robot
chr13	27557513	27557514	MACS_peak_3838	6.00	lobChIP_FOXA1_robot
chr13	27565376	27565377	MACS_peak_3839	6.00	lobChIP_FOXA1_robot
chr13	27565896	27565897	MACS_peak_3840	3.00	lobChIP_FOXA1_robot
chr13	27597024	27597025	MACS_peak_3841	6.00	lobChIP_FOXA1_robot
chr13	27941826	27941827	MACS_peak_3842	9.00	lobChIP_FOXA1_robot
chr13	27947224	27947225	MACS_peak_3843	8.00	lobChIP_FOXA1_robot
chr13	28200180	28200181	MACS_peak_3844	10.00	lobChIP_FOXA1_robot
chr13	28211623	28211624	MACS_peak_3845	11.00	lobChIP_FOXA1_robot
chr13	28216354	28216355	MACS_peak_3846	6.00	lobChIP_FOXA1_robot
chr13	28284121	28284122	MACS_peak_3847	7.00	lobChIP_FOXA1_robot
chr13	28535047	28535048	MACS_peak_3848	35.00	lobChIP_FOXA1_robot
chr13	28719346	28719347	MACS_peak_3849	31.00	lobChIP_FOXA1_robot
chr13	29212253	29212254	MACS_peak_3850	7.00	lobChIP_FOXA1_robot
chr13	29327739	29327740	MACS_peak_3851	6.00	lobChIP_FOXA1_robot
chr13	29590866	29590867	MACS_peak_3852	4.00	lobChIP_FOXA1_robot
chr13	30145937	30145938	MACS_peak_3853	8.00	lobChIP_FOXA1_robot
chr13	30384531	30384532	MACS_peak_3854	5.00	lobChIP_FOXA1_robot
chr13	30498231	30498232	MACS_peak_3855	10.00	lobChIP_FOXA1_robot
chr13	30969855	30969856	MACS_peak_3856	10.00	lobChIP_FOXA1_robot
chr13	31242619	31242620	MACS_peak_3857	5.00	lobChIP_FOXA1_robot
chr13	31407302	31407303	MACS_peak_3858	5.00	lobChIP_FOXA1_robot
chr13	31425153	31425154	MACS_peak_3859	5.00	lobChIP_FOXA1_robot
chr13	31746935	31746936	MACS_peak_3860	6.00	lobChIP_FOXA1_robot
chr13	32497689	32497690	MACS_peak_3861	5.00	lobChIP_FOXA1_robot
chr13	32614170	32614171	MACS_peak_3862	5.00	lobChIP_FOXA1_robot
chr13	32633860	32633861	MACS_peak_3863	6.00	lobChIP_FOXA1_robot
chr13	32649718	32649719	MACS_peak_3864	4.00	lobChIP_FOXA1_robot
chr13	32654594	32654595	MACS_peak_3865	11.00	lobChIP_FOXA1_robot
chr13	32662189	32662190	MACS_peak_3866	16.00	lobChIP_FOXA1_robot
chr13	32713691	32713692	MACS_peak_3867	12.00	lobChIP_FOXA1_robot
chr13	32717263	32717264	MACS_peak_3868	8.00	lobChIP_FOXA1_robot
chr13	32769959	32769960	MACS_peak_3869	5.00	lobChIP_FOXA1_robot
chr13	32829970	32829971	MACS_peak_3870	5.00	lobChIP_FOXA1_robot
chr13	32831891	32831892	MACS_peak_3871	7.00	lobChIP_FOXA1_robot
chr13	32894912	32894913	MACS_peak_3872	4.00	lobChIP_FOXA1_robot
chr13	33614472	33614473	MACS_peak_3873	6.00	lobChIP_FOXA1_robot
chr13	33676163	33676164	MACS_peak_3874	9.00	lobChIP_FOXA1_robot
chr13	33690905	33690906	MACS_peak_3875	4.00	lobChIP_FOXA1_robot
chr13	33701426	33701427	MACS_peak_3876	7.00	lobChIP_FOXA1_robot
chr13	33742691	33742692	MACS_peak_3877	16.00	lobChIP_FOXA1_robot
chr13	33781217	33781218	MACS_peak_3878	5.00	lobChIP_FOXA1_robot
chr13	33787205	33787206	MACS_peak_3879	3.00	lobChIP_FOXA1_robot
chr13	33803349	33803350	MACS_peak_3880	5.00	lobChIP_FOXA1_robot
chr13	33833119	33833120	MACS_peak_3881	5.00	lobChIP_FOXA1_robot
chr13	33834604	33834605	MACS_peak_3882	21.00	lobChIP_FOXA1_robot
chr13	33954831	33954832	MACS_peak_3883	6.00	lobChIP_FOXA1_robot
chr13	34019450	34019451	MACS_peak_3884	9.00	lobChIP_FOXA1_robot
chr13	34036385	34036386	MACS_peak_3885	5.00	lobChIP_FOXA1_robot
chr13	34070212	34070213	MACS_peak_3886	7.00	lobChIP_FOXA1_robot
chr13	34111509	34111510	MACS_peak_3887	3.00	lobChIP_FOXA1_robot
chr13	34183300	34183301	MACS_peak_3888	4.00	lobChIP_FOXA1_robot
chr13	34185604	34185605	MACS_peak_3889	3.00	lobChIP_FOXA1_robot
chr13	34215033	34215034	MACS_peak_3890	5.00	lobChIP_FOXA1_robot
chr13	34217045	34217046	MACS_peak_3891	26.00	lobChIP_FOXA1_robot
chr13	34253560	34253561	MACS_peak_3892	4.00	lobChIP_FOXA1_robot
chr13	34446225	34446226	MACS_peak_3893	3.00	lobChIP_FOXA1_robot
chr13	34447885	34447886	MACS_peak_3894	6.00	lobChIP_FOXA1_robot
chr13	34531866	34531867	MACS_peak_3895	4.00	lobChIP_FOXA1_robot
chr13	34543094	34543095	MACS_peak_3896	12.00	lobChIP_FOXA1_robot
chr13	34567411	34567412	MACS_peak_3897	9.00	lobChIP_FOXA1_robot
chr13	34586169	34586170	MACS_peak_3898	15.00	lobChIP_FOXA1_robot
chr13	34594096	34594097	MACS_peak_3899	6.00	lobChIP_FOXA1_robot
chr13	34612757	34612758	MACS_peak_3900	10.00	lobChIP_FOXA1_robot
chr13	35858086	35858087	MACS_peak_3901	11.00	lobChIP_FOXA1_robot
chr13	35924189	35924190	MACS_peak_3902	3.00	lobChIP_FOXA1_robot
chr13	36539066	36539067	MACS_peak_3903	5.00	lobChIP_FOXA1_robot
chr13	36930344	36930345	MACS_peak_3904	6.00	lobChIP_FOXA1_robot
chr13	37457221	37457222	MACS_peak_3905	10.00	lobChIP_FOXA1_robot
chr13	40638161	40638162	MACS_peak_3906	5.00	lobChIP_FOXA1_robot
chr13	41050865	41050866	MACS_peak_3907	5.00	lobChIP_FOXA1_robot
chr13	41078340	41078341	MACS_peak_3908	15.00	lobChIP_FOXA1_robot
chr13	41110873	41110874	MACS_peak_3909	5.00	lobChIP_FOXA1_robot
chr13	41114738	41114739	MACS_peak_3910	16.00	lobChIP_FOXA1_robot
chr13	41360793	41360794	MACS_peak_3911	5.00	lobChIP_FOXA1_robot
chr13	41455191	41455192	MACS_peak_3912	10.00	lobChIP_FOXA1_robot
chr13	41469943	41469944	MACS_peak_3913	8.00	lobChIP_FOXA1_robot
chr13	41575909	41575910	MACS_peak_3914	4.00	lobChIP_FOXA1_robot
chr13	41579547	41579548	MACS_peak_3915	3.00	lobChIP_FOXA1_robot
chr13	41582662	41582663	MACS_peak_3916	9.00	lobChIP_FOXA1_robot
chr13	41592054	41592055	MACS_peak_3917	7.00	lobChIP_FOXA1_robot
chr13	41593102	41593103	MACS_peak_3918	4.00	lobChIP_FOXA1_robot
chr13	41834174	41834175	MACS_peak_3919	9.00	lobChIP_FOXA1_robot
chr13	41863938	41863939	MACS_peak_3920	7.00	lobChIP_FOXA1_robot
chr13	42137748	42137749	MACS_peak_3921	3.00	lobChIP_FOXA1_robot
chr13	42180831	42180832	MACS_peak_3922	4.00	lobChIP_FOXA1_robot
chr13	42188201	42188202	MACS_peak_3923	6.00	lobChIP_FOXA1_robot
chr13	42222642	42222643	MACS_peak_3924	43.00	lobChIP_FOXA1_robot
chr13	42519368	42519369	MACS_peak_3925	24.00	lobChIP_FOXA1_robot
chr13	42938133	42938134	MACS_peak_3926	4.00	lobChIP_FOXA1_robot
chr13	43332620	43332621	MACS_peak_3927	3.00	lobChIP_FOXA1_robot
chr13	43579080	43579081	MACS_peak_3928	4.00	lobChIP_FOXA1_robot
chr13	43629057	43629058	MACS_peak_3929	6.00	lobChIP_FOXA1_robot
chr13	43843428	43843429	MACS_peak_3930	3.00	lobChIP_FOXA1_robot
chr13	44766775	44766776	MACS_peak_3931	6.00	lobChIP_FOXA1_robot
chr13	44978629	44978630	MACS_peak_3932	19.00	lobChIP_FOXA1_robot
chr13	45022724	45022725	MACS_peak_3933	6.00	lobChIP_FOXA1_robot
chr13	45024319	45024320	MACS_peak_3934	5.00	lobChIP_FOXA1_robot
chr13	45389879	45389880	MACS_peak_3935	4.00	lobChIP_FOXA1_robot
chr13	45428490	45428491	MACS_peak_3936	8.00	lobChIP_FOXA1_robot
chr13	46008922	46008923	MACS_peak_3937	7.00	lobChIP_FOXA1_robot
chr13	46015028	46015029	MACS_peak_3938	6.00	lobChIP_FOXA1_robot
chr13	46102156	46102157	MACS_peak_3939	12.00	lobChIP_FOXA1_robot
chr13	46605310	46605311	MACS_peak_3940	3.00	lobChIP_FOXA1_robot
chr13	46619632	46619633	MACS_peak_3941	4.00	lobChIP_FOXA1_robot
chr13	46671364	46671365	MACS_peak_3942	4.00	lobChIP_FOXA1_robot
chr13	46679206	46679207	MACS_peak_3943	11.00	lobChIP_FOXA1_robot
chr13	46717459	46717460	MACS_peak_3944	28.00	lobChIP_FOXA1_robot
chr13	47130038	47130039	MACS_peak_3945	4.00	lobChIP_FOXA1_robot
chr13	47196251	47196252	MACS_peak_3946	4.00	lobChIP_FOXA1_robot
chr13	47234031	47234032	MACS_peak_3947	5.00	lobChIP_FOXA1_robot
chr13	47240304	47240305	MACS_peak_3948	22.00	lobChIP_FOXA1_robot
chr13	47258860	47258861	MACS_peak_3949	4.00	lobChIP_FOXA1_robot
chr13	47262331	47262332	MACS_peak_3950	3.00	lobChIP_FOXA1_robot
chr13	48670824	48670825	MACS_peak_3951	6.00	lobChIP_FOXA1_robot
chr13	48685765	48685766	MACS_peak_3952	6.00	lobChIP_FOXA1_robot
chr13	48775331	48775332	MACS_peak_3953	5.00	lobChIP_FOXA1_robot
chr13	48816965	48816966	MACS_peak_3954	5.00	lobChIP_FOXA1_robot
chr13	48828333	48828334	MACS_peak_3955	3.00	lobChIP_FOXA1_robot
chr13	49355493	49355494	MACS_peak_3956	5.00	lobChIP_FOXA1_robot
chr13	49882837	49882838	MACS_peak_3957	3.00	lobChIP_FOXA1_robot
chr13	50039388	50039389	MACS_peak_3958	16.00	lobChIP_FOXA1_robot
chr13	50040637	50040638	MACS_peak_3959	4.00	lobChIP_FOXA1_robot
chr13	50161789	50161790	MACS_peak_3960	14.00	lobChIP_FOXA1_robot
chr13	50264562	50264563	MACS_peak_3961	5.00	lobChIP_FOXA1_robot
chr13	50313799	50313800	MACS_peak_3962	8.00	lobChIP_FOXA1_robot
chr13	50315219	50315220	MACS_peak_3963	5.00	lobChIP_FOXA1_robot
chr13	50380346	50380347	MACS_peak_3964	7.00	lobChIP_FOXA1_robot
chr13	50385155	50385156	MACS_peak_3965	10.00	lobChIP_FOXA1_robot
chr13	50430272	50430273	MACS_peak_3966	6.00	lobChIP_FOXA1_robot
chr13	50432825	50432826	MACS_peak_3967	41.00	lobChIP_FOXA1_robot
chr13	50699819	50699820	MACS_peak_3968	5.00	lobChIP_FOXA1_robot
chr13	50700653	50700654	MACS_peak_3969	11.00	lobChIP_FOXA1_robot
chr13	50761762	50761763	MACS_peak_3970	8.00	lobChIP_FOXA1_robot
chr13	50815810	50815811	MACS_peak_3971	7.00	lobChIP_FOXA1_robot
chr13	50818690	50818691	MACS_peak_3972	3.00	lobChIP_FOXA1_robot
chr13	50819375	50819376	MACS_peak_3973	17.00	lobChIP_FOXA1_robot
chr13	50857335	50857336	MACS_peak_3974	9.00	lobChIP_FOXA1_robot
chr13	50894613	50894614	MACS_peak_3975	7.00	lobChIP_FOXA1_robot
chr13	50940474	50940475	MACS_peak_3976	10.00	lobChIP_FOXA1_robot
chr13	51246387	51246388	MACS_peak_3977	6.00	lobChIP_FOXA1_robot
chr13	51514853	51514854	MACS_peak_3978	5.00	lobChIP_FOXA1_robot
chr13	51697124	51697125	MACS_peak_3979	10.00	lobChIP_FOXA1_robot
chr13	51992594	51992595	MACS_peak_3980	4.00	lobChIP_FOXA1_robot
chr13	52098610	52098611	MACS_peak_3981	15.00	lobChIP_FOXA1_robot
chr13	52194845	52194846	MACS_peak_3982	5.00	lobChIP_FOXA1_robot
chr13	52210724	52210725	MACS_peak_3983	21.00	lobChIP_FOXA1_robot
chr13	52243000	52243001	MACS_peak_3984	3.00	lobChIP_FOXA1_robot
chr13	52263878	52263879	MACS_peak_3985	6.00	lobChIP_FOXA1_robot
chr13	52431324	52431325	MACS_peak_3986	3.00	lobChIP_FOXA1_robot
chr13	52433313	52433314	MACS_peak_3987	6.00	lobChIP_FOXA1_robot
chr13	52553321	52553322	MACS_peak_3988	5.00	lobChIP_FOXA1_robot
chr13	52563260	52563261	MACS_peak_3989	5.00	lobChIP_FOXA1_robot
chr13	52572413	52572414	MACS_peak_3990	6.00	lobChIP_FOXA1_robot
chr13	52599822	52599823	MACS_peak_3991	21.00	lobChIP_FOXA1_robot
chr13	52737009	52737010	MACS_peak_3992	7.00	lobChIP_FOXA1_robot
chr13	52912368	52912369	MACS_peak_3993	8.00	lobChIP_FOXA1_robot
chr13	53002619	53002620	MACS_peak_3994	4.00	lobChIP_FOXA1_robot
chr13	53029100	53029101	MACS_peak_3995	3.00	lobChIP_FOXA1_robot
chr13	53176667	53176668	MACS_peak_3996	5.00	lobChIP_FOXA1_robot
chr13	55031905	55031906	MACS_peak_3997	3.00	lobChIP_FOXA1_robot
chr13	57291856	57291857	MACS_peak_3998	5.00	lobChIP_FOXA1_robot
chr13	59026554	59026555	MACS_peak_3999	8.00	lobChIP_FOXA1_robot
chr13	60265596	60265597	MACS_peak_4000	3.00	lobChIP_FOXA1_robot
chr13	60265955	60265956	MACS_peak_4001	11.00	lobChIP_FOXA1_robot
chr13	62239680	62239681	MACS_peak_4002	24.00	lobChIP_FOXA1_robot
chr13	62518850	62518851	MACS_peak_4003	5.00	lobChIP_FOXA1_robot
chr13	62536570	62536571	MACS_peak_4004	4.00	lobChIP_FOXA1_robot
chr13	63318978	63318979	MACS_peak_4005	3.00	lobChIP_FOXA1_robot
chr13	63634228	63634229	MACS_peak_4006	4.00	lobChIP_FOXA1_robot
chr13	65599006	65599007	MACS_peak_4007	5.00	lobChIP_FOXA1_robot
chr13	65652915	65652916	MACS_peak_4008	4.00	lobChIP_FOXA1_robot
chr13	66561263	66561264	MACS_peak_4009	4.00	lobChIP_FOXA1_robot
chr13	67632264	67632265	MACS_peak_4010	5.00	lobChIP_FOXA1_robot
chr13	68293355	68293356	MACS_peak_4011	4.00	lobChIP_FOXA1_robot
chr13	68943680	68943681	MACS_peak_4012	4.00	lobChIP_FOXA1_robot
chr13	68952626	68952627	MACS_peak_4013	4.00	lobChIP_FOXA1_robot
chr13	68954930	68954931	MACS_peak_4014	28.00	lobChIP_FOXA1_robot
chr13	70294585	70294586	MACS_peak_4015	4.00	lobChIP_FOXA1_robot
chr13	70780979	70780980	MACS_peak_4016	10.00	lobChIP_FOXA1_robot
chr13	71463517	71463518	MACS_peak_4017	5.00	lobChIP_FOXA1_robot
chr13	71464558	71464559	MACS_peak_4018	19.00	lobChIP_FOXA1_robot
chr13	71605211	71605212	MACS_peak_4019	7.00	lobChIP_FOXA1_robot
chr13	71621830	71621831	MACS_peak_4020	6.00	lobChIP_FOXA1_robot
chr13	72057635	72057636	MACS_peak_4021	5.00	lobChIP_FOXA1_robot
chr13	72136845	72136846	MACS_peak_4022	4.00	lobChIP_FOXA1_robot
chr13	72395238	72395239	MACS_peak_4023	4.00	lobChIP_FOXA1_robot
chr13	73119430	73119431	MACS_peak_4024	10.00	lobChIP_FOXA1_robot
chr13	73222925	73222926	MACS_peak_4025	4.00	lobChIP_FOXA1_robot
chr13	73366921	73366922	MACS_peak_4026	4.00	lobChIP_FOXA1_robot
chr13	73713542	73713543	MACS_peak_4027	5.00	lobChIP_FOXA1_robot
chr13	73784471	73784472	MACS_peak_4028	7.00	lobChIP_FOXA1_robot
chr13	74025768	74025769	MACS_peak_4029	5.00	lobChIP_FOXA1_robot
chr13	74353005	74353006	MACS_peak_4030	5.00	lobChIP_FOXA1_robot
chr13	74599186	74599187	MACS_peak_4031	5.00	lobChIP_FOXA1_robot
chr13	74801599	74801600	MACS_peak_4032	4.00	lobChIP_FOXA1_robot
chr13	75876350	75876351	MACS_peak_4033	8.00	lobChIP_FOXA1_robot
chr13	75914215	75914216	MACS_peak_4034	41.00	lobChIP_FOXA1_robot
chr13	75951391	75951392	MACS_peak_4035	5.00	lobChIP_FOXA1_robot
chr13	75960132	75960133	MACS_peak_4036	4.00	lobChIP_FOXA1_robot
chr13	75975158	75975159	MACS_peak_4037	10.00	lobChIP_FOXA1_robot
chr13	76006177	76006178	MACS_peak_4038	5.00	lobChIP_FOXA1_robot
chr13	76033433	76033434	MACS_peak_4039	10.00	lobChIP_FOXA1_robot
chr13	76041356	76041357	MACS_peak_4040	10.00	lobChIP_FOXA1_robot
chr13	76052984	76052985	MACS_peak_4041	9.00	lobChIP_FOXA1_robot
chr13	76321094	76321095	MACS_peak_4042	4.00	lobChIP_FOXA1_robot
chr13	76334790	76334791	MACS_peak_4043	9.00	lobChIP_FOXA1_robot
chr13	76336444	76336445	MACS_peak_4044	6.00	lobChIP_FOXA1_robot
chr13	76366949	76366950	MACS_peak_4045	4.00	lobChIP_FOXA1_robot
chr13	77864187	77864188	MACS_peak_4046	13.00	lobChIP_FOXA1_robot
chr13	77880753	77880754	MACS_peak_4047	7.00	lobChIP_FOXA1_robot
chr13	79759907	79759908	MACS_peak_4048	5.00	lobChIP_FOXA1_robot
chr13	79845218	79845219	MACS_peak_4049	3.00	lobChIP_FOXA1_robot
chr13	79968175	79968176	MACS_peak_4050	6.00	lobChIP_FOXA1_robot
chr13	80218737	80218738	MACS_peak_4051	5.00	lobChIP_FOXA1_robot
chr13	81456359	81456360	MACS_peak_4052	5.00	lobChIP_FOXA1_robot
chr13	81680688	81680689	MACS_peak_4053	5.00	lobChIP_FOXA1_robot
chr13	82067472	82067473	MACS_peak_4054	4.00	lobChIP_FOXA1_robot
chr13	82448522	82448523	MACS_peak_4055	145.00	lobChIP_FOXA1_robot
chr13	82575841	82575842	MACS_peak_4056	3.00	lobChIP_FOXA1_robot
chr13	83081150	83081151	MACS_peak_4057	6.00	lobChIP_FOXA1_robot
chr13	85568835	85568836	MACS_peak_4058	5.00	lobChIP_FOXA1_robot
chr13	85945556	85945557	MACS_peak_4059	5.00	lobChIP_FOXA1_robot
chr13	85947670	85947671	MACS_peak_4060	7.00	lobChIP_FOXA1_robot
chr13	87089292	87089293	MACS_peak_4061	4.00	lobChIP_FOXA1_robot
chr13	87638739	87638740	MACS_peak_4062	5.00	lobChIP_FOXA1_robot
chr13	89591786	89591787	MACS_peak_4063	4.00	lobChIP_FOXA1_robot
chr13	89714161	89714162	MACS_peak_4064	4.00	lobChIP_FOXA1_robot
chr13	89732173	89732174	MACS_peak_4065	3.00	lobChIP_FOXA1_robot
chr13	90167074	90167075	MACS_peak_4066	7.00	lobChIP_FOXA1_robot
chr13	90207218	90207219	MACS_peak_4067	5.00	lobChIP_FOXA1_robot
chr13	90552204	90552205	MACS_peak_4068	13.00	lobChIP_FOXA1_robot
chr13	90723677	90723678	MACS_peak_4069	3.00	lobChIP_FOXA1_robot
chr13	91519072	91519073	MACS_peak_4070	16.00	lobChIP_FOXA1_robot
chr13	91519749	91519750	MACS_peak_4071	5.00	lobChIP_FOXA1_robot
chr13	92014406	92014407	MACS_peak_4072	13.00	lobChIP_FOXA1_robot
chr13	92015374	92015375	MACS_peak_4073	4.00	lobChIP_FOXA1_robot
chr13	92706954	92706955	MACS_peak_4074	9.00	lobChIP_FOXA1_robot
chr13	92724110	92724111	MACS_peak_4075	4.00	lobChIP_FOXA1_robot
chr13	93854649	93854650	MACS_peak_4076	17.00	lobChIP_FOXA1_robot
chr13	93877014	93877015	MACS_peak_4077	15.00	lobChIP_FOXA1_robot
chr13	93901729	93901730	MACS_peak_4078	3.00	lobChIP_FOXA1_robot
chr13	93913129	93913130	MACS_peak_4079	5.00	lobChIP_FOXA1_robot
chr13	93923570	93923571	MACS_peak_4080	6.00	lobChIP_FOXA1_robot
chr13	94392581	94392582	MACS_peak_4081	4.00	lobChIP_FOXA1_robot
chr13	94419710	94419711	MACS_peak_4082	5.00	lobChIP_FOXA1_robot
chr13	94610469	94610470	MACS_peak_4083	5.00	lobChIP_FOXA1_robot
chr13	94615979	94615980	MACS_peak_4084	15.00	lobChIP_FOXA1_robot
chr13	94618732	94618733	MACS_peak_4085	14.00	lobChIP_FOXA1_robot
chr13	94625330	94625331	MACS_peak_4086	4.00	lobChIP_FOXA1_robot
chr13	94829720	94829721	MACS_peak_4087	4.00	lobChIP_FOXA1_robot
chr13	94831013	94831014	MACS_peak_4088	6.00	lobChIP_FOXA1_robot
chr13	94915589	94915590	MACS_peak_4089	7.00	lobChIP_FOXA1_robot
chr13	94944627	94944628	MACS_peak_4090	12.00	lobChIP_FOXA1_robot
chr13	94956029	94956030	MACS_peak_4091	5.00	lobChIP_FOXA1_robot
chr13	94957456	94957457	MACS_peak_4092	6.00	lobChIP_FOXA1_robot
chr13	95696883	95696884	MACS_peak_4093	6.00	lobChIP_FOXA1_robot
chr13	95776263	95776264	MACS_peak_4094	5.00	lobChIP_FOXA1_robot
chr13	95790774	95790775	MACS_peak_4095	4.00	lobChIP_FOXA1_robot
chr13	95813076	95813077	MACS_peak_4096	12.00	lobChIP_FOXA1_robot
chr13	95927620	95927621	MACS_peak_4097	5.00	lobChIP_FOXA1_robot
chr13	95950116	95950117	MACS_peak_4098	14.00	lobChIP_FOXA1_robot
chr13	95966945	95966946	MACS_peak_4099	5.00	lobChIP_FOXA1_robot
chr13	96146576	96146577	MACS_peak_4100	8.00	lobChIP_FOXA1_robot
chr13	96187772	96187773	MACS_peak_4101	8.00	lobChIP_FOXA1_robot
chr13	96624962	96624963	MACS_peak_4102	6.00	lobChIP_FOXA1_robot
chr13	97578302	97578303	MACS_peak_4103	23.00	lobChIP_FOXA1_robot
chr13	97793904	97793905	MACS_peak_4104	4.00	lobChIP_FOXA1_robot
chr13	97818274	97818275	MACS_peak_4105	7.00	lobChIP_FOXA1_robot
chr13	97875388	97875389	MACS_peak_4106	6.00	lobChIP_FOXA1_robot
chr13	97896861	97896862	MACS_peak_4107	9.00	lobChIP_FOXA1_robot
chr13	97910570	97910571	MACS_peak_4108	10.00	lobChIP_FOXA1_robot
chr13	97927238	97927239	MACS_peak_4109	21.00	lobChIP_FOXA1_robot
chr13	97960693	97960694	MACS_peak_4110	10.00	lobChIP_FOXA1_robot
chr13	98079152	98079153	MACS_peak_4111	4.00	lobChIP_FOXA1_robot
chr13	98081979	98081980	MACS_peak_4112	4.00	lobChIP_FOXA1_robot
chr13	98105140	98105141	MACS_peak_4113	5.00	lobChIP_FOXA1_robot
chr13	98143892	98143893	MACS_peak_4114	7.00	lobChIP_FOXA1_robot
chr13	98174641	98174642	MACS_peak_4115	7.00	lobChIP_FOXA1_robot
chr13	98702417	98702418	MACS_peak_4116	14.00	lobChIP_FOXA1_robot
chr13	98813820	98813821	MACS_peak_4117	24.00	lobChIP_FOXA1_robot
chr13	98825659	98825660	MACS_peak_4118	3.00	lobChIP_FOXA1_robot
chr13	98851994	98851995	MACS_peak_4119	8.00	lobChIP_FOXA1_robot
chr13	98896219	98896220	MACS_peak_4120	4.00	lobChIP_FOXA1_robot
chr13	98898834	98898835	MACS_peak_4121	7.00	lobChIP_FOXA1_robot
chr13	98919549	98919550	MACS_peak_4122	12.00	lobChIP_FOXA1_robot
chr13	98921846	98921847	MACS_peak_4123	7.00	lobChIP_FOXA1_robot
chr13	98925587	98925588	MACS_peak_4124	5.00	lobChIP_FOXA1_robot
chr13	98926876	98926877	MACS_peak_4125	5.00	lobChIP_FOXA1_robot
chr13	98944322	98944323	MACS_peak_4126	5.00	lobChIP_FOXA1_robot
chr13	98956266	98956267	MACS_peak_4127	5.00	lobChIP_FOXA1_robot
chr13	99142593	99142594	MACS_peak_4128	8.00	lobChIP_FOXA1_robot
chr13	99221956	99221957	MACS_peak_4129	9.00	lobChIP_FOXA1_robot
chr13	99636601	99636602	MACS_peak_4130	3.00	lobChIP_FOXA1_robot
chr13	100149124	100149125	MACS_peak_4131	4.00	lobChIP_FOXA1_robot
chr13	100210924	100210925	MACS_peak_4132	5.00	lobChIP_FOXA1_robot
chr13	100593643	100593644	MACS_peak_4133	8.00	lobChIP_FOXA1_robot
chr13	100608918	100608919	MACS_peak_4134	7.00	lobChIP_FOXA1_robot
chr13	100611617	100611618	MACS_peak_4135	6.00	lobChIP_FOXA1_robot
chr13	100630048	100630049	MACS_peak_4136	8.00	lobChIP_FOXA1_robot
chr13	100692169	100692170	MACS_peak_4137	3.00	lobChIP_FOXA1_robot
chr13	100887204	100887205	MACS_peak_4138	6.00	lobChIP_FOXA1_robot
chr13	100942914	100942915	MACS_peak_4139	6.00	lobChIP_FOXA1_robot
chr13	100966972	100966973	MACS_peak_4140	7.00	lobChIP_FOXA1_robot
chr13	101386780	101386781	MACS_peak_4141	57.00	lobChIP_FOXA1_robot
chr13	101706746	101706747	MACS_peak_4142	4.00	lobChIP_FOXA1_robot
chr13	102229468	102229469	MACS_peak_4143	5.00	lobChIP_FOXA1_robot
chr13	102231836	102231837	MACS_peak_4144	4.00	lobChIP_FOXA1_robot
chr13	102528508	102528509	MACS_peak_4145	3.00	lobChIP_FOXA1_robot
chr13	103210137	103210138	MACS_peak_4146	5.00	lobChIP_FOXA1_robot
chr13	103250259	103250260	MACS_peak_4147	4.00	lobChIP_FOXA1_robot
chr13	103422879	103422880	MACS_peak_4148	5.00	lobChIP_FOXA1_robot
chr13	103423658	103423659	MACS_peak_4149	4.00	lobChIP_FOXA1_robot
chr13	103483148	103483149	MACS_peak_4150	3.00	lobChIP_FOXA1_robot
chr13	103552872	103552873	MACS_peak_4151	9.00	lobChIP_FOXA1_robot
chr13	107552007	107552008	MACS_peak_4152	6.00	lobChIP_FOXA1_robot
chr13	107597930	107597931	MACS_peak_4153	5.00	lobChIP_FOXA1_robot
chr13	107844832	107844833	MACS_peak_4154	12.00	lobChIP_FOXA1_robot
chr13	108452995	108452996	MACS_peak_4155	3.00	lobChIP_FOXA1_robot
chr13	108807153	108807154	MACS_peak_4156	12.00	lobChIP_FOXA1_robot
chr13	108882928	108882929	MACS_peak_4157	24.00	lobChIP_FOXA1_robot
chr13	109568376	109568377	MACS_peak_4158	4.00	lobChIP_FOXA1_robot
chr13	109675182	109675183	MACS_peak_4159	6.00	lobChIP_FOXA1_robot
chr13	109962203	109962204	MACS_peak_4160	3.00	lobChIP_FOXA1_robot
chr13	111142920	111142921	MACS_peak_4161	6.00	lobChIP_FOXA1_robot
chr13	111208281	111208282	MACS_peak_4162	6.00	lobChIP_FOXA1_robot
chr13	111229384	111229385	MACS_peak_4163	5.00	lobChIP_FOXA1_robot
chr13	111335969	111335970	MACS_peak_4164	6.00	lobChIP_FOXA1_robot
chr13	111568881	111568882	MACS_peak_4165	4.00	lobChIP_FOXA1_robot
chr13	111824030	111824031	MACS_peak_4166	4.00	lobChIP_FOXA1_robot
chr13	113365853	113365854	MACS_peak_4167	3.00	lobChIP_FOXA1_robot
chr13	113411599	113411600	MACS_peak_4168	19.00	lobChIP_FOXA1_robot
chr13	113606044	113606045	MACS_peak_4169	5.00	lobChIP_FOXA1_robot
chr13	113607451	113607452	MACS_peak_4170	4.00	lobChIP_FOXA1_robot
chr13	113760137	113760138	MACS_peak_4171	10.00	lobChIP_FOXA1_robot
chr13	113793575	113793576	MACS_peak_4172	15.00	lobChIP_FOXA1_robot
chr13	113794075	113794076	MACS_peak_4173	12.00	lobChIP_FOXA1_robot
chr13	113824471	113824472	MACS_peak_4174	8.00	lobChIP_FOXA1_robot
chr13	113838370	113838371	MACS_peak_4175	8.00	lobChIP_FOXA1_robot
chr13	114034435	114034436	MACS_peak_4176	5.00	lobChIP_FOXA1_robot
chr13	114147803	114147804	MACS_peak_4177	11.00	lobChIP_FOXA1_robot
chr14	20844592	20844593	MACS_peak_4180	4.00	lobChIP_FOXA1_robot
chr14	20886184	20886185	MACS_peak_4181	5.00	lobChIP_FOXA1_robot
chr14	20886860	20886861	MACS_peak_4182	5.00	lobChIP_FOXA1_robot
chr14	20940735	20940736	MACS_peak_4183	3.00	lobChIP_FOXA1_robot
chr14	21075211	21075212	MACS_peak_4184	4.00	lobChIP_FOXA1_robot
chr14	21081732	21081733	MACS_peak_4185	7.00	lobChIP_FOXA1_robot
chr14	21082723	21082724	MACS_peak_4186	5.00	lobChIP_FOXA1_robot
chr14	21099490	21099491	MACS_peak_4187	5.00	lobChIP_FOXA1_robot
chr14	21140337	21140338	MACS_peak_4188	8.00	lobChIP_FOXA1_robot
chr14	21154783	21154784	MACS_peak_4189	19.00	lobChIP_FOXA1_robot
chr14	21155803	21155804	MACS_peak_4190	27.00	lobChIP_FOXA1_robot
chr14	21275913	21275914	MACS_peak_4191	3.00	lobChIP_FOXA1_robot
chr14	21516970	21516971	MACS_peak_4192	4.00	lobChIP_FOXA1_robot
chr14	21522809	21522810	MACS_peak_4193	21.00	lobChIP_FOXA1_robot
chr14	21524220	21524221	MACS_peak_4194	6.00	lobChIP_FOXA1_robot
chr14	21530481	21530482	MACS_peak_4195	4.00	lobChIP_FOXA1_robot
chr14	21552384	21552385	MACS_peak_4196	5.00	lobChIP_FOXA1_robot
chr14	21732908	21732909	MACS_peak_4197	3.00	lobChIP_FOXA1_robot
chr14	21734590	21734591	MACS_peak_4198	3.00	lobChIP_FOXA1_robot
chr14	21771010	21771011	MACS_peak_4199	4.00	lobChIP_FOXA1_robot
chr14	21776575	21776576	MACS_peak_4200	5.00	lobChIP_FOXA1_robot
chr14	21906046	21906047	MACS_peak_4201	4.00	lobChIP_FOXA1_robot
chr14	21924904	21924905	MACS_peak_4202	4.00	lobChIP_FOXA1_robot
chr14	21944022	21944023	MACS_peak_4203	3.00	lobChIP_FOXA1_robot
chr14	21997254	21997255	MACS_peak_4204	5.00	lobChIP_FOXA1_robot
chr14	22025757	22025758	MACS_peak_4205	17.00	lobChIP_FOXA1_robot
chr14	22175152	22175153	MACS_peak_4206	4.00	lobChIP_FOXA1_robot
chr14	22179783	22179784	MACS_peak_4207	7.00	lobChIP_FOXA1_robot
chr14	22213350	22213351	MACS_peak_4208	3.00	lobChIP_FOXA1_robot
chr14	22382136	22382137	MACS_peak_4209	5.00	lobChIP_FOXA1_robot
chr14	22515557	22515558	MACS_peak_4210	4.00	lobChIP_FOXA1_robot
chr14	22703823	22703824	MACS_peak_4211	5.00	lobChIP_FOXA1_robot
chr14	22741263	22741264	MACS_peak_4212	4.00	lobChIP_FOXA1_robot
chr14	22764766	22764767	MACS_peak_4213	25.00	lobChIP_FOXA1_robot
chr14	22802609	22802610	MACS_peak_4214	25.00	lobChIP_FOXA1_robot
chr14	22805315	22805316	MACS_peak_4215	3.00	lobChIP_FOXA1_robot
chr14	22906708	22906709	MACS_peak_4216	4.00	lobChIP_FOXA1_robot
chr14	23030231	23030232	MACS_peak_4217	11.00	lobChIP_FOXA1_robot
chr14	23039483	23039484	MACS_peak_4218	4.00	lobChIP_FOXA1_robot
chr14	23041689	23041690	MACS_peak_4219	13.00	lobChIP_FOXA1_robot
chr14	23067130	23067131	MACS_peak_4220	10.00	lobChIP_FOXA1_robot
chr14	23075507	23075508	MACS_peak_4221	4.00	lobChIP_FOXA1_robot
chr14	23122429	23122430	MACS_peak_4222	5.00	lobChIP_FOXA1_robot
chr14	23168760	23168761	MACS_peak_4223	8.00	lobChIP_FOXA1_robot
chr14	23169475	23169476	MACS_peak_4224	5.00	lobChIP_FOXA1_robot
chr14	23237793	23237794	MACS_peak_4225	3.00	lobChIP_FOXA1_robot
chr14	23265767	23265768	MACS_peak_4226	7.00	lobChIP_FOXA1_robot
chr14	23308791	23308792	MACS_peak_4227	4.00	lobChIP_FOXA1_robot
chr14	23340515	23340516	MACS_peak_4228	17.00	lobChIP_FOXA1_robot
chr14	23372532	23372533	MACS_peak_4229	35.00	lobChIP_FOXA1_robot
chr14	23435925	23435926	MACS_peak_4230	4.00	lobChIP_FOXA1_robot
chr14	23446074	23446075	MACS_peak_4231	35.00	lobChIP_FOXA1_robot
chr14	23447760	23447761	MACS_peak_4232	3.00	lobChIP_FOXA1_robot
chr14	23475599	23475600	MACS_peak_4233	5.00	lobChIP_FOXA1_robot
chr14	23564990	23564991	MACS_peak_4234	4.00	lobChIP_FOXA1_robot
chr14	23660768	23660769	MACS_peak_4235	5.00	lobChIP_FOXA1_robot
chr14	23707172	23707173	MACS_peak_4236	5.00	lobChIP_FOXA1_robot
chr14	23714586	23714587	MACS_peak_4237	7.00	lobChIP_FOXA1_robot
chr14	23755967	23755968	MACS_peak_4238	4.00	lobChIP_FOXA1_robot
chr14	23776080	23776081	MACS_peak_4239	4.00	lobChIP_FOXA1_robot
chr14	23926062	23926063	MACS_peak_4240	4.00	lobChIP_FOXA1_robot
chr14	23962101	23962102	MACS_peak_4241	8.00	lobChIP_FOXA1_robot
chr14	24052718	24052719	MACS_peak_4242	7.00	lobChIP_FOXA1_robot
chr14	24071681	24071682	MACS_peak_4243	3.00	lobChIP_FOXA1_robot
chr14	24078896	24078897	MACS_peak_4244	4.00	lobChIP_FOXA1_robot
chr14	24079530	24079531	MACS_peak_4245	5.00	lobChIP_FOXA1_robot
chr14	24080836	24080837	MACS_peak_4246	5.00	lobChIP_FOXA1_robot
chr14	24082852	24082853	MACS_peak_4247	28.00	lobChIP_FOXA1_robot
chr14	24087064	24087065	MACS_peak_4248	4.00	lobChIP_FOXA1_robot
chr14	24088043	24088044	MACS_peak_4249	5.00	lobChIP_FOXA1_robot
chr14	24107819	24107820	MACS_peak_4250	3.00	lobChIP_FOXA1_robot
chr14	24116258	24116259	MACS_peak_4251	3.00	lobChIP_FOXA1_robot
chr14	24159100	24159101	MACS_peak_4252	5.00	lobChIP_FOXA1_robot
chr14	24207689	24207690	MACS_peak_4253	5.00	lobChIP_FOXA1_robot
chr14	24297419	24297420	MACS_peak_4254	10.00	lobChIP_FOXA1_robot
chr14	24319471	24319472	MACS_peak_4255	4.00	lobChIP_FOXA1_robot
chr14	24323384	24323385	MACS_peak_4256	3.00	lobChIP_FOXA1_robot
chr14	24334002	24334003	MACS_peak_4257	3.00	lobChIP_FOXA1_robot
chr14	24353344	24353345	MACS_peak_4258	32.00	lobChIP_FOXA1_robot
chr14	24443368	24443369	MACS_peak_4259	7.00	lobChIP_FOXA1_robot
chr14	24565286	24565287	MACS_peak_4260	6.00	lobChIP_FOXA1_robot
chr14	24577978	24577979	MACS_peak_4261	3.00	lobChIP_FOXA1_robot
chr14	24584121	24584122	MACS_peak_4262	6.00	lobChIP_FOXA1_robot
chr14	24856143	24856144	MACS_peak_4263	11.00	lobChIP_FOXA1_robot
chr14	24861538	24861539	MACS_peak_4264	5.00	lobChIP_FOXA1_robot
chr14	24881705	24881706	MACS_peak_4265	3.00	lobChIP_FOXA1_robot
chr14	24912160	24912161	MACS_peak_4266	6.00	lobChIP_FOXA1_robot
chr14	25148280	25148281	MACS_peak_4267	4.00	lobChIP_FOXA1_robot
chr14	25154606	25154607	MACS_peak_4268	5.00	lobChIP_FOXA1_robot
chr14	25159018	25159019	MACS_peak_4269	4.00	lobChIP_FOXA1_robot
chr14	25286488	25286489	MACS_peak_4270	12.00	lobChIP_FOXA1_robot
chr14	25287380	25287381	MACS_peak_4271	9.00	lobChIP_FOXA1_robot
chr14	25288668	25288669	MACS_peak_4272	6.00	lobChIP_FOXA1_robot
chr14	25308897	25308898	MACS_peak_4273	57.00	lobChIP_FOXA1_robot
chr14	25348056	25348057	MACS_peak_4274	6.00	lobChIP_FOXA1_robot
chr14	25357951	25357952	MACS_peak_4275	4.00	lobChIP_FOXA1_robot
chr14	25361369	25361370	MACS_peak_4276	5.00	lobChIP_FOXA1_robot
chr14	25378242	25378243	MACS_peak_4277	19.00	lobChIP_FOXA1_robot
chr14	25379852	25379853	MACS_peak_4278	5.00	lobChIP_FOXA1_robot
chr14	25385378	25385379	MACS_peak_4279	18.00	lobChIP_FOXA1_robot
chr14	25418923	25418924	MACS_peak_4280	4.00	lobChIP_FOXA1_robot
chr14	25452052	25452053	MACS_peak_4281	42.00	lobChIP_FOXA1_robot
chr14	25473872	25473873	MACS_peak_4282	3.00	lobChIP_FOXA1_robot
chr14	25474446	25474447	MACS_peak_4283	4.00	lobChIP_FOXA1_robot
chr14	25476459	25476460	MACS_peak_4284	6.00	lobChIP_FOXA1_robot
chr14	25480674	25480675	MACS_peak_4285	5.00	lobChIP_FOXA1_robot
chr14	25484405	25484406	MACS_peak_4286	7.00	lobChIP_FOXA1_robot
chr14	25504591	25504592	MACS_peak_4287	8.00	lobChIP_FOXA1_robot
chr14	25572215	25572216	MACS_peak_4288	3.00	lobChIP_FOXA1_robot
chr14	25726241	25726242	MACS_peak_4289	3.00	lobChIP_FOXA1_robot
chr14	25794852	25794853	MACS_peak_4290	5.00	lobChIP_FOXA1_robot
chr14	26065693	26065694	MACS_peak_4291	4.00	lobChIP_FOXA1_robot
chr14	26254889	26254890	MACS_peak_4292	5.00	lobChIP_FOXA1_robot
chr14	26258818	26258819	MACS_peak_4293	4.00	lobChIP_FOXA1_robot
chr14	26318336	26318337	MACS_peak_4294	4.00	lobChIP_FOXA1_robot
chr14	26649174	26649175	MACS_peak_4295	3.00	lobChIP_FOXA1_robot
chr14	26676550	26676551	MACS_peak_4296	3.00	lobChIP_FOXA1_robot
chr14	26883251	26883252	MACS_peak_4297	4.00	lobChIP_FOXA1_robot
chr14	26913063	26913064	MACS_peak_4298	4.00	lobChIP_FOXA1_robot
chr14	26939876	26939877	MACS_peak_4299	4.00	lobChIP_FOXA1_robot
chr14	27017051	27017052	MACS_peak_4300	5.00	lobChIP_FOXA1_robot
chr14	27188926	27188927	MACS_peak_4301	4.00	lobChIP_FOXA1_robot
chr14	27243699	27243700	MACS_peak_4302	4.00	lobChIP_FOXA1_robot
chr14	27250969	27250970	MACS_peak_4303	6.00	lobChIP_FOXA1_robot
chr14	27489615	27489616	MACS_peak_4304	4.00	lobChIP_FOXA1_robot
chr14	27539026	27539027	MACS_peak_4305	6.00	lobChIP_FOXA1_robot
chr14	27550990	27550991	MACS_peak_4306	5.00	lobChIP_FOXA1_robot
chr14	27693076	27693077	MACS_peak_4307	4.00	lobChIP_FOXA1_robot
chr14	27957788	27957789	MACS_peak_4308	3.00	lobChIP_FOXA1_robot
chr14	28176016	28176017	MACS_peak_4309	3.00	lobChIP_FOXA1_robot
chr14	28196339	28196340	MACS_peak_4310	5.00	lobChIP_FOXA1_robot
chr14	28461715	28461716	MACS_peak_4311	4.00	lobChIP_FOXA1_robot
chr14	28673499	28673500	MACS_peak_4312	5.00	lobChIP_FOXA1_robot
chr14	28690947	28690948	MACS_peak_4313	3.00	lobChIP_FOXA1_robot
chr14	28839986	28839987	MACS_peak_4314	4.00	lobChIP_FOXA1_robot
chr14	28945999	28946000	MACS_peak_4315	3.00	lobChIP_FOXA1_robot
chr14	29356938	29356939	MACS_peak_4316	4.00	lobChIP_FOXA1_robot
chr14	29390924	29390925	MACS_peak_4317	4.00	lobChIP_FOXA1_robot
chr14	29498361	29498362	MACS_peak_4318	13.00	lobChIP_FOXA1_robot
chr14	29599261	29599262	MACS_peak_4319	4.00	lobChIP_FOXA1_robot
chr14	29841044	29841045	MACS_peak_4320	3.00	lobChIP_FOXA1_robot
chr14	29992181	29992182	MACS_peak_4321	4.00	lobChIP_FOXA1_robot
chr14	30051451	30051452	MACS_peak_4322	5.00	lobChIP_FOXA1_robot
chr14	30224683	30224684	MACS_peak_4323	3.00	lobChIP_FOXA1_robot
chr14	30365841	30365842	MACS_peak_4324	6.00	lobChIP_FOXA1_robot
chr14	30802211	30802212	MACS_peak_4325	4.00	lobChIP_FOXA1_robot
chr14	30824858	30824859	MACS_peak_4326	4.00	lobChIP_FOXA1_robot
chr14	30878724	30878725	MACS_peak_4327	4.00	lobChIP_FOXA1_robot
chr14	31008425	31008426	MACS_peak_4328	3.00	lobChIP_FOXA1_robot
chr14	31028133	31028134	MACS_peak_4329	13.00	lobChIP_FOXA1_robot
chr14	31034405	31034406	MACS_peak_4330	4.00	lobChIP_FOXA1_robot
chr14	31057674	31057675	MACS_peak_4331	4.00	lobChIP_FOXA1_robot
chr14	31099454	31099455	MACS_peak_4332	3.00	lobChIP_FOXA1_robot
chr14	31125454	31125455	MACS_peak_4333	4.00	lobChIP_FOXA1_robot
chr14	31161436	31161437	MACS_peak_4334	5.00	lobChIP_FOXA1_robot
chr14	31221931	31221932	MACS_peak_4335	4.00	lobChIP_FOXA1_robot
chr14	31358362	31358363	MACS_peak_4336	8.00	lobChIP_FOXA1_robot
chr14	31436692	31436693	MACS_peak_4337	12.00	lobChIP_FOXA1_robot
chr14	31493962	31493963	MACS_peak_4338	5.00	lobChIP_FOXA1_robot
chr14	31500933	31500934	MACS_peak_4339	4.00	lobChIP_FOXA1_robot
chr14	31502743	31502744	MACS_peak_4340	24.00	lobChIP_FOXA1_robot
chr14	31507330	31507331	MACS_peak_4341	4.00	lobChIP_FOXA1_robot
chr14	31522582	31522583	MACS_peak_4342	3.00	lobChIP_FOXA1_robot
chr14	31551405	31551406	MACS_peak_4343	5.00	lobChIP_FOXA1_robot
chr14	31560867	31560868	MACS_peak_4344	19.00	lobChIP_FOXA1_robot
chr14	31597090	31597091	MACS_peak_4345	8.00	lobChIP_FOXA1_robot
chr14	31609809	31609810	MACS_peak_4346	4.00	lobChIP_FOXA1_robot
chr14	31667814	31667815	MACS_peak_4347	6.00	lobChIP_FOXA1_robot
chr14	31674939	31674940	MACS_peak_4348	4.00	lobChIP_FOXA1_robot
chr14	31677030	31677031	MACS_peak_4349	4.00	lobChIP_FOXA1_robot
chr14	31693955	31693956	MACS_peak_4350	3.00	lobChIP_FOXA1_robot
chr14	31699015	31699016	MACS_peak_4351	69.00	lobChIP_FOXA1_robot
chr14	31727539	31727540	MACS_peak_4352	31.00	lobChIP_FOXA1_robot
chr14	31731368	31731369	MACS_peak_4353	7.00	lobChIP_FOXA1_robot
chr14	31733708	31733709	MACS_peak_4354	8.00	lobChIP_FOXA1_robot
chr14	31749830	31749831	MACS_peak_4355	15.00	lobChIP_FOXA1_robot
chr14	31762433	31762434	MACS_peak_4356	4.00	lobChIP_FOXA1_robot
chr14	31809035	31809036	MACS_peak_4357	3.00	lobChIP_FOXA1_robot
chr14	31825236	31825237	MACS_peak_4358	3.00	lobChIP_FOXA1_robot
chr14	31847422	31847423	MACS_peak_4359	13.00	lobChIP_FOXA1_robot
chr14	31859624	31859625	MACS_peak_4360	5.00	lobChIP_FOXA1_robot
chr14	31888999	31889000	MACS_peak_4361	5.00	lobChIP_FOXA1_robot
chr14	31890276	31890277	MACS_peak_4362	3.00	lobChIP_FOXA1_robot
chr14	31890711	31890712	MACS_peak_4363	7.00	lobChIP_FOXA1_robot
chr14	31896057	31896058	MACS_peak_4364	4.00	lobChIP_FOXA1_robot
chr14	31927738	31927739	MACS_peak_4365	17.00	lobChIP_FOXA1_robot
chr14	31953607	31953608	MACS_peak_4366	5.00	lobChIP_FOXA1_robot
chr14	31998473	31998474	MACS_peak_4367	14.00	lobChIP_FOXA1_robot
chr14	32019918	32019919	MACS_peak_4368	5.00	lobChIP_FOXA1_robot
chr14	32095030	32095031	MACS_peak_4369	6.00	lobChIP_FOXA1_robot
chr14	32102617	32102618	MACS_peak_4370	3.00	lobChIP_FOXA1_robot
chr14	32135170	32135171	MACS_peak_4371	5.00	lobChIP_FOXA1_robot
chr14	32165818	32165819	MACS_peak_4372	8.00	lobChIP_FOXA1_robot
chr14	32166962	32166963	MACS_peak_4373	4.00	lobChIP_FOXA1_robot
chr14	32186955	32186956	MACS_peak_4374	21.00	lobChIP_FOXA1_robot
chr14	32210148	32210149	MACS_peak_4375	4.00	lobChIP_FOXA1_robot
chr14	32217692	32217693	MACS_peak_4376	19.00	lobChIP_FOXA1_robot
chr14	32324712	32324713	MACS_peak_4377	15.00	lobChIP_FOXA1_robot
chr14	32328303	32328304	MACS_peak_4378	6.00	lobChIP_FOXA1_robot
chr14	32439714	32439715	MACS_peak_4379	3.00	lobChIP_FOXA1_robot
chr14	32479950	32479951	MACS_peak_4380	3.00	lobChIP_FOXA1_robot
chr14	32537349	32537350	MACS_peak_4381	5.00	lobChIP_FOXA1_robot
chr14	32548159	32548160	MACS_peak_4382	6.00	lobChIP_FOXA1_robot
chr14	32553173	32553174	MACS_peak_4383	15.00	lobChIP_FOXA1_robot
chr14	32574810	32574811	MACS_peak_4384	3.00	lobChIP_FOXA1_robot
chr14	32576651	32576652	MACS_peak_4385	7.00	lobChIP_FOXA1_robot
chr14	32582559	32582560	MACS_peak_4386	4.00	lobChIP_FOXA1_robot
chr14	32586759	32586760	MACS_peak_4387	3.00	lobChIP_FOXA1_robot
chr14	32609947	32609948	MACS_peak_4388	6.00	lobChIP_FOXA1_robot
chr14	32668962	32668963	MACS_peak_4389	7.00	lobChIP_FOXA1_robot
chr14	32686224	32686225	MACS_peak_4390	4.00	lobChIP_FOXA1_robot
chr14	32728685	32728686	MACS_peak_4391	5.00	lobChIP_FOXA1_robot
chr14	32747400	32747401	MACS_peak_4392	7.00	lobChIP_FOXA1_robot
chr14	32777526	32777527	MACS_peak_4393	16.00	lobChIP_FOXA1_robot
chr14	32809780	32809781	MACS_peak_4394	3.00	lobChIP_FOXA1_robot
chr14	32920916	32920917	MACS_peak_4395	3.00	lobChIP_FOXA1_robot
chr14	32945572	32945573	MACS_peak_4396	3.00	lobChIP_FOXA1_robot
chr14	32965765	32965766	MACS_peak_4397	3.00	lobChIP_FOXA1_robot
chr14	33111224	33111225	MACS_peak_4398	4.00	lobChIP_FOXA1_robot
chr14	33287657	33287658	MACS_peak_4399	4.00	lobChIP_FOXA1_robot
chr14	33446362	33446363	MACS_peak_4400	3.00	lobChIP_FOXA1_robot
chr14	33635871	33635872	MACS_peak_4401	5.00	lobChIP_FOXA1_robot
chr14	33750103	33750104	MACS_peak_4402	3.00	lobChIP_FOXA1_robot
chr14	33805764	33805765	MACS_peak_4403	3.00	lobChIP_FOXA1_robot
chr14	33810382	33810383	MACS_peak_4404	5.00	lobChIP_FOXA1_robot
chr14	34050778	34050779	MACS_peak_4405	3.00	lobChIP_FOXA1_robot
chr14	34369146	34369147	MACS_peak_4406	24.00	lobChIP_FOXA1_robot
chr14	34372730	34372731	MACS_peak_4407	3.00	lobChIP_FOXA1_robot
chr14	34456902	34456903	MACS_peak_4408	12.00	lobChIP_FOXA1_robot
chr14	34459440	34459441	MACS_peak_4409	3.00	lobChIP_FOXA1_robot
chr14	34460143	34460144	MACS_peak_4410	5.00	lobChIP_FOXA1_robot
chr14	34493537	34493538	MACS_peak_4411	32.00	lobChIP_FOXA1_robot
chr14	34504572	34504573	MACS_peak_4412	4.00	lobChIP_FOXA1_robot
chr14	34508665	34508666	MACS_peak_4413	8.00	lobChIP_FOXA1_robot
chr14	34513213	34513214	MACS_peak_4414	10.00	lobChIP_FOXA1_robot
chr14	34521317	34521318	MACS_peak_4415	7.00	lobChIP_FOXA1_robot
chr14	34524842	34524843	MACS_peak_4416	4.00	lobChIP_FOXA1_robot
chr14	34547575	34547576	MACS_peak_4417	5.00	lobChIP_FOXA1_robot
chr14	34551224	34551225	MACS_peak_4418	25.00	lobChIP_FOXA1_robot
chr14	34609202	34609203	MACS_peak_4419	9.00	lobChIP_FOXA1_robot
chr14	34631241	34631242	MACS_peak_4420	3.00	lobChIP_FOXA1_robot
chr14	34707061	34707062	MACS_peak_4421	4.00	lobChIP_FOXA1_robot
chr14	34788044	34788045	MACS_peak_4422	5.00	lobChIP_FOXA1_robot
chr14	34791666	34791667	MACS_peak_4423	3.00	lobChIP_FOXA1_robot
chr14	34817107	34817108	MACS_peak_4424	6.00	lobChIP_FOXA1_robot
chr14	34834853	34834854	MACS_peak_4425	4.00	lobChIP_FOXA1_robot
chr14	34837761	34837762	MACS_peak_4426	13.00	lobChIP_FOXA1_robot
chr14	34905801	34905802	MACS_peak_4427	4.00	lobChIP_FOXA1_robot
chr14	34919740	34919741	MACS_peak_4428	17.00	lobChIP_FOXA1_robot
chr14	34930217	34930218	MACS_peak_4429	3.00	lobChIP_FOXA1_robot
chr14	34953882	34953883	MACS_peak_4430	4.00	lobChIP_FOXA1_robot
chr14	34966852	34966853	MACS_peak_4431	6.00	lobChIP_FOXA1_robot
chr14	34967388	34967389	MACS_peak_4432	5.00	lobChIP_FOXA1_robot
chr14	35008898	35008899	MACS_peak_4433	9.00	lobChIP_FOXA1_robot
chr14	35032000	35032001	MACS_peak_4434	5.00	lobChIP_FOXA1_robot
chr14	35072903	35072904	MACS_peak_4435	5.00	lobChIP_FOXA1_robot
chr14	35118129	35118130	MACS_peak_4436	5.00	lobChIP_FOXA1_robot
chr14	35156863	35156864	MACS_peak_4437	13.00	lobChIP_FOXA1_robot
chr14	35160569	35160570	MACS_peak_4438	5.00	lobChIP_FOXA1_robot
chr14	35169553	35169554	MACS_peak_4439	7.00	lobChIP_FOXA1_robot
chr14	35174599	35174600	MACS_peak_4440	4.00	lobChIP_FOXA1_robot
chr14	35177231	35177232	MACS_peak_4441	8.00	lobChIP_FOXA1_robot
chr14	35238723	35238724	MACS_peak_4442	3.00	lobChIP_FOXA1_robot
chr14	35267316	35267317	MACS_peak_4443	3.00	lobChIP_FOXA1_robot
chr14	35302992	35302993	MACS_peak_4444	4.00	lobChIP_FOXA1_robot
chr14	35303722	35303723	MACS_peak_4445	4.00	lobChIP_FOXA1_robot
chr14	35306991	35306992	MACS_peak_4446	7.00	lobChIP_FOXA1_robot
chr14	35344859	35344860	MACS_peak_4447	3.00	lobChIP_FOXA1_robot
chr14	35450657	35450658	MACS_peak_4448	3.00	lobChIP_FOXA1_robot
chr14	35452848	35452849	MACS_peak_4449	4.00	lobChIP_FOXA1_robot
chr14	35474147	35474148	MACS_peak_4450	4.00	lobChIP_FOXA1_robot
chr14	35476860	35476861	MACS_peak_4451	9.00	lobChIP_FOXA1_robot
chr14	35494512	35494513	MACS_peak_4452	3.00	lobChIP_FOXA1_robot
chr14	35507281	35507282	MACS_peak_4453	3.00	lobChIP_FOXA1_robot
chr14	35517555	35517556	MACS_peak_4454	6.00	lobChIP_FOXA1_robot
chr14	35521181	35521182	MACS_peak_4455	7.00	lobChIP_FOXA1_robot
chr14	35613493	35613494	MACS_peak_4456	5.00	lobChIP_FOXA1_robot
chr14	35628625	35628626	MACS_peak_4457	6.00	lobChIP_FOXA1_robot
chr14	35629393	35629394	MACS_peak_4458	5.00	lobChIP_FOXA1_robot
chr14	35658910	35658911	MACS_peak_4459	7.00	lobChIP_FOXA1_robot
chr14	35673342	35673343	MACS_peak_4460	11.00	lobChIP_FOXA1_robot
chr14	35683347	35683348	MACS_peak_4461	5.00	lobChIP_FOXA1_robot
chr14	35805717	35805718	MACS_peak_4462	5.00	lobChIP_FOXA1_robot
chr14	35817012	35817013	MACS_peak_4463	13.00	lobChIP_FOXA1_robot
chr14	35823165	35823166	MACS_peak_4464	7.00	lobChIP_FOXA1_robot
chr14	35825735	35825736	MACS_peak_4465	6.00	lobChIP_FOXA1_robot
chr14	35867719	35867720	MACS_peak_4466	8.00	lobChIP_FOXA1_robot
chr14	35873154	35873155	MACS_peak_4467	4.00	lobChIP_FOXA1_robot
chr14	35876249	35876250	MACS_peak_4468	9.00	lobChIP_FOXA1_robot
chr14	35882814	35882815	MACS_peak_4469	3.00	lobChIP_FOXA1_robot
chr14	36054387	36054388	MACS_peak_4470	5.00	lobChIP_FOXA1_robot
chr14	36113879	36113880	MACS_peak_4471	3.00	lobChIP_FOXA1_robot
chr14	36124295	36124296	MACS_peak_4472	4.00	lobChIP_FOXA1_robot
chr14	36131313	36131314	MACS_peak_4473	10.00	lobChIP_FOXA1_robot
chr14	36189411	36189412	MACS_peak_4474	5.00	lobChIP_FOXA1_robot
chr14	36216158	36216159	MACS_peak_4475	5.00	lobChIP_FOXA1_robot
chr14	36216874	36216875	MACS_peak_4476	4.00	lobChIP_FOXA1_robot
chr14	36272732	36272733	MACS_peak_4477	3.00	lobChIP_FOXA1_robot
chr14	36276960	36276961	MACS_peak_4478	52.00	lobChIP_FOXA1_robot
chr14	36283821	36283822	MACS_peak_4479	6.00	lobChIP_FOXA1_robot
chr14	36468888	36468889	MACS_peak_4480	4.00	lobChIP_FOXA1_robot
chr14	36538897	36538898	MACS_peak_4481	7.00	lobChIP_FOXA1_robot
chr14	36540012	36540013	MACS_peak_4482	4.00	lobChIP_FOXA1_robot
chr14	36678079	36678080	MACS_peak_4483	124.00	lobChIP_FOXA1_robot
chr14	36788922	36788923	MACS_peak_4484	6.00	lobChIP_FOXA1_robot
chr14	36903832	36903833	MACS_peak_4485	3.00	lobChIP_FOXA1_robot
chr14	36924069	36924070	MACS_peak_4486	4.00	lobChIP_FOXA1_robot
chr14	37026433	37026434	MACS_peak_4487	3.00	lobChIP_FOXA1_robot
chr14	37074402	37074403	MACS_peak_4488	3.00	lobChIP_FOXA1_robot
chr14	37099660	37099661	MACS_peak_4489	3.00	lobChIP_FOXA1_robot
chr14	37148711	37148712	MACS_peak_4490	5.00	lobChIP_FOXA1_robot
chr14	37190636	37190637	MACS_peak_4491	4.00	lobChIP_FOXA1_robot
chr14	37240650	37240651	MACS_peak_4492	4.00	lobChIP_FOXA1_robot
chr14	37395563	37395564	MACS_peak_4493	3.00	lobChIP_FOXA1_robot
chr14	37449825	37449826	MACS_peak_4494	6.00	lobChIP_FOXA1_robot
chr14	37506107	37506108	MACS_peak_4495	5.00	lobChIP_FOXA1_robot
chr14	37584719	37584720	MACS_peak_4496	4.00	lobChIP_FOXA1_robot
chr14	37605602	37605603	MACS_peak_4497	3.00	lobChIP_FOXA1_robot
chr14	37608216	37608217	MACS_peak_4498	6.00	lobChIP_FOXA1_robot
chr14	37700042	37700043	MACS_peak_4499	6.00	lobChIP_FOXA1_robot
chr14	37715943	37715944	MACS_peak_4500	13.00	lobChIP_FOXA1_robot
chr14	37717239	37717240	MACS_peak_4501	4.00	lobChIP_FOXA1_robot
chr14	37721551	37721552	MACS_peak_4502	3.00	lobChIP_FOXA1_robot
chr14	37724575	37724576	MACS_peak_4503	4.00	lobChIP_FOXA1_robot
chr14	37725772	37725773	MACS_peak_4504	4.00	lobChIP_FOXA1_robot
chr14	37735968	37735969	MACS_peak_4505	4.00	lobChIP_FOXA1_robot
chr14	37740020	37740021	MACS_peak_4506	17.00	lobChIP_FOXA1_robot
chr14	37747003	37747004	MACS_peak_4507	5.00	lobChIP_FOXA1_robot
chr14	37748184	37748185	MACS_peak_4508	18.00	lobChIP_FOXA1_robot
chr14	37759032	37759033	MACS_peak_4509	4.00	lobChIP_FOXA1_robot
chr14	37799053	37799054	MACS_peak_4510	40.00	lobChIP_FOXA1_robot
chr14	37805290	37805291	MACS_peak_4511	4.00	lobChIP_FOXA1_robot
chr14	37810580	37810581	MACS_peak_4512	4.00	lobChIP_FOXA1_robot
chr14	37850557	37850558	MACS_peak_4513	3.00	lobChIP_FOXA1_robot
chr14	37854670	37854671	MACS_peak_4514	11.00	lobChIP_FOXA1_robot
chr14	37883131	37883132	MACS_peak_4515	18.00	lobChIP_FOXA1_robot
chr14	37917139	37917140	MACS_peak_4516	3.00	lobChIP_FOXA1_robot
chr14	37925712	37925713	MACS_peak_4517	3.00	lobChIP_FOXA1_robot
chr14	37936011	37936012	MACS_peak_4518	60.00	lobChIP_FOXA1_robot
chr14	37937432	37937433	MACS_peak_4519	28.00	lobChIP_FOXA1_robot
chr14	37962985	37962986	MACS_peak_4520	5.00	lobChIP_FOXA1_robot
chr14	37991585	37991586	MACS_peak_4521	3.00	lobChIP_FOXA1_robot
chr14	37996742	37996743	MACS_peak_4522	10.00	lobChIP_FOXA1_robot
chr14	38016259	38016260	MACS_peak_4523	112.00	lobChIP_FOXA1_robot
chr14	38034772	38034773	MACS_peak_4524	4.00	lobChIP_FOXA1_robot
chr14	38044451	38044452	MACS_peak_4525	4.00	lobChIP_FOXA1_robot
chr14	38053032	38053033	MACS_peak_4526	4.00	lobChIP_FOXA1_robot
chr14	38056346	38056347	MACS_peak_4527	5.00	lobChIP_FOXA1_robot
chr14	38057458	38057459	MACS_peak_4528	8.00	lobChIP_FOXA1_robot
chr14	38058020	38058021	MACS_peak_4529	20.00	lobChIP_FOXA1_robot
chr14	38059420	38059421	MACS_peak_4530	4.00	lobChIP_FOXA1_robot
chr14	38064646	38064647	MACS_peak_4531	4.00	lobChIP_FOXA1_robot
chr14	38072197	38072198	MACS_peak_4532	6.00	lobChIP_FOXA1_robot
chr14	38095685	38095686	MACS_peak_4533	4.00	lobChIP_FOXA1_robot
chr14	38110558	38110559	MACS_peak_4534	4.00	lobChIP_FOXA1_robot
chr14	38119758	38119759	MACS_peak_4535	11.00	lobChIP_FOXA1_robot
chr14	38127515	38127516	MACS_peak_4536	4.00	lobChIP_FOXA1_robot
chr14	38166926	38166927	MACS_peak_4537	26.00	lobChIP_FOXA1_robot
chr14	38168617	38168618	MACS_peak_4538	4.00	lobChIP_FOXA1_robot
chr14	38181538	38181539	MACS_peak_4539	5.00	lobChIP_FOXA1_robot
chr14	38257493	38257494	MACS_peak_4540	6.00	lobChIP_FOXA1_robot
chr14	38260722	38260723	MACS_peak_4541	13.00	lobChIP_FOXA1_robot
chr14	38288176	38288177	MACS_peak_4542	4.00	lobChIP_FOXA1_robot
chr14	38288548	38288549	MACS_peak_4543	8.00	lobChIP_FOXA1_robot
chr14	38334418	38334419	MACS_peak_4544	43.00	lobChIP_FOXA1_robot
chr14	38340858	38340859	MACS_peak_4545	4.00	lobChIP_FOXA1_robot
chr14	38342164	38342165	MACS_peak_4546	3.00	lobChIP_FOXA1_robot
chr14	38443015	38443016	MACS_peak_4547	4.00	lobChIP_FOXA1_robot
chr14	38569851	38569852	MACS_peak_4548	9.00	lobChIP_FOXA1_robot
chr14	38686373	38686374	MACS_peak_4549	4.00	lobChIP_FOXA1_robot
chr14	39007858	39007859	MACS_peak_4550	4.00	lobChIP_FOXA1_robot
chr14	39011405	39011406	MACS_peak_4551	3.00	lobChIP_FOXA1_robot
chr14	39075180	39075181	MACS_peak_4552	15.00	lobChIP_FOXA1_robot
chr14	39211936	39211937	MACS_peak_4553	5.00	lobChIP_FOXA1_robot
chr14	39324885	39324886	MACS_peak_4554	6.00	lobChIP_FOXA1_robot
chr14	39338151	39338152	MACS_peak_4555	12.00	lobChIP_FOXA1_robot
chr14	39350061	39350062	MACS_peak_4556	3.00	lobChIP_FOXA1_robot
chr14	39434003	39434004	MACS_peak_4557	4.00	lobChIP_FOXA1_robot
chr14	39442416	39442417	MACS_peak_4558	6.00	lobChIP_FOXA1_robot
chr14	39480902	39480903	MACS_peak_4559	5.00	lobChIP_FOXA1_robot
chr14	39483876	39483877	MACS_peak_4560	6.00	lobChIP_FOXA1_robot
chr14	39485013	39485014	MACS_peak_4561	4.00	lobChIP_FOXA1_robot
chr14	39493553	39493554	MACS_peak_4562	94.00	lobChIP_FOXA1_robot
chr14	39495169	39495170	MACS_peak_4563	18.00	lobChIP_FOXA1_robot
chr14	39573050	39573051	MACS_peak_4564	5.00	lobChIP_FOXA1_robot
chr14	39614768	39614769	MACS_peak_4565	7.00	lobChIP_FOXA1_robot
chr14	39630349	39630350	MACS_peak_4566	7.00	lobChIP_FOXA1_robot
chr14	39687815	39687816	MACS_peak_4567	8.00	lobChIP_FOXA1_robot
chr14	39688575	39688576	MACS_peak_4568	6.00	lobChIP_FOXA1_robot
chr14	39702292	39702293	MACS_peak_4569	7.00	lobChIP_FOXA1_robot
chr14	39703112	39703113	MACS_peak_4570	7.00	lobChIP_FOXA1_robot
chr14	39716150	39716151	MACS_peak_4571	4.00	lobChIP_FOXA1_robot
chr14	39737077	39737078	MACS_peak_4572	4.00	lobChIP_FOXA1_robot
chr14	39866284	39866285	MACS_peak_4573	3.00	lobChIP_FOXA1_robot
chr14	40075304	40075305	MACS_peak_4574	3.00	lobChIP_FOXA1_robot
chr14	40322526	40322527	MACS_peak_4575	4.00	lobChIP_FOXA1_robot
chr14	40350347	40350348	MACS_peak_4576	4.00	lobChIP_FOXA1_robot
chr14	40433481	40433482	MACS_peak_4577	3.00	lobChIP_FOXA1_robot
chr14	40434819	40434820	MACS_peak_4578	4.00	lobChIP_FOXA1_robot
chr14	40458569	40458570	MACS_peak_4579	5.00	lobChIP_FOXA1_robot
chr14	40694688	40694689	MACS_peak_4580	3.00	lobChIP_FOXA1_robot
chr14	40808154	40808155	MACS_peak_4581	4.00	lobChIP_FOXA1_robot
chr14	41067888	41067889	MACS_peak_4582	4.00	lobChIP_FOXA1_robot
chr14	41184572	41184573	MACS_peak_4583	3.00	lobChIP_FOXA1_robot
chr14	41239237	41239238	MACS_peak_4584	4.00	lobChIP_FOXA1_robot
chr14	41311025	41311026	MACS_peak_4585	4.00	lobChIP_FOXA1_robot
chr14	41521943	41521944	MACS_peak_4586	4.00	lobChIP_FOXA1_robot
chr14	41596363	41596364	MACS_peak_4587	3.00	lobChIP_FOXA1_robot
chr14	41711598	41711599	MACS_peak_4588	3.00	lobChIP_FOXA1_robot
chr14	41741977	41741978	MACS_peak_4589	11.00	lobChIP_FOXA1_robot
chr14	42027341	42027342	MACS_peak_4590	3.00	lobChIP_FOXA1_robot
chr14	42132573	42132574	MACS_peak_4591	3.00	lobChIP_FOXA1_robot
chr14	42156720	42156721	MACS_peak_4592	3.00	lobChIP_FOXA1_robot
chr14	42303059	42303060	MACS_peak_4593	4.00	lobChIP_FOXA1_robot
chr14	42348848	42348849	MACS_peak_4594	3.00	lobChIP_FOXA1_robot
chr14	42421961	42421962	MACS_peak_4595	4.00	lobChIP_FOXA1_robot
chr14	42453345	42453346	MACS_peak_4596	3.00	lobChIP_FOXA1_robot
chr14	42492802	42492803	MACS_peak_4597	4.00	lobChIP_FOXA1_robot
chr14	42788311	42788312	MACS_peak_4598	3.00	lobChIP_FOXA1_robot
chr14	42798915	42798916	MACS_peak_4599	5.00	lobChIP_FOXA1_robot
chr14	42800301	42800302	MACS_peak_4600	6.00	lobChIP_FOXA1_robot
chr14	42845859	42845860	MACS_peak_4601	3.00	lobChIP_FOXA1_robot
chr14	42915707	42915708	MACS_peak_4602	4.00	lobChIP_FOXA1_robot
chr14	42962928	42962929	MACS_peak_4603	4.00	lobChIP_FOXA1_robot
chr14	43076928	43076929	MACS_peak_4604	4.00	lobChIP_FOXA1_robot
chr14	43284162	43284163	MACS_peak_4605	5.00	lobChIP_FOXA1_robot
chr14	43509489	43509490	MACS_peak_4606	5.00	lobChIP_FOXA1_robot
chr14	43519978	43519979	MACS_peak_4607	4.00	lobChIP_FOXA1_robot
chr14	43564948	43564949	MACS_peak_4608	3.00	lobChIP_FOXA1_robot
chr14	43583675	43583676	MACS_peak_4609	5.00	lobChIP_FOXA1_robot
chr14	43584646	43584647	MACS_peak_4610	5.00	lobChIP_FOXA1_robot
chr14	43596355	43596356	MACS_peak_4611	3.00	lobChIP_FOXA1_robot
chr14	43600885	43600886	MACS_peak_4612	3.00	lobChIP_FOXA1_robot
chr14	43611852	43611853	MACS_peak_4613	4.00	lobChIP_FOXA1_robot
chr14	43614815	43614816	MACS_peak_4614	4.00	lobChIP_FOXA1_robot
chr14	43642559	43642560	MACS_peak_4615	7.00	lobChIP_FOXA1_robot
chr14	43654499	43654500	MACS_peak_4616	4.00	lobChIP_FOXA1_robot
chr14	43683910	43683911	MACS_peak_4617	4.00	lobChIP_FOXA1_robot
chr14	43695463	43695464	MACS_peak_4618	4.00	lobChIP_FOXA1_robot
chr14	43790790	43790791	MACS_peak_4619	4.00	lobChIP_FOXA1_robot
chr14	44248530	44248531	MACS_peak_4620	18.00	lobChIP_FOXA1_robot
chr14	44249779	44249780	MACS_peak_4621	20.00	lobChIP_FOXA1_robot
chr14	44365463	44365464	MACS_peak_4622	5.00	lobChIP_FOXA1_robot
chr14	44369914	44369915	MACS_peak_4623	4.00	lobChIP_FOXA1_robot
chr14	45464528	45464529	MACS_peak_4624	5.00	lobChIP_FOXA1_robot
chr14	45527080	45527081	MACS_peak_4625	6.00	lobChIP_FOXA1_robot
chr14	45582252	45582253	MACS_peak_4626	8.00	lobChIP_FOXA1_robot
chr14	49274757	49274758	MACS_peak_4627	8.00	lobChIP_FOXA1_robot
chr14	50063115	50063116	MACS_peak_4628	9.00	lobChIP_FOXA1_robot
chr14	50108719	50108720	MACS_peak_4629	20.00	lobChIP_FOXA1_robot
chr14	50127002	50127003	MACS_peak_4630	15.00	lobChIP_FOXA1_robot
chr14	50188648	50188649	MACS_peak_4631	9.00	lobChIP_FOXA1_robot
chr14	50229105	50229106	MACS_peak_4632	8.00	lobChIP_FOXA1_robot
chr14	50230840	50230841	MACS_peak_4633	3.00	lobChIP_FOXA1_robot
chr14	50237230	50237231	MACS_peak_4634	12.00	lobChIP_FOXA1_robot
chr14	50329224	50329225	MACS_peak_4635	4.00	lobChIP_FOXA1_robot
chr14	50372018	50372019	MACS_peak_4636	6.00	lobChIP_FOXA1_robot
chr14	50402438	50402439	MACS_peak_4637	3.00	lobChIP_FOXA1_robot
chr14	50454298	50454299	MACS_peak_4638	12.00	lobChIP_FOXA1_robot
chr14	50469896	50469897	MACS_peak_4639	3.00	lobChIP_FOXA1_robot
chr14	50527928	50527929	MACS_peak_4640	3.00	lobChIP_FOXA1_robot
chr14	50908710	50908711	MACS_peak_4641	13.00	lobChIP_FOXA1_robot
chr14	50988894	50988895	MACS_peak_4642	7.00	lobChIP_FOXA1_robot
chr14	51067019	51067020	MACS_peak_4643	7.00	lobChIP_FOXA1_robot
chr14	51300127	51300128	MACS_peak_4644	12.00	lobChIP_FOXA1_robot
chr14	51305972	51305973	MACS_peak_4645	6.00	lobChIP_FOXA1_robot
chr14	51341806	51341807	MACS_peak_4646	5.00	lobChIP_FOXA1_robot
chr14	51342311	51342312	MACS_peak_4647	5.00	lobChIP_FOXA1_robot
chr14	51402482	51402483	MACS_peak_4648	20.00	lobChIP_FOXA1_robot
chr14	51406203	51406204	MACS_peak_4649	10.00	lobChIP_FOXA1_robot
chr14	51412086	51412087	MACS_peak_4650	10.00	lobChIP_FOXA1_robot
chr14	51472154	51472155	MACS_peak_4651	6.00	lobChIP_FOXA1_robot
chr14	51502503	51502504	MACS_peak_4652	8.00	lobChIP_FOXA1_robot
chr14	52143290	52143291	MACS_peak_4653	7.00	lobChIP_FOXA1_robot
chr14	52168807	52168808	MACS_peak_4654	4.00	lobChIP_FOXA1_robot
chr14	52924832	52924833	MACS_peak_4655	9.00	lobChIP_FOXA1_robot
chr14	53167703	53167704	MACS_peak_4656	12.00	lobChIP_FOXA1_robot
chr14	53222384	53222385	MACS_peak_4657	5.00	lobChIP_FOXA1_robot
chr14	53288564	53288565	MACS_peak_4658	16.00	lobChIP_FOXA1_robot
chr14	53311219	53311220	MACS_peak_4659	9.00	lobChIP_FOXA1_robot
chr14	53319925	53319926	MACS_peak_4660	5.00	lobChIP_FOXA1_robot
chr14	53393063	53393064	MACS_peak_4661	10.00	lobChIP_FOXA1_robot
chr14	53401693	53401694	MACS_peak_4662	12.00	lobChIP_FOXA1_robot
chr14	53414937	53414938	MACS_peak_4663	5.00	lobChIP_FOXA1_robot
chr14	53917801	53917802	MACS_peak_4664	15.00	lobChIP_FOXA1_robot
chr14	53925212	53925213	MACS_peak_4665	16.00	lobChIP_FOXA1_robot
chr14	53945122	53945123	MACS_peak_4666	5.00	lobChIP_FOXA1_robot
chr14	54080757	54080758	MACS_peak_4667	20.00	lobChIP_FOXA1_robot
chr14	54170348	54170349	MACS_peak_4668	5.00	lobChIP_FOXA1_robot
chr14	54309800	54309801	MACS_peak_4669	5.00	lobChIP_FOXA1_robot
chr14	54317602	54317603	MACS_peak_4670	4.00	lobChIP_FOXA1_robot
chr14	54318605	54318606	MACS_peak_4671	8.00	lobChIP_FOXA1_robot
chr14	54322080	54322081	MACS_peak_4672	8.00	lobChIP_FOXA1_robot
chr14	54348113	54348114	MACS_peak_4673	3.00	lobChIP_FOXA1_robot
chr14	54349651	54349652	MACS_peak_4674	6.00	lobChIP_FOXA1_robot
chr14	54375398	54375399	MACS_peak_4675	5.00	lobChIP_FOXA1_robot
chr14	54394135	54394136	MACS_peak_4676	6.00	lobChIP_FOXA1_robot
chr14	54410141	54410142	MACS_peak_4677	8.00	lobChIP_FOXA1_robot
chr14	54414274	54414275	MACS_peak_4678	10.00	lobChIP_FOXA1_robot
chr14	54446914	54446915	MACS_peak_4679	4.00	lobChIP_FOXA1_robot
chr14	54468018	54468019	MACS_peak_4680	5.00	lobChIP_FOXA1_robot
chr14	54512916	54512917	MACS_peak_4681	4.00	lobChIP_FOXA1_robot
chr14	54514755	54514756	MACS_peak_4682	5.00	lobChIP_FOXA1_robot
chr14	54515691	54515692	MACS_peak_4683	10.00	lobChIP_FOXA1_robot
chr14	54555151	54555152	MACS_peak_4684	6.00	lobChIP_FOXA1_robot
chr14	54568956	54568957	MACS_peak_4685	19.00	lobChIP_FOXA1_robot
chr14	54575604	54575605	MACS_peak_4686	21.00	lobChIP_FOXA1_robot
chr14	54602667	54602668	MACS_peak_4687	6.00	lobChIP_FOXA1_robot
chr14	54605544	54605545	MACS_peak_4688	15.00	lobChIP_FOXA1_robot
chr14	54664391	54664392	MACS_peak_4689	9.00	lobChIP_FOXA1_robot
chr14	54758817	54758818	MACS_peak_4690	4.00	lobChIP_FOXA1_robot
chr14	54819853	54819854	MACS_peak_4691	5.00	lobChIP_FOXA1_robot
chr14	54821000	54821001	MACS_peak_4692	25.00	lobChIP_FOXA1_robot
chr14	54822732	54822733	MACS_peak_4693	5.00	lobChIP_FOXA1_robot
chr14	54827579	54827580	MACS_peak_4694	8.00	lobChIP_FOXA1_robot
chr14	54831438	54831439	MACS_peak_4695	6.00	lobChIP_FOXA1_robot
chr14	54866615	54866616	MACS_peak_4696	4.00	lobChIP_FOXA1_robot
chr14	54891311	54891312	MACS_peak_4697	7.00	lobChIP_FOXA1_robot
chr14	55004538	55004539	MACS_peak_4698	15.00	lobChIP_FOXA1_robot
chr14	55110318	55110319	MACS_peak_4699	5.00	lobChIP_FOXA1_robot
chr14	55117798	55117799	MACS_peak_4700	6.00	lobChIP_FOXA1_robot
chr14	55123017	55123018	MACS_peak_4701	29.00	lobChIP_FOXA1_robot
chr14	55306572	55306573	MACS_peak_4702	14.00	lobChIP_FOXA1_robot
chr14	55329758	55329759	MACS_peak_4703	4.00	lobChIP_FOXA1_robot
chr14	55570112	55570113	MACS_peak_4704	5.00	lobChIP_FOXA1_robot
chr14	55901766	55901767	MACS_peak_4705	6.00	lobChIP_FOXA1_robot
chr14	56039420	56039421	MACS_peak_4706	8.00	lobChIP_FOXA1_robot
chr14	56051028	56051029	MACS_peak_4707	4.00	lobChIP_FOXA1_robot
chr14	56059443	56059444	MACS_peak_4708	23.00	lobChIP_FOXA1_robot
chr14	56066563	56066564	MACS_peak_4709	18.00	lobChIP_FOXA1_robot
chr14	56072715	56072716	MACS_peak_4710	21.00	lobChIP_FOXA1_robot
chr14	56298943	56298944	MACS_peak_4711	6.00	lobChIP_FOXA1_robot
chr14	56584868	56584869	MACS_peak_4712	5.00	lobChIP_FOXA1_robot
chr14	57059744	57059745	MACS_peak_4713	5.00	lobChIP_FOXA1_robot
chr14	57126857	57126858	MACS_peak_4714	4.00	lobChIP_FOXA1_robot
chr14	57740145	57740146	MACS_peak_4715	4.00	lobChIP_FOXA1_robot
chr14	57759221	57759222	MACS_peak_4716	6.00	lobChIP_FOXA1_robot
chr14	57813636	57813637	MACS_peak_4717	9.00	lobChIP_FOXA1_robot
chr14	57850165	57850166	MACS_peak_4718	4.00	lobChIP_FOXA1_robot
chr14	58739519	58739520	MACS_peak_4719	11.00	lobChIP_FOXA1_robot
chr14	58754461	58754462	MACS_peak_4720	13.00	lobChIP_FOXA1_robot
chr14	58797910	58797911	MACS_peak_4721	3.00	lobChIP_FOXA1_robot
chr14	58894201	58894202	MACS_peak_4722	5.00	lobChIP_FOXA1_robot
chr14	59476813	59476814	MACS_peak_4723	9.00	lobChIP_FOXA1_robot
chr14	59480535	59480536	MACS_peak_4724	7.00	lobChIP_FOXA1_robot
chr14	59536307	59536308	MACS_peak_4725	13.00	lobChIP_FOXA1_robot
chr14	59729915	59729916	MACS_peak_4726	8.00	lobChIP_FOXA1_robot
chr14	59742634	59742635	MACS_peak_4727	5.00	lobChIP_FOXA1_robot
chr14	59750245	59750246	MACS_peak_4728	25.00	lobChIP_FOXA1_robot
chr14	59764037	59764038	MACS_peak_4729	14.00	lobChIP_FOXA1_robot
chr14	59768205	59768206	MACS_peak_4730	5.00	lobChIP_FOXA1_robot
chr14	59772722	59772723	MACS_peak_4731	6.00	lobChIP_FOXA1_robot
chr14	59781129	59781130	MACS_peak_4732	7.00	lobChIP_FOXA1_robot
chr14	60900847	60900848	MACS_peak_4733	6.00	lobChIP_FOXA1_robot
chr14	61110992	61110993	MACS_peak_4734	6.00	lobChIP_FOXA1_robot
chr14	61132084	61132085	MACS_peak_4735	7.00	lobChIP_FOXA1_robot
chr14	61153809	61153810	MACS_peak_4736	10.00	lobChIP_FOXA1_robot
chr14	61282352	61282353	MACS_peak_4737	10.00	lobChIP_FOXA1_robot
chr14	61286497	61286498	MACS_peak_4738	5.00	lobChIP_FOXA1_robot
chr14	61400815	61400816	MACS_peak_4739	4.00	lobChIP_FOXA1_robot
chr14	61431770	61431771	MACS_peak_4740	7.00	lobChIP_FOXA1_robot
chr14	61509045	61509046	MACS_peak_4741	20.00	lobChIP_FOXA1_robot
chr14	61569687	61569688	MACS_peak_4742	36.00	lobChIP_FOXA1_robot
chr14	61719651	61719652	MACS_peak_4743	4.00	lobChIP_FOXA1_robot
chr14	61905887	61905888	MACS_peak_4744	5.00	lobChIP_FOXA1_robot
chr14	61958086	61958087	MACS_peak_4745	4.00	lobChIP_FOXA1_robot
chr14	62003677	62003678	MACS_peak_4746	3.00	lobChIP_FOXA1_robot
chr14	62004475	62004476	MACS_peak_4747	16.00	lobChIP_FOXA1_robot
chr14	62020690	62020691	MACS_peak_4748	16.00	lobChIP_FOXA1_robot
chr14	62023329	62023330	MACS_peak_4749	4.00	lobChIP_FOXA1_robot
chr14	62034166	62034167	MACS_peak_4750	5.00	lobChIP_FOXA1_robot
chr14	62048164	62048165	MACS_peak_4751	7.00	lobChIP_FOXA1_robot
chr14	62178431	62178432	MACS_peak_4752	5.00	lobChIP_FOXA1_robot
chr14	62181885	62181886	MACS_peak_4753	28.00	lobChIP_FOXA1_robot
chr14	63156559	63156560	MACS_peak_4754	11.00	lobChIP_FOXA1_robot
chr14	63159092	63159093	MACS_peak_4755	15.00	lobChIP_FOXA1_robot
chr14	63267516	63267517	MACS_peak_4756	8.00	lobChIP_FOXA1_robot
chr14	63283919	63283920	MACS_peak_4757	7.00	lobChIP_FOXA1_robot
chr14	63938106	63938107	MACS_peak_4758	19.00	lobChIP_FOXA1_robot
chr14	63948735	63948736	MACS_peak_4759	7.00	lobChIP_FOXA1_robot
chr14	63949325	63949326	MACS_peak_4760	10.00	lobChIP_FOXA1_robot
chr14	64193459	64193460	MACS_peak_4761	7.00	lobChIP_FOXA1_robot
chr14	64224141	64224142	MACS_peak_4762	4.00	lobChIP_FOXA1_robot
chr14	64390054	64390055	MACS_peak_4763	9.00	lobChIP_FOXA1_robot
chr14	65405414	65405415	MACS_peak_4764	19.00	lobChIP_FOXA1_robot
chr14	65409588	65409589	MACS_peak_4765	13.00	lobChIP_FOXA1_robot
chr14	65693854	65693855	MACS_peak_4766	50.00	lobChIP_FOXA1_robot
chr14	65697364	65697365	MACS_peak_4767	7.00	lobChIP_FOXA1_robot
chr14	65698412	65698413	MACS_peak_4768	5.00	lobChIP_FOXA1_robot
chr14	65705397	65705398	MACS_peak_4769	5.00	lobChIP_FOXA1_robot
chr14	65709682	65709683	MACS_peak_4770	11.00	lobChIP_FOXA1_robot
chr14	65710646	65710647	MACS_peak_4771	5.00	lobChIP_FOXA1_robot
chr14	65721838	65721839	MACS_peak_4772	6.00	lobChIP_FOXA1_robot
chr14	65725239	65725240	MACS_peak_4773	5.00	lobChIP_FOXA1_robot
chr14	65745465	65745466	MACS_peak_4774	6.00	lobChIP_FOXA1_robot
chr14	65764662	65764663	MACS_peak_4775	11.00	lobChIP_FOXA1_robot
chr14	65973771	65973772	MACS_peak_4776	11.00	lobChIP_FOXA1_robot
chr14	66060989	66060990	MACS_peak_4777	6.00	lobChIP_FOXA1_robot
chr14	66143545	66143546	MACS_peak_4778	5.00	lobChIP_FOXA1_robot
chr14	67385608	67385609	MACS_peak_4779	5.00	lobChIP_FOXA1_robot
chr14	67427370	67427371	MACS_peak_4780	5.00	lobChIP_FOXA1_robot
chr14	67466921	67466922	MACS_peak_4781	3.00	lobChIP_FOXA1_robot
chr14	67634620	67634621	MACS_peak_4782	5.00	lobChIP_FOXA1_robot
chr14	67778891	67778892	MACS_peak_4783	10.00	lobChIP_FOXA1_robot
chr14	67780093	67780094	MACS_peak_4784	5.00	lobChIP_FOXA1_robot
chr14	68140448	68140449	MACS_peak_4785	5.00	lobChIP_FOXA1_robot
chr14	68165540	68165541	MACS_peak_4786	5.00	lobChIP_FOXA1_robot
chr14	68230308	68230309	MACS_peak_4787	6.00	lobChIP_FOXA1_robot
chr14	68450099	68450100	MACS_peak_4788	6.00	lobChIP_FOXA1_robot
chr14	68631643	68631644	MACS_peak_4789	3.00	lobChIP_FOXA1_robot
chr14	68639029	68639030	MACS_peak_4790	21.00	lobChIP_FOXA1_robot
chr14	68658556	68658557	MACS_peak_4791	3.00	lobChIP_FOXA1_robot
chr14	68662065	68662066	MACS_peak_4792	8.00	lobChIP_FOXA1_robot
chr14	68721999	68722000	MACS_peak_4793	13.00	lobChIP_FOXA1_robot
chr14	68761798	68761799	MACS_peak_4794	4.00	lobChIP_FOXA1_robot
chr14	68783344	68783345	MACS_peak_4795	8.00	lobChIP_FOXA1_robot
chr14	68816812	68816813	MACS_peak_4796	34.00	lobChIP_FOXA1_robot
chr14	68936324	68936325	MACS_peak_4797	51.00	lobChIP_FOXA1_robot
chr14	68953285	68953286	MACS_peak_4798	5.00	lobChIP_FOXA1_robot
chr14	69065655	69065656	MACS_peak_4799	4.00	lobChIP_FOXA1_robot
chr14	69245750	69245751	MACS_peak_4800	13.00	lobChIP_FOXA1_robot
chr14	69250743	69250744	MACS_peak_4801	8.00	lobChIP_FOXA1_robot
chr14	69560779	69560780	MACS_peak_4802	5.00	lobChIP_FOXA1_robot
chr14	69891050	69891051	MACS_peak_4803	8.00	lobChIP_FOXA1_robot
chr14	69897208	69897209	MACS_peak_4804	7.00	lobChIP_FOXA1_robot
chr14	70110303	70110304	MACS_peak_4805	5.00	lobChIP_FOXA1_robot
chr14	70124907	70124908	MACS_peak_4806	5.00	lobChIP_FOXA1_robot
chr14	70156957	70156958	MACS_peak_4807	5.00	lobChIP_FOXA1_robot
chr14	70276207	70276208	MACS_peak_4808	9.00	lobChIP_FOXA1_robot
chr14	70317593	70317594	MACS_peak_4809	11.00	lobChIP_FOXA1_robot
chr14	70343600	70343601	MACS_peak_4810	4.00	lobChIP_FOXA1_robot
chr14	70354821	70354822	MACS_peak_4811	4.00	lobChIP_FOXA1_robot
chr14	70404208	70404209	MACS_peak_4812	4.00	lobChIP_FOXA1_robot
chr14	70515041	70515042	MACS_peak_4813	7.00	lobChIP_FOXA1_robot
chr14	70785233	70785234	MACS_peak_4814	16.00	lobChIP_FOXA1_robot
chr14	70915578	70915579	MACS_peak_4815	5.00	lobChIP_FOXA1_robot
chr14	71176912	71176913	MACS_peak_4816	5.00	lobChIP_FOXA1_robot
chr14	71220878	71220879	MACS_peak_4817	5.00	lobChIP_FOXA1_robot
chr14	71278512	71278513	MACS_peak_4818	5.00	lobChIP_FOXA1_robot
chr14	71309387	71309388	MACS_peak_4819	4.00	lobChIP_FOXA1_robot
chr14	71431415	71431416	MACS_peak_4820	6.00	lobChIP_FOXA1_robot
chr14	71570804	71570805	MACS_peak_4821	5.00	lobChIP_FOXA1_robot
chr14	71583522	71583523	MACS_peak_4822	5.00	lobChIP_FOXA1_robot
chr14	71803454	71803455	MACS_peak_4823	4.00	lobChIP_FOXA1_robot
chr14	71940755	71940756	MACS_peak_4824	4.00	lobChIP_FOXA1_robot
chr14	72669009	72669010	MACS_peak_4825	4.00	lobChIP_FOXA1_robot
chr14	73243774	73243775	MACS_peak_4826	18.00	lobChIP_FOXA1_robot
chr14	73248751	73248752	MACS_peak_4827	5.00	lobChIP_FOXA1_robot
chr14	73524276	73524277	MACS_peak_4828	4.00	lobChIP_FOXA1_robot
chr14	73613663	73613664	MACS_peak_4829	4.00	lobChIP_FOXA1_robot
chr14	73616580	73616581	MACS_peak_4830	11.00	lobChIP_FOXA1_robot
chr14	73898938	73898939	MACS_peak_4831	5.00	lobChIP_FOXA1_robot
chr14	73922528	73922529	MACS_peak_4832	4.00	lobChIP_FOXA1_robot
chr14	73929053	73929054	MACS_peak_4833	6.00	lobChIP_FOXA1_robot
chr14	74130808	74130809	MACS_peak_4834	5.00	lobChIP_FOXA1_robot
chr14	74230334	74230335	MACS_peak_4835	8.00	lobChIP_FOXA1_robot
chr14	74238905	74238906	MACS_peak_4836	4.00	lobChIP_FOXA1_robot
chr14	74252651	74252652	MACS_peak_4837	5.00	lobChIP_FOXA1_robot
chr14	74305044	74305045	MACS_peak_4838	5.00	lobChIP_FOXA1_robot
chr14	74464005	74464006	MACS_peak_4839	7.00	lobChIP_FOXA1_robot
chr14	74551428	74551429	MACS_peak_4840	5.00	lobChIP_FOXA1_robot
chr14	74716534	74716535	MACS_peak_4841	5.00	lobChIP_FOXA1_robot
chr14	75100190	75100191	MACS_peak_4842	5.00	lobChIP_FOXA1_robot
chr14	75365703	75365704	MACS_peak_4843	4.00	lobChIP_FOXA1_robot
chr14	75394112	75394113	MACS_peak_4844	5.00	lobChIP_FOXA1_robot
chr14	75412590	75412591	MACS_peak_4845	12.00	lobChIP_FOXA1_robot
chr14	75620132	75620133	MACS_peak_4846	7.00	lobChIP_FOXA1_robot
chr14	75663411	75663412	MACS_peak_4847	13.00	lobChIP_FOXA1_robot
chr14	75750017	75750018	MACS_peak_4848	5.00	lobChIP_FOXA1_robot
chr14	75795587	75795588	MACS_peak_4849	4.00	lobChIP_FOXA1_robot
chr14	75797024	75797025	MACS_peak_4850	3.00	lobChIP_FOXA1_robot
chr14	75987911	75987912	MACS_peak_4851	7.00	lobChIP_FOXA1_robot
chr14	76002494	76002495	MACS_peak_4852	3.00	lobChIP_FOXA1_robot
chr14	76114785	76114786	MACS_peak_4853	15.00	lobChIP_FOXA1_robot
chr14	76116746	76116747	MACS_peak_4854	4.00	lobChIP_FOXA1_robot
chr14	76178127	76178128	MACS_peak_4855	4.00	lobChIP_FOXA1_robot
chr14	76292610	76292611	MACS_peak_4856	9.00	lobChIP_FOXA1_robot
chr14	76324957	76324958	MACS_peak_4857	6.00	lobChIP_FOXA1_robot
chr14	77154404	77154405	MACS_peak_4858	14.00	lobChIP_FOXA1_robot
chr14	77435157	77435158	MACS_peak_4859	5.00	lobChIP_FOXA1_robot
chr14	77495245	77495246	MACS_peak_4860	8.00	lobChIP_FOXA1_robot
chr14	77500704	77500705	MACS_peak_4861	34.00	lobChIP_FOXA1_robot
chr14	77968579	77968580	MACS_peak_4862	15.00	lobChIP_FOXA1_robot
chr14	78330683	78330684	MACS_peak_4863	3.00	lobChIP_FOXA1_robot
chr14	80469715	80469716	MACS_peak_4864	28.00	lobChIP_FOXA1_robot
chr14	80935143	80935144	MACS_peak_4865	90.00	lobChIP_FOXA1_robot
chr14	81044136	81044137	MACS_peak_4866	5.00	lobChIP_FOXA1_robot
chr14	81112770	81112771	MACS_peak_4867	3.00	lobChIP_FOXA1_robot
chr14	81769761	81769762	MACS_peak_4868	13.00	lobChIP_FOXA1_robot
chr14	81983920	81983921	MACS_peak_4869	11.00	lobChIP_FOXA1_robot
chr14	82003729	82003730	MACS_peak_4870	11.00	lobChIP_FOXA1_robot
chr14	82248375	82248376	MACS_peak_4871	3.00	lobChIP_FOXA1_robot
chr14	82452607	82452608	MACS_peak_4872	6.00	lobChIP_FOXA1_robot
chr14	85938130	85938131	MACS_peak_4873	6.00	lobChIP_FOXA1_robot
chr14	87175339	87175340	MACS_peak_4874	8.00	lobChIP_FOXA1_robot
chr14	87189467	87189468	MACS_peak_4875	16.00	lobChIP_FOXA1_robot
chr14	87309543	87309544	MACS_peak_4876	4.00	lobChIP_FOXA1_robot
chr14	87358993	87358994	MACS_peak_4877	14.00	lobChIP_FOXA1_robot
chr14	87364121	87364122	MACS_peak_4878	7.00	lobChIP_FOXA1_robot
chr14	87520267	87520268	MACS_peak_4879	4.00	lobChIP_FOXA1_robot
chr14	87900556	87900557	MACS_peak_4880	10.00	lobChIP_FOXA1_robot
chr14	88490765	88490766	MACS_peak_4881	9.00	lobChIP_FOXA1_robot
chr14	88510697	88510698	MACS_peak_4882	31.00	lobChIP_FOXA1_robot
chr14	88511340	88511341	MACS_peak_4883	6.00	lobChIP_FOXA1_robot
chr14	88855307	88855308	MACS_peak_4884	5.00	lobChIP_FOXA1_robot
chr14	88929999	88930000	MACS_peak_4885	5.00	lobChIP_FOXA1_robot
chr14	88958912	88958913	MACS_peak_4886	6.00	lobChIP_FOXA1_robot
chr14	89019333	89019334	MACS_peak_4887	7.00	lobChIP_FOXA1_robot
chr14	89080227	89080228	MACS_peak_4888	7.00	lobChIP_FOXA1_robot
chr14	89099055	89099056	MACS_peak_4889	5.00	lobChIP_FOXA1_robot
chr14	89507152	89507153	MACS_peak_4890	8.00	lobChIP_FOXA1_robot
chr14	89507768	89507769	MACS_peak_4891	12.00	lobChIP_FOXA1_robot
chr14	89637627	89637628	MACS_peak_4892	7.00	lobChIP_FOXA1_robot
chr14	89642631	89642632	MACS_peak_4893	9.00	lobChIP_FOXA1_robot
chr14	89657655	89657656	MACS_peak_4894	3.00	lobChIP_FOXA1_robot
chr14	89680795	89680796	MACS_peak_4895	6.00	lobChIP_FOXA1_robot
chr14	89698968	89698969	MACS_peak_4896	21.00	lobChIP_FOXA1_robot
chr14	89714242	89714243	MACS_peak_4897	11.00	lobChIP_FOXA1_robot
chr14	89806605	89806606	MACS_peak_4898	5.00	lobChIP_FOXA1_robot
chr14	89818144	89818145	MACS_peak_4899	91.00	lobChIP_FOXA1_robot
chr14	89857561	89857562	MACS_peak_4900	5.00	lobChIP_FOXA1_robot
chr14	89925076	89925077	MACS_peak_4901	3.00	lobChIP_FOXA1_robot
chr14	89960335	89960336	MACS_peak_4902	7.00	lobChIP_FOXA1_robot
chr14	90006546	90006547	MACS_peak_4903	11.00	lobChIP_FOXA1_robot
chr14	90055533	90055534	MACS_peak_4904	6.00	lobChIP_FOXA1_robot
chr14	90377564	90377565	MACS_peak_4905	9.00	lobChIP_FOXA1_robot
chr14	90980067	90980068	MACS_peak_4906	29.00	lobChIP_FOXA1_robot
chr14	91622868	91622869	MACS_peak_4907	14.00	lobChIP_FOXA1_robot
chr14	91827767	91827768	MACS_peak_4908	3.00	lobChIP_FOXA1_robot
chr14	91986061	91986062	MACS_peak_4909	4.00	lobChIP_FOXA1_robot
chr14	92001348	92001349	MACS_peak_4910	4.00	lobChIP_FOXA1_robot
chr14	92246959	92246960	MACS_peak_4911	3.00	lobChIP_FOXA1_robot
chr14	92814663	92814664	MACS_peak_4912	8.00	lobChIP_FOXA1_robot
chr14	93013697	93013698	MACS_peak_4913	18.00	lobChIP_FOXA1_robot
chr14	93023670	93023671	MACS_peak_4914	4.00	lobChIP_FOXA1_robot
chr14	93160509	93160510	MACS_peak_4915	11.00	lobChIP_FOXA1_robot
chr14	93190840	93190841	MACS_peak_4916	4.00	lobChIP_FOXA1_robot
chr14	93451398	93451399	MACS_peak_4917	6.00	lobChIP_FOXA1_robot
chr14	93644516	93644517	MACS_peak_4918	9.00	lobChIP_FOXA1_robot
chr14	94244975	94244976	MACS_peak_4919	15.00	lobChIP_FOXA1_robot
chr14	94266207	94266208	MACS_peak_4920	4.00	lobChIP_FOXA1_robot
chr14	94456459	94456460	MACS_peak_4921	4.00	lobChIP_FOXA1_robot
chr14	94494863	94494864	MACS_peak_4922	5.00	lobChIP_FOXA1_robot
chr14	94759614	94759615	MACS_peak_4923	5.00	lobChIP_FOXA1_robot
chr14	94786774	94786775	MACS_peak_4924	4.00	lobChIP_FOXA1_robot
chr14	94789782	94789783	MACS_peak_4925	10.00	lobChIP_FOXA1_robot
chr14	94806304	94806305	MACS_peak_4926	5.00	lobChIP_FOXA1_robot
chr14	94807003	94807004	MACS_peak_4927	9.00	lobChIP_FOXA1_robot
chr14	94823197	94823198	MACS_peak_4928	5.00	lobChIP_FOXA1_robot
chr14	94855013	94855014	MACS_peak_4929	4.00	lobChIP_FOXA1_robot
chr14	94861278	94861279	MACS_peak_4930	3.00	lobChIP_FOXA1_robot
chr14	94862444	94862445	MACS_peak_4931	30.00	lobChIP_FOXA1_robot
chr14	94866945	94866946	MACS_peak_4932	5.00	lobChIP_FOXA1_robot
chr14	94893803	94893804	MACS_peak_4933	3.00	lobChIP_FOXA1_robot
chr14	94919181	94919182	MACS_peak_4934	19.00	lobChIP_FOXA1_robot
chr14	94922490	94922491	MACS_peak_4935	6.00	lobChIP_FOXA1_robot
chr14	94923174	94923175	MACS_peak_4936	4.00	lobChIP_FOXA1_robot
chr14	94929778	94929779	MACS_peak_4937	5.00	lobChIP_FOXA1_robot
chr14	94968401	94968402	MACS_peak_4938	18.00	lobChIP_FOXA1_robot
chr14	94988744	94988745	MACS_peak_4939	7.00	lobChIP_FOXA1_robot
chr14	95030796	95030797	MACS_peak_4940	8.00	lobChIP_FOXA1_robot
chr14	95039284	95039285	MACS_peak_4941	12.00	lobChIP_FOXA1_robot
chr14	95060446	95060447	MACS_peak_4942	24.00	lobChIP_FOXA1_robot
chr14	95065490	95065491	MACS_peak_4943	5.00	lobChIP_FOXA1_robot
chr14	95067217	95067218	MACS_peak_4944	17.00	lobChIP_FOXA1_robot
chr14	95103275	95103276	MACS_peak_4945	19.00	lobChIP_FOXA1_robot
chr14	95293337	95293338	MACS_peak_4946	5.00	lobChIP_FOXA1_robot
chr14	95740079	95740080	MACS_peak_4947	4.00	lobChIP_FOXA1_robot
chr14	95762082	95762083	MACS_peak_4948	5.00	lobChIP_FOXA1_robot
chr14	95784728	95784729	MACS_peak_4949	3.00	lobChIP_FOXA1_robot
chr14	95806092	95806093	MACS_peak_4950	5.00	lobChIP_FOXA1_robot
chr14	95910590	95910591	MACS_peak_4951	15.00	lobChIP_FOXA1_robot
chr14	95989747	95989748	MACS_peak_4952	8.00	lobChIP_FOXA1_robot
chr14	95991432	95991433	MACS_peak_4953	9.00	lobChIP_FOXA1_robot
chr14	96003947	96003948	MACS_peak_4954	16.00	lobChIP_FOXA1_robot
chr14	96662218	96662219	MACS_peak_4955	5.00	lobChIP_FOXA1_robot
chr14	96811113	96811114	MACS_peak_4956	4.00	lobChIP_FOXA1_robot
chr14	96828694	96828695	MACS_peak_4957	6.00	lobChIP_FOXA1_robot
chr14	96928705	96928706	MACS_peak_4958	8.00	lobChIP_FOXA1_robot
chr14	96935328	96935329	MACS_peak_4959	4.00	lobChIP_FOXA1_robot
chr14	99782637	99782638	MACS_peak_4960	8.00	lobChIP_FOXA1_robot
chr14	100021351	100021352	MACS_peak_4961	4.00	lobChIP_FOXA1_robot
chr14	100250931	100250932	MACS_peak_4962	9.00	lobChIP_FOXA1_robot
chr14	100611654	100611655	MACS_peak_4963	4.00	lobChIP_FOXA1_robot
chr14	100671084	100671085	MACS_peak_4964	8.00	lobChIP_FOXA1_robot
chr14	100844367	100844368	MACS_peak_4965	3.00	lobChIP_FOXA1_robot
chr14	100963896	100963897	MACS_peak_4966	5.00	lobChIP_FOXA1_robot
chr14	101160530	101160531	MACS_peak_4967	5.00	lobChIP_FOXA1_robot
chr14	101216826	101216827	MACS_peak_4968	15.00	lobChIP_FOXA1_robot
chr14	101228649	101228650	MACS_peak_4969	10.00	lobChIP_FOXA1_robot
chr14	101283813	101283814	MACS_peak_4970	25.00	lobChIP_FOXA1_robot
chr14	101287474	101287475	MACS_peak_4971	5.00	lobChIP_FOXA1_robot
chr14	101313068	101313069	MACS_peak_4972	6.00	lobChIP_FOXA1_robot
chr14	101507379	101507380	MACS_peak_4973	11.00	lobChIP_FOXA1_robot
chr14	101693128	101693129	MACS_peak_4974	5.00	lobChIP_FOXA1_robot
chr14	101755055	101755056	MACS_peak_4975	10.00	lobChIP_FOXA1_robot
chr14	101755755	101755756	MACS_peak_4976	15.00	lobChIP_FOXA1_robot
chr14	101801879	101801880	MACS_peak_4977	8.00	lobChIP_FOXA1_robot
chr14	101879242	101879243	MACS_peak_4978	15.00	lobChIP_FOXA1_robot
chr14	102446555	102446556	MACS_peak_4979	3.00	lobChIP_FOXA1_robot
chr14	102651955	102651956	MACS_peak_4980	9.00	lobChIP_FOXA1_robot
chr14	102787956	102787957	MACS_peak_4981	4.00	lobChIP_FOXA1_robot
chr14	102931328	102931329	MACS_peak_4982	5.00	lobChIP_FOXA1_robot
chr14	102948377	102948378	MACS_peak_4983	5.00	lobChIP_FOXA1_robot
chr14	103121739	103121740	MACS_peak_4984	4.00	lobChIP_FOXA1_robot
chr14	103150727	103150728	MACS_peak_4985	5.00	lobChIP_FOXA1_robot
chr14	103157402	103157403	MACS_peak_4986	8.00	lobChIP_FOXA1_robot
chr14	103172530	103172531	MACS_peak_4987	4.00	lobChIP_FOXA1_robot
chr14	103261694	103261695	MACS_peak_4988	4.00	lobChIP_FOXA1_robot
chr14	103340057	103340058	MACS_peak_4989	30.00	lobChIP_FOXA1_robot
chr14	105267321	105267322	MACS_peak_4990	5.00	lobChIP_FOXA1_robot
chr14	107253682	107253683	MACS_peak_4991	4.00	lobChIP_FOXA1_robot
chr15	20457920	20457921	MACS_peak_4992	5.00	lobChIP_FOXA1_robot
chr15	23028111	23028112	MACS_peak_4993	5.00	lobChIP_FOXA1_robot
chr15	23935899	23935900	MACS_peak_4994	5.00	lobChIP_FOXA1_robot
chr15	25102538	25102539	MACS_peak_4995	3.00	lobChIP_FOXA1_robot
chr15	25208186	25208187	MACS_peak_4996	9.00	lobChIP_FOXA1_robot
chr15	25612400	25612401	MACS_peak_4997	4.00	lobChIP_FOXA1_robot
chr15	27553019	27553020	MACS_peak_4998	5.00	lobChIP_FOXA1_robot
chr15	27553506	27553507	MACS_peak_4999	3.00	lobChIP_FOXA1_robot
chr15	29968087	29968088	MACS_peak_5000	4.00	lobChIP_FOXA1_robot
chr15	30081588	30081589	MACS_peak_5001	3.00	lobChIP_FOXA1_robot
chr15	30106445	30106446	MACS_peak_5002	6.00	lobChIP_FOXA1_robot
chr15	30115420	30115421	MACS_peak_5003	5.00	lobChIP_FOXA1_robot
chr15	31157571	31157572	MACS_peak_5004	3.00	lobChIP_FOXA1_robot
chr15	31381060	31381061	MACS_peak_5005	6.00	lobChIP_FOXA1_robot
chr15	31450104	31450105	MACS_peak_5006	13.00	lobChIP_FOXA1_robot
chr15	31586011	31586012	MACS_peak_5007	11.00	lobChIP_FOXA1_robot
chr15	31588931	31588932	MACS_peak_5008	14.00	lobChIP_FOXA1_robot
chr15	31698026	31698027	MACS_peak_5009	7.00	lobChIP_FOXA1_robot
chr15	32175888	32175889	MACS_peak_5010	3.00	lobChIP_FOXA1_robot
chr15	32951895	32951896	MACS_peak_5011	10.00	lobChIP_FOXA1_robot
chr15	32980445	32980446	MACS_peak_5012	5.00	lobChIP_FOXA1_robot
chr15	32992924	32992925	MACS_peak_5013	6.00	lobChIP_FOXA1_robot
chr15	33001756	33001757	MACS_peak_5014	7.00	lobChIP_FOXA1_robot
chr15	33069765	33069766	MACS_peak_5015	4.00	lobChIP_FOXA1_robot
chr15	33131217	33131218	MACS_peak_5016	5.00	lobChIP_FOXA1_robot
chr15	33145322	33145323	MACS_peak_5017	9.00	lobChIP_FOXA1_robot
chr15	34450697	34450698	MACS_peak_5018	10.00	lobChIP_FOXA1_robot
chr15	34593498	34593499	MACS_peak_5019	5.00	lobChIP_FOXA1_robot
chr15	34864725	34864726	MACS_peak_5020	4.00	lobChIP_FOXA1_robot
chr15	35798714	35798715	MACS_peak_5021	6.00	lobChIP_FOXA1_robot
chr15	36028554	36028555	MACS_peak_5022	4.00	lobChIP_FOXA1_robot
chr15	36799138	36799139	MACS_peak_5023	77.00	lobChIP_FOXA1_robot
chr15	37107149	37107150	MACS_peak_5024	4.00	lobChIP_FOXA1_robot
chr15	37204416	37204417	MACS_peak_5025	3.00	lobChIP_FOXA1_robot
chr15	37204829	37204830	MACS_peak_5026	5.00	lobChIP_FOXA1_robot
chr15	37269982	37269983	MACS_peak_5027	8.00	lobChIP_FOXA1_robot
chr15	37301672	37301673	MACS_peak_5028	9.00	lobChIP_FOXA1_robot
chr15	37312066	37312067	MACS_peak_5029	4.00	lobChIP_FOXA1_robot
chr15	37327762	37327763	MACS_peak_5030	4.00	lobChIP_FOXA1_robot
chr15	37332613	37332614	MACS_peak_5031	7.00	lobChIP_FOXA1_robot
chr15	37338332	37338333	MACS_peak_5032	5.00	lobChIP_FOXA1_robot
chr15	37511723	37511724	MACS_peak_5033	4.00	lobChIP_FOXA1_robot
chr15	38002119	38002120	MACS_peak_5034	10.00	lobChIP_FOXA1_robot
chr15	38509177	38509178	MACS_peak_5035	7.00	lobChIP_FOXA1_robot
chr15	39165593	39165594	MACS_peak_5036	9.00	lobChIP_FOXA1_robot
chr15	39420632	39420633	MACS_peak_5037	9.00	lobChIP_FOXA1_robot
chr15	39503863	39503864	MACS_peak_5038	10.00	lobChIP_FOXA1_robot
chr15	39566239	39566240	MACS_peak_5039	46.00	lobChIP_FOXA1_robot
chr15	39652730	39652731	MACS_peak_5040	6.00	lobChIP_FOXA1_robot
chr15	39656124	39656125	MACS_peak_5041	6.00	lobChIP_FOXA1_robot
chr15	39727378	39727379	MACS_peak_5042	29.00	lobChIP_FOXA1_robot
chr15	39748035	39748036	MACS_peak_5043	10.00	lobChIP_FOXA1_robot
chr15	40022943	40022944	MACS_peak_5044	4.00	lobChIP_FOXA1_robot
chr15	40079985	40079986	MACS_peak_5045	11.00	lobChIP_FOXA1_robot
chr15	40213837	40213838	MACS_peak_5046	4.00	lobChIP_FOXA1_robot
chr15	40294718	40294719	MACS_peak_5047	4.00	lobChIP_FOXA1_robot
chr15	40373998	40373999	MACS_peak_5048	7.00	lobChIP_FOXA1_robot
chr15	40384656	40384657	MACS_peak_5049	4.00	lobChIP_FOXA1_robot
chr15	40391108	40391109	MACS_peak_5050	16.00	lobChIP_FOXA1_robot
chr15	40398830	40398831	MACS_peak_5051	6.00	lobChIP_FOXA1_robot
chr15	40942261	40942262	MACS_peak_5052	7.00	lobChIP_FOXA1_robot
chr15	41115950	41115951	MACS_peak_5053	13.00	lobChIP_FOXA1_robot
chr15	41237267	41237268	MACS_peak_5054	19.00	lobChIP_FOXA1_robot
chr15	41313238	41313239	MACS_peak_5055	4.00	lobChIP_FOXA1_robot
chr15	41417601	41417602	MACS_peak_5056	7.00	lobChIP_FOXA1_robot
chr15	41505341	41505342	MACS_peak_5057	4.00	lobChIP_FOXA1_robot
chr15	41548871	41548872	MACS_peak_5058	8.00	lobChIP_FOXA1_robot
chr15	41627741	41627742	MACS_peak_5059	4.00	lobChIP_FOXA1_robot
chr15	41860837	41860838	MACS_peak_5060	4.00	lobChIP_FOXA1_robot
chr15	42072601	42072602	MACS_peak_5061	4.00	lobChIP_FOXA1_robot
chr15	42220421	42220422	MACS_peak_5062	11.00	lobChIP_FOXA1_robot
chr15	42253248	42253249	MACS_peak_5063	5.00	lobChIP_FOXA1_robot
chr15	42338937	42338938	MACS_peak_5064	4.00	lobChIP_FOXA1_robot
chr15	42565722	42565723	MACS_peak_5065	5.00	lobChIP_FOXA1_robot
chr15	42578764	42578765	MACS_peak_5066	7.00	lobChIP_FOXA1_robot
chr15	44061965	44061966	MACS_peak_5067	5.00	lobChIP_FOXA1_robot
chr15	44134787	44134788	MACS_peak_5068	4.00	lobChIP_FOXA1_robot
chr15	44491815	44491816	MACS_peak_5069	4.00	lobChIP_FOXA1_robot
chr15	44720659	44720660	MACS_peak_5070	7.00	lobChIP_FOXA1_robot
chr15	44880996	44880997	MACS_peak_5071	12.00	lobChIP_FOXA1_robot
chr15	45104468	45104469	MACS_peak_5072	6.00	lobChIP_FOXA1_robot
chr15	45205398	45205399	MACS_peak_5073	18.00	lobChIP_FOXA1_robot
chr15	45272135	45272136	MACS_peak_5074	4.00	lobChIP_FOXA1_robot
chr15	45382018	45382019	MACS_peak_5075	4.00	lobChIP_FOXA1_robot
chr15	45492733	45492734	MACS_peak_5076	4.00	lobChIP_FOXA1_robot
chr15	45626893	45626894	MACS_peak_5077	5.00	lobChIP_FOXA1_robot
chr15	45679752	45679753	MACS_peak_5078	34.00	lobChIP_FOXA1_robot
chr15	45814047	45814048	MACS_peak_5079	4.00	lobChIP_FOXA1_robot
chr15	45933777	45933778	MACS_peak_5080	12.00	lobChIP_FOXA1_robot
chr15	46087180	46087181	MACS_peak_5081	8.00	lobChIP_FOXA1_robot
chr15	47416754	47416755	MACS_peak_5082	10.00	lobChIP_FOXA1_robot
chr15	47719791	47719792	MACS_peak_5083	7.00	lobChIP_FOXA1_robot
chr15	48647060	48647061	MACS_peak_5084	5.00	lobChIP_FOXA1_robot
chr15	48761313	48761314	MACS_peak_5085	12.00	lobChIP_FOXA1_robot
chr15	49242349	49242350	MACS_peak_5086	4.00	lobChIP_FOXA1_robot
chr15	49324507	49324508	MACS_peak_5087	8.00	lobChIP_FOXA1_robot
chr15	49328145	49328146	MACS_peak_5088	10.00	lobChIP_FOXA1_robot
chr15	50379487	50379488	MACS_peak_5089	7.00	lobChIP_FOXA1_robot
chr15	50636483	50636484	MACS_peak_5090	11.00	lobChIP_FOXA1_robot
chr15	51058885	51058886	MACS_peak_5091	7.00	lobChIP_FOXA1_robot
chr15	51352455	51352456	MACS_peak_5092	12.00	lobChIP_FOXA1_robot
chr15	51580992	51580993	MACS_peak_5093	4.00	lobChIP_FOXA1_robot
chr15	51604591	51604592	MACS_peak_5094	6.00	lobChIP_FOXA1_robot
chr15	51606092	51606093	MACS_peak_5095	4.00	lobChIP_FOXA1_robot
chr15	51786301	51786302	MACS_peak_5096	10.00	lobChIP_FOXA1_robot
chr15	52199859	52199860	MACS_peak_5097	4.00	lobChIP_FOXA1_robot
chr15	52262664	52262665	MACS_peak_5098	7.00	lobChIP_FOXA1_robot
chr15	52301412	52301413	MACS_peak_5099	8.00	lobChIP_FOXA1_robot
chr15	52346737	52346738	MACS_peak_5100	7.00	lobChIP_FOXA1_robot
chr15	52408517	52408518	MACS_peak_5101	5.00	lobChIP_FOXA1_robot
chr15	52543564	52543565	MACS_peak_5102	4.00	lobChIP_FOXA1_robot
chr15	52580733	52580734	MACS_peak_5103	22.00	lobChIP_FOXA1_robot
chr15	52907654	52907655	MACS_peak_5104	5.00	lobChIP_FOXA1_robot
chr15	53005850	53005851	MACS_peak_5105	4.00	lobChIP_FOXA1_robot
chr15	53726592	53726593	MACS_peak_5106	7.00	lobChIP_FOXA1_robot
chr15	53739402	53739403	MACS_peak_5107	6.00	lobChIP_FOXA1_robot
chr15	53744924	53744925	MACS_peak_5108	4.00	lobChIP_FOXA1_robot
chr15	53745894	53745895	MACS_peak_5109	24.00	lobChIP_FOXA1_robot
chr15	53747448	53747449	MACS_peak_5110	42.00	lobChIP_FOXA1_robot
chr15	54124321	54124322	MACS_peak_5111	34.00	lobChIP_FOXA1_robot
chr15	54125703	54125704	MACS_peak_5112	21.00	lobChIP_FOXA1_robot
chr15	54136639	54136640	MACS_peak_5113	6.00	lobChIP_FOXA1_robot
chr15	54162145	54162146	MACS_peak_5114	6.00	lobChIP_FOXA1_robot
chr15	55502139	55502140	MACS_peak_5115	4.00	lobChIP_FOXA1_robot
chr15	55542765	55542766	MACS_peak_5116	8.00	lobChIP_FOXA1_robot
chr15	55553196	55553197	MACS_peak_5117	5.00	lobChIP_FOXA1_robot
chr15	55729913	55729914	MACS_peak_5118	4.00	lobChIP_FOXA1_robot
chr15	55953435	55953436	MACS_peak_5119	3.00	lobChIP_FOXA1_robot
chr15	56269583	56269584	MACS_peak_5120	4.00	lobChIP_FOXA1_robot
chr15	56480124	56480125	MACS_peak_5121	5.00	lobChIP_FOXA1_robot
chr15	56537328	56537329	MACS_peak_5122	4.00	lobChIP_FOXA1_robot
chr15	57065105	57065106	MACS_peak_5123	4.00	lobChIP_FOXA1_robot
chr15	57150102	57150103	MACS_peak_5124	4.00	lobChIP_FOXA1_robot
chr15	57256238	57256239	MACS_peak_5125	4.00	lobChIP_FOXA1_robot
chr15	57326128	57326129	MACS_peak_5126	5.00	lobChIP_FOXA1_robot
chr15	57599994	57599995	MACS_peak_5127	8.00	lobChIP_FOXA1_robot
chr15	57683921	57683922	MACS_peak_5128	8.00	lobChIP_FOXA1_robot
chr15	57770154	57770155	MACS_peak_5129	5.00	lobChIP_FOXA1_robot
chr15	57789536	57789537	MACS_peak_5130	14.00	lobChIP_FOXA1_robot
chr15	57797437	57797438	MACS_peak_5131	6.00	lobChIP_FOXA1_robot
chr15	58025171	58025172	MACS_peak_5132	4.00	lobChIP_FOXA1_robot
chr15	58521835	58521836	MACS_peak_5133	6.00	lobChIP_FOXA1_robot
chr15	58578258	58578259	MACS_peak_5134	6.00	lobChIP_FOXA1_robot
chr15	58624403	58624404	MACS_peak_5135	3.00	lobChIP_FOXA1_robot
chr15	58747382	58747383	MACS_peak_5136	13.00	lobChIP_FOXA1_robot
chr15	58756247	58756248	MACS_peak_5137	9.00	lobChIP_FOXA1_robot
chr15	58759908	58759909	MACS_peak_5138	5.00	lobChIP_FOXA1_robot
chr15	58761221	58761222	MACS_peak_5139	10.00	lobChIP_FOXA1_robot
chr15	58820613	58820614	MACS_peak_5140	6.00	lobChIP_FOXA1_robot
chr15	58844085	58844086	MACS_peak_5141	9.00	lobChIP_FOXA1_robot
chr15	58918714	58918715	MACS_peak_5142	3.00	lobChIP_FOXA1_robot
chr15	59361800	59361801	MACS_peak_5143	12.00	lobChIP_FOXA1_robot
chr15	59458142	59458143	MACS_peak_5144	6.00	lobChIP_FOXA1_robot
chr15	59548392	59548393	MACS_peak_5145	4.00	lobChIP_FOXA1_robot
chr15	59569282	59569283	MACS_peak_5146	11.00	lobChIP_FOXA1_robot
chr15	59631665	59631666	MACS_peak_5147	5.00	lobChIP_FOXA1_robot
chr15	59653263	59653264	MACS_peak_5148	6.00	lobChIP_FOXA1_robot
chr15	59659062	59659063	MACS_peak_5149	12.00	lobChIP_FOXA1_robot
chr15	59686274	59686275	MACS_peak_5150	3.00	lobChIP_FOXA1_robot
chr15	59846523	59846524	MACS_peak_5151	58.00	lobChIP_FOXA1_robot
chr15	59914670	59914671	MACS_peak_5152	3.00	lobChIP_FOXA1_robot
chr15	60327383	60327384	MACS_peak_5153	3.00	lobChIP_FOXA1_robot
chr15	60386078	60386079	MACS_peak_5154	6.00	lobChIP_FOXA1_robot
chr15	60547886	60547887	MACS_peak_5155	5.00	lobChIP_FOXA1_robot
chr15	60575797	60575798	MACS_peak_5156	4.00	lobChIP_FOXA1_robot
chr15	60666980	60666981	MACS_peak_5157	7.00	lobChIP_FOXA1_robot
chr15	60672173	60672174	MACS_peak_5158	8.00	lobChIP_FOXA1_robot
chr15	60685403	60685404	MACS_peak_5159	7.00	lobChIP_FOXA1_robot
chr15	60785571	60785572	MACS_peak_5160	5.00	lobChIP_FOXA1_robot
chr15	60943647	60943648	MACS_peak_5161	6.00	lobChIP_FOXA1_robot
chr15	61027849	61027850	MACS_peak_5162	8.00	lobChIP_FOXA1_robot
chr15	62199888	62199889	MACS_peak_5163	4.00	lobChIP_FOXA1_robot
chr15	62722336	62722337	MACS_peak_5164	5.00	lobChIP_FOXA1_robot
chr15	62723805	62723806	MACS_peak_5165	8.00	lobChIP_FOXA1_robot
chr15	62801510	62801511	MACS_peak_5166	7.00	lobChIP_FOXA1_robot
chr15	62862512	62862513	MACS_peak_5167	58.00	lobChIP_FOXA1_robot
chr15	63049087	63049088	MACS_peak_5168	5.00	lobChIP_FOXA1_robot
chr15	63150771	63150772	MACS_peak_5169	7.00	lobChIP_FOXA1_robot
chr15	63178927	63178928	MACS_peak_5170	7.00	lobChIP_FOXA1_robot
chr15	63187511	63187512	MACS_peak_5171	7.00	lobChIP_FOXA1_robot
chr15	63189296	63189297	MACS_peak_5172	6.00	lobChIP_FOXA1_robot
chr15	63303701	63303702	MACS_peak_5173	5.00	lobChIP_FOXA1_robot
chr15	63341916	63341917	MACS_peak_5174	3.00	lobChIP_FOXA1_robot
chr15	63400271	63400272	MACS_peak_5175	10.00	lobChIP_FOXA1_robot
chr15	63478402	63478403	MACS_peak_5176	5.00	lobChIP_FOXA1_robot
chr15	63539481	63539482	MACS_peak_5177	5.00	lobChIP_FOXA1_robot
chr15	63553145	63553146	MACS_peak_5178	5.00	lobChIP_FOXA1_robot
chr15	63574122	63574123	MACS_peak_5179	5.00	lobChIP_FOXA1_robot
chr15	63694514	63694515	MACS_peak_5180	3.00	lobChIP_FOXA1_robot
chr15	63765380	63765381	MACS_peak_5181	7.00	lobChIP_FOXA1_robot
chr15	63766420	63766421	MACS_peak_5182	16.00	lobChIP_FOXA1_robot
chr15	63806801	63806802	MACS_peak_5183	6.00	lobChIP_FOXA1_robot
chr15	64010917	64010918	MACS_peak_5184	7.00	lobChIP_FOXA1_robot
chr15	64365223	64365224	MACS_peak_5185	15.00	lobChIP_FOXA1_robot
chr15	64540304	64540305	MACS_peak_5186	7.00	lobChIP_FOXA1_robot
chr15	64570131	64570132	MACS_peak_5187	5.00	lobChIP_FOXA1_robot
chr15	64748856	64748857	MACS_peak_5188	4.00	lobChIP_FOXA1_robot
chr15	64943940	64943941	MACS_peak_5189	6.00	lobChIP_FOXA1_robot
chr15	64985547	64985548	MACS_peak_5190	4.00	lobChIP_FOXA1_robot
chr15	65336674	65336675	MACS_peak_5191	4.00	lobChIP_FOXA1_robot
chr15	65337653	65337654	MACS_peak_5192	5.00	lobChIP_FOXA1_robot
chr15	65464012	65464013	MACS_peak_5193	6.00	lobChIP_FOXA1_robot
chr15	65597123	65597124	MACS_peak_5194	4.00	lobChIP_FOXA1_robot
chr15	65643980	65643981	MACS_peak_5195	5.00	lobChIP_FOXA1_robot
chr15	65660333	65660334	MACS_peak_5196	8.00	lobChIP_FOXA1_robot
chr15	66016922	66016923	MACS_peak_5197	6.00	lobChIP_FOXA1_robot
chr15	66059415	66059416	MACS_peak_5198	3.00	lobChIP_FOXA1_robot
chr15	66080265	66080266	MACS_peak_5199	8.00	lobChIP_FOXA1_robot
chr15	66093852	66093853	MACS_peak_5200	4.00	lobChIP_FOXA1_robot
chr15	66125130	66125131	MACS_peak_5201	35.00	lobChIP_FOXA1_robot
chr15	66335859	66335860	MACS_peak_5202	5.00	lobChIP_FOXA1_robot
chr15	66797470	66797471	MACS_peak_5203	4.00	lobChIP_FOXA1_robot
chr15	66909917	66909918	MACS_peak_5204	7.00	lobChIP_FOXA1_robot
chr15	67224556	67224557	MACS_peak_5205	7.00	lobChIP_FOXA1_robot
chr15	67340878	67340879	MACS_peak_5206	8.00	lobChIP_FOXA1_robot
chr15	67341291	67341292	MACS_peak_5207	7.00	lobChIP_FOXA1_robot
chr15	67356814	67356815	MACS_peak_5208	7.00	lobChIP_FOXA1_robot
chr15	67366590	67366591	MACS_peak_5209	5.00	lobChIP_FOXA1_robot
chr15	67427961	67427962	MACS_peak_5210	20.00	lobChIP_FOXA1_robot
chr15	67439495	67439496	MACS_peak_5211	13.00	lobChIP_FOXA1_robot
chr15	67443047	67443048	MACS_peak_5212	7.00	lobChIP_FOXA1_robot
chr15	67907759	67907760	MACS_peak_5213	8.00	lobChIP_FOXA1_robot
chr15	68053969	68053970	MACS_peak_5214	3.00	lobChIP_FOXA1_robot
chr15	68208071	68208072	MACS_peak_5215	34.00	lobChIP_FOXA1_robot
chr15	68238434	68238435	MACS_peak_5216	4.00	lobChIP_FOXA1_robot
chr15	68334633	68334634	MACS_peak_5217	5.00	lobChIP_FOXA1_robot
chr15	68567738	68567739	MACS_peak_5218	4.00	lobChIP_FOXA1_robot
chr15	68577842	68577843	MACS_peak_5219	5.00	lobChIP_FOXA1_robot
chr15	68601601	68601602	MACS_peak_5220	4.00	lobChIP_FOXA1_robot
chr15	69114567	69114568	MACS_peak_5221	5.00	lobChIP_FOXA1_robot
chr15	69418666	69418667	MACS_peak_5222	9.00	lobChIP_FOXA1_robot
chr15	69568427	69568428	MACS_peak_5223	5.00	lobChIP_FOXA1_robot
chr15	69849909	69849910	MACS_peak_5224	4.00	lobChIP_FOXA1_robot
chr15	69853822	69853823	MACS_peak_5225	87.00	lobChIP_FOXA1_robot
chr15	69884410	69884411	MACS_peak_5226	124.00	lobChIP_FOXA1_robot
chr15	69887161	69887162	MACS_peak_5227	97.00	lobChIP_FOXA1_robot
chr15	70371251	70371252	MACS_peak_5228	6.00	lobChIP_FOXA1_robot
chr15	70372328	70372329	MACS_peak_5229	5.00	lobChIP_FOXA1_robot
chr15	70390881	70390882	MACS_peak_5230	10.00	lobChIP_FOXA1_robot
chr15	70433005	70433006	MACS_peak_5231	97.00	lobChIP_FOXA1_robot
chr15	70660950	70660951	MACS_peak_5232	4.00	lobChIP_FOXA1_robot
chr15	70713683	70713684	MACS_peak_5233	6.00	lobChIP_FOXA1_robot
chr15	70800388	70800389	MACS_peak_5234	14.00	lobChIP_FOXA1_robot
chr15	70909694	70909695	MACS_peak_5235	76.00	lobChIP_FOXA1_robot
chr15	70944580	70944581	MACS_peak_5236	3.00	lobChIP_FOXA1_robot
chr15	70983395	70983396	MACS_peak_5237	14.00	lobChIP_FOXA1_robot
chr15	70996700	70996701	MACS_peak_5238	4.00	lobChIP_FOXA1_robot
chr15	71080865	71080866	MACS_peak_5239	4.00	lobChIP_FOXA1_robot
chr15	71104602	71104603	MACS_peak_5240	6.00	lobChIP_FOXA1_robot
chr15	71108188	71108189	MACS_peak_5241	3.00	lobChIP_FOXA1_robot
chr15	71117687	71117688	MACS_peak_5242	6.00	lobChIP_FOXA1_robot
chr15	71571730	71571731	MACS_peak_5243	5.00	lobChIP_FOXA1_robot
chr15	71737626	71737627	MACS_peak_5244	3.00	lobChIP_FOXA1_robot
chr15	71748472	71748473	MACS_peak_5245	26.00	lobChIP_FOXA1_robot
chr15	71804940	71804941	MACS_peak_5246	4.00	lobChIP_FOXA1_robot
chr15	71946556	71946557	MACS_peak_5247	33.00	lobChIP_FOXA1_robot
chr15	71953664	71953665	MACS_peak_5248	16.00	lobChIP_FOXA1_robot
chr15	71971991	71971992	MACS_peak_5249	4.00	lobChIP_FOXA1_robot
chr15	72040039	72040040	MACS_peak_5250	9.00	lobChIP_FOXA1_robot
chr15	72402910	72402911	MACS_peak_5251	13.00	lobChIP_FOXA1_robot
chr15	72434376	72434377	MACS_peak_5252	4.00	lobChIP_FOXA1_robot
chr15	72503280	72503281	MACS_peak_5253	11.00	lobChIP_FOXA1_robot
chr15	72766044	72766045	MACS_peak_5254	10.00	lobChIP_FOXA1_robot
chr15	73586484	73586485	MACS_peak_5255	6.00	lobChIP_FOXA1_robot
chr15	73778372	73778373	MACS_peak_5256	4.00	lobChIP_FOXA1_robot
chr15	73920567	73920568	MACS_peak_5257	5.00	lobChIP_FOXA1_robot
chr15	73921895	73921896	MACS_peak_5258	11.00	lobChIP_FOXA1_robot
chr15	74507761	74507762	MACS_peak_5259	5.00	lobChIP_FOXA1_robot
chr15	74688499	74688500	MACS_peak_5260	5.00	lobChIP_FOXA1_robot
chr15	74734505	74734506	MACS_peak_5261	7.00	lobChIP_FOXA1_robot
chr15	74736085	74736086	MACS_peak_5262	26.00	lobChIP_FOXA1_robot
chr15	74738879	74738880	MACS_peak_5263	12.00	lobChIP_FOXA1_robot
chr15	74753598	74753599	MACS_peak_5264	4.00	lobChIP_FOXA1_robot
chr15	75221381	75221382	MACS_peak_5265	9.00	lobChIP_FOXA1_robot
chr15	75351118	75351119	MACS_peak_5266	4.00	lobChIP_FOXA1_robot
chr15	75432653	75432654	MACS_peak_5267	15.00	lobChIP_FOXA1_robot
chr15	76178176	76178177	MACS_peak_5268	6.00	lobChIP_FOXA1_robot
chr15	76253929	76253930	MACS_peak_5269	15.00	lobChIP_FOXA1_robot
chr15	76690875	76690876	MACS_peak_5270	5.00	lobChIP_FOXA1_robot
chr15	76825906	76825907	MACS_peak_5271	8.00	lobChIP_FOXA1_robot
chr15	76826620	76826621	MACS_peak_5272	7.00	lobChIP_FOXA1_robot
chr15	76865263	76865264	MACS_peak_5273	5.00	lobChIP_FOXA1_robot
chr15	77179994	77179995	MACS_peak_5274	4.00	lobChIP_FOXA1_robot
chr15	77348060	77348061	MACS_peak_5275	4.00	lobChIP_FOXA1_robot
chr15	77476809	77476810	MACS_peak_5276	4.00	lobChIP_FOXA1_robot
chr15	77516865	77516866	MACS_peak_5277	6.00	lobChIP_FOXA1_robot
chr15	77574810	77574811	MACS_peak_5278	4.00	lobChIP_FOXA1_robot
chr15	78355631	78355632	MACS_peak_5279	3.00	lobChIP_FOXA1_robot
chr15	78357597	78357598	MACS_peak_5280	11.00	lobChIP_FOXA1_robot
chr15	78667362	78667363	MACS_peak_5281	5.00	lobChIP_FOXA1_robot
chr15	79244480	79244481	MACS_peak_5282	7.00	lobChIP_FOXA1_robot
chr15	79286372	79286373	MACS_peak_5283	10.00	lobChIP_FOXA1_robot
chr15	80166679	80166680	MACS_peak_5284	3.00	lobChIP_FOXA1_robot
chr15	80178639	80178640	MACS_peak_5285	10.00	lobChIP_FOXA1_robot
chr15	80179605	80179606	MACS_peak_5286	5.00	lobChIP_FOXA1_robot
chr15	80271501	80271502	MACS_peak_5287	9.00	lobChIP_FOXA1_robot
chr15	80498566	80498567	MACS_peak_5288	5.00	lobChIP_FOXA1_robot
chr15	80500544	80500545	MACS_peak_5289	4.00	lobChIP_FOXA1_robot
chr15	80538902	80538903	MACS_peak_5290	3.00	lobChIP_FOXA1_robot
chr15	80757485	80757486	MACS_peak_5291	9.00	lobChIP_FOXA1_robot
chr15	80877029	80877030	MACS_peak_5292	4.00	lobChIP_FOXA1_robot
chr15	80889043	80889044	MACS_peak_5293	5.00	lobChIP_FOXA1_robot
chr15	81097621	81097622	MACS_peak_5294	23.00	lobChIP_FOXA1_robot
chr15	81131722	81131723	MACS_peak_5295	14.00	lobChIP_FOXA1_robot
chr15	81134647	81134648	MACS_peak_5296	11.00	lobChIP_FOXA1_robot
chr15	81149404	81149405	MACS_peak_5297	6.00	lobChIP_FOXA1_robot
chr15	82555910	82555911	MACS_peak_5298	66.00	lobChIP_FOXA1_robot
chr15	83588484	83588485	MACS_peak_5299	12.00	lobChIP_FOXA1_robot
chr15	84599685	84599686	MACS_peak_5300	12.00	lobChIP_FOXA1_robot
chr15	85144831	85144832	MACS_peak_5301	4.00	lobChIP_FOXA1_robot
chr15	85150126	85150127	MACS_peak_5302	25.00	lobChIP_FOXA1_robot
chr15	85295658	85295659	MACS_peak_5303	8.00	lobChIP_FOXA1_robot
chr15	85297243	85297244	MACS_peak_5304	3.00	lobChIP_FOXA1_robot
chr15	85388318	85388319	MACS_peak_5305	6.00	lobChIP_FOXA1_robot
chr15	85467900	85467901	MACS_peak_5306	5.00	lobChIP_FOXA1_robot
chr15	85591069	85591070	MACS_peak_5307	26.00	lobChIP_FOXA1_robot
chr15	85942219	85942220	MACS_peak_5308	4.00	lobChIP_FOXA1_robot
chr15	85956812	85956813	MACS_peak_5309	7.00	lobChIP_FOXA1_robot
chr15	86251269	86251270	MACS_peak_5310	13.00	lobChIP_FOXA1_robot
chr15	86251958	86251959	MACS_peak_5311	4.00	lobChIP_FOXA1_robot
chr15	86324528	86324529	MACS_peak_5312	8.00	lobChIP_FOXA1_robot
chr15	86380507	86380508	MACS_peak_5313	6.00	lobChIP_FOXA1_robot
chr15	89181897	89181898	MACS_peak_5314	5.00	lobChIP_FOXA1_robot
chr15	89644805	89644806	MACS_peak_5315	4.00	lobChIP_FOXA1_robot
chr15	89663491	89663492	MACS_peak_5316	9.00	lobChIP_FOXA1_robot
chr15	89664805	89664806	MACS_peak_5317	16.00	lobChIP_FOXA1_robot
chr15	89668905	89668906	MACS_peak_5318	5.00	lobChIP_FOXA1_robot
chr15	89673128	89673129	MACS_peak_5319	6.00	lobChIP_FOXA1_robot
chr15	89709010	89709011	MACS_peak_5320	5.00	lobChIP_FOXA1_robot
chr15	90002552	90002553	MACS_peak_5321	4.00	lobChIP_FOXA1_robot
chr15	90052018	90052019	MACS_peak_5322	3.00	lobChIP_FOXA1_robot
chr15	90223911	90223912	MACS_peak_5323	5.00	lobChIP_FOXA1_robot
chr15	90300308	90300309	MACS_peak_5324	8.00	lobChIP_FOXA1_robot
chr15	90338217	90338218	MACS_peak_5325	6.00	lobChIP_FOXA1_robot
chr15	90352578	90352579	MACS_peak_5326	17.00	lobChIP_FOXA1_robot
chr15	90388861	90388862	MACS_peak_5327	4.00	lobChIP_FOXA1_robot
chr15	90572459	90572460	MACS_peak_5328	6.00	lobChIP_FOXA1_robot
chr15	90578950	90578951	MACS_peak_5329	6.00	lobChIP_FOXA1_robot
chr15	90581985	90581986	MACS_peak_5330	8.00	lobChIP_FOXA1_robot
chr15	90598279	90598280	MACS_peak_5331	5.00	lobChIP_FOXA1_robot
chr15	90669516	90669517	MACS_peak_5332	8.00	lobChIP_FOXA1_robot
chr15	90709288	90709289	MACS_peak_5333	8.00	lobChIP_FOXA1_robot
chr15	90944289	90944290	MACS_peak_5334	9.00	lobChIP_FOXA1_robot
chr15	90965569	90965570	MACS_peak_5335	4.00	lobChIP_FOXA1_robot
chr15	91033420	91033421	MACS_peak_5336	49.00	lobChIP_FOXA1_robot
chr15	91056911	91056912	MACS_peak_5337	17.00	lobChIP_FOXA1_robot
chr15	91099739	91099740	MACS_peak_5338	4.00	lobChIP_FOXA1_robot
chr15	91379737	91379738	MACS_peak_5339	12.00	lobChIP_FOXA1_robot
chr15	91404056	91404057	MACS_peak_5340	4.00	lobChIP_FOXA1_robot
chr15	91404690	91404691	MACS_peak_5341	21.00	lobChIP_FOXA1_robot
chr15	91417543	91417544	MACS_peak_5342	26.00	lobChIP_FOXA1_robot
chr15	92708180	92708181	MACS_peak_5343	6.00	lobChIP_FOXA1_robot
chr15	93160961	93160962	MACS_peak_5344	8.00	lobChIP_FOXA1_robot
chr15	93210570	93210571	MACS_peak_5345	8.00	lobChIP_FOXA1_robot
chr15	93321410	93321411	MACS_peak_5346	4.00	lobChIP_FOXA1_robot
chr15	93347447	93347448	MACS_peak_5347	21.00	lobChIP_FOXA1_robot
chr15	93354551	93354552	MACS_peak_5348	5.00	lobChIP_FOXA1_robot
chr15	93444098	93444099	MACS_peak_5349	3.00	lobChIP_FOXA1_robot
chr15	93457795	93457796	MACS_peak_5350	3.00	lobChIP_FOXA1_robot
chr15	93460953	93460954	MACS_peak_5351	5.00	lobChIP_FOXA1_robot
chr15	93461363	93461364	MACS_peak_5352	4.00	lobChIP_FOXA1_robot
chr15	93865160	93865161	MACS_peak_5353	5.00	lobChIP_FOXA1_robot
chr15	93884538	93884539	MACS_peak_5354	7.00	lobChIP_FOXA1_robot
chr15	94791673	94791674	MACS_peak_5355	5.00	lobChIP_FOXA1_robot
chr15	94796426	94796427	MACS_peak_5356	4.00	lobChIP_FOXA1_robot
chr15	95784352	95784353	MACS_peak_5357	3.00	lobChIP_FOXA1_robot
chr15	96714285	96714286	MACS_peak_5358	9.00	lobChIP_FOXA1_robot
chr15	96727382	96727383	MACS_peak_5359	3.00	lobChIP_FOXA1_robot
chr15	96752839	96752840	MACS_peak_5360	10.00	lobChIP_FOXA1_robot
chr15	96805223	96805224	MACS_peak_5361	11.00	lobChIP_FOXA1_robot
chr15	96808431	96808432	MACS_peak_5362	6.00	lobChIP_FOXA1_robot
chr15	96814031	96814032	MACS_peak_5363	3.00	lobChIP_FOXA1_robot
chr15	96869486	96869487	MACS_peak_5364	4.00	lobChIP_FOXA1_robot
chr15	96882183	96882184	MACS_peak_5365	11.00	lobChIP_FOXA1_robot
chr15	98521849	98521850	MACS_peak_5366	3.00	lobChIP_FOXA1_robot
chr15	99198274	99198275	MACS_peak_5367	11.00	lobChIP_FOXA1_robot
chr15	99207187	99207188	MACS_peak_5368	4.00	lobChIP_FOXA1_robot
chr15	99214636	99214637	MACS_peak_5369	7.00	lobChIP_FOXA1_robot
chr15	99277978	99277979	MACS_peak_5370	5.00	lobChIP_FOXA1_robot
chr15	99342927	99342928	MACS_peak_5371	8.00	lobChIP_FOXA1_robot
chr15	99356097	99356098	MACS_peak_5372	8.00	lobChIP_FOXA1_robot
chr15	99385654	99385655	MACS_peak_5373	4.00	lobChIP_FOXA1_robot
chr15	99387248	99387249	MACS_peak_5374	40.00	lobChIP_FOXA1_robot
chr15	99440292	99440293	MACS_peak_5375	8.00	lobChIP_FOXA1_robot
chr15	99675603	99675604	MACS_peak_5376	6.00	lobChIP_FOXA1_robot
chr15	99683640	99683641	MACS_peak_5377	4.00	lobChIP_FOXA1_robot
chr15	99721004	99721005	MACS_peak_5378	7.00	lobChIP_FOXA1_robot
chr15	99866668	99866669	MACS_peak_5379	5.00	lobChIP_FOXA1_robot
chr15	99961535	99961536	MACS_peak_5380	4.00	lobChIP_FOXA1_robot
chr15	100168663	100168664	MACS_peak_5381	3.00	lobChIP_FOXA1_robot
chr15	100207950	100207951	MACS_peak_5382	6.00	lobChIP_FOXA1_robot
chr15	100209551	100209552	MACS_peak_5383	4.00	lobChIP_FOXA1_robot
chr15	100274302	100274303	MACS_peak_5384	3.00	lobChIP_FOXA1_robot
chr15	100324409	100324410	MACS_peak_5385	5.00	lobChIP_FOXA1_robot
chr15	100891045	100891046	MACS_peak_5386	7.00	lobChIP_FOXA1_robot
chr15	101256103	101256104	MACS_peak_5387	14.00	lobChIP_FOXA1_robot
chr15	101262812	101262813	MACS_peak_5388	4.00	lobChIP_FOXA1_robot
chr15	101300023	101300024	MACS_peak_5389	4.00	lobChIP_FOXA1_robot
chr15	101611130	101611131	MACS_peak_5390	27.00	lobChIP_FOXA1_robot
chr15	101979946	101979947	MACS_peak_5391	8.00	lobChIP_FOXA1_robot
chr15	101985860	101985861	MACS_peak_5392	14.00	lobChIP_FOXA1_robot
chr15	102068720	102068721	MACS_peak_5393	10.00	lobChIP_FOXA1_robot
chr15	102078038	102078039	MACS_peak_5394	7.00	lobChIP_FOXA1_robot
chr16	95547	95548	MACS_peak_5395	4.00	lobChIP_FOXA1_robot
chr16	392748	392749	MACS_peak_5396	7.00	lobChIP_FOXA1_robot
chr16	507805	507806	MACS_peak_5397	4.00	lobChIP_FOXA1_robot
chr16	559352	559353	MACS_peak_5398	4.00	lobChIP_FOXA1_robot
chr16	962910	962911	MACS_peak_5399	5.00	lobChIP_FOXA1_robot
chr16	1128722	1128723	MACS_peak_5400	6.00	lobChIP_FOXA1_robot
chr16	1205103	1205104	MACS_peak_5401	14.00	lobChIP_FOXA1_robot
chr16	1341027	1341028	MACS_peak_5402	6.00	lobChIP_FOXA1_robot
chr16	1480926	1480927	MACS_peak_5403	11.00	lobChIP_FOXA1_robot
chr16	1739342	1739343	MACS_peak_5404	18.00	lobChIP_FOXA1_robot
chr16	1878810	1878811	MACS_peak_5405	6.00	lobChIP_FOXA1_robot
chr16	1920572	1920573	MACS_peak_5406	4.00	lobChIP_FOXA1_robot
chr16	1990731	1990732	MACS_peak_5407	7.00	lobChIP_FOXA1_robot
chr16	2089732	2089733	MACS_peak_5408	7.00	lobChIP_FOXA1_robot
chr16	2276559	2276560	MACS_peak_5409	15.00	lobChIP_FOXA1_robot
chr16	2439049	2439050	MACS_peak_5410	28.00	lobChIP_FOXA1_robot
chr16	2574480	2574481	MACS_peak_5411	6.00	lobChIP_FOXA1_robot
chr16	3207334	3207335	MACS_peak_5412	4.00	lobChIP_FOXA1_robot
chr16	3579778	3579779	MACS_peak_5413	5.00	lobChIP_FOXA1_robot
chr16	3646608	3646609	MACS_peak_5414	7.00	lobChIP_FOXA1_robot
chr16	3660097	3660098	MACS_peak_5415	8.00	lobChIP_FOXA1_robot
chr16	3696387	3696388	MACS_peak_5416	5.00	lobChIP_FOXA1_robot
chr16	3745915	3745916	MACS_peak_5417	6.00	lobChIP_FOXA1_robot
chr16	4062507	4062508	MACS_peak_5418	7.00	lobChIP_FOXA1_robot
chr16	4159393	4159394	MACS_peak_5419	7.00	lobChIP_FOXA1_robot
chr16	4207649	4207650	MACS_peak_5420	5.00	lobChIP_FOXA1_robot
chr16	4323173	4323174	MACS_peak_5421	14.00	lobChIP_FOXA1_robot
chr16	4395991	4395992	MACS_peak_5422	10.00	lobChIP_FOXA1_robot
chr16	4423132	4423133	MACS_peak_5423	8.00	lobChIP_FOXA1_robot
chr16	4551410	4551411	MACS_peak_5424	5.00	lobChIP_FOXA1_robot
chr16	4838788	4838789	MACS_peak_5425	3.00	lobChIP_FOXA1_robot
chr16	4993364	4993365	MACS_peak_5426	3.00	lobChIP_FOXA1_robot
chr16	4994779	4994780	MACS_peak_5427	7.00	lobChIP_FOXA1_robot
chr16	4997854	4997855	MACS_peak_5428	5.00	lobChIP_FOXA1_robot
chr16	5007960	5007961	MACS_peak_5429	4.00	lobChIP_FOXA1_robot
chr16	5082996	5082997	MACS_peak_5430	5.00	lobChIP_FOXA1_robot
chr16	5085500	5085501	MACS_peak_5431	11.00	lobChIP_FOXA1_robot
chr16	5194522	5194523	MACS_peak_5432	19.00	lobChIP_FOXA1_robot
chr16	6357596	6357597	MACS_peak_5433	5.00	lobChIP_FOXA1_robot
chr16	6451471	6451472	MACS_peak_5434	3.00	lobChIP_FOXA1_robot
chr16	7144966	7144967	MACS_peak_5435	5.00	lobChIP_FOXA1_robot
chr16	7236240	7236241	MACS_peak_5436	9.00	lobChIP_FOXA1_robot
chr16	7342105	7342106	MACS_peak_5437	5.00	lobChIP_FOXA1_robot
chr16	8223335	8223336	MACS_peak_5438	5.00	lobChIP_FOXA1_robot
chr16	8441436	8441437	MACS_peak_5439	5.00	lobChIP_FOXA1_robot
chr16	8465764	8465765	MACS_peak_5440	3.00	lobChIP_FOXA1_robot
chr16	8587900	8587901	MACS_peak_5441	5.00	lobChIP_FOXA1_robot
chr16	8601567	8601568	MACS_peak_5442	8.00	lobChIP_FOXA1_robot
chr16	8625324	8625325	MACS_peak_5443	3.00	lobChIP_FOXA1_robot
chr16	8772697	8772698	MACS_peak_5444	20.00	lobChIP_FOXA1_robot
chr16	8775174	8775175	MACS_peak_5445	3.00	lobChIP_FOXA1_robot
chr16	8814807	8814808	MACS_peak_5446	5.00	lobChIP_FOXA1_robot
chr16	8819455	8819456	MACS_peak_5447	5.00	lobChIP_FOXA1_robot
chr16	8834800	8834801	MACS_peak_5448	18.00	lobChIP_FOXA1_robot
chr16	8837478	8837479	MACS_peak_5449	6.00	lobChIP_FOXA1_robot
chr16	8957640	8957641	MACS_peak_5450	7.00	lobChIP_FOXA1_robot
chr16	9053899	9053900	MACS_peak_5451	6.00	lobChIP_FOXA1_robot
chr16	9054319	9054320	MACS_peak_5452	11.00	lobChIP_FOXA1_robot
chr16	9081460	9081461	MACS_peak_5453	25.00	lobChIP_FOXA1_robot
chr16	9354044	9354045	MACS_peak_5454	3.00	lobChIP_FOXA1_robot
chr16	9410651	9410652	MACS_peak_5455	6.00	lobChIP_FOXA1_robot
chr16	9459367	9459368	MACS_peak_5456	5.00	lobChIP_FOXA1_robot
chr16	9639509	9639510	MACS_peak_5457	3.00	lobChIP_FOXA1_robot
chr16	9840381	9840382	MACS_peak_5458	4.00	lobChIP_FOXA1_robot
chr16	9941607	9941608	MACS_peak_5459	3.00	lobChIP_FOXA1_robot
chr16	9943138	9943139	MACS_peak_5460	4.00	lobChIP_FOXA1_robot
chr16	10113078	10113079	MACS_peak_5461	3.00	lobChIP_FOXA1_robot
chr16	10265188	10265189	MACS_peak_5462	5.00	lobChIP_FOXA1_robot
chr16	10351090	10351091	MACS_peak_5463	6.00	lobChIP_FOXA1_robot
chr16	10356353	10356354	MACS_peak_5464	4.00	lobChIP_FOXA1_robot
chr16	10360053	10360054	MACS_peak_5465	10.00	lobChIP_FOXA1_robot
chr16	10363706	10363707	MACS_peak_5466	15.00	lobChIP_FOXA1_robot
chr16	10412758	10412759	MACS_peak_5467	7.00	lobChIP_FOXA1_robot
chr16	10417597	10417598	MACS_peak_5468	3.00	lobChIP_FOXA1_robot
chr16	10456535	10456536	MACS_peak_5469	5.00	lobChIP_FOXA1_robot
chr16	10608790	10608791	MACS_peak_5470	3.00	lobChIP_FOXA1_robot
chr16	10614434	10614435	MACS_peak_5471	7.00	lobChIP_FOXA1_robot
chr16	10695940	10695941	MACS_peak_5472	7.00	lobChIP_FOXA1_robot
chr16	10704992	10704993	MACS_peak_5473	12.00	lobChIP_FOXA1_robot
chr16	10719997	10719998	MACS_peak_5474	7.00	lobChIP_FOXA1_robot
chr16	10741770	10741771	MACS_peak_5475	47.00	lobChIP_FOXA1_robot
chr16	10791741	10791742	MACS_peak_5476	5.00	lobChIP_FOXA1_robot
chr16	11110755	11110756	MACS_peak_5477	9.00	lobChIP_FOXA1_robot
chr16	11118395	11118396	MACS_peak_5478	3.00	lobChIP_FOXA1_robot
chr16	11150546	11150547	MACS_peak_5479	4.00	lobChIP_FOXA1_robot
chr16	11233332	11233333	MACS_peak_5480	49.00	lobChIP_FOXA1_robot
chr16	11361299	11361300	MACS_peak_5481	13.00	lobChIP_FOXA1_robot
chr16	11373614	11373615	MACS_peak_5482	12.00	lobChIP_FOXA1_robot
chr16	11390867	11390868	MACS_peak_5483	9.00	lobChIP_FOXA1_robot
chr16	11439095	11439096	MACS_peak_5484	4.00	lobChIP_FOXA1_robot
chr16	11458398	11458399	MACS_peak_5485	23.00	lobChIP_FOXA1_robot
chr16	11465770	11465771	MACS_peak_5486	4.00	lobChIP_FOXA1_robot
chr16	11668569	11668570	MACS_peak_5487	4.00	lobChIP_FOXA1_robot
chr16	11669639	11669640	MACS_peak_5488	9.00	lobChIP_FOXA1_robot
chr16	11670662	11670663	MACS_peak_5489	14.00	lobChIP_FOXA1_robot
chr16	11678165	11678166	MACS_peak_5490	3.00	lobChIP_FOXA1_robot
chr16	11683269	11683270	MACS_peak_5491	3.00	lobChIP_FOXA1_robot
chr16	11724826	11724827	MACS_peak_5492	3.00	lobChIP_FOXA1_robot
chr16	11783886	11783887	MACS_peak_5493	5.00	lobChIP_FOXA1_robot
chr16	11794380	11794381	MACS_peak_5494	10.00	lobChIP_FOXA1_robot
chr16	11837523	11837524	MACS_peak_5495	3.00	lobChIP_FOXA1_robot
chr16	11838438	11838439	MACS_peak_5496	7.00	lobChIP_FOXA1_robot
chr16	11849117	11849118	MACS_peak_5497	10.00	lobChIP_FOXA1_robot
chr16	11853471	11853472	MACS_peak_5498	6.00	lobChIP_FOXA1_robot
chr16	11879871	11879872	MACS_peak_5499	9.00	lobChIP_FOXA1_robot
chr16	11904227	11904228	MACS_peak_5500	17.00	lobChIP_FOXA1_robot
chr16	11916268	11916269	MACS_peak_5501	7.00	lobChIP_FOXA1_robot
chr16	11928215	11928216	MACS_peak_5502	5.00	lobChIP_FOXA1_robot
chr16	11963890	11963891	MACS_peak_5503	3.00	lobChIP_FOXA1_robot
chr16	12010136	12010137	MACS_peak_5504	4.00	lobChIP_FOXA1_robot
chr16	12010665	12010666	MACS_peak_5505	6.00	lobChIP_FOXA1_robot
chr16	12046920	12046921	MACS_peak_5506	3.00	lobChIP_FOXA1_robot
chr16	12185783	12185784	MACS_peak_5507	4.00	lobChIP_FOXA1_robot
chr16	12191493	12191494	MACS_peak_5508	4.00	lobChIP_FOXA1_robot
chr16	12289029	12289030	MACS_peak_5509	3.00	lobChIP_FOXA1_robot
chr16	12305471	12305472	MACS_peak_5510	11.00	lobChIP_FOXA1_robot
chr16	12306522	12306523	MACS_peak_5511	5.00	lobChIP_FOXA1_robot
chr16	12324319	12324320	MACS_peak_5512	3.00	lobChIP_FOXA1_robot
chr16	12379202	12379203	MACS_peak_5513	4.00	lobChIP_FOXA1_robot
chr16	12409611	12409612	MACS_peak_5514	13.00	lobChIP_FOXA1_robot
chr16	12667906	12667907	MACS_peak_5515	14.00	lobChIP_FOXA1_robot
chr16	12683392	12683393	MACS_peak_5516	4.00	lobChIP_FOXA1_robot
chr16	12706619	12706620	MACS_peak_5517	4.00	lobChIP_FOXA1_robot
chr16	12707417	12707418	MACS_peak_5518	9.00	lobChIP_FOXA1_robot
chr16	12737946	12737947	MACS_peak_5519	12.00	lobChIP_FOXA1_robot
chr16	12738968	12738969	MACS_peak_5520	4.00	lobChIP_FOXA1_robot
chr16	12772996	12772997	MACS_peak_5521	6.00	lobChIP_FOXA1_robot
chr16	12848175	12848176	MACS_peak_5522	16.00	lobChIP_FOXA1_robot
chr16	12878908	12878909	MACS_peak_5523	3.00	lobChIP_FOXA1_robot
chr16	12894022	12894023	MACS_peak_5524	4.00	lobChIP_FOXA1_robot
chr16	12897462	12897463	MACS_peak_5525	6.00	lobChIP_FOXA1_robot
chr16	13192750	13192751	MACS_peak_5526	5.00	lobChIP_FOXA1_robot
chr16	13204404	13204405	MACS_peak_5527	8.00	lobChIP_FOXA1_robot
chr16	13339785	13339786	MACS_peak_5528	67.00	lobChIP_FOXA1_robot
chr16	13364039	13364040	MACS_peak_5529	16.00	lobChIP_FOXA1_robot
chr16	13979835	13979836	MACS_peak_5530	20.00	lobChIP_FOXA1_robot
chr16	14013156	14013157	MACS_peak_5531	5.00	lobChIP_FOXA1_robot
chr16	14038341	14038342	MACS_peak_5532	4.00	lobChIP_FOXA1_robot
chr16	14051809	14051810	MACS_peak_5533	9.00	lobChIP_FOXA1_robot
chr16	14106688	14106689	MACS_peak_5534	5.00	lobChIP_FOXA1_robot
chr16	14114206	14114207	MACS_peak_5535	6.00	lobChIP_FOXA1_robot
chr16	14182112	14182113	MACS_peak_5536	8.00	lobChIP_FOXA1_robot
chr16	14200499	14200500	MACS_peak_5537	22.00	lobChIP_FOXA1_robot
chr16	14296833	14296834	MACS_peak_5538	5.00	lobChIP_FOXA1_robot
chr16	14383758	14383759	MACS_peak_5539	4.00	lobChIP_FOXA1_robot
chr16	14412329	14412330	MACS_peak_5540	7.00	lobChIP_FOXA1_robot
chr16	14432087	14432088	MACS_peak_5541	3.00	lobChIP_FOXA1_robot
chr16	14458531	14458532	MACS_peak_5542	11.00	lobChIP_FOXA1_robot
chr16	14479297	14479298	MACS_peak_5543	9.00	lobChIP_FOXA1_robot
chr16	14493511	14493512	MACS_peak_5544	6.00	lobChIP_FOXA1_robot
chr16	14567539	14567540	MACS_peak_5545	5.00	lobChIP_FOXA1_robot
chr16	14569464	14569465	MACS_peak_5546	7.00	lobChIP_FOXA1_robot
chr16	14569897	14569898	MACS_peak_5547	4.00	lobChIP_FOXA1_robot
chr16	14591625	14591626	MACS_peak_5548	18.00	lobChIP_FOXA1_robot
chr16	14598554	14598555	MACS_peak_5549	6.00	lobChIP_FOXA1_robot
chr16	14605109	14605110	MACS_peak_5550	29.00	lobChIP_FOXA1_robot
chr16	14639488	14639489	MACS_peak_5551	44.00	lobChIP_FOXA1_robot
chr16	14643831	14643832	MACS_peak_5552	9.00	lobChIP_FOXA1_robot
chr16	14656733	14656734	MACS_peak_5553	8.00	lobChIP_FOXA1_robot
chr16	14659421	14659422	MACS_peak_5554	5.00	lobChIP_FOXA1_robot
chr16	14660105	14660106	MACS_peak_5555	5.00	lobChIP_FOXA1_robot
chr16	14728374	14728375	MACS_peak_5556	4.00	lobChIP_FOXA1_robot
chr16	14750538	14750539	MACS_peak_5557	5.00	lobChIP_FOXA1_robot
chr16	14766408	14766409	MACS_peak_5558	5.00	lobChIP_FOXA1_robot
chr16	14968259	14968260	MACS_peak_5559	6.00	lobChIP_FOXA1_robot
chr16	15146260	15146261	MACS_peak_5560	14.00	lobChIP_FOXA1_robot
chr16	15576307	15576308	MACS_peak_5561	5.00	lobChIP_FOXA1_robot
chr16	15583595	15583596	MACS_peak_5562	4.00	lobChIP_FOXA1_robot
chr16	15666524	15666525	MACS_peak_5563	6.00	lobChIP_FOXA1_robot
chr16	15670355	15670356	MACS_peak_5564	4.00	lobChIP_FOXA1_robot
chr16	15684953	15684954	MACS_peak_5565	10.00	lobChIP_FOXA1_robot
chr16	15702447	15702448	MACS_peak_5566	11.00	lobChIP_FOXA1_robot
chr16	15704474	15704475	MACS_peak_5567	10.00	lobChIP_FOXA1_robot
chr16	15709574	15709575	MACS_peak_5568	4.00	lobChIP_FOXA1_robot
chr16	15723052	15723053	MACS_peak_5569	19.00	lobChIP_FOXA1_robot
chr16	15735403	15735404	MACS_peak_5570	3.00	lobChIP_FOXA1_robot
chr16	15766939	15766940	MACS_peak_5571	20.00	lobChIP_FOXA1_robot
chr16	15969092	15969093	MACS_peak_5572	8.00	lobChIP_FOXA1_robot
chr16	15981559	15981560	MACS_peak_5573	3.00	lobChIP_FOXA1_robot
chr16	16010397	16010398	MACS_peak_5574	4.00	lobChIP_FOXA1_robot
chr16	16022201	16022202	MACS_peak_5575	3.00	lobChIP_FOXA1_robot
chr16	16027036	16027037	MACS_peak_5576	5.00	lobChIP_FOXA1_robot
chr16	16040315	16040316	MACS_peak_5577	6.00	lobChIP_FOXA1_robot
chr16	16090766	16090767	MACS_peak_5578	7.00	lobChIP_FOXA1_robot
chr16	16108966	16108967	MACS_peak_5579	11.00	lobChIP_FOXA1_robot
chr16	16243716	16243717	MACS_peak_5580	5.00	lobChIP_FOXA1_robot
chr16	16288903	16288904	MACS_peak_5581	5.00	lobChIP_FOXA1_robot
chr16	16307954	16307955	MACS_peak_5582	5.00	lobChIP_FOXA1_robot
chr16	16345490	16345491	MACS_peak_5583	5.00	lobChIP_FOXA1_robot
chr16	17472529	17472530	MACS_peak_5584	5.00	lobChIP_FOXA1_robot
chr16	17888236	17888237	MACS_peak_5585	3.00	lobChIP_FOXA1_robot
chr16	18839062	18839063	MACS_peak_5586	5.00	lobChIP_FOXA1_robot
chr16	18842715	18842716	MACS_peak_5587	4.00	lobChIP_FOXA1_robot
chr16	18943839	18943840	MACS_peak_5588	6.00	lobChIP_FOXA1_robot
chr16	18948490	18948491	MACS_peak_5589	8.00	lobChIP_FOXA1_robot
chr16	18957330	18957331	MACS_peak_5590	6.00	lobChIP_FOXA1_robot
chr16	19000425	19000426	MACS_peak_5591	30.00	lobChIP_FOXA1_robot
chr16	19010206	19010207	MACS_peak_5592	4.00	lobChIP_FOXA1_robot
chr16	19028715	19028716	MACS_peak_5593	5.00	lobChIP_FOXA1_robot
chr16	19104954	19104955	MACS_peak_5594	12.00	lobChIP_FOXA1_robot
chr16	19122294	19122295	MACS_peak_5595	7.00	lobChIP_FOXA1_robot
chr16	19490792	19490793	MACS_peak_5596	9.00	lobChIP_FOXA1_robot
chr16	19544202	19544203	MACS_peak_5597	4.00	lobChIP_FOXA1_robot
chr16	19553503	19553504	MACS_peak_5598	4.00	lobChIP_FOXA1_robot
chr16	19673905	19673906	MACS_peak_5599	8.00	lobChIP_FOXA1_robot
chr16	19711748	19711749	MACS_peak_5600	19.00	lobChIP_FOXA1_robot
chr16	19756123	19756124	MACS_peak_5601	25.00	lobChIP_FOXA1_robot
chr16	19769441	19769442	MACS_peak_5602	4.00	lobChIP_FOXA1_robot
chr16	19769906	19769907	MACS_peak_5603	6.00	lobChIP_FOXA1_robot
chr16	19796933	19796934	MACS_peak_5604	6.00	lobChIP_FOXA1_robot
chr16	19822735	19822736	MACS_peak_5605	4.00	lobChIP_FOXA1_robot
chr16	20339257	20339258	MACS_peak_5606	5.00	lobChIP_FOXA1_robot
chr16	20438067	20438068	MACS_peak_5607	7.00	lobChIP_FOXA1_robot
chr16	20608234	20608235	MACS_peak_5608	8.00	lobChIP_FOXA1_robot
chr16	20610234	20610235	MACS_peak_5609	5.00	lobChIP_FOXA1_robot
chr16	20689287	20689288	MACS_peak_5610	5.00	lobChIP_FOXA1_robot
chr16	20694077	20694078	MACS_peak_5611	4.00	lobChIP_FOXA1_robot
chr16	20815148	20815149	MACS_peak_5612	5.00	lobChIP_FOXA1_robot
chr16	20862519	20862520	MACS_peak_5613	4.00	lobChIP_FOXA1_robot
chr16	20866393	20866394	MACS_peak_5614	7.00	lobChIP_FOXA1_robot
chr16	20886180	20886181	MACS_peak_5615	6.00	lobChIP_FOXA1_robot
chr16	21245021	21245022	MACS_peak_5616	5.00	lobChIP_FOXA1_robot
chr16	21245942	21245943	MACS_peak_5617	4.00	lobChIP_FOXA1_robot
chr16	21257733	21257734	MACS_peak_5618	4.00	lobChIP_FOXA1_robot
chr16	21312053	21312054	MACS_peak_5619	4.00	lobChIP_FOXA1_robot
chr16	21350104	21350105	MACS_peak_5620	5.00	lobChIP_FOXA1_robot
chr16	21532915	21532916	MACS_peak_5621	4.00	lobChIP_FOXA1_robot
chr16	21536845	21536846	MACS_peak_5622	7.00	lobChIP_FOXA1_robot
chr16	21538527	21538528	MACS_peak_5623	3.00	lobChIP_FOXA1_robot
chr16	21577411	21577412	MACS_peak_5624	17.00	lobChIP_FOXA1_robot
chr16	21595489	21595490	MACS_peak_5625	4.00	lobChIP_FOXA1_robot
chr16	21639642	21639643	MACS_peak_5626	10.00	lobChIP_FOXA1_robot
chr16	21650192	21650193	MACS_peak_5627	32.00	lobChIP_FOXA1_robot
chr16	21948802	21948803	MACS_peak_5628	44.00	lobChIP_FOXA1_robot
chr16	21963858	21963859	MACS_peak_5629	3.00	lobChIP_FOXA1_robot
chr16	22017713	22017714	MACS_peak_5630	4.00	lobChIP_FOXA1_robot
chr16	22038170	22038171	MACS_peak_5631	14.00	lobChIP_FOXA1_robot
chr16	22041477	22041478	MACS_peak_5632	5.00	lobChIP_FOXA1_robot
chr16	22203883	22203884	MACS_peak_5633	4.00	lobChIP_FOXA1_robot
chr16	22303750	22303751	MACS_peak_5634	4.00	lobChIP_FOXA1_robot
chr16	22380012	22380013	MACS_peak_5635	7.00	lobChIP_FOXA1_robot
chr16	22640258	22640259	MACS_peak_5636	4.00	lobChIP_FOXA1_robot
chr16	22678827	22678828	MACS_peak_5637	3.00	lobChIP_FOXA1_robot
chr16	23104328	23104329	MACS_peak_5638	7.00	lobChIP_FOXA1_robot
chr16	23115910	23115911	MACS_peak_5639	13.00	lobChIP_FOXA1_robot
chr16	23137363	23137364	MACS_peak_5640	4.00	lobChIP_FOXA1_robot
chr16	23153603	23153604	MACS_peak_5641	4.00	lobChIP_FOXA1_robot
chr16	23155694	23155695	MACS_peak_5642	4.00	lobChIP_FOXA1_robot
chr16	23252636	23252637	MACS_peak_5643	16.00	lobChIP_FOXA1_robot
chr16	23264340	23264341	MACS_peak_5644	5.00	lobChIP_FOXA1_robot
chr16	23265035	23265036	MACS_peak_5645	8.00	lobChIP_FOXA1_robot
chr16	23336361	23336362	MACS_peak_5646	7.00	lobChIP_FOXA1_robot
chr16	23551807	23551808	MACS_peak_5647	21.00	lobChIP_FOXA1_robot
chr16	23927154	23927155	MACS_peak_5648	5.00	lobChIP_FOXA1_robot
chr16	24697419	24697420	MACS_peak_5649	4.00	lobChIP_FOXA1_robot
chr16	24749013	24749014	MACS_peak_5650	10.00	lobChIP_FOXA1_robot
chr16	24998414	24998415	MACS_peak_5651	4.00	lobChIP_FOXA1_robot
chr16	25005764	25005765	MACS_peak_5652	8.00	lobChIP_FOXA1_robot
chr16	25025621	25025622	MACS_peak_5653	4.00	lobChIP_FOXA1_robot
chr16	25189540	25189541	MACS_peak_5654	6.00	lobChIP_FOXA1_robot
chr16	25217261	25217262	MACS_peak_5655	4.00	lobChIP_FOXA1_robot
chr16	25230627	25230628	MACS_peak_5656	7.00	lobChIP_FOXA1_robot
chr16	25309987	25309988	MACS_peak_5657	12.00	lobChIP_FOXA1_robot
chr16	27254499	27254500	MACS_peak_5658	3.00	lobChIP_FOXA1_robot
chr16	27269778	27269779	MACS_peak_5659	8.00	lobChIP_FOXA1_robot
chr16	27275742	27275743	MACS_peak_5660	4.00	lobChIP_FOXA1_robot
chr16	27285368	27285369	MACS_peak_5661	6.00	lobChIP_FOXA1_robot
chr16	27324749	27324750	MACS_peak_5662	5.00	lobChIP_FOXA1_robot
chr16	27376052	27376053	MACS_peak_5663	35.00	lobChIP_FOXA1_robot
chr16	27590658	27590659	MACS_peak_5664	6.00	lobChIP_FOXA1_robot
chr16	28175703	28175704	MACS_peak_5665	4.00	lobChIP_FOXA1_robot
chr16	28336168	28336169	MACS_peak_5666	5.00	lobChIP_FOXA1_robot
chr16	28537862	28537863	MACS_peak_5667	8.00	lobChIP_FOXA1_robot
chr16	28539934	28539935	MACS_peak_5668	6.00	lobChIP_FOXA1_robot
chr16	28552591	28552592	MACS_peak_5669	12.00	lobChIP_FOXA1_robot
chr16	28934865	28934866	MACS_peak_5670	5.00	lobChIP_FOXA1_robot
chr16	29168515	29168516	MACS_peak_5671	12.00	lobChIP_FOXA1_robot
chr16	29776955	29776956	MACS_peak_5672	10.00	lobChIP_FOXA1_robot
chr16	29955532	29955533	MACS_peak_5673	5.00	lobChIP_FOXA1_robot
chr16	30151396	30151397	MACS_peak_5674	25.00	lobChIP_FOXA1_robot
chr16	30347437	30347438	MACS_peak_5675	4.00	lobChIP_FOXA1_robot
chr16	30366643	30366644	MACS_peak_5676	4.00	lobChIP_FOXA1_robot
chr16	30413380	30413381	MACS_peak_5677	8.00	lobChIP_FOXA1_robot
chr16	30449183	30449184	MACS_peak_5678	10.00	lobChIP_FOXA1_robot
chr16	30453783	30453784	MACS_peak_5679	6.00	lobChIP_FOXA1_robot
chr16	30459151	30459152	MACS_peak_5680	5.00	lobChIP_FOXA1_robot
chr16	30488514	30488515	MACS_peak_5681	6.00	lobChIP_FOXA1_robot
chr16	30500239	30500240	MACS_peak_5682	5.00	lobChIP_FOXA1_robot
chr16	30835722	30835723	MACS_peak_5683	4.00	lobChIP_FOXA1_robot
chr16	30850356	30850357	MACS_peak_5684	10.00	lobChIP_FOXA1_robot
chr16	30912109	30912110	MACS_peak_5685	5.00	lobChIP_FOXA1_robot
chr16	30965631	30965632	MACS_peak_5686	4.00	lobChIP_FOXA1_robot
chr16	31111731	31111732	MACS_peak_5687	4.00	lobChIP_FOXA1_robot
chr16	31193752	31193753	MACS_peak_5688	5.00	lobChIP_FOXA1_robot
chr16	31712457	31712458	MACS_peak_5689	7.00	lobChIP_FOXA1_robot
chr16	31888897	31888898	MACS_peak_5690	5.00	lobChIP_FOXA1_robot
chr16	33956569	33956570	MACS_peak_5692	5.00	lobChIP_FOXA1_robot
chr16	33961126	33961127	MACS_peak_5693	5.00	lobChIP_FOXA1_robot
chr16	46823179	46823180	MACS_peak_5695	13.00	lobChIP_FOXA1_robot
chr16	46889535	46889536	MACS_peak_5696	5.00	lobChIP_FOXA1_robot
chr16	46898446	46898447	MACS_peak_5697	9.00	lobChIP_FOXA1_robot
chr16	46910666	46910667	MACS_peak_5698	46.00	lobChIP_FOXA1_robot
chr16	46916585	46916586	MACS_peak_5699	6.00	lobChIP_FOXA1_robot
chr16	46917481	46917482	MACS_peak_5700	6.00	lobChIP_FOXA1_robot
chr16	46930113	46930114	MACS_peak_5701	7.00	lobChIP_FOXA1_robot
chr16	46953025	46953026	MACS_peak_5702	5.00	lobChIP_FOXA1_robot
chr16	46982720	46982721	MACS_peak_5703	7.00	lobChIP_FOXA1_robot
chr16	47010757	47010758	MACS_peak_5704	5.00	lobChIP_FOXA1_robot
chr16	47188996	47188997	MACS_peak_5705	6.00	lobChIP_FOXA1_robot
chr16	47509789	47509790	MACS_peak_5706	5.00	lobChIP_FOXA1_robot
chr16	47527196	47527197	MACS_peak_5707	3.00	lobChIP_FOXA1_robot
chr16	47533702	47533703	MACS_peak_5708	4.00	lobChIP_FOXA1_robot
chr16	47572524	47572525	MACS_peak_5709	14.00	lobChIP_FOXA1_robot
chr16	47668198	47668199	MACS_peak_5710	37.00	lobChIP_FOXA1_robot
chr16	47691181	47691182	MACS_peak_5711	11.00	lobChIP_FOXA1_robot
chr16	48110770	48110771	MACS_peak_5712	3.00	lobChIP_FOXA1_robot
chr16	48199390	48199391	MACS_peak_5713	5.00	lobChIP_FOXA1_robot
chr16	48229259	48229260	MACS_peak_5714	4.00	lobChIP_FOXA1_robot
chr16	48238628	48238629	MACS_peak_5715	15.00	lobChIP_FOXA1_robot
chr16	48263252	48263253	MACS_peak_5716	16.00	lobChIP_FOXA1_robot
chr16	48269748	48269749	MACS_peak_5717	4.00	lobChIP_FOXA1_robot
chr16	48270369	48270370	MACS_peak_5718	8.00	lobChIP_FOXA1_robot
chr16	48475921	48475922	MACS_peak_5719	21.00	lobChIP_FOXA1_robot
chr16	48564810	48564811	MACS_peak_5720	3.00	lobChIP_FOXA1_robot
chr16	48596002	48596003	MACS_peak_5721	3.00	lobChIP_FOXA1_robot
chr16	49968610	49968611	MACS_peak_5722	7.00	lobChIP_FOXA1_robot
chr16	50069500	50069501	MACS_peak_5723	17.00	lobChIP_FOXA1_robot
chr16	50189127	50189128	MACS_peak_5724	20.00	lobChIP_FOXA1_robot
chr16	50232804	50232805	MACS_peak_5725	10.00	lobChIP_FOXA1_robot
chr16	50236782	50236783	MACS_peak_5726	5.00	lobChIP_FOXA1_robot
chr16	50293633	50293634	MACS_peak_5727	5.00	lobChIP_FOXA1_robot
chr16	50298523	50298524	MACS_peak_5728	4.00	lobChIP_FOXA1_robot
chr16	50363902	50363903	MACS_peak_5729	4.00	lobChIP_FOXA1_robot
chr16	50497761	50497762	MACS_peak_5730	5.00	lobChIP_FOXA1_robot
chr16	50502061	50502062	MACS_peak_5731	9.00	lobChIP_FOXA1_robot
chr16	50536845	50536846	MACS_peak_5732	3.00	lobChIP_FOXA1_robot
chr16	50554598	50554599	MACS_peak_5733	12.00	lobChIP_FOXA1_robot
chr16	50564671	50564672	MACS_peak_5734	4.00	lobChIP_FOXA1_robot
chr16	50573306	50573307	MACS_peak_5735	11.00	lobChIP_FOXA1_robot
chr16	50581560	50581561	MACS_peak_5736	14.00	lobChIP_FOXA1_robot
chr16	50585215	50585216	MACS_peak_5737	3.00	lobChIP_FOXA1_robot
chr16	50651495	50651496	MACS_peak_5738	10.00	lobChIP_FOXA1_robot
chr16	51043269	51043270	MACS_peak_5739	4.00	lobChIP_FOXA1_robot
chr16	51092086	51092087	MACS_peak_5740	10.00	lobChIP_FOXA1_robot
chr16	51092682	51092683	MACS_peak_5741	5.00	lobChIP_FOXA1_robot
chr16	51154809	51154810	MACS_peak_5742	12.00	lobChIP_FOXA1_robot
chr16	51272119	51272120	MACS_peak_5743	26.00	lobChIP_FOXA1_robot
chr16	51278155	51278156	MACS_peak_5744	8.00	lobChIP_FOXA1_robot
chr16	51361955	51361956	MACS_peak_5745	4.00	lobChIP_FOXA1_robot
chr16	51436987	51436988	MACS_peak_5746	7.00	lobChIP_FOXA1_robot
chr16	51566970	51566971	MACS_peak_5747	5.00	lobChIP_FOXA1_robot
chr16	52061445	52061446	MACS_peak_5748	6.00	lobChIP_FOXA1_robot
chr16	52133466	52133467	MACS_peak_5749	8.00	lobChIP_FOXA1_robot
chr16	52432569	52432570	MACS_peak_5750	10.00	lobChIP_FOXA1_robot
chr16	52442587	52442588	MACS_peak_5751	19.00	lobChIP_FOXA1_robot
chr16	52486189	52486190	MACS_peak_5752	8.00	lobChIP_FOXA1_robot
chr16	52490490	52490491	MACS_peak_5753	7.00	lobChIP_FOXA1_robot
chr16	52522907	52522908	MACS_peak_5754	9.00	lobChIP_FOXA1_robot
chr16	52526922	52526923	MACS_peak_5755	18.00	lobChIP_FOXA1_robot
chr16	52527829	52527830	MACS_peak_5756	5.00	lobChIP_FOXA1_robot
chr16	52528671	52528672	MACS_peak_5757	4.00	lobChIP_FOXA1_robot
chr16	52530581	52530582	MACS_peak_5758	54.00	lobChIP_FOXA1_robot
chr16	52586801	52586802	MACS_peak_5759	5.00	lobChIP_FOXA1_robot
chr16	52596133	52596134	MACS_peak_5760	20.00	lobChIP_FOXA1_robot
chr16	52599067	52599068	MACS_peak_5761	8.00	lobChIP_FOXA1_robot
chr16	52607589	52607590	MACS_peak_5762	3.00	lobChIP_FOXA1_robot
chr16	52612256	52612257	MACS_peak_5763	26.00	lobChIP_FOXA1_robot
chr16	52631820	52631821	MACS_peak_5764	12.00	lobChIP_FOXA1_robot
chr16	52646056	52646057	MACS_peak_5765	23.00	lobChIP_FOXA1_robot
chr16	53082540	53082541	MACS_peak_5766	6.00	lobChIP_FOXA1_robot
chr16	53090512	53090513	MACS_peak_5767	8.00	lobChIP_FOXA1_robot
chr16	53200162	53200163	MACS_peak_5768	5.00	lobChIP_FOXA1_robot
chr16	53545407	53545408	MACS_peak_5769	9.00	lobChIP_FOXA1_robot
chr16	53673076	53673077	MACS_peak_5770	4.00	lobChIP_FOXA1_robot
chr16	53706596	53706597	MACS_peak_5771	11.00	lobChIP_FOXA1_robot
chr16	53790761	53790762	MACS_peak_5772	6.00	lobChIP_FOXA1_robot
chr16	53800603	53800604	MACS_peak_5773	4.00	lobChIP_FOXA1_robot
chr16	53807629	53807630	MACS_peak_5774	4.00	lobChIP_FOXA1_robot
chr16	53809912	53809913	MACS_peak_5775	4.00	lobChIP_FOXA1_robot
chr16	53815318	53815319	MACS_peak_5776	7.00	lobChIP_FOXA1_robot
chr16	53838465	53838466	MACS_peak_5777	4.00	lobChIP_FOXA1_robot
chr16	53882659	53882660	MACS_peak_5778	7.00	lobChIP_FOXA1_robot
chr16	53933108	53933109	MACS_peak_5779	9.00	lobChIP_FOXA1_robot
chr16	54025719	54025720	MACS_peak_5780	4.00	lobChIP_FOXA1_robot
chr16	54032168	54032169	MACS_peak_5781	3.00	lobChIP_FOXA1_robot
chr16	54101759	54101760	MACS_peak_5782	14.00	lobChIP_FOXA1_robot
chr16	54114782	54114783	MACS_peak_5783	8.00	lobChIP_FOXA1_robot
chr16	54137400	54137401	MACS_peak_5784	11.00	lobChIP_FOXA1_robot
chr16	54140628	54140629	MACS_peak_5785	4.00	lobChIP_FOXA1_robot
chr16	54168047	54168048	MACS_peak_5786	5.00	lobChIP_FOXA1_robot
chr16	54195619	54195620	MACS_peak_5787	5.00	lobChIP_FOXA1_robot
chr16	54199501	54199502	MACS_peak_5788	5.00	lobChIP_FOXA1_robot
chr16	54209896	54209897	MACS_peak_5789	31.00	lobChIP_FOXA1_robot
chr16	54215030	54215031	MACS_peak_5790	12.00	lobChIP_FOXA1_robot
chr16	54300551	54300552	MACS_peak_5791	5.00	lobChIP_FOXA1_robot
chr16	54464604	54464605	MACS_peak_5792	16.00	lobChIP_FOXA1_robot
chr16	54662998	54662999	MACS_peak_5793	43.00	lobChIP_FOXA1_robot
chr16	54881769	54881770	MACS_peak_5794	5.00	lobChIP_FOXA1_robot
chr16	55172472	55172473	MACS_peak_5795	3.00	lobChIP_FOXA1_robot
chr16	55540052	55540053	MACS_peak_5796	5.00	lobChIP_FOXA1_robot
chr16	55605243	55605244	MACS_peak_5797	3.00	lobChIP_FOXA1_robot
chr16	55655849	55655850	MACS_peak_5798	3.00	lobChIP_FOXA1_robot
chr16	55657190	55657191	MACS_peak_5799	9.00	lobChIP_FOXA1_robot
chr16	55671051	55671052	MACS_peak_5800	6.00	lobChIP_FOXA1_robot
chr16	55675717	55675718	MACS_peak_5801	3.00	lobChIP_FOXA1_robot
chr16	56640849	56640850	MACS_peak_5802	8.00	lobChIP_FOXA1_robot
chr16	56803251	56803252	MACS_peak_5803	4.00	lobChIP_FOXA1_robot
chr16	56842453	56842454	MACS_peak_5804	8.00	lobChIP_FOXA1_robot
chr16	56849124	56849125	MACS_peak_5805	4.00	lobChIP_FOXA1_robot
chr16	56852047	56852048	MACS_peak_5806	19.00	lobChIP_FOXA1_robot
chr16	56873082	56873083	MACS_peak_5807	14.00	lobChIP_FOXA1_robot
chr16	56953327	56953328	MACS_peak_5808	10.00	lobChIP_FOXA1_robot
chr16	56956480	56956481	MACS_peak_5809	4.00	lobChIP_FOXA1_robot
chr16	56967021	56967022	MACS_peak_5810	19.00	lobChIP_FOXA1_robot
chr16	56983271	56983272	MACS_peak_5811	8.00	lobChIP_FOXA1_robot
chr16	57038686	57038687	MACS_peak_5812	5.00	lobChIP_FOXA1_robot
chr16	57190382	57190383	MACS_peak_5813	6.00	lobChIP_FOXA1_robot
chr16	57228705	57228706	MACS_peak_5814	9.00	lobChIP_FOXA1_robot
chr16	57314100	57314101	MACS_peak_5815	23.00	lobChIP_FOXA1_robot
chr16	57360224	57360225	MACS_peak_5816	4.00	lobChIP_FOXA1_robot
chr16	57407078	57407079	MACS_peak_5817	4.00	lobChIP_FOXA1_robot
chr16	57451201	57451202	MACS_peak_5818	4.00	lobChIP_FOXA1_robot
chr16	57641031	57641032	MACS_peak_5819	7.00	lobChIP_FOXA1_robot
chr16	57738030	57738031	MACS_peak_5820	12.00	lobChIP_FOXA1_robot
chr16	57743721	57743722	MACS_peak_5821	9.00	lobChIP_FOXA1_robot
chr16	57815935	57815936	MACS_peak_5822	3.00	lobChIP_FOXA1_robot
chr16	57838120	57838121	MACS_peak_5823	6.00	lobChIP_FOXA1_robot
chr16	57842154	57842155	MACS_peak_5824	5.00	lobChIP_FOXA1_robot
chr16	57844254	57844255	MACS_peak_5825	5.00	lobChIP_FOXA1_robot
chr16	57860678	57860679	MACS_peak_5826	8.00	lobChIP_FOXA1_robot
chr16	57875038	57875039	MACS_peak_5827	6.00	lobChIP_FOXA1_robot
chr16	57898917	57898918	MACS_peak_5828	4.00	lobChIP_FOXA1_robot
chr16	57927971	57927972	MACS_peak_5829	4.00	lobChIP_FOXA1_robot
chr16	57928736	57928737	MACS_peak_5830	31.00	lobChIP_FOXA1_robot
chr16	58112109	58112110	MACS_peak_5831	5.00	lobChIP_FOXA1_robot
chr16	58486180	58486181	MACS_peak_5832	5.00	lobChIP_FOXA1_robot
chr16	58490393	58490394	MACS_peak_5833	5.00	lobChIP_FOXA1_robot
chr16	58755046	58755047	MACS_peak_5834	30.00	lobChIP_FOXA1_robot
chr16	58807958	58807959	MACS_peak_5835	12.00	lobChIP_FOXA1_robot
chr16	58882147	58882148	MACS_peak_5836	5.00	lobChIP_FOXA1_robot
chr16	59214316	59214317	MACS_peak_5837	10.00	lobChIP_FOXA1_robot
chr16	59736758	59736759	MACS_peak_5838	4.00	lobChIP_FOXA1_robot
chr16	60540887	60540888	MACS_peak_5839	3.00	lobChIP_FOXA1_robot
chr16	61990119	61990120	MACS_peak_5840	4.00	lobChIP_FOXA1_robot
chr16	62923072	62923073	MACS_peak_5841	4.00	lobChIP_FOXA1_robot
chr16	63349332	63349333	MACS_peak_5842	5.00	lobChIP_FOXA1_robot
chr16	63977397	63977398	MACS_peak_5843	3.00	lobChIP_FOXA1_robot
chr16	64195310	64195311	MACS_peak_5844	9.00	lobChIP_FOXA1_robot
chr16	65163216	65163217	MACS_peak_5845	8.00	lobChIP_FOXA1_robot
chr16	65357211	65357212	MACS_peak_5846	4.00	lobChIP_FOXA1_robot
chr16	65675811	65675812	MACS_peak_5847	4.00	lobChIP_FOXA1_robot
chr16	66467687	66467688	MACS_peak_5848	8.00	lobChIP_FOXA1_robot
chr16	66469255	66469256	MACS_peak_5849	4.00	lobChIP_FOXA1_robot
chr16	66547129	66547130	MACS_peak_5850	6.00	lobChIP_FOXA1_robot
chr16	66550949	66550950	MACS_peak_5851	13.00	lobChIP_FOXA1_robot
chr16	66939272	66939273	MACS_peak_5852	5.00	lobChIP_FOXA1_robot
chr16	67029815	67029816	MACS_peak_5853	4.00	lobChIP_FOXA1_robot
chr16	67145251	67145252	MACS_peak_5854	26.00	lobChIP_FOXA1_robot
chr16	67148132	67148133	MACS_peak_5855	8.00	lobChIP_FOXA1_robot
chr16	67512030	67512031	MACS_peak_5856	6.00	lobChIP_FOXA1_robot
chr16	67892024	67892025	MACS_peak_5857	5.00	lobChIP_FOXA1_robot
chr16	67977407	67977408	MACS_peak_5858	8.00	lobChIP_FOXA1_robot
chr16	68113814	68113815	MACS_peak_5859	7.00	lobChIP_FOXA1_robot
chr16	68147191	68147192	MACS_peak_5860	8.00	lobChIP_FOXA1_robot
chr16	68166009	68166010	MACS_peak_5861	5.00	lobChIP_FOXA1_robot
chr16	68271617	68271618	MACS_peak_5862	8.00	lobChIP_FOXA1_robot
chr16	68299277	68299278	MACS_peak_5863	12.00	lobChIP_FOXA1_robot
chr16	68315837	68315838	MACS_peak_5864	7.00	lobChIP_FOXA1_robot
chr16	68334398	68334399	MACS_peak_5865	3.00	lobChIP_FOXA1_robot
chr16	68778804	68778805	MACS_peak_5866	16.00	lobChIP_FOXA1_robot
chr16	68783407	68783408	MACS_peak_5867	9.00	lobChIP_FOXA1_robot
chr16	68824339	68824340	MACS_peak_5868	13.00	lobChIP_FOXA1_robot
chr16	68826742	68826743	MACS_peak_5869	14.00	lobChIP_FOXA1_robot
chr16	68993101	68993102	MACS_peak_5870	14.00	lobChIP_FOXA1_robot
chr16	69026587	69026588	MACS_peak_5871	10.00	lobChIP_FOXA1_robot
chr16	69074670	69074671	MACS_peak_5872	5.00	lobChIP_FOXA1_robot
chr16	69255888	69255889	MACS_peak_5873	12.00	lobChIP_FOXA1_robot
chr16	69384963	69384964	MACS_peak_5874	3.00	lobChIP_FOXA1_robot
chr16	69430621	69430622	MACS_peak_5875	53.00	lobChIP_FOXA1_robot
chr16	69438238	69438239	MACS_peak_5876	5.00	lobChIP_FOXA1_robot
chr16	69499243	69499244	MACS_peak_5877	4.00	lobChIP_FOXA1_robot
chr16	69500044	69500045	MACS_peak_5878	6.00	lobChIP_FOXA1_robot
chr16	69555510	69555511	MACS_peak_5879	4.00	lobChIP_FOXA1_robot
chr16	69579764	69579765	MACS_peak_5880	4.00	lobChIP_FOXA1_robot
chr16	69649627	69649628	MACS_peak_5881	16.00	lobChIP_FOXA1_robot
chr16	69706134	69706135	MACS_peak_5882	9.00	lobChIP_FOXA1_robot
chr16	69714434	69714435	MACS_peak_5883	11.00	lobChIP_FOXA1_robot
chr16	69757855	69757856	MACS_peak_5884	10.00	lobChIP_FOXA1_robot
chr16	69848724	69848725	MACS_peak_5885	6.00	lobChIP_FOXA1_robot
chr16	69856425	69856426	MACS_peak_5886	5.00	lobChIP_FOXA1_robot
chr16	69861080	69861081	MACS_peak_5887	4.00	lobChIP_FOXA1_robot
chr16	69864833	69864834	MACS_peak_5888	6.00	lobChIP_FOXA1_robot
chr16	69892329	69892330	MACS_peak_5889	4.00	lobChIP_FOXA1_robot
chr16	69893005	69893006	MACS_peak_5890	4.00	lobChIP_FOXA1_robot
chr16	70248023	70248024	MACS_peak_5891	9.00	lobChIP_FOXA1_robot
chr16	70333411	70333412	MACS_peak_5892	5.00	lobChIP_FOXA1_robot
chr16	70462712	70462713	MACS_peak_5893	4.00	lobChIP_FOXA1_robot
chr16	70716117	70716118	MACS_peak_5894	5.00	lobChIP_FOXA1_robot
chr16	70745487	70745488	MACS_peak_5895	5.00	lobChIP_FOXA1_robot
chr16	70759468	70759469	MACS_peak_5896	10.00	lobChIP_FOXA1_robot
chr16	70780020	70780021	MACS_peak_5897	6.00	lobChIP_FOXA1_robot
chr16	70780790	70780791	MACS_peak_5898	14.00	lobChIP_FOXA1_robot
chr16	70794869	70794870	MACS_peak_5899	5.00	lobChIP_FOXA1_robot
chr16	71599914	71599915	MACS_peak_5900	5.00	lobChIP_FOXA1_robot
chr16	71606777	71606778	MACS_peak_5901	5.00	lobChIP_FOXA1_robot
chr16	71619293	71619294	MACS_peak_5902	14.00	lobChIP_FOXA1_robot
chr16	71627580	71627581	MACS_peak_5903	8.00	lobChIP_FOXA1_robot
chr16	71628212	71628213	MACS_peak_5904	7.00	lobChIP_FOXA1_robot
chr16	71629582	71629583	MACS_peak_5905	10.00	lobChIP_FOXA1_robot
chr16	71632496	71632497	MACS_peak_5906	6.00	lobChIP_FOXA1_robot
chr16	71704612	71704613	MACS_peak_5907	7.00	lobChIP_FOXA1_robot
chr16	71849117	71849118	MACS_peak_5908	5.00	lobChIP_FOXA1_robot
chr16	71937983	71937984	MACS_peak_5909	5.00	lobChIP_FOXA1_robot
chr16	72051135	72051136	MACS_peak_5910	5.00	lobChIP_FOXA1_robot
chr16	72106885	72106886	MACS_peak_5911	7.00	lobChIP_FOXA1_robot
chr16	72343028	72343029	MACS_peak_5912	19.00	lobChIP_FOXA1_robot
chr16	72396675	72396676	MACS_peak_5913	5.00	lobChIP_FOXA1_robot
chr16	72449519	72449520	MACS_peak_5914	12.00	lobChIP_FOXA1_robot
chr16	72490387	72490388	MACS_peak_5915	7.00	lobChIP_FOXA1_robot
chr16	72602544	72602545	MACS_peak_5916	6.00	lobChIP_FOXA1_robot
chr16	72711829	72711830	MACS_peak_5917	6.00	lobChIP_FOXA1_robot
chr16	72713297	72713298	MACS_peak_5918	4.00	lobChIP_FOXA1_robot
chr16	72911522	72911523	MACS_peak_5919	5.00	lobChIP_FOXA1_robot
chr16	72948478	72948479	MACS_peak_5920	9.00	lobChIP_FOXA1_robot
chr16	72959042	72959043	MACS_peak_5921	22.00	lobChIP_FOXA1_robot
chr16	72961800	72961801	MACS_peak_5922	11.00	lobChIP_FOXA1_robot
chr16	73010582	73010583	MACS_peak_5923	6.00	lobChIP_FOXA1_robot
chr16	73050088	73050089	MACS_peak_5924	7.00	lobChIP_FOXA1_robot
chr16	73054483	73054484	MACS_peak_5925	15.00	lobChIP_FOXA1_robot
chr16	73087796	73087797	MACS_peak_5926	4.00	lobChIP_FOXA1_robot
chr16	73089878	73089879	MACS_peak_5927	18.00	lobChIP_FOXA1_robot
chr16	73104560	73104561	MACS_peak_5928	4.00	lobChIP_FOXA1_robot
chr16	73221332	73221333	MACS_peak_5929	6.00	lobChIP_FOXA1_robot
chr16	74031210	74031211	MACS_peak_5930	3.00	lobChIP_FOXA1_robot
chr16	74330460	74330461	MACS_peak_5931	8.00	lobChIP_FOXA1_robot
chr16	74545519	74545520	MACS_peak_5932	3.00	lobChIP_FOXA1_robot
chr16	74669454	74669455	MACS_peak_5933	6.00	lobChIP_FOXA1_robot
chr16	75022981	75022982	MACS_peak_5934	6.00	lobChIP_FOXA1_robot
chr16	75025711	75025712	MACS_peak_5935	9.00	lobChIP_FOXA1_robot
chr16	75088317	75088318	MACS_peak_5936	8.00	lobChIP_FOXA1_robot
chr16	75104513	75104514	MACS_peak_5937	17.00	lobChIP_FOXA1_robot
chr16	75121418	75121419	MACS_peak_5938	6.00	lobChIP_FOXA1_robot
chr16	75339444	75339445	MACS_peak_5939	6.00	lobChIP_FOXA1_robot
chr16	75340455	75340456	MACS_peak_5940	8.00	lobChIP_FOXA1_robot
chr16	75344062	75344063	MACS_peak_5941	6.00	lobChIP_FOXA1_robot
chr16	75458121	75458122	MACS_peak_5942	5.00	lobChIP_FOXA1_robot
chr16	75480488	75480489	MACS_peak_5943	8.00	lobChIP_FOXA1_robot
chr16	75628282	75628283	MACS_peak_5944	4.00	lobChIP_FOXA1_robot
chr16	75682607	75682608	MACS_peak_5945	5.00	lobChIP_FOXA1_robot
chr16	76608829	76608830	MACS_peak_5946	17.00	lobChIP_FOXA1_robot
chr16	76684669	76684670	MACS_peak_5947	7.00	lobChIP_FOXA1_robot
chr16	77222235	77222236	MACS_peak_5948	5.00	lobChIP_FOXA1_robot
chr16	77339070	77339071	MACS_peak_5949	8.00	lobChIP_FOXA1_robot
chr16	77387774	77387775	MACS_peak_5950	3.00	lobChIP_FOXA1_robot
chr16	77392545	77392546	MACS_peak_5951	4.00	lobChIP_FOXA1_robot
chr16	77393133	77393134	MACS_peak_5952	10.00	lobChIP_FOXA1_robot
chr16	77750705	77750706	MACS_peak_5953	5.00	lobChIP_FOXA1_robot
chr16	77771001	77771002	MACS_peak_5954	5.00	lobChIP_FOXA1_robot
chr16	77783527	77783528	MACS_peak_5955	5.00	lobChIP_FOXA1_robot
chr16	77816852	77816853	MACS_peak_5956	3.00	lobChIP_FOXA1_robot
chr16	77847155	77847156	MACS_peak_5957	6.00	lobChIP_FOXA1_robot
chr16	78157105	78157106	MACS_peak_5958	3.00	lobChIP_FOXA1_robot
chr16	78163396	78163397	MACS_peak_5959	4.00	lobChIP_FOXA1_robot
chr16	78168058	78168059	MACS_peak_5960	19.00	lobChIP_FOXA1_robot
chr16	78169115	78169116	MACS_peak_5961	6.00	lobChIP_FOXA1_robot
chr16	78175254	78175255	MACS_peak_5962	6.00	lobChIP_FOXA1_robot
chr16	78234833	78234834	MACS_peak_5963	6.00	lobChIP_FOXA1_robot
chr16	78286843	78286844	MACS_peak_5964	14.00	lobChIP_FOXA1_robot
chr16	78331130	78331131	MACS_peak_5965	16.00	lobChIP_FOXA1_robot
chr16	78514677	78514678	MACS_peak_5966	6.00	lobChIP_FOXA1_robot
chr16	78517484	78517485	MACS_peak_5967	6.00	lobChIP_FOXA1_robot
chr16	78519492	78519493	MACS_peak_5968	5.00	lobChIP_FOXA1_robot
chr16	78630998	78630999	MACS_peak_5969	9.00	lobChIP_FOXA1_robot
chr16	78763918	78763919	MACS_peak_5970	6.00	lobChIP_FOXA1_robot
chr16	78768710	78768711	MACS_peak_5971	10.00	lobChIP_FOXA1_robot
chr16	79635617	79635618	MACS_peak_5972	4.00	lobChIP_FOXA1_robot
chr16	80299816	80299817	MACS_peak_5973	5.00	lobChIP_FOXA1_robot
chr16	80441563	80441564	MACS_peak_5974	9.00	lobChIP_FOXA1_robot
chr16	80948489	80948490	MACS_peak_5975	5.00	lobChIP_FOXA1_robot
chr16	81025604	81025605	MACS_peak_5976	15.00	lobChIP_FOXA1_robot
chr16	81209036	81209037	MACS_peak_5977	6.00	lobChIP_FOXA1_robot
chr16	81383323	81383324	MACS_peak_5978	6.00	lobChIP_FOXA1_robot
chr16	81464988	81464989	MACS_peak_5979	4.00	lobChIP_FOXA1_robot
chr16	81485844	81485845	MACS_peak_5980	4.00	lobChIP_FOXA1_robot
chr16	81491552	81491553	MACS_peak_5981	5.00	lobChIP_FOXA1_robot
chr16	81519188	81519189	MACS_peak_5982	3.00	lobChIP_FOXA1_robot
chr16	81592564	81592565	MACS_peak_5983	7.00	lobChIP_FOXA1_robot
chr16	81802013	81802014	MACS_peak_5984	19.00	lobChIP_FOXA1_robot
chr16	82084892	82084893	MACS_peak_5985	5.00	lobChIP_FOXA1_robot
chr16	82089902	82089903	MACS_peak_5986	12.00	lobChIP_FOXA1_robot
chr16	82116564	82116565	MACS_peak_5987	6.00	lobChIP_FOXA1_robot
chr16	82333949	82333950	MACS_peak_5988	4.00	lobChIP_FOXA1_robot
chr16	82363581	82363582	MACS_peak_5989	8.00	lobChIP_FOXA1_robot
chr16	82558768	82558769	MACS_peak_5990	15.00	lobChIP_FOXA1_robot
chr16	82913440	82913441	MACS_peak_5991	4.00	lobChIP_FOXA1_robot
chr16	83976690	83976691	MACS_peak_5992	14.00	lobChIP_FOXA1_robot
chr16	84077066	84077067	MACS_peak_5993	4.00	lobChIP_FOXA1_robot
chr16	84082177	84082178	MACS_peak_5994	6.00	lobChIP_FOXA1_robot
chr16	84130372	84130373	MACS_peak_5995	24.00	lobChIP_FOXA1_robot
chr16	84448859	84448860	MACS_peak_5996	6.00	lobChIP_FOXA1_robot
chr16	84620628	84620629	MACS_peak_5997	5.00	lobChIP_FOXA1_robot
chr16	84633299	84633300	MACS_peak_5998	5.00	lobChIP_FOXA1_robot
chr16	84747127	84747128	MACS_peak_5999	7.00	lobChIP_FOXA1_robot
chr16	84763800	84763801	MACS_peak_6000	3.00	lobChIP_FOXA1_robot
chr16	84765612	84765613	MACS_peak_6001	7.00	lobChIP_FOXA1_robot
chr16	84797235	84797236	MACS_peak_6002	3.00	lobChIP_FOXA1_robot
chr16	84885075	84885076	MACS_peak_6003	7.00	lobChIP_FOXA1_robot
chr16	85010580	85010581	MACS_peak_6004	3.00	lobChIP_FOXA1_robot
chr16	85028607	85028608	MACS_peak_6005	21.00	lobChIP_FOXA1_robot
chr16	85032996	85032997	MACS_peak_6006	15.00	lobChIP_FOXA1_robot
chr16	85034720	85034721	MACS_peak_6007	5.00	lobChIP_FOXA1_robot
chr16	85035699	85035700	MACS_peak_6008	5.00	lobChIP_FOXA1_robot
chr16	85322740	85322741	MACS_peak_6009	4.00	lobChIP_FOXA1_robot
chr16	85341436	85341437	MACS_peak_6010	18.00	lobChIP_FOXA1_robot
chr16	85456575	85456576	MACS_peak_6011	7.00	lobChIP_FOXA1_robot
chr16	85482668	85482669	MACS_peak_6012	7.00	lobChIP_FOXA1_robot
chr16	85548736	85548737	MACS_peak_6013	12.00	lobChIP_FOXA1_robot
chr16	85558260	85558261	MACS_peak_6014	5.00	lobChIP_FOXA1_robot
chr16	85569150	85569151	MACS_peak_6015	8.00	lobChIP_FOXA1_robot
chr16	85881720	85881721	MACS_peak_6016	8.00	lobChIP_FOXA1_robot
chr16	86134113	86134114	MACS_peak_6017	5.00	lobChIP_FOXA1_robot
chr16	87274602	87274603	MACS_peak_6018	7.00	lobChIP_FOXA1_robot
chr16	87279993	87279994	MACS_peak_6019	7.00	lobChIP_FOXA1_robot
chr16	87298544	87298545	MACS_peak_6020	4.00	lobChIP_FOXA1_robot
chr16	87389473	87389474	MACS_peak_6021	4.00	lobChIP_FOXA1_robot
chr16	87413024	87413025	MACS_peak_6022	7.00	lobChIP_FOXA1_robot
chr16	87416018	87416019	MACS_peak_6023	52.00	lobChIP_FOXA1_robot
chr16	87422002	87422003	MACS_peak_6024	8.00	lobChIP_FOXA1_robot
chr16	87570184	87570185	MACS_peak_6025	3.00	lobChIP_FOXA1_robot
chr16	87739789	87739790	MACS_peak_6026	18.00	lobChIP_FOXA1_robot
chr16	87822629	87822630	MACS_peak_6027	6.00	lobChIP_FOXA1_robot
chr16	87823300	87823301	MACS_peak_6028	14.00	lobChIP_FOXA1_robot
chr16	87881808	87881809	MACS_peak_6029	4.00	lobChIP_FOXA1_robot
chr16	87887984	87887985	MACS_peak_6030	6.00	lobChIP_FOXA1_robot
chr16	87902109	87902110	MACS_peak_6031	4.00	lobChIP_FOXA1_robot
chr16	87904408	87904409	MACS_peak_6032	3.00	lobChIP_FOXA1_robot
chr16	87970265	87970266	MACS_peak_6033	5.00	lobChIP_FOXA1_robot
chr16	87981914	87981915	MACS_peak_6034	6.00	lobChIP_FOXA1_robot
chr16	88152741	88152742	MACS_peak_6035	5.00	lobChIP_FOXA1_robot
chr16	88555067	88555068	MACS_peak_6036	16.00	lobChIP_FOXA1_robot
chr16	88664474	88664475	MACS_peak_6037	4.00	lobChIP_FOXA1_robot
chr16	88685098	88685099	MACS_peak_6038	4.00	lobChIP_FOXA1_robot
chr16	88830182	88830183	MACS_peak_6039	13.00	lobChIP_FOXA1_robot
chr16	89253117	89253118	MACS_peak_6040	6.00	lobChIP_FOXA1_robot
chr16	89264394	89264395	MACS_peak_6041	7.00	lobChIP_FOXA1_robot
chr16	89298607	89298608	MACS_peak_6042	4.00	lobChIP_FOXA1_robot
chr16	89312995	89312996	MACS_peak_6043	9.00	lobChIP_FOXA1_robot
chr16	89412305	89412306	MACS_peak_6044	3.00	lobChIP_FOXA1_robot
chr16	89522225	89522226	MACS_peak_6045	5.00	lobChIP_FOXA1_robot
chr16	89523029	89523030	MACS_peak_6046	5.00	lobChIP_FOXA1_robot
chr16	89545335	89545336	MACS_peak_6047	5.00	lobChIP_FOXA1_robot
chr16	89571690	89571691	MACS_peak_6048	3.00	lobChIP_FOXA1_robot
chr16	89889587	89889588	MACS_peak_6049	3.00	lobChIP_FOXA1_robot
chr16	89904342	89904343	MACS_peak_6050	8.00	lobChIP_FOXA1_robot
chr16	90124499	90124500	MACS_peak_6051	6.00	lobChIP_FOXA1_robot
chr17	132485	132486	MACS_peak_6052	5.00	lobChIP_FOXA1_robot
chr17	198638	198639	MACS_peak_6053	4.00	lobChIP_FOXA1_robot
chr17	202596	202597	MACS_peak_6054	4.00	lobChIP_FOXA1_robot
chr17	474023	474024	MACS_peak_6055	4.00	lobChIP_FOXA1_robot
chr17	619711	619712	MACS_peak_6056	5.00	lobChIP_FOXA1_robot
chr17	833790	833791	MACS_peak_6057	25.00	lobChIP_FOXA1_robot
chr17	858161	858162	MACS_peak_6058	7.00	lobChIP_FOXA1_robot
chr17	1018516	1018517	MACS_peak_6059	5.00	lobChIP_FOXA1_robot
chr17	1360399	1360400	MACS_peak_6060	3.00	lobChIP_FOXA1_robot
chr17	1493544	1493545	MACS_peak_6061	7.00	lobChIP_FOXA1_robot
chr17	1639835	1639836	MACS_peak_6062	16.00	lobChIP_FOXA1_robot
chr17	1673556	1673557	MACS_peak_6063	15.00	lobChIP_FOXA1_robot
chr17	1970138	1970139	MACS_peak_6064	17.00	lobChIP_FOXA1_robot
chr17	1998688	1998689	MACS_peak_6065	3.00	lobChIP_FOXA1_robot
chr17	2046289	2046290	MACS_peak_6066	5.00	lobChIP_FOXA1_robot
chr17	2078160	2078161	MACS_peak_6067	6.00	lobChIP_FOXA1_robot
chr17	2389036	2389037	MACS_peak_6068	6.00	lobChIP_FOXA1_robot
chr17	2855568	2855569	MACS_peak_6069	7.00	lobChIP_FOXA1_robot
chr17	2858416	2858417	MACS_peak_6070	6.00	lobChIP_FOXA1_robot
chr17	2863574	2863575	MACS_peak_6071	8.00	lobChIP_FOXA1_robot
chr17	3377742	3377743	MACS_peak_6072	5.00	lobChIP_FOXA1_robot
chr17	3698902	3698903	MACS_peak_6073	6.00	lobChIP_FOXA1_robot
chr17	3706439	3706440	MACS_peak_6074	10.00	lobChIP_FOXA1_robot
chr17	4219524	4219525	MACS_peak_6075	5.00	lobChIP_FOXA1_robot
chr17	4558950	4558951	MACS_peak_6076	3.00	lobChIP_FOXA1_robot
chr17	4582977	4582978	MACS_peak_6077	4.00	lobChIP_FOXA1_robot
chr17	4624962	4624963	MACS_peak_6078	9.00	lobChIP_FOXA1_robot
chr17	4663263	4663264	MACS_peak_6079	12.00	lobChIP_FOXA1_robot
chr17	4681907	4681908	MACS_peak_6080	5.00	lobChIP_FOXA1_robot
chr17	4691252	4691253	MACS_peak_6081	14.00	lobChIP_FOXA1_robot
chr17	5135674	5135675	MACS_peak_6082	85.00	lobChIP_FOXA1_robot
chr17	5217377	5217378	MACS_peak_6083	11.00	lobChIP_FOXA1_robot
chr17	5308989	5308990	MACS_peak_6084	5.00	lobChIP_FOXA1_robot
chr17	5419608	5419609	MACS_peak_6085	6.00	lobChIP_FOXA1_robot
chr17	5740535	5740536	MACS_peak_6086	7.00	lobChIP_FOXA1_robot
chr17	6043957	6043958	MACS_peak_6087	4.00	lobChIP_FOXA1_robot
chr17	6586751	6586752	MACS_peak_6088	13.00	lobChIP_FOXA1_robot
chr17	6603527	6603528	MACS_peak_6089	7.00	lobChIP_FOXA1_robot
chr17	6699443	6699444	MACS_peak_6090	6.00	lobChIP_FOXA1_robot
chr17	7038997	7038998	MACS_peak_6091	5.00	lobChIP_FOXA1_robot
chr17	7082764	7082765	MACS_peak_6092	4.00	lobChIP_FOXA1_robot
chr17	7170436	7170437	MACS_peak_6093	5.00	lobChIP_FOXA1_robot
chr17	7766639	7766640	MACS_peak_6094	5.00	lobChIP_FOXA1_robot
chr17	7935448	7935449	MACS_peak_6095	4.00	lobChIP_FOXA1_robot
chr17	7959959	7959960	MACS_peak_6096	63.00	lobChIP_FOXA1_robot
chr17	8076342	8076343	MACS_peak_6097	4.00	lobChIP_FOXA1_robot
chr17	8114604	8114605	MACS_peak_6098	35.00	lobChIP_FOXA1_robot
chr17	8177430	8177431	MACS_peak_6099	3.00	lobChIP_FOXA1_robot
chr17	8323755	8323756	MACS_peak_6100	12.00	lobChIP_FOXA1_robot
chr17	8351998	8351999	MACS_peak_6101	3.00	lobChIP_FOXA1_robot
chr17	8353810	8353811	MACS_peak_6102	6.00	lobChIP_FOXA1_robot
chr17	8435519	8435520	MACS_peak_6103	32.00	lobChIP_FOXA1_robot
chr17	8446690	8446691	MACS_peak_6104	11.00	lobChIP_FOXA1_robot
chr17	8517101	8517102	MACS_peak_6105	4.00	lobChIP_FOXA1_robot
chr17	8552689	8552690	MACS_peak_6106	4.00	lobChIP_FOXA1_robot
chr17	8626118	8626119	MACS_peak_6107	4.00	lobChIP_FOXA1_robot
chr17	8657395	8657396	MACS_peak_6108	16.00	lobChIP_FOXA1_robot
chr17	8692512	8692513	MACS_peak_6109	11.00	lobChIP_FOXA1_robot
chr17	8775535	8775536	MACS_peak_6110	4.00	lobChIP_FOXA1_robot
chr17	9054619	9054620	MACS_peak_6111	18.00	lobChIP_FOXA1_robot
chr17	9161653	9161654	MACS_peak_6112	7.00	lobChIP_FOXA1_robot
chr17	9264727	9264728	MACS_peak_6113	5.00	lobChIP_FOXA1_robot
chr17	9632542	9632543	MACS_peak_6114	8.00	lobChIP_FOXA1_robot
chr17	9665009	9665010	MACS_peak_6115	8.00	lobChIP_FOXA1_robot
chr17	9745820	9745821	MACS_peak_6116	5.00	lobChIP_FOXA1_robot
chr17	10351035	10351036	MACS_peak_6117	4.00	lobChIP_FOXA1_robot
chr17	10647954	10647955	MACS_peak_6118	9.00	lobChIP_FOXA1_robot
chr17	11512009	11512010	MACS_peak_6119	5.00	lobChIP_FOXA1_robot
chr17	12307089	12307090	MACS_peak_6120	3.00	lobChIP_FOXA1_robot
chr17	13010373	13010374	MACS_peak_6121	5.00	lobChIP_FOXA1_robot
chr17	13225737	13225738	MACS_peak_6122	5.00	lobChIP_FOXA1_robot
chr17	13307366	13307367	MACS_peak_6123	4.00	lobChIP_FOXA1_robot
chr17	13314375	13314376	MACS_peak_6124	12.00	lobChIP_FOXA1_robot
chr17	13324383	13324384	MACS_peak_6125	16.00	lobChIP_FOXA1_robot
chr17	13837029	13837030	MACS_peak_6126	5.00	lobChIP_FOXA1_robot
chr17	13855715	13855716	MACS_peak_6127	8.00	lobChIP_FOXA1_robot
chr17	13956703	13956704	MACS_peak_6128	5.00	lobChIP_FOXA1_robot
chr17	14229265	14229266	MACS_peak_6129	10.00	lobChIP_FOXA1_robot
chr17	14265310	14265311	MACS_peak_6130	7.00	lobChIP_FOXA1_robot
chr17	14276564	14276565	MACS_peak_6131	18.00	lobChIP_FOXA1_robot
chr17	14278305	14278306	MACS_peak_6132	4.00	lobChIP_FOXA1_robot
chr17	14280430	14280431	MACS_peak_6133	6.00	lobChIP_FOXA1_robot
chr17	14602386	14602387	MACS_peak_6134	3.00	lobChIP_FOXA1_robot
chr17	14603345	14603346	MACS_peak_6135	22.00	lobChIP_FOXA1_robot
chr17	15336598	15336599	MACS_peak_6136	5.00	lobChIP_FOXA1_robot
chr17	15337799	15337800	MACS_peak_6137	4.00	lobChIP_FOXA1_robot
chr17	15869542	15869543	MACS_peak_6138	6.00	lobChIP_FOXA1_robot
chr17	15944382	15944383	MACS_peak_6139	6.00	lobChIP_FOXA1_robot
chr17	15985086	15985087	MACS_peak_6140	5.00	lobChIP_FOXA1_robot
chr17	16075161	16075162	MACS_peak_6141	6.00	lobChIP_FOXA1_robot
chr17	16192622	16192623	MACS_peak_6142	9.00	lobChIP_FOXA1_robot
chr17	16200551	16200552	MACS_peak_6143	5.00	lobChIP_FOXA1_robot
chr17	16348995	16348996	MACS_peak_6144	5.00	lobChIP_FOXA1_robot
chr17	16438824	16438825	MACS_peak_6145	4.00	lobChIP_FOXA1_robot
chr17	17273370	17273371	MACS_peak_6146	5.00	lobChIP_FOXA1_robot
chr17	17283012	17283013	MACS_peak_6147	4.00	lobChIP_FOXA1_robot
chr17	17285182	17285183	MACS_peak_6148	3.00	lobChIP_FOXA1_robot
chr17	17312015	17312016	MACS_peak_6149	5.00	lobChIP_FOXA1_robot
chr17	17321533	17321534	MACS_peak_6150	4.00	lobChIP_FOXA1_robot
chr17	17346715	17346716	MACS_peak_6151	18.00	lobChIP_FOXA1_robot
chr17	17349742	17349743	MACS_peak_6152	54.00	lobChIP_FOXA1_robot
chr17	17437447	17437448	MACS_peak_6153	46.00	lobChIP_FOXA1_robot
chr17	17442086	17442087	MACS_peak_6154	16.00	lobChIP_FOXA1_robot
chr17	17485790	17485791	MACS_peak_6155	4.00	lobChIP_FOXA1_robot
chr17	17921466	17921467	MACS_peak_6156	5.00	lobChIP_FOXA1_robot
chr17	17978364	17978365	MACS_peak_6157	3.00	lobChIP_FOXA1_robot
chr17	18828762	18828763	MACS_peak_6158	4.00	lobChIP_FOXA1_robot
chr17	18909567	18909568	MACS_peak_6159	8.00	lobChIP_FOXA1_robot
chr17	19453305	19453306	MACS_peak_6160	6.00	lobChIP_FOXA1_robot
chr17	19545994	19545995	MACS_peak_6161	3.00	lobChIP_FOXA1_robot
chr17	19550782	19550783	MACS_peak_6162	6.00	lobChIP_FOXA1_robot
chr17	19561450	19561451	MACS_peak_6163	4.00	lobChIP_FOXA1_robot
chr17	19563836	19563837	MACS_peak_6164	24.00	lobChIP_FOXA1_robot
chr17	19575425	19575426	MACS_peak_6165	3.00	lobChIP_FOXA1_robot
chr17	19637450	19637451	MACS_peak_6166	9.00	lobChIP_FOXA1_robot
chr17	19651868	19651869	MACS_peak_6167	11.00	lobChIP_FOXA1_robot
chr17	19685007	19685008	MACS_peak_6168	12.00	lobChIP_FOXA1_robot
chr17	19841031	19841032	MACS_peak_6169	5.00	lobChIP_FOXA1_robot
chr17	20868998	20868999	MACS_peak_6170	4.00	lobChIP_FOXA1_robot
chr17	20997757	20997758	MACS_peak_6171	6.00	lobChIP_FOXA1_robot
chr17	21678464	21678465	MACS_peak_6172	4.00	lobChIP_FOXA1_robot
chr17	25574605	25574606	MACS_peak_6173	8.00	lobChIP_FOXA1_robot
chr17	25592441	25592442	MACS_peak_6174	4.00	lobChIP_FOXA1_robot
chr17	25785365	25785366	MACS_peak_6175	4.00	lobChIP_FOXA1_robot
chr17	25879124	25879125	MACS_peak_6176	6.00	lobChIP_FOXA1_robot
chr17	25897426	25897427	MACS_peak_6177	5.00	lobChIP_FOXA1_robot
chr17	26385145	26385146	MACS_peak_6178	9.00	lobChIP_FOXA1_robot
chr17	26516832	26516833	MACS_peak_6179	5.00	lobChIP_FOXA1_robot
chr17	26662476	26662477	MACS_peak_6180	4.00	lobChIP_FOXA1_robot
chr17	26697710	26697711	MACS_peak_6181	28.00	lobChIP_FOXA1_robot
chr17	26989718	26989719	MACS_peak_6182	4.00	lobChIP_FOXA1_robot
chr17	27046588	27046589	MACS_peak_6183	4.00	lobChIP_FOXA1_robot
chr17	27335780	27335781	MACS_peak_6184	5.00	lobChIP_FOXA1_robot
chr17	27485912	27485913	MACS_peak_6185	7.00	lobChIP_FOXA1_robot
chr17	27882941	27882942	MACS_peak_6186	7.00	lobChIP_FOXA1_robot
chr17	28071998	28071999	MACS_peak_6187	8.00	lobChIP_FOXA1_robot
chr17	28258182	28258183	MACS_peak_6188	6.00	lobChIP_FOXA1_robot
chr17	28553166	28553167	MACS_peak_6189	5.00	lobChIP_FOXA1_robot
chr17	28660723	28660724	MACS_peak_6190	5.00	lobChIP_FOXA1_robot
chr17	28671500	28671501	MACS_peak_6191	16.00	lobChIP_FOXA1_robot
chr17	28673611	28673612	MACS_peak_6192	5.00	lobChIP_FOXA1_robot
chr17	28703816	28703817	MACS_peak_6193	4.00	lobChIP_FOXA1_robot
chr17	28722577	28722578	MACS_peak_6194	6.00	lobChIP_FOXA1_robot
chr17	28777892	28777893	MACS_peak_6195	5.00	lobChIP_FOXA1_robot
chr17	29116918	29116919	MACS_peak_6196	6.00	lobChIP_FOXA1_robot
chr17	29243293	29243294	MACS_peak_6197	16.00	lobChIP_FOXA1_robot
chr17	29277921	29277922	MACS_peak_6198	5.00	lobChIP_FOXA1_robot
chr17	29601413	29601414	MACS_peak_6199	11.00	lobChIP_FOXA1_robot
chr17	29655048	29655049	MACS_peak_6200	4.00	lobChIP_FOXA1_robot
chr17	29706959	29706960	MACS_peak_6201	4.00	lobChIP_FOXA1_robot
chr17	29795366	29795367	MACS_peak_6202	4.00	lobChIP_FOXA1_robot
chr17	29802699	29802700	MACS_peak_6203	19.00	lobChIP_FOXA1_robot
chr17	30130834	30130835	MACS_peak_6204	17.00	lobChIP_FOXA1_robot
chr17	30170255	30170256	MACS_peak_6205	3.00	lobChIP_FOXA1_robot
chr17	30629299	30629300	MACS_peak_6206	6.00	lobChIP_FOXA1_robot
chr17	31158712	31158713	MACS_peak_6207	4.00	lobChIP_FOXA1_robot
chr17	31254378	31254379	MACS_peak_6208	7.00	lobChIP_FOXA1_robot
chr17	31611220	31611221	MACS_peak_6209	11.00	lobChIP_FOXA1_robot
chr17	33390801	33390802	MACS_peak_6210	8.00	lobChIP_FOXA1_robot
chr17	33402594	33402595	MACS_peak_6211	4.00	lobChIP_FOXA1_robot
chr17	33414575	33414576	MACS_peak_6212	14.00	lobChIP_FOXA1_robot
chr17	33415261	33415262	MACS_peak_6213	6.00	lobChIP_FOXA1_robot
chr17	34300668	34300669	MACS_peak_6215	47.00	lobChIP_FOXA1_robot
chr17	34304557	34304558	MACS_peak_6216	3.00	lobChIP_FOXA1_robot
chr17	34307150	34307151	MACS_peak_6217	11.00	lobChIP_FOXA1_robot
chr17	34328013	34328014	MACS_peak_6218	6.00	lobChIP_FOXA1_robot
chr17	35364475	35364476	MACS_peak_6219	3.00	lobChIP_FOXA1_robot
chr17	35400732	35400733	MACS_peak_6220	4.00	lobChIP_FOXA1_robot
chr17	35424984	35424985	MACS_peak_6221	3.00	lobChIP_FOXA1_robot
chr17	35504648	35504649	MACS_peak_6222	19.00	lobChIP_FOXA1_robot
chr17	35848578	35848579	MACS_peak_6223	6.00	lobChIP_FOXA1_robot
chr17	35862287	35862288	MACS_peak_6224	14.00	lobChIP_FOXA1_robot
chr17	36036238	36036239	MACS_peak_6225	8.00	lobChIP_FOXA1_robot
chr17	36043582	36043583	MACS_peak_6226	24.00	lobChIP_FOXA1_robot
chr17	36049407	36049408	MACS_peak_6227	11.00	lobChIP_FOXA1_robot
chr17	36070508	36070509	MACS_peak_6228	5.00	lobChIP_FOXA1_robot
chr17	36077040	36077041	MACS_peak_6229	13.00	lobChIP_FOXA1_robot
chr17	36190044	36190045	MACS_peak_6230	4.00	lobChIP_FOXA1_robot
chr17	36514699	36514700	MACS_peak_6231	3.00	lobChIP_FOXA1_robot
chr17	36770491	36770492	MACS_peak_6232	13.00	lobChIP_FOXA1_robot
chr17	36777131	36777132	MACS_peak_6233	9.00	lobChIP_FOXA1_robot
chr17	36813876	36813877	MACS_peak_6234	3.00	lobChIP_FOXA1_robot
chr17	36860251	36860252	MACS_peak_6235	7.00	lobChIP_FOXA1_robot
chr17	36902942	36902943	MACS_peak_6236	3.00	lobChIP_FOXA1_robot
chr17	37027988	37027989	MACS_peak_6237	4.00	lobChIP_FOXA1_robot
chr17	37139486	37139487	MACS_peak_6238	18.00	lobChIP_FOXA1_robot
chr17	37404826	37404827	MACS_peak_6239	4.00	lobChIP_FOXA1_robot
chr17	37413081	37413082	MACS_peak_6240	7.00	lobChIP_FOXA1_robot
chr17	37610066	37610067	MACS_peak_6241	18.00	lobChIP_FOXA1_robot
chr17	37617486	37617487	MACS_peak_6242	18.00	lobChIP_FOXA1_robot
chr17	37851891	37851892	MACS_peak_6243	12.00	lobChIP_FOXA1_robot
chr17	37859802	37859803	MACS_peak_6244	7.00	lobChIP_FOXA1_robot
chr17	37893156	37893157	MACS_peak_6245	13.00	lobChIP_FOXA1_robot
chr17	37895935	37895936	MACS_peak_6246	6.00	lobChIP_FOXA1_robot
chr17	38080717	38080718	MACS_peak_6247	5.00	lobChIP_FOXA1_robot
chr17	38117169	38117170	MACS_peak_6248	7.00	lobChIP_FOXA1_robot
chr17	38255096	38255097	MACS_peak_6249	3.00	lobChIP_FOXA1_robot
chr17	38257573	38257574	MACS_peak_6250	15.00	lobChIP_FOXA1_robot
chr17	38270077	38270078	MACS_peak_6251	7.00	lobChIP_FOXA1_robot
chr17	38271049	38271050	MACS_peak_6252	16.00	lobChIP_FOXA1_robot
chr17	38329839	38329840	MACS_peak_6253	4.00	lobChIP_FOXA1_robot
chr17	38442884	38442885	MACS_peak_6254	6.00	lobChIP_FOXA1_robot
chr17	38491272	38491273	MACS_peak_6255	6.00	lobChIP_FOXA1_robot
chr17	38509240	38509241	MACS_peak_6256	10.00	lobChIP_FOXA1_robot
chr17	38576030	38576031	MACS_peak_6257	7.00	lobChIP_FOXA1_robot
chr17	38605197	38605198	MACS_peak_6258	5.00	lobChIP_FOXA1_robot
chr17	38642468	38642469	MACS_peak_6259	7.00	lobChIP_FOXA1_robot
chr17	38653086	38653087	MACS_peak_6260	4.00	lobChIP_FOXA1_robot
chr17	38673123	38673124	MACS_peak_6261	15.00	lobChIP_FOXA1_robot
chr17	38689893	38689894	MACS_peak_6262	5.00	lobChIP_FOXA1_robot
chr17	38705724	38705725	MACS_peak_6263	7.00	lobChIP_FOXA1_robot
chr17	38708233	38708234	MACS_peak_6264	26.00	lobChIP_FOXA1_robot
chr17	39058453	39058454	MACS_peak_6265	20.00	lobChIP_FOXA1_robot
chr17	39060045	39060046	MACS_peak_6266	7.00	lobChIP_FOXA1_robot
chr17	39071798	39071799	MACS_peak_6267	4.00	lobChIP_FOXA1_robot
chr17	39089056	39089057	MACS_peak_6268	7.00	lobChIP_FOXA1_robot
chr17	39105972	39105973	MACS_peak_6269	6.00	lobChIP_FOXA1_robot
chr17	39109094	39109095	MACS_peak_6270	3.00	lobChIP_FOXA1_robot
chr17	39113287	39113288	MACS_peak_6271	35.00	lobChIP_FOXA1_robot
chr17	39114515	39114516	MACS_peak_6272	72.00	lobChIP_FOXA1_robot
chr17	39157189	39157190	MACS_peak_6273	5.00	lobChIP_FOXA1_robot
chr17	39165678	39165679	MACS_peak_6274	21.00	lobChIP_FOXA1_robot
chr17	39205650	39205651	MACS_peak_6275	4.00	lobChIP_FOXA1_robot
chr17	39215650	39215651	MACS_peak_6276	16.00	lobChIP_FOXA1_robot
chr17	39560499	39560500	MACS_peak_6277	9.00	lobChIP_FOXA1_robot
chr17	39569475	39569476	MACS_peak_6278	10.00	lobChIP_FOXA1_robot
chr17	39572419	39572420	MACS_peak_6279	7.00	lobChIP_FOXA1_robot
chr17	39572866	39572867	MACS_peak_6280	5.00	lobChIP_FOXA1_robot
chr17	39832715	39832716	MACS_peak_6281	5.00	lobChIP_FOXA1_robot
chr17	39844203	39844204	MACS_peak_6282	4.00	lobChIP_FOXA1_robot
chr17	39933959	39933960	MACS_peak_6283	14.00	lobChIP_FOXA1_robot
chr17	40141875	40141876	MACS_peak_6284	11.00	lobChIP_FOXA1_robot
chr17	40155058	40155059	MACS_peak_6285	9.00	lobChIP_FOXA1_robot
chr17	40240119	40240120	MACS_peak_6286	23.00	lobChIP_FOXA1_robot
chr17	40421669	40421670	MACS_peak_6287	6.00	lobChIP_FOXA1_robot
chr17	40531903	40531904	MACS_peak_6288	4.00	lobChIP_FOXA1_robot
chr17	40580841	40580842	MACS_peak_6289	4.00	lobChIP_FOXA1_robot
chr17	40593675	40593676	MACS_peak_6290	4.00	lobChIP_FOXA1_robot
chr17	40680103	40680104	MACS_peak_6291	9.00	lobChIP_FOXA1_robot
chr17	40682962	40682963	MACS_peak_6292	23.00	lobChIP_FOXA1_robot
chr17	40717070	40717071	MACS_peak_6293	4.00	lobChIP_FOXA1_robot
chr17	40897005	40897006	MACS_peak_6294	7.00	lobChIP_FOXA1_robot
chr17	40942265	40942266	MACS_peak_6295	6.00	lobChIP_FOXA1_robot
chr17	40957274	40957275	MACS_peak_6296	5.00	lobChIP_FOXA1_robot
chr17	41038657	41038658	MACS_peak_6297	5.00	lobChIP_FOXA1_robot
chr17	41046323	41046324	MACS_peak_6298	3.00	lobChIP_FOXA1_robot
chr17	41071071	41071072	MACS_peak_6299	27.00	lobChIP_FOXA1_robot
chr17	41120110	41120111	MACS_peak_6300	12.00	lobChIP_FOXA1_robot
chr17	41162963	41162964	MACS_peak_6301	5.00	lobChIP_FOXA1_robot
chr17	41192948	41192949	MACS_peak_6302	7.00	lobChIP_FOXA1_robot
chr17	41221090	41221091	MACS_peak_6303	8.00	lobChIP_FOXA1_robot
chr17	41321938	41321939	MACS_peak_6304	6.00	lobChIP_FOXA1_robot
chr17	41324317	41324318	MACS_peak_6305	6.00	lobChIP_FOXA1_robot
chr17	41328580	41328581	MACS_peak_6306	4.00	lobChIP_FOXA1_robot
chr17	41369217	41369218	MACS_peak_6307	3.00	lobChIP_FOXA1_robot
chr17	41438514	41438515	MACS_peak_6308	4.00	lobChIP_FOXA1_robot
chr17	41487219	41487220	MACS_peak_6309	5.00	lobChIP_FOXA1_robot
chr17	41534403	41534404	MACS_peak_6310	10.00	lobChIP_FOXA1_robot
chr17	41543686	41543687	MACS_peak_6311	5.00	lobChIP_FOXA1_robot
chr17	41557514	41557515	MACS_peak_6312	18.00	lobChIP_FOXA1_robot
chr17	41608337	41608338	MACS_peak_6313	7.00	lobChIP_FOXA1_robot
chr17	41622077	41622078	MACS_peak_6314	36.00	lobChIP_FOXA1_robot
chr17	41651117	41651118	MACS_peak_6315	11.00	lobChIP_FOXA1_robot
chr17	41656573	41656574	MACS_peak_6316	4.00	lobChIP_FOXA1_robot
chr17	41662321	41662322	MACS_peak_6317	10.00	lobChIP_FOXA1_robot
chr17	41849555	41849556	MACS_peak_6318	3.00	lobChIP_FOXA1_robot
chr17	41850358	41850359	MACS_peak_6319	6.00	lobChIP_FOXA1_robot
chr17	41879427	41879428	MACS_peak_6320	27.00	lobChIP_FOXA1_robot
chr17	42042356	42042357	MACS_peak_6321	14.00	lobChIP_FOXA1_robot
chr17	42061339	42061340	MACS_peak_6322	17.00	lobChIP_FOXA1_robot
chr17	42186079	42186080	MACS_peak_6323	8.00	lobChIP_FOXA1_robot
chr17	42204423	42204424	MACS_peak_6324	4.00	lobChIP_FOXA1_robot
chr17	42215961	42215962	MACS_peak_6325	3.00	lobChIP_FOXA1_robot
chr17	43058820	43058821	MACS_peak_6326	4.00	lobChIP_FOXA1_robot
chr17	43128161	43128162	MACS_peak_6327	4.00	lobChIP_FOXA1_robot
chr17	43179573	43179574	MACS_peak_6328	7.00	lobChIP_FOXA1_robot
chr17	43205422	43205423	MACS_peak_6329	4.00	lobChIP_FOXA1_robot
chr17	43225266	43225267	MACS_peak_6330	14.00	lobChIP_FOXA1_robot
chr17	43248905	43248906	MACS_peak_6331	10.00	lobChIP_FOXA1_robot
chr17	43279462	43279463	MACS_peak_6332	6.00	lobChIP_FOXA1_robot
chr17	43449503	43449504	MACS_peak_6333	3.00	lobChIP_FOXA1_robot
chr17	43782946	43782947	MACS_peak_6334	9.00	lobChIP_FOXA1_robot
chr17	44065969	44065970	MACS_peak_6335	5.00	lobChIP_FOXA1_robot
chr17	44082074	44082075	MACS_peak_6336	7.00	lobChIP_FOXA1_robot
chr17	44091235	44091236	MACS_peak_6337	4.00	lobChIP_FOXA1_robot
chr17	44177365	44177366	MACS_peak_6338	6.00	lobChIP_FOXA1_robot
chr17	44268815	44268816	MACS_peak_6339	4.00	lobChIP_FOXA1_robot
chr17	44836172	44836173	MACS_peak_6340	7.00	lobChIP_FOXA1_robot
chr17	44860718	44860719	MACS_peak_6341	5.00	lobChIP_FOXA1_robot
chr17	44971542	44971543	MACS_peak_6342	3.00	lobChIP_FOXA1_robot
chr17	45368981	45368982	MACS_peak_6343	9.00	lobChIP_FOXA1_robot
chr17	45403851	45403852	MACS_peak_6344	4.00	lobChIP_FOXA1_robot
chr17	45407497	45407498	MACS_peak_6345	4.00	lobChIP_FOXA1_robot
chr17	45698062	45698063	MACS_peak_6346	38.00	lobChIP_FOXA1_robot
chr17	45764490	45764491	MACS_peak_6347	7.00	lobChIP_FOXA1_robot
chr17	46085966	46085967	MACS_peak_6348	9.00	lobChIP_FOXA1_robot
chr17	46125661	46125662	MACS_peak_6349	4.00	lobChIP_FOXA1_robot
chr17	46873151	46873152	MACS_peak_6350	5.00	lobChIP_FOXA1_robot
chr17	46949192	46949193	MACS_peak_6351	20.00	lobChIP_FOXA1_robot
chr17	47049784	47049785	MACS_peak_6352	5.00	lobChIP_FOXA1_robot
chr17	47082640	47082641	MACS_peak_6353	8.00	lobChIP_FOXA1_robot
chr17	47146438	47146439	MACS_peak_6354	7.00	lobChIP_FOXA1_robot
chr17	47184277	47184278	MACS_peak_6355	28.00	lobChIP_FOXA1_robot
chr17	47438065	47438066	MACS_peak_6356	4.00	lobChIP_FOXA1_robot
chr17	47485645	47485646	MACS_peak_6357	5.00	lobChIP_FOXA1_robot
chr17	47674444	47674445	MACS_peak_6358	10.00	lobChIP_FOXA1_robot
chr17	47707404	47707405	MACS_peak_6359	3.00	lobChIP_FOXA1_robot
chr17	47812473	47812474	MACS_peak_6360	6.00	lobChIP_FOXA1_robot
chr17	48163429	48163430	MACS_peak_6361	11.00	lobChIP_FOXA1_robot
chr17	48296275	48296276	MACS_peak_6362	5.00	lobChIP_FOXA1_robot
chr17	48500293	48500294	MACS_peak_6363	7.00	lobChIP_FOXA1_robot
chr17	48501168	48501169	MACS_peak_6364	17.00	lobChIP_FOXA1_robot
chr17	48517828	48517829	MACS_peak_6365	6.00	lobChIP_FOXA1_robot
chr17	48536575	48536576	MACS_peak_6366	6.00	lobChIP_FOXA1_robot
chr17	48620431	48620432	MACS_peak_6367	15.00	lobChIP_FOXA1_robot
chr17	48713653	48713654	MACS_peak_6368	7.00	lobChIP_FOXA1_robot
chr17	48737753	48737754	MACS_peak_6369	9.00	lobChIP_FOXA1_robot
chr17	48748683	48748684	MACS_peak_6370	7.00	lobChIP_FOXA1_robot
chr17	48762250	48762251	MACS_peak_6371	9.00	lobChIP_FOXA1_robot
chr17	48764126	48764127	MACS_peak_6372	7.00	lobChIP_FOXA1_robot
chr17	48770633	48770634	MACS_peak_6373	21.00	lobChIP_FOXA1_robot
chr17	48771610	48771611	MACS_peak_6374	4.00	lobChIP_FOXA1_robot
chr17	48772715	48772716	MACS_peak_6375	5.00	lobChIP_FOXA1_robot
chr17	48786331	48786332	MACS_peak_6376	4.00	lobChIP_FOXA1_robot
chr17	48883911	48883912	MACS_peak_6377	5.00	lobChIP_FOXA1_robot
chr17	48933597	48933598	MACS_peak_6378	8.00	lobChIP_FOXA1_robot
chr17	48942415	48942416	MACS_peak_6379	5.00	lobChIP_FOXA1_robot
chr17	48968234	48968235	MACS_peak_6380	36.00	lobChIP_FOXA1_robot
chr17	48983015	48983016	MACS_peak_6381	4.00	lobChIP_FOXA1_robot
chr17	49022070	49022071	MACS_peak_6382	7.00	lobChIP_FOXA1_robot
chr17	49195916	49195917	MACS_peak_6383	5.00	lobChIP_FOXA1_robot
chr17	49203607	49203608	MACS_peak_6384	4.00	lobChIP_FOXA1_robot
chr17	49223782	49223783	MACS_peak_6385	4.00	lobChIP_FOXA1_robot
chr17	49230865	49230866	MACS_peak_6386	4.00	lobChIP_FOXA1_robot
chr17	49380786	49380787	MACS_peak_6387	3.00	lobChIP_FOXA1_robot
chr17	49399906	49399907	MACS_peak_6388	4.00	lobChIP_FOXA1_robot
chr17	49423767	49423768	MACS_peak_6389	5.00	lobChIP_FOXA1_robot
chr17	49513286	49513287	MACS_peak_6390	4.00	lobChIP_FOXA1_robot
chr17	49685789	49685790	MACS_peak_6391	5.00	lobChIP_FOXA1_robot
chr17	49819063	49819064	MACS_peak_6392	5.00	lobChIP_FOXA1_robot
chr17	50075314	50075315	MACS_peak_6393	6.00	lobChIP_FOXA1_robot
chr17	50496762	50496763	MACS_peak_6394	7.00	lobChIP_FOXA1_robot
chr17	52002038	52002039	MACS_peak_6395	11.00	lobChIP_FOXA1_robot
chr17	52563392	52563393	MACS_peak_6396	5.00	lobChIP_FOXA1_robot
chr17	52896445	52896446	MACS_peak_6397	5.00	lobChIP_FOXA1_robot
chr17	53082917	53082918	MACS_peak_6398	3.00	lobChIP_FOXA1_robot
chr17	53194167	53194168	MACS_peak_6399	3.00	lobChIP_FOXA1_robot
chr17	53361681	53361682	MACS_peak_6400	9.00	lobChIP_FOXA1_robot
chr17	53363545	53363546	MACS_peak_6401	32.00	lobChIP_FOXA1_robot
chr17	53364584	53364585	MACS_peak_6402	45.00	lobChIP_FOXA1_robot
chr17	53379648	53379649	MACS_peak_6403	12.00	lobChIP_FOXA1_robot
chr17	53383830	53383831	MACS_peak_6404	19.00	lobChIP_FOXA1_robot
chr17	53386082	53386083	MACS_peak_6405	4.00	lobChIP_FOXA1_robot
chr17	53472032	53472033	MACS_peak_6406	17.00	lobChIP_FOXA1_robot
chr17	53485623	53485624	MACS_peak_6407	15.00	lobChIP_FOXA1_robot
chr17	53496049	53496050	MACS_peak_6408	3.00	lobChIP_FOXA1_robot
chr17	53510742	53510743	MACS_peak_6409	19.00	lobChIP_FOXA1_robot
chr17	53522420	53522421	MACS_peak_6410	5.00	lobChIP_FOXA1_robot
chr17	53524297	53524298	MACS_peak_6411	39.00	lobChIP_FOXA1_robot
chr17	53532405	53532406	MACS_peak_6412	12.00	lobChIP_FOXA1_robot
chr17	53618034	53618035	MACS_peak_6413	4.00	lobChIP_FOXA1_robot
chr17	53654255	53654256	MACS_peak_6414	4.00	lobChIP_FOXA1_robot
chr17	53840134	53840135	MACS_peak_6415	4.00	lobChIP_FOXA1_robot
chr17	54144850	54144851	MACS_peak_6416	4.00	lobChIP_FOXA1_robot
chr17	54466837	54466838	MACS_peak_6417	3.00	lobChIP_FOXA1_robot
chr17	54849963	54849964	MACS_peak_6418	9.00	lobChIP_FOXA1_robot
chr17	54964017	54964018	MACS_peak_6419	7.00	lobChIP_FOXA1_robot
chr17	55083918	55083919	MACS_peak_6420	4.00	lobChIP_FOXA1_robot
chr17	55163240	55163241	MACS_peak_6421	21.00	lobChIP_FOXA1_robot
chr17	55163941	55163942	MACS_peak_6422	5.00	lobChIP_FOXA1_robot
chr17	55198095	55198096	MACS_peak_6423	4.00	lobChIP_FOXA1_robot
chr17	55298879	55298880	MACS_peak_6424	9.00	lobChIP_FOXA1_robot
chr17	55335611	55335612	MACS_peak_6425	6.00	lobChIP_FOXA1_robot
chr17	55362735	55362736	MACS_peak_6426	5.00	lobChIP_FOXA1_robot
chr17	55422450	55422451	MACS_peak_6427	10.00	lobChIP_FOXA1_robot
chr17	55444319	55444320	MACS_peak_6428	9.00	lobChIP_FOXA1_robot
chr17	55447637	55447638	MACS_peak_6429	4.00	lobChIP_FOXA1_robot
chr17	55449605	55449606	MACS_peak_6430	31.00	lobChIP_FOXA1_robot
chr17	55450680	55450681	MACS_peak_6431	5.00	lobChIP_FOXA1_robot
chr17	55484806	55484807	MACS_peak_6432	14.00	lobChIP_FOXA1_robot
chr17	55485845	55485846	MACS_peak_6433	5.00	lobChIP_FOXA1_robot
chr17	55487708	55487709	MACS_peak_6434	6.00	lobChIP_FOXA1_robot
chr17	55491611	55491612	MACS_peak_6435	22.00	lobChIP_FOXA1_robot
chr17	55522530	55522531	MACS_peak_6436	8.00	lobChIP_FOXA1_robot
chr17	55536916	55536917	MACS_peak_6437	8.00	lobChIP_FOXA1_robot
chr17	55546760	55546761	MACS_peak_6438	4.00	lobChIP_FOXA1_robot
chr17	55593815	55593816	MACS_peak_6439	6.00	lobChIP_FOXA1_robot
chr17	55595465	55595466	MACS_peak_6440	8.00	lobChIP_FOXA1_robot
chr17	55638934	55638935	MACS_peak_6441	28.00	lobChIP_FOXA1_robot
chr17	55655346	55655347	MACS_peak_6442	6.00	lobChIP_FOXA1_robot
chr17	55686863	55686864	MACS_peak_6443	4.00	lobChIP_FOXA1_robot
chr17	55911071	55911072	MACS_peak_6444	6.00	lobChIP_FOXA1_robot
chr17	55927524	55927525	MACS_peak_6445	3.00	lobChIP_FOXA1_robot
chr17	55928768	55928769	MACS_peak_6446	9.00	lobChIP_FOXA1_robot
chr17	55929284	55929285	MACS_peak_6447	13.00	lobChIP_FOXA1_robot
chr17	55938644	55938645	MACS_peak_6448	7.00	lobChIP_FOXA1_robot
chr17	55956604	55956605	MACS_peak_6449	8.00	lobChIP_FOXA1_robot
chr17	56015660	56015661	MACS_peak_6450	5.00	lobChIP_FOXA1_robot
chr17	56024624	56024625	MACS_peak_6451	6.00	lobChIP_FOXA1_robot
chr17	56038374	56038375	MACS_peak_6452	6.00	lobChIP_FOXA1_robot
chr17	56052675	56052676	MACS_peak_6453	33.00	lobChIP_FOXA1_robot
chr17	56426353	56426354	MACS_peak_6454	4.00	lobChIP_FOXA1_robot
chr17	56429580	56429581	MACS_peak_6455	3.00	lobChIP_FOXA1_robot
chr17	56458712	56458713	MACS_peak_6456	4.00	lobChIP_FOXA1_robot
chr17	56460414	56460415	MACS_peak_6457	5.00	lobChIP_FOXA1_robot
chr17	56461485	56461486	MACS_peak_6458	5.00	lobChIP_FOXA1_robot
chr17	56468995	56468996	MACS_peak_6459	40.00	lobChIP_FOXA1_robot
chr17	56472150	56472151	MACS_peak_6460	7.00	lobChIP_FOXA1_robot
chr17	56490485	56490486	MACS_peak_6461	6.00	lobChIP_FOXA1_robot
chr17	56493542	56493543	MACS_peak_6462	3.00	lobChIP_FOXA1_robot
chr17	56613081	56613082	MACS_peak_6463	4.00	lobChIP_FOXA1_robot
chr17	56708986	56708987	MACS_peak_6464	8.00	lobChIP_FOXA1_robot
chr17	56736660	56736661	MACS_peak_6465	7.00	lobChIP_FOXA1_robot
chr17	56906898	56906899	MACS_peak_6466	7.00	lobChIP_FOXA1_robot
chr17	57035096	57035097	MACS_peak_6467	5.00	lobChIP_FOXA1_robot
chr17	57108448	57108449	MACS_peak_6468	5.00	lobChIP_FOXA1_robot
chr17	57232508	57232509	MACS_peak_6469	3.00	lobChIP_FOXA1_robot
chr17	57233652	57233653	MACS_peak_6470	5.00	lobChIP_FOXA1_robot
chr17	57374798	57374799	MACS_peak_6471	5.00	lobChIP_FOXA1_robot
chr17	57444343	57444344	MACS_peak_6472	22.00	lobChIP_FOXA1_robot
chr17	57446007	57446008	MACS_peak_6473	4.00	lobChIP_FOXA1_robot
chr17	57470466	57470467	MACS_peak_6474	4.00	lobChIP_FOXA1_robot
chr17	57542706	57542707	MACS_peak_6475	31.00	lobChIP_FOXA1_robot
chr17	57547395	57547396	MACS_peak_6476	13.00	lobChIP_FOXA1_robot
chr17	57591889	57591890	MACS_peak_6477	5.00	lobChIP_FOXA1_robot
chr17	57803300	57803301	MACS_peak_6478	12.00	lobChIP_FOXA1_robot
chr17	57832059	57832060	MACS_peak_6479	7.00	lobChIP_FOXA1_robot
chr17	57833656	57833657	MACS_peak_6480	13.00	lobChIP_FOXA1_robot
chr17	57862086	57862087	MACS_peak_6481	3.00	lobChIP_FOXA1_robot
chr17	57862571	57862572	MACS_peak_6482	6.00	lobChIP_FOXA1_robot
chr17	57864092	57864093	MACS_peak_6483	11.00	lobChIP_FOXA1_robot
chr17	57869736	57869737	MACS_peak_6484	5.00	lobChIP_FOXA1_robot
chr17	57880981	57880982	MACS_peak_6485	10.00	lobChIP_FOXA1_robot
chr17	57906916	57906917	MACS_peak_6486	4.00	lobChIP_FOXA1_robot
chr17	57910538	57910539	MACS_peak_6487	6.00	lobChIP_FOXA1_robot
chr17	57915139	57915140	MACS_peak_6488	4.00	lobChIP_FOXA1_robot
chr17	57920821	57920822	MACS_peak_6489	5.00	lobChIP_FOXA1_robot
chr17	57922854	57922855	MACS_peak_6490	9.00	lobChIP_FOXA1_robot
chr17	57923885	57923886	MACS_peak_6491	21.00	lobChIP_FOXA1_robot
chr17	57924948	57924949	MACS_peak_6492	3.00	lobChIP_FOXA1_robot
chr17	57925982	57925983	MACS_peak_6493	5.00	lobChIP_FOXA1_robot
chr17	57930659	57930660	MACS_peak_6494	13.00	lobChIP_FOXA1_robot
chr17	57933181	57933182	MACS_peak_6495	27.00	lobChIP_FOXA1_robot
chr17	57971812	57971813	MACS_peak_6496	6.00	lobChIP_FOXA1_robot
chr17	57991759	57991760	MACS_peak_6497	7.00	lobChIP_FOXA1_robot
chr17	58116062	58116063	MACS_peak_6498	4.00	lobChIP_FOXA1_robot
chr17	58303631	58303632	MACS_peak_6499	3.00	lobChIP_FOXA1_robot
chr17	58433591	58433592	MACS_peak_6500	9.00	lobChIP_FOXA1_robot
chr17	58522531	58522532	MACS_peak_6501	4.00	lobChIP_FOXA1_robot
chr17	58664025	58664026	MACS_peak_6502	3.00	lobChIP_FOXA1_robot
chr17	58807458	58807459	MACS_peak_6503	3.00	lobChIP_FOXA1_robot
chr17	58823028	58823029	MACS_peak_6504	4.00	lobChIP_FOXA1_robot
chr17	58997703	58997704	MACS_peak_6505	7.00	lobChIP_FOXA1_robot
chr17	59227528	59227529	MACS_peak_6506	5.00	lobChIP_FOXA1_robot
chr17	59413579	59413580	MACS_peak_6507	81.00	lobChIP_FOXA1_robot
chr17	59415574	59415575	MACS_peak_6508	3.00	lobChIP_FOXA1_robot
chr17	59434199	59434200	MACS_peak_6509	25.00	lobChIP_FOXA1_robot
chr17	59441078	59441079	MACS_peak_6510	19.00	lobChIP_FOXA1_robot
chr17	59442943	59442944	MACS_peak_6511	5.00	lobChIP_FOXA1_robot
chr17	59444389	59444390	MACS_peak_6512	9.00	lobChIP_FOXA1_robot
chr17	59504371	59504372	MACS_peak_6513	4.00	lobChIP_FOXA1_robot
chr17	59532542	59532543	MACS_peak_6514	16.00	lobChIP_FOXA1_robot
chr17	59538421	59538422	MACS_peak_6515	15.00	lobChIP_FOXA1_robot
chr17	59566892	59566893	MACS_peak_6516	6.00	lobChIP_FOXA1_robot
chr17	59574666	59574667	MACS_peak_6517	4.00	lobChIP_FOXA1_robot
chr17	59646136	59646137	MACS_peak_6518	31.00	lobChIP_FOXA1_robot
chr17	59800351	59800352	MACS_peak_6519	4.00	lobChIP_FOXA1_robot
chr17	60167761	60167762	MACS_peak_6520	4.00	lobChIP_FOXA1_robot
chr17	60238302	60238303	MACS_peak_6521	3.00	lobChIP_FOXA1_robot
chr17	60241410	60241411	MACS_peak_6522	9.00	lobChIP_FOXA1_robot
chr17	60242907	60242908	MACS_peak_6523	8.00	lobChIP_FOXA1_robot
chr17	60282585	60282586	MACS_peak_6524	5.00	lobChIP_FOXA1_robot
chr17	60384853	60384854	MACS_peak_6525	5.00	lobChIP_FOXA1_robot
chr17	60615873	60615874	MACS_peak_6526	4.00	lobChIP_FOXA1_robot
chr17	61598064	61598065	MACS_peak_6527	10.00	lobChIP_FOXA1_robot
chr17	61655850	61655851	MACS_peak_6528	8.00	lobChIP_FOXA1_robot
chr17	61839816	61839817	MACS_peak_6529	4.00	lobChIP_FOXA1_robot
chr17	62152295	62152296	MACS_peak_6530	9.00	lobChIP_FOXA1_robot
chr17	62159615	62159616	MACS_peak_6531	13.00	lobChIP_FOXA1_robot
chr17	62162063	62162064	MACS_peak_6532	10.00	lobChIP_FOXA1_robot
chr17	62182973	62182974	MACS_peak_6533	5.00	lobChIP_FOXA1_robot
chr17	62206200	62206201	MACS_peak_6534	4.00	lobChIP_FOXA1_robot
chr17	62211928	62211929	MACS_peak_6535	5.00	lobChIP_FOXA1_robot
chr17	62227873	62227874	MACS_peak_6536	10.00	lobChIP_FOXA1_robot
chr17	62255675	62255676	MACS_peak_6537	8.00	lobChIP_FOXA1_robot
chr17	62294470	62294471	MACS_peak_6538	7.00	lobChIP_FOXA1_robot
chr17	62396248	62396249	MACS_peak_6539	5.00	lobChIP_FOXA1_robot
chr17	62804815	62804816	MACS_peak_6540	6.00	lobChIP_FOXA1_robot
chr17	62923796	62923797	MACS_peak_6541	6.00	lobChIP_FOXA1_robot
chr17	62938036	62938037	MACS_peak_6542	3.00	lobChIP_FOXA1_robot
chr17	62955382	62955383	MACS_peak_6543	7.00	lobChIP_FOXA1_robot
chr17	63019149	63019150	MACS_peak_6544	4.00	lobChIP_FOXA1_robot
chr17	63071034	63071035	MACS_peak_6545	7.00	lobChIP_FOXA1_robot
chr17	63512257	63512258	MACS_peak_6546	4.00	lobChIP_FOXA1_robot
chr17	63538881	63538882	MACS_peak_6547	4.00	lobChIP_FOXA1_robot
chr17	63553736	63553737	MACS_peak_6548	18.00	lobChIP_FOXA1_robot
chr17	63554954	63554955	MACS_peak_6549	7.00	lobChIP_FOXA1_robot
chr17	63558282	63558283	MACS_peak_6550	4.00	lobChIP_FOXA1_robot
chr17	63581492	63581493	MACS_peak_6551	5.00	lobChIP_FOXA1_robot
chr17	63584701	63584702	MACS_peak_6552	6.00	lobChIP_FOXA1_robot
chr17	63624371	63624372	MACS_peak_6553	4.00	lobChIP_FOXA1_robot
chr17	63630275	63630276	MACS_peak_6554	13.00	lobChIP_FOXA1_robot
chr17	63652972	63652973	MACS_peak_6555	4.00	lobChIP_FOXA1_robot
chr17	63654008	63654009	MACS_peak_6556	4.00	lobChIP_FOXA1_robot
chr17	63667211	63667212	MACS_peak_6557	7.00	lobChIP_FOXA1_robot
chr17	63687002	63687003	MACS_peak_6558	6.00	lobChIP_FOXA1_robot
chr17	63695336	63695337	MACS_peak_6559	5.00	lobChIP_FOXA1_robot
chr17	63719399	63719400	MACS_peak_6560	3.00	lobChIP_FOXA1_robot
chr17	63719877	63719878	MACS_peak_6561	5.00	lobChIP_FOXA1_robot
chr17	63747010	63747011	MACS_peak_6562	5.00	lobChIP_FOXA1_robot
chr17	63781432	63781433	MACS_peak_6563	15.00	lobChIP_FOXA1_robot
chr17	63788862	63788863	MACS_peak_6564	3.00	lobChIP_FOXA1_robot
chr17	64197094	64197095	MACS_peak_6565	7.00	lobChIP_FOXA1_robot
chr17	64225643	64225644	MACS_peak_6566	10.00	lobChIP_FOXA1_robot
chr17	64226652	64226653	MACS_peak_6567	6.00	lobChIP_FOXA1_robot
chr17	64231682	64231683	MACS_peak_6568	38.00	lobChIP_FOXA1_robot
chr17	64337853	64337854	MACS_peak_6569	6.00	lobChIP_FOXA1_robot
chr17	64364389	64364390	MACS_peak_6570	5.00	lobChIP_FOXA1_robot
chr17	64411780	64411781	MACS_peak_6571	5.00	lobChIP_FOXA1_robot
chr17	64447701	64447702	MACS_peak_6572	6.00	lobChIP_FOXA1_robot
chr17	64456069	64456070	MACS_peak_6573	4.00	lobChIP_FOXA1_robot
chr17	64459196	64459197	MACS_peak_6574	3.00	lobChIP_FOXA1_robot
chr17	64543803	64543804	MACS_peak_6575	3.00	lobChIP_FOXA1_robot
chr17	64708174	64708175	MACS_peak_6576	6.00	lobChIP_FOXA1_robot
chr17	64773594	64773595	MACS_peak_6577	10.00	lobChIP_FOXA1_robot
chr17	64920235	64920236	MACS_peak_6578	5.00	lobChIP_FOXA1_robot
chr17	64954519	64954520	MACS_peak_6579	7.00	lobChIP_FOXA1_robot
chr17	65072571	65072572	MACS_peak_6580	4.00	lobChIP_FOXA1_robot
chr17	65235126	65235127	MACS_peak_6581	6.00	lobChIP_FOXA1_robot
chr17	65241960	65241961	MACS_peak_6582	6.00	lobChIP_FOXA1_robot
chr17	65243336	65243337	MACS_peak_6583	11.00	lobChIP_FOXA1_robot
chr17	65387019	65387020	MACS_peak_6584	6.00	lobChIP_FOXA1_robot
chr17	65635960	65635961	MACS_peak_6585	5.00	lobChIP_FOXA1_robot
chr17	65721676	65721677	MACS_peak_6586	4.00	lobChIP_FOXA1_robot
chr17	65725024	65725025	MACS_peak_6587	4.00	lobChIP_FOXA1_robot
chr17	65959619	65959620	MACS_peak_6588	3.00	lobChIP_FOXA1_robot
chr17	65990823	65990824	MACS_peak_6589	4.00	lobChIP_FOXA1_robot
chr17	66030300	66030301	MACS_peak_6590	4.00	lobChIP_FOXA1_robot
chr17	66262208	66262209	MACS_peak_6591	12.00	lobChIP_FOXA1_robot
chr17	66374982	66374983	MACS_peak_6592	14.00	lobChIP_FOXA1_robot
chr17	66376083	66376084	MACS_peak_6593	5.00	lobChIP_FOXA1_robot
chr17	66380228	66380229	MACS_peak_6594	9.00	lobChIP_FOXA1_robot
chr17	66433909	66433910	MACS_peak_6595	3.00	lobChIP_FOXA1_robot
chr17	66448341	66448342	MACS_peak_6596	7.00	lobChIP_FOXA1_robot
chr17	66470643	66470644	MACS_peak_6597	5.00	lobChIP_FOXA1_robot
chr17	66478880	66478881	MACS_peak_6598	9.00	lobChIP_FOXA1_robot
chr17	66481503	66481504	MACS_peak_6599	5.00	lobChIP_FOXA1_robot
chr17	66755361	66755362	MACS_peak_6600	8.00	lobChIP_FOXA1_robot
chr17	66774309	66774310	MACS_peak_6601	5.00	lobChIP_FOXA1_robot
chr17	66785524	66785525	MACS_peak_6602	19.00	lobChIP_FOXA1_robot
chr17	66790209	66790210	MACS_peak_6603	57.00	lobChIP_FOXA1_robot
chr17	66816992	66816993	MACS_peak_6604	43.00	lobChIP_FOXA1_robot
chr17	66818218	66818219	MACS_peak_6605	7.00	lobChIP_FOXA1_robot
chr17	67043839	67043840	MACS_peak_6606	13.00	lobChIP_FOXA1_robot
chr17	67069027	67069028	MACS_peak_6607	15.00	lobChIP_FOXA1_robot
chr17	67118319	67118320	MACS_peak_6608	4.00	lobChIP_FOXA1_robot
chr17	67172048	67172049	MACS_peak_6609	6.00	lobChIP_FOXA1_robot
chr17	67216441	67216442	MACS_peak_6610	3.00	lobChIP_FOXA1_robot
chr17	67265038	67265039	MACS_peak_6611	6.00	lobChIP_FOXA1_robot
chr17	67310645	67310646	MACS_peak_6612	8.00	lobChIP_FOXA1_robot
chr17	67323278	67323279	MACS_peak_6613	5.00	lobChIP_FOXA1_robot
chr17	67331403	67331404	MACS_peak_6614	3.00	lobChIP_FOXA1_robot
chr17	67387173	67387174	MACS_peak_6615	6.00	lobChIP_FOXA1_robot
chr17	67564178	67564179	MACS_peak_6616	4.00	lobChIP_FOXA1_robot
chr17	69678116	69678117	MACS_peak_6617	23.00	lobChIP_FOXA1_robot
chr17	69780846	69780847	MACS_peak_6618	5.00	lobChIP_FOXA1_robot
chr17	70089148	70089149	MACS_peak_6619	11.00	lobChIP_FOXA1_robot
chr17	70119476	70119477	MACS_peak_6620	6.00	lobChIP_FOXA1_robot
chr17	70122794	70122795	MACS_peak_6621	4.00	lobChIP_FOXA1_robot
chr17	70141060	70141061	MACS_peak_6622	3.00	lobChIP_FOXA1_robot
chr17	70147901	70147902	MACS_peak_6623	5.00	lobChIP_FOXA1_robot
chr17	70169836	70169837	MACS_peak_6624	5.00	lobChIP_FOXA1_robot
chr17	70335462	70335463	MACS_peak_6625	6.00	lobChIP_FOXA1_robot
chr17	70383101	70383102	MACS_peak_6626	8.00	lobChIP_FOXA1_robot
chr17	70393437	70393438	MACS_peak_6627	9.00	lobChIP_FOXA1_robot
chr17	70395981	70395982	MACS_peak_6628	57.00	lobChIP_FOXA1_robot
chr17	70400087	70400088	MACS_peak_6629	16.00	lobChIP_FOXA1_robot
chr17	70409456	70409457	MACS_peak_6630	36.00	lobChIP_FOXA1_robot
chr17	70443673	70443674	MACS_peak_6631	8.00	lobChIP_FOXA1_robot
chr17	70444375	70444376	MACS_peak_6632	4.00	lobChIP_FOXA1_robot
chr17	70498188	70498189	MACS_peak_6633	14.00	lobChIP_FOXA1_robot
chr17	70515172	70515173	MACS_peak_6634	28.00	lobChIP_FOXA1_robot
chr17	70535640	70535641	MACS_peak_6635	3.00	lobChIP_FOXA1_robot
chr17	70576858	70576859	MACS_peak_6636	6.00	lobChIP_FOXA1_robot
chr17	70612700	70612701	MACS_peak_6637	4.00	lobChIP_FOXA1_robot
chr17	70628589	70628590	MACS_peak_6638	7.00	lobChIP_FOXA1_robot
chr17	70657086	70657087	MACS_peak_6639	6.00	lobChIP_FOXA1_robot
chr17	70679302	70679303	MACS_peak_6640	3.00	lobChIP_FOXA1_robot
chr17	70680227	70680228	MACS_peak_6641	4.00	lobChIP_FOXA1_robot
chr17	70694264	70694265	MACS_peak_6642	5.00	lobChIP_FOXA1_robot
chr17	70698674	70698675	MACS_peak_6643	5.00	lobChIP_FOXA1_robot
chr17	70712487	70712488	MACS_peak_6644	10.00	lobChIP_FOXA1_robot
chr17	70924934	70924935	MACS_peak_6645	4.00	lobChIP_FOXA1_robot
chr17	70931269	70931270	MACS_peak_6646	5.00	lobChIP_FOXA1_robot
chr17	70968622	70968623	MACS_peak_6647	28.00	lobChIP_FOXA1_robot
chr17	71006817	71006818	MACS_peak_6648	5.00	lobChIP_FOXA1_robot
chr17	71086836	71086837	MACS_peak_6649	10.00	lobChIP_FOXA1_robot
chr17	71148327	71148328	MACS_peak_6650	9.00	lobChIP_FOXA1_robot
chr17	71183618	71183619	MACS_peak_6651	81.00	lobChIP_FOXA1_robot
chr17	71197507	71197508	MACS_peak_6652	8.00	lobChIP_FOXA1_robot
chr17	71258155	71258156	MACS_peak_6653	5.00	lobChIP_FOXA1_robot
chr17	71269151	71269152	MACS_peak_6654	8.00	lobChIP_FOXA1_robot
chr17	71285364	71285365	MACS_peak_6655	6.00	lobChIP_FOXA1_robot
chr17	71292702	71292703	MACS_peak_6656	5.00	lobChIP_FOXA1_robot
chr17	71309908	71309909	MACS_peak_6657	6.00	lobChIP_FOXA1_robot
chr17	71317171	71317172	MACS_peak_6658	5.00	lobChIP_FOXA1_robot
chr17	71423539	71423540	MACS_peak_6659	5.00	lobChIP_FOXA1_robot
chr17	71792068	71792069	MACS_peak_6660	6.00	lobChIP_FOXA1_robot
chr17	71794415	71794416	MACS_peak_6661	25.00	lobChIP_FOXA1_robot
chr17	72414999	72415000	MACS_peak_6662	5.00	lobChIP_FOXA1_robot
chr17	72426850	72426851	MACS_peak_6663	6.00	lobChIP_FOXA1_robot
chr17	72439231	72439232	MACS_peak_6664	4.00	lobChIP_FOXA1_robot
chr17	72711447	72711448	MACS_peak_6665	8.00	lobChIP_FOXA1_robot
chr17	72732773	72732774	MACS_peak_6666	5.00	lobChIP_FOXA1_robot
chr17	72734686	72734687	MACS_peak_6667	12.00	lobChIP_FOXA1_robot
chr17	72746831	72746832	MACS_peak_6668	6.00	lobChIP_FOXA1_robot
chr17	72759171	72759172	MACS_peak_6669	8.00	lobChIP_FOXA1_robot
chr17	72968293	72968294	MACS_peak_6670	4.00	lobChIP_FOXA1_robot
chr17	73009677	73009678	MACS_peak_6671	4.00	lobChIP_FOXA1_robot
chr17	73068238	73068239	MACS_peak_6672	5.00	lobChIP_FOXA1_robot
chr17	73080380	73080381	MACS_peak_6673	17.00	lobChIP_FOXA1_robot
chr17	73155469	73155470	MACS_peak_6674	4.00	lobChIP_FOXA1_robot
chr17	73196186	73196187	MACS_peak_6675	11.00	lobChIP_FOXA1_robot
chr17	73263998	73263999	MACS_peak_6676	3.00	lobChIP_FOXA1_robot
chr17	73290564	73290565	MACS_peak_6677	8.00	lobChIP_FOXA1_robot
chr17	73291416	73291417	MACS_peak_6678	6.00	lobChIP_FOXA1_robot
chr17	73309866	73309867	MACS_peak_6679	4.00	lobChIP_FOXA1_robot
chr17	73346381	73346382	MACS_peak_6680	4.00	lobChIP_FOXA1_robot
chr17	73369056	73369057	MACS_peak_6681	3.00	lobChIP_FOXA1_robot
chr17	73379265	73379266	MACS_peak_6682	45.00	lobChIP_FOXA1_robot
chr17	73455895	73455896	MACS_peak_6683	3.00	lobChIP_FOXA1_robot
chr17	73508007	73508008	MACS_peak_6684	10.00	lobChIP_FOXA1_robot
chr17	73521750	73521751	MACS_peak_6685	10.00	lobChIP_FOXA1_robot
chr17	73553690	73553691	MACS_peak_6686	9.00	lobChIP_FOXA1_robot
chr17	73843350	73843351	MACS_peak_6687	4.00	lobChIP_FOXA1_robot
chr17	73865491	73865492	MACS_peak_6688	8.00	lobChIP_FOXA1_robot
chr17	73956000	73956001	MACS_peak_6689	5.00	lobChIP_FOXA1_robot
chr17	73972592	73972593	MACS_peak_6690	8.00	lobChIP_FOXA1_robot
chr17	73999658	73999659	MACS_peak_6691	10.00	lobChIP_FOXA1_robot
chr17	74102302	74102303	MACS_peak_6692	8.00	lobChIP_FOXA1_robot
chr17	74134660	74134661	MACS_peak_6693	4.00	lobChIP_FOXA1_robot
chr17	74170309	74170310	MACS_peak_6694	7.00	lobChIP_FOXA1_robot
chr17	74234147	74234148	MACS_peak_6695	6.00	lobChIP_FOXA1_robot
chr17	74248349	74248350	MACS_peak_6696	3.00	lobChIP_FOXA1_robot
chr17	74261099	74261100	MACS_peak_6697	7.00	lobChIP_FOXA1_robot
chr17	74348003	74348004	MACS_peak_6698	10.00	lobChIP_FOXA1_robot
chr17	74353360	74353361	MACS_peak_6699	11.00	lobChIP_FOXA1_robot
chr17	74363867	74363868	MACS_peak_6700	9.00	lobChIP_FOXA1_robot
chr17	74378615	74378616	MACS_peak_6701	3.00	lobChIP_FOXA1_robot
chr17	74479795	74479796	MACS_peak_6702	4.00	lobChIP_FOXA1_robot
chr17	74495503	74495504	MACS_peak_6703	11.00	lobChIP_FOXA1_robot
chr17	74529277	74529278	MACS_peak_6704	15.00	lobChIP_FOXA1_robot
chr17	74547322	74547323	MACS_peak_6705	4.00	lobChIP_FOXA1_robot
chr17	74637874	74637875	MACS_peak_6706	7.00	lobChIP_FOXA1_robot
chr17	74758899	74758900	MACS_peak_6707	55.00	lobChIP_FOXA1_robot
chr17	74911207	74911208	MACS_peak_6708	39.00	lobChIP_FOXA1_robot
chr17	74998606	74998607	MACS_peak_6709	5.00	lobChIP_FOXA1_robot
chr17	75098061	75098062	MACS_peak_6710	25.00	lobChIP_FOXA1_robot
chr17	75112867	75112868	MACS_peak_6711	4.00	lobChIP_FOXA1_robot
chr17	75118082	75118083	MACS_peak_6712	27.00	lobChIP_FOXA1_robot
chr17	75156642	75156643	MACS_peak_6713	13.00	lobChIP_FOXA1_robot
chr17	75279336	75279337	MACS_peak_6714	5.00	lobChIP_FOXA1_robot
chr17	75282791	75282792	MACS_peak_6715	26.00	lobChIP_FOXA1_robot
chr17	75296590	75296591	MACS_peak_6716	5.00	lobChIP_FOXA1_robot
chr17	75426467	75426468	MACS_peak_6717	8.00	lobChIP_FOXA1_robot
chr17	75508180	75508181	MACS_peak_6718	18.00	lobChIP_FOXA1_robot
chr17	75821666	75821667	MACS_peak_6719	4.00	lobChIP_FOXA1_robot
chr17	75985845	75985846	MACS_peak_6720	4.00	lobChIP_FOXA1_robot
chr17	76125299	76125300	MACS_peak_6721	4.00	lobChIP_FOXA1_robot
chr17	76184150	76184151	MACS_peak_6722	15.00	lobChIP_FOXA1_robot
chr17	76371992	76371993	MACS_peak_6723	3.00	lobChIP_FOXA1_robot
chr17	76392957	76392958	MACS_peak_6724	4.00	lobChIP_FOXA1_robot
chr17	76395911	76395912	MACS_peak_6725	10.00	lobChIP_FOXA1_robot
chr17	76417781	76417782	MACS_peak_6726	63.00	lobChIP_FOXA1_robot
chr17	76532088	76532089	MACS_peak_6727	3.00	lobChIP_FOXA1_robot
chr17	76646527	76646528	MACS_peak_6728	23.00	lobChIP_FOXA1_robot
chr17	76662189	76662190	MACS_peak_6729	6.00	lobChIP_FOXA1_robot
chr17	76743011	76743012	MACS_peak_6730	4.00	lobChIP_FOXA1_robot
chr17	76744467	76744468	MACS_peak_6731	16.00	lobChIP_FOXA1_robot
chr17	76828233	76828234	MACS_peak_6732	5.00	lobChIP_FOXA1_robot
chr17	76834761	76834762	MACS_peak_6733	4.00	lobChIP_FOXA1_robot
chr17	76915294	76915295	MACS_peak_6734	3.00	lobChIP_FOXA1_robot
chr17	76958744	76958745	MACS_peak_6735	6.00	lobChIP_FOXA1_robot
chr17	77023045	77023046	MACS_peak_6736	5.00	lobChIP_FOXA1_robot
chr17	77707002	77707003	MACS_peak_6737	5.00	lobChIP_FOXA1_robot
chr17	77750547	77750548	MACS_peak_6738	8.00	lobChIP_FOXA1_robot
chr17	77795271	77795272	MACS_peak_6739	14.00	lobChIP_FOXA1_robot
chr17	77879902	77879903	MACS_peak_6740	5.00	lobChIP_FOXA1_robot
chr17	77905757	77905758	MACS_peak_6741	5.00	lobChIP_FOXA1_robot
chr17	77960674	77960675	MACS_peak_6742	4.00	lobChIP_FOXA1_robot
chr17	77962085	77962086	MACS_peak_6743	7.00	lobChIP_FOXA1_robot
chr17	77966281	77966282	MACS_peak_6744	4.00	lobChIP_FOXA1_robot
chr17	77967638	77967639	MACS_peak_6745	6.00	lobChIP_FOXA1_robot
chr17	77993343	77993344	MACS_peak_6746	9.00	lobChIP_FOXA1_robot
chr17	78235327	78235328	MACS_peak_6747	15.00	lobChIP_FOXA1_robot
chr17	78602407	78602408	MACS_peak_6748	4.00	lobChIP_FOXA1_robot
chr17	78603889	78603890	MACS_peak_6749	5.00	lobChIP_FOXA1_robot
chr17	78630896	78630897	MACS_peak_6750	4.00	lobChIP_FOXA1_robot
chr17	78631962	78631963	MACS_peak_6751	5.00	lobChIP_FOXA1_robot
chr17	78641468	78641469	MACS_peak_6752	9.00	lobChIP_FOXA1_robot
chr17	78646130	78646131	MACS_peak_6753	18.00	lobChIP_FOXA1_robot
chr17	78647449	78647450	MACS_peak_6754	8.00	lobChIP_FOXA1_robot
chr17	78765514	78765515	MACS_peak_6755	5.00	lobChIP_FOXA1_robot
chr17	79057199	79057200	MACS_peak_6756	4.00	lobChIP_FOXA1_robot
chr17	79272857	79272858	MACS_peak_6757	6.00	lobChIP_FOXA1_robot
chr17	79313973	79313974	MACS_peak_6758	5.00	lobChIP_FOXA1_robot
chr17	79367944	79367945	MACS_peak_6759	27.00	lobChIP_FOXA1_robot
chr17	79371178	79371179	MACS_peak_6760	3.00	lobChIP_FOXA1_robot
chr17	79371754	79371755	MACS_peak_6761	9.00	lobChIP_FOXA1_robot
chr17	79458234	79458235	MACS_peak_6762	5.00	lobChIP_FOXA1_robot
chr17	79543744	79543745	MACS_peak_6763	9.00	lobChIP_FOXA1_robot
chr17	79551182	79551183	MACS_peak_6764	5.00	lobChIP_FOXA1_robot
chr17	79694758	79694759	MACS_peak_6765	4.00	lobChIP_FOXA1_robot
chr17	79950444	79950445	MACS_peak_6766	5.00	lobChIP_FOXA1_robot
chr17	79953008	79953009	MACS_peak_6767	7.00	lobChIP_FOXA1_robot
chr17	79958514	79958515	MACS_peak_6768	5.00	lobChIP_FOXA1_robot
chr17	80189865	80189866	MACS_peak_6769	5.00	lobChIP_FOXA1_robot
chr17	80200717	80200718	MACS_peak_6770	4.00	lobChIP_FOXA1_robot
chr17	80253017	80253018	MACS_peak_6771	3.00	lobChIP_FOXA1_robot
chr17	80253674	80253675	MACS_peak_6772	8.00	lobChIP_FOXA1_robot
chr17	80304371	80304372	MACS_peak_6773	4.00	lobChIP_FOXA1_robot
chr17	80415204	80415205	MACS_peak_6774	5.00	lobChIP_FOXA1_robot
chr17	80420024	80420025	MACS_peak_6775	5.00	lobChIP_FOXA1_robot
chr17	80475336	80475337	MACS_peak_6776	7.00	lobChIP_FOXA1_robot
chr17	80506403	80506404	MACS_peak_6777	3.00	lobChIP_FOXA1_robot
chr17	80508042	80508043	MACS_peak_6778	5.00	lobChIP_FOXA1_robot
chr17	80524196	80524197	MACS_peak_6779	4.00	lobChIP_FOXA1_robot
chr17	80526479	80526480	MACS_peak_6780	31.00	lobChIP_FOXA1_robot
chr17	80696337	80696338	MACS_peak_6781	11.00	lobChIP_FOXA1_robot
chr17	80847519	80847520	MACS_peak_6782	22.00	lobChIP_FOXA1_robot
chr17	81099517	81099518	MACS_peak_6783	15.00	lobChIP_FOXA1_robot
chr17	81109786	81109787	MACS_peak_6784	5.00	lobChIP_FOXA1_robot
chr17	81122362	81122363	MACS_peak_6785	30.00	lobChIP_FOXA1_robot
chr18	189478	189479	MACS_peak_6786	6.00	lobChIP_FOXA1_robot
chr18	362548	362549	MACS_peak_6787	6.00	lobChIP_FOXA1_robot
chr18	499622	499623	MACS_peak_6788	7.00	lobChIP_FOXA1_robot
chr18	624196	624197	MACS_peak_6789	6.00	lobChIP_FOXA1_robot
chr18	682960	682961	MACS_peak_6790	11.00	lobChIP_FOXA1_robot
chr18	770785	770786	MACS_peak_6791	5.00	lobChIP_FOXA1_robot
chr18	775022	775023	MACS_peak_6792	6.00	lobChIP_FOXA1_robot
chr18	1270290	1270291	MACS_peak_6793	5.00	lobChIP_FOXA1_robot
chr18	1624855	1624856	MACS_peak_6794	10.00	lobChIP_FOXA1_robot
chr18	2886581	2886582	MACS_peak_6795	6.00	lobChIP_FOXA1_robot
chr18	2985236	2985237	MACS_peak_6796	5.00	lobChIP_FOXA1_robot
chr18	2986405	2986406	MACS_peak_6797	13.00	lobChIP_FOXA1_robot
chr18	3037586	3037587	MACS_peak_6798	14.00	lobChIP_FOXA1_robot
chr18	3052248	3052249	MACS_peak_6799	4.00	lobChIP_FOXA1_robot
chr18	3059457	3059458	MACS_peak_6800	4.00	lobChIP_FOXA1_robot
chr18	3117785	3117786	MACS_peak_6801	5.00	lobChIP_FOXA1_robot
chr18	3200530	3200531	MACS_peak_6802	9.00	lobChIP_FOXA1_robot
chr18	3218099	3218100	MACS_peak_6803	5.00	lobChIP_FOXA1_robot
chr18	3233209	3233210	MACS_peak_6804	4.00	lobChIP_FOXA1_robot
chr18	3233960	3233961	MACS_peak_6805	12.00	lobChIP_FOXA1_robot
chr18	3256860	3256861	MACS_peak_6806	8.00	lobChIP_FOXA1_robot
chr18	3449147	3449148	MACS_peak_6807	11.00	lobChIP_FOXA1_robot
chr18	3456885	3456886	MACS_peak_6808	15.00	lobChIP_FOXA1_robot
chr18	3593902	3593903	MACS_peak_6809	4.00	lobChIP_FOXA1_robot
chr18	3596018	3596019	MACS_peak_6810	17.00	lobChIP_FOXA1_robot
chr18	3603184	3603185	MACS_peak_6811	3.00	lobChIP_FOXA1_robot
chr18	3604854	3604855	MACS_peak_6812	6.00	lobChIP_FOXA1_robot
chr18	3618318	3618319	MACS_peak_6813	43.00	lobChIP_FOXA1_robot
chr18	3634434	3634435	MACS_peak_6814	4.00	lobChIP_FOXA1_robot
chr18	3664778	3664779	MACS_peak_6815	7.00	lobChIP_FOXA1_robot
chr18	3665989	3665990	MACS_peak_6816	6.00	lobChIP_FOXA1_robot
chr18	3723238	3723239	MACS_peak_6817	5.00	lobChIP_FOXA1_robot
chr18	5298501	5298502	MACS_peak_6818	5.00	lobChIP_FOXA1_robot
chr18	5303852	5303853	MACS_peak_6819	8.00	lobChIP_FOXA1_robot
chr18	5339670	5339671	MACS_peak_6820	11.00	lobChIP_FOXA1_robot
chr18	5747097	5747098	MACS_peak_6821	10.00	lobChIP_FOXA1_robot
chr18	6211137	6211138	MACS_peak_6822	4.00	lobChIP_FOXA1_robot
chr18	6376987	6376988	MACS_peak_6823	3.00	lobChIP_FOXA1_robot
chr18	6835581	6835582	MACS_peak_6824	7.00	lobChIP_FOXA1_robot
chr18	6962721	6962722	MACS_peak_6825	4.00	lobChIP_FOXA1_robot
chr18	7626637	7626638	MACS_peak_6826	6.00	lobChIP_FOXA1_robot
chr18	7628696	7628697	MACS_peak_6827	5.00	lobChIP_FOXA1_robot
chr18	7637465	7637466	MACS_peak_6828	9.00	lobChIP_FOXA1_robot
chr18	7646420	7646421	MACS_peak_6829	14.00	lobChIP_FOXA1_robot
chr18	7658185	7658186	MACS_peak_6830	4.00	lobChIP_FOXA1_robot
chr18	7749829	7749830	MACS_peak_6831	7.00	lobChIP_FOXA1_robot
chr18	7755253	7755254	MACS_peak_6832	4.00	lobChIP_FOXA1_robot
chr18	7756329	7756330	MACS_peak_6833	6.00	lobChIP_FOXA1_robot
chr18	7761265	7761266	MACS_peak_6834	3.00	lobChIP_FOXA1_robot
chr18	7804393	7804394	MACS_peak_6835	10.00	lobChIP_FOXA1_robot
chr18	7829848	7829849	MACS_peak_6836	5.00	lobChIP_FOXA1_robot
chr18	7915119	7915120	MACS_peak_6837	8.00	lobChIP_FOXA1_robot
chr18	7919689	7919690	MACS_peak_6838	4.00	lobChIP_FOXA1_robot
chr18	8176940	8176941	MACS_peak_6839	3.00	lobChIP_FOXA1_robot
chr18	8177868	8177869	MACS_peak_6840	3.00	lobChIP_FOXA1_robot
chr18	8238642	8238643	MACS_peak_6841	5.00	lobChIP_FOXA1_robot
chr18	8341751	8341752	MACS_peak_6842	24.00	lobChIP_FOXA1_robot
chr18	8358160	8358161	MACS_peak_6843	5.00	lobChIP_FOXA1_robot
chr18	8730483	8730484	MACS_peak_6844	7.00	lobChIP_FOXA1_robot
chr18	8959810	8959811	MACS_peak_6845	7.00	lobChIP_FOXA1_robot
chr18	8960913	8960914	MACS_peak_6846	3.00	lobChIP_FOXA1_robot
chr18	9067918	9067919	MACS_peak_6847	7.00	lobChIP_FOXA1_robot
chr18	9073812	9073813	MACS_peak_6848	6.00	lobChIP_FOXA1_robot
chr18	9092795	9092796	MACS_peak_6849	10.00	lobChIP_FOXA1_robot
chr18	9094860	9094861	MACS_peak_6850	13.00	lobChIP_FOXA1_robot
chr18	9101952	9101953	MACS_peak_6851	6.00	lobChIP_FOXA1_robot
chr18	9104686	9104687	MACS_peak_6852	7.00	lobChIP_FOXA1_robot
chr18	9215416	9215417	MACS_peak_6853	7.00	lobChIP_FOXA1_robot
chr18	9514272	9514273	MACS_peak_6854	8.00	lobChIP_FOXA1_robot
chr18	9535534	9535535	MACS_peak_6855	8.00	lobChIP_FOXA1_robot
chr18	9609008	9609009	MACS_peak_6856	17.00	lobChIP_FOXA1_robot
chr18	9647911	9647912	MACS_peak_6857	8.00	lobChIP_FOXA1_robot
chr18	9743054	9743055	MACS_peak_6858	4.00	lobChIP_FOXA1_robot
chr18	9814607	9814608	MACS_peak_6859	7.00	lobChIP_FOXA1_robot
chr18	9916436	9916437	MACS_peak_6860	5.00	lobChIP_FOXA1_robot
chr18	10024296	10024297	MACS_peak_6861	5.00	lobChIP_FOXA1_robot
chr18	10464274	10464275	MACS_peak_6862	7.00	lobChIP_FOXA1_robot
chr18	10525662	10525663	MACS_peak_6863	5.00	lobChIP_FOXA1_robot
chr18	10705978	10705979	MACS_peak_6864	7.00	lobChIP_FOXA1_robot
chr18	10893371	10893372	MACS_peak_6865	5.00	lobChIP_FOXA1_robot
chr18	10901612	10901613	MACS_peak_6866	5.00	lobChIP_FOXA1_robot
chr18	10985907	10985908	MACS_peak_6867	4.00	lobChIP_FOXA1_robot
chr18	11005828	11005829	MACS_peak_6868	13.00	lobChIP_FOXA1_robot
chr18	11113210	11113211	MACS_peak_6869	5.00	lobChIP_FOXA1_robot
chr18	11120188	11120189	MACS_peak_6870	4.00	lobChIP_FOXA1_robot
chr18	11120737	11120738	MACS_peak_6871	5.00	lobChIP_FOXA1_robot
chr18	11177502	11177503	MACS_peak_6872	4.00	lobChIP_FOXA1_robot
chr18	11283741	11283742	MACS_peak_6873	4.00	lobChIP_FOXA1_robot
chr18	11394868	11394869	MACS_peak_6874	4.00	lobChIP_FOXA1_robot
chr18	11494254	11494255	MACS_peak_6875	6.00	lobChIP_FOXA1_robot
chr18	11500973	11500974	MACS_peak_6876	21.00	lobChIP_FOXA1_robot
chr18	11840729	11840730	MACS_peak_6877	6.00	lobChIP_FOXA1_robot
chr18	11925994	11925995	MACS_peak_6878	3.00	lobChIP_FOXA1_robot
chr18	11994552	11994553	MACS_peak_6879	7.00	lobChIP_FOXA1_robot
chr18	12000470	12000471	MACS_peak_6880	5.00	lobChIP_FOXA1_robot
chr18	12008443	12008444	MACS_peak_6881	12.00	lobChIP_FOXA1_robot
chr18	12337051	12337052	MACS_peak_6882	14.00	lobChIP_FOXA1_robot
chr18	12411098	12411099	MACS_peak_6883	5.00	lobChIP_FOXA1_robot
chr18	12608493	12608494	MACS_peak_6884	10.00	lobChIP_FOXA1_robot
chr18	12640521	12640522	MACS_peak_6885	7.00	lobChIP_FOXA1_robot
chr18	12699951	12699952	MACS_peak_6886	10.00	lobChIP_FOXA1_robot
chr18	12730358	12730359	MACS_peak_6887	13.00	lobChIP_FOXA1_robot
chr18	12741419	12741420	MACS_peak_6888	12.00	lobChIP_FOXA1_robot
chr18	13176031	13176032	MACS_peak_6889	13.00	lobChIP_FOXA1_robot
chr18	13179749	13179750	MACS_peak_6890	20.00	lobChIP_FOXA1_robot
chr18	13540320	13540321	MACS_peak_6891	5.00	lobChIP_FOXA1_robot
chr18	13542207	13542208	MACS_peak_6892	16.00	lobChIP_FOXA1_robot
chr18	19106736	19106737	MACS_peak_6894	4.00	lobChIP_FOXA1_robot
chr18	19118398	19118399	MACS_peak_6895	6.00	lobChIP_FOXA1_robot
chr18	19130529	19130530	MACS_peak_6896	4.00	lobChIP_FOXA1_robot
chr18	19218470	19218471	MACS_peak_6897	12.00	lobChIP_FOXA1_robot
chr18	19264831	19264832	MACS_peak_6898	9.00	lobChIP_FOXA1_robot
chr18	19315695	19315696	MACS_peak_6899	7.00	lobChIP_FOXA1_robot
chr18	19571193	19571194	MACS_peak_6900	11.00	lobChIP_FOXA1_robot
chr18	19581927	19581928	MACS_peak_6901	7.00	lobChIP_FOXA1_robot
chr18	19598506	19598507	MACS_peak_6902	4.00	lobChIP_FOXA1_robot
chr18	19629118	19629119	MACS_peak_6903	5.00	lobChIP_FOXA1_robot
chr18	19638272	19638273	MACS_peak_6904	8.00	lobChIP_FOXA1_robot
chr18	19650221	19650222	MACS_peak_6905	4.00	lobChIP_FOXA1_robot
chr18	19749448	19749449	MACS_peak_6906	5.00	lobChIP_FOXA1_robot
chr18	19755446	19755447	MACS_peak_6907	14.00	lobChIP_FOXA1_robot
chr18	20284518	20284519	MACS_peak_6908	10.00	lobChIP_FOXA1_robot
chr18	20679776	20679777	MACS_peak_6909	18.00	lobChIP_FOXA1_robot
chr18	20681919	20681920	MACS_peak_6910	6.00	lobChIP_FOXA1_robot
chr18	20758833	20758834	MACS_peak_6911	5.00	lobChIP_FOXA1_robot
chr18	20957860	20957861	MACS_peak_6912	5.00	lobChIP_FOXA1_robot
chr18	20965314	20965315	MACS_peak_6913	11.00	lobChIP_FOXA1_robot
chr18	21022771	21022772	MACS_peak_6914	5.00	lobChIP_FOXA1_robot
chr18	21125582	21125583	MACS_peak_6915	8.00	lobChIP_FOXA1_robot
chr18	21152178	21152179	MACS_peak_6916	5.00	lobChIP_FOXA1_robot
chr18	21209581	21209582	MACS_peak_6917	12.00	lobChIP_FOXA1_robot
chr18	21439646	21439647	MACS_peak_6918	3.00	lobChIP_FOXA1_robot
chr18	21463586	21463587	MACS_peak_6919	5.00	lobChIP_FOXA1_robot
chr18	21631633	21631634	MACS_peak_6920	12.00	lobChIP_FOXA1_robot
chr18	21643816	21643817	MACS_peak_6921	9.00	lobChIP_FOXA1_robot
chr18	21651286	21651287	MACS_peak_6922	10.00	lobChIP_FOXA1_robot
chr18	21651913	21651914	MACS_peak_6923	12.00	lobChIP_FOXA1_robot
chr18	21695320	21695321	MACS_peak_6924	6.00	lobChIP_FOXA1_robot
chr18	21795960	21795961	MACS_peak_6925	11.00	lobChIP_FOXA1_robot
chr18	22070584	22070585	MACS_peak_6926	3.00	lobChIP_FOXA1_robot
chr18	22071473	22071474	MACS_peak_6927	3.00	lobChIP_FOXA1_robot
chr18	23544850	23544851	MACS_peak_6928	3.00	lobChIP_FOXA1_robot
chr18	24155636	24155637	MACS_peak_6929	6.00	lobChIP_FOXA1_robot
chr18	24322820	24322821	MACS_peak_6930	4.00	lobChIP_FOXA1_robot
chr18	24716295	24716296	MACS_peak_6931	3.00	lobChIP_FOXA1_robot
chr18	24723128	24723129	MACS_peak_6932	6.00	lobChIP_FOXA1_robot
chr18	24778367	24778368	MACS_peak_6933	8.00	lobChIP_FOXA1_robot
chr18	25602999	25603000	MACS_peak_6934	4.00	lobChIP_FOXA1_robot
chr18	25707926	25707927	MACS_peak_6935	11.00	lobChIP_FOXA1_robot
chr18	25708498	25708499	MACS_peak_6936	6.00	lobChIP_FOXA1_robot
chr18	25747911	25747912	MACS_peak_6937	23.00	lobChIP_FOXA1_robot
chr18	28338375	28338376	MACS_peak_6938	5.00	lobChIP_FOXA1_robot
chr18	28783422	28783423	MACS_peak_6939	3.00	lobChIP_FOXA1_robot
chr18	28784965	28784966	MACS_peak_6940	4.00	lobChIP_FOXA1_robot
chr18	28810635	28810636	MACS_peak_6941	5.00	lobChIP_FOXA1_robot
chr18	28825091	28825092	MACS_peak_6942	6.00	lobChIP_FOXA1_robot
chr18	28842346	28842347	MACS_peak_6943	7.00	lobChIP_FOXA1_robot
chr18	28855064	28855065	MACS_peak_6944	5.00	lobChIP_FOXA1_robot
chr18	28858649	28858650	MACS_peak_6945	6.00	lobChIP_FOXA1_robot
chr18	28953778	28953779	MACS_peak_6946	6.00	lobChIP_FOXA1_robot
chr18	28982112	28982113	MACS_peak_6947	10.00	lobChIP_FOXA1_robot
chr18	29092315	29092316	MACS_peak_6948	29.00	lobChIP_FOXA1_robot
chr18	29138642	29138643	MACS_peak_6949	3.00	lobChIP_FOXA1_robot
chr18	29166737	29166738	MACS_peak_6950	6.00	lobChIP_FOXA1_robot
chr18	29167693	29167694	MACS_peak_6951	4.00	lobChIP_FOXA1_robot
chr18	29171700	29171701	MACS_peak_6952	9.00	lobChIP_FOXA1_robot
chr18	29523938	29523939	MACS_peak_6953	12.00	lobChIP_FOXA1_robot
chr18	29599728	29599729	MACS_peak_6954	4.00	lobChIP_FOXA1_robot
chr18	29952434	29952435	MACS_peak_6955	5.00	lobChIP_FOXA1_robot
chr18	30378428	30378429	MACS_peak_6956	5.00	lobChIP_FOXA1_robot
chr18	30383775	30383776	MACS_peak_6957	53.00	lobChIP_FOXA1_robot
chr18	30384703	30384704	MACS_peak_6958	8.00	lobChIP_FOXA1_robot
chr18	30594275	30594276	MACS_peak_6959	16.00	lobChIP_FOXA1_robot
chr18	31331753	31331754	MACS_peak_6960	4.00	lobChIP_FOXA1_robot
chr18	32072803	32072804	MACS_peak_6961	12.00	lobChIP_FOXA1_robot
chr18	32123489	32123490	MACS_peak_6962	4.00	lobChIP_FOXA1_robot
chr18	32492259	32492260	MACS_peak_6963	7.00	lobChIP_FOXA1_robot
chr18	32494015	32494016	MACS_peak_6964	4.00	lobChIP_FOXA1_robot
chr18	32509375	32509376	MACS_peak_6965	17.00	lobChIP_FOXA1_robot
chr18	32539337	32539338	MACS_peak_6966	8.00	lobChIP_FOXA1_robot
chr18	32605808	32605809	MACS_peak_6967	5.00	lobChIP_FOXA1_robot
chr18	32662997	32662998	MACS_peak_6968	4.00	lobChIP_FOXA1_robot
chr18	32664952	32664953	MACS_peak_6969	5.00	lobChIP_FOXA1_robot
chr18	32665853	32665854	MACS_peak_6970	7.00	lobChIP_FOXA1_robot
chr18	32751436	32751437	MACS_peak_6971	5.00	lobChIP_FOXA1_robot
chr18	33049804	33049805	MACS_peak_6972	6.00	lobChIP_FOXA1_robot
chr18	33059523	33059524	MACS_peak_6973	3.00	lobChIP_FOXA1_robot
chr18	33062025	33062026	MACS_peak_6974	8.00	lobChIP_FOXA1_robot
chr18	33133580	33133581	MACS_peak_6975	8.00	lobChIP_FOXA1_robot
chr18	33283228	33283229	MACS_peak_6976	17.00	lobChIP_FOXA1_robot
chr18	33291335	33291336	MACS_peak_6977	7.00	lobChIP_FOXA1_robot
chr18	33296441	33296442	MACS_peak_6978	7.00	lobChIP_FOXA1_robot
chr18	33304339	33304340	MACS_peak_6979	4.00	lobChIP_FOXA1_robot
chr18	33756327	33756328	MACS_peak_6980	6.00	lobChIP_FOXA1_robot
chr18	33797430	33797431	MACS_peak_6981	4.00	lobChIP_FOXA1_robot
chr18	33824328	33824329	MACS_peak_6982	4.00	lobChIP_FOXA1_robot
chr18	36821746	36821747	MACS_peak_6983	14.00	lobChIP_FOXA1_robot
chr18	36924706	36924707	MACS_peak_6984	3.00	lobChIP_FOXA1_robot
chr18	36999381	36999382	MACS_peak_6985	5.00	lobChIP_FOXA1_robot
chr18	37253373	37253374	MACS_peak_6986	4.00	lobChIP_FOXA1_robot
chr18	37282042	37282043	MACS_peak_6987	6.00	lobChIP_FOXA1_robot
chr18	39021162	39021163	MACS_peak_6988	3.00	lobChIP_FOXA1_robot
chr18	39496375	39496376	MACS_peak_6989	4.00	lobChIP_FOXA1_robot
chr18	39589639	39589640	MACS_peak_6990	4.00	lobChIP_FOXA1_robot
chr18	39692179	39692180	MACS_peak_6991	4.00	lobChIP_FOXA1_robot
chr18	39692850	39692851	MACS_peak_6992	9.00	lobChIP_FOXA1_robot
chr18	39748617	39748618	MACS_peak_6993	12.00	lobChIP_FOXA1_robot
chr18	39822675	39822676	MACS_peak_6994	4.00	lobChIP_FOXA1_robot
chr18	39919486	39919487	MACS_peak_6995	4.00	lobChIP_FOXA1_robot
chr18	39920567	39920568	MACS_peak_6996	6.00	lobChIP_FOXA1_robot
chr18	39921986	39921987	MACS_peak_6997	15.00	lobChIP_FOXA1_robot
chr18	40026354	40026355	MACS_peak_6998	9.00	lobChIP_FOXA1_robot
chr18	42128558	42128559	MACS_peak_6999	3.00	lobChIP_FOXA1_robot
chr18	43632268	43632269	MACS_peak_7000	16.00	lobChIP_FOXA1_robot
chr18	43779667	43779668	MACS_peak_7001	6.00	lobChIP_FOXA1_robot
chr18	44622384	44622385	MACS_peak_7002	6.00	lobChIP_FOXA1_robot
chr18	45101539	45101540	MACS_peak_7003	24.00	lobChIP_FOXA1_robot
chr18	45157256	45157257	MACS_peak_7004	3.00	lobChIP_FOXA1_robot
chr18	45408268	45408269	MACS_peak_7005	4.00	lobChIP_FOXA1_robot
chr18	46102484	46102485	MACS_peak_7006	6.00	lobChIP_FOXA1_robot
chr18	46161635	46161636	MACS_peak_7007	3.00	lobChIP_FOXA1_robot
chr18	46428467	46428468	MACS_peak_7008	6.00	lobChIP_FOXA1_robot
chr18	46436409	46436410	MACS_peak_7009	5.00	lobChIP_FOXA1_robot
chr18	46473144	46473145	MACS_peak_7010	10.00	lobChIP_FOXA1_robot
chr18	46474940	46474941	MACS_peak_7011	8.00	lobChIP_FOXA1_robot
chr18	46520559	46520560	MACS_peak_7012	6.00	lobChIP_FOXA1_robot
chr18	46597103	46597104	MACS_peak_7013	8.00	lobChIP_FOXA1_robot
chr18	46781292	46781293	MACS_peak_7014	5.00	lobChIP_FOXA1_robot
chr18	46816725	46816726	MACS_peak_7015	5.00	lobChIP_FOXA1_robot
chr18	46895136	46895137	MACS_peak_7016	5.00	lobChIP_FOXA1_robot
chr18	46904078	46904079	MACS_peak_7017	4.00	lobChIP_FOXA1_robot
chr18	47074739	47074740	MACS_peak_7018	3.00	lobChIP_FOXA1_robot
chr18	47165620	47165621	MACS_peak_7019	7.00	lobChIP_FOXA1_robot
chr18	47250407	47250408	MACS_peak_7020	4.00	lobChIP_FOXA1_robot
chr18	47258184	47258185	MACS_peak_7021	4.00	lobChIP_FOXA1_robot
chr18	47358795	47358796	MACS_peak_7022	19.00	lobChIP_FOXA1_robot
chr18	47361570	47361571	MACS_peak_7023	31.00	lobChIP_FOXA1_robot
chr18	47364527	47364528	MACS_peak_7024	13.00	lobChIP_FOXA1_robot
chr18	47437868	47437869	MACS_peak_7025	11.00	lobChIP_FOXA1_robot
chr18	47493756	47493757	MACS_peak_7026	6.00	lobChIP_FOXA1_robot
chr18	47617232	47617233	MACS_peak_7027	12.00	lobChIP_FOXA1_robot
chr18	47920332	47920333	MACS_peak_7028	5.00	lobChIP_FOXA1_robot
chr18	47931391	47931392	MACS_peak_7029	5.00	lobChIP_FOXA1_robot
chr18	47951952	47951953	MACS_peak_7030	3.00	lobChIP_FOXA1_robot
chr18	48429345	48429346	MACS_peak_7031	17.00	lobChIP_FOXA1_robot
chr18	48500303	48500304	MACS_peak_7032	4.00	lobChIP_FOXA1_robot
chr18	48806271	48806272	MACS_peak_7033	5.00	lobChIP_FOXA1_robot
chr18	49596939	49596940	MACS_peak_7034	7.00	lobChIP_FOXA1_robot
chr18	49608911	49608912	MACS_peak_7035	4.00	lobChIP_FOXA1_robot
chr18	50551825	50551826	MACS_peak_7036	4.00	lobChIP_FOXA1_robot
chr18	51773387	51773388	MACS_peak_7037	4.00	lobChIP_FOXA1_robot
chr18	51780877	51780878	MACS_peak_7038	7.00	lobChIP_FOXA1_robot
chr18	51790688	51790689	MACS_peak_7039	4.00	lobChIP_FOXA1_robot
chr18	52434527	52434528	MACS_peak_7040	8.00	lobChIP_FOXA1_robot
chr18	52613552	52613553	MACS_peak_7041	6.00	lobChIP_FOXA1_robot
chr18	52615831	52615832	MACS_peak_7042	21.00	lobChIP_FOXA1_robot
chr18	52624807	52624808	MACS_peak_7043	5.00	lobChIP_FOXA1_robot
chr18	54309215	54309216	MACS_peak_7044	8.00	lobChIP_FOXA1_robot
chr18	55091266	55091267	MACS_peak_7045	27.00	lobChIP_FOXA1_robot
chr18	55112788	55112789	MACS_peak_7046	5.00	lobChIP_FOXA1_robot
chr18	55200754	55200755	MACS_peak_7047	5.00	lobChIP_FOXA1_robot
chr18	55405130	55405131	MACS_peak_7048	6.00	lobChIP_FOXA1_robot
chr18	55416170	55416171	MACS_peak_7049	15.00	lobChIP_FOXA1_robot
chr18	55439906	55439907	MACS_peak_7050	6.00	lobChIP_FOXA1_robot
chr18	55464575	55464576	MACS_peak_7051	9.00	lobChIP_FOXA1_robot
chr18	55466197	55466198	MACS_peak_7052	5.00	lobChIP_FOXA1_robot
chr18	55593361	55593362	MACS_peak_7053	4.00	lobChIP_FOXA1_robot
chr18	55594888	55594889	MACS_peak_7054	9.00	lobChIP_FOXA1_robot
chr18	55618834	55618835	MACS_peak_7055	5.00	lobChIP_FOXA1_robot
chr18	55648381	55648382	MACS_peak_7056	11.00	lobChIP_FOXA1_robot
chr18	55680916	55680917	MACS_peak_7057	39.00	lobChIP_FOXA1_robot
chr18	55691706	55691707	MACS_peak_7058	9.00	lobChIP_FOXA1_robot
chr18	55710871	55710872	MACS_peak_7059	6.00	lobChIP_FOXA1_robot
chr18	55713909	55713910	MACS_peak_7060	6.00	lobChIP_FOXA1_robot
chr18	55764604	55764605	MACS_peak_7061	5.00	lobChIP_FOXA1_robot
chr18	55902387	55902388	MACS_peak_7062	6.00	lobChIP_FOXA1_robot
chr18	56075141	56075142	MACS_peak_7063	55.00	lobChIP_FOXA1_robot
chr18	56076167	56076168	MACS_peak_7064	5.00	lobChIP_FOXA1_robot
chr18	56080179	56080180	MACS_peak_7065	5.00	lobChIP_FOXA1_robot
chr18	56081392	56081393	MACS_peak_7066	62.00	lobChIP_FOXA1_robot
chr18	56099514	56099515	MACS_peak_7067	5.00	lobChIP_FOXA1_robot
chr18	56217317	56217318	MACS_peak_7068	44.00	lobChIP_FOXA1_robot
chr18	56219576	56219577	MACS_peak_7069	8.00	lobChIP_FOXA1_robot
chr18	56272754	56272755	MACS_peak_7070	22.00	lobChIP_FOXA1_robot
chr18	56336479	56336480	MACS_peak_7071	5.00	lobChIP_FOXA1_robot
chr18	56337097	56337098	MACS_peak_7072	11.00	lobChIP_FOXA1_robot
chr18	56428543	56428544	MACS_peak_7073	3.00	lobChIP_FOXA1_robot
chr18	56620305	56620306	MACS_peak_7074	16.00	lobChIP_FOXA1_robot
chr18	56809207	56809208	MACS_peak_7075	5.00	lobChIP_FOXA1_robot
chr18	57121010	57121011	MACS_peak_7076	23.00	lobChIP_FOXA1_robot
chr18	57183820	57183821	MACS_peak_7077	7.00	lobChIP_FOXA1_robot
chr18	57549310	57549311	MACS_peak_7078	6.00	lobChIP_FOXA1_robot
chr18	58045184	58045185	MACS_peak_7079	3.00	lobChIP_FOXA1_robot
chr18	60119621	60119622	MACS_peak_7080	18.00	lobChIP_FOXA1_robot
chr18	60128211	60128212	MACS_peak_7081	9.00	lobChIP_FOXA1_robot
chr18	60391295	60391296	MACS_peak_7082	4.00	lobChIP_FOXA1_robot
chr18	60408496	60408497	MACS_peak_7083	8.00	lobChIP_FOXA1_robot
chr18	60409479	60409480	MACS_peak_7084	4.00	lobChIP_FOXA1_robot
chr18	60463668	60463669	MACS_peak_7085	5.00	lobChIP_FOXA1_robot
chr18	60467962	60467963	MACS_peak_7086	7.00	lobChIP_FOXA1_robot
chr18	60477246	60477247	MACS_peak_7087	5.00	lobChIP_FOXA1_robot
chr18	61035143	61035144	MACS_peak_7088	5.00	lobChIP_FOXA1_robot
chr18	61349349	61349350	MACS_peak_7089	4.00	lobChIP_FOXA1_robot
chr18	63122776	63122777	MACS_peak_7090	5.00	lobChIP_FOXA1_robot
chr18	64542473	64542474	MACS_peak_7091	7.00	lobChIP_FOXA1_robot
chr18	65052910	65052911	MACS_peak_7092	4.00	lobChIP_FOXA1_robot
chr18	66554848	66554849	MACS_peak_7093	6.00	lobChIP_FOXA1_robot
chr18	67017284	67017285	MACS_peak_7094	14.00	lobChIP_FOXA1_robot
chr18	67025974	67025975	MACS_peak_7095	17.00	lobChIP_FOXA1_robot
chr18	67235531	67235532	MACS_peak_7096	6.00	lobChIP_FOXA1_robot
chr18	67326795	67326796	MACS_peak_7097	3.00	lobChIP_FOXA1_robot
chr18	67420324	67420325	MACS_peak_7098	19.00	lobChIP_FOXA1_robot
chr18	67813557	67813558	MACS_peak_7099	4.00	lobChIP_FOXA1_robot
chr18	67981001	67981002	MACS_peak_7100	4.00	lobChIP_FOXA1_robot
chr18	68001028	68001029	MACS_peak_7101	5.00	lobChIP_FOXA1_robot
chr18	68002442	68002443	MACS_peak_7102	11.00	lobChIP_FOXA1_robot
chr18	68003228	68003229	MACS_peak_7103	8.00	lobChIP_FOXA1_robot
chr18	68041508	68041509	MACS_peak_7104	4.00	lobChIP_FOXA1_robot
chr18	68044413	68044414	MACS_peak_7105	3.00	lobChIP_FOXA1_robot
chr18	68044751	68044752	MACS_peak_7106	5.00	lobChIP_FOXA1_robot
chr18	68066405	68066406	MACS_peak_7107	4.00	lobChIP_FOXA1_robot
chr18	68098602	68098603	MACS_peak_7108	14.00	lobChIP_FOXA1_robot
chr18	68129544	68129545	MACS_peak_7109	4.00	lobChIP_FOXA1_robot
chr18	68191312	68191313	MACS_peak_7110	6.00	lobChIP_FOXA1_robot
chr18	68795556	68795557	MACS_peak_7111	3.00	lobChIP_FOXA1_robot
chr18	69828249	69828250	MACS_peak_7112	4.00	lobChIP_FOXA1_robot
chr18	70857108	70857109	MACS_peak_7113	8.00	lobChIP_FOXA1_robot
chr18	71863594	71863595	MACS_peak_7114	5.00	lobChIP_FOXA1_robot
chr18	71893096	71893097	MACS_peak_7115	4.00	lobChIP_FOXA1_robot
chr18	71901714	71901715	MACS_peak_7116	9.00	lobChIP_FOXA1_robot
chr18	71916141	71916142	MACS_peak_7117	4.00	lobChIP_FOXA1_robot
chr18	71933673	71933674	MACS_peak_7118	3.00	lobChIP_FOXA1_robot
chr18	71950048	71950049	MACS_peak_7119	5.00	lobChIP_FOXA1_robot
chr18	71967207	71967208	MACS_peak_7120	3.00	lobChIP_FOXA1_robot
chr18	71984138	71984139	MACS_peak_7121	14.00	lobChIP_FOXA1_robot
chr18	72209468	72209469	MACS_peak_7122	5.00	lobChIP_FOXA1_robot
chr18	72419121	72419122	MACS_peak_7123	4.00	lobChIP_FOXA1_robot
chr18	72428545	72428546	MACS_peak_7124	6.00	lobChIP_FOXA1_robot
chr18	72446059	72446060	MACS_peak_7125	3.00	lobChIP_FOXA1_robot
chr18	72552704	72552705	MACS_peak_7126	5.00	lobChIP_FOXA1_robot
chr18	72749237	72749238	MACS_peak_7127	5.00	lobChIP_FOXA1_robot
chr18	72988353	72988354	MACS_peak_7128	3.00	lobChIP_FOXA1_robot
chr18	73002772	73002773	MACS_peak_7129	13.00	lobChIP_FOXA1_robot
chr18	74088374	74088375	MACS_peak_7130	8.00	lobChIP_FOXA1_robot
chr18	74205828	74205829	MACS_peak_7131	9.00	lobChIP_FOXA1_robot
chr18	74207901	74207902	MACS_peak_7132	4.00	lobChIP_FOXA1_robot
chr18	74214742	74214743	MACS_peak_7133	6.00	lobChIP_FOXA1_robot
chr18	74266021	74266022	MACS_peak_7134	3.00	lobChIP_FOXA1_robot
chr18	74557150	74557151	MACS_peak_7135	4.00	lobChIP_FOXA1_robot
chr18	75079340	75079341	MACS_peak_7136	8.00	lobChIP_FOXA1_robot
chr18	77694824	77694825	MACS_peak_7137	39.00	lobChIP_FOXA1_robot
chr18	77864545	77864546	MACS_peak_7138	4.00	lobChIP_FOXA1_robot
chr19	335192	335193	MACS_peak_7139	5.00	lobChIP_FOXA1_robot
chr19	513091	513092	MACS_peak_7140	5.00	lobChIP_FOXA1_robot
chr19	660840	660841	MACS_peak_7141	4.00	lobChIP_FOXA1_robot
chr19	752843	752844	MACS_peak_7142	7.00	lobChIP_FOXA1_robot
chr19	800686	800687	MACS_peak_7143	5.00	lobChIP_FOXA1_robot
chr19	909416	909417	MACS_peak_7144	8.00	lobChIP_FOXA1_robot
chr19	935121	935122	MACS_peak_7145	8.00	lobChIP_FOXA1_robot
chr19	952744	952745	MACS_peak_7146	4.00	lobChIP_FOXA1_robot
chr19	1253678	1253679	MACS_peak_7147	6.00	lobChIP_FOXA1_robot
chr19	1871518	1871519	MACS_peak_7148	7.00	lobChIP_FOXA1_robot
chr19	1897532	1897533	MACS_peak_7149	3.00	lobChIP_FOXA1_robot
chr19	2428060	2428061	MACS_peak_7150	10.00	lobChIP_FOXA1_robot
chr19	2458276	2458277	MACS_peak_7151	4.00	lobChIP_FOXA1_robot
chr19	2507228	2507229	MACS_peak_7152	7.00	lobChIP_FOXA1_robot
chr19	2533857	2533858	MACS_peak_7153	5.00	lobChIP_FOXA1_robot
chr19	2535760	2535761	MACS_peak_7154	7.00	lobChIP_FOXA1_robot
chr19	2588464	2588465	MACS_peak_7155	3.00	lobChIP_FOXA1_robot
chr19	2592089	2592090	MACS_peak_7156	4.00	lobChIP_FOXA1_robot
chr19	2639307	2639308	MACS_peak_7157	4.00	lobChIP_FOXA1_robot
chr19	3065860	3065861	MACS_peak_7158	6.00	lobChIP_FOXA1_robot
chr19	3398820	3398821	MACS_peak_7159	4.00	lobChIP_FOXA1_robot
chr19	3414155	3414156	MACS_peak_7160	5.00	lobChIP_FOXA1_robot
chr19	3968718	3968719	MACS_peak_7161	10.00	lobChIP_FOXA1_robot
chr19	4147511	4147512	MACS_peak_7162	7.00	lobChIP_FOXA1_robot
chr19	4625097	4625098	MACS_peak_7163	3.00	lobChIP_FOXA1_robot
chr19	4715938	4715939	MACS_peak_7164	8.00	lobChIP_FOXA1_robot
chr19	4870307	4870308	MACS_peak_7165	3.00	lobChIP_FOXA1_robot
chr19	5315429	5315430	MACS_peak_7166	3.00	lobChIP_FOXA1_robot
chr19	5470555	5470556	MACS_peak_7167	3.00	lobChIP_FOXA1_robot
chr19	5870403	5870404	MACS_peak_7168	6.00	lobChIP_FOXA1_robot
chr19	5871175	5871176	MACS_peak_7169	11.00	lobChIP_FOXA1_robot
chr19	5880753	5880754	MACS_peak_7170	9.00	lobChIP_FOXA1_robot
chr19	5909369	5909370	MACS_peak_7171	9.00	lobChIP_FOXA1_robot
chr19	5910185	5910186	MACS_peak_7172	9.00	lobChIP_FOXA1_robot
chr19	5954859	5954860	MACS_peak_7173	5.00	lobChIP_FOXA1_robot
chr19	5962574	5962575	MACS_peak_7174	3.00	lobChIP_FOXA1_robot
chr19	5975130	5975131	MACS_peak_7175	7.00	lobChIP_FOXA1_robot
chr19	6022561	6022562	MACS_peak_7176	5.00	lobChIP_FOXA1_robot
chr19	6066101	6066102	MACS_peak_7177	7.00	lobChIP_FOXA1_robot
chr19	6199933	6199934	MACS_peak_7178	8.00	lobChIP_FOXA1_robot
chr19	6273996	6273997	MACS_peak_7179	4.00	lobChIP_FOXA1_robot
chr19	7062767	7062768	MACS_peak_7180	6.00	lobChIP_FOXA1_robot
chr19	7145649	7145650	MACS_peak_7181	4.00	lobChIP_FOXA1_robot
chr19	7214519	7214520	MACS_peak_7182	9.00	lobChIP_FOXA1_robot
chr19	7224431	7224432	MACS_peak_7183	6.00	lobChIP_FOXA1_robot
chr19	7296321	7296322	MACS_peak_7184	9.00	lobChIP_FOXA1_robot
chr19	7463817	7463818	MACS_peak_7185	20.00	lobChIP_FOXA1_robot
chr19	7515296	7515297	MACS_peak_7186	4.00	lobChIP_FOXA1_robot
chr19	7717170	7717171	MACS_peak_7187	4.00	lobChIP_FOXA1_robot
chr19	8151158	8151159	MACS_peak_7188	6.00	lobChIP_FOXA1_robot
chr19	8253788	8253789	MACS_peak_7189	7.00	lobChIP_FOXA1_robot
chr19	8311517	8311518	MACS_peak_7190	17.00	lobChIP_FOXA1_robot
chr19	8323139	8323140	MACS_peak_7191	10.00	lobChIP_FOXA1_robot
chr19	8422335	8422336	MACS_peak_7192	4.00	lobChIP_FOXA1_robot
chr19	8439279	8439280	MACS_peak_7193	20.00	lobChIP_FOXA1_robot
chr19	8453152	8453153	MACS_peak_7194	25.00	lobChIP_FOXA1_robot
chr19	8487027	8487028	MACS_peak_7195	7.00	lobChIP_FOXA1_robot
chr19	8524868	8524869	MACS_peak_7196	6.00	lobChIP_FOXA1_robot
chr19	8741105	8741106	MACS_peak_7197	12.00	lobChIP_FOXA1_robot
chr19	8749836	8749837	MACS_peak_7198	4.00	lobChIP_FOXA1_robot
chr19	8750165	8750166	MACS_peak_7199	3.00	lobChIP_FOXA1_robot
chr19	9681057	9681058	MACS_peak_7200	5.00	lobChIP_FOXA1_robot
chr19	9982522	9982523	MACS_peak_7201	7.00	lobChIP_FOXA1_robot
chr19	10302236	10302237	MACS_peak_7202	5.00	lobChIP_FOXA1_robot
chr19	10349268	10349269	MACS_peak_7203	8.00	lobChIP_FOXA1_robot
chr19	10444677	10444678	MACS_peak_7204	4.00	lobChIP_FOXA1_robot
chr19	10952209	10952210	MACS_peak_7205	4.00	lobChIP_FOXA1_robot
chr19	11199435	11199436	MACS_peak_7206	11.00	lobChIP_FOXA1_robot
chr19	11202940	11202941	MACS_peak_7207	5.00	lobChIP_FOXA1_robot
chr19	11205970	11205971	MACS_peak_7208	6.00	lobChIP_FOXA1_robot
chr19	11376591	11376592	MACS_peak_7209	12.00	lobChIP_FOXA1_robot
chr19	11455177	11455178	MACS_peak_7210	6.00	lobChIP_FOXA1_robot
chr19	11601709	11601710	MACS_peak_7211	4.00	lobChIP_FOXA1_robot
chr19	11670158	11670159	MACS_peak_7212	9.00	lobChIP_FOXA1_robot
chr19	11999217	11999218	MACS_peak_7213	5.00	lobChIP_FOXA1_robot
chr19	12034557	12034558	MACS_peak_7214	12.00	lobChIP_FOXA1_robot
chr19	12126726	12126727	MACS_peak_7215	6.00	lobChIP_FOXA1_robot
chr19	12223309	12223310	MACS_peak_7216	11.00	lobChIP_FOXA1_robot
chr19	12319303	12319304	MACS_peak_7217	5.00	lobChIP_FOXA1_robot
chr19	12401953	12401954	MACS_peak_7218	3.00	lobChIP_FOXA1_robot
chr19	12436750	12436751	MACS_peak_7219	19.00	lobChIP_FOXA1_robot
chr19	12441767	12441768	MACS_peak_7220	4.00	lobChIP_FOXA1_robot
chr19	12442269	12442270	MACS_peak_7221	5.00	lobChIP_FOXA1_robot
chr19	12512250	12512251	MACS_peak_7222	5.00	lobChIP_FOXA1_robot
chr19	12894990	12894991	MACS_peak_7223	4.00	lobChIP_FOXA1_robot
chr19	12902120	12902121	MACS_peak_7224	4.00	lobChIP_FOXA1_robot
chr19	13137470	13137471	MACS_peak_7225	19.00	lobChIP_FOXA1_robot
chr19	13162567	13162568	MACS_peak_7226	7.00	lobChIP_FOXA1_robot
chr19	13958250	13958251	MACS_peak_7227	3.00	lobChIP_FOXA1_robot
chr19	13963448	13963449	MACS_peak_7228	5.00	lobChIP_FOXA1_robot
chr19	14131032	14131033	MACS_peak_7229	5.00	lobChIP_FOXA1_robot
chr19	14623959	14623960	MACS_peak_7230	6.00	lobChIP_FOXA1_robot
chr19	14860266	14860267	MACS_peak_7231	6.00	lobChIP_FOXA1_robot
chr19	15408407	15408408	MACS_peak_7232	21.00	lobChIP_FOXA1_robot
chr19	15752093	15752094	MACS_peak_7233	6.00	lobChIP_FOXA1_robot
chr19	15784161	15784162	MACS_peak_7234	8.00	lobChIP_FOXA1_robot
chr19	16190157	16190158	MACS_peak_7235	5.00	lobChIP_FOXA1_robot
chr19	16204647	16204648	MACS_peak_7236	5.00	lobChIP_FOXA1_robot
chr19	16205925	16205926	MACS_peak_7237	4.00	lobChIP_FOXA1_robot
chr19	16278108	16278109	MACS_peak_7238	5.00	lobChIP_FOXA1_robot
chr19	16373618	16373619	MACS_peak_7239	4.00	lobChIP_FOXA1_robot
chr19	16599933	16599934	MACS_peak_7240	5.00	lobChIP_FOXA1_robot
chr19	16603863	16603864	MACS_peak_7241	5.00	lobChIP_FOXA1_robot
chr19	17501889	17501890	MACS_peak_7242	4.00	lobChIP_FOXA1_robot
chr19	17876019	17876020	MACS_peak_7243	6.00	lobChIP_FOXA1_robot
chr19	17908534	17908535	MACS_peak_7244	5.00	lobChIP_FOXA1_robot
chr19	18140522	18140523	MACS_peak_7245	6.00	lobChIP_FOXA1_robot
chr19	18430730	18430731	MACS_peak_7246	7.00	lobChIP_FOXA1_robot
chr19	18473857	18473858	MACS_peak_7247	5.00	lobChIP_FOXA1_robot
chr19	18491053	18491054	MACS_peak_7248	21.00	lobChIP_FOXA1_robot
chr19	18492924	18492925	MACS_peak_7249	7.00	lobChIP_FOXA1_robot
chr19	18496878	18496879	MACS_peak_7250	11.00	lobChIP_FOXA1_robot
chr19	18586680	18586681	MACS_peak_7251	5.00	lobChIP_FOXA1_robot
chr19	18625852	18625853	MACS_peak_7252	13.00	lobChIP_FOXA1_robot
chr19	18844297	18844298	MACS_peak_7253	4.00	lobChIP_FOXA1_robot
chr19	19977719	19977720	MACS_peak_7254	5.00	lobChIP_FOXA1_robot
chr19	21006347	21006348	MACS_peak_7255	4.00	lobChIP_FOXA1_robot
chr19	21393040	21393041	MACS_peak_7256	24.00	lobChIP_FOXA1_robot
chr19	23586504	23586505	MACS_peak_7257	6.00	lobChIP_FOXA1_robot
chr19	24065487	24065488	MACS_peak_7258	5.00	lobChIP_FOXA1_robot
chr19	24323012	24323013	MACS_peak_7259	7.00	lobChIP_FOXA1_robot
chr19	24324500	24324501	MACS_peak_7260	4.00	lobChIP_FOXA1_robot
chr19	30296141	30296142	MACS_peak_7264	4.00	lobChIP_FOXA1_robot
chr19	30302442	30302443	MACS_peak_7265	5.00	lobChIP_FOXA1_robot
chr19	30639046	30639047	MACS_peak_7266	6.00	lobChIP_FOXA1_robot
chr19	32625801	32625802	MACS_peak_7267	4.00	lobChIP_FOXA1_robot
chr19	32841608	32841609	MACS_peak_7268	6.00	lobChIP_FOXA1_robot
chr19	32864873	32864874	MACS_peak_7269	19.00	lobChIP_FOXA1_robot
chr19	32994326	32994327	MACS_peak_7270	6.00	lobChIP_FOXA1_robot
chr19	33030424	33030425	MACS_peak_7271	9.00	lobChIP_FOXA1_robot
chr19	33161051	33161052	MACS_peak_7272	6.00	lobChIP_FOXA1_robot
chr19	33193132	33193133	MACS_peak_7273	48.00	lobChIP_FOXA1_robot
chr19	33516548	33516549	MACS_peak_7274	7.00	lobChIP_FOXA1_robot
chr19	33532350	33532351	MACS_peak_7275	5.00	lobChIP_FOXA1_robot
chr19	33592285	33592286	MACS_peak_7276	10.00	lobChIP_FOXA1_robot
chr19	33767124	33767125	MACS_peak_7277	13.00	lobChIP_FOXA1_robot
chr19	33784507	33784508	MACS_peak_7278	8.00	lobChIP_FOXA1_robot
chr19	33888234	33888235	MACS_peak_7279	14.00	lobChIP_FOXA1_robot
chr19	33894718	33894719	MACS_peak_7280	12.00	lobChIP_FOXA1_robot
chr19	34438003	34438004	MACS_peak_7281	4.00	lobChIP_FOXA1_robot
chr19	34711561	34711562	MACS_peak_7282	6.00	lobChIP_FOXA1_robot
chr19	34766133	34766134	MACS_peak_7283	4.00	lobChIP_FOXA1_robot
chr19	34893199	34893200	MACS_peak_7284	12.00	lobChIP_FOXA1_robot
chr19	35016193	35016194	MACS_peak_7285	5.00	lobChIP_FOXA1_robot
chr19	35236378	35236379	MACS_peak_7286	5.00	lobChIP_FOXA1_robot
chr19	35486037	35486038	MACS_peak_7287	26.00	lobChIP_FOXA1_robot
chr19	35497435	35497436	MACS_peak_7288	48.00	lobChIP_FOXA1_robot
chr19	35628452	35628453	MACS_peak_7289	5.00	lobChIP_FOXA1_robot
chr19	35739657	35739658	MACS_peak_7290	4.00	lobChIP_FOXA1_robot
chr19	35871579	35871580	MACS_peak_7291	4.00	lobChIP_FOXA1_robot
chr19	35880714	35880715	MACS_peak_7292	4.00	lobChIP_FOXA1_robot
chr19	35976621	35976622	MACS_peak_7293	8.00	lobChIP_FOXA1_robot
chr19	35977503	35977504	MACS_peak_7294	8.00	lobChIP_FOXA1_robot
chr19	36152501	36152502	MACS_peak_7295	6.00	lobChIP_FOXA1_robot
chr19	36310945	36310946	MACS_peak_7296	6.00	lobChIP_FOXA1_robot
chr19	36611131	36611132	MACS_peak_7297	7.00	lobChIP_FOXA1_robot
chr19	37062815	37062816	MACS_peak_7298	6.00	lobChIP_FOXA1_robot
chr19	38270806	38270807	MACS_peak_7299	3.00	lobChIP_FOXA1_robot
chr19	38365655	38365656	MACS_peak_7300	4.00	lobChIP_FOXA1_robot
chr19	38432734	38432735	MACS_peak_7301	5.00	lobChIP_FOXA1_robot
chr19	38442014	38442015	MACS_peak_7302	6.00	lobChIP_FOXA1_robot
chr19	38570922	38570923	MACS_peak_7303	6.00	lobChIP_FOXA1_robot
chr19	38741503	38741504	MACS_peak_7304	10.00	lobChIP_FOXA1_robot
chr19	39142865	39142866	MACS_peak_7305	5.00	lobChIP_FOXA1_robot
chr19	39144103	39144104	MACS_peak_7306	9.00	lobChIP_FOXA1_robot
chr19	39147227	39147228	MACS_peak_7307	4.00	lobChIP_FOXA1_robot
chr19	39149499	39149500	MACS_peak_7308	6.00	lobChIP_FOXA1_robot
chr19	39150348	39150349	MACS_peak_7309	5.00	lobChIP_FOXA1_robot
chr19	39174427	39174428	MACS_peak_7310	4.00	lobChIP_FOXA1_robot
chr19	39184092	39184093	MACS_peak_7311	5.00	lobChIP_FOXA1_robot
chr19	39892308	39892309	MACS_peak_7312	3.00	lobChIP_FOXA1_robot
chr19	40186568	40186569	MACS_peak_7313	10.00	lobChIP_FOXA1_robot
chr19	40450129	40450130	MACS_peak_7314	4.00	lobChIP_FOXA1_robot
chr19	40591265	40591266	MACS_peak_7315	5.00	lobChIP_FOXA1_robot
chr19	40616635	40616636	MACS_peak_7316	5.00	lobChIP_FOXA1_robot
chr19	40625531	40625532	MACS_peak_7317	14.00	lobChIP_FOXA1_robot
chr19	40732928	40732929	MACS_peak_7318	7.00	lobChIP_FOXA1_robot
chr19	41109850	41109851	MACS_peak_7319	4.00	lobChIP_FOXA1_robot
chr19	41427324	41427325	MACS_peak_7320	19.00	lobChIP_FOXA1_robot
chr19	41673221	41673222	MACS_peak_7321	6.00	lobChIP_FOXA1_robot
chr19	42392118	42392119	MACS_peak_7322	6.00	lobChIP_FOXA1_robot
chr19	42408722	42408723	MACS_peak_7323	20.00	lobChIP_FOXA1_robot
chr19	42948189	42948190	MACS_peak_7324	10.00	lobChIP_FOXA1_robot
chr19	42985125	42985126	MACS_peak_7325	9.00	lobChIP_FOXA1_robot
chr19	42993346	42993347	MACS_peak_7326	28.00	lobChIP_FOXA1_robot
chr19	43002038	43002039	MACS_peak_7327	4.00	lobChIP_FOXA1_robot
chr19	43943411	43943412	MACS_peak_7328	5.00	lobChIP_FOXA1_robot
chr19	44030166	44030167	MACS_peak_7329	5.00	lobChIP_FOXA1_robot
chr19	44462248	44462249	MACS_peak_7330	5.00	lobChIP_FOXA1_robot
chr19	44758819	44758820	MACS_peak_7331	3.00	lobChIP_FOXA1_robot
chr19	45100383	45100384	MACS_peak_7332	5.00	lobChIP_FOXA1_robot
chr19	45115306	45115307	MACS_peak_7333	5.00	lobChIP_FOXA1_robot
chr19	45119087	45119088	MACS_peak_7334	5.00	lobChIP_FOXA1_robot
chr19	45140119	45140120	MACS_peak_7335	5.00	lobChIP_FOXA1_robot
chr19	45198242	45198243	MACS_peak_7336	34.00	lobChIP_FOXA1_robot
chr19	45198829	45198830	MACS_peak_7337	4.00	lobChIP_FOXA1_robot
chr19	45222069	45222070	MACS_peak_7338	5.00	lobChIP_FOXA1_robot
chr19	45300096	45300097	MACS_peak_7339	14.00	lobChIP_FOXA1_robot
chr19	45360119	45360120	MACS_peak_7340	4.00	lobChIP_FOXA1_robot
chr19	45362586	45362587	MACS_peak_7341	5.00	lobChIP_FOXA1_robot
chr19	45372310	45372311	MACS_peak_7342	6.00	lobChIP_FOXA1_robot
chr19	45427693	45427694	MACS_peak_7343	24.00	lobChIP_FOXA1_robot
chr19	45428877	45428878	MACS_peak_7344	4.00	lobChIP_FOXA1_robot
chr19	45439297	45439298	MACS_peak_7345	10.00	lobChIP_FOXA1_robot
chr19	45549079	45549080	MACS_peak_7346	18.00	lobChIP_FOXA1_robot
chr19	45776457	45776458	MACS_peak_7347	19.00	lobChIP_FOXA1_robot
chr19	45779839	45779840	MACS_peak_7348	5.00	lobChIP_FOXA1_robot
chr19	46008407	46008408	MACS_peak_7349	5.00	lobChIP_FOXA1_robot
chr19	46009683	46009684	MACS_peak_7350	3.00	lobChIP_FOXA1_robot
chr19	46143377	46143378	MACS_peak_7351	7.00	lobChIP_FOXA1_robot
chr19	46161286	46161287	MACS_peak_7352	6.00	lobChIP_FOXA1_robot
chr19	46377528	46377529	MACS_peak_7353	3.00	lobChIP_FOXA1_robot
chr19	46424551	46424552	MACS_peak_7354	3.00	lobChIP_FOXA1_robot
chr19	46696569	46696570	MACS_peak_7355	11.00	lobChIP_FOXA1_robot
chr19	47274352	47274353	MACS_peak_7356	11.00	lobChIP_FOXA1_robot
chr19	47370117	47370118	MACS_peak_7357	5.00	lobChIP_FOXA1_robot
chr19	47394240	47394241	MACS_peak_7358	8.00	lobChIP_FOXA1_robot
chr19	47439658	47439659	MACS_peak_7359	4.00	lobChIP_FOXA1_robot
chr19	47457988	47457989	MACS_peak_7360	5.00	lobChIP_FOXA1_robot
chr19	47487910	47487911	MACS_peak_7361	23.00	lobChIP_FOXA1_robot
chr19	47599484	47599485	MACS_peak_7362	9.00	lobChIP_FOXA1_robot
chr19	47693742	47693743	MACS_peak_7363	8.00	lobChIP_FOXA1_robot
chr19	47754090	47754091	MACS_peak_7364	5.00	lobChIP_FOXA1_robot
chr19	48052876	48052877	MACS_peak_7365	5.00	lobChIP_FOXA1_robot
chr19	48166197	48166198	MACS_peak_7366	4.00	lobChIP_FOXA1_robot
chr19	48167356	48167357	MACS_peak_7367	4.00	lobChIP_FOXA1_robot
chr19	48760074	48760075	MACS_peak_7368	5.00	lobChIP_FOXA1_robot
chr19	48857954	48857955	MACS_peak_7369	12.00	lobChIP_FOXA1_robot
chr19	49068594	49068595	MACS_peak_7370	9.00	lobChIP_FOXA1_robot
chr19	49275321	49275322	MACS_peak_7371	4.00	lobChIP_FOXA1_robot
chr19	49376376	49376377	MACS_peak_7372	8.00	lobChIP_FOXA1_robot
chr19	49955087	49955088	MACS_peak_7373	7.00	lobChIP_FOXA1_robot
chr19	49996115	49996116	MACS_peak_7374	7.00	lobChIP_FOXA1_robot
chr19	50380722	50380723	MACS_peak_7375	5.00	lobChIP_FOXA1_robot
chr19	50737811	50737812	MACS_peak_7376	15.00	lobChIP_FOXA1_robot
chr19	50963940	50963941	MACS_peak_7377	5.00	lobChIP_FOXA1_robot
chr19	51858325	51858326	MACS_peak_7378	5.00	lobChIP_FOXA1_robot
chr19	51866160	51866161	MACS_peak_7379	27.00	lobChIP_FOXA1_robot
chr19	51934547	51934548	MACS_peak_7380	4.00	lobChIP_FOXA1_robot
chr19	52461094	52461095	MACS_peak_7381	4.00	lobChIP_FOXA1_robot
chr19	52514128	52514129	MACS_peak_7382	6.00	lobChIP_FOXA1_robot
chr19	52527929	52527930	MACS_peak_7383	5.00	lobChIP_FOXA1_robot
chr19	53206232	53206233	MACS_peak_7384	4.00	lobChIP_FOXA1_robot
chr19	54000875	54000876	MACS_peak_7385	6.00	lobChIP_FOXA1_robot
chr19	54034969	54034970	MACS_peak_7386	5.00	lobChIP_FOXA1_robot
chr19	54038981	54038982	MACS_peak_7387	7.00	lobChIP_FOXA1_robot
chr19	54083029	54083030	MACS_peak_7388	5.00	lobChIP_FOXA1_robot
chr19	54123234	54123235	MACS_peak_7389	23.00	lobChIP_FOXA1_robot
chr19	54279023	54279024	MACS_peak_7390	7.00	lobChIP_FOXA1_robot
chr19	54312075	54312076	MACS_peak_7391	4.00	lobChIP_FOXA1_robot
chr19	54367856	54367857	MACS_peak_7392	30.00	lobChIP_FOXA1_robot
chr19	54713952	54713953	MACS_peak_7393	8.00	lobChIP_FOXA1_robot
chr19	55185687	55185688	MACS_peak_7394	8.00	lobChIP_FOXA1_robot
chr19	55721636	55721637	MACS_peak_7395	7.00	lobChIP_FOXA1_robot
chr19	56037942	56037943	MACS_peak_7396	3.00	lobChIP_FOXA1_robot
chr19	56060850	56060851	MACS_peak_7397	12.00	lobChIP_FOXA1_robot
chr19	56064802	56064803	MACS_peak_7398	5.00	lobChIP_FOXA1_robot
chr19	56092951	56092952	MACS_peak_7399	3.00	lobChIP_FOXA1_robot
chr19	56649601	56649602	MACS_peak_7400	6.00	lobChIP_FOXA1_robot
chr19	56653759	56653760	MACS_peak_7401	7.00	lobChIP_FOXA1_robot
chr19	56867006	56867007	MACS_peak_7402	7.00	lobChIP_FOXA1_robot
chr19	57412113	57412114	MACS_peak_7403	5.00	lobChIP_FOXA1_robot
chr19	57430192	57430193	MACS_peak_7404	10.00	lobChIP_FOXA1_robot
chr19	57438917	57438918	MACS_peak_7405	16.00	lobChIP_FOXA1_robot
chr19	57442396	57442397	MACS_peak_7406	24.00	lobChIP_FOXA1_robot
chr19	57446313	57446314	MACS_peak_7407	24.00	lobChIP_FOXA1_robot
chr19	57467090	57467091	MACS_peak_7408	3.00	lobChIP_FOXA1_robot
chr19	57487886	57487887	MACS_peak_7409	10.00	lobChIP_FOXA1_robot
chr19	57538821	57538822	MACS_peak_7410	3.00	lobChIP_FOXA1_robot
chr19	57587665	57587666	MACS_peak_7411	5.00	lobChIP_FOXA1_robot
chr19	58385984	58385985	MACS_peak_7412	5.00	lobChIP_FOXA1_robot
chr2	176086	176087	MACS_peak_7413	14.00	lobChIP_FOXA1_robot
chr2	246875	246876	MACS_peak_7414	4.00	lobChIP_FOXA1_robot
chr2	249935	249936	MACS_peak_7415	5.00	lobChIP_FOXA1_robot
chr2	260847	260848	MACS_peak_7416	47.00	lobChIP_FOXA1_robot
chr2	263067	263068	MACS_peak_7417	6.00	lobChIP_FOXA1_robot
chr2	279564	279565	MACS_peak_7418	3.00	lobChIP_FOXA1_robot
chr2	378504	378505	MACS_peak_7419	7.00	lobChIP_FOXA1_robot
chr2	1242029	1242030	MACS_peak_7420	4.00	lobChIP_FOXA1_robot
chr2	2395183	2395184	MACS_peak_7421	4.00	lobChIP_FOXA1_robot
chr2	2447609	2447610	MACS_peak_7422	3.00	lobChIP_FOXA1_robot
chr2	2540339	2540340	MACS_peak_7423	5.00	lobChIP_FOXA1_robot
chr2	2905833	2905834	MACS_peak_7424	4.00	lobChIP_FOXA1_robot
chr2	3384136	3384137	MACS_peak_7425	8.00	lobChIP_FOXA1_robot
chr2	3427952	3427953	MACS_peak_7426	6.00	lobChIP_FOXA1_robot
chr2	3469615	3469616	MACS_peak_7427	5.00	lobChIP_FOXA1_robot
chr2	3508789	3508790	MACS_peak_7428	6.00	lobChIP_FOXA1_robot
chr2	3521961	3521962	MACS_peak_7429	8.00	lobChIP_FOXA1_robot
chr2	3527829	3527830	MACS_peak_7430	3.00	lobChIP_FOXA1_robot
chr2	3633480	3633481	MACS_peak_7431	9.00	lobChIP_FOXA1_robot
chr2	3668723	3668724	MACS_peak_7432	27.00	lobChIP_FOXA1_robot
chr2	3704734	3704735	MACS_peak_7433	6.00	lobChIP_FOXA1_robot
chr2	4315718	4315719	MACS_peak_7434	4.00	lobChIP_FOXA1_robot
chr2	5483029	5483030	MACS_peak_7435	8.00	lobChIP_FOXA1_robot
chr2	6084264	6084265	MACS_peak_7436	5.00	lobChIP_FOXA1_robot
chr2	7069286	7069287	MACS_peak_7437	5.00	lobChIP_FOXA1_robot
chr2	7172054	7172055	MACS_peak_7438	11.00	lobChIP_FOXA1_robot
chr2	7966041	7966042	MACS_peak_7439	7.00	lobChIP_FOXA1_robot
chr2	8040077	8040078	MACS_peak_7440	3.00	lobChIP_FOXA1_robot
chr2	8241981	8241982	MACS_peak_7441	3.00	lobChIP_FOXA1_robot
chr2	8243428	8243429	MACS_peak_7442	33.00	lobChIP_FOXA1_robot
chr2	8259951	8259952	MACS_peak_7443	9.00	lobChIP_FOXA1_robot
chr2	8262838	8262839	MACS_peak_7444	4.00	lobChIP_FOXA1_robot
chr2	8266128	8266129	MACS_peak_7445	6.00	lobChIP_FOXA1_robot
chr2	8274816	8274817	MACS_peak_7446	4.00	lobChIP_FOXA1_robot
chr2	8279396	8279397	MACS_peak_7447	24.00	lobChIP_FOXA1_robot
chr2	8518610	8518611	MACS_peak_7448	5.00	lobChIP_FOXA1_robot
chr2	8554130	8554131	MACS_peak_7449	3.00	lobChIP_FOXA1_robot
chr2	8633669	8633670	MACS_peak_7450	4.00	lobChIP_FOXA1_robot
chr2	8640929	8640930	MACS_peak_7451	4.00	lobChIP_FOXA1_robot
chr2	8641677	8641678	MACS_peak_7452	4.00	lobChIP_FOXA1_robot
chr2	8672050	8672051	MACS_peak_7453	4.00	lobChIP_FOXA1_robot
chr2	8712366	8712367	MACS_peak_7454	6.00	lobChIP_FOXA1_robot
chr2	8751764	8751765	MACS_peak_7455	4.00	lobChIP_FOXA1_robot
chr2	8753147	8753148	MACS_peak_7456	8.00	lobChIP_FOXA1_robot
chr2	8765658	8765659	MACS_peak_7457	5.00	lobChIP_FOXA1_robot
chr2	8767375	8767376	MACS_peak_7458	5.00	lobChIP_FOXA1_robot
chr2	8796566	8796567	MACS_peak_7459	15.00	lobChIP_FOXA1_robot
chr2	8817774	8817775	MACS_peak_7460	13.00	lobChIP_FOXA1_robot
chr2	8820769	8820770	MACS_peak_7461	7.00	lobChIP_FOXA1_robot
chr2	8944330	8944331	MACS_peak_7462	7.00	lobChIP_FOXA1_robot
chr2	8960548	8960549	MACS_peak_7463	4.00	lobChIP_FOXA1_robot
chr2	8980805	8980806	MACS_peak_7464	11.00	lobChIP_FOXA1_robot
chr2	9102983	9102984	MACS_peak_7465	5.00	lobChIP_FOXA1_robot
chr2	9308474	9308475	MACS_peak_7466	5.00	lobChIP_FOXA1_robot
chr2	9319446	9319447	MACS_peak_7467	5.00	lobChIP_FOXA1_robot
chr2	9350339	9350340	MACS_peak_7468	4.00	lobChIP_FOXA1_robot
chr2	9372333	9372334	MACS_peak_7469	6.00	lobChIP_FOXA1_robot
chr2	9468083	9468084	MACS_peak_7470	14.00	lobChIP_FOXA1_robot
chr2	9477123	9477124	MACS_peak_7471	7.00	lobChIP_FOXA1_robot
chr2	9521353	9521354	MACS_peak_7472	5.00	lobChIP_FOXA1_robot
chr2	10030009	10030010	MACS_peak_7473	6.00	lobChIP_FOXA1_robot
chr2	10087507	10087508	MACS_peak_7474	3.00	lobChIP_FOXA1_robot
chr2	10115545	10115546	MACS_peak_7475	4.00	lobChIP_FOXA1_robot
chr2	10159469	10159470	MACS_peak_7476	5.00	lobChIP_FOXA1_robot
chr2	10260647	10260648	MACS_peak_7477	7.00	lobChIP_FOXA1_robot
chr2	10369014	10369015	MACS_peak_7478	5.00	lobChIP_FOXA1_robot
chr2	10478597	10478598	MACS_peak_7479	4.00	lobChIP_FOXA1_robot
chr2	10506209	10506210	MACS_peak_7480	9.00	lobChIP_FOXA1_robot
chr2	10508801	10508802	MACS_peak_7481	6.00	lobChIP_FOXA1_robot
chr2	10606217	10606218	MACS_peak_7482	5.00	lobChIP_FOXA1_robot
chr2	10627456	10627457	MACS_peak_7483	22.00	lobChIP_FOXA1_robot
chr2	10724816	10724817	MACS_peak_7484	83.00	lobChIP_FOXA1_robot
chr2	11076491	11076492	MACS_peak_7485	17.00	lobChIP_FOXA1_robot
chr2	11199901	11199902	MACS_peak_7486	4.00	lobChIP_FOXA1_robot
chr2	11309537	11309538	MACS_peak_7487	11.00	lobChIP_FOXA1_robot
chr2	11394321	11394322	MACS_peak_7488	8.00	lobChIP_FOXA1_robot
chr2	11480853	11480854	MACS_peak_7489	5.00	lobChIP_FOXA1_robot
chr2	11483016	11483017	MACS_peak_7490	14.00	lobChIP_FOXA1_robot
chr2	11494635	11494636	MACS_peak_7491	69.00	lobChIP_FOXA1_robot
chr2	11497681	11497682	MACS_peak_7492	12.00	lobChIP_FOXA1_robot
chr2	11506572	11506573	MACS_peak_7493	3.00	lobChIP_FOXA1_robot
chr2	11510992	11510993	MACS_peak_7494	8.00	lobChIP_FOXA1_robot
chr2	11515829	11515830	MACS_peak_7495	3.00	lobChIP_FOXA1_robot
chr2	11520921	11520922	MACS_peak_7496	7.00	lobChIP_FOXA1_robot
chr2	11528105	11528106	MACS_peak_7497	57.00	lobChIP_FOXA1_robot
chr2	11629096	11629097	MACS_peak_7498	5.00	lobChIP_FOXA1_robot
chr2	11639561	11639562	MACS_peak_7499	9.00	lobChIP_FOXA1_robot
chr2	11646170	11646171	MACS_peak_7500	16.00	lobChIP_FOXA1_robot
chr2	11673655	11673656	MACS_peak_7501	7.00	lobChIP_FOXA1_robot
chr2	11679625	11679626	MACS_peak_7502	5.00	lobChIP_FOXA1_robot
chr2	11680321	11680322	MACS_peak_7503	3.00	lobChIP_FOXA1_robot
chr2	11684221	11684222	MACS_peak_7504	5.00	lobChIP_FOXA1_robot
chr2	11723518	11723519	MACS_peak_7505	6.00	lobChIP_FOXA1_robot
chr2	11857025	11857026	MACS_peak_7506	4.00	lobChIP_FOXA1_robot
chr2	11959544	11959545	MACS_peak_7507	15.00	lobChIP_FOXA1_robot
chr2	11963061	11963062	MACS_peak_7508	7.00	lobChIP_FOXA1_robot
chr2	11969725	11969726	MACS_peak_7509	7.00	lobChIP_FOXA1_robot
chr2	11970876	11970877	MACS_peak_7510	13.00	lobChIP_FOXA1_robot
chr2	12596811	12596812	MACS_peak_7511	4.00	lobChIP_FOXA1_robot
chr2	12655016	12655017	MACS_peak_7512	7.00	lobChIP_FOXA1_robot
chr2	13170281	13170282	MACS_peak_7513	6.00	lobChIP_FOXA1_robot
chr2	13211805	13211806	MACS_peak_7514	3.00	lobChIP_FOXA1_robot
chr2	13834231	13834232	MACS_peak_7515	9.00	lobChIP_FOXA1_robot
chr2	14441644	14441645	MACS_peak_7516	3.00	lobChIP_FOXA1_robot
chr2	14834274	14834275	MACS_peak_7517	6.00	lobChIP_FOXA1_robot
chr2	15313166	15313167	MACS_peak_7518	9.00	lobChIP_FOXA1_robot
chr2	15500536	15500537	MACS_peak_7519	7.00	lobChIP_FOXA1_robot
chr2	15662841	15662842	MACS_peak_7520	4.00	lobChIP_FOXA1_robot
chr2	15765210	15765211	MACS_peak_7521	5.00	lobChIP_FOXA1_robot
chr2	15769432	15769433	MACS_peak_7522	6.00	lobChIP_FOXA1_robot
chr2	15781988	15781989	MACS_peak_7523	14.00	lobChIP_FOXA1_robot
chr2	15874092	15874093	MACS_peak_7524	5.00	lobChIP_FOXA1_robot
chr2	15906672	15906673	MACS_peak_7525	32.00	lobChIP_FOXA1_robot
chr2	15916845	15916846	MACS_peak_7526	21.00	lobChIP_FOXA1_robot
chr2	15922388	15922389	MACS_peak_7527	16.00	lobChIP_FOXA1_robot
chr2	16208465	16208466	MACS_peak_7528	10.00	lobChIP_FOXA1_robot
chr2	16210596	16210597	MACS_peak_7529	15.00	lobChIP_FOXA1_robot
chr2	16244630	16244631	MACS_peak_7530	15.00	lobChIP_FOXA1_robot
chr2	16440450	16440451	MACS_peak_7531	5.00	lobChIP_FOXA1_robot
chr2	16512017	16512018	MACS_peak_7532	6.00	lobChIP_FOXA1_robot
chr2	16574692	16574693	MACS_peak_7533	15.00	lobChIP_FOXA1_robot
chr2	16607197	16607198	MACS_peak_7534	34.00	lobChIP_FOXA1_robot
chr2	16607952	16607953	MACS_peak_7535	3.00	lobChIP_FOXA1_robot
chr2	16790459	16790460	MACS_peak_7536	4.00	lobChIP_FOXA1_robot
chr2	16999313	16999314	MACS_peak_7537	7.00	lobChIP_FOXA1_robot
chr2	17389896	17389897	MACS_peak_7538	5.00	lobChIP_FOXA1_robot
chr2	17660596	17660597	MACS_peak_7539	3.00	lobChIP_FOXA1_robot
chr2	17661238	17661239	MACS_peak_7540	20.00	lobChIP_FOXA1_robot
chr2	17723980	17723981	MACS_peak_7541	4.00	lobChIP_FOXA1_robot
chr2	17727660	17727661	MACS_peak_7542	13.00	lobChIP_FOXA1_robot
chr2	17758087	17758088	MACS_peak_7543	4.00	lobChIP_FOXA1_robot
chr2	17760049	17760050	MACS_peak_7544	6.00	lobChIP_FOXA1_robot
chr2	17761081	17761082	MACS_peak_7545	16.00	lobChIP_FOXA1_robot
chr2	17774573	17774574	MACS_peak_7546	4.00	lobChIP_FOXA1_robot
chr2	17775323	17775324	MACS_peak_7547	37.00	lobChIP_FOXA1_robot
chr2	17949696	17949697	MACS_peak_7548	5.00	lobChIP_FOXA1_robot
chr2	17962904	17962905	MACS_peak_7549	7.00	lobChIP_FOXA1_robot
chr2	18327129	18327130	MACS_peak_7550	4.00	lobChIP_FOXA1_robot
chr2	18414479	18414480	MACS_peak_7551	7.00	lobChIP_FOXA1_robot
chr2	18690131	18690132	MACS_peak_7552	3.00	lobChIP_FOXA1_robot
chr2	18747635	18747636	MACS_peak_7553	4.00	lobChIP_FOXA1_robot
chr2	18944156	18944157	MACS_peak_7554	4.00	lobChIP_FOXA1_robot
chr2	18981815	18981816	MACS_peak_7555	129.00	lobChIP_FOXA1_robot
chr2	19070991	19070992	MACS_peak_7556	15.00	lobChIP_FOXA1_robot
chr2	19248800	19248801	MACS_peak_7557	8.00	lobChIP_FOXA1_robot
chr2	19340695	19340696	MACS_peak_7558	24.00	lobChIP_FOXA1_robot
chr2	19688327	19688328	MACS_peak_7559	4.00	lobChIP_FOXA1_robot
chr2	19705490	19705491	MACS_peak_7560	27.00	lobChIP_FOXA1_robot
chr2	19769221	19769222	MACS_peak_7561	9.00	lobChIP_FOXA1_robot
chr2	20100053	20100054	MACS_peak_7562	8.00	lobChIP_FOXA1_robot
chr2	20101962	20101963	MACS_peak_7563	5.00	lobChIP_FOXA1_robot
chr2	20136163	20136164	MACS_peak_7564	3.00	lobChIP_FOXA1_robot
chr2	20205248	20205249	MACS_peak_7565	4.00	lobChIP_FOXA1_robot
chr2	20208605	20208606	MACS_peak_7566	10.00	lobChIP_FOXA1_robot
chr2	20213097	20213098	MACS_peak_7567	6.00	lobChIP_FOXA1_robot
chr2	20298136	20298137	MACS_peak_7568	6.00	lobChIP_FOXA1_robot
chr2	20384066	20384067	MACS_peak_7569	6.00	lobChIP_FOXA1_robot
chr2	20395918	20395919	MACS_peak_7570	5.00	lobChIP_FOXA1_robot
chr2	20408059	20408060	MACS_peak_7571	6.00	lobChIP_FOXA1_robot
chr2	20617133	20617134	MACS_peak_7572	4.00	lobChIP_FOXA1_robot
chr2	20638461	20638462	MACS_peak_7573	15.00	lobChIP_FOXA1_robot
chr2	20643225	20643226	MACS_peak_7574	4.00	lobChIP_FOXA1_robot
chr2	20644184	20644185	MACS_peak_7575	3.00	lobChIP_FOXA1_robot
chr2	20650140	20650141	MACS_peak_7576	5.00	lobChIP_FOXA1_robot
chr2	20738611	20738612	MACS_peak_7577	4.00	lobChIP_FOXA1_robot
chr2	20779258	20779259	MACS_peak_7578	4.00	lobChIP_FOXA1_robot
chr2	20780553	20780554	MACS_peak_7579	14.00	lobChIP_FOXA1_robot
chr2	20785413	20785414	MACS_peak_7580	11.00	lobChIP_FOXA1_robot
chr2	20793367	20793368	MACS_peak_7581	46.00	lobChIP_FOXA1_robot
chr2	20796895	20796896	MACS_peak_7582	6.00	lobChIP_FOXA1_robot
chr2	20811319	20811320	MACS_peak_7583	11.00	lobChIP_FOXA1_robot
chr2	20928000	20928001	MACS_peak_7584	6.00	lobChIP_FOXA1_robot
chr2	20945953	20945954	MACS_peak_7585	4.00	lobChIP_FOXA1_robot
chr2	21135340	21135341	MACS_peak_7586	9.00	lobChIP_FOXA1_robot
chr2	21226091	21226092	MACS_peak_7587	5.00	lobChIP_FOXA1_robot
chr2	21235516	21235517	MACS_peak_7588	4.00	lobChIP_FOXA1_robot
chr2	21265113	21265114	MACS_peak_7589	11.00	lobChIP_FOXA1_robot
chr2	21266043	21266044	MACS_peak_7590	11.00	lobChIP_FOXA1_robot
chr2	21267047	21267048	MACS_peak_7591	8.00	lobChIP_FOXA1_robot
chr2	21270123	21270124	MACS_peak_7592	20.00	lobChIP_FOXA1_robot
chr2	21279382	21279383	MACS_peak_7593	43.00	lobChIP_FOXA1_robot
chr2	21293362	21293363	MACS_peak_7594	19.00	lobChIP_FOXA1_robot
chr2	21326593	21326594	MACS_peak_7595	14.00	lobChIP_FOXA1_robot
chr2	21378842	21378843	MACS_peak_7596	16.00	lobChIP_FOXA1_robot
chr2	21444046	21444047	MACS_peak_7597	4.00	lobChIP_FOXA1_robot
chr2	21645989	21645990	MACS_peak_7598	3.00	lobChIP_FOXA1_robot
chr2	22278894	22278895	MACS_peak_7599	21.00	lobChIP_FOXA1_robot
chr2	23582962	23582963	MACS_peak_7600	5.00	lobChIP_FOXA1_robot
chr2	24238571	24238572	MACS_peak_7601	10.00	lobChIP_FOXA1_robot
chr2	24274415	24274416	MACS_peak_7602	7.00	lobChIP_FOXA1_robot
chr2	24347153	24347154	MACS_peak_7603	4.00	lobChIP_FOXA1_robot
chr2	24381211	24381212	MACS_peak_7604	7.00	lobChIP_FOXA1_robot
chr2	24461257	24461258	MACS_peak_7605	4.00	lobChIP_FOXA1_robot
chr2	24917260	24917261	MACS_peak_7606	3.00	lobChIP_FOXA1_robot
chr2	24940587	24940588	MACS_peak_7607	7.00	lobChIP_FOXA1_robot
chr2	25675485	25675486	MACS_peak_7608	4.00	lobChIP_FOXA1_robot
chr2	25745119	25745120	MACS_peak_7609	12.00	lobChIP_FOXA1_robot
chr2	25841771	25841772	MACS_peak_7610	11.00	lobChIP_FOXA1_robot
chr2	25938452	25938453	MACS_peak_7611	4.00	lobChIP_FOXA1_robot
chr2	26010014	26010015	MACS_peak_7612	11.00	lobChIP_FOXA1_robot
chr2	26010500	26010501	MACS_peak_7613	4.00	lobChIP_FOXA1_robot
chr2	26031652	26031653	MACS_peak_7614	23.00	lobChIP_FOXA1_robot
chr2	26040141	26040142	MACS_peak_7615	6.00	lobChIP_FOXA1_robot
chr2	26052417	26052418	MACS_peak_7616	7.00	lobChIP_FOXA1_robot
chr2	26102092	26102093	MACS_peak_7617	9.00	lobChIP_FOXA1_robot
chr2	26106241	26106242	MACS_peak_7618	5.00	lobChIP_FOXA1_robot
chr2	26108972	26108973	MACS_peak_7619	6.00	lobChIP_FOXA1_robot
chr2	26214590	26214591	MACS_peak_7620	4.00	lobChIP_FOXA1_robot
chr2	26235224	26235225	MACS_peak_7621	12.00	lobChIP_FOXA1_robot
chr2	26299262	26299263	MACS_peak_7622	6.00	lobChIP_FOXA1_robot
chr2	26303436	26303437	MACS_peak_7623	10.00	lobChIP_FOXA1_robot
chr2	26461668	26461669	MACS_peak_7624	9.00	lobChIP_FOXA1_robot
chr2	26497112	26497113	MACS_peak_7625	4.00	lobChIP_FOXA1_robot
chr2	26853959	26853960	MACS_peak_7626	10.00	lobChIP_FOXA1_robot
chr2	26858746	26858747	MACS_peak_7627	4.00	lobChIP_FOXA1_robot
chr2	26944735	26944736	MACS_peak_7628	9.00	lobChIP_FOXA1_robot
chr2	27148529	27148530	MACS_peak_7629	4.00	lobChIP_FOXA1_robot
chr2	27318773	27318774	MACS_peak_7630	5.00	lobChIP_FOXA1_robot
chr2	27394562	27394563	MACS_peak_7631	13.00	lobChIP_FOXA1_robot
chr2	27400695	27400696	MACS_peak_7632	26.00	lobChIP_FOXA1_robot
chr2	27403767	27403768	MACS_peak_7633	6.00	lobChIP_FOXA1_robot
chr2	27417899	27417900	MACS_peak_7634	8.00	lobChIP_FOXA1_robot
chr2	27516148	27516149	MACS_peak_7635	17.00	lobChIP_FOXA1_robot
chr2	27517930	27517931	MACS_peak_7636	5.00	lobChIP_FOXA1_robot
chr2	27602667	27602668	MACS_peak_7637	5.00	lobChIP_FOXA1_robot
chr2	27948380	27948381	MACS_peak_7638	5.00	lobChIP_FOXA1_robot
chr2	27969321	27969322	MACS_peak_7639	7.00	lobChIP_FOXA1_robot
chr2	27986285	27986286	MACS_peak_7640	10.00	lobChIP_FOXA1_robot
chr2	28098531	28098532	MACS_peak_7641	19.00	lobChIP_FOXA1_robot
chr2	28138997	28138998	MACS_peak_7642	11.00	lobChIP_FOXA1_robot
chr2	28234504	28234505	MACS_peak_7643	6.00	lobChIP_FOXA1_robot
chr2	28257167	28257168	MACS_peak_7644	4.00	lobChIP_FOXA1_robot
chr2	28541488	28541489	MACS_peak_7645	5.00	lobChIP_FOXA1_robot
chr2	28542218	28542219	MACS_peak_7646	5.00	lobChIP_FOXA1_robot
chr2	28619674	28619675	MACS_peak_7647	7.00	lobChIP_FOXA1_robot
chr2	28631374	28631375	MACS_peak_7648	8.00	lobChIP_FOXA1_robot
chr2	28884680	28884681	MACS_peak_7649	6.00	lobChIP_FOXA1_robot
chr2	28974714	28974715	MACS_peak_7650	4.00	lobChIP_FOXA1_robot
chr2	28976377	28976378	MACS_peak_7651	10.00	lobChIP_FOXA1_robot
chr2	29096007	29096008	MACS_peak_7652	4.00	lobChIP_FOXA1_robot
chr2	29346694	29346695	MACS_peak_7653	4.00	lobChIP_FOXA1_robot
chr2	30156547	30156548	MACS_peak_7654	5.00	lobChIP_FOXA1_robot
chr2	30364271	30364272	MACS_peak_7655	3.00	lobChIP_FOXA1_robot
chr2	30676670	30676671	MACS_peak_7656	7.00	lobChIP_FOXA1_robot
chr2	30729032	30729033	MACS_peak_7657	4.00	lobChIP_FOXA1_robot
chr2	30908962	30908963	MACS_peak_7658	4.00	lobChIP_FOXA1_robot
chr2	31230293	31230294	MACS_peak_7659	3.00	lobChIP_FOXA1_robot
chr2	31783963	31783964	MACS_peak_7660	4.00	lobChIP_FOXA1_robot
chr2	32037508	32037509	MACS_peak_7661	15.00	lobChIP_FOXA1_robot
chr2	32150742	32150743	MACS_peak_7662	7.00	lobChIP_FOXA1_robot
chr2	32214655	32214656	MACS_peak_7663	3.00	lobChIP_FOXA1_robot
chr2	32218150	32218151	MACS_peak_7664	4.00	lobChIP_FOXA1_robot
chr2	32231552	32231553	MACS_peak_7665	9.00	lobChIP_FOXA1_robot
chr2	32457255	32457256	MACS_peak_7666	5.00	lobChIP_FOXA1_robot
chr2	32490727	32490728	MACS_peak_7667	4.00	lobChIP_FOXA1_robot
chr2	32502873	32502874	MACS_peak_7668	12.00	lobChIP_FOXA1_robot
chr2	32554419	32554420	MACS_peak_7669	16.00	lobChIP_FOXA1_robot
chr2	32682137	32682138	MACS_peak_7670	4.00	lobChIP_FOXA1_robot
chr2	32964526	32964527	MACS_peak_7671	4.00	lobChIP_FOXA1_robot
chr2	32969145	32969146	MACS_peak_7672	5.00	lobChIP_FOXA1_robot
chr2	32977513	32977514	MACS_peak_7673	3.00	lobChIP_FOXA1_robot
chr2	33002921	33002922	MACS_peak_7674	8.00	lobChIP_FOXA1_robot
chr2	33030467	33030468	MACS_peak_7675	6.00	lobChIP_FOXA1_robot
chr2	33500854	33500855	MACS_peak_7676	5.00	lobChIP_FOXA1_robot
chr2	33516569	33516570	MACS_peak_7677	8.00	lobChIP_FOXA1_robot
chr2	34578906	34578907	MACS_peak_7678	14.00	lobChIP_FOXA1_robot
chr2	34599105	34599106	MACS_peak_7679	9.00	lobChIP_FOXA1_robot
chr2	34998835	34998836	MACS_peak_7680	3.00	lobChIP_FOXA1_robot
chr2	35656547	35656548	MACS_peak_7681	3.00	lobChIP_FOXA1_robot
chr2	35687105	35687106	MACS_peak_7682	3.00	lobChIP_FOXA1_robot
chr2	36585165	36585166	MACS_peak_7683	4.00	lobChIP_FOXA1_robot
chr2	36588131	36588132	MACS_peak_7684	10.00	lobChIP_FOXA1_robot
chr2	36602953	36602954	MACS_peak_7685	5.00	lobChIP_FOXA1_robot
chr2	36603548	36603549	MACS_peak_7686	8.00	lobChIP_FOXA1_robot
chr2	36660661	36660662	MACS_peak_7687	24.00	lobChIP_FOXA1_robot
chr2	36681000	36681001	MACS_peak_7688	7.00	lobChIP_FOXA1_robot
chr2	36709803	36709804	MACS_peak_7689	6.00	lobChIP_FOXA1_robot
chr2	36713983	36713984	MACS_peak_7690	19.00	lobChIP_FOXA1_robot
chr2	36729364	36729365	MACS_peak_7691	34.00	lobChIP_FOXA1_robot
chr2	36733052	36733053	MACS_peak_7692	10.00	lobChIP_FOXA1_robot
chr2	36877410	36877411	MACS_peak_7693	7.00	lobChIP_FOXA1_robot
chr2	36887326	36887327	MACS_peak_7694	6.00	lobChIP_FOXA1_robot
chr2	36981996	36981997	MACS_peak_7695	4.00	lobChIP_FOXA1_robot
chr2	36996113	36996114	MACS_peak_7696	4.00	lobChIP_FOXA1_robot
chr2	37194914	37194915	MACS_peak_7697	4.00	lobChIP_FOXA1_robot
chr2	37215312	37215313	MACS_peak_7698	10.00	lobChIP_FOXA1_robot
chr2	37331020	37331021	MACS_peak_7699	4.00	lobChIP_FOXA1_robot
chr2	37398910	37398911	MACS_peak_7700	16.00	lobChIP_FOXA1_robot
chr2	37417311	37417312	MACS_peak_7701	4.00	lobChIP_FOXA1_robot
chr2	37578185	37578186	MACS_peak_7702	4.00	lobChIP_FOXA1_robot
chr2	37617765	37617766	MACS_peak_7703	4.00	lobChIP_FOXA1_robot
chr2	37745002	37745003	MACS_peak_7704	4.00	lobChIP_FOXA1_robot
chr2	38145916	38145917	MACS_peak_7705	9.00	lobChIP_FOXA1_robot
chr2	38147993	38147994	MACS_peak_7706	8.00	lobChIP_FOXA1_robot
chr2	38155670	38155671	MACS_peak_7707	8.00	lobChIP_FOXA1_robot
chr2	38634010	38634011	MACS_peak_7708	5.00	lobChIP_FOXA1_robot
chr2	38663548	38663549	MACS_peak_7709	10.00	lobChIP_FOXA1_robot
chr2	38724529	38724530	MACS_peak_7710	7.00	lobChIP_FOXA1_robot
chr2	38770201	38770202	MACS_peak_7711	5.00	lobChIP_FOXA1_robot
chr2	38826971	38826972	MACS_peak_7712	14.00	lobChIP_FOXA1_robot
chr2	38835032	38835033	MACS_peak_7713	3.00	lobChIP_FOXA1_robot
chr2	38838554	38838555	MACS_peak_7714	25.00	lobChIP_FOXA1_robot
chr2	38893212	38893213	MACS_peak_7715	7.00	lobChIP_FOXA1_robot
chr2	38912676	38912677	MACS_peak_7716	13.00	lobChIP_FOXA1_robot
chr2	38918100	38918101	MACS_peak_7717	5.00	lobChIP_FOXA1_robot
chr2	38969162	38969163	MACS_peak_7718	5.00	lobChIP_FOXA1_robot
chr2	38973154	38973155	MACS_peak_7719	5.00	lobChIP_FOXA1_robot
chr2	38978697	38978698	MACS_peak_7720	3.00	lobChIP_FOXA1_robot
chr2	39021488	39021489	MACS_peak_7721	4.00	lobChIP_FOXA1_robot
chr2	39134871	39134872	MACS_peak_7722	6.00	lobChIP_FOXA1_robot
chr2	39149810	39149811	MACS_peak_7723	8.00	lobChIP_FOXA1_robot
chr2	39208894	39208895	MACS_peak_7724	24.00	lobChIP_FOXA1_robot
chr2	39300151	39300152	MACS_peak_7725	5.00	lobChIP_FOXA1_robot
chr2	39348320	39348321	MACS_peak_7726	3.00	lobChIP_FOXA1_robot
chr2	39361507	39361508	MACS_peak_7727	7.00	lobChIP_FOXA1_robot
chr2	39365488	39365489	MACS_peak_7728	8.00	lobChIP_FOXA1_robot
chr2	39378367	39378368	MACS_peak_7729	8.00	lobChIP_FOXA1_robot
chr2	39444245	39444246	MACS_peak_7730	10.00	lobChIP_FOXA1_robot
chr2	39457192	39457193	MACS_peak_7731	4.00	lobChIP_FOXA1_robot
chr2	39607027	39607028	MACS_peak_7732	9.00	lobChIP_FOXA1_robot
chr2	39620398	39620399	MACS_peak_7733	7.00	lobChIP_FOXA1_robot
chr2	39675484	39675485	MACS_peak_7734	4.00	lobChIP_FOXA1_robot
chr2	39695174	39695175	MACS_peak_7735	6.00	lobChIP_FOXA1_robot
chr2	39719480	39719481	MACS_peak_7736	10.00	lobChIP_FOXA1_robot
chr2	39732270	39732271	MACS_peak_7737	5.00	lobChIP_FOXA1_robot
chr2	39742817	39742818	MACS_peak_7738	4.00	lobChIP_FOXA1_robot
chr2	39743575	39743576	MACS_peak_7739	7.00	lobChIP_FOXA1_robot
chr2	39753357	39753358	MACS_peak_7740	7.00	lobChIP_FOXA1_robot
chr2	39768449	39768450	MACS_peak_7741	4.00	lobChIP_FOXA1_robot
chr2	39773815	39773816	MACS_peak_7742	4.00	lobChIP_FOXA1_robot
chr2	39774236	39774237	MACS_peak_7743	5.00	lobChIP_FOXA1_robot
chr2	39778009	39778010	MACS_peak_7744	31.00	lobChIP_FOXA1_robot
chr2	40579344	40579345	MACS_peak_7745	3.00	lobChIP_FOXA1_robot
chr2	40699180	40699181	MACS_peak_7746	3.00	lobChIP_FOXA1_robot
chr2	41053526	41053527	MACS_peak_7747	4.00	lobChIP_FOXA1_robot
chr2	42055376	42055377	MACS_peak_7748	19.00	lobChIP_FOXA1_robot
chr2	42075423	42075424	MACS_peak_7749	29.00	lobChIP_FOXA1_robot
chr2	42273345	42273346	MACS_peak_7750	5.00	lobChIP_FOXA1_robot
chr2	42283316	42283317	MACS_peak_7751	10.00	lobChIP_FOXA1_robot
chr2	42290990	42290991	MACS_peak_7752	8.00	lobChIP_FOXA1_robot
chr2	42291813	42291814	MACS_peak_7753	4.00	lobChIP_FOXA1_robot
chr2	42319705	42319706	MACS_peak_7754	7.00	lobChIP_FOXA1_robot
chr2	42323200	42323201	MACS_peak_7755	11.00	lobChIP_FOXA1_robot
chr2	42333294	42333295	MACS_peak_7756	5.00	lobChIP_FOXA1_robot
chr2	42335068	42335069	MACS_peak_7757	5.00	lobChIP_FOXA1_robot
chr2	42344174	42344175	MACS_peak_7758	5.00	lobChIP_FOXA1_robot
chr2	42345133	42345134	MACS_peak_7759	11.00	lobChIP_FOXA1_robot
chr2	42354980	42354981	MACS_peak_7760	10.00	lobChIP_FOXA1_robot
chr2	42360266	42360267	MACS_peak_7761	7.00	lobChIP_FOXA1_robot
chr2	42363099	42363100	MACS_peak_7762	25.00	lobChIP_FOXA1_robot
chr2	42364372	42364373	MACS_peak_7763	8.00	lobChIP_FOXA1_robot
chr2	42369171	42369172	MACS_peak_7764	10.00	lobChIP_FOXA1_robot
chr2	42415284	42415285	MACS_peak_7765	4.00	lobChIP_FOXA1_robot
chr2	42418694	42418695	MACS_peak_7766	7.00	lobChIP_FOXA1_robot
chr2	42422890	42422891	MACS_peak_7767	22.00	lobChIP_FOXA1_robot
chr2	42424176	42424177	MACS_peak_7768	6.00	lobChIP_FOXA1_robot
chr2	42444647	42444648	MACS_peak_7769	11.00	lobChIP_FOXA1_robot
chr2	42449773	42449774	MACS_peak_7770	25.00	lobChIP_FOXA1_robot
chr2	42471585	42471586	MACS_peak_7771	9.00	lobChIP_FOXA1_robot
chr2	42512244	42512245	MACS_peak_7772	5.00	lobChIP_FOXA1_robot
chr2	42513943	42513944	MACS_peak_7773	9.00	lobChIP_FOXA1_robot
chr2	42554524	42554525	MACS_peak_7774	4.00	lobChIP_FOXA1_robot
chr2	42584273	42584274	MACS_peak_7775	4.00	lobChIP_FOXA1_robot
chr2	42702192	42702193	MACS_peak_7776	4.00	lobChIP_FOXA1_robot
chr2	42901294	42901295	MACS_peak_7777	6.00	lobChIP_FOXA1_robot
chr2	42903116	42903117	MACS_peak_7778	8.00	lobChIP_FOXA1_robot
chr2	43014997	43014998	MACS_peak_7779	5.00	lobChIP_FOXA1_robot
chr2	43155882	43155883	MACS_peak_7780	5.00	lobChIP_FOXA1_robot
chr2	43157314	43157315	MACS_peak_7781	4.00	lobChIP_FOXA1_robot
chr2	43169070	43169071	MACS_peak_7782	5.00	lobChIP_FOXA1_robot
chr2	43205659	43205660	MACS_peak_7783	10.00	lobChIP_FOXA1_robot
chr2	43206900	43206901	MACS_peak_7784	30.00	lobChIP_FOXA1_robot
chr2	43214413	43214414	MACS_peak_7785	6.00	lobChIP_FOXA1_robot
chr2	43315951	43315952	MACS_peak_7786	20.00	lobChIP_FOXA1_robot
chr2	43390300	43390301	MACS_peak_7787	8.00	lobChIP_FOXA1_robot
chr2	43411789	43411790	MACS_peak_7788	5.00	lobChIP_FOXA1_robot
chr2	43437861	43437862	MACS_peak_7789	5.00	lobChIP_FOXA1_robot
chr2	43454438	43454439	MACS_peak_7790	7.00	lobChIP_FOXA1_robot
chr2	43485815	43485816	MACS_peak_7791	20.00	lobChIP_FOXA1_robot
chr2	43519645	43519646	MACS_peak_7792	3.00	lobChIP_FOXA1_robot
chr2	43521165	43521166	MACS_peak_7793	18.00	lobChIP_FOXA1_robot
chr2	43528576	43528577	MACS_peak_7794	4.00	lobChIP_FOXA1_robot
chr2	43538640	43538641	MACS_peak_7795	13.00	lobChIP_FOXA1_robot
chr2	43587503	43587504	MACS_peak_7796	5.00	lobChIP_FOXA1_robot
chr2	43603166	43603167	MACS_peak_7797	23.00	lobChIP_FOXA1_robot
chr2	43603783	43603784	MACS_peak_7798	4.00	lobChIP_FOXA1_robot
chr2	43617186	43617187	MACS_peak_7799	10.00	lobChIP_FOXA1_robot
chr2	43626374	43626375	MACS_peak_7800	5.00	lobChIP_FOXA1_robot
chr2	43628068	43628069	MACS_peak_7801	4.00	lobChIP_FOXA1_robot
chr2	43638524	43638525	MACS_peak_7802	3.00	lobChIP_FOXA1_robot
chr2	43647569	43647570	MACS_peak_7803	9.00	lobChIP_FOXA1_robot
chr2	43651424	43651425	MACS_peak_7804	8.00	lobChIP_FOXA1_robot
chr2	43653554	43653555	MACS_peak_7805	6.00	lobChIP_FOXA1_robot
chr2	43678658	43678659	MACS_peak_7806	9.00	lobChIP_FOXA1_robot
chr2	43683534	43683535	MACS_peak_7807	10.00	lobChIP_FOXA1_robot
chr2	43686801	43686802	MACS_peak_7808	3.00	lobChIP_FOXA1_robot
chr2	43690472	43690473	MACS_peak_7809	14.00	lobChIP_FOXA1_robot
chr2	43861904	43861905	MACS_peak_7810	37.00	lobChIP_FOXA1_robot
chr2	43972782	43972783	MACS_peak_7811	6.00	lobChIP_FOXA1_robot
chr2	43986528	43986529	MACS_peak_7812	15.00	lobChIP_FOXA1_robot
chr2	44033748	44033749	MACS_peak_7813	6.00	lobChIP_FOXA1_robot
chr2	44035619	44035620	MACS_peak_7814	3.00	lobChIP_FOXA1_robot
chr2	44069825	44069826	MACS_peak_7815	20.00	lobChIP_FOXA1_robot
chr2	44108933	44108934	MACS_peak_7816	5.00	lobChIP_FOXA1_robot
chr2	44112008	44112009	MACS_peak_7817	5.00	lobChIP_FOXA1_robot
chr2	44134081	44134082	MACS_peak_7818	5.00	lobChIP_FOXA1_robot
chr2	44288329	44288330	MACS_peak_7819	5.00	lobChIP_FOXA1_robot
chr2	44292900	44292901	MACS_peak_7820	8.00	lobChIP_FOXA1_robot
chr2	44314539	44314540	MACS_peak_7821	9.00	lobChIP_FOXA1_robot
chr2	44397879	44397880	MACS_peak_7822	4.00	lobChIP_FOXA1_robot
chr2	44460659	44460660	MACS_peak_7823	4.00	lobChIP_FOXA1_robot
chr2	44751929	44751930	MACS_peak_7824	4.00	lobChIP_FOXA1_robot
chr2	45465674	45465675	MACS_peak_7825	3.00	lobChIP_FOXA1_robot
chr2	45484698	45484699	MACS_peak_7826	9.00	lobChIP_FOXA1_robot
chr2	45500815	45500816	MACS_peak_7827	34.00	lobChIP_FOXA1_robot
chr2	45501819	45501820	MACS_peak_7828	15.00	lobChIP_FOXA1_robot
chr2	45527337	45527338	MACS_peak_7829	7.00	lobChIP_FOXA1_robot
chr2	45600745	45600746	MACS_peak_7830	3.00	lobChIP_FOXA1_robot
chr2	45666647	45666648	MACS_peak_7831	5.00	lobChIP_FOXA1_robot
chr2	45690490	45690491	MACS_peak_7832	4.00	lobChIP_FOXA1_robot
chr2	45697099	45697100	MACS_peak_7833	13.00	lobChIP_FOXA1_robot
chr2	45790775	45790776	MACS_peak_7834	3.00	lobChIP_FOXA1_robot
chr2	45814131	45814132	MACS_peak_7835	8.00	lobChIP_FOXA1_robot
chr2	45838646	45838647	MACS_peak_7836	4.00	lobChIP_FOXA1_robot
chr2	46005892	46005893	MACS_peak_7837	10.00	lobChIP_FOXA1_robot
chr2	46170952	46170953	MACS_peak_7838	5.00	lobChIP_FOXA1_robot
chr2	46219688	46219689	MACS_peak_7839	9.00	lobChIP_FOXA1_robot
chr2	46229846	46229847	MACS_peak_7840	9.00	lobChIP_FOXA1_robot
chr2	46265555	46265556	MACS_peak_7841	5.00	lobChIP_FOXA1_robot
chr2	46266636	46266637	MACS_peak_7842	3.00	lobChIP_FOXA1_robot
chr2	46367957	46367958	MACS_peak_7843	4.00	lobChIP_FOXA1_robot
chr2	46400949	46400950	MACS_peak_7844	32.00	lobChIP_FOXA1_robot
chr2	46526507	46526508	MACS_peak_7845	3.00	lobChIP_FOXA1_robot
chr2	46534494	46534495	MACS_peak_7846	16.00	lobChIP_FOXA1_robot
chr2	46535305	46535306	MACS_peak_7847	3.00	lobChIP_FOXA1_robot
chr2	46535913	46535914	MACS_peak_7848	5.00	lobChIP_FOXA1_robot
chr2	46542382	46542383	MACS_peak_7849	3.00	lobChIP_FOXA1_robot
chr2	46547436	46547437	MACS_peak_7850	6.00	lobChIP_FOXA1_robot
chr2	46553678	46553679	MACS_peak_7851	4.00	lobChIP_FOXA1_robot
chr2	46563380	46563381	MACS_peak_7852	6.00	lobChIP_FOXA1_robot
chr2	46622030	46622031	MACS_peak_7853	9.00	lobChIP_FOXA1_robot
chr2	46636435	46636436	MACS_peak_7854	20.00	lobChIP_FOXA1_robot
chr2	46644401	46644402	MACS_peak_7855	5.00	lobChIP_FOXA1_robot
chr2	46816027	46816028	MACS_peak_7856	3.00	lobChIP_FOXA1_robot
chr2	46930899	46930900	MACS_peak_7857	4.00	lobChIP_FOXA1_robot
chr2	46951409	46951410	MACS_peak_7858	6.00	lobChIP_FOXA1_robot
chr2	46964926	46964927	MACS_peak_7859	21.00	lobChIP_FOXA1_robot
chr2	46998008	46998009	MACS_peak_7860	3.00	lobChIP_FOXA1_robot
chr2	47182257	47182258	MACS_peak_7861	7.00	lobChIP_FOXA1_robot
chr2	47194494	47194495	MACS_peak_7862	5.00	lobChIP_FOXA1_robot
chr2	47198097	47198098	MACS_peak_7863	6.00	lobChIP_FOXA1_robot
chr2	47257423	47257424	MACS_peak_7864	9.00	lobChIP_FOXA1_robot
chr2	47261810	47261811	MACS_peak_7865	7.00	lobChIP_FOXA1_robot
chr2	47262368	47262369	MACS_peak_7866	3.00	lobChIP_FOXA1_robot
chr2	47298528	47298529	MACS_peak_7867	3.00	lobChIP_FOXA1_robot
chr2	47304726	47304727	MACS_peak_7868	3.00	lobChIP_FOXA1_robot
chr2	47306267	47306268	MACS_peak_7869	24.00	lobChIP_FOXA1_robot
chr2	47419168	47419169	MACS_peak_7870	9.00	lobChIP_FOXA1_robot
chr2	47509949	47509950	MACS_peak_7871	6.00	lobChIP_FOXA1_robot
chr2	47537192	47537193	MACS_peak_7872	13.00	lobChIP_FOXA1_robot
chr2	47540445	47540446	MACS_peak_7873	9.00	lobChIP_FOXA1_robot
chr2	47572425	47572426	MACS_peak_7874	7.00	lobChIP_FOXA1_robot
chr2	47629988	47629989	MACS_peak_7875	9.00	lobChIP_FOXA1_robot
chr2	47979754	47979755	MACS_peak_7876	5.00	lobChIP_FOXA1_robot
chr2	48131374	48131375	MACS_peak_7877	4.00	lobChIP_FOXA1_robot
chr2	48447113	48447114	MACS_peak_7878	4.00	lobChIP_FOXA1_robot
chr2	48449232	48449233	MACS_peak_7879	45.00	lobChIP_FOXA1_robot
chr2	48497452	48497453	MACS_peak_7880	4.00	lobChIP_FOXA1_robot
chr2	48571478	48571479	MACS_peak_7881	8.00	lobChIP_FOXA1_robot
chr2	48675929	48675930	MACS_peak_7882	7.00	lobChIP_FOXA1_robot
chr2	49987862	49987863	MACS_peak_7883	4.00	lobChIP_FOXA1_robot
chr2	52389146	52389147	MACS_peak_7884	5.00	lobChIP_FOXA1_robot
chr2	54054145	54054146	MACS_peak_7885	34.00	lobChIP_FOXA1_robot
chr2	54078186	54078187	MACS_peak_7886	3.00	lobChIP_FOXA1_robot
chr2	54196597	54196598	MACS_peak_7887	8.00	lobChIP_FOXA1_robot
chr2	54235793	54235794	MACS_peak_7888	9.00	lobChIP_FOXA1_robot
chr2	54243300	54243301	MACS_peak_7889	5.00	lobChIP_FOXA1_robot
chr2	54266311	54266312	MACS_peak_7890	4.00	lobChIP_FOXA1_robot
chr2	54271274	54271275	MACS_peak_7891	27.00	lobChIP_FOXA1_robot
chr2	54276582	54276583	MACS_peak_7892	5.00	lobChIP_FOXA1_robot
chr2	54334547	54334548	MACS_peak_7893	7.00	lobChIP_FOXA1_robot
chr2	54345540	54345541	MACS_peak_7894	7.00	lobChIP_FOXA1_robot
chr2	54349421	54349422	MACS_peak_7895	8.00	lobChIP_FOXA1_robot
chr2	54366703	54366704	MACS_peak_7896	3.00	lobChIP_FOXA1_robot
chr2	54435031	54435032	MACS_peak_7897	22.00	lobChIP_FOXA1_robot
chr2	54461380	54461381	MACS_peak_7898	34.00	lobChIP_FOXA1_robot
chr2	54474440	54474441	MACS_peak_7899	5.00	lobChIP_FOXA1_robot
chr2	54488779	54488780	MACS_peak_7900	12.00	lobChIP_FOXA1_robot
chr2	54674014	54674015	MACS_peak_7901	7.00	lobChIP_FOXA1_robot
chr2	54680209	54680210	MACS_peak_7902	3.00	lobChIP_FOXA1_robot
chr2	54681953	54681954	MACS_peak_7903	22.00	lobChIP_FOXA1_robot
chr2	54750757	54750758	MACS_peak_7904	3.00	lobChIP_FOXA1_robot
chr2	54773109	54773110	MACS_peak_7905	12.00	lobChIP_FOXA1_robot
chr2	54799588	54799589	MACS_peak_7906	28.00	lobChIP_FOXA1_robot
chr2	54801403	54801404	MACS_peak_7907	12.00	lobChIP_FOXA1_robot
chr2	54806995	54806996	MACS_peak_7908	10.00	lobChIP_FOXA1_robot
chr2	54817207	54817208	MACS_peak_7909	23.00	lobChIP_FOXA1_robot
chr2	54837032	54837033	MACS_peak_7910	4.00	lobChIP_FOXA1_robot
chr2	54846305	54846306	MACS_peak_7911	4.00	lobChIP_FOXA1_robot
chr2	54857593	54857594	MACS_peak_7912	6.00	lobChIP_FOXA1_robot
chr2	54897027	54897028	MACS_peak_7913	11.00	lobChIP_FOXA1_robot
chr2	54933941	54933942	MACS_peak_7914	8.00	lobChIP_FOXA1_robot
chr2	55013518	55013519	MACS_peak_7915	5.00	lobChIP_FOXA1_robot
chr2	55085621	55085622	MACS_peak_7916	5.00	lobChIP_FOXA1_robot
chr2	55099028	55099029	MACS_peak_7917	5.00	lobChIP_FOXA1_robot
chr2	55274108	55274109	MACS_peak_7918	4.00	lobChIP_FOXA1_robot
chr2	55336892	55336893	MACS_peak_7919	29.00	lobChIP_FOXA1_robot
chr2	55407693	55407694	MACS_peak_7920	10.00	lobChIP_FOXA1_robot
chr2	55466501	55466502	MACS_peak_7921	7.00	lobChIP_FOXA1_robot
chr2	55466975	55466976	MACS_peak_7922	23.00	lobChIP_FOXA1_robot
chr2	55843916	55843917	MACS_peak_7923	5.00	lobChIP_FOXA1_robot
chr2	56066477	56066478	MACS_peak_7924	18.00	lobChIP_FOXA1_robot
chr2	57559966	57559967	MACS_peak_7925	4.00	lobChIP_FOXA1_robot
chr2	57765297	57765298	MACS_peak_7926	3.00	lobChIP_FOXA1_robot
chr2	58121089	58121090	MACS_peak_7927	4.00	lobChIP_FOXA1_robot
chr2	58268072	58268073	MACS_peak_7928	4.00	lobChIP_FOXA1_robot
chr2	58308421	58308422	MACS_peak_7929	5.00	lobChIP_FOXA1_robot
chr2	58397833	58397834	MACS_peak_7930	4.00	lobChIP_FOXA1_robot
chr2	58430697	58430698	MACS_peak_7931	4.00	lobChIP_FOXA1_robot
chr2	58431751	58431752	MACS_peak_7932	5.00	lobChIP_FOXA1_robot
chr2	59119133	59119134	MACS_peak_7933	5.00	lobChIP_FOXA1_robot
chr2	59300626	59300627	MACS_peak_7934	5.00	lobChIP_FOXA1_robot
chr2	59834002	59834003	MACS_peak_7935	4.00	lobChIP_FOXA1_robot
chr2	60321714	60321715	MACS_peak_7936	3.00	lobChIP_FOXA1_robot
chr2	61026391	61026392	MACS_peak_7937	4.00	lobChIP_FOXA1_robot
chr2	61112963	61112964	MACS_peak_7938	11.00	lobChIP_FOXA1_robot
chr2	61153357	61153358	MACS_peak_7939	7.00	lobChIP_FOXA1_robot
chr2	61196196	61196197	MACS_peak_7940	4.00	lobChIP_FOXA1_robot
chr2	61243788	61243789	MACS_peak_7941	4.00	lobChIP_FOXA1_robot
chr2	61494034	61494035	MACS_peak_7942	5.00	lobChIP_FOXA1_robot
chr2	61583997	61583998	MACS_peak_7943	5.00	lobChIP_FOXA1_robot
chr2	61614916	61614917	MACS_peak_7944	4.00	lobChIP_FOXA1_robot
chr2	61898917	61898918	MACS_peak_7945	7.00	lobChIP_FOXA1_robot
chr2	62250494	62250495	MACS_peak_7946	7.00	lobChIP_FOXA1_robot
chr2	62519302	62519303	MACS_peak_7947	3.00	lobChIP_FOXA1_robot
chr2	62636167	62636168	MACS_peak_7948	4.00	lobChIP_FOXA1_robot
chr2	62694361	62694362	MACS_peak_7949	7.00	lobChIP_FOXA1_robot
chr2	62695058	62695059	MACS_peak_7950	5.00	lobChIP_FOXA1_robot
chr2	62716662	62716663	MACS_peak_7951	4.00	lobChIP_FOXA1_robot
chr2	62753887	62753888	MACS_peak_7952	4.00	lobChIP_FOXA1_robot
chr2	62755332	62755333	MACS_peak_7953	10.00	lobChIP_FOXA1_robot
chr2	62767628	62767629	MACS_peak_7954	4.00	lobChIP_FOXA1_robot
chr2	62792858	62792859	MACS_peak_7955	6.00	lobChIP_FOXA1_robot
chr2	62796277	62796278	MACS_peak_7956	9.00	lobChIP_FOXA1_robot
chr2	62797435	62797436	MACS_peak_7957	30.00	lobChIP_FOXA1_robot
chr2	62805287	62805288	MACS_peak_7958	8.00	lobChIP_FOXA1_robot
chr2	62806171	62806172	MACS_peak_7959	5.00	lobChIP_FOXA1_robot
chr2	62869928	62869929	MACS_peak_7960	5.00	lobChIP_FOXA1_robot
chr2	62881240	62881241	MACS_peak_7961	12.00	lobChIP_FOXA1_robot
chr2	62966512	62966513	MACS_peak_7962	6.00	lobChIP_FOXA1_robot
chr2	62991032	62991033	MACS_peak_7963	5.00	lobChIP_FOXA1_robot
chr2	63022156	63022157	MACS_peak_7964	8.00	lobChIP_FOXA1_robot
chr2	63059915	63059916	MACS_peak_7965	3.00	lobChIP_FOXA1_robot
chr2	63098857	63098858	MACS_peak_7966	7.00	lobChIP_FOXA1_robot
chr2	63198432	63198433	MACS_peak_7967	4.00	lobChIP_FOXA1_robot
chr2	63221975	63221976	MACS_peak_7968	3.00	lobChIP_FOXA1_robot
chr2	63420228	63420229	MACS_peak_7969	113.00	lobChIP_FOXA1_robot
chr2	63421479	63421480	MACS_peak_7970	5.00	lobChIP_FOXA1_robot
chr2	63823837	63823838	MACS_peak_7971	7.00	lobChIP_FOXA1_robot
chr2	63961761	63961762	MACS_peak_7972	26.00	lobChIP_FOXA1_robot
chr2	64027693	64027694	MACS_peak_7973	3.00	lobChIP_FOXA1_robot
chr2	64055147	64055148	MACS_peak_7974	12.00	lobChIP_FOXA1_robot
chr2	64069920	64069921	MACS_peak_7975	7.00	lobChIP_FOXA1_robot
chr2	64177849	64177850	MACS_peak_7976	5.00	lobChIP_FOXA1_robot
chr2	64209049	64209050	MACS_peak_7977	32.00	lobChIP_FOXA1_robot
chr2	64211553	64211554	MACS_peak_7978	70.00	lobChIP_FOXA1_robot
chr2	64218788	64218789	MACS_peak_7979	35.00	lobChIP_FOXA1_robot
chr2	64220420	64220421	MACS_peak_7980	5.00	lobChIP_FOXA1_robot
chr2	64249551	64249552	MACS_peak_7981	4.00	lobChIP_FOXA1_robot
chr2	64251671	64251672	MACS_peak_7982	19.00	lobChIP_FOXA1_robot
chr2	64355654	64355655	MACS_peak_7983	3.00	lobChIP_FOXA1_robot
chr2	64385908	64385909	MACS_peak_7984	4.00	lobChIP_FOXA1_robot
chr2	64470528	64470529	MACS_peak_7985	4.00	lobChIP_FOXA1_robot
chr2	64653479	64653480	MACS_peak_7986	5.00	lobChIP_FOXA1_robot
chr2	64744735	64744736	MACS_peak_7987	4.00	lobChIP_FOXA1_robot
chr2	64769988	64769989	MACS_peak_7988	4.00	lobChIP_FOXA1_robot
chr2	64868562	64868563	MACS_peak_7989	4.00	lobChIP_FOXA1_robot
chr2	64872070	64872071	MACS_peak_7990	4.00	lobChIP_FOXA1_robot
chr2	64873557	64873558	MACS_peak_7991	23.00	lobChIP_FOXA1_robot
chr2	64877999	64878000	MACS_peak_7992	7.00	lobChIP_FOXA1_robot
chr2	64896605	64896606	MACS_peak_7993	4.00	lobChIP_FOXA1_robot
chr2	64911482	64911483	MACS_peak_7994	6.00	lobChIP_FOXA1_robot
chr2	64912773	64912774	MACS_peak_7995	13.00	lobChIP_FOXA1_robot
chr2	64957750	64957751	MACS_peak_7996	9.00	lobChIP_FOXA1_robot
chr2	64969637	64969638	MACS_peak_7997	3.00	lobChIP_FOXA1_robot
chr2	65150030	65150031	MACS_peak_7998	3.00	lobChIP_FOXA1_robot
chr2	65170964	65170965	MACS_peak_7999	12.00	lobChIP_FOXA1_robot
chr2	65209905	65209906	MACS_peak_8000	15.00	lobChIP_FOXA1_robot
chr2	65253419	65253420	MACS_peak_8001	5.00	lobChIP_FOXA1_robot
chr2	65255745	65255746	MACS_peak_8002	6.00	lobChIP_FOXA1_robot
chr2	65258991	65258992	MACS_peak_8003	6.00	lobChIP_FOXA1_robot
chr2	65345493	65345494	MACS_peak_8004	3.00	lobChIP_FOXA1_robot
chr2	65528382	65528383	MACS_peak_8005	17.00	lobChIP_FOXA1_robot
chr2	65590461	65590462	MACS_peak_8006	5.00	lobChIP_FOXA1_robot
chr2	65600673	65600674	MACS_peak_8007	68.00	lobChIP_FOXA1_robot
chr2	65623406	65623407	MACS_peak_8008	6.00	lobChIP_FOXA1_robot
chr2	65804322	65804323	MACS_peak_8009	6.00	lobChIP_FOXA1_robot
chr2	66496617	66496618	MACS_peak_8010	4.00	lobChIP_FOXA1_robot
chr2	66727180	66727181	MACS_peak_8011	6.00	lobChIP_FOXA1_robot
chr2	66750381	66750382	MACS_peak_8012	5.00	lobChIP_FOXA1_robot
chr2	67285297	67285298	MACS_peak_8013	4.00	lobChIP_FOXA1_robot
chr2	67536640	67536641	MACS_peak_8014	5.00	lobChIP_FOXA1_robot
chr2	68118837	68118838	MACS_peak_8015	3.00	lobChIP_FOXA1_robot
chr2	68181595	68181596	MACS_peak_8016	6.00	lobChIP_FOXA1_robot
chr2	68348330	68348331	MACS_peak_8017	4.00	lobChIP_FOXA1_robot
chr2	68744278	68744279	MACS_peak_8018	10.00	lobChIP_FOXA1_robot
chr2	68745859	68745860	MACS_peak_8019	7.00	lobChIP_FOXA1_robot
chr2	68998463	68998464	MACS_peak_8020	3.00	lobChIP_FOXA1_robot
chr2	69115382	69115383	MACS_peak_8021	4.00	lobChIP_FOXA1_robot
chr2	69247999	69248000	MACS_peak_8022	4.00	lobChIP_FOXA1_robot
chr2	69273452	69273453	MACS_peak_8023	5.00	lobChIP_FOXA1_robot
chr2	69310040	69310041	MACS_peak_8024	15.00	lobChIP_FOXA1_robot
chr2	69324856	69324857	MACS_peak_8025	5.00	lobChIP_FOXA1_robot
chr2	69394083	69394084	MACS_peak_8026	12.00	lobChIP_FOXA1_robot
chr2	69405378	69405379	MACS_peak_8027	5.00	lobChIP_FOXA1_robot
chr2	69443882	69443883	MACS_peak_8028	11.00	lobChIP_FOXA1_robot
chr2	69624464	69624465	MACS_peak_8029	5.00	lobChIP_FOXA1_robot
chr2	69711104	69711105	MACS_peak_8030	10.00	lobChIP_FOXA1_robot
chr2	69727399	69727400	MACS_peak_8031	3.00	lobChIP_FOXA1_robot
chr2	69902469	69902470	MACS_peak_8032	4.00	lobChIP_FOXA1_robot
chr2	69912972	69912973	MACS_peak_8033	4.00	lobChIP_FOXA1_robot
chr2	69914156	69914157	MACS_peak_8034	4.00	lobChIP_FOXA1_robot
chr2	69961636	69961637	MACS_peak_8035	4.00	lobChIP_FOXA1_robot
chr2	69971119	69971120	MACS_peak_8036	7.00	lobChIP_FOXA1_robot
chr2	70008756	70008757	MACS_peak_8037	20.00	lobChIP_FOXA1_robot
chr2	70154424	70154425	MACS_peak_8038	5.00	lobChIP_FOXA1_robot
chr2	70173814	70173815	MACS_peak_8039	5.00	lobChIP_FOXA1_robot
chr2	70207744	70207745	MACS_peak_8040	4.00	lobChIP_FOXA1_robot
chr2	70313873	70313874	MACS_peak_8041	18.00	lobChIP_FOXA1_robot
chr2	70359715	70359716	MACS_peak_8042	3.00	lobChIP_FOXA1_robot
chr2	70415691	70415692	MACS_peak_8043	8.00	lobChIP_FOXA1_robot
chr2	70423139	70423140	MACS_peak_8044	4.00	lobChIP_FOXA1_robot
chr2	70439859	70439860	MACS_peak_8045	5.00	lobChIP_FOXA1_robot
chr2	70476169	70476170	MACS_peak_8046	5.00	lobChIP_FOXA1_robot
chr2	70476941	70476942	MACS_peak_8047	8.00	lobChIP_FOXA1_robot
chr2	70784498	70784499	MACS_peak_8048	12.00	lobChIP_FOXA1_robot
chr2	70792451	70792452	MACS_peak_8049	16.00	lobChIP_FOXA1_robot
chr2	71034541	71034542	MACS_peak_8050	29.00	lobChIP_FOXA1_robot
chr2	71147060	71147061	MACS_peak_8051	8.00	lobChIP_FOXA1_robot
chr2	71560270	71560271	MACS_peak_8052	4.00	lobChIP_FOXA1_robot
chr2	71882233	71882234	MACS_peak_8053	3.00	lobChIP_FOXA1_robot
chr2	72186890	72186891	MACS_peak_8054	4.00	lobChIP_FOXA1_robot
chr2	72222649	72222650	MACS_peak_8055	4.00	lobChIP_FOXA1_robot
chr2	72305259	72305260	MACS_peak_8056	42.00	lobChIP_FOXA1_robot
chr2	72734297	72734298	MACS_peak_8057	5.00	lobChIP_FOXA1_robot
chr2	72775546	72775547	MACS_peak_8058	11.00	lobChIP_FOXA1_robot
chr2	72908739	72908740	MACS_peak_8059	8.00	lobChIP_FOXA1_robot
chr2	72922151	72922152	MACS_peak_8060	9.00	lobChIP_FOXA1_robot
chr2	72951056	72951057	MACS_peak_8061	7.00	lobChIP_FOXA1_robot
chr2	72970817	72970818	MACS_peak_8062	9.00	lobChIP_FOXA1_robot
chr2	72984510	72984511	MACS_peak_8063	23.00	lobChIP_FOXA1_robot
chr2	73248748	73248749	MACS_peak_8064	5.00	lobChIP_FOXA1_robot
chr2	73259512	73259513	MACS_peak_8065	17.00	lobChIP_FOXA1_robot
chr2	73295864	73295865	MACS_peak_8066	5.00	lobChIP_FOXA1_robot
chr2	73418972	73418973	MACS_peak_8067	6.00	lobChIP_FOXA1_robot
chr2	73427114	73427115	MACS_peak_8068	6.00	lobChIP_FOXA1_robot
chr2	73459795	73459796	MACS_peak_8069	3.00	lobChIP_FOXA1_robot
chr2	73489651	73489652	MACS_peak_8070	16.00	lobChIP_FOXA1_robot
chr2	73612150	73612151	MACS_peak_8071	5.00	lobChIP_FOXA1_robot
chr2	73869983	73869984	MACS_peak_8072	5.00	lobChIP_FOXA1_robot
chr2	74007292	74007293	MACS_peak_8073	5.00	lobChIP_FOXA1_robot
chr2	74070068	74070069	MACS_peak_8074	5.00	lobChIP_FOXA1_robot
chr2	74207936	74207937	MACS_peak_8075	8.00	lobChIP_FOXA1_robot
chr2	74287247	74287248	MACS_peak_8076	3.00	lobChIP_FOXA1_robot
chr2	74520072	74520073	MACS_peak_8077	11.00	lobChIP_FOXA1_robot
chr2	74713488	74713489	MACS_peak_8078	9.00	lobChIP_FOXA1_robot
chr2	74716362	74716363	MACS_peak_8079	5.00	lobChIP_FOXA1_robot
chr2	74866874	74866875	MACS_peak_8080	11.00	lobChIP_FOXA1_robot
chr2	74979305	74979306	MACS_peak_8081	6.00	lobChIP_FOXA1_robot
chr2	74983666	74983667	MACS_peak_8082	5.00	lobChIP_FOXA1_robot
chr2	74992210	74992211	MACS_peak_8083	46.00	lobChIP_FOXA1_robot
chr2	75005104	75005105	MACS_peak_8084	4.00	lobChIP_FOXA1_robot
chr2	75033867	75033868	MACS_peak_8085	13.00	lobChIP_FOXA1_robot
chr2	75040109	75040110	MACS_peak_8086	5.00	lobChIP_FOXA1_robot
chr2	75043847	75043848	MACS_peak_8087	4.00	lobChIP_FOXA1_robot
chr2	75067604	75067605	MACS_peak_8088	4.00	lobChIP_FOXA1_robot
chr2	75070575	75070576	MACS_peak_8089	4.00	lobChIP_FOXA1_robot
chr2	75083625	75083626	MACS_peak_8090	22.00	lobChIP_FOXA1_robot
chr2	75129607	75129608	MACS_peak_8091	7.00	lobChIP_FOXA1_robot
chr2	75164857	75164858	MACS_peak_8092	12.00	lobChIP_FOXA1_robot
chr2	75167363	75167364	MACS_peak_8093	50.00	lobChIP_FOXA1_robot
chr2	75280847	75280848	MACS_peak_8094	4.00	lobChIP_FOXA1_robot
chr2	75332673	75332674	MACS_peak_8095	3.00	lobChIP_FOXA1_robot
chr2	75380586	75380587	MACS_peak_8096	31.00	lobChIP_FOXA1_robot
chr2	75692941	75692942	MACS_peak_8097	4.00	lobChIP_FOXA1_robot
chr2	75708676	75708677	MACS_peak_8098	69.00	lobChIP_FOXA1_robot
chr2	75743708	75743709	MACS_peak_8099	22.00	lobChIP_FOXA1_robot
chr2	75748845	75748846	MACS_peak_8100	35.00	lobChIP_FOXA1_robot
chr2	75749880	75749881	MACS_peak_8101	10.00	lobChIP_FOXA1_robot
chr2	75750913	75750914	MACS_peak_8102	10.00	lobChIP_FOXA1_robot
chr2	75776968	75776969	MACS_peak_8103	8.00	lobChIP_FOXA1_robot
chr2	75798330	75798331	MACS_peak_8104	14.00	lobChIP_FOXA1_robot
chr2	75804525	75804526	MACS_peak_8105	7.00	lobChIP_FOXA1_robot
chr2	75810149	75810150	MACS_peak_8106	5.00	lobChIP_FOXA1_robot
chr2	75941987	75941988	MACS_peak_8107	5.00	lobChIP_FOXA1_robot
chr2	76399524	76399525	MACS_peak_8108	12.00	lobChIP_FOXA1_robot
chr2	76442735	76442736	MACS_peak_8109	5.00	lobChIP_FOXA1_robot
chr2	77043653	77043654	MACS_peak_8110	3.00	lobChIP_FOXA1_robot
chr2	77418321	77418322	MACS_peak_8111	7.00	lobChIP_FOXA1_robot
chr2	77465621	77465622	MACS_peak_8112	4.00	lobChIP_FOXA1_robot
chr2	77832261	77832262	MACS_peak_8113	8.00	lobChIP_FOXA1_robot
chr2	78065703	78065704	MACS_peak_8114	8.00	lobChIP_FOXA1_robot
chr2	78389553	78389554	MACS_peak_8115	29.00	lobChIP_FOXA1_robot
chr2	78487456	78487457	MACS_peak_8116	4.00	lobChIP_FOXA1_robot
chr2	78488184	78488185	MACS_peak_8117	24.00	lobChIP_FOXA1_robot
chr2	78643961	78643962	MACS_peak_8118	12.00	lobChIP_FOXA1_robot
chr2	79199313	79199314	MACS_peak_8119	4.00	lobChIP_FOXA1_robot
chr2	80773144	80773145	MACS_peak_8120	3.00	lobChIP_FOXA1_robot
chr2	81474954	81474955	MACS_peak_8121	6.00	lobChIP_FOXA1_robot
chr2	82755181	82755182	MACS_peak_8122	3.00	lobChIP_FOXA1_robot
chr2	83298600	83298601	MACS_peak_8123	6.00	lobChIP_FOXA1_robot
chr2	83344397	83344398	MACS_peak_8124	4.00	lobChIP_FOXA1_robot
chr2	83569679	83569680	MACS_peak_8125	7.00	lobChIP_FOXA1_robot
chr2	84339013	84339014	MACS_peak_8126	4.00	lobChIP_FOXA1_robot
chr2	84521110	84521111	MACS_peak_8127	22.00	lobChIP_FOXA1_robot
chr2	84524589	84524590	MACS_peak_8128	4.00	lobChIP_FOXA1_robot
chr2	84527462	84527463	MACS_peak_8129	82.00	lobChIP_FOXA1_robot
chr2	84687229	84687230	MACS_peak_8130	4.00	lobChIP_FOXA1_robot
chr2	84840469	84840470	MACS_peak_8131	4.00	lobChIP_FOXA1_robot
chr2	85134887	85134888	MACS_peak_8132	5.00	lobChIP_FOXA1_robot
chr2	85187114	85187115	MACS_peak_8133	3.00	lobChIP_FOXA1_robot
chr2	85191007	85191008	MACS_peak_8134	9.00	lobChIP_FOXA1_robot
chr2	85552264	85552265	MACS_peak_8135	14.00	lobChIP_FOXA1_robot
chr2	85592861	85592862	MACS_peak_8136	7.00	lobChIP_FOXA1_robot
chr2	85647131	85647132	MACS_peak_8137	5.00	lobChIP_FOXA1_robot
chr2	85648611	85648612	MACS_peak_8138	34.00	lobChIP_FOXA1_robot
chr2	85696124	85696125	MACS_peak_8139	5.00	lobChIP_FOXA1_robot
chr2	85726383	85726384	MACS_peak_8140	4.00	lobChIP_FOXA1_robot
chr2	85774081	85774082	MACS_peak_8141	8.00	lobChIP_FOXA1_robot
chr2	85812106	85812107	MACS_peak_8142	3.00	lobChIP_FOXA1_robot
chr2	85814766	85814767	MACS_peak_8143	16.00	lobChIP_FOXA1_robot
chr2	85817409	85817410	MACS_peak_8144	6.00	lobChIP_FOXA1_robot
chr2	85969180	85969181	MACS_peak_8145	8.00	lobChIP_FOXA1_robot
chr2	85981086	85981087	MACS_peak_8146	4.00	lobChIP_FOXA1_robot
chr2	85988943	85988944	MACS_peak_8147	5.00	lobChIP_FOXA1_robot
chr2	86021342	86021343	MACS_peak_8148	4.00	lobChIP_FOXA1_robot
chr2	86033831	86033832	MACS_peak_8149	3.00	lobChIP_FOXA1_robot
chr2	86182180	86182181	MACS_peak_8150	5.00	lobChIP_FOXA1_robot
chr2	86668288	86668289	MACS_peak_8151	6.00	lobChIP_FOXA1_robot
chr2	86783807	86783808	MACS_peak_8152	26.00	lobChIP_FOXA1_robot
chr2	86860002	86860003	MACS_peak_8153	10.00	lobChIP_FOXA1_robot
chr2	86876335	86876336	MACS_peak_8154	4.00	lobChIP_FOXA1_robot
chr2	86933450	86933451	MACS_peak_8155	4.00	lobChIP_FOXA1_robot
chr2	87034453	87034454	MACS_peak_8156	3.00	lobChIP_FOXA1_robot
chr2	87957607	87957608	MACS_peak_8157	4.00	lobChIP_FOXA1_robot
chr2	88316083	88316084	MACS_peak_8158	5.00	lobChIP_FOXA1_robot
chr2	88360285	88360286	MACS_peak_8159	4.00	lobChIP_FOXA1_robot
chr2	88428070	88428071	MACS_peak_8160	47.00	lobChIP_FOXA1_robot
chr2	88431309	88431310	MACS_peak_8161	4.00	lobChIP_FOXA1_robot
chr2	88506581	88506582	MACS_peak_8162	12.00	lobChIP_FOXA1_robot
chr2	88510011	88510012	MACS_peak_8163	4.00	lobChIP_FOXA1_robot
chr2	88512134	88512135	MACS_peak_8164	6.00	lobChIP_FOXA1_robot
chr2	88629908	88629909	MACS_peak_8165	12.00	lobChIP_FOXA1_robot
chr2	88968579	88968580	MACS_peak_8166	4.00	lobChIP_FOXA1_robot
chr2	89006805	89006806	MACS_peak_8167	8.00	lobChIP_FOXA1_robot
chr2	91766489	91766490	MACS_peak_8168	4.00	lobChIP_FOXA1_robot
chr2	91805218	91805219	MACS_peak_8169	3.00	lobChIP_FOXA1_robot
chr2	91809539	91809540	MACS_peak_8170	26.00	lobChIP_FOXA1_robot
chr2	95916513	95916514	MACS_peak_8174	4.00	lobChIP_FOXA1_robot
chr2	96734001	96734002	MACS_peak_8175	6.00	lobChIP_FOXA1_robot
chr2	96853252	96853253	MACS_peak_8176	7.00	lobChIP_FOXA1_robot
chr2	96926306	96926307	MACS_peak_8177	13.00	lobChIP_FOXA1_robot
chr2	96927490	96927491	MACS_peak_8178	7.00	lobChIP_FOXA1_robot
chr2	97346522	97346523	MACS_peak_8179	5.00	lobChIP_FOXA1_robot
chr2	97405965	97405966	MACS_peak_8180	9.00	lobChIP_FOXA1_robot
chr2	97470928	97470929	MACS_peak_8181	4.00	lobChIP_FOXA1_robot
chr2	97485142	97485143	MACS_peak_8182	6.00	lobChIP_FOXA1_robot
chr2	98552967	98552968	MACS_peak_8183	6.00	lobChIP_FOXA1_robot
chr2	98583028	98583029	MACS_peak_8184	3.00	lobChIP_FOXA1_robot
chr2	98584728	98584729	MACS_peak_8185	3.00	lobChIP_FOXA1_robot
chr2	98701454	98701455	MACS_peak_8186	14.00	lobChIP_FOXA1_robot
chr2	99105376	99105377	MACS_peak_8187	7.00	lobChIP_FOXA1_robot
chr2	99321354	99321355	MACS_peak_8188	15.00	lobChIP_FOXA1_robot
chr2	99351664	99351665	MACS_peak_8189	11.00	lobChIP_FOXA1_robot
chr2	99353081	99353082	MACS_peak_8190	6.00	lobChIP_FOXA1_robot
chr2	99385327	99385328	MACS_peak_8191	11.00	lobChIP_FOXA1_robot
chr2	99412640	99412641	MACS_peak_8192	5.00	lobChIP_FOXA1_robot
chr2	99593572	99593573	MACS_peak_8193	4.00	lobChIP_FOXA1_robot
chr2	99895396	99895397	MACS_peak_8194	42.00	lobChIP_FOXA1_robot
chr2	100083614	100083615	MACS_peak_8195	5.00	lobChIP_FOXA1_robot
chr2	100351929	100351930	MACS_peak_8196	3.00	lobChIP_FOXA1_robot
chr2	100684384	100684385	MACS_peak_8197	58.00	lobChIP_FOXA1_robot
chr2	101223036	101223037	MACS_peak_8198	5.00	lobChIP_FOXA1_robot
chr2	101384428	101384429	MACS_peak_8199	4.00	lobChIP_FOXA1_robot
chr2	101396898	101396899	MACS_peak_8200	4.00	lobChIP_FOXA1_robot
chr2	101397323	101397324	MACS_peak_8201	6.00	lobChIP_FOXA1_robot
chr2	101413383	101413384	MACS_peak_8202	4.00	lobChIP_FOXA1_robot
chr2	101442236	101442237	MACS_peak_8203	4.00	lobChIP_FOXA1_robot
chr2	101492130	101492131	MACS_peak_8204	5.00	lobChIP_FOXA1_robot
chr2	101498224	101498225	MACS_peak_8205	5.00	lobChIP_FOXA1_robot
chr2	101520462	101520463	MACS_peak_8206	5.00	lobChIP_FOXA1_robot
chr2	101681865	101681866	MACS_peak_8207	6.00	lobChIP_FOXA1_robot
chr2	101682888	101682889	MACS_peak_8208	10.00	lobChIP_FOXA1_robot
chr2	101723300	101723301	MACS_peak_8209	18.00	lobChIP_FOXA1_robot
chr2	101727108	101727109	MACS_peak_8210	37.00	lobChIP_FOXA1_robot
chr2	101733907	101733908	MACS_peak_8211	62.00	lobChIP_FOXA1_robot
chr2	101741757	101741758	MACS_peak_8212	11.00	lobChIP_FOXA1_robot
chr2	101824179	101824180	MACS_peak_8213	7.00	lobChIP_FOXA1_robot
chr2	101880085	101880086	MACS_peak_8214	5.00	lobChIP_FOXA1_robot
chr2	101950041	101950042	MACS_peak_8215	14.00	lobChIP_FOXA1_robot
chr2	102173910	102173911	MACS_peak_8216	15.00	lobChIP_FOXA1_robot
chr2	102175495	102175496	MACS_peak_8217	5.00	lobChIP_FOXA1_robot
chr2	102183427	102183428	MACS_peak_8218	167.00	lobChIP_FOXA1_robot
chr2	102185602	102185603	MACS_peak_8219	15.00	lobChIP_FOXA1_robot
chr2	102272476	102272477	MACS_peak_8220	25.00	lobChIP_FOXA1_robot
chr2	102316430	102316431	MACS_peak_8221	5.00	lobChIP_FOXA1_robot
chr2	102348113	102348114	MACS_peak_8222	11.00	lobChIP_FOXA1_robot
chr2	102354831	102354832	MACS_peak_8223	4.00	lobChIP_FOXA1_robot
chr2	102357193	102357194	MACS_peak_8224	12.00	lobChIP_FOXA1_robot
chr2	102368620	102368621	MACS_peak_8225	7.00	lobChIP_FOXA1_robot
chr2	102371445	102371446	MACS_peak_8226	6.00	lobChIP_FOXA1_robot
chr2	102380518	102380519	MACS_peak_8227	25.00	lobChIP_FOXA1_robot
chr2	102384073	102384074	MACS_peak_8228	7.00	lobChIP_FOXA1_robot
chr2	102388599	102388600	MACS_peak_8229	7.00	lobChIP_FOXA1_robot
chr2	102419743	102419744	MACS_peak_8230	6.00	lobChIP_FOXA1_robot
chr2	102431402	102431403	MACS_peak_8231	5.00	lobChIP_FOXA1_robot
chr2	102467652	102467653	MACS_peak_8232	5.00	lobChIP_FOXA1_robot
chr2	102616140	102616141	MACS_peak_8233	6.00	lobChIP_FOXA1_robot
chr2	102647390	102647391	MACS_peak_8234	26.00	lobChIP_FOXA1_robot
chr2	102648992	102648993	MACS_peak_8235	5.00	lobChIP_FOXA1_robot
chr2	102650681	102650682	MACS_peak_8236	7.00	lobChIP_FOXA1_robot
chr2	102662948	102662949	MACS_peak_8237	17.00	lobChIP_FOXA1_robot
chr2	102674528	102674529	MACS_peak_8238	22.00	lobChIP_FOXA1_robot
chr2	102717012	102717013	MACS_peak_8239	5.00	lobChIP_FOXA1_robot
chr2	102717486	102717487	MACS_peak_8240	14.00	lobChIP_FOXA1_robot
chr2	102722906	102722907	MACS_peak_8241	5.00	lobChIP_FOXA1_robot
chr2	102724342	102724343	MACS_peak_8242	4.00	lobChIP_FOXA1_robot
chr2	102731828	102731829	MACS_peak_8243	10.00	lobChIP_FOXA1_robot
chr2	102772641	102772642	MACS_peak_8244	59.00	lobChIP_FOXA1_robot
chr2	102842182	102842183	MACS_peak_8245	6.00	lobChIP_FOXA1_robot
chr2	102857388	102857389	MACS_peak_8246	7.00	lobChIP_FOXA1_robot
chr2	103340741	103340742	MACS_peak_8247	5.00	lobChIP_FOXA1_robot
chr2	103414153	103414154	MACS_peak_8248	5.00	lobChIP_FOXA1_robot
chr2	104631275	104631276	MACS_peak_8249	4.00	lobChIP_FOXA1_robot
chr2	105093091	105093092	MACS_peak_8250	6.00	lobChIP_FOXA1_robot
chr2	105658058	105658059	MACS_peak_8251	3.00	lobChIP_FOXA1_robot
chr2	105956044	105956045	MACS_peak_8252	4.00	lobChIP_FOXA1_robot
chr2	106007667	106007668	MACS_peak_8253	5.00	lobChIP_FOXA1_robot
chr2	106012985	106012986	MACS_peak_8254	5.00	lobChIP_FOXA1_robot
chr2	106072291	106072292	MACS_peak_8255	3.00	lobChIP_FOXA1_robot
chr2	106100349	106100350	MACS_peak_8256	6.00	lobChIP_FOXA1_robot
chr2	106261001	106261002	MACS_peak_8257	6.00	lobChIP_FOXA1_robot
chr2	106439654	106439655	MACS_peak_8258	5.00	lobChIP_FOXA1_robot
chr2	106460444	106460445	MACS_peak_8259	3.00	lobChIP_FOXA1_robot
chr2	106527559	106527560	MACS_peak_8260	4.00	lobChIP_FOXA1_robot
chr2	106647586	106647587	MACS_peak_8261	7.00	lobChIP_FOXA1_robot
chr2	106651034	106651035	MACS_peak_8262	7.00	lobChIP_FOXA1_robot
chr2	106814511	106814512	MACS_peak_8263	5.00	lobChIP_FOXA1_robot
chr2	107148299	107148300	MACS_peak_8264	4.00	lobChIP_FOXA1_robot
chr2	108053956	108053957	MACS_peak_8265	4.00	lobChIP_FOXA1_robot
chr2	108326961	108326962	MACS_peak_8266	3.00	lobChIP_FOXA1_robot
chr2	108425238	108425239	MACS_peak_8267	7.00	lobChIP_FOXA1_robot
chr2	108809862	108809863	MACS_peak_8268	5.00	lobChIP_FOXA1_robot
chr2	108888685	108888686	MACS_peak_8269	6.00	lobChIP_FOXA1_robot
chr2	108986679	108986680	MACS_peak_8270	8.00	lobChIP_FOXA1_robot
chr2	109002308	109002309	MACS_peak_8271	10.00	lobChIP_FOXA1_robot
chr2	109029624	109029625	MACS_peak_8272	4.00	lobChIP_FOXA1_robot
chr2	109149666	109149667	MACS_peak_8273	15.00	lobChIP_FOXA1_robot
chr2	109428142	109428143	MACS_peak_8274	5.00	lobChIP_FOXA1_robot
chr2	109598820	109598821	MACS_peak_8275	3.00	lobChIP_FOXA1_robot
chr2	109636733	109636734	MACS_peak_8276	6.00	lobChIP_FOXA1_robot
chr2	109822386	109822387	MACS_peak_8277	4.00	lobChIP_FOXA1_robot
chr2	110316242	110316243	MACS_peak_8278	8.00	lobChIP_FOXA1_robot
chr2	110371040	110371041	MACS_peak_8279	4.00	lobChIP_FOXA1_robot
chr2	110402374	110402375	MACS_peak_8280	33.00	lobChIP_FOXA1_robot
chr2	110438497	110438498	MACS_peak_8281	8.00	lobChIP_FOXA1_robot
chr2	110448695	110448696	MACS_peak_8282	8.00	lobChIP_FOXA1_robot
chr2	110903891	110903892	MACS_peak_8283	11.00	lobChIP_FOXA1_robot
chr2	111421926	111421927	MACS_peak_8284	3.00	lobChIP_FOXA1_robot
chr2	111526337	111526338	MACS_peak_8285	16.00	lobChIP_FOXA1_robot
chr2	111569486	111569487	MACS_peak_8286	4.00	lobChIP_FOXA1_robot
chr2	111619379	111619380	MACS_peak_8287	4.00	lobChIP_FOXA1_robot
chr2	111765498	111765499	MACS_peak_8288	9.00	lobChIP_FOXA1_robot
chr2	111920585	111920586	MACS_peak_8289	3.00	lobChIP_FOXA1_robot
chr2	111936919	111936920	MACS_peak_8290	5.00	lobChIP_FOXA1_robot
chr2	112280131	112280132	MACS_peak_8291	8.00	lobChIP_FOXA1_robot
chr2	112286775	112286776	MACS_peak_8292	5.00	lobChIP_FOXA1_robot
chr2	112317127	112317128	MACS_peak_8293	6.00	lobChIP_FOXA1_robot
chr2	112374987	112374988	MACS_peak_8294	4.00	lobChIP_FOXA1_robot
chr2	112597617	112597618	MACS_peak_8295	7.00	lobChIP_FOXA1_robot
chr2	112676181	112676182	MACS_peak_8296	9.00	lobChIP_FOXA1_robot
chr2	112712745	112712746	MACS_peak_8297	6.00	lobChIP_FOXA1_robot
chr2	112732845	112732846	MACS_peak_8298	8.00	lobChIP_FOXA1_robot
chr2	112745056	112745057	MACS_peak_8299	12.00	lobChIP_FOXA1_robot
chr2	112927418	112927419	MACS_peak_8300	15.00	lobChIP_FOXA1_robot
chr2	113072612	113072613	MACS_peak_8301	25.00	lobChIP_FOXA1_robot
chr2	113401808	113401809	MACS_peak_8302	4.00	lobChIP_FOXA1_robot
chr2	113519349	113519350	MACS_peak_8303	11.00	lobChIP_FOXA1_robot
chr2	113561891	113561892	MACS_peak_8304	6.00	lobChIP_FOXA1_robot
chr2	113575757	113575758	MACS_peak_8305	14.00	lobChIP_FOXA1_robot
chr2	113846282	113846283	MACS_peak_8306	12.00	lobChIP_FOXA1_robot
chr2	113882927	113882928	MACS_peak_8307	4.00	lobChIP_FOXA1_robot
chr2	113884648	113884649	MACS_peak_8308	15.00	lobChIP_FOXA1_robot
chr2	113914201	113914202	MACS_peak_8309	5.00	lobChIP_FOXA1_robot
chr2	113923955	113923956	MACS_peak_8310	5.00	lobChIP_FOXA1_robot
chr2	113976069	113976070	MACS_peak_8311	4.00	lobChIP_FOXA1_robot
chr2	114576171	114576172	MACS_peak_8312	6.00	lobChIP_FOXA1_robot
chr2	114634275	114634276	MACS_peak_8313	4.00	lobChIP_FOXA1_robot
chr2	114635039	114635040	MACS_peak_8314	3.00	lobChIP_FOXA1_robot
chr2	114725302	114725303	MACS_peak_8315	4.00	lobChIP_FOXA1_robot
chr2	114735389	114735390	MACS_peak_8316	24.00	lobChIP_FOXA1_robot
chr2	114764137	114764138	MACS_peak_8317	6.00	lobChIP_FOXA1_robot
chr2	115988565	115988566	MACS_peak_8318	3.00	lobChIP_FOXA1_robot
chr2	116251190	116251191	MACS_peak_8319	4.00	lobChIP_FOXA1_robot
chr2	117910909	117910910	MACS_peak_8320	4.00	lobChIP_FOXA1_robot
chr2	118586702	118586703	MACS_peak_8321	4.00	lobChIP_FOXA1_robot
chr2	118691728	118691729	MACS_peak_8322	5.00	lobChIP_FOXA1_robot
chr2	118843512	118843513	MACS_peak_8323	33.00	lobChIP_FOXA1_robot
chr2	118843983	118843984	MACS_peak_8324	6.00	lobChIP_FOXA1_robot
chr2	118844735	118844736	MACS_peak_8325	4.00	lobChIP_FOXA1_robot
chr2	118845806	118845807	MACS_peak_8326	3.00	lobChIP_FOXA1_robot
chr2	118848810	118848811	MACS_peak_8327	5.00	lobChIP_FOXA1_robot
chr2	119079424	119079425	MACS_peak_8328	3.00	lobChIP_FOXA1_robot
chr2	119426740	119426741	MACS_peak_8329	6.00	lobChIP_FOXA1_robot
chr2	120109997	120109998	MACS_peak_8330	6.00	lobChIP_FOXA1_robot
chr2	120123821	120123822	MACS_peak_8331	4.00	lobChIP_FOXA1_robot
chr2	120183558	120183559	MACS_peak_8332	12.00	lobChIP_FOXA1_robot
chr2	120227722	120227723	MACS_peak_8333	3.00	lobChIP_FOXA1_robot
chr2	120435642	120435643	MACS_peak_8334	5.00	lobChIP_FOXA1_robot
chr2	120452670	120452671	MACS_peak_8335	7.00	lobChIP_FOXA1_robot
chr2	120523224	120523225	MACS_peak_8336	4.00	lobChIP_FOXA1_robot
chr2	120568021	120568022	MACS_peak_8337	7.00	lobChIP_FOXA1_robot
chr2	120753726	120753727	MACS_peak_8338	3.00	lobChIP_FOXA1_robot
chr2	120774995	120774996	MACS_peak_8339	5.00	lobChIP_FOXA1_robot
chr2	120824902	120824903	MACS_peak_8340	37.00	lobChIP_FOXA1_robot
chr2	120891991	120891992	MACS_peak_8341	6.00	lobChIP_FOXA1_robot
chr2	121015356	121015357	MACS_peak_8342	5.00	lobChIP_FOXA1_robot
chr2	121030557	121030558	MACS_peak_8343	7.00	lobChIP_FOXA1_robot
chr2	121106204	121106205	MACS_peak_8344	12.00	lobChIP_FOXA1_robot
chr2	121156882	121156883	MACS_peak_8345	12.00	lobChIP_FOXA1_robot
chr2	121199757	121199758	MACS_peak_8346	7.00	lobChIP_FOXA1_robot
chr2	121223814	121223815	MACS_peak_8347	42.00	lobChIP_FOXA1_robot
chr2	121228002	121228003	MACS_peak_8348	10.00	lobChIP_FOXA1_robot
chr2	121283083	121283084	MACS_peak_8349	3.00	lobChIP_FOXA1_robot
chr2	121298026	121298027	MACS_peak_8350	9.00	lobChIP_FOXA1_robot
chr2	121301040	121301041	MACS_peak_8351	4.00	lobChIP_FOXA1_robot
chr2	121312427	121312428	MACS_peak_8352	6.00	lobChIP_FOXA1_robot
chr2	121322379	121322380	MACS_peak_8353	33.00	lobChIP_FOXA1_robot
chr2	121377315	121377316	MACS_peak_8354	7.00	lobChIP_FOXA1_robot
chr2	121692744	121692745	MACS_peak_8355	6.00	lobChIP_FOXA1_robot
chr2	121772549	121772550	MACS_peak_8356	8.00	lobChIP_FOXA1_robot
chr2	121794085	121794086	MACS_peak_8357	25.00	lobChIP_FOXA1_robot
chr2	121867351	121867352	MACS_peak_8358	8.00	lobChIP_FOXA1_robot
chr2	122040642	122040643	MACS_peak_8359	6.00	lobChIP_FOXA1_robot
chr2	122113275	122113276	MACS_peak_8360	6.00	lobChIP_FOXA1_robot
chr2	122288644	122288645	MACS_peak_8361	9.00	lobChIP_FOXA1_robot
chr2	122325724	122325725	MACS_peak_8362	4.00	lobChIP_FOXA1_robot
chr2	122338492	122338493	MACS_peak_8363	6.00	lobChIP_FOXA1_robot
chr2	122355833	122355834	MACS_peak_8364	7.00	lobChIP_FOXA1_robot
chr2	122366741	122366742	MACS_peak_8365	6.00	lobChIP_FOXA1_robot
chr2	123190946	123190947	MACS_peak_8366	19.00	lobChIP_FOXA1_robot
chr2	123506519	123506520	MACS_peak_8367	4.00	lobChIP_FOXA1_robot
chr2	123508494	123508495	MACS_peak_8368	4.00	lobChIP_FOXA1_robot
chr2	123715936	123715937	MACS_peak_8369	4.00	lobChIP_FOXA1_robot
chr2	124691010	124691011	MACS_peak_8370	4.00	lobChIP_FOXA1_robot
chr2	125324259	125324260	MACS_peak_8371	7.00	lobChIP_FOXA1_robot
chr2	125328686	125328687	MACS_peak_8372	5.00	lobChIP_FOXA1_robot
chr2	125844304	125844305	MACS_peak_8373	9.00	lobChIP_FOXA1_robot
chr2	126651748	126651749	MACS_peak_8374	6.00	lobChIP_FOXA1_robot
chr2	127095189	127095190	MACS_peak_8375	4.00	lobChIP_FOXA1_robot
chr2	127317997	127317998	MACS_peak_8376	3.00	lobChIP_FOXA1_robot
chr2	127748513	127748514	MACS_peak_8377	6.00	lobChIP_FOXA1_robot
chr2	127754429	127754430	MACS_peak_8378	21.00	lobChIP_FOXA1_robot
chr2	127873506	127873507	MACS_peak_8379	4.00	lobChIP_FOXA1_robot
chr2	127984350	127984351	MACS_peak_8380	6.00	lobChIP_FOXA1_robot
chr2	128012536	128012537	MACS_peak_8381	3.00	lobChIP_FOXA1_robot
chr2	128115829	128115830	MACS_peak_8382	4.00	lobChIP_FOXA1_robot
chr2	128131640	128131641	MACS_peak_8383	4.00	lobChIP_FOXA1_robot
chr2	128143215	128143216	MACS_peak_8384	5.00	lobChIP_FOXA1_robot
chr2	128168984	128168985	MACS_peak_8385	5.00	lobChIP_FOXA1_robot
chr2	128169751	128169752	MACS_peak_8386	5.00	lobChIP_FOXA1_robot
chr2	128171559	128171560	MACS_peak_8387	13.00	lobChIP_FOXA1_robot
chr2	128175976	128175977	MACS_peak_8388	8.00	lobChIP_FOXA1_robot
chr2	128195977	128195978	MACS_peak_8389	5.00	lobChIP_FOXA1_robot
chr2	128212608	128212609	MACS_peak_8390	14.00	lobChIP_FOXA1_robot
chr2	128256304	128256305	MACS_peak_8391	3.00	lobChIP_FOXA1_robot
chr2	128493461	128493462	MACS_peak_8392	5.00	lobChIP_FOXA1_robot
chr2	128521495	128521496	MACS_peak_8393	4.00	lobChIP_FOXA1_robot
chr2	128542940	128542941	MACS_peak_8394	8.00	lobChIP_FOXA1_robot
chr2	128743042	128743043	MACS_peak_8395	8.00	lobChIP_FOXA1_robot
chr2	128919377	128919378	MACS_peak_8396	4.00	lobChIP_FOXA1_robot
chr2	128927861	128927862	MACS_peak_8397	13.00	lobChIP_FOXA1_robot
chr2	128936408	128936409	MACS_peak_8398	32.00	lobChIP_FOXA1_robot
chr2	129064197	129064198	MACS_peak_8399	4.00	lobChIP_FOXA1_robot
chr2	129067233	129067234	MACS_peak_8400	7.00	lobChIP_FOXA1_robot
chr2	129136834	129136835	MACS_peak_8401	9.00	lobChIP_FOXA1_robot
chr2	129157438	129157439	MACS_peak_8402	4.00	lobChIP_FOXA1_robot
chr2	129294924	129294925	MACS_peak_8403	4.00	lobChIP_FOXA1_robot
chr2	129352868	129352869	MACS_peak_8404	9.00	lobChIP_FOXA1_robot
chr2	130175038	130175039	MACS_peak_8405	4.00	lobChIP_FOXA1_robot
chr2	131502308	131502309	MACS_peak_8406	8.00	lobChIP_FOXA1_robot
chr2	131834423	131834424	MACS_peak_8407	4.00	lobChIP_FOXA1_robot
chr2	133028434	133028435	MACS_peak_8411	12.00	lobChIP_FOXA1_robot
chr2	134523561	134523562	MACS_peak_8412	5.00	lobChIP_FOXA1_robot
chr2	134753935	134753936	MACS_peak_8413	5.00	lobChIP_FOXA1_robot
chr2	134881841	134881842	MACS_peak_8414	4.00	lobChIP_FOXA1_robot
chr2	134890305	134890306	MACS_peak_8415	5.00	lobChIP_FOXA1_robot
chr2	134999732	134999733	MACS_peak_8416	4.00	lobChIP_FOXA1_robot
chr2	135117224	135117225	MACS_peak_8417	7.00	lobChIP_FOXA1_robot
chr2	135169902	135169903	MACS_peak_8418	23.00	lobChIP_FOXA1_robot
chr2	135190152	135190153	MACS_peak_8419	15.00	lobChIP_FOXA1_robot
chr2	135511682	135511683	MACS_peak_8420	3.00	lobChIP_FOXA1_robot
chr2	135581194	135581195	MACS_peak_8421	8.00	lobChIP_FOXA1_robot
chr2	135655444	135655445	MACS_peak_8422	4.00	lobChIP_FOXA1_robot
chr2	135674927	135674928	MACS_peak_8423	4.00	lobChIP_FOXA1_robot
chr2	135849417	135849418	MACS_peak_8424	22.00	lobChIP_FOXA1_robot
chr2	135908411	135908412	MACS_peak_8425	5.00	lobChIP_FOXA1_robot
chr2	136034261	136034262	MACS_peak_8426	6.00	lobChIP_FOXA1_robot
chr2	136269837	136269838	MACS_peak_8427	4.00	lobChIP_FOXA1_robot
chr2	136781114	136781115	MACS_peak_8428	9.00	lobChIP_FOXA1_robot
chr2	136828555	136828556	MACS_peak_8429	3.00	lobChIP_FOXA1_robot
chr2	137104210	137104211	MACS_peak_8430	5.00	lobChIP_FOXA1_robot
chr2	138670075	138670076	MACS_peak_8431	4.00	lobChIP_FOXA1_robot
chr2	138686599	138686600	MACS_peak_8432	30.00	lobChIP_FOXA1_robot
chr2	138700747	138700748	MACS_peak_8433	29.00	lobChIP_FOXA1_robot
chr2	138714754	138714755	MACS_peak_8434	6.00	lobChIP_FOXA1_robot
chr2	138721913	138721914	MACS_peak_8435	33.00	lobChIP_FOXA1_robot
chr2	138731087	138731088	MACS_peak_8436	3.00	lobChIP_FOXA1_robot
chr2	138736557	138736558	MACS_peak_8437	3.00	lobChIP_FOXA1_robot
chr2	138835714	138835715	MACS_peak_8438	4.00	lobChIP_FOXA1_robot
chr2	138839453	138839454	MACS_peak_8439	4.00	lobChIP_FOXA1_robot
chr2	138868248	138868249	MACS_peak_8440	13.00	lobChIP_FOXA1_robot
chr2	138934811	138934812	MACS_peak_8441	4.00	lobChIP_FOXA1_robot
chr2	138955674	138955675	MACS_peak_8442	3.00	lobChIP_FOXA1_robot
chr2	139033652	139033653	MACS_peak_8443	5.00	lobChIP_FOXA1_robot
chr2	139039781	139039782	MACS_peak_8444	7.00	lobChIP_FOXA1_robot
chr2	139119473	139119474	MACS_peak_8445	4.00	lobChIP_FOXA1_robot
chr2	139131710	139131711	MACS_peak_8446	4.00	lobChIP_FOXA1_robot
chr2	139179034	139179035	MACS_peak_8447	5.00	lobChIP_FOXA1_robot
chr2	139195100	139195101	MACS_peak_8448	5.00	lobChIP_FOXA1_robot
chr2	139233188	139233189	MACS_peak_8449	10.00	lobChIP_FOXA1_robot
chr2	139248981	139248982	MACS_peak_8450	25.00	lobChIP_FOXA1_robot
chr2	139249681	139249682	MACS_peak_8451	5.00	lobChIP_FOXA1_robot
chr2	139264991	139264992	MACS_peak_8452	6.00	lobChIP_FOXA1_robot
chr2	139270413	139270414	MACS_peak_8453	6.00	lobChIP_FOXA1_robot
chr2	139477973	139477974	MACS_peak_8454	3.00	lobChIP_FOXA1_robot
chr2	139482733	139482734	MACS_peak_8455	15.00	lobChIP_FOXA1_robot
chr2	139535738	139535739	MACS_peak_8456	6.00	lobChIP_FOXA1_robot
chr2	139572298	139572299	MACS_peak_8457	5.00	lobChIP_FOXA1_robot
chr2	139923456	139923457	MACS_peak_8458	4.00	lobChIP_FOXA1_robot
chr2	141101294	141101295	MACS_peak_8459	4.00	lobChIP_FOXA1_robot
chr2	141462249	141462250	MACS_peak_8460	4.00	lobChIP_FOXA1_robot
chr2	142275268	142275269	MACS_peak_8461	5.00	lobChIP_FOXA1_robot
chr2	142807493	142807494	MACS_peak_8462	3.00	lobChIP_FOXA1_robot
chr2	143030317	143030318	MACS_peak_8463	3.00	lobChIP_FOXA1_robot
chr2	143360422	143360423	MACS_peak_8464	4.00	lobChIP_FOXA1_robot
chr2	143476780	143476781	MACS_peak_8465	4.00	lobChIP_FOXA1_robot
chr2	143582122	143582123	MACS_peak_8466	4.00	lobChIP_FOXA1_robot
chr2	143621308	143621309	MACS_peak_8467	26.00	lobChIP_FOXA1_robot
chr2	143628169	143628170	MACS_peak_8468	3.00	lobChIP_FOXA1_robot
chr2	143632172	143632173	MACS_peak_8469	13.00	lobChIP_FOXA1_robot
chr2	143635982	143635983	MACS_peak_8470	4.00	lobChIP_FOXA1_robot
chr2	143709228	143709229	MACS_peak_8471	17.00	lobChIP_FOXA1_robot
chr2	143710498	143710499	MACS_peak_8472	4.00	lobChIP_FOXA1_robot
chr2	143738289	143738290	MACS_peak_8473	4.00	lobChIP_FOXA1_robot
chr2	144051814	144051815	MACS_peak_8474	15.00	lobChIP_FOXA1_robot
chr2	145222438	145222439	MACS_peak_8475	3.00	lobChIP_FOXA1_robot
chr2	146318944	146318945	MACS_peak_8476	5.00	lobChIP_FOXA1_robot
chr2	146509967	146509968	MACS_peak_8477	3.00	lobChIP_FOXA1_robot
chr2	146512988	146512989	MACS_peak_8478	5.00	lobChIP_FOXA1_robot
chr2	146519085	146519086	MACS_peak_8479	34.00	lobChIP_FOXA1_robot
chr2	146535183	146535184	MACS_peak_8480	5.00	lobChIP_FOXA1_robot
chr2	146558171	146558172	MACS_peak_8481	29.00	lobChIP_FOXA1_robot
chr2	146580955	146580956	MACS_peak_8482	24.00	lobChIP_FOXA1_robot
chr2	146603012	146603013	MACS_peak_8483	8.00	lobChIP_FOXA1_robot
chr2	146603674	146603675	MACS_peak_8484	9.00	lobChIP_FOXA1_robot
chr2	146972157	146972158	MACS_peak_8485	5.00	lobChIP_FOXA1_robot
chr2	147212568	147212569	MACS_peak_8486	5.00	lobChIP_FOXA1_robot
chr2	148173847	148173848	MACS_peak_8487	4.00	lobChIP_FOXA1_robot
chr2	148567272	148567273	MACS_peak_8488	7.00	lobChIP_FOXA1_robot
chr2	148684859	148684860	MACS_peak_8489	16.00	lobChIP_FOXA1_robot
chr2	148696993	148696994	MACS_peak_8490	4.00	lobChIP_FOXA1_robot
chr2	148890757	148890758	MACS_peak_8491	4.00	lobChIP_FOXA1_robot
chr2	149050319	149050320	MACS_peak_8492	4.00	lobChIP_FOXA1_robot
chr2	149168681	149168682	MACS_peak_8493	6.00	lobChIP_FOXA1_robot
chr2	149174286	149174287	MACS_peak_8494	3.00	lobChIP_FOXA1_robot
chr2	149403061	149403062	MACS_peak_8495	4.00	lobChIP_FOXA1_robot
chr2	149414305	149414306	MACS_peak_8496	4.00	lobChIP_FOXA1_robot
chr2	149504772	149504773	MACS_peak_8497	7.00	lobChIP_FOXA1_robot
chr2	149877098	149877099	MACS_peak_8498	6.00	lobChIP_FOXA1_robot
chr2	150186826	150186827	MACS_peak_8499	4.00	lobChIP_FOXA1_robot
chr2	150419131	150419132	MACS_peak_8500	6.00	lobChIP_FOXA1_robot
chr2	150846173	150846174	MACS_peak_8501	12.00	lobChIP_FOXA1_robot
chr2	151071216	151071217	MACS_peak_8502	4.00	lobChIP_FOXA1_robot
chr2	152190760	152190761	MACS_peak_8503	3.00	lobChIP_FOXA1_robot
chr2	152266448	152266449	MACS_peak_8504	5.00	lobChIP_FOXA1_robot
chr2	152495336	152495337	MACS_peak_8505	5.00	lobChIP_FOXA1_robot
chr2	152498655	152498656	MACS_peak_8506	5.00	lobChIP_FOXA1_robot
chr2	152679271	152679272	MACS_peak_8507	3.00	lobChIP_FOXA1_robot
chr2	152706429	152706430	MACS_peak_8508	4.00	lobChIP_FOXA1_robot
chr2	152709711	152709712	MACS_peak_8509	24.00	lobChIP_FOXA1_robot
chr2	152846059	152846060	MACS_peak_8510	3.00	lobChIP_FOXA1_robot
chr2	152852397	152852398	MACS_peak_8511	35.00	lobChIP_FOXA1_robot
chr2	152853171	152853172	MACS_peak_8512	3.00	lobChIP_FOXA1_robot
chr2	152882436	152882437	MACS_peak_8513	3.00	lobChIP_FOXA1_robot
chr2	153199368	153199369	MACS_peak_8514	4.00	lobChIP_FOXA1_robot
chr2	153201308	153201309	MACS_peak_8515	41.00	lobChIP_FOXA1_robot
chr2	153216326	153216327	MACS_peak_8516	18.00	lobChIP_FOXA1_robot
chr2	153234713	153234714	MACS_peak_8517	9.00	lobChIP_FOXA1_robot
chr2	153250111	153250112	MACS_peak_8518	3.00	lobChIP_FOXA1_robot
chr2	153261949	153261950	MACS_peak_8519	6.00	lobChIP_FOXA1_robot
chr2	153271868	153271869	MACS_peak_8520	4.00	lobChIP_FOXA1_robot
chr2	153282751	153282752	MACS_peak_8521	17.00	lobChIP_FOXA1_robot
chr2	153330098	153330099	MACS_peak_8522	4.00	lobChIP_FOXA1_robot
chr2	153338644	153338645	MACS_peak_8523	9.00	lobChIP_FOXA1_robot
chr2	153342873	153342874	MACS_peak_8524	27.00	lobChIP_FOXA1_robot
chr2	153506630	153506631	MACS_peak_8525	3.00	lobChIP_FOXA1_robot
chr2	154038333	154038334	MACS_peak_8526	4.00	lobChIP_FOXA1_robot
chr2	154691406	154691407	MACS_peak_8527	4.00	lobChIP_FOXA1_robot
chr2	155251280	155251281	MACS_peak_8528	4.00	lobChIP_FOXA1_robot
chr2	155304329	155304330	MACS_peak_8529	6.00	lobChIP_FOXA1_robot
chr2	155306544	155306545	MACS_peak_8530	21.00	lobChIP_FOXA1_robot
chr2	155319379	155319380	MACS_peak_8531	4.00	lobChIP_FOXA1_robot
chr2	155689440	155689441	MACS_peak_8532	5.00	lobChIP_FOXA1_robot
chr2	156030144	156030145	MACS_peak_8533	3.00	lobChIP_FOXA1_robot
chr2	156672620	156672621	MACS_peak_8534	3.00	lobChIP_FOXA1_robot
chr2	156832334	156832335	MACS_peak_8535	5.00	lobChIP_FOXA1_robot
chr2	157045860	157045861	MACS_peak_8536	11.00	lobChIP_FOXA1_robot
chr2	157907540	157907541	MACS_peak_8537	3.00	lobChIP_FOXA1_robot
chr2	158695236	158695237	MACS_peak_8538	6.00	lobChIP_FOXA1_robot
chr2	158913712	158913713	MACS_peak_8539	4.00	lobChIP_FOXA1_robot
chr2	158936393	158936394	MACS_peak_8540	7.00	lobChIP_FOXA1_robot
chr2	158983993	158983994	MACS_peak_8541	3.00	lobChIP_FOXA1_robot
chr2	158987649	158987650	MACS_peak_8542	19.00	lobChIP_FOXA1_robot
chr2	158988693	158988694	MACS_peak_8543	18.00	lobChIP_FOXA1_robot
chr2	159046199	159046200	MACS_peak_8544	21.00	lobChIP_FOXA1_robot
chr2	159047809	159047810	MACS_peak_8545	14.00	lobChIP_FOXA1_robot
chr2	159089178	159089179	MACS_peak_8546	16.00	lobChIP_FOXA1_robot
chr2	159314835	159314836	MACS_peak_8547	3.00	lobChIP_FOXA1_robot
chr2	159359833	159359834	MACS_peak_8548	8.00	lobChIP_FOXA1_robot
chr2	159395965	159395966	MACS_peak_8549	4.00	lobChIP_FOXA1_robot
chr2	159443282	159443283	MACS_peak_8550	6.00	lobChIP_FOXA1_robot
chr2	159456026	159456027	MACS_peak_8551	3.00	lobChIP_FOXA1_robot
chr2	159580580	159580581	MACS_peak_8552	7.00	lobChIP_FOXA1_robot
chr2	159596146	159596147	MACS_peak_8553	5.00	lobChIP_FOXA1_robot
chr2	159806014	159806015	MACS_peak_8554	14.00	lobChIP_FOXA1_robot
chr2	159851666	159851667	MACS_peak_8555	37.00	lobChIP_FOXA1_robot
chr2	159853715	159853716	MACS_peak_8556	5.00	lobChIP_FOXA1_robot
chr2	159855623	159855624	MACS_peak_8557	34.00	lobChIP_FOXA1_robot
chr2	159867112	159867113	MACS_peak_8558	3.00	lobChIP_FOXA1_robot
chr2	159920397	159920398	MACS_peak_8559	3.00	lobChIP_FOXA1_robot
chr2	159981055	159981056	MACS_peak_8560	5.00	lobChIP_FOXA1_robot
chr2	160146897	160146898	MACS_peak_8561	4.00	lobChIP_FOXA1_robot
chr2	160259519	160259520	MACS_peak_8562	3.00	lobChIP_FOXA1_robot
chr2	160471563	160471564	MACS_peak_8563	5.00	lobChIP_FOXA1_robot
chr2	160655029	160655030	MACS_peak_8564	12.00	lobChIP_FOXA1_robot
chr2	160656623	160656624	MACS_peak_8565	6.00	lobChIP_FOXA1_robot
chr2	160730821	160730822	MACS_peak_8566	7.00	lobChIP_FOXA1_robot
chr2	161137843	161137844	MACS_peak_8567	4.00	lobChIP_FOXA1_robot
chr2	161186050	161186051	MACS_peak_8568	5.00	lobChIP_FOXA1_robot
chr2	161290422	161290423	MACS_peak_8569	10.00	lobChIP_FOXA1_robot
chr2	161290986	161290987	MACS_peak_8570	10.00	lobChIP_FOXA1_robot
chr2	161310738	161310739	MACS_peak_8571	5.00	lobChIP_FOXA1_robot
chr2	161348853	161348854	MACS_peak_8572	4.00	lobChIP_FOXA1_robot
chr2	161410972	161410973	MACS_peak_8573	5.00	lobChIP_FOXA1_robot
chr2	161972488	161972489	MACS_peak_8574	11.00	lobChIP_FOXA1_robot
chr2	161991938	161991939	MACS_peak_8575	13.00	lobChIP_FOXA1_robot
chr2	162016605	162016606	MACS_peak_8576	21.00	lobChIP_FOXA1_robot
chr2	162017507	162017508	MACS_peak_8577	7.00	lobChIP_FOXA1_robot
chr2	162214180	162214181	MACS_peak_8578	6.00	lobChIP_FOXA1_robot
chr2	162701533	162701534	MACS_peak_8579	16.00	lobChIP_FOXA1_robot
chr2	162941927	162941928	MACS_peak_8580	5.00	lobChIP_FOXA1_robot
chr2	162964249	162964250	MACS_peak_8581	4.00	lobChIP_FOXA1_robot
chr2	162969110	162969111	MACS_peak_8582	5.00	lobChIP_FOXA1_robot
chr2	163349457	163349458	MACS_peak_8583	4.00	lobChIP_FOXA1_robot
chr2	163356725	163356726	MACS_peak_8584	5.00	lobChIP_FOXA1_robot
chr2	163431112	163431113	MACS_peak_8585	7.00	lobChIP_FOXA1_robot
chr2	163431420	163431421	MACS_peak_8586	4.00	lobChIP_FOXA1_robot
chr2	163436704	163436705	MACS_peak_8587	13.00	lobChIP_FOXA1_robot
chr2	163439152	163439153	MACS_peak_8588	69.00	lobChIP_FOXA1_robot
chr2	163464622	163464623	MACS_peak_8589	5.00	lobChIP_FOXA1_robot
chr2	163566972	163566973	MACS_peak_8590	6.00	lobChIP_FOXA1_robot
chr2	164272142	164272143	MACS_peak_8591	4.00	lobChIP_FOXA1_robot
chr2	164331016	164331017	MACS_peak_8592	18.00	lobChIP_FOXA1_robot
chr2	164428125	164428126	MACS_peak_8593	17.00	lobChIP_FOXA1_robot
chr2	164524255	164524256	MACS_peak_8594	7.00	lobChIP_FOXA1_robot
chr2	164591982	164591983	MACS_peak_8595	20.00	lobChIP_FOXA1_robot
chr2	164629752	164629753	MACS_peak_8596	3.00	lobChIP_FOXA1_robot
chr2	164800165	164800166	MACS_peak_8597	5.00	lobChIP_FOXA1_robot
chr2	165203735	165203736	MACS_peak_8598	4.00	lobChIP_FOXA1_robot
chr2	165381335	165381336	MACS_peak_8599	5.00	lobChIP_FOXA1_robot
chr2	165416914	165416915	MACS_peak_8600	9.00	lobChIP_FOXA1_robot
chr2	165424329	165424330	MACS_peak_8601	9.00	lobChIP_FOXA1_robot
chr2	165456697	165456698	MACS_peak_8602	5.00	lobChIP_FOXA1_robot
chr2	165520234	165520235	MACS_peak_8603	5.00	lobChIP_FOXA1_robot
chr2	165550135	165550136	MACS_peak_8604	27.00	lobChIP_FOXA1_robot
chr2	165568057	165568058	MACS_peak_8605	14.00	lobChIP_FOXA1_robot
chr2	165599408	165599409	MACS_peak_8606	6.00	lobChIP_FOXA1_robot
chr2	165614620	165614621	MACS_peak_8607	8.00	lobChIP_FOXA1_robot
chr2	165667462	165667463	MACS_peak_8608	29.00	lobChIP_FOXA1_robot
chr2	165675108	165675109	MACS_peak_8609	8.00	lobChIP_FOXA1_robot
chr2	165697001	165697002	MACS_peak_8610	6.00	lobChIP_FOXA1_robot
chr2	165716659	165716660	MACS_peak_8611	3.00	lobChIP_FOXA1_robot
chr2	165729635	165729636	MACS_peak_8612	4.00	lobChIP_FOXA1_robot
chr2	165735309	165735310	MACS_peak_8613	6.00	lobChIP_FOXA1_robot
chr2	165744037	165744038	MACS_peak_8614	8.00	lobChIP_FOXA1_robot
chr2	165744617	165744618	MACS_peak_8615	3.00	lobChIP_FOXA1_robot
chr2	165755140	165755141	MACS_peak_8616	4.00	lobChIP_FOXA1_robot
chr2	165768582	165768583	MACS_peak_8617	9.00	lobChIP_FOXA1_robot
chr2	165790420	165790421	MACS_peak_8618	22.00	lobChIP_FOXA1_robot
chr2	165791670	165791671	MACS_peak_8619	7.00	lobChIP_FOXA1_robot
chr2	165794733	165794734	MACS_peak_8620	10.00	lobChIP_FOXA1_robot
chr2	165967065	165967066	MACS_peak_8621	16.00	lobChIP_FOXA1_robot
chr2	165971980	165971981	MACS_peak_8622	6.00	lobChIP_FOXA1_robot
chr2	166623163	166623164	MACS_peak_8623	5.00	lobChIP_FOXA1_robot
chr2	166634854	166634855	MACS_peak_8624	4.00	lobChIP_FOXA1_robot
chr2	166731277	166731278	MACS_peak_8625	3.00	lobChIP_FOXA1_robot
chr2	166801925	166801926	MACS_peak_8626	3.00	lobChIP_FOXA1_robot
chr2	166984329	166984330	MACS_peak_8627	3.00	lobChIP_FOXA1_robot
chr2	166991915	166991916	MACS_peak_8628	30.00	lobChIP_FOXA1_robot
chr2	167013588	167013589	MACS_peak_8629	4.00	lobChIP_FOXA1_robot
chr2	167086374	167086375	MACS_peak_8630	13.00	lobChIP_FOXA1_robot
chr2	167090764	167090765	MACS_peak_8631	7.00	lobChIP_FOXA1_robot
chr2	167191887	167191888	MACS_peak_8632	4.00	lobChIP_FOXA1_robot
chr2	167218009	167218010	MACS_peak_8633	4.00	lobChIP_FOXA1_robot
chr2	167228878	167228879	MACS_peak_8634	16.00	lobChIP_FOXA1_robot
chr2	167275755	167275756	MACS_peak_8635	6.00	lobChIP_FOXA1_robot
chr2	167465880	167465881	MACS_peak_8636	7.00	lobChIP_FOXA1_robot
chr2	167471307	167471308	MACS_peak_8637	5.00	lobChIP_FOXA1_robot
chr2	167960104	167960105	MACS_peak_8638	4.00	lobChIP_FOXA1_robot
chr2	168367060	168367061	MACS_peak_8639	21.00	lobChIP_FOXA1_robot
chr2	168465208	168465209	MACS_peak_8640	4.00	lobChIP_FOXA1_robot
chr2	168651214	168651215	MACS_peak_8641	28.00	lobChIP_FOXA1_robot
chr2	168672025	168672026	MACS_peak_8642	25.00	lobChIP_FOXA1_robot
chr2	168673571	168673572	MACS_peak_8643	15.00	lobChIP_FOXA1_robot
chr2	168684271	168684272	MACS_peak_8644	9.00	lobChIP_FOXA1_robot
chr2	169006437	169006438	MACS_peak_8645	5.00	lobChIP_FOXA1_robot
chr2	169023719	169023720	MACS_peak_8646	3.00	lobChIP_FOXA1_robot
chr2	169037793	169037794	MACS_peak_8647	12.00	lobChIP_FOXA1_robot
chr2	169042156	169042157	MACS_peak_8648	6.00	lobChIP_FOXA1_robot
chr2	169047406	169047407	MACS_peak_8649	4.00	lobChIP_FOXA1_robot
chr2	169053656	169053657	MACS_peak_8650	4.00	lobChIP_FOXA1_robot
chr2	169055266	169055267	MACS_peak_8651	5.00	lobChIP_FOXA1_robot
chr2	169067794	169067795	MACS_peak_8652	4.00	lobChIP_FOXA1_robot
chr2	169077888	169077889	MACS_peak_8653	12.00	lobChIP_FOXA1_robot
chr2	169129000	169129001	MACS_peak_8654	8.00	lobChIP_FOXA1_robot
chr2	169135268	169135269	MACS_peak_8655	10.00	lobChIP_FOXA1_robot
chr2	169324421	169324422	MACS_peak_8656	8.00	lobChIP_FOXA1_robot
chr2	169349922	169349923	MACS_peak_8657	5.00	lobChIP_FOXA1_robot
chr2	169351030	169351031	MACS_peak_8658	8.00	lobChIP_FOXA1_robot
chr2	169753957	169753958	MACS_peak_8659	4.00	lobChIP_FOXA1_robot
chr2	169831696	169831697	MACS_peak_8660	6.00	lobChIP_FOXA1_robot
chr2	169868854	169868855	MACS_peak_8661	5.00	lobChIP_FOXA1_robot
chr2	170323344	170323345	MACS_peak_8662	6.00	lobChIP_FOXA1_robot
chr2	170323902	170323903	MACS_peak_8663	13.00	lobChIP_FOXA1_robot
chr2	170342571	170342572	MACS_peak_8664	6.00	lobChIP_FOXA1_robot
chr2	170418259	170418260	MACS_peak_8665	7.00	lobChIP_FOXA1_robot
chr2	170430747	170430748	MACS_peak_8666	5.00	lobChIP_FOXA1_robot
chr2	170540299	170540300	MACS_peak_8667	14.00	lobChIP_FOXA1_robot
chr2	170572216	170572217	MACS_peak_8668	16.00	lobChIP_FOXA1_robot
chr2	170574041	170574042	MACS_peak_8669	7.00	lobChIP_FOXA1_robot
chr2	170618361	170618362	MACS_peak_8670	5.00	lobChIP_FOXA1_robot
chr2	170672615	170672616	MACS_peak_8671	6.00	lobChIP_FOXA1_robot
chr2	170748192	170748193	MACS_peak_8672	7.00	lobChIP_FOXA1_robot
chr2	170860088	170860089	MACS_peak_8673	5.00	lobChIP_FOXA1_robot
chr2	171013775	171013776	MACS_peak_8674	14.00	lobChIP_FOXA1_robot
chr2	171183153	171183154	MACS_peak_8675	6.00	lobChIP_FOXA1_robot
chr2	171312797	171312798	MACS_peak_8676	17.00	lobChIP_FOXA1_robot
chr2	171386915	171386916	MACS_peak_8677	15.00	lobChIP_FOXA1_robot
chr2	171535937	171535938	MACS_peak_8678	3.00	lobChIP_FOXA1_robot
chr2	171568711	171568712	MACS_peak_8679	11.00	lobChIP_FOXA1_robot
chr2	171571650	171571651	MACS_peak_8680	42.00	lobChIP_FOXA1_robot
chr2	171623425	171623426	MACS_peak_8681	10.00	lobChIP_FOXA1_robot
chr2	171936091	171936092	MACS_peak_8682	4.00	lobChIP_FOXA1_robot
chr2	171946040	171946041	MACS_peak_8683	6.00	lobChIP_FOXA1_robot
chr2	171959625	171959626	MACS_peak_8684	7.00	lobChIP_FOXA1_robot
chr2	172006558	172006559	MACS_peak_8685	5.00	lobChIP_FOXA1_robot
chr2	172023306	172023307	MACS_peak_8686	10.00	lobChIP_FOXA1_robot
chr2	172024348	172024349	MACS_peak_8687	8.00	lobChIP_FOXA1_robot
chr2	172128641	172128642	MACS_peak_8688	4.00	lobChIP_FOXA1_robot
chr2	172221206	172221207	MACS_peak_8689	3.00	lobChIP_FOXA1_robot
chr2	172546897	172546898	MACS_peak_8690	3.00	lobChIP_FOXA1_robot
chr2	172585991	172585992	MACS_peak_8691	24.00	lobChIP_FOXA1_robot
chr2	172615482	172615483	MACS_peak_8692	3.00	lobChIP_FOXA1_robot
chr2	172629101	172629102	MACS_peak_8693	10.00	lobChIP_FOXA1_robot
chr2	172664704	172664705	MACS_peak_8694	24.00	lobChIP_FOXA1_robot
chr2	172746765	172746766	MACS_peak_8695	5.00	lobChIP_FOXA1_robot
chr2	172749842	172749843	MACS_peak_8696	5.00	lobChIP_FOXA1_robot
chr2	172893416	172893417	MACS_peak_8697	5.00	lobChIP_FOXA1_robot
chr2	172907120	172907121	MACS_peak_8698	5.00	lobChIP_FOXA1_robot
chr2	172914794	172914795	MACS_peak_8699	3.00	lobChIP_FOXA1_robot
chr2	172917178	172917179	MACS_peak_8700	11.00	lobChIP_FOXA1_robot
chr2	172938500	172938501	MACS_peak_8701	30.00	lobChIP_FOXA1_robot
chr2	172983968	172983969	MACS_peak_8702	6.00	lobChIP_FOXA1_robot
chr2	173099853	173099854	MACS_peak_8703	5.00	lobChIP_FOXA1_robot
chr2	173194971	173194972	MACS_peak_8704	7.00	lobChIP_FOXA1_robot
chr2	173205942	173205943	MACS_peak_8705	10.00	lobChIP_FOXA1_robot
chr2	173293442	173293443	MACS_peak_8706	3.00	lobChIP_FOXA1_robot
chr2	173810363	173810364	MACS_peak_8707	4.00	lobChIP_FOXA1_robot
chr2	173903178	173903179	MACS_peak_8708	4.00	lobChIP_FOXA1_robot
chr2	173913774	173913775	MACS_peak_8709	14.00	lobChIP_FOXA1_robot
chr2	173917461	173917462	MACS_peak_8710	5.00	lobChIP_FOXA1_robot
chr2	173918198	173918199	MACS_peak_8711	8.00	lobChIP_FOXA1_robot
chr2	173953039	173953040	MACS_peak_8712	9.00	lobChIP_FOXA1_robot
chr2	173974253	173974254	MACS_peak_8713	5.00	lobChIP_FOXA1_robot
chr2	174091896	174091897	MACS_peak_8714	4.00	lobChIP_FOXA1_robot
chr2	174103268	174103269	MACS_peak_8715	4.00	lobChIP_FOXA1_robot
chr2	174191569	174191570	MACS_peak_8716	4.00	lobChIP_FOXA1_robot
chr2	174212215	174212216	MACS_peak_8717	5.00	lobChIP_FOXA1_robot
chr2	174328460	174328461	MACS_peak_8718	6.00	lobChIP_FOXA1_robot
chr2	174642788	174642789	MACS_peak_8719	11.00	lobChIP_FOXA1_robot
chr2	174823139	174823140	MACS_peak_8720	6.00	lobChIP_FOXA1_robot
chr2	174831549	174831550	MACS_peak_8721	5.00	lobChIP_FOXA1_robot
chr2	174888917	174888918	MACS_peak_8722	3.00	lobChIP_FOXA1_robot
chr2	174916698	174916699	MACS_peak_8723	8.00	lobChIP_FOXA1_robot
chr2	174981723	174981724	MACS_peak_8724	3.00	lobChIP_FOXA1_robot
chr2	175039633	175039634	MACS_peak_8725	6.00	lobChIP_FOXA1_robot
chr2	175098678	175098679	MACS_peak_8726	25.00	lobChIP_FOXA1_robot
chr2	175206225	175206226	MACS_peak_8727	5.00	lobChIP_FOXA1_robot
chr2	175260410	175260411	MACS_peak_8728	4.00	lobChIP_FOXA1_robot
chr2	175293143	175293144	MACS_peak_8729	12.00	lobChIP_FOXA1_robot
chr2	175312248	175312249	MACS_peak_8730	5.00	lobChIP_FOXA1_robot
chr2	175481938	175481939	MACS_peak_8731	5.00	lobChIP_FOXA1_robot
chr2	175893321	175893322	MACS_peak_8732	11.00	lobChIP_FOXA1_robot
chr2	175901474	175901475	MACS_peak_8733	9.00	lobChIP_FOXA1_robot
chr2	175927916	175927917	MACS_peak_8734	53.00	lobChIP_FOXA1_robot
chr2	176032852	176032853	MACS_peak_8735	8.00	lobChIP_FOXA1_robot
chr2	176326754	176326755	MACS_peak_8736	6.00	lobChIP_FOXA1_robot
chr2	176471395	176471396	MACS_peak_8737	27.00	lobChIP_FOXA1_robot
chr2	176484407	176484408	MACS_peak_8738	5.00	lobChIP_FOXA1_robot
chr2	176615118	176615119	MACS_peak_8739	5.00	lobChIP_FOXA1_robot
chr2	177052165	177052166	MACS_peak_8740	13.00	lobChIP_FOXA1_robot
chr2	177182232	177182233	MACS_peak_8741	4.00	lobChIP_FOXA1_robot
chr2	178033091	178033092	MACS_peak_8742	3.00	lobChIP_FOXA1_robot
chr2	178035083	178035084	MACS_peak_8743	6.00	lobChIP_FOXA1_robot
chr2	178044290	178044291	MACS_peak_8744	5.00	lobChIP_FOXA1_robot
chr2	178052513	178052514	MACS_peak_8745	4.00	lobChIP_FOXA1_robot
chr2	178053810	178053811	MACS_peak_8746	12.00	lobChIP_FOXA1_robot
chr2	178068207	178068208	MACS_peak_8747	6.00	lobChIP_FOXA1_robot
chr2	178110019	178110020	MACS_peak_8748	4.00	lobChIP_FOXA1_robot
chr2	178128213	178128214	MACS_peak_8749	4.00	lobChIP_FOXA1_robot
chr2	178129808	178129809	MACS_peak_8750	23.00	lobChIP_FOXA1_robot
chr2	178131424	178131425	MACS_peak_8751	9.00	lobChIP_FOXA1_robot
chr2	178200281	178200282	MACS_peak_8752	4.00	lobChIP_FOXA1_robot
chr2	178259085	178259086	MACS_peak_8753	10.00	lobChIP_FOXA1_robot
chr2	178261603	178261604	MACS_peak_8754	26.00	lobChIP_FOXA1_robot
chr2	178262919	178262920	MACS_peak_8755	28.00	lobChIP_FOXA1_robot
chr2	178269932	178269933	MACS_peak_8756	15.00	lobChIP_FOXA1_robot
chr2	178292739	178292740	MACS_peak_8757	4.00	lobChIP_FOXA1_robot
chr2	178444597	178444598	MACS_peak_8758	9.00	lobChIP_FOXA1_robot
chr2	178467796	178467797	MACS_peak_8759	4.00	lobChIP_FOXA1_robot
chr2	178585993	178585994	MACS_peak_8760	6.00	lobChIP_FOXA1_robot
chr2	178835991	178835992	MACS_peak_8761	7.00	lobChIP_FOXA1_robot
chr2	178935846	178935847	MACS_peak_8762	5.00	lobChIP_FOXA1_robot
chr2	179058415	179058416	MACS_peak_8763	48.00	lobChIP_FOXA1_robot
chr2	179334389	179334390	MACS_peak_8764	4.00	lobChIP_FOXA1_robot
chr2	179972839	179972840	MACS_peak_8765	4.00	lobChIP_FOXA1_robot
chr2	180044261	180044262	MACS_peak_8766	3.00	lobChIP_FOXA1_robot
chr2	180097348	180097349	MACS_peak_8767	4.00	lobChIP_FOXA1_robot
chr2	180105395	180105396	MACS_peak_8768	5.00	lobChIP_FOXA1_robot
chr2	180116604	180116605	MACS_peak_8769	3.00	lobChIP_FOXA1_robot
chr2	180127943	180127944	MACS_peak_8770	4.00	lobChIP_FOXA1_robot
chr2	180137290	180137291	MACS_peak_8771	25.00	lobChIP_FOXA1_robot
chr2	180197615	180197616	MACS_peak_8772	4.00	lobChIP_FOXA1_robot
chr2	180282816	180282817	MACS_peak_8773	4.00	lobChIP_FOXA1_robot
chr2	180408260	180408261	MACS_peak_8774	33.00	lobChIP_FOXA1_robot
chr2	180415182	180415183	MACS_peak_8775	7.00	lobChIP_FOXA1_robot
chr2	180423154	180423155	MACS_peak_8776	9.00	lobChIP_FOXA1_robot
chr2	180425104	180425105	MACS_peak_8777	11.00	lobChIP_FOXA1_robot
chr2	180439111	180439112	MACS_peak_8778	6.00	lobChIP_FOXA1_robot
chr2	180496647	180496648	MACS_peak_8779	5.00	lobChIP_FOXA1_robot
chr2	180818992	180818993	MACS_peak_8780	9.00	lobChIP_FOXA1_robot
chr2	180841927	180841928	MACS_peak_8781	5.00	lobChIP_FOXA1_robot
chr2	181119709	181119710	MACS_peak_8782	4.00	lobChIP_FOXA1_robot
chr2	181154544	181154545	MACS_peak_8783	4.00	lobChIP_FOXA1_robot
chr2	181628527	181628528	MACS_peak_8784	36.00	lobChIP_FOXA1_robot
chr2	182412002	182412003	MACS_peak_8785	3.00	lobChIP_FOXA1_robot
chr2	182655804	182655805	MACS_peak_8786	7.00	lobChIP_FOXA1_robot
chr2	182656243	182656244	MACS_peak_8787	5.00	lobChIP_FOXA1_robot
chr2	182765196	182765197	MACS_peak_8788	10.00	lobChIP_FOXA1_robot
chr2	182771892	182771893	MACS_peak_8789	7.00	lobChIP_FOXA1_robot
chr2	183482009	183482010	MACS_peak_8790	3.00	lobChIP_FOXA1_robot
chr2	183755443	183755444	MACS_peak_8791	3.00	lobChIP_FOXA1_robot
chr2	183819635	183819636	MACS_peak_8792	4.00	lobChIP_FOXA1_robot
chr2	185412134	185412135	MACS_peak_8793	5.00	lobChIP_FOXA1_robot
chr2	185462096	185462097	MACS_peak_8794	5.00	lobChIP_FOXA1_robot
chr2	185624200	185624201	MACS_peak_8795	4.00	lobChIP_FOXA1_robot
chr2	186183331	186183332	MACS_peak_8796	3.00	lobChIP_FOXA1_robot
chr2	186510341	186510342	MACS_peak_8797	5.00	lobChIP_FOXA1_robot
chr2	186678586	186678587	MACS_peak_8798	16.00	lobChIP_FOXA1_robot
chr2	186994859	186994860	MACS_peak_8799	6.00	lobChIP_FOXA1_robot
chr2	187046532	187046533	MACS_peak_8800	5.00	lobChIP_FOXA1_robot
chr2	187139894	187139895	MACS_peak_8801	4.00	lobChIP_FOXA1_robot
chr2	187152112	187152113	MACS_peak_8802	3.00	lobChIP_FOXA1_robot
chr2	187206961	187206962	MACS_peak_8803	4.00	lobChIP_FOXA1_robot
chr2	187247777	187247778	MACS_peak_8804	31.00	lobChIP_FOXA1_robot
chr2	187291063	187291064	MACS_peak_8805	10.00	lobChIP_FOXA1_robot
chr2	187398735	187398736	MACS_peak_8806	6.00	lobChIP_FOXA1_robot
chr2	187409201	187409202	MACS_peak_8807	14.00	lobChIP_FOXA1_robot
chr2	187426503	187426504	MACS_peak_8808	4.00	lobChIP_FOXA1_robot
chr2	187460015	187460016	MACS_peak_8809	4.00	lobChIP_FOXA1_robot
chr2	187645922	187645923	MACS_peak_8810	6.00	lobChIP_FOXA1_robot
chr2	187707999	187708000	MACS_peak_8811	3.00	lobChIP_FOXA1_robot
chr2	188365840	188365841	MACS_peak_8812	3.00	lobChIP_FOXA1_robot
chr2	188374719	188374720	MACS_peak_8813	25.00	lobChIP_FOXA1_robot
chr2	188376789	188376790	MACS_peak_8814	7.00	lobChIP_FOXA1_robot
chr2	188382609	188382610	MACS_peak_8815	8.00	lobChIP_FOXA1_robot
chr2	188392133	188392134	MACS_peak_8816	4.00	lobChIP_FOXA1_robot
chr2	188394979	188394980	MACS_peak_8817	5.00	lobChIP_FOXA1_robot
chr2	188406405	188406406	MACS_peak_8818	21.00	lobChIP_FOXA1_robot
chr2	188408956	188408957	MACS_peak_8819	5.00	lobChIP_FOXA1_robot
chr2	188410386	188410387	MACS_peak_8820	6.00	lobChIP_FOXA1_robot
chr2	188414184	188414185	MACS_peak_8821	5.00	lobChIP_FOXA1_robot
chr2	188414973	188414974	MACS_peak_8822	5.00	lobChIP_FOXA1_robot
chr2	188438915	188438916	MACS_peak_8823	5.00	lobChIP_FOXA1_robot
chr2	188708173	188708174	MACS_peak_8824	4.00	lobChIP_FOXA1_robot
chr2	189915026	189915027	MACS_peak_8825	83.00	lobChIP_FOXA1_robot
chr2	190234412	190234413	MACS_peak_8826	5.00	lobChIP_FOXA1_robot
chr2	190367630	190367631	MACS_peak_8827	5.00	lobChIP_FOXA1_robot
chr2	190386908	190386909	MACS_peak_8828	11.00	lobChIP_FOXA1_robot
chr2	190416918	190416919	MACS_peak_8829	3.00	lobChIP_FOXA1_robot
chr2	190417954	190417955	MACS_peak_8830	28.00	lobChIP_FOXA1_robot
chr2	190420098	190420099	MACS_peak_8831	6.00	lobChIP_FOXA1_robot
chr2	190439771	190439772	MACS_peak_8832	5.00	lobChIP_FOXA1_robot
chr2	190474109	190474110	MACS_peak_8833	3.00	lobChIP_FOXA1_robot
chr2	190477892	190477893	MACS_peak_8834	5.00	lobChIP_FOXA1_robot
chr2	190526070	190526071	MACS_peak_8835	4.00	lobChIP_FOXA1_robot
chr2	190628011	190628012	MACS_peak_8836	4.00	lobChIP_FOXA1_robot
chr2	190701557	190701558	MACS_peak_8837	4.00	lobChIP_FOXA1_robot
chr2	190817606	190817607	MACS_peak_8838	4.00	lobChIP_FOXA1_robot
chr2	191001179	191001180	MACS_peak_8839	5.00	lobChIP_FOXA1_robot
chr2	191135168	191135169	MACS_peak_8840	9.00	lobChIP_FOXA1_robot
chr2	191180245	191180246	MACS_peak_8841	5.00	lobChIP_FOXA1_robot
chr2	191199308	191199309	MACS_peak_8842	16.00	lobChIP_FOXA1_robot
chr2	191217784	191217785	MACS_peak_8843	13.00	lobChIP_FOXA1_robot
chr2	191237355	191237356	MACS_peak_8844	8.00	lobChIP_FOXA1_robot
chr2	191364157	191364158	MACS_peak_8845	5.00	lobChIP_FOXA1_robot
chr2	191561973	191561974	MACS_peak_8846	4.00	lobChIP_FOXA1_robot
chr2	191673921	191673922	MACS_peak_8847	7.00	lobChIP_FOXA1_robot
chr2	191680249	191680250	MACS_peak_8848	4.00	lobChIP_FOXA1_robot
chr2	191757771	191757772	MACS_peak_8849	5.00	lobChIP_FOXA1_robot
chr2	192127903	192127904	MACS_peak_8850	4.00	lobChIP_FOXA1_robot
chr2	192247654	192247655	MACS_peak_8851	5.00	lobChIP_FOXA1_robot
chr2	192304262	192304263	MACS_peak_8852	5.00	lobChIP_FOXA1_robot
chr2	192489830	192489831	MACS_peak_8853	4.00	lobChIP_FOXA1_robot
chr2	192502702	192502703	MACS_peak_8854	24.00	lobChIP_FOXA1_robot
chr2	192503211	192503212	MACS_peak_8855	5.00	lobChIP_FOXA1_robot
chr2	192711914	192711915	MACS_peak_8856	3.00	lobChIP_FOXA1_robot
chr2	192729577	192729578	MACS_peak_8857	7.00	lobChIP_FOXA1_robot
chr2	192731756	192731757	MACS_peak_8858	16.00	lobChIP_FOXA1_robot
chr2	192734054	192734055	MACS_peak_8859	4.00	lobChIP_FOXA1_robot
chr2	192747224	192747225	MACS_peak_8860	11.00	lobChIP_FOXA1_robot
chr2	192782599	192782600	MACS_peak_8861	5.00	lobChIP_FOXA1_robot
chr2	192927873	192927874	MACS_peak_8862	5.00	lobChIP_FOXA1_robot
chr2	192934189	192934190	MACS_peak_8863	4.00	lobChIP_FOXA1_robot
chr2	193025410	193025411	MACS_peak_8864	4.00	lobChIP_FOXA1_robot
chr2	193443657	193443658	MACS_peak_8865	5.00	lobChIP_FOXA1_robot
chr2	193991865	193991866	MACS_peak_8866	5.00	lobChIP_FOXA1_robot
chr2	194293193	194293194	MACS_peak_8867	22.00	lobChIP_FOXA1_robot
chr2	194600591	194600592	MACS_peak_8868	4.00	lobChIP_FOXA1_robot
chr2	195063509	195063510	MACS_peak_8869	5.00	lobChIP_FOXA1_robot
chr2	195109072	195109073	MACS_peak_8870	5.00	lobChIP_FOXA1_robot
chr2	196172798	196172799	MACS_peak_8871	4.00	lobChIP_FOXA1_robot
chr2	196409440	196409441	MACS_peak_8872	5.00	lobChIP_FOXA1_robot
chr2	196621065	196621066	MACS_peak_8873	4.00	lobChIP_FOXA1_robot
chr2	197124673	197124674	MACS_peak_8874	15.00	lobChIP_FOXA1_robot
chr2	197594316	197594317	MACS_peak_8875	4.00	lobChIP_FOXA1_robot
chr2	197613922	197613923	MACS_peak_8876	3.00	lobChIP_FOXA1_robot
chr2	198023705	198023706	MACS_peak_8877	5.00	lobChIP_FOXA1_robot
chr2	198348051	198348052	MACS_peak_8878	3.00	lobChIP_FOXA1_robot
chr2	198413456	198413457	MACS_peak_8879	6.00	lobChIP_FOXA1_robot
chr2	198478397	198478398	MACS_peak_8880	66.00	lobChIP_FOXA1_robot
chr2	198522519	198522520	MACS_peak_8881	5.00	lobChIP_FOXA1_robot
chr2	199714064	199714065	MACS_peak_8882	5.00	lobChIP_FOXA1_robot
chr2	200041397	200041398	MACS_peak_8883	5.00	lobChIP_FOXA1_robot
chr2	200085771	200085772	MACS_peak_8884	3.00	lobChIP_FOXA1_robot
chr2	200389874	200389875	MACS_peak_8885	12.00	lobChIP_FOXA1_robot
chr2	200582950	200582951	MACS_peak_8886	8.00	lobChIP_FOXA1_robot
chr2	200603698	200603699	MACS_peak_8887	9.00	lobChIP_FOXA1_robot
chr2	200779218	200779219	MACS_peak_8888	4.00	lobChIP_FOXA1_robot
chr2	200942039	200942040	MACS_peak_8889	28.00	lobChIP_FOXA1_robot
chr2	201084552	201084553	MACS_peak_8890	4.00	lobChIP_FOXA1_robot
chr2	201086026	201086027	MACS_peak_8891	22.00	lobChIP_FOXA1_robot
chr2	201095648	201095649	MACS_peak_8892	4.00	lobChIP_FOXA1_robot
chr2	201167896	201167897	MACS_peak_8893	8.00	lobChIP_FOXA1_robot
chr2	201170939	201170940	MACS_peak_8894	7.00	lobChIP_FOXA1_robot
chr2	201173301	201173302	MACS_peak_8895	3.00	lobChIP_FOXA1_robot
chr2	201174563	201174564	MACS_peak_8896	5.00	lobChIP_FOXA1_robot
chr2	201183871	201183872	MACS_peak_8897	36.00	lobChIP_FOXA1_robot
chr2	201184849	201184850	MACS_peak_8898	40.00	lobChIP_FOXA1_robot
chr2	201186652	201186653	MACS_peak_8899	7.00	lobChIP_FOXA1_robot
chr2	201232122	201232123	MACS_peak_8900	11.00	lobChIP_FOXA1_robot
chr2	201270855	201270856	MACS_peak_8901	19.00	lobChIP_FOXA1_robot
chr2	201457366	201457367	MACS_peak_8902	9.00	lobChIP_FOXA1_robot
chr2	201468947	201468948	MACS_peak_8903	7.00	lobChIP_FOXA1_robot
chr2	201480190	201480191	MACS_peak_8904	4.00	lobChIP_FOXA1_robot
chr2	201542756	201542757	MACS_peak_8905	4.00	lobChIP_FOXA1_robot
chr2	201567442	201567443	MACS_peak_8906	10.00	lobChIP_FOXA1_robot
chr2	201606753	201606754	MACS_peak_8907	10.00	lobChIP_FOXA1_robot
chr2	201613049	201613050	MACS_peak_8908	23.00	lobChIP_FOXA1_robot
chr2	201621571	201621572	MACS_peak_8909	17.00	lobChIP_FOXA1_robot
chr2	201623274	201623275	MACS_peak_8910	14.00	lobChIP_FOXA1_robot
chr2	201642229	201642230	MACS_peak_8911	5.00	lobChIP_FOXA1_robot
chr2	201651395	201651396	MACS_peak_8912	6.00	lobChIP_FOXA1_robot
chr2	201796119	201796120	MACS_peak_8913	4.00	lobChIP_FOXA1_robot
chr2	201796755	201796756	MACS_peak_8914	4.00	lobChIP_FOXA1_robot
chr2	201809818	201809819	MACS_peak_8915	4.00	lobChIP_FOXA1_robot
chr2	201873176	201873177	MACS_peak_8916	8.00	lobChIP_FOXA1_robot
chr2	201905936	201905937	MACS_peak_8917	6.00	lobChIP_FOXA1_robot
chr2	201922707	201922708	MACS_peak_8918	15.00	lobChIP_FOXA1_robot
chr2	201934221	201934222	MACS_peak_8919	7.00	lobChIP_FOXA1_robot
chr2	201938071	201938072	MACS_peak_8920	5.00	lobChIP_FOXA1_robot
chr2	201972341	201972342	MACS_peak_8921	5.00	lobChIP_FOXA1_robot
chr2	201980934	201980935	MACS_peak_8922	3.00	lobChIP_FOXA1_robot
chr2	201984503	201984504	MACS_peak_8923	5.00	lobChIP_FOXA1_robot
chr2	201993947	201993948	MACS_peak_8924	6.00	lobChIP_FOXA1_robot
chr2	202014153	202014154	MACS_peak_8925	6.00	lobChIP_FOXA1_robot
chr2	202023122	202023123	MACS_peak_8926	6.00	lobChIP_FOXA1_robot
chr2	202047742	202047743	MACS_peak_8927	7.00	lobChIP_FOXA1_robot
chr2	202313028	202313029	MACS_peak_8928	5.00	lobChIP_FOXA1_robot
chr2	202600309	202600310	MACS_peak_8929	6.00	lobChIP_FOXA1_robot
chr2	202619675	202619676	MACS_peak_8930	14.00	lobChIP_FOXA1_robot
chr2	202640216	202640217	MACS_peak_8931	5.00	lobChIP_FOXA1_robot
chr2	202717828	202717829	MACS_peak_8932	7.00	lobChIP_FOXA1_robot
chr2	202789312	202789313	MACS_peak_8933	5.00	lobChIP_FOXA1_robot
chr2	202978328	202978329	MACS_peak_8934	5.00	lobChIP_FOXA1_robot
chr2	203143842	203143843	MACS_peak_8935	4.00	lobChIP_FOXA1_robot
chr2	203359316	203359317	MACS_peak_8936	5.00	lobChIP_FOXA1_robot
chr2	203453600	203453601	MACS_peak_8937	5.00	lobChIP_FOXA1_robot
chr2	203746470	203746471	MACS_peak_8938	10.00	lobChIP_FOXA1_robot
chr2	204040610	204040611	MACS_peak_8939	3.00	lobChIP_FOXA1_robot
chr2	204087995	204087996	MACS_peak_8940	4.00	lobChIP_FOXA1_robot
chr2	204217796	204217797	MACS_peak_8941	12.00	lobChIP_FOXA1_robot
chr2	204449839	204449840	MACS_peak_8942	4.00	lobChIP_FOXA1_robot
chr2	204513289	204513290	MACS_peak_8943	8.00	lobChIP_FOXA1_robot
chr2	204521435	204521436	MACS_peak_8944	5.00	lobChIP_FOXA1_robot
chr2	204579060	204579061	MACS_peak_8945	3.00	lobChIP_FOXA1_robot
chr2	204585969	204585970	MACS_peak_8946	5.00	lobChIP_FOXA1_robot
chr2	204648806	204648807	MACS_peak_8947	29.00	lobChIP_FOXA1_robot
chr2	204677639	204677640	MACS_peak_8948	3.00	lobChIP_FOXA1_robot
chr2	204680223	204680224	MACS_peak_8949	7.00	lobChIP_FOXA1_robot
chr2	204895887	204895888	MACS_peak_8950	5.00	lobChIP_FOXA1_robot
chr2	205204697	205204698	MACS_peak_8951	11.00	lobChIP_FOXA1_robot
chr2	205209224	205209225	MACS_peak_8952	3.00	lobChIP_FOXA1_robot
chr2	205272580	205272581	MACS_peak_8953	7.00	lobChIP_FOXA1_robot
chr2	205302923	205302924	MACS_peak_8954	7.00	lobChIP_FOXA1_robot
chr2	205348357	205348358	MACS_peak_8955	4.00	lobChIP_FOXA1_robot
chr2	205352737	205352738	MACS_peak_8956	3.00	lobChIP_FOXA1_robot
chr2	205478512	205478513	MACS_peak_8957	8.00	lobChIP_FOXA1_robot
chr2	205509929	205509930	MACS_peak_8958	6.00	lobChIP_FOXA1_robot
chr2	205529603	205529604	MACS_peak_8959	5.00	lobChIP_FOXA1_robot
chr2	205534996	205534997	MACS_peak_8960	8.00	lobChIP_FOXA1_robot
chr2	205548222	205548223	MACS_peak_8961	3.00	lobChIP_FOXA1_robot
chr2	205762505	205762506	MACS_peak_8962	6.00	lobChIP_FOXA1_robot
chr2	205765411	205765412	MACS_peak_8963	18.00	lobChIP_FOXA1_robot
chr2	205812468	205812469	MACS_peak_8964	10.00	lobChIP_FOXA1_robot
chr2	205895636	205895637	MACS_peak_8965	8.00	lobChIP_FOXA1_robot
chr2	205967927	205967928	MACS_peak_8966	10.00	lobChIP_FOXA1_robot
chr2	206055415	206055416	MACS_peak_8967	8.00	lobChIP_FOXA1_robot
chr2	206081451	206081452	MACS_peak_8968	11.00	lobChIP_FOXA1_robot
chr2	206085502	206085503	MACS_peak_8969	4.00	lobChIP_FOXA1_robot
chr2	206138509	206138510	MACS_peak_8970	26.00	lobChIP_FOXA1_robot
chr2	206565449	206565450	MACS_peak_8971	4.00	lobChIP_FOXA1_robot
chr2	206583909	206583910	MACS_peak_8972	16.00	lobChIP_FOXA1_robot
chr2	206840235	206840236	MACS_peak_8973	13.00	lobChIP_FOXA1_robot
chr2	206854627	206854628	MACS_peak_8974	14.00	lobChIP_FOXA1_robot
chr2	206877212	206877213	MACS_peak_8975	6.00	lobChIP_FOXA1_robot
chr2	206951490	206951491	MACS_peak_8976	4.00	lobChIP_FOXA1_robot
chr2	207267943	207267944	MACS_peak_8977	9.00	lobChIP_FOXA1_robot
chr2	207972643	207972644	MACS_peak_8978	4.00	lobChIP_FOXA1_robot
chr2	208028424	208028425	MACS_peak_8979	5.00	lobChIP_FOXA1_robot
chr2	208273227	208273228	MACS_peak_8980	4.00	lobChIP_FOXA1_robot
chr2	208398520	208398521	MACS_peak_8981	4.00	lobChIP_FOXA1_robot
chr2	208484664	208484665	MACS_peak_8982	4.00	lobChIP_FOXA1_robot
chr2	208599661	208599662	MACS_peak_8983	16.00	lobChIP_FOXA1_robot
chr2	208612760	208612761	MACS_peak_8984	9.00	lobChIP_FOXA1_robot
chr2	208630510	208630511	MACS_peak_8985	4.00	lobChIP_FOXA1_robot
chr2	208640238	208640239	MACS_peak_8986	4.00	lobChIP_FOXA1_robot
chr2	208647064	208647065	MACS_peak_8987	11.00	lobChIP_FOXA1_robot
chr2	208661582	208661583	MACS_peak_8988	5.00	lobChIP_FOXA1_robot
chr2	208666198	208666199	MACS_peak_8989	5.00	lobChIP_FOXA1_robot
chr2	208693889	208693890	MACS_peak_8990	4.00	lobChIP_FOXA1_robot
chr2	208698077	208698078	MACS_peak_8991	3.00	lobChIP_FOXA1_robot
chr2	208710114	208710115	MACS_peak_8992	35.00	lobChIP_FOXA1_robot
chr2	208712737	208712738	MACS_peak_8993	29.00	lobChIP_FOXA1_robot
chr2	208735195	208735196	MACS_peak_8994	11.00	lobChIP_FOXA1_robot
chr2	208740764	208740765	MACS_peak_8995	6.00	lobChIP_FOXA1_robot
chr2	208762926	208762927	MACS_peak_8996	6.00	lobChIP_FOXA1_robot
chr2	208771830	208771831	MACS_peak_8997	4.00	lobChIP_FOXA1_robot
chr2	208791072	208791073	MACS_peak_8998	5.00	lobChIP_FOXA1_robot
chr2	208878853	208878854	MACS_peak_8999	5.00	lobChIP_FOXA1_robot
chr2	208880319	208880320	MACS_peak_9000	11.00	lobChIP_FOXA1_robot
chr2	208890259	208890260	MACS_peak_9001	8.00	lobChIP_FOXA1_robot
chr2	208896650	208896651	MACS_peak_9002	11.00	lobChIP_FOXA1_robot
chr2	208898855	208898856	MACS_peak_9003	3.00	lobChIP_FOXA1_robot
chr2	208987926	208987927	MACS_peak_9004	7.00	lobChIP_FOXA1_robot
chr2	209008173	209008174	MACS_peak_9005	4.00	lobChIP_FOXA1_robot
chr2	209118836	209118837	MACS_peak_9006	6.00	lobChIP_FOXA1_robot
chr2	209128647	209128648	MACS_peak_9007	5.00	lobChIP_FOXA1_robot
chr2	209235218	209235219	MACS_peak_9008	8.00	lobChIP_FOXA1_robot
chr2	209341127	209341128	MACS_peak_9009	3.00	lobChIP_FOXA1_robot
chr2	209659319	209659320	MACS_peak_9010	4.00	lobChIP_FOXA1_robot
chr2	210373191	210373192	MACS_peak_9011	4.00	lobChIP_FOXA1_robot
chr2	210893868	210893869	MACS_peak_9012	4.00	lobChIP_FOXA1_robot
chr2	210996880	210996881	MACS_peak_9013	6.00	lobChIP_FOXA1_robot
chr2	211323273	211323274	MACS_peak_9014	3.00	lobChIP_FOXA1_robot
chr2	211388574	211388575	MACS_peak_9015	4.00	lobChIP_FOXA1_robot
chr2	211406285	211406286	MACS_peak_9016	10.00	lobChIP_FOXA1_robot
chr2	211413966	211413967	MACS_peak_9017	41.00	lobChIP_FOXA1_robot
chr2	211482255	211482256	MACS_peak_9018	4.00	lobChIP_FOXA1_robot
chr2	211496180	211496181	MACS_peak_9019	3.00	lobChIP_FOXA1_robot
chr2	211828246	211828247	MACS_peak_9020	9.00	lobChIP_FOXA1_robot
chr2	212774371	212774372	MACS_peak_9021	4.00	lobChIP_FOXA1_robot
chr2	213224320	213224321	MACS_peak_9022	7.00	lobChIP_FOXA1_robot
chr2	213288967	213288968	MACS_peak_9023	8.00	lobChIP_FOXA1_robot
chr2	213316787	213316788	MACS_peak_9024	7.00	lobChIP_FOXA1_robot
chr2	214104106	214104107	MACS_peak_9025	13.00	lobChIP_FOXA1_robot
chr2	214576078	214576079	MACS_peak_9026	6.00	lobChIP_FOXA1_robot
chr2	214613412	214613413	MACS_peak_9027	4.00	lobChIP_FOXA1_robot
chr2	215604461	215604462	MACS_peak_9028	3.00	lobChIP_FOXA1_robot
chr2	216050104	216050105	MACS_peak_9029	3.00	lobChIP_FOXA1_robot
chr2	216201050	216201051	MACS_peak_9030	3.00	lobChIP_FOXA1_robot
chr2	216280172	216280173	MACS_peak_9031	17.00	lobChIP_FOXA1_robot
chr2	216282224	216282225	MACS_peak_9032	50.00	lobChIP_FOXA1_robot
chr2	216283442	216283443	MACS_peak_9033	7.00	lobChIP_FOXA1_robot
chr2	216284744	216284745	MACS_peak_9034	17.00	lobChIP_FOXA1_robot
chr2	216394284	216394285	MACS_peak_9035	5.00	lobChIP_FOXA1_robot
chr2	216400607	216400608	MACS_peak_9036	4.00	lobChIP_FOXA1_robot
chr2	216417084	216417085	MACS_peak_9037	6.00	lobChIP_FOXA1_robot
chr2	216482003	216482004	MACS_peak_9038	81.00	lobChIP_FOXA1_robot
chr2	216484072	216484073	MACS_peak_9039	15.00	lobChIP_FOXA1_robot
chr2	216540281	216540282	MACS_peak_9040	3.00	lobChIP_FOXA1_robot
chr2	216545937	216545938	MACS_peak_9041	25.00	lobChIP_FOXA1_robot
chr2	216557340	216557341	MACS_peak_9042	7.00	lobChIP_FOXA1_robot
chr2	216558493	216558494	MACS_peak_9043	11.00	lobChIP_FOXA1_robot
chr2	216576182	216576183	MACS_peak_9044	5.00	lobChIP_FOXA1_robot
chr2	216576661	216576662	MACS_peak_9045	6.00	lobChIP_FOXA1_robot
chr2	216578329	216578330	MACS_peak_9046	8.00	lobChIP_FOXA1_robot
chr2	216591907	216591908	MACS_peak_9047	4.00	lobChIP_FOXA1_robot
chr2	216592878	216592879	MACS_peak_9048	5.00	lobChIP_FOXA1_robot
chr2	216614572	216614573	MACS_peak_9049	4.00	lobChIP_FOXA1_robot
chr2	216656506	216656507	MACS_peak_9050	5.00	lobChIP_FOXA1_robot
chr2	216658764	216658765	MACS_peak_9051	47.00	lobChIP_FOXA1_robot
chr2	216691976	216691977	MACS_peak_9052	11.00	lobChIP_FOXA1_robot
chr2	216698720	216698721	MACS_peak_9053	17.00	lobChIP_FOXA1_robot
chr2	216708617	216708618	MACS_peak_9054	4.00	lobChIP_FOXA1_robot
chr2	216741549	216741550	MACS_peak_9055	19.00	lobChIP_FOXA1_robot
chr2	216816416	216816417	MACS_peak_9056	5.00	lobChIP_FOXA1_robot
chr2	216847314	216847315	MACS_peak_9057	19.00	lobChIP_FOXA1_robot
chr2	216853511	216853512	MACS_peak_9058	5.00	lobChIP_FOXA1_robot
chr2	216877100	216877101	MACS_peak_9059	10.00	lobChIP_FOXA1_robot
chr2	216943635	216943636	MACS_peak_9060	18.00	lobChIP_FOXA1_robot
chr2	216979770	216979771	MACS_peak_9061	4.00	lobChIP_FOXA1_robot
chr2	217053392	217053393	MACS_peak_9062	5.00	lobChIP_FOXA1_robot
chr2	217381630	217381631	MACS_peak_9063	4.00	lobChIP_FOXA1_robot
chr2	217387948	217387949	MACS_peak_9064	5.00	lobChIP_FOXA1_robot
chr2	217431225	217431226	MACS_peak_9065	3.00	lobChIP_FOXA1_robot
chr2	217436498	217436499	MACS_peak_9066	22.00	lobChIP_FOXA1_robot
chr2	217438028	217438029	MACS_peak_9067	5.00	lobChIP_FOXA1_robot
chr2	217485029	217485030	MACS_peak_9068	4.00	lobChIP_FOXA1_robot
chr2	217651713	217651714	MACS_peak_9069	3.00	lobChIP_FOXA1_robot
chr2	218190277	218190278	MACS_peak_9070	5.00	lobChIP_FOXA1_robot
chr2	218191171	218191172	MACS_peak_9071	6.00	lobChIP_FOXA1_robot
chr2	218196433	218196434	MACS_peak_9072	7.00	lobChIP_FOXA1_robot
chr2	218767875	218767876	MACS_peak_9073	6.00	lobChIP_FOXA1_robot
chr2	218820151	218820152	MACS_peak_9074	4.00	lobChIP_FOXA1_robot
chr2	219158186	219158187	MACS_peak_9075	6.00	lobChIP_FOXA1_robot
chr2	219177129	219177130	MACS_peak_9076	10.00	lobChIP_FOXA1_robot
chr2	219309541	219309542	MACS_peak_9077	13.00	lobChIP_FOXA1_robot
chr2	219313857	219313858	MACS_peak_9078	6.00	lobChIP_FOXA1_robot
chr2	219592713	219592714	MACS_peak_9079	7.00	lobChIP_FOXA1_robot
chr2	219598601	219598602	MACS_peak_9080	9.00	lobChIP_FOXA1_robot
chr2	219620659	219620660	MACS_peak_9081	28.00	lobChIP_FOXA1_robot
chr2	219630908	219630909	MACS_peak_9082	6.00	lobChIP_FOXA1_robot
chr2	219645891	219645892	MACS_peak_9083	9.00	lobChIP_FOXA1_robot
chr2	219741454	219741455	MACS_peak_9084	4.00	lobChIP_FOXA1_robot
chr2	219866780	219866781	MACS_peak_9085	13.00	lobChIP_FOXA1_robot
chr2	219936121	219936122	MACS_peak_9086	12.00	lobChIP_FOXA1_robot
chr2	219946075	219946076	MACS_peak_9087	10.00	lobChIP_FOXA1_robot
chr2	219976522	219976523	MACS_peak_9088	21.00	lobChIP_FOXA1_robot
chr2	219981648	219981649	MACS_peak_9089	8.00	lobChIP_FOXA1_robot
chr2	219991308	219991309	MACS_peak_9090	7.00	lobChIP_FOXA1_robot
chr2	219992359	219992360	MACS_peak_9091	6.00	lobChIP_FOXA1_robot
chr2	219996263	219996264	MACS_peak_9092	9.00	lobChIP_FOXA1_robot
chr2	220014575	220014576	MACS_peak_9093	4.00	lobChIP_FOXA1_robot
chr2	220042560	220042561	MACS_peak_9094	5.00	lobChIP_FOXA1_robot
chr2	220057618	220057619	MACS_peak_9095	11.00	lobChIP_FOXA1_robot
chr2	220073284	220073285	MACS_peak_9096	4.00	lobChIP_FOXA1_robot
chr2	220114155	220114156	MACS_peak_9097	28.00	lobChIP_FOXA1_robot
chr2	220263748	220263749	MACS_peak_9098	17.00	lobChIP_FOXA1_robot
chr2	220436809	220436810	MACS_peak_9099	5.00	lobChIP_FOXA1_robot
chr2	220441772	220441773	MACS_peak_9100	10.00	lobChIP_FOXA1_robot
chr2	222676777	222676778	MACS_peak_9101	3.00	lobChIP_FOXA1_robot
chr2	223459598	223459599	MACS_peak_9102	6.00	lobChIP_FOXA1_robot
chr2	223598399	223598400	MACS_peak_9103	8.00	lobChIP_FOXA1_robot
chr2	223642427	223642428	MACS_peak_9104	4.00	lobChIP_FOXA1_robot
chr2	223661573	223661574	MACS_peak_9105	59.00	lobChIP_FOXA1_robot
chr2	223707477	223707478	MACS_peak_9106	10.00	lobChIP_FOXA1_robot
chr2	223743218	223743219	MACS_peak_9107	7.00	lobChIP_FOXA1_robot
chr2	223768635	223768636	MACS_peak_9108	4.00	lobChIP_FOXA1_robot
chr2	223872963	223872964	MACS_peak_9109	6.00	lobChIP_FOXA1_robot
chr2	224624143	224624144	MACS_peak_9110	4.00	lobChIP_FOXA1_robot
chr2	224624894	224624895	MACS_peak_9111	5.00	lobChIP_FOXA1_robot
chr2	224718233	224718234	MACS_peak_9112	6.00	lobChIP_FOXA1_robot
chr2	224720849	224720850	MACS_peak_9113	5.00	lobChIP_FOXA1_robot
chr2	224726253	224726254	MACS_peak_9114	15.00	lobChIP_FOXA1_robot
chr2	224773307	224773308	MACS_peak_9115	13.00	lobChIP_FOXA1_robot
chr2	224794551	224794552	MACS_peak_9116	5.00	lobChIP_FOXA1_robot
chr2	224862959	224862960	MACS_peak_9117	4.00	lobChIP_FOXA1_robot
chr2	224888138	224888139	MACS_peak_9118	9.00	lobChIP_FOXA1_robot
chr2	225011207	225011208	MACS_peak_9119	7.00	lobChIP_FOXA1_robot
chr2	225016171	225016172	MACS_peak_9120	8.00	lobChIP_FOXA1_robot
chr2	225350261	225350262	MACS_peak_9121	8.00	lobChIP_FOXA1_robot
chr2	225378582	225378583	MACS_peak_9122	3.00	lobChIP_FOXA1_robot
chr2	225534879	225534880	MACS_peak_9123	4.00	lobChIP_FOXA1_robot
chr2	225658518	225658519	MACS_peak_9124	4.00	lobChIP_FOXA1_robot
chr2	226336353	226336354	MACS_peak_9125	17.00	lobChIP_FOXA1_robot
chr2	226979990	226979991	MACS_peak_9126	9.00	lobChIP_FOXA1_robot
chr2	226983201	226983202	MACS_peak_9127	6.00	lobChIP_FOXA1_robot
chr2	226983968	226983969	MACS_peak_9128	5.00	lobChIP_FOXA1_robot
chr2	226984804	226984805	MACS_peak_9129	6.00	lobChIP_FOXA1_robot
chr2	227024832	227024833	MACS_peak_9130	48.00	lobChIP_FOXA1_robot
chr2	227033533	227033534	MACS_peak_9131	10.00	lobChIP_FOXA1_robot
chr2	227172067	227172068	MACS_peak_9132	3.00	lobChIP_FOXA1_robot
chr2	227268415	227268416	MACS_peak_9133	3.00	lobChIP_FOXA1_robot
chr2	227276600	227276601	MACS_peak_9134	12.00	lobChIP_FOXA1_robot
chr2	227321014	227321015	MACS_peak_9135	12.00	lobChIP_FOXA1_robot
chr2	227342885	227342886	MACS_peak_9136	6.00	lobChIP_FOXA1_robot
chr2	227348895	227348896	MACS_peak_9137	5.00	lobChIP_FOXA1_robot
chr2	227380266	227380267	MACS_peak_9138	13.00	lobChIP_FOXA1_robot
chr2	227424381	227424382	MACS_peak_9139	24.00	lobChIP_FOXA1_robot
chr2	227448558	227448559	MACS_peak_9140	7.00	lobChIP_FOXA1_robot
chr2	227483609	227483610	MACS_peak_9141	17.00	lobChIP_FOXA1_robot
chr2	227515117	227515118	MACS_peak_9142	5.00	lobChIP_FOXA1_robot
chr2	227522186	227522187	MACS_peak_9143	8.00	lobChIP_FOXA1_robot
chr2	227566724	227566725	MACS_peak_9144	3.00	lobChIP_FOXA1_robot
chr2	227598111	227598112	MACS_peak_9145	17.00	lobChIP_FOXA1_robot
chr2	227603641	227603642	MACS_peak_9146	6.00	lobChIP_FOXA1_robot
chr2	227605144	227605145	MACS_peak_9147	6.00	lobChIP_FOXA1_robot
chr2	227625615	227625616	MACS_peak_9148	7.00	lobChIP_FOXA1_robot
chr2	227637549	227637550	MACS_peak_9149	5.00	lobChIP_FOXA1_robot
chr2	227665439	227665440	MACS_peak_9150	28.00	lobChIP_FOXA1_robot
chr2	227666503	227666504	MACS_peak_9151	4.00	lobChIP_FOXA1_robot
chr2	227703662	227703663	MACS_peak_9152	11.00	lobChIP_FOXA1_robot
chr2	227850164	227850165	MACS_peak_9153	4.00	lobChIP_FOXA1_robot
chr2	227903459	227903460	MACS_peak_9154	18.00	lobChIP_FOXA1_robot
chr2	228103047	228103048	MACS_peak_9155	4.00	lobChIP_FOXA1_robot
chr2	228120315	228120316	MACS_peak_9156	4.00	lobChIP_FOXA1_robot
chr2	228178217	228178218	MACS_peak_9157	3.00	lobChIP_FOXA1_robot
chr2	228204013	228204014	MACS_peak_9158	8.00	lobChIP_FOXA1_robot
chr2	228316299	228316300	MACS_peak_9159	21.00	lobChIP_FOXA1_robot
chr2	228319498	228319499	MACS_peak_9160	10.00	lobChIP_FOXA1_robot
chr2	228331425	228331426	MACS_peak_9161	10.00	lobChIP_FOXA1_robot
chr2	228333117	228333118	MACS_peak_9162	4.00	lobChIP_FOXA1_robot
chr2	228509063	228509064	MACS_peak_9163	26.00	lobChIP_FOXA1_robot
chr2	228518281	228518282	MACS_peak_9164	5.00	lobChIP_FOXA1_robot
chr2	228568144	228568145	MACS_peak_9165	5.00	lobChIP_FOXA1_robot
chr2	228612946	228612947	MACS_peak_9166	4.00	lobChIP_FOXA1_robot
chr2	228658310	228658311	MACS_peak_9167	5.00	lobChIP_FOXA1_robot
chr2	228660896	228660897	MACS_peak_9168	7.00	lobChIP_FOXA1_robot
chr2	228678451	228678452	MACS_peak_9169	21.00	lobChIP_FOXA1_robot
chr2	228683730	228683731	MACS_peak_9170	16.00	lobChIP_FOXA1_robot
chr2	228686463	228686464	MACS_peak_9171	4.00	lobChIP_FOXA1_robot
chr2	230024999	230025000	MACS_peak_9172	12.00	lobChIP_FOXA1_robot
chr2	230309866	230309867	MACS_peak_9174	5.00	lobChIP_FOXA1_robot
chr2	230764835	230764836	MACS_peak_9175	14.00	lobChIP_FOXA1_robot
chr2	230877258	230877259	MACS_peak_9176	4.00	lobChIP_FOXA1_robot
chr2	230983754	230983755	MACS_peak_9177	8.00	lobChIP_FOXA1_robot
chr2	230994268	230994269	MACS_peak_9178	3.00	lobChIP_FOXA1_robot
chr2	230995759	230995760	MACS_peak_9179	3.00	lobChIP_FOXA1_robot
chr2	231220950	231220951	MACS_peak_9180	12.00	lobChIP_FOXA1_robot
chr2	231290233	231290234	MACS_peak_9181	4.00	lobChIP_FOXA1_robot
chr2	231356856	231356857	MACS_peak_9182	4.00	lobChIP_FOXA1_robot
chr2	231378179	231378180	MACS_peak_9183	9.00	lobChIP_FOXA1_robot
chr2	231515358	231515359	MACS_peak_9184	11.00	lobChIP_FOXA1_robot
chr2	231603842	231603843	MACS_peak_9185	64.00	lobChIP_FOXA1_robot
chr2	231604752	231604753	MACS_peak_9186	4.00	lobChIP_FOXA1_robot
chr2	231872181	231872182	MACS_peak_9187	6.00	lobChIP_FOXA1_robot
chr2	231899138	231899139	MACS_peak_9188	20.00	lobChIP_FOXA1_robot
chr2	231912607	231912608	MACS_peak_9189	6.00	lobChIP_FOXA1_robot
chr2	231915206	231915207	MACS_peak_9190	25.00	lobChIP_FOXA1_robot
chr2	231930837	231930838	MACS_peak_9191	11.00	lobChIP_FOXA1_robot
chr2	231933380	231933381	MACS_peak_9192	13.00	lobChIP_FOXA1_robot
chr2	232203383	232203384	MACS_peak_9193	6.00	lobChIP_FOXA1_robot
chr2	232230151	232230152	MACS_peak_9194	5.00	lobChIP_FOXA1_robot
chr2	232651164	232651165	MACS_peak_9195	5.00	lobChIP_FOXA1_robot
chr2	232822706	232822707	MACS_peak_9196	4.00	lobChIP_FOXA1_robot
chr2	233165960	233165961	MACS_peak_9197	25.00	lobChIP_FOXA1_robot
chr2	233722115	233722116	MACS_peak_9198	7.00	lobChIP_FOXA1_robot
chr2	233725806	233725807	MACS_peak_9199	5.00	lobChIP_FOXA1_robot
chr2	233878030	233878031	MACS_peak_9200	10.00	lobChIP_FOXA1_robot
chr2	233932027	233932028	MACS_peak_9201	7.00	lobChIP_FOXA1_robot
chr2	234654239	234654240	MACS_peak_9202	32.00	lobChIP_FOXA1_robot
chr2	234665561	234665562	MACS_peak_9203	55.00	lobChIP_FOXA1_robot
chr2	234890673	234890674	MACS_peak_9204	8.00	lobChIP_FOXA1_robot
chr2	235282439	235282440	MACS_peak_9205	3.00	lobChIP_FOXA1_robot
chr2	235371404	235371405	MACS_peak_9206	5.00	lobChIP_FOXA1_robot
chr2	235568049	235568050	MACS_peak_9207	4.00	lobChIP_FOXA1_robot
chr2	235584926	235584927	MACS_peak_9208	17.00	lobChIP_FOXA1_robot
chr2	235586629	235586630	MACS_peak_9209	5.00	lobChIP_FOXA1_robot
chr2	235588550	235588551	MACS_peak_9210	6.00	lobChIP_FOXA1_robot
chr2	235598066	235598067	MACS_peak_9211	7.00	lobChIP_FOXA1_robot
chr2	235601119	235601120	MACS_peak_9212	17.00	lobChIP_FOXA1_robot
chr2	235619456	235619457	MACS_peak_9213	8.00	lobChIP_FOXA1_robot
chr2	235642563	235642564	MACS_peak_9214	4.00	lobChIP_FOXA1_robot
chr2	235704435	235704436	MACS_peak_9215	15.00	lobChIP_FOXA1_robot
chr2	235732586	235732587	MACS_peak_9216	6.00	lobChIP_FOXA1_robot
chr2	235739713	235739714	MACS_peak_9217	4.00	lobChIP_FOXA1_robot
chr2	235746249	235746250	MACS_peak_9218	4.00	lobChIP_FOXA1_robot
chr2	235754771	235754772	MACS_peak_9219	18.00	lobChIP_FOXA1_robot
chr2	235776481	235776482	MACS_peak_9220	4.00	lobChIP_FOXA1_robot
chr2	235780003	235780004	MACS_peak_9221	15.00	lobChIP_FOXA1_robot
chr2	235784589	235784590	MACS_peak_9222	4.00	lobChIP_FOXA1_robot
chr2	235789407	235789408	MACS_peak_9223	12.00	lobChIP_FOXA1_robot
chr2	235890291	235890292	MACS_peak_9224	4.00	lobChIP_FOXA1_robot
chr2	235904456	235904457	MACS_peak_9225	8.00	lobChIP_FOXA1_robot
chr2	235907413	235907414	MACS_peak_9226	3.00	lobChIP_FOXA1_robot
chr2	235909099	235909100	MACS_peak_9227	8.00	lobChIP_FOXA1_robot
chr2	235909732	235909733	MACS_peak_9228	6.00	lobChIP_FOXA1_robot
chr2	235914427	235914428	MACS_peak_9229	19.00	lobChIP_FOXA1_robot
chr2	236025794	236025795	MACS_peak_9230	6.00	lobChIP_FOXA1_robot
chr2	236414095	236414096	MACS_peak_9231	12.00	lobChIP_FOXA1_robot
chr2	236450783	236450784	MACS_peak_9232	25.00	lobChIP_FOXA1_robot
chr2	236451620	236451621	MACS_peak_9233	6.00	lobChIP_FOXA1_robot
chr2	236507676	236507677	MACS_peak_9234	5.00	lobChIP_FOXA1_robot
chr2	236508711	236508712	MACS_peak_9235	6.00	lobChIP_FOXA1_robot
chr2	236526886	236526887	MACS_peak_9236	4.00	lobChIP_FOXA1_robot
chr2	236797710	236797711	MACS_peak_9237	9.00	lobChIP_FOXA1_robot
chr2	236830684	236830685	MACS_peak_9238	3.00	lobChIP_FOXA1_robot
chr2	236956793	236956794	MACS_peak_9239	4.00	lobChIP_FOXA1_robot
chr2	237026777	237026778	MACS_peak_9240	58.00	lobChIP_FOXA1_robot
chr2	237159058	237159059	MACS_peak_9241	5.00	lobChIP_FOXA1_robot
chr2	237266377	237266378	MACS_peak_9242	9.00	lobChIP_FOXA1_robot
chr2	237266851	237266852	MACS_peak_9243	4.00	lobChIP_FOXA1_robot
chr2	237864646	237864647	MACS_peak_9244	7.00	lobChIP_FOXA1_robot
chr2	237984709	237984710	MACS_peak_9245	5.00	lobChIP_FOXA1_robot
chr2	238206271	238206272	MACS_peak_9246	4.00	lobChIP_FOXA1_robot
chr2	238410720	238410721	MACS_peak_9247	5.00	lobChIP_FOXA1_robot
chr2	238411342	238411343	MACS_peak_9248	7.00	lobChIP_FOXA1_robot
chr2	238523001	238523002	MACS_peak_9249	11.00	lobChIP_FOXA1_robot
chr2	238581828	238581829	MACS_peak_9250	5.00	lobChIP_FOXA1_robot
chr2	238589942	238589943	MACS_peak_9251	7.00	lobChIP_FOXA1_robot
chr2	238592167	238592168	MACS_peak_9252	4.00	lobChIP_FOXA1_robot
chr2	238642723	238642724	MACS_peak_9253	8.00	lobChIP_FOXA1_robot
chr2	238652767	238652768	MACS_peak_9254	4.00	lobChIP_FOXA1_robot
chr2	238683696	238683697	MACS_peak_9255	7.00	lobChIP_FOXA1_robot
chr2	238769849	238769850	MACS_peak_9256	8.00	lobChIP_FOXA1_robot
chr2	238801589	238801590	MACS_peak_9257	4.00	lobChIP_FOXA1_robot
chr2	238988648	238988649	MACS_peak_9258	37.00	lobChIP_FOXA1_robot
chr2	239171863	239171864	MACS_peak_9259	3.00	lobChIP_FOXA1_robot
chr2	239194629	239194630	MACS_peak_9260	19.00	lobChIP_FOXA1_robot
chr2	239312806	239312807	MACS_peak_9261	8.00	lobChIP_FOXA1_robot
chr2	239371668	239371669	MACS_peak_9262	6.00	lobChIP_FOXA1_robot
chr2	239426539	239426540	MACS_peak_9263	4.00	lobChIP_FOXA1_robot
chr2	239437788	239437789	MACS_peak_9264	5.00	lobChIP_FOXA1_robot
chr2	239773101	239773102	MACS_peak_9265	5.00	lobChIP_FOXA1_robot
chr2	239777921	239777922	MACS_peak_9266	5.00	lobChIP_FOXA1_robot
chr2	240202562	240202563	MACS_peak_9267	3.00	lobChIP_FOXA1_robot
chr2	240204204	240204205	MACS_peak_9268	8.00	lobChIP_FOXA1_robot
chr2	240351495	240351496	MACS_peak_9269	4.00	lobChIP_FOXA1_robot
chr2	240367148	240367149	MACS_peak_9270	12.00	lobChIP_FOXA1_robot
chr2	240496529	240496530	MACS_peak_9271	5.00	lobChIP_FOXA1_robot
chr2	240630097	240630098	MACS_peak_9272	6.00	lobChIP_FOXA1_robot
chr2	240737600	240737601	MACS_peak_9273	7.00	lobChIP_FOXA1_robot
chr2	241195608	241195609	MACS_peak_9274	6.00	lobChIP_FOXA1_robot
chr2	241500721	241500722	MACS_peak_9275	3.00	lobChIP_FOXA1_robot
chr2	241504493	241504494	MACS_peak_9276	5.00	lobChIP_FOXA1_robot
chr2	242033081	242033082	MACS_peak_9277	68.00	lobChIP_FOXA1_robot
chr2	242224770	242224771	MACS_peak_9278	13.00	lobChIP_FOXA1_robot
chr2	242251381	242251382	MACS_peak_9279	3.00	lobChIP_FOXA1_robot
chr2	242372449	242372450	MACS_peak_9280	33.00	lobChIP_FOXA1_robot
chr2	242467689	242467690	MACS_peak_9281	10.00	lobChIP_FOXA1_robot
chr2	242489962	242489963	MACS_peak_9282	6.00	lobChIP_FOXA1_robot
chr20	118555	118556	MACS_peak_9283	8.00	lobChIP_FOXA1_robot
chr20	351209	351210	MACS_peak_9284	8.00	lobChIP_FOXA1_robot
chr20	352607	352608	MACS_peak_9285	6.00	lobChIP_FOXA1_robot
chr20	377520	377521	MACS_peak_9286	5.00	lobChIP_FOXA1_robot
chr20	382827	382828	MACS_peak_9287	3.00	lobChIP_FOXA1_robot
chr20	430628	430629	MACS_peak_9288	4.00	lobChIP_FOXA1_robot
chr20	459827	459828	MACS_peak_9289	4.00	lobChIP_FOXA1_robot
chr20	491293	491294	MACS_peak_9290	13.00	lobChIP_FOXA1_robot
chr20	599917	599918	MACS_peak_9291	6.00	lobChIP_FOXA1_robot
chr20	627224	627225	MACS_peak_9292	6.00	lobChIP_FOXA1_robot
chr20	1103294	1103295	MACS_peak_9293	5.00	lobChIP_FOXA1_robot
chr20	1131108	1131109	MACS_peak_9294	7.00	lobChIP_FOXA1_robot
chr20	1364883	1364884	MACS_peak_9295	20.00	lobChIP_FOXA1_robot
chr20	1418141	1418142	MACS_peak_9296	3.00	lobChIP_FOXA1_robot
chr20	1617502	1617503	MACS_peak_9297	8.00	lobChIP_FOXA1_robot
chr20	1808657	1808658	MACS_peak_9298	7.00	lobChIP_FOXA1_robot
chr20	1986643	1986644	MACS_peak_9299	3.00	lobChIP_FOXA1_robot
chr20	2047307	2047308	MACS_peak_9300	15.00	lobChIP_FOXA1_robot
chr20	2241831	2241832	MACS_peak_9301	4.00	lobChIP_FOXA1_robot
chr20	2245895	2245896	MACS_peak_9302	5.00	lobChIP_FOXA1_robot
chr20	2256825	2256826	MACS_peak_9303	9.00	lobChIP_FOXA1_robot
chr20	2327456	2327457	MACS_peak_9304	5.00	lobChIP_FOXA1_robot
chr20	2456803	2456804	MACS_peak_9305	6.00	lobChIP_FOXA1_robot
chr20	2490582	2490583	MACS_peak_9306	6.00	lobChIP_FOXA1_robot
chr20	2732090	2732091	MACS_peak_9307	14.00	lobChIP_FOXA1_robot
chr20	2928304	2928305	MACS_peak_9308	11.00	lobChIP_FOXA1_robot
chr20	2963213	2963214	MACS_peak_9309	4.00	lobChIP_FOXA1_robot
chr20	3022980	3022981	MACS_peak_9310	4.00	lobChIP_FOXA1_robot
chr20	3084523	3084524	MACS_peak_9311	4.00	lobChIP_FOXA1_robot
chr20	3203674	3203675	MACS_peak_9312	29.00	lobChIP_FOXA1_robot
chr20	3205855	3205856	MACS_peak_9313	4.00	lobChIP_FOXA1_robot
chr20	3213068	3213069	MACS_peak_9314	5.00	lobChIP_FOXA1_robot
chr20	3226052	3226053	MACS_peak_9315	13.00	lobChIP_FOXA1_robot
chr20	3386841	3386842	MACS_peak_9316	9.00	lobChIP_FOXA1_robot
chr20	3458398	3458399	MACS_peak_9317	3.00	lobChIP_FOXA1_robot
chr20	3473074	3473075	MACS_peak_9318	7.00	lobChIP_FOXA1_robot
chr20	3490932	3490933	MACS_peak_9319	5.00	lobChIP_FOXA1_robot
chr20	3522574	3522575	MACS_peak_9320	10.00	lobChIP_FOXA1_robot
chr20	3525608	3525609	MACS_peak_9321	3.00	lobChIP_FOXA1_robot
chr20	3528714	3528715	MACS_peak_9322	3.00	lobChIP_FOXA1_robot
chr20	3534616	3534617	MACS_peak_9323	8.00	lobChIP_FOXA1_robot
chr20	3613354	3613355	MACS_peak_9324	8.00	lobChIP_FOXA1_robot
chr20	3778848	3778849	MACS_peak_9325	3.00	lobChIP_FOXA1_robot
chr20	3799231	3799232	MACS_peak_9326	5.00	lobChIP_FOXA1_robot
chr20	3869593	3869594	MACS_peak_9327	4.00	lobChIP_FOXA1_robot
chr20	3961677	3961678	MACS_peak_9328	5.00	lobChIP_FOXA1_robot
chr20	3998449	3998450	MACS_peak_9329	6.00	lobChIP_FOXA1_robot
chr20	4109760	4109761	MACS_peak_9330	5.00	lobChIP_FOXA1_robot
chr20	4111169	4111170	MACS_peak_9331	4.00	lobChIP_FOXA1_robot
chr20	4116086	4116087	MACS_peak_9332	5.00	lobChIP_FOXA1_robot
chr20	4152961	4152962	MACS_peak_9333	5.00	lobChIP_FOXA1_robot
chr20	4203141	4203142	MACS_peak_9334	36.00	lobChIP_FOXA1_robot
chr20	4672728	4672729	MACS_peak_9335	19.00	lobChIP_FOXA1_robot
chr20	4700034	4700035	MACS_peak_9336	4.00	lobChIP_FOXA1_robot
chr20	4700856	4700857	MACS_peak_9337	7.00	lobChIP_FOXA1_robot
chr20	4747707	4747708	MACS_peak_9338	4.00	lobChIP_FOXA1_robot
chr20	4750259	4750260	MACS_peak_9339	5.00	lobChIP_FOXA1_robot
chr20	4884952	4884953	MACS_peak_9340	4.00	lobChIP_FOXA1_robot
chr20	4886442	4886443	MACS_peak_9341	14.00	lobChIP_FOXA1_robot
chr20	4918322	4918323	MACS_peak_9342	5.00	lobChIP_FOXA1_robot
chr20	4942678	4942679	MACS_peak_9343	13.00	lobChIP_FOXA1_robot
chr20	4948091	4948092	MACS_peak_9344	56.00	lobChIP_FOXA1_robot
chr20	4953779	4953780	MACS_peak_9345	5.00	lobChIP_FOXA1_robot
chr20	4954501	4954502	MACS_peak_9346	6.00	lobChIP_FOXA1_robot
chr20	4991124	4991125	MACS_peak_9347	5.00	lobChIP_FOXA1_robot
chr20	5009457	5009458	MACS_peak_9348	12.00	lobChIP_FOXA1_robot
chr20	5051283	5051284	MACS_peak_9349	5.00	lobChIP_FOXA1_robot
chr20	5124024	5124025	MACS_peak_9350	8.00	lobChIP_FOXA1_robot
chr20	5133757	5133758	MACS_peak_9351	14.00	lobChIP_FOXA1_robot
chr20	5495046	5495047	MACS_peak_9352	18.00	lobChIP_FOXA1_robot
chr20	5635440	5635441	MACS_peak_9353	7.00	lobChIP_FOXA1_robot
chr20	5695394	5695395	MACS_peak_9354	6.00	lobChIP_FOXA1_robot
chr20	5705452	5705453	MACS_peak_9355	21.00	lobChIP_FOXA1_robot
chr20	5746769	5746770	MACS_peak_9356	3.00	lobChIP_FOXA1_robot
chr20	5760377	5760378	MACS_peak_9357	22.00	lobChIP_FOXA1_robot
chr20	5763311	5763312	MACS_peak_9358	53.00	lobChIP_FOXA1_robot
chr20	5785057	5785058	MACS_peak_9359	43.00	lobChIP_FOXA1_robot
chr20	5801446	5801447	MACS_peak_9360	4.00	lobChIP_FOXA1_robot
chr20	5812434	5812435	MACS_peak_9361	12.00	lobChIP_FOXA1_robot
chr20	5825202	5825203	MACS_peak_9362	6.00	lobChIP_FOXA1_robot
chr20	5958071	5958072	MACS_peak_9363	6.00	lobChIP_FOXA1_robot
chr20	5961552	5961553	MACS_peak_9364	4.00	lobChIP_FOXA1_robot
chr20	5986188	5986189	MACS_peak_9365	6.00	lobChIP_FOXA1_robot
chr20	5988441	5988442	MACS_peak_9366	24.00	lobChIP_FOXA1_robot
chr20	5988992	5988993	MACS_peak_9367	9.00	lobChIP_FOXA1_robot
chr20	6076408	6076409	MACS_peak_9368	8.00	lobChIP_FOXA1_robot
chr20	6098797	6098798	MACS_peak_9369	5.00	lobChIP_FOXA1_robot
chr20	6100820	6100821	MACS_peak_9370	3.00	lobChIP_FOXA1_robot
chr20	6104092	6104093	MACS_peak_9371	3.00	lobChIP_FOXA1_robot
chr20	6447312	6447313	MACS_peak_9372	5.00	lobChIP_FOXA1_robot
chr20	6470211	6470212	MACS_peak_9373	4.00	lobChIP_FOXA1_robot
chr20	6685054	6685055	MACS_peak_9374	11.00	lobChIP_FOXA1_robot
chr20	6988299	6988300	MACS_peak_9375	4.00	lobChIP_FOXA1_robot
chr20	7019837	7019838	MACS_peak_9376	4.00	lobChIP_FOXA1_robot
chr20	7127824	7127825	MACS_peak_9377	3.00	lobChIP_FOXA1_robot
chr20	7565839	7565840	MACS_peak_9378	8.00	lobChIP_FOXA1_robot
chr20	7962207	7962208	MACS_peak_9379	4.00	lobChIP_FOXA1_robot
chr20	8028999	8029000	MACS_peak_9380	6.00	lobChIP_FOXA1_robot
chr20	8075561	8075562	MACS_peak_9381	6.00	lobChIP_FOXA1_robot
chr20	8110469	8110470	MACS_peak_9382	11.00	lobChIP_FOXA1_robot
chr20	8111428	8111429	MACS_peak_9383	4.00	lobChIP_FOXA1_robot
chr20	8124568	8124569	MACS_peak_9384	3.00	lobChIP_FOXA1_robot
chr20	8125454	8125455	MACS_peak_9385	30.00	lobChIP_FOXA1_robot
chr20	8136168	8136169	MACS_peak_9386	10.00	lobChIP_FOXA1_robot
chr20	8137346	8137347	MACS_peak_9387	47.00	lobChIP_FOXA1_robot
chr20	8167893	8167894	MACS_peak_9388	4.00	lobChIP_FOXA1_robot
chr20	8173920	8173921	MACS_peak_9389	4.00	lobChIP_FOXA1_robot
chr20	8186817	8186818	MACS_peak_9390	25.00	lobChIP_FOXA1_robot
chr20	8201117	8201118	MACS_peak_9391	5.00	lobChIP_FOXA1_robot
chr20	8224730	8224731	MACS_peak_9392	3.00	lobChIP_FOXA1_robot
chr20	8231372	8231373	MACS_peak_9393	46.00	lobChIP_FOXA1_robot
chr20	8236715	8236716	MACS_peak_9394	11.00	lobChIP_FOXA1_robot
chr20	8311740	8311741	MACS_peak_9395	34.00	lobChIP_FOXA1_robot
chr20	8312988	8312989	MACS_peak_9396	9.00	lobChIP_FOXA1_robot
chr20	8345328	8345329	MACS_peak_9397	10.00	lobChIP_FOXA1_robot
chr20	8347164	8347165	MACS_peak_9398	12.00	lobChIP_FOXA1_robot
chr20	8395715	8395716	MACS_peak_9399	4.00	lobChIP_FOXA1_robot
chr20	8445298	8445299	MACS_peak_9400	6.00	lobChIP_FOXA1_robot
chr20	8643113	8643114	MACS_peak_9401	4.00	lobChIP_FOXA1_robot
chr20	8713247	8713248	MACS_peak_9402	5.00	lobChIP_FOXA1_robot
chr20	8771249	8771250	MACS_peak_9403	3.00	lobChIP_FOXA1_robot
chr20	8788917	8788918	MACS_peak_9404	3.00	lobChIP_FOXA1_robot
chr20	8841908	8841909	MACS_peak_9405	6.00	lobChIP_FOXA1_robot
chr20	9151579	9151580	MACS_peak_9406	4.00	lobChIP_FOXA1_robot
chr20	9282606	9282607	MACS_peak_9407	5.00	lobChIP_FOXA1_robot
chr20	9390291	9390292	MACS_peak_9408	4.00	lobChIP_FOXA1_robot
chr20	9575106	9575107	MACS_peak_9409	7.00	lobChIP_FOXA1_robot
chr20	10231678	10231679	MACS_peak_9410	4.00	lobChIP_FOXA1_robot
chr20	10244525	10244526	MACS_peak_9411	25.00	lobChIP_FOXA1_robot
chr20	10340511	10340512	MACS_peak_9412	4.00	lobChIP_FOXA1_robot
chr20	10348703	10348704	MACS_peak_9413	11.00	lobChIP_FOXA1_robot
chr20	10350173	10350174	MACS_peak_9414	4.00	lobChIP_FOXA1_robot
chr20	10351176	10351177	MACS_peak_9415	20.00	lobChIP_FOXA1_robot
chr20	10384891	10384892	MACS_peak_9416	4.00	lobChIP_FOXA1_robot
chr20	10415004	10415005	MACS_peak_9417	6.00	lobChIP_FOXA1_robot
chr20	10492804	10492805	MACS_peak_9418	9.00	lobChIP_FOXA1_robot
chr20	10521557	10521558	MACS_peak_9419	10.00	lobChIP_FOXA1_robot
chr20	10522892	10522893	MACS_peak_9420	6.00	lobChIP_FOXA1_robot
chr20	10536851	10536852	MACS_peak_9421	6.00	lobChIP_FOXA1_robot
chr20	10546549	10546550	MACS_peak_9422	25.00	lobChIP_FOXA1_robot
chr20	10549000	10549001	MACS_peak_9423	6.00	lobChIP_FOXA1_robot
chr20	10577745	10577746	MACS_peak_9424	6.00	lobChIP_FOXA1_robot
chr20	10579728	10579729	MACS_peak_9425	10.00	lobChIP_FOXA1_robot
chr20	10585398	10585399	MACS_peak_9426	14.00	lobChIP_FOXA1_robot
chr20	10617278	10617279	MACS_peak_9427	5.00	lobChIP_FOXA1_robot
chr20	10686902	10686903	MACS_peak_9428	8.00	lobChIP_FOXA1_robot
chr20	10689592	10689593	MACS_peak_9429	13.00	lobChIP_FOXA1_robot
chr20	10691888	10691889	MACS_peak_9430	14.00	lobChIP_FOXA1_robot
chr20	10692648	10692649	MACS_peak_9431	19.00	lobChIP_FOXA1_robot
chr20	10695859	10695860	MACS_peak_9432	8.00	lobChIP_FOXA1_robot
chr20	10697694	10697695	MACS_peak_9433	6.00	lobChIP_FOXA1_robot
chr20	10781178	10781179	MACS_peak_9434	4.00	lobChIP_FOXA1_robot
chr20	10829893	10829894	MACS_peak_9435	4.00	lobChIP_FOXA1_robot
chr20	10837140	10837141	MACS_peak_9436	8.00	lobChIP_FOXA1_robot
chr20	10930716	10930717	MACS_peak_9437	6.00	lobChIP_FOXA1_robot
chr20	10941197	10941198	MACS_peak_9438	4.00	lobChIP_FOXA1_robot
chr20	11294904	11294905	MACS_peak_9439	4.00	lobChIP_FOXA1_robot
chr20	11297520	11297521	MACS_peak_9440	3.00	lobChIP_FOXA1_robot
chr20	11668493	11668494	MACS_peak_9441	5.00	lobChIP_FOXA1_robot
chr20	12177205	12177206	MACS_peak_9442	4.00	lobChIP_FOXA1_robot
chr20	12296514	12296515	MACS_peak_9443	5.00	lobChIP_FOXA1_robot
chr20	12374785	12374786	MACS_peak_9444	7.00	lobChIP_FOXA1_robot
chr20	12895764	12895765	MACS_peak_9445	7.00	lobChIP_FOXA1_robot
chr20	12909639	12909640	MACS_peak_9446	3.00	lobChIP_FOXA1_robot
chr20	12950160	12950161	MACS_peak_9447	6.00	lobChIP_FOXA1_robot
chr20	12957496	12957497	MACS_peak_9448	3.00	lobChIP_FOXA1_robot
chr20	12958755	12958756	MACS_peak_9449	22.00	lobChIP_FOXA1_robot
chr20	12959799	12959800	MACS_peak_9450	22.00	lobChIP_FOXA1_robot
chr20	13039777	13039778	MACS_peak_9451	14.00	lobChIP_FOXA1_robot
chr20	13059180	13059181	MACS_peak_9452	4.00	lobChIP_FOXA1_robot
chr20	13070755	13070756	MACS_peak_9453	15.00	lobChIP_FOXA1_robot
chr20	13075812	13075813	MACS_peak_9454	9.00	lobChIP_FOXA1_robot
chr20	13092224	13092225	MACS_peak_9455	4.00	lobChIP_FOXA1_robot
chr20	13102681	13102682	MACS_peak_9456	11.00	lobChIP_FOXA1_robot
chr20	13224632	13224633	MACS_peak_9457	56.00	lobChIP_FOXA1_robot
chr20	13226147	13226148	MACS_peak_9458	7.00	lobChIP_FOXA1_robot
chr20	13238240	13238241	MACS_peak_9459	4.00	lobChIP_FOXA1_robot
chr20	13242786	13242787	MACS_peak_9460	5.00	lobChIP_FOXA1_robot
chr20	13243539	13243540	MACS_peak_9461	4.00	lobChIP_FOXA1_robot
chr20	13303016	13303017	MACS_peak_9462	4.00	lobChIP_FOXA1_robot
chr20	13303426	13303427	MACS_peak_9463	15.00	lobChIP_FOXA1_robot
chr20	13317390	13317391	MACS_peak_9464	4.00	lobChIP_FOXA1_robot
chr20	13320073	13320074	MACS_peak_9465	6.00	lobChIP_FOXA1_robot
chr20	13336253	13336254	MACS_peak_9466	4.00	lobChIP_FOXA1_robot
chr20	13399571	13399572	MACS_peak_9467	11.00	lobChIP_FOXA1_robot
chr20	13403490	13403491	MACS_peak_9468	6.00	lobChIP_FOXA1_robot
chr20	13404739	13404740	MACS_peak_9469	40.00	lobChIP_FOXA1_robot
chr20	13411458	13411459	MACS_peak_9470	4.00	lobChIP_FOXA1_robot
chr20	13433475	13433476	MACS_peak_9471	9.00	lobChIP_FOXA1_robot
chr20	13527265	13527266	MACS_peak_9472	6.00	lobChIP_FOXA1_robot
chr20	13568225	13568226	MACS_peak_9473	5.00	lobChIP_FOXA1_robot
chr20	13594807	13594808	MACS_peak_9474	4.00	lobChIP_FOXA1_robot
chr20	13662363	13662364	MACS_peak_9475	4.00	lobChIP_FOXA1_robot
chr20	13673756	13673757	MACS_peak_9476	8.00	lobChIP_FOXA1_robot
chr20	13769290	13769291	MACS_peak_9477	12.00	lobChIP_FOXA1_robot
chr20	14294339	14294340	MACS_peak_9478	3.00	lobChIP_FOXA1_robot
chr20	14387321	14387322	MACS_peak_9479	5.00	lobChIP_FOXA1_robot
chr20	14859855	14859856	MACS_peak_9480	16.00	lobChIP_FOXA1_robot
chr20	14936859	14936860	MACS_peak_9481	8.00	lobChIP_FOXA1_robot
chr20	14960324	14960325	MACS_peak_9482	16.00	lobChIP_FOXA1_robot
chr20	14965963	14965964	MACS_peak_9483	29.00	lobChIP_FOXA1_robot
chr20	14967138	14967139	MACS_peak_9484	11.00	lobChIP_FOXA1_robot
chr20	14971860	14971861	MACS_peak_9485	26.00	lobChIP_FOXA1_robot
chr20	15041442	15041443	MACS_peak_9486	12.00	lobChIP_FOXA1_robot
chr20	15042832	15042833	MACS_peak_9487	20.00	lobChIP_FOXA1_robot
chr20	15139262	15139263	MACS_peak_9488	4.00	lobChIP_FOXA1_robot
chr20	15166607	15166608	MACS_peak_9489	9.00	lobChIP_FOXA1_robot
chr20	15237124	15237125	MACS_peak_9490	4.00	lobChIP_FOXA1_robot
chr20	15684027	15684028	MACS_peak_9491	5.00	lobChIP_FOXA1_robot
chr20	15845514	15845515	MACS_peak_9492	4.00	lobChIP_FOXA1_robot
chr20	16252243	16252244	MACS_peak_9493	5.00	lobChIP_FOXA1_robot
chr20	16284194	16284195	MACS_peak_9494	5.00	lobChIP_FOXA1_robot
chr20	16349482	16349483	MACS_peak_9495	8.00	lobChIP_FOXA1_robot
chr20	16456131	16456132	MACS_peak_9496	30.00	lobChIP_FOXA1_robot
chr20	16456780	16456781	MACS_peak_9497	15.00	lobChIP_FOXA1_robot
chr20	16508982	16508983	MACS_peak_9498	4.00	lobChIP_FOXA1_robot
chr20	16560852	16560853	MACS_peak_9499	6.00	lobChIP_FOXA1_robot
chr20	16728265	16728266	MACS_peak_9500	4.00	lobChIP_FOXA1_robot
chr20	16742203	16742204	MACS_peak_9501	4.00	lobChIP_FOXA1_robot
chr20	17145304	17145305	MACS_peak_9502	5.00	lobChIP_FOXA1_robot
chr20	17424889	17424890	MACS_peak_9503	8.00	lobChIP_FOXA1_robot
chr20	17442143	17442144	MACS_peak_9504	3.00	lobChIP_FOXA1_robot
chr20	17512070	17512071	MACS_peak_9505	14.00	lobChIP_FOXA1_robot
chr20	17539173	17539174	MACS_peak_9506	4.00	lobChIP_FOXA1_robot
chr20	17551630	17551631	MACS_peak_9507	3.00	lobChIP_FOXA1_robot
chr20	17586103	17586104	MACS_peak_9508	9.00	lobChIP_FOXA1_robot
chr20	17651913	17651914	MACS_peak_9509	4.00	lobChIP_FOXA1_robot
chr20	17657865	17657866	MACS_peak_9510	4.00	lobChIP_FOXA1_robot
chr20	17665103	17665104	MACS_peak_9511	42.00	lobChIP_FOXA1_robot
chr20	17689547	17689548	MACS_peak_9512	3.00	lobChIP_FOXA1_robot
chr20	17702897	17702898	MACS_peak_9513	75.00	lobChIP_FOXA1_robot
chr20	17752176	17752177	MACS_peak_9514	4.00	lobChIP_FOXA1_robot
chr20	17802768	17802769	MACS_peak_9515	5.00	lobChIP_FOXA1_robot
chr20	17836819	17836820	MACS_peak_9516	4.00	lobChIP_FOXA1_robot
chr20	17839918	17839919	MACS_peak_9517	21.00	lobChIP_FOXA1_robot
chr20	17844562	17844563	MACS_peak_9518	10.00	lobChIP_FOXA1_robot
chr20	17873035	17873036	MACS_peak_9519	8.00	lobChIP_FOXA1_robot
chr20	17877555	17877556	MACS_peak_9520	9.00	lobChIP_FOXA1_robot
chr20	17899096	17899097	MACS_peak_9521	12.00	lobChIP_FOXA1_robot
chr20	17938228	17938229	MACS_peak_9522	10.00	lobChIP_FOXA1_robot
chr20	17939359	17939360	MACS_peak_9523	16.00	lobChIP_FOXA1_robot
chr20	17944427	17944428	MACS_peak_9524	5.00	lobChIP_FOXA1_robot
chr20	17966505	17966506	MACS_peak_9525	22.00	lobChIP_FOXA1_robot
chr20	18084328	18084329	MACS_peak_9526	9.00	lobChIP_FOXA1_robot
chr20	18119649	18119650	MACS_peak_9527	4.00	lobChIP_FOXA1_robot
chr20	18125260	18125261	MACS_peak_9528	4.00	lobChIP_FOXA1_robot
chr20	18205200	18205201	MACS_peak_9529	3.00	lobChIP_FOXA1_robot
chr20	18554437	18554438	MACS_peak_9530	8.00	lobChIP_FOXA1_robot
chr20	18601008	18601009	MACS_peak_9531	4.00	lobChIP_FOXA1_robot
chr20	19590602	19590603	MACS_peak_9532	9.00	lobChIP_FOXA1_robot
chr20	19765249	19765250	MACS_peak_9533	7.00	lobChIP_FOXA1_robot
chr20	19775711	19775712	MACS_peak_9534	5.00	lobChIP_FOXA1_robot
chr20	19860992	19860993	MACS_peak_9535	4.00	lobChIP_FOXA1_robot
chr20	19865712	19865713	MACS_peak_9536	7.00	lobChIP_FOXA1_robot
chr20	19899262	19899263	MACS_peak_9537	36.00	lobChIP_FOXA1_robot
chr20	19945360	19945361	MACS_peak_9538	15.00	lobChIP_FOXA1_robot
chr20	19957584	19957585	MACS_peak_9539	20.00	lobChIP_FOXA1_robot
chr20	19965538	19965539	MACS_peak_9540	6.00	lobChIP_FOXA1_robot
chr20	19982968	19982969	MACS_peak_9541	5.00	lobChIP_FOXA1_robot
chr20	19991380	19991381	MACS_peak_9542	11.00	lobChIP_FOXA1_robot
chr20	19995429	19995430	MACS_peak_9543	6.00	lobChIP_FOXA1_robot
chr20	19996631	19996632	MACS_peak_9544	8.00	lobChIP_FOXA1_robot
chr20	20177374	20177375	MACS_peak_9545	4.00	lobChIP_FOXA1_robot
chr20	20529644	20529645	MACS_peak_9546	12.00	lobChIP_FOXA1_robot
chr20	20554624	20554625	MACS_peak_9547	4.00	lobChIP_FOXA1_robot
chr20	20679857	20679858	MACS_peak_9548	34.00	lobChIP_FOXA1_robot
chr20	20681763	20681764	MACS_peak_9549	5.00	lobChIP_FOXA1_robot
chr20	20724359	20724360	MACS_peak_9550	8.00	lobChIP_FOXA1_robot
chr20	20876267	20876268	MACS_peak_9551	4.00	lobChIP_FOXA1_robot
chr20	20922419	20922420	MACS_peak_9552	8.00	lobChIP_FOXA1_robot
chr20	20952667	20952668	MACS_peak_9553	23.00	lobChIP_FOXA1_robot
chr20	20960790	20960791	MACS_peak_9554	28.00	lobChIP_FOXA1_robot
chr20	20998479	20998480	MACS_peak_9555	25.00	lobChIP_FOXA1_robot
chr20	21001396	21001397	MACS_peak_9556	6.00	lobChIP_FOXA1_robot
chr20	21012655	21012656	MACS_peak_9557	4.00	lobChIP_FOXA1_robot
chr20	21033918	21033919	MACS_peak_9558	11.00	lobChIP_FOXA1_robot
chr20	21058814	21058815	MACS_peak_9559	21.00	lobChIP_FOXA1_robot
chr20	21065787	21065788	MACS_peak_9560	3.00	lobChIP_FOXA1_robot
chr20	21079250	21079251	MACS_peak_9561	3.00	lobChIP_FOXA1_robot
chr20	21106639	21106640	MACS_peak_9562	4.00	lobChIP_FOXA1_robot
chr20	21115756	21115757	MACS_peak_9563	55.00	lobChIP_FOXA1_robot
chr20	21120603	21120604	MACS_peak_9564	6.00	lobChIP_FOXA1_robot
chr20	21216819	21216820	MACS_peak_9565	5.00	lobChIP_FOXA1_robot
chr20	21218105	21218106	MACS_peak_9566	3.00	lobChIP_FOXA1_robot
chr20	21317191	21317192	MACS_peak_9567	8.00	lobChIP_FOXA1_robot
chr20	21320422	21320423	MACS_peak_9568	3.00	lobChIP_FOXA1_robot
chr20	21338725	21338726	MACS_peak_9569	5.00	lobChIP_FOXA1_robot
chr20	21545131	21545132	MACS_peak_9570	4.00	lobChIP_FOXA1_robot
chr20	21962827	21962828	MACS_peak_9571	8.00	lobChIP_FOXA1_robot
chr20	22152866	22152867	MACS_peak_9572	3.00	lobChIP_FOXA1_robot
chr20	22384217	22384218	MACS_peak_9573	3.00	lobChIP_FOXA1_robot
chr20	22384776	22384777	MACS_peak_9574	5.00	lobChIP_FOXA1_robot
chr20	22395157	22395158	MACS_peak_9575	5.00	lobChIP_FOXA1_robot
chr20	22410200	22410201	MACS_peak_9576	10.00	lobChIP_FOXA1_robot
chr20	22411534	22411535	MACS_peak_9577	18.00	lobChIP_FOXA1_robot
chr20	22433254	22433255	MACS_peak_9578	22.00	lobChIP_FOXA1_robot
chr20	22446862	22446863	MACS_peak_9579	4.00	lobChIP_FOXA1_robot
chr20	22451320	22451321	MACS_peak_9580	21.00	lobChIP_FOXA1_robot
chr20	22471584	22471585	MACS_peak_9581	10.00	lobChIP_FOXA1_robot
chr20	22472214	22472215	MACS_peak_9582	4.00	lobChIP_FOXA1_robot
chr20	22479254	22479255	MACS_peak_9583	4.00	lobChIP_FOXA1_robot
chr20	22488034	22488035	MACS_peak_9584	9.00	lobChIP_FOXA1_robot
chr20	22493253	22493254	MACS_peak_9585	56.00	lobChIP_FOXA1_robot
chr20	22504966	22504967	MACS_peak_9586	10.00	lobChIP_FOXA1_robot
chr20	22505885	22505886	MACS_peak_9587	9.00	lobChIP_FOXA1_robot
chr20	22514946	22514947	MACS_peak_9588	3.00	lobChIP_FOXA1_robot
chr20	22517842	22517843	MACS_peak_9589	5.00	lobChIP_FOXA1_robot
chr20	22559462	22559463	MACS_peak_9590	20.00	lobChIP_FOXA1_robot
chr20	22561056	22561057	MACS_peak_9591	5.00	lobChIP_FOXA1_robot
chr20	22564676	22564677	MACS_peak_9592	5.00	lobChIP_FOXA1_robot
chr20	22566220	22566221	MACS_peak_9593	16.00	lobChIP_FOXA1_robot
chr20	22661118	22661119	MACS_peak_9594	8.00	lobChIP_FOXA1_robot
chr20	22678271	22678272	MACS_peak_9595	4.00	lobChIP_FOXA1_robot
chr20	23129066	23129067	MACS_peak_9596	5.00	lobChIP_FOXA1_robot
chr20	23213436	23213437	MACS_peak_9597	8.00	lobChIP_FOXA1_robot
chr20	23249689	23249690	MACS_peak_9598	7.00	lobChIP_FOXA1_robot
chr20	23251013	23251014	MACS_peak_9599	9.00	lobChIP_FOXA1_robot
chr20	23339776	23339777	MACS_peak_9600	3.00	lobChIP_FOXA1_robot
chr20	23349072	23349073	MACS_peak_9601	4.00	lobChIP_FOXA1_robot
chr20	23367338	23367339	MACS_peak_9602	9.00	lobChIP_FOXA1_robot
chr20	23416782	23416783	MACS_peak_9603	4.00	lobChIP_FOXA1_robot
chr20	23566750	23566751	MACS_peak_9604	4.00	lobChIP_FOXA1_robot
chr20	23609901	23609902	MACS_peak_9605	4.00	lobChIP_FOXA1_robot
chr20	23639878	23639879	MACS_peak_9606	3.00	lobChIP_FOXA1_robot
chr20	23642287	23642288	MACS_peak_9607	25.00	lobChIP_FOXA1_robot
chr20	23860740	23860741	MACS_peak_9608	3.00	lobChIP_FOXA1_robot
chr20	23976769	23976770	MACS_peak_9609	6.00	lobChIP_FOXA1_robot
chr20	24270810	24270811	MACS_peak_9610	4.00	lobChIP_FOXA1_robot
chr20	24601900	24601901	MACS_peak_9611	4.00	lobChIP_FOXA1_robot
chr20	24968174	24968175	MACS_peak_9612	7.00	lobChIP_FOXA1_robot
chr20	24971434	24971435	MACS_peak_9613	6.00	lobChIP_FOXA1_robot
chr20	24973770	24973771	MACS_peak_9614	4.00	lobChIP_FOXA1_robot
chr20	25013265	25013266	MACS_peak_9615	6.00	lobChIP_FOXA1_robot
chr20	25036069	25036070	MACS_peak_9616	4.00	lobChIP_FOXA1_robot
chr20	25095980	25095981	MACS_peak_9617	9.00	lobChIP_FOXA1_robot
chr20	25154680	25154681	MACS_peak_9618	6.00	lobChIP_FOXA1_robot
chr20	25214195	25214196	MACS_peak_9619	13.00	lobChIP_FOXA1_robot
chr20	25291139	25291140	MACS_peak_9620	6.00	lobChIP_FOXA1_robot
chr20	25319226	25319227	MACS_peak_9621	25.00	lobChIP_FOXA1_robot
chr20	25359247	25359248	MACS_peak_9622	80.00	lobChIP_FOXA1_robot
chr20	25375839	25375840	MACS_peak_9623	3.00	lobChIP_FOXA1_robot
chr20	25488906	25488907	MACS_peak_9624	42.00	lobChIP_FOXA1_robot
chr20	25519829	25519830	MACS_peak_9625	4.00	lobChIP_FOXA1_robot
chr20	25555346	25555347	MACS_peak_9626	4.00	lobChIP_FOXA1_robot
chr20	25594825	25594826	MACS_peak_9627	4.00	lobChIP_FOXA1_robot
chr20	25650717	25650718	MACS_peak_9628	3.00	lobChIP_FOXA1_robot
chr20	30160714	30160715	MACS_peak_9629	3.00	lobChIP_FOXA1_robot
chr20	30162206	30162207	MACS_peak_9630	6.00	lobChIP_FOXA1_robot
chr20	30168775	30168776	MACS_peak_9631	3.00	lobChIP_FOXA1_robot
chr20	30182423	30182424	MACS_peak_9632	3.00	lobChIP_FOXA1_robot
chr20	30192094	30192095	MACS_peak_9633	5.00	lobChIP_FOXA1_robot
chr20	30195114	30195115	MACS_peak_9634	5.00	lobChIP_FOXA1_robot
chr20	30196508	30196509	MACS_peak_9635	31.00	lobChIP_FOXA1_robot
chr20	30198719	30198720	MACS_peak_9636	5.00	lobChIP_FOXA1_robot
chr20	30252264	30252265	MACS_peak_9637	22.00	lobChIP_FOXA1_robot
chr20	30284044	30284045	MACS_peak_9638	6.00	lobChIP_FOXA1_robot
chr20	30292583	30292584	MACS_peak_9639	41.00	lobChIP_FOXA1_robot
chr20	30294711	30294712	MACS_peak_9640	56.00	lobChIP_FOXA1_robot
chr20	30297988	30297989	MACS_peak_9641	6.00	lobChIP_FOXA1_robot
chr20	30299404	30299405	MACS_peak_9642	11.00	lobChIP_FOXA1_robot
chr20	30300680	30300681	MACS_peak_9643	11.00	lobChIP_FOXA1_robot
chr20	30307768	30307769	MACS_peak_9644	41.00	lobChIP_FOXA1_robot
chr20	30308762	30308763	MACS_peak_9645	6.00	lobChIP_FOXA1_robot
chr20	30467953	30467954	MACS_peak_9646	7.00	lobChIP_FOXA1_robot
chr20	30698467	30698468	MACS_peak_9647	3.00	lobChIP_FOXA1_robot
chr20	30844474	30844475	MACS_peak_9648	7.00	lobChIP_FOXA1_robot
chr20	30851240	30851241	MACS_peak_9649	5.00	lobChIP_FOXA1_robot
chr20	30877536	30877537	MACS_peak_9650	13.00	lobChIP_FOXA1_robot
chr20	30913838	30913839	MACS_peak_9651	29.00	lobChIP_FOXA1_robot
chr20	30918201	30918202	MACS_peak_9652	55.00	lobChIP_FOXA1_robot
chr20	30930289	30930290	MACS_peak_9653	5.00	lobChIP_FOXA1_robot
chr20	30943139	30943140	MACS_peak_9654	13.00	lobChIP_FOXA1_robot
chr20	31025593	31025594	MACS_peak_9655	6.00	lobChIP_FOXA1_robot
chr20	31061448	31061449	MACS_peak_9656	9.00	lobChIP_FOXA1_robot
chr20	31073210	31073211	MACS_peak_9657	6.00	lobChIP_FOXA1_robot
chr20	31097705	31097706	MACS_peak_9658	3.00	lobChIP_FOXA1_robot
chr20	31099079	31099080	MACS_peak_9659	8.00	lobChIP_FOXA1_robot
chr20	31131662	31131663	MACS_peak_9660	17.00	lobChIP_FOXA1_robot
chr20	31133141	31133142	MACS_peak_9661	8.00	lobChIP_FOXA1_robot
chr20	31273740	31273741	MACS_peak_9662	15.00	lobChIP_FOXA1_robot
chr20	31593120	31593121	MACS_peak_9663	4.00	lobChIP_FOXA1_robot
chr20	31594002	31594003	MACS_peak_9664	12.00	lobChIP_FOXA1_robot
chr20	31595414	31595415	MACS_peak_9665	10.00	lobChIP_FOXA1_robot
chr20	31597427	31597428	MACS_peak_9666	6.00	lobChIP_FOXA1_robot
chr20	31631541	31631542	MACS_peak_9667	6.00	lobChIP_FOXA1_robot
chr20	31694030	31694031	MACS_peak_9668	10.00	lobChIP_FOXA1_robot
chr20	31715823	31715824	MACS_peak_9669	12.00	lobChIP_FOXA1_robot
chr20	31927652	31927653	MACS_peak_9670	4.00	lobChIP_FOXA1_robot
chr20	31961718	31961719	MACS_peak_9671	31.00	lobChIP_FOXA1_robot
chr20	31989656	31989657	MACS_peak_9672	6.00	lobChIP_FOXA1_robot
chr20	32125810	32125811	MACS_peak_9673	7.00	lobChIP_FOXA1_robot
chr20	32127158	32127159	MACS_peak_9674	8.00	lobChIP_FOXA1_robot
chr20	32127766	32127767	MACS_peak_9675	3.00	lobChIP_FOXA1_robot
chr20	32143009	32143010	MACS_peak_9676	5.00	lobChIP_FOXA1_robot
chr20	32150080	32150081	MACS_peak_9677	22.00	lobChIP_FOXA1_robot
chr20	32151730	32151731	MACS_peak_9678	5.00	lobChIP_FOXA1_robot
chr20	32277067	32277068	MACS_peak_9679	5.00	lobChIP_FOXA1_robot
chr20	32290135	32290136	MACS_peak_9680	4.00	lobChIP_FOXA1_robot
chr20	32309813	32309814	MACS_peak_9681	4.00	lobChIP_FOXA1_robot
chr20	32321796	32321797	MACS_peak_9682	8.00	lobChIP_FOXA1_robot
chr20	32385006	32385007	MACS_peak_9683	9.00	lobChIP_FOXA1_robot
chr20	32497825	32497826	MACS_peak_9684	10.00	lobChIP_FOXA1_robot
chr20	32591421	32591422	MACS_peak_9685	16.00	lobChIP_FOXA1_robot
chr20	32592684	32592685	MACS_peak_9686	39.00	lobChIP_FOXA1_robot
chr20	32712895	32712896	MACS_peak_9687	4.00	lobChIP_FOXA1_robot
chr20	32723556	32723557	MACS_peak_9688	7.00	lobChIP_FOXA1_robot
chr20	32791852	32791853	MACS_peak_9689	4.00	lobChIP_FOXA1_robot
chr20	32842886	32842887	MACS_peak_9690	5.00	lobChIP_FOXA1_robot
chr20	32888837	32888838	MACS_peak_9691	4.00	lobChIP_FOXA1_robot
chr20	32898468	32898469	MACS_peak_9692	48.00	lobChIP_FOXA1_robot
chr20	32899311	32899312	MACS_peak_9693	20.00	lobChIP_FOXA1_robot
chr20	32906921	32906922	MACS_peak_9694	5.00	lobChIP_FOXA1_robot
chr20	32931984	32931985	MACS_peak_9695	4.00	lobChIP_FOXA1_robot
chr20	32991193	32991194	MACS_peak_9696	4.00	lobChIP_FOXA1_robot
chr20	32995768	32995769	MACS_peak_9697	4.00	lobChIP_FOXA1_robot
chr20	33073109	33073110	MACS_peak_9698	5.00	lobChIP_FOXA1_robot
chr20	33099485	33099486	MACS_peak_9699	4.00	lobChIP_FOXA1_robot
chr20	33113754	33113755	MACS_peak_9700	10.00	lobChIP_FOXA1_robot
chr20	33209602	33209603	MACS_peak_9701	24.00	lobChIP_FOXA1_robot
chr20	33225511	33225512	MACS_peak_9702	10.00	lobChIP_FOXA1_robot
chr20	33226356	33226357	MACS_peak_9703	5.00	lobChIP_FOXA1_robot
chr20	33289932	33289933	MACS_peak_9704	4.00	lobChIP_FOXA1_robot
chr20	33291432	33291433	MACS_peak_9705	11.00	lobChIP_FOXA1_robot
chr20	33295330	33295331	MACS_peak_9706	6.00	lobChIP_FOXA1_robot
chr20	33305401	33305402	MACS_peak_9707	10.00	lobChIP_FOXA1_robot
chr20	33410657	33410658	MACS_peak_9708	4.00	lobChIP_FOXA1_robot
chr20	33413947	33413948	MACS_peak_9709	4.00	lobChIP_FOXA1_robot
chr20	33640670	33640671	MACS_peak_9710	6.00	lobChIP_FOXA1_robot
chr20	33717720	33717721	MACS_peak_9711	6.00	lobChIP_FOXA1_robot
chr20	33767527	33767528	MACS_peak_9712	4.00	lobChIP_FOXA1_robot
chr20	33908404	33908405	MACS_peak_9713	7.00	lobChIP_FOXA1_robot
chr20	33915077	33915078	MACS_peak_9714	12.00	lobChIP_FOXA1_robot
chr20	33921761	33921762	MACS_peak_9715	4.00	lobChIP_FOXA1_robot
chr20	33975227	33975228	MACS_peak_9716	3.00	lobChIP_FOXA1_robot
chr20	34055632	34055633	MACS_peak_9717	3.00	lobChIP_FOXA1_robot
chr20	34056788	34056789	MACS_peak_9718	7.00	lobChIP_FOXA1_robot
chr20	34213898	34213899	MACS_peak_9719	4.00	lobChIP_FOXA1_robot
chr20	34224261	34224262	MACS_peak_9720	9.00	lobChIP_FOXA1_robot
chr20	34327953	34327954	MACS_peak_9721	4.00	lobChIP_FOXA1_robot
chr20	34328704	34328705	MACS_peak_9722	5.00	lobChIP_FOXA1_robot
chr20	34332155	34332156	MACS_peak_9723	12.00	lobChIP_FOXA1_robot
chr20	34771258	34771259	MACS_peak_9724	5.00	lobChIP_FOXA1_robot
chr20	34846097	34846098	MACS_peak_9725	12.00	lobChIP_FOXA1_robot
chr20	35114485	35114486	MACS_peak_9726	5.00	lobChIP_FOXA1_robot
chr20	35184949	35184950	MACS_peak_9727	6.00	lobChIP_FOXA1_robot
chr20	35251945	35251946	MACS_peak_9728	19.00	lobChIP_FOXA1_robot
chr20	35363894	35363895	MACS_peak_9729	5.00	lobChIP_FOXA1_robot
chr20	35582819	35582820	MACS_peak_9730	9.00	lobChIP_FOXA1_robot
chr20	35681600	35681601	MACS_peak_9731	4.00	lobChIP_FOXA1_robot
chr20	35748321	35748322	MACS_peak_9732	5.00	lobChIP_FOXA1_robot
chr20	35912841	35912842	MACS_peak_9733	6.00	lobChIP_FOXA1_robot
chr20	36008889	36008890	MACS_peak_9734	13.00	lobChIP_FOXA1_robot
chr20	36027682	36027683	MACS_peak_9735	3.00	lobChIP_FOXA1_robot
chr20	36136256	36136257	MACS_peak_9736	5.00	lobChIP_FOXA1_robot
chr20	36330251	36330252	MACS_peak_9737	5.00	lobChIP_FOXA1_robot
chr20	36352222	36352223	MACS_peak_9738	5.00	lobChIP_FOXA1_robot
chr20	36356224	36356225	MACS_peak_9739	4.00	lobChIP_FOXA1_robot
chr20	36514368	36514369	MACS_peak_9740	6.00	lobChIP_FOXA1_robot
chr20	36688490	36688491	MACS_peak_9741	18.00	lobChIP_FOXA1_robot
chr20	36703784	36703785	MACS_peak_9742	9.00	lobChIP_FOXA1_robot
chr20	36706859	36706860	MACS_peak_9743	3.00	lobChIP_FOXA1_robot
chr20	36711485	36711486	MACS_peak_9744	15.00	lobChIP_FOXA1_robot
chr20	36722364	36722365	MACS_peak_9745	5.00	lobChIP_FOXA1_robot
chr20	36738688	36738689	MACS_peak_9746	4.00	lobChIP_FOXA1_robot
chr20	36751541	36751542	MACS_peak_9747	5.00	lobChIP_FOXA1_robot
chr20	36752434	36752435	MACS_peak_9748	4.00	lobChIP_FOXA1_robot
chr20	36753647	36753648	MACS_peak_9749	4.00	lobChIP_FOXA1_robot
chr20	36754157	36754158	MACS_peak_9750	8.00	lobChIP_FOXA1_robot
chr20	36771891	36771892	MACS_peak_9751	6.00	lobChIP_FOXA1_robot
chr20	36802014	36802015	MACS_peak_9752	4.00	lobChIP_FOXA1_robot
chr20	36825404	36825405	MACS_peak_9753	3.00	lobChIP_FOXA1_robot
chr20	36871349	36871350	MACS_peak_9754	3.00	lobChIP_FOXA1_robot
chr20	37077212	37077213	MACS_peak_9755	10.00	lobChIP_FOXA1_robot
chr20	37092135	37092136	MACS_peak_9756	13.00	lobChIP_FOXA1_robot
chr20	37214397	37214398	MACS_peak_9757	6.00	lobChIP_FOXA1_robot
chr20	37233670	37233671	MACS_peak_9758	22.00	lobChIP_FOXA1_robot
chr20	37295551	37295552	MACS_peak_9759	4.00	lobChIP_FOXA1_robot
chr20	37296346	37296347	MACS_peak_9760	6.00	lobChIP_FOXA1_robot
chr20	37513003	37513004	MACS_peak_9761	4.00	lobChIP_FOXA1_robot
chr20	37542473	37542474	MACS_peak_9762	18.00	lobChIP_FOXA1_robot
chr20	37611088	37611089	MACS_peak_9763	5.00	lobChIP_FOXA1_robot
chr20	37624308	37624309	MACS_peak_9764	8.00	lobChIP_FOXA1_robot
chr20	37629234	37629235	MACS_peak_9765	8.00	lobChIP_FOXA1_robot
chr20	37780927	37780928	MACS_peak_9766	6.00	lobChIP_FOXA1_robot
chr20	37781554	37781555	MACS_peak_9767	5.00	lobChIP_FOXA1_robot
chr20	38018079	38018080	MACS_peak_9768	4.00	lobChIP_FOXA1_robot
chr20	38032335	38032336	MACS_peak_9769	3.00	lobChIP_FOXA1_robot
chr20	38401238	38401239	MACS_peak_9770	6.00	lobChIP_FOXA1_robot
chr20	38860581	38860582	MACS_peak_9771	4.00	lobChIP_FOXA1_robot
chr20	38880146	38880147	MACS_peak_9772	6.00	lobChIP_FOXA1_robot
chr20	39631942	39631943	MACS_peak_9773	27.00	lobChIP_FOXA1_robot
chr20	39748803	39748804	MACS_peak_9774	6.00	lobChIP_FOXA1_robot
chr20	39950493	39950494	MACS_peak_9775	4.00	lobChIP_FOXA1_robot
chr20	40057104	40057105	MACS_peak_9776	7.00	lobChIP_FOXA1_robot
chr20	40126023	40126024	MACS_peak_9777	15.00	lobChIP_FOXA1_robot
chr20	40162348	40162349	MACS_peak_9778	22.00	lobChIP_FOXA1_robot
chr20	40206000	40206001	MACS_peak_9779	8.00	lobChIP_FOXA1_robot
chr20	40223298	40223299	MACS_peak_9780	14.00	lobChIP_FOXA1_robot
chr20	40245212	40245213	MACS_peak_9781	3.00	lobChIP_FOXA1_robot
chr20	40279314	40279315	MACS_peak_9782	9.00	lobChIP_FOXA1_robot
chr20	40322759	40322760	MACS_peak_9783	3.00	lobChIP_FOXA1_robot
chr20	40537801	40537802	MACS_peak_9784	3.00	lobChIP_FOXA1_robot
chr20	41396463	41396464	MACS_peak_9785	4.00	lobChIP_FOXA1_robot
chr20	42093047	42093048	MACS_peak_9786	7.00	lobChIP_FOXA1_robot
chr20	42176304	42176305	MACS_peak_9787	5.00	lobChIP_FOXA1_robot
chr20	42185515	42185516	MACS_peak_9788	4.00	lobChIP_FOXA1_robot
chr20	42186463	42186464	MACS_peak_9789	4.00	lobChIP_FOXA1_robot
chr20	42281758	42281759	MACS_peak_9790	4.00	lobChIP_FOXA1_robot
chr20	42794778	42794779	MACS_peak_9791	15.00	lobChIP_FOXA1_robot
chr20	42897210	42897211	MACS_peak_9792	5.00	lobChIP_FOXA1_robot
chr20	42903210	42903211	MACS_peak_9793	5.00	lobChIP_FOXA1_robot
chr20	42915965	42915966	MACS_peak_9794	5.00	lobChIP_FOXA1_robot
chr20	42931157	42931158	MACS_peak_9795	15.00	lobChIP_FOXA1_robot
chr20	42999364	42999365	MACS_peak_9796	15.00	lobChIP_FOXA1_robot
chr20	43023283	43023284	MACS_peak_9797	91.00	lobChIP_FOXA1_robot
chr20	43029779	43029780	MACS_peak_9798	5.00	lobChIP_FOXA1_robot
chr20	43040038	43040039	MACS_peak_9799	11.00	lobChIP_FOXA1_robot
chr20	43057271	43057272	MACS_peak_9800	32.00	lobChIP_FOXA1_robot
chr20	43090057	43090058	MACS_peak_9801	5.00	lobChIP_FOXA1_robot
chr20	43133431	43133432	MACS_peak_9802	3.00	lobChIP_FOXA1_robot
chr20	43150273	43150274	MACS_peak_9803	4.00	lobChIP_FOXA1_robot
chr20	43281067	43281068	MACS_peak_9804	5.00	lobChIP_FOXA1_robot
chr20	43350310	43350311	MACS_peak_9805	4.00	lobChIP_FOXA1_robot
chr20	43463258	43463259	MACS_peak_9806	4.00	lobChIP_FOXA1_robot
chr20	43590475	43590476	MACS_peak_9807	4.00	lobChIP_FOXA1_robot
chr20	43815654	43815655	MACS_peak_9808	3.00	lobChIP_FOXA1_robot
chr20	43965911	43965912	MACS_peak_9809	6.00	lobChIP_FOXA1_robot
chr20	43972285	43972286	MACS_peak_9810	6.00	lobChIP_FOXA1_robot
chr20	43972909	43972910	MACS_peak_9811	4.00	lobChIP_FOXA1_robot
chr20	43988122	43988123	MACS_peak_9812	4.00	lobChIP_FOXA1_robot
chr20	44205699	44205700	MACS_peak_9813	7.00	lobChIP_FOXA1_robot
chr20	44353978	44353979	MACS_peak_9814	4.00	lobChIP_FOXA1_robot
chr20	44400079	44400080	MACS_peak_9815	9.00	lobChIP_FOXA1_robot
chr20	44458622	44458623	MACS_peak_9816	4.00	lobChIP_FOXA1_robot
chr20	44491648	44491649	MACS_peak_9817	4.00	lobChIP_FOXA1_robot
chr20	44492821	44492822	MACS_peak_9818	3.00	lobChIP_FOXA1_robot
chr20	44553348	44553349	MACS_peak_9819	5.00	lobChIP_FOXA1_robot
chr20	44556171	44556172	MACS_peak_9820	7.00	lobChIP_FOXA1_robot
chr20	44643823	44643824	MACS_peak_9821	12.00	lobChIP_FOXA1_robot
chr20	44713983	44713984	MACS_peak_9822	5.00	lobChIP_FOXA1_robot
chr20	44939828	44939829	MACS_peak_9823	3.00	lobChIP_FOXA1_robot
chr20	44992684	44992685	MACS_peak_9824	8.00	lobChIP_FOXA1_robot
chr20	44993345	44993346	MACS_peak_9825	4.00	lobChIP_FOXA1_robot
chr20	45152484	45152485	MACS_peak_9826	8.00	lobChIP_FOXA1_robot
chr20	45191326	45191327	MACS_peak_9827	7.00	lobChIP_FOXA1_robot
chr20	45213479	45213480	MACS_peak_9828	10.00	lobChIP_FOXA1_robot
chr20	45227092	45227093	MACS_peak_9829	3.00	lobChIP_FOXA1_robot
chr20	45236973	45236974	MACS_peak_9830	4.00	lobChIP_FOXA1_robot
chr20	45255684	45255685	MACS_peak_9831	6.00	lobChIP_FOXA1_robot
chr20	45269554	45269555	MACS_peak_9832	5.00	lobChIP_FOXA1_robot
chr20	45295730	45295731	MACS_peak_9833	18.00	lobChIP_FOXA1_robot
chr20	45299689	45299690	MACS_peak_9834	15.00	lobChIP_FOXA1_robot
chr20	45303069	45303070	MACS_peak_9835	9.00	lobChIP_FOXA1_robot
chr20	45332200	45332201	MACS_peak_9836	4.00	lobChIP_FOXA1_robot
chr20	45411173	45411174	MACS_peak_9837	5.00	lobChIP_FOXA1_robot
chr20	45443752	45443753	MACS_peak_9838	5.00	lobChIP_FOXA1_robot
chr20	45587979	45587980	MACS_peak_9839	4.00	lobChIP_FOXA1_robot
chr20	45637969	45637970	MACS_peak_9840	13.00	lobChIP_FOXA1_robot
chr20	45810073	45810074	MACS_peak_9841	5.00	lobChIP_FOXA1_robot
chr20	45829279	45829280	MACS_peak_9842	8.00	lobChIP_FOXA1_robot
chr20	45831690	45831691	MACS_peak_9843	3.00	lobChIP_FOXA1_robot
chr20	45862345	45862346	MACS_peak_9844	11.00	lobChIP_FOXA1_robot
chr20	45947256	45947257	MACS_peak_9845	4.00	lobChIP_FOXA1_robot
chr20	45950186	45950187	MACS_peak_9846	9.00	lobChIP_FOXA1_robot
chr20	45963006	45963007	MACS_peak_9847	9.00	lobChIP_FOXA1_robot
chr20	45983924	45983925	MACS_peak_9848	5.00	lobChIP_FOXA1_robot
chr20	45985429	45985430	MACS_peak_9849	5.00	lobChIP_FOXA1_robot
chr20	45986979	45986980	MACS_peak_9850	4.00	lobChIP_FOXA1_robot
chr20	45988026	45988027	MACS_peak_9851	5.00	lobChIP_FOXA1_robot
chr20	45989040	45989041	MACS_peak_9852	9.00	lobChIP_FOXA1_robot
chr20	46045904	46045905	MACS_peak_9853	3.00	lobChIP_FOXA1_robot
chr20	46046798	46046799	MACS_peak_9854	10.00	lobChIP_FOXA1_robot
chr20	46080044	46080045	MACS_peak_9855	8.00	lobChIP_FOXA1_robot
chr20	46082094	46082095	MACS_peak_9856	5.00	lobChIP_FOXA1_robot
chr20	46084113	46084114	MACS_peak_9857	55.00	lobChIP_FOXA1_robot
chr20	46093302	46093303	MACS_peak_9858	24.00	lobChIP_FOXA1_robot
chr20	46122053	46122054	MACS_peak_9859	4.00	lobChIP_FOXA1_robot
chr20	46185133	46185134	MACS_peak_9860	4.00	lobChIP_FOXA1_robot
chr20	46205618	46205619	MACS_peak_9861	11.00	lobChIP_FOXA1_robot
chr20	46234246	46234247	MACS_peak_9862	3.00	lobChIP_FOXA1_robot
chr20	46234702	46234703	MACS_peak_9863	13.00	lobChIP_FOXA1_robot
chr20	46271555	46271556	MACS_peak_9864	9.00	lobChIP_FOXA1_robot
chr20	46273376	46273377	MACS_peak_9865	5.00	lobChIP_FOXA1_robot
chr20	46340711	46340712	MACS_peak_9866	3.00	lobChIP_FOXA1_robot
chr20	46540099	46540100	MACS_peak_9867	5.00	lobChIP_FOXA1_robot
chr20	46542025	46542026	MACS_peak_9868	7.00	lobChIP_FOXA1_robot
chr20	46602841	46602842	MACS_peak_9869	4.00	lobChIP_FOXA1_robot
chr20	46924464	46924465	MACS_peak_9870	3.00	lobChIP_FOXA1_robot
chr20	47001934	47001935	MACS_peak_9871	6.00	lobChIP_FOXA1_robot
chr20	47028028	47028029	MACS_peak_9872	4.00	lobChIP_FOXA1_robot
chr20	47113606	47113607	MACS_peak_9873	6.00	lobChIP_FOXA1_robot
chr20	47312921	47312922	MACS_peak_9874	3.00	lobChIP_FOXA1_robot
chr20	47428974	47428975	MACS_peak_9875	7.00	lobChIP_FOXA1_robot
chr20	47537224	47537225	MACS_peak_9876	3.00	lobChIP_FOXA1_robot
chr20	47603758	47603759	MACS_peak_9877	6.00	lobChIP_FOXA1_robot
chr20	47625774	47625775	MACS_peak_9878	36.00	lobChIP_FOXA1_robot
chr20	47654498	47654499	MACS_peak_9879	4.00	lobChIP_FOXA1_robot
chr20	47777436	47777437	MACS_peak_9880	8.00	lobChIP_FOXA1_robot
chr20	47795958	47795959	MACS_peak_9881	11.00	lobChIP_FOXA1_robot
chr20	47802113	47802114	MACS_peak_9882	7.00	lobChIP_FOXA1_robot
chr20	47897971	47897972	MACS_peak_9883	6.00	lobChIP_FOXA1_robot
chr20	47899690	47899691	MACS_peak_9884	4.00	lobChIP_FOXA1_robot
chr20	48096452	48096453	MACS_peak_9885	5.00	lobChIP_FOXA1_robot
chr20	48170008	48170009	MACS_peak_9886	4.00	lobChIP_FOXA1_robot
chr20	48200319	48200320	MACS_peak_9887	7.00	lobChIP_FOXA1_robot
chr20	48221232	48221233	MACS_peak_9888	9.00	lobChIP_FOXA1_robot
chr20	48242546	48242547	MACS_peak_9889	5.00	lobChIP_FOXA1_robot
chr20	48258366	48258367	MACS_peak_9890	7.00	lobChIP_FOXA1_robot
chr20	48292433	48292434	MACS_peak_9891	7.00	lobChIP_FOXA1_robot
chr20	48307599	48307600	MACS_peak_9892	5.00	lobChIP_FOXA1_robot
chr20	48323802	48323803	MACS_peak_9893	4.00	lobChIP_FOXA1_robot
chr20	48345209	48345210	MACS_peak_9894	11.00	lobChIP_FOXA1_robot
chr20	48385093	48385094	MACS_peak_9895	40.00	lobChIP_FOXA1_robot
chr20	48389054	48389055	MACS_peak_9896	7.00	lobChIP_FOXA1_robot
chr20	48449392	48449393	MACS_peak_9897	4.00	lobChIP_FOXA1_robot
chr20	48533188	48533189	MACS_peak_9898	20.00	lobChIP_FOXA1_robot
chr20	48650702	48650703	MACS_peak_9899	3.00	lobChIP_FOXA1_robot
chr20	48782635	48782636	MACS_peak_9900	9.00	lobChIP_FOXA1_robot
chr20	48804151	48804152	MACS_peak_9901	3.00	lobChIP_FOXA1_robot
chr20	48808913	48808914	MACS_peak_9902	8.00	lobChIP_FOXA1_robot
chr20	48889281	48889282	MACS_peak_9903	7.00	lobChIP_FOXA1_robot
chr20	48922024	48922025	MACS_peak_9904	5.00	lobChIP_FOXA1_robot
chr20	48922591	48922592	MACS_peak_9905	7.00	lobChIP_FOXA1_robot
chr20	48983381	48983382	MACS_peak_9906	28.00	lobChIP_FOXA1_robot
chr20	49006102	49006103	MACS_peak_9907	14.00	lobChIP_FOXA1_robot
chr20	49006965	49006966	MACS_peak_9908	12.00	lobChIP_FOXA1_robot
chr20	49015690	49015691	MACS_peak_9909	10.00	lobChIP_FOXA1_robot
chr20	49016790	49016791	MACS_peak_9910	30.00	lobChIP_FOXA1_robot
chr20	49017411	49017412	MACS_peak_9911	5.00	lobChIP_FOXA1_robot
chr20	49021995	49021996	MACS_peak_9912	7.00	lobChIP_FOXA1_robot
chr20	49022695	49022696	MACS_peak_9913	11.00	lobChIP_FOXA1_robot
chr20	49051459	49051460	MACS_peak_9914	8.00	lobChIP_FOXA1_robot
chr20	49057329	49057330	MACS_peak_9915	3.00	lobChIP_FOXA1_robot
chr20	49063306	49063307	MACS_peak_9916	4.00	lobChIP_FOXA1_robot
chr20	49084697	49084698	MACS_peak_9917	3.00	lobChIP_FOXA1_robot
chr20	49180672	49180673	MACS_peak_9918	7.00	lobChIP_FOXA1_robot
chr20	49287215	49287216	MACS_peak_9919	5.00	lobChIP_FOXA1_robot
chr20	49295260	49295261	MACS_peak_9920	4.00	lobChIP_FOXA1_robot
chr20	49346160	49346161	MACS_peak_9921	4.00	lobChIP_FOXA1_robot
chr20	49363111	49363112	MACS_peak_9922	9.00	lobChIP_FOXA1_robot
chr20	49378421	49378422	MACS_peak_9923	9.00	lobChIP_FOXA1_robot
chr20	49380909	49380910	MACS_peak_9924	14.00	lobChIP_FOXA1_robot
chr20	49410179	49410180	MACS_peak_9925	4.00	lobChIP_FOXA1_robot
chr20	49435176	49435177	MACS_peak_9926	13.00	lobChIP_FOXA1_robot
chr20	49511919	49511920	MACS_peak_9927	6.00	lobChIP_FOXA1_robot
chr20	49845348	49845349	MACS_peak_9928	9.00	lobChIP_FOXA1_robot
chr20	50045232	50045233	MACS_peak_9929	5.00	lobChIP_FOXA1_robot
chr20	50245727	50245728	MACS_peak_9930	6.00	lobChIP_FOXA1_robot
chr20	50278385	50278386	MACS_peak_9931	7.00	lobChIP_FOXA1_robot
chr20	50318118	50318119	MACS_peak_9932	11.00	lobChIP_FOXA1_robot
chr20	50319035	50319036	MACS_peak_9933	9.00	lobChIP_FOXA1_robot
chr20	50320567	50320568	MACS_peak_9934	43.00	lobChIP_FOXA1_robot
chr20	50342301	50342302	MACS_peak_9935	3.00	lobChIP_FOXA1_robot
chr20	50356556	50356557	MACS_peak_9936	5.00	lobChIP_FOXA1_robot
chr20	50359548	50359549	MACS_peak_9937	3.00	lobChIP_FOXA1_robot
chr20	50361208	50361209	MACS_peak_9938	22.00	lobChIP_FOXA1_robot
chr20	50361933	50361934	MACS_peak_9939	4.00	lobChIP_FOXA1_robot
chr20	50366967	50366968	MACS_peak_9940	6.00	lobChIP_FOXA1_robot
chr20	50369292	50369293	MACS_peak_9941	6.00	lobChIP_FOXA1_robot
chr20	50375938	50375939	MACS_peak_9942	6.00	lobChIP_FOXA1_robot
chr20	50377736	50377737	MACS_peak_9943	17.00	lobChIP_FOXA1_robot
chr20	50397956	50397957	MACS_peak_9944	18.00	lobChIP_FOXA1_robot
chr20	50427756	50427757	MACS_peak_9945	8.00	lobChIP_FOXA1_robot
chr20	50432076	50432077	MACS_peak_9946	4.00	lobChIP_FOXA1_robot
chr20	50547212	50547213	MACS_peak_9947	4.00	lobChIP_FOXA1_robot
chr20	50618804	50618805	MACS_peak_9948	11.00	lobChIP_FOXA1_robot
chr20	50633612	50633613	MACS_peak_9949	12.00	lobChIP_FOXA1_robot
chr20	50642726	50642727	MACS_peak_9950	10.00	lobChIP_FOXA1_robot
chr20	50648295	50648296	MACS_peak_9951	12.00	lobChIP_FOXA1_robot
chr20	50807314	50807315	MACS_peak_9952	3.00	lobChIP_FOXA1_robot
chr20	50848075	50848076	MACS_peak_9953	8.00	lobChIP_FOXA1_robot
chr20	50851286	50851287	MACS_peak_9954	4.00	lobChIP_FOXA1_robot
chr20	51050103	51050104	MACS_peak_9955	6.00	lobChIP_FOXA1_robot
chr20	51530556	51530557	MACS_peak_9956	4.00	lobChIP_FOXA1_robot
chr20	51541437	51541438	MACS_peak_9957	15.00	lobChIP_FOXA1_robot
chr20	51694260	51694261	MACS_peak_9958	5.00	lobChIP_FOXA1_robot
chr20	51698060	51698061	MACS_peak_9959	8.00	lobChIP_FOXA1_robot
chr20	52130383	52130384	MACS_peak_9960	9.00	lobChIP_FOXA1_robot
chr20	52132412	52132413	MACS_peak_9961	10.00	lobChIP_FOXA1_robot
chr20	52168657	52168658	MACS_peak_9962	9.00	lobChIP_FOXA1_robot
chr20	52196285	52196286	MACS_peak_9963	13.00	lobChIP_FOXA1_robot
chr20	52240285	52240286	MACS_peak_9964	15.00	lobChIP_FOXA1_robot
chr20	52256517	52256518	MACS_peak_9965	6.00	lobChIP_FOXA1_robot
chr20	52259395	52259396	MACS_peak_9966	10.00	lobChIP_FOXA1_robot
chr20	52268850	52268851	MACS_peak_9967	4.00	lobChIP_FOXA1_robot
chr20	52278443	52278444	MACS_peak_9968	3.00	lobChIP_FOXA1_robot
chr20	52294405	52294406	MACS_peak_9969	6.00	lobChIP_FOXA1_robot
chr20	52295938	52295939	MACS_peak_9970	4.00	lobChIP_FOXA1_robot
chr20	52354776	52354777	MACS_peak_9971	6.00	lobChIP_FOXA1_robot
chr20	52356171	52356172	MACS_peak_9972	3.00	lobChIP_FOXA1_robot
chr20	52368423	52368424	MACS_peak_9973	4.00	lobChIP_FOXA1_robot
chr20	52380547	52380548	MACS_peak_9974	12.00	lobChIP_FOXA1_robot
chr20	52385615	52385616	MACS_peak_9975	15.00	lobChIP_FOXA1_robot
chr20	52388452	52388453	MACS_peak_9976	6.00	lobChIP_FOXA1_robot
chr20	52400506	52400507	MACS_peak_9977	5.00	lobChIP_FOXA1_robot
chr20	52403557	52403558	MACS_peak_9978	3.00	lobChIP_FOXA1_robot
chr20	52417526	52417527	MACS_peak_9979	23.00	lobChIP_FOXA1_robot
chr20	52462904	52462905	MACS_peak_9980	9.00	lobChIP_FOXA1_robot
chr20	52480831	52480832	MACS_peak_9981	7.00	lobChIP_FOXA1_robot
chr20	52486452	52486453	MACS_peak_9982	9.00	lobChIP_FOXA1_robot
chr20	52495320	52495321	MACS_peak_9983	8.00	lobChIP_FOXA1_robot
chr20	52571582	52571583	MACS_peak_9984	4.00	lobChIP_FOXA1_robot
chr20	52684343	52684344	MACS_peak_9985	20.00	lobChIP_FOXA1_robot
chr20	52687115	52687116	MACS_peak_9986	11.00	lobChIP_FOXA1_robot
chr20	52701615	52701616	MACS_peak_9987	11.00	lobChIP_FOXA1_robot
chr20	52715940	52715941	MACS_peak_9988	4.00	lobChIP_FOXA1_robot
chr20	52720823	52720824	MACS_peak_9989	6.00	lobChIP_FOXA1_robot
chr20	52724368	52724369	MACS_peak_9990	18.00	lobChIP_FOXA1_robot
chr20	52726766	52726767	MACS_peak_9991	4.00	lobChIP_FOXA1_robot
chr20	52732611	52732612	MACS_peak_9992	8.00	lobChIP_FOXA1_robot
chr20	52740567	52740568	MACS_peak_9993	30.00	lobChIP_FOXA1_robot
chr20	52749424	52749425	MACS_peak_9994	16.00	lobChIP_FOXA1_robot
chr20	52750273	52750274	MACS_peak_9995	10.00	lobChIP_FOXA1_robot
chr20	52757643	52757644	MACS_peak_9996	8.00	lobChIP_FOXA1_robot
chr20	52765704	52765705	MACS_peak_9997	6.00	lobChIP_FOXA1_robot
chr20	52786044	52786045	MACS_peak_9998	6.00	lobChIP_FOXA1_robot
chr20	52794933	52794934	MACS_peak_9999	8.00	lobChIP_FOXA1_robot
chr20	52811479	52811480	MACS_peak_10000	9.00	lobChIP_FOXA1_robot
chr20	53081018	53081019	MACS_peak_10001	4.00	lobChIP_FOXA1_robot
chr20	54049000	54049001	MACS_peak_10002	4.00	lobChIP_FOXA1_robot
chr20	54087997	54087998	MACS_peak_10003	6.00	lobChIP_FOXA1_robot
chr20	54327068	54327069	MACS_peak_10004	13.00	lobChIP_FOXA1_robot
chr20	54995311	54995312	MACS_peak_10005	6.00	lobChIP_FOXA1_robot
chr20	54997648	54997649	MACS_peak_10006	4.00	lobChIP_FOXA1_robot
chr20	55019111	55019112	MACS_peak_10007	4.00	lobChIP_FOXA1_robot
chr20	55049610	55049611	MACS_peak_10008	4.00	lobChIP_FOXA1_robot
chr20	55130003	55130004	MACS_peak_10009	5.00	lobChIP_FOXA1_robot
chr20	55363522	55363523	MACS_peak_10010	11.00	lobChIP_FOXA1_robot
chr20	55404971	55404972	MACS_peak_10011	4.00	lobChIP_FOXA1_robot
chr20	55427147	55427148	MACS_peak_10012	3.00	lobChIP_FOXA1_robot
chr20	55705465	55705466	MACS_peak_10013	4.00	lobChIP_FOXA1_robot
chr20	55839428	55839429	MACS_peak_10014	37.00	lobChIP_FOXA1_robot
chr20	55956902	55956903	MACS_peak_10015	4.00	lobChIP_FOXA1_robot
chr20	56102965	56102966	MACS_peak_10016	4.00	lobChIP_FOXA1_robot
chr20	56109921	56109922	MACS_peak_10017	3.00	lobChIP_FOXA1_robot
chr20	56133555	56133556	MACS_peak_10018	4.00	lobChIP_FOXA1_robot
chr20	56135759	56135760	MACS_peak_10019	7.00	lobChIP_FOXA1_robot
chr20	56809138	56809139	MACS_peak_10020	3.00	lobChIP_FOXA1_robot
chr20	56970916	56970917	MACS_peak_10021	5.00	lobChIP_FOXA1_robot
chr20	56984099	56984100	MACS_peak_10022	6.00	lobChIP_FOXA1_robot
chr20	57226274	57226275	MACS_peak_10023	4.00	lobChIP_FOXA1_robot
chr20	57585986	57585987	MACS_peak_10024	5.00	lobChIP_FOXA1_robot
chr20	57587720	57587721	MACS_peak_10025	17.00	lobChIP_FOXA1_robot
chr20	57591421	57591422	MACS_peak_10026	26.00	lobChIP_FOXA1_robot
chr20	57592180	57592181	MACS_peak_10027	4.00	lobChIP_FOXA1_robot
chr20	57623280	57623281	MACS_peak_10028	6.00	lobChIP_FOXA1_robot
chr20	57869410	57869411	MACS_peak_10029	4.00	lobChIP_FOXA1_robot
chr20	57925931	57925932	MACS_peak_10030	3.00	lobChIP_FOXA1_robot
chr20	58628586	58628587	MACS_peak_10031	10.00	lobChIP_FOXA1_robot
chr20	58737366	58737367	MACS_peak_10032	3.00	lobChIP_FOXA1_robot
chr20	59118576	59118577	MACS_peak_10033	4.00	lobChIP_FOXA1_robot
chr20	59958546	59958547	MACS_peak_10034	4.00	lobChIP_FOXA1_robot
chr20	60346915	60346916	MACS_peak_10035	13.00	lobChIP_FOXA1_robot
chr20	60422623	60422624	MACS_peak_10036	5.00	lobChIP_FOXA1_robot
chr20	60663715	60663716	MACS_peak_10037	6.00	lobChIP_FOXA1_robot
chr20	60946291	60946292	MACS_peak_10038	3.00	lobChIP_FOXA1_robot
chr20	61005420	61005421	MACS_peak_10039	6.00	lobChIP_FOXA1_robot
chr20	61312931	61312932	MACS_peak_10040	9.00	lobChIP_FOXA1_robot
chr20	61503773	61503774	MACS_peak_10041	3.00	lobChIP_FOXA1_robot
chr20	61548389	61548390	MACS_peak_10042	9.00	lobChIP_FOXA1_robot
chr20	61555404	61555405	MACS_peak_10043	7.00	lobChIP_FOXA1_robot
chr20	61586157	61586158	MACS_peak_10044	3.00	lobChIP_FOXA1_robot
chr20	61587064	61587065	MACS_peak_10045	8.00	lobChIP_FOXA1_robot
chr20	61708857	61708858	MACS_peak_10046	3.00	lobChIP_FOXA1_robot
chr20	62140601	62140602	MACS_peak_10047	3.00	lobChIP_FOXA1_robot
chr20	62358348	62358349	MACS_peak_10048	6.00	lobChIP_FOXA1_robot
chr20	62528121	62528122	MACS_peak_10049	6.00	lobChIP_FOXA1_robot
chr20	62604012	62604013	MACS_peak_10050	6.00	lobChIP_FOXA1_robot
chr20	62655638	62655639	MACS_peak_10051	16.00	lobChIP_FOXA1_robot
chr20	62781205	62781206	MACS_peak_10052	5.00	lobChIP_FOXA1_robot
chr20	62820698	62820699	MACS_peak_10053	6.00	lobChIP_FOXA1_robot
chr20	62869765	62869766	MACS_peak_10054	12.00	lobChIP_FOXA1_robot
chr20	62874343	62874344	MACS_peak_10055	12.00	lobChIP_FOXA1_robot
chr21	9832020	9832021	MACS_peak_10056	5.00	lobChIP_FOXA1_robot
chr21	9832621	9832622	MACS_peak_10057	6.00	lobChIP_FOXA1_robot
chr21	9882662	9882663	MACS_peak_10058	4.00	lobChIP_FOXA1_robot
chr21	11083247	11083248	MACS_peak_10060	4.00	lobChIP_FOXA1_robot
chr21	11115210	11115211	MACS_peak_10061	4.00	lobChIP_FOXA1_robot
chr21	16072558	16072559	MACS_peak_10062	29.00	lobChIP_FOXA1_robot
chr21	16237352	16237353	MACS_peak_10063	3.00	lobChIP_FOXA1_robot
chr21	16244811	16244812	MACS_peak_10064	6.00	lobChIP_FOXA1_robot
chr21	16250230	16250231	MACS_peak_10065	7.00	lobChIP_FOXA1_robot
chr21	16251510	16251511	MACS_peak_10066	50.00	lobChIP_FOXA1_robot
chr21	16261578	16261579	MACS_peak_10067	6.00	lobChIP_FOXA1_robot
chr21	16263773	16263774	MACS_peak_10068	8.00	lobChIP_FOXA1_robot
chr21	16315568	16315569	MACS_peak_10069	23.00	lobChIP_FOXA1_robot
chr21	16414127	16414128	MACS_peak_10070	4.00	lobChIP_FOXA1_robot
chr21	16440876	16440877	MACS_peak_10071	5.00	lobChIP_FOXA1_robot
chr21	16494297	16494298	MACS_peak_10072	8.00	lobChIP_FOXA1_robot
chr21	16513047	16513048	MACS_peak_10073	20.00	lobChIP_FOXA1_robot
chr21	16545483	16545484	MACS_peak_10074	16.00	lobChIP_FOXA1_robot
chr21	16568297	16568298	MACS_peak_10075	3.00	lobChIP_FOXA1_robot
chr21	16582801	16582802	MACS_peak_10076	5.00	lobChIP_FOXA1_robot
chr21	16584010	16584011	MACS_peak_10077	33.00	lobChIP_FOXA1_robot
chr21	16594579	16594580	MACS_peak_10078	6.00	lobChIP_FOXA1_robot
chr21	16662940	16662941	MACS_peak_10079	4.00	lobChIP_FOXA1_robot
chr21	16813695	16813696	MACS_peak_10080	9.00	lobChIP_FOXA1_robot
chr21	16816644	16816645	MACS_peak_10081	12.00	lobChIP_FOXA1_robot
chr21	16886905	16886906	MACS_peak_10082	24.00	lobChIP_FOXA1_robot
chr21	17114136	17114137	MACS_peak_10083	8.00	lobChIP_FOXA1_robot
chr21	17452446	17452447	MACS_peak_10084	5.00	lobChIP_FOXA1_robot
chr21	17959859	17959860	MACS_peak_10085	4.00	lobChIP_FOXA1_robot
chr21	18343546	18343547	MACS_peak_10086	5.00	lobChIP_FOXA1_robot
chr21	18741249	18741250	MACS_peak_10087	6.00	lobChIP_FOXA1_robot
chr21	18813845	18813846	MACS_peak_10088	3.00	lobChIP_FOXA1_robot
chr21	18827111	18827112	MACS_peak_10089	3.00	lobChIP_FOXA1_robot
chr21	18828003	18828004	MACS_peak_10090	3.00	lobChIP_FOXA1_robot
chr21	18834163	18834164	MACS_peak_10091	4.00	lobChIP_FOXA1_robot
chr21	18835027	18835028	MACS_peak_10092	4.00	lobChIP_FOXA1_robot
chr21	18840206	18840207	MACS_peak_10093	13.00	lobChIP_FOXA1_robot
chr21	18845152	18845153	MACS_peak_10094	10.00	lobChIP_FOXA1_robot
chr21	18868194	18868195	MACS_peak_10095	5.00	lobChIP_FOXA1_robot
chr21	18873953	18873954	MACS_peak_10096	54.00	lobChIP_FOXA1_robot
chr21	18895524	18895525	MACS_peak_10097	17.00	lobChIP_FOXA1_robot
chr21	18899717	18899718	MACS_peak_10098	10.00	lobChIP_FOXA1_robot
chr21	18914897	18914898	MACS_peak_10099	28.00	lobChIP_FOXA1_robot
chr21	18922265	18922266	MACS_peak_10100	17.00	lobChIP_FOXA1_robot
chr21	19159312	19159313	MACS_peak_10101	11.00	lobChIP_FOXA1_robot
chr21	19872313	19872314	MACS_peak_10102	5.00	lobChIP_FOXA1_robot
chr21	20118372	20118373	MACS_peak_10103	5.00	lobChIP_FOXA1_robot
chr21	24077915	24077916	MACS_peak_10104	3.00	lobChIP_FOXA1_robot
chr21	29473813	29473814	MACS_peak_10105	5.00	lobChIP_FOXA1_robot
chr21	30547907	30547908	MACS_peak_10106	6.00	lobChIP_FOXA1_robot
chr21	30554698	30554699	MACS_peak_10107	4.00	lobChIP_FOXA1_robot
chr21	30566909	30566910	MACS_peak_10108	5.00	lobChIP_FOXA1_robot
chr21	30644439	30644440	MACS_peak_10109	6.00	lobChIP_FOXA1_robot
chr21	30795464	30795465	MACS_peak_10110	5.00	lobChIP_FOXA1_robot
chr21	30920490	30920491	MACS_peak_10111	6.00	lobChIP_FOXA1_robot
chr21	31097615	31097616	MACS_peak_10112	5.00	lobChIP_FOXA1_robot
chr21	31416887	31416888	MACS_peak_10113	4.00	lobChIP_FOXA1_robot
chr21	33288045	33288046	MACS_peak_10114	8.00	lobChIP_FOXA1_robot
chr21	33703331	33703332	MACS_peak_10115	4.00	lobChIP_FOXA1_robot
chr21	33736800	33736801	MACS_peak_10116	3.00	lobChIP_FOXA1_robot
chr21	33965452	33965453	MACS_peak_10117	8.00	lobChIP_FOXA1_robot
chr21	33969692	33969693	MACS_peak_10118	13.00	lobChIP_FOXA1_robot
chr21	34006948	34006949	MACS_peak_10119	3.00	lobChIP_FOXA1_robot
chr21	34108244	34108245	MACS_peak_10120	4.00	lobChIP_FOXA1_robot
chr21	34280793	34280794	MACS_peak_10121	5.00	lobChIP_FOXA1_robot
chr21	34949125	34949126	MACS_peak_10122	9.00	lobChIP_FOXA1_robot
chr21	35060166	35060167	MACS_peak_10123	25.00	lobChIP_FOXA1_robot
chr21	35752893	35752894	MACS_peak_10124	8.00	lobChIP_FOXA1_robot
chr21	35894823	35894824	MACS_peak_10125	8.00	lobChIP_FOXA1_robot
chr21	35899220	35899221	MACS_peak_10126	9.00	lobChIP_FOXA1_robot
chr21	36109201	36109202	MACS_peak_10127	4.00	lobChIP_FOXA1_robot
chr21	36144062	36144063	MACS_peak_10128	4.00	lobChIP_FOXA1_robot
chr21	36147129	36147130	MACS_peak_10129	6.00	lobChIP_FOXA1_robot
chr21	36169308	36169309	MACS_peak_10130	5.00	lobChIP_FOXA1_robot
chr21	36173602	36173603	MACS_peak_10131	5.00	lobChIP_FOXA1_robot
chr21	36208699	36208700	MACS_peak_10132	3.00	lobChIP_FOXA1_robot
chr21	36209078	36209079	MACS_peak_10133	3.00	lobChIP_FOXA1_robot
chr21	36237751	36237752	MACS_peak_10134	17.00	lobChIP_FOXA1_robot
chr21	36281068	36281069	MACS_peak_10135	10.00	lobChIP_FOXA1_robot
chr21	36392244	36392245	MACS_peak_10136	5.00	lobChIP_FOXA1_robot
chr21	36396397	36396398	MACS_peak_10137	4.00	lobChIP_FOXA1_robot
chr21	36399760	36399761	MACS_peak_10138	4.00	lobChIP_FOXA1_robot
chr21	36412022	36412023	MACS_peak_10139	9.00	lobChIP_FOXA1_robot
chr21	36438772	36438773	MACS_peak_10140	3.00	lobChIP_FOXA1_robot
chr21	37141958	37141959	MACS_peak_10141	5.00	lobChIP_FOXA1_robot
chr21	37326053	37326054	MACS_peak_10142	24.00	lobChIP_FOXA1_robot
chr21	37357213	37357214	MACS_peak_10143	3.00	lobChIP_FOXA1_robot
chr21	37367939	37367940	MACS_peak_10144	6.00	lobChIP_FOXA1_robot
chr21	37513900	37513901	MACS_peak_10145	13.00	lobChIP_FOXA1_robot
chr21	37536866	37536867	MACS_peak_10146	13.00	lobChIP_FOXA1_robot
chr21	37560468	37560469	MACS_peak_10147	7.00	lobChIP_FOXA1_robot
chr21	37574705	37574706	MACS_peak_10148	4.00	lobChIP_FOXA1_robot
chr21	37607617	37607618	MACS_peak_10149	18.00	lobChIP_FOXA1_robot
chr21	37985582	37985583	MACS_peak_10150	6.00	lobChIP_FOXA1_robot
chr21	38034020	38034021	MACS_peak_10151	21.00	lobChIP_FOXA1_robot
chr21	38103268	38103269	MACS_peak_10152	10.00	lobChIP_FOXA1_robot
chr21	38232291	38232292	MACS_peak_10153	10.00	lobChIP_FOXA1_robot
chr21	38233616	38233617	MACS_peak_10154	7.00	lobChIP_FOXA1_robot
chr21	38360244	38360245	MACS_peak_10155	13.00	lobChIP_FOXA1_robot
chr21	38432859	38432860	MACS_peak_10156	10.00	lobChIP_FOXA1_robot
chr21	38455357	38455358	MACS_peak_10157	17.00	lobChIP_FOXA1_robot
chr21	38489668	38489669	MACS_peak_10158	9.00	lobChIP_FOXA1_robot
chr21	38526465	38526466	MACS_peak_10159	3.00	lobChIP_FOXA1_robot
chr21	38749293	38749294	MACS_peak_10160	6.00	lobChIP_FOXA1_robot
chr21	38938407	38938408	MACS_peak_10161	5.00	lobChIP_FOXA1_robot
chr21	39896306	39896307	MACS_peak_10162	6.00	lobChIP_FOXA1_robot
chr21	39896808	39896809	MACS_peak_10163	15.00	lobChIP_FOXA1_robot
chr21	39933715	39933716	MACS_peak_10164	8.00	lobChIP_FOXA1_robot
chr21	40157017	40157018	MACS_peak_10165	5.00	lobChIP_FOXA1_robot
chr21	40159606	40159607	MACS_peak_10166	4.00	lobChIP_FOXA1_robot
chr21	40160004	40160005	MACS_peak_10167	8.00	lobChIP_FOXA1_robot
chr21	40174783	40174784	MACS_peak_10168	30.00	lobChIP_FOXA1_robot
chr21	40289085	40289086	MACS_peak_10169	27.00	lobChIP_FOXA1_robot
chr21	40290766	40290767	MACS_peak_10170	3.00	lobChIP_FOXA1_robot
chr21	40292741	40292742	MACS_peak_10171	4.00	lobChIP_FOXA1_robot
chr21	40330854	40330855	MACS_peak_10172	7.00	lobChIP_FOXA1_robot
chr21	40406163	40406164	MACS_peak_10173	4.00	lobChIP_FOXA1_robot
chr21	40437889	40437890	MACS_peak_10174	8.00	lobChIP_FOXA1_robot
chr21	40443895	40443896	MACS_peak_10175	17.00	lobChIP_FOXA1_robot
chr21	40467203	40467204	MACS_peak_10176	26.00	lobChIP_FOXA1_robot
chr21	40504520	40504521	MACS_peak_10177	4.00	lobChIP_FOXA1_robot
chr21	41880088	41880089	MACS_peak_10178	5.00	lobChIP_FOXA1_robot
chr21	42694659	42694660	MACS_peak_10179	32.00	lobChIP_FOXA1_robot
chr21	42697269	42697270	MACS_peak_10180	6.00	lobChIP_FOXA1_robot
chr21	42870055	42870056	MACS_peak_10181	5.00	lobChIP_FOXA1_robot
chr21	43025948	43025949	MACS_peak_10182	7.00	lobChIP_FOXA1_robot
chr21	43127092	43127093	MACS_peak_10183	8.00	lobChIP_FOXA1_robot
chr21	43170833	43170834	MACS_peak_10184	10.00	lobChIP_FOXA1_robot
chr21	43189111	43189112	MACS_peak_10185	5.00	lobChIP_FOXA1_robot
chr21	43346336	43346337	MACS_peak_10186	6.00	lobChIP_FOXA1_robot
chr21	43540249	43540250	MACS_peak_10187	7.00	lobChIP_FOXA1_robot
chr21	43735569	43735570	MACS_peak_10188	7.00	lobChIP_FOXA1_robot
chr21	43806365	43806366	MACS_peak_10189	8.00	lobChIP_FOXA1_robot
chr21	44092260	44092261	MACS_peak_10190	10.00	lobChIP_FOXA1_robot
chr21	44132019	44132020	MACS_peak_10191	5.00	lobChIP_FOXA1_robot
chr21	44153516	44153517	MACS_peak_10192	4.00	lobChIP_FOXA1_robot
chr21	44154613	44154614	MACS_peak_10193	15.00	lobChIP_FOXA1_robot
chr21	44241318	44241319	MACS_peak_10194	4.00	lobChIP_FOXA1_robot
chr21	44345761	44345762	MACS_peak_10195	4.00	lobChIP_FOXA1_robot
chr21	44376075	44376076	MACS_peak_10196	8.00	lobChIP_FOXA1_robot
chr21	44392395	44392396	MACS_peak_10197	4.00	lobChIP_FOXA1_robot
chr21	44493443	44493444	MACS_peak_10198	5.00	lobChIP_FOXA1_robot
chr21	44582848	44582849	MACS_peak_10199	4.00	lobChIP_FOXA1_robot
chr21	44708304	44708305	MACS_peak_10200	14.00	lobChIP_FOXA1_robot
chr21	44719929	44719930	MACS_peak_10201	3.00	lobChIP_FOXA1_robot
chr21	44753372	44753373	MACS_peak_10202	5.00	lobChIP_FOXA1_robot
chr21	44763217	44763218	MACS_peak_10203	4.00	lobChIP_FOXA1_robot
chr21	44861004	44861005	MACS_peak_10204	11.00	lobChIP_FOXA1_robot
chr21	44885079	44885080	MACS_peak_10205	16.00	lobChIP_FOXA1_robot
chr21	44897911	44897912	MACS_peak_10206	4.00	lobChIP_FOXA1_robot
chr21	44922419	44922420	MACS_peak_10207	6.00	lobChIP_FOXA1_robot
chr21	44939806	44939807	MACS_peak_10208	11.00	lobChIP_FOXA1_robot
chr21	44943989	44943990	MACS_peak_10209	6.00	lobChIP_FOXA1_robot
chr21	45006100	45006101	MACS_peak_10210	6.00	lobChIP_FOXA1_robot
chr21	45010307	45010308	MACS_peak_10211	9.00	lobChIP_FOXA1_robot
chr21	45029714	45029715	MACS_peak_10212	5.00	lobChIP_FOXA1_robot
chr21	45664098	45664099	MACS_peak_10213	12.00	lobChIP_FOXA1_robot
chr21	45877814	45877815	MACS_peak_10214	17.00	lobChIP_FOXA1_robot
chr21	46156750	46156751	MACS_peak_10215	10.00	lobChIP_FOXA1_robot
chr21	46213403	46213404	MACS_peak_10216	3.00	lobChIP_FOXA1_robot
chr21	46286844	46286845	MACS_peak_10217	4.00	lobChIP_FOXA1_robot
chr21	46290001	46290002	MACS_peak_10218	5.00	lobChIP_FOXA1_robot
chr21	46290520	46290521	MACS_peak_10219	7.00	lobChIP_FOXA1_robot
chr21	46750178	46750179	MACS_peak_10220	8.00	lobChIP_FOXA1_robot
chr21	46776919	46776920	MACS_peak_10221	5.00	lobChIP_FOXA1_robot
chr21	46872708	46872709	MACS_peak_10222	3.00	lobChIP_FOXA1_robot
chr21	47176086	47176087	MACS_peak_10223	4.00	lobChIP_FOXA1_robot
chr21	47454050	47454051	MACS_peak_10224	6.00	lobChIP_FOXA1_robot
chr21	47929781	47929782	MACS_peak_10225	13.00	lobChIP_FOXA1_robot
chr22	18631362	18631363	MACS_peak_10226	28.00	lobChIP_FOXA1_robot
chr22	18718656	18718657	MACS_peak_10227	3.00	lobChIP_FOXA1_robot
chr22	19591432	19591433	MACS_peak_10229	4.00	lobChIP_FOXA1_robot
chr22	19868965	19868966	MACS_peak_10230	7.00	lobChIP_FOXA1_robot
chr22	19925364	19925365	MACS_peak_10231	3.00	lobChIP_FOXA1_robot
chr22	20658869	20658870	MACS_peak_10232	7.00	lobChIP_FOXA1_robot
chr22	20878511	20878512	MACS_peak_10233	5.00	lobChIP_FOXA1_robot
chr22	21128322	21128323	MACS_peak_10234	23.00	lobChIP_FOXA1_robot
chr22	21131221	21131222	MACS_peak_10235	4.00	lobChIP_FOXA1_robot
chr22	21132212	21132213	MACS_peak_10236	29.00	lobChIP_FOXA1_robot
chr22	21145165	21145166	MACS_peak_10237	11.00	lobChIP_FOXA1_robot
chr22	21181271	21181272	MACS_peak_10238	5.00	lobChIP_FOXA1_robot
chr22	21305717	21305718	MACS_peak_10239	6.00	lobChIP_FOXA1_robot
chr22	21811608	21811609	MACS_peak_10240	11.00	lobChIP_FOXA1_robot
chr22	22211957	22211958	MACS_peak_10241	8.00	lobChIP_FOXA1_robot
chr22	22217080	22217081	MACS_peak_10242	3.00	lobChIP_FOXA1_robot
chr22	22591298	22591299	MACS_peak_10243	5.00	lobChIP_FOXA1_robot
chr22	22741693	22741694	MACS_peak_10244	14.00	lobChIP_FOXA1_robot
chr22	22835487	22835488	MACS_peak_10245	7.00	lobChIP_FOXA1_robot
chr22	23589539	23589540	MACS_peak_10246	13.00	lobChIP_FOXA1_robot
chr22	23864020	23864021	MACS_peak_10247	12.00	lobChIP_FOXA1_robot
chr22	23947250	23947251	MACS_peak_10248	4.00	lobChIP_FOXA1_robot
chr22	23976168	23976169	MACS_peak_10249	4.00	lobChIP_FOXA1_robot
chr22	24116560	24116561	MACS_peak_10250	6.00	lobChIP_FOXA1_robot
chr22	24439657	24439658	MACS_peak_10251	4.00	lobChIP_FOXA1_robot
chr22	24604180	24604181	MACS_peak_10252	17.00	lobChIP_FOXA1_robot
chr22	24685268	24685269	MACS_peak_10253	7.00	lobChIP_FOXA1_robot
chr22	24756915	24756916	MACS_peak_10254	11.00	lobChIP_FOXA1_robot
chr22	24901529	24901530	MACS_peak_10255	9.00	lobChIP_FOXA1_robot
chr22	24903117	24903118	MACS_peak_10256	30.00	lobChIP_FOXA1_robot
chr22	24910310	24910311	MACS_peak_10257	8.00	lobChIP_FOXA1_robot
chr22	24921572	24921573	MACS_peak_10258	4.00	lobChIP_FOXA1_robot
chr22	24946265	24946266	MACS_peak_10259	3.00	lobChIP_FOXA1_robot
chr22	24968034	24968035	MACS_peak_10260	4.00	lobChIP_FOXA1_robot
chr22	24975057	24975058	MACS_peak_10261	23.00	lobChIP_FOXA1_robot
chr22	25001763	25001764	MACS_peak_10262	7.00	lobChIP_FOXA1_robot
chr22	25123252	25123253	MACS_peak_10263	6.00	lobChIP_FOXA1_robot
chr22	25198254	25198255	MACS_peak_10264	4.00	lobChIP_FOXA1_robot
chr22	25225529	25225530	MACS_peak_10265	5.00	lobChIP_FOXA1_robot
chr22	25290672	25290673	MACS_peak_10266	4.00	lobChIP_FOXA1_robot
chr22	25482075	25482076	MACS_peak_10267	5.00	lobChIP_FOXA1_robot
chr22	25484873	25484874	MACS_peak_10268	4.00	lobChIP_FOXA1_robot
chr22	25506324	25506325	MACS_peak_10269	10.00	lobChIP_FOXA1_robot
chr22	25792145	25792146	MACS_peak_10270	5.00	lobChIP_FOXA1_robot
chr22	25797078	25797079	MACS_peak_10271	7.00	lobChIP_FOXA1_robot
chr22	25799807	25799808	MACS_peak_10272	5.00	lobChIP_FOXA1_robot
chr22	26032603	26032604	MACS_peak_10273	3.00	lobChIP_FOXA1_robot
chr22	26055647	26055648	MACS_peak_10274	7.00	lobChIP_FOXA1_robot
chr22	26059761	26059762	MACS_peak_10275	10.00	lobChIP_FOXA1_robot
chr22	26350106	26350107	MACS_peak_10276	10.00	lobChIP_FOXA1_robot
chr22	26592832	26592833	MACS_peak_10277	5.00	lobChIP_FOXA1_robot
chr22	26875793	26875794	MACS_peak_10278	12.00	lobChIP_FOXA1_robot
chr22	28266722	28266723	MACS_peak_10279	8.00	lobChIP_FOXA1_robot
chr22	28282446	28282447	MACS_peak_10280	7.00	lobChIP_FOXA1_robot
chr22	28309157	28309158	MACS_peak_10281	5.00	lobChIP_FOXA1_robot
chr22	28361684	28361685	MACS_peak_10282	4.00	lobChIP_FOXA1_robot
chr22	28473290	28473291	MACS_peak_10283	6.00	lobChIP_FOXA1_robot
chr22	28627642	28627643	MACS_peak_10284	4.00	lobChIP_FOXA1_robot
chr22	28642025	28642026	MACS_peak_10285	6.00	lobChIP_FOXA1_robot
chr22	28763051	28763052	MACS_peak_10286	24.00	lobChIP_FOXA1_robot
chr22	28820890	28820891	MACS_peak_10287	13.00	lobChIP_FOXA1_robot
chr22	28821583	28821584	MACS_peak_10288	4.00	lobChIP_FOXA1_robot
chr22	29108289	29108290	MACS_peak_10289	8.00	lobChIP_FOXA1_robot
chr22	29156106	29156107	MACS_peak_10290	13.00	lobChIP_FOXA1_robot
chr22	29180088	29180089	MACS_peak_10291	8.00	lobChIP_FOXA1_robot
chr22	29204968	29204969	MACS_peak_10292	25.00	lobChIP_FOXA1_robot
chr22	29206939	29206940	MACS_peak_10293	6.00	lobChIP_FOXA1_robot
chr22	29209953	29209954	MACS_peak_10294	9.00	lobChIP_FOXA1_robot
chr22	29214881	29214882	MACS_peak_10295	5.00	lobChIP_FOXA1_robot
chr22	29215691	29215692	MACS_peak_10296	20.00	lobChIP_FOXA1_robot
chr22	29218992	29218993	MACS_peak_10297	6.00	lobChIP_FOXA1_robot
chr22	29261103	29261104	MACS_peak_10298	7.00	lobChIP_FOXA1_robot
chr22	29282238	29282239	MACS_peak_10299	4.00	lobChIP_FOXA1_robot
chr22	29283903	29283904	MACS_peak_10300	6.00	lobChIP_FOXA1_robot
chr22	29308064	29308065	MACS_peak_10301	12.00	lobChIP_FOXA1_robot
chr22	29314162	29314163	MACS_peak_10302	3.00	lobChIP_FOXA1_robot
chr22	29317159	29317160	MACS_peak_10303	4.00	lobChIP_FOXA1_robot
chr22	29320160	29320161	MACS_peak_10304	21.00	lobChIP_FOXA1_robot
chr22	29378146	29378147	MACS_peak_10305	28.00	lobChIP_FOXA1_robot
chr22	29491619	29491620	MACS_peak_10306	9.00	lobChIP_FOXA1_robot
chr22	29492825	29492826	MACS_peak_10307	16.00	lobChIP_FOXA1_robot
chr22	29616425	29616426	MACS_peak_10308	4.00	lobChIP_FOXA1_robot
chr22	29665948	29665949	MACS_peak_10309	5.00	lobChIP_FOXA1_robot
chr22	30021654	30021655	MACS_peak_10310	8.00	lobChIP_FOXA1_robot
chr22	30100755	30100756	MACS_peak_10311	5.00	lobChIP_FOXA1_robot
chr22	30166019	30166020	MACS_peak_10312	9.00	lobChIP_FOXA1_robot
chr22	30182154	30182155	MACS_peak_10313	5.00	lobChIP_FOXA1_robot
chr22	30367173	30367174	MACS_peak_10314	3.00	lobChIP_FOXA1_robot
chr22	30450919	30450920	MACS_peak_10315	4.00	lobChIP_FOXA1_robot
chr22	30515253	30515254	MACS_peak_10316	5.00	lobChIP_FOXA1_robot
chr22	30592042	30592043	MACS_peak_10317	4.00	lobChIP_FOXA1_robot
chr22	30608666	30608667	MACS_peak_10318	9.00	lobChIP_FOXA1_robot
chr22	30800483	30800484	MACS_peak_10319	5.00	lobChIP_FOXA1_robot
chr22	30815077	30815078	MACS_peak_10320	4.00	lobChIP_FOXA1_robot
chr22	30893693	30893694	MACS_peak_10321	7.00	lobChIP_FOXA1_robot
chr22	31451776	31451777	MACS_peak_10322	14.00	lobChIP_FOXA1_robot
chr22	31475043	31475044	MACS_peak_10323	5.00	lobChIP_FOXA1_robot
chr22	31527887	31527888	MACS_peak_10324	8.00	lobChIP_FOXA1_robot
chr22	31615891	31615892	MACS_peak_10325	7.00	lobChIP_FOXA1_robot
chr22	31617891	31617892	MACS_peak_10326	24.00	lobChIP_FOXA1_robot
chr22	31619872	31619873	MACS_peak_10327	3.00	lobChIP_FOXA1_robot
chr22	31627462	31627463	MACS_peak_10328	4.00	lobChIP_FOXA1_robot
chr22	31686653	31686654	MACS_peak_10329	5.00	lobChIP_FOXA1_robot
chr22	31735236	31735237	MACS_peak_10330	5.00	lobChIP_FOXA1_robot
chr22	31793832	31793833	MACS_peak_10331	4.00	lobChIP_FOXA1_robot
chr22	31926699	31926700	MACS_peak_10332	14.00	lobChIP_FOXA1_robot
chr22	31975977	31975978	MACS_peak_10333	19.00	lobChIP_FOXA1_robot
chr22	31981791	31981792	MACS_peak_10334	3.00	lobChIP_FOXA1_robot
chr22	31982712	31982713	MACS_peak_10335	7.00	lobChIP_FOXA1_robot
chr22	32042667	32042668	MACS_peak_10336	5.00	lobChIP_FOXA1_robot
chr22	32209433	32209434	MACS_peak_10337	6.00	lobChIP_FOXA1_robot
chr22	32908864	32908865	MACS_peak_10338	7.00	lobChIP_FOXA1_robot
chr22	33040646	33040647	MACS_peak_10339	6.00	lobChIP_FOXA1_robot
chr22	33076393	33076394	MACS_peak_10340	5.00	lobChIP_FOXA1_robot
chr22	33096394	33096395	MACS_peak_10341	5.00	lobChIP_FOXA1_robot
chr22	33117708	33117709	MACS_peak_10342	8.00	lobChIP_FOXA1_robot
chr22	33176622	33176623	MACS_peak_10343	11.00	lobChIP_FOXA1_robot
chr22	33186157	33186158	MACS_peak_10344	5.00	lobChIP_FOXA1_robot
chr22	33191063	33191064	MACS_peak_10345	6.00	lobChIP_FOXA1_robot
chr22	33345859	33345860	MACS_peak_10346	8.00	lobChIP_FOXA1_robot
chr22	33402865	33402866	MACS_peak_10347	14.00	lobChIP_FOXA1_robot
chr22	33448617	33448618	MACS_peak_10348	3.00	lobChIP_FOXA1_robot
chr22	33526616	33526617	MACS_peak_10349	12.00	lobChIP_FOXA1_robot
chr22	33666831	33666832	MACS_peak_10350	4.00	lobChIP_FOXA1_robot
chr22	33668364	33668365	MACS_peak_10351	10.00	lobChIP_FOXA1_robot
chr22	33732066	33732067	MACS_peak_10352	17.00	lobChIP_FOXA1_robot
chr22	33910057	33910058	MACS_peak_10353	11.00	lobChIP_FOXA1_robot
chr22	33936437	33936438	MACS_peak_10354	36.00	lobChIP_FOXA1_robot
chr22	33974048	33974049	MACS_peak_10355	12.00	lobChIP_FOXA1_robot
chr22	34142739	34142740	MACS_peak_10356	5.00	lobChIP_FOXA1_robot
chr22	35724769	35724770	MACS_peak_10357	5.00	lobChIP_FOXA1_robot
chr22	35793716	35793717	MACS_peak_10358	8.00	lobChIP_FOXA1_robot
chr22	36197454	36197455	MACS_peak_10359	6.00	lobChIP_FOXA1_robot
chr22	36215633	36215634	MACS_peak_10360	8.00	lobChIP_FOXA1_robot
chr22	36220565	36220566	MACS_peak_10361	5.00	lobChIP_FOXA1_robot
chr22	36345514	36345515	MACS_peak_10362	7.00	lobChIP_FOXA1_robot
chr22	36426479	36426480	MACS_peak_10363	6.00	lobChIP_FOXA1_robot
chr22	36648073	36648074	MACS_peak_10364	3.00	lobChIP_FOXA1_robot
chr22	36675572	36675573	MACS_peak_10365	6.00	lobChIP_FOXA1_robot
chr22	36747259	36747260	MACS_peak_10366	6.00	lobChIP_FOXA1_robot
chr22	36757084	36757085	MACS_peak_10367	4.00	lobChIP_FOXA1_robot
chr22	36759547	36759548	MACS_peak_10368	5.00	lobChIP_FOXA1_robot
chr22	36766284	36766285	MACS_peak_10369	10.00	lobChIP_FOXA1_robot
chr22	36815494	36815495	MACS_peak_10370	13.00	lobChIP_FOXA1_robot
chr22	36827228	36827229	MACS_peak_10371	4.00	lobChIP_FOXA1_robot
chr22	36829515	36829516	MACS_peak_10372	12.00	lobChIP_FOXA1_robot
chr22	36830966	36830967	MACS_peak_10373	7.00	lobChIP_FOXA1_robot
chr22	37056288	37056289	MACS_peak_10374	5.00	lobChIP_FOXA1_robot
chr22	37428409	37428410	MACS_peak_10375	3.00	lobChIP_FOXA1_robot
chr22	37429062	37429063	MACS_peak_10376	15.00	lobChIP_FOXA1_robot
chr22	37596027	37596028	MACS_peak_10377	5.00	lobChIP_FOXA1_robot
chr22	37851018	37851019	MACS_peak_10378	6.00	lobChIP_FOXA1_robot
chr22	37897810	37897811	MACS_peak_10379	4.00	lobChIP_FOXA1_robot
chr22	37908548	37908549	MACS_peak_10380	4.00	lobChIP_FOXA1_robot
chr22	37941793	37941794	MACS_peak_10381	11.00	lobChIP_FOXA1_robot
chr22	38156353	38156354	MACS_peak_10382	7.00	lobChIP_FOXA1_robot
chr22	38199090	38199091	MACS_peak_10383	7.00	lobChIP_FOXA1_robot
chr22	38352072	38352073	MACS_peak_10384	5.00	lobChIP_FOXA1_robot
chr22	38574249	38574250	MACS_peak_10385	5.00	lobChIP_FOXA1_robot
chr22	38583557	38583558	MACS_peak_10386	6.00	lobChIP_FOXA1_robot
chr22	38674788	38674789	MACS_peak_10387	4.00	lobChIP_FOXA1_robot
chr22	38705765	38705766	MACS_peak_10388	6.00	lobChIP_FOXA1_robot
chr22	39102880	39102881	MACS_peak_10389	4.00	lobChIP_FOXA1_robot
chr22	39320807	39320808	MACS_peak_10390	9.00	lobChIP_FOXA1_robot
chr22	39571006	39571007	MACS_peak_10391	7.00	lobChIP_FOXA1_robot
chr22	40552192	40552193	MACS_peak_10392	18.00	lobChIP_FOXA1_robot
chr22	40570959	40570960	MACS_peak_10393	5.00	lobChIP_FOXA1_robot
chr22	40577691	40577692	MACS_peak_10394	3.00	lobChIP_FOXA1_robot
chr22	40833806	40833807	MACS_peak_10395	5.00	lobChIP_FOXA1_robot
chr22	40894801	40894802	MACS_peak_10396	9.00	lobChIP_FOXA1_robot
chr22	40895428	40895429	MACS_peak_10397	3.00	lobChIP_FOXA1_robot
chr22	41040897	41040898	MACS_peak_10398	11.00	lobChIP_FOXA1_robot
chr22	41074530	41074531	MACS_peak_10399	5.00	lobChIP_FOXA1_robot
chr22	41107714	41107715	MACS_peak_10400	6.00	lobChIP_FOXA1_robot
chr22	41489689	41489690	MACS_peak_10401	5.00	lobChIP_FOXA1_robot
chr22	41684503	41684504	MACS_peak_10402	5.00	lobChIP_FOXA1_robot
chr22	41756300	41756301	MACS_peak_10403	8.00	lobChIP_FOXA1_robot
chr22	41793439	41793440	MACS_peak_10404	7.00	lobChIP_FOXA1_robot
chr22	42916750	42916751	MACS_peak_10405	8.00	lobChIP_FOXA1_robot
chr22	43150861	43150862	MACS_peak_10406	5.00	lobChIP_FOXA1_robot
chr22	43201254	43201255	MACS_peak_10407	12.00	lobChIP_FOXA1_robot
chr22	43309980	43309981	MACS_peak_10408	13.00	lobChIP_FOXA1_robot
chr22	43336412	43336413	MACS_peak_10409	7.00	lobChIP_FOXA1_robot
chr22	43390015	43390016	MACS_peak_10410	10.00	lobChIP_FOXA1_robot
chr22	43414716	43414717	MACS_peak_10411	4.00	lobChIP_FOXA1_robot
chr22	43443793	43443794	MACS_peak_10412	5.00	lobChIP_FOXA1_robot
chr22	43773918	43773919	MACS_peak_10413	4.00	lobChIP_FOXA1_robot
chr22	43774465	43774466	MACS_peak_10414	3.00	lobChIP_FOXA1_robot
chr22	43793138	43793139	MACS_peak_10415	10.00	lobChIP_FOXA1_robot
chr22	45041100	45041101	MACS_peak_10416	15.00	lobChIP_FOXA1_robot
chr22	45668211	45668212	MACS_peak_10417	4.00	lobChIP_FOXA1_robot
chr22	45669555	45669556	MACS_peak_10418	8.00	lobChIP_FOXA1_robot
chr22	45698117	45698118	MACS_peak_10419	4.00	lobChIP_FOXA1_robot
chr22	45809576	45809577	MACS_peak_10420	4.00	lobChIP_FOXA1_robot
chr22	45993687	45993688	MACS_peak_10421	7.00	lobChIP_FOXA1_robot
chr22	46025804	46025805	MACS_peak_10422	5.00	lobChIP_FOXA1_robot
chr22	46138967	46138968	MACS_peak_10423	41.00	lobChIP_FOXA1_robot
chr22	46440496	46440497	MACS_peak_10424	5.00	lobChIP_FOXA1_robot
chr22	46574802	46574803	MACS_peak_10425	8.00	lobChIP_FOXA1_robot
chr22	46773659	46773660	MACS_peak_10426	6.00	lobChIP_FOXA1_robot
chr22	46924160	46924161	MACS_peak_10427	7.00	lobChIP_FOXA1_robot
chr22	47005376	47005377	MACS_peak_10428	4.00	lobChIP_FOXA1_robot
chr22	47035016	47035017	MACS_peak_10429	5.00	lobChIP_FOXA1_robot
chr22	47100892	47100893	MACS_peak_10430	5.00	lobChIP_FOXA1_robot
chr22	47197377	47197378	MACS_peak_10431	6.00	lobChIP_FOXA1_robot
chr22	47212262	47212263	MACS_peak_10432	4.00	lobChIP_FOXA1_robot
chr22	47483096	47483097	MACS_peak_10433	4.00	lobChIP_FOXA1_robot
chr22	47498764	47498765	MACS_peak_10434	7.00	lobChIP_FOXA1_robot
chr22	48007350	48007351	MACS_peak_10435	3.00	lobChIP_FOXA1_robot
chr22	48766032	48766033	MACS_peak_10436	8.00	lobChIP_FOXA1_robot
chr22	49314623	49314624	MACS_peak_10437	13.00	lobChIP_FOXA1_robot
chr22	50331627	50331628	MACS_peak_10438	4.00	lobChIP_FOXA1_robot
chr22	50444912	50444913	MACS_peak_10439	5.00	lobChIP_FOXA1_robot
chr22	50580443	50580444	MACS_peak_10440	6.00	lobChIP_FOXA1_robot
chr22	50732406	50732407	MACS_peak_10441	3.00	lobChIP_FOXA1_robot
chr22	50825932	50825933	MACS_peak_10442	13.00	lobChIP_FOXA1_robot
chr22	50835635	50835636	MACS_peak_10443	51.00	lobChIP_FOXA1_robot
chr22	51080607	51080608	MACS_peak_10444	7.00	lobChIP_FOXA1_robot
chr3	138081	138082	MACS_peak_10445	4.00	lobChIP_FOXA1_robot
chr3	290862	290863	MACS_peak_10446	3.00	lobChIP_FOXA1_robot
chr3	306484	306485	MACS_peak_10447	12.00	lobChIP_FOXA1_robot
chr3	2956481	2956482	MACS_peak_10448	3.00	lobChIP_FOXA1_robot
chr3	3192859	3192860	MACS_peak_10449	4.00	lobChIP_FOXA1_robot
chr3	3412241	3412242	MACS_peak_10450	3.00	lobChIP_FOXA1_robot
chr3	4468596	4468597	MACS_peak_10451	8.00	lobChIP_FOXA1_robot
chr3	4507608	4507609	MACS_peak_10452	3.00	lobChIP_FOXA1_robot
chr3	4554882	4554883	MACS_peak_10453	4.00	lobChIP_FOXA1_robot
chr3	4728997	4728998	MACS_peak_10454	7.00	lobChIP_FOXA1_robot
chr3	4786439	4786440	MACS_peak_10455	6.00	lobChIP_FOXA1_robot
chr3	5028630	5028631	MACS_peak_10456	9.00	lobChIP_FOXA1_robot
chr3	5058359	5058360	MACS_peak_10457	7.00	lobChIP_FOXA1_robot
chr3	5064159	5064160	MACS_peak_10458	8.00	lobChIP_FOXA1_robot
chr3	5180052	5180053	MACS_peak_10459	6.00	lobChIP_FOXA1_robot
chr3	5373855	5373856	MACS_peak_10460	6.00	lobChIP_FOXA1_robot
chr3	8486248	8486249	MACS_peak_10461	12.00	lobChIP_FOXA1_robot
chr3	8528841	8528842	MACS_peak_10462	15.00	lobChIP_FOXA1_robot
chr3	8531308	8531309	MACS_peak_10463	3.00	lobChIP_FOXA1_robot
chr3	8541194	8541195	MACS_peak_10464	4.00	lobChIP_FOXA1_robot
chr3	8697295	8697296	MACS_peak_10465	9.00	lobChIP_FOXA1_robot
chr3	9006976	9006977	MACS_peak_10466	8.00	lobChIP_FOXA1_robot
chr3	9246237	9246238	MACS_peak_10467	4.00	lobChIP_FOXA1_robot
chr3	9395287	9395288	MACS_peak_10468	18.00	lobChIP_FOXA1_robot
chr3	9470371	9470372	MACS_peak_10469	5.00	lobChIP_FOXA1_robot
chr3	9691080	9691081	MACS_peak_10470	5.00	lobChIP_FOXA1_robot
chr3	9939941	9939942	MACS_peak_10471	8.00	lobChIP_FOXA1_robot
chr3	10018928	10018929	MACS_peak_10472	5.00	lobChIP_FOXA1_robot
chr3	10244338	10244339	MACS_peak_10473	5.00	lobChIP_FOXA1_robot
chr3	10245728	10245729	MACS_peak_10474	9.00	lobChIP_FOXA1_robot
chr3	10266640	10266641	MACS_peak_10475	5.00	lobChIP_FOXA1_robot
chr3	10292005	10292006	MACS_peak_10476	5.00	lobChIP_FOXA1_robot
chr3	10334589	10334590	MACS_peak_10477	6.00	lobChIP_FOXA1_robot
chr3	10550449	10550450	MACS_peak_10478	6.00	lobChIP_FOXA1_robot
chr3	10786829	10786830	MACS_peak_10479	6.00	lobChIP_FOXA1_robot
chr3	10854182	10854183	MACS_peak_10480	21.00	lobChIP_FOXA1_robot
chr3	10864932	10864933	MACS_peak_10481	5.00	lobChIP_FOXA1_robot
chr3	11522571	11522572	MACS_peak_10482	5.00	lobChIP_FOXA1_robot
chr3	11642024	11642025	MACS_peak_10483	5.00	lobChIP_FOXA1_robot
chr3	11660456	11660457	MACS_peak_10484	5.00	lobChIP_FOXA1_robot
chr3	11695125	11695126	MACS_peak_10485	6.00	lobChIP_FOXA1_robot
chr3	12350156	12350157	MACS_peak_10486	4.00	lobChIP_FOXA1_robot
chr3	12358650	12358651	MACS_peak_10487	3.00	lobChIP_FOXA1_robot
chr3	12450677	12450678	MACS_peak_10488	27.00	lobChIP_FOXA1_robot
chr3	12451926	12451927	MACS_peak_10489	14.00	lobChIP_FOXA1_robot
chr3	12689248	12689249	MACS_peak_10490	8.00	lobChIP_FOXA1_robot
chr3	12712478	12712479	MACS_peak_10491	4.00	lobChIP_FOXA1_robot
chr3	12797341	12797342	MACS_peak_10492	6.00	lobChIP_FOXA1_robot
chr3	13006431	13006432	MACS_peak_10493	11.00	lobChIP_FOXA1_robot
chr3	13236471	13236472	MACS_peak_10494	4.00	lobChIP_FOXA1_robot
chr3	13293095	13293096	MACS_peak_10495	4.00	lobChIP_FOXA1_robot
chr3	13304344	13304345	MACS_peak_10496	7.00	lobChIP_FOXA1_robot
chr3	13503230	13503231	MACS_peak_10497	5.00	lobChIP_FOXA1_robot
chr3	13512464	13512465	MACS_peak_10498	6.00	lobChIP_FOXA1_robot
chr3	13517851	13517852	MACS_peak_10499	8.00	lobChIP_FOXA1_robot
chr3	14307175	14307176	MACS_peak_10500	6.00	lobChIP_FOXA1_robot
chr3	14464924	14464925	MACS_peak_10501	4.00	lobChIP_FOXA1_robot
chr3	14984673	14984674	MACS_peak_10502	6.00	lobChIP_FOXA1_robot
chr3	15212825	15212826	MACS_peak_10503	37.00	lobChIP_FOXA1_robot
chr3	15265388	15265389	MACS_peak_10504	6.00	lobChIP_FOXA1_robot
chr3	15643156	15643157	MACS_peak_10505	13.00	lobChIP_FOXA1_robot
chr3	15648123	15648124	MACS_peak_10506	4.00	lobChIP_FOXA1_robot
chr3	15810596	15810597	MACS_peak_10507	5.00	lobChIP_FOXA1_robot
chr3	15833234	15833235	MACS_peak_10508	6.00	lobChIP_FOXA1_robot
chr3	16009315	16009316	MACS_peak_10509	3.00	lobChIP_FOXA1_robot
chr3	16102082	16102083	MACS_peak_10510	10.00	lobChIP_FOXA1_robot
chr3	16110714	16110715	MACS_peak_10511	8.00	lobChIP_FOXA1_robot
chr3	16112982	16112983	MACS_peak_10512	5.00	lobChIP_FOXA1_robot
chr3	16144496	16144497	MACS_peak_10513	4.00	lobChIP_FOXA1_robot
chr3	16365934	16365935	MACS_peak_10514	7.00	lobChIP_FOXA1_robot
chr3	16372952	16372953	MACS_peak_10515	20.00	lobChIP_FOXA1_robot
chr3	16374562	16374563	MACS_peak_10516	13.00	lobChIP_FOXA1_robot
chr3	17462396	17462397	MACS_peak_10517	3.00	lobChIP_FOXA1_robot
chr3	20362181	20362182	MACS_peak_10518	4.00	lobChIP_FOXA1_robot
chr3	22461047	22461048	MACS_peak_10519	4.00	lobChIP_FOXA1_robot
chr3	23942499	23942500	MACS_peak_10520	4.00	lobChIP_FOXA1_robot
chr3	23955949	23955950	MACS_peak_10521	3.00	lobChIP_FOXA1_robot
chr3	23991403	23991404	MACS_peak_10522	6.00	lobChIP_FOXA1_robot
chr3	24314504	24314505	MACS_peak_10523	4.00	lobChIP_FOXA1_robot
chr3	24315219	24315220	MACS_peak_10524	7.00	lobChIP_FOXA1_robot
chr3	24405023	24405024	MACS_peak_10525	10.00	lobChIP_FOXA1_robot
chr3	24406663	24406664	MACS_peak_10526	4.00	lobChIP_FOXA1_robot
chr3	24414960	24414961	MACS_peak_10527	3.00	lobChIP_FOXA1_robot
chr3	24417621	24417622	MACS_peak_10528	7.00	lobChIP_FOXA1_robot
chr3	24492259	24492260	MACS_peak_10529	6.00	lobChIP_FOXA1_robot
chr3	24493330	24493331	MACS_peak_10530	18.00	lobChIP_FOXA1_robot
chr3	24564796	24564797	MACS_peak_10531	3.00	lobChIP_FOXA1_robot
chr3	24661963	24661964	MACS_peak_10532	4.00	lobChIP_FOXA1_robot
chr3	25472078	25472079	MACS_peak_10533	5.00	lobChIP_FOXA1_robot
chr3	25876722	25876723	MACS_peak_10534	3.00	lobChIP_FOXA1_robot
chr3	26150866	26150867	MACS_peak_10535	4.00	lobChIP_FOXA1_robot
chr3	27156437	27156438	MACS_peak_10536	5.00	lobChIP_FOXA1_robot
chr3	27156993	27156994	MACS_peak_10537	6.00	lobChIP_FOXA1_robot
chr3	27168890	27168891	MACS_peak_10538	17.00	lobChIP_FOXA1_robot
chr3	27172500	27172501	MACS_peak_10539	14.00	lobChIP_FOXA1_robot
chr3	27174863	27174864	MACS_peak_10540	4.00	lobChIP_FOXA1_robot
chr3	27323537	27323538	MACS_peak_10541	18.00	lobChIP_FOXA1_robot
chr3	27358844	27358845	MACS_peak_10542	12.00	lobChIP_FOXA1_robot
chr3	27377233	27377234	MACS_peak_10543	9.00	lobChIP_FOXA1_robot
chr3	27402174	27402175	MACS_peak_10544	54.00	lobChIP_FOXA1_robot
chr3	27407918	27407919	MACS_peak_10545	6.00	lobChIP_FOXA1_robot
chr3	27418071	27418072	MACS_peak_10546	17.00	lobChIP_FOXA1_robot
chr3	27575066	27575067	MACS_peak_10547	5.00	lobChIP_FOXA1_robot
chr3	27575625	27575626	MACS_peak_10548	9.00	lobChIP_FOXA1_robot
chr3	27664822	27664823	MACS_peak_10549	18.00	lobChIP_FOXA1_robot
chr3	28156627	28156628	MACS_peak_10550	7.00	lobChIP_FOXA1_robot
chr3	28181701	28181702	MACS_peak_10551	5.00	lobChIP_FOXA1_robot
chr3	28362659	28362660	MACS_peak_10552	13.00	lobChIP_FOXA1_robot
chr3	30016600	30016601	MACS_peak_10553	3.00	lobChIP_FOXA1_robot
chr3	30235472	30235473	MACS_peak_10554	28.00	lobChIP_FOXA1_robot
chr3	30269626	30269627	MACS_peak_10555	4.00	lobChIP_FOXA1_robot
chr3	30300480	30300481	MACS_peak_10556	61.00	lobChIP_FOXA1_robot
chr3	30331186	30331187	MACS_peak_10557	4.00	lobChIP_FOXA1_robot
chr3	30368282	30368283	MACS_peak_10558	5.00	lobChIP_FOXA1_robot
chr3	30385884	30385885	MACS_peak_10559	7.00	lobChIP_FOXA1_robot
chr3	30389632	30389633	MACS_peak_10560	7.00	lobChIP_FOXA1_robot
chr3	30481773	30481774	MACS_peak_10561	4.00	lobChIP_FOXA1_robot
chr3	30518396	30518397	MACS_peak_10562	4.00	lobChIP_FOXA1_robot
chr3	30534356	30534357	MACS_peak_10563	15.00	lobChIP_FOXA1_robot
chr3	30553271	30553272	MACS_peak_10564	7.00	lobChIP_FOXA1_robot
chr3	30559936	30559937	MACS_peak_10565	5.00	lobChIP_FOXA1_robot
chr3	30588452	30588453	MACS_peak_10566	5.00	lobChIP_FOXA1_robot
chr3	30599609	30599610	MACS_peak_10567	4.00	lobChIP_FOXA1_robot
chr3	30638993	30638994	MACS_peak_10568	19.00	lobChIP_FOXA1_robot
chr3	30647842	30647843	MACS_peak_10569	9.00	lobChIP_FOXA1_robot
chr3	30667741	30667742	MACS_peak_10570	19.00	lobChIP_FOXA1_robot
chr3	30701026	30701027	MACS_peak_10571	12.00	lobChIP_FOXA1_robot
chr3	30734111	30734112	MACS_peak_10572	8.00	lobChIP_FOXA1_robot
chr3	30762703	30762704	MACS_peak_10573	12.00	lobChIP_FOXA1_robot
chr3	30812308	30812309	MACS_peak_10574	3.00	lobChIP_FOXA1_robot
chr3	31289864	31289865	MACS_peak_10575	5.00	lobChIP_FOXA1_robot
chr3	31316684	31316685	MACS_peak_10576	13.00	lobChIP_FOXA1_robot
chr3	31330331	31330332	MACS_peak_10577	10.00	lobChIP_FOXA1_robot
chr3	31388359	31388360	MACS_peak_10578	6.00	lobChIP_FOXA1_robot
chr3	31418778	31418779	MACS_peak_10579	34.00	lobChIP_FOXA1_robot
chr3	31678390	31678391	MACS_peak_10580	5.00	lobChIP_FOXA1_robot
chr3	31728916	31728917	MACS_peak_10581	3.00	lobChIP_FOXA1_robot
chr3	31821164	31821165	MACS_peak_10582	5.00	lobChIP_FOXA1_robot
chr3	31880913	31880914	MACS_peak_10583	4.00	lobChIP_FOXA1_robot
chr3	31961270	31961271	MACS_peak_10584	12.00	lobChIP_FOXA1_robot
chr3	31982746	31982747	MACS_peak_10585	9.00	lobChIP_FOXA1_robot
chr3	31986802	31986803	MACS_peak_10586	31.00	lobChIP_FOXA1_robot
chr3	32002680	32002681	MACS_peak_10587	7.00	lobChIP_FOXA1_robot
chr3	32010609	32010610	MACS_peak_10588	6.00	lobChIP_FOXA1_robot
chr3	32016011	32016012	MACS_peak_10589	3.00	lobChIP_FOXA1_robot
chr3	32059898	32059899	MACS_peak_10590	4.00	lobChIP_FOXA1_robot
chr3	32086967	32086968	MACS_peak_10591	17.00	lobChIP_FOXA1_robot
chr3	32137108	32137109	MACS_peak_10592	29.00	lobChIP_FOXA1_robot
chr3	32171450	32171451	MACS_peak_10593	4.00	lobChIP_FOXA1_robot
chr3	32227732	32227733	MACS_peak_10594	5.00	lobChIP_FOXA1_robot
chr3	32257757	32257758	MACS_peak_10595	40.00	lobChIP_FOXA1_robot
chr3	32323423	32323424	MACS_peak_10596	4.00	lobChIP_FOXA1_robot
chr3	32334124	32334125	MACS_peak_10597	4.00	lobChIP_FOXA1_robot
chr3	32338460	32338461	MACS_peak_10598	8.00	lobChIP_FOXA1_robot
chr3	32346099	32346100	MACS_peak_10599	6.00	lobChIP_FOXA1_robot
chr3	32407619	32407620	MACS_peak_10600	4.00	lobChIP_FOXA1_robot
chr3	32447397	32447398	MACS_peak_10601	5.00	lobChIP_FOXA1_robot
chr3	32542033	32542034	MACS_peak_10602	3.00	lobChIP_FOXA1_robot
chr3	32599354	32599355	MACS_peak_10603	49.00	lobChIP_FOXA1_robot
chr3	32672033	32672034	MACS_peak_10604	3.00	lobChIP_FOXA1_robot
chr3	32816030	32816031	MACS_peak_10605	6.00	lobChIP_FOXA1_robot
chr3	32836340	32836341	MACS_peak_10606	11.00	lobChIP_FOXA1_robot
chr3	32865049	32865050	MACS_peak_10607	4.00	lobChIP_FOXA1_robot
chr3	32869307	32869308	MACS_peak_10608	10.00	lobChIP_FOXA1_robot
chr3	32881792	32881793	MACS_peak_10609	11.00	lobChIP_FOXA1_robot
chr3	32925200	32925201	MACS_peak_10610	5.00	lobChIP_FOXA1_robot
chr3	33109096	33109097	MACS_peak_10611	11.00	lobChIP_FOXA1_robot
chr3	33111542	33111543	MACS_peak_10612	7.00	lobChIP_FOXA1_robot
chr3	33124476	33124477	MACS_peak_10613	3.00	lobChIP_FOXA1_robot
chr3	33140818	33140819	MACS_peak_10614	5.00	lobChIP_FOXA1_robot
chr3	33483343	33483344	MACS_peak_10615	7.00	lobChIP_FOXA1_robot
chr3	33618961	33618962	MACS_peak_10616	4.00	lobChIP_FOXA1_robot
chr3	33620584	33620585	MACS_peak_10617	9.00	lobChIP_FOXA1_robot
chr3	33639012	33639013	MACS_peak_10618	5.00	lobChIP_FOXA1_robot
chr3	34053748	34053749	MACS_peak_10619	15.00	lobChIP_FOXA1_robot
chr3	34055530	34055531	MACS_peak_10620	6.00	lobChIP_FOXA1_robot
chr3	36961413	36961414	MACS_peak_10621	6.00	lobChIP_FOXA1_robot
chr3	37409229	37409230	MACS_peak_10622	5.00	lobChIP_FOXA1_robot
chr3	37905857	37905858	MACS_peak_10623	11.00	lobChIP_FOXA1_robot
chr3	37949447	37949448	MACS_peak_10624	4.00	lobChIP_FOXA1_robot
chr3	37951186	37951187	MACS_peak_10625	4.00	lobChIP_FOXA1_robot
chr3	37976504	37976505	MACS_peak_10626	6.00	lobChIP_FOXA1_robot
chr3	37977905	37977906	MACS_peak_10627	14.00	lobChIP_FOXA1_robot
chr3	38028635	38028636	MACS_peak_10628	6.00	lobChIP_FOXA1_robot
chr3	38046503	38046504	MACS_peak_10629	7.00	lobChIP_FOXA1_robot
chr3	38100207	38100208	MACS_peak_10630	4.00	lobChIP_FOXA1_robot
chr3	38175303	38175304	MACS_peak_10631	7.00	lobChIP_FOXA1_robot
chr3	38429522	38429523	MACS_peak_10632	10.00	lobChIP_FOXA1_robot
chr3	38613670	38613671	MACS_peak_10633	8.00	lobChIP_FOXA1_robot
chr3	39093483	39093484	MACS_peak_10634	8.00	lobChIP_FOXA1_robot
chr3	39359010	39359011	MACS_peak_10635	6.00	lobChIP_FOXA1_robot
chr3	39847338	39847339	MACS_peak_10636	4.00	lobChIP_FOXA1_robot
chr3	40129124	40129125	MACS_peak_10637	10.00	lobChIP_FOXA1_robot
chr3	40871449	40871450	MACS_peak_10638	4.00	lobChIP_FOXA1_robot
chr3	40885000	40885001	MACS_peak_10639	20.00	lobChIP_FOXA1_robot
chr3	40937993	40937994	MACS_peak_10640	5.00	lobChIP_FOXA1_robot
chr3	40953717	40953718	MACS_peak_10641	6.00	lobChIP_FOXA1_robot
chr3	41244374	41244375	MACS_peak_10642	5.00	lobChIP_FOXA1_robot
chr3	41309687	41309688	MACS_peak_10643	18.00	lobChIP_FOXA1_robot
chr3	41316999	41317000	MACS_peak_10644	8.00	lobChIP_FOXA1_robot
chr3	41556407	41556408	MACS_peak_10645	8.00	lobChIP_FOXA1_robot
chr3	42060815	42060816	MACS_peak_10646	4.00	lobChIP_FOXA1_robot
chr3	42074409	42074410	MACS_peak_10647	5.00	lobChIP_FOXA1_robot
chr3	42085063	42085064	MACS_peak_10648	4.00	lobChIP_FOXA1_robot
chr3	42096975	42096976	MACS_peak_10649	4.00	lobChIP_FOXA1_robot
chr3	42172002	42172003	MACS_peak_10650	88.00	lobChIP_FOXA1_robot
chr3	42463883	42463884	MACS_peak_10651	20.00	lobChIP_FOXA1_robot
chr3	42774001	42774002	MACS_peak_10652	6.00	lobChIP_FOXA1_robot
chr3	42861024	42861025	MACS_peak_10653	3.00	lobChIP_FOXA1_robot
chr3	42871474	42871475	MACS_peak_10654	11.00	lobChIP_FOXA1_robot
chr3	42895443	42895444	MACS_peak_10655	20.00	lobChIP_FOXA1_robot
chr3	42897029	42897030	MACS_peak_10656	9.00	lobChIP_FOXA1_robot
chr3	42956196	42956197	MACS_peak_10657	4.00	lobChIP_FOXA1_robot
chr3	43336734	43336735	MACS_peak_10658	5.00	lobChIP_FOXA1_robot
chr3	43354006	43354007	MACS_peak_10659	4.00	lobChIP_FOXA1_robot
chr3	43358684	43358685	MACS_peak_10660	11.00	lobChIP_FOXA1_robot
chr3	44362994	44362995	MACS_peak_10661	18.00	lobChIP_FOXA1_robot
chr3	44400775	44400776	MACS_peak_10662	8.00	lobChIP_FOXA1_robot
chr3	45043892	45043893	MACS_peak_10663	4.00	lobChIP_FOXA1_robot
chr3	45233829	45233830	MACS_peak_10664	15.00	lobChIP_FOXA1_robot
chr3	45452719	45452720	MACS_peak_10665	4.00	lobChIP_FOXA1_robot
chr3	45659214	45659215	MACS_peak_10666	4.00	lobChIP_FOXA1_robot
chr3	45680715	45680716	MACS_peak_10667	14.00	lobChIP_FOXA1_robot
chr3	45688806	45688807	MACS_peak_10668	5.00	lobChIP_FOXA1_robot
chr3	45698492	45698493	MACS_peak_10669	7.00	lobChIP_FOXA1_robot
chr3	45701282	45701283	MACS_peak_10670	3.00	lobChIP_FOXA1_robot
chr3	46028102	46028103	MACS_peak_10671	6.00	lobChIP_FOXA1_robot
chr3	46977207	46977208	MACS_peak_10672	5.00	lobChIP_FOXA1_robot
chr3	46989874	46989875	MACS_peak_10673	6.00	lobChIP_FOXA1_robot
chr3	47016371	47016372	MACS_peak_10674	4.00	lobChIP_FOXA1_robot
chr3	47060043	47060044	MACS_peak_10675	9.00	lobChIP_FOXA1_robot
chr3	47077193	47077194	MACS_peak_10676	6.00	lobChIP_FOXA1_robot
chr3	47091288	47091289	MACS_peak_10677	4.00	lobChIP_FOXA1_robot
chr3	47107471	47107472	MACS_peak_10678	6.00	lobChIP_FOXA1_robot
chr3	47324037	47324038	MACS_peak_10679	9.00	lobChIP_FOXA1_robot
chr3	47365938	47365939	MACS_peak_10680	4.00	lobChIP_FOXA1_robot
chr3	47421283	47421284	MACS_peak_10681	5.00	lobChIP_FOXA1_robot
chr3	47466424	47466425	MACS_peak_10682	11.00	lobChIP_FOXA1_robot
chr3	47696321	47696322	MACS_peak_10683	3.00	lobChIP_FOXA1_robot
chr3	47758755	47758756	MACS_peak_10684	3.00	lobChIP_FOXA1_robot
chr3	47996363	47996364	MACS_peak_10685	11.00	lobChIP_FOXA1_robot
chr3	48041872	48041873	MACS_peak_10686	7.00	lobChIP_FOXA1_robot
chr3	48081533	48081534	MACS_peak_10687	3.00	lobChIP_FOXA1_robot
chr3	48471584	48471585	MACS_peak_10688	7.00	lobChIP_FOXA1_robot
chr3	48472899	48472900	MACS_peak_10689	7.00	lobChIP_FOXA1_robot
chr3	48674806	48674807	MACS_peak_10690	3.00	lobChIP_FOXA1_robot
chr3	48934686	48934687	MACS_peak_10691	4.00	lobChIP_FOXA1_robot
chr3	49336209	49336210	MACS_peak_10692	5.00	lobChIP_FOXA1_robot
chr3	49607961	49607962	MACS_peak_10693	7.00	lobChIP_FOXA1_robot
chr3	49708844	49708845	MACS_peak_10694	5.00	lobChIP_FOXA1_robot
chr3	50246452	50246453	MACS_peak_10695	4.00	lobChIP_FOXA1_robot
chr3	50248216	50248217	MACS_peak_10696	5.00	lobChIP_FOXA1_robot
chr3	50337313	50337314	MACS_peak_10697	3.00	lobChIP_FOXA1_robot
chr3	50347305	50347306	MACS_peak_10698	4.00	lobChIP_FOXA1_robot
chr3	50461124	50461125	MACS_peak_10699	5.00	lobChIP_FOXA1_robot
chr3	50483484	50483485	MACS_peak_10700	14.00	lobChIP_FOXA1_robot
chr3	50626618	50626619	MACS_peak_10701	4.00	lobChIP_FOXA1_robot
chr3	50649280	50649281	MACS_peak_10702	5.00	lobChIP_FOXA1_robot
chr3	51040173	51040174	MACS_peak_10703	8.00	lobChIP_FOXA1_robot
chr3	51132453	51132454	MACS_peak_10704	5.00	lobChIP_FOXA1_robot
chr3	51229390	51229391	MACS_peak_10705	5.00	lobChIP_FOXA1_robot
chr3	52115249	52115250	MACS_peak_10706	21.00	lobChIP_FOXA1_robot
chr3	52244914	52244915	MACS_peak_10707	24.00	lobChIP_FOXA1_robot
chr3	52272384	52272385	MACS_peak_10708	7.00	lobChIP_FOXA1_robot
chr3	52319561	52319562	MACS_peak_10709	6.00	lobChIP_FOXA1_robot
chr3	52373815	52373816	MACS_peak_10710	6.00	lobChIP_FOXA1_robot
chr3	52459354	52459355	MACS_peak_10711	7.00	lobChIP_FOXA1_robot
chr3	52879426	52879427	MACS_peak_10712	3.00	lobChIP_FOXA1_robot
chr3	52927868	52927869	MACS_peak_10713	9.00	lobChIP_FOXA1_robot
chr3	53512656	53512657	MACS_peak_10714	7.00	lobChIP_FOXA1_robot
chr3	53877034	53877035	MACS_peak_10715	13.00	lobChIP_FOXA1_robot
chr3	54356625	54356626	MACS_peak_10716	4.00	lobChIP_FOXA1_robot
chr3	54732345	54732346	MACS_peak_10717	5.00	lobChIP_FOXA1_robot
chr3	56792792	56792793	MACS_peak_10718	5.00	lobChIP_FOXA1_robot
chr3	56816407	56816408	MACS_peak_10719	10.00	lobChIP_FOXA1_robot
chr3	56817805	56817806	MACS_peak_10720	5.00	lobChIP_FOXA1_robot
chr3	56833471	56833472	MACS_peak_10721	3.00	lobChIP_FOXA1_robot
chr3	56853388	56853389	MACS_peak_10722	11.00	lobChIP_FOXA1_robot
chr3	56858273	56858274	MACS_peak_10723	19.00	lobChIP_FOXA1_robot
chr3	56865105	56865106	MACS_peak_10724	18.00	lobChIP_FOXA1_robot
chr3	57023322	57023323	MACS_peak_10725	4.00	lobChIP_FOXA1_robot
chr3	57023939	57023940	MACS_peak_10726	8.00	lobChIP_FOXA1_robot
chr3	57024758	57024759	MACS_peak_10727	15.00	lobChIP_FOXA1_robot
chr3	57026713	57026714	MACS_peak_10728	8.00	lobChIP_FOXA1_robot
chr3	57436788	57436789	MACS_peak_10729	7.00	lobChIP_FOXA1_robot
chr3	57581218	57581219	MACS_peak_10730	3.00	lobChIP_FOXA1_robot
chr3	57777232	57777233	MACS_peak_10731	6.00	lobChIP_FOXA1_robot
chr3	57784015	57784016	MACS_peak_10732	12.00	lobChIP_FOXA1_robot
chr3	57802625	57802626	MACS_peak_10733	4.00	lobChIP_FOXA1_robot
chr3	57812961	57812962	MACS_peak_10734	10.00	lobChIP_FOXA1_robot
chr3	57932798	57932799	MACS_peak_10735	8.00	lobChIP_FOXA1_robot
chr3	57978391	57978392	MACS_peak_10736	17.00	lobChIP_FOXA1_robot
chr3	58008018	58008019	MACS_peak_10737	3.00	lobChIP_FOXA1_robot
chr3	58025964	58025965	MACS_peak_10738	17.00	lobChIP_FOXA1_robot
chr3	58028011	58028012	MACS_peak_10739	6.00	lobChIP_FOXA1_robot
chr3	58045482	58045483	MACS_peak_10740	9.00	lobChIP_FOXA1_robot
chr3	58087795	58087796	MACS_peak_10741	9.00	lobChIP_FOXA1_robot
chr3	58130702	58130703	MACS_peak_10742	16.00	lobChIP_FOXA1_robot
chr3	58159930	58159931	MACS_peak_10743	7.00	lobChIP_FOXA1_robot
chr3	58167095	58167096	MACS_peak_10744	10.00	lobChIP_FOXA1_robot
chr3	58175061	58175062	MACS_peak_10745	4.00	lobChIP_FOXA1_robot
chr3	58177488	58177489	MACS_peak_10746	7.00	lobChIP_FOXA1_robot
chr3	58179231	58179232	MACS_peak_10747	14.00	lobChIP_FOXA1_robot
chr3	58220607	58220608	MACS_peak_10748	8.00	lobChIP_FOXA1_robot
chr3	58319796	58319797	MACS_peak_10749	4.00	lobChIP_FOXA1_robot
chr3	58438143	58438144	MACS_peak_10750	7.00	lobChIP_FOXA1_robot
chr3	58457255	58457256	MACS_peak_10751	46.00	lobChIP_FOXA1_robot
chr3	58521677	58521678	MACS_peak_10752	8.00	lobChIP_FOXA1_robot
chr3	58699049	58699050	MACS_peak_10753	4.00	lobChIP_FOXA1_robot
chr3	59157984	59157985	MACS_peak_10754	6.00	lobChIP_FOXA1_robot
chr3	59306169	59306170	MACS_peak_10755	6.00	lobChIP_FOXA1_robot
chr3	59325019	59325020	MACS_peak_10756	5.00	lobChIP_FOXA1_robot
chr3	59784332	59784333	MACS_peak_10757	8.00	lobChIP_FOXA1_robot
chr3	60827194	60827195	MACS_peak_10758	18.00	lobChIP_FOXA1_robot
chr3	60953085	60953086	MACS_peak_10759	14.00	lobChIP_FOXA1_robot
chr3	61016324	61016325	MACS_peak_10760	4.00	lobChIP_FOXA1_robot
chr3	61620587	61620588	MACS_peak_10761	21.00	lobChIP_FOXA1_robot
chr3	61630915	61630916	MACS_peak_10762	11.00	lobChIP_FOXA1_robot
chr3	61711928	61711929	MACS_peak_10763	13.00	lobChIP_FOXA1_robot
chr3	61715084	61715085	MACS_peak_10764	6.00	lobChIP_FOXA1_robot
chr3	62043223	62043224	MACS_peak_10765	8.00	lobChIP_FOXA1_robot
chr3	64234231	64234232	MACS_peak_10766	3.00	lobChIP_FOXA1_robot
chr3	64487390	64487391	MACS_peak_10767	17.00	lobChIP_FOXA1_robot
chr3	65433199	65433200	MACS_peak_10768	4.00	lobChIP_FOXA1_robot
chr3	65646259	65646260	MACS_peak_10769	5.00	lobChIP_FOXA1_robot
chr3	65911133	65911134	MACS_peak_10770	7.00	lobChIP_FOXA1_robot
chr3	65925025	65925026	MACS_peak_10771	4.00	lobChIP_FOXA1_robot
chr3	65942024	65942025	MACS_peak_10772	7.00	lobChIP_FOXA1_robot
chr3	66059625	66059626	MACS_peak_10773	4.00	lobChIP_FOXA1_robot
chr3	66513652	66513653	MACS_peak_10774	10.00	lobChIP_FOXA1_robot
chr3	66527657	66527658	MACS_peak_10775	8.00	lobChIP_FOXA1_robot
chr3	66532298	66532299	MACS_peak_10776	11.00	lobChIP_FOXA1_robot
chr3	66644622	66644623	MACS_peak_10777	5.00	lobChIP_FOXA1_robot
chr3	67167928	67167929	MACS_peak_10778	3.00	lobChIP_FOXA1_robot
chr3	67343144	67343145	MACS_peak_10779	5.00	lobChIP_FOXA1_robot
chr3	67346545	67346546	MACS_peak_10780	8.00	lobChIP_FOXA1_robot
chr3	67698018	67698019	MACS_peak_10781	8.00	lobChIP_FOXA1_robot
chr3	67700259	67700260	MACS_peak_10782	3.00	lobChIP_FOXA1_robot
chr3	67926946	67926947	MACS_peak_10783	5.00	lobChIP_FOXA1_robot
chr3	68995576	68995577	MACS_peak_10784	9.00	lobChIP_FOXA1_robot
chr3	69228691	69228692	MACS_peak_10785	4.00	lobChIP_FOXA1_robot
chr3	69229163	69229164	MACS_peak_10786	5.00	lobChIP_FOXA1_robot
chr3	69261850	69261851	MACS_peak_10787	5.00	lobChIP_FOXA1_robot
chr3	71076294	71076295	MACS_peak_10788	7.00	lobChIP_FOXA1_robot
chr3	71098344	71098345	MACS_peak_10789	5.00	lobChIP_FOXA1_robot
chr3	71099522	71099523	MACS_peak_10790	3.00	lobChIP_FOXA1_robot
chr3	71100001	71100002	MACS_peak_10791	6.00	lobChIP_FOXA1_robot
chr3	71104905	71104906	MACS_peak_10792	4.00	lobChIP_FOXA1_robot
chr3	71109856	71109857	MACS_peak_10793	40.00	lobChIP_FOXA1_robot
chr3	71136667	71136668	MACS_peak_10794	7.00	lobChIP_FOXA1_robot
chr3	71137693	71137694	MACS_peak_10795	9.00	lobChIP_FOXA1_robot
chr3	71178336	71178337	MACS_peak_10796	3.00	lobChIP_FOXA1_robot
chr3	71180808	71180809	MACS_peak_10797	5.00	lobChIP_FOXA1_robot
chr3	71182766	71182767	MACS_peak_10798	4.00	lobChIP_FOXA1_robot
chr3	71184809	71184810	MACS_peak_10799	5.00	lobChIP_FOXA1_robot
chr3	71190688	71190689	MACS_peak_10800	4.00	lobChIP_FOXA1_robot
chr3	71202243	71202244	MACS_peak_10801	8.00	lobChIP_FOXA1_robot
chr3	71203164	71203165	MACS_peak_10802	5.00	lobChIP_FOXA1_robot
chr3	71244830	71244831	MACS_peak_10803	4.00	lobChIP_FOXA1_robot
chr3	71257649	71257650	MACS_peak_10804	50.00	lobChIP_FOXA1_robot
chr3	71293808	71293809	MACS_peak_10805	4.00	lobChIP_FOXA1_robot
chr3	71294595	71294596	MACS_peak_10806	8.00	lobChIP_FOXA1_robot
chr3	71303956	71303957	MACS_peak_10807	8.00	lobChIP_FOXA1_robot
chr3	71307336	71307337	MACS_peak_10808	3.00	lobChIP_FOXA1_robot
chr3	71307869	71307870	MACS_peak_10809	5.00	lobChIP_FOXA1_robot
chr3	71429282	71429283	MACS_peak_10810	4.00	lobChIP_FOXA1_robot
chr3	71594674	71594675	MACS_peak_10811	6.00	lobChIP_FOXA1_robot
chr3	71597279	71597280	MACS_peak_10812	7.00	lobChIP_FOXA1_robot
chr3	71629290	71629291	MACS_peak_10813	16.00	lobChIP_FOXA1_robot
chr3	71744664	71744665	MACS_peak_10814	5.00	lobChIP_FOXA1_robot
chr3	72201943	72201944	MACS_peak_10815	4.00	lobChIP_FOXA1_robot
chr3	72621162	72621163	MACS_peak_10816	9.00	lobChIP_FOXA1_robot
chr3	72629009	72629010	MACS_peak_10817	6.00	lobChIP_FOXA1_robot
chr3	72639998	72639999	MACS_peak_10818	5.00	lobChIP_FOXA1_robot
chr3	73002837	73002838	MACS_peak_10819	7.00	lobChIP_FOXA1_robot
chr3	73013892	73013893	MACS_peak_10820	3.00	lobChIP_FOXA1_robot
chr3	73016904	73016905	MACS_peak_10821	8.00	lobChIP_FOXA1_robot
chr3	73017647	73017648	MACS_peak_10822	6.00	lobChIP_FOXA1_robot
chr3	73101212	73101213	MACS_peak_10823	4.00	lobChIP_FOXA1_robot
chr3	73159955	73159956	MACS_peak_10824	4.00	lobChIP_FOXA1_robot
chr3	73198056	73198057	MACS_peak_10825	5.00	lobChIP_FOXA1_robot
chr3	73620638	73620639	MACS_peak_10826	15.00	lobChIP_FOXA1_robot
chr3	76399095	76399096	MACS_peak_10827	3.00	lobChIP_FOXA1_robot
chr3	76735204	76735205	MACS_peak_10828	5.00	lobChIP_FOXA1_robot
chr3	77022170	77022171	MACS_peak_10829	8.00	lobChIP_FOXA1_robot
chr3	78695074	78695075	MACS_peak_10830	5.00	lobChIP_FOXA1_robot
chr3	78708738	78708739	MACS_peak_10831	7.00	lobChIP_FOXA1_robot
chr3	78771512	78771513	MACS_peak_10832	4.00	lobChIP_FOXA1_robot
chr3	78882188	78882189	MACS_peak_10833	5.00	lobChIP_FOXA1_robot
chr3	78893686	78893687	MACS_peak_10834	16.00	lobChIP_FOXA1_robot
chr3	78898285	78898286	MACS_peak_10835	18.00	lobChIP_FOXA1_robot
chr3	78898902	78898903	MACS_peak_10836	14.00	lobChIP_FOXA1_robot
chr3	78919766	78919767	MACS_peak_10837	5.00	lobChIP_FOXA1_robot
chr3	78928211	78928212	MACS_peak_10838	5.00	lobChIP_FOXA1_robot
chr3	78955584	78955585	MACS_peak_10839	12.00	lobChIP_FOXA1_robot
chr3	78968682	78968683	MACS_peak_10840	14.00	lobChIP_FOXA1_robot
chr3	78974033	78974034	MACS_peak_10841	3.00	lobChIP_FOXA1_robot
chr3	79021459	79021460	MACS_peak_10842	32.00	lobChIP_FOXA1_robot
chr3	79037764	79037765	MACS_peak_10843	3.00	lobChIP_FOXA1_robot
chr3	79041846	79041847	MACS_peak_10844	3.00	lobChIP_FOXA1_robot
chr3	79042769	79042770	MACS_peak_10845	8.00	lobChIP_FOXA1_robot
chr3	79043887	79043888	MACS_peak_10846	5.00	lobChIP_FOXA1_robot
chr3	79055423	79055424	MACS_peak_10847	14.00	lobChIP_FOXA1_robot
chr3	80644795	80644796	MACS_peak_10848	3.00	lobChIP_FOXA1_robot
chr3	80667682	80667683	MACS_peak_10849	19.00	lobChIP_FOXA1_robot
chr3	80782898	80782899	MACS_peak_10850	5.00	lobChIP_FOXA1_robot
chr3	81298932	81298933	MACS_peak_10851	13.00	lobChIP_FOXA1_robot
chr3	81329567	81329568	MACS_peak_10852	7.00	lobChIP_FOXA1_robot
chr3	81349732	81349733	MACS_peak_10853	70.00	lobChIP_FOXA1_robot
chr3	81766334	81766335	MACS_peak_10854	5.00	lobChIP_FOXA1_robot
chr3	81773101	81773102	MACS_peak_10855	4.00	lobChIP_FOXA1_robot
chr3	82310363	82310364	MACS_peak_10856	5.00	lobChIP_FOXA1_robot
chr3	82347171	82347172	MACS_peak_10857	5.00	lobChIP_FOXA1_robot
chr3	82396910	82396911	MACS_peak_10858	4.00	lobChIP_FOXA1_robot
chr3	82510710	82510711	MACS_peak_10859	11.00	lobChIP_FOXA1_robot
chr3	82514133	82514134	MACS_peak_10860	5.00	lobChIP_FOXA1_robot
chr3	82977286	82977287	MACS_peak_10861	7.00	lobChIP_FOXA1_robot
chr3	83930579	83930580	MACS_peak_10862	4.00	lobChIP_FOXA1_robot
chr3	84459326	84459327	MACS_peak_10863	3.00	lobChIP_FOXA1_robot
chr3	85056554	85056555	MACS_peak_10864	18.00	lobChIP_FOXA1_robot
chr3	85775705	85775706	MACS_peak_10865	7.00	lobChIP_FOXA1_robot
chr3	86408052	86408053	MACS_peak_10866	7.00	lobChIP_FOXA1_robot
chr3	87030631	87030632	MACS_peak_10867	3.00	lobChIP_FOXA1_robot
chr3	87382709	87382710	MACS_peak_10868	6.00	lobChIP_FOXA1_robot
chr3	94243349	94243350	MACS_peak_10870	5.00	lobChIP_FOXA1_robot
chr3	95060374	95060375	MACS_peak_10871	5.00	lobChIP_FOXA1_robot
chr3	95503309	95503310	MACS_peak_10872	4.00	lobChIP_FOXA1_robot
chr3	95673099	95673100	MACS_peak_10873	3.00	lobChIP_FOXA1_robot
chr3	96930783	96930784	MACS_peak_10874	3.00	lobChIP_FOXA1_robot
chr3	97713160	97713161	MACS_peak_10875	4.00	lobChIP_FOXA1_robot
chr3	97729393	97729394	MACS_peak_10876	6.00	lobChIP_FOXA1_robot
chr3	97730731	97730732	MACS_peak_10877	3.00	lobChIP_FOXA1_robot
chr3	98247912	98247913	MACS_peak_10878	3.00	lobChIP_FOXA1_robot
chr3	98294062	98294063	MACS_peak_10879	9.00	lobChIP_FOXA1_robot
chr3	98312712	98312713	MACS_peak_10880	5.00	lobChIP_FOXA1_robot
chr3	98407898	98407899	MACS_peak_10881	8.00	lobChIP_FOXA1_robot
chr3	98496360	98496361	MACS_peak_10882	9.00	lobChIP_FOXA1_robot
chr3	98497746	98497747	MACS_peak_10883	4.00	lobChIP_FOXA1_robot
chr3	98503220	98503221	MACS_peak_10884	4.00	lobChIP_FOXA1_robot
chr3	98503935	98503936	MACS_peak_10885	4.00	lobChIP_FOXA1_robot
chr3	98530020	98530021	MACS_peak_10886	10.00	lobChIP_FOXA1_robot
chr3	98532068	98532069	MACS_peak_10887	4.00	lobChIP_FOXA1_robot
chr3	98641870	98641871	MACS_peak_10888	6.00	lobChIP_FOXA1_robot
chr3	98696454	98696455	MACS_peak_10889	7.00	lobChIP_FOXA1_robot
chr3	98697146	98697147	MACS_peak_10890	7.00	lobChIP_FOXA1_robot
chr3	99544483	99544484	MACS_peak_10891	3.00	lobChIP_FOXA1_robot
chr3	99565492	99565493	MACS_peak_10892	4.00	lobChIP_FOXA1_robot
chr3	99566750	99566751	MACS_peak_10893	4.00	lobChIP_FOXA1_robot
chr3	99576399	99576400	MACS_peak_10894	9.00	lobChIP_FOXA1_robot
chr3	99614699	99614700	MACS_peak_10895	7.00	lobChIP_FOXA1_robot
chr3	99620523	99620524	MACS_peak_10896	13.00	lobChIP_FOXA1_robot
chr3	99628746	99628747	MACS_peak_10897	5.00	lobChIP_FOXA1_robot
chr3	99639928	99639929	MACS_peak_10898	9.00	lobChIP_FOXA1_robot
chr3	99643064	99643065	MACS_peak_10899	6.00	lobChIP_FOXA1_robot
chr3	99650098	99650099	MACS_peak_10900	30.00	lobChIP_FOXA1_robot
chr3	99673930	99673931	MACS_peak_10901	4.00	lobChIP_FOXA1_robot
chr3	99722063	99722064	MACS_peak_10902	4.00	lobChIP_FOXA1_robot
chr3	99742186	99742187	MACS_peak_10903	7.00	lobChIP_FOXA1_robot
chr3	99760264	99760265	MACS_peak_10904	5.00	lobChIP_FOXA1_robot
chr3	99782808	99782809	MACS_peak_10905	5.00	lobChIP_FOXA1_robot
chr3	99783292	99783293	MACS_peak_10906	3.00	lobChIP_FOXA1_robot
chr3	99795141	99795142	MACS_peak_10907	27.00	lobChIP_FOXA1_robot
chr3	99807872	99807873	MACS_peak_10908	3.00	lobChIP_FOXA1_robot
chr3	99833701	99833702	MACS_peak_10909	6.00	lobChIP_FOXA1_robot
chr3	100093690	100093691	MACS_peak_10910	5.00	lobChIP_FOXA1_robot
chr3	100105614	100105615	MACS_peak_10911	16.00	lobChIP_FOXA1_robot
chr3	100321527	100321528	MACS_peak_10912	11.00	lobChIP_FOXA1_robot
chr3	101267737	101267738	MACS_peak_10913	7.00	lobChIP_FOXA1_robot
chr3	101292709	101292710	MACS_peak_10914	4.00	lobChIP_FOXA1_robot
chr3	101533993	101533994	MACS_peak_10915	4.00	lobChIP_FOXA1_robot
chr3	101567372	101567373	MACS_peak_10916	4.00	lobChIP_FOXA1_robot
chr3	101573709	101573710	MACS_peak_10917	15.00	lobChIP_FOXA1_robot
chr3	102604403	102604404	MACS_peak_10918	5.00	lobChIP_FOXA1_robot
chr3	102805964	102805965	MACS_peak_10919	4.00	lobChIP_FOXA1_robot
chr3	103566678	103566679	MACS_peak_10920	3.00	lobChIP_FOXA1_robot
chr3	103695924	103695925	MACS_peak_10921	11.00	lobChIP_FOXA1_robot
chr3	104325876	104325877	MACS_peak_10922	5.00	lobChIP_FOXA1_robot
chr3	104331589	104331590	MACS_peak_10923	4.00	lobChIP_FOXA1_robot
chr3	105106194	105106195	MACS_peak_10924	42.00	lobChIP_FOXA1_robot
chr3	105148413	105148414	MACS_peak_10925	5.00	lobChIP_FOXA1_robot
chr3	105159179	105159180	MACS_peak_10926	5.00	lobChIP_FOXA1_robot
chr3	105222212	105222213	MACS_peak_10927	28.00	lobChIP_FOXA1_robot
chr3	105528634	105528635	MACS_peak_10928	4.00	lobChIP_FOXA1_robot
chr3	105564500	105564501	MACS_peak_10929	5.00	lobChIP_FOXA1_robot
chr3	105681514	105681515	MACS_peak_10930	10.00	lobChIP_FOXA1_robot
chr3	106230064	106230065	MACS_peak_10931	5.00	lobChIP_FOXA1_robot
chr3	107403615	107403616	MACS_peak_10932	11.00	lobChIP_FOXA1_robot
chr3	107408345	107408346	MACS_peak_10933	6.00	lobChIP_FOXA1_robot
chr3	107472985	107472986	MACS_peak_10934	5.00	lobChIP_FOXA1_robot
chr3	107515832	107515833	MACS_peak_10935	6.00	lobChIP_FOXA1_robot
chr3	107554008	107554009	MACS_peak_10936	10.00	lobChIP_FOXA1_robot
chr3	108055014	108055015	MACS_peak_10937	4.00	lobChIP_FOXA1_robot
chr3	110116209	110116210	MACS_peak_10938	6.00	lobChIP_FOXA1_robot
chr3	110462187	110462188	MACS_peak_10939	16.00	lobChIP_FOXA1_robot
chr3	110492706	110492707	MACS_peak_10940	15.00	lobChIP_FOXA1_robot
chr3	110530016	110530017	MACS_peak_10941	5.00	lobChIP_FOXA1_robot
chr3	110647323	110647324	MACS_peak_10942	3.00	lobChIP_FOXA1_robot
chr3	110657819	110657820	MACS_peak_10943	3.00	lobChIP_FOXA1_robot
chr3	110659100	110659101	MACS_peak_10944	6.00	lobChIP_FOXA1_robot
chr3	110665869	110665870	MACS_peak_10945	16.00	lobChIP_FOXA1_robot
chr3	110714777	110714778	MACS_peak_10946	4.00	lobChIP_FOXA1_robot
chr3	110766806	110766807	MACS_peak_10947	6.00	lobChIP_FOXA1_robot
chr3	110787749	110787750	MACS_peak_10948	3.00	lobChIP_FOXA1_robot
chr3	110826051	110826052	MACS_peak_10949	8.00	lobChIP_FOXA1_robot
chr3	111445973	111445974	MACS_peak_10950	7.00	lobChIP_FOXA1_robot
chr3	111452111	111452112	MACS_peak_10951	14.00	lobChIP_FOXA1_robot
chr3	111454535	111454536	MACS_peak_10952	5.00	lobChIP_FOXA1_robot
chr3	112192142	112192143	MACS_peak_10953	4.00	lobChIP_FOXA1_robot
chr3	112822667	112822668	MACS_peak_10954	9.00	lobChIP_FOXA1_robot
chr3	113382339	113382340	MACS_peak_10955	22.00	lobChIP_FOXA1_robot
chr3	113383459	113383460	MACS_peak_10956	9.00	lobChIP_FOXA1_robot
chr3	113405030	113405031	MACS_peak_10957	5.00	lobChIP_FOXA1_robot
chr3	113549694	113549695	MACS_peak_10958	4.00	lobChIP_FOXA1_robot
chr3	114081844	114081845	MACS_peak_10959	6.00	lobChIP_FOXA1_robot
chr3	114425598	114425599	MACS_peak_10960	12.00	lobChIP_FOXA1_robot
chr3	114464421	114464422	MACS_peak_10961	10.00	lobChIP_FOXA1_robot
chr3	114818484	114818485	MACS_peak_10962	4.00	lobChIP_FOXA1_robot
chr3	115425310	115425311	MACS_peak_10963	4.00	lobChIP_FOXA1_robot
chr3	115533094	115533095	MACS_peak_10964	8.00	lobChIP_FOXA1_robot
chr3	115541639	115541640	MACS_peak_10965	9.00	lobChIP_FOXA1_robot
chr3	115562394	115562395	MACS_peak_10966	13.00	lobChIP_FOXA1_robot
chr3	118250977	118250978	MACS_peak_10967	4.00	lobChIP_FOXA1_robot
chr3	118432475	118432476	MACS_peak_10968	14.00	lobChIP_FOXA1_robot
chr3	119514796	119514797	MACS_peak_10969	7.00	lobChIP_FOXA1_robot
chr3	119900024	119900025	MACS_peak_10970	3.00	lobChIP_FOXA1_robot
chr3	120020044	120020045	MACS_peak_10971	5.00	lobChIP_FOXA1_robot
chr3	120069372	120069373	MACS_peak_10972	3.00	lobChIP_FOXA1_robot
chr3	120401158	120401159	MACS_peak_10973	7.00	lobChIP_FOXA1_robot
chr3	120408468	120408469	MACS_peak_10974	5.00	lobChIP_FOXA1_robot
chr3	120515018	120515019	MACS_peak_10975	9.00	lobChIP_FOXA1_robot
chr3	120757953	120757954	MACS_peak_10976	4.00	lobChIP_FOXA1_robot
chr3	121449138	121449139	MACS_peak_10977	4.00	lobChIP_FOXA1_robot
chr3	122084577	122084578	MACS_peak_10978	30.00	lobChIP_FOXA1_robot
chr3	122370680	122370681	MACS_peak_10979	17.00	lobChIP_FOXA1_robot
chr3	122410206	122410207	MACS_peak_10980	4.00	lobChIP_FOXA1_robot
chr3	122411748	122411749	MACS_peak_10981	7.00	lobChIP_FOXA1_robot
chr3	123170317	123170318	MACS_peak_10982	5.00	lobChIP_FOXA1_robot
chr3	123247157	123247158	MACS_peak_10983	4.00	lobChIP_FOXA1_robot
chr3	123324182	123324183	MACS_peak_10984	22.00	lobChIP_FOXA1_robot
chr3	123325575	123325576	MACS_peak_10985	8.00	lobChIP_FOXA1_robot
chr3	123451406	123451407	MACS_peak_10986	7.00	lobChIP_FOXA1_robot
chr3	123538064	123538065	MACS_peak_10987	6.00	lobChIP_FOXA1_robot
chr3	124232248	124232249	MACS_peak_10988	8.00	lobChIP_FOXA1_robot
chr3	124240122	124240123	MACS_peak_10989	7.00	lobChIP_FOXA1_robot
chr3	124271345	124271346	MACS_peak_10990	7.00	lobChIP_FOXA1_robot
chr3	124336301	124336302	MACS_peak_10991	9.00	lobChIP_FOXA1_robot
chr3	124354230	124354231	MACS_peak_10992	27.00	lobChIP_FOXA1_robot
chr3	124418085	124418086	MACS_peak_10993	15.00	lobChIP_FOXA1_robot
chr3	124418638	124418639	MACS_peak_10994	4.00	lobChIP_FOXA1_robot
chr3	124480943	124480944	MACS_peak_10995	4.00	lobChIP_FOXA1_robot
chr3	124533646	124533647	MACS_peak_10996	6.00	lobChIP_FOXA1_robot
chr3	124553088	124553089	MACS_peak_10997	13.00	lobChIP_FOXA1_robot
chr3	124553710	124553711	MACS_peak_10998	8.00	lobChIP_FOXA1_robot
chr3	124597758	124597759	MACS_peak_10999	12.00	lobChIP_FOXA1_robot
chr3	124605510	124605511	MACS_peak_11000	4.00	lobChIP_FOXA1_robot
chr3	124721030	124721031	MACS_peak_11001	6.00	lobChIP_FOXA1_robot
chr3	124722471	124722472	MACS_peak_11002	10.00	lobChIP_FOXA1_robot
chr3	124781987	124781988	MACS_peak_11003	17.00	lobChIP_FOXA1_robot
chr3	125785684	125785685	MACS_peak_11004	14.00	lobChIP_FOXA1_robot
chr3	126002906	126002907	MACS_peak_11005	4.00	lobChIP_FOXA1_robot
chr3	126045720	126045721	MACS_peak_11006	3.00	lobChIP_FOXA1_robot
chr3	126236111	126236112	MACS_peak_11007	4.00	lobChIP_FOXA1_robot
chr3	126569790	126569791	MACS_peak_11008	5.00	lobChIP_FOXA1_robot
chr3	126832499	126832500	MACS_peak_11009	23.00	lobChIP_FOXA1_robot
chr3	126860939	126860940	MACS_peak_11010	5.00	lobChIP_FOXA1_robot
chr3	127755324	127755325	MACS_peak_11011	6.00	lobChIP_FOXA1_robot
chr3	127887061	127887062	MACS_peak_11012	7.00	lobChIP_FOXA1_robot
chr3	127889964	127889965	MACS_peak_11013	4.00	lobChIP_FOXA1_robot
chr3	128073530	128073531	MACS_peak_11014	4.00	lobChIP_FOXA1_robot
chr3	128079679	128079680	MACS_peak_11015	6.00	lobChIP_FOXA1_robot
chr3	128104361	128104362	MACS_peak_11016	3.00	lobChIP_FOXA1_robot
chr3	128427001	128427002	MACS_peak_11017	7.00	lobChIP_FOXA1_robot
chr3	128773549	128773550	MACS_peak_11018	5.00	lobChIP_FOXA1_robot
chr3	128838677	128838678	MACS_peak_11019	7.00	lobChIP_FOXA1_robot
chr3	129107811	129107812	MACS_peak_11020	5.00	lobChIP_FOXA1_robot
chr3	129118712	129118713	MACS_peak_11021	5.00	lobChIP_FOXA1_robot
chr3	129132700	129132701	MACS_peak_11022	5.00	lobChIP_FOXA1_robot
chr3	129214015	129214016	MACS_peak_11023	6.00	lobChIP_FOXA1_robot
chr3	129312840	129312841	MACS_peak_11024	14.00	lobChIP_FOXA1_robot
chr3	129381941	129381942	MACS_peak_11025	5.00	lobChIP_FOXA1_robot
chr3	129406006	129406007	MACS_peak_11026	5.00	lobChIP_FOXA1_robot
chr3	129451430	129451431	MACS_peak_11027	4.00	lobChIP_FOXA1_robot
chr3	129690434	129690435	MACS_peak_11028	7.00	lobChIP_FOXA1_robot
chr3	130452305	130452306	MACS_peak_11029	5.00	lobChIP_FOXA1_robot
chr3	130682979	130682980	MACS_peak_11030	5.00	lobChIP_FOXA1_robot
chr3	130752534	130752535	MACS_peak_11031	7.00	lobChIP_FOXA1_robot
chr3	131113465	131113466	MACS_peak_11032	8.00	lobChIP_FOXA1_robot
chr3	132361286	132361287	MACS_peak_11033	5.00	lobChIP_FOXA1_robot
chr3	132381279	132381280	MACS_peak_11034	5.00	lobChIP_FOXA1_robot
chr3	133163119	133163120	MACS_peak_11035	6.00	lobChIP_FOXA1_robot
chr3	133171911	133171912	MACS_peak_11036	12.00	lobChIP_FOXA1_robot
chr3	133188247	133188248	MACS_peak_11037	6.00	lobChIP_FOXA1_robot
chr3	133218190	133218191	MACS_peak_11038	9.00	lobChIP_FOXA1_robot
chr3	133255370	133255371	MACS_peak_11039	3.00	lobChIP_FOXA1_robot
chr3	133283846	133283847	MACS_peak_11040	4.00	lobChIP_FOXA1_robot
chr3	133389419	133389420	MACS_peak_11041	18.00	lobChIP_FOXA1_robot
chr3	133394748	133394749	MACS_peak_11042	3.00	lobChIP_FOXA1_robot
chr3	133430153	133430154	MACS_peak_11043	7.00	lobChIP_FOXA1_robot
chr3	133450034	133450035	MACS_peak_11044	6.00	lobChIP_FOXA1_robot
chr3	133452257	133452258	MACS_peak_11045	5.00	lobChIP_FOXA1_robot
chr3	133461607	133461608	MACS_peak_11046	46.00	lobChIP_FOXA1_robot
chr3	133465171	133465172	MACS_peak_11047	5.00	lobChIP_FOXA1_robot
chr3	133956896	133956897	MACS_peak_11048	5.00	lobChIP_FOXA1_robot
chr3	134138662	134138663	MACS_peak_11049	6.00	lobChIP_FOXA1_robot
chr3	134203977	134203978	MACS_peak_11050	4.00	lobChIP_FOXA1_robot
chr3	135861613	135861614	MACS_peak_11051	5.00	lobChIP_FOXA1_robot
chr3	135974794	135974795	MACS_peak_11052	4.00	lobChIP_FOXA1_robot
chr3	136011488	136011489	MACS_peak_11053	4.00	lobChIP_FOXA1_robot
chr3	136305690	136305691	MACS_peak_11054	3.00	lobChIP_FOXA1_robot
chr3	136351793	136351794	MACS_peak_11055	4.00	lobChIP_FOXA1_robot
chr3	136422516	136422517	MACS_peak_11056	7.00	lobChIP_FOXA1_robot
chr3	136692394	136692395	MACS_peak_11057	5.00	lobChIP_FOXA1_robot
chr3	137228741	137228742	MACS_peak_11058	4.00	lobChIP_FOXA1_robot
chr3	138047867	138047868	MACS_peak_11059	5.00	lobChIP_FOXA1_robot
chr3	138118858	138118859	MACS_peak_11060	14.00	lobChIP_FOXA1_robot
chr3	138392598	138392599	MACS_peak_11061	3.00	lobChIP_FOXA1_robot
chr3	138405847	138405848	MACS_peak_11062	27.00	lobChIP_FOXA1_robot
chr3	138407795	138407796	MACS_peak_11063	6.00	lobChIP_FOXA1_robot
chr3	138493486	138493487	MACS_peak_11064	3.00	lobChIP_FOXA1_robot
chr3	138542710	138542711	MACS_peak_11065	10.00	lobChIP_FOXA1_robot
chr3	138581977	138581978	MACS_peak_11066	7.00	lobChIP_FOXA1_robot
chr3	138634121	138634122	MACS_peak_11067	4.00	lobChIP_FOXA1_robot
chr3	138683297	138683298	MACS_peak_11068	5.00	lobChIP_FOXA1_robot
chr3	139373535	139373536	MACS_peak_11069	3.00	lobChIP_FOXA1_robot
chr3	140418792	140418793	MACS_peak_11070	22.00	lobChIP_FOXA1_robot
chr3	140430324	140430325	MACS_peak_11071	8.00	lobChIP_FOXA1_robot
chr3	140666242	140666243	MACS_peak_11072	6.00	lobChIP_FOXA1_robot
chr3	140676174	140676175	MACS_peak_11073	77.00	lobChIP_FOXA1_robot
chr3	140866756	140866757	MACS_peak_11074	5.00	lobChIP_FOXA1_robot
chr3	141080034	141080035	MACS_peak_11075	15.00	lobChIP_FOXA1_robot
chr3	141087136	141087137	MACS_peak_11076	4.00	lobChIP_FOXA1_robot
chr3	141119280	141119281	MACS_peak_11077	6.00	lobChIP_FOXA1_robot
chr3	141128368	141128369	MACS_peak_11078	10.00	lobChIP_FOXA1_robot
chr3	141131975	141131976	MACS_peak_11079	6.00	lobChIP_FOXA1_robot
chr3	141150499	141150500	MACS_peak_11080	11.00	lobChIP_FOXA1_robot
chr3	141181476	141181477	MACS_peak_11081	6.00	lobChIP_FOXA1_robot
chr3	141462403	141462404	MACS_peak_11082	5.00	lobChIP_FOXA1_robot
chr3	141490248	141490249	MACS_peak_11083	5.00	lobChIP_FOXA1_robot
chr3	142034883	142034884	MACS_peak_11084	19.00	lobChIP_FOXA1_robot
chr3	142082893	142082894	MACS_peak_11085	6.00	lobChIP_FOXA1_robot
chr3	142166292	142166293	MACS_peak_11086	8.00	lobChIP_FOXA1_robot
chr3	142565773	142565774	MACS_peak_11087	9.00	lobChIP_FOXA1_robot
chr3	142648234	142648235	MACS_peak_11088	5.00	lobChIP_FOXA1_robot
chr3	142659080	142659081	MACS_peak_11089	27.00	lobChIP_FOXA1_robot
chr3	142660575	142660576	MACS_peak_11090	9.00	lobChIP_FOXA1_robot
chr3	142759957	142759958	MACS_peak_11091	4.00	lobChIP_FOXA1_robot
chr3	143692691	143692692	MACS_peak_11092	7.00	lobChIP_FOXA1_robot
chr3	143881026	143881027	MACS_peak_11093	22.00	lobChIP_FOXA1_robot
chr3	145367170	145367171	MACS_peak_11094	4.00	lobChIP_FOXA1_robot
chr3	145556141	145556142	MACS_peak_11095	5.00	lobChIP_FOXA1_robot
chr3	145783489	145783490	MACS_peak_11096	4.00	lobChIP_FOXA1_robot
chr3	145833619	145833620	MACS_peak_11097	5.00	lobChIP_FOXA1_robot
chr3	145880518	145880519	MACS_peak_11098	4.00	lobChIP_FOXA1_robot
chr3	145940349	145940350	MACS_peak_11099	7.00	lobChIP_FOXA1_robot
chr3	146299001	146299002	MACS_peak_11100	12.00	lobChIP_FOXA1_robot
chr3	146344760	146344761	MACS_peak_11101	7.00	lobChIP_FOXA1_robot
chr3	146355825	146355826	MACS_peak_11102	25.00	lobChIP_FOXA1_robot
chr3	146373250	146373251	MACS_peak_11103	5.00	lobChIP_FOXA1_robot
chr3	146902724	146902725	MACS_peak_11104	6.00	lobChIP_FOXA1_robot
chr3	147086725	147086726	MACS_peak_11105	5.00	lobChIP_FOXA1_robot
chr3	148252386	148252387	MACS_peak_11106	5.00	lobChIP_FOXA1_robot
chr3	148318857	148318858	MACS_peak_11107	10.00	lobChIP_FOXA1_robot
chr3	148320144	148320145	MACS_peak_11108	27.00	lobChIP_FOXA1_robot
chr3	148427615	148427616	MACS_peak_11109	13.00	lobChIP_FOXA1_robot
chr3	148455224	148455225	MACS_peak_11110	4.00	lobChIP_FOXA1_robot
chr3	148728840	148728841	MACS_peak_11111	5.00	lobChIP_FOXA1_robot
chr3	148736101	148736102	MACS_peak_11112	10.00	lobChIP_FOXA1_robot
chr3	148922582	148922583	MACS_peak_11113	6.00	lobChIP_FOXA1_robot
chr3	148932688	148932689	MACS_peak_11114	13.00	lobChIP_FOXA1_robot
chr3	148933438	148933439	MACS_peak_11115	10.00	lobChIP_FOXA1_robot
chr3	148939804	148939805	MACS_peak_11116	7.00	lobChIP_FOXA1_robot
chr3	148941868	148941869	MACS_peak_11117	6.00	lobChIP_FOXA1_robot
chr3	148954009	148954010	MACS_peak_11118	11.00	lobChIP_FOXA1_robot
chr3	148965357	148965358	MACS_peak_11119	8.00	lobChIP_FOXA1_robot
chr3	148995502	148995503	MACS_peak_11120	4.00	lobChIP_FOXA1_robot
chr3	149018001	149018002	MACS_peak_11121	4.00	lobChIP_FOXA1_robot
chr3	149074246	149074247	MACS_peak_11122	3.00	lobChIP_FOXA1_robot
chr3	149087226	149087227	MACS_peak_11123	6.00	lobChIP_FOXA1_robot
chr3	149089255	149089256	MACS_peak_11124	12.00	lobChIP_FOXA1_robot
chr3	149091701	149091702	MACS_peak_11125	5.00	lobChIP_FOXA1_robot
chr3	149095643	149095644	MACS_peak_11126	6.00	lobChIP_FOXA1_robot
chr3	149104892	149104893	MACS_peak_11127	25.00	lobChIP_FOXA1_robot
chr3	149116318	149116319	MACS_peak_11128	6.00	lobChIP_FOXA1_robot
chr3	149118543	149118544	MACS_peak_11129	7.00	lobChIP_FOXA1_robot
chr3	149119536	149119537	MACS_peak_11130	81.00	lobChIP_FOXA1_robot
chr3	149167979	149167980	MACS_peak_11131	4.00	lobChIP_FOXA1_robot
chr3	149265815	149265816	MACS_peak_11132	5.00	lobChIP_FOXA1_robot
chr3	149317268	149317269	MACS_peak_11133	5.00	lobChIP_FOXA1_robot
chr3	149325612	149325613	MACS_peak_11134	15.00	lobChIP_FOXA1_robot
chr3	149347015	149347016	MACS_peak_11135	6.00	lobChIP_FOXA1_robot
chr3	149358986	149358987	MACS_peak_11136	4.00	lobChIP_FOXA1_robot
chr3	149359707	149359708	MACS_peak_11137	10.00	lobChIP_FOXA1_robot
chr3	149563599	149563600	MACS_peak_11138	13.00	lobChIP_FOXA1_robot
chr3	150090642	150090643	MACS_peak_11139	6.00	lobChIP_FOXA1_robot
chr3	150451573	150451574	MACS_peak_11140	4.00	lobChIP_FOXA1_robot
chr3	150455149	150455150	MACS_peak_11141	18.00	lobChIP_FOXA1_robot
chr3	150455972	150455973	MACS_peak_11142	4.00	lobChIP_FOXA1_robot
chr3	150916344	150916345	MACS_peak_11143	5.00	lobChIP_FOXA1_robot
chr3	151001989	151001990	MACS_peak_11144	7.00	lobChIP_FOXA1_robot
chr3	151347990	151347991	MACS_peak_11145	3.00	lobChIP_FOXA1_robot
chr3	151488144	151488145	MACS_peak_11146	14.00	lobChIP_FOXA1_robot
chr3	151534250	151534251	MACS_peak_11147	11.00	lobChIP_FOXA1_robot
chr3	151689674	151689675	MACS_peak_11148	6.00	lobChIP_FOXA1_robot
chr3	152025622	152025623	MACS_peak_11149	3.00	lobChIP_FOXA1_robot
chr3	152046505	152046506	MACS_peak_11150	4.00	lobChIP_FOXA1_robot
chr3	152103704	152103705	MACS_peak_11151	4.00	lobChIP_FOXA1_robot
chr3	152110230	152110231	MACS_peak_11152	7.00	lobChIP_FOXA1_robot
chr3	152450572	152450573	MACS_peak_11153	3.00	lobChIP_FOXA1_robot
chr3	152856793	152856794	MACS_peak_11154	6.00	lobChIP_FOXA1_robot
chr3	152858724	152858725	MACS_peak_11155	15.00	lobChIP_FOXA1_robot
chr3	152975647	152975648	MACS_peak_11156	9.00	lobChIP_FOXA1_robot
chr3	153068335	153068336	MACS_peak_11157	10.00	lobChIP_FOXA1_robot
chr3	153666175	153666176	MACS_peak_11158	3.00	lobChIP_FOXA1_robot
chr3	153740536	153740537	MACS_peak_11159	4.00	lobChIP_FOXA1_robot
chr3	153741119	153741120	MACS_peak_11160	13.00	lobChIP_FOXA1_robot
chr3	153843704	153843705	MACS_peak_11161	5.00	lobChIP_FOXA1_robot
chr3	153857975	153857976	MACS_peak_11162	20.00	lobChIP_FOXA1_robot
chr3	153882359	153882360	MACS_peak_11163	3.00	lobChIP_FOXA1_robot
chr3	153969666	153969667	MACS_peak_11164	9.00	lobChIP_FOXA1_robot
chr3	155323561	155323562	MACS_peak_11165	6.00	lobChIP_FOXA1_robot
chr3	156324638	156324639	MACS_peak_11166	6.00	lobChIP_FOXA1_robot
chr3	156446368	156446369	MACS_peak_11167	4.00	lobChIP_FOXA1_robot
chr3	156513879	156513880	MACS_peak_11168	7.00	lobChIP_FOXA1_robot
chr3	156530579	156530580	MACS_peak_11169	4.00	lobChIP_FOXA1_robot
chr3	156806641	156806642	MACS_peak_11170	3.00	lobChIP_FOXA1_robot
chr3	156838498	156838499	MACS_peak_11171	14.00	lobChIP_FOXA1_robot
chr3	156848547	156848548	MACS_peak_11172	3.00	lobChIP_FOXA1_robot
chr3	156854660	156854661	MACS_peak_11173	4.00	lobChIP_FOXA1_robot
chr3	157828635	157828636	MACS_peak_11174	6.00	lobChIP_FOXA1_robot
chr3	158086362	158086363	MACS_peak_11175	14.00	lobChIP_FOXA1_robot
chr3	158169424	158169425	MACS_peak_11176	17.00	lobChIP_FOXA1_robot
chr3	158170981	158170982	MACS_peak_11177	13.00	lobChIP_FOXA1_robot
chr3	158189643	158189644	MACS_peak_11178	4.00	lobChIP_FOXA1_robot
chr3	158487271	158487272	MACS_peak_11179	6.00	lobChIP_FOXA1_robot
chr3	159503240	159503241	MACS_peak_11180	6.00	lobChIP_FOXA1_robot
chr3	159505077	159505078	MACS_peak_11181	5.00	lobChIP_FOXA1_robot
chr3	159647446	159647447	MACS_peak_11182	4.00	lobChIP_FOXA1_robot
chr3	159809345	159809346	MACS_peak_11183	3.00	lobChIP_FOXA1_robot
chr3	159827034	159827035	MACS_peak_11184	9.00	lobChIP_FOXA1_robot
chr3	159928998	159928999	MACS_peak_11185	5.00	lobChIP_FOXA1_robot
chr3	160049074	160049075	MACS_peak_11186	5.00	lobChIP_FOXA1_robot
chr3	160090769	160090770	MACS_peak_11187	4.00	lobChIP_FOXA1_robot
chr3	160224916	160224917	MACS_peak_11188	5.00	lobChIP_FOXA1_robot
chr3	160990382	160990383	MACS_peak_11189	5.00	lobChIP_FOXA1_robot
chr3	161040816	161040817	MACS_peak_11190	9.00	lobChIP_FOXA1_robot
chr3	161041893	161041894	MACS_peak_11191	29.00	lobChIP_FOXA1_robot
chr3	161050748	161050749	MACS_peak_11192	4.00	lobChIP_FOXA1_robot
chr3	161138723	161138724	MACS_peak_11193	8.00	lobChIP_FOXA1_robot
chr3	161162060	161162061	MACS_peak_11194	5.00	lobChIP_FOXA1_robot
chr3	161634897	161634898	MACS_peak_11195	5.00	lobChIP_FOXA1_robot
chr3	161925260	161925261	MACS_peak_11196	3.00	lobChIP_FOXA1_robot
chr3	163266549	163266550	MACS_peak_11197	4.00	lobChIP_FOXA1_robot
chr3	165262963	165262964	MACS_peak_11198	4.00	lobChIP_FOXA1_robot
chr3	165569998	165569999	MACS_peak_11199	3.00	lobChIP_FOXA1_robot
chr3	166171412	166171413	MACS_peak_11200	15.00	lobChIP_FOXA1_robot
chr3	166547817	166547818	MACS_peak_11201	57.00	lobChIP_FOXA1_robot
chr3	167186157	167186158	MACS_peak_11202	9.00	lobChIP_FOXA1_robot
chr3	167434834	167434835	MACS_peak_11203	7.00	lobChIP_FOXA1_robot
chr3	167466263	167466264	MACS_peak_11204	3.00	lobChIP_FOXA1_robot
chr3	167531066	167531067	MACS_peak_11205	4.00	lobChIP_FOXA1_robot
chr3	167809998	167809999	MACS_peak_11206	5.00	lobChIP_FOXA1_robot
chr3	167835936	167835937	MACS_peak_11207	5.00	lobChIP_FOXA1_robot
chr3	168292675	168292676	MACS_peak_11208	5.00	lobChIP_FOXA1_robot
chr3	168577198	168577199	MACS_peak_11209	7.00	lobChIP_FOXA1_robot
chr3	168706956	168706957	MACS_peak_11210	4.00	lobChIP_FOXA1_robot
chr3	169404845	169404846	MACS_peak_11211	4.00	lobChIP_FOXA1_robot
chr3	170049174	170049175	MACS_peak_11212	6.00	lobChIP_FOXA1_robot
chr3	170146082	170146083	MACS_peak_11213	10.00	lobChIP_FOXA1_robot
chr3	170483436	170483437	MACS_peak_11214	7.00	lobChIP_FOXA1_robot
chr3	170585719	170585720	MACS_peak_11215	5.00	lobChIP_FOXA1_robot
chr3	170690923	170690924	MACS_peak_11216	6.00	lobChIP_FOXA1_robot
chr3	170715795	170715796	MACS_peak_11217	5.00	lobChIP_FOXA1_robot
chr3	170720283	170720284	MACS_peak_11218	46.00	lobChIP_FOXA1_robot
chr3	170731792	170731793	MACS_peak_11219	41.00	lobChIP_FOXA1_robot
chr3	170762404	170762405	MACS_peak_11220	9.00	lobChIP_FOXA1_robot
chr3	170763384	170763385	MACS_peak_11221	30.00	lobChIP_FOXA1_robot
chr3	170773571	170773572	MACS_peak_11222	24.00	lobChIP_FOXA1_robot
chr3	170848309	170848310	MACS_peak_11223	12.00	lobChIP_FOXA1_robot
chr3	170944768	170944769	MACS_peak_11224	22.00	lobChIP_FOXA1_robot
chr3	171004626	171004627	MACS_peak_11225	5.00	lobChIP_FOXA1_robot
chr3	171283647	171283648	MACS_peak_11226	4.00	lobChIP_FOXA1_robot
chr3	171397268	171397269	MACS_peak_11227	6.00	lobChIP_FOXA1_robot
chr3	171399009	171399010	MACS_peak_11228	4.00	lobChIP_FOXA1_robot
chr3	171419839	171419840	MACS_peak_11229	3.00	lobChIP_FOXA1_robot
chr3	171432716	171432717	MACS_peak_11230	5.00	lobChIP_FOXA1_robot
chr3	171524428	171524429	MACS_peak_11231	26.00	lobChIP_FOXA1_robot
chr3	171525799	171525800	MACS_peak_11232	15.00	lobChIP_FOXA1_robot
chr3	171529433	171529434	MACS_peak_11233	5.00	lobChIP_FOXA1_robot
chr3	171544479	171544480	MACS_peak_11234	6.00	lobChIP_FOXA1_robot
chr3	171559125	171559126	MACS_peak_11235	81.00	lobChIP_FOXA1_robot
chr3	171560823	171560824	MACS_peak_11236	6.00	lobChIP_FOXA1_robot
chr3	171563300	171563301	MACS_peak_11237	11.00	lobChIP_FOXA1_robot
chr3	171601176	171601177	MACS_peak_11238	46.00	lobChIP_FOXA1_robot
chr3	171619905	171619906	MACS_peak_11239	4.00	lobChIP_FOXA1_robot
chr3	171685189	171685190	MACS_peak_11240	15.00	lobChIP_FOXA1_robot
chr3	171691092	171691093	MACS_peak_11241	4.00	lobChIP_FOXA1_robot
chr3	171709106	171709107	MACS_peak_11242	7.00	lobChIP_FOXA1_robot
chr3	171724268	171724269	MACS_peak_11243	12.00	lobChIP_FOXA1_robot
chr3	171779683	171779684	MACS_peak_11244	5.00	lobChIP_FOXA1_robot
chr3	171784263	171784264	MACS_peak_11245	4.00	lobChIP_FOXA1_robot
chr3	171798505	171798506	MACS_peak_11246	7.00	lobChIP_FOXA1_robot
chr3	171801847	171801848	MACS_peak_11247	12.00	lobChIP_FOXA1_robot
chr3	171815087	171815088	MACS_peak_11248	13.00	lobChIP_FOXA1_robot
chr3	171816708	171816709	MACS_peak_11249	5.00	lobChIP_FOXA1_robot
chr3	171819075	171819076	MACS_peak_11250	21.00	lobChIP_FOXA1_robot
chr3	171820992	171820993	MACS_peak_11251	19.00	lobChIP_FOXA1_robot
chr3	171829112	171829113	MACS_peak_11252	4.00	lobChIP_FOXA1_robot
chr3	171837434	171837435	MACS_peak_11253	4.00	lobChIP_FOXA1_robot
chr3	171845917	171845918	MACS_peak_11254	4.00	lobChIP_FOXA1_robot
chr3	171847883	171847884	MACS_peak_11255	8.00	lobChIP_FOXA1_robot
chr3	171858650	171858651	MACS_peak_11256	9.00	lobChIP_FOXA1_robot
chr3	171897814	171897815	MACS_peak_11257	9.00	lobChIP_FOXA1_robot
chr3	171910838	171910839	MACS_peak_11258	5.00	lobChIP_FOXA1_robot
chr3	171921849	171921850	MACS_peak_11259	20.00	lobChIP_FOXA1_robot
chr3	171942777	171942778	MACS_peak_11260	4.00	lobChIP_FOXA1_robot
chr3	172271661	172271662	MACS_peak_11261	7.00	lobChIP_FOXA1_robot
chr3	172280935	172280936	MACS_peak_11262	6.00	lobChIP_FOXA1_robot
chr3	172319218	172319219	MACS_peak_11263	5.00	lobChIP_FOXA1_robot
chr3	172330726	172330727	MACS_peak_11264	5.00	lobChIP_FOXA1_robot
chr3	172371180	172371181	MACS_peak_11265	8.00	lobChIP_FOXA1_robot
chr3	172409297	172409298	MACS_peak_11266	15.00	lobChIP_FOXA1_robot
chr3	172414308	172414309	MACS_peak_11267	11.00	lobChIP_FOXA1_robot
chr3	172468251	172468252	MACS_peak_11268	41.00	lobChIP_FOXA1_robot
chr3	175334179	175334180	MACS_peak_11269	3.00	lobChIP_FOXA1_robot
chr3	177056603	177056604	MACS_peak_11270	5.00	lobChIP_FOXA1_robot
chr3	177077842	177077843	MACS_peak_11271	9.00	lobChIP_FOXA1_robot
chr3	177193279	177193280	MACS_peak_11272	6.00	lobChIP_FOXA1_robot
chr3	177292159	177292160	MACS_peak_11273	5.00	lobChIP_FOXA1_robot
chr3	177545417	177545418	MACS_peak_11274	7.00	lobChIP_FOXA1_robot
chr3	178865162	178865163	MACS_peak_11275	8.00	lobChIP_FOXA1_robot
chr3	178865801	178865802	MACS_peak_11276	6.00	lobChIP_FOXA1_robot
chr3	178867111	178867112	MACS_peak_11277	6.00	lobChIP_FOXA1_robot
chr3	178969002	178969003	MACS_peak_11278	15.00	lobChIP_FOXA1_robot
chr3	179362086	179362087	MACS_peak_11279	6.00	lobChIP_FOXA1_robot
chr3	179381055	179381056	MACS_peak_11280	4.00	lobChIP_FOXA1_robot
chr3	179457854	179457855	MACS_peak_11281	15.00	lobChIP_FOXA1_robot
chr3	181446344	181446345	MACS_peak_11282	4.00	lobChIP_FOXA1_robot
chr3	181854109	181854110	MACS_peak_11283	14.00	lobChIP_FOXA1_robot
chr3	181866363	181866364	MACS_peak_11284	5.00	lobChIP_FOXA1_robot
chr3	181930863	181930864	MACS_peak_11285	22.00	lobChIP_FOXA1_robot
chr3	182560190	182560191	MACS_peak_11286	5.00	lobChIP_FOXA1_robot
chr3	182697343	182697344	MACS_peak_11287	5.00	lobChIP_FOXA1_robot
chr3	182741095	182741096	MACS_peak_11288	3.00	lobChIP_FOXA1_robot
chr3	183009596	183009597	MACS_peak_11289	15.00	lobChIP_FOXA1_robot
chr3	183182406	183182407	MACS_peak_11290	5.00	lobChIP_FOXA1_robot
chr3	183463455	183463456	MACS_peak_11291	3.00	lobChIP_FOXA1_robot
chr3	183630007	183630008	MACS_peak_11292	12.00	lobChIP_FOXA1_robot
chr3	183631160	183631161	MACS_peak_11293	4.00	lobChIP_FOXA1_robot
chr3	184089529	184089530	MACS_peak_11294	8.00	lobChIP_FOXA1_robot
chr3	184968526	184968527	MACS_peak_11295	3.00	lobChIP_FOXA1_robot
chr3	184994372	184994373	MACS_peak_11296	5.00	lobChIP_FOXA1_robot
chr3	185046556	185046557	MACS_peak_11297	4.00	lobChIP_FOXA1_robot
chr3	185514542	185514543	MACS_peak_11298	13.00	lobChIP_FOXA1_robot
chr3	185540249	185540250	MACS_peak_11299	5.00	lobChIP_FOXA1_robot
chr3	185549105	185549106	MACS_peak_11300	7.00	lobChIP_FOXA1_robot
chr3	185552864	185552865	MACS_peak_11301	5.00	lobChIP_FOXA1_robot
chr3	185576446	185576447	MACS_peak_11302	7.00	lobChIP_FOXA1_robot
chr3	185684583	185684584	MACS_peak_11303	25.00	lobChIP_FOXA1_robot
chr3	185691104	185691105	MACS_peak_11304	10.00	lobChIP_FOXA1_robot
chr3	185827076	185827077	MACS_peak_11305	9.00	lobChIP_FOXA1_robot
chr3	186072186	186072187	MACS_peak_11306	3.00	lobChIP_FOXA1_robot
chr3	186317154	186317155	MACS_peak_11307	9.00	lobChIP_FOXA1_robot
chr3	186324245	186324246	MACS_peak_11308	40.00	lobChIP_FOXA1_robot
chr3	186330717	186330718	MACS_peak_11309	36.00	lobChIP_FOXA1_robot
chr3	186460922	186460923	MACS_peak_11310	16.00	lobChIP_FOXA1_robot
chr3	186471133	186471134	MACS_peak_11311	5.00	lobChIP_FOXA1_robot
chr3	186505957	186505958	MACS_peak_11312	4.00	lobChIP_FOXA1_robot
chr3	186625100	186625101	MACS_peak_11313	5.00	lobChIP_FOXA1_robot
chr3	186759318	186759319	MACS_peak_11314	62.00	lobChIP_FOXA1_robot
chr3	186780411	186780412	MACS_peak_11315	5.00	lobChIP_FOXA1_robot
chr3	186883016	186883017	MACS_peak_11316	5.00	lobChIP_FOXA1_robot
chr3	187385503	187385504	MACS_peak_11317	7.00	lobChIP_FOXA1_robot
chr3	187437537	187437538	MACS_peak_11318	7.00	lobChIP_FOXA1_robot
chr3	187468655	187468656	MACS_peak_11319	13.00	lobChIP_FOXA1_robot
chr3	187527029	187527030	MACS_peak_11320	4.00	lobChIP_FOXA1_robot
chr3	187686831	187686832	MACS_peak_11321	5.00	lobChIP_FOXA1_robot
chr3	187710599	187710600	MACS_peak_11322	4.00	lobChIP_FOXA1_robot
chr3	187715906	187715907	MACS_peak_11323	16.00	lobChIP_FOXA1_robot
chr3	187720595	187720596	MACS_peak_11324	4.00	lobChIP_FOXA1_robot
chr3	187721282	187721283	MACS_peak_11325	21.00	lobChIP_FOXA1_robot
chr3	187739582	187739583	MACS_peak_11326	7.00	lobChIP_FOXA1_robot
chr3	187754395	187754396	MACS_peak_11327	35.00	lobChIP_FOXA1_robot
chr3	187755110	187755111	MACS_peak_11328	4.00	lobChIP_FOXA1_robot
chr3	187776542	187776543	MACS_peak_11329	4.00	lobChIP_FOXA1_robot
chr3	187800919	187800920	MACS_peak_11330	9.00	lobChIP_FOXA1_robot
chr3	187841041	187841042	MACS_peak_11331	12.00	lobChIP_FOXA1_robot
chr3	187888938	187888939	MACS_peak_11332	16.00	lobChIP_FOXA1_robot
chr3	187905032	187905033	MACS_peak_11333	9.00	lobChIP_FOXA1_robot
chr3	187982161	187982162	MACS_peak_11334	3.00	lobChIP_FOXA1_robot
chr3	187988324	187988325	MACS_peak_11335	14.00	lobChIP_FOXA1_robot
chr3	188008477	188008478	MACS_peak_11336	15.00	lobChIP_FOXA1_robot
chr3	188012815	188012816	MACS_peak_11337	13.00	lobChIP_FOXA1_robot
chr3	188015086	188015087	MACS_peak_11338	10.00	lobChIP_FOXA1_robot
chr3	188191878	188191879	MACS_peak_11339	5.00	lobChIP_FOXA1_robot
chr3	188236765	188236766	MACS_peak_11340	9.00	lobChIP_FOXA1_robot
chr3	188260152	188260153	MACS_peak_11341	10.00	lobChIP_FOXA1_robot
chr3	188265056	188265057	MACS_peak_11342	8.00	lobChIP_FOXA1_robot
chr3	188425763	188425764	MACS_peak_11343	3.00	lobChIP_FOXA1_robot
chr3	188430390	188430391	MACS_peak_11344	3.00	lobChIP_FOXA1_robot
chr3	188438661	188438662	MACS_peak_11345	11.00	lobChIP_FOXA1_robot
chr3	188464266	188464267	MACS_peak_11346	58.00	lobChIP_FOXA1_robot
chr3	188490675	188490676	MACS_peak_11347	4.00	lobChIP_FOXA1_robot
chr3	188557271	188557272	MACS_peak_11348	39.00	lobChIP_FOXA1_robot
chr3	188558168	188558169	MACS_peak_11349	5.00	lobChIP_FOXA1_robot
chr3	188599173	188599174	MACS_peak_11350	19.00	lobChIP_FOXA1_robot
chr3	188776042	188776043	MACS_peak_11351	8.00	lobChIP_FOXA1_robot
chr3	189659694	189659695	MACS_peak_11352	3.00	lobChIP_FOXA1_robot
chr3	189789006	189789007	MACS_peak_11353	4.00	lobChIP_FOXA1_robot
chr3	190025232	190025233	MACS_peak_11354	39.00	lobChIP_FOXA1_robot
chr3	190048808	190048809	MACS_peak_11355	18.00	lobChIP_FOXA1_robot
chr3	190063273	190063274	MACS_peak_11356	33.00	lobChIP_FOXA1_robot
chr3	190092111	190092112	MACS_peak_11357	5.00	lobChIP_FOXA1_robot
chr3	190244141	190244142	MACS_peak_11358	9.00	lobChIP_FOXA1_robot
chr3	190245251	190245252	MACS_peak_11359	4.00	lobChIP_FOXA1_robot
chr3	190252920	190252921	MACS_peak_11360	12.00	lobChIP_FOXA1_robot
chr3	190266299	190266300	MACS_peak_11361	14.00	lobChIP_FOXA1_robot
chr3	190333066	190333067	MACS_peak_11362	7.00	lobChIP_FOXA1_robot
chr3	190980294	190980295	MACS_peak_11363	4.00	lobChIP_FOXA1_robot
chr3	191480449	191480450	MACS_peak_11364	5.00	lobChIP_FOXA1_robot
chr3	192569795	192569796	MACS_peak_11365	23.00	lobChIP_FOXA1_robot
chr3	192614931	192614932	MACS_peak_11366	4.00	lobChIP_FOXA1_robot
chr3	193411621	193411622	MACS_peak_11367	5.00	lobChIP_FOXA1_robot
chr3	193430173	193430174	MACS_peak_11368	51.00	lobChIP_FOXA1_robot
chr3	193462056	193462057	MACS_peak_11369	19.00	lobChIP_FOXA1_robot
chr3	193793741	193793742	MACS_peak_11370	5.00	lobChIP_FOXA1_robot
chr3	193819546	193819547	MACS_peak_11371	10.00	lobChIP_FOXA1_robot
chr3	193839921	193839922	MACS_peak_11372	6.00	lobChIP_FOXA1_robot
chr3	193849222	193849223	MACS_peak_11373	19.00	lobChIP_FOXA1_robot
chr3	193852614	193852615	MACS_peak_11374	6.00	lobChIP_FOXA1_robot
chr3	194038757	194038758	MACS_peak_11375	3.00	lobChIP_FOXA1_robot
chr3	194053822	194053823	MACS_peak_11376	4.00	lobChIP_FOXA1_robot
chr3	194107502	194107503	MACS_peak_11377	5.00	lobChIP_FOXA1_robot
chr3	194167656	194167657	MACS_peak_11378	31.00	lobChIP_FOXA1_robot
chr3	194215171	194215172	MACS_peak_11379	6.00	lobChIP_FOXA1_robot
chr3	194257615	194257616	MACS_peak_11380	5.00	lobChIP_FOXA1_robot
chr3	194443483	194443484	MACS_peak_11381	5.00	lobChIP_FOXA1_robot
chr3	194447844	194447845	MACS_peak_11382	6.00	lobChIP_FOXA1_robot
chr3	194470552	194470553	MACS_peak_11383	5.00	lobChIP_FOXA1_robot
chr3	194752380	194752381	MACS_peak_11384	8.00	lobChIP_FOXA1_robot
chr3	194826279	194826280	MACS_peak_11385	9.00	lobChIP_FOXA1_robot
chr3	194875871	194875872	MACS_peak_11386	4.00	lobChIP_FOXA1_robot
chr3	194881146	194881147	MACS_peak_11387	3.00	lobChIP_FOXA1_robot
chr3	194885581	194885582	MACS_peak_11388	14.00	lobChIP_FOXA1_robot
chr3	194886689	194886690	MACS_peak_11389	14.00	lobChIP_FOXA1_robot
chr3	194995431	194995432	MACS_peak_11390	4.00	lobChIP_FOXA1_robot
chr3	195275347	195275348	MACS_peak_11391	5.00	lobChIP_FOXA1_robot
chr3	195283460	195283461	MACS_peak_11392	4.00	lobChIP_FOXA1_robot
chr3	195847670	195847671	MACS_peak_11393	3.00	lobChIP_FOXA1_robot
chr3	196074306	196074307	MACS_peak_11394	21.00	lobChIP_FOXA1_robot
chr3	196464957	196464958	MACS_peak_11395	6.00	lobChIP_FOXA1_robot
chr3	196517149	196517150	MACS_peak_11396	5.00	lobChIP_FOXA1_robot
chr3	196689112	196689113	MACS_peak_11397	6.00	lobChIP_FOXA1_robot
chr3	196689707	196689708	MACS_peak_11398	6.00	lobChIP_FOXA1_robot
chr3	196708702	196708703	MACS_peak_11399	7.00	lobChIP_FOXA1_robot
chr3	196713128	196713129	MACS_peak_11400	4.00	lobChIP_FOXA1_robot
chr3	196817346	196817347	MACS_peak_11401	5.00	lobChIP_FOXA1_robot
chr3	197043142	197043143	MACS_peak_11402	7.00	lobChIP_FOXA1_robot
chr3	197082825	197082826	MACS_peak_11403	9.00	lobChIP_FOXA1_robot
chr3	197097683	197097684	MACS_peak_11404	7.00	lobChIP_FOXA1_robot
chr3	197192515	197192516	MACS_peak_11405	6.00	lobChIP_FOXA1_robot
chr3	197193148	197193149	MACS_peak_11406	7.00	lobChIP_FOXA1_robot
chr3	197300282	197300283	MACS_peak_11407	10.00	lobChIP_FOXA1_robot
chr3	197317508	197317509	MACS_peak_11408	19.00	lobChIP_FOXA1_robot
chr3	197548140	197548141	MACS_peak_11409	30.00	lobChIP_FOXA1_robot
chr3	197607712	197607713	MACS_peak_11410	14.00	lobChIP_FOXA1_robot
chr4	29208	29209	MACS_peak_11411	5.00	lobChIP_FOXA1_robot
chr4	48419	48420	MACS_peak_11412	3.00	lobChIP_FOXA1_robot
chr4	48964	48965	MACS_peak_11413	4.00	lobChIP_FOXA1_robot
chr4	52896	52897	MACS_peak_11414	3.00	lobChIP_FOXA1_robot
chr4	56688	56689	MACS_peak_11415	5.00	lobChIP_FOXA1_robot
chr4	535702	535703	MACS_peak_11416	7.00	lobChIP_FOXA1_robot
chr4	771994	771995	MACS_peak_11417	4.00	lobChIP_FOXA1_robot
chr4	809725	809726	MACS_peak_11418	13.00	lobChIP_FOXA1_robot
chr4	923872	923873	MACS_peak_11419	18.00	lobChIP_FOXA1_robot
chr4	987287	987288	MACS_peak_11420	4.00	lobChIP_FOXA1_robot
chr4	1914116	1914117	MACS_peak_11421	3.00	lobChIP_FOXA1_robot
chr4	2545830	2545831	MACS_peak_11422	5.00	lobChIP_FOXA1_robot
chr4	2763388	2763389	MACS_peak_11423	7.00	lobChIP_FOXA1_robot
chr4	2847629	2847630	MACS_peak_11424	4.00	lobChIP_FOXA1_robot
chr4	2868410	2868411	MACS_peak_11425	7.00	lobChIP_FOXA1_robot
chr4	2962975	2962976	MACS_peak_11426	9.00	lobChIP_FOXA1_robot
chr4	3180994	3180995	MACS_peak_11427	9.00	lobChIP_FOXA1_robot
chr4	3209734	3209735	MACS_peak_11428	4.00	lobChIP_FOXA1_robot
chr4	3344393	3344394	MACS_peak_11429	11.00	lobChIP_FOXA1_robot
chr4	3356316	3356317	MACS_peak_11430	14.00	lobChIP_FOXA1_robot
chr4	3673716	3673717	MACS_peak_11431	12.00	lobChIP_FOXA1_robot
chr4	3821128	3821129	MACS_peak_11432	8.00	lobChIP_FOXA1_robot
chr4	3838856	3838857	MACS_peak_11433	5.00	lobChIP_FOXA1_robot
chr4	4190397	4190398	MACS_peak_11434	16.00	lobChIP_FOXA1_robot
chr4	4250056	4250057	MACS_peak_11435	5.00	lobChIP_FOXA1_robot
chr4	4261522	4261523	MACS_peak_11436	8.00	lobChIP_FOXA1_robot
chr4	4463753	4463754	MACS_peak_11437	9.00	lobChIP_FOXA1_robot
chr4	4464860	4464861	MACS_peak_11438	5.00	lobChIP_FOXA1_robot
chr4	4488914	4488915	MACS_peak_11439	6.00	lobChIP_FOXA1_robot
chr4	4492764	4492765	MACS_peak_11440	14.00	lobChIP_FOXA1_robot
chr4	4536576	4536577	MACS_peak_11441	4.00	lobChIP_FOXA1_robot
chr4	4543111	4543112	MACS_peak_11442	5.00	lobChIP_FOXA1_robot
chr4	4714928	4714929	MACS_peak_11443	4.00	lobChIP_FOXA1_robot
chr4	5541479	5541480	MACS_peak_11444	7.00	lobChIP_FOXA1_robot
chr4	6202984	6202985	MACS_peak_11445	8.00	lobChIP_FOXA1_robot
chr4	6323261	6323262	MACS_peak_11446	13.00	lobChIP_FOXA1_robot
chr4	6324674	6324675	MACS_peak_11447	5.00	lobChIP_FOXA1_robot
chr4	6695542	6695543	MACS_peak_11448	15.00	lobChIP_FOXA1_robot
chr4	6697620	6697621	MACS_peak_11449	15.00	lobChIP_FOXA1_robot
chr4	6735451	6735452	MACS_peak_11450	8.00	lobChIP_FOXA1_robot
chr4	6753448	6753449	MACS_peak_11451	4.00	lobChIP_FOXA1_robot
chr4	6757041	6757042	MACS_peak_11452	4.00	lobChIP_FOXA1_robot
chr4	6895504	6895505	MACS_peak_11453	4.00	lobChIP_FOXA1_robot
chr4	6919557	6919558	MACS_peak_11454	13.00	lobChIP_FOXA1_robot
chr4	7036582	7036583	MACS_peak_11455	5.00	lobChIP_FOXA1_robot
chr4	7161861	7161862	MACS_peak_11456	6.00	lobChIP_FOXA1_robot
chr4	7751517	7751518	MACS_peak_11457	10.00	lobChIP_FOXA1_robot
chr4	7807434	7807435	MACS_peak_11458	16.00	lobChIP_FOXA1_robot
chr4	7859978	7859979	MACS_peak_11459	5.00	lobChIP_FOXA1_robot
chr4	7881884	7881885	MACS_peak_11460	6.00	lobChIP_FOXA1_robot
chr4	7971933	7971934	MACS_peak_11461	5.00	lobChIP_FOXA1_robot
chr4	8441119	8441120	MACS_peak_11462	4.00	lobChIP_FOXA1_robot
chr4	10019178	10019179	MACS_peak_11463	23.00	lobChIP_FOXA1_robot
chr4	10027253	10027254	MACS_peak_11464	7.00	lobChIP_FOXA1_robot
chr4	10272271	10272272	MACS_peak_11465	3.00	lobChIP_FOXA1_robot
chr4	12501519	12501520	MACS_peak_11466	8.00	lobChIP_FOXA1_robot
chr4	12935433	12935434	MACS_peak_11467	10.00	lobChIP_FOXA1_robot
chr4	12949623	12949624	MACS_peak_11468	4.00	lobChIP_FOXA1_robot
chr4	12951483	12951484	MACS_peak_11469	7.00	lobChIP_FOXA1_robot
chr4	12958856	12958857	MACS_peak_11470	5.00	lobChIP_FOXA1_robot
chr4	13369012	13369013	MACS_peak_11471	8.00	lobChIP_FOXA1_robot
chr4	13598993	13598994	MACS_peak_11472	18.00	lobChIP_FOXA1_robot
chr4	14868372	14868373	MACS_peak_11473	7.00	lobChIP_FOXA1_robot
chr4	15079776	15079777	MACS_peak_11474	6.00	lobChIP_FOXA1_robot
chr4	15151811	15151812	MACS_peak_11475	10.00	lobChIP_FOXA1_robot
chr4	15553826	15553827	MACS_peak_11476	4.00	lobChIP_FOXA1_robot
chr4	15701701	15701702	MACS_peak_11477	9.00	lobChIP_FOXA1_robot
chr4	15742586	15742587	MACS_peak_11478	7.00	lobChIP_FOXA1_robot
chr4	16031766	16031767	MACS_peak_11479	3.00	lobChIP_FOXA1_robot
chr4	16035740	16035741	MACS_peak_11480	10.00	lobChIP_FOXA1_robot
chr4	16131084	16131085	MACS_peak_11481	7.00	lobChIP_FOXA1_robot
chr4	16224730	16224731	MACS_peak_11482	4.00	lobChIP_FOXA1_robot
chr4	16226593	16226594	MACS_peak_11483	3.00	lobChIP_FOXA1_robot
chr4	16810691	16810692	MACS_peak_11484	3.00	lobChIP_FOXA1_robot
chr4	17492473	17492474	MACS_peak_11485	3.00	lobChIP_FOXA1_robot
chr4	17546172	17546173	MACS_peak_11486	17.00	lobChIP_FOXA1_robot
chr4	17558336	17558337	MACS_peak_11487	12.00	lobChIP_FOXA1_robot
chr4	17559690	17559691	MACS_peak_11488	5.00	lobChIP_FOXA1_robot
chr4	17560934	17560935	MACS_peak_11489	21.00	lobChIP_FOXA1_robot
chr4	18015903	18015904	MACS_peak_11490	3.00	lobChIP_FOXA1_robot
chr4	18569245	18569246	MACS_peak_11491	8.00	lobChIP_FOXA1_robot
chr4	18572232	18572233	MACS_peak_11492	4.00	lobChIP_FOXA1_robot
chr4	21207793	21207794	MACS_peak_11493	8.00	lobChIP_FOXA1_robot
chr4	21770148	21770149	MACS_peak_11494	11.00	lobChIP_FOXA1_robot
chr4	21780377	21780378	MACS_peak_11495	4.00	lobChIP_FOXA1_robot
chr4	23857973	23857974	MACS_peak_11496	8.00	lobChIP_FOXA1_robot
chr4	23859023	23859024	MACS_peak_11497	3.00	lobChIP_FOXA1_robot
chr4	23863738	23863739	MACS_peak_11498	5.00	lobChIP_FOXA1_robot
chr4	23866364	23866365	MACS_peak_11499	8.00	lobChIP_FOXA1_robot
chr4	23879519	23879520	MACS_peak_11500	19.00	lobChIP_FOXA1_robot
chr4	23882810	23882811	MACS_peak_11501	4.00	lobChIP_FOXA1_robot
chr4	24140109	24140110	MACS_peak_11502	8.00	lobChIP_FOXA1_robot
chr4	24543314	24543315	MACS_peak_11503	5.00	lobChIP_FOXA1_robot
chr4	24566875	24566876	MACS_peak_11504	6.00	lobChIP_FOXA1_robot
chr4	25161604	25161605	MACS_peak_11505	4.00	lobChIP_FOXA1_robot
chr4	25180626	25180627	MACS_peak_11506	4.00	lobChIP_FOXA1_robot
chr4	25813347	25813348	MACS_peak_11507	4.00	lobChIP_FOXA1_robot
chr4	25815330	25815331	MACS_peak_11508	4.00	lobChIP_FOXA1_robot
chr4	25921527	25921528	MACS_peak_11509	4.00	lobChIP_FOXA1_robot
chr4	26023829	26023830	MACS_peak_11510	4.00	lobChIP_FOXA1_robot
chr4	26121930	26121931	MACS_peak_11511	11.00	lobChIP_FOXA1_robot
chr4	26147046	26147047	MACS_peak_11512	5.00	lobChIP_FOXA1_robot
chr4	26374381	26374382	MACS_peak_11513	11.00	lobChIP_FOXA1_robot
chr4	26465968	26465969	MACS_peak_11514	7.00	lobChIP_FOXA1_robot
chr4	26824027	26824028	MACS_peak_11515	14.00	lobChIP_FOXA1_robot
chr4	30053354	30053355	MACS_peak_11516	5.00	lobChIP_FOXA1_robot
chr4	32663006	32663007	MACS_peak_11517	11.00	lobChIP_FOXA1_robot
chr4	32770458	32770459	MACS_peak_11518	5.00	lobChIP_FOXA1_robot
chr4	32843063	32843064	MACS_peak_11519	4.00	lobChIP_FOXA1_robot
chr4	34734932	34734933	MACS_peak_11520	8.00	lobChIP_FOXA1_robot
chr4	34747330	34747331	MACS_peak_11521	4.00	lobChIP_FOXA1_robot
chr4	37009970	37009971	MACS_peak_11522	22.00	lobChIP_FOXA1_robot
chr4	37432543	37432544	MACS_peak_11523	5.00	lobChIP_FOXA1_robot
chr4	37433118	37433119	MACS_peak_11524	4.00	lobChIP_FOXA1_robot
chr4	37585457	37585458	MACS_peak_11525	7.00	lobChIP_FOXA1_robot
chr4	37649925	37649926	MACS_peak_11526	5.00	lobChIP_FOXA1_robot
chr4	37664900	37664901	MACS_peak_11527	6.00	lobChIP_FOXA1_robot
chr4	37665338	37665339	MACS_peak_11528	4.00	lobChIP_FOXA1_robot
chr4	37735331	37735332	MACS_peak_11529	5.00	lobChIP_FOXA1_robot
chr4	37767645	37767646	MACS_peak_11530	13.00	lobChIP_FOXA1_robot
chr4	37805198	37805199	MACS_peak_11531	5.00	lobChIP_FOXA1_robot
chr4	37875241	37875242	MACS_peak_11532	8.00	lobChIP_FOXA1_robot
chr4	37955870	37955871	MACS_peak_11533	3.00	lobChIP_FOXA1_robot
chr4	38134917	38134918	MACS_peak_11534	25.00	lobChIP_FOXA1_robot
chr4	38142809	38142810	MACS_peak_11535	4.00	lobChIP_FOXA1_robot
chr4	38208908	38208909	MACS_peak_11536	5.00	lobChIP_FOXA1_robot
chr4	38225490	38225491	MACS_peak_11537	14.00	lobChIP_FOXA1_robot
chr4	38367574	38367575	MACS_peak_11538	4.00	lobChIP_FOXA1_robot
chr4	38387607	38387608	MACS_peak_11539	7.00	lobChIP_FOXA1_robot
chr4	38426352	38426353	MACS_peak_11540	4.00	lobChIP_FOXA1_robot
chr4	38492897	38492898	MACS_peak_11541	13.00	lobChIP_FOXA1_robot
chr4	38668790	38668791	MACS_peak_11542	4.00	lobChIP_FOXA1_robot
chr4	38675401	38675402	MACS_peak_11543	23.00	lobChIP_FOXA1_robot
chr4	38676530	38676531	MACS_peak_11544	3.00	lobChIP_FOXA1_robot
chr4	38874493	38874494	MACS_peak_11545	9.00	lobChIP_FOXA1_robot
chr4	38906071	38906072	MACS_peak_11546	8.00	lobChIP_FOXA1_robot
chr4	38932091	38932092	MACS_peak_11547	5.00	lobChIP_FOXA1_robot
chr4	39045010	39045011	MACS_peak_11548	24.00	lobChIP_FOXA1_robot
chr4	39047737	39047738	MACS_peak_11549	5.00	lobChIP_FOXA1_robot
chr4	39322231	39322232	MACS_peak_11550	8.00	lobChIP_FOXA1_robot
chr4	39339641	39339642	MACS_peak_11551	14.00	lobChIP_FOXA1_robot
chr4	39341754	39341755	MACS_peak_11552	7.00	lobChIP_FOXA1_robot
chr4	39342747	39342748	MACS_peak_11553	7.00	lobChIP_FOXA1_robot
chr4	39377186	39377187	MACS_peak_11554	5.00	lobChIP_FOXA1_robot
chr4	39415517	39415518	MACS_peak_11555	18.00	lobChIP_FOXA1_robot
chr4	39417359	39417360	MACS_peak_11556	3.00	lobChIP_FOXA1_robot
chr4	39420818	39420819	MACS_peak_11557	5.00	lobChIP_FOXA1_robot
chr4	39431570	39431571	MACS_peak_11558	7.00	lobChIP_FOXA1_robot
chr4	39454234	39454235	MACS_peak_11559	3.00	lobChIP_FOXA1_robot
chr4	39516727	39516728	MACS_peak_11560	8.00	lobChIP_FOXA1_robot
chr4	39560088	39560089	MACS_peak_11561	29.00	lobChIP_FOXA1_robot
chr4	39614827	39614828	MACS_peak_11562	5.00	lobChIP_FOXA1_robot
chr4	39619348	39619349	MACS_peak_11563	4.00	lobChIP_FOXA1_robot
chr4	39620825	39620826	MACS_peak_11564	5.00	lobChIP_FOXA1_robot
chr4	39698840	39698841	MACS_peak_11565	7.00	lobChIP_FOXA1_robot
chr4	40048160	40048161	MACS_peak_11566	9.00	lobChIP_FOXA1_robot
chr4	40059140	40059141	MACS_peak_11567	5.00	lobChIP_FOXA1_robot
chr4	40193384	40193385	MACS_peak_11568	25.00	lobChIP_FOXA1_robot
chr4	40193909	40193910	MACS_peak_11569	3.00	lobChIP_FOXA1_robot
chr4	40449461	40449462	MACS_peak_11570	5.00	lobChIP_FOXA1_robot
chr4	40476175	40476176	MACS_peak_11571	4.00	lobChIP_FOXA1_robot
chr4	40579425	40579426	MACS_peak_11572	7.00	lobChIP_FOXA1_robot
chr4	41079886	41079887	MACS_peak_11573	8.00	lobChIP_FOXA1_robot
chr4	41134529	41134530	MACS_peak_11574	37.00	lobChIP_FOXA1_robot
chr4	41166887	41166888	MACS_peak_11575	5.00	lobChIP_FOXA1_robot
chr4	41179337	41179338	MACS_peak_11576	4.00	lobChIP_FOXA1_robot
chr4	42826717	42826718	MACS_peak_11577	9.00	lobChIP_FOXA1_robot
chr4	43362427	43362428	MACS_peak_11578	8.00	lobChIP_FOXA1_robot
chr4	43845765	43845766	MACS_peak_11579	5.00	lobChIP_FOXA1_robot
chr4	47652993	47652994	MACS_peak_11580	4.00	lobChIP_FOXA1_robot
chr4	47718215	47718216	MACS_peak_11581	14.00	lobChIP_FOXA1_robot
chr4	47738270	47738271	MACS_peak_11582	5.00	lobChIP_FOXA1_robot
chr4	47879020	47879021	MACS_peak_11583	4.00	lobChIP_FOXA1_robot
chr4	47908439	47908440	MACS_peak_11584	5.00	lobChIP_FOXA1_robot
chr4	48045228	48045229	MACS_peak_11585	6.00	lobChIP_FOXA1_robot
chr4	48331455	48331456	MACS_peak_11586	10.00	lobChIP_FOXA1_robot
chr4	48398276	48398277	MACS_peak_11587	4.00	lobChIP_FOXA1_robot
chr4	48701675	48701676	MACS_peak_11588	9.00	lobChIP_FOXA1_robot
chr4	48960336	48960337	MACS_peak_11589	6.00	lobChIP_FOXA1_robot
chr4	49151781	49151782	MACS_peak_11591	30.00	lobChIP_FOXA1_robot
chr4	52775568	52775569	MACS_peak_11593	7.00	lobChIP_FOXA1_robot
chr4	53068655	53068656	MACS_peak_11594	3.00	lobChIP_FOXA1_robot
chr4	53235255	53235256	MACS_peak_11595	8.00	lobChIP_FOXA1_robot
chr4	53236100	53236101	MACS_peak_11596	21.00	lobChIP_FOXA1_robot
chr4	53801603	53801604	MACS_peak_11597	5.00	lobChIP_FOXA1_robot
chr4	53814051	53814052	MACS_peak_11598	7.00	lobChIP_FOXA1_robot
chr4	53875465	53875466	MACS_peak_11599	5.00	lobChIP_FOXA1_robot
chr4	53886154	53886155	MACS_peak_11600	15.00	lobChIP_FOXA1_robot
chr4	53988905	53988906	MACS_peak_11601	3.00	lobChIP_FOXA1_robot
chr4	54054561	54054562	MACS_peak_11602	18.00	lobChIP_FOXA1_robot
chr4	54322153	54322154	MACS_peak_11603	28.00	lobChIP_FOXA1_robot
chr4	54325945	54325946	MACS_peak_11604	27.00	lobChIP_FOXA1_robot
chr4	54647999	54648000	MACS_peak_11605	12.00	lobChIP_FOXA1_robot
chr4	54873102	54873103	MACS_peak_11606	16.00	lobChIP_FOXA1_robot
chr4	54948551	54948552	MACS_peak_11607	14.00	lobChIP_FOXA1_robot
chr4	55988687	55988688	MACS_peak_11608	5.00	lobChIP_FOXA1_robot
chr4	56305444	56305445	MACS_peak_11609	5.00	lobChIP_FOXA1_robot
chr4	56435233	56435234	MACS_peak_11610	6.00	lobChIP_FOXA1_robot
chr4	56814442	56814443	MACS_peak_11611	4.00	lobChIP_FOXA1_robot
chr4	56815746	56815747	MACS_peak_11612	4.00	lobChIP_FOXA1_robot
chr4	56870640	56870641	MACS_peak_11613	22.00	lobChIP_FOXA1_robot
chr4	56890111	56890112	MACS_peak_11614	4.00	lobChIP_FOXA1_robot
chr4	57081769	57081770	MACS_peak_11615	4.00	lobChIP_FOXA1_robot
chr4	57111881	57111882	MACS_peak_11616	4.00	lobChIP_FOXA1_robot
chr4	57311104	57311105	MACS_peak_11617	4.00	lobChIP_FOXA1_robot
chr4	57967205	57967206	MACS_peak_11618	4.00	lobChIP_FOXA1_robot
chr4	62328603	62328604	MACS_peak_11619	10.00	lobChIP_FOXA1_robot
chr4	64583338	64583339	MACS_peak_11620	7.00	lobChIP_FOXA1_robot
chr4	65370208	65370209	MACS_peak_11621	8.00	lobChIP_FOXA1_robot
chr4	66882141	66882142	MACS_peak_11622	16.00	lobChIP_FOXA1_robot
chr4	67880552	67880553	MACS_peak_11623	7.00	lobChIP_FOXA1_robot
chr4	69113629	69113630	MACS_peak_11624	4.00	lobChIP_FOXA1_robot
chr4	69215811	69215812	MACS_peak_11625	8.00	lobChIP_FOXA1_robot
chr4	69431614	69431615	MACS_peak_11626	3.00	lobChIP_FOXA1_robot
chr4	69522090	69522091	MACS_peak_11627	10.00	lobChIP_FOXA1_robot
chr4	69524576	69524577	MACS_peak_11628	3.00	lobChIP_FOXA1_robot
chr4	69598970	69598971	MACS_peak_11629	16.00	lobChIP_FOXA1_robot
chr4	69681532	69681533	MACS_peak_11630	23.00	lobChIP_FOXA1_robot
chr4	69683850	69683851	MACS_peak_11631	4.00	lobChIP_FOXA1_robot
chr4	69730950	69730951	MACS_peak_11632	9.00	lobChIP_FOXA1_robot
chr4	69736699	69736700	MACS_peak_11633	6.00	lobChIP_FOXA1_robot
chr4	69738298	69738299	MACS_peak_11634	7.00	lobChIP_FOXA1_robot
chr4	69774206	69774207	MACS_peak_11635	4.00	lobChIP_FOXA1_robot
chr4	69886290	69886291	MACS_peak_11636	17.00	lobChIP_FOXA1_robot
chr4	69952153	69952154	MACS_peak_11637	5.00	lobChIP_FOXA1_robot
chr4	69961373	69961374	MACS_peak_11638	11.00	lobChIP_FOXA1_robot
chr4	69962086	69962087	MACS_peak_11639	8.00	lobChIP_FOXA1_robot
chr4	69964466	69964467	MACS_peak_11640	10.00	lobChIP_FOXA1_robot
chr4	69968570	69968571	MACS_peak_11641	3.00	lobChIP_FOXA1_robot
chr4	70254988	70254989	MACS_peak_11642	4.00	lobChIP_FOXA1_robot
chr4	70647713	70647714	MACS_peak_11643	17.00	lobChIP_FOXA1_robot
chr4	70689387	70689388	MACS_peak_11644	25.00	lobChIP_FOXA1_robot
chr4	70698553	70698554	MACS_peak_11645	23.00	lobChIP_FOXA1_robot
chr4	70726780	70726781	MACS_peak_11646	5.00	lobChIP_FOXA1_robot
chr4	71048752	71048753	MACS_peak_11647	36.00	lobChIP_FOXA1_robot
chr4	71058873	71058874	MACS_peak_11648	4.00	lobChIP_FOXA1_robot
chr4	71069442	71069443	MACS_peak_11649	3.00	lobChIP_FOXA1_robot
chr4	71099896	71099897	MACS_peak_11650	4.00	lobChIP_FOXA1_robot
chr4	71105432	71105433	MACS_peak_11651	8.00	lobChIP_FOXA1_robot
chr4	71117756	71117757	MACS_peak_11652	3.00	lobChIP_FOXA1_robot
chr4	71425816	71425817	MACS_peak_11653	16.00	lobChIP_FOXA1_robot
chr4	71430027	71430028	MACS_peak_11654	7.00	lobChIP_FOXA1_robot
chr4	71575465	71575466	MACS_peak_11655	10.00	lobChIP_FOXA1_robot
chr4	71659241	71659242	MACS_peak_11656	4.00	lobChIP_FOXA1_robot
chr4	71668856	71668857	MACS_peak_11657	5.00	lobChIP_FOXA1_robot
chr4	71933189	71933190	MACS_peak_11658	25.00	lobChIP_FOXA1_robot
chr4	72474097	72474098	MACS_peak_11659	8.00	lobChIP_FOXA1_robot
chr4	73533947	73533948	MACS_peak_11660	4.00	lobChIP_FOXA1_robot
chr4	74160187	74160188	MACS_peak_11661	5.00	lobChIP_FOXA1_robot
chr4	74211564	74211565	MACS_peak_11662	28.00	lobChIP_FOXA1_robot
chr4	74213457	74213458	MACS_peak_11663	8.00	lobChIP_FOXA1_robot
chr4	74241163	74241164	MACS_peak_11664	12.00	lobChIP_FOXA1_robot
chr4	74244748	74244749	MACS_peak_11665	8.00	lobChIP_FOXA1_robot
chr4	74250754	74250755	MACS_peak_11666	4.00	lobChIP_FOXA1_robot
chr4	74254853	74254854	MACS_peak_11667	12.00	lobChIP_FOXA1_robot
chr4	74264526	74264527	MACS_peak_11668	3.00	lobChIP_FOXA1_robot
chr4	74267357	74267358	MACS_peak_11669	7.00	lobChIP_FOXA1_robot
chr4	74281871	74281872	MACS_peak_11670	5.00	lobChIP_FOXA1_robot
chr4	74298495	74298496	MACS_peak_11671	42.00	lobChIP_FOXA1_robot
chr4	74301376	74301377	MACS_peak_11672	3.00	lobChIP_FOXA1_robot
chr4	74347237	74347238	MACS_peak_11673	8.00	lobChIP_FOXA1_robot
chr4	74473261	74473262	MACS_peak_11674	5.00	lobChIP_FOXA1_robot
chr4	74782855	74782856	MACS_peak_11675	5.00	lobChIP_FOXA1_robot
chr4	74871345	74871346	MACS_peak_11676	9.00	lobChIP_FOXA1_robot
chr4	74965558	74965559	MACS_peak_11677	5.00	lobChIP_FOXA1_robot
chr4	74966469	74966470	MACS_peak_11678	5.00	lobChIP_FOXA1_robot
chr4	75057006	75057007	MACS_peak_11679	9.00	lobChIP_FOXA1_robot
chr4	75067972	75067973	MACS_peak_11680	76.00	lobChIP_FOXA1_robot
chr4	75069103	75069104	MACS_peak_11681	4.00	lobChIP_FOXA1_robot
chr4	75344940	75344941	MACS_peak_11682	27.00	lobChIP_FOXA1_robot
chr4	75550070	75550071	MACS_peak_11683	3.00	lobChIP_FOXA1_robot
chr4	75579436	75579437	MACS_peak_11684	8.00	lobChIP_FOXA1_robot
chr4	75585264	75585265	MACS_peak_11685	10.00	lobChIP_FOXA1_robot
chr4	75586995	75586996	MACS_peak_11686	8.00	lobChIP_FOXA1_robot
chr4	76430969	76430970	MACS_peak_11687	4.00	lobChIP_FOXA1_robot
chr4	76441107	76441108	MACS_peak_11688	6.00	lobChIP_FOXA1_robot
chr4	76599131	76599132	MACS_peak_11689	5.00	lobChIP_FOXA1_robot
chr4	77101087	77101088	MACS_peak_11690	6.00	lobChIP_FOXA1_robot
chr4	77129685	77129686	MACS_peak_11691	9.00	lobChIP_FOXA1_robot
chr4	77193498	77193499	MACS_peak_11692	8.00	lobChIP_FOXA1_robot
chr4	77196829	77196830	MACS_peak_11693	4.00	lobChIP_FOXA1_robot
chr4	77233717	77233718	MACS_peak_11694	3.00	lobChIP_FOXA1_robot
chr4	77491658	77491659	MACS_peak_11695	3.00	lobChIP_FOXA1_robot
chr4	77492643	77492644	MACS_peak_11696	11.00	lobChIP_FOXA1_robot
chr4	77752226	77752227	MACS_peak_11697	9.00	lobChIP_FOXA1_robot
chr4	77771373	77771374	MACS_peak_11698	8.00	lobChIP_FOXA1_robot
chr4	77820309	77820310	MACS_peak_11699	13.00	lobChIP_FOXA1_robot
chr4	77852957	77852958	MACS_peak_11700	8.00	lobChIP_FOXA1_robot
chr4	78558240	78558241	MACS_peak_11701	7.00	lobChIP_FOXA1_robot
chr4	78687111	78687112	MACS_peak_11702	5.00	lobChIP_FOXA1_robot
chr4	78703474	78703475	MACS_peak_11703	3.00	lobChIP_FOXA1_robot
chr4	78803246	78803247	MACS_peak_11704	9.00	lobChIP_FOXA1_robot
chr4	78842980	78842981	MACS_peak_11705	7.00	lobChIP_FOXA1_robot
chr4	78917421	78917422	MACS_peak_11706	12.00	lobChIP_FOXA1_robot
chr4	78936200	78936201	MACS_peak_11707	38.00	lobChIP_FOXA1_robot
chr4	79057710	79057711	MACS_peak_11708	3.00	lobChIP_FOXA1_robot
chr4	79071933	79071934	MACS_peak_11709	10.00	lobChIP_FOXA1_robot
chr4	79077199	79077200	MACS_peak_11710	5.00	lobChIP_FOXA1_robot
chr4	79087158	79087159	MACS_peak_11711	6.00	lobChIP_FOXA1_robot
chr4	79088959	79088960	MACS_peak_11712	4.00	lobChIP_FOXA1_robot
chr4	79097848	79097849	MACS_peak_11713	11.00	lobChIP_FOXA1_robot
chr4	79122358	79122359	MACS_peak_11714	8.00	lobChIP_FOXA1_robot
chr4	79434864	79434865	MACS_peak_11715	4.00	lobChIP_FOXA1_robot
chr4	79488808	79488809	MACS_peak_11716	4.00	lobChIP_FOXA1_robot
chr4	79583296	79583297	MACS_peak_11717	14.00	lobChIP_FOXA1_robot
chr4	79946611	79946612	MACS_peak_11718	6.00	lobChIP_FOXA1_robot
chr4	80992302	80992303	MACS_peak_11719	11.00	lobChIP_FOXA1_robot
chr4	81536497	81536498	MACS_peak_11720	22.00	lobChIP_FOXA1_robot
chr4	82543835	82543836	MACS_peak_11721	4.00	lobChIP_FOXA1_robot
chr4	82547423	82547424	MACS_peak_11722	5.00	lobChIP_FOXA1_robot
chr4	82597455	82597456	MACS_peak_11723	3.00	lobChIP_FOXA1_robot
chr4	83453168	83453169	MACS_peak_11724	17.00	lobChIP_FOXA1_robot
chr4	83806300	83806301	MACS_peak_11725	5.00	lobChIP_FOXA1_robot
chr4	83806697	83806698	MACS_peak_11726	11.00	lobChIP_FOXA1_robot
chr4	83932283	83932284	MACS_peak_11727	4.00	lobChIP_FOXA1_robot
chr4	84119024	84119025	MACS_peak_11728	4.00	lobChIP_FOXA1_robot
chr4	84278515	84278516	MACS_peak_11729	10.00	lobChIP_FOXA1_robot
chr4	84282897	84282898	MACS_peak_11730	8.00	lobChIP_FOXA1_robot
chr4	84284191	84284192	MACS_peak_11731	8.00	lobChIP_FOXA1_robot
chr4	84407980	84407981	MACS_peak_11732	5.00	lobChIP_FOXA1_robot
chr4	84499606	84499607	MACS_peak_11733	9.00	lobChIP_FOXA1_robot
chr4	84570238	84570239	MACS_peak_11734	5.00	lobChIP_FOXA1_robot
chr4	84614598	84614599	MACS_peak_11735	11.00	lobChIP_FOXA1_robot
chr4	85965147	85965148	MACS_peak_11736	5.00	lobChIP_FOXA1_robot
chr4	85972957	85972958	MACS_peak_11737	5.00	lobChIP_FOXA1_robot
chr4	86850692	86850693	MACS_peak_11738	4.00	lobChIP_FOXA1_robot
chr4	87030193	87030194	MACS_peak_11739	12.00	lobChIP_FOXA1_robot
chr4	87723332	87723333	MACS_peak_11740	4.00	lobChIP_FOXA1_robot
chr4	87848336	87848337	MACS_peak_11741	9.00	lobChIP_FOXA1_robot
chr4	87849277	87849278	MACS_peak_11742	8.00	lobChIP_FOXA1_robot
chr4	87857862	87857863	MACS_peak_11743	3.00	lobChIP_FOXA1_robot
chr4	87895091	87895092	MACS_peak_11744	13.00	lobChIP_FOXA1_robot
chr4	87915599	87915600	MACS_peak_11745	5.00	lobChIP_FOXA1_robot
chr4	87934000	87934001	MACS_peak_11746	5.00	lobChIP_FOXA1_robot
chr4	87946926	87946927	MACS_peak_11747	4.00	lobChIP_FOXA1_robot
chr4	88010656	88010657	MACS_peak_11748	7.00	lobChIP_FOXA1_robot
chr4	88026443	88026444	MACS_peak_11749	3.00	lobChIP_FOXA1_robot
chr4	88199648	88199649	MACS_peak_11750	19.00	lobChIP_FOXA1_robot
chr4	88330833	88330834	MACS_peak_11751	12.00	lobChIP_FOXA1_robot
chr4	88338315	88338316	MACS_peak_11752	5.00	lobChIP_FOXA1_robot
chr4	88340997	88340998	MACS_peak_11753	14.00	lobChIP_FOXA1_robot
chr4	88348626	88348627	MACS_peak_11754	3.00	lobChIP_FOXA1_robot
chr4	88787484	88787485	MACS_peak_11755	25.00	lobChIP_FOXA1_robot
chr4	88832649	88832650	MACS_peak_11756	8.00	lobChIP_FOXA1_robot
chr4	89028903	89028904	MACS_peak_11757	7.00	lobChIP_FOXA1_robot
chr4	89030574	89030575	MACS_peak_11758	28.00	lobChIP_FOXA1_robot
chr4	89137697	89137698	MACS_peak_11759	5.00	lobChIP_FOXA1_robot
chr4	89436252	89436253	MACS_peak_11760	14.00	lobChIP_FOXA1_robot
chr4	89675347	89675348	MACS_peak_11761	6.00	lobChIP_FOXA1_robot
chr4	90033547	90033548	MACS_peak_11762	5.00	lobChIP_FOXA1_robot
chr4	90080222	90080223	MACS_peak_11763	4.00	lobChIP_FOXA1_robot
chr4	90566810	90566811	MACS_peak_11764	4.00	lobChIP_FOXA1_robot
chr4	90596247	90596248	MACS_peak_11765	7.00	lobChIP_FOXA1_robot
chr4	90602474	90602475	MACS_peak_11766	6.00	lobChIP_FOXA1_robot
chr4	93421651	93421652	MACS_peak_11767	4.00	lobChIP_FOXA1_robot
chr4	93520250	93520251	MACS_peak_11768	5.00	lobChIP_FOXA1_robot
chr4	93891343	93891344	MACS_peak_11769	7.00	lobChIP_FOXA1_robot
chr4	93942947	93942948	MACS_peak_11770	3.00	lobChIP_FOXA1_robot
chr4	95064081	95064082	MACS_peak_11771	4.00	lobChIP_FOXA1_robot
chr4	95220221	95220222	MACS_peak_11772	9.00	lobChIP_FOXA1_robot
chr4	95333858	95333859	MACS_peak_11773	6.00	lobChIP_FOXA1_robot
chr4	95346844	95346845	MACS_peak_11774	12.00	lobChIP_FOXA1_robot
chr4	95350182	95350183	MACS_peak_11775	7.00	lobChIP_FOXA1_robot
chr4	95394198	95394199	MACS_peak_11776	19.00	lobChIP_FOXA1_robot
chr4	95394835	95394836	MACS_peak_11777	5.00	lobChIP_FOXA1_robot
chr4	95469145	95469146	MACS_peak_11778	5.00	lobChIP_FOXA1_robot
chr4	95487817	95487818	MACS_peak_11779	16.00	lobChIP_FOXA1_robot
chr4	95517147	95517148	MACS_peak_11780	5.00	lobChIP_FOXA1_robot
chr4	95818464	95818465	MACS_peak_11781	3.00	lobChIP_FOXA1_robot
chr4	96141932	96141933	MACS_peak_11782	12.00	lobChIP_FOXA1_robot
chr4	96622511	96622512	MACS_peak_11783	4.00	lobChIP_FOXA1_robot
chr4	97137315	97137316	MACS_peak_11784	4.00	lobChIP_FOXA1_robot
chr4	97514912	97514913	MACS_peak_11785	8.00	lobChIP_FOXA1_robot
chr4	98831638	98831639	MACS_peak_11786	4.00	lobChIP_FOXA1_robot
chr4	99014157	99014158	MACS_peak_11787	6.00	lobChIP_FOXA1_robot
chr4	99239856	99239857	MACS_peak_11788	7.00	lobChIP_FOXA1_robot
chr4	99505603	99505604	MACS_peak_11789	7.00	lobChIP_FOXA1_robot
chr4	99564410	99564411	MACS_peak_11790	3.00	lobChIP_FOXA1_robot
chr4	99699600	99699601	MACS_peak_11791	8.00	lobChIP_FOXA1_robot
chr4	99856095	99856096	MACS_peak_11792	6.00	lobChIP_FOXA1_robot
chr4	99921254	99921255	MACS_peak_11793	11.00	lobChIP_FOXA1_robot
chr4	99948675	99948676	MACS_peak_11794	6.00	lobChIP_FOXA1_robot
chr4	99988860	99988861	MACS_peak_11795	3.00	lobChIP_FOXA1_robot
chr4	100078799	100078800	MACS_peak_11796	111.00	lobChIP_FOXA1_robot
chr4	100087663	100087664	MACS_peak_11797	3.00	lobChIP_FOXA1_robot
chr4	100324884	100324885	MACS_peak_11798	4.00	lobChIP_FOXA1_robot
chr4	100493845	100493846	MACS_peak_11799	6.00	lobChIP_FOXA1_robot
chr4	100495880	100495881	MACS_peak_11800	7.00	lobChIP_FOXA1_robot
chr4	100785178	100785179	MACS_peak_11801	4.00	lobChIP_FOXA1_robot
chr4	101744913	101744914	MACS_peak_11802	5.00	lobChIP_FOXA1_robot
chr4	101919337	101919338	MACS_peak_11803	4.00	lobChIP_FOXA1_robot
chr4	102036464	102036465	MACS_peak_11804	11.00	lobChIP_FOXA1_robot
chr4	102090232	102090233	MACS_peak_11805	8.00	lobChIP_FOXA1_robot
chr4	102110280	102110281	MACS_peak_11806	5.00	lobChIP_FOXA1_robot
chr4	102132367	102132368	MACS_peak_11807	5.00	lobChIP_FOXA1_robot
chr4	102177739	102177740	MACS_peak_11808	3.00	lobChIP_FOXA1_robot
chr4	102185951	102185952	MACS_peak_11809	19.00	lobChIP_FOXA1_robot
chr4	102186897	102186898	MACS_peak_11810	4.00	lobChIP_FOXA1_robot
chr4	102192589	102192590	MACS_peak_11811	8.00	lobChIP_FOXA1_robot
chr4	102277656	102277657	MACS_peak_11812	8.00	lobChIP_FOXA1_robot
chr4	102806979	102806980	MACS_peak_11813	13.00	lobChIP_FOXA1_robot
chr4	102894597	102894598	MACS_peak_11814	6.00	lobChIP_FOXA1_robot
chr4	103363583	103363584	MACS_peak_11815	9.00	lobChIP_FOXA1_robot
chr4	103416833	103416834	MACS_peak_11816	3.00	lobChIP_FOXA1_robot
chr4	103490526	103490527	MACS_peak_11817	4.00	lobChIP_FOXA1_robot
chr4	103634484	103634485	MACS_peak_11818	12.00	lobChIP_FOXA1_robot
chr4	103656204	103656205	MACS_peak_11819	5.00	lobChIP_FOXA1_robot
chr4	103749457	103749458	MACS_peak_11820	4.00	lobChIP_FOXA1_robot
chr4	104123307	104123308	MACS_peak_11821	4.00	lobChIP_FOXA1_robot
chr4	104346155	104346156	MACS_peak_11822	5.00	lobChIP_FOXA1_robot
chr4	105050091	105050092	MACS_peak_11823	4.00	lobChIP_FOXA1_robot
chr4	105649769	105649770	MACS_peak_11824	4.00	lobChIP_FOXA1_robot
chr4	106093305	106093306	MACS_peak_11825	10.00	lobChIP_FOXA1_robot
chr4	106507397	106507398	MACS_peak_11826	5.00	lobChIP_FOXA1_robot
chr4	106759479	106759480	MACS_peak_11827	3.00	lobChIP_FOXA1_robot
chr4	106819642	106819643	MACS_peak_11828	22.00	lobChIP_FOXA1_robot
chr4	106851919	106851920	MACS_peak_11829	5.00	lobChIP_FOXA1_robot
chr4	106958234	106958235	MACS_peak_11830	8.00	lobChIP_FOXA1_robot
chr4	107127983	107127984	MACS_peak_11831	3.00	lobChIP_FOXA1_robot
chr4	107179563	107179564	MACS_peak_11832	30.00	lobChIP_FOXA1_robot
chr4	107183352	107183353	MACS_peak_11833	5.00	lobChIP_FOXA1_robot
chr4	107188381	107188382	MACS_peak_11834	4.00	lobChIP_FOXA1_robot
chr4	107208671	107208672	MACS_peak_11835	17.00	lobChIP_FOXA1_robot
chr4	107254438	107254439	MACS_peak_11836	3.00	lobChIP_FOXA1_robot
chr4	107265724	107265725	MACS_peak_11837	9.00	lobChIP_FOXA1_robot
chr4	107286892	107286893	MACS_peak_11838	4.00	lobChIP_FOXA1_robot
chr4	108135995	108135996	MACS_peak_11839	6.00	lobChIP_FOXA1_robot
chr4	108605254	108605255	MACS_peak_11840	10.00	lobChIP_FOXA1_robot
chr4	109413043	109413044	MACS_peak_11841	5.00	lobChIP_FOXA1_robot
chr4	109513999	109514000	MACS_peak_11842	13.00	lobChIP_FOXA1_robot
chr4	110894918	110894919	MACS_peak_11843	4.00	lobChIP_FOXA1_robot
chr4	110985559	110985560	MACS_peak_11844	7.00	lobChIP_FOXA1_robot
chr4	111561969	111561970	MACS_peak_11845	6.00	lobChIP_FOXA1_robot
chr4	111842976	111842977	MACS_peak_11846	3.00	lobChIP_FOXA1_robot
chr4	112563094	112563095	MACS_peak_11847	3.00	lobChIP_FOXA1_robot
chr4	112765624	112765625	MACS_peak_11848	4.00	lobChIP_FOXA1_robot
chr4	113021034	113021035	MACS_peak_11849	7.00	lobChIP_FOXA1_robot
chr4	113033876	113033877	MACS_peak_11850	5.00	lobChIP_FOXA1_robot
chr4	113034374	113034375	MACS_peak_11851	24.00	lobChIP_FOXA1_robot
chr4	113124862	113124863	MACS_peak_11852	7.00	lobChIP_FOXA1_robot
chr4	114063200	114063201	MACS_peak_11853	19.00	lobChIP_FOXA1_robot
chr4	114092382	114092383	MACS_peak_11854	5.00	lobChIP_FOXA1_robot
chr4	114102581	114102582	MACS_peak_11855	6.00	lobChIP_FOXA1_robot
chr4	114255431	114255432	MACS_peak_11856	5.00	lobChIP_FOXA1_robot
chr4	114325604	114325605	MACS_peak_11857	13.00	lobChIP_FOXA1_robot
chr4	114562395	114562396	MACS_peak_11858	5.00	lobChIP_FOXA1_robot
chr4	114880564	114880565	MACS_peak_11859	10.00	lobChIP_FOXA1_robot
chr4	116765642	116765643	MACS_peak_11860	6.00	lobChIP_FOXA1_robot
chr4	117056649	117056650	MACS_peak_11861	4.00	lobChIP_FOXA1_robot
chr4	118321457	118321458	MACS_peak_11862	5.00	lobChIP_FOXA1_robot
chr4	119624508	119624509	MACS_peak_11863	5.00	lobChIP_FOXA1_robot
chr4	119679044	119679045	MACS_peak_11864	5.00	lobChIP_FOXA1_robot
chr4	119779092	119779093	MACS_peak_11865	6.00	lobChIP_FOXA1_robot
chr4	120002326	120002327	MACS_peak_11866	5.00	lobChIP_FOXA1_robot
chr4	120014307	120014308	MACS_peak_11867	4.00	lobChIP_FOXA1_robot
chr4	120400007	120400008	MACS_peak_11868	6.00	lobChIP_FOXA1_robot
chr4	120425778	120425779	MACS_peak_11869	13.00	lobChIP_FOXA1_robot
chr4	120446687	120446688	MACS_peak_11870	8.00	lobChIP_FOXA1_robot
chr4	120628638	120628639	MACS_peak_11871	4.00	lobChIP_FOXA1_robot
chr4	120713228	120713229	MACS_peak_11872	4.00	lobChIP_FOXA1_robot
chr4	122597994	122597995	MACS_peak_11873	10.00	lobChIP_FOXA1_robot
chr4	122612701	122612702	MACS_peak_11874	8.00	lobChIP_FOXA1_robot
chr4	123894704	123894705	MACS_peak_11875	4.00	lobChIP_FOXA1_robot
chr4	123965133	123965134	MACS_peak_11876	9.00	lobChIP_FOXA1_robot
chr4	123985455	123985456	MACS_peak_11877	3.00	lobChIP_FOXA1_robot
chr4	124037780	124037781	MACS_peak_11878	5.00	lobChIP_FOXA1_robot
chr4	124270857	124270858	MACS_peak_11879	5.00	lobChIP_FOXA1_robot
chr4	124318411	124318412	MACS_peak_11880	4.00	lobChIP_FOXA1_robot
chr4	124333560	124333561	MACS_peak_11881	6.00	lobChIP_FOXA1_robot
chr4	124337305	124337306	MACS_peak_11882	9.00	lobChIP_FOXA1_robot
chr4	124339816	124339817	MACS_peak_11883	15.00	lobChIP_FOXA1_robot
chr4	124400255	124400256	MACS_peak_11884	15.00	lobChIP_FOXA1_robot
chr4	124401229	124401230	MACS_peak_11885	17.00	lobChIP_FOXA1_robot
chr4	124426406	124426407	MACS_peak_11886	7.00	lobChIP_FOXA1_robot
chr4	124426984	124426985	MACS_peak_11887	23.00	lobChIP_FOXA1_robot
chr4	124446589	124446590	MACS_peak_11888	6.00	lobChIP_FOXA1_robot
chr4	124476709	124476710	MACS_peak_11889	8.00	lobChIP_FOXA1_robot
chr4	124520542	124520543	MACS_peak_11890	12.00	lobChIP_FOXA1_robot
chr4	124521396	124521397	MACS_peak_11891	24.00	lobChIP_FOXA1_robot
chr4	124541480	124541481	MACS_peak_11892	4.00	lobChIP_FOXA1_robot
chr4	124560195	124560196	MACS_peak_11893	5.00	lobChIP_FOXA1_robot
chr4	124571051	124571052	MACS_peak_11894	6.00	lobChIP_FOXA1_robot
chr4	124778056	124778057	MACS_peak_11895	8.00	lobChIP_FOXA1_robot
chr4	124778513	124778514	MACS_peak_11896	15.00	lobChIP_FOXA1_robot
chr4	125448101	125448102	MACS_peak_11897	6.00	lobChIP_FOXA1_robot
chr4	125451980	125451981	MACS_peak_11898	13.00	lobChIP_FOXA1_robot
chr4	125800512	125800513	MACS_peak_11899	7.00	lobChIP_FOXA1_robot
chr4	128605094	128605095	MACS_peak_11900	4.00	lobChIP_FOXA1_robot
chr4	128981739	128981740	MACS_peak_11901	4.00	lobChIP_FOXA1_robot
chr4	129049172	129049173	MACS_peak_11902	3.00	lobChIP_FOXA1_robot
chr4	129084785	129084786	MACS_peak_11903	5.00	lobChIP_FOXA1_robot
chr4	129206944	129206945	MACS_peak_11904	5.00	lobChIP_FOXA1_robot
chr4	129271135	129271136	MACS_peak_11905	17.00	lobChIP_FOXA1_robot
chr4	129350527	129350528	MACS_peak_11906	6.00	lobChIP_FOXA1_robot
chr4	129474223	129474224	MACS_peak_11907	4.00	lobChIP_FOXA1_robot
chr4	129525841	129525842	MACS_peak_11908	5.00	lobChIP_FOXA1_robot
chr4	129879370	129879371	MACS_peak_11909	3.00	lobChIP_FOXA1_robot
chr4	135054236	135054237	MACS_peak_11910	4.00	lobChIP_FOXA1_robot
chr4	138324088	138324089	MACS_peak_11911	3.00	lobChIP_FOXA1_robot
chr4	138450443	138450444	MACS_peak_11912	3.00	lobChIP_FOXA1_robot
chr4	139120754	139120755	MACS_peak_11913	5.00	lobChIP_FOXA1_robot
chr4	139289254	139289255	MACS_peak_11914	4.00	lobChIP_FOXA1_robot
chr4	139888619	139888620	MACS_peak_11915	6.00	lobChIP_FOXA1_robot
chr4	140004162	140004163	MACS_peak_11916	7.00	lobChIP_FOXA1_robot
chr4	140074967	140074968	MACS_peak_11917	9.00	lobChIP_FOXA1_robot
chr4	140416965	140416966	MACS_peak_11918	4.00	lobChIP_FOXA1_robot
chr4	140587022	140587023	MACS_peak_11919	9.00	lobChIP_FOXA1_robot
chr4	140610716	140610717	MACS_peak_11920	9.00	lobChIP_FOXA1_robot
chr4	140613999	140614000	MACS_peak_11921	9.00	lobChIP_FOXA1_robot
chr4	140763205	140763206	MACS_peak_11922	8.00	lobChIP_FOXA1_robot
chr4	140778354	140778355	MACS_peak_11923	6.00	lobChIP_FOXA1_robot
chr4	140843005	140843006	MACS_peak_11924	5.00	lobChIP_FOXA1_robot
chr4	140923565	140923566	MACS_peak_11925	3.00	lobChIP_FOXA1_robot
chr4	140941346	140941347	MACS_peak_11926	18.00	lobChIP_FOXA1_robot
chr4	140958679	140958680	MACS_peak_11927	4.00	lobChIP_FOXA1_robot
chr4	140985687	140985688	MACS_peak_11928	4.00	lobChIP_FOXA1_robot
chr4	141005899	141005900	MACS_peak_11929	3.00	lobChIP_FOXA1_robot
chr4	141349370	141349371	MACS_peak_11930	5.00	lobChIP_FOXA1_robot
chr4	141536850	141536851	MACS_peak_11931	6.00	lobChIP_FOXA1_robot
chr4	141562034	141562035	MACS_peak_11932	4.00	lobChIP_FOXA1_robot
chr4	141562454	141562455	MACS_peak_11933	5.00	lobChIP_FOXA1_robot
chr4	141567369	141567370	MACS_peak_11934	5.00	lobChIP_FOXA1_robot
chr4	141649344	141649345	MACS_peak_11935	23.00	lobChIP_FOXA1_robot
chr4	142476974	142476975	MACS_peak_11936	9.00	lobChIP_FOXA1_robot
chr4	144146224	144146225	MACS_peak_11937	6.00	lobChIP_FOXA1_robot
chr4	144310893	144310894	MACS_peak_11938	33.00	lobChIP_FOXA1_robot
chr4	144804727	144804728	MACS_peak_11939	4.00	lobChIP_FOXA1_robot
chr4	146616212	146616213	MACS_peak_11940	3.00	lobChIP_FOXA1_robot
chr4	146860060	146860061	MACS_peak_11941	3.00	lobChIP_FOXA1_robot
chr4	148160984	148160985	MACS_peak_11942	6.00	lobChIP_FOXA1_robot
chr4	149297525	149297526	MACS_peak_11943	13.00	lobChIP_FOXA1_robot
chr4	150185475	150185476	MACS_peak_11944	5.00	lobChIP_FOXA1_robot
chr4	150976723	150976724	MACS_peak_11945	12.00	lobChIP_FOXA1_robot
chr4	151002237	151002238	MACS_peak_11946	8.00	lobChIP_FOXA1_robot
chr4	151040840	151040841	MACS_peak_11947	20.00	lobChIP_FOXA1_robot
chr4	151178559	151178560	MACS_peak_11948	6.00	lobChIP_FOXA1_robot
chr4	151277331	151277332	MACS_peak_11949	3.00	lobChIP_FOXA1_robot
chr4	151278147	151278148	MACS_peak_11950	3.00	lobChIP_FOXA1_robot
chr4	151384216	151384217	MACS_peak_11951	7.00	lobChIP_FOXA1_robot
chr4	151410339	151410340	MACS_peak_11952	6.00	lobChIP_FOXA1_robot
chr4	151636107	151636108	MACS_peak_11953	19.00	lobChIP_FOXA1_robot
chr4	151788497	151788498	MACS_peak_11954	8.00	lobChIP_FOXA1_robot
chr4	151895365	151895366	MACS_peak_11955	8.00	lobChIP_FOXA1_robot
chr4	151956702	151956703	MACS_peak_11956	4.00	lobChIP_FOXA1_robot
chr4	152020641	152020642	MACS_peak_11957	3.00	lobChIP_FOXA1_robot
chr4	152043830	152043831	MACS_peak_11958	5.00	lobChIP_FOXA1_robot
chr4	152126366	152126367	MACS_peak_11959	5.00	lobChIP_FOXA1_robot
chr4	152173300	152173301	MACS_peak_11960	6.00	lobChIP_FOXA1_robot
chr4	152187995	152187996	MACS_peak_11961	4.00	lobChIP_FOXA1_robot
chr4	152188452	152188453	MACS_peak_11962	3.00	lobChIP_FOXA1_robot
chr4	152241795	152241796	MACS_peak_11963	4.00	lobChIP_FOXA1_robot
chr4	152245609	152245610	MACS_peak_11964	5.00	lobChIP_FOXA1_robot
chr4	152247009	152247010	MACS_peak_11965	7.00	lobChIP_FOXA1_robot
chr4	153347410	153347411	MACS_peak_11966	7.00	lobChIP_FOXA1_robot
chr4	153407040	153407041	MACS_peak_11967	12.00	lobChIP_FOXA1_robot
chr4	153408339	153408340	MACS_peak_11968	11.00	lobChIP_FOXA1_robot
chr4	153743744	153743745	MACS_peak_11969	3.00	lobChIP_FOXA1_robot
chr4	153880569	153880570	MACS_peak_11970	4.00	lobChIP_FOXA1_robot
chr4	153960088	153960089	MACS_peak_11971	7.00	lobChIP_FOXA1_robot
chr4	153988718	153988719	MACS_peak_11972	3.00	lobChIP_FOXA1_robot
chr4	154130795	154130796	MACS_peak_11973	7.00	lobChIP_FOXA1_robot
chr4	154406702	154406703	MACS_peak_11974	9.00	lobChIP_FOXA1_robot
chr4	154433484	154433485	MACS_peak_11975	10.00	lobChIP_FOXA1_robot
chr4	154485865	154485866	MACS_peak_11976	7.00	lobChIP_FOXA1_robot
chr4	154531951	154531952	MACS_peak_11977	3.00	lobChIP_FOXA1_robot
chr4	155473410	155473411	MACS_peak_11978	5.00	lobChIP_FOXA1_robot
chr4	155481561	155481562	MACS_peak_11979	14.00	lobChIP_FOXA1_robot
chr4	155498600	155498601	MACS_peak_11980	8.00	lobChIP_FOXA1_robot
chr4	155512016	155512017	MACS_peak_11981	21.00	lobChIP_FOXA1_robot
chr4	155517116	155517117	MACS_peak_11982	10.00	lobChIP_FOXA1_robot
chr4	155519395	155519396	MACS_peak_11983	39.00	lobChIP_FOXA1_robot
chr4	155529526	155529527	MACS_peak_11984	5.00	lobChIP_FOXA1_robot
chr4	155534111	155534112	MACS_peak_11985	7.00	lobChIP_FOXA1_robot
chr4	155535736	155535737	MACS_peak_11986	3.00	lobChIP_FOXA1_robot
chr4	155539054	155539055	MACS_peak_11987	106.00	lobChIP_FOXA1_robot
chr4	155581444	155581445	MACS_peak_11988	23.00	lobChIP_FOXA1_robot
chr4	155759418	155759419	MACS_peak_11989	10.00	lobChIP_FOXA1_robot
chr4	156876212	156876213	MACS_peak_11990	4.00	lobChIP_FOXA1_robot
chr4	157575763	157575764	MACS_peak_11991	9.00	lobChIP_FOXA1_robot
chr4	157701055	157701056	MACS_peak_11992	4.00	lobChIP_FOXA1_robot
chr4	157713793	157713794	MACS_peak_11993	6.00	lobChIP_FOXA1_robot
chr4	157852658	157852659	MACS_peak_11994	7.00	lobChIP_FOXA1_robot
chr4	157863337	157863338	MACS_peak_11995	19.00	lobChIP_FOXA1_robot
chr4	157870693	157870694	MACS_peak_11996	6.00	lobChIP_FOXA1_robot
chr4	158342879	158342880	MACS_peak_11997	5.00	lobChIP_FOXA1_robot
chr4	158731442	158731443	MACS_peak_11998	5.00	lobChIP_FOXA1_robot
chr4	158914338	158914339	MACS_peak_11999	12.00	lobChIP_FOXA1_robot
chr4	158916154	158916155	MACS_peak_12000	80.00	lobChIP_FOXA1_robot
chr4	158941564	158941565	MACS_peak_12001	6.00	lobChIP_FOXA1_robot
chr4	159015715	159015716	MACS_peak_12002	3.00	lobChIP_FOXA1_robot
chr4	159164418	159164419	MACS_peak_12003	6.00	lobChIP_FOXA1_robot
chr4	159733868	159733869	MACS_peak_12004	6.00	lobChIP_FOXA1_robot
chr4	159748094	159748095	MACS_peak_12005	10.00	lobChIP_FOXA1_robot
chr4	159823904	159823905	MACS_peak_12006	6.00	lobChIP_FOXA1_robot
chr4	159927567	159927568	MACS_peak_12007	4.00	lobChIP_FOXA1_robot
chr4	160027310	160027311	MACS_peak_12008	8.00	lobChIP_FOXA1_robot
chr4	160096870	160096871	MACS_peak_12009	3.00	lobChIP_FOXA1_robot
chr4	160201847	160201848	MACS_peak_12010	3.00	lobChIP_FOXA1_robot
chr4	160204254	160204255	MACS_peak_12011	6.00	lobChIP_FOXA1_robot
chr4	160289388	160289389	MACS_peak_12012	3.00	lobChIP_FOXA1_robot
chr4	160341538	160341539	MACS_peak_12013	14.00	lobChIP_FOXA1_robot
chr4	162721082	162721083	MACS_peak_12014	12.00	lobChIP_FOXA1_robot
chr4	163221996	163221997	MACS_peak_12015	4.00	lobChIP_FOXA1_robot
chr4	164983517	164983518	MACS_peak_12016	4.00	lobChIP_FOXA1_robot
chr4	165540295	165540296	MACS_peak_12017	4.00	lobChIP_FOXA1_robot
chr4	166085668	166085669	MACS_peak_12018	4.00	lobChIP_FOXA1_robot
chr4	166290977	166290978	MACS_peak_12019	17.00	lobChIP_FOXA1_robot
chr4	166291903	166291904	MACS_peak_12020	5.00	lobChIP_FOXA1_robot
chr4	166424282	166424283	MACS_peak_12021	18.00	lobChIP_FOXA1_robot
chr4	168098057	168098058	MACS_peak_12022	11.00	lobChIP_FOXA1_robot
chr4	168708943	168708944	MACS_peak_12023	4.00	lobChIP_FOXA1_robot
chr4	169075072	169075073	MACS_peak_12024	5.00	lobChIP_FOXA1_robot
chr4	169422722	169422723	MACS_peak_12025	5.00	lobChIP_FOXA1_robot
chr4	169602611	169602612	MACS_peak_12026	4.00	lobChIP_FOXA1_robot
chr4	169620597	169620598	MACS_peak_12027	7.00	lobChIP_FOXA1_robot
chr4	169718292	169718293	MACS_peak_12028	3.00	lobChIP_FOXA1_robot
chr4	169926790	169926791	MACS_peak_12029	10.00	lobChIP_FOXA1_robot
chr4	169937418	169937419	MACS_peak_12030	3.00	lobChIP_FOXA1_robot
chr4	169992102	169992103	MACS_peak_12031	9.00	lobChIP_FOXA1_robot
chr4	170165120	170165121	MACS_peak_12032	9.00	lobChIP_FOXA1_robot
chr4	170170860	170170861	MACS_peak_12033	11.00	lobChIP_FOXA1_robot
chr4	170188435	170188436	MACS_peak_12034	4.00	lobChIP_FOXA1_robot
chr4	170190552	170190553	MACS_peak_12035	7.00	lobChIP_FOXA1_robot
chr4	170542998	170542999	MACS_peak_12036	6.00	lobChIP_FOXA1_robot
chr4	172333361	172333362	MACS_peak_12037	3.00	lobChIP_FOXA1_robot
chr4	173647425	173647426	MACS_peak_12038	7.00	lobChIP_FOXA1_robot
chr4	174404777	174404778	MACS_peak_12039	4.00	lobChIP_FOXA1_robot
chr4	175157747	175157748	MACS_peak_12040	3.00	lobChIP_FOXA1_robot
chr4	175181464	175181465	MACS_peak_12041	5.00	lobChIP_FOXA1_robot
chr4	177140345	177140346	MACS_peak_12042	6.00	lobChIP_FOXA1_robot
chr4	178362147	178362148	MACS_peak_12043	4.00	lobChIP_FOXA1_robot
chr4	179681014	179681015	MACS_peak_12044	5.00	lobChIP_FOXA1_robot
chr4	180337096	180337097	MACS_peak_12045	6.00	lobChIP_FOXA1_robot
chr4	183798920	183798921	MACS_peak_12046	6.00	lobChIP_FOXA1_robot
chr4	183817114	183817115	MACS_peak_12047	6.00	lobChIP_FOXA1_robot
chr4	183986102	183986103	MACS_peak_12048	3.00	lobChIP_FOXA1_robot
chr4	184001877	184001878	MACS_peak_12049	19.00	lobChIP_FOXA1_robot
chr4	184022236	184022237	MACS_peak_12050	7.00	lobChIP_FOXA1_robot
chr4	184023505	184023506	MACS_peak_12051	9.00	lobChIP_FOXA1_robot
chr4	184032002	184032003	MACS_peak_12052	4.00	lobChIP_FOXA1_robot
chr4	184032725	184032726	MACS_peak_12053	23.00	lobChIP_FOXA1_robot
chr4	184076042	184076043	MACS_peak_12054	4.00	lobChIP_FOXA1_robot
chr4	184093065	184093066	MACS_peak_12055	5.00	lobChIP_FOXA1_robot
chr4	184112446	184112447	MACS_peak_12056	33.00	lobChIP_FOXA1_robot
chr4	184163047	184163048	MACS_peak_12057	5.00	lobChIP_FOXA1_robot
chr4	184181844	184181845	MACS_peak_12058	8.00	lobChIP_FOXA1_robot
chr4	184365364	184365365	MACS_peak_12059	3.00	lobChIP_FOXA1_robot
chr4	184389781	184389782	MACS_peak_12060	6.00	lobChIP_FOXA1_robot
chr4	184540026	184540027	MACS_peak_12061	9.00	lobChIP_FOXA1_robot
chr4	184540785	184540786	MACS_peak_12062	8.00	lobChIP_FOXA1_robot
chr4	185025831	185025832	MACS_peak_12063	20.00	lobChIP_FOXA1_robot
chr4	185269918	185269919	MACS_peak_12064	5.00	lobChIP_FOXA1_robot
chr4	185305302	185305303	MACS_peak_12065	6.00	lobChIP_FOXA1_robot
chr4	185384064	185384065	MACS_peak_12066	6.00	lobChIP_FOXA1_robot
chr4	185386206	185386207	MACS_peak_12067	6.00	lobChIP_FOXA1_robot
chr4	185395802	185395803	MACS_peak_12068	4.00	lobChIP_FOXA1_robot
chr4	185517983	185517984	MACS_peak_12069	4.00	lobChIP_FOXA1_robot
chr4	185547519	185547520	MACS_peak_12070	3.00	lobChIP_FOXA1_robot
chr4	185729379	185729380	MACS_peak_12071	4.00	lobChIP_FOXA1_robot
chr4	185734647	185734648	MACS_peak_12072	7.00	lobChIP_FOXA1_robot
chr4	185759636	185759637	MACS_peak_12073	30.00	lobChIP_FOXA1_robot
chr4	186123760	186123761	MACS_peak_12074	5.00	lobChIP_FOXA1_robot
chr4	186157161	186157162	MACS_peak_12075	6.00	lobChIP_FOXA1_robot
chr4	186175686	186175687	MACS_peak_12076	4.00	lobChIP_FOXA1_robot
chr4	186240108	186240109	MACS_peak_12077	6.00	lobChIP_FOXA1_robot
chr4	186254422	186254423	MACS_peak_12078	4.00	lobChIP_FOXA1_robot
chr4	186301686	186301687	MACS_peak_12079	9.00	lobChIP_FOXA1_robot
chr4	186435251	186435252	MACS_peak_12080	5.00	lobChIP_FOXA1_robot
chr4	186489086	186489087	MACS_peak_12081	4.00	lobChIP_FOXA1_robot
chr4	186572586	186572587	MACS_peak_12082	5.00	lobChIP_FOXA1_robot
chr4	186635012	186635013	MACS_peak_12083	14.00	lobChIP_FOXA1_robot
chr4	186643215	186643216	MACS_peak_12084	12.00	lobChIP_FOXA1_robot
chr4	186695618	186695619	MACS_peak_12085	14.00	lobChIP_FOXA1_robot
chr4	186714024	186714025	MACS_peak_12086	4.00	lobChIP_FOXA1_robot
chr4	186726587	186726588	MACS_peak_12087	12.00	lobChIP_FOXA1_robot
chr4	186732533	186732534	MACS_peak_12088	23.00	lobChIP_FOXA1_robot
chr4	186751659	186751660	MACS_peak_12089	3.00	lobChIP_FOXA1_robot
chr4	186757700	186757701	MACS_peak_12090	5.00	lobChIP_FOXA1_robot
chr4	186879484	186879485	MACS_peak_12091	4.00	lobChIP_FOXA1_robot
chr4	187148619	187148620	MACS_peak_12092	8.00	lobChIP_FOXA1_robot
chr4	187186939	187186940	MACS_peak_12093	4.00	lobChIP_FOXA1_robot
chr4	187565394	187565395	MACS_peak_12094	6.00	lobChIP_FOXA1_robot
chr4	187566009	187566010	MACS_peak_12095	5.00	lobChIP_FOXA1_robot
chr4	187606226	187606227	MACS_peak_12096	10.00	lobChIP_FOXA1_robot
chr4	187607172	187607173	MACS_peak_12097	12.00	lobChIP_FOXA1_robot
chr4	187609856	187609857	MACS_peak_12098	7.00	lobChIP_FOXA1_robot
chr4	187631795	187631796	MACS_peak_12099	4.00	lobChIP_FOXA1_robot
chr4	187641278	187641279	MACS_peak_12100	5.00	lobChIP_FOXA1_robot
chr4	187654771	187654772	MACS_peak_12101	4.00	lobChIP_FOXA1_robot
chr4	187687039	187687040	MACS_peak_12102	5.00	lobChIP_FOXA1_robot
chr4	187702982	187702983	MACS_peak_12103	4.00	lobChIP_FOXA1_robot
chr4	187726885	187726886	MACS_peak_12104	8.00	lobChIP_FOXA1_robot
chr4	187825516	187825517	MACS_peak_12105	4.00	lobChIP_FOXA1_robot
chr4	187847208	187847209	MACS_peak_12106	3.00	lobChIP_FOXA1_robot
chr4	190510994	190510995	MACS_peak_12107	4.00	lobChIP_FOXA1_robot
chr4	190512285	190512286	MACS_peak_12108	4.00	lobChIP_FOXA1_robot
chr4	190547314	190547315	MACS_peak_12109	4.00	lobChIP_FOXA1_robot
chr4	190591435	190591436	MACS_peak_12110	4.00	lobChIP_FOXA1_robot
chr4	190603806	190603807	MACS_peak_12111	5.00	lobChIP_FOXA1_robot
chr4	190644687	190644688	MACS_peak_12112	3.00	lobChIP_FOXA1_robot
chr4	190660738	190660739	MACS_peak_12113	3.00	lobChIP_FOXA1_robot
chr4	190825739	190825740	MACS_peak_12114	3.00	lobChIP_FOXA1_robot
chr4	190879513	190879514	MACS_peak_12115	5.00	lobChIP_FOXA1_robot
chr5	919709	919710	MACS_peak_12116	11.00	lobChIP_FOXA1_robot
chr5	1140765	1140766	MACS_peak_12117	5.00	lobChIP_FOXA1_robot
chr5	1555640	1555641	MACS_peak_12118	12.00	lobChIP_FOXA1_robot
chr5	1564822	1564823	MACS_peak_12119	4.00	lobChIP_FOXA1_robot
chr5	1800299	1800300	MACS_peak_12120	5.00	lobChIP_FOXA1_robot
chr5	2352765	2352766	MACS_peak_12121	5.00	lobChIP_FOXA1_robot
chr5	2802972	2802973	MACS_peak_12122	6.00	lobChIP_FOXA1_robot
chr5	5136259	5136260	MACS_peak_12123	7.00	lobChIP_FOXA1_robot
chr5	5361444	5361445	MACS_peak_12124	4.00	lobChIP_FOXA1_robot
chr5	5642018	5642019	MACS_peak_12125	3.00	lobChIP_FOXA1_robot
chr5	6496140	6496141	MACS_peak_12126	7.00	lobChIP_FOXA1_robot
chr5	6716008	6716009	MACS_peak_12127	4.00	lobChIP_FOXA1_robot
chr5	6874999	6875000	MACS_peak_12128	4.00	lobChIP_FOXA1_robot
chr5	6901124	6901125	MACS_peak_12129	10.00	lobChIP_FOXA1_robot
chr5	7385394	7385395	MACS_peak_12130	5.00	lobChIP_FOXA1_robot
chr5	7878780	7878781	MACS_peak_12131	6.00	lobChIP_FOXA1_robot
chr5	10157662	10157663	MACS_peak_12132	4.00	lobChIP_FOXA1_robot
chr5	10347840	10347841	MACS_peak_12133	5.00	lobChIP_FOXA1_robot
chr5	10354513	10354514	MACS_peak_12134	3.00	lobChIP_FOXA1_robot
chr5	10760669	10760670	MACS_peak_12135	3.00	lobChIP_FOXA1_robot
chr5	10860066	10860067	MACS_peak_12136	7.00	lobChIP_FOXA1_robot
chr5	10976274	10976275	MACS_peak_12137	6.00	lobChIP_FOXA1_robot
chr5	11596960	11596961	MACS_peak_12138	27.00	lobChIP_FOXA1_robot
chr5	11733819	11733820	MACS_peak_12139	4.00	lobChIP_FOXA1_robot
chr5	14009301	14009302	MACS_peak_12140	9.00	lobChIP_FOXA1_robot
chr5	14160298	14160299	MACS_peak_12141	3.00	lobChIP_FOXA1_robot
chr5	14208868	14208869	MACS_peak_12142	12.00	lobChIP_FOXA1_robot
chr5	14213215	14213216	MACS_peak_12143	9.00	lobChIP_FOXA1_robot
chr5	14570256	14570257	MACS_peak_12144	13.00	lobChIP_FOXA1_robot
chr5	14753645	14753646	MACS_peak_12145	6.00	lobChIP_FOXA1_robot
chr5	14774794	14774795	MACS_peak_12146	15.00	lobChIP_FOXA1_robot
chr5	14915589	14915590	MACS_peak_12147	5.00	lobChIP_FOXA1_robot
chr5	15008783	15008784	MACS_peak_12148	5.00	lobChIP_FOXA1_robot
chr5	15009804	15009805	MACS_peak_12149	8.00	lobChIP_FOXA1_robot
chr5	16272144	16272145	MACS_peak_12150	14.00	lobChIP_FOXA1_robot
chr5	16462084	16462085	MACS_peak_12151	5.00	lobChIP_FOXA1_robot
chr5	16588778	16588779	MACS_peak_12152	4.00	lobChIP_FOXA1_robot
chr5	16708017	16708018	MACS_peak_12153	3.00	lobChIP_FOXA1_robot
chr5	16721367	16721368	MACS_peak_12154	11.00	lobChIP_FOXA1_robot
chr5	16744906	16744907	MACS_peak_12155	8.00	lobChIP_FOXA1_robot
chr5	16758240	16758241	MACS_peak_12156	8.00	lobChIP_FOXA1_robot
chr5	16809901	16809902	MACS_peak_12157	4.00	lobChIP_FOXA1_robot
chr5	16881474	16881475	MACS_peak_12158	13.00	lobChIP_FOXA1_robot
chr5	16894590	16894591	MACS_peak_12159	4.00	lobChIP_FOXA1_robot
chr5	17169900	17169901	MACS_peak_12160	14.00	lobChIP_FOXA1_robot
chr5	17305338	17305339	MACS_peak_12161	3.00	lobChIP_FOXA1_robot
chr5	18913798	18913799	MACS_peak_12162	3.00	lobChIP_FOXA1_robot
chr5	19881046	19881047	MACS_peak_12163	62.00	lobChIP_FOXA1_robot
chr5	23670272	23670273	MACS_peak_12164	3.00	lobChIP_FOXA1_robot
chr5	28232040	28232041	MACS_peak_12165	5.00	lobChIP_FOXA1_robot
chr5	28308967	28308968	MACS_peak_12166	3.00	lobChIP_FOXA1_robot
chr5	28862944	28862945	MACS_peak_12167	4.00	lobChIP_FOXA1_robot
chr5	29759368	29759369	MACS_peak_12168	6.00	lobChIP_FOXA1_robot
chr5	29780687	29780688	MACS_peak_12169	5.00	lobChIP_FOXA1_robot
chr5	30505868	30505869	MACS_peak_12170	18.00	lobChIP_FOXA1_robot
chr5	30601261	30601262	MACS_peak_12171	6.00	lobChIP_FOXA1_robot
chr5	30605983	30605984	MACS_peak_12172	38.00	lobChIP_FOXA1_robot
chr5	32233946	32233947	MACS_peak_12173	10.00	lobChIP_FOXA1_robot
chr5	32268333	32268334	MACS_peak_12174	5.00	lobChIP_FOXA1_robot
chr5	32274294	32274295	MACS_peak_12175	5.00	lobChIP_FOXA1_robot
chr5	32275634	32275635	MACS_peak_12176	8.00	lobChIP_FOXA1_robot
chr5	32314939	32314940	MACS_peak_12177	4.00	lobChIP_FOXA1_robot
chr5	32331602	32331603	MACS_peak_12178	8.00	lobChIP_FOXA1_robot
chr5	32370689	32370690	MACS_peak_12179	4.00	lobChIP_FOXA1_robot
chr5	32531870	32531871	MACS_peak_12180	5.00	lobChIP_FOXA1_robot
chr5	32559746	32559747	MACS_peak_12181	5.00	lobChIP_FOXA1_robot
chr5	32574926	32574927	MACS_peak_12182	19.00	lobChIP_FOXA1_robot
chr5	34001575	34001576	MACS_peak_12183	5.00	lobChIP_FOXA1_robot
chr5	34036016	34036017	MACS_peak_12184	11.00	lobChIP_FOXA1_robot
chr5	34246198	34246199	MACS_peak_12185	12.00	lobChIP_FOXA1_robot
chr5	34485374	34485375	MACS_peak_12186	4.00	lobChIP_FOXA1_robot
chr5	34487645	34487646	MACS_peak_12187	11.00	lobChIP_FOXA1_robot
chr5	34585868	34585869	MACS_peak_12188	5.00	lobChIP_FOXA1_robot
chr5	34683576	34683577	MACS_peak_12189	7.00	lobChIP_FOXA1_robot
chr5	34686151	34686152	MACS_peak_12190	7.00	lobChIP_FOXA1_robot
chr5	34709715	34709716	MACS_peak_12191	3.00	lobChIP_FOXA1_robot
chr5	34731575	34731576	MACS_peak_12192	7.00	lobChIP_FOXA1_robot
chr5	34848474	34848475	MACS_peak_12193	4.00	lobChIP_FOXA1_robot
chr5	35116253	35116254	MACS_peak_12194	4.00	lobChIP_FOXA1_robot
chr5	35134603	35134604	MACS_peak_12195	7.00	lobChIP_FOXA1_robot
chr5	35159658	35159659	MACS_peak_12196	8.00	lobChIP_FOXA1_robot
chr5	35416631	35416632	MACS_peak_12197	3.00	lobChIP_FOXA1_robot
chr5	35421349	35421350	MACS_peak_12198	4.00	lobChIP_FOXA1_robot
chr5	35484017	35484018	MACS_peak_12199	11.00	lobChIP_FOXA1_robot
chr5	35741463	35741464	MACS_peak_12200	5.00	lobChIP_FOXA1_robot
chr5	35758460	35758461	MACS_peak_12201	4.00	lobChIP_FOXA1_robot
chr5	36154042	36154043	MACS_peak_12202	7.00	lobChIP_FOXA1_robot
chr5	36181063	36181064	MACS_peak_12203	26.00	lobChIP_FOXA1_robot
chr5	36239649	36239650	MACS_peak_12204	16.00	lobChIP_FOXA1_robot
chr5	36944867	36944868	MACS_peak_12205	12.00	lobChIP_FOXA1_robot
chr5	37086694	37086695	MACS_peak_12206	15.00	lobChIP_FOXA1_robot
chr5	37090596	37090597	MACS_peak_12207	21.00	lobChIP_FOXA1_robot
chr5	37094067	37094068	MACS_peak_12208	7.00	lobChIP_FOXA1_robot
chr5	37349373	37349374	MACS_peak_12209	25.00	lobChIP_FOXA1_robot
chr5	37668705	37668706	MACS_peak_12210	5.00	lobChIP_FOXA1_robot
chr5	37718220	37718221	MACS_peak_12211	13.00	lobChIP_FOXA1_robot
chr5	37832431	37832432	MACS_peak_12212	5.00	lobChIP_FOXA1_robot
chr5	38060633	38060634	MACS_peak_12213	10.00	lobChIP_FOXA1_robot
chr5	38104791	38104792	MACS_peak_12214	6.00	lobChIP_FOXA1_robot
chr5	38148253	38148254	MACS_peak_12215	4.00	lobChIP_FOXA1_robot
chr5	38442098	38442099	MACS_peak_12216	3.00	lobChIP_FOXA1_robot
chr5	38528404	38528405	MACS_peak_12217	6.00	lobChIP_FOXA1_robot
chr5	38533557	38533558	MACS_peak_12218	24.00	lobChIP_FOXA1_robot
chr5	38536209	38536210	MACS_peak_12219	6.00	lobChIP_FOXA1_robot
chr5	38547085	38547086	MACS_peak_12220	11.00	lobChIP_FOXA1_robot
chr5	38550175	38550176	MACS_peak_12221	13.00	lobChIP_FOXA1_robot
chr5	38559750	38559751	MACS_peak_12222	4.00	lobChIP_FOXA1_robot
chr5	38672635	38672636	MACS_peak_12223	7.00	lobChIP_FOXA1_robot
chr5	38783313	38783314	MACS_peak_12224	4.00	lobChIP_FOXA1_robot
chr5	38859153	38859154	MACS_peak_12225	9.00	lobChIP_FOXA1_robot
chr5	39284929	39284930	MACS_peak_12226	11.00	lobChIP_FOXA1_robot
chr5	39413321	39413322	MACS_peak_12227	4.00	lobChIP_FOXA1_robot
chr5	39437738	39437739	MACS_peak_12228	12.00	lobChIP_FOXA1_robot
chr5	39527420	39527421	MACS_peak_12229	11.00	lobChIP_FOXA1_robot
chr5	40447200	40447201	MACS_peak_12230	6.00	lobChIP_FOXA1_robot
chr5	40720369	40720370	MACS_peak_12231	8.00	lobChIP_FOXA1_robot
chr5	40784616	40784617	MACS_peak_12232	6.00	lobChIP_FOXA1_robot
chr5	40791915	40791916	MACS_peak_12233	4.00	lobChIP_FOXA1_robot
chr5	40841684	40841685	MACS_peak_12234	5.00	lobChIP_FOXA1_robot
chr5	41182599	41182600	MACS_peak_12235	3.00	lobChIP_FOXA1_robot
chr5	41638959	41638960	MACS_peak_12236	6.00	lobChIP_FOXA1_robot
chr5	41929252	41929253	MACS_peak_12237	6.00	lobChIP_FOXA1_robot
chr5	42108784	42108785	MACS_peak_12238	41.00	lobChIP_FOXA1_robot
chr5	42812064	42812065	MACS_peak_12239	13.00	lobChIP_FOXA1_robot
chr5	42855282	42855283	MACS_peak_12240	5.00	lobChIP_FOXA1_robot
chr5	42875142	42875143	MACS_peak_12241	8.00	lobChIP_FOXA1_robot
chr5	42881649	42881650	MACS_peak_12242	4.00	lobChIP_FOXA1_robot
chr5	42882373	42882374	MACS_peak_12243	4.00	lobChIP_FOXA1_robot
chr5	42941103	42941104	MACS_peak_12244	4.00	lobChIP_FOXA1_robot
chr5	42998164	42998165	MACS_peak_12245	4.00	lobChIP_FOXA1_robot
chr5	43422447	43422448	MACS_peak_12246	15.00	lobChIP_FOXA1_robot
chr5	43432868	43432869	MACS_peak_12247	22.00	lobChIP_FOXA1_robot
chr5	43476733	43476734	MACS_peak_12248	3.00	lobChIP_FOXA1_robot
chr5	43717509	43717510	MACS_peak_12249	4.00	lobChIP_FOXA1_robot
chr5	43997909	43997910	MACS_peak_12250	12.00	lobChIP_FOXA1_robot
chr5	44001937	44001938	MACS_peak_12251	4.00	lobChIP_FOXA1_robot
chr5	44002958	44002959	MACS_peak_12252	13.00	lobChIP_FOXA1_robot
chr5	45372781	45372782	MACS_peak_12253	4.00	lobChIP_FOXA1_robot
chr5	52003684	52003685	MACS_peak_12254	7.00	lobChIP_FOXA1_robot
chr5	52005506	52005507	MACS_peak_12255	5.00	lobChIP_FOXA1_robot
chr5	52090521	52090522	MACS_peak_12256	4.00	lobChIP_FOXA1_robot
chr5	52094243	52094244	MACS_peak_12257	4.00	lobChIP_FOXA1_robot
chr5	52161024	52161025	MACS_peak_12258	6.00	lobChIP_FOXA1_robot
chr5	52177549	52177550	MACS_peak_12259	5.00	lobChIP_FOXA1_robot
chr5	52293416	52293417	MACS_peak_12260	12.00	lobChIP_FOXA1_robot
chr5	52300856	52300857	MACS_peak_12261	4.00	lobChIP_FOXA1_robot
chr5	52315306	52315307	MACS_peak_12262	4.00	lobChIP_FOXA1_robot
chr5	52321695	52321696	MACS_peak_12263	3.00	lobChIP_FOXA1_robot
chr5	52409033	52409034	MACS_peak_12264	5.00	lobChIP_FOXA1_robot
chr5	52729339	52729340	MACS_peak_12265	5.00	lobChIP_FOXA1_robot
chr5	52749994	52749995	MACS_peak_12266	8.00	lobChIP_FOXA1_robot
chr5	52772992	52772993	MACS_peak_12267	24.00	lobChIP_FOXA1_robot
chr5	52783804	52783805	MACS_peak_12268	3.00	lobChIP_FOXA1_robot
chr5	53304393	53304394	MACS_peak_12269	15.00	lobChIP_FOXA1_robot
chr5	53602893	53602894	MACS_peak_12270	4.00	lobChIP_FOXA1_robot
chr5	54443294	54443295	MACS_peak_12271	5.00	lobChIP_FOXA1_robot
chr5	54845347	54845348	MACS_peak_12272	6.00	lobChIP_FOXA1_robot
chr5	54868147	54868148	MACS_peak_12273	5.00	lobChIP_FOXA1_robot
chr5	54872306	54872307	MACS_peak_12274	10.00	lobChIP_FOXA1_robot
chr5	54897216	54897217	MACS_peak_12275	7.00	lobChIP_FOXA1_robot
chr5	54917316	54917317	MACS_peak_12276	6.00	lobChIP_FOXA1_robot
chr5	54988494	54988495	MACS_peak_12277	5.00	lobChIP_FOXA1_robot
chr5	55230146	55230147	MACS_peak_12278	33.00	lobChIP_FOXA1_robot
chr5	55285027	55285028	MACS_peak_12279	16.00	lobChIP_FOXA1_robot
chr5	55321323	55321324	MACS_peak_12280	13.00	lobChIP_FOXA1_robot
chr5	55339672	55339673	MACS_peak_12281	5.00	lobChIP_FOXA1_robot
chr5	55385557	55385558	MACS_peak_12282	3.00	lobChIP_FOXA1_robot
chr5	55393947	55393948	MACS_peak_12283	7.00	lobChIP_FOXA1_robot
chr5	55419494	55419495	MACS_peak_12284	4.00	lobChIP_FOXA1_robot
chr5	55420041	55420042	MACS_peak_12285	6.00	lobChIP_FOXA1_robot
chr5	55498505	55498506	MACS_peak_12286	12.00	lobChIP_FOXA1_robot
chr5	55507393	55507394	MACS_peak_12287	4.00	lobChIP_FOXA1_robot
chr5	55508151	55508152	MACS_peak_12288	5.00	lobChIP_FOXA1_robot
chr5	55510984	55510985	MACS_peak_12289	6.00	lobChIP_FOXA1_robot
chr5	55576066	55576067	MACS_peak_12290	3.00	lobChIP_FOXA1_robot
chr5	55646637	55646638	MACS_peak_12291	4.00	lobChIP_FOXA1_robot
chr5	55654899	55654900	MACS_peak_12292	10.00	lobChIP_FOXA1_robot
chr5	55684250	55684251	MACS_peak_12293	19.00	lobChIP_FOXA1_robot
chr5	55737438	55737439	MACS_peak_12294	10.00	lobChIP_FOXA1_robot
chr5	55760996	55760997	MACS_peak_12295	8.00	lobChIP_FOXA1_robot
chr5	55776234	55776235	MACS_peak_12296	12.00	lobChIP_FOXA1_robot
chr5	55933387	55933388	MACS_peak_12297	7.00	lobChIP_FOXA1_robot
chr5	55934168	55934169	MACS_peak_12298	8.00	lobChIP_FOXA1_robot
chr5	56631156	56631157	MACS_peak_12299	4.00	lobChIP_FOXA1_robot
chr5	56659735	56659736	MACS_peak_12300	4.00	lobChIP_FOXA1_robot
chr5	56946413	56946414	MACS_peak_12301	5.00	lobChIP_FOXA1_robot
chr5	56989521	56989522	MACS_peak_12302	4.00	lobChIP_FOXA1_robot
chr5	57758600	57758601	MACS_peak_12303	4.00	lobChIP_FOXA1_robot
chr5	57893134	57893135	MACS_peak_12304	4.00	lobChIP_FOXA1_robot
chr5	58145995	58145996	MACS_peak_12305	5.00	lobChIP_FOXA1_robot
chr5	58166781	58166782	MACS_peak_12306	5.00	lobChIP_FOXA1_robot
chr5	58336876	58336877	MACS_peak_12307	5.00	lobChIP_FOXA1_robot
chr5	58357294	58357295	MACS_peak_12308	6.00	lobChIP_FOXA1_robot
chr5	58856605	58856606	MACS_peak_12309	4.00	lobChIP_FOXA1_robot
chr5	59052347	59052348	MACS_peak_12310	7.00	lobChIP_FOXA1_robot
chr5	59064710	59064711	MACS_peak_12311	6.00	lobChIP_FOXA1_robot
chr5	59713732	59713733	MACS_peak_12312	15.00	lobChIP_FOXA1_robot
chr5	59743447	59743448	MACS_peak_12313	48.00	lobChIP_FOXA1_robot
chr5	59759197	59759198	MACS_peak_12314	4.00	lobChIP_FOXA1_robot
chr5	59769693	59769694	MACS_peak_12315	5.00	lobChIP_FOXA1_robot
chr5	59838006	59838007	MACS_peak_12316	6.00	lobChIP_FOXA1_robot
chr5	59943685	59943686	MACS_peak_12317	5.00	lobChIP_FOXA1_robot
chr5	59967834	59967835	MACS_peak_12318	11.00	lobChIP_FOXA1_robot
chr5	59968878	59968879	MACS_peak_12319	13.00	lobChIP_FOXA1_robot
chr5	59983879	59983880	MACS_peak_12320	4.00	lobChIP_FOXA1_robot
chr5	59999997	59999998	MACS_peak_12321	5.00	lobChIP_FOXA1_robot
chr5	60106785	60106786	MACS_peak_12322	3.00	lobChIP_FOXA1_robot
chr5	60459107	60459108	MACS_peak_12323	5.00	lobChIP_FOXA1_robot
chr5	60600915	60600916	MACS_peak_12324	3.00	lobChIP_FOXA1_robot
chr5	61345163	61345164	MACS_peak_12325	4.00	lobChIP_FOXA1_robot
chr5	61618432	61618433	MACS_peak_12326	19.00	lobChIP_FOXA1_robot
chr5	61896744	61896745	MACS_peak_12327	4.00	lobChIP_FOXA1_robot
chr5	64114064	64114065	MACS_peak_12328	5.00	lobChIP_FOXA1_robot
chr5	64196606	64196607	MACS_peak_12329	5.00	lobChIP_FOXA1_robot
chr5	64235615	64235616	MACS_peak_12330	6.00	lobChIP_FOXA1_robot
chr5	64776570	64776571	MACS_peak_12331	4.00	lobChIP_FOXA1_robot
chr5	64927059	64927060	MACS_peak_12332	6.00	lobChIP_FOXA1_robot
chr5	64947188	64947189	MACS_peak_12333	4.00	lobChIP_FOXA1_robot
chr5	65226600	65226601	MACS_peak_12334	4.00	lobChIP_FOXA1_robot
chr5	66037439	66037440	MACS_peak_12335	9.00	lobChIP_FOXA1_robot
chr5	66255625	66255626	MACS_peak_12336	7.00	lobChIP_FOXA1_robot
chr5	66290826	66290827	MACS_peak_12337	5.00	lobChIP_FOXA1_robot
chr5	66391297	66391298	MACS_peak_12338	8.00	lobChIP_FOXA1_robot
chr5	66440348	66440349	MACS_peak_12339	3.00	lobChIP_FOXA1_robot
chr5	66531743	66531744	MACS_peak_12340	7.00	lobChIP_FOXA1_robot
chr5	67066909	67066910	MACS_peak_12341	4.00	lobChIP_FOXA1_robot
chr5	67376557	67376558	MACS_peak_12342	5.00	lobChIP_FOXA1_robot
chr5	67535431	67535432	MACS_peak_12343	4.00	lobChIP_FOXA1_robot
chr5	68346332	68346333	MACS_peak_12344	5.00	lobChIP_FOXA1_robot
chr5	68627975	68627976	MACS_peak_12345	8.00	lobChIP_FOXA1_robot
chr5	68740078	68740079	MACS_peak_12346	4.00	lobChIP_FOXA1_robot
chr5	68748315	68748316	MACS_peak_12347	15.00	lobChIP_FOXA1_robot
chr5	68768525	68768526	MACS_peak_12348	11.00	lobChIP_FOXA1_robot
chr5	70778183	70778184	MACS_peak_12349	8.00	lobChIP_FOXA1_robot
chr5	70779414	70779415	MACS_peak_12350	6.00	lobChIP_FOXA1_robot
chr5	71537618	71537619	MACS_peak_12352	5.00	lobChIP_FOXA1_robot
chr5	71591634	71591635	MACS_peak_12353	7.00	lobChIP_FOXA1_robot
chr5	72251883	72251884	MACS_peak_12354	12.00	lobChIP_FOXA1_robot
chr5	72318526	72318527	MACS_peak_12355	5.00	lobChIP_FOXA1_robot
chr5	72821746	72821747	MACS_peak_12356	5.00	lobChIP_FOXA1_robot
chr5	72837358	72837359	MACS_peak_12357	3.00	lobChIP_FOXA1_robot
chr5	72840611	72840612	MACS_peak_12358	22.00	lobChIP_FOXA1_robot
chr5	72901313	72901314	MACS_peak_12359	5.00	lobChIP_FOXA1_robot
chr5	72919919	72919920	MACS_peak_12360	38.00	lobChIP_FOXA1_robot
chr5	72959468	72959469	MACS_peak_12361	4.00	lobChIP_FOXA1_robot
chr5	72959904	72959905	MACS_peak_12362	4.00	lobChIP_FOXA1_robot
chr5	72968985	72968986	MACS_peak_12363	5.00	lobChIP_FOXA1_robot
chr5	72974444	72974445	MACS_peak_12364	8.00	lobChIP_FOXA1_robot
chr5	73025291	73025292	MACS_peak_12365	25.00	lobChIP_FOXA1_robot
chr5	73060639	73060640	MACS_peak_12366	10.00	lobChIP_FOXA1_robot
chr5	73066418	73066419	MACS_peak_12367	3.00	lobChIP_FOXA1_robot
chr5	73088761	73088762	MACS_peak_12368	9.00	lobChIP_FOXA1_robot
chr5	73092679	73092680	MACS_peak_12369	5.00	lobChIP_FOXA1_robot
chr5	73118959	73118960	MACS_peak_12370	6.00	lobChIP_FOXA1_robot
chr5	73119406	73119407	MACS_peak_12371	4.00	lobChIP_FOXA1_robot
chr5	73157560	73157561	MACS_peak_12372	4.00	lobChIP_FOXA1_robot
chr5	73254179	73254180	MACS_peak_12373	5.00	lobChIP_FOXA1_robot
chr5	73281189	73281190	MACS_peak_12374	5.00	lobChIP_FOXA1_robot
chr5	73359866	73359867	MACS_peak_12375	5.00	lobChIP_FOXA1_robot
chr5	73400507	73400508	MACS_peak_12376	5.00	lobChIP_FOXA1_robot
chr5	73704261	73704262	MACS_peak_12377	10.00	lobChIP_FOXA1_robot
chr5	73711838	73711839	MACS_peak_12378	6.00	lobChIP_FOXA1_robot
chr5	73720571	73720572	MACS_peak_12379	5.00	lobChIP_FOXA1_robot
chr5	73796290	73796291	MACS_peak_12380	4.00	lobChIP_FOXA1_robot
chr5	73798101	73798102	MACS_peak_12381	5.00	lobChIP_FOXA1_robot
chr5	73811391	73811392	MACS_peak_12382	3.00	lobChIP_FOXA1_robot
chr5	73873163	73873164	MACS_peak_12383	5.00	lobChIP_FOXA1_robot
chr5	73877004	73877005	MACS_peak_12384	17.00	lobChIP_FOXA1_robot
chr5	73934235	73934236	MACS_peak_12385	5.00	lobChIP_FOXA1_robot
chr5	74129618	74129619	MACS_peak_12386	4.00	lobChIP_FOXA1_robot
chr5	74169996	74169997	MACS_peak_12387	7.00	lobChIP_FOXA1_robot
chr5	74172324	74172325	MACS_peak_12388	19.00	lobChIP_FOXA1_robot
chr5	74173581	74173582	MACS_peak_12389	8.00	lobChIP_FOXA1_robot
chr5	74212796	74212797	MACS_peak_12390	12.00	lobChIP_FOXA1_robot
chr5	74214168	74214169	MACS_peak_12391	6.00	lobChIP_FOXA1_robot
chr5	74242304	74242305	MACS_peak_12392	3.00	lobChIP_FOXA1_robot
chr5	74250373	74250374	MACS_peak_12393	11.00	lobChIP_FOXA1_robot
chr5	74306877	74306878	MACS_peak_12394	7.00	lobChIP_FOXA1_robot
chr5	74627404	74627405	MACS_peak_12395	14.00	lobChIP_FOXA1_robot
chr5	74819342	74819343	MACS_peak_12396	22.00	lobChIP_FOXA1_robot
chr5	75595010	75595011	MACS_peak_12397	4.00	lobChIP_FOXA1_robot
chr5	75675364	75675365	MACS_peak_12398	4.00	lobChIP_FOXA1_robot
chr5	75685781	75685782	MACS_peak_12399	4.00	lobChIP_FOXA1_robot
chr5	75700166	75700167	MACS_peak_12400	3.00	lobChIP_FOXA1_robot
chr5	75753936	75753937	MACS_peak_12401	4.00	lobChIP_FOXA1_robot
chr5	75768663	75768664	MACS_peak_12402	6.00	lobChIP_FOXA1_robot
chr5	75775952	75775953	MACS_peak_12403	7.00	lobChIP_FOXA1_robot
chr5	75789594	75789595	MACS_peak_12404	11.00	lobChIP_FOXA1_robot
chr5	75833037	75833038	MACS_peak_12405	5.00	lobChIP_FOXA1_robot
chr5	75844806	75844807	MACS_peak_12406	5.00	lobChIP_FOXA1_robot
chr5	76067770	76067771	MACS_peak_12407	5.00	lobChIP_FOXA1_robot
chr5	76101948	76101949	MACS_peak_12408	17.00	lobChIP_FOXA1_robot
chr5	76116534	76116535	MACS_peak_12409	5.00	lobChIP_FOXA1_robot
chr5	76483112	76483113	MACS_peak_12410	5.00	lobChIP_FOXA1_robot
chr5	77570885	77570886	MACS_peak_12411	7.00	lobChIP_FOXA1_robot
chr5	77589557	77589558	MACS_peak_12412	5.00	lobChIP_FOXA1_robot
chr5	77716554	77716555	MACS_peak_12413	6.00	lobChIP_FOXA1_robot
chr5	77842649	77842650	MACS_peak_12414	6.00	lobChIP_FOXA1_robot
chr5	77853007	77853008	MACS_peak_12415	4.00	lobChIP_FOXA1_robot
chr5	77884281	77884282	MACS_peak_12416	10.00	lobChIP_FOXA1_robot
chr5	77898015	77898016	MACS_peak_12417	11.00	lobChIP_FOXA1_robot
chr5	78017081	78017082	MACS_peak_12418	4.00	lobChIP_FOXA1_robot
chr5	78054176	78054177	MACS_peak_12419	9.00	lobChIP_FOXA1_robot
chr5	78254541	78254542	MACS_peak_12420	8.00	lobChIP_FOXA1_robot
chr5	78277595	78277596	MACS_peak_12421	10.00	lobChIP_FOXA1_robot
chr5	78281774	78281775	MACS_peak_12422	15.00	lobChIP_FOXA1_robot
chr5	78298369	78298370	MACS_peak_12423	6.00	lobChIP_FOXA1_robot
chr5	78319963	78319964	MACS_peak_12424	16.00	lobChIP_FOXA1_robot
chr5	78326689	78326690	MACS_peak_12425	11.00	lobChIP_FOXA1_robot
chr5	78469685	78469686	MACS_peak_12426	9.00	lobChIP_FOXA1_robot
chr5	78566460	78566461	MACS_peak_12427	9.00	lobChIP_FOXA1_robot
chr5	78716419	78716420	MACS_peak_12428	6.00	lobChIP_FOXA1_robot
chr5	78831601	78831602	MACS_peak_12429	25.00	lobChIP_FOXA1_robot
chr5	78842983	78842984	MACS_peak_12430	3.00	lobChIP_FOXA1_robot
chr5	78877454	78877455	MACS_peak_12431	9.00	lobChIP_FOXA1_robot
chr5	79379561	79379562	MACS_peak_12432	11.00	lobChIP_FOXA1_robot
chr5	79461411	79461412	MACS_peak_12433	4.00	lobChIP_FOXA1_robot
chr5	79477681	79477682	MACS_peak_12434	5.00	lobChIP_FOXA1_robot
chr5	79480326	79480327	MACS_peak_12435	19.00	lobChIP_FOXA1_robot
chr5	79500892	79500893	MACS_peak_12436	16.00	lobChIP_FOXA1_robot
chr5	79525322	79525323	MACS_peak_12437	37.00	lobChIP_FOXA1_robot
chr5	79770886	79770887	MACS_peak_12438	4.00	lobChIP_FOXA1_robot
chr5	79896160	79896161	MACS_peak_12439	5.00	lobChIP_FOXA1_robot
chr5	80252991	80252992	MACS_peak_12440	13.00	lobChIP_FOXA1_robot
chr5	80260480	80260481	MACS_peak_12441	4.00	lobChIP_FOXA1_robot
chr5	80261926	80261927	MACS_peak_12442	4.00	lobChIP_FOXA1_robot
chr5	80263534	80263535	MACS_peak_12443	10.00	lobChIP_FOXA1_robot
chr5	80270912	80270913	MACS_peak_12444	3.00	lobChIP_FOXA1_robot
chr5	80283336	80283337	MACS_peak_12445	5.00	lobChIP_FOXA1_robot
chr5	80341219	80341220	MACS_peak_12446	5.00	lobChIP_FOXA1_robot
chr5	80667520	80667521	MACS_peak_12447	12.00	lobChIP_FOXA1_robot
chr5	80747007	80747008	MACS_peak_12448	5.00	lobChIP_FOXA1_robot
chr5	81653117	81653118	MACS_peak_12449	4.00	lobChIP_FOXA1_robot
chr5	81671560	81671561	MACS_peak_12450	7.00	lobChIP_FOXA1_robot
chr5	81683329	81683330	MACS_peak_12451	59.00	lobChIP_FOXA1_robot
chr5	81931715	81931716	MACS_peak_12452	16.00	lobChIP_FOXA1_robot
chr5	82003751	82003752	MACS_peak_12453	4.00	lobChIP_FOXA1_robot
chr5	82272872	82272873	MACS_peak_12454	5.00	lobChIP_FOXA1_robot
chr5	82381091	82381092	MACS_peak_12455	16.00	lobChIP_FOXA1_robot
chr5	82420644	82420645	MACS_peak_12456	28.00	lobChIP_FOXA1_robot
chr5	82561201	82561202	MACS_peak_12457	5.00	lobChIP_FOXA1_robot
chr5	82588744	82588745	MACS_peak_12458	3.00	lobChIP_FOXA1_robot
chr5	82766529	82766530	MACS_peak_12459	6.00	lobChIP_FOXA1_robot
chr5	82854996	82854997	MACS_peak_12460	6.00	lobChIP_FOXA1_robot
chr5	82859615	82859616	MACS_peak_12461	6.00	lobChIP_FOXA1_robot
chr5	82873033	82873034	MACS_peak_12462	10.00	lobChIP_FOXA1_robot
chr5	83387448	83387449	MACS_peak_12463	48.00	lobChIP_FOXA1_robot
chr5	84966413	84966414	MACS_peak_12464	5.00	lobChIP_FOXA1_robot
chr5	85076256	85076257	MACS_peak_12465	4.00	lobChIP_FOXA1_robot
chr5	85206484	85206485	MACS_peak_12466	4.00	lobChIP_FOXA1_robot
chr5	89605151	89605152	MACS_peak_12467	7.00	lobChIP_FOXA1_robot
chr5	89844073	89844074	MACS_peak_12468	6.00	lobChIP_FOXA1_robot
chr5	90162865	90162866	MACS_peak_12469	12.00	lobChIP_FOXA1_robot
chr5	90195167	90195168	MACS_peak_12470	5.00	lobChIP_FOXA1_robot
chr5	90207173	90207174	MACS_peak_12471	18.00	lobChIP_FOXA1_robot
chr5	90211338	90211339	MACS_peak_12472	6.00	lobChIP_FOXA1_robot
chr5	90245777	90245778	MACS_peak_12473	12.00	lobChIP_FOXA1_robot
chr5	90323568	90323569	MACS_peak_12474	6.00	lobChIP_FOXA1_robot
chr5	91888634	91888635	MACS_peak_12475	4.00	lobChIP_FOXA1_robot
chr5	91977264	91977265	MACS_peak_12476	3.00	lobChIP_FOXA1_robot
chr5	92223189	92223190	MACS_peak_12477	9.00	lobChIP_FOXA1_robot
chr5	92243870	92243871	MACS_peak_12478	6.00	lobChIP_FOXA1_robot
chr5	92251463	92251464	MACS_peak_12479	4.00	lobChIP_FOXA1_robot
chr5	92263113	92263114	MACS_peak_12480	14.00	lobChIP_FOXA1_robot
chr5	92278703	92278704	MACS_peak_12481	9.00	lobChIP_FOXA1_robot
chr5	92280965	92280966	MACS_peak_12482	52.00	lobChIP_FOXA1_robot
chr5	92305689	92305690	MACS_peak_12483	23.00	lobChIP_FOXA1_robot
chr5	92334442	92334443	MACS_peak_12484	5.00	lobChIP_FOXA1_robot
chr5	92389603	92389604	MACS_peak_12485	6.00	lobChIP_FOXA1_robot
chr5	92414913	92414914	MACS_peak_12486	6.00	lobChIP_FOXA1_robot
chr5	92528021	92528022	MACS_peak_12487	7.00	lobChIP_FOXA1_robot
chr5	92582209	92582210	MACS_peak_12488	37.00	lobChIP_FOXA1_robot
chr5	93238090	93238091	MACS_peak_12489	5.00	lobChIP_FOXA1_robot
chr5	93250959	93250960	MACS_peak_12490	4.00	lobChIP_FOXA1_robot
chr5	94598750	94598751	MACS_peak_12491	6.00	lobChIP_FOXA1_robot
chr5	95066544	95066545	MACS_peak_12492	4.00	lobChIP_FOXA1_robot
chr5	95083942	95083943	MACS_peak_12493	3.00	lobChIP_FOXA1_robot
chr5	95095610	95095611	MACS_peak_12494	13.00	lobChIP_FOXA1_robot
chr5	95193193	95193194	MACS_peak_12495	10.00	lobChIP_FOXA1_robot
chr5	95197281	95197282	MACS_peak_12496	5.00	lobChIP_FOXA1_robot
chr5	95198544	95198545	MACS_peak_12497	17.00	lobChIP_FOXA1_robot
chr5	95200031	95200032	MACS_peak_12498	4.00	lobChIP_FOXA1_robot
chr5	95214888	95214889	MACS_peak_12499	18.00	lobChIP_FOXA1_robot
chr5	95223913	95223914	MACS_peak_12500	8.00	lobChIP_FOXA1_robot
chr5	95244466	95244467	MACS_peak_12501	7.00	lobChIP_FOXA1_robot
chr5	95260639	95260640	MACS_peak_12502	20.00	lobChIP_FOXA1_robot
chr5	95292764	95292765	MACS_peak_12503	3.00	lobChIP_FOXA1_robot
chr5	95298022	95298023	MACS_peak_12504	4.00	lobChIP_FOXA1_robot
chr5	95301851	95301852	MACS_peak_12505	19.00	lobChIP_FOXA1_robot
chr5	95306078	95306079	MACS_peak_12506	6.00	lobChIP_FOXA1_robot
chr5	95322925	95322926	MACS_peak_12507	22.00	lobChIP_FOXA1_robot
chr5	95323915	95323916	MACS_peak_12508	13.00	lobChIP_FOXA1_robot
chr5	95324806	95324807	MACS_peak_12509	5.00	lobChIP_FOXA1_robot
chr5	95325210	95325211	MACS_peak_12510	13.00	lobChIP_FOXA1_robot
chr5	95333192	95333193	MACS_peak_12511	5.00	lobChIP_FOXA1_robot
chr5	95337264	95337265	MACS_peak_12512	5.00	lobChIP_FOXA1_robot
chr5	95429849	95429850	MACS_peak_12513	6.00	lobChIP_FOXA1_robot
chr5	95453858	95453859	MACS_peak_12514	9.00	lobChIP_FOXA1_robot
chr5	95459344	95459345	MACS_peak_12515	9.00	lobChIP_FOXA1_robot
chr5	95460343	95460344	MACS_peak_12516	7.00	lobChIP_FOXA1_robot
chr5	95461293	95461294	MACS_peak_12517	9.00	lobChIP_FOXA1_robot
chr5	95523370	95523371	MACS_peak_12518	17.00	lobChIP_FOXA1_robot
chr5	95525211	95525212	MACS_peak_12519	4.00	lobChIP_FOXA1_robot
chr5	95532028	95532029	MACS_peak_12520	4.00	lobChIP_FOXA1_robot
chr5	95535608	95535609	MACS_peak_12521	5.00	lobChIP_FOXA1_robot
chr5	95540992	95540993	MACS_peak_12522	4.00	lobChIP_FOXA1_robot
chr5	95581311	95581312	MACS_peak_12523	32.00	lobChIP_FOXA1_robot
chr5	95615730	95615731	MACS_peak_12524	4.00	lobChIP_FOXA1_robot
chr5	95633808	95633809	MACS_peak_12525	27.00	lobChIP_FOXA1_robot
chr5	95642903	95642904	MACS_peak_12526	4.00	lobChIP_FOXA1_robot
chr5	96117495	96117496	MACS_peak_12527	6.00	lobChIP_FOXA1_robot
chr5	97840473	97840474	MACS_peak_12528	4.00	lobChIP_FOXA1_robot
chr5	98104238	98104239	MACS_peak_12529	4.00	lobChIP_FOXA1_robot
chr5	98274326	98274327	MACS_peak_12530	3.00	lobChIP_FOXA1_robot
chr5	98381801	98381802	MACS_peak_12531	4.00	lobChIP_FOXA1_robot
chr5	98396338	98396339	MACS_peak_12532	4.00	lobChIP_FOXA1_robot
chr5	99057432	99057433	MACS_peak_12533	18.00	lobChIP_FOXA1_robot
chr5	101416225	101416226	MACS_peak_12534	8.00	lobChIP_FOXA1_robot
chr5	101552616	101552617	MACS_peak_12535	5.00	lobChIP_FOXA1_robot
chr5	101558707	101558708	MACS_peak_12536	50.00	lobChIP_FOXA1_robot
chr5	101559674	101559675	MACS_peak_12537	4.00	lobChIP_FOXA1_robot
chr5	102106677	102106678	MACS_peak_12538	4.00	lobChIP_FOXA1_robot
chr5	102131856	102131857	MACS_peak_12539	3.00	lobChIP_FOXA1_robot
chr5	102350057	102350058	MACS_peak_12540	4.00	lobChIP_FOXA1_robot
chr5	103534056	103534057	MACS_peak_12541	4.00	lobChIP_FOXA1_robot
chr5	104039287	104039288	MACS_peak_12542	4.00	lobChIP_FOXA1_robot
chr5	105153977	105153978	MACS_peak_12543	4.00	lobChIP_FOXA1_robot
chr5	105658780	105658781	MACS_peak_12544	6.00	lobChIP_FOXA1_robot
chr5	107272459	107272460	MACS_peak_12545	6.00	lobChIP_FOXA1_robot
chr5	108083448	108083449	MACS_peak_12546	4.00	lobChIP_FOXA1_robot
chr5	108706016	108706017	MACS_peak_12547	4.00	lobChIP_FOXA1_robot
chr5	108746278	108746279	MACS_peak_12548	5.00	lobChIP_FOXA1_robot
chr5	108804606	108804607	MACS_peak_12549	5.00	lobChIP_FOXA1_robot
chr5	109043411	109043412	MACS_peak_12550	8.00	lobChIP_FOXA1_robot
chr5	109237275	109237276	MACS_peak_12551	20.00	lobChIP_FOXA1_robot
chr5	109335994	109335995	MACS_peak_12552	5.00	lobChIP_FOXA1_robot
chr5	110603760	110603761	MACS_peak_12553	4.00	lobChIP_FOXA1_robot
chr5	110740175	110740176	MACS_peak_12554	4.00	lobChIP_FOXA1_robot
chr5	111066865	111066866	MACS_peak_12555	15.00	lobChIP_FOXA1_robot
chr5	111070455	111070456	MACS_peak_12556	8.00	lobChIP_FOXA1_robot
chr5	111078514	111078515	MACS_peak_12557	5.00	lobChIP_FOXA1_robot
chr5	111090055	111090056	MACS_peak_12558	4.00	lobChIP_FOXA1_robot
chr5	112294372	112294373	MACS_peak_12559	6.00	lobChIP_FOXA1_robot
chr5	112304619	112304620	MACS_peak_12560	5.00	lobChIP_FOXA1_robot
chr5	112314229	112314230	MACS_peak_12561	8.00	lobChIP_FOXA1_robot
chr5	112356345	112356346	MACS_peak_12562	6.00	lobChIP_FOXA1_robot
chr5	112635412	112635413	MACS_peak_12563	4.00	lobChIP_FOXA1_robot
chr5	113271345	113271346	MACS_peak_12564	18.00	lobChIP_FOXA1_robot
chr5	114877499	114877500	MACS_peak_12565	4.00	lobChIP_FOXA1_robot
chr5	114878046	114878047	MACS_peak_12566	3.00	lobChIP_FOXA1_robot
chr5	114879257	114879258	MACS_peak_12567	4.00	lobChIP_FOXA1_robot
chr5	115165875	115165876	MACS_peak_12568	10.00	lobChIP_FOXA1_robot
chr5	115167140	115167141	MACS_peak_12569	9.00	lobChIP_FOXA1_robot
chr5	115860796	115860797	MACS_peak_12570	20.00	lobChIP_FOXA1_robot
chr5	115868839	115868840	MACS_peak_12571	36.00	lobChIP_FOXA1_robot
chr5	115988118	115988119	MACS_peak_12572	4.00	lobChIP_FOXA1_robot
chr5	116194913	116194914	MACS_peak_12573	5.00	lobChIP_FOXA1_robot
chr5	116198878	116198879	MACS_peak_12574	19.00	lobChIP_FOXA1_robot
chr5	116200219	116200220	MACS_peak_12575	27.00	lobChIP_FOXA1_robot
chr5	116217964	116217965	MACS_peak_12576	5.00	lobChIP_FOXA1_robot
chr5	117879783	117879784	MACS_peak_12577	7.00	lobChIP_FOXA1_robot
chr5	118345007	118345008	MACS_peak_12578	4.00	lobChIP_FOXA1_robot
chr5	118638863	118638864	MACS_peak_12579	8.00	lobChIP_FOXA1_robot
chr5	118724427	118724428	MACS_peak_12580	5.00	lobChIP_FOXA1_robot
chr5	118858801	118858802	MACS_peak_12581	6.00	lobChIP_FOXA1_robot
chr5	118860424	118860425	MACS_peak_12582	6.00	lobChIP_FOXA1_robot
chr5	119188022	119188023	MACS_peak_12583	8.00	lobChIP_FOXA1_robot
chr5	121411898	121411899	MACS_peak_12584	7.00	lobChIP_FOXA1_robot
chr5	121851934	121851935	MACS_peak_12585	3.00	lobChIP_FOXA1_robot
chr5	122128741	122128742	MACS_peak_12586	4.00	lobChIP_FOXA1_robot
chr5	122251708	122251709	MACS_peak_12587	5.00	lobChIP_FOXA1_robot
chr5	122252350	122252351	MACS_peak_12588	6.00	lobChIP_FOXA1_robot
chr5	123085952	123085953	MACS_peak_12589	3.00	lobChIP_FOXA1_robot
chr5	123499015	123499016	MACS_peak_12590	5.00	lobChIP_FOXA1_robot
chr5	123917026	123917027	MACS_peak_12591	7.00	lobChIP_FOXA1_robot
chr5	123932784	123932785	MACS_peak_12592	14.00	lobChIP_FOXA1_robot
chr5	123934905	123934906	MACS_peak_12593	7.00	lobChIP_FOXA1_robot
chr5	123935541	123935542	MACS_peak_12594	47.00	lobChIP_FOXA1_robot
chr5	123970033	123970034	MACS_peak_12595	6.00	lobChIP_FOXA1_robot
chr5	123988287	123988288	MACS_peak_12596	6.00	lobChIP_FOXA1_robot
chr5	123991824	123991825	MACS_peak_12597	20.00	lobChIP_FOXA1_robot
chr5	123992539	123992540	MACS_peak_12598	4.00	lobChIP_FOXA1_robot
chr5	123997446	123997447	MACS_peak_12599	69.00	lobChIP_FOXA1_robot
chr5	123998905	123998906	MACS_peak_12600	7.00	lobChIP_FOXA1_robot
chr5	124012199	124012200	MACS_peak_12601	7.00	lobChIP_FOXA1_robot
chr5	124019788	124019789	MACS_peak_12602	6.00	lobChIP_FOXA1_robot
chr5	124085198	124085199	MACS_peak_12603	10.00	lobChIP_FOXA1_robot
chr5	124100825	124100826	MACS_peak_12604	11.00	lobChIP_FOXA1_robot
chr5	125701387	125701388	MACS_peak_12605	3.00	lobChIP_FOXA1_robot
chr5	125706086	125706087	MACS_peak_12606	3.00	lobChIP_FOXA1_robot
chr5	125875012	125875013	MACS_peak_12607	10.00	lobChIP_FOXA1_robot
chr5	125984966	125984967	MACS_peak_12608	7.00	lobChIP_FOXA1_robot
chr5	125992303	125992304	MACS_peak_12609	6.00	lobChIP_FOXA1_robot
chr5	126340034	126340035	MACS_peak_12610	3.00	lobChIP_FOXA1_robot
chr5	126358751	126358752	MACS_peak_12611	8.00	lobChIP_FOXA1_robot
chr5	126433320	126433321	MACS_peak_12612	5.00	lobChIP_FOXA1_robot
chr5	126542223	126542224	MACS_peak_12613	4.00	lobChIP_FOXA1_robot
chr5	126647059	126647060	MACS_peak_12614	3.00	lobChIP_FOXA1_robot
chr5	127270778	127270779	MACS_peak_12615	21.00	lobChIP_FOXA1_robot
chr5	127294474	127294475	MACS_peak_12616	14.00	lobChIP_FOXA1_robot
chr5	127309209	127309210	MACS_peak_12617	5.00	lobChIP_FOXA1_robot
chr5	128847442	128847443	MACS_peak_12618	3.00	lobChIP_FOXA1_robot
chr5	129727495	129727496	MACS_peak_12619	5.00	lobChIP_FOXA1_robot
chr5	130598438	130598439	MACS_peak_12620	5.00	lobChIP_FOXA1_robot
chr5	130715585	130715586	MACS_peak_12621	5.00	lobChIP_FOXA1_robot
chr5	130722623	130722624	MACS_peak_12622	8.00	lobChIP_FOXA1_robot
chr5	130786754	130786755	MACS_peak_12623	4.00	lobChIP_FOXA1_robot
chr5	130809530	130809531	MACS_peak_12624	6.00	lobChIP_FOXA1_robot
chr5	130946610	130946611	MACS_peak_12625	8.00	lobChIP_FOXA1_robot
chr5	130966512	130966513	MACS_peak_12626	7.00	lobChIP_FOXA1_robot
chr5	131332660	131332661	MACS_peak_12627	9.00	lobChIP_FOXA1_robot
chr5	131337431	131337432	MACS_peak_12628	3.00	lobChIP_FOXA1_robot
chr5	132157756	132157757	MACS_peak_12629	6.00	lobChIP_FOXA1_robot
chr5	132175568	132175569	MACS_peak_12630	6.00	lobChIP_FOXA1_robot
chr5	132208910	132208911	MACS_peak_12631	3.00	lobChIP_FOXA1_robot
chr5	132241893	132241894	MACS_peak_12632	12.00	lobChIP_FOXA1_robot
chr5	132368687	132368688	MACS_peak_12633	5.00	lobChIP_FOXA1_robot
chr5	132376465	132376466	MACS_peak_12634	5.00	lobChIP_FOXA1_robot
chr5	132385476	132385477	MACS_peak_12635	35.00	lobChIP_FOXA1_robot
chr5	132386519	132386520	MACS_peak_12636	3.00	lobChIP_FOXA1_robot
chr5	132592144	132592145	MACS_peak_12637	4.00	lobChIP_FOXA1_robot
chr5	132755585	132755586	MACS_peak_12638	4.00	lobChIP_FOXA1_robot
chr5	133305848	133305849	MACS_peak_12639	11.00	lobChIP_FOXA1_robot
chr5	133373985	133373986	MACS_peak_12640	5.00	lobChIP_FOXA1_robot
chr5	133415540	133415541	MACS_peak_12641	6.00	lobChIP_FOXA1_robot
chr5	133434483	133434484	MACS_peak_12642	6.00	lobChIP_FOXA1_robot
chr5	133485409	133485410	MACS_peak_12643	6.00	lobChIP_FOXA1_robot
chr5	133578004	133578005	MACS_peak_12644	5.00	lobChIP_FOXA1_robot
chr5	133581190	133581191	MACS_peak_12645	7.00	lobChIP_FOXA1_robot
chr5	133751740	133751741	MACS_peak_12646	7.00	lobChIP_FOXA1_robot
chr5	133862611	133862612	MACS_peak_12647	8.00	lobChIP_FOXA1_robot
chr5	134258248	134258249	MACS_peak_12648	5.00	lobChIP_FOXA1_robot
chr5	134300507	134300508	MACS_peak_12649	7.00	lobChIP_FOXA1_robot
chr5	134389864	134389865	MACS_peak_12650	6.00	lobChIP_FOXA1_robot
chr5	134481856	134481857	MACS_peak_12651	5.00	lobChIP_FOXA1_robot
chr5	134492712	134492713	MACS_peak_12652	11.00	lobChIP_FOXA1_robot
chr5	134493399	134493400	MACS_peak_12653	5.00	lobChIP_FOXA1_robot
chr5	134494948	134494949	MACS_peak_12654	6.00	lobChIP_FOXA1_robot
chr5	134598902	134598903	MACS_peak_12655	5.00	lobChIP_FOXA1_robot
chr5	134818302	134818303	MACS_peak_12656	4.00	lobChIP_FOXA1_robot
chr5	134825613	134825614	MACS_peak_12657	6.00	lobChIP_FOXA1_robot
chr5	135143432	135143433	MACS_peak_12658	5.00	lobChIP_FOXA1_robot
chr5	135309200	135309201	MACS_peak_12659	11.00	lobChIP_FOXA1_robot
chr5	135310142	135310143	MACS_peak_12660	5.00	lobChIP_FOXA1_robot
chr5	135389563	135389564	MACS_peak_12661	20.00	lobChIP_FOXA1_robot
chr5	135394382	135394383	MACS_peak_12662	23.00	lobChIP_FOXA1_robot
chr5	135459251	135459252	MACS_peak_12663	7.00	lobChIP_FOXA1_robot
chr5	135485814	135485815	MACS_peak_12664	14.00	lobChIP_FOXA1_robot
chr5	136550497	136550498	MACS_peak_12665	5.00	lobChIP_FOXA1_robot
chr5	137087078	137087079	MACS_peak_12666	4.00	lobChIP_FOXA1_robot
chr5	137549360	137549361	MACS_peak_12667	4.00	lobChIP_FOXA1_robot
chr5	137607183	137607184	MACS_peak_12668	5.00	lobChIP_FOXA1_robot
chr5	137623510	137623511	MACS_peak_12669	5.00	lobChIP_FOXA1_robot
chr5	137673598	137673599	MACS_peak_12670	9.00	lobChIP_FOXA1_robot
chr5	137842099	137842100	MACS_peak_12671	3.00	lobChIP_FOXA1_robot
chr5	137843303	137843304	MACS_peak_12672	7.00	lobChIP_FOXA1_robot
chr5	137864115	137864116	MACS_peak_12673	3.00	lobChIP_FOXA1_robot
chr5	138106871	138106872	MACS_peak_12674	6.00	lobChIP_FOXA1_robot
chr5	138204948	138204949	MACS_peak_12675	8.00	lobChIP_FOXA1_robot
chr5	138299875	138299876	MACS_peak_12676	4.00	lobChIP_FOXA1_robot
chr5	138632046	138632047	MACS_peak_12677	5.00	lobChIP_FOXA1_robot
chr5	139409694	139409695	MACS_peak_12678	6.00	lobChIP_FOXA1_robot
chr5	139641008	139641009	MACS_peak_12679	5.00	lobChIP_FOXA1_robot
chr5	139673050	139673051	MACS_peak_12680	3.00	lobChIP_FOXA1_robot
chr5	139703785	139703786	MACS_peak_12681	4.00	lobChIP_FOXA1_robot
chr5	139707380	139707381	MACS_peak_12682	4.00	lobChIP_FOXA1_robot
chr5	139788770	139788771	MACS_peak_12683	4.00	lobChIP_FOXA1_robot
chr5	139986682	139986683	MACS_peak_12684	8.00	lobChIP_FOXA1_robot
chr5	139990115	139990116	MACS_peak_12685	4.00	lobChIP_FOXA1_robot
chr5	140016928	140016929	MACS_peak_12686	3.00	lobChIP_FOXA1_robot
chr5	140969855	140969856	MACS_peak_12687	4.00	lobChIP_FOXA1_robot
chr5	141545868	141545869	MACS_peak_12688	5.00	lobChIP_FOXA1_robot
chr5	141549397	141549398	MACS_peak_12689	5.00	lobChIP_FOXA1_robot
chr5	141595572	141595573	MACS_peak_12690	3.00	lobChIP_FOXA1_robot
chr5	141600972	141600973	MACS_peak_12691	4.00	lobChIP_FOXA1_robot
chr5	141670818	141670819	MACS_peak_12692	4.00	lobChIP_FOXA1_robot
chr5	141801026	141801027	MACS_peak_12693	11.00	lobChIP_FOXA1_robot
chr5	142060650	142060651	MACS_peak_12694	8.00	lobChIP_FOXA1_robot
chr5	142120240	142120241	MACS_peak_12695	34.00	lobChIP_FOXA1_robot
chr5	142175689	142175690	MACS_peak_12696	12.00	lobChIP_FOXA1_robot
chr5	142187197	142187198	MACS_peak_12697	7.00	lobChIP_FOXA1_robot
chr5	142200131	142200132	MACS_peak_12698	6.00	lobChIP_FOXA1_robot
chr5	142213264	142213265	MACS_peak_12699	14.00	lobChIP_FOXA1_robot
chr5	142214307	142214308	MACS_peak_12700	4.00	lobChIP_FOXA1_robot
chr5	142268137	142268138	MACS_peak_12701	5.00	lobChIP_FOXA1_robot
chr5	142275709	142275710	MACS_peak_12702	10.00	lobChIP_FOXA1_robot
chr5	142302162	142302163	MACS_peak_12703	34.00	lobChIP_FOXA1_robot
chr5	142345550	142345551	MACS_peak_12704	3.00	lobChIP_FOXA1_robot
chr5	142466746	142466747	MACS_peak_12705	3.00	lobChIP_FOXA1_robot
chr5	142659297	142659298	MACS_peak_12706	4.00	lobChIP_FOXA1_robot
chr5	142735484	142735485	MACS_peak_12707	4.00	lobChIP_FOXA1_robot
chr5	142781654	142781655	MACS_peak_12708	4.00	lobChIP_FOXA1_robot
chr5	142859229	142859230	MACS_peak_12709	4.00	lobChIP_FOXA1_robot
chr5	142871424	142871425	MACS_peak_12710	5.00	lobChIP_FOXA1_robot
chr5	142874859	142874860	MACS_peak_12711	4.00	lobChIP_FOXA1_robot
chr5	142886904	142886905	MACS_peak_12712	23.00	lobChIP_FOXA1_robot
chr5	142927093	142927094	MACS_peak_12713	11.00	lobChIP_FOXA1_robot
chr5	142934967	142934968	MACS_peak_12714	12.00	lobChIP_FOXA1_robot
chr5	142940410	142940411	MACS_peak_12715	5.00	lobChIP_FOXA1_robot
chr5	142940972	142940973	MACS_peak_12716	5.00	lobChIP_FOXA1_robot
chr5	142949170	142949171	MACS_peak_12717	4.00	lobChIP_FOXA1_robot
chr5	142974803	142974804	MACS_peak_12718	13.00	lobChIP_FOXA1_robot
chr5	143009555	143009556	MACS_peak_12719	5.00	lobChIP_FOXA1_robot
chr5	143014911	143014912	MACS_peak_12720	7.00	lobChIP_FOXA1_robot
chr5	143016975	143016976	MACS_peak_12721	19.00	lobChIP_FOXA1_robot
chr5	143037657	143037658	MACS_peak_12722	6.00	lobChIP_FOXA1_robot
chr5	143039379	143039380	MACS_peak_12723	24.00	lobChIP_FOXA1_robot
chr5	143053689	143053690	MACS_peak_12724	31.00	lobChIP_FOXA1_robot
chr5	143059463	143059464	MACS_peak_12725	5.00	lobChIP_FOXA1_robot
chr5	143060559	143060560	MACS_peak_12726	17.00	lobChIP_FOXA1_robot
chr5	144192759	144192760	MACS_peak_12727	5.00	lobChIP_FOXA1_robot
chr5	144617571	144617572	MACS_peak_12728	3.00	lobChIP_FOXA1_robot
chr5	145209502	145209503	MACS_peak_12729	4.00	lobChIP_FOXA1_robot
chr5	145210376	145210377	MACS_peak_12730	4.00	lobChIP_FOXA1_robot
chr5	145235378	145235379	MACS_peak_12731	4.00	lobChIP_FOXA1_robot
chr5	145267507	145267508	MACS_peak_12732	4.00	lobChIP_FOXA1_robot
chr5	145326921	145326922	MACS_peak_12733	5.00	lobChIP_FOXA1_robot
chr5	145348719	145348720	MACS_peak_12734	5.00	lobChIP_FOXA1_robot
chr5	145355405	145355406	MACS_peak_12735	4.00	lobChIP_FOXA1_robot
chr5	145358988	145358989	MACS_peak_12736	31.00	lobChIP_FOXA1_robot
chr5	145359825	145359826	MACS_peak_12737	5.00	lobChIP_FOXA1_robot
chr5	145361642	145361643	MACS_peak_12738	7.00	lobChIP_FOXA1_robot
chr5	145404740	145404741	MACS_peak_12739	5.00	lobChIP_FOXA1_robot
chr5	145517811	145517812	MACS_peak_12740	5.00	lobChIP_FOXA1_robot
chr5	145548820	145548821	MACS_peak_12741	4.00	lobChIP_FOXA1_robot
chr5	146147281	146147282	MACS_peak_12742	9.00	lobChIP_FOXA1_robot
chr5	146520303	146520304	MACS_peak_12743	10.00	lobChIP_FOXA1_robot
chr5	146547277	146547278	MACS_peak_12744	9.00	lobChIP_FOXA1_robot
chr5	146550432	146550433	MACS_peak_12745	15.00	lobChIP_FOXA1_robot
chr5	146555628	146555629	MACS_peak_12746	16.00	lobChIP_FOXA1_robot
chr5	147215082	147215083	MACS_peak_12747	101.00	lobChIP_FOXA1_robot
chr5	147217790	147217791	MACS_peak_12748	6.00	lobChIP_FOXA1_robot
chr5	147525582	147525583	MACS_peak_12749	6.00	lobChIP_FOXA1_robot
chr5	147527221	147527222	MACS_peak_12750	6.00	lobChIP_FOXA1_robot
chr5	147774219	147774220	MACS_peak_12751	16.00	lobChIP_FOXA1_robot
chr5	147778082	147778083	MACS_peak_12752	4.00	lobChIP_FOXA1_robot
chr5	148532029	148532030	MACS_peak_12753	5.00	lobChIP_FOXA1_robot
chr5	148553947	148553948	MACS_peak_12754	3.00	lobChIP_FOXA1_robot
chr5	148825426	148825427	MACS_peak_12755	4.00	lobChIP_FOXA1_robot
chr5	148834076	148834077	MACS_peak_12756	5.00	lobChIP_FOXA1_robot
chr5	148862461	148862462	MACS_peak_12757	7.00	lobChIP_FOXA1_robot
chr5	149363022	149363023	MACS_peak_12758	4.00	lobChIP_FOXA1_robot
chr5	149405872	149405873	MACS_peak_12759	11.00	lobChIP_FOXA1_robot
chr5	149517109	149517110	MACS_peak_12760	7.00	lobChIP_FOXA1_robot
chr5	149687651	149687652	MACS_peak_12761	7.00	lobChIP_FOXA1_robot
chr5	149702850	149702851	MACS_peak_12762	4.00	lobChIP_FOXA1_robot
chr5	149890231	149890232	MACS_peak_12763	6.00	lobChIP_FOXA1_robot
chr5	149902181	149902182	MACS_peak_12764	25.00	lobChIP_FOXA1_robot
chr5	149922675	149922676	MACS_peak_12765	16.00	lobChIP_FOXA1_robot
chr5	149995599	149995600	MACS_peak_12766	5.00	lobChIP_FOXA1_robot
chr5	150017293	150017294	MACS_peak_12767	6.00	lobChIP_FOXA1_robot
chr5	150121950	150121951	MACS_peak_12768	3.00	lobChIP_FOXA1_robot
chr5	150295684	150295685	MACS_peak_12769	4.00	lobChIP_FOXA1_robot
chr5	150384270	150384271	MACS_peak_12770	7.00	lobChIP_FOXA1_robot
chr5	150614600	150614601	MACS_peak_12771	5.00	lobChIP_FOXA1_robot
chr5	150997783	150997784	MACS_peak_12772	27.00	lobChIP_FOXA1_robot
chr5	151199601	151199602	MACS_peak_12773	11.00	lobChIP_FOXA1_robot
chr5	151200689	151200690	MACS_peak_12774	8.00	lobChIP_FOXA1_robot
chr5	153665457	153665458	MACS_peak_12775	6.00	lobChIP_FOXA1_robot
chr5	153666914	153666915	MACS_peak_12776	13.00	lobChIP_FOXA1_robot
chr5	153676586	153676587	MACS_peak_12777	6.00	lobChIP_FOXA1_robot
chr5	153746169	153746170	MACS_peak_12778	5.00	lobChIP_FOXA1_robot
chr5	153766572	153766573	MACS_peak_12779	5.00	lobChIP_FOXA1_robot
chr5	154033206	154033207	MACS_peak_12780	8.00	lobChIP_FOXA1_robot
chr5	154080928	154080929	MACS_peak_12781	5.00	lobChIP_FOXA1_robot
chr5	154109178	154109179	MACS_peak_12782	5.00	lobChIP_FOXA1_robot
chr5	154213767	154213768	MACS_peak_12783	16.00	lobChIP_FOXA1_robot
chr5	154217269	154217270	MACS_peak_12784	7.00	lobChIP_FOXA1_robot
chr5	154222683	154222684	MACS_peak_12785	23.00	lobChIP_FOXA1_robot
chr5	154224644	154224645	MACS_peak_12786	7.00	lobChIP_FOXA1_robot
chr5	154239368	154239369	MACS_peak_12787	5.00	lobChIP_FOXA1_robot
chr5	154840084	154840085	MACS_peak_12788	20.00	lobChIP_FOXA1_robot
chr5	155225070	155225071	MACS_peak_12789	4.00	lobChIP_FOXA1_robot
chr5	156486210	156486211	MACS_peak_12790	7.00	lobChIP_FOXA1_robot
chr5	156715463	156715464	MACS_peak_12791	5.00	lobChIP_FOXA1_robot
chr5	156817786	156817787	MACS_peak_12792	3.00	lobChIP_FOXA1_robot
chr5	156905328	156905329	MACS_peak_12793	4.00	lobChIP_FOXA1_robot
chr5	157249607	157249608	MACS_peak_12794	7.00	lobChIP_FOXA1_robot
chr5	157273107	157273108	MACS_peak_12795	4.00	lobChIP_FOXA1_robot
chr5	157888420	157888421	MACS_peak_12796	3.00	lobChIP_FOXA1_robot
chr5	158609982	158609983	MACS_peak_12797	19.00	lobChIP_FOXA1_robot
chr5	158891197	158891198	MACS_peak_12798	17.00	lobChIP_FOXA1_robot
chr5	159743137	159743138	MACS_peak_12799	6.00	lobChIP_FOXA1_robot
chr5	159894001	159894002	MACS_peak_12800	10.00	lobChIP_FOXA1_robot
chr5	159894644	159894645	MACS_peak_12801	5.00	lobChIP_FOXA1_robot
chr5	159903014	159903015	MACS_peak_12802	7.00	lobChIP_FOXA1_robot
chr5	159907814	159907815	MACS_peak_12803	4.00	lobChIP_FOXA1_robot
chr5	160315025	160315026	MACS_peak_12804	10.00	lobChIP_FOXA1_robot
chr5	161604528	161604529	MACS_peak_12805	5.00	lobChIP_FOXA1_robot
chr5	162806770	162806771	MACS_peak_12806	6.00	lobChIP_FOXA1_robot
chr5	162846890	162846891	MACS_peak_12807	6.00	lobChIP_FOXA1_robot
chr5	165055056	165055057	MACS_peak_12808	9.00	lobChIP_FOXA1_robot
chr5	167742067	167742068	MACS_peak_12809	7.00	lobChIP_FOXA1_robot
chr5	167789279	167789280	MACS_peak_12810	5.00	lobChIP_FOXA1_robot
chr5	167821006	167821007	MACS_peak_12811	7.00	lobChIP_FOXA1_robot
chr5	168005733	168005734	MACS_peak_12812	4.00	lobChIP_FOXA1_robot
chr5	168019149	168019150	MACS_peak_12813	25.00	lobChIP_FOXA1_robot
chr5	168052016	168052017	MACS_peak_12814	8.00	lobChIP_FOXA1_robot
chr5	168416927	168416928	MACS_peak_12815	9.00	lobChIP_FOXA1_robot
chr5	168510492	168510493	MACS_peak_12816	4.00	lobChIP_FOXA1_robot
chr5	169011335	169011336	MACS_peak_12817	3.00	lobChIP_FOXA1_robot
chr5	170767763	170767764	MACS_peak_12818	5.00	lobChIP_FOXA1_robot
chr5	171662420	171662421	MACS_peak_12819	8.00	lobChIP_FOXA1_robot
chr5	171810481	171810482	MACS_peak_12820	9.00	lobChIP_FOXA1_robot
chr5	171855771	171855772	MACS_peak_12821	12.00	lobChIP_FOXA1_robot
chr5	171991373	171991374	MACS_peak_12822	5.00	lobChIP_FOXA1_robot
chr5	172087453	172087454	MACS_peak_12823	4.00	lobChIP_FOXA1_robot
chr5	172151767	172151768	MACS_peak_12824	8.00	lobChIP_FOXA1_robot
chr5	172203045	172203046	MACS_peak_12825	5.00	lobChIP_FOXA1_robot
chr5	172203566	172203567	MACS_peak_12826	8.00	lobChIP_FOXA1_robot
chr5	172206887	172206888	MACS_peak_12827	3.00	lobChIP_FOXA1_robot
chr5	172209738	172209739	MACS_peak_12828	20.00	lobChIP_FOXA1_robot
chr5	172285906	172285907	MACS_peak_12829	5.00	lobChIP_FOXA1_robot
chr5	172321933	172321934	MACS_peak_12830	28.00	lobChIP_FOXA1_robot
chr5	172521340	172521341	MACS_peak_12831	5.00	lobChIP_FOXA1_robot
chr5	172566803	172566804	MACS_peak_12832	5.00	lobChIP_FOXA1_robot
chr5	172718057	172718058	MACS_peak_12833	4.00	lobChIP_FOXA1_robot
chr5	173146890	173146891	MACS_peak_12834	10.00	lobChIP_FOXA1_robot
chr5	173193615	173193616	MACS_peak_12835	14.00	lobChIP_FOXA1_robot
chr5	173216464	173216465	MACS_peak_12836	4.00	lobChIP_FOXA1_robot
chr5	173233453	173233454	MACS_peak_12837	4.00	lobChIP_FOXA1_robot
chr5	173251300	173251301	MACS_peak_12838	14.00	lobChIP_FOXA1_robot
chr5	173251931	173251932	MACS_peak_12839	4.00	lobChIP_FOXA1_robot
chr5	173253707	173253708	MACS_peak_12840	3.00	lobChIP_FOXA1_robot
chr5	173359898	173359899	MACS_peak_12841	7.00	lobChIP_FOXA1_robot
chr5	173369185	173369186	MACS_peak_12842	8.00	lobChIP_FOXA1_robot
chr5	173380623	173380624	MACS_peak_12843	9.00	lobChIP_FOXA1_robot
chr5	173396626	173396627	MACS_peak_12844	8.00	lobChIP_FOXA1_robot
chr5	173596790	173596791	MACS_peak_12845	4.00	lobChIP_FOXA1_robot
chr5	174477922	174477923	MACS_peak_12846	5.00	lobChIP_FOXA1_robot
chr5	174895141	174895142	MACS_peak_12847	28.00	lobChIP_FOXA1_robot
chr5	175133585	175133586	MACS_peak_12848	6.00	lobChIP_FOXA1_robot
chr5	175281705	175281706	MACS_peak_12849	13.00	lobChIP_FOXA1_robot
chr5	175288692	175288693	MACS_peak_12850	6.00	lobChIP_FOXA1_robot
chr5	175866098	175866099	MACS_peak_12851	5.00	lobChIP_FOXA1_robot
chr5	175869250	175869251	MACS_peak_12852	4.00	lobChIP_FOXA1_robot
chr5	177015796	177015797	MACS_peak_12853	8.00	lobChIP_FOXA1_robot
chr5	177710764	177710765	MACS_peak_12854	4.00	lobChIP_FOXA1_robot
chr5	178056192	178056193	MACS_peak_12855	4.00	lobChIP_FOXA1_robot
chr5	178057601	178057602	MACS_peak_12856	18.00	lobChIP_FOXA1_robot
chr5	178206021	178206022	MACS_peak_12857	8.00	lobChIP_FOXA1_robot
chr5	178214302	178214303	MACS_peak_12858	6.00	lobChIP_FOXA1_robot
chr5	178379687	178379688	MACS_peak_12859	4.00	lobChIP_FOXA1_robot
chr5	178961725	178961726	MACS_peak_12860	5.00	lobChIP_FOXA1_robot
chr5	178986061	178986062	MACS_peak_12861	6.00	lobChIP_FOXA1_robot
chr5	179111061	179111062	MACS_peak_12862	13.00	lobChIP_FOXA1_robot
chr5	179246217	179246218	MACS_peak_12863	7.00	lobChIP_FOXA1_robot
chr5	179251839	179251840	MACS_peak_12864	5.00	lobChIP_FOXA1_robot
chr5	179387315	179387316	MACS_peak_12865	6.00	lobChIP_FOXA1_robot
chr5	179508686	179508687	MACS_peak_12866	5.00	lobChIP_FOXA1_robot
chr5	179686248	179686249	MACS_peak_12867	4.00	lobChIP_FOXA1_robot
chr5	180145828	180145829	MACS_peak_12868	5.00	lobChIP_FOXA1_robot
chr5	180614735	180614736	MACS_peak_12869	4.00	lobChIP_FOXA1_robot
chr6	220491	220492	MACS_peak_12870	16.00	lobChIP_FOXA1_robot
chr6	221643	221644	MACS_peak_12871	4.00	lobChIP_FOXA1_robot
chr6	292398	292399	MACS_peak_12872	4.00	lobChIP_FOXA1_robot
chr6	422896	422897	MACS_peak_12873	13.00	lobChIP_FOXA1_robot
chr6	621315	621316	MACS_peak_12874	4.00	lobChIP_FOXA1_robot
chr6	901088	901089	MACS_peak_12875	11.00	lobChIP_FOXA1_robot
chr6	1000099	1000100	MACS_peak_12876	6.00	lobChIP_FOXA1_robot
chr6	1025075	1025076	MACS_peak_12877	6.00	lobChIP_FOXA1_robot
chr6	1044773	1044774	MACS_peak_12878	4.00	lobChIP_FOXA1_robot
chr6	1055450	1055451	MACS_peak_12879	40.00	lobChIP_FOXA1_robot
chr6	1117967	1117968	MACS_peak_12880	5.00	lobChIP_FOXA1_robot
chr6	1187891	1187892	MACS_peak_12881	4.00	lobChIP_FOXA1_robot
chr6	1197901	1197902	MACS_peak_12882	5.00	lobChIP_FOXA1_robot
chr6	1212513	1212514	MACS_peak_12883	4.00	lobChIP_FOXA1_robot
chr6	1228406	1228407	MACS_peak_12884	24.00	lobChIP_FOXA1_robot
chr6	1231030	1231031	MACS_peak_12885	22.00	lobChIP_FOXA1_robot
chr6	1252153	1252154	MACS_peak_12886	12.00	lobChIP_FOXA1_robot
chr6	1264460	1264461	MACS_peak_12887	20.00	lobChIP_FOXA1_robot
chr6	1267698	1267699	MACS_peak_12888	9.00	lobChIP_FOXA1_robot
chr6	1268821	1268822	MACS_peak_12889	24.00	lobChIP_FOXA1_robot
chr6	1312120	1312121	MACS_peak_12890	7.00	lobChIP_FOXA1_robot
chr6	1323198	1323199	MACS_peak_12891	13.00	lobChIP_FOXA1_robot
chr6	1325993	1325994	MACS_peak_12892	30.00	lobChIP_FOXA1_robot
chr6	1336687	1336688	MACS_peak_12893	4.00	lobChIP_FOXA1_robot
chr6	1702827	1702828	MACS_peak_12894	3.00	lobChIP_FOXA1_robot
chr6	2017383	2017384	MACS_peak_12895	6.00	lobChIP_FOXA1_robot
chr6	2054157	2054158	MACS_peak_12896	8.00	lobChIP_FOXA1_robot
chr6	2105602	2105603	MACS_peak_12897	4.00	lobChIP_FOXA1_robot
chr6	2225882	2225883	MACS_peak_12898	13.00	lobChIP_FOXA1_robot
chr6	2246520	2246521	MACS_peak_12899	4.00	lobChIP_FOXA1_robot
chr6	2259378	2259379	MACS_peak_12900	6.00	lobChIP_FOXA1_robot
chr6	2515452	2515453	MACS_peak_12901	3.00	lobChIP_FOXA1_robot
chr6	2515901	2515902	MACS_peak_12902	5.00	lobChIP_FOXA1_robot
chr6	2565928	2565929	MACS_peak_12903	11.00	lobChIP_FOXA1_robot
chr6	2839588	2839589	MACS_peak_12904	8.00	lobChIP_FOXA1_robot
chr6	2877008	2877009	MACS_peak_12905	7.00	lobChIP_FOXA1_robot
chr6	2961397	2961398	MACS_peak_12906	5.00	lobChIP_FOXA1_robot
chr6	2994115	2994116	MACS_peak_12907	17.00	lobChIP_FOXA1_robot
chr6	2999514	2999515	MACS_peak_12908	4.00	lobChIP_FOXA1_robot
chr6	3013932	3013933	MACS_peak_12909	5.00	lobChIP_FOXA1_robot
chr6	3076062	3076063	MACS_peak_12910	4.00	lobChIP_FOXA1_robot
chr6	3098789	3098790	MACS_peak_12911	3.00	lobChIP_FOXA1_robot
chr6	3136008	3136009	MACS_peak_12912	12.00	lobChIP_FOXA1_robot
chr6	3139622	3139623	MACS_peak_12913	20.00	lobChIP_FOXA1_robot
chr6	3221336	3221337	MACS_peak_12914	4.00	lobChIP_FOXA1_robot
chr6	3357452	3357453	MACS_peak_12915	20.00	lobChIP_FOXA1_robot
chr6	3367202	3367203	MACS_peak_12916	3.00	lobChIP_FOXA1_robot
chr6	3380768	3380769	MACS_peak_12917	27.00	lobChIP_FOXA1_robot
chr6	3383297	3383298	MACS_peak_12918	3.00	lobChIP_FOXA1_robot
chr6	3383862	3383863	MACS_peak_12919	5.00	lobChIP_FOXA1_robot
chr6	3403662	3403663	MACS_peak_12920	16.00	lobChIP_FOXA1_robot
chr6	3410354	3410355	MACS_peak_12921	9.00	lobChIP_FOXA1_robot
chr6	3412546	3412547	MACS_peak_12922	7.00	lobChIP_FOXA1_robot
chr6	3501075	3501076	MACS_peak_12923	4.00	lobChIP_FOXA1_robot
chr6	3502417	3502418	MACS_peak_12924	9.00	lobChIP_FOXA1_robot
chr6	3789458	3789459	MACS_peak_12925	6.00	lobChIP_FOXA1_robot
chr6	3790279	3790280	MACS_peak_12926	7.00	lobChIP_FOXA1_robot
chr6	3798245	3798246	MACS_peak_12927	8.00	lobChIP_FOXA1_robot
chr6	3839616	3839617	MACS_peak_12928	8.00	lobChIP_FOXA1_robot
chr6	3841404	3841405	MACS_peak_12929	18.00	lobChIP_FOXA1_robot
chr6	3870242	3870243	MACS_peak_12930	9.00	lobChIP_FOXA1_robot
chr6	3912261	3912262	MACS_peak_12931	11.00	lobChIP_FOXA1_robot
chr6	3913976	3913977	MACS_peak_12932	12.00	lobChIP_FOXA1_robot
chr6	3963543	3963544	MACS_peak_12933	3.00	lobChIP_FOXA1_robot
chr6	4103103	4103104	MACS_peak_12934	7.00	lobChIP_FOXA1_robot
chr6	4791895	4791896	MACS_peak_12935	4.00	lobChIP_FOXA1_robot
chr6	4795158	4795159	MACS_peak_12936	4.00	lobChIP_FOXA1_robot
chr6	4810440	4810441	MACS_peak_12937	13.00	lobChIP_FOXA1_robot
chr6	4849120	4849121	MACS_peak_12938	5.00	lobChIP_FOXA1_robot
chr6	5002551	5002552	MACS_peak_12939	4.00	lobChIP_FOXA1_robot
chr6	5017593	5017594	MACS_peak_12940	5.00	lobChIP_FOXA1_robot
chr6	5020576	5020577	MACS_peak_12941	6.00	lobChIP_FOXA1_robot
chr6	5059844	5059845	MACS_peak_12942	12.00	lobChIP_FOXA1_robot
chr6	5068487	5068488	MACS_peak_12943	15.00	lobChIP_FOXA1_robot
chr6	5080038	5080039	MACS_peak_12944	3.00	lobChIP_FOXA1_robot
chr6	5115552	5115553	MACS_peak_12945	13.00	lobChIP_FOXA1_robot
chr6	5190923	5190924	MACS_peak_12946	4.00	lobChIP_FOXA1_robot
chr6	5206341	5206342	MACS_peak_12947	6.00	lobChIP_FOXA1_robot
chr6	5713981	5713982	MACS_peak_12948	10.00	lobChIP_FOXA1_robot
chr6	6312116	6312117	MACS_peak_12949	4.00	lobChIP_FOXA1_robot
chr6	6688823	6688824	MACS_peak_12950	4.00	lobChIP_FOXA1_robot
chr6	6750434	6750435	MACS_peak_12951	5.00	lobChIP_FOXA1_robot
chr6	6759559	6759560	MACS_peak_12952	7.00	lobChIP_FOXA1_robot
chr6	7139413	7139414	MACS_peak_12953	17.00	lobChIP_FOXA1_robot
chr6	7140102	7140103	MACS_peak_12954	5.00	lobChIP_FOXA1_robot
chr6	7165542	7165543	MACS_peak_12955	7.00	lobChIP_FOXA1_robot
chr6	7417936	7417937	MACS_peak_12956	4.00	lobChIP_FOXA1_robot
chr6	7419464	7419465	MACS_peak_12957	4.00	lobChIP_FOXA1_robot
chr6	7467736	7467737	MACS_peak_12958	3.00	lobChIP_FOXA1_robot
chr6	7541477	7541478	MACS_peak_12959	10.00	lobChIP_FOXA1_robot
chr6	7548999	7549000	MACS_peak_12960	23.00	lobChIP_FOXA1_robot
chr6	7549884	7549885	MACS_peak_12961	6.00	lobChIP_FOXA1_robot
chr6	7550800	7550801	MACS_peak_12962	21.00	lobChIP_FOXA1_robot
chr6	7562061	7562062	MACS_peak_12963	13.00	lobChIP_FOXA1_robot
chr6	7581083	7581084	MACS_peak_12964	3.00	lobChIP_FOXA1_robot
chr6	7698563	7698564	MACS_peak_12965	4.00	lobChIP_FOXA1_robot
chr6	7975613	7975614	MACS_peak_12966	11.00	lobChIP_FOXA1_robot
chr6	8027232	8027233	MACS_peak_12967	4.00	lobChIP_FOXA1_robot
chr6	8047011	8047012	MACS_peak_12968	3.00	lobChIP_FOXA1_robot
chr6	8064840	8064841	MACS_peak_12969	3.00	lobChIP_FOXA1_robot
chr6	8085460	8085461	MACS_peak_12970	7.00	lobChIP_FOXA1_robot
chr6	8138082	8138083	MACS_peak_12971	8.00	lobChIP_FOXA1_robot
chr6	8158371	8158372	MACS_peak_12972	5.00	lobChIP_FOXA1_robot
chr6	8619811	8619812	MACS_peak_12973	5.00	lobChIP_FOXA1_robot
chr6	8959230	8959231	MACS_peak_12974	18.00	lobChIP_FOXA1_robot
chr6	9400182	9400183	MACS_peak_12975	4.00	lobChIP_FOXA1_robot
chr6	10458129	10458130	MACS_peak_12976	4.00	lobChIP_FOXA1_robot
chr6	10505984	10505985	MACS_peak_12977	25.00	lobChIP_FOXA1_robot
chr6	10528525	10528526	MACS_peak_12978	5.00	lobChIP_FOXA1_robot
chr6	10613501	10613502	MACS_peak_12979	59.00	lobChIP_FOXA1_robot
chr6	10743386	10743387	MACS_peak_12980	4.00	lobChIP_FOXA1_robot
chr6	10793661	10793662	MACS_peak_12981	3.00	lobChIP_FOXA1_robot
chr6	10954957	10954958	MACS_peak_12982	5.00	lobChIP_FOXA1_robot
chr6	11006610	11006611	MACS_peak_12983	3.00	lobChIP_FOXA1_robot
chr6	11008270	11008271	MACS_peak_12984	14.00	lobChIP_FOXA1_robot
chr6	11012747	11012748	MACS_peak_12985	31.00	lobChIP_FOXA1_robot
chr6	11039596	11039597	MACS_peak_12986	3.00	lobChIP_FOXA1_robot
chr6	11055473	11055474	MACS_peak_12987	7.00	lobChIP_FOXA1_robot
chr6	11067165	11067166	MACS_peak_12988	10.00	lobChIP_FOXA1_robot
chr6	11179993	11179994	MACS_peak_12989	6.00	lobChIP_FOXA1_robot
chr6	11184136	11184137	MACS_peak_12990	5.00	lobChIP_FOXA1_robot
chr6	11203288	11203289	MACS_peak_12991	10.00	lobChIP_FOXA1_robot
chr6	11207609	11207610	MACS_peak_12992	6.00	lobChIP_FOXA1_robot
chr6	11217656	11217657	MACS_peak_12993	4.00	lobChIP_FOXA1_robot
chr6	11219164	11219165	MACS_peak_12994	12.00	lobChIP_FOXA1_robot
chr6	11226444	11226445	MACS_peak_12995	23.00	lobChIP_FOXA1_robot
chr6	11233371	11233372	MACS_peak_12996	4.00	lobChIP_FOXA1_robot
chr6	11270729	11270730	MACS_peak_12997	7.00	lobChIP_FOXA1_robot
chr6	11275977	11275978	MACS_peak_12998	6.00	lobChIP_FOXA1_robot
chr6	11277560	11277561	MACS_peak_12999	16.00	lobChIP_FOXA1_robot
chr6	11299535	11299536	MACS_peak_13000	10.00	lobChIP_FOXA1_robot
chr6	11359566	11359567	MACS_peak_13001	8.00	lobChIP_FOXA1_robot
chr6	11368094	11368095	MACS_peak_13002	5.00	lobChIP_FOXA1_robot
chr6	11392889	11392890	MACS_peak_13003	33.00	lobChIP_FOXA1_robot
chr6	11398843	11398844	MACS_peak_13004	10.00	lobChIP_FOXA1_robot
chr6	11425900	11425901	MACS_peak_13005	6.00	lobChIP_FOXA1_robot
chr6	11435152	11435153	MACS_peak_13006	20.00	lobChIP_FOXA1_robot
chr6	11463391	11463392	MACS_peak_13007	8.00	lobChIP_FOXA1_robot
chr6	11700619	11700620	MACS_peak_13008	6.00	lobChIP_FOXA1_robot
chr6	12069350	12069351	MACS_peak_13009	5.00	lobChIP_FOXA1_robot
chr6	12298365	12298366	MACS_peak_13010	5.00	lobChIP_FOXA1_robot
chr6	12324646	12324647	MACS_peak_13011	5.00	lobChIP_FOXA1_robot
chr6	12332334	12332335	MACS_peak_13012	7.00	lobChIP_FOXA1_robot
chr6	12343918	12343919	MACS_peak_13013	6.00	lobChIP_FOXA1_robot
chr6	12349047	12349048	MACS_peak_13014	14.00	lobChIP_FOXA1_robot
chr6	12372163	12372164	MACS_peak_13015	5.00	lobChIP_FOXA1_robot
chr6	12384908	12384909	MACS_peak_13016	8.00	lobChIP_FOXA1_robot
chr6	12484998	12484999	MACS_peak_13017	4.00	lobChIP_FOXA1_robot
chr6	12492819	12492820	MACS_peak_13018	18.00	lobChIP_FOXA1_robot
chr6	13290522	13290523	MACS_peak_13019	4.00	lobChIP_FOXA1_robot
chr6	13326289	13326290	MACS_peak_13020	8.00	lobChIP_FOXA1_robot
chr6	13367097	13367098	MACS_peak_13021	4.00	lobChIP_FOXA1_robot
chr6	13408052	13408053	MACS_peak_13022	15.00	lobChIP_FOXA1_robot
chr6	13413983	13413984	MACS_peak_13023	15.00	lobChIP_FOXA1_robot
chr6	13485260	13485261	MACS_peak_13024	10.00	lobChIP_FOXA1_robot
chr6	13575622	13575623	MACS_peak_13025	4.00	lobChIP_FOXA1_robot
chr6	13605352	13605353	MACS_peak_13026	28.00	lobChIP_FOXA1_robot
chr6	13661248	13661249	MACS_peak_13027	14.00	lobChIP_FOXA1_robot
chr6	13665205	13665206	MACS_peak_13028	6.00	lobChIP_FOXA1_robot
chr6	13679146	13679147	MACS_peak_13029	3.00	lobChIP_FOXA1_robot
chr6	13699369	13699370	MACS_peak_13030	6.00	lobChIP_FOXA1_robot
chr6	13818805	13818806	MACS_peak_13031	6.00	lobChIP_FOXA1_robot
chr6	13976643	13976644	MACS_peak_13032	4.00	lobChIP_FOXA1_robot
chr6	14046815	14046816	MACS_peak_13033	4.00	lobChIP_FOXA1_robot
chr6	14170187	14170188	MACS_peak_13034	4.00	lobChIP_FOXA1_robot
chr6	14533783	14533784	MACS_peak_13035	3.00	lobChIP_FOXA1_robot
chr6	15027206	15027207	MACS_peak_13036	6.00	lobChIP_FOXA1_robot
chr6	15214313	15214314	MACS_peak_13037	4.00	lobChIP_FOXA1_robot
chr6	15254297	15254298	MACS_peak_13038	5.00	lobChIP_FOXA1_robot
chr6	15279454	15279455	MACS_peak_13039	9.00	lobChIP_FOXA1_robot
chr6	15280335	15280336	MACS_peak_13040	6.00	lobChIP_FOXA1_robot
chr6	15325154	15325155	MACS_peak_13041	4.00	lobChIP_FOXA1_robot
chr6	15362704	15362705	MACS_peak_13042	6.00	lobChIP_FOXA1_robot
chr6	15402346	15402347	MACS_peak_13043	6.00	lobChIP_FOXA1_robot
chr6	15429242	15429243	MACS_peak_13044	3.00	lobChIP_FOXA1_robot
chr6	15431180	15431181	MACS_peak_13045	3.00	lobChIP_FOXA1_robot
chr6	15432153	15432154	MACS_peak_13046	5.00	lobChIP_FOXA1_robot
chr6	15434729	15434730	MACS_peak_13047	5.00	lobChIP_FOXA1_robot
chr6	15435214	15435215	MACS_peak_13048	5.00	lobChIP_FOXA1_robot
chr6	15949761	15949762	MACS_peak_13049	6.00	lobChIP_FOXA1_robot
chr6	15956619	15956620	MACS_peak_13050	4.00	lobChIP_FOXA1_robot
chr6	16272729	16272730	MACS_peak_13051	3.00	lobChIP_FOXA1_robot
chr6	16345658	16345659	MACS_peak_13052	5.00	lobChIP_FOXA1_robot
chr6	16431083	16431084	MACS_peak_13053	4.00	lobChIP_FOXA1_robot
chr6	16539919	16539920	MACS_peak_13054	7.00	lobChIP_FOXA1_robot
chr6	16576771	16576772	MACS_peak_13055	9.00	lobChIP_FOXA1_robot
chr6	16586796	16586797	MACS_peak_13056	11.00	lobChIP_FOXA1_robot
chr6	16677384	16677385	MACS_peak_13057	4.00	lobChIP_FOXA1_robot
chr6	16681998	16681999	MACS_peak_13058	6.00	lobChIP_FOXA1_robot
chr6	16683887	16683888	MACS_peak_13059	4.00	lobChIP_FOXA1_robot
chr6	16684791	16684792	MACS_peak_13060	4.00	lobChIP_FOXA1_robot
chr6	16691201	16691202	MACS_peak_13061	9.00	lobChIP_FOXA1_robot
chr6	16699784	16699785	MACS_peak_13062	7.00	lobChIP_FOXA1_robot
chr6	16711817	16711818	MACS_peak_13063	5.00	lobChIP_FOXA1_robot
chr6	16716911	16716912	MACS_peak_13064	4.00	lobChIP_FOXA1_robot
chr6	16725761	16725762	MACS_peak_13065	5.00	lobChIP_FOXA1_robot
chr6	16726212	16726213	MACS_peak_13066	7.00	lobChIP_FOXA1_robot
chr6	16727219	16727220	MACS_peak_13067	3.00	lobChIP_FOXA1_robot
chr6	16729696	16729697	MACS_peak_13068	6.00	lobChIP_FOXA1_robot
chr6	16731242	16731243	MACS_peak_13069	14.00	lobChIP_FOXA1_robot
chr6	16737255	16737256	MACS_peak_13070	5.00	lobChIP_FOXA1_robot
chr6	16739406	16739407	MACS_peak_13071	4.00	lobChIP_FOXA1_robot
chr6	16753023	16753024	MACS_peak_13072	4.00	lobChIP_FOXA1_robot
chr6	16755327	16755328	MACS_peak_13073	19.00	lobChIP_FOXA1_robot
chr6	16800295	16800296	MACS_peak_13074	5.00	lobChIP_FOXA1_robot
chr6	16808365	16808366	MACS_peak_13075	9.00	lobChIP_FOXA1_robot
chr6	16822265	16822266	MACS_peak_13076	7.00	lobChIP_FOXA1_robot
chr6	17097039	17097040	MACS_peak_13077	14.00	lobChIP_FOXA1_robot
chr6	17159532	17159533	MACS_peak_13078	4.00	lobChIP_FOXA1_robot
chr6	17271778	17271779	MACS_peak_13079	3.00	lobChIP_FOXA1_robot
chr6	17312185	17312186	MACS_peak_13080	8.00	lobChIP_FOXA1_robot
chr6	17355140	17355141	MACS_peak_13081	14.00	lobChIP_FOXA1_robot
chr6	17367303	17367304	MACS_peak_13082	6.00	lobChIP_FOXA1_robot
chr6	17398069	17398070	MACS_peak_13083	3.00	lobChIP_FOXA1_robot
chr6	17530600	17530601	MACS_peak_13084	3.00	lobChIP_FOXA1_robot
chr6	17603449	17603450	MACS_peak_13085	6.00	lobChIP_FOXA1_robot
chr6	17713003	17713004	MACS_peak_13086	8.00	lobChIP_FOXA1_robot
chr6	17714553	17714554	MACS_peak_13087	10.00	lobChIP_FOXA1_robot
chr6	17876413	17876414	MACS_peak_13088	4.00	lobChIP_FOXA1_robot
chr6	17909183	17909184	MACS_peak_13089	5.00	lobChIP_FOXA1_robot
chr6	17930257	17930258	MACS_peak_13090	5.00	lobChIP_FOXA1_robot
chr6	17932631	17932632	MACS_peak_13091	6.00	lobChIP_FOXA1_robot
chr6	18057338	18057339	MACS_peak_13092	10.00	lobChIP_FOXA1_robot
chr6	18081622	18081623	MACS_peak_13093	10.00	lobChIP_FOXA1_robot
chr6	18389380	18389381	MACS_peak_13094	4.00	lobChIP_FOXA1_robot
chr6	18468627	18468628	MACS_peak_13095	23.00	lobChIP_FOXA1_robot
chr6	19811876	19811877	MACS_peak_13096	6.00	lobChIP_FOXA1_robot
chr6	20350375	20350376	MACS_peak_13097	5.00	lobChIP_FOXA1_robot
chr6	20543352	20543353	MACS_peak_13098	5.00	lobChIP_FOXA1_robot
chr6	20573297	20573298	MACS_peak_13099	5.00	lobChIP_FOXA1_robot
chr6	20594698	20594699	MACS_peak_13100	19.00	lobChIP_FOXA1_robot
chr6	20631371	20631372	MACS_peak_13101	9.00	lobChIP_FOXA1_robot
chr6	20658435	20658436	MACS_peak_13102	6.00	lobChIP_FOXA1_robot
chr6	20681816	20681817	MACS_peak_13103	8.00	lobChIP_FOXA1_robot
chr6	20707641	20707642	MACS_peak_13104	8.00	lobChIP_FOXA1_robot
chr6	20740673	20740674	MACS_peak_13105	4.00	lobChIP_FOXA1_robot
chr6	20848332	20848333	MACS_peak_13106	4.00	lobChIP_FOXA1_robot
chr6	20877821	20877822	MACS_peak_13107	6.00	lobChIP_FOXA1_robot
chr6	20933562	20933563	MACS_peak_13108	5.00	lobChIP_FOXA1_robot
chr6	21001120	21001121	MACS_peak_13109	4.00	lobChIP_FOXA1_robot
chr6	21004192	21004193	MACS_peak_13110	57.00	lobChIP_FOXA1_robot
chr6	21023237	21023238	MACS_peak_13111	4.00	lobChIP_FOXA1_robot
chr6	21082533	21082534	MACS_peak_13112	3.00	lobChIP_FOXA1_robot
chr6	21104006	21104007	MACS_peak_13113	19.00	lobChIP_FOXA1_robot
chr6	21111858	21111859	MACS_peak_13114	15.00	lobChIP_FOXA1_robot
chr6	21241326	21241327	MACS_peak_13115	4.00	lobChIP_FOXA1_robot
chr6	21381950	21381951	MACS_peak_13116	17.00	lobChIP_FOXA1_robot
chr6	21451857	21451858	MACS_peak_13117	13.00	lobChIP_FOXA1_robot
chr6	21598026	21598027	MACS_peak_13118	4.00	lobChIP_FOXA1_robot
chr6	23792629	23792630	MACS_peak_13119	4.00	lobChIP_FOXA1_robot
chr6	24110967	24110968	MACS_peak_13120	4.00	lobChIP_FOXA1_robot
chr6	24146076	24146077	MACS_peak_13121	6.00	lobChIP_FOXA1_robot
chr6	24148472	24148473	MACS_peak_13122	25.00	lobChIP_FOXA1_robot
chr6	24150309	24150310	MACS_peak_13123	12.00	lobChIP_FOXA1_robot
chr6	24172889	24172890	MACS_peak_13124	3.00	lobChIP_FOXA1_robot
chr6	24195668	24195669	MACS_peak_13125	6.00	lobChIP_FOXA1_robot
chr6	24241410	24241411	MACS_peak_13126	7.00	lobChIP_FOXA1_robot
chr6	24283741	24283742	MACS_peak_13127	4.00	lobChIP_FOXA1_robot
chr6	24313779	24313780	MACS_peak_13128	22.00	lobChIP_FOXA1_robot
chr6	24322985	24322986	MACS_peak_13129	6.00	lobChIP_FOXA1_robot
chr6	24324258	24324259	MACS_peak_13130	4.00	lobChIP_FOXA1_robot
chr6	24330593	24330594	MACS_peak_13131	27.00	lobChIP_FOXA1_robot
chr6	24332949	24332950	MACS_peak_13132	4.00	lobChIP_FOXA1_robot
chr6	24341656	24341657	MACS_peak_13133	59.00	lobChIP_FOXA1_robot
chr6	24362705	24362706	MACS_peak_13134	4.00	lobChIP_FOXA1_robot
chr6	24385976	24385977	MACS_peak_13135	19.00	lobChIP_FOXA1_robot
chr6	24505110	24505111	MACS_peak_13136	3.00	lobChIP_FOXA1_robot
chr6	24581535	24581536	MACS_peak_13137	4.00	lobChIP_FOXA1_robot
chr6	24767096	24767097	MACS_peak_13138	5.00	lobChIP_FOXA1_robot
chr6	24771664	24771665	MACS_peak_13139	6.00	lobChIP_FOXA1_robot
chr6	24779818	24779819	MACS_peak_13140	5.00	lobChIP_FOXA1_robot
chr6	24803765	24803766	MACS_peak_13141	8.00	lobChIP_FOXA1_robot
chr6	24824602	24824603	MACS_peak_13142	8.00	lobChIP_FOXA1_robot
chr6	25084138	25084139	MACS_peak_13143	6.00	lobChIP_FOXA1_robot
chr6	25270944	25270945	MACS_peak_13144	4.00	lobChIP_FOXA1_robot
chr6	25597585	25597586	MACS_peak_13145	5.00	lobChIP_FOXA1_robot
chr6	25642725	25642726	MACS_peak_13146	6.00	lobChIP_FOXA1_robot
chr6	25832255	25832256	MACS_peak_13147	4.00	lobChIP_FOXA1_robot
chr6	25900452	25900453	MACS_peak_13148	4.00	lobChIP_FOXA1_robot
chr6	25907832	25907833	MACS_peak_13149	14.00	lobChIP_FOXA1_robot
chr6	25929368	25929369	MACS_peak_13150	24.00	lobChIP_FOXA1_robot
chr6	25931051	25931052	MACS_peak_13151	8.00	lobChIP_FOXA1_robot
chr6	26027032	26027033	MACS_peak_13152	4.00	lobChIP_FOXA1_robot
chr6	26031035	26031036	MACS_peak_13153	4.00	lobChIP_FOXA1_robot
chr6	26033341	26033342	MACS_peak_13154	3.00	lobChIP_FOXA1_robot
chr6	26044535	26044536	MACS_peak_13155	4.00	lobChIP_FOXA1_robot
chr6	26046568	26046569	MACS_peak_13156	6.00	lobChIP_FOXA1_robot
chr6	26055428	26055429	MACS_peak_13157	5.00	lobChIP_FOXA1_robot
chr6	26103521	26103522	MACS_peak_13158	5.00	lobChIP_FOXA1_robot
chr6	26105136	26105137	MACS_peak_13159	13.00	lobChIP_FOXA1_robot
chr6	26119433	26119434	MACS_peak_13160	3.00	lobChIP_FOXA1_robot
chr6	26121472	26121473	MACS_peak_13161	3.00	lobChIP_FOXA1_robot
chr6	26123294	26123295	MACS_peak_13162	4.00	lobChIP_FOXA1_robot
chr6	26123952	26123953	MACS_peak_13163	4.00	lobChIP_FOXA1_robot
chr6	26158256	26158257	MACS_peak_13164	5.00	lobChIP_FOXA1_robot
chr6	26198436	26198437	MACS_peak_13165	6.00	lobChIP_FOXA1_robot
chr6	26200393	26200394	MACS_peak_13166	5.00	lobChIP_FOXA1_robot
chr6	26257563	26257564	MACS_peak_13167	5.00	lobChIP_FOXA1_robot
chr6	26258722	26258723	MACS_peak_13168	4.00	lobChIP_FOXA1_robot
chr6	26296661	26296662	MACS_peak_13169	5.00	lobChIP_FOXA1_robot
chr6	26312118	26312119	MACS_peak_13170	7.00	lobChIP_FOXA1_robot
chr6	26319964	26319965	MACS_peak_13171	4.00	lobChIP_FOXA1_robot
chr6	26331892	26331893	MACS_peak_13172	4.00	lobChIP_FOXA1_robot
chr6	26366028	26366029	MACS_peak_13173	3.00	lobChIP_FOXA1_robot
chr6	26388440	26388441	MACS_peak_13174	5.00	lobChIP_FOXA1_robot
chr6	26415908	26415909	MACS_peak_13175	4.00	lobChIP_FOXA1_robot
chr6	26476221	26476222	MACS_peak_13176	4.00	lobChIP_FOXA1_robot
chr6	26502580	26502581	MACS_peak_13177	5.00	lobChIP_FOXA1_robot
chr6	26517375	26517376	MACS_peak_13178	5.00	lobChIP_FOXA1_robot
chr6	26544898	26544899	MACS_peak_13179	26.00	lobChIP_FOXA1_robot
chr6	26562330	26562331	MACS_peak_13180	4.00	lobChIP_FOXA1_robot
chr6	26565639	26565640	MACS_peak_13181	18.00	lobChIP_FOXA1_robot
chr6	26706315	26706316	MACS_peak_13182	6.00	lobChIP_FOXA1_robot
chr6	26925727	26925728	MACS_peak_13183	5.00	lobChIP_FOXA1_robot
chr6	27008231	27008232	MACS_peak_13184	4.00	lobChIP_FOXA1_robot
chr6	27100076	27100077	MACS_peak_13185	6.00	lobChIP_FOXA1_robot
chr6	27125853	27125854	MACS_peak_13186	3.00	lobChIP_FOXA1_robot
chr6	27470529	27470530	MACS_peak_13187	3.00	lobChIP_FOXA1_robot
chr6	27520842	27520843	MACS_peak_13188	3.00	lobChIP_FOXA1_robot
chr6	27760882	27760883	MACS_peak_13189	3.00	lobChIP_FOXA1_robot
chr6	27763652	27763653	MACS_peak_13190	4.00	lobChIP_FOXA1_robot
chr6	27782016	27782017	MACS_peak_13191	7.00	lobChIP_FOXA1_robot
chr6	27799673	27799674	MACS_peak_13192	4.00	lobChIP_FOXA1_robot
chr6	27804896	27804897	MACS_peak_13193	3.00	lobChIP_FOXA1_robot
chr6	27807498	27807499	MACS_peak_13194	5.00	lobChIP_FOXA1_robot
chr6	27860458	27860459	MACS_peak_13195	4.00	lobChIP_FOXA1_robot
chr6	27865130	27865131	MACS_peak_13196	4.00	lobChIP_FOXA1_robot
chr6	28290724	28290725	MACS_peak_13197	8.00	lobChIP_FOXA1_robot
chr6	28291927	28291928	MACS_peak_13198	7.00	lobChIP_FOXA1_robot
chr6	28915870	28915871	MACS_peak_13199	5.00	lobChIP_FOXA1_robot
chr6	28920966	28920967	MACS_peak_13200	5.00	lobChIP_FOXA1_robot
chr6	28949034	28949035	MACS_peak_13201	5.00	lobChIP_FOXA1_robot
chr6	28950093	28950094	MACS_peak_13202	11.00	lobChIP_FOXA1_robot
chr6	29932750	29932751	MACS_peak_13203	4.00	lobChIP_FOXA1_robot
chr6	29935522	29935523	MACS_peak_13204	4.00	lobChIP_FOXA1_robot
chr6	30102052	30102053	MACS_peak_13205	4.00	lobChIP_FOXA1_robot
chr6	30103723	30103724	MACS_peak_13206	6.00	lobChIP_FOXA1_robot
chr6	30137869	30137870	MACS_peak_13207	4.00	lobChIP_FOXA1_robot
chr6	30174900	30174901	MACS_peak_13208	4.00	lobChIP_FOXA1_robot
chr6	30279770	30279771	MACS_peak_13209	3.00	lobChIP_FOXA1_robot
chr6	30293429	30293430	MACS_peak_13210	5.00	lobChIP_FOXA1_robot
chr6	30346301	30346302	MACS_peak_13211	5.00	lobChIP_FOXA1_robot
chr6	30495891	30495892	MACS_peak_13212	7.00	lobChIP_FOXA1_robot
chr6	30524869	30524870	MACS_peak_13213	5.00	lobChIP_FOXA1_robot
chr6	30580581	30580582	MACS_peak_13214	4.00	lobChIP_FOXA1_robot
chr6	30623271	30623272	MACS_peak_13215	6.00	lobChIP_FOXA1_robot
chr6	30716247	30716248	MACS_peak_13216	6.00	lobChIP_FOXA1_robot
chr6	30717402	30717403	MACS_peak_13217	5.00	lobChIP_FOXA1_robot
chr6	30727451	30727452	MACS_peak_13218	3.00	lobChIP_FOXA1_robot
chr6	30732079	30732080	MACS_peak_13219	22.00	lobChIP_FOXA1_robot
chr6	30737511	30737512	MACS_peak_13220	6.00	lobChIP_FOXA1_robot
chr6	30750322	30750323	MACS_peak_13221	11.00	lobChIP_FOXA1_robot
chr6	30767735	30767736	MACS_peak_13222	13.00	lobChIP_FOXA1_robot
chr6	30789937	30789938	MACS_peak_13223	5.00	lobChIP_FOXA1_robot
chr6	30790619	30790620	MACS_peak_13224	29.00	lobChIP_FOXA1_robot
chr6	30825508	30825509	MACS_peak_13225	4.00	lobChIP_FOXA1_robot
chr6	31061102	31061103	MACS_peak_13226	5.00	lobChIP_FOXA1_robot
chr6	31171241	31171242	MACS_peak_13227	10.00	lobChIP_FOXA1_robot
chr6	31335203	31335204	MACS_peak_13228	4.00	lobChIP_FOXA1_robot
chr6	31588188	31588189	MACS_peak_13229	4.00	lobChIP_FOXA1_robot
chr6	31622591	31622592	MACS_peak_13230	5.00	lobChIP_FOXA1_robot
chr6	31623524	31623525	MACS_peak_13231	8.00	lobChIP_FOXA1_robot
chr6	31642777	31642778	MACS_peak_13232	5.00	lobChIP_FOXA1_robot
chr6	31682077	31682078	MACS_peak_13233	4.00	lobChIP_FOXA1_robot
chr6	31705589	31705590	MACS_peak_13234	20.00	lobChIP_FOXA1_robot
chr6	31707334	31707335	MACS_peak_13235	28.00	lobChIP_FOXA1_robot
chr6	31787526	31787527	MACS_peak_13236	3.00	lobChIP_FOXA1_robot
chr6	31833289	31833290	MACS_peak_13237	6.00	lobChIP_FOXA1_robot
chr6	31889065	31889066	MACS_peak_13238	4.00	lobChIP_FOXA1_robot
chr6	31895194	31895195	MACS_peak_13239	17.00	lobChIP_FOXA1_robot
chr6	31913596	31913597	MACS_peak_13240	8.00	lobChIP_FOXA1_robot
chr6	32266601	32266602	MACS_peak_13241	9.00	lobChIP_FOXA1_robot
chr6	32342007	32342008	MACS_peak_13242	4.00	lobChIP_FOXA1_robot
chr6	33021080	33021081	MACS_peak_13243	7.00	lobChIP_FOXA1_robot
chr6	33166425	33166426	MACS_peak_13244	6.00	lobChIP_FOXA1_robot
chr6	33177089	33177090	MACS_peak_13245	3.00	lobChIP_FOXA1_robot
chr6	33239381	33239382	MACS_peak_13246	4.00	lobChIP_FOXA1_robot
chr6	33257346	33257347	MACS_peak_13247	4.00	lobChIP_FOXA1_robot
chr6	33575786	33575787	MACS_peak_13248	4.00	lobChIP_FOXA1_robot
chr6	33808070	33808071	MACS_peak_13249	8.00	lobChIP_FOXA1_robot
chr6	33956248	33956249	MACS_peak_13250	69.00	lobChIP_FOXA1_robot
chr6	34383136	34383137	MACS_peak_13251	3.00	lobChIP_FOXA1_robot
chr6	34566309	34566310	MACS_peak_13252	3.00	lobChIP_FOXA1_robot
chr6	34625356	34625357	MACS_peak_13253	4.00	lobChIP_FOXA1_robot
chr6	34630432	34630433	MACS_peak_13254	4.00	lobChIP_FOXA1_robot
chr6	34635644	34635645	MACS_peak_13255	6.00	lobChIP_FOXA1_robot
chr6	34642283	34642284	MACS_peak_13256	8.00	lobChIP_FOXA1_robot
chr6	34713853	34713854	MACS_peak_13257	5.00	lobChIP_FOXA1_robot
chr6	34759181	34759182	MACS_peak_13258	6.00	lobChIP_FOXA1_robot
chr6	34921615	34921616	MACS_peak_13259	37.00	lobChIP_FOXA1_robot
chr6	35134453	35134454	MACS_peak_13260	5.00	lobChIP_FOXA1_robot
chr6	35300182	35300183	MACS_peak_13261	9.00	lobChIP_FOXA1_robot
chr6	35328321	35328322	MACS_peak_13262	5.00	lobChIP_FOXA1_robot
chr6	35336632	35336633	MACS_peak_13263	7.00	lobChIP_FOXA1_robot
chr6	35339616	35339617	MACS_peak_13264	4.00	lobChIP_FOXA1_robot
chr6	35383286	35383287	MACS_peak_13265	34.00	lobChIP_FOXA1_robot
chr6	35396477	35396478	MACS_peak_13266	5.00	lobChIP_FOXA1_robot
chr6	35571169	35571170	MACS_peak_13267	6.00	lobChIP_FOXA1_robot
chr6	35886986	35886987	MACS_peak_13268	5.00	lobChIP_FOXA1_robot
chr6	36031306	36031307	MACS_peak_13269	6.00	lobChIP_FOXA1_robot
chr6	36063921	36063922	MACS_peak_13270	10.00	lobChIP_FOXA1_robot
chr6	36302944	36302945	MACS_peak_13271	6.00	lobChIP_FOXA1_robot
chr6	36459510	36459511	MACS_peak_13272	11.00	lobChIP_FOXA1_robot
chr6	36461844	36461845	MACS_peak_13273	7.00	lobChIP_FOXA1_robot
chr6	36491863	36491864	MACS_peak_13274	7.00	lobChIP_FOXA1_robot
chr6	36534475	36534476	MACS_peak_13275	5.00	lobChIP_FOXA1_robot
chr6	36577163	36577164	MACS_peak_13276	6.00	lobChIP_FOXA1_robot
chr6	36618849	36618850	MACS_peak_13277	4.00	lobChIP_FOXA1_robot
chr6	36651565	36651566	MACS_peak_13278	58.00	lobChIP_FOXA1_robot
chr6	36797819	36797820	MACS_peak_13279	9.00	lobChIP_FOXA1_robot
chr6	36841530	36841531	MACS_peak_13280	4.00	lobChIP_FOXA1_robot
chr6	36991328	36991329	MACS_peak_13281	38.00	lobChIP_FOXA1_robot
chr6	37051531	37051532	MACS_peak_13282	3.00	lobChIP_FOXA1_robot
chr6	37071372	37071373	MACS_peak_13283	4.00	lobChIP_FOXA1_robot
chr6	37072390	37072391	MACS_peak_13284	7.00	lobChIP_FOXA1_robot
chr6	37073082	37073083	MACS_peak_13285	5.00	lobChIP_FOXA1_robot
chr6	37078146	37078147	MACS_peak_13286	8.00	lobChIP_FOXA1_robot
chr6	37078632	37078633	MACS_peak_13287	5.00	lobChIP_FOXA1_robot
chr6	37094163	37094164	MACS_peak_13288	4.00	lobChIP_FOXA1_robot
chr6	37094668	37094669	MACS_peak_13289	4.00	lobChIP_FOXA1_robot
chr6	37104200	37104201	MACS_peak_13290	6.00	lobChIP_FOXA1_robot
chr6	37116071	37116072	MACS_peak_13291	35.00	lobChIP_FOXA1_robot
chr6	37123629	37123630	MACS_peak_13292	6.00	lobChIP_FOXA1_robot
chr6	37129093	37129094	MACS_peak_13293	5.00	lobChIP_FOXA1_robot
chr6	37137557	37137558	MACS_peak_13294	4.00	lobChIP_FOXA1_robot
chr6	37140005	37140006	MACS_peak_13295	19.00	lobChIP_FOXA1_robot
chr6	37230635	37230636	MACS_peak_13296	4.00	lobChIP_FOXA1_robot
chr6	37277812	37277813	MACS_peak_13297	6.00	lobChIP_FOXA1_robot
chr6	37307671	37307672	MACS_peak_13298	5.00	lobChIP_FOXA1_robot
chr6	37790322	37790323	MACS_peak_13299	3.00	lobChIP_FOXA1_robot
chr6	37888604	37888605	MACS_peak_13300	12.00	lobChIP_FOXA1_robot
chr6	37898622	37898623	MACS_peak_13301	5.00	lobChIP_FOXA1_robot
chr6	37956141	37956142	MACS_peak_13302	10.00	lobChIP_FOXA1_robot
chr6	37986560	37986561	MACS_peak_13303	5.00	lobChIP_FOXA1_robot
chr6	38013537	38013538	MACS_peak_13304	9.00	lobChIP_FOXA1_robot
chr6	38046681	38046682	MACS_peak_13305	5.00	lobChIP_FOXA1_robot
chr6	38386873	38386874	MACS_peak_13306	4.00	lobChIP_FOXA1_robot
chr6	38470388	38470389	MACS_peak_13307	4.00	lobChIP_FOXA1_robot
chr6	38671086	38671087	MACS_peak_13308	5.00	lobChIP_FOXA1_robot
chr6	38873585	38873586	MACS_peak_13309	5.00	lobChIP_FOXA1_robot
chr6	39399817	39399818	MACS_peak_13310	3.00	lobChIP_FOXA1_robot
chr6	39618370	39618371	MACS_peak_13311	3.00	lobChIP_FOXA1_robot
chr6	39637031	39637032	MACS_peak_13312	5.00	lobChIP_FOXA1_robot
chr6	39855707	39855708	MACS_peak_13313	7.00	lobChIP_FOXA1_robot
chr6	40682759	40682760	MACS_peak_13314	24.00	lobChIP_FOXA1_robot
chr6	40865569	40865570	MACS_peak_13315	6.00	lobChIP_FOXA1_robot
chr6	40867548	40867549	MACS_peak_13316	5.00	lobChIP_FOXA1_robot
chr6	40940766	40940767	MACS_peak_13317	9.00	lobChIP_FOXA1_robot
chr6	40965418	40965419	MACS_peak_13318	17.00	lobChIP_FOXA1_robot
chr6	41031781	41031782	MACS_peak_13319	17.00	lobChIP_FOXA1_robot
chr6	41033462	41033463	MACS_peak_13320	8.00	lobChIP_FOXA1_robot
chr6	41288419	41288420	MACS_peak_13321	8.00	lobChIP_FOXA1_robot
chr6	41424677	41424678	MACS_peak_13322	21.00	lobChIP_FOXA1_robot
chr6	41431257	41431258	MACS_peak_13323	3.00	lobChIP_FOXA1_robot
chr6	41467001	41467002	MACS_peak_13324	4.00	lobChIP_FOXA1_robot
chr6	41471876	41471877	MACS_peak_13325	4.00	lobChIP_FOXA1_robot
chr6	41485966	41485967	MACS_peak_13326	5.00	lobChIP_FOXA1_robot
chr6	41487671	41487672	MACS_peak_13327	8.00	lobChIP_FOXA1_robot
chr6	41536546	41536547	MACS_peak_13328	15.00	lobChIP_FOXA1_robot
chr6	41544015	41544016	MACS_peak_13329	5.00	lobChIP_FOXA1_robot
chr6	41546359	41546360	MACS_peak_13330	4.00	lobChIP_FOXA1_robot
chr6	41715303	41715304	MACS_peak_13331	14.00	lobChIP_FOXA1_robot
chr6	41719659	41719660	MACS_peak_13332	7.00	lobChIP_FOXA1_robot
chr6	41768079	41768080	MACS_peak_13333	3.00	lobChIP_FOXA1_robot
chr6	42078637	42078638	MACS_peak_13334	11.00	lobChIP_FOXA1_robot
chr6	42085167	42085168	MACS_peak_13335	14.00	lobChIP_FOXA1_robot
chr6	42106842	42106843	MACS_peak_13336	6.00	lobChIP_FOXA1_robot
chr6	42172175	42172176	MACS_peak_13337	25.00	lobChIP_FOXA1_robot
chr6	42194787	42194788	MACS_peak_13338	4.00	lobChIP_FOXA1_robot
chr6	42537549	42537550	MACS_peak_13339	7.00	lobChIP_FOXA1_robot
chr6	42694479	42694480	MACS_peak_13340	5.00	lobChIP_FOXA1_robot
chr6	42773430	42773431	MACS_peak_13341	5.00	lobChIP_FOXA1_robot
chr6	42813179	42813180	MACS_peak_13342	10.00	lobChIP_FOXA1_robot
chr6	42847658	42847659	MACS_peak_13343	8.00	lobChIP_FOXA1_robot
chr6	42907341	42907342	MACS_peak_13344	4.00	lobChIP_FOXA1_robot
chr6	43058058	43058059	MACS_peak_13345	20.00	lobChIP_FOXA1_robot
chr6	43235728	43235729	MACS_peak_13346	5.00	lobChIP_FOXA1_robot
chr6	43258799	43258800	MACS_peak_13347	9.00	lobChIP_FOXA1_robot
chr6	43335396	43335397	MACS_peak_13348	7.00	lobChIP_FOXA1_robot
chr6	43404759	43404760	MACS_peak_13349	7.00	lobChIP_FOXA1_robot
chr6	43444519	43444520	MACS_peak_13350	3.00	lobChIP_FOXA1_robot
chr6	43639899	43639900	MACS_peak_13351	7.00	lobChIP_FOXA1_robot
chr6	43640616	43640617	MACS_peak_13352	9.00	lobChIP_FOXA1_robot
chr6	43667509	43667510	MACS_peak_13353	7.00	lobChIP_FOXA1_robot
chr6	43672714	43672715	MACS_peak_13354	40.00	lobChIP_FOXA1_robot
chr6	43711938	43711939	MACS_peak_13355	4.00	lobChIP_FOXA1_robot
chr6	43772409	43772410	MACS_peak_13356	13.00	lobChIP_FOXA1_robot
chr6	43791979	43791980	MACS_peak_13357	5.00	lobChIP_FOXA1_robot
chr6	43816858	43816859	MACS_peak_13358	5.00	lobChIP_FOXA1_robot
chr6	43868885	43868886	MACS_peak_13359	4.00	lobChIP_FOXA1_robot
chr6	43885243	43885244	MACS_peak_13360	4.00	lobChIP_FOXA1_robot
chr6	43894962	43894963	MACS_peak_13361	5.00	lobChIP_FOXA1_robot
chr6	43958650	43958651	MACS_peak_13362	5.00	lobChIP_FOXA1_robot
chr6	43970057	43970058	MACS_peak_13363	5.00	lobChIP_FOXA1_robot
chr6	44060358	44060359	MACS_peak_13364	5.00	lobChIP_FOXA1_robot
chr6	44062625	44062626	MACS_peak_13365	4.00	lobChIP_FOXA1_robot
chr6	44067164	44067165	MACS_peak_13366	6.00	lobChIP_FOXA1_robot
chr6	44079859	44079860	MACS_peak_13367	6.00	lobChIP_FOXA1_robot
chr6	44090665	44090666	MACS_peak_13368	3.00	lobChIP_FOXA1_robot
chr6	44101671	44101672	MACS_peak_13369	5.00	lobChIP_FOXA1_robot
chr6	44124314	44124315	MACS_peak_13370	20.00	lobChIP_FOXA1_robot
chr6	44205464	44205465	MACS_peak_13371	5.00	lobChIP_FOXA1_robot
chr6	44349761	44349762	MACS_peak_13372	4.00	lobChIP_FOXA1_robot
chr6	44358892	44358893	MACS_peak_13373	3.00	lobChIP_FOXA1_robot
chr6	44414532	44414533	MACS_peak_13374	9.00	lobChIP_FOXA1_robot
chr6	44888924	44888925	MACS_peak_13375	6.00	lobChIP_FOXA1_robot
chr6	44908406	44908407	MACS_peak_13376	15.00	lobChIP_FOXA1_robot
chr6	44919071	44919072	MACS_peak_13377	8.00	lobChIP_FOXA1_robot
chr6	45090889	45090890	MACS_peak_13378	4.00	lobChIP_FOXA1_robot
chr6	45100810	45100811	MACS_peak_13379	6.00	lobChIP_FOXA1_robot
chr6	45233085	45233086	MACS_peak_13380	4.00	lobChIP_FOXA1_robot
chr6	45401431	45401432	MACS_peak_13381	6.00	lobChIP_FOXA1_robot
chr6	45413780	45413781	MACS_peak_13382	12.00	lobChIP_FOXA1_robot
chr6	45710989	45710990	MACS_peak_13383	4.00	lobChIP_FOXA1_robot
chr6	46617075	46617076	MACS_peak_13384	5.00	lobChIP_FOXA1_robot
chr6	46760953	46760954	MACS_peak_13385	3.00	lobChIP_FOXA1_robot
chr6	47015465	47015466	MACS_peak_13386	4.00	lobChIP_FOXA1_robot
chr6	47041093	47041094	MACS_peak_13387	52.00	lobChIP_FOXA1_robot
chr6	47043978	47043979	MACS_peak_13388	17.00	lobChIP_FOXA1_robot
chr6	47051794	47051795	MACS_peak_13389	5.00	lobChIP_FOXA1_robot
chr6	47062665	47062666	MACS_peak_13390	9.00	lobChIP_FOXA1_robot
chr6	47079566	47079567	MACS_peak_13391	7.00	lobChIP_FOXA1_robot
chr6	47095028	47095029	MACS_peak_13392	5.00	lobChIP_FOXA1_robot
chr6	47102637	47102638	MACS_peak_13393	14.00	lobChIP_FOXA1_robot
chr6	47103624	47103625	MACS_peak_13394	9.00	lobChIP_FOXA1_robot
chr6	47104902	47104903	MACS_peak_13395	4.00	lobChIP_FOXA1_robot
chr6	47115345	47115346	MACS_peak_13396	6.00	lobChIP_FOXA1_robot
chr6	47148522	47148523	MACS_peak_13397	6.00	lobChIP_FOXA1_robot
chr6	47330802	47330803	MACS_peak_13398	6.00	lobChIP_FOXA1_robot
chr6	47428260	47428261	MACS_peak_13399	5.00	lobChIP_FOXA1_robot
chr6	47459132	47459133	MACS_peak_13400	3.00	lobChIP_FOXA1_robot
chr6	48035319	48035320	MACS_peak_13401	4.00	lobChIP_FOXA1_robot
chr6	48048115	48048116	MACS_peak_13402	7.00	lobChIP_FOXA1_robot
chr6	48079043	48079044	MACS_peak_13403	5.00	lobChIP_FOXA1_robot
chr6	48646434	48646435	MACS_peak_13404	4.00	lobChIP_FOXA1_robot
chr6	49064694	49064695	MACS_peak_13405	5.00	lobChIP_FOXA1_robot
chr6	49395678	49395679	MACS_peak_13406	4.00	lobChIP_FOXA1_robot
chr6	49421011	49421012	MACS_peak_13407	8.00	lobChIP_FOXA1_robot
chr6	49481229	49481230	MACS_peak_13408	6.00	lobChIP_FOXA1_robot
chr6	49489756	49489757	MACS_peak_13409	5.00	lobChIP_FOXA1_robot
chr6	49490807	49490808	MACS_peak_13410	68.00	lobChIP_FOXA1_robot
chr6	49509523	49509524	MACS_peak_13411	3.00	lobChIP_FOXA1_robot
chr6	49524423	49524424	MACS_peak_13412	15.00	lobChIP_FOXA1_robot
chr6	49734705	49734706	MACS_peak_13413	5.00	lobChIP_FOXA1_robot
chr6	50061813	50061814	MACS_peak_13414	18.00	lobChIP_FOXA1_robot
chr6	50366840	50366841	MACS_peak_13415	4.00	lobChIP_FOXA1_robot
chr6	50374468	50374469	MACS_peak_13416	4.00	lobChIP_FOXA1_robot
chr6	51540989	51540990	MACS_peak_13417	6.00	lobChIP_FOXA1_robot
chr6	51884936	51884937	MACS_peak_13418	3.00	lobChIP_FOXA1_robot
chr6	52108253	52108254	MACS_peak_13419	7.00	lobChIP_FOXA1_robot
chr6	52114129	52114130	MACS_peak_13420	6.00	lobChIP_FOXA1_robot
chr6	52136799	52136800	MACS_peak_13421	6.00	lobChIP_FOXA1_robot
chr6	52190858	52190859	MACS_peak_13422	9.00	lobChIP_FOXA1_robot
chr6	52193677	52193678	MACS_peak_13423	7.00	lobChIP_FOXA1_robot
chr6	52217424	52217425	MACS_peak_13424	7.00	lobChIP_FOXA1_robot
chr6	52234236	52234237	MACS_peak_13425	13.00	lobChIP_FOXA1_robot
chr6	52236507	52236508	MACS_peak_13426	5.00	lobChIP_FOXA1_robot
chr6	52254270	52254271	MACS_peak_13427	6.00	lobChIP_FOXA1_robot
chr6	52382904	52382905	MACS_peak_13428	5.00	lobChIP_FOXA1_robot
chr6	52409036	52409037	MACS_peak_13429	19.00	lobChIP_FOXA1_robot
chr6	52454024	52454025	MACS_peak_13430	8.00	lobChIP_FOXA1_robot
chr6	52461313	52461314	MACS_peak_13431	12.00	lobChIP_FOXA1_robot
chr6	52463262	52463263	MACS_peak_13432	6.00	lobChIP_FOXA1_robot
chr6	52481256	52481257	MACS_peak_13433	4.00	lobChIP_FOXA1_robot
chr6	52499038	52499039	MACS_peak_13434	4.00	lobChIP_FOXA1_robot
chr6	52590683	52590684	MACS_peak_13435	97.00	lobChIP_FOXA1_robot
chr6	52610649	52610650	MACS_peak_13436	5.00	lobChIP_FOXA1_robot
chr6	52652991	52652992	MACS_peak_13437	3.00	lobChIP_FOXA1_robot
chr6	52680195	52680196	MACS_peak_13438	4.00	lobChIP_FOXA1_robot
chr6	52914454	52914455	MACS_peak_13439	4.00	lobChIP_FOXA1_robot
chr6	52984823	52984824	MACS_peak_13440	10.00	lobChIP_FOXA1_robot
chr6	52999853	52999854	MACS_peak_13441	7.00	lobChIP_FOXA1_robot
chr6	53167057	53167058	MACS_peak_13442	13.00	lobChIP_FOXA1_robot
chr6	53171756	53171757	MACS_peak_13443	3.00	lobChIP_FOXA1_robot
chr6	53186690	53186691	MACS_peak_13444	11.00	lobChIP_FOXA1_robot
chr6	53198816	53198817	MACS_peak_13445	7.00	lobChIP_FOXA1_robot
chr6	53222611	53222612	MACS_peak_13446	9.00	lobChIP_FOXA1_robot
chr6	53275572	53275573	MACS_peak_13447	3.00	lobChIP_FOXA1_robot
chr6	53297526	53297527	MACS_peak_13448	4.00	lobChIP_FOXA1_robot
chr6	53298519	53298520	MACS_peak_13449	6.00	lobChIP_FOXA1_robot
chr6	53480534	53480535	MACS_peak_13450	6.00	lobChIP_FOXA1_robot
chr6	53499044	53499045	MACS_peak_13451	16.00	lobChIP_FOXA1_robot
chr6	53504478	53504479	MACS_peak_13452	4.00	lobChIP_FOXA1_robot
chr6	53517937	53517938	MACS_peak_13453	6.00	lobChIP_FOXA1_robot
chr6	53679294	53679295	MACS_peak_13454	16.00	lobChIP_FOXA1_robot
chr6	53696593	53696594	MACS_peak_13455	3.00	lobChIP_FOXA1_robot
chr6	53719727	53719728	MACS_peak_13456	4.00	lobChIP_FOXA1_robot
chr6	53803537	53803538	MACS_peak_13457	4.00	lobChIP_FOXA1_robot
chr6	54000915	54000916	MACS_peak_13458	9.00	lobChIP_FOXA1_robot
chr6	54387760	54387761	MACS_peak_13459	4.00	lobChIP_FOXA1_robot
chr6	54634863	54634864	MACS_peak_13460	4.00	lobChIP_FOXA1_robot
chr6	54794536	54794537	MACS_peak_13461	20.00	lobChIP_FOXA1_robot
chr6	55692894	55692895	MACS_peak_13462	4.00	lobChIP_FOXA1_robot
chr6	55888173	55888174	MACS_peak_13463	3.00	lobChIP_FOXA1_robot
chr6	56245497	56245498	MACS_peak_13464	6.00	lobChIP_FOXA1_robot
chr6	56264666	56264667	MACS_peak_13465	49.00	lobChIP_FOXA1_robot
chr6	56347558	56347559	MACS_peak_13466	6.00	lobChIP_FOXA1_robot
chr6	56376275	56376276	MACS_peak_13467	4.00	lobChIP_FOXA1_robot
chr6	56386366	56386367	MACS_peak_13468	14.00	lobChIP_FOXA1_robot
chr6	56390826	56390827	MACS_peak_13469	4.00	lobChIP_FOXA1_robot
chr6	56406423	56406424	MACS_peak_13470	5.00	lobChIP_FOXA1_robot
chr6	56406875	56406876	MACS_peak_13471	5.00	lobChIP_FOXA1_robot
chr6	56489758	56489759	MACS_peak_13472	9.00	lobChIP_FOXA1_robot
chr6	56507368	56507369	MACS_peak_13473	6.00	lobChIP_FOXA1_robot
chr6	56507880	56507881	MACS_peak_13474	5.00	lobChIP_FOXA1_robot
chr6	56517556	56517557	MACS_peak_13475	3.00	lobChIP_FOXA1_robot
chr6	56548953	56548954	MACS_peak_13476	46.00	lobChIP_FOXA1_robot
chr6	56550012	56550013	MACS_peak_13477	7.00	lobChIP_FOXA1_robot
chr6	56551363	56551364	MACS_peak_13478	7.00	lobChIP_FOXA1_robot
chr6	56592744	56592745	MACS_peak_13479	4.00	lobChIP_FOXA1_robot
chr6	56593840	56593841	MACS_peak_13480	15.00	lobChIP_FOXA1_robot
chr6	56659984	56659985	MACS_peak_13481	4.00	lobChIP_FOXA1_robot
chr6	56686060	56686061	MACS_peak_13482	11.00	lobChIP_FOXA1_robot
chr6	56733664	56733665	MACS_peak_13483	9.00	lobChIP_FOXA1_robot
chr6	56844095	56844096	MACS_peak_13484	3.00	lobChIP_FOXA1_robot
chr6	56943668	56943669	MACS_peak_13485	4.00	lobChIP_FOXA1_robot
chr6	56951793	56951794	MACS_peak_13486	3.00	lobChIP_FOXA1_robot
chr6	56971917	56971918	MACS_peak_13487	4.00	lobChIP_FOXA1_robot
chr6	56992785	56992786	MACS_peak_13488	6.00	lobChIP_FOXA1_robot
chr6	57006717	57006718	MACS_peak_13489	6.00	lobChIP_FOXA1_robot
chr6	57008216	57008217	MACS_peak_13490	5.00	lobChIP_FOXA1_robot
chr6	57117875	57117876	MACS_peak_13491	4.00	lobChIP_FOXA1_robot
chr6	57236810	57236811	MACS_peak_13492	15.00	lobChIP_FOXA1_robot
chr6	57336009	57336010	MACS_peak_13493	4.00	lobChIP_FOXA1_robot
chr6	57361381	57361382	MACS_peak_13494	5.00	lobChIP_FOXA1_robot
chr6	57365692	57365693	MACS_peak_13495	24.00	lobChIP_FOXA1_robot
chr6	57366726	57366727	MACS_peak_13496	5.00	lobChIP_FOXA1_robot
chr6	57445499	57445500	MACS_peak_13497	3.00	lobChIP_FOXA1_robot
chr6	57533733	57533734	MACS_peak_13498	5.00	lobChIP_FOXA1_robot
chr6	58506242	58506243	MACS_peak_13499	3.00	lobChIP_FOXA1_robot
chr6	58526803	58526804	MACS_peak_13500	5.00	lobChIP_FOXA1_robot
chr6	61947979	61947980	MACS_peak_13502	5.00	lobChIP_FOXA1_robot
chr6	64101566	64101567	MACS_peak_13503	17.00	lobChIP_FOXA1_robot
chr6	64121385	64121386	MACS_peak_13504	3.00	lobChIP_FOXA1_robot
chr6	64158300	64158301	MACS_peak_13505	7.00	lobChIP_FOXA1_robot
chr6	64162104	64162105	MACS_peak_13506	5.00	lobChIP_FOXA1_robot
chr6	64168705	64168706	MACS_peak_13507	41.00	lobChIP_FOXA1_robot
chr6	64220257	64220258	MACS_peak_13508	13.00	lobChIP_FOXA1_robot
chr6	64224004	64224005	MACS_peak_13509	22.00	lobChIP_FOXA1_robot
chr6	64224596	64224597	MACS_peak_13510	16.00	lobChIP_FOXA1_robot
chr6	64226334	64226335	MACS_peak_13511	14.00	lobChIP_FOXA1_robot
chr6	64231398	64231399	MACS_peak_13512	4.00	lobChIP_FOXA1_robot
chr6	64232613	64232614	MACS_peak_13513	4.00	lobChIP_FOXA1_robot
chr6	64237774	64237775	MACS_peak_13514	6.00	lobChIP_FOXA1_robot
chr6	64271007	64271008	MACS_peak_13515	19.00	lobChIP_FOXA1_robot
chr6	64275602	64275603	MACS_peak_13516	18.00	lobChIP_FOXA1_robot
chr6	64299882	64299883	MACS_peak_13517	7.00	lobChIP_FOXA1_robot
chr6	64387309	64387310	MACS_peak_13518	4.00	lobChIP_FOXA1_robot
chr6	64434854	64434855	MACS_peak_13519	6.00	lobChIP_FOXA1_robot
chr6	64516281	64516282	MACS_peak_13520	7.00	lobChIP_FOXA1_robot
chr6	64794194	64794195	MACS_peak_13521	7.00	lobChIP_FOXA1_robot
chr6	64968898	64968899	MACS_peak_13522	6.00	lobChIP_FOXA1_robot
chr6	65011694	65011695	MACS_peak_13523	31.00	lobChIP_FOXA1_robot
chr6	65013884	65013885	MACS_peak_13524	7.00	lobChIP_FOXA1_robot
chr6	66109639	66109640	MACS_peak_13525	4.00	lobChIP_FOXA1_robot
chr6	66909346	66909347	MACS_peak_13526	3.00	lobChIP_FOXA1_robot
chr6	67859609	67859610	MACS_peak_13527	3.00	lobChIP_FOXA1_robot
chr6	70952071	70952072	MACS_peak_13528	8.00	lobChIP_FOXA1_robot
chr6	71109129	71109130	MACS_peak_13529	6.00	lobChIP_FOXA1_robot
chr6	71121390	71121391	MACS_peak_13530	5.00	lobChIP_FOXA1_robot
chr6	71136701	71136702	MACS_peak_13531	10.00	lobChIP_FOXA1_robot
chr6	71741818	71741819	MACS_peak_13532	6.00	lobChIP_FOXA1_robot
chr6	74225772	74225773	MACS_peak_13533	4.00	lobChIP_FOXA1_robot
chr6	74234003	74234004	MACS_peak_13534	18.00	lobChIP_FOXA1_robot
chr6	74258590	74258591	MACS_peak_13535	5.00	lobChIP_FOXA1_robot
chr6	74270266	74270267	MACS_peak_13536	3.00	lobChIP_FOXA1_robot
chr6	74289911	74289912	MACS_peak_13537	5.00	lobChIP_FOXA1_robot
chr6	74290721	74290722	MACS_peak_13538	7.00	lobChIP_FOXA1_robot
chr6	74295438	74295439	MACS_peak_13539	7.00	lobChIP_FOXA1_robot
chr6	74358109	74358110	MACS_peak_13540	6.00	lobChIP_FOXA1_robot
chr6	74362455	74362456	MACS_peak_13541	5.00	lobChIP_FOXA1_robot
chr6	74405566	74405567	MACS_peak_13542	6.00	lobChIP_FOXA1_robot
chr6	75244416	75244417	MACS_peak_13543	71.00	lobChIP_FOXA1_robot
chr6	76002190	76002191	MACS_peak_13544	3.00	lobChIP_FOXA1_robot
chr6	76234249	76234250	MACS_peak_13545	7.00	lobChIP_FOXA1_robot
chr6	76313003	76313004	MACS_peak_13546	5.00	lobChIP_FOXA1_robot
chr6	78195883	78195884	MACS_peak_13547	3.00	lobChIP_FOXA1_robot
chr6	78202479	78202480	MACS_peak_13548	4.00	lobChIP_FOXA1_robot
chr6	78544450	78544451	MACS_peak_13549	22.00	lobChIP_FOXA1_robot
chr6	79867741	79867742	MACS_peak_13550	8.00	lobChIP_FOXA1_robot
chr6	80235738	80235739	MACS_peak_13551	6.00	lobChIP_FOXA1_robot
chr6	80268011	80268012	MACS_peak_13552	4.00	lobChIP_FOXA1_robot
chr6	80318703	80318704	MACS_peak_13553	3.00	lobChIP_FOXA1_robot
chr6	80327308	80327309	MACS_peak_13554	11.00	lobChIP_FOXA1_robot
chr6	80382592	80382593	MACS_peak_13555	10.00	lobChIP_FOXA1_robot
chr6	80398384	80398385	MACS_peak_13556	4.00	lobChIP_FOXA1_robot
chr6	81237391	81237392	MACS_peak_13557	8.00	lobChIP_FOXA1_robot
chr6	82201326	82201327	MACS_peak_13558	4.00	lobChIP_FOXA1_robot
chr6	82541109	82541110	MACS_peak_13559	4.00	lobChIP_FOXA1_robot
chr6	82572874	82572875	MACS_peak_13560	4.00	lobChIP_FOXA1_robot
chr6	82574034	82574035	MACS_peak_13561	17.00	lobChIP_FOXA1_robot
chr6	82794395	82794396	MACS_peak_13562	12.00	lobChIP_FOXA1_robot
chr6	82796763	82796764	MACS_peak_13563	4.00	lobChIP_FOXA1_robot
chr6	82807553	82807554	MACS_peak_13564	6.00	lobChIP_FOXA1_robot
chr6	83093417	83093418	MACS_peak_13565	3.00	lobChIP_FOXA1_robot
chr6	83718613	83718614	MACS_peak_13566	7.00	lobChIP_FOXA1_robot
chr6	84979929	84979930	MACS_peak_13567	4.00	lobChIP_FOXA1_robot
chr6	85282278	85282279	MACS_peak_13568	5.00	lobChIP_FOXA1_robot
chr6	86106127	86106128	MACS_peak_13569	5.00	lobChIP_FOXA1_robot
chr6	86115150	86115151	MACS_peak_13570	19.00	lobChIP_FOXA1_robot
chr6	86161817	86161818	MACS_peak_13571	5.00	lobChIP_FOXA1_robot
chr6	86290407	86290408	MACS_peak_13572	5.00	lobChIP_FOXA1_robot
chr6	86457624	86457625	MACS_peak_13573	5.00	lobChIP_FOXA1_robot
chr6	87809682	87809683	MACS_peak_13574	4.00	lobChIP_FOXA1_robot
chr6	88052700	88052701	MACS_peak_13575	8.00	lobChIP_FOXA1_robot
chr6	88195892	88195893	MACS_peak_13576	6.00	lobChIP_FOXA1_robot
chr6	88244261	88244262	MACS_peak_13577	13.00	lobChIP_FOXA1_robot
chr6	88245275	88245276	MACS_peak_13578	5.00	lobChIP_FOXA1_robot
chr6	88283513	88283514	MACS_peak_13579	15.00	lobChIP_FOXA1_robot
chr6	88293774	88293775	MACS_peak_13580	18.00	lobChIP_FOXA1_robot
chr6	88376986	88376987	MACS_peak_13581	4.00	lobChIP_FOXA1_robot
chr6	88379136	88379137	MACS_peak_13582	4.00	lobChIP_FOXA1_robot
chr6	88381658	88381659	MACS_peak_13583	14.00	lobChIP_FOXA1_robot
chr6	88382539	88382540	MACS_peak_13584	6.00	lobChIP_FOXA1_robot
chr6	88415174	88415175	MACS_peak_13585	5.00	lobChIP_FOXA1_robot
chr6	88433128	88433129	MACS_peak_13586	4.00	lobChIP_FOXA1_robot
chr6	88441735	88441736	MACS_peak_13587	4.00	lobChIP_FOXA1_robot
chr6	88482722	88482723	MACS_peak_13588	6.00	lobChIP_FOXA1_robot
chr6	88495093	88495094	MACS_peak_13589	28.00	lobChIP_FOXA1_robot
chr6	88508687	88508688	MACS_peak_13590	11.00	lobChIP_FOXA1_robot
chr6	88516678	88516679	MACS_peak_13591	12.00	lobChIP_FOXA1_robot
chr6	88530459	88530460	MACS_peak_13592	4.00	lobChIP_FOXA1_robot
chr6	88531775	88531776	MACS_peak_13593	12.00	lobChIP_FOXA1_robot
chr6	89419794	89419795	MACS_peak_13594	15.00	lobChIP_FOXA1_robot
chr6	89533904	89533905	MACS_peak_13595	5.00	lobChIP_FOXA1_robot
chr6	89545173	89545174	MACS_peak_13596	5.00	lobChIP_FOXA1_robot
chr6	89682933	89682934	MACS_peak_13597	25.00	lobChIP_FOXA1_robot
chr6	89695874	89695875	MACS_peak_13598	14.00	lobChIP_FOXA1_robot
chr6	89716696	89716697	MACS_peak_13599	6.00	lobChIP_FOXA1_robot
chr6	89744860	89744861	MACS_peak_13600	8.00	lobChIP_FOXA1_robot
chr6	89780992	89780993	MACS_peak_13601	12.00	lobChIP_FOXA1_robot
chr6	89850208	89850209	MACS_peak_13602	7.00	lobChIP_FOXA1_robot
chr6	90277718	90277719	MACS_peak_13603	6.00	lobChIP_FOXA1_robot
chr6	90650325	90650326	MACS_peak_13604	13.00	lobChIP_FOXA1_robot
chr6	90651287	90651288	MACS_peak_13605	5.00	lobChIP_FOXA1_robot
chr6	90813366	90813367	MACS_peak_13606	7.00	lobChIP_FOXA1_robot
chr6	91306429	91306430	MACS_peak_13607	5.00	lobChIP_FOXA1_robot
chr6	93074894	93074895	MACS_peak_13608	17.00	lobChIP_FOXA1_robot
chr6	96022922	96022923	MACS_peak_13609	6.00	lobChIP_FOXA1_robot
chr6	96253067	96253068	MACS_peak_13610	37.00	lobChIP_FOXA1_robot
chr6	96253794	96253795	MACS_peak_13611	4.00	lobChIP_FOXA1_robot
chr6	96263602	96263603	MACS_peak_13612	11.00	lobChIP_FOXA1_robot
chr6	96325684	96325685	MACS_peak_13613	10.00	lobChIP_FOXA1_robot
chr6	96455762	96455763	MACS_peak_13614	4.00	lobChIP_FOXA1_robot
chr6	97584447	97584448	MACS_peak_13615	4.00	lobChIP_FOXA1_robot
chr6	98740930	98740931	MACS_peak_13616	5.00	lobChIP_FOXA1_robot
chr6	99118361	99118362	MACS_peak_13617	5.00	lobChIP_FOXA1_robot
chr6	99392533	99392534	MACS_peak_13618	28.00	lobChIP_FOXA1_robot
chr6	101310216	101310217	MACS_peak_13619	6.00	lobChIP_FOXA1_robot
chr6	101396796	101396797	MACS_peak_13620	10.00	lobChIP_FOXA1_robot
chr6	102523038	102523039	MACS_peak_13621	4.00	lobChIP_FOXA1_robot
chr6	102574554	102574555	MACS_peak_13622	7.00	lobChIP_FOXA1_robot
chr6	102820986	102820987	MACS_peak_13623	6.00	lobChIP_FOXA1_robot
chr6	103949316	103949317	MACS_peak_13624	5.00	lobChIP_FOXA1_robot
chr6	105483342	105483343	MACS_peak_13625	6.00	lobChIP_FOXA1_robot
chr6	105514307	105514308	MACS_peak_13626	24.00	lobChIP_FOXA1_robot
chr6	105822047	105822048	MACS_peak_13627	9.00	lobChIP_FOXA1_robot
chr6	105876761	105876762	MACS_peak_13628	6.00	lobChIP_FOXA1_robot
chr6	106344279	106344280	MACS_peak_13629	5.00	lobChIP_FOXA1_robot
chr6	106416705	106416706	MACS_peak_13630	6.00	lobChIP_FOXA1_robot
chr6	106503545	106503546	MACS_peak_13631	8.00	lobChIP_FOXA1_robot
chr6	106709814	106709815	MACS_peak_13632	6.00	lobChIP_FOXA1_robot
chr6	106911185	106911186	MACS_peak_13633	7.00	lobChIP_FOXA1_robot
chr6	106988573	106988574	MACS_peak_13634	13.00	lobChIP_FOXA1_robot
chr6	106995976	106995977	MACS_peak_13635	5.00	lobChIP_FOXA1_robot
chr6	107005921	107005922	MACS_peak_13636	5.00	lobChIP_FOXA1_robot
chr6	107006483	107006484	MACS_peak_13637	5.00	lobChIP_FOXA1_robot
chr6	107065940	107065941	MACS_peak_13638	15.00	lobChIP_FOXA1_robot
chr6	107070367	107070368	MACS_peak_13639	14.00	lobChIP_FOXA1_robot
chr6	107150499	107150500	MACS_peak_13640	7.00	lobChIP_FOXA1_robot
chr6	107169394	107169395	MACS_peak_13641	5.00	lobChIP_FOXA1_robot
chr6	107246423	107246424	MACS_peak_13642	7.00	lobChIP_FOXA1_robot
chr6	107533109	107533110	MACS_peak_13643	5.00	lobChIP_FOXA1_robot
chr6	107556397	107556398	MACS_peak_13644	5.00	lobChIP_FOXA1_robot
chr6	107585841	107585842	MACS_peak_13645	30.00	lobChIP_FOXA1_robot
chr6	107588293	107588294	MACS_peak_13646	7.00	lobChIP_FOXA1_robot
chr6	107636140	107636141	MACS_peak_13647	8.00	lobChIP_FOXA1_robot
chr6	107653082	107653083	MACS_peak_13648	5.00	lobChIP_FOXA1_robot
chr6	107814570	107814571	MACS_peak_13649	8.00	lobChIP_FOXA1_robot
chr6	107860523	107860524	MACS_peak_13650	4.00	lobChIP_FOXA1_robot
chr6	107861747	107861748	MACS_peak_13651	20.00	lobChIP_FOXA1_robot
chr6	107909078	107909079	MACS_peak_13652	4.00	lobChIP_FOXA1_robot
chr6	107987861	107987862	MACS_peak_13653	6.00	lobChIP_FOXA1_robot
chr6	108148415	108148416	MACS_peak_13654	6.00	lobChIP_FOXA1_robot
chr6	108253309	108253310	MACS_peak_13655	10.00	lobChIP_FOXA1_robot
chr6	108274021	108274022	MACS_peak_13656	19.00	lobChIP_FOXA1_robot
chr6	108323134	108323135	MACS_peak_13657	7.00	lobChIP_FOXA1_robot
chr6	108449299	108449300	MACS_peak_13658	9.00	lobChIP_FOXA1_robot
chr6	108684481	108684482	MACS_peak_13659	4.00	lobChIP_FOXA1_robot
chr6	108786039	108786040	MACS_peak_13660	4.00	lobChIP_FOXA1_robot
chr6	109028687	109028688	MACS_peak_13661	7.00	lobChIP_FOXA1_robot
chr6	109042606	109042607	MACS_peak_13662	5.00	lobChIP_FOXA1_robot
chr6	109050321	109050322	MACS_peak_13663	5.00	lobChIP_FOXA1_robot
chr6	109063000	109063001	MACS_peak_13664	4.00	lobChIP_FOXA1_robot
chr6	109065909	109065910	MACS_peak_13665	3.00	lobChIP_FOXA1_robot
chr6	109074163	109074164	MACS_peak_13666	6.00	lobChIP_FOXA1_robot
chr6	109085312	109085313	MACS_peak_13667	8.00	lobChIP_FOXA1_robot
chr6	109225222	109225223	MACS_peak_13668	44.00	lobChIP_FOXA1_robot
chr6	109371778	109371779	MACS_peak_13669	5.00	lobChIP_FOXA1_robot
chr6	109372650	109372651	MACS_peak_13670	7.00	lobChIP_FOXA1_robot
chr6	109485458	109485459	MACS_peak_13671	5.00	lobChIP_FOXA1_robot
chr6	109573618	109573619	MACS_peak_13672	5.00	lobChIP_FOXA1_robot
chr6	109579526	109579527	MACS_peak_13673	4.00	lobChIP_FOXA1_robot
chr6	109616089	109616090	MACS_peak_13674	4.00	lobChIP_FOXA1_robot
chr6	109639658	109639659	MACS_peak_13675	5.00	lobChIP_FOXA1_robot
chr6	109705568	109705569	MACS_peak_13676	8.00	lobChIP_FOXA1_robot
chr6	110651100	110651101	MACS_peak_13677	4.00	lobChIP_FOXA1_robot
chr6	110682096	110682097	MACS_peak_13678	7.00	lobChIP_FOXA1_robot
chr6	110876320	110876321	MACS_peak_13679	21.00	lobChIP_FOXA1_robot
chr6	110975472	110975473	MACS_peak_13680	21.00	lobChIP_FOXA1_robot
chr6	111002944	111002945	MACS_peak_13681	9.00	lobChIP_FOXA1_robot
chr6	111025333	111025334	MACS_peak_13682	39.00	lobChIP_FOXA1_robot
chr6	111068603	111068604	MACS_peak_13683	4.00	lobChIP_FOXA1_robot
chr6	111076669	111076670	MACS_peak_13684	3.00	lobChIP_FOXA1_robot
chr6	111198431	111198432	MACS_peak_13685	5.00	lobChIP_FOXA1_robot
chr6	111200589	111200590	MACS_peak_13686	5.00	lobChIP_FOXA1_robot
chr6	111273591	111273592	MACS_peak_13687	7.00	lobChIP_FOXA1_robot
chr6	111426878	111426879	MACS_peak_13688	5.00	lobChIP_FOXA1_robot
chr6	111429044	111429045	MACS_peak_13689	16.00	lobChIP_FOXA1_robot
chr6	111445979	111445980	MACS_peak_13690	3.00	lobChIP_FOXA1_robot
chr6	111659613	111659614	MACS_peak_13691	4.00	lobChIP_FOXA1_robot
chr6	111677853	111677854	MACS_peak_13692	3.00	lobChIP_FOXA1_robot
chr6	111694935	111694936	MACS_peak_13693	5.00	lobChIP_FOXA1_robot
chr6	111784493	111784494	MACS_peak_13694	3.00	lobChIP_FOXA1_robot
chr6	112351894	112351895	MACS_peak_13695	4.00	lobChIP_FOXA1_robot
chr6	112364302	112364303	MACS_peak_13696	5.00	lobChIP_FOXA1_robot
chr6	112795358	112795359	MACS_peak_13697	3.00	lobChIP_FOXA1_robot
chr6	112894587	112894588	MACS_peak_13698	8.00	lobChIP_FOXA1_robot
chr6	113433233	113433234	MACS_peak_13699	3.00	lobChIP_FOXA1_robot
chr6	113673585	113673586	MACS_peak_13700	32.00	lobChIP_FOXA1_robot
chr6	113674217	113674218	MACS_peak_13701	4.00	lobChIP_FOXA1_robot
chr6	113682223	113682224	MACS_peak_13702	5.00	lobChIP_FOXA1_robot
chr6	113686444	113686445	MACS_peak_13703	5.00	lobChIP_FOXA1_robot
chr6	113811745	113811746	MACS_peak_13704	5.00	lobChIP_FOXA1_robot
chr6	113885755	113885756	MACS_peak_13705	4.00	lobChIP_FOXA1_robot
chr6	113964323	113964324	MACS_peak_13706	51.00	lobChIP_FOXA1_robot
chr6	113990453	113990454	MACS_peak_13707	10.00	lobChIP_FOXA1_robot
chr6	114196764	114196765	MACS_peak_13708	3.00	lobChIP_FOXA1_robot
chr6	114466261	114466262	MACS_peak_13709	6.00	lobChIP_FOXA1_robot
chr6	114481166	114481167	MACS_peak_13710	10.00	lobChIP_FOXA1_robot
chr6	114489115	114489116	MACS_peak_13711	33.00	lobChIP_FOXA1_robot
chr6	114492148	114492149	MACS_peak_13712	19.00	lobChIP_FOXA1_robot
chr6	114593936	114593937	MACS_peak_13713	3.00	lobChIP_FOXA1_robot
chr6	115834003	115834004	MACS_peak_13714	5.00	lobChIP_FOXA1_robot
chr6	116258805	116258806	MACS_peak_13715	8.00	lobChIP_FOXA1_robot
chr6	116303778	116303779	MACS_peak_13716	6.00	lobChIP_FOXA1_robot
chr6	116338144	116338145	MACS_peak_13717	8.00	lobChIP_FOXA1_robot
chr6	116392321	116392322	MACS_peak_13718	20.00	lobChIP_FOXA1_robot
chr6	116393635	116393636	MACS_peak_13719	8.00	lobChIP_FOXA1_robot
chr6	116395403	116395404	MACS_peak_13720	5.00	lobChIP_FOXA1_robot
chr6	116449365	116449366	MACS_peak_13721	5.00	lobChIP_FOXA1_robot
chr6	116467491	116467492	MACS_peak_13722	7.00	lobChIP_FOXA1_robot
chr6	116849923	116849924	MACS_peak_13723	3.00	lobChIP_FOXA1_robot
chr6	116872078	116872079	MACS_peak_13724	9.00	lobChIP_FOXA1_robot
chr6	116952821	116952822	MACS_peak_13725	6.00	lobChIP_FOXA1_robot
chr6	117163650	117163651	MACS_peak_13726	7.00	lobChIP_FOXA1_robot
chr6	117276229	117276230	MACS_peak_13727	7.00	lobChIP_FOXA1_robot
chr6	117453473	117453474	MACS_peak_13728	3.00	lobChIP_FOXA1_robot
chr6	117763151	117763152	MACS_peak_13729	19.00	lobChIP_FOXA1_robot
chr6	117802547	117802548	MACS_peak_13730	10.00	lobChIP_FOXA1_robot
chr6	117825922	117825923	MACS_peak_13731	4.00	lobChIP_FOXA1_robot
chr6	117827714	117827715	MACS_peak_13732	4.00	lobChIP_FOXA1_robot
chr6	117836740	117836741	MACS_peak_13733	9.00	lobChIP_FOXA1_robot
chr6	117866431	117866432	MACS_peak_13734	4.00	lobChIP_FOXA1_robot
chr6	117870131	117870132	MACS_peak_13735	5.00	lobChIP_FOXA1_robot
chr6	117932224	117932225	MACS_peak_13736	4.00	lobChIP_FOXA1_robot
chr6	118025638	118025639	MACS_peak_13737	4.00	lobChIP_FOXA1_robot
chr6	118790451	118790452	MACS_peak_13738	8.00	lobChIP_FOXA1_robot
chr6	119197055	119197056	MACS_peak_13739	11.00	lobChIP_FOXA1_robot
chr6	119244448	119244449	MACS_peak_13740	4.00	lobChIP_FOXA1_robot
chr6	119451269	119451270	MACS_peak_13741	30.00	lobChIP_FOXA1_robot
chr6	119468772	119468773	MACS_peak_13742	5.00	lobChIP_FOXA1_robot
chr6	119483707	119483708	MACS_peak_13743	3.00	lobChIP_FOXA1_robot
chr6	119491780	119491781	MACS_peak_13744	24.00	lobChIP_FOXA1_robot
chr6	119494906	119494907	MACS_peak_13745	5.00	lobChIP_FOXA1_robot
chr6	119536661	119536662	MACS_peak_13746	5.00	lobChIP_FOXA1_robot
chr6	119580319	119580320	MACS_peak_13747	4.00	lobChIP_FOXA1_robot
chr6	119582602	119582603	MACS_peak_13748	161.00	lobChIP_FOXA1_robot
chr6	119667353	119667354	MACS_peak_13749	4.00	lobChIP_FOXA1_robot
chr6	119668982	119668983	MACS_peak_13750	19.00	lobChIP_FOXA1_robot
chr6	119821163	119821164	MACS_peak_13751	5.00	lobChIP_FOXA1_robot
chr6	120323576	120323577	MACS_peak_13752	6.00	lobChIP_FOXA1_robot
chr6	120775240	120775241	MACS_peak_13753	4.00	lobChIP_FOXA1_robot
chr6	121106464	121106465	MACS_peak_13754	4.00	lobChIP_FOXA1_robot
chr6	121751742	121751743	MACS_peak_13755	3.00	lobChIP_FOXA1_robot
chr6	122206717	122206718	MACS_peak_13756	8.00	lobChIP_FOXA1_robot
chr6	122288917	122288918	MACS_peak_13757	4.00	lobChIP_FOXA1_robot
chr6	122797406	122797407	MACS_peak_13758	4.00	lobChIP_FOXA1_robot
chr6	123112164	123112165	MACS_peak_13759	4.00	lobChIP_FOXA1_robot
chr6	123113188	123113189	MACS_peak_13760	4.00	lobChIP_FOXA1_robot
chr6	123119733	123119734	MACS_peak_13761	4.00	lobChIP_FOXA1_robot
chr6	123136662	123136663	MACS_peak_13762	5.00	lobChIP_FOXA1_robot
chr6	123218999	123219000	MACS_peak_13763	4.00	lobChIP_FOXA1_robot
chr6	123378166	123378167	MACS_peak_13764	10.00	lobChIP_FOXA1_robot
chr6	124721858	124721859	MACS_peak_13765	5.00	lobChIP_FOXA1_robot
chr6	125829639	125829640	MACS_peak_13766	14.00	lobChIP_FOXA1_robot
chr6	126220887	126220888	MACS_peak_13767	4.00	lobChIP_FOXA1_robot
chr6	126243676	126243677	MACS_peak_13768	4.00	lobChIP_FOXA1_robot
chr6	126265778	126265779	MACS_peak_13769	10.00	lobChIP_FOXA1_robot
chr6	126266684	126266685	MACS_peak_13770	4.00	lobChIP_FOXA1_robot
chr6	126398768	126398769	MACS_peak_13771	6.00	lobChIP_FOXA1_robot
chr6	126699078	126699079	MACS_peak_13772	5.00	lobChIP_FOXA1_robot
chr6	126701567	126701568	MACS_peak_13773	21.00	lobChIP_FOXA1_robot
chr6	126978908	126978909	MACS_peak_13774	5.00	lobChIP_FOXA1_robot
chr6	127343672	127343673	MACS_peak_13775	5.00	lobChIP_FOXA1_robot
chr6	127344323	127344324	MACS_peak_13776	69.00	lobChIP_FOXA1_robot
chr6	127498093	127498094	MACS_peak_13777	3.00	lobChIP_FOXA1_robot
chr6	127618874	127618875	MACS_peak_13778	7.00	lobChIP_FOXA1_robot
chr6	127668524	127668525	MACS_peak_13779	6.00	lobChIP_FOXA1_robot
chr6	127741864	127741865	MACS_peak_13780	7.00	lobChIP_FOXA1_robot
chr6	127779667	127779668	MACS_peak_13781	5.00	lobChIP_FOXA1_robot
chr6	128058412	128058413	MACS_peak_13782	5.00	lobChIP_FOXA1_robot
chr6	128328899	128328900	MACS_peak_13783	19.00	lobChIP_FOXA1_robot
chr6	128381463	128381464	MACS_peak_13784	4.00	lobChIP_FOXA1_robot
chr6	128384249	128384250	MACS_peak_13785	49.00	lobChIP_FOXA1_robot
chr6	128385526	128385527	MACS_peak_13786	10.00	lobChIP_FOXA1_robot
chr6	128564098	128564099	MACS_peak_13787	5.00	lobChIP_FOXA1_robot
chr6	128671787	128671788	MACS_peak_13788	4.00	lobChIP_FOXA1_robot
chr6	128686901	128686902	MACS_peak_13789	3.00	lobChIP_FOXA1_robot
chr6	128815623	128815624	MACS_peak_13790	5.00	lobChIP_FOXA1_robot
chr6	128820250	128820251	MACS_peak_13791	6.00	lobChIP_FOXA1_robot
chr6	128820686	128820687	MACS_peak_13792	7.00	lobChIP_FOXA1_robot
chr6	128825694	128825695	MACS_peak_13793	3.00	lobChIP_FOXA1_robot
chr6	128826961	128826962	MACS_peak_13794	5.00	lobChIP_FOXA1_robot
chr6	128829045	128829046	MACS_peak_13795	16.00	lobChIP_FOXA1_robot
chr6	128830160	128830161	MACS_peak_13796	7.00	lobChIP_FOXA1_robot
chr6	128883937	128883938	MACS_peak_13797	3.00	lobChIP_FOXA1_robot
chr6	128981552	128981553	MACS_peak_13798	7.00	lobChIP_FOXA1_robot
chr6	129589106	129589107	MACS_peak_13799	3.00	lobChIP_FOXA1_robot
chr6	129951917	129951918	MACS_peak_13800	7.00	lobChIP_FOXA1_robot
chr6	129958651	129958652	MACS_peak_13801	5.00	lobChIP_FOXA1_robot
chr6	129959651	129959652	MACS_peak_13802	5.00	lobChIP_FOXA1_robot
chr6	129965878	129965879	MACS_peak_13803	7.00	lobChIP_FOXA1_robot
chr6	129967185	129967186	MACS_peak_13804	6.00	lobChIP_FOXA1_robot
chr6	129983415	129983416	MACS_peak_13805	5.00	lobChIP_FOXA1_robot
chr6	130002883	130002884	MACS_peak_13806	3.00	lobChIP_FOXA1_robot
chr6	130005474	130005475	MACS_peak_13807	4.00	lobChIP_FOXA1_robot
chr6	130011024	130011025	MACS_peak_13808	5.00	lobChIP_FOXA1_robot
chr6	130015357	130015358	MACS_peak_13809	8.00	lobChIP_FOXA1_robot
chr6	130017084	130017085	MACS_peak_13810	22.00	lobChIP_FOXA1_robot
chr6	130026536	130026537	MACS_peak_13811	6.00	lobChIP_FOXA1_robot
chr6	130225026	130225027	MACS_peak_13812	4.00	lobChIP_FOXA1_robot
chr6	130241605	130241606	MACS_peak_13813	4.00	lobChIP_FOXA1_robot
chr6	130629292	130629293	MACS_peak_13814	10.00	lobChIP_FOXA1_robot
chr6	131133234	131133235	MACS_peak_13815	4.00	lobChIP_FOXA1_robot
chr6	131136581	131136582	MACS_peak_13816	51.00	lobChIP_FOXA1_robot
chr6	131146321	131146322	MACS_peak_13817	7.00	lobChIP_FOXA1_robot
chr6	131148402	131148403	MACS_peak_13818	4.00	lobChIP_FOXA1_robot
chr6	131184772	131184773	MACS_peak_13819	41.00	lobChIP_FOXA1_robot
chr6	131185742	131185743	MACS_peak_13820	5.00	lobChIP_FOXA1_robot
chr6	131255164	131255165	MACS_peak_13821	24.00	lobChIP_FOXA1_robot
chr6	131257357	131257358	MACS_peak_13822	7.00	lobChIP_FOXA1_robot
chr6	131266920	131266921	MACS_peak_13823	7.00	lobChIP_FOXA1_robot
chr6	131291128	131291129	MACS_peak_13824	6.00	lobChIP_FOXA1_robot
chr6	131360307	131360308	MACS_peak_13825	9.00	lobChIP_FOXA1_robot
chr6	131386498	131386499	MACS_peak_13826	3.00	lobChIP_FOXA1_robot
chr6	131687496	131687497	MACS_peak_13827	4.00	lobChIP_FOXA1_robot
chr6	131840882	131840883	MACS_peak_13828	3.00	lobChIP_FOXA1_robot
chr6	131895494	131895495	MACS_peak_13829	8.00	lobChIP_FOXA1_robot
chr6	132043226	132043227	MACS_peak_13830	5.00	lobChIP_FOXA1_robot
chr6	132076117	132076118	MACS_peak_13831	16.00	lobChIP_FOXA1_robot
chr6	132090975	132090976	MACS_peak_13832	9.00	lobChIP_FOXA1_robot
chr6	132108382	132108383	MACS_peak_13833	4.00	lobChIP_FOXA1_robot
chr6	132143746	132143747	MACS_peak_13834	4.00	lobChIP_FOXA1_robot
chr6	132157922	132157923	MACS_peak_13835	11.00	lobChIP_FOXA1_robot
chr6	132165533	132165534	MACS_peak_13836	21.00	lobChIP_FOXA1_robot
chr6	132189033	132189034	MACS_peak_13837	7.00	lobChIP_FOXA1_robot
chr6	132216420	132216421	MACS_peak_13838	25.00	lobChIP_FOXA1_robot
chr6	132259396	132259397	MACS_peak_13839	40.00	lobChIP_FOXA1_robot
chr6	132268328	132268329	MACS_peak_13840	4.00	lobChIP_FOXA1_robot
chr6	132272657	132272658	MACS_peak_13841	11.00	lobChIP_FOXA1_robot
chr6	132276321	132276322	MACS_peak_13842	4.00	lobChIP_FOXA1_robot
chr6	132280184	132280185	MACS_peak_13843	23.00	lobChIP_FOXA1_robot
chr6	132291756	132291757	MACS_peak_13844	4.00	lobChIP_FOXA1_robot
chr6	132297812	132297813	MACS_peak_13845	5.00	lobChIP_FOXA1_robot
chr6	132302282	132302283	MACS_peak_13846	22.00	lobChIP_FOXA1_robot
chr6	132342841	132342842	MACS_peak_13847	4.00	lobChIP_FOXA1_robot
chr6	132343280	132343281	MACS_peak_13848	5.00	lobChIP_FOXA1_robot
chr6	132375909	132375910	MACS_peak_13849	4.00	lobChIP_FOXA1_robot
chr6	132482660	132482661	MACS_peak_13850	3.00	lobChIP_FOXA1_robot
chr6	132786097	132786098	MACS_peak_13851	4.00	lobChIP_FOXA1_robot
chr6	132832741	132832742	MACS_peak_13852	5.00	lobChIP_FOXA1_robot
chr6	132834648	132834649	MACS_peak_13853	4.00	lobChIP_FOXA1_robot
chr6	133051968	133051969	MACS_peak_13854	4.00	lobChIP_FOXA1_robot
chr6	133056005	133056006	MACS_peak_13855	10.00	lobChIP_FOXA1_robot
chr6	133285470	133285471	MACS_peak_13856	10.00	lobChIP_FOXA1_robot
chr6	133355939	133355940	MACS_peak_13857	21.00	lobChIP_FOXA1_robot
chr6	133420917	133420918	MACS_peak_13858	6.00	lobChIP_FOXA1_robot
chr6	133790284	133790285	MACS_peak_13859	4.00	lobChIP_FOXA1_robot
chr6	134430925	134430926	MACS_peak_13860	8.00	lobChIP_FOXA1_robot
chr6	134444989	134444990	MACS_peak_13861	5.00	lobChIP_FOXA1_robot
chr6	134516435	134516436	MACS_peak_13862	5.00	lobChIP_FOXA1_robot
chr6	134519365	134519366	MACS_peak_13863	31.00	lobChIP_FOXA1_robot
chr6	134521250	134521251	MACS_peak_13864	41.00	lobChIP_FOXA1_robot
chr6	134571408	134571409	MACS_peak_13865	6.00	lobChIP_FOXA1_robot
chr6	134671326	134671327	MACS_peak_13866	3.00	lobChIP_FOXA1_robot
chr6	134851205	134851206	MACS_peak_13867	15.00	lobChIP_FOXA1_robot
chr6	134993054	134993055	MACS_peak_13868	3.00	lobChIP_FOXA1_robot
chr6	135009375	135009376	MACS_peak_13869	4.00	lobChIP_FOXA1_robot
chr6	135572772	135572773	MACS_peak_13870	24.00	lobChIP_FOXA1_robot
chr6	135748167	135748168	MACS_peak_13871	11.00	lobChIP_FOXA1_robot
chr6	135844532	135844533	MACS_peak_13872	4.00	lobChIP_FOXA1_robot
chr6	136002667	136002668	MACS_peak_13873	4.00	lobChIP_FOXA1_robot
chr6	136928648	136928649	MACS_peak_13874	5.00	lobChIP_FOXA1_robot
chr6	137209195	137209196	MACS_peak_13875	45.00	lobChIP_FOXA1_robot
chr6	137316095	137316096	MACS_peak_13876	4.00	lobChIP_FOXA1_robot
chr6	137322670	137322671	MACS_peak_13877	11.00	lobChIP_FOXA1_robot
chr6	137510782	137510783	MACS_peak_13878	5.00	lobChIP_FOXA1_robot
chr6	137539550	137539551	MACS_peak_13879	5.00	lobChIP_FOXA1_robot
chr6	137562861	137562862	MACS_peak_13880	36.00	lobChIP_FOXA1_robot
chr6	137568295	137568296	MACS_peak_13881	3.00	lobChIP_FOXA1_robot
chr6	137577943	137577944	MACS_peak_13882	4.00	lobChIP_FOXA1_robot
chr6	137582764	137582765	MACS_peak_13883	5.00	lobChIP_FOXA1_robot
chr6	137636367	137636368	MACS_peak_13884	8.00	lobChIP_FOXA1_robot
chr6	137671535	137671536	MACS_peak_13885	14.00	lobChIP_FOXA1_robot
chr6	137675255	137675256	MACS_peak_13886	29.00	lobChIP_FOXA1_robot
chr6	137750266	137750267	MACS_peak_13887	5.00	lobChIP_FOXA1_robot
chr6	137970132	137970133	MACS_peak_13888	5.00	lobChIP_FOXA1_robot
chr6	138018979	138018980	MACS_peak_13889	13.00	lobChIP_FOXA1_robot
chr6	138028649	138028650	MACS_peak_13890	5.00	lobChIP_FOXA1_robot
chr6	138044648	138044649	MACS_peak_13891	13.00	lobChIP_FOXA1_robot
chr6	138051178	138051179	MACS_peak_13892	5.00	lobChIP_FOXA1_robot
chr6	138053331	138053332	MACS_peak_13893	12.00	lobChIP_FOXA1_robot
chr6	138103679	138103680	MACS_peak_13894	7.00	lobChIP_FOXA1_robot
chr6	138116236	138116237	MACS_peak_13895	40.00	lobChIP_FOXA1_robot
chr6	138121290	138121291	MACS_peak_13896	5.00	lobChIP_FOXA1_robot
chr6	138129409	138129410	MACS_peak_13897	5.00	lobChIP_FOXA1_robot
chr6	138170001	138170002	MACS_peak_13898	4.00	lobChIP_FOXA1_robot
chr6	138194655	138194656	MACS_peak_13899	11.00	lobChIP_FOXA1_robot
chr6	138406178	138406179	MACS_peak_13900	5.00	lobChIP_FOXA1_robot
chr6	138407851	138407852	MACS_peak_13901	22.00	lobChIP_FOXA1_robot
chr6	138544122	138544123	MACS_peak_13902	14.00	lobChIP_FOXA1_robot
chr6	138552895	138552896	MACS_peak_13903	8.00	lobChIP_FOXA1_robot
chr6	138570221	138570222	MACS_peak_13904	6.00	lobChIP_FOXA1_robot
chr6	138579012	138579013	MACS_peak_13905	12.00	lobChIP_FOXA1_robot
chr6	138708922	138708923	MACS_peak_13906	5.00	lobChIP_FOXA1_robot
chr6	138789993	138789994	MACS_peak_13907	13.00	lobChIP_FOXA1_robot
chr6	138825428	138825429	MACS_peak_13908	3.00	lobChIP_FOXA1_robot
chr6	138826279	138826280	MACS_peak_13909	9.00	lobChIP_FOXA1_robot
chr6	138848278	138848279	MACS_peak_13910	7.00	lobChIP_FOXA1_robot
chr6	138862644	138862645	MACS_peak_13911	7.00	lobChIP_FOXA1_robot
chr6	138864974	138864975	MACS_peak_13912	14.00	lobChIP_FOXA1_robot
chr6	138884001	138884002	MACS_peak_13913	5.00	lobChIP_FOXA1_robot
chr6	138891280	138891281	MACS_peak_13914	4.00	lobChIP_FOXA1_robot
chr6	138891775	138891776	MACS_peak_13915	5.00	lobChIP_FOXA1_robot
chr6	139135487	139135488	MACS_peak_13916	8.00	lobChIP_FOXA1_robot
chr6	139238897	139238898	MACS_peak_13917	4.00	lobChIP_FOXA1_robot
chr6	139276576	139276577	MACS_peak_13918	4.00	lobChIP_FOXA1_robot
chr6	139310479	139310480	MACS_peak_13919	13.00	lobChIP_FOXA1_robot
chr6	139630529	139630530	MACS_peak_13920	6.00	lobChIP_FOXA1_robot
chr6	139696892	139696893	MACS_peak_13921	6.00	lobChIP_FOXA1_robot
chr6	139829947	139829948	MACS_peak_13922	7.00	lobChIP_FOXA1_robot
chr6	140087080	140087081	MACS_peak_13923	6.00	lobChIP_FOXA1_robot
chr6	140247274	140247275	MACS_peak_13924	3.00	lobChIP_FOXA1_robot
chr6	140701247	140701248	MACS_peak_13925	6.00	lobChIP_FOXA1_robot
chr6	140702208	140702209	MACS_peak_13926	11.00	lobChIP_FOXA1_robot
chr6	140777676	140777677	MACS_peak_13927	4.00	lobChIP_FOXA1_robot
chr6	141034590	141034591	MACS_peak_13928	5.00	lobChIP_FOXA1_robot
chr6	141587096	141587097	MACS_peak_13929	5.00	lobChIP_FOXA1_robot
chr6	141848272	141848273	MACS_peak_13930	6.00	lobChIP_FOXA1_robot
chr6	141882498	141882499	MACS_peak_13931	4.00	lobChIP_FOXA1_robot
chr6	142325037	142325038	MACS_peak_13932	6.00	lobChIP_FOXA1_robot
chr6	142325887	142325888	MACS_peak_13933	6.00	lobChIP_FOXA1_robot
chr6	142553791	142553792	MACS_peak_13934	7.00	lobChIP_FOXA1_robot
chr6	142586806	142586807	MACS_peak_13935	12.00	lobChIP_FOXA1_robot
chr6	142601804	142601805	MACS_peak_13936	4.00	lobChIP_FOXA1_robot
chr6	142606889	142606890	MACS_peak_13937	25.00	lobChIP_FOXA1_robot
chr6	142613213	142613214	MACS_peak_13938	4.00	lobChIP_FOXA1_robot
chr6	142618196	142618197	MACS_peak_13939	7.00	lobChIP_FOXA1_robot
chr6	142619233	142619234	MACS_peak_13940	14.00	lobChIP_FOXA1_robot
chr6	142619880	142619881	MACS_peak_13941	4.00	lobChIP_FOXA1_robot
chr6	142623763	142623764	MACS_peak_13942	5.00	lobChIP_FOXA1_robot
chr6	142626937	142626938	MACS_peak_13943	3.00	lobChIP_FOXA1_robot
chr6	142627977	142627978	MACS_peak_13944	3.00	lobChIP_FOXA1_robot
chr6	142629182	142629183	MACS_peak_13945	69.00	lobChIP_FOXA1_robot
chr6	142644751	142644752	MACS_peak_13946	25.00	lobChIP_FOXA1_robot
chr6	142652344	142652345	MACS_peak_13947	9.00	lobChIP_FOXA1_robot
chr6	142670858	142670859	MACS_peak_13948	5.00	lobChIP_FOXA1_robot
chr6	142683014	142683015	MACS_peak_13949	8.00	lobChIP_FOXA1_robot
chr6	142696665	142696666	MACS_peak_13950	52.00	lobChIP_FOXA1_robot
chr6	143041515	143041516	MACS_peak_13951	4.00	lobChIP_FOXA1_robot
chr6	143111533	143111534	MACS_peak_13952	5.00	lobChIP_FOXA1_robot
chr6	143167187	143167188	MACS_peak_13953	3.00	lobChIP_FOXA1_robot
chr6	143189287	143189288	MACS_peak_13954	5.00	lobChIP_FOXA1_robot
chr6	143190361	143190362	MACS_peak_13955	8.00	lobChIP_FOXA1_robot
chr6	143206590	143206591	MACS_peak_13956	5.00	lobChIP_FOXA1_robot
chr6	143223875	143223876	MACS_peak_13957	4.00	lobChIP_FOXA1_robot
chr6	143249124	143249125	MACS_peak_13958	5.00	lobChIP_FOXA1_robot
chr6	143264116	143264117	MACS_peak_13959	17.00	lobChIP_FOXA1_robot
chr6	143380940	143380941	MACS_peak_13960	3.00	lobChIP_FOXA1_robot
chr6	143382805	143382806	MACS_peak_13961	3.00	lobChIP_FOXA1_robot
chr6	143717826	143717827	MACS_peak_13962	12.00	lobChIP_FOXA1_robot
chr6	143718488	143718489	MACS_peak_13963	6.00	lobChIP_FOXA1_robot
chr6	143730636	143730637	MACS_peak_13964	7.00	lobChIP_FOXA1_robot
chr6	143732193	143732194	MACS_peak_13965	19.00	lobChIP_FOXA1_robot
chr6	143753945	143753946	MACS_peak_13966	9.00	lobChIP_FOXA1_robot
chr6	143856543	143856544	MACS_peak_13967	16.00	lobChIP_FOXA1_robot
chr6	143876148	143876149	MACS_peak_13968	5.00	lobChIP_FOXA1_robot
chr6	143951622	143951623	MACS_peak_13969	4.00	lobChIP_FOXA1_robot
chr6	143960822	143960823	MACS_peak_13970	6.00	lobChIP_FOXA1_robot
chr6	143976877	143976878	MACS_peak_13971	19.00	lobChIP_FOXA1_robot
chr6	143990823	143990824	MACS_peak_13972	4.00	lobChIP_FOXA1_robot
chr6	143996703	143996704	MACS_peak_13973	4.00	lobChIP_FOXA1_robot
chr6	144012569	144012570	MACS_peak_13974	6.00	lobChIP_FOXA1_robot
chr6	144051499	144051500	MACS_peak_13975	3.00	lobChIP_FOXA1_robot
chr6	144066078	144066079	MACS_peak_13976	6.00	lobChIP_FOXA1_robot
chr6	144066741	144066742	MACS_peak_13977	56.00	lobChIP_FOXA1_robot
chr6	144068562	144068563	MACS_peak_13978	15.00	lobChIP_FOXA1_robot
chr6	144538546	144538547	MACS_peak_13979	4.00	lobChIP_FOXA1_robot
chr6	144581861	144581862	MACS_peak_13980	3.00	lobChIP_FOXA1_robot
chr6	144585468	144585469	MACS_peak_13981	10.00	lobChIP_FOXA1_robot
chr6	144608485	144608486	MACS_peak_13982	7.00	lobChIP_FOXA1_robot
chr6	144619182	144619183	MACS_peak_13983	5.00	lobChIP_FOXA1_robot
chr6	144637559	144637560	MACS_peak_13984	12.00	lobChIP_FOXA1_robot
chr6	144660663	144660664	MACS_peak_13985	4.00	lobChIP_FOXA1_robot
chr6	144662033	144662034	MACS_peak_13986	6.00	lobChIP_FOXA1_robot
chr6	144664962	144664963	MACS_peak_13987	30.00	lobChIP_FOXA1_robot
chr6	144696732	144696733	MACS_peak_13988	14.00	lobChIP_FOXA1_robot
chr6	144733183	144733184	MACS_peak_13989	4.00	lobChIP_FOXA1_robot
chr6	144736374	144736375	MACS_peak_13990	12.00	lobChIP_FOXA1_robot
chr6	144765609	144765610	MACS_peak_13991	7.00	lobChIP_FOXA1_robot
chr6	144881453	144881454	MACS_peak_13992	5.00	lobChIP_FOXA1_robot
chr6	144900287	144900288	MACS_peak_13993	3.00	lobChIP_FOXA1_robot
chr6	144968154	144968155	MACS_peak_13994	3.00	lobChIP_FOXA1_robot
chr6	144969618	144969619	MACS_peak_13995	18.00	lobChIP_FOXA1_robot
chr6	144970402	144970403	MACS_peak_13996	5.00	lobChIP_FOXA1_robot
chr6	144973843	144973844	MACS_peak_13997	8.00	lobChIP_FOXA1_robot
chr6	144975175	144975176	MACS_peak_13998	38.00	lobChIP_FOXA1_robot
chr6	144977270	144977271	MACS_peak_13999	4.00	lobChIP_FOXA1_robot
chr6	145111494	145111495	MACS_peak_14000	3.00	lobChIP_FOXA1_robot
chr6	145346786	145346787	MACS_peak_14001	12.00	lobChIP_FOXA1_robot
chr6	145347638	145347639	MACS_peak_14002	9.00	lobChIP_FOXA1_robot
chr6	145588461	145588462	MACS_peak_14003	73.00	lobChIP_FOXA1_robot
chr6	145872285	145872286	MACS_peak_14004	3.00	lobChIP_FOXA1_robot
chr6	145914401	145914402	MACS_peak_14005	3.00	lobChIP_FOXA1_robot
chr6	146085084	146085085	MACS_peak_14006	7.00	lobChIP_FOXA1_robot
chr6	146161670	146161671	MACS_peak_14007	5.00	lobChIP_FOXA1_robot
chr6	147266154	147266155	MACS_peak_14008	4.00	lobChIP_FOXA1_robot
chr6	147302040	147302041	MACS_peak_14009	11.00	lobChIP_FOXA1_robot
chr6	147305383	147305384	MACS_peak_14010	7.00	lobChIP_FOXA1_robot
chr6	147306473	147306474	MACS_peak_14011	7.00	lobChIP_FOXA1_robot
chr6	147381946	147381947	MACS_peak_14012	5.00	lobChIP_FOXA1_robot
chr6	147513597	147513598	MACS_peak_14013	27.00	lobChIP_FOXA1_robot
chr6	148004474	148004475	MACS_peak_14014	4.00	lobChIP_FOXA1_robot
chr6	148126472	148126473	MACS_peak_14015	6.00	lobChIP_FOXA1_robot
chr6	148663525	148663526	MACS_peak_14016	5.00	lobChIP_FOXA1_robot
chr6	149241627	149241628	MACS_peak_14017	4.00	lobChIP_FOXA1_robot
chr6	149300769	149300770	MACS_peak_14018	6.00	lobChIP_FOXA1_robot
chr6	149350223	149350224	MACS_peak_14019	4.00	lobChIP_FOXA1_robot
chr6	149354397	149354398	MACS_peak_14020	7.00	lobChIP_FOXA1_robot
chr6	149376096	149376097	MACS_peak_14021	4.00	lobChIP_FOXA1_robot
chr6	149382633	149382634	MACS_peak_14022	5.00	lobChIP_FOXA1_robot
chr6	149482252	149482253	MACS_peak_14023	6.00	lobChIP_FOXA1_robot
chr6	149498368	149498369	MACS_peak_14024	17.00	lobChIP_FOXA1_robot
chr6	149507127	149507128	MACS_peak_14025	7.00	lobChIP_FOXA1_robot
chr6	149593521	149593522	MACS_peak_14026	3.00	lobChIP_FOXA1_robot
chr6	149693409	149693410	MACS_peak_14027	5.00	lobChIP_FOXA1_robot
chr6	149762703	149762704	MACS_peak_14028	6.00	lobChIP_FOXA1_robot
chr6	149806126	149806127	MACS_peak_14029	4.00	lobChIP_FOXA1_robot
chr6	149818687	149818688	MACS_peak_14030	5.00	lobChIP_FOXA1_robot
chr6	150152870	150152871	MACS_peak_14031	4.00	lobChIP_FOXA1_robot
chr6	150168916	150168917	MACS_peak_14032	4.00	lobChIP_FOXA1_robot
chr6	150176529	150176530	MACS_peak_14033	5.00	lobChIP_FOXA1_robot
chr6	150182022	150182023	MACS_peak_14034	7.00	lobChIP_FOXA1_robot
chr6	150195492	150195493	MACS_peak_14035	4.00	lobChIP_FOXA1_robot
chr6	150856650	150856651	MACS_peak_14036	4.00	lobChIP_FOXA1_robot
chr6	150934248	150934249	MACS_peak_14037	4.00	lobChIP_FOXA1_robot
chr6	151199902	151199903	MACS_peak_14038	12.00	lobChIP_FOXA1_robot
chr6	151203515	151203516	MACS_peak_14039	6.00	lobChIP_FOXA1_robot
chr6	151314171	151314172	MACS_peak_14040	5.00	lobChIP_FOXA1_robot
chr6	151326079	151326080	MACS_peak_14041	4.00	lobChIP_FOXA1_robot
chr6	151348630	151348631	MACS_peak_14042	3.00	lobChIP_FOXA1_robot
chr6	151357604	151357605	MACS_peak_14043	6.00	lobChIP_FOXA1_robot
chr6	151381452	151381453	MACS_peak_14044	6.00	lobChIP_FOXA1_robot
chr6	151389099	151389100	MACS_peak_14045	21.00	lobChIP_FOXA1_robot
chr6	151391062	151391063	MACS_peak_14046	6.00	lobChIP_FOXA1_robot
chr6	151406477	151406478	MACS_peak_14047	5.00	lobChIP_FOXA1_robot
chr6	151417438	151417439	MACS_peak_14048	4.00	lobChIP_FOXA1_robot
chr6	151441386	151441387	MACS_peak_14049	4.00	lobChIP_FOXA1_robot
chr6	151447481	151447482	MACS_peak_14050	7.00	lobChIP_FOXA1_robot
chr6	151482187	151482188	MACS_peak_14051	6.00	lobChIP_FOXA1_robot
chr6	151512191	151512192	MACS_peak_14052	3.00	lobChIP_FOXA1_robot
chr6	151547275	151547276	MACS_peak_14053	3.00	lobChIP_FOXA1_robot
chr6	151590916	151590917	MACS_peak_14054	7.00	lobChIP_FOXA1_robot
chr6	151628784	151628785	MACS_peak_14055	17.00	lobChIP_FOXA1_robot
chr6	151643957	151643958	MACS_peak_14056	4.00	lobChIP_FOXA1_robot
chr6	151713019	151713020	MACS_peak_14057	5.00	lobChIP_FOXA1_robot
chr6	151822821	151822822	MACS_peak_14058	7.00	lobChIP_FOXA1_robot
chr6	151954847	151954848	MACS_peak_14059	4.00	lobChIP_FOXA1_robot
chr6	151961069	151961070	MACS_peak_14060	3.00	lobChIP_FOXA1_robot
chr6	151979413	151979414	MACS_peak_14061	7.00	lobChIP_FOXA1_robot
chr6	152004207	152004208	MACS_peak_14062	6.00	lobChIP_FOXA1_robot
chr6	152457461	152457462	MACS_peak_14063	25.00	lobChIP_FOXA1_robot
chr6	152529421	152529422	MACS_peak_14064	13.00	lobChIP_FOXA1_robot
chr6	152589484	152589485	MACS_peak_14065	5.00	lobChIP_FOXA1_robot
chr6	152618229	152618230	MACS_peak_14066	4.00	lobChIP_FOXA1_robot
chr6	152622730	152622731	MACS_peak_14067	8.00	lobChIP_FOXA1_robot
chr6	152702862	152702863	MACS_peak_14068	6.00	lobChIP_FOXA1_robot
chr6	154421286	154421287	MACS_peak_14069	7.00	lobChIP_FOXA1_robot
chr6	154614181	154614182	MACS_peak_14070	4.00	lobChIP_FOXA1_robot
chr6	154735652	154735653	MACS_peak_14071	7.00	lobChIP_FOXA1_robot
chr6	154751850	154751851	MACS_peak_14072	3.00	lobChIP_FOXA1_robot
chr6	154761310	154761311	MACS_peak_14073	13.00	lobChIP_FOXA1_robot
chr6	154781701	154781702	MACS_peak_14074	9.00	lobChIP_FOXA1_robot
chr6	154808994	154808995	MACS_peak_14075	7.00	lobChIP_FOXA1_robot
chr6	154822063	154822064	MACS_peak_14076	5.00	lobChIP_FOXA1_robot
chr6	154830276	154830277	MACS_peak_14077	9.00	lobChIP_FOXA1_robot
chr6	154832654	154832655	MACS_peak_14078	5.00	lobChIP_FOXA1_robot
chr6	155119334	155119335	MACS_peak_14079	3.00	lobChIP_FOXA1_robot
chr6	155173974	155173975	MACS_peak_14080	6.00	lobChIP_FOXA1_robot
chr6	155281741	155281742	MACS_peak_14081	5.00	lobChIP_FOXA1_robot
chr6	155282996	155282997	MACS_peak_14082	3.00	lobChIP_FOXA1_robot
chr6	155302410	155302411	MACS_peak_14083	7.00	lobChIP_FOXA1_robot
chr6	155649997	155649998	MACS_peak_14084	41.00	lobChIP_FOXA1_robot
chr6	155720653	155720654	MACS_peak_14085	11.00	lobChIP_FOXA1_robot
chr6	155723458	155723459	MACS_peak_14086	3.00	lobChIP_FOXA1_robot
chr6	155765422	155765423	MACS_peak_14087	6.00	lobChIP_FOXA1_robot
chr6	155808156	155808157	MACS_peak_14088	52.00	lobChIP_FOXA1_robot
chr6	155812731	155812732	MACS_peak_14089	20.00	lobChIP_FOXA1_robot
chr6	155815001	155815002	MACS_peak_14090	4.00	lobChIP_FOXA1_robot
chr6	155816419	155816420	MACS_peak_14091	4.00	lobChIP_FOXA1_robot
chr6	155850145	155850146	MACS_peak_14092	5.00	lobChIP_FOXA1_robot
chr6	155882646	155882647	MACS_peak_14093	5.00	lobChIP_FOXA1_robot
chr6	156594662	156594663	MACS_peak_14094	6.00	lobChIP_FOXA1_robot
chr6	157081587	157081588	MACS_peak_14095	4.00	lobChIP_FOXA1_robot
chr6	157098145	157098146	MACS_peak_14096	4.00	lobChIP_FOXA1_robot
chr6	157101754	157101755	MACS_peak_14097	5.00	lobChIP_FOXA1_robot
chr6	157144149	157144150	MACS_peak_14098	5.00	lobChIP_FOXA1_robot
chr6	157254445	157254446	MACS_peak_14099	5.00	lobChIP_FOXA1_robot
chr6	157274690	157274691	MACS_peak_14100	6.00	lobChIP_FOXA1_robot
chr6	157284836	157284837	MACS_peak_14101	5.00	lobChIP_FOXA1_robot
chr6	157418094	157418095	MACS_peak_14102	3.00	lobChIP_FOXA1_robot
chr6	157508745	157508746	MACS_peak_14103	6.00	lobChIP_FOXA1_robot
chr6	157845371	157845372	MACS_peak_14104	3.00	lobChIP_FOXA1_robot
chr6	157934540	157934541	MACS_peak_14105	8.00	lobChIP_FOXA1_robot
chr6	157952267	157952268	MACS_peak_14106	8.00	lobChIP_FOXA1_robot
chr6	157961124	157961125	MACS_peak_14107	7.00	lobChIP_FOXA1_robot
chr6	158104064	158104065	MACS_peak_14108	7.00	lobChIP_FOXA1_robot
chr6	158149638	158149639	MACS_peak_14109	4.00	lobChIP_FOXA1_robot
chr6	158246668	158246669	MACS_peak_14110	14.00	lobChIP_FOXA1_robot
chr6	158375692	158375693	MACS_peak_14111	4.00	lobChIP_FOXA1_robot
chr6	158396157	158396158	MACS_peak_14112	4.00	lobChIP_FOXA1_robot
chr6	158403425	158403426	MACS_peak_14113	10.00	lobChIP_FOXA1_robot
chr6	158406607	158406608	MACS_peak_14114	35.00	lobChIP_FOXA1_robot
chr6	158429303	158429304	MACS_peak_14115	3.00	lobChIP_FOXA1_robot
chr6	158432782	158432783	MACS_peak_14116	34.00	lobChIP_FOXA1_robot
chr6	158436152	158436153	MACS_peak_14117	4.00	lobChIP_FOXA1_robot
chr6	158455447	158455448	MACS_peak_14118	11.00	lobChIP_FOXA1_robot
chr6	158461537	158461538	MACS_peak_14119	19.00	lobChIP_FOXA1_robot
chr6	158526123	158526124	MACS_peak_14120	3.00	lobChIP_FOXA1_robot
chr6	158564012	158564013	MACS_peak_14121	7.00	lobChIP_FOXA1_robot
chr6	158604571	158604572	MACS_peak_14122	4.00	lobChIP_FOXA1_robot
chr6	158717811	158717812	MACS_peak_14123	3.00	lobChIP_FOXA1_robot
chr6	158743892	158743893	MACS_peak_14124	13.00	lobChIP_FOXA1_robot
chr6	158747200	158747201	MACS_peak_14125	3.00	lobChIP_FOXA1_robot
chr6	158753497	158753498	MACS_peak_14126	5.00	lobChIP_FOXA1_robot
chr6	158763369	158763370	MACS_peak_14127	6.00	lobChIP_FOXA1_robot
chr6	158963534	158963535	MACS_peak_14128	7.00	lobChIP_FOXA1_robot
chr6	158976077	158976078	MACS_peak_14129	14.00	lobChIP_FOXA1_robot
chr6	158993085	158993086	MACS_peak_14130	7.00	lobChIP_FOXA1_robot
chr6	159246877	159246878	MACS_peak_14131	5.00	lobChIP_FOXA1_robot
chr6	159265519	159265520	MACS_peak_14132	4.00	lobChIP_FOXA1_robot
chr6	159274606	159274607	MACS_peak_14133	8.00	lobChIP_FOXA1_robot
chr6	159367525	159367526	MACS_peak_14134	6.00	lobChIP_FOXA1_robot
chr6	160112535	160112536	MACS_peak_14135	6.00	lobChIP_FOXA1_robot
chr6	160115536	160115537	MACS_peak_14136	6.00	lobChIP_FOXA1_robot
chr6	160147907	160147908	MACS_peak_14137	5.00	lobChIP_FOXA1_robot
chr6	160296298	160296299	MACS_peak_14138	5.00	lobChIP_FOXA1_robot
chr6	160464111	160464112	MACS_peak_14139	7.00	lobChIP_FOXA1_robot
chr6	160558430	160558431	MACS_peak_14140	19.00	lobChIP_FOXA1_robot
chr6	160559433	160559434	MACS_peak_14141	4.00	lobChIP_FOXA1_robot
chr6	160815458	160815459	MACS_peak_14142	5.00	lobChIP_FOXA1_robot
chr6	161231372	161231373	MACS_peak_14143	6.00	lobChIP_FOXA1_robot
chr6	162295693	162295694	MACS_peak_14144	7.00	lobChIP_FOXA1_robot
chr6	162671459	162671460	MACS_peak_14145	5.00	lobChIP_FOXA1_robot
chr6	162794407	162794408	MACS_peak_14146	8.00	lobChIP_FOXA1_robot
chr6	163304293	163304294	MACS_peak_14147	4.00	lobChIP_FOXA1_robot
chr6	163572562	163572563	MACS_peak_14148	3.00	lobChIP_FOXA1_robot
chr6	163867719	163867720	MACS_peak_14149	5.00	lobChIP_FOXA1_robot
chr6	164116323	164116324	MACS_peak_14150	12.00	lobChIP_FOXA1_robot
chr6	164211790	164211791	MACS_peak_14151	3.00	lobChIP_FOXA1_robot
chr6	165853628	165853629	MACS_peak_14152	5.00	lobChIP_FOXA1_robot
chr6	166088715	166088716	MACS_peak_14153	3.00	lobChIP_FOXA1_robot
chr6	166436520	166436521	MACS_peak_14154	4.00	lobChIP_FOXA1_robot
chr6	166444715	166444716	MACS_peak_14155	10.00	lobChIP_FOXA1_robot
chr6	166765309	166765310	MACS_peak_14156	9.00	lobChIP_FOXA1_robot
chr6	166774871	166774872	MACS_peak_14157	10.00	lobChIP_FOXA1_robot
chr6	166801536	166801537	MACS_peak_14158	10.00	lobChIP_FOXA1_robot
chr6	167347578	167347579	MACS_peak_14159	3.00	lobChIP_FOXA1_robot
chr6	167362368	167362369	MACS_peak_14160	6.00	lobChIP_FOXA1_robot
chr6	167491306	167491307	MACS_peak_14161	4.00	lobChIP_FOXA1_robot
chr6	167516708	167516709	MACS_peak_14162	6.00	lobChIP_FOXA1_robot
chr6	167536501	167536502	MACS_peak_14163	6.00	lobChIP_FOXA1_robot
chr6	167544733	167544734	MACS_peak_14164	9.00	lobChIP_FOXA1_robot
chr6	167554765	167554766	MACS_peak_14165	8.00	lobChIP_FOXA1_robot
chr6	167630038	167630039	MACS_peak_14166	6.00	lobChIP_FOXA1_robot
chr6	167652601	167652602	MACS_peak_14167	4.00	lobChIP_FOXA1_robot
chr6	167682349	167682350	MACS_peak_14168	7.00	lobChIP_FOXA1_robot
chr6	167686255	167686256	MACS_peak_14169	8.00	lobChIP_FOXA1_robot
chr6	167722351	167722352	MACS_peak_14170	5.00	lobChIP_FOXA1_robot
chr6	167723397	167723398	MACS_peak_14171	4.00	lobChIP_FOXA1_robot
chr6	167723878	167723879	MACS_peak_14172	12.00	lobChIP_FOXA1_robot
chr6	167755726	167755727	MACS_peak_14173	57.00	lobChIP_FOXA1_robot
chr6	167810201	167810202	MACS_peak_14174	11.00	lobChIP_FOXA1_robot
chr6	167811972	167811973	MACS_peak_14175	6.00	lobChIP_FOXA1_robot
chr6	167909827	167909828	MACS_peak_14176	5.00	lobChIP_FOXA1_robot
chr6	168068860	168068861	MACS_peak_14177	5.00	lobChIP_FOXA1_robot
chr6	168076931	168076932	MACS_peak_14178	23.00	lobChIP_FOXA1_robot
chr6	168083943	168083944	MACS_peak_14179	17.00	lobChIP_FOXA1_robot
chr6	168129220	168129221	MACS_peak_14180	5.00	lobChIP_FOXA1_robot
chr6	168134741	168134742	MACS_peak_14181	15.00	lobChIP_FOXA1_robot
chr6	168143778	168143779	MACS_peak_14182	4.00	lobChIP_FOXA1_robot
chr6	168163245	168163246	MACS_peak_14183	7.00	lobChIP_FOXA1_robot
chr6	168165905	168165906	MACS_peak_14184	4.00	lobChIP_FOXA1_robot
chr6	168207149	168207150	MACS_peak_14185	6.00	lobChIP_FOXA1_robot
chr6	168250727	168250728	MACS_peak_14186	3.00	lobChIP_FOXA1_robot
chr6	168256657	168256658	MACS_peak_14187	6.00	lobChIP_FOXA1_robot
chr6	168278073	168278074	MACS_peak_14188	4.00	lobChIP_FOXA1_robot
chr6	168310402	168310403	MACS_peak_14189	9.00	lobChIP_FOXA1_robot
chr6	168334210	168334211	MACS_peak_14190	6.00	lobChIP_FOXA1_robot
chr6	168364413	168364414	MACS_peak_14191	9.00	lobChIP_FOXA1_robot
chr6	168365525	168365526	MACS_peak_14192	28.00	lobChIP_FOXA1_robot
chr6	168425045	168425046	MACS_peak_14193	3.00	lobChIP_FOXA1_robot
chr6	168574144	168574145	MACS_peak_14194	5.00	lobChIP_FOXA1_robot
chr6	168597311	168597312	MACS_peak_14195	5.00	lobChIP_FOXA1_robot
chr6	168646078	168646079	MACS_peak_14196	17.00	lobChIP_FOXA1_robot
chr6	168648952	168648953	MACS_peak_14197	7.00	lobChIP_FOXA1_robot
chr6	168662702	168662703	MACS_peak_14198	10.00	lobChIP_FOXA1_robot
chr6	168665091	168665092	MACS_peak_14199	3.00	lobChIP_FOXA1_robot
chr6	168665784	168665785	MACS_peak_14200	7.00	lobChIP_FOXA1_robot
chr6	168675383	168675384	MACS_peak_14201	6.00	lobChIP_FOXA1_robot
chr6	168702460	168702461	MACS_peak_14202	20.00	lobChIP_FOXA1_robot
chr6	168718874	168718875	MACS_peak_14203	4.00	lobChIP_FOXA1_robot
chr6	168727840	168727841	MACS_peak_14204	8.00	lobChIP_FOXA1_robot
chr6	168900099	168900100	MACS_peak_14205	5.00	lobChIP_FOXA1_robot
chr6	169295236	169295237	MACS_peak_14206	4.00	lobChIP_FOXA1_robot
chr6	169692674	169692675	MACS_peak_14207	14.00	lobChIP_FOXA1_robot
chr6	170120479	170120480	MACS_peak_14208	4.00	lobChIP_FOXA1_robot
chr6	170123176	170123177	MACS_peak_14209	3.00	lobChIP_FOXA1_robot
chr6	170126076	170126077	MACS_peak_14210	6.00	lobChIP_FOXA1_robot
chr6	170199851	170199852	MACS_peak_14211	15.00	lobChIP_FOXA1_robot
chr6	170200812	170200813	MACS_peak_14212	9.00	lobChIP_FOXA1_robot
chr6	170589232	170589233	MACS_peak_14213	4.00	lobChIP_FOXA1_robot
chr7	359061	359062	MACS_peak_14214	12.00	lobChIP_FOXA1_robot
chr7	382547	382548	MACS_peak_14215	3.00	lobChIP_FOXA1_robot
chr7	413124	413125	MACS_peak_14216	5.00	lobChIP_FOXA1_robot
chr7	475055	475056	MACS_peak_14217	6.00	lobChIP_FOXA1_robot
chr7	524476	524477	MACS_peak_14218	3.00	lobChIP_FOXA1_robot
chr7	658266	658267	MACS_peak_14219	5.00	lobChIP_FOXA1_robot
chr7	852494	852495	MACS_peak_14220	4.00	lobChIP_FOXA1_robot
chr7	1196775	1196776	MACS_peak_14221	60.00	lobChIP_FOXA1_robot
chr7	1546732	1546733	MACS_peak_14222	4.00	lobChIP_FOXA1_robot
chr7	1560326	1560327	MACS_peak_14223	4.00	lobChIP_FOXA1_robot
chr7	1572535	1572536	MACS_peak_14224	5.00	lobChIP_FOXA1_robot
chr7	1576984	1576985	MACS_peak_14225	9.00	lobChIP_FOXA1_robot
chr7	1753638	1753639	MACS_peak_14226	9.00	lobChIP_FOXA1_robot
chr7	1843494	1843495	MACS_peak_14227	6.00	lobChIP_FOXA1_robot
chr7	2067423	2067424	MACS_peak_14228	14.00	lobChIP_FOXA1_robot
chr7	2149681	2149682	MACS_peak_14229	3.00	lobChIP_FOXA1_robot
chr7	2451974	2451975	MACS_peak_14230	7.00	lobChIP_FOXA1_robot
chr7	2452573	2452574	MACS_peak_14231	5.00	lobChIP_FOXA1_robot
chr7	2551256	2551257	MACS_peak_14232	10.00	lobChIP_FOXA1_robot
chr7	2667852	2667853	MACS_peak_14233	4.00	lobChIP_FOXA1_robot
chr7	2797900	2797901	MACS_peak_14234	6.00	lobChIP_FOXA1_robot
chr7	2842451	2842452	MACS_peak_14235	8.00	lobChIP_FOXA1_robot
chr7	2844178	2844179	MACS_peak_14236	3.00	lobChIP_FOXA1_robot
chr7	2897226	2897227	MACS_peak_14237	9.00	lobChIP_FOXA1_robot
chr7	3234463	3234464	MACS_peak_14238	4.00	lobChIP_FOXA1_robot
chr7	3954187	3954188	MACS_peak_14239	5.00	lobChIP_FOXA1_robot
chr7	3993057	3993058	MACS_peak_14240	5.00	lobChIP_FOXA1_robot
chr7	4046542	4046543	MACS_peak_14241	6.00	lobChIP_FOXA1_robot
chr7	4446487	4446488	MACS_peak_14242	3.00	lobChIP_FOXA1_robot
chr7	4779217	4779218	MACS_peak_14243	6.00	lobChIP_FOXA1_robot
chr7	4784676	4784677	MACS_peak_14244	5.00	lobChIP_FOXA1_robot
chr7	5324173	5324174	MACS_peak_14245	6.00	lobChIP_FOXA1_robot
chr7	5463640	5463641	MACS_peak_14246	5.00	lobChIP_FOXA1_robot
chr7	5466454	5466455	MACS_peak_14247	6.00	lobChIP_FOXA1_robot
chr7	5553923	5553924	MACS_peak_14248	43.00	lobChIP_FOXA1_robot
chr7	5554421	5554422	MACS_peak_14249	5.00	lobChIP_FOXA1_robot
chr7	5731856	5731857	MACS_peak_14250	5.00	lobChIP_FOXA1_robot
chr7	5849880	5849881	MACS_peak_14251	3.00	lobChIP_FOXA1_robot
chr7	6090604	6090605	MACS_peak_14252	9.00	lobChIP_FOXA1_robot
chr7	6095295	6095296	MACS_peak_14253	5.00	lobChIP_FOXA1_robot
chr7	6098393	6098394	MACS_peak_14254	7.00	lobChIP_FOXA1_robot
chr7	6117072	6117073	MACS_peak_14255	6.00	lobChIP_FOXA1_robot
chr7	6157273	6157274	MACS_peak_14256	4.00	lobChIP_FOXA1_robot
chr7	6278121	6278122	MACS_peak_14257	109.00	lobChIP_FOXA1_robot
chr7	6345154	6345155	MACS_peak_14258	5.00	lobChIP_FOXA1_robot
chr7	6416573	6416574	MACS_peak_14259	4.00	lobChIP_FOXA1_robot
chr7	6488169	6488170	MACS_peak_14260	11.00	lobChIP_FOXA1_robot
chr7	6521982	6521983	MACS_peak_14261	4.00	lobChIP_FOXA1_robot
chr7	6615251	6615252	MACS_peak_14262	4.00	lobChIP_FOXA1_robot
chr7	6618350	6618351	MACS_peak_14263	12.00	lobChIP_FOXA1_robot
chr7	7154564	7154565	MACS_peak_14264	31.00	lobChIP_FOXA1_robot
chr7	7158684	7158685	MACS_peak_14265	14.00	lobChIP_FOXA1_robot
chr7	7165787	7165788	MACS_peak_14266	9.00	lobChIP_FOXA1_robot
chr7	7176855	7176856	MACS_peak_14267	6.00	lobChIP_FOXA1_robot
chr7	7196443	7196444	MACS_peak_14268	4.00	lobChIP_FOXA1_robot
chr7	7213997	7213998	MACS_peak_14269	9.00	lobChIP_FOXA1_robot
chr7	7216895	7216896	MACS_peak_14270	16.00	lobChIP_FOXA1_robot
chr7	7539686	7539687	MACS_peak_14271	6.00	lobChIP_FOXA1_robot
chr7	7625609	7625610	MACS_peak_14272	3.00	lobChIP_FOXA1_robot
chr7	7682541	7682542	MACS_peak_14273	5.00	lobChIP_FOXA1_robot
chr7	7733707	7733708	MACS_peak_14274	4.00	lobChIP_FOXA1_robot
chr7	7736674	7736675	MACS_peak_14275	7.00	lobChIP_FOXA1_robot
chr7	7771480	7771481	MACS_peak_14276	6.00	lobChIP_FOXA1_robot
chr7	7791140	7791141	MACS_peak_14277	4.00	lobChIP_FOXA1_robot
chr7	7802736	7802737	MACS_peak_14278	5.00	lobChIP_FOXA1_robot
chr7	7951154	7951155	MACS_peak_14279	11.00	lobChIP_FOXA1_robot
chr7	7962526	7962527	MACS_peak_14280	9.00	lobChIP_FOXA1_robot
chr7	8215739	8215740	MACS_peak_14281	5.00	lobChIP_FOXA1_robot
chr7	9802683	9802684	MACS_peak_14282	3.00	lobChIP_FOXA1_robot
chr7	11044023	11044024	MACS_peak_14283	4.00	lobChIP_FOXA1_robot
chr7	12028036	12028037	MACS_peak_14284	7.00	lobChIP_FOXA1_robot
chr7	12251548	12251549	MACS_peak_14285	6.00	lobChIP_FOXA1_robot
chr7	12680087	12680088	MACS_peak_14286	10.00	lobChIP_FOXA1_robot
chr7	12687510	12687511	MACS_peak_14287	7.00	lobChIP_FOXA1_robot
chr7	12709986	12709987	MACS_peak_14288	8.00	lobChIP_FOXA1_robot
chr7	12791561	12791562	MACS_peak_14289	5.00	lobChIP_FOXA1_robot
chr7	12981280	12981281	MACS_peak_14290	14.00	lobChIP_FOXA1_robot
chr7	12982452	12982453	MACS_peak_14291	11.00	lobChIP_FOXA1_robot
chr7	13057227	13057228	MACS_peak_14292	18.00	lobChIP_FOXA1_robot
chr7	13061453	13061454	MACS_peak_14293	4.00	lobChIP_FOXA1_robot
chr7	13082469	13082470	MACS_peak_14294	3.00	lobChIP_FOXA1_robot
chr7	13090544	13090545	MACS_peak_14295	5.00	lobChIP_FOXA1_robot
chr7	13225022	13225023	MACS_peak_14296	23.00	lobChIP_FOXA1_robot
chr7	14609798	14609799	MACS_peak_14297	5.00	lobChIP_FOXA1_robot
chr7	15595340	15595341	MACS_peak_14298	6.00	lobChIP_FOXA1_robot
chr7	16461934	16461935	MACS_peak_14299	4.00	lobChIP_FOXA1_robot
chr7	16683761	16683762	MACS_peak_14300	5.00	lobChIP_FOXA1_robot
chr7	16691723	16691724	MACS_peak_14301	5.00	lobChIP_FOXA1_robot
chr7	16954464	16954465	MACS_peak_14302	3.00	lobChIP_FOXA1_robot
chr7	16967840	16967841	MACS_peak_14303	5.00	lobChIP_FOXA1_robot
chr7	16998561	16998562	MACS_peak_14304	6.00	lobChIP_FOXA1_robot
chr7	17018148	17018149	MACS_peak_14305	12.00	lobChIP_FOXA1_robot
chr7	17029672	17029673	MACS_peak_14306	28.00	lobChIP_FOXA1_robot
chr7	17056223	17056224	MACS_peak_14307	11.00	lobChIP_FOXA1_robot
chr7	17061045	17061046	MACS_peak_14308	12.00	lobChIP_FOXA1_robot
chr7	17139210	17139211	MACS_peak_14309	14.00	lobChIP_FOXA1_robot
chr7	17142518	17142519	MACS_peak_14310	7.00	lobChIP_FOXA1_robot
chr7	17170084	17170085	MACS_peak_14311	26.00	lobChIP_FOXA1_robot
chr7	17180202	17180203	MACS_peak_14312	5.00	lobChIP_FOXA1_robot
chr7	17182986	17182987	MACS_peak_14313	7.00	lobChIP_FOXA1_robot
chr7	17224651	17224652	MACS_peak_14314	18.00	lobChIP_FOXA1_robot
chr7	17246158	17246159	MACS_peak_14315	33.00	lobChIP_FOXA1_robot
chr7	17279561	17279562	MACS_peak_14316	38.00	lobChIP_FOXA1_robot
chr7	17315949	17315950	MACS_peak_14317	6.00	lobChIP_FOXA1_robot
chr7	17339557	17339558	MACS_peak_14318	5.00	lobChIP_FOXA1_robot
chr7	17360425	17360426	MACS_peak_14319	4.00	lobChIP_FOXA1_robot
chr7	17532289	17532290	MACS_peak_14320	7.00	lobChIP_FOXA1_robot
chr7	17545860	17545861	MACS_peak_14321	29.00	lobChIP_FOXA1_robot
chr7	17720718	17720719	MACS_peak_14322	10.00	lobChIP_FOXA1_robot
chr7	17796882	17796883	MACS_peak_14323	4.00	lobChIP_FOXA1_robot
chr7	20259708	20259709	MACS_peak_14324	16.00	lobChIP_FOXA1_robot
chr7	20319375	20319376	MACS_peak_14325	5.00	lobChIP_FOXA1_robot
chr7	20777744	20777745	MACS_peak_14326	5.00	lobChIP_FOXA1_robot
chr7	21403834	21403835	MACS_peak_14327	4.00	lobChIP_FOXA1_robot
chr7	21406097	21406098	MACS_peak_14328	8.00	lobChIP_FOXA1_robot
chr7	21994436	21994437	MACS_peak_14329	6.00	lobChIP_FOXA1_robot
chr7	22386166	22386167	MACS_peak_14330	8.00	lobChIP_FOXA1_robot
chr7	22391207	22391208	MACS_peak_14331	6.00	lobChIP_FOXA1_robot
chr7	22399114	22399115	MACS_peak_14332	14.00	lobChIP_FOXA1_robot
chr7	22418074	22418075	MACS_peak_14333	15.00	lobChIP_FOXA1_robot
chr7	22481892	22481893	MACS_peak_14334	19.00	lobChIP_FOXA1_robot
chr7	22599631	22599632	MACS_peak_14335	5.00	lobChIP_FOXA1_robot
chr7	22713132	22713133	MACS_peak_14336	11.00	lobChIP_FOXA1_robot
chr7	22813110	22813111	MACS_peak_14337	7.00	lobChIP_FOXA1_robot
chr7	22826228	22826229	MACS_peak_14338	19.00	lobChIP_FOXA1_robot
chr7	22854383	22854384	MACS_peak_14339	10.00	lobChIP_FOXA1_robot
chr7	22939872	22939873	MACS_peak_14340	4.00	lobChIP_FOXA1_robot
chr7	23114742	23114743	MACS_peak_14341	11.00	lobChIP_FOXA1_robot
chr7	23246880	23246881	MACS_peak_14342	5.00	lobChIP_FOXA1_robot
chr7	23312972	23312973	MACS_peak_14343	4.00	lobChIP_FOXA1_robot
chr7	23333475	23333476	MACS_peak_14344	8.00	lobChIP_FOXA1_robot
chr7	23360003	23360004	MACS_peak_14345	5.00	lobChIP_FOXA1_robot
chr7	23374420	23374421	MACS_peak_14346	36.00	lobChIP_FOXA1_robot
chr7	23376777	23376778	MACS_peak_14347	6.00	lobChIP_FOXA1_robot
chr7	23383867	23383868	MACS_peak_14348	5.00	lobChIP_FOXA1_robot
chr7	23396861	23396862	MACS_peak_14349	13.00	lobChIP_FOXA1_robot
chr7	23398326	23398327	MACS_peak_14350	6.00	lobChIP_FOXA1_robot
chr7	24517512	24517513	MACS_peak_14351	3.00	lobChIP_FOXA1_robot
chr7	24620378	24620379	MACS_peak_14352	5.00	lobChIP_FOXA1_robot
chr7	24684807	24684808	MACS_peak_14353	4.00	lobChIP_FOXA1_robot
chr7	24882683	24882684	MACS_peak_14354	8.00	lobChIP_FOXA1_robot
chr7	25065129	25065130	MACS_peak_14355	3.00	lobChIP_FOXA1_robot
chr7	25068558	25068559	MACS_peak_14356	5.00	lobChIP_FOXA1_robot
chr7	25289539	25289540	MACS_peak_14357	5.00	lobChIP_FOXA1_robot
chr7	25608259	25608260	MACS_peak_14358	3.00	lobChIP_FOXA1_robot
chr7	25872146	25872147	MACS_peak_14359	7.00	lobChIP_FOXA1_robot
chr7	25992424	25992425	MACS_peak_14360	5.00	lobChIP_FOXA1_robot
chr7	26041415	26041416	MACS_peak_14361	15.00	lobChIP_FOXA1_robot
chr7	26085276	26085277	MACS_peak_14362	4.00	lobChIP_FOXA1_robot
chr7	26452812	26452813	MACS_peak_14363	4.00	lobChIP_FOXA1_robot
chr7	26728163	26728164	MACS_peak_14364	5.00	lobChIP_FOXA1_robot
chr7	26989025	26989026	MACS_peak_14365	38.00	lobChIP_FOXA1_robot
chr7	27130907	27130908	MACS_peak_14366	9.00	lobChIP_FOXA1_robot
chr7	27160746	27160747	MACS_peak_14367	7.00	lobChIP_FOXA1_robot
chr7	27162204	27162205	MACS_peak_14368	4.00	lobChIP_FOXA1_robot
chr7	27175756	27175757	MACS_peak_14369	7.00	lobChIP_FOXA1_robot
chr7	27645943	27645944	MACS_peak_14370	5.00	lobChIP_FOXA1_robot
chr7	27702820	27702821	MACS_peak_14371	8.00	lobChIP_FOXA1_robot
chr7	27774971	27774972	MACS_peak_14372	9.00	lobChIP_FOXA1_robot
chr7	27778832	27778833	MACS_peak_14373	4.00	lobChIP_FOXA1_robot
chr7	27784403	27784404	MACS_peak_14374	6.00	lobChIP_FOXA1_robot
chr7	27824236	27824237	MACS_peak_14375	4.00	lobChIP_FOXA1_robot
chr7	28074490	28074491	MACS_peak_14376	14.00	lobChIP_FOXA1_robot
chr7	28195821	28195822	MACS_peak_14377	4.00	lobChIP_FOXA1_robot
chr7	29140277	29140278	MACS_peak_14378	12.00	lobChIP_FOXA1_robot
chr7	29155968	29155969	MACS_peak_14379	6.00	lobChIP_FOXA1_robot
chr7	29203661	29203662	MACS_peak_14380	11.00	lobChIP_FOXA1_robot
chr7	29230184	29230185	MACS_peak_14381	14.00	lobChIP_FOXA1_robot
chr7	29244648	29244649	MACS_peak_14382	5.00	lobChIP_FOXA1_robot
chr7	29248334	29248335	MACS_peak_14383	3.00	lobChIP_FOXA1_robot
chr7	29270918	29270919	MACS_peak_14384	5.00	lobChIP_FOXA1_robot
chr7	29329297	29329298	MACS_peak_14385	10.00	lobChIP_FOXA1_robot
chr7	29332037	29332038	MACS_peak_14386	4.00	lobChIP_FOXA1_robot
chr7	29367899	29367900	MACS_peak_14387	4.00	lobChIP_FOXA1_robot
chr7	29376834	29376835	MACS_peak_14388	17.00	lobChIP_FOXA1_robot
chr7	29445858	29445859	MACS_peak_14389	4.00	lobChIP_FOXA1_robot
chr7	29489722	29489723	MACS_peak_14390	11.00	lobChIP_FOXA1_robot
chr7	29498061	29498062	MACS_peak_14391	4.00	lobChIP_FOXA1_robot
chr7	29511625	29511626	MACS_peak_14392	3.00	lobChIP_FOXA1_robot
chr7	29518582	29518583	MACS_peak_14393	5.00	lobChIP_FOXA1_robot
chr7	29523056	29523057	MACS_peak_14394	10.00	lobChIP_FOXA1_robot
chr7	30027740	30027741	MACS_peak_14395	13.00	lobChIP_FOXA1_robot
chr7	30278693	30278694	MACS_peak_14396	6.00	lobChIP_FOXA1_robot
chr7	30295105	30295106	MACS_peak_14397	8.00	lobChIP_FOXA1_robot
chr7	30302844	30302845	MACS_peak_14398	8.00	lobChIP_FOXA1_robot
chr7	30344327	30344328	MACS_peak_14399	3.00	lobChIP_FOXA1_robot
chr7	30588591	30588592	MACS_peak_14400	12.00	lobChIP_FOXA1_robot
chr7	31030586	31030587	MACS_peak_14401	3.00	lobChIP_FOXA1_robot
chr7	32555688	32555689	MACS_peak_14402	4.00	lobChIP_FOXA1_robot
chr7	32574826	32574827	MACS_peak_14403	3.00	lobChIP_FOXA1_robot
chr7	32627503	32627504	MACS_peak_14404	9.00	lobChIP_FOXA1_robot
chr7	32708208	32708209	MACS_peak_14405	25.00	lobChIP_FOXA1_robot
chr7	32709903	32709904	MACS_peak_14406	8.00	lobChIP_FOXA1_robot
chr7	33255764	33255765	MACS_peak_14407	9.00	lobChIP_FOXA1_robot
chr7	33259963	33259964	MACS_peak_14408	4.00	lobChIP_FOXA1_robot
chr7	34494225	34494226	MACS_peak_14409	9.00	lobChIP_FOXA1_robot
chr7	34788333	34788334	MACS_peak_14410	6.00	lobChIP_FOXA1_robot
chr7	34797825	34797826	MACS_peak_14411	5.00	lobChIP_FOXA1_robot
chr7	34978840	34978841	MACS_peak_14412	3.00	lobChIP_FOXA1_robot
chr7	35871048	35871049	MACS_peak_14413	4.00	lobChIP_FOXA1_robot
chr7	36191804	36191805	MACS_peak_14414	3.00	lobChIP_FOXA1_robot
chr7	36201821	36201822	MACS_peak_14415	5.00	lobChIP_FOXA1_robot
chr7	36204886	36204887	MACS_peak_14416	10.00	lobChIP_FOXA1_robot
chr7	36235577	36235578	MACS_peak_14417	17.00	lobChIP_FOXA1_robot
chr7	36298266	36298267	MACS_peak_14418	4.00	lobChIP_FOXA1_robot
chr7	36312794	36312795	MACS_peak_14419	8.00	lobChIP_FOXA1_robot
chr7	36327147	36327148	MACS_peak_14420	9.00	lobChIP_FOXA1_robot
chr7	36329923	36329924	MACS_peak_14421	4.00	lobChIP_FOXA1_robot
chr7	36835346	36835347	MACS_peak_14422	8.00	lobChIP_FOXA1_robot
chr7	36985869	36985870	MACS_peak_14423	7.00	lobChIP_FOXA1_robot
chr7	37011252	37011253	MACS_peak_14424	82.00	lobChIP_FOXA1_robot
chr7	37055907	37055908	MACS_peak_14425	5.00	lobChIP_FOXA1_robot
chr7	37114000	37114001	MACS_peak_14426	10.00	lobChIP_FOXA1_robot
chr7	37132832	37132833	MACS_peak_14427	5.00	lobChIP_FOXA1_robot
chr7	37134891	37134892	MACS_peak_14428	5.00	lobChIP_FOXA1_robot
chr7	37148894	37148895	MACS_peak_14429	5.00	lobChIP_FOXA1_robot
chr7	37189968	37189969	MACS_peak_14430	7.00	lobChIP_FOXA1_robot
chr7	37231619	37231620	MACS_peak_14431	10.00	lobChIP_FOXA1_robot
chr7	37982933	37982934	MACS_peak_14432	5.00	lobChIP_FOXA1_robot
chr7	38602465	38602466	MACS_peak_14433	4.00	lobChIP_FOXA1_robot
chr7	39416549	39416550	MACS_peak_14434	5.00	lobChIP_FOXA1_robot
chr7	39580138	39580139	MACS_peak_14435	7.00	lobChIP_FOXA1_robot
chr7	39605446	39605447	MACS_peak_14436	4.00	lobChIP_FOXA1_robot
chr7	39667683	39667684	MACS_peak_14437	8.00	lobChIP_FOXA1_robot
chr7	40138645	40138646	MACS_peak_14438	6.00	lobChIP_FOXA1_robot
chr7	40139245	40139246	MACS_peak_14439	4.00	lobChIP_FOXA1_robot
chr7	40443430	40443431	MACS_peak_14440	29.00	lobChIP_FOXA1_robot
chr7	40681499	40681500	MACS_peak_14441	4.00	lobChIP_FOXA1_robot
chr7	40773009	40773010	MACS_peak_14442	9.00	lobChIP_FOXA1_robot
chr7	40800100	40800101	MACS_peak_14443	8.00	lobChIP_FOXA1_robot
chr7	40800889	40800890	MACS_peak_14444	5.00	lobChIP_FOXA1_robot
chr7	41506583	41506584	MACS_peak_14445	4.00	lobChIP_FOXA1_robot
chr7	41982212	41982213	MACS_peak_14446	8.00	lobChIP_FOXA1_robot
chr7	42011630	42011631	MACS_peak_14447	5.00	lobChIP_FOXA1_robot
chr7	42131430	42131431	MACS_peak_14448	12.00	lobChIP_FOXA1_robot
chr7	43022350	43022351	MACS_peak_14449	4.00	lobChIP_FOXA1_robot
chr7	43524604	43524605	MACS_peak_14450	4.00	lobChIP_FOXA1_robot
chr7	43526800	43526801	MACS_peak_14451	20.00	lobChIP_FOXA1_robot
chr7	43541645	43541646	MACS_peak_14452	6.00	lobChIP_FOXA1_robot
chr7	43626181	43626182	MACS_peak_14453	6.00	lobChIP_FOXA1_robot
chr7	43675964	43675965	MACS_peak_14454	10.00	lobChIP_FOXA1_robot
chr7	43699260	43699261	MACS_peak_14455	10.00	lobChIP_FOXA1_robot
chr7	43714387	43714388	MACS_peak_14456	5.00	lobChIP_FOXA1_robot
chr7	43804804	43804805	MACS_peak_14457	9.00	lobChIP_FOXA1_robot
chr7	43821976	43821977	MACS_peak_14458	8.00	lobChIP_FOXA1_robot
chr7	43944971	43944972	MACS_peak_14459	5.00	lobChIP_FOXA1_robot
chr7	44510617	44510618	MACS_peak_14460	4.00	lobChIP_FOXA1_robot
chr7	44513068	44513069	MACS_peak_14461	5.00	lobChIP_FOXA1_robot
chr7	45011847	45011848	MACS_peak_14462	4.00	lobChIP_FOXA1_robot
chr7	45076071	45076072	MACS_peak_14463	5.00	lobChIP_FOXA1_robot
chr7	45678738	45678739	MACS_peak_14464	9.00	lobChIP_FOXA1_robot
chr7	45731024	45731025	MACS_peak_14465	38.00	lobChIP_FOXA1_robot
chr7	45921759	45921760	MACS_peak_14466	17.00	lobChIP_FOXA1_robot
chr7	45925130	45925131	MACS_peak_14467	15.00	lobChIP_FOXA1_robot
chr7	45933281	45933282	MACS_peak_14468	4.00	lobChIP_FOXA1_robot
chr7	46403382	46403383	MACS_peak_14469	5.00	lobChIP_FOXA1_robot
chr7	47332719	47332720	MACS_peak_14470	50.00	lobChIP_FOXA1_robot
chr7	47510764	47510765	MACS_peak_14471	6.00	lobChIP_FOXA1_robot
chr7	47515300	47515301	MACS_peak_14472	7.00	lobChIP_FOXA1_robot
chr7	47578573	47578574	MACS_peak_14473	3.00	lobChIP_FOXA1_robot
chr7	47611118	47611119	MACS_peak_14474	4.00	lobChIP_FOXA1_robot
chr7	47613458	47613459	MACS_peak_14475	9.00	lobChIP_FOXA1_robot
chr7	47615934	47615935	MACS_peak_14476	4.00	lobChIP_FOXA1_robot
chr7	47620154	47620155	MACS_peak_14477	6.00	lobChIP_FOXA1_robot
chr7	47631418	47631419	MACS_peak_14478	5.00	lobChIP_FOXA1_robot
chr7	47644755	47644756	MACS_peak_14479	27.00	lobChIP_FOXA1_robot
chr7	47662662	47662663	MACS_peak_14480	4.00	lobChIP_FOXA1_robot
chr7	47663071	47663072	MACS_peak_14481	5.00	lobChIP_FOXA1_robot
chr7	47723266	47723267	MACS_peak_14482	4.00	lobChIP_FOXA1_robot
chr7	47755501	47755502	MACS_peak_14483	8.00	lobChIP_FOXA1_robot
chr7	47774223	47774224	MACS_peak_14484	10.00	lobChIP_FOXA1_robot
chr7	47803408	47803409	MACS_peak_14485	47.00	lobChIP_FOXA1_robot
chr7	47818063	47818064	MACS_peak_14486	3.00	lobChIP_FOXA1_robot
chr7	47822983	47822984	MACS_peak_14487	6.00	lobChIP_FOXA1_robot
chr7	47842740	47842741	MACS_peak_14488	6.00	lobChIP_FOXA1_robot
chr7	47991180	47991181	MACS_peak_14489	16.00	lobChIP_FOXA1_robot
chr7	48007743	48007744	MACS_peak_14490	4.00	lobChIP_FOXA1_robot
chr7	48179804	48179805	MACS_peak_14491	12.00	lobChIP_FOXA1_robot
chr7	48184056	48184057	MACS_peak_14492	11.00	lobChIP_FOXA1_robot
chr7	50786738	50786739	MACS_peak_14493	17.00	lobChIP_FOXA1_robot
chr7	50806793	50806794	MACS_peak_14494	5.00	lobChIP_FOXA1_robot
chr7	50825548	50825549	MACS_peak_14495	3.00	lobChIP_FOXA1_robot
chr7	51230595	51230596	MACS_peak_14496	6.00	lobChIP_FOXA1_robot
chr7	51382907	51382908	MACS_peak_14497	5.00	lobChIP_FOXA1_robot
chr7	51396342	51396343	MACS_peak_14498	5.00	lobChIP_FOXA1_robot
chr7	55055657	55055658	MACS_peak_14499	4.00	lobChIP_FOXA1_robot
chr7	55195400	55195401	MACS_peak_14500	3.00	lobChIP_FOXA1_robot
chr7	55246512	55246513	MACS_peak_14501	7.00	lobChIP_FOXA1_robot
chr7	55256319	55256320	MACS_peak_14502	6.00	lobChIP_FOXA1_robot
chr7	55766953	55766954	MACS_peak_14503	5.00	lobChIP_FOXA1_robot
chr7	56097426	56097427	MACS_peak_14504	6.00	lobChIP_FOXA1_robot
chr7	56114649	56114650	MACS_peak_14505	8.00	lobChIP_FOXA1_robot
chr7	56116234	56116235	MACS_peak_14506	5.00	lobChIP_FOXA1_robot
chr7	64460022	64460023	MACS_peak_14509	16.00	lobChIP_FOXA1_robot
chr7	64687129	64687130	MACS_peak_14510	4.00	lobChIP_FOXA1_robot
chr7	64861178	64861179	MACS_peak_14511	3.00	lobChIP_FOXA1_robot
chr7	64862770	64862771	MACS_peak_14512	6.00	lobChIP_FOXA1_robot
chr7	64910232	64910233	MACS_peak_14513	27.00	lobChIP_FOXA1_robot
chr7	64940740	64940741	MACS_peak_14514	6.00	lobChIP_FOXA1_robot
chr7	65210838	65210839	MACS_peak_14515	6.00	lobChIP_FOXA1_robot
chr7	65226594	65226595	MACS_peak_14516	4.00	lobChIP_FOXA1_robot
chr7	65372708	65372709	MACS_peak_14517	4.00	lobChIP_FOXA1_robot
chr7	65607480	65607481	MACS_peak_14518	11.00	lobChIP_FOXA1_robot
chr7	65904958	65904959	MACS_peak_14519	8.00	lobChIP_FOXA1_robot
chr7	66238066	66238067	MACS_peak_14520	9.00	lobChIP_FOXA1_robot
chr7	66247989	66247990	MACS_peak_14521	8.00	lobChIP_FOXA1_robot
chr7	67229752	67229753	MACS_peak_14522	4.00	lobChIP_FOXA1_robot
chr7	68654954	68654955	MACS_peak_14523	4.00	lobChIP_FOXA1_robot
chr7	70210156	70210157	MACS_peak_14524	3.00	lobChIP_FOXA1_robot
chr7	71082016	71082017	MACS_peak_14525	5.00	lobChIP_FOXA1_robot
chr7	71387616	71387617	MACS_peak_14526	5.00	lobChIP_FOXA1_robot
chr7	71909700	71909701	MACS_peak_14527	3.00	lobChIP_FOXA1_robot
chr7	72148840	72148841	MACS_peak_14528	10.00	lobChIP_FOXA1_robot
chr7	72772519	72772520	MACS_peak_14529	13.00	lobChIP_FOXA1_robot
chr7	73003877	73003878	MACS_peak_14530	10.00	lobChIP_FOXA1_robot
chr7	73027257	73027258	MACS_peak_14531	8.00	lobChIP_FOXA1_robot
chr7	73994832	73994833	MACS_peak_14532	5.00	lobChIP_FOXA1_robot
chr7	74024872	74024873	MACS_peak_14533	6.00	lobChIP_FOXA1_robot
chr7	74106800	74106801	MACS_peak_14534	4.00	lobChIP_FOXA1_robot
chr7	75282347	75282348	MACS_peak_14535	7.00	lobChIP_FOXA1_robot
chr7	75305478	75305479	MACS_peak_14536	9.00	lobChIP_FOXA1_robot
chr7	75357334	75357335	MACS_peak_14537	6.00	lobChIP_FOXA1_robot
chr7	75536917	75536918	MACS_peak_14538	15.00	lobChIP_FOXA1_robot
chr7	75550128	75550129	MACS_peak_14539	4.00	lobChIP_FOXA1_robot
chr7	75585677	75585678	MACS_peak_14540	4.00	lobChIP_FOXA1_robot
chr7	75588290	75588291	MACS_peak_14541	6.00	lobChIP_FOXA1_robot
chr7	75738015	75738016	MACS_peak_14542	10.00	lobChIP_FOXA1_robot
chr7	75747742	75747743	MACS_peak_14543	12.00	lobChIP_FOXA1_robot
chr7	75874561	75874562	MACS_peak_14544	5.00	lobChIP_FOXA1_robot
chr7	75878873	75878874	MACS_peak_14545	10.00	lobChIP_FOXA1_robot
chr7	76989628	76989629	MACS_peak_14546	14.00	lobChIP_FOXA1_robot
chr7	77026386	77026387	MACS_peak_14547	4.00	lobChIP_FOXA1_robot
chr7	77038861	77038862	MACS_peak_14548	19.00	lobChIP_FOXA1_robot
chr7	77039542	77039543	MACS_peak_14549	6.00	lobChIP_FOXA1_robot
chr7	77088755	77088756	MACS_peak_14550	30.00	lobChIP_FOXA1_robot
chr7	77111463	77111464	MACS_peak_14551	4.00	lobChIP_FOXA1_robot
chr7	77204695	77204696	MACS_peak_14552	5.00	lobChIP_FOXA1_robot
chr7	77321525	77321526	MACS_peak_14553	4.00	lobChIP_FOXA1_robot
chr7	77496239	77496240	MACS_peak_14554	4.00	lobChIP_FOXA1_robot
chr7	77755362	77755363	MACS_peak_14555	10.00	lobChIP_FOXA1_robot
chr7	77759448	77759449	MACS_peak_14556	5.00	lobChIP_FOXA1_robot
chr7	78704017	78704018	MACS_peak_14557	5.00	lobChIP_FOXA1_robot
chr7	78841257	78841258	MACS_peak_14558	5.00	lobChIP_FOXA1_robot
chr7	78846998	78846999	MACS_peak_14559	7.00	lobChIP_FOXA1_robot
chr7	78923113	78923114	MACS_peak_14560	7.00	lobChIP_FOXA1_robot
chr7	78946962	78946963	MACS_peak_14561	5.00	lobChIP_FOXA1_robot
chr7	79006225	79006226	MACS_peak_14562	3.00	lobChIP_FOXA1_robot
chr7	79091189	79091190	MACS_peak_14563	8.00	lobChIP_FOXA1_robot
chr7	79117122	79117123	MACS_peak_14564	4.00	lobChIP_FOXA1_robot
chr7	79136594	79136595	MACS_peak_14565	5.00	lobChIP_FOXA1_robot
chr7	79253960	79253961	MACS_peak_14566	3.00	lobChIP_FOXA1_robot
chr7	79359672	79359673	MACS_peak_14567	3.00	lobChIP_FOXA1_robot
chr7	79440868	79440869	MACS_peak_14568	4.00	lobChIP_FOXA1_robot
chr7	79839925	79839926	MACS_peak_14569	20.00	lobChIP_FOXA1_robot
chr7	79991678	79991679	MACS_peak_14570	4.00	lobChIP_FOXA1_robot
chr7	80023352	80023353	MACS_peak_14571	5.00	lobChIP_FOXA1_robot
chr7	80144878	80144879	MACS_peak_14572	6.00	lobChIP_FOXA1_robot
chr7	80241386	80241387	MACS_peak_14573	19.00	lobChIP_FOXA1_robot
chr7	80264052	80264053	MACS_peak_14574	3.00	lobChIP_FOXA1_robot
chr7	80272342	80272343	MACS_peak_14575	8.00	lobChIP_FOXA1_robot
chr7	80308238	80308239	MACS_peak_14576	5.00	lobChIP_FOXA1_robot
chr7	80389736	80389737	MACS_peak_14577	4.00	lobChIP_FOXA1_robot
chr7	80452600	80452601	MACS_peak_14578	12.00	lobChIP_FOXA1_robot
chr7	80481369	80481370	MACS_peak_14579	5.00	lobChIP_FOXA1_robot
chr7	80785334	80785335	MACS_peak_14580	12.00	lobChIP_FOXA1_robot
chr7	80804534	80804535	MACS_peak_14581	4.00	lobChIP_FOXA1_robot
chr7	82234776	82234777	MACS_peak_14582	4.00	lobChIP_FOXA1_robot
chr7	83652123	83652124	MACS_peak_14583	7.00	lobChIP_FOXA1_robot
chr7	83807444	83807445	MACS_peak_14584	5.00	lobChIP_FOXA1_robot
chr7	84054355	84054356	MACS_peak_14585	5.00	lobChIP_FOXA1_robot
chr7	84545716	84545717	MACS_peak_14586	10.00	lobChIP_FOXA1_robot
chr7	85221361	85221362	MACS_peak_14587	24.00	lobChIP_FOXA1_robot
chr7	85332108	85332109	MACS_peak_14588	4.00	lobChIP_FOXA1_robot
chr7	86476306	86476307	MACS_peak_14589	13.00	lobChIP_FOXA1_robot
chr7	86582728	86582729	MACS_peak_14590	9.00	lobChIP_FOXA1_robot
chr7	87043943	87043944	MACS_peak_14591	4.00	lobChIP_FOXA1_robot
chr7	87094669	87094670	MACS_peak_14592	8.00	lobChIP_FOXA1_robot
chr7	87178075	87178076	MACS_peak_14593	10.00	lobChIP_FOXA1_robot
chr7	87228739	87228740	MACS_peak_14594	6.00	lobChIP_FOXA1_robot
chr7	87462579	87462580	MACS_peak_14595	5.00	lobChIP_FOXA1_robot
chr7	87780614	87780615	MACS_peak_14596	6.00	lobChIP_FOXA1_robot
chr7	87856493	87856494	MACS_peak_14597	7.00	lobChIP_FOXA1_robot
chr7	88542404	88542405	MACS_peak_14598	4.00	lobChIP_FOXA1_robot
chr7	89802927	89802928	MACS_peak_14599	5.00	lobChIP_FOXA1_robot
chr7	89899930	89899931	MACS_peak_14600	7.00	lobChIP_FOXA1_robot
chr7	90347214	90347215	MACS_peak_14601	4.00	lobChIP_FOXA1_robot
chr7	90388664	90388665	MACS_peak_14602	28.00	lobChIP_FOXA1_robot
chr7	90396616	90396617	MACS_peak_14603	4.00	lobChIP_FOXA1_robot
chr7	91195134	91195135	MACS_peak_14604	6.00	lobChIP_FOXA1_robot
chr7	91197846	91197847	MACS_peak_14605	3.00	lobChIP_FOXA1_robot
chr7	91671556	91671557	MACS_peak_14606	10.00	lobChIP_FOXA1_robot
chr7	91691416	91691417	MACS_peak_14607	5.00	lobChIP_FOXA1_robot
chr7	92262907	92262908	MACS_peak_14608	6.00	lobChIP_FOXA1_robot
chr7	92307775	92307776	MACS_peak_14609	49.00	lobChIP_FOXA1_robot
chr7	92312522	92312523	MACS_peak_14610	8.00	lobChIP_FOXA1_robot
chr7	92325063	92325064	MACS_peak_14611	21.00	lobChIP_FOXA1_robot
chr7	92326006	92326007	MACS_peak_14612	4.00	lobChIP_FOXA1_robot
chr7	92369561	92369562	MACS_peak_14613	5.00	lobChIP_FOXA1_robot
chr7	92381710	92381711	MACS_peak_14614	27.00	lobChIP_FOXA1_robot
chr7	92854435	92854436	MACS_peak_14615	3.00	lobChIP_FOXA1_robot
chr7	92857760	92857761	MACS_peak_14616	6.00	lobChIP_FOXA1_robot
chr7	93208367	93208368	MACS_peak_14617	4.00	lobChIP_FOXA1_robot
chr7	94070275	94070276	MACS_peak_14618	7.00	lobChIP_FOXA1_robot
chr7	94137014	94137015	MACS_peak_14619	4.00	lobChIP_FOXA1_robot
chr7	94191358	94191359	MACS_peak_14620	15.00	lobChIP_FOXA1_robot
chr7	94210001	94210002	MACS_peak_14621	8.00	lobChIP_FOXA1_robot
chr7	94253327	94253328	MACS_peak_14622	39.00	lobChIP_FOXA1_robot
chr7	94259246	94259247	MACS_peak_14623	6.00	lobChIP_FOXA1_robot
chr7	94260345	94260346	MACS_peak_14624	6.00	lobChIP_FOXA1_robot
chr7	94272724	94272725	MACS_peak_14625	20.00	lobChIP_FOXA1_robot
chr7	94280101	94280102	MACS_peak_14626	9.00	lobChIP_FOXA1_robot
chr7	94292394	94292395	MACS_peak_14627	4.00	lobChIP_FOXA1_robot
chr7	94331058	94331059	MACS_peak_14628	5.00	lobChIP_FOXA1_robot
chr7	94571757	94571758	MACS_peak_14629	5.00	lobChIP_FOXA1_robot
chr7	94601819	94601820	MACS_peak_14630	7.00	lobChIP_FOXA1_robot
chr7	94610273	94610274	MACS_peak_14631	30.00	lobChIP_FOXA1_robot
chr7	94658221	94658222	MACS_peak_14632	24.00	lobChIP_FOXA1_robot
chr7	94755400	94755401	MACS_peak_14633	3.00	lobChIP_FOXA1_robot
chr7	95060500	95060501	MACS_peak_14634	3.00	lobChIP_FOXA1_robot
chr7	95069905	95069906	MACS_peak_14635	21.00	lobChIP_FOXA1_robot
chr7	95099879	95099880	MACS_peak_14636	10.00	lobChIP_FOXA1_robot
chr7	95108101	95108102	MACS_peak_14637	7.00	lobChIP_FOXA1_robot
chr7	95119818	95119819	MACS_peak_14638	4.00	lobChIP_FOXA1_robot
chr7	95129048	95129049	MACS_peak_14639	11.00	lobChIP_FOXA1_robot
chr7	95130048	95130049	MACS_peak_14640	4.00	lobChIP_FOXA1_robot
chr7	95219926	95219927	MACS_peak_14641	17.00	lobChIP_FOXA1_robot
chr7	95238201	95238202	MACS_peak_14642	9.00	lobChIP_FOXA1_robot
chr7	95804281	95804282	MACS_peak_14643	3.00	lobChIP_FOXA1_robot
chr7	95929905	95929906	MACS_peak_14644	5.00	lobChIP_FOXA1_robot
chr7	95932666	95932667	MACS_peak_14645	22.00	lobChIP_FOXA1_robot
chr7	95956914	95956915	MACS_peak_14646	5.00	lobChIP_FOXA1_robot
chr7	96178136	96178137	MACS_peak_14647	4.00	lobChIP_FOXA1_robot
chr7	96328914	96328915	MACS_peak_14648	4.00	lobChIP_FOXA1_robot
chr7	96463106	96463107	MACS_peak_14649	9.00	lobChIP_FOXA1_robot
chr7	96600213	96600214	MACS_peak_14650	8.00	lobChIP_FOXA1_robot
chr7	97601926	97601927	MACS_peak_14651	4.00	lobChIP_FOXA1_robot
chr7	97740166	97740167	MACS_peak_14652	10.00	lobChIP_FOXA1_robot
chr7	97748769	97748770	MACS_peak_14653	5.00	lobChIP_FOXA1_robot
chr7	97987146	97987147	MACS_peak_14654	3.00	lobChIP_FOXA1_robot
chr7	98017428	98017429	MACS_peak_14655	4.00	lobChIP_FOXA1_robot
chr7	98048463	98048464	MACS_peak_14656	12.00	lobChIP_FOXA1_robot
chr7	98117731	98117732	MACS_peak_14657	6.00	lobChIP_FOXA1_robot
chr7	98520507	98520508	MACS_peak_14658	4.00	lobChIP_FOXA1_robot
chr7	98690058	98690059	MACS_peak_14659	8.00	lobChIP_FOXA1_robot
chr7	98707568	98707569	MACS_peak_14660	10.00	lobChIP_FOXA1_robot
chr7	98788444	98788445	MACS_peak_14661	4.00	lobChIP_FOXA1_robot
chr7	98805151	98805152	MACS_peak_14662	5.00	lobChIP_FOXA1_robot
chr7	98805771	98805772	MACS_peak_14663	4.00	lobChIP_FOXA1_robot
chr7	98808058	98808059	MACS_peak_14664	6.00	lobChIP_FOXA1_robot
chr7	98809592	98809593	MACS_peak_14665	7.00	lobChIP_FOXA1_robot
chr7	98868487	98868488	MACS_peak_14666	5.00	lobChIP_FOXA1_robot
chr7	99191994	99191995	MACS_peak_14667	4.00	lobChIP_FOXA1_robot
chr7	99275344	99275345	MACS_peak_14668	6.00	lobChIP_FOXA1_robot
chr7	99283212	99283213	MACS_peak_14669	5.00	lobChIP_FOXA1_robot
chr7	99291215	99291216	MACS_peak_14670	6.00	lobChIP_FOXA1_robot
chr7	99292117	99292118	MACS_peak_14671	15.00	lobChIP_FOXA1_robot
chr7	99370304	99370305	MACS_peak_14672	5.00	lobChIP_FOXA1_robot
chr7	99392979	99392980	MACS_peak_14673	4.00	lobChIP_FOXA1_robot
chr7	99425121	99425122	MACS_peak_14674	17.00	lobChIP_FOXA1_robot
chr7	99425861	99425862	MACS_peak_14675	15.00	lobChIP_FOXA1_robot
chr7	99427258	99427259	MACS_peak_14676	5.00	lobChIP_FOXA1_robot
chr7	99601590	99601591	MACS_peak_14677	23.00	lobChIP_FOXA1_robot
chr7	99634446	99634447	MACS_peak_14678	11.00	lobChIP_FOXA1_robot
chr7	99678579	99678580	MACS_peak_14679	12.00	lobChIP_FOXA1_robot
chr7	99698157	99698158	MACS_peak_14680	5.00	lobChIP_FOXA1_robot
chr7	99839834	99839835	MACS_peak_14681	7.00	lobChIP_FOXA1_robot
chr7	99842760	99842761	MACS_peak_14682	5.00	lobChIP_FOXA1_robot
chr7	99845181	99845182	MACS_peak_14683	7.00	lobChIP_FOXA1_robot
chr7	100143907	100143908	MACS_peak_14684	15.00	lobChIP_FOXA1_robot
chr7	100250794	100250795	MACS_peak_14685	8.00	lobChIP_FOXA1_robot
chr7	100317475	100317476	MACS_peak_14686	6.00	lobChIP_FOXA1_robot
chr7	100399674	100399675	MACS_peak_14687	9.00	lobChIP_FOXA1_robot
chr7	100438565	100438566	MACS_peak_14688	5.00	lobChIP_FOXA1_robot
chr7	100670997	100670998	MACS_peak_14689	5.00	lobChIP_FOXA1_robot
chr7	100768233	100768234	MACS_peak_14690	13.00	lobChIP_FOXA1_robot
chr7	100769653	100769654	MACS_peak_14691	8.00	lobChIP_FOXA1_robot
chr7	100844366	100844367	MACS_peak_14692	4.00	lobChIP_FOXA1_robot
chr7	100887695	100887696	MACS_peak_14693	4.00	lobChIP_FOXA1_robot
chr7	100995715	100995716	MACS_peak_14694	9.00	lobChIP_FOXA1_robot
chr7	101094402	101094403	MACS_peak_14695	7.00	lobChIP_FOXA1_robot
chr7	101241010	101241011	MACS_peak_14696	11.00	lobChIP_FOXA1_robot
chr7	101337172	101337173	MACS_peak_14697	6.00	lobChIP_FOXA1_robot
chr7	101526382	101526383	MACS_peak_14698	11.00	lobChIP_FOXA1_robot
chr7	101619011	101619012	MACS_peak_14699	7.00	lobChIP_FOXA1_robot
chr7	101656350	101656351	MACS_peak_14700	8.00	lobChIP_FOXA1_robot
chr7	101764958	101764959	MACS_peak_14701	6.00	lobChIP_FOXA1_robot
chr7	101901407	101901408	MACS_peak_14702	4.00	lobChIP_FOXA1_robot
chr7	102452653	102452654	MACS_peak_14703	5.00	lobChIP_FOXA1_robot
chr7	102509775	102509776	MACS_peak_14704	5.00	lobChIP_FOXA1_robot
chr7	102514767	102514768	MACS_peak_14705	5.00	lobChIP_FOXA1_robot
chr7	102747118	102747119	MACS_peak_14706	5.00	lobChIP_FOXA1_robot
chr7	103029079	103029080	MACS_peak_14707	5.00	lobChIP_FOXA1_robot
chr7	103029433	103029434	MACS_peak_14708	7.00	lobChIP_FOXA1_robot
chr7	103128005	103128006	MACS_peak_14709	12.00	lobChIP_FOXA1_robot
chr7	103301233	103301234	MACS_peak_14710	47.00	lobChIP_FOXA1_robot
chr7	103450069	103450070	MACS_peak_14711	4.00	lobChIP_FOXA1_robot
chr7	103507602	103507603	MACS_peak_14712	12.00	lobChIP_FOXA1_robot
chr7	103651183	103651184	MACS_peak_14713	5.00	lobChIP_FOXA1_robot
chr7	103675719	103675720	MACS_peak_14714	3.00	lobChIP_FOXA1_robot
chr7	103705584	103705585	MACS_peak_14715	62.00	lobChIP_FOXA1_robot
chr7	103912640	103912641	MACS_peak_14716	7.00	lobChIP_FOXA1_robot
chr7	104838771	104838772	MACS_peak_14717	5.00	lobChIP_FOXA1_robot
chr7	104944550	104944551	MACS_peak_14718	4.00	lobChIP_FOXA1_robot
chr7	104945064	104945065	MACS_peak_14719	5.00	lobChIP_FOXA1_robot
chr7	105008149	105008150	MACS_peak_14720	11.00	lobChIP_FOXA1_robot
chr7	105070414	105070415	MACS_peak_14721	4.00	lobChIP_FOXA1_robot
chr7	105289469	105289470	MACS_peak_14722	5.00	lobChIP_FOXA1_robot
chr7	105469441	105469442	MACS_peak_14723	9.00	lobChIP_FOXA1_robot
chr7	105471235	105471236	MACS_peak_14724	3.00	lobChIP_FOXA1_robot
chr7	105712575	105712576	MACS_peak_14725	3.00	lobChIP_FOXA1_robot
chr7	105968442	105968443	MACS_peak_14726	3.00	lobChIP_FOXA1_robot
chr7	106809074	106809075	MACS_peak_14727	7.00	lobChIP_FOXA1_robot
chr7	107384025	107384026	MACS_peak_14728	5.00	lobChIP_FOXA1_robot
chr7	107420122	107420123	MACS_peak_14729	7.00	lobChIP_FOXA1_robot
chr7	107589448	107589449	MACS_peak_14730	9.00	lobChIP_FOXA1_robot
chr7	107608061	107608062	MACS_peak_14731	17.00	lobChIP_FOXA1_robot
chr7	107612680	107612681	MACS_peak_14732	4.00	lobChIP_FOXA1_robot
chr7	107636061	107636062	MACS_peak_14733	3.00	lobChIP_FOXA1_robot
chr7	107653127	107653128	MACS_peak_14734	6.00	lobChIP_FOXA1_robot
chr7	107655734	107655735	MACS_peak_14735	17.00	lobChIP_FOXA1_robot
chr7	107668714	107668715	MACS_peak_14736	24.00	lobChIP_FOXA1_robot
chr7	107675227	107675228	MACS_peak_14737	29.00	lobChIP_FOXA1_robot
chr7	107690575	107690576	MACS_peak_14738	5.00	lobChIP_FOXA1_robot
chr7	107696206	107696207	MACS_peak_14739	7.00	lobChIP_FOXA1_robot
chr7	107707703	107707704	MACS_peak_14740	4.00	lobChIP_FOXA1_robot
chr7	107731998	107731999	MACS_peak_14741	6.00	lobChIP_FOXA1_robot
chr7	107735526	107735527	MACS_peak_14742	5.00	lobChIP_FOXA1_robot
chr7	107992328	107992329	MACS_peak_14743	7.00	lobChIP_FOXA1_robot
chr7	110163072	110163073	MACS_peak_14744	11.00	lobChIP_FOXA1_robot
chr7	110317680	110317681	MACS_peak_14745	6.00	lobChIP_FOXA1_robot
chr7	110832848	110832849	MACS_peak_14746	6.00	lobChIP_FOXA1_robot
chr7	111485154	111485155	MACS_peak_14747	4.00	lobChIP_FOXA1_robot
chr7	111559858	111559859	MACS_peak_14748	8.00	lobChIP_FOXA1_robot
chr7	111560557	111560558	MACS_peak_14749	7.00	lobChIP_FOXA1_robot
chr7	111571426	111571427	MACS_peak_14750	5.00	lobChIP_FOXA1_robot
chr7	111581728	111581729	MACS_peak_14751	5.00	lobChIP_FOXA1_robot
chr7	111649443	111649444	MACS_peak_14752	5.00	lobChIP_FOXA1_robot
chr7	111726786	111726787	MACS_peak_14753	4.00	lobChIP_FOXA1_robot
chr7	111800924	111800925	MACS_peak_14754	15.00	lobChIP_FOXA1_robot
chr7	111802851	111802852	MACS_peak_14755	3.00	lobChIP_FOXA1_robot
chr7	111834318	111834319	MACS_peak_14756	9.00	lobChIP_FOXA1_robot
chr7	111851658	111851659	MACS_peak_14757	4.00	lobChIP_FOXA1_robot
chr7	111876901	111876902	MACS_peak_14758	5.00	lobChIP_FOXA1_robot
chr7	111998066	111998067	MACS_peak_14759	6.00	lobChIP_FOXA1_robot
chr7	112538716	112538717	MACS_peak_14760	8.00	lobChIP_FOXA1_robot
chr7	113911869	113911870	MACS_peak_14761	5.00	lobChIP_FOXA1_robot
chr7	114054528	114054529	MACS_peak_14762	5.00	lobChIP_FOXA1_robot
chr7	115318307	115318308	MACS_peak_14763	5.00	lobChIP_FOXA1_robot
chr7	115809243	115809244	MACS_peak_14764	5.00	lobChIP_FOXA1_robot
chr7	115810404	115810405	MACS_peak_14765	10.00	lobChIP_FOXA1_robot
chr7	115813849	115813850	MACS_peak_14766	13.00	lobChIP_FOXA1_robot
chr7	115879778	115879779	MACS_peak_14767	7.00	lobChIP_FOXA1_robot
chr7	116310940	116310941	MACS_peak_14768	7.00	lobChIP_FOXA1_robot
chr7	116312758	116312759	MACS_peak_14769	4.00	lobChIP_FOXA1_robot
chr7	116327284	116327285	MACS_peak_14770	6.00	lobChIP_FOXA1_robot
chr7	116350926	116350927	MACS_peak_14771	3.00	lobChIP_FOXA1_robot
chr7	116351332	116351333	MACS_peak_14772	10.00	lobChIP_FOXA1_robot
chr7	116378650	116378651	MACS_peak_14773	7.00	lobChIP_FOXA1_robot
chr7	116382127	116382128	MACS_peak_14774	6.00	lobChIP_FOXA1_robot
chr7	116412784	116412785	MACS_peak_14775	8.00	lobChIP_FOXA1_robot
chr7	116441245	116441246	MACS_peak_14776	4.00	lobChIP_FOXA1_robot
chr7	116446144	116446145	MACS_peak_14777	9.00	lobChIP_FOXA1_robot
chr7	116449840	116449841	MACS_peak_14778	42.00	lobChIP_FOXA1_robot
chr7	116450991	116450992	MACS_peak_14779	41.00	lobChIP_FOXA1_robot
chr7	116592857	116592858	MACS_peak_14780	4.00	lobChIP_FOXA1_robot
chr7	116717843	116717844	MACS_peak_14781	3.00	lobChIP_FOXA1_robot
chr7	116764776	116764777	MACS_peak_14782	11.00	lobChIP_FOXA1_robot
chr7	117578262	117578263	MACS_peak_14783	5.00	lobChIP_FOXA1_robot
chr7	118865581	118865582	MACS_peak_14784	3.00	lobChIP_FOXA1_robot
chr7	119185346	119185347	MACS_peak_14785	8.00	lobChIP_FOXA1_robot
chr7	121784307	121784308	MACS_peak_14786	4.00	lobChIP_FOXA1_robot
chr7	121944821	121944822	MACS_peak_14787	5.00	lobChIP_FOXA1_robot
chr7	122172836	122172837	MACS_peak_14788	6.00	lobChIP_FOXA1_robot
chr7	122397123	122397124	MACS_peak_14789	5.00	lobChIP_FOXA1_robot
chr7	122498582	122498583	MACS_peak_14790	7.00	lobChIP_FOXA1_robot
chr7	122523992	122523993	MACS_peak_14791	3.00	lobChIP_FOXA1_robot
chr7	122528240	122528241	MACS_peak_14792	3.00	lobChIP_FOXA1_robot
chr7	122831551	122831552	MACS_peak_14793	10.00	lobChIP_FOXA1_robot
chr7	123321823	123321824	MACS_peak_14794	4.00	lobChIP_FOXA1_robot
chr7	123365048	123365049	MACS_peak_14795	3.00	lobChIP_FOXA1_robot
chr7	123366113	123366114	MACS_peak_14796	3.00	lobChIP_FOXA1_robot
chr7	123367443	123367444	MACS_peak_14797	4.00	lobChIP_FOXA1_robot
chr7	123378518	123378519	MACS_peak_14798	6.00	lobChIP_FOXA1_robot
chr7	123438264	123438265	MACS_peak_14799	7.00	lobChIP_FOXA1_robot
chr7	123464987	123464988	MACS_peak_14800	6.00	lobChIP_FOXA1_robot
chr7	124100742	124100743	MACS_peak_14801	4.00	lobChIP_FOXA1_robot
chr7	124130258	124130259	MACS_peak_14802	11.00	lobChIP_FOXA1_robot
chr7	124403069	124403070	MACS_peak_14803	4.00	lobChIP_FOXA1_robot
chr7	124416360	124416361	MACS_peak_14804	6.00	lobChIP_FOXA1_robot
chr7	124437116	124437117	MACS_peak_14805	6.00	lobChIP_FOXA1_robot
chr7	127547988	127547989	MACS_peak_14806	5.00	lobChIP_FOXA1_robot
chr7	127553172	127553173	MACS_peak_14807	4.00	lobChIP_FOXA1_robot
chr7	128075549	128075550	MACS_peak_14808	8.00	lobChIP_FOXA1_robot
chr7	128093682	128093683	MACS_peak_14809	4.00	lobChIP_FOXA1_robot
chr7	128417776	128417777	MACS_peak_14810	5.00	lobChIP_FOXA1_robot
chr7	128576260	128576261	MACS_peak_14811	3.00	lobChIP_FOXA1_robot
chr7	128841272	128841273	MACS_peak_14812	17.00	lobChIP_FOXA1_robot
chr7	129209883	129209884	MACS_peak_14813	5.00	lobChIP_FOXA1_robot
chr7	129753952	129753953	MACS_peak_14814	10.00	lobChIP_FOXA1_robot
chr7	129868042	129868043	MACS_peak_14815	5.00	lobChIP_FOXA1_robot
chr7	130009337	130009338	MACS_peak_14816	4.00	lobChIP_FOXA1_robot
chr7	130013572	130013573	MACS_peak_14817	3.00	lobChIP_FOXA1_robot
chr7	130572159	130572160	MACS_peak_14818	5.00	lobChIP_FOXA1_robot
chr7	130579249	130579250	MACS_peak_14819	6.00	lobChIP_FOXA1_robot
chr7	130584442	130584443	MACS_peak_14820	4.00	lobChIP_FOXA1_robot
chr7	130589230	130589231	MACS_peak_14821	4.00	lobChIP_FOXA1_robot
chr7	130600601	130600602	MACS_peak_14822	9.00	lobChIP_FOXA1_robot
chr7	130603129	130603130	MACS_peak_14823	4.00	lobChIP_FOXA1_robot
chr7	130607571	130607572	MACS_peak_14824	4.00	lobChIP_FOXA1_robot
chr7	130681867	130681868	MACS_peak_14825	6.00	lobChIP_FOXA1_robot
chr7	130742198	130742199	MACS_peak_14826	9.00	lobChIP_FOXA1_robot
chr7	130750301	130750302	MACS_peak_14827	3.00	lobChIP_FOXA1_robot
chr7	130751570	130751571	MACS_peak_14828	4.00	lobChIP_FOXA1_robot
chr7	130761766	130761767	MACS_peak_14829	8.00	lobChIP_FOXA1_robot
chr7	130774103	130774104	MACS_peak_14830	4.00	lobChIP_FOXA1_robot
chr7	130852779	130852780	MACS_peak_14831	4.00	lobChIP_FOXA1_robot
chr7	130911073	130911074	MACS_peak_14832	9.00	lobChIP_FOXA1_robot
chr7	130990074	130990075	MACS_peak_14833	8.00	lobChIP_FOXA1_robot
chr7	131016722	131016723	MACS_peak_14834	10.00	lobChIP_FOXA1_robot
chr7	131192842	131192843	MACS_peak_14835	3.00	lobChIP_FOXA1_robot
chr7	131903754	131903755	MACS_peak_14836	10.00	lobChIP_FOXA1_robot
chr7	131935825	131935826	MACS_peak_14837	4.00	lobChIP_FOXA1_robot
chr7	132740215	132740216	MACS_peak_14838	3.00	lobChIP_FOXA1_robot
chr7	133012923	133012924	MACS_peak_14839	14.00	lobChIP_FOXA1_robot
chr7	133500874	133500875	MACS_peak_14840	19.00	lobChIP_FOXA1_robot
chr7	133548701	133548702	MACS_peak_14841	7.00	lobChIP_FOXA1_robot
chr7	133556650	133556651	MACS_peak_14842	7.00	lobChIP_FOXA1_robot
chr7	133977960	133977961	MACS_peak_14843	4.00	lobChIP_FOXA1_robot
chr7	134576860	134576861	MACS_peak_14844	18.00	lobChIP_FOXA1_robot
chr7	134586633	134586634	MACS_peak_14845	6.00	lobChIP_FOXA1_robot
chr7	134651541	134651542	MACS_peak_14846	5.00	lobChIP_FOXA1_robot
chr7	134676685	134676686	MACS_peak_14847	42.00	lobChIP_FOXA1_robot
chr7	134682309	134682310	MACS_peak_14848	18.00	lobChIP_FOXA1_robot
chr7	134779334	134779335	MACS_peak_14849	4.00	lobChIP_FOXA1_robot
chr7	134780150	134780151	MACS_peak_14850	5.00	lobChIP_FOXA1_robot
chr7	134832803	134832804	MACS_peak_14851	9.00	lobChIP_FOXA1_robot
chr7	134833448	134833449	MACS_peak_14852	4.00	lobChIP_FOXA1_robot
chr7	134942687	134942688	MACS_peak_14853	8.00	lobChIP_FOXA1_robot
chr7	137380836	137380837	MACS_peak_14854	28.00	lobChIP_FOXA1_robot
chr7	137576120	137576121	MACS_peak_14855	6.00	lobChIP_FOXA1_robot
chr7	137616777	137616778	MACS_peak_14856	18.00	lobChIP_FOXA1_robot
chr7	137627700	137627701	MACS_peak_14857	3.00	lobChIP_FOXA1_robot
chr7	137633248	137633249	MACS_peak_14858	4.00	lobChIP_FOXA1_robot
chr7	137633796	137633797	MACS_peak_14859	9.00	lobChIP_FOXA1_robot
chr7	137656367	137656368	MACS_peak_14860	7.00	lobChIP_FOXA1_robot
chr7	137676837	137676838	MACS_peak_14861	8.00	lobChIP_FOXA1_robot
chr7	137729815	137729816	MACS_peak_14862	3.00	lobChIP_FOXA1_robot
chr7	137734514	137734515	MACS_peak_14863	10.00	lobChIP_FOXA1_robot
chr7	137748345	137748346	MACS_peak_14864	12.00	lobChIP_FOXA1_robot
chr7	137750053	137750054	MACS_peak_14865	6.00	lobChIP_FOXA1_robot
chr7	137750792	137750793	MACS_peak_14866	8.00	lobChIP_FOXA1_robot
chr7	137751862	137751863	MACS_peak_14867	13.00	lobChIP_FOXA1_robot
chr7	137774430	137774431	MACS_peak_14868	13.00	lobChIP_FOXA1_robot
chr7	137782325	137782326	MACS_peak_14869	6.00	lobChIP_FOXA1_robot
chr7	137805319	137805320	MACS_peak_14870	5.00	lobChIP_FOXA1_robot
chr7	137836911	137836912	MACS_peak_14871	9.00	lobChIP_FOXA1_robot
chr7	137843684	137843685	MACS_peak_14872	4.00	lobChIP_FOXA1_robot
chr7	137920871	137920872	MACS_peak_14873	4.00	lobChIP_FOXA1_robot
chr7	137922881	137922882	MACS_peak_14874	11.00	lobChIP_FOXA1_robot
chr7	137930698	137930699	MACS_peak_14875	15.00	lobChIP_FOXA1_robot
chr7	137942592	137942593	MACS_peak_14876	15.00	lobChIP_FOXA1_robot
chr7	137979391	137979392	MACS_peak_14877	5.00	lobChIP_FOXA1_robot
chr7	137982397	137982398	MACS_peak_14878	7.00	lobChIP_FOXA1_robot
chr7	138001532	138001533	MACS_peak_14879	9.00	lobChIP_FOXA1_robot
chr7	138013095	138013096	MACS_peak_14880	4.00	lobChIP_FOXA1_robot
chr7	138037502	138037503	MACS_peak_14881	5.00	lobChIP_FOXA1_robot
chr7	138073776	138073777	MACS_peak_14882	18.00	lobChIP_FOXA1_robot
chr7	138079455	138079456	MACS_peak_14883	6.00	lobChIP_FOXA1_robot
chr7	138081288	138081289	MACS_peak_14884	4.00	lobChIP_FOXA1_robot
chr7	138102762	138102763	MACS_peak_14885	21.00	lobChIP_FOXA1_robot
chr7	138162191	138162192	MACS_peak_14886	8.00	lobChIP_FOXA1_robot
chr7	138250642	138250643	MACS_peak_14887	3.00	lobChIP_FOXA1_robot
chr7	138298701	138298702	MACS_peak_14888	5.00	lobChIP_FOXA1_robot
chr7	138801862	138801863	MACS_peak_14889	8.00	lobChIP_FOXA1_robot
chr7	139345269	139345270	MACS_peak_14890	7.00	lobChIP_FOXA1_robot
chr7	139360103	139360104	MACS_peak_14891	12.00	lobChIP_FOXA1_robot
chr7	139364867	139364868	MACS_peak_14892	4.00	lobChIP_FOXA1_robot
chr7	139651066	139651067	MACS_peak_14893	5.00	lobChIP_FOXA1_robot
chr7	139746180	139746181	MACS_peak_14894	5.00	lobChIP_FOXA1_robot
chr7	139749159	139749160	MACS_peak_14895	38.00	lobChIP_FOXA1_robot
chr7	139929518	139929519	MACS_peak_14896	16.00	lobChIP_FOXA1_robot
chr7	139949057	139949058	MACS_peak_14897	14.00	lobChIP_FOXA1_robot
chr7	139952737	139952738	MACS_peak_14898	14.00	lobChIP_FOXA1_robot
chr7	140010344	140010345	MACS_peak_14899	9.00	lobChIP_FOXA1_robot
chr7	140529762	140529763	MACS_peak_14900	5.00	lobChIP_FOXA1_robot
chr7	140613121	140613122	MACS_peak_14901	7.00	lobChIP_FOXA1_robot
chr7	140672900	140672901	MACS_peak_14902	3.00	lobChIP_FOXA1_robot
chr7	140676031	140676032	MACS_peak_14903	24.00	lobChIP_FOXA1_robot
chr7	140742470	140742471	MACS_peak_14904	4.00	lobChIP_FOXA1_robot
chr7	141286257	141286258	MACS_peak_14905	5.00	lobChIP_FOXA1_robot
chr7	141395765	141395766	MACS_peak_14906	9.00	lobChIP_FOXA1_robot
chr7	141804758	141804759	MACS_peak_14907	11.00	lobChIP_FOXA1_robot
chr7	141832884	141832885	MACS_peak_14908	5.00	lobChIP_FOXA1_robot
chr7	142740070	142740071	MACS_peak_14909	5.00	lobChIP_FOXA1_robot
chr7	143092794	143092795	MACS_peak_14910	5.00	lobChIP_FOXA1_robot
chr7	143589908	143589909	MACS_peak_14911	3.00	lobChIP_FOXA1_robot
chr7	143676661	143676662	MACS_peak_14912	5.00	lobChIP_FOXA1_robot
chr7	144279506	144279507	MACS_peak_14913	3.00	lobChIP_FOXA1_robot
chr7	147529702	147529703	MACS_peak_14914	5.00	lobChIP_FOXA1_robot
chr7	148125788	148125789	MACS_peak_14915	40.00	lobChIP_FOXA1_robot
chr7	148144700	148144701	MACS_peak_14916	5.00	lobChIP_FOXA1_robot
chr7	148224109	148224110	MACS_peak_14917	5.00	lobChIP_FOXA1_robot
chr7	148680894	148680895	MACS_peak_14918	4.00	lobChIP_FOXA1_robot
chr7	149119251	149119252	MACS_peak_14919	6.00	lobChIP_FOXA1_robot
chr7	150075248	150075249	MACS_peak_14920	8.00	lobChIP_FOXA1_robot
chr7	150080141	150080142	MACS_peak_14921	6.00	lobChIP_FOXA1_robot
chr7	150112669	150112670	MACS_peak_14922	4.00	lobChIP_FOXA1_robot
chr7	150462900	150462901	MACS_peak_14923	5.00	lobChIP_FOXA1_robot
chr7	151191068	151191069	MACS_peak_14924	5.00	lobChIP_FOXA1_robot
chr7	151541704	151541705	MACS_peak_14925	5.00	lobChIP_FOXA1_robot
chr7	151778499	151778500	MACS_peak_14926	4.00	lobChIP_FOXA1_robot
chr7	151902517	151902518	MACS_peak_14927	6.00	lobChIP_FOXA1_robot
chr7	152107058	152107059	MACS_peak_14928	4.00	lobChIP_FOXA1_robot
chr7	152335258	152335259	MACS_peak_14929	3.00	lobChIP_FOXA1_robot
chr7	152607700	152607701	MACS_peak_14930	4.00	lobChIP_FOXA1_robot
chr7	153999747	153999748	MACS_peak_14931	6.00	lobChIP_FOXA1_robot
chr7	154080449	154080450	MACS_peak_14932	4.00	lobChIP_FOXA1_robot
chr7	155744505	155744506	MACS_peak_14933	5.00	lobChIP_FOXA1_robot
chr7	156172555	156172556	MACS_peak_14934	4.00	lobChIP_FOXA1_robot
chr7	156267004	156267005	MACS_peak_14935	5.00	lobChIP_FOXA1_robot
chr7	156309082	156309083	MACS_peak_14936	6.00	lobChIP_FOXA1_robot
chr7	156401847	156401848	MACS_peak_14937	13.00	lobChIP_FOXA1_robot
chr7	156402432	156402433	MACS_peak_14938	5.00	lobChIP_FOXA1_robot
chr7	156407710	156407711	MACS_peak_14939	41.00	lobChIP_FOXA1_robot
chr7	156459485	156459486	MACS_peak_14940	11.00	lobChIP_FOXA1_robot
chr7	156540572	156540573	MACS_peak_14941	25.00	lobChIP_FOXA1_robot
chr7	156610276	156610277	MACS_peak_14942	6.00	lobChIP_FOXA1_robot
chr7	156804472	156804473	MACS_peak_14943	6.00	lobChIP_FOXA1_robot
chr7	158225625	158225626	MACS_peak_14944	7.00	lobChIP_FOXA1_robot
chr7	158291661	158291662	MACS_peak_14945	7.00	lobChIP_FOXA1_robot
chr7	158373164	158373165	MACS_peak_14946	6.00	lobChIP_FOXA1_robot
chr7	158383550	158383551	MACS_peak_14947	10.00	lobChIP_FOXA1_robot
chr7	158419236	158419237	MACS_peak_14948	5.00	lobChIP_FOXA1_robot
chr7	158435030	158435031	MACS_peak_14949	4.00	lobChIP_FOXA1_robot
chr7	158439893	158439894	MACS_peak_14950	4.00	lobChIP_FOXA1_robot
chr7	158611805	158611806	MACS_peak_14951	6.00	lobChIP_FOXA1_robot
chr7	158756880	158756881	MACS_peak_14952	6.00	lobChIP_FOXA1_robot
chr8	335075	335076	MACS_peak_14953	7.00	lobChIP_FOXA1_robot
chr8	350905	350906	MACS_peak_14954	6.00	lobChIP_FOXA1_robot
chr8	438884	438885	MACS_peak_14955	9.00	lobChIP_FOXA1_robot
chr8	644476	644477	MACS_peak_14956	13.00	lobChIP_FOXA1_robot
chr8	980357	980358	MACS_peak_14957	7.00	lobChIP_FOXA1_robot
chr8	1693648	1693649	MACS_peak_14958	6.00	lobChIP_FOXA1_robot
chr8	3792846	3792847	MACS_peak_14959	5.00	lobChIP_FOXA1_robot
chr8	3834453	3834454	MACS_peak_14960	7.00	lobChIP_FOXA1_robot
chr8	3845332	3845333	MACS_peak_14961	15.00	lobChIP_FOXA1_robot
chr8	5997613	5997614	MACS_peak_14962	11.00	lobChIP_FOXA1_robot
chr8	6249272	6249273	MACS_peak_14963	8.00	lobChIP_FOXA1_robot
chr8	6321567	6321568	MACS_peak_14964	14.00	lobChIP_FOXA1_robot
chr8	6323271	6323272	MACS_peak_14965	4.00	lobChIP_FOXA1_robot
chr8	6417373	6417374	MACS_peak_14966	5.00	lobChIP_FOXA1_robot
chr8	6418111	6418112	MACS_peak_14967	6.00	lobChIP_FOXA1_robot
chr8	6594133	6594134	MACS_peak_14968	5.00	lobChIP_FOXA1_robot
chr8	6605877	6605878	MACS_peak_14969	9.00	lobChIP_FOXA1_robot
chr8	6710081	6710082	MACS_peak_14970	12.00	lobChIP_FOXA1_robot
chr8	6758594	6758595	MACS_peak_14971	7.00	lobChIP_FOXA1_robot
chr8	8093170	8093171	MACS_peak_14972	4.00	lobChIP_FOXA1_robot
chr8	8111678	8111679	MACS_peak_14973	5.00	lobChIP_FOXA1_robot
chr8	8121130	8121131	MACS_peak_14974	4.00	lobChIP_FOXA1_robot
chr8	8128065	8128066	MACS_peak_14975	15.00	lobChIP_FOXA1_robot
chr8	8146060	8146061	MACS_peak_14976	9.00	lobChIP_FOXA1_robot
chr8	8264996	8264997	MACS_peak_14977	4.00	lobChIP_FOXA1_robot
chr8	8285769	8285770	MACS_peak_14978	4.00	lobChIP_FOXA1_robot
chr8	8287058	8287059	MACS_peak_14979	56.00	lobChIP_FOXA1_robot
chr8	8324876	8324877	MACS_peak_14980	3.00	lobChIP_FOXA1_robot
chr8	8341132	8341133	MACS_peak_14981	6.00	lobChIP_FOXA1_robot
chr8	8514672	8514673	MACS_peak_14982	5.00	lobChIP_FOXA1_robot
chr8	8663868	8663869	MACS_peak_14983	26.00	lobChIP_FOXA1_robot
chr8	8729656	8729657	MACS_peak_14984	11.00	lobChIP_FOXA1_robot
chr8	8732829	8732830	MACS_peak_14985	4.00	lobChIP_FOXA1_robot
chr8	8775398	8775399	MACS_peak_14986	4.00	lobChIP_FOXA1_robot
chr8	8835590	8835591	MACS_peak_14987	6.00	lobChIP_FOXA1_robot
chr8	8852668	8852669	MACS_peak_14988	3.00	lobChIP_FOXA1_robot
chr8	8953662	8953663	MACS_peak_14989	37.00	lobChIP_FOXA1_robot
chr8	8960320	8960321	MACS_peak_14990	77.00	lobChIP_FOXA1_robot
chr8	9018352	9018353	MACS_peak_14991	7.00	lobChIP_FOXA1_robot
chr8	9212378	9212379	MACS_peak_14992	6.00	lobChIP_FOXA1_robot
chr8	9279152	9279153	MACS_peak_14993	10.00	lobChIP_FOXA1_robot
chr8	9410091	9410092	MACS_peak_14994	4.00	lobChIP_FOXA1_robot
chr8	9637861	9637862	MACS_peak_14995	4.00	lobChIP_FOXA1_robot
chr8	9639062	9639063	MACS_peak_14996	20.00	lobChIP_FOXA1_robot
chr8	9640108	9640109	MACS_peak_14997	20.00	lobChIP_FOXA1_robot
chr8	9651422	9651423	MACS_peak_14998	52.00	lobChIP_FOXA1_robot
chr8	9675988	9675989	MACS_peak_14999	10.00	lobChIP_FOXA1_robot
chr8	10950152	10950153	MACS_peak_15000	5.00	lobChIP_FOXA1_robot
chr8	11326623	11326624	MACS_peak_15001	5.00	lobChIP_FOXA1_robot
chr8	11486452	11486453	MACS_peak_15002	9.00	lobChIP_FOXA1_robot
chr8	11576343	11576344	MACS_peak_15003	6.00	lobChIP_FOXA1_robot
chr8	11666543	11666544	MACS_peak_15004	8.00	lobChIP_FOXA1_robot
chr8	11734495	11734496	MACS_peak_15005	5.00	lobChIP_FOXA1_robot
chr8	11749630	11749631	MACS_peak_15006	4.00	lobChIP_FOXA1_robot
chr8	11759434	11759435	MACS_peak_15007	18.00	lobChIP_FOXA1_robot
chr8	12584319	12584320	MACS_peak_15008	6.00	lobChIP_FOXA1_robot
chr8	12954136	12954137	MACS_peak_15009	23.00	lobChIP_FOXA1_robot
chr8	12973881	12973882	MACS_peak_15010	3.00	lobChIP_FOXA1_robot
chr8	12996971	12996972	MACS_peak_15011	16.00	lobChIP_FOXA1_robot
chr8	12998239	12998240	MACS_peak_15012	19.00	lobChIP_FOXA1_robot
chr8	13067954	13067955	MACS_peak_15013	15.00	lobChIP_FOXA1_robot
chr8	13076724	13076725	MACS_peak_15014	3.00	lobChIP_FOXA1_robot
chr8	13091565	13091566	MACS_peak_15015	6.00	lobChIP_FOXA1_robot
chr8	13092744	13092745	MACS_peak_15016	7.00	lobChIP_FOXA1_robot
chr8	13123446	13123447	MACS_peak_15017	26.00	lobChIP_FOXA1_robot
chr8	13128941	13128942	MACS_peak_15018	4.00	lobChIP_FOXA1_robot
chr8	13132917	13132918	MACS_peak_15019	39.00	lobChIP_FOXA1_robot
chr8	13187993	13187994	MACS_peak_15020	10.00	lobChIP_FOXA1_robot
chr8	13191515	13191516	MACS_peak_15021	5.00	lobChIP_FOXA1_robot
chr8	13211566	13211567	MACS_peak_15022	16.00	lobChIP_FOXA1_robot
chr8	13215005	13215006	MACS_peak_15023	3.00	lobChIP_FOXA1_robot
chr8	13306877	13306878	MACS_peak_15024	10.00	lobChIP_FOXA1_robot
chr8	13402500	13402501	MACS_peak_15025	7.00	lobChIP_FOXA1_robot
chr8	15086924	15086925	MACS_peak_15026	3.00	lobChIP_FOXA1_robot
chr8	15315341	15315342	MACS_peak_15027	5.00	lobChIP_FOXA1_robot
chr8	16909311	16909312	MACS_peak_15028	12.00	lobChIP_FOXA1_robot
chr8	16911133	16911134	MACS_peak_15029	4.00	lobChIP_FOXA1_robot
chr8	17013239	17013240	MACS_peak_15030	7.00	lobChIP_FOXA1_robot
chr8	17117151	17117152	MACS_peak_15031	3.00	lobChIP_FOXA1_robot
chr8	17179205	17179206	MACS_peak_15032	51.00	lobChIP_FOXA1_robot
chr8	17186836	17186837	MACS_peak_15033	7.00	lobChIP_FOXA1_robot
chr8	17208826	17208827	MACS_peak_15034	6.00	lobChIP_FOXA1_robot
chr8	17251065	17251066	MACS_peak_15035	4.00	lobChIP_FOXA1_robot
chr8	17353942	17353943	MACS_peak_15036	7.00	lobChIP_FOXA1_robot
chr8	17355695	17355696	MACS_peak_15037	3.00	lobChIP_FOXA1_robot
chr8	17435622	17435623	MACS_peak_15038	5.00	lobChIP_FOXA1_robot
chr8	17459566	17459567	MACS_peak_15039	4.00	lobChIP_FOXA1_robot
chr8	17472377	17472378	MACS_peak_15040	17.00	lobChIP_FOXA1_robot
chr8	17644579	17644580	MACS_peak_15041	6.00	lobChIP_FOXA1_robot
chr8	17646403	17646404	MACS_peak_15042	12.00	lobChIP_FOXA1_robot
chr8	17647183	17647184	MACS_peak_15043	4.00	lobChIP_FOXA1_robot
chr8	17652622	17652623	MACS_peak_15044	21.00	lobChIP_FOXA1_robot
chr8	17653933	17653934	MACS_peak_15045	5.00	lobChIP_FOXA1_robot
chr8	17660638	17660639	MACS_peak_15046	8.00	lobChIP_FOXA1_robot
chr8	17661509	17661510	MACS_peak_15047	6.00	lobChIP_FOXA1_robot
chr8	17663475	17663476	MACS_peak_15048	21.00	lobChIP_FOXA1_robot
chr8	17678841	17678842	MACS_peak_15049	4.00	lobChIP_FOXA1_robot
chr8	17693829	17693830	MACS_peak_15050	15.00	lobChIP_FOXA1_robot
chr8	17694430	17694431	MACS_peak_15051	4.00	lobChIP_FOXA1_robot
chr8	17701726	17701727	MACS_peak_15052	4.00	lobChIP_FOXA1_robot
chr8	17749987	17749988	MACS_peak_15053	7.00	lobChIP_FOXA1_robot
chr8	17753759	17753760	MACS_peak_15054	27.00	lobChIP_FOXA1_robot
chr8	17764907	17764908	MACS_peak_15055	27.00	lobChIP_FOXA1_robot
chr8	17773529	17773530	MACS_peak_15056	8.00	lobChIP_FOXA1_robot
chr8	17774013	17774014	MACS_peak_15057	4.00	lobChIP_FOXA1_robot
chr8	18192108	18192109	MACS_peak_15058	3.00	lobChIP_FOXA1_robot
chr8	18524298	18524299	MACS_peak_15059	9.00	lobChIP_FOXA1_robot
chr8	18827128	18827129	MACS_peak_15060	4.00	lobChIP_FOXA1_robot
chr8	19128932	19128933	MACS_peak_15061	8.00	lobChIP_FOXA1_robot
chr8	19176542	19176543	MACS_peak_15062	17.00	lobChIP_FOXA1_robot
chr8	19202786	19202787	MACS_peak_15063	4.00	lobChIP_FOXA1_robot
chr8	19296146	19296147	MACS_peak_15064	9.00	lobChIP_FOXA1_robot
chr8	19315989	19315990	MACS_peak_15065	4.00	lobChIP_FOXA1_robot
chr8	19523032	19523033	MACS_peak_15066	6.00	lobChIP_FOXA1_robot
chr8	19745461	19745462	MACS_peak_15067	6.00	lobChIP_FOXA1_robot
chr8	20646710	20646711	MACS_peak_15068	3.00	lobChIP_FOXA1_robot
chr8	21888442	21888443	MACS_peak_15069	5.00	lobChIP_FOXA1_robot
chr8	21969068	21969069	MACS_peak_15070	13.00	lobChIP_FOXA1_robot
chr8	22001318	22001319	MACS_peak_15071	7.00	lobChIP_FOXA1_robot
chr8	22077727	22077728	MACS_peak_15072	4.00	lobChIP_FOXA1_robot
chr8	22101172	22101173	MACS_peak_15073	4.00	lobChIP_FOXA1_robot
chr8	22131826	22131827	MACS_peak_15074	7.00	lobChIP_FOXA1_robot
chr8	22214071	22214072	MACS_peak_15075	4.00	lobChIP_FOXA1_robot
chr8	22223191	22223192	MACS_peak_15076	5.00	lobChIP_FOXA1_robot
chr8	22287563	22287564	MACS_peak_15077	12.00	lobChIP_FOXA1_robot
chr8	22310652	22310653	MACS_peak_15078	4.00	lobChIP_FOXA1_robot
chr8	22407190	22407191	MACS_peak_15079	4.00	lobChIP_FOXA1_robot
chr8	22414078	22414079	MACS_peak_15080	16.00	lobChIP_FOXA1_robot
chr8	22773192	22773193	MACS_peak_15081	6.00	lobChIP_FOXA1_robot
chr8	22898063	22898064	MACS_peak_15082	4.00	lobChIP_FOXA1_robot
chr8	22940887	22940888	MACS_peak_15083	4.00	lobChIP_FOXA1_robot
chr8	22963658	22963659	MACS_peak_15084	4.00	lobChIP_FOXA1_robot
chr8	23283987	23283988	MACS_peak_15085	4.00	lobChIP_FOXA1_robot
chr8	23285125	23285126	MACS_peak_15086	3.00	lobChIP_FOXA1_robot
chr8	24206828	24206829	MACS_peak_15087	5.00	lobChIP_FOXA1_robot
chr8	25043849	25043850	MACS_peak_15088	5.00	lobChIP_FOXA1_robot
chr8	25056670	25056671	MACS_peak_15089	6.00	lobChIP_FOXA1_robot
chr8	25057485	25057486	MACS_peak_15090	4.00	lobChIP_FOXA1_robot
chr8	25160306	25160307	MACS_peak_15091	5.00	lobChIP_FOXA1_robot
chr8	25177491	25177492	MACS_peak_15092	14.00	lobChIP_FOXA1_robot
chr8	25250359	25250360	MACS_peak_15093	7.00	lobChIP_FOXA1_robot
chr8	25314724	25314725	MACS_peak_15094	4.00	lobChIP_FOXA1_robot
chr8	25339369	25339370	MACS_peak_15095	8.00	lobChIP_FOXA1_robot
chr8	26059737	26059738	MACS_peak_15096	3.00	lobChIP_FOXA1_robot
chr8	26152614	26152615	MACS_peak_15097	5.00	lobChIP_FOXA1_robot
chr8	26187576	26187577	MACS_peak_15098	9.00	lobChIP_FOXA1_robot
chr8	26498892	26498893	MACS_peak_15099	4.00	lobChIP_FOXA1_robot
chr8	26499403	26499404	MACS_peak_15100	5.00	lobChIP_FOXA1_robot
chr8	27180815	27180816	MACS_peak_15101	12.00	lobChIP_FOXA1_robot
chr8	27316885	27316886	MACS_peak_15102	72.00	lobChIP_FOXA1_robot
chr8	27431861	27431862	MACS_peak_15103	5.00	lobChIP_FOXA1_robot
chr8	27452636	27452637	MACS_peak_15104	8.00	lobChIP_FOXA1_robot
chr8	27460998	27460999	MACS_peak_15105	35.00	lobChIP_FOXA1_robot
chr8	27541110	27541111	MACS_peak_15106	11.00	lobChIP_FOXA1_robot
chr8	27608149	27608150	MACS_peak_15107	9.00	lobChIP_FOXA1_robot
chr8	27730272	27730273	MACS_peak_15108	8.00	lobChIP_FOXA1_robot
chr8	27927625	27927626	MACS_peak_15109	5.00	lobChIP_FOXA1_robot
chr8	27930045	27930046	MACS_peak_15110	21.00	lobChIP_FOXA1_robot
chr8	27941424	27941425	MACS_peak_15111	4.00	lobChIP_FOXA1_robot
chr8	27985359	27985360	MACS_peak_15112	10.00	lobChIP_FOXA1_robot
chr8	28022955	28022956	MACS_peak_15113	10.00	lobChIP_FOXA1_robot
chr8	28055041	28055042	MACS_peak_15114	7.00	lobChIP_FOXA1_robot
chr8	28350681	28350682	MACS_peak_15115	7.00	lobChIP_FOXA1_robot
chr8	28551773	28551774	MACS_peak_15116	4.00	lobChIP_FOXA1_robot
chr8	28570863	28570864	MACS_peak_15117	9.00	lobChIP_FOXA1_robot
chr8	29087612	29087613	MACS_peak_15118	4.00	lobChIP_FOXA1_robot
chr8	29140616	29140617	MACS_peak_15119	5.00	lobChIP_FOXA1_robot
chr8	29147286	29147287	MACS_peak_15120	4.00	lobChIP_FOXA1_robot
chr8	29184934	29184935	MACS_peak_15121	8.00	lobChIP_FOXA1_robot
chr8	29355921	29355922	MACS_peak_15122	5.00	lobChIP_FOXA1_robot
chr8	29524031	29524032	MACS_peak_15123	4.00	lobChIP_FOXA1_robot
chr8	29631060	29631061	MACS_peak_15124	5.00	lobChIP_FOXA1_robot
chr8	29672156	29672157	MACS_peak_15125	4.00	lobChIP_FOXA1_robot
chr8	29802351	29802352	MACS_peak_15126	24.00	lobChIP_FOXA1_robot
chr8	29813937	29813938	MACS_peak_15127	11.00	lobChIP_FOXA1_robot
chr8	29815937	29815938	MACS_peak_15128	3.00	lobChIP_FOXA1_robot
chr8	29832846	29832847	MACS_peak_15129	10.00	lobChIP_FOXA1_robot
chr8	29856299	29856300	MACS_peak_15130	4.00	lobChIP_FOXA1_robot
chr8	30140707	30140708	MACS_peak_15131	4.00	lobChIP_FOXA1_robot
chr8	30172130	30172131	MACS_peak_15132	5.00	lobChIP_FOXA1_robot
chr8	30173312	30173313	MACS_peak_15133	12.00	lobChIP_FOXA1_robot
chr8	30182331	30182332	MACS_peak_15134	14.00	lobChIP_FOXA1_robot
chr8	30183725	30183726	MACS_peak_15135	3.00	lobChIP_FOXA1_robot
chr8	30271604	30271605	MACS_peak_15136	6.00	lobChIP_FOXA1_robot
chr8	30343207	30343208	MACS_peak_15137	6.00	lobChIP_FOXA1_robot
chr8	30358743	30358744	MACS_peak_15138	4.00	lobChIP_FOXA1_robot
chr8	30361786	30361787	MACS_peak_15139	21.00	lobChIP_FOXA1_robot
chr8	30378735	30378736	MACS_peak_15140	32.00	lobChIP_FOXA1_robot
chr8	30389156	30389157	MACS_peak_15141	25.00	lobChIP_FOXA1_robot
chr8	30440249	30440250	MACS_peak_15142	6.00	lobChIP_FOXA1_robot
chr8	30474182	30474183	MACS_peak_15143	4.00	lobChIP_FOXA1_robot
chr8	30547546	30547547	MACS_peak_15144	6.00	lobChIP_FOXA1_robot
chr8	30755597	30755598	MACS_peak_15145	4.00	lobChIP_FOXA1_robot
chr8	30947435	30947436	MACS_peak_15146	4.00	lobChIP_FOXA1_robot
chr8	30971119	30971120	MACS_peak_15147	7.00	lobChIP_FOXA1_robot
chr8	32949411	32949412	MACS_peak_15148	10.00	lobChIP_FOXA1_robot
chr8	33371198	33371199	MACS_peak_15149	4.00	lobChIP_FOXA1_robot
chr8	33994608	33994609	MACS_peak_15150	3.00	lobChIP_FOXA1_robot
chr8	34441678	34441679	MACS_peak_15151	5.00	lobChIP_FOXA1_robot
chr8	37100671	37100672	MACS_peak_15152	10.00	lobChIP_FOXA1_robot
chr8	37265969	37265970	MACS_peak_15153	6.00	lobChIP_FOXA1_robot
chr8	37410493	37410494	MACS_peak_15154	13.00	lobChIP_FOXA1_robot
chr8	37449109	37449110	MACS_peak_15155	14.00	lobChIP_FOXA1_robot
chr8	37456290	37456291	MACS_peak_15156	4.00	lobChIP_FOXA1_robot
chr8	37483001	37483002	MACS_peak_15157	9.00	lobChIP_FOXA1_robot
chr8	37532996	37532997	MACS_peak_15158	17.00	lobChIP_FOXA1_robot
chr8	37548921	37548922	MACS_peak_15159	9.00	lobChIP_FOXA1_robot
chr8	37857578	37857579	MACS_peak_15160	7.00	lobChIP_FOXA1_robot
chr8	37925235	37925236	MACS_peak_15161	8.00	lobChIP_FOXA1_robot
chr8	38036739	38036740	MACS_peak_15162	3.00	lobChIP_FOXA1_robot
chr8	38042145	38042146	MACS_peak_15163	6.00	lobChIP_FOXA1_robot
chr8	38411106	38411107	MACS_peak_15164	11.00	lobChIP_FOXA1_robot
chr8	38415315	38415316	MACS_peak_15165	9.00	lobChIP_FOXA1_robot
chr8	38624432	38624433	MACS_peak_15166	4.00	lobChIP_FOXA1_robot
chr8	38859092	38859093	MACS_peak_15167	3.00	lobChIP_FOXA1_robot
chr8	38859747	38859748	MACS_peak_15168	6.00	lobChIP_FOXA1_robot
chr8	39066577	39066578	MACS_peak_15169	16.00	lobChIP_FOXA1_robot
chr8	39899206	39899207	MACS_peak_15170	3.00	lobChIP_FOXA1_robot
chr8	39912409	39912410	MACS_peak_15171	15.00	lobChIP_FOXA1_robot
chr8	39917175	39917176	MACS_peak_15172	5.00	lobChIP_FOXA1_robot
chr8	39921129	39921130	MACS_peak_15173	4.00	lobChIP_FOXA1_robot
chr8	39962035	39962036	MACS_peak_15174	14.00	lobChIP_FOXA1_robot
chr8	39976775	39976776	MACS_peak_15175	3.00	lobChIP_FOXA1_robot
chr8	40016646	40016647	MACS_peak_15176	11.00	lobChIP_FOXA1_robot
chr8	40020102	40020103	MACS_peak_15177	14.00	lobChIP_FOXA1_robot
chr8	40028299	40028300	MACS_peak_15178	11.00	lobChIP_FOXA1_robot
chr8	40032538	40032539	MACS_peak_15179	12.00	lobChIP_FOXA1_robot
chr8	41137426	41137427	MACS_peak_15180	7.00	lobChIP_FOXA1_robot
chr8	41462004	41462005	MACS_peak_15181	5.00	lobChIP_FOXA1_robot
chr8	41651316	41651317	MACS_peak_15182	4.00	lobChIP_FOXA1_robot
chr8	41675390	41675391	MACS_peak_15183	5.00	lobChIP_FOXA1_robot
chr8	41787200	41787201	MACS_peak_15184	7.00	lobChIP_FOXA1_robot
chr8	41841468	41841469	MACS_peak_15185	4.00	lobChIP_FOXA1_robot
chr8	41867823	41867824	MACS_peak_15186	10.00	lobChIP_FOXA1_robot
chr8	41871282	41871283	MACS_peak_15187	4.00	lobChIP_FOXA1_robot
chr8	41900694	41900695	MACS_peak_15188	3.00	lobChIP_FOXA1_robot
chr8	42224435	42224436	MACS_peak_15189	25.00	lobChIP_FOXA1_robot
chr8	42381348	42381349	MACS_peak_15190	3.00	lobChIP_FOXA1_robot
chr8	42394748	42394749	MACS_peak_15191	4.00	lobChIP_FOXA1_robot
chr8	42398338	42398339	MACS_peak_15192	7.00	lobChIP_FOXA1_robot
chr8	42426890	42426891	MACS_peak_15193	5.00	lobChIP_FOXA1_robot
chr8	42514027	42514028	MACS_peak_15194	9.00	lobChIP_FOXA1_robot
chr8	42575758	42575759	MACS_peak_15195	5.00	lobChIP_FOXA1_robot
chr8	42656702	42656703	MACS_peak_15196	7.00	lobChIP_FOXA1_robot
chr8	42764998	42764999	MACS_peak_15197	45.00	lobChIP_FOXA1_robot
chr8	42906140	42906141	MACS_peak_15198	29.00	lobChIP_FOXA1_robot
chr8	48290246	48290247	MACS_peak_15205	11.00	lobChIP_FOXA1_robot
chr8	48437621	48437622	MACS_peak_15206	4.00	lobChIP_FOXA1_robot
chr8	48556568	48556569	MACS_peak_15207	6.00	lobChIP_FOXA1_robot
chr8	48572059	48572060	MACS_peak_15208	5.00	lobChIP_FOXA1_robot
chr8	48647851	48647852	MACS_peak_15209	3.00	lobChIP_FOXA1_robot
chr8	48670902	48670903	MACS_peak_15210	7.00	lobChIP_FOXA1_robot
chr8	48905686	48905687	MACS_peak_15211	12.00	lobChIP_FOXA1_robot
chr8	48955010	48955011	MACS_peak_15212	10.00	lobChIP_FOXA1_robot
chr8	49062943	49062944	MACS_peak_15213	7.00	lobChIP_FOXA1_robot
chr8	52513358	52513359	MACS_peak_15214	3.00	lobChIP_FOXA1_robot
chr8	52811665	52811666	MACS_peak_15215	7.00	lobChIP_FOXA1_robot
chr8	52886102	52886103	MACS_peak_15216	6.00	lobChIP_FOXA1_robot
chr8	53302967	53302968	MACS_peak_15217	6.00	lobChIP_FOXA1_robot
chr8	53303507	53303508	MACS_peak_15218	6.00	lobChIP_FOXA1_robot
chr8	53379752	53379753	MACS_peak_15219	4.00	lobChIP_FOXA1_robot
chr8	53542115	53542116	MACS_peak_15220	5.00	lobChIP_FOXA1_robot
chr8	53607984	53607985	MACS_peak_15221	11.00	lobChIP_FOXA1_robot
chr8	53615509	53615510	MACS_peak_15222	6.00	lobChIP_FOXA1_robot
chr8	53653606	53653607	MACS_peak_15223	4.00	lobChIP_FOXA1_robot
chr8	54267328	54267329	MACS_peak_15224	3.00	lobChIP_FOXA1_robot
chr8	54645206	54645207	MACS_peak_15225	5.00	lobChIP_FOXA1_robot
chr8	54857788	54857789	MACS_peak_15226	12.00	lobChIP_FOXA1_robot
chr8	56585326	56585327	MACS_peak_15227	3.00	lobChIP_FOXA1_robot
chr8	56901707	56901708	MACS_peak_15228	5.00	lobChIP_FOXA1_robot
chr8	58493144	58493145	MACS_peak_15229	12.00	lobChIP_FOXA1_robot
chr8	58501087	58501088	MACS_peak_15230	13.00	lobChIP_FOXA1_robot
chr8	58716937	58716938	MACS_peak_15231	15.00	lobChIP_FOXA1_robot
chr8	58721386	58721387	MACS_peak_15232	9.00	lobChIP_FOXA1_robot
chr8	58781831	58781832	MACS_peak_15233	10.00	lobChIP_FOXA1_robot
chr8	58980407	58980408	MACS_peak_15234	30.00	lobChIP_FOXA1_robot
chr8	58991650	58991651	MACS_peak_15235	6.00	lobChIP_FOXA1_robot
chr8	59008640	59008641	MACS_peak_15236	11.00	lobChIP_FOXA1_robot
chr8	59032782	59032783	MACS_peak_15237	4.00	lobChIP_FOXA1_robot
chr8	59044337	59044338	MACS_peak_15238	7.00	lobChIP_FOXA1_robot
chr8	59049577	59049578	MACS_peak_15239	10.00	lobChIP_FOXA1_robot
chr8	59231701	59231702	MACS_peak_15240	4.00	lobChIP_FOXA1_robot
chr8	59383940	59383941	MACS_peak_15241	11.00	lobChIP_FOXA1_robot
chr8	59390726	59390727	MACS_peak_15242	4.00	lobChIP_FOXA1_robot
chr8	59412965	59412966	MACS_peak_15243	6.00	lobChIP_FOXA1_robot
chr8	59425975	59425976	MACS_peak_15244	19.00	lobChIP_FOXA1_robot
chr8	59812678	59812679	MACS_peak_15245	3.00	lobChIP_FOXA1_robot
chr8	60929599	60929600	MACS_peak_15246	4.00	lobChIP_FOXA1_robot
chr8	61194717	61194718	MACS_peak_15247	8.00	lobChIP_FOXA1_robot
chr8	61596739	61596740	MACS_peak_15248	8.00	lobChIP_FOXA1_robot
chr8	61631593	61631594	MACS_peak_15249	3.00	lobChIP_FOXA1_robot
chr8	61862552	61862553	MACS_peak_15250	5.00	lobChIP_FOXA1_robot
chr8	62224445	62224446	MACS_peak_15251	6.00	lobChIP_FOXA1_robot
chr8	62623758	62623759	MACS_peak_15252	7.00	lobChIP_FOXA1_robot
chr8	62625593	62625594	MACS_peak_15253	4.00	lobChIP_FOXA1_robot
chr8	62679977	62679978	MACS_peak_15254	40.00	lobChIP_FOXA1_robot
chr8	62695490	62695491	MACS_peak_15255	13.00	lobChIP_FOXA1_robot
chr8	63580936	63580937	MACS_peak_15256	4.00	lobChIP_FOXA1_robot
chr8	63941722	63941723	MACS_peak_15257	4.00	lobChIP_FOXA1_robot
chr8	63952581	63952582	MACS_peak_15258	10.00	lobChIP_FOXA1_robot
chr8	64013860	64013861	MACS_peak_15259	13.00	lobChIP_FOXA1_robot
chr8	65112097	65112098	MACS_peak_15260	3.00	lobChIP_FOXA1_robot
chr8	65352195	65352196	MACS_peak_15261	7.00	lobChIP_FOXA1_robot
chr8	66095649	66095650	MACS_peak_15262	3.00	lobChIP_FOXA1_robot
chr8	66629776	66629777	MACS_peak_15263	8.00	lobChIP_FOXA1_robot
chr8	66637523	66637524	MACS_peak_15264	7.00	lobChIP_FOXA1_robot
chr8	66841658	66841659	MACS_peak_15265	6.00	lobChIP_FOXA1_robot
chr8	67576066	67576067	MACS_peak_15266	3.00	lobChIP_FOXA1_robot
chr8	67609110	67609111	MACS_peak_15267	9.00	lobChIP_FOXA1_robot
chr8	67793380	67793381	MACS_peak_15268	4.00	lobChIP_FOXA1_robot
chr8	67981124	67981125	MACS_peak_15269	9.00	lobChIP_FOXA1_robot
chr8	68022235	68022236	MACS_peak_15270	15.00	lobChIP_FOXA1_robot
chr8	68315673	68315674	MACS_peak_15271	4.00	lobChIP_FOXA1_robot
chr8	68604997	68604998	MACS_peak_15272	4.00	lobChIP_FOXA1_robot
chr8	68612676	68612677	MACS_peak_15273	10.00	lobChIP_FOXA1_robot
chr8	68649932	68649933	MACS_peak_15274	17.00	lobChIP_FOXA1_robot
chr8	69560822	69560823	MACS_peak_15275	5.00	lobChIP_FOXA1_robot
chr8	70204521	70204522	MACS_peak_15276	26.00	lobChIP_FOXA1_robot
chr8	71064848	71064849	MACS_peak_15278	25.00	lobChIP_FOXA1_robot
chr8	71245030	71245031	MACS_peak_15279	4.00	lobChIP_FOXA1_robot
chr8	71524582	71524583	MACS_peak_15280	14.00	lobChIP_FOXA1_robot
chr8	71580423	71580424	MACS_peak_15281	5.00	lobChIP_FOXA1_robot
chr8	71592726	71592727	MACS_peak_15282	4.00	lobChIP_FOXA1_robot
chr8	71683011	71683012	MACS_peak_15283	6.00	lobChIP_FOXA1_robot
chr8	71684502	71684503	MACS_peak_15284	6.00	lobChIP_FOXA1_robot
chr8	73284282	73284283	MACS_peak_15285	4.00	lobChIP_FOXA1_robot
chr8	73607379	73607380	MACS_peak_15286	3.00	lobChIP_FOXA1_robot
chr8	74091854	74091855	MACS_peak_15287	5.00	lobChIP_FOXA1_robot
chr8	74097210	74097211	MACS_peak_15288	8.00	lobChIP_FOXA1_robot
chr8	74115529	74115530	MACS_peak_15289	3.00	lobChIP_FOXA1_robot
chr8	74270823	74270824	MACS_peak_15290	15.00	lobChIP_FOXA1_robot
chr8	74457118	74457119	MACS_peak_15291	7.00	lobChIP_FOXA1_robot
chr8	74751004	74751005	MACS_peak_15292	6.00	lobChIP_FOXA1_robot
chr8	75306973	75306974	MACS_peak_15293	5.00	lobChIP_FOXA1_robot
chr8	75825960	75825961	MACS_peak_15294	7.00	lobChIP_FOXA1_robot
chr8	76005170	76005171	MACS_peak_15295	4.00	lobChIP_FOXA1_robot
chr8	76396971	76396972	MACS_peak_15296	4.00	lobChIP_FOXA1_robot
chr8	78312249	78312250	MACS_peak_15297	3.00	lobChIP_FOXA1_robot
chr8	80875871	80875872	MACS_peak_15298	12.00	lobChIP_FOXA1_robot
chr8	80938691	80938692	MACS_peak_15299	10.00	lobChIP_FOXA1_robot
chr8	80996658	80996659	MACS_peak_15300	3.00	lobChIP_FOXA1_robot
chr8	81059811	81059812	MACS_peak_15301	4.00	lobChIP_FOXA1_robot
chr8	81309496	81309497	MACS_peak_15302	5.00	lobChIP_FOXA1_robot
chr8	81416236	81416237	MACS_peak_15303	3.00	lobChIP_FOXA1_robot
chr8	81479131	81479132	MACS_peak_15304	4.00	lobChIP_FOXA1_robot
chr8	81590083	81590084	MACS_peak_15305	19.00	lobChIP_FOXA1_robot
chr8	81733189	81733190	MACS_peak_15306	7.00	lobChIP_FOXA1_robot
chr8	81796672	81796673	MACS_peak_15307	4.00	lobChIP_FOXA1_robot
chr8	81877739	81877740	MACS_peak_15308	9.00	lobChIP_FOXA1_robot
chr8	81942140	81942141	MACS_peak_15309	10.00	lobChIP_FOXA1_robot
chr8	82106469	82106470	MACS_peak_15310	5.00	lobChIP_FOXA1_robot
chr8	82505440	82505441	MACS_peak_15311	3.00	lobChIP_FOXA1_robot
chr8	82518230	82518231	MACS_peak_15312	5.00	lobChIP_FOXA1_robot
chr8	82912152	82912153	MACS_peak_15313	11.00	lobChIP_FOXA1_robot
chr8	83119130	83119131	MACS_peak_15314	6.00	lobChIP_FOXA1_robot
chr8	86180389	86180390	MACS_peak_15315	7.00	lobChIP_FOXA1_robot
chr8	86259172	86259173	MACS_peak_15316	48.00	lobChIP_FOXA1_robot
chr8	86464224	86464225	MACS_peak_15317	7.00	lobChIP_FOXA1_robot
chr8	87140638	87140639	MACS_peak_15318	7.00	lobChIP_FOXA1_robot
chr8	87223099	87223100	MACS_peak_15319	8.00	lobChIP_FOXA1_robot
chr8	87251572	87251573	MACS_peak_15320	6.00	lobChIP_FOXA1_robot
chr8	87268074	87268075	MACS_peak_15321	12.00	lobChIP_FOXA1_robot
chr8	87302438	87302439	MACS_peak_15322	12.00	lobChIP_FOXA1_robot
chr8	87320776	87320777	MACS_peak_15323	5.00	lobChIP_FOXA1_robot
chr8	87333097	87333098	MACS_peak_15324	5.00	lobChIP_FOXA1_robot
chr8	87369140	87369141	MACS_peak_15325	3.00	lobChIP_FOXA1_robot
chr8	90096642	90096643	MACS_peak_15326	5.00	lobChIP_FOXA1_robot
chr8	90811636	90811637	MACS_peak_15327	5.00	lobChIP_FOXA1_robot
chr8	90995770	90995771	MACS_peak_15328	6.00	lobChIP_FOXA1_robot
chr8	91176787	91176788	MACS_peak_15329	4.00	lobChIP_FOXA1_robot
chr8	91655694	91655695	MACS_peak_15330	5.00	lobChIP_FOXA1_robot
chr8	92120192	92120193	MACS_peak_15331	24.00	lobChIP_FOXA1_robot
chr8	93934778	93934779	MACS_peak_15332	4.00	lobChIP_FOXA1_robot
chr8	93977142	93977143	MACS_peak_15333	9.00	lobChIP_FOXA1_robot
chr8	94045746	94045747	MACS_peak_15334	4.00	lobChIP_FOXA1_robot
chr8	94667908	94667909	MACS_peak_15335	9.00	lobChIP_FOXA1_robot
chr8	94678645	94678646	MACS_peak_15336	49.00	lobChIP_FOXA1_robot
chr8	94685192	94685193	MACS_peak_15337	4.00	lobChIP_FOXA1_robot
chr8	94970136	94970137	MACS_peak_15338	6.00	lobChIP_FOXA1_robot
chr8	95417776	95417777	MACS_peak_15339	3.00	lobChIP_FOXA1_robot
chr8	95907986	95907987	MACS_peak_15340	5.00	lobChIP_FOXA1_robot
chr8	95972211	95972212	MACS_peak_15341	31.00	lobChIP_FOXA1_robot
chr8	95973048	95973049	MACS_peak_15342	8.00	lobChIP_FOXA1_robot
chr8	96003541	96003542	MACS_peak_15343	5.00	lobChIP_FOXA1_robot
chr8	96087276	96087277	MACS_peak_15344	22.00	lobChIP_FOXA1_robot
chr8	96113232	96113233	MACS_peak_15345	22.00	lobChIP_FOXA1_robot
chr8	96125009	96125010	MACS_peak_15346	9.00	lobChIP_FOXA1_robot
chr8	96212854	96212855	MACS_peak_15347	4.00	lobChIP_FOXA1_robot
chr8	96222542	96222543	MACS_peak_15348	5.00	lobChIP_FOXA1_robot
chr8	96223997	96223998	MACS_peak_15349	6.00	lobChIP_FOXA1_robot
chr8	97536320	97536321	MACS_peak_15350	3.00	lobChIP_FOXA1_robot
chr8	97536939	97536940	MACS_peak_15351	4.00	lobChIP_FOXA1_robot
chr8	97567805	97567806	MACS_peak_15352	6.00	lobChIP_FOXA1_robot
chr8	97603624	97603625	MACS_peak_15353	5.00	lobChIP_FOXA1_robot
chr8	97781882	97781883	MACS_peak_15354	4.00	lobChIP_FOXA1_robot
chr8	97940833	97940834	MACS_peak_15355	7.00	lobChIP_FOXA1_robot
chr8	98074273	98074274	MACS_peak_15356	9.00	lobChIP_FOXA1_robot
chr8	98160744	98160745	MACS_peak_15357	9.00	lobChIP_FOXA1_robot
chr8	98175840	98175841	MACS_peak_15358	35.00	lobChIP_FOXA1_robot
chr8	98183911	98183912	MACS_peak_15359	7.00	lobChIP_FOXA1_robot
chr8	98219737	98219738	MACS_peak_15360	6.00	lobChIP_FOXA1_robot
chr8	98220342	98220343	MACS_peak_15361	4.00	lobChIP_FOXA1_robot
chr8	98279874	98279875	MACS_peak_15362	41.00	lobChIP_FOXA1_robot
chr8	98375884	98375885	MACS_peak_15363	6.00	lobChIP_FOXA1_robot
chr8	98795637	98795638	MACS_peak_15364	5.00	lobChIP_FOXA1_robot
chr8	99081036	99081037	MACS_peak_15365	13.00	lobChIP_FOXA1_robot
chr8	99176070	99176071	MACS_peak_15366	4.00	lobChIP_FOXA1_robot
chr8	99296600	99296601	MACS_peak_15367	4.00	lobChIP_FOXA1_robot
chr8	99304252	99304253	MACS_peak_15368	7.00	lobChIP_FOXA1_robot
chr8	99310204	99310205	MACS_peak_15369	27.00	lobChIP_FOXA1_robot
chr8	99610583	99610584	MACS_peak_15370	12.00	lobChIP_FOXA1_robot
chr8	99887996	99887997	MACS_peak_15371	4.00	lobChIP_FOXA1_robot
chr8	99889420	99889421	MACS_peak_15372	6.00	lobChIP_FOXA1_robot
chr8	99979423	99979424	MACS_peak_15373	4.00	lobChIP_FOXA1_robot
chr8	100046602	100046603	MACS_peak_15374	5.00	lobChIP_FOXA1_robot
chr8	100202931	100202932	MACS_peak_15375	5.00	lobChIP_FOXA1_robot
chr8	100511104	100511105	MACS_peak_15376	5.00	lobChIP_FOXA1_robot
chr8	100703401	100703402	MACS_peak_15377	3.00	lobChIP_FOXA1_robot
chr8	101347105	101347106	MACS_peak_15378	8.00	lobChIP_FOXA1_robot
chr8	101450909	101450910	MACS_peak_15379	5.00	lobChIP_FOXA1_robot
chr8	101500324	101500325	MACS_peak_15380	19.00	lobChIP_FOXA1_robot
chr8	101541434	101541435	MACS_peak_15381	8.00	lobChIP_FOXA1_robot
chr8	101675973	101675974	MACS_peak_15382	9.00	lobChIP_FOXA1_robot
chr8	102171574	102171575	MACS_peak_15383	7.00	lobChIP_FOXA1_robot
chr8	102373223	102373224	MACS_peak_15384	5.00	lobChIP_FOXA1_robot
chr8	102620306	102620307	MACS_peak_15385	7.00	lobChIP_FOXA1_robot
chr8	103343539	103343540	MACS_peak_15386	17.00	lobChIP_FOXA1_robot
chr8	103412690	103412691	MACS_peak_15387	4.00	lobChIP_FOXA1_robot
chr8	103428964	103428965	MACS_peak_15388	10.00	lobChIP_FOXA1_robot
chr8	103504581	103504582	MACS_peak_15389	11.00	lobChIP_FOXA1_robot
chr8	103788833	103788834	MACS_peak_15390	4.00	lobChIP_FOXA1_robot
chr8	105597995	105597996	MACS_peak_15391	8.00	lobChIP_FOXA1_robot
chr8	106255070	106255071	MACS_peak_15392	4.00	lobChIP_FOXA1_robot
chr8	106909523	106909524	MACS_peak_15393	5.00	lobChIP_FOXA1_robot
chr8	107636924	107636925	MACS_peak_15394	13.00	lobChIP_FOXA1_robot
chr8	107642885	107642886	MACS_peak_15395	10.00	lobChIP_FOXA1_robot
chr8	108129084	108129085	MACS_peak_15396	17.00	lobChIP_FOXA1_robot
chr8	109467708	109467709	MACS_peak_15397	5.00	lobChIP_FOXA1_robot
chr8	110241641	110241642	MACS_peak_15398	18.00	lobChIP_FOXA1_robot
chr8	110289388	110289389	MACS_peak_15399	4.00	lobChIP_FOXA1_robot
chr8	110349136	110349137	MACS_peak_15400	5.00	lobChIP_FOXA1_robot
chr8	110487285	110487286	MACS_peak_15401	5.00	lobChIP_FOXA1_robot
chr8	110592589	110592590	MACS_peak_15402	5.00	lobChIP_FOXA1_robot
chr8	110605684	110605685	MACS_peak_15403	14.00	lobChIP_FOXA1_robot
chr8	110620166	110620167	MACS_peak_15404	12.00	lobChIP_FOXA1_robot
chr8	110652753	110652754	MACS_peak_15405	21.00	lobChIP_FOXA1_robot
chr8	111342273	111342274	MACS_peak_15406	3.00	lobChIP_FOXA1_robot
chr8	112576719	112576720	MACS_peak_15407	11.00	lobChIP_FOXA1_robot
chr8	113039101	113039102	MACS_peak_15408	29.00	lobChIP_FOXA1_robot
chr8	117737975	117737976	MACS_peak_15409	10.00	lobChIP_FOXA1_robot
chr8	117877557	117877558	MACS_peak_15410	9.00	lobChIP_FOXA1_robot
chr8	117902307	117902308	MACS_peak_15411	5.00	lobChIP_FOXA1_robot
chr8	118273588	118273589	MACS_peak_15412	4.00	lobChIP_FOXA1_robot
chr8	118662784	118662785	MACS_peak_15413	5.00	lobChIP_FOXA1_robot
chr8	118675683	118675684	MACS_peak_15414	9.00	lobChIP_FOXA1_robot
chr8	118681887	118681888	MACS_peak_15415	11.00	lobChIP_FOXA1_robot
chr8	118684406	118684407	MACS_peak_15416	12.00	lobChIP_FOXA1_robot
chr8	118898860	118898861	MACS_peak_15417	5.00	lobChIP_FOXA1_robot
chr8	118960006	118960007	MACS_peak_15418	7.00	lobChIP_FOXA1_robot
chr8	118993039	118993040	MACS_peak_15419	5.00	lobChIP_FOXA1_robot
chr8	119001875	119001876	MACS_peak_15420	8.00	lobChIP_FOXA1_robot
chr8	119004469	119004470	MACS_peak_15421	5.00	lobChIP_FOXA1_robot
chr8	119042144	119042145	MACS_peak_15422	25.00	lobChIP_FOXA1_robot
chr8	119356404	119356405	MACS_peak_15423	10.00	lobChIP_FOXA1_robot
chr8	120221808	120221809	MACS_peak_15424	7.00	lobChIP_FOXA1_robot
chr8	120238685	120238686	MACS_peak_15425	12.00	lobChIP_FOXA1_robot
chr8	120244542	120244543	MACS_peak_15426	5.00	lobChIP_FOXA1_robot
chr8	120648103	120648104	MACS_peak_15427	6.00	lobChIP_FOXA1_robot
chr8	120666602	120666603	MACS_peak_15428	3.00	lobChIP_FOXA1_robot
chr8	120667545	120667546	MACS_peak_15429	37.00	lobChIP_FOXA1_robot
chr8	120780059	120780060	MACS_peak_15430	6.00	lobChIP_FOXA1_robot
chr8	120883577	120883578	MACS_peak_15431	4.00	lobChIP_FOXA1_robot
chr8	121493615	121493616	MACS_peak_15432	9.00	lobChIP_FOXA1_robot
chr8	121723745	121723746	MACS_peak_15433	20.00	lobChIP_FOXA1_robot
chr8	121758810	121758811	MACS_peak_15434	5.00	lobChIP_FOXA1_robot
chr8	121760397	121760398	MACS_peak_15435	13.00	lobChIP_FOXA1_robot
chr8	121772502	121772503	MACS_peak_15436	7.00	lobChIP_FOXA1_robot
chr8	121774984	121774985	MACS_peak_15437	5.00	lobChIP_FOXA1_robot
chr8	121787636	121787637	MACS_peak_15438	15.00	lobChIP_FOXA1_robot
chr8	121789427	121789428	MACS_peak_15439	31.00	lobChIP_FOXA1_robot
chr8	121790441	121790442	MACS_peak_15440	5.00	lobChIP_FOXA1_robot
chr8	121795285	121795286	MACS_peak_15441	4.00	lobChIP_FOXA1_robot
chr8	121798128	121798129	MACS_peak_15442	5.00	lobChIP_FOXA1_robot
chr8	121822035	121822036	MACS_peak_15443	43.00	lobChIP_FOXA1_robot
chr8	121840581	121840582	MACS_peak_15444	6.00	lobChIP_FOXA1_robot
chr8	121843326	121843327	MACS_peak_15445	3.00	lobChIP_FOXA1_robot
chr8	121851693	121851694	MACS_peak_15446	18.00	lobChIP_FOXA1_robot
chr8	121855597	121855598	MACS_peak_15447	5.00	lobChIP_FOXA1_robot
chr8	121858585	121858586	MACS_peak_15448	9.00	lobChIP_FOXA1_robot
chr8	121864133	121864134	MACS_peak_15449	10.00	lobChIP_FOXA1_robot
chr8	121931865	121931866	MACS_peak_15450	7.00	lobChIP_FOXA1_robot
chr8	122117865	122117866	MACS_peak_15451	8.00	lobChIP_FOXA1_robot
chr8	122166815	122166816	MACS_peak_15452	5.00	lobChIP_FOXA1_robot
chr8	122361196	122361197	MACS_peak_15453	21.00	lobChIP_FOXA1_robot
chr8	122368691	122368692	MACS_peak_15454	9.00	lobChIP_FOXA1_robot
chr8	122439150	122439151	MACS_peak_15455	9.00	lobChIP_FOXA1_robot
chr8	123282285	123282286	MACS_peak_15456	7.00	lobChIP_FOXA1_robot
chr8	123690837	123690838	MACS_peak_15457	5.00	lobChIP_FOXA1_robot
chr8	123740949	123740950	MACS_peak_15458	22.00	lobChIP_FOXA1_robot
chr8	123759356	123759357	MACS_peak_15459	8.00	lobChIP_FOXA1_robot
chr8	123772638	123772639	MACS_peak_15460	5.00	lobChIP_FOXA1_robot
chr8	124185436	124185437	MACS_peak_15461	10.00	lobChIP_FOXA1_robot
chr8	124409000	124409001	MACS_peak_15462	4.00	lobChIP_FOXA1_robot
chr8	124416146	124416147	MACS_peak_15463	20.00	lobChIP_FOXA1_robot
chr8	124417936	124417937	MACS_peak_15464	23.00	lobChIP_FOXA1_robot
chr8	124425307	124425308	MACS_peak_15465	6.00	lobChIP_FOXA1_robot
chr8	124426211	124426212	MACS_peak_15466	3.00	lobChIP_FOXA1_robot
chr8	124732394	124732395	MACS_peak_15467	7.00	lobChIP_FOXA1_robot
chr8	124970045	124970046	MACS_peak_15468	4.00	lobChIP_FOXA1_robot
chr8	125575993	125575994	MACS_peak_15469	5.00	lobChIP_FOXA1_robot
chr8	125630541	125630542	MACS_peak_15470	4.00	lobChIP_FOXA1_robot
chr8	125643572	125643573	MACS_peak_15471	6.00	lobChIP_FOXA1_robot
chr8	125672710	125672711	MACS_peak_15472	3.00	lobChIP_FOXA1_robot
chr8	125681706	125681707	MACS_peak_15473	8.00	lobChIP_FOXA1_robot
chr8	125682515	125682516	MACS_peak_15474	6.00	lobChIP_FOXA1_robot
chr8	125702313	125702314	MACS_peak_15475	5.00	lobChIP_FOXA1_robot
chr8	125704810	125704811	MACS_peak_15476	5.00	lobChIP_FOXA1_robot
chr8	125732766	125732767	MACS_peak_15477	14.00	lobChIP_FOXA1_robot
chr8	125755636	125755637	MACS_peak_15478	4.00	lobChIP_FOXA1_robot
chr8	126123257	126123258	MACS_peak_15479	6.00	lobChIP_FOXA1_robot
chr8	126138079	126138080	MACS_peak_15480	5.00	lobChIP_FOXA1_robot
chr8	126204270	126204271	MACS_peak_15481	5.00	lobChIP_FOXA1_robot
chr8	126245098	126245099	MACS_peak_15482	4.00	lobChIP_FOXA1_robot
chr8	126285629	126285630	MACS_peak_15483	6.00	lobChIP_FOXA1_robot
chr8	126325185	126325186	MACS_peak_15484	4.00	lobChIP_FOXA1_robot
chr8	126423007	126423008	MACS_peak_15485	12.00	lobChIP_FOXA1_robot
chr8	126429109	126429110	MACS_peak_15486	3.00	lobChIP_FOXA1_robot
chr8	126442496	126442497	MACS_peak_15487	20.00	lobChIP_FOXA1_robot
chr8	126514079	126514080	MACS_peak_15488	21.00	lobChIP_FOXA1_robot
chr8	126558596	126558597	MACS_peak_15489	5.00	lobChIP_FOXA1_robot
chr8	126698692	126698693	MACS_peak_15490	7.00	lobChIP_FOXA1_robot
chr8	127987176	127987177	MACS_peak_15491	4.00	lobChIP_FOXA1_robot
chr8	128089109	128089110	MACS_peak_15492	4.00	lobChIP_FOXA1_robot
chr8	128227668	128227669	MACS_peak_15493	68.00	lobChIP_FOXA1_robot
chr8	128231336	128231337	MACS_peak_15494	21.00	lobChIP_FOXA1_robot
chr8	128235167	128235168	MACS_peak_15495	6.00	lobChIP_FOXA1_robot
chr8	128413419	128413420	MACS_peak_15496	10.00	lobChIP_FOXA1_robot
chr8	128481844	128481845	MACS_peak_15497	5.00	lobChIP_FOXA1_robot
chr8	128845491	128845492	MACS_peak_15498	4.00	lobChIP_FOXA1_robot
chr8	128864389	128864390	MACS_peak_15499	4.00	lobChIP_FOXA1_robot
chr8	129129992	129129993	MACS_peak_15500	21.00	lobChIP_FOXA1_robot
chr8	129165479	129165480	MACS_peak_15501	6.00	lobChIP_FOXA1_robot
chr8	129169690	129169691	MACS_peak_15502	12.00	lobChIP_FOXA1_robot
chr8	129179793	129179794	MACS_peak_15503	29.00	lobChIP_FOXA1_robot
chr8	129181004	129181005	MACS_peak_15504	3.00	lobChIP_FOXA1_robot
chr8	129189286	129189287	MACS_peak_15505	32.00	lobChIP_FOXA1_robot
chr8	129197513	129197514	MACS_peak_15506	42.00	lobChIP_FOXA1_robot
chr8	129209367	129209368	MACS_peak_15507	33.00	lobChIP_FOXA1_robot
chr8	129209998	129209999	MACS_peak_15508	8.00	lobChIP_FOXA1_robot
chr8	129237758	129237759	MACS_peak_15509	7.00	lobChIP_FOXA1_robot
chr8	129353872	129353873	MACS_peak_15510	3.00	lobChIP_FOXA1_robot
chr8	129370130	129370131	MACS_peak_15511	9.00	lobChIP_FOXA1_robot
chr8	129381883	129381884	MACS_peak_15512	3.00	lobChIP_FOXA1_robot
chr8	129474771	129474772	MACS_peak_15513	3.00	lobChIP_FOXA1_robot
chr8	129488110	129488111	MACS_peak_15514	16.00	lobChIP_FOXA1_robot
chr8	129527081	129527082	MACS_peak_15515	6.00	lobChIP_FOXA1_robot
chr8	129567404	129567405	MACS_peak_15516	40.00	lobChIP_FOXA1_robot
chr8	129568491	129568492	MACS_peak_15517	3.00	lobChIP_FOXA1_robot
chr8	129568895	129568896	MACS_peak_15518	49.00	lobChIP_FOXA1_robot
chr8	129597546	129597547	MACS_peak_15519	70.00	lobChIP_FOXA1_robot
chr8	129615083	129615084	MACS_peak_15520	35.00	lobChIP_FOXA1_robot
chr8	131009142	131009143	MACS_peak_15521	10.00	lobChIP_FOXA1_robot
chr8	131269577	131269578	MACS_peak_15522	9.00	lobChIP_FOXA1_robot
chr8	131277089	131277090	MACS_peak_15523	7.00	lobChIP_FOXA1_robot
chr8	131291845	131291846	MACS_peak_15524	4.00	lobChIP_FOXA1_robot
chr8	131439192	131439193	MACS_peak_15525	10.00	lobChIP_FOXA1_robot
chr8	132531836	132531837	MACS_peak_15526	12.00	lobChIP_FOXA1_robot
chr8	132594903	132594904	MACS_peak_15527	4.00	lobChIP_FOXA1_robot
chr8	132928889	132928890	MACS_peak_15528	9.00	lobChIP_FOXA1_robot
chr8	134124562	134124563	MACS_peak_15529	7.00	lobChIP_FOXA1_robot
chr8	134229077	134229078	MACS_peak_15530	8.00	lobChIP_FOXA1_robot
chr8	134353869	134353870	MACS_peak_15531	5.00	lobChIP_FOXA1_robot
chr8	134362704	134362705	MACS_peak_15532	4.00	lobChIP_FOXA1_robot
chr8	134372668	134372669	MACS_peak_15533	5.00	lobChIP_FOXA1_robot
chr8	134388989	134388990	MACS_peak_15534	6.00	lobChIP_FOXA1_robot
chr8	134441199	134441200	MACS_peak_15535	6.00	lobChIP_FOXA1_robot
chr8	134502075	134502076	MACS_peak_15536	4.00	lobChIP_FOXA1_robot
chr8	134564218	134564219	MACS_peak_15537	5.00	lobChIP_FOXA1_robot
chr8	134565316	134565317	MACS_peak_15538	3.00	lobChIP_FOXA1_robot
chr8	134575336	134575337	MACS_peak_15539	9.00	lobChIP_FOXA1_robot
chr8	134576696	134576697	MACS_peak_15540	4.00	lobChIP_FOXA1_robot
chr8	134807267	134807268	MACS_peak_15541	4.00	lobChIP_FOXA1_robot
chr8	135626623	135626624	MACS_peak_15542	6.00	lobChIP_FOXA1_robot
chr8	135664899	135664900	MACS_peak_15543	4.00	lobChIP_FOXA1_robot
chr8	135726588	135726589	MACS_peak_15544	4.00	lobChIP_FOXA1_robot
chr8	135766477	135766478	MACS_peak_15545	10.00	lobChIP_FOXA1_robot
chr8	135778253	135778254	MACS_peak_15546	4.00	lobChIP_FOXA1_robot
chr8	135807369	135807370	MACS_peak_15547	5.00	lobChIP_FOXA1_robot
chr8	136527943	136527944	MACS_peak_15548	14.00	lobChIP_FOXA1_robot
chr8	136550593	136550594	MACS_peak_15549	4.00	lobChIP_FOXA1_robot
chr8	136602010	136602011	MACS_peak_15550	9.00	lobChIP_FOXA1_robot
chr8	137042079	137042080	MACS_peak_15551	30.00	lobChIP_FOXA1_robot
chr8	137053286	137053287	MACS_peak_15552	5.00	lobChIP_FOXA1_robot
chr8	137501631	137501632	MACS_peak_15553	9.00	lobChIP_FOXA1_robot
chr8	139163460	139163461	MACS_peak_15554	4.00	lobChIP_FOXA1_robot
chr8	139272416	139272417	MACS_peak_15555	25.00	lobChIP_FOXA1_robot
chr8	139580201	139580202	MACS_peak_15556	5.00	lobChIP_FOXA1_robot
chr8	139852658	139852659	MACS_peak_15557	3.00	lobChIP_FOXA1_robot
chr8	140209143	140209144	MACS_peak_15558	7.00	lobChIP_FOXA1_robot
chr8	140353768	140353769	MACS_peak_15559	6.00	lobChIP_FOXA1_robot
chr8	140388664	140388665	MACS_peak_15560	4.00	lobChIP_FOXA1_robot
chr8	140399345	140399346	MACS_peak_15561	10.00	lobChIP_FOXA1_robot
chr8	140854791	140854792	MACS_peak_15562	4.00	lobChIP_FOXA1_robot
chr8	140867408	140867409	MACS_peak_15563	5.00	lobChIP_FOXA1_robot
chr8	141059146	141059147	MACS_peak_15564	6.00	lobChIP_FOXA1_robot
chr8	141063897	141063898	MACS_peak_15565	48.00	lobChIP_FOXA1_robot
chr8	141075828	141075829	MACS_peak_15566	14.00	lobChIP_FOXA1_robot
chr8	141101868	141101869	MACS_peak_15567	5.00	lobChIP_FOXA1_robot
chr8	141164146	141164147	MACS_peak_15568	5.00	lobChIP_FOXA1_robot
chr8	141404103	141404104	MACS_peak_15569	4.00	lobChIP_FOXA1_robot
chr8	141474382	141474383	MACS_peak_15570	5.00	lobChIP_FOXA1_robot
chr8	141636432	141636433	MACS_peak_15571	25.00	lobChIP_FOXA1_robot
chr8	141637416	141637417	MACS_peak_15572	17.00	lobChIP_FOXA1_robot
chr8	141654794	141654795	MACS_peak_15573	5.00	lobChIP_FOXA1_robot
chr8	141932356	141932357	MACS_peak_15574	3.00	lobChIP_FOXA1_robot
chr8	141957084	141957085	MACS_peak_15575	4.00	lobChIP_FOXA1_robot
chr8	141979353	141979354	MACS_peak_15576	4.00	lobChIP_FOXA1_robot
chr8	141981238	141981239	MACS_peak_15577	4.00	lobChIP_FOXA1_robot
chr8	141983400	141983401	MACS_peak_15578	27.00	lobChIP_FOXA1_robot
chr8	142153004	142153005	MACS_peak_15579	7.00	lobChIP_FOXA1_robot
chr8	142156888	142156889	MACS_peak_15580	4.00	lobChIP_FOXA1_robot
chr8	142164366	142164367	MACS_peak_15581	4.00	lobChIP_FOXA1_robot
chr8	142238731	142238732	MACS_peak_15582	58.00	lobChIP_FOXA1_robot
chr8	142409401	142409402	MACS_peak_15583	8.00	lobChIP_FOXA1_robot
chr8	143844402	143844403	MACS_peak_15584	12.00	lobChIP_FOXA1_robot
chr8	146252305	146252306	MACS_peak_15585	6.00	lobChIP_FOXA1_robot
chr9	336156	336157	MACS_peak_15586	5.00	lobChIP_FOXA1_robot
chr9	489404	489405	MACS_peak_15587	8.00	lobChIP_FOXA1_robot
chr9	494970	494971	MACS_peak_15588	10.00	lobChIP_FOXA1_robot
chr9	673867	673868	MACS_peak_15589	19.00	lobChIP_FOXA1_robot
chr9	677219	677220	MACS_peak_15590	32.00	lobChIP_FOXA1_robot
chr9	682869	682870	MACS_peak_15591	13.00	lobChIP_FOXA1_robot
chr9	708981	708982	MACS_peak_15592	8.00	lobChIP_FOXA1_robot
chr9	734798	734799	MACS_peak_15593	5.00	lobChIP_FOXA1_robot
chr9	792829	792830	MACS_peak_15594	5.00	lobChIP_FOXA1_robot
chr9	1232577	1232578	MACS_peak_15595	4.00	lobChIP_FOXA1_robot
chr9	1460364	1460365	MACS_peak_15596	7.00	lobChIP_FOXA1_robot
chr9	1517398	1517399	MACS_peak_15597	3.00	lobChIP_FOXA1_robot
chr9	2720501	2720502	MACS_peak_15598	4.00	lobChIP_FOXA1_robot
chr9	2802723	2802724	MACS_peak_15599	4.00	lobChIP_FOXA1_robot
chr9	3039795	3039796	MACS_peak_15600	4.00	lobChIP_FOXA1_robot
chr9	3177543	3177544	MACS_peak_15601	5.00	lobChIP_FOXA1_robot
chr9	3343974	3343975	MACS_peak_15602	4.00	lobChIP_FOXA1_robot
chr9	3920733	3920734	MACS_peak_15603	9.00	lobChIP_FOXA1_robot
chr9	3962605	3962606	MACS_peak_15604	6.00	lobChIP_FOXA1_robot
chr9	4657896	4657897	MACS_peak_15605	19.00	lobChIP_FOXA1_robot
chr9	4678311	4678312	MACS_peak_15606	20.00	lobChIP_FOXA1_robot
chr9	4700193	4700194	MACS_peak_15607	5.00	lobChIP_FOXA1_robot
chr9	4833243	4833244	MACS_peak_15608	4.00	lobChIP_FOXA1_robot
chr9	4837957	4837958	MACS_peak_15609	3.00	lobChIP_FOXA1_robot
chr9	4839663	4839664	MACS_peak_15610	21.00	lobChIP_FOXA1_robot
chr9	5721802	5721803	MACS_peak_15611	35.00	lobChIP_FOXA1_robot
chr9	5828341	5828342	MACS_peak_15612	5.00	lobChIP_FOXA1_robot
chr9	5843905	5843906	MACS_peak_15613	6.00	lobChIP_FOXA1_robot
chr9	5894075	5894076	MACS_peak_15614	9.00	lobChIP_FOXA1_robot
chr9	6272350	6272351	MACS_peak_15615	5.00	lobChIP_FOXA1_robot
chr9	6591018	6591019	MACS_peak_15616	38.00	lobChIP_FOXA1_robot
chr9	6628933	6628934	MACS_peak_15617	7.00	lobChIP_FOXA1_robot
chr9	6665092	6665093	MACS_peak_15618	5.00	lobChIP_FOXA1_robot
chr9	6673863	6673864	MACS_peak_15619	5.00	lobChIP_FOXA1_robot
chr9	6781667	6781668	MACS_peak_15620	8.00	lobChIP_FOXA1_robot
chr9	6788382	6788383	MACS_peak_15621	5.00	lobChIP_FOXA1_robot
chr9	6788940	6788941	MACS_peak_15622	7.00	lobChIP_FOXA1_robot
chr9	6877929	6877930	MACS_peak_15623	11.00	lobChIP_FOXA1_robot
chr9	6898026	6898027	MACS_peak_15624	11.00	lobChIP_FOXA1_robot
chr9	6899584	6899585	MACS_peak_15625	6.00	lobChIP_FOXA1_robot
chr9	6974027	6974028	MACS_peak_15626	4.00	lobChIP_FOXA1_robot
chr9	8270449	8270450	MACS_peak_15627	5.00	lobChIP_FOXA1_robot
chr9	9054867	9054868	MACS_peak_15628	5.00	lobChIP_FOXA1_robot
chr9	12794369	12794370	MACS_peak_15629	8.00	lobChIP_FOXA1_robot
chr9	12814080	12814081	MACS_peak_15630	8.00	lobChIP_FOXA1_robot
chr9	12817092	12817093	MACS_peak_15631	8.00	lobChIP_FOXA1_robot
chr9	12911832	12911833	MACS_peak_15632	3.00	lobChIP_FOXA1_robot
chr9	13203331	13203332	MACS_peak_15633	8.00	lobChIP_FOXA1_robot
chr9	13740380	13740381	MACS_peak_15634	3.00	lobChIP_FOXA1_robot
chr9	14098255	14098256	MACS_peak_15635	4.00	lobChIP_FOXA1_robot
chr9	14390267	14390268	MACS_peak_15636	3.00	lobChIP_FOXA1_robot
chr9	14913586	14913587	MACS_peak_15637	10.00	lobChIP_FOXA1_robot
chr9	14968192	14968193	MACS_peak_15638	5.00	lobChIP_FOXA1_robot
chr9	15100315	15100316	MACS_peak_15639	6.00	lobChIP_FOXA1_robot
chr9	15350610	15350611	MACS_peak_15640	4.00	lobChIP_FOXA1_robot
chr9	15424149	15424150	MACS_peak_15641	5.00	lobChIP_FOXA1_robot
chr9	15480282	15480283	MACS_peak_15642	8.00	lobChIP_FOXA1_robot
chr9	16413706	16413707	MACS_peak_15643	12.00	lobChIP_FOXA1_robot
chr9	18284801	18284802	MACS_peak_15644	5.00	lobChIP_FOXA1_robot
chr9	19050685	19050686	MACS_peak_15645	7.00	lobChIP_FOXA1_robot
chr9	19129787	19129788	MACS_peak_15646	5.00	lobChIP_FOXA1_robot
chr9	19156139	19156140	MACS_peak_15647	4.00	lobChIP_FOXA1_robot
chr9	19161852	19161853	MACS_peak_15648	10.00	lobChIP_FOXA1_robot
chr9	19173734	19173735	MACS_peak_15649	5.00	lobChIP_FOXA1_robot
chr9	19202407	19202408	MACS_peak_15650	27.00	lobChIP_FOXA1_robot
chr9	19652962	19652963	MACS_peak_15651	4.00	lobChIP_FOXA1_robot
chr9	20382937	20382938	MACS_peak_15652	6.00	lobChIP_FOXA1_robot
chr9	20391324	20391325	MACS_peak_15653	4.00	lobChIP_FOXA1_robot
chr9	20477262	20477263	MACS_peak_15654	5.00	lobChIP_FOXA1_robot
chr9	20612187	20612188	MACS_peak_15655	14.00	lobChIP_FOXA1_robot
chr9	20690001	20690002	MACS_peak_15656	4.00	lobChIP_FOXA1_robot
chr9	20692731	20692732	MACS_peak_15657	5.00	lobChIP_FOXA1_robot
chr9	20781298	20781299	MACS_peak_15658	4.00	lobChIP_FOXA1_robot
chr9	21336582	21336583	MACS_peak_15659	4.00	lobChIP_FOXA1_robot
chr9	21461541	21461542	MACS_peak_15660	5.00	lobChIP_FOXA1_robot
chr9	21814178	21814179	MACS_peak_15661	3.00	lobChIP_FOXA1_robot
chr9	21853602	21853603	MACS_peak_15662	8.00	lobChIP_FOXA1_robot
chr9	22159128	22159129	MACS_peak_15663	10.00	lobChIP_FOXA1_robot
chr9	25775508	25775509	MACS_peak_15664	4.00	lobChIP_FOXA1_robot
chr9	27065576	27065577	MACS_peak_15665	6.00	lobChIP_FOXA1_robot
chr9	27261809	27261810	MACS_peak_15666	4.00	lobChIP_FOXA1_robot
chr9	27379971	27379972	MACS_peak_15667	5.00	lobChIP_FOXA1_robot
chr9	27436584	27436585	MACS_peak_15668	8.00	lobChIP_FOXA1_robot
chr9	27569603	27569604	MACS_peak_15669	11.00	lobChIP_FOXA1_robot
chr9	28213202	28213203	MACS_peak_15670	12.00	lobChIP_FOXA1_robot
chr9	30706739	30706740	MACS_peak_15671	4.00	lobChIP_FOXA1_robot
chr9	30896636	30896637	MACS_peak_15672	11.00	lobChIP_FOXA1_robot
chr9	31825057	31825058	MACS_peak_15673	15.00	lobChIP_FOXA1_robot
chr9	32004910	32004911	MACS_peak_15674	4.00	lobChIP_FOXA1_robot
chr9	32137050	32137051	MACS_peak_15675	14.00	lobChIP_FOXA1_robot
chr9	32248510	32248511	MACS_peak_15676	5.00	lobChIP_FOXA1_robot
chr9	32300564	32300565	MACS_peak_15677	7.00	lobChIP_FOXA1_robot
chr9	32361960	32361961	MACS_peak_15678	5.00	lobChIP_FOXA1_robot
chr9	32456821	32456822	MACS_peak_15679	10.00	lobChIP_FOXA1_robot
chr9	33029618	33029619	MACS_peak_15680	8.00	lobChIP_FOXA1_robot
chr9	33033005	33033006	MACS_peak_15681	8.00	lobChIP_FOXA1_robot
chr9	33294014	33294015	MACS_peak_15682	6.00	lobChIP_FOXA1_robot
chr9	33415748	33415749	MACS_peak_15683	44.00	lobChIP_FOXA1_robot
chr9	33807346	33807347	MACS_peak_15684	4.00	lobChIP_FOXA1_robot
chr9	33940394	33940395	MACS_peak_15685	8.00	lobChIP_FOXA1_robot
chr9	34035812	34035813	MACS_peak_15686	5.00	lobChIP_FOXA1_robot
chr9	34073358	34073359	MACS_peak_15687	16.00	lobChIP_FOXA1_robot
chr9	34142971	34142972	MACS_peak_15688	4.00	lobChIP_FOXA1_robot
chr9	34191447	34191448	MACS_peak_15689	5.00	lobChIP_FOXA1_robot
chr9	34512776	34512777	MACS_peak_15690	16.00	lobChIP_FOXA1_robot
chr9	35180163	35180164	MACS_peak_15691	6.00	lobChIP_FOXA1_robot
chr9	35257872	35257873	MACS_peak_15692	4.00	lobChIP_FOXA1_robot
chr9	35423684	35423685	MACS_peak_15693	5.00	lobChIP_FOXA1_robot
chr9	35518376	35518377	MACS_peak_15694	6.00	lobChIP_FOXA1_robot
chr9	35581330	35581331	MACS_peak_15695	6.00	lobChIP_FOXA1_robot
chr9	35652648	35652649	MACS_peak_15696	13.00	lobChIP_FOXA1_robot
chr9	36207675	36207676	MACS_peak_15697	4.00	lobChIP_FOXA1_robot
chr9	36420147	36420148	MACS_peak_15698	8.00	lobChIP_FOXA1_robot
chr9	36457983	36457984	MACS_peak_15699	5.00	lobChIP_FOXA1_robot
chr9	36597640	36597641	MACS_peak_15700	3.00	lobChIP_FOXA1_robot
chr9	36765453	36765454	MACS_peak_15701	4.00	lobChIP_FOXA1_robot
chr9	36868162	36868163	MACS_peak_15702	17.00	lobChIP_FOXA1_robot
chr9	36924195	36924196	MACS_peak_15703	5.00	lobChIP_FOXA1_robot
chr9	37097506	37097507	MACS_peak_15704	7.00	lobChIP_FOXA1_robot
chr9	37116849	37116850	MACS_peak_15705	4.00	lobChIP_FOXA1_robot
chr9	37239275	37239276	MACS_peak_15706	5.00	lobChIP_FOXA1_robot
chr9	37454487	37454488	MACS_peak_15707	10.00	lobChIP_FOXA1_robot
chr9	37908825	37908826	MACS_peak_15708	17.00	lobChIP_FOXA1_robot
chr9	37964578	37964579	MACS_peak_15709	4.00	lobChIP_FOXA1_robot
chr9	38047773	38047774	MACS_peak_15710	17.00	lobChIP_FOXA1_robot
chr9	38381260	38381261	MACS_peak_15711	17.00	lobChIP_FOXA1_robot
chr9	38386658	38386659	MACS_peak_15712	4.00	lobChIP_FOXA1_robot
chr9	38443429	38443430	MACS_peak_15713	9.00	lobChIP_FOXA1_robot
chr9	68418349	68418350	MACS_peak_15716	5.00	lobChIP_FOXA1_robot
chr9	69836028	69836029	MACS_peak_15717	4.00	lobChIP_FOXA1_robot
chr9	71292351	71292352	MACS_peak_15718	4.00	lobChIP_FOXA1_robot
chr9	71347545	71347546	MACS_peak_15719	12.00	lobChIP_FOXA1_robot
chr9	71369928	71369929	MACS_peak_15720	3.00	lobChIP_FOXA1_robot
chr9	71432170	71432171	MACS_peak_15721	11.00	lobChIP_FOXA1_robot
chr9	71584735	71584736	MACS_peak_15722	8.00	lobChIP_FOXA1_robot
chr9	71719827	71719828	MACS_peak_15723	13.00	lobChIP_FOXA1_robot
chr9	71735592	71735593	MACS_peak_15724	5.00	lobChIP_FOXA1_robot
chr9	71793570	71793571	MACS_peak_15725	17.00	lobChIP_FOXA1_robot
chr9	71801316	71801317	MACS_peak_15726	18.00	lobChIP_FOXA1_robot
chr9	71805322	71805323	MACS_peak_15727	9.00	lobChIP_FOXA1_robot
chr9	72135094	72135095	MACS_peak_15728	6.00	lobChIP_FOXA1_robot
chr9	72361949	72361950	MACS_peak_15729	3.00	lobChIP_FOXA1_robot
chr9	72363355	72363356	MACS_peak_15730	9.00	lobChIP_FOXA1_robot
chr9	72384872	72384873	MACS_peak_15731	4.00	lobChIP_FOXA1_robot
chr9	73035107	73035108	MACS_peak_15732	4.00	lobChIP_FOXA1_robot
chr9	73055449	73055450	MACS_peak_15733	4.00	lobChIP_FOXA1_robot
chr9	73056649	73056650	MACS_peak_15734	9.00	lobChIP_FOXA1_robot
chr9	73085441	73085442	MACS_peak_15735	4.00	lobChIP_FOXA1_robot
chr9	73711654	73711655	MACS_peak_15736	14.00	lobChIP_FOXA1_robot
chr9	73894278	73894279	MACS_peak_15737	9.00	lobChIP_FOXA1_robot
chr9	74269292	74269293	MACS_peak_15738	7.00	lobChIP_FOXA1_robot
chr9	74293042	74293043	MACS_peak_15739	6.00	lobChIP_FOXA1_robot
chr9	74341004	74341005	MACS_peak_15740	14.00	lobChIP_FOXA1_robot
chr9	74357431	74357432	MACS_peak_15741	9.00	lobChIP_FOXA1_robot
chr9	74373895	74373896	MACS_peak_15742	12.00	lobChIP_FOXA1_robot
chr9	74381854	74381855	MACS_peak_15743	3.00	lobChIP_FOXA1_robot
chr9	74393111	74393112	MACS_peak_15744	8.00	lobChIP_FOXA1_robot
chr9	74491204	74491205	MACS_peak_15745	7.00	lobChIP_FOXA1_robot
chr9	74736633	74736634	MACS_peak_15746	6.00	lobChIP_FOXA1_robot
chr9	74744922	74744923	MACS_peak_15747	4.00	lobChIP_FOXA1_robot
chr9	74822483	74822484	MACS_peak_15748	3.00	lobChIP_FOXA1_robot
chr9	74968941	74968942	MACS_peak_15749	6.00	lobChIP_FOXA1_robot
chr9	74977132	74977133	MACS_peak_15750	4.00	lobChIP_FOXA1_robot
chr9	75093364	75093365	MACS_peak_15751	10.00	lobChIP_FOXA1_robot
chr9	75443533	75443534	MACS_peak_15752	5.00	lobChIP_FOXA1_robot
chr9	75450109	75450110	MACS_peak_15753	7.00	lobChIP_FOXA1_robot
chr9	75540637	75540638	MACS_peak_15754	6.00	lobChIP_FOXA1_robot
chr9	75549298	75549299	MACS_peak_15755	15.00	lobChIP_FOXA1_robot
chr9	75594466	75594467	MACS_peak_15756	25.00	lobChIP_FOXA1_robot
chr9	75595692	75595693	MACS_peak_15757	8.00	lobChIP_FOXA1_robot
chr9	75612860	75612861	MACS_peak_15758	16.00	lobChIP_FOXA1_robot
chr9	75689213	75689214	MACS_peak_15759	3.00	lobChIP_FOXA1_robot
chr9	76158872	76158873	MACS_peak_15760	13.00	lobChIP_FOXA1_robot
chr9	76194904	76194905	MACS_peak_15761	7.00	lobChIP_FOXA1_robot
chr9	76289847	76289848	MACS_peak_15762	7.00	lobChIP_FOXA1_robot
chr9	76640550	76640551	MACS_peak_15763	22.00	lobChIP_FOXA1_robot
chr9	77616970	77616971	MACS_peak_15764	5.00	lobChIP_FOXA1_robot
chr9	77701756	77701757	MACS_peak_15765	6.00	lobChIP_FOXA1_robot
chr9	78988301	78988302	MACS_peak_15766	4.00	lobChIP_FOXA1_robot
chr9	78990330	78990331	MACS_peak_15767	6.00	lobChIP_FOXA1_robot
chr9	79017083	79017084	MACS_peak_15768	6.00	lobChIP_FOXA1_robot
chr9	79018908	79018909	MACS_peak_15769	5.00	lobChIP_FOXA1_robot
chr9	79038930	79038931	MACS_peak_15770	13.00	lobChIP_FOXA1_robot
chr9	79071265	79071266	MACS_peak_15771	6.00	lobChIP_FOXA1_robot
chr9	79345055	79345056	MACS_peak_15772	7.00	lobChIP_FOXA1_robot
chr9	79786273	79786274	MACS_peak_15773	28.00	lobChIP_FOXA1_robot
chr9	80128008	80128009	MACS_peak_15774	17.00	lobChIP_FOXA1_robot
chr9	80378648	80378649	MACS_peak_15775	6.00	lobChIP_FOXA1_robot
chr9	80423019	80423020	MACS_peak_15776	11.00	lobChIP_FOXA1_robot
chr9	80466889	80466890	MACS_peak_15777	13.00	lobChIP_FOXA1_robot
chr9	80619581	80619582	MACS_peak_15778	6.00	lobChIP_FOXA1_robot
chr9	80902267	80902268	MACS_peak_15779	7.00	lobChIP_FOXA1_robot
chr9	80969702	80969703	MACS_peak_15780	8.00	lobChIP_FOXA1_robot
chr9	81014261	81014262	MACS_peak_15781	5.00	lobChIP_FOXA1_robot
chr9	81022036	81022037	MACS_peak_15782	8.00	lobChIP_FOXA1_robot
chr9	82104691	82104692	MACS_peak_15783	28.00	lobChIP_FOXA1_robot
chr9	82188707	82188708	MACS_peak_15784	5.00	lobChIP_FOXA1_robot
chr9	82388430	82388431	MACS_peak_15785	4.00	lobChIP_FOXA1_robot
chr9	82395693	82395694	MACS_peak_15786	4.00	lobChIP_FOXA1_robot
chr9	83613408	83613409	MACS_peak_15787	4.00	lobChIP_FOXA1_robot
chr9	84150621	84150622	MACS_peak_15788	34.00	lobChIP_FOXA1_robot
chr9	84153999	84154000	MACS_peak_15789	7.00	lobChIP_FOXA1_robot
chr9	84161617	84161618	MACS_peak_15790	9.00	lobChIP_FOXA1_robot
chr9	84164997	84164998	MACS_peak_15791	10.00	lobChIP_FOXA1_robot
chr9	84202843	84202844	MACS_peak_15792	29.00	lobChIP_FOXA1_robot
chr9	84220227	84220228	MACS_peak_15793	8.00	lobChIP_FOXA1_robot
chr9	84225540	84225541	MACS_peak_15794	24.00	lobChIP_FOXA1_robot
chr9	84244101	84244102	MACS_peak_15795	11.00	lobChIP_FOXA1_robot
chr9	84249537	84249538	MACS_peak_15796	5.00	lobChIP_FOXA1_robot
chr9	84271989	84271990	MACS_peak_15797	42.00	lobChIP_FOXA1_robot
chr9	84300465	84300466	MACS_peak_15798	4.00	lobChIP_FOXA1_robot
chr9	84301924	84301925	MACS_peak_15799	35.00	lobChIP_FOXA1_robot
chr9	84304728	84304729	MACS_peak_15800	3.00	lobChIP_FOXA1_robot
chr9	84429592	84429593	MACS_peak_15801	10.00	lobChIP_FOXA1_robot
chr9	85175523	85175524	MACS_peak_15802	6.00	lobChIP_FOXA1_robot
chr9	85214719	85214720	MACS_peak_15803	4.00	lobChIP_FOXA1_robot
chr9	86002597	86002598	MACS_peak_15804	4.00	lobChIP_FOXA1_robot
chr9	86039178	86039179	MACS_peak_15805	4.00	lobChIP_FOXA1_robot
chr9	86087396	86087397	MACS_peak_15806	4.00	lobChIP_FOXA1_robot
chr9	86244702	86244703	MACS_peak_15807	5.00	lobChIP_FOXA1_robot
chr9	86601669	86601670	MACS_peak_15808	11.00	lobChIP_FOXA1_robot
chr9	86607648	86607649	MACS_peak_15809	5.00	lobChIP_FOXA1_robot
chr9	88708807	88708808	MACS_peak_15810	93.00	lobChIP_FOXA1_robot
chr9	88951147	88951148	MACS_peak_15811	4.00	lobChIP_FOXA1_robot
chr9	89020270	89020271	MACS_peak_15812	44.00	lobChIP_FOXA1_robot
chr9	89558537	89558538	MACS_peak_15813	8.00	lobChIP_FOXA1_robot
chr9	90011206	90011207	MACS_peak_15814	24.00	lobChIP_FOXA1_robot
chr9	90095013	90095014	MACS_peak_15815	3.00	lobChIP_FOXA1_robot
chr9	90185153	90185154	MACS_peak_15816	39.00	lobChIP_FOXA1_robot
chr9	90185857	90185858	MACS_peak_15817	11.00	lobChIP_FOXA1_robot
chr9	90228405	90228406	MACS_peak_15818	12.00	lobChIP_FOXA1_robot
chr9	90329929	90329930	MACS_peak_15819	15.00	lobChIP_FOXA1_robot
chr9	90340777	90340778	MACS_peak_15820	4.00	lobChIP_FOXA1_robot
chr9	90408443	90408444	MACS_peak_15821	5.00	lobChIP_FOXA1_robot
chr9	91321343	91321344	MACS_peak_15822	18.00	lobChIP_FOXA1_robot
chr9	91966294	91966295	MACS_peak_15823	7.00	lobChIP_FOXA1_robot
chr9	91972361	91972362	MACS_peak_15824	7.00	lobChIP_FOXA1_robot
chr9	92171722	92171723	MACS_peak_15825	8.00	lobChIP_FOXA1_robot
chr9	92263397	92263398	MACS_peak_15826	12.00	lobChIP_FOXA1_robot
chr9	92756558	92756559	MACS_peak_15827	5.00	lobChIP_FOXA1_robot
chr9	92762493	92762494	MACS_peak_15828	9.00	lobChIP_FOXA1_robot
chr9	92931941	92931942	MACS_peak_15829	7.00	lobChIP_FOXA1_robot
chr9	93799728	93799729	MACS_peak_15830	12.00	lobChIP_FOXA1_robot
chr9	93956254	93956255	MACS_peak_15831	3.00	lobChIP_FOXA1_robot
chr9	93960197	93960198	MACS_peak_15832	28.00	lobChIP_FOXA1_robot
chr9	94157509	94157510	MACS_peak_15833	4.00	lobChIP_FOXA1_robot
chr9	94169357	94169358	MACS_peak_15834	5.00	lobChIP_FOXA1_robot
chr9	94181618	94181619	MACS_peak_15835	5.00	lobChIP_FOXA1_robot
chr9	94293273	94293274	MACS_peak_15836	7.00	lobChIP_FOXA1_robot
chr9	94756846	94756847	MACS_peak_15837	10.00	lobChIP_FOXA1_robot
chr9	95379324	95379325	MACS_peak_15838	7.00	lobChIP_FOXA1_robot
chr9	95387118	95387119	MACS_peak_15839	11.00	lobChIP_FOXA1_robot
chr9	95674257	95674258	MACS_peak_15840	10.00	lobChIP_FOXA1_robot
chr9	96075459	96075460	MACS_peak_15841	17.00	lobChIP_FOXA1_robot
chr9	96647255	96647256	MACS_peak_15842	6.00	lobChIP_FOXA1_robot
chr9	97270341	97270342	MACS_peak_15843	8.00	lobChIP_FOXA1_robot
chr9	97326000	97326001	MACS_peak_15844	6.00	lobChIP_FOXA1_robot
chr9	97370406	97370407	MACS_peak_15845	4.00	lobChIP_FOXA1_robot
chr9	97370995	97370996	MACS_peak_15846	9.00	lobChIP_FOXA1_robot
chr9	97374447	97374448	MACS_peak_15847	5.00	lobChIP_FOXA1_robot
chr9	97456625	97456626	MACS_peak_15848	4.00	lobChIP_FOXA1_robot
chr9	97466225	97466226	MACS_peak_15849	5.00	lobChIP_FOXA1_robot
chr9	97469423	97469424	MACS_peak_15850	4.00	lobChIP_FOXA1_robot
chr9	97589158	97589159	MACS_peak_15851	5.00	lobChIP_FOXA1_robot
chr9	97589976	97589977	MACS_peak_15852	16.00	lobChIP_FOXA1_robot
chr9	97616320	97616321	MACS_peak_15853	4.00	lobChIP_FOXA1_robot
chr9	97629198	97629199	MACS_peak_15854	5.00	lobChIP_FOXA1_robot
chr9	97629662	97629663	MACS_peak_15855	20.00	lobChIP_FOXA1_robot
chr9	97648269	97648270	MACS_peak_15856	5.00	lobChIP_FOXA1_robot
chr9	97675410	97675411	MACS_peak_15857	3.00	lobChIP_FOXA1_robot
chr9	97676109	97676110	MACS_peak_15858	9.00	lobChIP_FOXA1_robot
chr9	97684800	97684801	MACS_peak_15859	11.00	lobChIP_FOXA1_robot
chr9	97704116	97704117	MACS_peak_15860	10.00	lobChIP_FOXA1_robot
chr9	97713385	97713386	MACS_peak_15861	4.00	lobChIP_FOXA1_robot
chr9	97752350	97752351	MACS_peak_15862	7.00	lobChIP_FOXA1_robot
chr9	97753647	97753648	MACS_peak_15863	5.00	lobChIP_FOXA1_robot
chr9	97754381	97754382	MACS_peak_15864	6.00	lobChIP_FOXA1_robot
chr9	97755624	97755625	MACS_peak_15865	7.00	lobChIP_FOXA1_robot
chr9	97785919	97785920	MACS_peak_15866	5.00	lobChIP_FOXA1_robot
chr9	97786597	97786598	MACS_peak_15867	5.00	lobChIP_FOXA1_robot
chr9	97794059	97794060	MACS_peak_15868	13.00	lobChIP_FOXA1_robot
chr9	97827044	97827045	MACS_peak_15869	4.00	lobChIP_FOXA1_robot
chr9	97892376	97892377	MACS_peak_15870	7.00	lobChIP_FOXA1_robot
chr9	97906442	97906443	MACS_peak_15871	6.00	lobChIP_FOXA1_robot
chr9	97951671	97951672	MACS_peak_15872	6.00	lobChIP_FOXA1_robot
chr9	98044820	98044821	MACS_peak_15873	5.00	lobChIP_FOXA1_robot
chr9	98266492	98266493	MACS_peak_15874	13.00	lobChIP_FOXA1_robot
chr9	98486996	98486997	MACS_peak_15875	4.00	lobChIP_FOXA1_robot
chr9	98533923	98533924	MACS_peak_15876	10.00	lobChIP_FOXA1_robot
chr9	98803336	98803337	MACS_peak_15877	6.00	lobChIP_FOXA1_robot
chr9	99102080	99102081	MACS_peak_15878	11.00	lobChIP_FOXA1_robot
chr9	99148643	99148644	MACS_peak_15879	9.00	lobChIP_FOXA1_robot
chr9	99182772	99182773	MACS_peak_15880	4.00	lobChIP_FOXA1_robot
chr9	99197850	99197851	MACS_peak_15881	9.00	lobChIP_FOXA1_robot
chr9	99253052	99253053	MACS_peak_15882	10.00	lobChIP_FOXA1_robot
chr9	99253731	99253732	MACS_peak_15883	21.00	lobChIP_FOXA1_robot
chr9	99284769	99284770	MACS_peak_15884	4.00	lobChIP_FOXA1_robot
chr9	99321756	99321757	MACS_peak_15885	4.00	lobChIP_FOXA1_robot
chr9	99328692	99328693	MACS_peak_15886	6.00	lobChIP_FOXA1_robot
chr9	99380597	99380598	MACS_peak_15887	11.00	lobChIP_FOXA1_robot
chr9	99406587	99406588	MACS_peak_15888	32.00	lobChIP_FOXA1_robot
chr9	99618869	99618870	MACS_peak_15889	9.00	lobChIP_FOXA1_robot
chr9	99874044	99874045	MACS_peak_15890	3.00	lobChIP_FOXA1_robot
chr9	100147452	100147453	MACS_peak_15891	7.00	lobChIP_FOXA1_robot
chr9	100157432	100157433	MACS_peak_15892	9.00	lobChIP_FOXA1_robot
chr9	100167252	100167253	MACS_peak_15893	47.00	lobChIP_FOXA1_robot
chr9	100395337	100395338	MACS_peak_15894	3.00	lobChIP_FOXA1_robot
chr9	100522430	100522431	MACS_peak_15895	3.00	lobChIP_FOXA1_robot
chr9	100551682	100551683	MACS_peak_15896	7.00	lobChIP_FOXA1_robot
chr9	100920749	100920750	MACS_peak_15897	13.00	lobChIP_FOXA1_robot
chr9	100988922	100988923	MACS_peak_15898	10.00	lobChIP_FOXA1_robot
chr9	100990808	100990809	MACS_peak_15899	7.00	lobChIP_FOXA1_robot
chr9	101253681	101253682	MACS_peak_15900	8.00	lobChIP_FOXA1_robot
chr9	103126559	103126560	MACS_peak_15901	8.00	lobChIP_FOXA1_robot
chr9	103131242	103131243	MACS_peak_15902	3.00	lobChIP_FOXA1_robot
chr9	103791037	103791038	MACS_peak_15903	5.00	lobChIP_FOXA1_robot
chr9	103825247	103825248	MACS_peak_15904	3.00	lobChIP_FOXA1_robot
chr9	103924794	103924795	MACS_peak_15905	5.00	lobChIP_FOXA1_robot
chr9	103947244	103947245	MACS_peak_15906	7.00	lobChIP_FOXA1_robot
chr9	104077578	104077579	MACS_peak_15907	16.00	lobChIP_FOXA1_robot
chr9	104086793	104086794	MACS_peak_15908	18.00	lobChIP_FOXA1_robot
chr9	104089777	104089778	MACS_peak_15909	3.00	lobChIP_FOXA1_robot
chr9	104112793	104112794	MACS_peak_15910	7.00	lobChIP_FOXA1_robot
chr9	104116862	104116863	MACS_peak_15911	15.00	lobChIP_FOXA1_robot
chr9	104122202	104122203	MACS_peak_15912	9.00	lobChIP_FOXA1_robot
chr9	104128568	104128569	MACS_peak_15913	5.00	lobChIP_FOXA1_robot
chr9	104150321	104150322	MACS_peak_15914	4.00	lobChIP_FOXA1_robot
chr9	104151250	104151251	MACS_peak_15915	28.00	lobChIP_FOXA1_robot
chr9	104184097	104184098	MACS_peak_15916	8.00	lobChIP_FOXA1_robot
chr9	104195800	104195801	MACS_peak_15917	11.00	lobChIP_FOXA1_robot
chr9	104207865	104207866	MACS_peak_15918	5.00	lobChIP_FOXA1_robot
chr9	105602946	105602947	MACS_peak_15919	3.00	lobChIP_FOXA1_robot
chr9	106123255	106123256	MACS_peak_15920	4.00	lobChIP_FOXA1_robot
chr9	107444216	107444217	MACS_peak_15921	4.00	lobChIP_FOXA1_robot
chr9	107528466	107528467	MACS_peak_15922	6.00	lobChIP_FOXA1_robot
chr9	107618952	107618953	MACS_peak_15923	16.00	lobChIP_FOXA1_robot
chr9	107623254	107623255	MACS_peak_15924	4.00	lobChIP_FOXA1_robot
chr9	107648765	107648766	MACS_peak_15925	19.00	lobChIP_FOXA1_robot
chr9	107652089	107652090	MACS_peak_15926	6.00	lobChIP_FOXA1_robot
chr9	107652866	107652867	MACS_peak_15927	4.00	lobChIP_FOXA1_robot
chr9	107674967	107674968	MACS_peak_15928	34.00	lobChIP_FOXA1_robot
chr9	107755131	107755132	MACS_peak_15929	4.00	lobChIP_FOXA1_robot
chr9	107827733	107827734	MACS_peak_15930	11.00	lobChIP_FOXA1_robot
chr9	108014497	108014498	MACS_peak_15931	7.00	lobChIP_FOXA1_robot
chr9	108053887	108053888	MACS_peak_15932	10.00	lobChIP_FOXA1_robot
chr9	108081345	108081346	MACS_peak_15933	6.00	lobChIP_FOXA1_robot
chr9	108093063	108093064	MACS_peak_15934	4.00	lobChIP_FOXA1_robot
chr9	108180936	108180937	MACS_peak_15935	10.00	lobChIP_FOXA1_robot
chr9	108260707	108260708	MACS_peak_15936	4.00	lobChIP_FOXA1_robot
chr9	108269537	108269538	MACS_peak_15937	4.00	lobChIP_FOXA1_robot
chr9	108578853	108578854	MACS_peak_15938	3.00	lobChIP_FOXA1_robot
chr9	108972840	108972841	MACS_peak_15939	4.00	lobChIP_FOXA1_robot
chr9	109556664	109556665	MACS_peak_15940	6.00	lobChIP_FOXA1_robot
chr9	109975106	109975107	MACS_peak_15941	6.00	lobChIP_FOXA1_robot
chr9	110006204	110006205	MACS_peak_15942	7.00	lobChIP_FOXA1_robot
chr9	110011712	110011713	MACS_peak_15943	31.00	lobChIP_FOXA1_robot
chr9	110014491	110014492	MACS_peak_15944	12.00	lobChIP_FOXA1_robot
chr9	110022916	110022917	MACS_peak_15945	5.00	lobChIP_FOXA1_robot
chr9	110032163	110032164	MACS_peak_15946	6.00	lobChIP_FOXA1_robot
chr9	110047207	110047208	MACS_peak_15947	13.00	lobChIP_FOXA1_robot
chr9	110048599	110048600	MACS_peak_15948	6.00	lobChIP_FOXA1_robot
chr9	110130424	110130425	MACS_peak_15949	4.00	lobChIP_FOXA1_robot
chr9	110267261	110267262	MACS_peak_15950	6.00	lobChIP_FOXA1_robot
chr9	110309361	110309362	MACS_peak_15951	22.00	lobChIP_FOXA1_robot
chr9	110871573	110871574	MACS_peak_15952	5.00	lobChIP_FOXA1_robot
chr9	110932033	110932034	MACS_peak_15953	3.00	lobChIP_FOXA1_robot
chr9	111205873	111205874	MACS_peak_15954	5.00	lobChIP_FOXA1_robot
chr9	111207855	111207856	MACS_peak_15955	15.00	lobChIP_FOXA1_robot
chr9	111236125	111236126	MACS_peak_15956	3.00	lobChIP_FOXA1_robot
chr9	111538405	111538406	MACS_peak_15957	7.00	lobChIP_FOXA1_robot
chr9	111578344	111578345	MACS_peak_15958	6.00	lobChIP_FOXA1_robot
chr9	111631519	111631520	MACS_peak_15959	4.00	lobChIP_FOXA1_robot
chr9	111648404	111648405	MACS_peak_15960	11.00	lobChIP_FOXA1_robot
chr9	111652235	111652236	MACS_peak_15961	14.00	lobChIP_FOXA1_robot
chr9	111656716	111656717	MACS_peak_15962	16.00	lobChIP_FOXA1_robot
chr9	111660282	111660283	MACS_peak_15963	3.00	lobChIP_FOXA1_robot
chr9	111702474	111702475	MACS_peak_15964	3.00	lobChIP_FOXA1_robot
chr9	111716402	111716403	MACS_peak_15965	5.00	lobChIP_FOXA1_robot
chr9	111773231	111773232	MACS_peak_15966	3.00	lobChIP_FOXA1_robot
chr9	111785288	111785289	MACS_peak_15967	6.00	lobChIP_FOXA1_robot
chr9	111798246	111798247	MACS_peak_15968	3.00	lobChIP_FOXA1_robot
chr9	111835669	111835670	MACS_peak_15969	5.00	lobChIP_FOXA1_robot
chr9	111844489	111844490	MACS_peak_15970	15.00	lobChIP_FOXA1_robot
chr9	111944966	111944967	MACS_peak_15971	4.00	lobChIP_FOXA1_robot
chr9	112013603	112013604	MACS_peak_15972	5.00	lobChIP_FOXA1_robot
chr9	112027306	112027307	MACS_peak_15973	6.00	lobChIP_FOXA1_robot
chr9	112097269	112097270	MACS_peak_15974	4.00	lobChIP_FOXA1_robot
chr9	112268525	112268526	MACS_peak_15975	4.00	lobChIP_FOXA1_robot
chr9	112269916	112269917	MACS_peak_15976	9.00	lobChIP_FOXA1_robot
chr9	112943314	112943315	MACS_peak_15977	5.00	lobChIP_FOXA1_robot
chr9	112956717	112956718	MACS_peak_15978	25.00	lobChIP_FOXA1_robot
chr9	112960094	112960095	MACS_peak_15979	5.00	lobChIP_FOXA1_robot
chr9	112975936	112975937	MACS_peak_15980	9.00	lobChIP_FOXA1_robot
chr9	112977456	112977457	MACS_peak_15981	7.00	lobChIP_FOXA1_robot
chr9	113044554	113044555	MACS_peak_15982	5.00	lobChIP_FOXA1_robot
chr9	113532690	113532691	MACS_peak_15983	6.00	lobChIP_FOXA1_robot
chr9	113913921	113913922	MACS_peak_15984	6.00	lobChIP_FOXA1_robot
chr9	114076742	114076743	MACS_peak_15985	4.00	lobChIP_FOXA1_robot
chr9	114111256	114111257	MACS_peak_15986	23.00	lobChIP_FOXA1_robot
chr9	114112262	114112263	MACS_peak_15987	28.00	lobChIP_FOXA1_robot
chr9	114115035	114115036	MACS_peak_15988	14.00	lobChIP_FOXA1_robot
chr9	114385052	114385053	MACS_peak_15989	6.00	lobChIP_FOXA1_robot
chr9	114392577	114392578	MACS_peak_15990	6.00	lobChIP_FOXA1_robot
chr9	114397374	114397375	MACS_peak_15991	15.00	lobChIP_FOXA1_robot
chr9	114796080	114796081	MACS_peak_15992	10.00	lobChIP_FOXA1_robot
chr9	114881925	114881926	MACS_peak_15993	5.00	lobChIP_FOXA1_robot
chr9	114888393	114888394	MACS_peak_15994	8.00	lobChIP_FOXA1_robot
chr9	115034136	115034137	MACS_peak_15995	4.00	lobChIP_FOXA1_robot
chr9	115100310	115100311	MACS_peak_15996	3.00	lobChIP_FOXA1_robot
chr9	115104128	115104129	MACS_peak_15997	4.00	lobChIP_FOXA1_robot
chr9	115114655	115114656	MACS_peak_15998	8.00	lobChIP_FOXA1_robot
chr9	115145095	115145096	MACS_peak_15999	16.00	lobChIP_FOXA1_robot
chr9	115251593	115251594	MACS_peak_16000	10.00	lobChIP_FOXA1_robot
chr9	115258391	115258392	MACS_peak_16001	3.00	lobChIP_FOXA1_robot
chr9	115268072	115268073	MACS_peak_16002	6.00	lobChIP_FOXA1_robot
chr9	115378335	115378336	MACS_peak_16003	5.00	lobChIP_FOXA1_robot
chr9	115603715	115603716	MACS_peak_16004	10.00	lobChIP_FOXA1_robot
chr9	115990749	115990750	MACS_peak_16005	4.00	lobChIP_FOXA1_robot
chr9	116145524	116145525	MACS_peak_16006	6.00	lobChIP_FOXA1_robot
chr9	116232586	116232587	MACS_peak_16007	5.00	lobChIP_FOXA1_robot
chr9	116397587	116397588	MACS_peak_16008	3.00	lobChIP_FOXA1_robot
chr9	116412831	116412832	MACS_peak_16009	6.00	lobChIP_FOXA1_robot
chr9	116415292	116415293	MACS_peak_16010	5.00	lobChIP_FOXA1_robot
chr9	116843316	116843317	MACS_peak_16011	19.00	lobChIP_FOXA1_robot
chr9	116880713	116880714	MACS_peak_16012	28.00	lobChIP_FOXA1_robot
chr9	116904411	116904412	MACS_peak_16013	10.00	lobChIP_FOXA1_robot
chr9	116937003	116937004	MACS_peak_16014	5.00	lobChIP_FOXA1_robot
chr9	117091656	117091657	MACS_peak_16015	6.00	lobChIP_FOXA1_robot
chr9	117484138	117484139	MACS_peak_16016	11.00	lobChIP_FOXA1_robot
chr9	117505435	117505436	MACS_peak_16017	10.00	lobChIP_FOXA1_robot
chr9	117650508	117650509	MACS_peak_16018	5.00	lobChIP_FOXA1_robot
chr9	117657170	117657171	MACS_peak_16019	3.00	lobChIP_FOXA1_robot
chr9	117670462	117670463	MACS_peak_16020	8.00	lobChIP_FOXA1_robot
chr9	118136059	118136060	MACS_peak_16021	4.00	lobChIP_FOXA1_robot
chr9	118257771	118257772	MACS_peak_16022	36.00	lobChIP_FOXA1_robot
chr9	118706805	118706806	MACS_peak_16023	22.00	lobChIP_FOXA1_robot
chr9	118709073	118709074	MACS_peak_16024	13.00	lobChIP_FOXA1_robot
chr9	118758849	118758850	MACS_peak_16025	4.00	lobChIP_FOXA1_robot
chr9	119098925	119098926	MACS_peak_16026	4.00	lobChIP_FOXA1_robot
chr9	119336772	119336773	MACS_peak_16027	9.00	lobChIP_FOXA1_robot
chr9	120253910	120253911	MACS_peak_16028	10.00	lobChIP_FOXA1_robot
chr9	123148772	123148773	MACS_peak_16029	3.00	lobChIP_FOXA1_robot
chr9	123300800	123300801	MACS_peak_16030	6.00	lobChIP_FOXA1_robot
chr9	123333762	123333763	MACS_peak_16031	14.00	lobChIP_FOXA1_robot
chr9	123334271	123334272	MACS_peak_16032	8.00	lobChIP_FOXA1_robot
chr9	123402871	123402872	MACS_peak_16033	13.00	lobChIP_FOXA1_robot
chr9	123412771	123412772	MACS_peak_16034	4.00	lobChIP_FOXA1_robot
chr9	123446868	123446869	MACS_peak_16035	4.00	lobChIP_FOXA1_robot
chr9	123493097	123493098	MACS_peak_16036	8.00	lobChIP_FOXA1_robot
chr9	123497941	123497942	MACS_peak_16037	7.00	lobChIP_FOXA1_robot
chr9	123529203	123529204	MACS_peak_16038	7.00	lobChIP_FOXA1_robot
chr9	123541228	123541229	MACS_peak_16039	49.00	lobChIP_FOXA1_robot
chr9	123546130	123546131	MACS_peak_16040	8.00	lobChIP_FOXA1_robot
chr9	123760621	123760622	MACS_peak_16041	8.00	lobChIP_FOXA1_robot
chr9	123788775	123788776	MACS_peak_16042	5.00	lobChIP_FOXA1_robot
chr9	123811061	123811062	MACS_peak_16043	5.00	lobChIP_FOXA1_robot
chr9	123812631	123812632	MACS_peak_16044	5.00	lobChIP_FOXA1_robot
chr9	123814246	123814247	MACS_peak_16045	39.00	lobChIP_FOXA1_robot
chr9	123941814	123941815	MACS_peak_16046	6.00	lobChIP_FOXA1_robot
chr9	123946198	123946199	MACS_peak_16047	4.00	lobChIP_FOXA1_robot
chr9	124084700	124084701	MACS_peak_16048	28.00	lobChIP_FOXA1_robot
chr9	124085489	124085490	MACS_peak_16049	3.00	lobChIP_FOXA1_robot
chr9	124114265	124114266	MACS_peak_16050	4.00	lobChIP_FOXA1_robot
chr9	124130533	124130534	MACS_peak_16051	17.00	lobChIP_FOXA1_robot
chr9	124134016	124134017	MACS_peak_16052	4.00	lobChIP_FOXA1_robot
chr9	124134934	124134935	MACS_peak_16053	40.00	lobChIP_FOXA1_robot
chr9	124953066	124953067	MACS_peak_16054	9.00	lobChIP_FOXA1_robot
chr9	124961168	124961169	MACS_peak_16055	5.00	lobChIP_FOXA1_robot
chr9	125623342	125623343	MACS_peak_16056	4.00	lobChIP_FOXA1_robot
chr9	125774567	125774568	MACS_peak_16057	5.00	lobChIP_FOXA1_robot
chr9	125849793	125849794	MACS_peak_16058	14.00	lobChIP_FOXA1_robot
chr9	125894496	125894497	MACS_peak_16059	6.00	lobChIP_FOXA1_robot
chr9	126073746	126073747	MACS_peak_16060	3.00	lobChIP_FOXA1_robot
chr9	126078819	126078820	MACS_peak_16061	5.00	lobChIP_FOXA1_robot
chr9	126227862	126227863	MACS_peak_16062	6.00	lobChIP_FOXA1_robot
chr9	126229417	126229418	MACS_peak_16063	5.00	lobChIP_FOXA1_robot
chr9	126329450	126329451	MACS_peak_16064	6.00	lobChIP_FOXA1_robot
chr9	126440500	126440501	MACS_peak_16065	17.00	lobChIP_FOXA1_robot
chr9	126441110	126441111	MACS_peak_16066	22.00	lobChIP_FOXA1_robot
chr9	126501168	126501169	MACS_peak_16067	6.00	lobChIP_FOXA1_robot
chr9	126502417	126502418	MACS_peak_16068	6.00	lobChIP_FOXA1_robot
chr9	126508382	126508383	MACS_peak_16069	5.00	lobChIP_FOXA1_robot
chr9	126684679	126684680	MACS_peak_16070	5.00	lobChIP_FOXA1_robot
chr9	126685391	126685392	MACS_peak_16071	4.00	lobChIP_FOXA1_robot
chr9	126985728	126985729	MACS_peak_16072	4.00	lobChIP_FOXA1_robot
chr9	127021894	127021895	MACS_peak_16073	6.00	lobChIP_FOXA1_robot
chr9	127046775	127046776	MACS_peak_16074	7.00	lobChIP_FOXA1_robot
chr9	127148606	127148607	MACS_peak_16075	45.00	lobChIP_FOXA1_robot
chr9	127153338	127153339	MACS_peak_16076	3.00	lobChIP_FOXA1_robot
chr9	127282610	127282611	MACS_peak_16077	5.00	lobChIP_FOXA1_robot
chr9	127328255	127328256	MACS_peak_16078	5.00	lobChIP_FOXA1_robot
chr9	127329659	127329660	MACS_peak_16079	4.00	lobChIP_FOXA1_robot
chr9	127428092	127428093	MACS_peak_16080	3.00	lobChIP_FOXA1_robot
chr9	127457393	127457394	MACS_peak_16081	5.00	lobChIP_FOXA1_robot
chr9	127483527	127483528	MACS_peak_16082	9.00	lobChIP_FOXA1_robot
chr9	127496486	127496487	MACS_peak_16083	7.00	lobChIP_FOXA1_robot
chr9	127712482	127712483	MACS_peak_16084	12.00	lobChIP_FOXA1_robot
chr9	128035101	128035102	MACS_peak_16085	12.00	lobChIP_FOXA1_robot
chr9	128046998	128046999	MACS_peak_16086	5.00	lobChIP_FOXA1_robot
chr9	128059464	128059465	MACS_peak_16087	44.00	lobChIP_FOXA1_robot
chr9	128071279	128071280	MACS_peak_16088	4.00	lobChIP_FOXA1_robot
chr9	128300519	128300520	MACS_peak_16089	19.00	lobChIP_FOXA1_robot
chr9	128310553	128310554	MACS_peak_16090	8.00	lobChIP_FOXA1_robot
chr9	128444375	128444376	MACS_peak_16091	6.00	lobChIP_FOXA1_robot
chr9	128521606	128521607	MACS_peak_16092	7.00	lobChIP_FOXA1_robot
chr9	128613207	128613208	MACS_peak_16093	4.00	lobChIP_FOXA1_robot
chr9	128913495	128913496	MACS_peak_16094	4.00	lobChIP_FOXA1_robot
chr9	129097437	129097438	MACS_peak_16095	7.00	lobChIP_FOXA1_robot
chr9	129133918	129133919	MACS_peak_16096	4.00	lobChIP_FOXA1_robot
chr9	129138901	129138902	MACS_peak_16097	4.00	lobChIP_FOXA1_robot
chr9	129139362	129139363	MACS_peak_16098	5.00	lobChIP_FOXA1_robot
chr9	129571586	129571587	MACS_peak_16099	13.00	lobChIP_FOXA1_robot
chr9	129681605	129681606	MACS_peak_16100	11.00	lobChIP_FOXA1_robot
chr9	129871813	129871814	MACS_peak_16101	7.00	lobChIP_FOXA1_robot
chr9	130071185	130071186	MACS_peak_16102	7.00	lobChIP_FOXA1_robot
chr9	130150820	130150821	MACS_peak_16103	7.00	lobChIP_FOXA1_robot
chr9	130497868	130497869	MACS_peak_16104	4.00	lobChIP_FOXA1_robot
chr9	130555915	130555916	MACS_peak_16105	3.00	lobChIP_FOXA1_robot
chr9	130663344	130663345	MACS_peak_16106	7.00	lobChIP_FOXA1_robot
chr9	130717874	130717875	MACS_peak_16107	8.00	lobChIP_FOXA1_robot
chr9	130835728	130835729	MACS_peak_16108	9.00	lobChIP_FOXA1_robot
chr9	131057986	131057987	MACS_peak_16109	4.00	lobChIP_FOXA1_robot
chr9	131600944	131600945	MACS_peak_16110	5.00	lobChIP_FOXA1_robot
chr9	131638265	131638266	MACS_peak_16111	6.00	lobChIP_FOXA1_robot
chr9	132096743	132096744	MACS_peak_16112	4.00	lobChIP_FOXA1_robot
chr9	132106272	132106273	MACS_peak_16113	4.00	lobChIP_FOXA1_robot
chr9	132107611	132107612	MACS_peak_16114	5.00	lobChIP_FOXA1_robot
chr9	132176923	132176924	MACS_peak_16115	6.00	lobChIP_FOXA1_robot
chr9	132241761	132241762	MACS_peak_16116	5.00	lobChIP_FOXA1_robot
chr9	132258140	132258141	MACS_peak_16117	5.00	lobChIP_FOXA1_robot
chr9	132319948	132319949	MACS_peak_16118	8.00	lobChIP_FOXA1_robot
chr9	132787040	132787041	MACS_peak_16119	4.00	lobChIP_FOXA1_robot
chr9	132790947	132790948	MACS_peak_16120	8.00	lobChIP_FOXA1_robot
chr9	133473769	133473770	MACS_peak_16121	4.00	lobChIP_FOXA1_robot
chr9	133484155	133484156	MACS_peak_16122	6.00	lobChIP_FOXA1_robot
chr9	133569131	133569132	MACS_peak_16123	7.00	lobChIP_FOXA1_robot
chr9	133655087	133655088	MACS_peak_16124	5.00	lobChIP_FOXA1_robot
chr9	134278464	134278465	MACS_peak_16125	5.00	lobChIP_FOXA1_robot
chr9	134287605	134287606	MACS_peak_16126	3.00	lobChIP_FOXA1_robot
chr9	134420850	134420851	MACS_peak_16127	9.00	lobChIP_FOXA1_robot
chr9	134422215	134422216	MACS_peak_16128	18.00	lobChIP_FOXA1_robot
chr9	134481812	134481813	MACS_peak_16129	5.00	lobChIP_FOXA1_robot
chr9	134533057	134533058	MACS_peak_16130	3.00	lobChIP_FOXA1_robot
chr9	134553500	134553501	MACS_peak_16131	7.00	lobChIP_FOXA1_robot
chr9	134611028	134611029	MACS_peak_16132	5.00	lobChIP_FOXA1_robot
chr9	134884628	134884629	MACS_peak_16133	10.00	lobChIP_FOXA1_robot
chr9	136009266	136009267	MACS_peak_16134	6.00	lobChIP_FOXA1_robot
chr9	136610904	136610905	MACS_peak_16135	16.00	lobChIP_FOXA1_robot
chr9	136750544	136750545	MACS_peak_16136	7.00	lobChIP_FOXA1_robot
chr9	136757536	136757537	MACS_peak_16137	25.00	lobChIP_FOXA1_robot
chr9	136843928	136843929	MACS_peak_16138	6.00	lobChIP_FOXA1_robot
chr9	137151233	137151234	MACS_peak_16139	9.00	lobChIP_FOXA1_robot
chr9	137220512	137220513	MACS_peak_16140	4.00	lobChIP_FOXA1_robot
chr9	139136198	139136199	MACS_peak_16141	6.00	lobChIP_FOXA1_robot
chr9	139466719	139466720	MACS_peak_16142	6.00	lobChIP_FOXA1_robot
chr9	139521291	139521292	MACS_peak_16143	42.00	lobChIP_FOXA1_robot
chr9	139592602	139592603	MACS_peak_16144	7.00	lobChIP_FOXA1_robot
chr9	139951709	139951710	MACS_peak_16145	4.00	lobChIP_FOXA1_robot
chr9	140171813	140171814	MACS_peak_16146	4.00	lobChIP_FOXA1_robot
chr9	140559428	140559429	MACS_peak_16147	5.00	lobChIP_FOXA1_robot
chrX	2959147	2959148	MACS_peak_16148	7.00	lobChIP_FOXA1_robot
chrX	3102366	3102367	MACS_peak_16149	3.00	lobChIP_FOXA1_robot
chrX	3405301	3405302	MACS_peak_16150	5.00	lobChIP_FOXA1_robot
chrX	4749145	4749146	MACS_peak_16151	3.00	lobChIP_FOXA1_robot
chrX	4777397	4777398	MACS_peak_16152	4.00	lobChIP_FOXA1_robot
chrX	4782631	4782632	MACS_peak_16153	6.00	lobChIP_FOXA1_robot
chrX	5588336	5588337	MACS_peak_16154	3.00	lobChIP_FOXA1_robot
chrX	7038702	7038703	MACS_peak_16155	7.00	lobChIP_FOXA1_robot
chrX	7740293	7740294	MACS_peak_16156	4.00	lobChIP_FOXA1_robot
chrX	9235718	9235719	MACS_peak_16157	12.00	lobChIP_FOXA1_robot
chrX	9431179	9431180	MACS_peak_16158	6.00	lobChIP_FOXA1_robot
chrX	9445409	9445410	MACS_peak_16159	11.00	lobChIP_FOXA1_robot
chrX	9757110	9757111	MACS_peak_16160	6.00	lobChIP_FOXA1_robot
chrX	9998742	9998743	MACS_peak_16161	5.00	lobChIP_FOXA1_robot
chrX	10069434	10069435	MACS_peak_16162	23.00	lobChIP_FOXA1_robot
chrX	10125271	10125272	MACS_peak_16163	4.00	lobChIP_FOXA1_robot
chrX	10136492	10136493	MACS_peak_16164	3.00	lobChIP_FOXA1_robot
chrX	10158674	10158675	MACS_peak_16165	3.00	lobChIP_FOXA1_robot
chrX	10918878	10918879	MACS_peak_16166	14.00	lobChIP_FOXA1_robot
chrX	10999836	10999837	MACS_peak_16167	6.00	lobChIP_FOXA1_robot
chrX	11773977	11773978	MACS_peak_16168	13.00	lobChIP_FOXA1_robot
chrX	12862107	12862108	MACS_peak_16169	5.00	lobChIP_FOXA1_robot
chrX	12966125	12966126	MACS_peak_16170	4.00	lobChIP_FOXA1_robot
chrX	15586590	15586591	MACS_peak_16171	6.00	lobChIP_FOXA1_robot
chrX	15762579	15762580	MACS_peak_16172	6.00	lobChIP_FOXA1_robot
chrX	16764021	16764022	MACS_peak_16173	5.00	lobChIP_FOXA1_robot
chrX	16844651	16844652	MACS_peak_16174	3.00	lobChIP_FOXA1_robot
chrX	17465868	17465869	MACS_peak_16175	4.00	lobChIP_FOXA1_robot
chrX	17753396	17753397	MACS_peak_16176	4.00	lobChIP_FOXA1_robot
chrX	18649254	18649255	MACS_peak_16177	8.00	lobChIP_FOXA1_robot
chrX	19264199	19264200	MACS_peak_16178	4.00	lobChIP_FOXA1_robot
chrX	19602110	19602111	MACS_peak_16179	5.00	lobChIP_FOXA1_robot
chrX	20240179	20240180	MACS_peak_16180	8.00	lobChIP_FOXA1_robot
chrX	20288751	20288752	MACS_peak_16181	3.00	lobChIP_FOXA1_robot
chrX	20289493	20289494	MACS_peak_16182	10.00	lobChIP_FOXA1_robot
chrX	20338986	20338987	MACS_peak_16183	15.00	lobChIP_FOXA1_robot
chrX	20379340	20379341	MACS_peak_16184	4.00	lobChIP_FOXA1_robot
chrX	20379858	20379859	MACS_peak_16185	4.00	lobChIP_FOXA1_robot
chrX	20380992	20380993	MACS_peak_16186	8.00	lobChIP_FOXA1_robot
chrX	20381730	20381731	MACS_peak_16187	5.00	lobChIP_FOXA1_robot
chrX	20400415	20400416	MACS_peak_16188	5.00	lobChIP_FOXA1_robot
chrX	20412212	20412213	MACS_peak_16189	10.00	lobChIP_FOXA1_robot
chrX	20489784	20489785	MACS_peak_16190	10.00	lobChIP_FOXA1_robot
chrX	20495543	20495544	MACS_peak_16191	5.00	lobChIP_FOXA1_robot
chrX	20497424	20497425	MACS_peak_16192	13.00	lobChIP_FOXA1_robot
chrX	20498183	20498184	MACS_peak_16193	15.00	lobChIP_FOXA1_robot
chrX	21753239	21753240	MACS_peak_16194	3.00	lobChIP_FOXA1_robot
chrX	23840688	23840689	MACS_peak_16195	7.00	lobChIP_FOXA1_robot
chrX	23860218	23860219	MACS_peak_16196	5.00	lobChIP_FOXA1_robot
chrX	23863772	23863773	MACS_peak_16197	7.00	lobChIP_FOXA1_robot
chrX	23890244	23890245	MACS_peak_16198	6.00	lobChIP_FOXA1_robot
chrX	24166797	24166798	MACS_peak_16199	7.00	lobChIP_FOXA1_robot
chrX	24204077	24204078	MACS_peak_16200	7.00	lobChIP_FOXA1_robot
chrX	24205885	24205886	MACS_peak_16201	9.00	lobChIP_FOXA1_robot
chrX	24207841	24207842	MACS_peak_16202	32.00	lobChIP_FOXA1_robot
chrX	24208699	24208700	MACS_peak_16203	5.00	lobChIP_FOXA1_robot
chrX	25542435	25542436	MACS_peak_16204	6.00	lobChIP_FOXA1_robot
chrX	25554644	25554645	MACS_peak_16205	12.00	lobChIP_FOXA1_robot
chrX	27457457	27457458	MACS_peak_16206	4.00	lobChIP_FOXA1_robot
chrX	30646050	30646051	MACS_peak_16207	23.00	lobChIP_FOXA1_robot
chrX	30661704	30661705	MACS_peak_16208	5.00	lobChIP_FOXA1_robot
chrX	30697795	30697796	MACS_peak_16209	10.00	lobChIP_FOXA1_robot
chrX	30712326	30712327	MACS_peak_16210	4.00	lobChIP_FOXA1_robot
chrX	30853692	30853693	MACS_peak_16211	4.00	lobChIP_FOXA1_robot
chrX	30922415	30922416	MACS_peak_16212	4.00	lobChIP_FOXA1_robot
chrX	30948905	30948906	MACS_peak_16213	11.00	lobChIP_FOXA1_robot
chrX	30979747	30979748	MACS_peak_16214	3.00	lobChIP_FOXA1_robot
chrX	31013435	31013436	MACS_peak_16215	6.00	lobChIP_FOXA1_robot
chrX	31239506	31239507	MACS_peak_16216	3.00	lobChIP_FOXA1_robot
chrX	31368723	31368724	MACS_peak_16217	11.00	lobChIP_FOXA1_robot
chrX	37818726	37818727	MACS_peak_16218	13.00	lobChIP_FOXA1_robot
chrX	38202617	38202618	MACS_peak_16219	7.00	lobChIP_FOXA1_robot
chrX	39842263	39842264	MACS_peak_16220	4.00	lobChIP_FOXA1_robot
chrX	40598161	40598162	MACS_peak_16221	5.00	lobChIP_FOXA1_robot
chrX	40882072	40882073	MACS_peak_16222	4.00	lobChIP_FOXA1_robot
chrX	41682869	41682870	MACS_peak_16223	5.00	lobChIP_FOXA1_robot
chrX	42319200	42319201	MACS_peak_16224	4.00	lobChIP_FOXA1_robot
chrX	43553275	43553276	MACS_peak_16225	15.00	lobChIP_FOXA1_robot
chrX	43556151	43556152	MACS_peak_16226	3.00	lobChIP_FOXA1_robot
chrX	43561602	43561603	MACS_peak_16227	5.00	lobChIP_FOXA1_robot
chrX	44288371	44288372	MACS_peak_16228	7.00	lobChIP_FOXA1_robot
chrX	44552635	44552636	MACS_peak_16229	5.00	lobChIP_FOXA1_robot
chrX	44734045	44734046	MACS_peak_16230	4.00	lobChIP_FOXA1_robot
chrX	44773925	44773926	MACS_peak_16231	4.00	lobChIP_FOXA1_robot
chrX	44774938	44774939	MACS_peak_16232	5.00	lobChIP_FOXA1_robot
chrX	44802388	44802389	MACS_peak_16233	3.00	lobChIP_FOXA1_robot
chrX	45366426	45366427	MACS_peak_16234	4.00	lobChIP_FOXA1_robot
chrX	45374908	45374909	MACS_peak_16235	5.00	lobChIP_FOXA1_robot
chrX	45754916	45754917	MACS_peak_16236	6.00	lobChIP_FOXA1_robot
chrX	46046085	46046086	MACS_peak_16237	4.00	lobChIP_FOXA1_robot
chrX	46080526	46080527	MACS_peak_16238	4.00	lobChIP_FOXA1_robot
chrX	46179583	46179584	MACS_peak_16239	4.00	lobChIP_FOXA1_robot
chrX	46180043	46180044	MACS_peak_16240	7.00	lobChIP_FOXA1_robot
chrX	46672089	46672090	MACS_peak_16241	9.00	lobChIP_FOXA1_robot
chrX	46720227	46720228	MACS_peak_16242	8.00	lobChIP_FOXA1_robot
chrX	46777994	46777995	MACS_peak_16243	12.00	lobChIP_FOXA1_robot
chrX	46918718	46918719	MACS_peak_16244	7.00	lobChIP_FOXA1_robot
chrX	46928306	46928307	MACS_peak_16245	5.00	lobChIP_FOXA1_robot
chrX	46940237	46940238	MACS_peak_16246	15.00	lobChIP_FOXA1_robot
chrX	48571674	48571675	MACS_peak_16247	6.00	lobChIP_FOXA1_robot
chrX	49549574	49549575	MACS_peak_16248	5.00	lobChIP_FOXA1_robot
chrX	49577066	49577067	MACS_peak_16249	13.00	lobChIP_FOXA1_robot
chrX	49626858	49626859	MACS_peak_16250	4.00	lobChIP_FOXA1_robot
chrX	49642125	49642126	MACS_peak_16251	10.00	lobChIP_FOXA1_robot
chrX	49873102	49873103	MACS_peak_16252	6.00	lobChIP_FOXA1_robot
chrX	50174758	50174759	MACS_peak_16253	8.00	lobChIP_FOXA1_robot
chrX	50176307	50176308	MACS_peak_16254	3.00	lobChIP_FOXA1_robot
chrX	53103426	53103427	MACS_peak_16255	8.00	lobChIP_FOXA1_robot
chrX	53410810	53410811	MACS_peak_16256	10.00	lobChIP_FOXA1_robot
chrX	53908963	53908964	MACS_peak_16257	4.00	lobChIP_FOXA1_robot
chrX	53909770	53909771	MACS_peak_16258	5.00	lobChIP_FOXA1_robot
chrX	54072206	54072207	MACS_peak_16259	9.00	lobChIP_FOXA1_robot
chrX	55169020	55169021	MACS_peak_16260	5.00	lobChIP_FOXA1_robot
chrX	57622829	57622830	MACS_peak_16261	4.00	lobChIP_FOXA1_robot
chrX	57670170	57670171	MACS_peak_16262	4.00	lobChIP_FOXA1_robot
chrX	68776227	68776228	MACS_peak_16268	5.00	lobChIP_FOXA1_robot
chrX	69440437	69440438	MACS_peak_16269	40.00	lobChIP_FOXA1_robot
chrX	69475068	69475069	MACS_peak_16270	10.00	lobChIP_FOXA1_robot
chrX	69745726	69745727	MACS_peak_16271	8.00	lobChIP_FOXA1_robot
chrX	69747043	69747044	MACS_peak_16272	7.00	lobChIP_FOXA1_robot
chrX	70613083	70613084	MACS_peak_16273	34.00	lobChIP_FOXA1_robot
chrX	70707871	70707872	MACS_peak_16274	7.00	lobChIP_FOXA1_robot
chrX	73399393	73399394	MACS_peak_16275	3.00	lobChIP_FOXA1_robot
chrX	73462149	73462150	MACS_peak_16276	7.00	lobChIP_FOXA1_robot
chrX	76308300	76308301	MACS_peak_16277	5.00	lobChIP_FOXA1_robot
chrX	77043627	77043628	MACS_peak_16278	5.00	lobChIP_FOXA1_robot
chrX	84747766	84747767	MACS_peak_16279	5.00	lobChIP_FOXA1_robot
chrX	85269045	85269046	MACS_peak_16280	5.00	lobChIP_FOXA1_robot
chrX	85625482	85625483	MACS_peak_16281	5.00	lobChIP_FOXA1_robot
chrX	85626109	85626110	MACS_peak_16282	3.00	lobChIP_FOXA1_robot
chrX	87695154	87695155	MACS_peak_16283	7.00	lobChIP_FOXA1_robot
chrX	87815969	87815970	MACS_peak_16284	11.00	lobChIP_FOXA1_robot
chrX	87859989	87859990	MACS_peak_16285	14.00	lobChIP_FOXA1_robot
chrX	87868627	87868628	MACS_peak_16286	5.00	lobChIP_FOXA1_robot
chrX	90363994	90363995	MACS_peak_16287	13.00	lobChIP_FOXA1_robot
chrX	98439339	98439340	MACS_peak_16288	4.00	lobChIP_FOXA1_robot
chrX	100138761	100138762	MACS_peak_16289	4.00	lobChIP_FOXA1_robot
chrX	103060978	103060979	MACS_peak_16290	6.00	lobChIP_FOXA1_robot
chrX	103061773	103061774	MACS_peak_16291	11.00	lobChIP_FOXA1_robot
chrX	105249844	105249845	MACS_peak_16292	7.00	lobChIP_FOXA1_robot
chrX	105936565	105936566	MACS_peak_16293	13.00	lobChIP_FOXA1_robot
chrX	105959496	105959497	MACS_peak_16294	6.00	lobChIP_FOXA1_robot
chrX	105962156	105962157	MACS_peak_16295	6.00	lobChIP_FOXA1_robot
chrX	106176880	106176881	MACS_peak_16296	5.00	lobChIP_FOXA1_robot
chrX	106198459	106198460	MACS_peak_16297	14.00	lobChIP_FOXA1_robot
chrX	106817694	106817695	MACS_peak_16298	5.00	lobChIP_FOXA1_robot
chrX	107279633	107279634	MACS_peak_16299	4.00	lobChIP_FOXA1_robot
chrX	107504963	107504964	MACS_peak_16300	4.00	lobChIP_FOXA1_robot
chrX	107759662	107759663	MACS_peak_16301	13.00	lobChIP_FOXA1_robot
chrX	108016541	108016542	MACS_peak_16302	3.00	lobChIP_FOXA1_robot
chrX	108952531	108952532	MACS_peak_16304	5.00	lobChIP_FOXA1_robot
chrX	108983432	108983433	MACS_peak_16305	40.00	lobChIP_FOXA1_robot
chrX	109066341	109066342	MACS_peak_16306	7.00	lobChIP_FOXA1_robot
chrX	109074194	109074195	MACS_peak_16307	18.00	lobChIP_FOXA1_robot
chrX	109087477	109087478	MACS_peak_16308	4.00	lobChIP_FOXA1_robot
chrX	109102463	109102464	MACS_peak_16309	4.00	lobChIP_FOXA1_robot
chrX	109115562	109115563	MACS_peak_16310	9.00	lobChIP_FOXA1_robot
chrX	109279864	109279865	MACS_peak_16311	14.00	lobChIP_FOXA1_robot
chrX	109329667	109329668	MACS_peak_16312	10.00	lobChIP_FOXA1_robot
chrX	117103833	117103834	MACS_peak_16313	4.00	lobChIP_FOXA1_robot
chrX	117290512	117290513	MACS_peak_16314	4.00	lobChIP_FOXA1_robot
chrX	117631300	117631301	MACS_peak_16315	3.00	lobChIP_FOXA1_robot
chrX	117800637	117800638	MACS_peak_16316	5.00	lobChIP_FOXA1_robot
chrX	117860997	117860998	MACS_peak_16317	10.00	lobChIP_FOXA1_robot
chrX	118523354	118523355	MACS_peak_16318	5.00	lobChIP_FOXA1_robot
chrX	118639997	118639998	MACS_peak_16319	11.00	lobChIP_FOXA1_robot
chrX	119077761	119077762	MACS_peak_16320	6.00	lobChIP_FOXA1_robot
chrX	119379184	119379185	MACS_peak_16321	14.00	lobChIP_FOXA1_robot
chrX	119683960	119683961	MACS_peak_16322	4.00	lobChIP_FOXA1_robot
chrX	119889173	119889174	MACS_peak_16323	5.00	lobChIP_FOXA1_robot
chrX	120379769	120379770	MACS_peak_16324	9.00	lobChIP_FOXA1_robot
chrX	121949701	121949702	MACS_peak_16325	3.00	lobChIP_FOXA1_robot
chrX	122227885	122227886	MACS_peak_16326	5.00	lobChIP_FOXA1_robot
chrX	122913931	122913932	MACS_peak_16327	4.00	lobChIP_FOXA1_robot
chrX	122937571	122937572	MACS_peak_16328	8.00	lobChIP_FOXA1_robot
chrX	124406984	124406985	MACS_peak_16329	4.00	lobChIP_FOXA1_robot
chrX	124408751	124408752	MACS_peak_16330	4.00	lobChIP_FOXA1_robot
chrX	124410098	124410099	MACS_peak_16331	4.00	lobChIP_FOXA1_robot
chrX	127888423	127888424	MACS_peak_16332	7.00	lobChIP_FOXA1_robot
chrX	127893891	127893892	MACS_peak_16333	10.00	lobChIP_FOXA1_robot
chrX	128063388	128063389	MACS_peak_16334	5.00	lobChIP_FOXA1_robot
chrX	128134077	128134078	MACS_peak_16335	7.00	lobChIP_FOXA1_robot
chrX	128257940	128257941	MACS_peak_16336	6.00	lobChIP_FOXA1_robot
chrX	128259639	128259640	MACS_peak_16337	5.00	lobChIP_FOXA1_robot
chrX	128274056	128274057	MACS_peak_16338	18.00	lobChIP_FOXA1_robot
chrX	128278849	128278850	MACS_peak_16339	13.00	lobChIP_FOXA1_robot
chrX	128280102	128280103	MACS_peak_16340	3.00	lobChIP_FOXA1_robot
chrX	128300626	128300627	MACS_peak_16341	6.00	lobChIP_FOXA1_robot
chrX	128659270	128659271	MACS_peak_16342	18.00	lobChIP_FOXA1_robot
chrX	128675053	128675054	MACS_peak_16343	4.00	lobChIP_FOXA1_robot
chrX	128719953	128719954	MACS_peak_16344	4.00	lobChIP_FOXA1_robot
chrX	128832359	128832360	MACS_peak_16345	7.00	lobChIP_FOXA1_robot
chrX	129649389	129649390	MACS_peak_16346	3.00	lobChIP_FOXA1_robot
chrX	130421753	130421754	MACS_peak_16347	6.00	lobChIP_FOXA1_robot
chrX	131232977	131232978	MACS_peak_16348	5.00	lobChIP_FOXA1_robot
chrX	131465223	131465224	MACS_peak_16349	25.00	lobChIP_FOXA1_robot
chrX	131502161	131502162	MACS_peak_16350	14.00	lobChIP_FOXA1_robot
chrX	131545652	131545653	MACS_peak_16351	4.00	lobChIP_FOXA1_robot
chrX	131560243	131560244	MACS_peak_16352	4.00	lobChIP_FOXA1_robot
chrX	131614870	131614871	MACS_peak_16353	5.00	lobChIP_FOXA1_robot
chrX	131616878	131616879	MACS_peak_16354	17.00	lobChIP_FOXA1_robot
chrX	131625475	131625476	MACS_peak_16355	7.00	lobChIP_FOXA1_robot
chrX	131627428	131627429	MACS_peak_16356	12.00	lobChIP_FOXA1_robot
chrX	131654457	131654458	MACS_peak_16357	8.00	lobChIP_FOXA1_robot
chrX	131667103	131667104	MACS_peak_16358	7.00	lobChIP_FOXA1_robot
chrX	131696200	131696201	MACS_peak_16359	7.00	lobChIP_FOXA1_robot
chrX	131724701	131724702	MACS_peak_16360	13.00	lobChIP_FOXA1_robot
chrX	131749389	131749390	MACS_peak_16361	5.00	lobChIP_FOXA1_robot
chrX	132101240	132101241	MACS_peak_16362	7.00	lobChIP_FOXA1_robot
chrX	133121502	133121503	MACS_peak_16363	8.00	lobChIP_FOXA1_robot
chrX	133128808	133128809	MACS_peak_16364	6.00	lobChIP_FOXA1_robot
chrX	133140995	133140996	MACS_peak_16365	21.00	lobChIP_FOXA1_robot
chrX	133156916	133156917	MACS_peak_16366	8.00	lobChIP_FOXA1_robot
chrX	133158123	133158124	MACS_peak_16367	13.00	lobChIP_FOXA1_robot
chrX	133597121	133597122	MACS_peak_16368	16.00	lobChIP_FOXA1_robot
chrX	134082996	134082997	MACS_peak_16369	9.00	lobChIP_FOXA1_robot
chrX	134085573	134085574	MACS_peak_16370	7.00	lobChIP_FOXA1_robot
chrX	135578709	135578710	MACS_peak_16371	7.00	lobChIP_FOXA1_robot
chrX	139755521	139755522	MACS_peak_16372	6.00	lobChIP_FOXA1_robot
chrX	148801753	148801754	MACS_peak_16373	9.00	lobChIP_FOXA1_robot
chrX	149739048	149739049	MACS_peak_16374	7.00	lobChIP_FOXA1_robot
chrX	149975440	149975441	MACS_peak_16375	5.00	lobChIP_FOXA1_robot
chrX	151155665	151155666	MACS_peak_16376	5.00	lobChIP_FOXA1_robot
chrX	152911537	152911538	MACS_peak_16377	5.00	lobChIP_FOXA1_robot
chrX	153769361	153769362	MACS_peak_16378	5.00	lobChIP_FOXA1_robot
chrX	153770266	153770267	MACS_peak_16379	5.00	lobChIP_FOXA1_robot
chrX	154028265	154028266	MACS_peak_16380	9.00	lobChIP_FOXA1_robot
chrX	154216411	154216412	MACS_peak_16381	15.00	lobChIP_FOXA1_robot
chrX	154254868	154254869	MACS_peak_16382	7.00	lobChIP_FOXA1_robot
chrX	154296708	154296709	MACS_peak_16383	4.00	lobChIP_FOXA1_robot
chrX	154464476	154464477	MACS_peak_16384	4.00	lobChIP_FOXA1_robot
chrY	7178766	7178767	MACS_peak_16385	7.00	lobChIP_FOXA1_robot
chrY	8898207	8898208	MACS_peak_16386	6.00	lobChIP_FOXA1_robot
chrY	9960247	9960248	MACS_peak_16387	5.00	lobChIP_FOXA1_robot
chrY	9994816	9994817	MACS_peak_16388	4.00	lobChIP_FOXA1_robot
chrY	10020623	10020624	MACS_peak_16389	5.00	lobChIP_FOXA1_robot
chrY	13137840	13137841	MACS_peak_16392	8.00	lobChIP_FOXA1_robot
chrY	16745789	16745790	MACS_peak_16397	6.00	lobChIP_FOXA1_robot
chrY	59012018	59012019	MACS_peak_16398	5.00	lobChIP_FOXA1_robot
chr1	1060287	1060288	MACS_peak_1	19.00	lobChIP_FOXA1inPBS
chr1	1356753	1356754	MACS_peak_2	14.00	lobChIP_FOXA1inPBS
chr1	1365282	1365283	MACS_peak_3	11.00	lobChIP_FOXA1inPBS
chr1	1714564	1714565	MACS_peak_4	13.00	lobChIP_FOXA1inPBS
chr1	1765450	1765451	MACS_peak_5	8.00	lobChIP_FOXA1inPBS
chr1	1790838	1790839	MACS_peak_6	11.00	lobChIP_FOXA1inPBS
chr1	2066298	2066299	MACS_peak_7	16.00	lobChIP_FOXA1inPBS
chr1	2121595	2121596	MACS_peak_8	11.00	lobChIP_FOXA1inPBS
chr1	2151555	2151556	MACS_peak_9	12.00	lobChIP_FOXA1inPBS
chr1	2164248	2164249	MACS_peak_10	19.00	lobChIP_FOXA1inPBS
chr1	3154851	3154852	MACS_peak_11	13.00	lobChIP_FOXA1inPBS
chr1	3178121	3178122	MACS_peak_12	8.00	lobChIP_FOXA1inPBS
chr1	3541356	3541357	MACS_peak_13	14.00	lobChIP_FOXA1inPBS
chr1	4003054	4003055	MACS_peak_14	23.00	lobChIP_FOXA1inPBS
chr1	4771149	4771150	MACS_peak_15	10.00	lobChIP_FOXA1inPBS
chr1	5658264	5658265	MACS_peak_16	17.00	lobChIP_FOXA1inPBS
chr1	5991042	5991043	MACS_peak_17	6.00	lobChIP_FOXA1inPBS
chr1	6009348	6009349	MACS_peak_18	13.00	lobChIP_FOXA1inPBS
chr1	6015745	6015746	MACS_peak_19	12.00	lobChIP_FOXA1inPBS
chr1	6300358	6300359	MACS_peak_20	19.00	lobChIP_FOXA1inPBS
chr1	6355455	6355456	MACS_peak_21	10.00	lobChIP_FOXA1inPBS
chr1	6473103	6473104	MACS_peak_22	16.00	lobChIP_FOXA1inPBS
chr1	6642616	6642617	MACS_peak_23	18.00	lobChIP_FOXA1inPBS
chr1	6920380	6920381	MACS_peak_24	7.00	lobChIP_FOXA1inPBS
chr1	6970104	6970105	MACS_peak_25	16.00	lobChIP_FOXA1inPBS
chr1	7551354	7551355	MACS_peak_26	8.00	lobChIP_FOXA1inPBS
chr1	7711036	7711037	MACS_peak_27	7.00	lobChIP_FOXA1inPBS
chr1	7812713	7812714	MACS_peak_28	8.00	lobChIP_FOXA1inPBS
chr1	7814510	7814511	MACS_peak_29	7.00	lobChIP_FOXA1inPBS
chr1	7998228	7998229	MACS_peak_30	5.00	lobChIP_FOXA1inPBS
chr1	8066557	8066558	MACS_peak_31	11.00	lobChIP_FOXA1inPBS
chr1	8087617	8087618	MACS_peak_32	18.00	lobChIP_FOXA1inPBS
chr1	8100792	8100793	MACS_peak_33	12.00	lobChIP_FOXA1inPBS
chr1	8121490	8121491	MACS_peak_34	28.00	lobChIP_FOXA1inPBS
chr1	8144441	8144442	MACS_peak_35	41.00	lobChIP_FOXA1inPBS
chr1	8155144	8155145	MACS_peak_36	14.00	lobChIP_FOXA1inPBS
chr1	8177186	8177187	MACS_peak_37	24.00	lobChIP_FOXA1inPBS
chr1	8181052	8181053	MACS_peak_38	9.00	lobChIP_FOXA1inPBS
chr1	8214863	8214864	MACS_peak_39	8.00	lobChIP_FOXA1inPBS
chr1	8215658	8215659	MACS_peak_40	12.00	lobChIP_FOXA1inPBS
chr1	8230399	8230400	MACS_peak_41	10.00	lobChIP_FOXA1inPBS
chr1	8242626	8242627	MACS_peak_42	12.00	lobChIP_FOXA1inPBS
chr1	8250282	8250283	MACS_peak_43	17.00	lobChIP_FOXA1inPBS
chr1	8254828	8254829	MACS_peak_44	14.00	lobChIP_FOXA1inPBS
chr1	8260501	8260502	MACS_peak_45	34.00	lobChIP_FOXA1inPBS
chr1	8272293	8272294	MACS_peak_46	9.00	lobChIP_FOXA1inPBS
chr1	8319296	8319297	MACS_peak_47	55.00	lobChIP_FOXA1inPBS
chr1	8372415	8372416	MACS_peak_48	7.00	lobChIP_FOXA1inPBS
chr1	8438452	8438453	MACS_peak_49	7.00	lobChIP_FOXA1inPBS
chr1	8533740	8533741	MACS_peak_50	6.00	lobChIP_FOXA1inPBS
chr1	8631818	8631819	MACS_peak_51	16.00	lobChIP_FOXA1inPBS
chr1	8689521	8689522	MACS_peak_52	14.00	lobChIP_FOXA1inPBS
chr1	8704121	8704122	MACS_peak_53	14.00	lobChIP_FOXA1inPBS
chr1	8730526	8730527	MACS_peak_54	13.00	lobChIP_FOXA1inPBS
chr1	8741620	8741621	MACS_peak_55	12.00	lobChIP_FOXA1inPBS
chr1	8762478	8762479	MACS_peak_56	16.00	lobChIP_FOXA1inPBS
chr1	8766536	8766537	MACS_peak_57	10.00	lobChIP_FOXA1inPBS
chr1	8772939	8772940	MACS_peak_58	13.00	lobChIP_FOXA1inPBS
chr1	8777439	8777440	MACS_peak_59	12.00	lobChIP_FOXA1inPBS
chr1	8800369	8800370	MACS_peak_60	18.00	lobChIP_FOXA1inPBS
chr1	8802242	8802243	MACS_peak_61	137.00	lobChIP_FOXA1inPBS
chr1	8936057	8936058	MACS_peak_62	5.00	lobChIP_FOXA1inPBS
chr1	8940292	8940293	MACS_peak_63	16.00	lobChIP_FOXA1inPBS
chr1	9062023	9062024	MACS_peak_64	7.00	lobChIP_FOXA1inPBS
chr1	9118710	9118711	MACS_peak_65	8.00	lobChIP_FOXA1inPBS
chr1	9173342	9173343	MACS_peak_66	22.00	lobChIP_FOXA1inPBS
chr1	9232354	9232355	MACS_peak_67	15.00	lobChIP_FOXA1inPBS
chr1	9254291	9254292	MACS_peak_68	36.00	lobChIP_FOXA1inPBS
chr1	9391984	9391985	MACS_peak_69	17.00	lobChIP_FOXA1inPBS
chr1	9495393	9495394	MACS_peak_70	50.00	lobChIP_FOXA1inPBS
chr1	9496607	9496608	MACS_peak_71	7.00	lobChIP_FOXA1inPBS
chr1	9498654	9498655	MACS_peak_72	13.00	lobChIP_FOXA1inPBS
chr1	9499558	9499559	MACS_peak_73	12.00	lobChIP_FOXA1inPBS
chr1	9596667	9596668	MACS_peak_74	9.00	lobChIP_FOXA1inPBS
chr1	9598125	9598126	MACS_peak_75	20.00	lobChIP_FOXA1inPBS
chr1	9610438	9610439	MACS_peak_76	54.00	lobChIP_FOXA1inPBS
chr1	9614104	9614105	MACS_peak_77	25.00	lobChIP_FOXA1inPBS
chr1	9876490	9876491	MACS_peak_78	8.00	lobChIP_FOXA1inPBS
chr1	9974592	9974593	MACS_peak_79	18.00	lobChIP_FOXA1inPBS
chr1	9989659	9989660	MACS_peak_80	13.00	lobChIP_FOXA1inPBS
chr1	10116469	10116470	MACS_peak_81	24.00	lobChIP_FOXA1inPBS
chr1	10229326	10229327	MACS_peak_82	13.00	lobChIP_FOXA1inPBS
chr1	10231339	10231340	MACS_peak_83	13.00	lobChIP_FOXA1inPBS
chr1	10233072	10233073	MACS_peak_84	9.00	lobChIP_FOXA1inPBS
chr1	10550041	10550042	MACS_peak_85	21.00	lobChIP_FOXA1inPBS
chr1	10659810	10659811	MACS_peak_86	21.00	lobChIP_FOXA1inPBS
chr1	10764791	10764792	MACS_peak_87	14.00	lobChIP_FOXA1inPBS
chr1	10997585	10997586	MACS_peak_88	8.00	lobChIP_FOXA1inPBS
chr1	11327963	11327964	MACS_peak_89	11.00	lobChIP_FOXA1inPBS
chr1	11330004	11330005	MACS_peak_90	9.00	lobChIP_FOXA1inPBS
chr1	11968310	11968311	MACS_peak_91	13.00	lobChIP_FOXA1inPBS
chr1	11969806	11969807	MACS_peak_92	10.00	lobChIP_FOXA1inPBS
chr1	11988086	11988087	MACS_peak_93	9.00	lobChIP_FOXA1inPBS
chr1	12407071	12407072	MACS_peak_94	8.00	lobChIP_FOXA1inPBS
chr1	12416199	12416200	MACS_peak_95	12.00	lobChIP_FOXA1inPBS
chr1	12440516	12440517	MACS_peak_96	7.00	lobChIP_FOXA1inPBS
chr1	12441401	12441402	MACS_peak_97	46.00	lobChIP_FOXA1inPBS
chr1	12462492	12462493	MACS_peak_98	64.00	lobChIP_FOXA1inPBS
chr1	12465716	12465717	MACS_peak_99	9.00	lobChIP_FOXA1inPBS
chr1	12497171	12497172	MACS_peak_100	12.00	lobChIP_FOXA1inPBS
chr1	12536064	12536065	MACS_peak_101	13.00	lobChIP_FOXA1inPBS
chr1	12538297	12538298	MACS_peak_102	8.00	lobChIP_FOXA1inPBS
chr1	12585564	12585565	MACS_peak_103	7.00	lobChIP_FOXA1inPBS
chr1	12588506	12588507	MACS_peak_104	44.00	lobChIP_FOXA1inPBS
chr1	12589613	12589614	MACS_peak_105	17.00	lobChIP_FOXA1inPBS
chr1	12609991	12609992	MACS_peak_106	12.00	lobChIP_FOXA1inPBS
chr1	12664575	12664576	MACS_peak_107	20.00	lobChIP_FOXA1inPBS
chr1	12668755	12668756	MACS_peak_108	16.00	lobChIP_FOXA1inPBS
chr1	12674609	12674610	MACS_peak_109	11.00	lobChIP_FOXA1inPBS
chr1	12676132	12676133	MACS_peak_110	51.00	lobChIP_FOXA1inPBS
chr1	12714871	12714872	MACS_peak_111	32.00	lobChIP_FOXA1inPBS
chr1	13977945	13977946	MACS_peak_112	13.00	lobChIP_FOXA1inPBS
chr1	14862310	14862311	MACS_peak_113	16.00	lobChIP_FOXA1inPBS
chr1	15026141	15026142	MACS_peak_114	7.00	lobChIP_FOXA1inPBS
chr1	15279438	15279439	MACS_peak_115	8.00	lobChIP_FOXA1inPBS
chr1	15284857	15284858	MACS_peak_116	7.00	lobChIP_FOXA1inPBS
chr1	15347938	15347939	MACS_peak_117	13.00	lobChIP_FOXA1inPBS
chr1	15466271	15466272	MACS_peak_118	5.00	lobChIP_FOXA1inPBS
chr1	15472138	15472139	MACS_peak_119	16.00	lobChIP_FOXA1inPBS
chr1	15508141	15508142	MACS_peak_120	8.00	lobChIP_FOXA1inPBS
chr1	15516459	15516460	MACS_peak_121	11.00	lobChIP_FOXA1inPBS
chr1	15524558	15524559	MACS_peak_122	10.00	lobChIP_FOXA1inPBS
chr1	15611683	15611684	MACS_peak_123	42.00	lobChIP_FOXA1inPBS
chr1	15679078	15679079	MACS_peak_124	8.00	lobChIP_FOXA1inPBS
chr1	15680590	15680591	MACS_peak_125	9.00	lobChIP_FOXA1inPBS
chr1	15720919	15720920	MACS_peak_126	7.00	lobChIP_FOXA1inPBS
chr1	15911793	15911794	MACS_peak_127	8.00	lobChIP_FOXA1inPBS
chr1	15928164	15928165	MACS_peak_128	9.00	lobChIP_FOXA1inPBS
chr1	15932384	15932385	MACS_peak_129	9.00	lobChIP_FOXA1inPBS
chr1	15936830	15936831	MACS_peak_130	11.00	lobChIP_FOXA1inPBS
chr1	15942511	15942512	MACS_peak_131	14.00	lobChIP_FOXA1inPBS
chr1	15945370	15945371	MACS_peak_132	21.00	lobChIP_FOXA1inPBS
chr1	15946994	15946995	MACS_peak_133	14.00	lobChIP_FOXA1inPBS
chr1	16107377	16107378	MACS_peak_134	8.00	lobChIP_FOXA1inPBS
chr1	16158685	16158686	MACS_peak_135	21.00	lobChIP_FOXA1inPBS
chr1	16177066	16177067	MACS_peak_136	11.00	lobChIP_FOXA1inPBS
chr1	16305785	16305786	MACS_peak_137	9.00	lobChIP_FOXA1inPBS
chr1	16330600	16330601	MACS_peak_138	30.00	lobChIP_FOXA1inPBS
chr1	16447739	16447740	MACS_peak_139	26.00	lobChIP_FOXA1inPBS
chr1	16470580	16470581	MACS_peak_140	10.00	lobChIP_FOXA1inPBS
chr1	16490856	16490857	MACS_peak_141	8.00	lobChIP_FOXA1inPBS
chr1	16661367	16661368	MACS_peak_142	11.00	lobChIP_FOXA1inPBS
chr1	16693559	16693560	MACS_peak_143	12.00	lobChIP_FOXA1inPBS
chr1	16699271	16699272	MACS_peak_144	7.00	lobChIP_FOXA1inPBS
chr1	17029866	17029867	MACS_peak_145	8.00	lobChIP_FOXA1inPBS
chr1	17766106	17766107	MACS_peak_146	8.00	lobChIP_FOXA1inPBS
chr1	17824789	17824790	MACS_peak_147	16.00	lobChIP_FOXA1inPBS
chr1	17891322	17891323	MACS_peak_148	6.00	lobChIP_FOXA1inPBS
chr1	17908150	17908151	MACS_peak_149	36.00	lobChIP_FOXA1inPBS
chr1	17918707	17918708	MACS_peak_150	21.00	lobChIP_FOXA1inPBS
chr1	18791031	18791032	MACS_peak_151	12.00	lobChIP_FOXA1inPBS
chr1	18813413	18813414	MACS_peak_152	10.00	lobChIP_FOXA1inPBS
chr1	18841989	18841990	MACS_peak_153	8.00	lobChIP_FOXA1inPBS
chr1	19275638	19275639	MACS_peak_154	10.00	lobChIP_FOXA1inPBS
chr1	19315503	19315504	MACS_peak_155	15.00	lobChIP_FOXA1inPBS
chr1	19465873	19465874	MACS_peak_156	12.00	lobChIP_FOXA1inPBS
chr1	19549701	19549702	MACS_peak_157	15.00	lobChIP_FOXA1inPBS
chr1	19596534	19596535	MACS_peak_158	10.00	lobChIP_FOXA1inPBS
chr1	19736377	19736378	MACS_peak_159	39.00	lobChIP_FOXA1inPBS
chr1	19751450	19751451	MACS_peak_160	11.00	lobChIP_FOXA1inPBS
chr1	19752329	19752330	MACS_peak_161	9.00	lobChIP_FOXA1inPBS
chr1	19753869	19753870	MACS_peak_162	9.00	lobChIP_FOXA1inPBS
chr1	19756886	19756887	MACS_peak_163	15.00	lobChIP_FOXA1inPBS
chr1	19831431	19831432	MACS_peak_164	10.00	lobChIP_FOXA1inPBS
chr1	19874512	19874513	MACS_peak_165	19.00	lobChIP_FOXA1inPBS
chr1	19946497	19946498	MACS_peak_166	32.00	lobChIP_FOXA1inPBS
chr1	20301719	20301720	MACS_peak_167	16.00	lobChIP_FOXA1inPBS
chr1	20306932	20306933	MACS_peak_168	8.00	lobChIP_FOXA1inPBS
chr1	20314373	20314374	MACS_peak_169	52.00	lobChIP_FOXA1inPBS
chr1	20864941	20864942	MACS_peak_170	10.00	lobChIP_FOXA1inPBS
chr1	20914550	20914551	MACS_peak_171	6.00	lobChIP_FOXA1inPBS
chr1	21016218	21016219	MACS_peak_172	8.00	lobChIP_FOXA1inPBS
chr1	21111381	21111382	MACS_peak_173	15.00	lobChIP_FOXA1inPBS
chr1	21140857	21140858	MACS_peak_174	8.00	lobChIP_FOXA1inPBS
chr1	21223471	21223472	MACS_peak_175	11.00	lobChIP_FOXA1inPBS
chr1	21451510	21451511	MACS_peak_176	6.00	lobChIP_FOXA1inPBS
chr1	21452909	21452910	MACS_peak_177	6.00	lobChIP_FOXA1inPBS
chr1	21578567	21578568	MACS_peak_178	31.00	lobChIP_FOXA1inPBS
chr1	21603079	21603080	MACS_peak_179	13.00	lobChIP_FOXA1inPBS
chr1	21646291	21646292	MACS_peak_180	19.00	lobChIP_FOXA1inPBS
chr1	21653384	21653385	MACS_peak_181	6.00	lobChIP_FOXA1inPBS
chr1	21665350	21665351	MACS_peak_182	35.00	lobChIP_FOXA1inPBS
chr1	21950198	21950199	MACS_peak_183	6.00	lobChIP_FOXA1inPBS
chr1	21993527	21993528	MACS_peak_184	10.00	lobChIP_FOXA1inPBS
chr1	22032247	22032248	MACS_peak_185	23.00	lobChIP_FOXA1inPBS
chr1	22102693	22102694	MACS_peak_186	50.00	lobChIP_FOXA1inPBS
chr1	22182562	22182563	MACS_peak_187	11.00	lobChIP_FOXA1inPBS
chr1	22257750	22257751	MACS_peak_188	17.00	lobChIP_FOXA1inPBS
chr1	22299668	22299669	MACS_peak_189	8.00	lobChIP_FOXA1inPBS
chr1	22324551	22324552	MACS_peak_190	11.00	lobChIP_FOXA1inPBS
chr1	22470578	22470579	MACS_peak_191	12.00	lobChIP_FOXA1inPBS
chr1	22675095	22675096	MACS_peak_192	54.00	lobChIP_FOXA1inPBS
chr1	22739386	22739387	MACS_peak_193	9.00	lobChIP_FOXA1inPBS
chr1	22740852	22740853	MACS_peak_194	51.00	lobChIP_FOXA1inPBS
chr1	23007364	23007365	MACS_peak_195	7.00	lobChIP_FOXA1inPBS
chr1	23441754	23441755	MACS_peak_196	29.00	lobChIP_FOXA1inPBS
chr1	23475703	23475704	MACS_peak_197	7.00	lobChIP_FOXA1inPBS
chr1	23514466	23514467	MACS_peak_198	17.00	lobChIP_FOXA1inPBS
chr1	23704089	23704090	MACS_peak_199	9.00	lobChIP_FOXA1inPBS
chr1	23785929	23785930	MACS_peak_200	15.00	lobChIP_FOXA1inPBS
chr1	23787841	23787842	MACS_peak_201	10.00	lobChIP_FOXA1inPBS
chr1	23805504	23805505	MACS_peak_202	14.00	lobChIP_FOXA1inPBS
chr1	23824489	23824490	MACS_peak_203	12.00	lobChIP_FOXA1inPBS
chr1	23888928	23888929	MACS_peak_204	33.00	lobChIP_FOXA1inPBS
chr1	23890612	23890613	MACS_peak_205	12.00	lobChIP_FOXA1inPBS
chr1	23918114	23918115	MACS_peak_206	20.00	lobChIP_FOXA1inPBS
chr1	23918712	23918713	MACS_peak_207	9.00	lobChIP_FOXA1inPBS
chr1	24054107	24054108	MACS_peak_208	10.00	lobChIP_FOXA1inPBS
chr1	24092455	24092456	MACS_peak_209	20.00	lobChIP_FOXA1inPBS
chr1	24279624	24279625	MACS_peak_210	12.00	lobChIP_FOXA1inPBS
chr1	24300298	24300299	MACS_peak_211	8.00	lobChIP_FOXA1inPBS
chr1	24345490	24345491	MACS_peak_212	7.00	lobChIP_FOXA1inPBS
chr1	24348159	24348160	MACS_peak_213	19.00	lobChIP_FOXA1inPBS
chr1	24441421	24441422	MACS_peak_214	16.00	lobChIP_FOXA1inPBS
chr1	24446448	24446449	MACS_peak_215	7.00	lobChIP_FOXA1inPBS
chr1	24867702	24867703	MACS_peak_216	51.00	lobChIP_FOXA1inPBS
chr1	24878810	24878811	MACS_peak_217	9.00	lobChIP_FOXA1inPBS
chr1	24885861	24885862	MACS_peak_218	13.00	lobChIP_FOXA1inPBS
chr1	24894689	24894690	MACS_peak_219	20.00	lobChIP_FOXA1inPBS
chr1	25032384	25032385	MACS_peak_220	20.00	lobChIP_FOXA1inPBS
chr1	25094093	25094094	MACS_peak_221	26.00	lobChIP_FOXA1inPBS
chr1	25151928	25151929	MACS_peak_222	18.00	lobChIP_FOXA1inPBS
chr1	25237830	25237831	MACS_peak_223	8.00	lobChIP_FOXA1inPBS
chr1	25495131	25495132	MACS_peak_224	55.00	lobChIP_FOXA1inPBS
chr1	25501013	25501014	MACS_peak_225	15.00	lobChIP_FOXA1inPBS
chr1	25523925	25523926	MACS_peak_226	10.00	lobChIP_FOXA1inPBS
chr1	25758819	25758820	MACS_peak_227	7.00	lobChIP_FOXA1inPBS
chr1	25827791	25827792	MACS_peak_228	23.00	lobChIP_FOXA1inPBS
chr1	25851609	25851610	MACS_peak_229	8.00	lobChIP_FOXA1inPBS
chr1	25927710	25927711	MACS_peak_230	8.00	lobChIP_FOXA1inPBS
chr1	26187865	26187866	MACS_peak_231	10.00	lobChIP_FOXA1inPBS
chr1	26217017	26217018	MACS_peak_232	7.00	lobChIP_FOXA1inPBS
chr1	26231380	26231381	MACS_peak_233	7.00	lobChIP_FOXA1inPBS
chr1	26556966	26556967	MACS_peak_234	7.00	lobChIP_FOXA1inPBS
chr1	27033036	27033037	MACS_peak_235	9.00	lobChIP_FOXA1inPBS
chr1	27110888	27110889	MACS_peak_236	11.00	lobChIP_FOXA1inPBS
chr1	27158434	27158435	MACS_peak_237	30.00	lobChIP_FOXA1inPBS
chr1	27240773	27240774	MACS_peak_238	22.00	lobChIP_FOXA1inPBS
chr1	27241686	27241687	MACS_peak_239	8.00	lobChIP_FOXA1inPBS
chr1	27317204	27317205	MACS_peak_240	8.00	lobChIP_FOXA1inPBS
chr1	27330451	27330452	MACS_peak_241	7.00	lobChIP_FOXA1inPBS
chr1	27354236	27354237	MACS_peak_242	9.00	lobChIP_FOXA1inPBS
chr1	27518628	27518629	MACS_peak_243	9.00	lobChIP_FOXA1inPBS
chr1	27754819	27754820	MACS_peak_244	15.00	lobChIP_FOXA1inPBS
chr1	27816894	27816895	MACS_peak_245	7.00	lobChIP_FOXA1inPBS
chr1	27868987	27868988	MACS_peak_246	17.00	lobChIP_FOXA1inPBS
chr1	28270052	28270053	MACS_peak_247	16.00	lobChIP_FOXA1inPBS
chr1	28306251	28306252	MACS_peak_248	15.00	lobChIP_FOXA1inPBS
chr1	28479805	28479806	MACS_peak_249	17.00	lobChIP_FOXA1inPBS
chr1	28496819	28496820	MACS_peak_250	10.00	lobChIP_FOXA1inPBS
chr1	28622618	28622619	MACS_peak_251	18.00	lobChIP_FOXA1inPBS
chr1	28673019	28673020	MACS_peak_252	6.00	lobChIP_FOXA1inPBS
chr1	28678297	28678298	MACS_peak_253	7.00	lobChIP_FOXA1inPBS
chr1	28834844	28834845	MACS_peak_254	9.00	lobChIP_FOXA1inPBS
chr1	28845529	28845530	MACS_peak_255	11.00	lobChIP_FOXA1inPBS
chr1	28961045	28961046	MACS_peak_256	67.00	lobChIP_FOXA1inPBS
chr1	28975276	28975277	MACS_peak_257	8.00	lobChIP_FOXA1inPBS
chr1	29245881	29245882	MACS_peak_258	13.00	lobChIP_FOXA1inPBS
chr1	29432576	29432577	MACS_peak_259	14.00	lobChIP_FOXA1inPBS
chr1	29472668	29472669	MACS_peak_260	11.00	lobChIP_FOXA1inPBS
chr1	29512948	29512949	MACS_peak_261	18.00	lobChIP_FOXA1inPBS
chr1	29598427	29598428	MACS_peak_262	9.00	lobChIP_FOXA1inPBS
chr1	29684865	29684866	MACS_peak_263	11.00	lobChIP_FOXA1inPBS
chr1	30702591	30702592	MACS_peak_264	13.00	lobChIP_FOXA1inPBS
chr1	31537066	31537067	MACS_peak_265	34.00	lobChIP_FOXA1inPBS
chr1	31642861	31642862	MACS_peak_266	16.00	lobChIP_FOXA1inPBS
chr1	31643696	31643697	MACS_peak_267	11.00	lobChIP_FOXA1inPBS
chr1	31767827	31767828	MACS_peak_268	12.00	lobChIP_FOXA1inPBS
chr1	31920268	31920269	MACS_peak_269	37.00	lobChIP_FOXA1inPBS
chr1	31922739	31922740	MACS_peak_270	13.00	lobChIP_FOXA1inPBS
chr1	31975019	31975020	MACS_peak_271	9.00	lobChIP_FOXA1inPBS
chr1	31977738	31977739	MACS_peak_272	10.00	lobChIP_FOXA1inPBS
chr1	32321135	32321136	MACS_peak_273	8.00	lobChIP_FOXA1inPBS
chr1	32376700	32376701	MACS_peak_274	15.00	lobChIP_FOXA1inPBS
chr1	32644133	32644134	MACS_peak_275	23.00	lobChIP_FOXA1inPBS
chr1	32721056	32721057	MACS_peak_276	9.00	lobChIP_FOXA1inPBS
chr1	32775712	32775713	MACS_peak_277	11.00	lobChIP_FOXA1inPBS
chr1	32787478	32787479	MACS_peak_278	60.00	lobChIP_FOXA1inPBS
chr1	32860776	32860777	MACS_peak_279	7.00	lobChIP_FOXA1inPBS
chr1	33180157	33180158	MACS_peak_280	25.00	lobChIP_FOXA1inPBS
chr1	33671594	33671595	MACS_peak_281	8.00	lobChIP_FOXA1inPBS
chr1	33722566	33722567	MACS_peak_282	19.00	lobChIP_FOXA1inPBS
chr1	33724623	33724624	MACS_peak_283	31.00	lobChIP_FOXA1inPBS
chr1	33808065	33808066	MACS_peak_284	26.00	lobChIP_FOXA1inPBS
chr1	33813040	33813041	MACS_peak_285	24.00	lobChIP_FOXA1inPBS
chr1	34579748	34579749	MACS_peak_286	13.00	lobChIP_FOXA1inPBS
chr1	35048180	35048181	MACS_peak_287	23.00	lobChIP_FOXA1inPBS
chr1	35151028	35151029	MACS_peak_288	9.00	lobChIP_FOXA1inPBS
chr1	35291363	35291364	MACS_peak_289	9.00	lobChIP_FOXA1inPBS
chr1	35667938	35667939	MACS_peak_290	9.00	lobChIP_FOXA1inPBS
chr1	35811431	35811432	MACS_peak_291	8.00	lobChIP_FOXA1inPBS
chr1	36021955	36021956	MACS_peak_292	25.00	lobChIP_FOXA1inPBS
chr1	36041367	36041368	MACS_peak_293	15.00	lobChIP_FOXA1inPBS
chr1	36094855	36094856	MACS_peak_294	15.00	lobChIP_FOXA1inPBS
chr1	36103411	36103412	MACS_peak_295	7.00	lobChIP_FOXA1inPBS
chr1	36105200	36105201	MACS_peak_296	8.00	lobChIP_FOXA1inPBS
chr1	36275113	36275114	MACS_peak_297	9.00	lobChIP_FOXA1inPBS
chr1	36705573	36705574	MACS_peak_298	9.00	lobChIP_FOXA1inPBS
chr1	36714413	36714414	MACS_peak_299	25.00	lobChIP_FOXA1inPBS
chr1	36735120	36735121	MACS_peak_300	65.00	lobChIP_FOXA1inPBS
chr1	36840233	36840234	MACS_peak_301	6.00	lobChIP_FOXA1inPBS
chr1	36952723	36952724	MACS_peak_302	50.00	lobChIP_FOXA1inPBS
chr1	37927578	37927579	MACS_peak_303	8.00	lobChIP_FOXA1inPBS
chr1	38000295	38000296	MACS_peak_304	11.00	lobChIP_FOXA1inPBS
chr1	38108735	38108736	MACS_peak_305	8.00	lobChIP_FOXA1inPBS
chr1	38119823	38119824	MACS_peak_306	10.00	lobChIP_FOXA1inPBS
chr1	38245788	38245789	MACS_peak_307	15.00	lobChIP_FOXA1inPBS
chr1	38328265	38328266	MACS_peak_308	15.00	lobChIP_FOXA1inPBS
chr1	38380636	38380637	MACS_peak_309	11.00	lobChIP_FOXA1inPBS
chr1	38453580	38453581	MACS_peak_310	33.00	lobChIP_FOXA1inPBS
chr1	38503547	38503548	MACS_peak_311	8.00	lobChIP_FOXA1inPBS
chr1	38605924	38605925	MACS_peak_312	10.00	lobChIP_FOXA1inPBS
chr1	38771416	38771417	MACS_peak_313	6.00	lobChIP_FOXA1inPBS
chr1	39354550	39354551	MACS_peak_314	6.00	lobChIP_FOXA1inPBS
chr1	39434942	39434943	MACS_peak_315	80.00	lobChIP_FOXA1inPBS
chr1	39451780	39451781	MACS_peak_316	19.00	lobChIP_FOXA1inPBS
chr1	39452473	39452474	MACS_peak_317	19.00	lobChIP_FOXA1inPBS
chr1	39458614	39458615	MACS_peak_318	7.00	lobChIP_FOXA1inPBS
chr1	39482381	39482382	MACS_peak_319	9.00	lobChIP_FOXA1inPBS
chr1	39558189	39558190	MACS_peak_320	8.00	lobChIP_FOXA1inPBS
chr1	39582710	39582711	MACS_peak_321	14.00	lobChIP_FOXA1inPBS
chr1	39624116	39624117	MACS_peak_322	10.00	lobChIP_FOXA1inPBS
chr1	39640258	39640259	MACS_peak_323	10.00	lobChIP_FOXA1inPBS
chr1	39666028	39666029	MACS_peak_324	14.00	lobChIP_FOXA1inPBS
chr1	39680091	39680092	MACS_peak_325	15.00	lobChIP_FOXA1inPBS
chr1	39844197	39844198	MACS_peak_326	7.00	lobChIP_FOXA1inPBS
chr1	39873649	39873650	MACS_peak_327	11.00	lobChIP_FOXA1inPBS
chr1	40265827	40265828	MACS_peak_328	9.00	lobChIP_FOXA1inPBS
chr1	40318098	40318099	MACS_peak_329	11.00	lobChIP_FOXA1inPBS
chr1	40318712	40318713	MACS_peak_330	8.00	lobChIP_FOXA1inPBS
chr1	40849907	40849908	MACS_peak_331	15.00	lobChIP_FOXA1inPBS
chr1	40855932	40855933	MACS_peak_332	14.00	lobChIP_FOXA1inPBS
chr1	40870672	40870673	MACS_peak_333	8.00	lobChIP_FOXA1inPBS
chr1	41001552	41001553	MACS_peak_334	8.00	lobChIP_FOXA1inPBS
chr1	41175910	41175911	MACS_peak_335	6.00	lobChIP_FOXA1inPBS
chr1	41178212	41178213	MACS_peak_336	56.00	lobChIP_FOXA1inPBS
chr1	41181566	41181567	MACS_peak_337	12.00	lobChIP_FOXA1inPBS
chr1	41215726	41215727	MACS_peak_338	9.00	lobChIP_FOXA1inPBS
chr1	41224432	41224433	MACS_peak_339	8.00	lobChIP_FOXA1inPBS
chr1	41290740	41290741	MACS_peak_340	14.00	lobChIP_FOXA1inPBS
chr1	41334165	41334166	MACS_peak_341	11.00	lobChIP_FOXA1inPBS
chr1	41385326	41385327	MACS_peak_342	18.00	lobChIP_FOXA1inPBS
chr1	41616755	41616756	MACS_peak_343	10.00	lobChIP_FOXA1inPBS
chr1	41705931	41705932	MACS_peak_344	31.00	lobChIP_FOXA1inPBS
chr1	42680418	42680419	MACS_peak_345	17.00	lobChIP_FOXA1inPBS
chr1	42781233	42781234	MACS_peak_346	10.00	lobChIP_FOXA1inPBS
chr1	42785979	42785980	MACS_peak_347	8.00	lobChIP_FOXA1inPBS
chr1	42790870	42790871	MACS_peak_348	16.00	lobChIP_FOXA1inPBS
chr1	42802116	42802117	MACS_peak_349	6.00	lobChIP_FOXA1inPBS
chr1	42806002	42806003	MACS_peak_350	20.00	lobChIP_FOXA1inPBS
chr1	42905770	42905771	MACS_peak_351	7.00	lobChIP_FOXA1inPBS
chr1	43086159	43086160	MACS_peak_352	16.00	lobChIP_FOXA1inPBS
chr1	43427564	43427565	MACS_peak_353	14.00	lobChIP_FOXA1inPBS
chr1	43446478	43446479	MACS_peak_354	11.00	lobChIP_FOXA1inPBS
chr1	43500109	43500110	MACS_peak_355	6.00	lobChIP_FOXA1inPBS
chr1	43505213	43505214	MACS_peak_356	49.00	lobChIP_FOXA1inPBS
chr1	43540177	43540178	MACS_peak_357	10.00	lobChIP_FOXA1inPBS
chr1	43667165	43667166	MACS_peak_358	9.00	lobChIP_FOXA1inPBS
chr1	43712632	43712633	MACS_peak_359	6.00	lobChIP_FOXA1inPBS
chr1	43779726	43779727	MACS_peak_360	9.00	lobChIP_FOXA1inPBS
chr1	43931645	43931646	MACS_peak_361	13.00	lobChIP_FOXA1inPBS
chr1	43940147	43940148	MACS_peak_362	7.00	lobChIP_FOXA1inPBS
chr1	43959704	43959705	MACS_peak_363	17.00	lobChIP_FOXA1inPBS
chr1	44014219	44014220	MACS_peak_364	12.00	lobChIP_FOXA1inPBS
chr1	44098166	44098167	MACS_peak_365	23.00	lobChIP_FOXA1inPBS
chr1	44223586	44223587	MACS_peak_366	19.00	lobChIP_FOXA1inPBS
chr1	44307692	44307693	MACS_peak_367	7.00	lobChIP_FOXA1inPBS
chr1	44446412	44446413	MACS_peak_368	8.00	lobChIP_FOXA1inPBS
chr1	44453062	44453063	MACS_peak_369	25.00	lobChIP_FOXA1inPBS
chr1	44475508	44475509	MACS_peak_370	21.00	lobChIP_FOXA1inPBS
chr1	44749446	44749447	MACS_peak_371	52.00	lobChIP_FOXA1inPBS
chr1	44763902	44763903	MACS_peak_372	8.00	lobChIP_FOXA1inPBS
chr1	44856083	44856084	MACS_peak_373	8.00	lobChIP_FOXA1inPBS
chr1	44870724	44870725	MACS_peak_374	6.00	lobChIP_FOXA1inPBS
chr1	45143044	45143045	MACS_peak_375	8.00	lobChIP_FOXA1inPBS
chr1	45147958	45147959	MACS_peak_376	30.00	lobChIP_FOXA1inPBS
chr1	45167029	45167030	MACS_peak_377	33.00	lobChIP_FOXA1inPBS
chr1	45412589	45412590	MACS_peak_378	32.00	lobChIP_FOXA1inPBS
chr1	45415106	45415107	MACS_peak_379	13.00	lobChIP_FOXA1inPBS
chr1	45428572	45428573	MACS_peak_380	9.00	lobChIP_FOXA1inPBS
chr1	45506966	45506967	MACS_peak_381	9.00	lobChIP_FOXA1inPBS
chr1	45572644	45572645	MACS_peak_382	9.00	lobChIP_FOXA1inPBS
chr1	45600307	45600308	MACS_peak_383	21.00	lobChIP_FOXA1inPBS
chr1	45637889	45637890	MACS_peak_384	106.00	lobChIP_FOXA1inPBS
chr1	45726203	45726204	MACS_peak_385	7.00	lobChIP_FOXA1inPBS
chr1	45732215	45732216	MACS_peak_386	15.00	lobChIP_FOXA1inPBS
chr1	45763883	45763884	MACS_peak_387	10.00	lobChIP_FOXA1inPBS
chr1	45787088	45787089	MACS_peak_388	7.00	lobChIP_FOXA1inPBS
chr1	45801698	45801699	MACS_peak_389	11.00	lobChIP_FOXA1inPBS
chr1	45967958	45967959	MACS_peak_390	26.00	lobChIP_FOXA1inPBS
chr1	45992048	45992049	MACS_peak_391	42.00	lobChIP_FOXA1inPBS
chr1	46123986	46123987	MACS_peak_392	18.00	lobChIP_FOXA1inPBS
chr1	46337982	46337983	MACS_peak_393	37.00	lobChIP_FOXA1inPBS
chr1	46444865	46444866	MACS_peak_394	18.00	lobChIP_FOXA1inPBS
chr1	46645420	46645421	MACS_peak_395	10.00	lobChIP_FOXA1inPBS
chr1	47779271	47779272	MACS_peak_396	10.00	lobChIP_FOXA1inPBS
chr1	47788468	47788469	MACS_peak_397	6.00	lobChIP_FOXA1inPBS
chr1	47844030	47844031	MACS_peak_398	35.00	lobChIP_FOXA1inPBS
chr1	47845471	47845472	MACS_peak_399	17.00	lobChIP_FOXA1inPBS
chr1	47846555	47846556	MACS_peak_400	9.00	lobChIP_FOXA1inPBS
chr1	47943937	47943938	MACS_peak_401	62.00	lobChIP_FOXA1inPBS
chr1	47974273	47974274	MACS_peak_402	10.00	lobChIP_FOXA1inPBS
chr1	48303796	48303797	MACS_peak_403	7.00	lobChIP_FOXA1inPBS
chr1	48318971	48318972	MACS_peak_404	12.00	lobChIP_FOXA1inPBS
chr1	48320429	48320430	MACS_peak_405	8.00	lobChIP_FOXA1inPBS
chr1	48332782	48332783	MACS_peak_406	15.00	lobChIP_FOXA1inPBS
chr1	48359311	48359312	MACS_peak_407	14.00	lobChIP_FOXA1inPBS
chr1	48377796	48377797	MACS_peak_408	5.00	lobChIP_FOXA1inPBS
chr1	48379123	48379124	MACS_peak_409	18.00	lobChIP_FOXA1inPBS
chr1	48581689	48581690	MACS_peak_410	9.00	lobChIP_FOXA1inPBS
chr1	48688355	48688356	MACS_peak_411	10.00	lobChIP_FOXA1inPBS
chr1	48775370	48775371	MACS_peak_412	13.00	lobChIP_FOXA1inPBS
chr1	48960198	48960199	MACS_peak_413	8.00	lobChIP_FOXA1inPBS
chr1	50680723	50680724	MACS_peak_414	7.00	lobChIP_FOXA1inPBS
chr1	50745840	50745841	MACS_peak_415	11.00	lobChIP_FOXA1inPBS
chr1	50940744	50940745	MACS_peak_416	7.00	lobChIP_FOXA1inPBS
chr1	51110617	51110618	MACS_peak_417	13.00	lobChIP_FOXA1inPBS
chr1	51350960	51350961	MACS_peak_418	7.00	lobChIP_FOXA1inPBS
chr1	51636135	51636136	MACS_peak_419	29.00	lobChIP_FOXA1inPBS
chr1	51682920	51682921	MACS_peak_420	13.00	lobChIP_FOXA1inPBS
chr1	51715270	51715271	MACS_peak_421	13.00	lobChIP_FOXA1inPBS
chr1	51983855	51983856	MACS_peak_422	19.00	lobChIP_FOXA1inPBS
chr1	52047191	52047192	MACS_peak_423	9.00	lobChIP_FOXA1inPBS
chr1	52116445	52116446	MACS_peak_424	7.00	lobChIP_FOXA1inPBS
chr1	52156556	52156557	MACS_peak_425	54.00	lobChIP_FOXA1inPBS
chr1	52199723	52199724	MACS_peak_426	7.00	lobChIP_FOXA1inPBS
chr1	52211419	52211420	MACS_peak_427	9.00	lobChIP_FOXA1inPBS
chr1	52434432	52434433	MACS_peak_428	14.00	lobChIP_FOXA1inPBS
chr1	52443041	52443042	MACS_peak_429	18.00	lobChIP_FOXA1inPBS
chr1	52503390	52503391	MACS_peak_430	33.00	lobChIP_FOXA1inPBS
chr1	52545990	52545991	MACS_peak_431	13.00	lobChIP_FOXA1inPBS
chr1	52564041	52564042	MACS_peak_432	16.00	lobChIP_FOXA1inPBS
chr1	52606924	52606925	MACS_peak_433	12.00	lobChIP_FOXA1inPBS
chr1	52655952	52655953	MACS_peak_434	15.00	lobChIP_FOXA1inPBS
chr1	52729333	52729334	MACS_peak_435	7.00	lobChIP_FOXA1inPBS
chr1	52747403	52747404	MACS_peak_436	8.00	lobChIP_FOXA1inPBS
chr1	52938921	52938922	MACS_peak_437	15.00	lobChIP_FOXA1inPBS
chr1	53018113	53018114	MACS_peak_438	6.00	lobChIP_FOXA1inPBS
chr1	53169102	53169103	MACS_peak_439	8.00	lobChIP_FOXA1inPBS
chr1	53280338	53280339	MACS_peak_440	15.00	lobChIP_FOXA1inPBS
chr1	53573475	53573476	MACS_peak_441	63.00	lobChIP_FOXA1inPBS
chr1	53626871	53626872	MACS_peak_442	9.00	lobChIP_FOXA1inPBS
chr1	53649315	53649316	MACS_peak_443	33.00	lobChIP_FOXA1inPBS
chr1	54151095	54151096	MACS_peak_444	9.00	lobChIP_FOXA1inPBS
chr1	54375783	54375784	MACS_peak_445	9.00	lobChIP_FOXA1inPBS
chr1	54422663	54422664	MACS_peak_446	34.00	lobChIP_FOXA1inPBS
chr1	54467420	54467421	MACS_peak_447	7.00	lobChIP_FOXA1inPBS
chr1	54488869	54488870	MACS_peak_448	7.00	lobChIP_FOXA1inPBS
chr1	54493658	54493659	MACS_peak_449	6.00	lobChIP_FOXA1inPBS
chr1	54536358	54536359	MACS_peak_450	8.00	lobChIP_FOXA1inPBS
chr1	54547469	54547470	MACS_peak_451	17.00	lobChIP_FOXA1inPBS
chr1	54560114	54560115	MACS_peak_452	12.00	lobChIP_FOXA1inPBS
chr1	54591599	54591600	MACS_peak_453	7.00	lobChIP_FOXA1inPBS
chr1	54813777	54813778	MACS_peak_454	7.00	lobChIP_FOXA1inPBS
chr1	54814237	54814238	MACS_peak_455	9.00	lobChIP_FOXA1inPBS
chr1	54849218	54849219	MACS_peak_456	15.00	lobChIP_FOXA1inPBS
chr1	54873444	54873445	MACS_peak_457	18.00	lobChIP_FOXA1inPBS
chr1	55326784	55326785	MACS_peak_458	16.00	lobChIP_FOXA1inPBS
chr1	55332912	55332913	MACS_peak_459	12.00	lobChIP_FOXA1inPBS
chr1	55333567	55333568	MACS_peak_460	14.00	lobChIP_FOXA1inPBS
chr1	55335081	55335082	MACS_peak_461	20.00	lobChIP_FOXA1inPBS
chr1	55348481	55348482	MACS_peak_462	6.00	lobChIP_FOXA1inPBS
chr1	55354278	55354279	MACS_peak_463	116.00	lobChIP_FOXA1inPBS
chr1	55385510	55385511	MACS_peak_464	9.00	lobChIP_FOXA1inPBS
chr1	55416648	55416649	MACS_peak_465	8.00	lobChIP_FOXA1inPBS
chr1	55594233	55594234	MACS_peak_466	8.00	lobChIP_FOXA1inPBS
chr1	55677386	55677387	MACS_peak_467	17.00	lobChIP_FOXA1inPBS
chr1	55892446	55892447	MACS_peak_468	9.00	lobChIP_FOXA1inPBS
chr1	55894574	55894575	MACS_peak_469	8.00	lobChIP_FOXA1inPBS
chr1	55896644	55896645	MACS_peak_470	8.00	lobChIP_FOXA1inPBS
chr1	55903561	55903562	MACS_peak_471	12.00	lobChIP_FOXA1inPBS
chr1	55913347	55913348	MACS_peak_472	11.00	lobChIP_FOXA1inPBS
chr1	55996624	55996625	MACS_peak_473	6.00	lobChIP_FOXA1inPBS
chr1	56655937	56655938	MACS_peak_474	7.00	lobChIP_FOXA1inPBS
chr1	56763838	56763839	MACS_peak_475	25.00	lobChIP_FOXA1inPBS
chr1	56797665	56797666	MACS_peak_476	29.00	lobChIP_FOXA1inPBS
chr1	56880574	56880575	MACS_peak_477	30.00	lobChIP_FOXA1inPBS
chr1	56882453	56882454	MACS_peak_478	10.00	lobChIP_FOXA1inPBS
chr1	56900007	56900008	MACS_peak_479	6.00	lobChIP_FOXA1inPBS
chr1	56910853	56910854	MACS_peak_480	9.00	lobChIP_FOXA1inPBS
chr1	56948351	56948352	MACS_peak_481	13.00	lobChIP_FOXA1inPBS
chr1	56960202	56960203	MACS_peak_482	27.00	lobChIP_FOXA1inPBS
chr1	56961960	56961961	MACS_peak_483	15.00	lobChIP_FOXA1inPBS
chr1	56975869	56975870	MACS_peak_484	12.00	lobChIP_FOXA1inPBS
chr1	57001915	57001916	MACS_peak_485	7.00	lobChIP_FOXA1inPBS
chr1	57005220	57005221	MACS_peak_486	10.00	lobChIP_FOXA1inPBS
chr1	57029347	57029348	MACS_peak_487	8.00	lobChIP_FOXA1inPBS
chr1	57035441	57035442	MACS_peak_488	44.00	lobChIP_FOXA1inPBS
chr1	57042813	57042814	MACS_peak_489	9.00	lobChIP_FOXA1inPBS
chr1	57045681	57045682	MACS_peak_490	10.00	lobChIP_FOXA1inPBS
chr1	57105073	57105074	MACS_peak_491	18.00	lobChIP_FOXA1inPBS
chr1	57154356	57154357	MACS_peak_492	7.00	lobChIP_FOXA1inPBS
chr1	57277115	57277116	MACS_peak_493	7.00	lobChIP_FOXA1inPBS
chr1	57284617	57284618	MACS_peak_494	15.00	lobChIP_FOXA1inPBS
chr1	57287229	57287230	MACS_peak_495	10.00	lobChIP_FOXA1inPBS
chr1	57320435	57320436	MACS_peak_496	12.00	lobChIP_FOXA1inPBS
chr1	57376427	57376428	MACS_peak_497	9.00	lobChIP_FOXA1inPBS
chr1	57377373	57377374	MACS_peak_498	11.00	lobChIP_FOXA1inPBS
chr1	57385556	57385557	MACS_peak_499	25.00	lobChIP_FOXA1inPBS
chr1	57431734	57431735	MACS_peak_500	27.00	lobChIP_FOXA1inPBS
chr1	57526356	57526357	MACS_peak_501	9.00	lobChIP_FOXA1inPBS
chr1	57746016	57746017	MACS_peak_502	31.00	lobChIP_FOXA1inPBS
chr1	57747115	57747116	MACS_peak_503	10.00	lobChIP_FOXA1inPBS
chr1	57930508	57930509	MACS_peak_504	50.00	lobChIP_FOXA1inPBS
chr1	58089362	58089363	MACS_peak_505	14.00	lobChIP_FOXA1inPBS
chr1	58912232	58912233	MACS_peak_506	8.00	lobChIP_FOXA1inPBS
chr1	58917390	58917391	MACS_peak_507	8.00	lobChIP_FOXA1inPBS
chr1	59035856	59035857	MACS_peak_508	10.00	lobChIP_FOXA1inPBS
chr1	59218741	59218742	MACS_peak_509	17.00	lobChIP_FOXA1inPBS
chr1	59351176	59351177	MACS_peak_510	76.00	lobChIP_FOXA1inPBS
chr1	59366950	59366951	MACS_peak_511	12.00	lobChIP_FOXA1inPBS
chr1	59448271	59448272	MACS_peak_512	8.00	lobChIP_FOXA1inPBS
chr1	59518064	59518065	MACS_peak_513	48.00	lobChIP_FOXA1inPBS
chr1	59536495	59536496	MACS_peak_514	7.00	lobChIP_FOXA1inPBS
chr1	59649721	59649722	MACS_peak_515	7.00	lobChIP_FOXA1inPBS
chr1	59680592	59680593	MACS_peak_516	18.00	lobChIP_FOXA1inPBS
chr1	59783155	59783156	MACS_peak_517	11.00	lobChIP_FOXA1inPBS
chr1	59812347	59812348	MACS_peak_518	12.00	lobChIP_FOXA1inPBS
chr1	59874454	59874455	MACS_peak_519	11.00	lobChIP_FOXA1inPBS
chr1	59892363	59892364	MACS_peak_520	8.00	lobChIP_FOXA1inPBS
chr1	59898546	59898547	MACS_peak_521	7.00	lobChIP_FOXA1inPBS
chr1	59972972	59972973	MACS_peak_522	5.00	lobChIP_FOXA1inPBS
chr1	59977885	59977886	MACS_peak_523	16.00	lobChIP_FOXA1inPBS
chr1	60015716	60015717	MACS_peak_524	13.00	lobChIP_FOXA1inPBS
chr1	60123768	60123769	MACS_peak_525	9.00	lobChIP_FOXA1inPBS
chr1	60132730	60132731	MACS_peak_526	8.00	lobChIP_FOXA1inPBS
chr1	60140504	60140505	MACS_peak_527	13.00	lobChIP_FOXA1inPBS
chr1	60204108	60204109	MACS_peak_528	12.00	lobChIP_FOXA1inPBS
chr1	60322529	60322530	MACS_peak_529	42.00	lobChIP_FOXA1inPBS
chr1	60402367	60402368	MACS_peak_530	14.00	lobChIP_FOXA1inPBS
chr1	61406565	61406566	MACS_peak_531	10.00	lobChIP_FOXA1inPBS
chr1	61521169	61521170	MACS_peak_532	11.00	lobChIP_FOXA1inPBS
chr1	61524343	61524344	MACS_peak_533	7.00	lobChIP_FOXA1inPBS
chr1	61555244	61555245	MACS_peak_534	41.00	lobChIP_FOXA1inPBS
chr1	61580272	61580273	MACS_peak_535	26.00	lobChIP_FOXA1inPBS
chr1	61586811	61586812	MACS_peak_536	11.00	lobChIP_FOXA1inPBS
chr1	61588227	61588228	MACS_peak_537	11.00	lobChIP_FOXA1inPBS
chr1	61598993	61598994	MACS_peak_538	13.00	lobChIP_FOXA1inPBS
chr1	61617982	61617983	MACS_peak_539	6.00	lobChIP_FOXA1inPBS
chr1	61623608	61623609	MACS_peak_540	9.00	lobChIP_FOXA1inPBS
chr1	61649932	61649933	MACS_peak_541	9.00	lobChIP_FOXA1inPBS
chr1	61689649	61689650	MACS_peak_542	9.00	lobChIP_FOXA1inPBS
chr1	61708742	61708743	MACS_peak_543	6.00	lobChIP_FOXA1inPBS
chr1	61765352	61765353	MACS_peak_544	19.00	lobChIP_FOXA1inPBS
chr1	61767764	61767765	MACS_peak_545	14.00	lobChIP_FOXA1inPBS
chr1	61788672	61788673	MACS_peak_546	9.00	lobChIP_FOXA1inPBS
chr1	61803729	61803730	MACS_peak_547	29.00	lobChIP_FOXA1inPBS
chr1	61804769	61804770	MACS_peak_548	16.00	lobChIP_FOXA1inPBS
chr1	62165770	62165771	MACS_peak_549	6.00	lobChIP_FOXA1inPBS
chr1	62380621	62380622	MACS_peak_550	9.00	lobChIP_FOXA1inPBS
chr1	62506810	62506811	MACS_peak_551	11.00	lobChIP_FOXA1inPBS
chr1	62707487	62707488	MACS_peak_552	37.00	lobChIP_FOXA1inPBS
chr1	62852208	62852209	MACS_peak_553	20.00	lobChIP_FOXA1inPBS
chr1	62989742	62989743	MACS_peak_554	13.00	lobChIP_FOXA1inPBS
chr1	62995097	62995098	MACS_peak_555	11.00	lobChIP_FOXA1inPBS
chr1	63057259	63057260	MACS_peak_556	12.00	lobChIP_FOXA1inPBS
chr1	63059851	63059852	MACS_peak_557	10.00	lobChIP_FOXA1inPBS
chr1	63087704	63087705	MACS_peak_558	40.00	lobChIP_FOXA1inPBS
chr1	63115990	63115991	MACS_peak_559	5.00	lobChIP_FOXA1inPBS
chr1	63199670	63199671	MACS_peak_560	14.00	lobChIP_FOXA1inPBS
chr1	63231848	63231849	MACS_peak_561	9.00	lobChIP_FOXA1inPBS
chr1	63255072	63255073	MACS_peak_562	6.00	lobChIP_FOXA1inPBS
chr1	63872878	63872879	MACS_peak_563	7.00	lobChIP_FOXA1inPBS
chr1	64058721	64058722	MACS_peak_564	9.00	lobChIP_FOXA1inPBS
chr1	64107023	64107024	MACS_peak_565	27.00	lobChIP_FOXA1inPBS
chr1	65061937	65061938	MACS_peak_566	68.00	lobChIP_FOXA1inPBS
chr1	65364031	65364032	MACS_peak_567	12.00	lobChIP_FOXA1inPBS
chr1	65372418	65372419	MACS_peak_568	8.00	lobChIP_FOXA1inPBS
chr1	65376557	65376558	MACS_peak_569	12.00	lobChIP_FOXA1inPBS
chr1	65381784	65381785	MACS_peak_570	28.00	lobChIP_FOXA1inPBS
chr1	65419213	65419214	MACS_peak_571	8.00	lobChIP_FOXA1inPBS
chr1	65447347	65447348	MACS_peak_572	6.00	lobChIP_FOXA1inPBS
chr1	65453365	65453366	MACS_peak_573	11.00	lobChIP_FOXA1inPBS
chr1	65593365	65593366	MACS_peak_574	11.00	lobChIP_FOXA1inPBS
chr1	65624624	65624625	MACS_peak_575	50.00	lobChIP_FOXA1inPBS
chr1	65644120	65644121	MACS_peak_576	11.00	lobChIP_FOXA1inPBS
chr1	65933413	65933414	MACS_peak_577	12.00	lobChIP_FOXA1inPBS
chr1	65937255	65937256	MACS_peak_578	11.00	lobChIP_FOXA1inPBS
chr1	65939933	65939934	MACS_peak_579	10.00	lobChIP_FOXA1inPBS
chr1	65950223	65950224	MACS_peak_580	22.00	lobChIP_FOXA1inPBS
chr1	66020147	66020148	MACS_peak_581	10.00	lobChIP_FOXA1inPBS
chr1	66613053	66613054	MACS_peak_582	8.00	lobChIP_FOXA1inPBS
chr1	67009279	67009280	MACS_peak_583	13.00	lobChIP_FOXA1inPBS
chr1	67446615	67446616	MACS_peak_584	14.00	lobChIP_FOXA1inPBS
chr1	67518544	67518545	MACS_peak_585	11.00	lobChIP_FOXA1inPBS
chr1	67833328	67833329	MACS_peak_586	9.00	lobChIP_FOXA1inPBS
chr1	67915096	67915097	MACS_peak_587	6.00	lobChIP_FOXA1inPBS
chr1	68000412	68000413	MACS_peak_588	65.00	lobChIP_FOXA1inPBS
chr1	68044541	68044542	MACS_peak_589	17.00	lobChIP_FOXA1inPBS
chr1	68129916	68129917	MACS_peak_590	25.00	lobChIP_FOXA1inPBS
chr1	68148499	68148500	MACS_peak_591	8.00	lobChIP_FOXA1inPBS
chr1	68163586	68163587	MACS_peak_592	14.00	lobChIP_FOXA1inPBS
chr1	70799069	70799070	MACS_peak_593	9.00	lobChIP_FOXA1inPBS
chr1	72948819	72948820	MACS_peak_594	25.00	lobChIP_FOXA1inPBS
chr1	74822434	74822435	MACS_peak_595	13.00	lobChIP_FOXA1inPBS
chr1	75155699	75155700	MACS_peak_596	61.00	lobChIP_FOXA1inPBS
chr1	75669527	75669528	MACS_peak_597	23.00	lobChIP_FOXA1inPBS
chr1	76084225	76084226	MACS_peak_598	7.00	lobChIP_FOXA1inPBS
chr1	76095111	76095112	MACS_peak_599	6.00	lobChIP_FOXA1inPBS
chr1	76201883	76201884	MACS_peak_600	13.00	lobChIP_FOXA1inPBS
chr1	76292422	76292423	MACS_peak_601	9.00	lobChIP_FOXA1inPBS
chr1	76320838	76320839	MACS_peak_602	8.00	lobChIP_FOXA1inPBS
chr1	77139860	77139861	MACS_peak_603	8.00	lobChIP_FOXA1inPBS
chr1	77204192	77204193	MACS_peak_604	10.00	lobChIP_FOXA1inPBS
chr1	77762635	77762636	MACS_peak_605	7.00	lobChIP_FOXA1inPBS
chr1	78121263	78121264	MACS_peak_606	11.00	lobChIP_FOXA1inPBS
chr1	78144785	78144786	MACS_peak_607	11.00	lobChIP_FOXA1inPBS
chr1	78175600	78175601	MACS_peak_608	10.00	lobChIP_FOXA1inPBS
chr1	78445595	78445596	MACS_peak_609	8.00	lobChIP_FOXA1inPBS
chr1	78470132	78470133	MACS_peak_610	10.00	lobChIP_FOXA1inPBS
chr1	78472425	78472426	MACS_peak_611	15.00	lobChIP_FOXA1inPBS
chr1	78580429	78580430	MACS_peak_612	6.00	lobChIP_FOXA1inPBS
chr1	79070573	79070574	MACS_peak_613	20.00	lobChIP_FOXA1inPBS
chr1	79073844	79073845	MACS_peak_614	8.00	lobChIP_FOXA1inPBS
chr1	81122027	81122028	MACS_peak_615	7.00	lobChIP_FOXA1inPBS
chr1	81348319	81348320	MACS_peak_616	8.00	lobChIP_FOXA1inPBS
chr1	82314708	82314709	MACS_peak_617	23.00	lobChIP_FOXA1inPBS
chr1	82572602	82572603	MACS_peak_618	14.00	lobChIP_FOXA1inPBS
chr1	85186305	85186306	MACS_peak_619	18.00	lobChIP_FOXA1inPBS
chr1	85743647	85743648	MACS_peak_620	12.00	lobChIP_FOXA1inPBS
chr1	85794878	85794879	MACS_peak_621	17.00	lobChIP_FOXA1inPBS
chr1	85927422	85927423	MACS_peak_622	6.00	lobChIP_FOXA1inPBS
chr1	86050017	86050018	MACS_peak_623	11.00	lobChIP_FOXA1inPBS
chr1	86094636	86094637	MACS_peak_624	7.00	lobChIP_FOXA1inPBS
chr1	86095489	86095490	MACS_peak_625	11.00	lobChIP_FOXA1inPBS
chr1	86134198	86134199	MACS_peak_626	8.00	lobChIP_FOXA1inPBS
chr1	86146811	86146812	MACS_peak_627	6.00	lobChIP_FOXA1inPBS
chr1	86175350	86175351	MACS_peak_628	12.00	lobChIP_FOXA1inPBS
chr1	86965914	86965915	MACS_peak_629	6.00	lobChIP_FOXA1inPBS
chr1	87161212	87161213	MACS_peak_630	8.00	lobChIP_FOXA1inPBS
chr1	87219619	87219620	MACS_peak_631	13.00	lobChIP_FOXA1inPBS
chr1	87224455	87224456	MACS_peak_632	10.00	lobChIP_FOXA1inPBS
chr1	87240396	87240397	MACS_peak_633	41.00	lobChIP_FOXA1inPBS
chr1	87248387	87248388	MACS_peak_634	20.00	lobChIP_FOXA1inPBS
chr1	87254146	87254147	MACS_peak_635	12.00	lobChIP_FOXA1inPBS
chr1	87258899	87258900	MACS_peak_636	39.00	lobChIP_FOXA1inPBS
chr1	87289647	87289648	MACS_peak_637	114.00	lobChIP_FOXA1inPBS
chr1	87326287	87326288	MACS_peak_638	15.00	lobChIP_FOXA1inPBS
chr1	87399436	87399437	MACS_peak_639	6.00	lobChIP_FOXA1inPBS
chr1	87433985	87433986	MACS_peak_640	12.00	lobChIP_FOXA1inPBS
chr1	87459235	87459236	MACS_peak_641	14.00	lobChIP_FOXA1inPBS
chr1	87461332	87461333	MACS_peak_642	11.00	lobChIP_FOXA1inPBS
chr1	87491043	87491044	MACS_peak_643	12.00	lobChIP_FOXA1inPBS
chr1	88252823	88252824	MACS_peak_644	30.00	lobChIP_FOXA1inPBS
chr1	88261786	88261787	MACS_peak_645	11.00	lobChIP_FOXA1inPBS
chr1	88641664	88641665	MACS_peak_646	8.00	lobChIP_FOXA1inPBS
chr1	89113303	89113304	MACS_peak_647	7.00	lobChIP_FOXA1inPBS
chr1	89126336	89126337	MACS_peak_648	6.00	lobChIP_FOXA1inPBS
chr1	89164165	89164166	MACS_peak_649	8.00	lobChIP_FOXA1inPBS
chr1	89279326	89279327	MACS_peak_650	13.00	lobChIP_FOXA1inPBS
chr1	89377551	89377552	MACS_peak_651	10.00	lobChIP_FOXA1inPBS
chr1	89386720	89386721	MACS_peak_652	30.00	lobChIP_FOXA1inPBS
chr1	89401373	89401374	MACS_peak_653	7.00	lobChIP_FOXA1inPBS
chr1	89488128	89488129	MACS_peak_654	8.00	lobChIP_FOXA1inPBS
chr1	89511262	89511263	MACS_peak_655	12.00	lobChIP_FOXA1inPBS
chr1	89822764	89822765	MACS_peak_656	21.00	lobChIP_FOXA1inPBS
chr1	90058388	90058389	MACS_peak_657	8.00	lobChIP_FOXA1inPBS
chr1	90276596	90276597	MACS_peak_658	20.00	lobChIP_FOXA1inPBS
chr1	90292270	90292271	MACS_peak_659	36.00	lobChIP_FOXA1inPBS
chr1	90299561	90299562	MACS_peak_660	10.00	lobChIP_FOXA1inPBS
chr1	90301994	90301995	MACS_peak_661	13.00	lobChIP_FOXA1inPBS
chr1	90336924	90336925	MACS_peak_662	14.00	lobChIP_FOXA1inPBS
chr1	90371804	90371805	MACS_peak_663	12.00	lobChIP_FOXA1inPBS
chr1	90408742	90408743	MACS_peak_664	17.00	lobChIP_FOXA1inPBS
chr1	90416027	90416028	MACS_peak_665	7.00	lobChIP_FOXA1inPBS
chr1	91187286	91187287	MACS_peak_666	8.00	lobChIP_FOXA1inPBS
chr1	91214460	91214461	MACS_peak_667	20.00	lobChIP_FOXA1inPBS
chr1	91389626	91389627	MACS_peak_668	7.00	lobChIP_FOXA1inPBS
chr1	91425349	91425350	MACS_peak_669	22.00	lobChIP_FOXA1inPBS
chr1	91480248	91480249	MACS_peak_670	8.00	lobChIP_FOXA1inPBS
chr1	91481920	91481921	MACS_peak_671	26.00	lobChIP_FOXA1inPBS
chr1	91502847	91502848	MACS_peak_672	8.00	lobChIP_FOXA1inPBS
chr1	91969295	91969296	MACS_peak_673	8.00	lobChIP_FOXA1inPBS
chr1	92211725	92211726	MACS_peak_674	9.00	lobChIP_FOXA1inPBS
chr1	92232677	92232678	MACS_peak_675	9.00	lobChIP_FOXA1inPBS
chr1	92293341	92293342	MACS_peak_676	22.00	lobChIP_FOXA1inPBS
chr1	92297354	92297355	MACS_peak_677	7.00	lobChIP_FOXA1inPBS
chr1	92310828	92310829	MACS_peak_678	9.00	lobChIP_FOXA1inPBS
chr1	92350065	92350066	MACS_peak_679	7.00	lobChIP_FOXA1inPBS
chr1	92504488	92504489	MACS_peak_680	11.00	lobChIP_FOXA1inPBS
chr1	92907674	92907675	MACS_peak_681	16.00	lobChIP_FOXA1inPBS
chr1	92970949	92970950	MACS_peak_682	13.00	lobChIP_FOXA1inPBS
chr1	93352630	93352631	MACS_peak_683	20.00	lobChIP_FOXA1inPBS
chr1	93411415	93411416	MACS_peak_684	20.00	lobChIP_FOXA1inPBS
chr1	93416889	93416890	MACS_peak_685	14.00	lobChIP_FOXA1inPBS
chr1	93595220	93595221	MACS_peak_686	13.00	lobChIP_FOXA1inPBS
chr1	93637448	93637449	MACS_peak_687	13.00	lobChIP_FOXA1inPBS
chr1	93638545	93638546	MACS_peak_688	10.00	lobChIP_FOXA1inPBS
chr1	93666432	93666433	MACS_peak_689	9.00	lobChIP_FOXA1inPBS
chr1	93694385	93694386	MACS_peak_690	16.00	lobChIP_FOXA1inPBS
chr1	93809931	93809932	MACS_peak_691	6.00	lobChIP_FOXA1inPBS
chr1	93827935	93827936	MACS_peak_692	7.00	lobChIP_FOXA1inPBS
chr1	93926774	93926775	MACS_peak_693	9.00	lobChIP_FOXA1inPBS
chr1	94046814	94046815	MACS_peak_694	10.00	lobChIP_FOXA1inPBS
chr1	94050999	94051000	MACS_peak_695	21.00	lobChIP_FOXA1inPBS
chr1	94080280	94080281	MACS_peak_696	8.00	lobChIP_FOXA1inPBS
chr1	94083826	94083827	MACS_peak_697	8.00	lobChIP_FOXA1inPBS
chr1	94224702	94224703	MACS_peak_698	5.00	lobChIP_FOXA1inPBS
chr1	94267216	94267217	MACS_peak_699	11.00	lobChIP_FOXA1inPBS
chr1	94293646	94293647	MACS_peak_700	7.00	lobChIP_FOXA1inPBS
chr1	94310059	94310060	MACS_peak_701	10.00	lobChIP_FOXA1inPBS
chr1	94314002	94314003	MACS_peak_702	11.00	lobChIP_FOXA1inPBS
chr1	94371547	94371548	MACS_peak_703	9.00	lobChIP_FOXA1inPBS
chr1	94424029	94424030	MACS_peak_704	6.00	lobChIP_FOXA1inPBS
chr1	94430416	94430417	MACS_peak_705	11.00	lobChIP_FOXA1inPBS
chr1	94445657	94445658	MACS_peak_706	14.00	lobChIP_FOXA1inPBS
chr1	94534760	94534761	MACS_peak_707	17.00	lobChIP_FOXA1inPBS
chr1	94544802	94544803	MACS_peak_708	15.00	lobChIP_FOXA1inPBS
chr1	94582093	94582094	MACS_peak_709	12.00	lobChIP_FOXA1inPBS
chr1	94591668	94591669	MACS_peak_710	14.00	lobChIP_FOXA1inPBS
chr1	94700364	94700365	MACS_peak_711	16.00	lobChIP_FOXA1inPBS
chr1	94774871	94774872	MACS_peak_712	10.00	lobChIP_FOXA1inPBS
chr1	94791303	94791304	MACS_peak_713	7.00	lobChIP_FOXA1inPBS
chr1	94821492	94821493	MACS_peak_714	11.00	lobChIP_FOXA1inPBS
chr1	94849883	94849884	MACS_peak_715	13.00	lobChIP_FOXA1inPBS
chr1	94850477	94850478	MACS_peak_716	8.00	lobChIP_FOXA1inPBS
chr1	94891539	94891540	MACS_peak_717	42.00	lobChIP_FOXA1inPBS
chr1	95028628	95028629	MACS_peak_718	16.00	lobChIP_FOXA1inPBS
chr1	95163592	95163593	MACS_peak_719	22.00	lobChIP_FOXA1inPBS
chr1	95164728	95164729	MACS_peak_720	24.00	lobChIP_FOXA1inPBS
chr1	95202353	95202354	MACS_peak_721	12.00	lobChIP_FOXA1inPBS
chr1	95224965	95224966	MACS_peak_722	13.00	lobChIP_FOXA1inPBS
chr1	95258736	95258737	MACS_peak_723	7.00	lobChIP_FOXA1inPBS
chr1	95263822	95263823	MACS_peak_724	13.00	lobChIP_FOXA1inPBS
chr1	95273818	95273819	MACS_peak_725	14.00	lobChIP_FOXA1inPBS
chr1	95278644	95278645	MACS_peak_726	11.00	lobChIP_FOXA1inPBS
chr1	95394772	95394773	MACS_peak_727	14.00	lobChIP_FOXA1inPBS
chr1	95428217	95428218	MACS_peak_728	9.00	lobChIP_FOXA1inPBS
chr1	95479744	95479745	MACS_peak_729	44.00	lobChIP_FOXA1inPBS
chr1	95588063	95588064	MACS_peak_730	27.00	lobChIP_FOXA1inPBS
chr1	95836341	95836342	MACS_peak_731	10.00	lobChIP_FOXA1inPBS
chr1	96090992	96090993	MACS_peak_732	14.00	lobChIP_FOXA1inPBS
chr1	97097683	97097684	MACS_peak_733	77.00	lobChIP_FOXA1inPBS
chr1	97224232	97224233	MACS_peak_734	8.00	lobChIP_FOXA1inPBS
chr1	97991366	97991367	MACS_peak_735	11.00	lobChIP_FOXA1inPBS
chr1	99128667	99128668	MACS_peak_736	12.00	lobChIP_FOXA1inPBS
chr1	100020487	100020488	MACS_peak_737	13.00	lobChIP_FOXA1inPBS
chr1	100024931	100024932	MACS_peak_738	10.00	lobChIP_FOXA1inPBS
chr1	100032274	100032275	MACS_peak_739	20.00	lobChIP_FOXA1inPBS
chr1	100054894	100054895	MACS_peak_740	11.00	lobChIP_FOXA1inPBS
chr1	100101016	100101017	MACS_peak_741	15.00	lobChIP_FOXA1inPBS
chr1	100204679	100204680	MACS_peak_742	13.00	lobChIP_FOXA1inPBS
chr1	100220486	100220487	MACS_peak_743	18.00	lobChIP_FOXA1inPBS
chr1	100225527	100225528	MACS_peak_744	20.00	lobChIP_FOXA1inPBS
chr1	100816837	100816838	MACS_peak_745	13.00	lobChIP_FOXA1inPBS
chr1	100878717	100878718	MACS_peak_746	8.00	lobChIP_FOXA1inPBS
chr1	100951531	100951532	MACS_peak_747	74.00	lobChIP_FOXA1inPBS
chr1	100981583	100981584	MACS_peak_748	23.00	lobChIP_FOXA1inPBS
chr1	101048781	101048782	MACS_peak_749	11.00	lobChIP_FOXA1inPBS
chr1	101055445	101055446	MACS_peak_750	10.00	lobChIP_FOXA1inPBS
chr1	101197035	101197036	MACS_peak_751	15.00	lobChIP_FOXA1inPBS
chr1	101469816	101469817	MACS_peak_752	57.00	lobChIP_FOXA1inPBS
chr1	101602387	101602388	MACS_peak_753	12.00	lobChIP_FOXA1inPBS
chr1	101754269	101754270	MACS_peak_754	10.00	lobChIP_FOXA1inPBS
chr1	101775031	101775032	MACS_peak_755	7.00	lobChIP_FOXA1inPBS
chr1	102197980	102197981	MACS_peak_756	6.00	lobChIP_FOXA1inPBS
chr1	104098765	104098766	MACS_peak_757	6.00	lobChIP_FOXA1inPBS
chr1	108069452	108069453	MACS_peak_758	44.00	lobChIP_FOXA1inPBS
chr1	108184185	108184186	MACS_peak_759	18.00	lobChIP_FOXA1inPBS
chr1	108223842	108223843	MACS_peak_760	36.00	lobChIP_FOXA1inPBS
chr1	108225890	108225891	MACS_peak_761	13.00	lobChIP_FOXA1inPBS
chr1	108577295	108577296	MACS_peak_762	9.00	lobChIP_FOXA1inPBS
chr1	108594379	108594380	MACS_peak_763	6.00	lobChIP_FOXA1inPBS
chr1	109265239	109265240	MACS_peak_764	12.00	lobChIP_FOXA1inPBS
chr1	109322223	109322224	MACS_peak_765	6.00	lobChIP_FOXA1inPBS
chr1	109507307	109507308	MACS_peak_766	16.00	lobChIP_FOXA1inPBS
chr1	109659157	109659158	MACS_peak_767	12.00	lobChIP_FOXA1inPBS
chr1	109694547	109694548	MACS_peak_768	7.00	lobChIP_FOXA1inPBS
chr1	109719168	109719169	MACS_peak_769	14.00	lobChIP_FOXA1inPBS
chr1	109724148	109724149	MACS_peak_770	40.00	lobChIP_FOXA1inPBS
chr1	109817591	109817592	MACS_peak_771	19.00	lobChIP_FOXA1inPBS
chr1	109820000	109820001	MACS_peak_772	8.00	lobChIP_FOXA1inPBS
chr1	109842038	109842039	MACS_peak_773	30.00	lobChIP_FOXA1inPBS
chr1	109908353	109908354	MACS_peak_774	6.00	lobChIP_FOXA1inPBS
chr1	109913752	109913753	MACS_peak_775	8.00	lobChIP_FOXA1inPBS
chr1	109922106	109922107	MACS_peak_776	7.00	lobChIP_FOXA1inPBS
chr1	109935893	109935894	MACS_peak_777	31.00	lobChIP_FOXA1inPBS
chr1	109983975	109983976	MACS_peak_778	14.00	lobChIP_FOXA1inPBS
chr1	110110706	110110707	MACS_peak_779	10.00	lobChIP_FOXA1inPBS
chr1	110366963	110366964	MACS_peak_780	5.00	lobChIP_FOXA1inPBS
chr1	110416176	110416177	MACS_peak_781	6.00	lobChIP_FOXA1inPBS
chr1	110420317	110420318	MACS_peak_782	8.00	lobChIP_FOXA1inPBS
chr1	110425461	110425462	MACS_peak_783	18.00	lobChIP_FOXA1inPBS
chr1	110491081	110491082	MACS_peak_784	15.00	lobChIP_FOXA1inPBS
chr1	110503269	110503270	MACS_peak_785	42.00	lobChIP_FOXA1inPBS
chr1	110521546	110521547	MACS_peak_786	10.00	lobChIP_FOXA1inPBS
chr1	110577052	110577053	MACS_peak_787	9.00	lobChIP_FOXA1inPBS
chr1	110863856	110863857	MACS_peak_788	7.00	lobChIP_FOXA1inPBS
chr1	111323878	111323879	MACS_peak_789	9.00	lobChIP_FOXA1inPBS
chr1	111573288	111573289	MACS_peak_790	27.00	lobChIP_FOXA1inPBS
chr1	111587977	111587978	MACS_peak_791	23.00	lobChIP_FOXA1inPBS
chr1	111653056	111653057	MACS_peak_792	26.00	lobChIP_FOXA1inPBS
chr1	111685399	111685400	MACS_peak_793	8.00	lobChIP_FOXA1inPBS
chr1	111737857	111737858	MACS_peak_794	14.00	lobChIP_FOXA1inPBS
chr1	111770917	111770918	MACS_peak_795	24.00	lobChIP_FOXA1inPBS
chr1	111960560	111960561	MACS_peak_796	33.00	lobChIP_FOXA1inPBS
chr1	112052107	112052108	MACS_peak_797	12.00	lobChIP_FOXA1inPBS
chr1	112060986	112060987	MACS_peak_798	7.00	lobChIP_FOXA1inPBS
chr1	112077072	112077073	MACS_peak_799	8.00	lobChIP_FOXA1inPBS
chr1	112078594	112078595	MACS_peak_800	7.00	lobChIP_FOXA1inPBS
chr1	112102629	112102630	MACS_peak_801	7.00	lobChIP_FOXA1inPBS
chr1	112182449	112182450	MACS_peak_802	5.00	lobChIP_FOXA1inPBS
chr1	112205355	112205356	MACS_peak_803	6.00	lobChIP_FOXA1inPBS
chr1	112282186	112282187	MACS_peak_804	9.00	lobChIP_FOXA1inPBS
chr1	112289992	112289993	MACS_peak_805	9.00	lobChIP_FOXA1inPBS
chr1	112519655	112519656	MACS_peak_806	49.00	lobChIP_FOXA1inPBS
chr1	112937300	112937301	MACS_peak_807	16.00	lobChIP_FOXA1inPBS
chr1	112939846	112939847	MACS_peak_808	8.00	lobChIP_FOXA1inPBS
chr1	113003441	113003442	MACS_peak_809	10.00	lobChIP_FOXA1inPBS
chr1	113178173	113178174	MACS_peak_810	9.00	lobChIP_FOXA1inPBS
chr1	113547075	113547076	MACS_peak_811	14.00	lobChIP_FOXA1inPBS
chr1	113564612	113564613	MACS_peak_812	9.00	lobChIP_FOXA1inPBS
chr1	113689033	113689034	MACS_peak_813	7.00	lobChIP_FOXA1inPBS
chr1	113796820	113796821	MACS_peak_814	7.00	lobChIP_FOXA1inPBS
chr1	113931435	113931436	MACS_peak_815	31.00	lobChIP_FOXA1inPBS
chr1	113937001	113937002	MACS_peak_816	17.00	lobChIP_FOXA1inPBS
chr1	113976819	113976820	MACS_peak_817	14.00	lobChIP_FOXA1inPBS
chr1	113977300	113977301	MACS_peak_818	9.00	lobChIP_FOXA1inPBS
chr1	113980863	113980864	MACS_peak_819	9.00	lobChIP_FOXA1inPBS
chr1	114050707	114050708	MACS_peak_820	10.00	lobChIP_FOXA1inPBS
chr1	114060151	114060152	MACS_peak_821	16.00	lobChIP_FOXA1inPBS
chr1	114347591	114347592	MACS_peak_822	9.00	lobChIP_FOXA1inPBS
chr1	114352713	114352714	MACS_peak_823	13.00	lobChIP_FOXA1inPBS
chr1	114471706	114471707	MACS_peak_824	17.00	lobChIP_FOXA1inPBS
chr1	114996222	114996223	MACS_peak_825	10.00	lobChIP_FOXA1inPBS
chr1	115000506	115000507	MACS_peak_826	9.00	lobChIP_FOXA1inPBS
chr1	115001323	115001324	MACS_peak_827	8.00	lobChIP_FOXA1inPBS
chr1	115010190	115010191	MACS_peak_828	47.00	lobChIP_FOXA1inPBS
chr1	115124356	115124357	MACS_peak_829	9.00	lobChIP_FOXA1inPBS
chr1	115211721	115211722	MACS_peak_830	15.00	lobChIP_FOXA1inPBS
chr1	115579910	115579911	MACS_peak_831	17.00	lobChIP_FOXA1inPBS
chr1	116109481	116109482	MACS_peak_832	9.00	lobChIP_FOXA1inPBS
chr1	116254122	116254123	MACS_peak_833	15.00	lobChIP_FOXA1inPBS
chr1	116665890	116665891	MACS_peak_834	12.00	lobChIP_FOXA1inPBS
chr1	116722246	116722247	MACS_peak_835	6.00	lobChIP_FOXA1inPBS
chr1	116835730	116835731	MACS_peak_836	19.00	lobChIP_FOXA1inPBS
chr1	116858376	116858377	MACS_peak_837	8.00	lobChIP_FOXA1inPBS
chr1	116864757	116864758	MACS_peak_838	6.00	lobChIP_FOXA1inPBS
chr1	116920800	116920801	MACS_peak_839	10.00	lobChIP_FOXA1inPBS
chr1	116963189	116963190	MACS_peak_840	17.00	lobChIP_FOXA1inPBS
chr1	116975657	116975658	MACS_peak_841	9.00	lobChIP_FOXA1inPBS
chr1	116991579	116991580	MACS_peak_842	8.00	lobChIP_FOXA1inPBS
chr1	116994825	116994826	MACS_peak_843	10.00	lobChIP_FOXA1inPBS
chr1	117008769	117008770	MACS_peak_844	52.00	lobChIP_FOXA1inPBS
chr1	117018005	117018006	MACS_peak_845	9.00	lobChIP_FOXA1inPBS
chr1	117071006	117071007	MACS_peak_846	6.00	lobChIP_FOXA1inPBS
chr1	117396451	117396452	MACS_peak_847	11.00	lobChIP_FOXA1inPBS
chr1	117464335	117464336	MACS_peak_848	11.00	lobChIP_FOXA1inPBS
chr1	117914614	117914615	MACS_peak_849	10.00	lobChIP_FOXA1inPBS
chr1	118277886	118277887	MACS_peak_850	9.00	lobChIP_FOXA1inPBS
chr1	118297570	118297571	MACS_peak_851	9.00	lobChIP_FOXA1inPBS
chr1	118301754	118301755	MACS_peak_852	9.00	lobChIP_FOXA1inPBS
chr1	118364559	118364560	MACS_peak_853	14.00	lobChIP_FOXA1inPBS
chr1	118400243	118400244	MACS_peak_854	9.00	lobChIP_FOXA1inPBS
chr1	118471521	118471522	MACS_peak_855	12.00	lobChIP_FOXA1inPBS
chr1	118659728	118659729	MACS_peak_856	7.00	lobChIP_FOXA1inPBS
chr1	119402834	119402835	MACS_peak_857	10.00	lobChIP_FOXA1inPBS
chr1	119457129	119457130	MACS_peak_858	10.00	lobChIP_FOXA1inPBS
chr1	119503401	119503402	MACS_peak_859	18.00	lobChIP_FOXA1inPBS
chr1	119506461	119506462	MACS_peak_860	18.00	lobChIP_FOXA1inPBS
chr1	119879824	119879825	MACS_peak_861	15.00	lobChIP_FOXA1inPBS
chr1	119884247	119884248	MACS_peak_862	50.00	lobChIP_FOXA1inPBS
chr1	120010785	120010786	MACS_peak_863	9.00	lobChIP_FOXA1inPBS
chr1	120183228	120183229	MACS_peak_864	10.00	lobChIP_FOXA1inPBS
chr1	120309221	120309222	MACS_peak_865	34.00	lobChIP_FOXA1inPBS
chr1	120359173	120359174	MACS_peak_866	12.00	lobChIP_FOXA1inPBS
chr1	120436639	120436640	MACS_peak_867	7.00	lobChIP_FOXA1inPBS
chr1	120575167	120575168	MACS_peak_868	10.00	lobChIP_FOXA1inPBS
chr1	121261483	121261484	MACS_peak_869	10.00	lobChIP_FOXA1inPBS
chr1	121264062	121264063	MACS_peak_870	21.00	lobChIP_FOXA1inPBS
chr1	121280928	121280929	MACS_peak_871	14.00	lobChIP_FOXA1inPBS
chr1	121328445	121328446	MACS_peak_872	45.00	lobChIP_FOXA1inPBS
chr1	144534286	144534287	MACS_peak_873	13.00	lobChIP_FOXA1inPBS
chr1	144924031	144924032	MACS_peak_874	15.00	lobChIP_FOXA1inPBS
chr1	144937371	144937372	MACS_peak_875	17.00	lobChIP_FOXA1inPBS
chr1	145246349	145246350	MACS_peak_876	21.00	lobChIP_FOXA1inPBS
chr1	145384690	145384691	MACS_peak_877	17.00	lobChIP_FOXA1inPBS
chr1	145390982	145390983	MACS_peak_878	21.00	lobChIP_FOXA1inPBS
chr1	145422122	145422123	MACS_peak_879	18.00	lobChIP_FOXA1inPBS
chr1	145462352	145462353	MACS_peak_880	8.00	lobChIP_FOXA1inPBS
chr1	145542730	145542731	MACS_peak_881	21.00	lobChIP_FOXA1inPBS
chr1	145723702	145723703	MACS_peak_882	10.00	lobChIP_FOXA1inPBS
chr1	145743171	145743172	MACS_peak_883	8.00	lobChIP_FOXA1inPBS
chr1	146565184	146565185	MACS_peak_884	14.00	lobChIP_FOXA1inPBS
chr1	146582720	146582721	MACS_peak_885	9.00	lobChIP_FOXA1inPBS
chr1	146640916	146640917	MACS_peak_886	7.00	lobChIP_FOXA1inPBS
chr1	146687241	146687242	MACS_peak_887	26.00	lobChIP_FOXA1inPBS
chr1	146709966	146709967	MACS_peak_888	18.00	lobChIP_FOXA1inPBS
chr1	146710735	146710736	MACS_peak_889	10.00	lobChIP_FOXA1inPBS
chr1	146874582	146874583	MACS_peak_890	9.00	lobChIP_FOXA1inPBS
chr1	147084394	147084395	MACS_peak_891	30.00	lobChIP_FOXA1inPBS
chr1	147097238	147097239	MACS_peak_892	32.00	lobChIP_FOXA1inPBS
chr1	147189479	147189480	MACS_peak_893	7.00	lobChIP_FOXA1inPBS
chr1	147327348	147327349	MACS_peak_894	7.00	lobChIP_FOXA1inPBS
chr1	148931512	148931513	MACS_peak_895	7.00	lobChIP_FOXA1inPBS
chr1	149223946	149223947	MACS_peak_896	11.00	lobChIP_FOXA1inPBS
chr1	149980800	149980801	MACS_peak_897	50.00	lobChIP_FOXA1inPBS
chr1	150038867	150038868	MACS_peak_898	9.00	lobChIP_FOXA1inPBS
chr1	150208318	150208319	MACS_peak_899	7.00	lobChIP_FOXA1inPBS
chr1	150230809	150230810	MACS_peak_900	10.00	lobChIP_FOXA1inPBS
chr1	150266177	150266178	MACS_peak_901	7.00	lobChIP_FOXA1inPBS
chr1	150334081	150334082	MACS_peak_902	9.00	lobChIP_FOXA1inPBS
chr1	150339774	150339775	MACS_peak_903	7.00	lobChIP_FOXA1inPBS
chr1	150359114	150359115	MACS_peak_904	18.00	lobChIP_FOXA1inPBS
chr1	150378387	150378388	MACS_peak_905	11.00	lobChIP_FOXA1inPBS
chr1	150383283	150383284	MACS_peak_906	10.00	lobChIP_FOXA1inPBS
chr1	150446407	150446408	MACS_peak_907	16.00	lobChIP_FOXA1inPBS
chr1	150504871	150504872	MACS_peak_908	7.00	lobChIP_FOXA1inPBS
chr1	150516515	150516516	MACS_peak_909	11.00	lobChIP_FOXA1inPBS
chr1	150540757	150540758	MACS_peak_910	10.00	lobChIP_FOXA1inPBS
chr1	150548489	150548490	MACS_peak_911	16.00	lobChIP_FOXA1inPBS
chr1	150584087	150584088	MACS_peak_912	13.00	lobChIP_FOXA1inPBS
chr1	150824937	150824938	MACS_peak_913	15.00	lobChIP_FOXA1inPBS
chr1	150847375	150847376	MACS_peak_914	10.00	lobChIP_FOXA1inPBS
chr1	150944125	150944126	MACS_peak_915	14.00	lobChIP_FOXA1inPBS
chr1	150980729	150980730	MACS_peak_916	7.00	lobChIP_FOXA1inPBS
chr1	150985673	150985674	MACS_peak_917	14.00	lobChIP_FOXA1inPBS
chr1	151044291	151044292	MACS_peak_918	16.00	lobChIP_FOXA1inPBS
chr1	151486477	151486478	MACS_peak_919	8.00	lobChIP_FOXA1inPBS
chr1	151538082	151538083	MACS_peak_920	13.00	lobChIP_FOXA1inPBS
chr1	151702257	151702258	MACS_peak_921	9.00	lobChIP_FOXA1inPBS
chr1	151948091	151948092	MACS_peak_922	9.00	lobChIP_FOXA1inPBS
chr1	152901345	152901346	MACS_peak_923	11.00	lobChIP_FOXA1inPBS
chr1	153505251	153505252	MACS_peak_924	9.00	lobChIP_FOXA1inPBS
chr1	153603734	153603735	MACS_peak_925	10.00	lobChIP_FOXA1inPBS
chr1	153612076	153612077	MACS_peak_926	8.00	lobChIP_FOXA1inPBS
chr1	153627918	153627919	MACS_peak_927	11.00	lobChIP_FOXA1inPBS
chr1	153702936	153702937	MACS_peak_928	7.00	lobChIP_FOXA1inPBS
chr1	153715023	153715024	MACS_peak_929	7.00	lobChIP_FOXA1inPBS
chr1	153741027	153741028	MACS_peak_930	9.00	lobChIP_FOXA1inPBS
chr1	153926534	153926535	MACS_peak_931	20.00	lobChIP_FOXA1inPBS
chr1	154210019	154210020	MACS_peak_932	12.00	lobChIP_FOXA1inPBS
chr1	154306867	154306868	MACS_peak_933	10.00	lobChIP_FOXA1inPBS
chr1	154324003	154324004	MACS_peak_934	9.00	lobChIP_FOXA1inPBS
chr1	154359776	154359777	MACS_peak_935	18.00	lobChIP_FOXA1inPBS
chr1	154381115	154381116	MACS_peak_936	17.00	lobChIP_FOXA1inPBS
chr1	154390917	154390918	MACS_peak_937	30.00	lobChIP_FOXA1inPBS
chr1	154392349	154392350	MACS_peak_938	11.00	lobChIP_FOXA1inPBS
chr1	155063971	155063972	MACS_peak_939	8.00	lobChIP_FOXA1inPBS
chr1	155080027	155080028	MACS_peak_940	11.00	lobChIP_FOXA1inPBS
chr1	155099212	155099213	MACS_peak_941	12.00	lobChIP_FOXA1inPBS
chr1	155099851	155099852	MACS_peak_942	31.00	lobChIP_FOXA1inPBS
chr1	155428110	155428111	MACS_peak_943	13.00	lobChIP_FOXA1inPBS
chr1	155618562	155618563	MACS_peak_944	17.00	lobChIP_FOXA1inPBS
chr1	155714828	155714829	MACS_peak_945	8.00	lobChIP_FOXA1inPBS
chr1	155862133	155862134	MACS_peak_946	8.00	lobChIP_FOXA1inPBS
chr1	155914679	155914680	MACS_peak_947	46.00	lobChIP_FOXA1inPBS
chr1	155937255	155937256	MACS_peak_948	13.00	lobChIP_FOXA1inPBS
chr1	155953618	155953619	MACS_peak_949	7.00	lobChIP_FOXA1inPBS
chr1	155970944	155970945	MACS_peak_950	7.00	lobChIP_FOXA1inPBS
chr1	156056170	156056171	MACS_peak_951	18.00	lobChIP_FOXA1inPBS
chr1	156093735	156093736	MACS_peak_952	9.00	lobChIP_FOXA1inPBS
chr1	156154181	156154182	MACS_peak_953	12.00	lobChIP_FOXA1inPBS
chr1	156161378	156161379	MACS_peak_954	11.00	lobChIP_FOXA1inPBS
chr1	156195320	156195321	MACS_peak_955	30.00	lobChIP_FOXA1inPBS
chr1	156338325	156338326	MACS_peak_956	7.00	lobChIP_FOXA1inPBS
chr1	156441481	156441482	MACS_peak_957	26.00	lobChIP_FOXA1inPBS
chr1	156723192	156723193	MACS_peak_958	11.00	lobChIP_FOXA1inPBS
chr1	156822662	156822663	MACS_peak_959	7.00	lobChIP_FOXA1inPBS
chr1	157210482	157210483	MACS_peak_960	27.00	lobChIP_FOXA1inPBS
chr1	159252106	159252107	MACS_peak_961	6.00	lobChIP_FOXA1inPBS
chr1	159941021	159941022	MACS_peak_962	10.00	lobChIP_FOXA1inPBS
chr1	160015558	160015559	MACS_peak_963	7.00	lobChIP_FOXA1inPBS
chr1	160023658	160023659	MACS_peak_964	10.00	lobChIP_FOXA1inPBS
chr1	160050695	160050696	MACS_peak_965	15.00	lobChIP_FOXA1inPBS
chr1	160221918	160221919	MACS_peak_966	15.00	lobChIP_FOXA1inPBS
chr1	160231479	160231480	MACS_peak_967	10.00	lobChIP_FOXA1inPBS
chr1	160265795	160265796	MACS_peak_968	8.00	lobChIP_FOXA1inPBS
chr1	160948444	160948445	MACS_peak_969	9.00	lobChIP_FOXA1inPBS
chr1	161188487	161188488	MACS_peak_970	7.00	lobChIP_FOXA1inPBS
chr1	161193434	161193435	MACS_peak_971	13.00	lobChIP_FOXA1inPBS
chr1	161194213	161194214	MACS_peak_972	12.00	lobChIP_FOXA1inPBS
chr1	161225921	161225922	MACS_peak_973	7.00	lobChIP_FOXA1inPBS
chr1	161368015	161368016	MACS_peak_974	29.00	lobChIP_FOXA1inPBS
chr1	161672104	161672105	MACS_peak_975	16.00	lobChIP_FOXA1inPBS
chr1	161735969	161735970	MACS_peak_976	35.00	lobChIP_FOXA1inPBS
chr1	161854456	161854457	MACS_peak_977	11.00	lobChIP_FOXA1inPBS
chr1	161875206	161875207	MACS_peak_978	8.00	lobChIP_FOXA1inPBS
chr1	161883745	161883746	MACS_peak_979	16.00	lobChIP_FOXA1inPBS
chr1	162061966	162061967	MACS_peak_980	19.00	lobChIP_FOXA1inPBS
chr1	162110858	162110859	MACS_peak_981	9.00	lobChIP_FOXA1inPBS
chr1	162122285	162122286	MACS_peak_982	11.00	lobChIP_FOXA1inPBS
chr1	162203082	162203083	MACS_peak_983	8.00	lobChIP_FOXA1inPBS
chr1	162217336	162217337	MACS_peak_984	8.00	lobChIP_FOXA1inPBS
chr1	162327637	162327638	MACS_peak_985	36.00	lobChIP_FOXA1inPBS
chr1	162530489	162530490	MACS_peak_986	28.00	lobChIP_FOXA1inPBS
chr1	162651923	162651924	MACS_peak_987	7.00	lobChIP_FOXA1inPBS
chr1	162716618	162716619	MACS_peak_988	16.00	lobChIP_FOXA1inPBS
chr1	162844020	162844021	MACS_peak_989	21.00	lobChIP_FOXA1inPBS
chr1	162863275	162863276	MACS_peak_990	12.00	lobChIP_FOXA1inPBS
chr1	163514440	163514441	MACS_peak_991	10.00	lobChIP_FOXA1inPBS
chr1	165223425	165223426	MACS_peak_992	8.00	lobChIP_FOXA1inPBS
chr1	165798319	165798320	MACS_peak_993	8.00	lobChIP_FOXA1inPBS
chr1	165880183	165880184	MACS_peak_994	10.00	lobChIP_FOXA1inPBS
chr1	167208069	167208070	MACS_peak_995	6.00	lobChIP_FOXA1inPBS
chr1	167597424	167597425	MACS_peak_996	7.00	lobChIP_FOXA1inPBS
chr1	167786634	167786635	MACS_peak_997	62.00	lobChIP_FOXA1inPBS
chr1	167895984	167895985	MACS_peak_998	9.00	lobChIP_FOXA1inPBS
chr1	167896951	167896952	MACS_peak_999	9.00	lobChIP_FOXA1inPBS
chr1	167953235	167953236	MACS_peak_1000	6.00	lobChIP_FOXA1inPBS
chr1	168116001	168116002	MACS_peak_1001	7.00	lobChIP_FOXA1inPBS
chr1	168186316	168186317	MACS_peak_1002	11.00	lobChIP_FOXA1inPBS
chr1	168464986	168464987	MACS_peak_1003	25.00	lobChIP_FOXA1inPBS
chr1	168467019	168467020	MACS_peak_1004	13.00	lobChIP_FOXA1inPBS
chr1	168521149	168521150	MACS_peak_1005	28.00	lobChIP_FOXA1inPBS
chr1	168525023	168525024	MACS_peak_1006	13.00	lobChIP_FOXA1inPBS
chr1	168526734	168526735	MACS_peak_1007	30.00	lobChIP_FOXA1inPBS
chr1	168538828	168538829	MACS_peak_1008	11.00	lobChIP_FOXA1inPBS
chr1	168658588	168658589	MACS_peak_1009	46.00	lobChIP_FOXA1inPBS
chr1	168770867	168770868	MACS_peak_1010	19.00	lobChIP_FOXA1inPBS
chr1	168831027	168831028	MACS_peak_1011	14.00	lobChIP_FOXA1inPBS
chr1	169079311	169079312	MACS_peak_1012	29.00	lobChIP_FOXA1inPBS
chr1	169085630	169085631	MACS_peak_1013	11.00	lobChIP_FOXA1inPBS
chr1	169220290	169220291	MACS_peak_1014	18.00	lobChIP_FOXA1inPBS
chr1	169477266	169477267	MACS_peak_1015	5.00	lobChIP_FOXA1inPBS
chr1	169479444	169479445	MACS_peak_1016	6.00	lobChIP_FOXA1inPBS
chr1	169539509	169539510	MACS_peak_1017	51.00	lobChIP_FOXA1inPBS
chr1	169554129	169554130	MACS_peak_1018	13.00	lobChIP_FOXA1inPBS
chr1	169557700	169557701	MACS_peak_1019	14.00	lobChIP_FOXA1inPBS
chr1	169714560	169714561	MACS_peak_1020	18.00	lobChIP_FOXA1inPBS
chr1	169818960	169818961	MACS_peak_1021	7.00	lobChIP_FOXA1inPBS
chr1	170305545	170305546	MACS_peak_1022	9.00	lobChIP_FOXA1inPBS
chr1	170503880	170503881	MACS_peak_1023	11.00	lobChIP_FOXA1inPBS
chr1	172238443	172238444	MACS_peak_1024	25.00	lobChIP_FOXA1inPBS
chr1	172329880	172329881	MACS_peak_1025	32.00	lobChIP_FOXA1inPBS
chr1	172368750	172368751	MACS_peak_1026	14.00	lobChIP_FOXA1inPBS
chr1	172401208	172401209	MACS_peak_1027	7.00	lobChIP_FOXA1inPBS
chr1	172420448	172420449	MACS_peak_1028	11.00	lobChIP_FOXA1inPBS
chr1	172439452	172439453	MACS_peak_1029	28.00	lobChIP_FOXA1inPBS
chr1	172526642	172526643	MACS_peak_1030	10.00	lobChIP_FOXA1inPBS
chr1	172550877	172550878	MACS_peak_1031	7.00	lobChIP_FOXA1inPBS
chr1	172570681	172570682	MACS_peak_1032	6.00	lobChIP_FOXA1inPBS
chr1	172938910	172938911	MACS_peak_1033	13.00	lobChIP_FOXA1inPBS
chr1	172945793	172945794	MACS_peak_1034	15.00	lobChIP_FOXA1inPBS
chr1	173097167	173097168	MACS_peak_1035	10.00	lobChIP_FOXA1inPBS
chr1	173444599	173444600	MACS_peak_1036	6.00	lobChIP_FOXA1inPBS
chr1	173684005	173684006	MACS_peak_1037	7.00	lobChIP_FOXA1inPBS
chr1	173990797	173990798	MACS_peak_1038	6.00	lobChIP_FOXA1inPBS
chr1	174227382	174227383	MACS_peak_1039	10.00	lobChIP_FOXA1inPBS
chr1	174236566	174236567	MACS_peak_1040	58.00	lobChIP_FOXA1inPBS
chr1	174635548	174635549	MACS_peak_1041	24.00	lobChIP_FOXA1inPBS
chr1	174782002	174782003	MACS_peak_1042	58.00	lobChIP_FOXA1inPBS
chr1	174923067	174923068	MACS_peak_1043	12.00	lobChIP_FOXA1inPBS
chr1	174992590	174992591	MACS_peak_1044	10.00	lobChIP_FOXA1inPBS
chr1	175009643	175009644	MACS_peak_1045	9.00	lobChIP_FOXA1inPBS
chr1	175121328	175121329	MACS_peak_1046	7.00	lobChIP_FOXA1inPBS
chr1	175124116	175124117	MACS_peak_1047	10.00	lobChIP_FOXA1inPBS
chr1	175809279	175809280	MACS_peak_1048	14.00	lobChIP_FOXA1inPBS
chr1	175979271	175979272	MACS_peak_1049	13.00	lobChIP_FOXA1inPBS
chr1	176119698	176119699	MACS_peak_1050	23.00	lobChIP_FOXA1inPBS
chr1	177541082	177541083	MACS_peak_1051	33.00	lobChIP_FOXA1inPBS
chr1	177616681	177616682	MACS_peak_1052	10.00	lobChIP_FOXA1inPBS
chr1	177841823	177841824	MACS_peak_1053	29.00	lobChIP_FOXA1inPBS
chr1	178022165	178022166	MACS_peak_1054	10.00	lobChIP_FOXA1inPBS
chr1	178061535	178061536	MACS_peak_1055	10.00	lobChIP_FOXA1inPBS
chr1	178159176	178159177	MACS_peak_1056	8.00	lobChIP_FOXA1inPBS
chr1	178520638	178520639	MACS_peak_1057	9.00	lobChIP_FOXA1inPBS
chr1	178590178	178590179	MACS_peak_1058	12.00	lobChIP_FOXA1inPBS
chr1	178651794	178651795	MACS_peak_1059	7.00	lobChIP_FOXA1inPBS
chr1	178714598	178714599	MACS_peak_1060	7.00	lobChIP_FOXA1inPBS
chr1	178715624	178715625	MACS_peak_1061	9.00	lobChIP_FOXA1inPBS
chr1	178745783	178745784	MACS_peak_1062	5.00	lobChIP_FOXA1inPBS
chr1	178886129	178886130	MACS_peak_1063	20.00	lobChIP_FOXA1inPBS
chr1	178888931	178888932	MACS_peak_1064	32.00	lobChIP_FOXA1inPBS
chr1	178892800	178892801	MACS_peak_1065	5.00	lobChIP_FOXA1inPBS
chr1	179074196	179074197	MACS_peak_1066	9.00	lobChIP_FOXA1inPBS
chr1	179121274	179121275	MACS_peak_1067	25.00	lobChIP_FOXA1inPBS
chr1	179196004	179196005	MACS_peak_1068	8.00	lobChIP_FOXA1inPBS
chr1	179312043	179312044	MACS_peak_1069	10.00	lobChIP_FOXA1inPBS
chr1	179339062	179339063	MACS_peak_1070	15.00	lobChIP_FOXA1inPBS
chr1	179353356	179353357	MACS_peak_1071	33.00	lobChIP_FOXA1inPBS
chr1	179568919	179568920	MACS_peak_1072	24.00	lobChIP_FOXA1inPBS
chr1	179842734	179842735	MACS_peak_1073	9.00	lobChIP_FOXA1inPBS
chr1	179850493	179850494	MACS_peak_1074	5.00	lobChIP_FOXA1inPBS
chr1	180126989	180126990	MACS_peak_1075	18.00	lobChIP_FOXA1inPBS
chr1	180784550	180784551	MACS_peak_1076	44.00	lobChIP_FOXA1inPBS
chr1	180796525	180796526	MACS_peak_1077	15.00	lobChIP_FOXA1inPBS
chr1	180982445	180982446	MACS_peak_1078	9.00	lobChIP_FOXA1inPBS
chr1	181012182	181012183	MACS_peak_1079	24.00	lobChIP_FOXA1inPBS
chr1	181053469	181053470	MACS_peak_1080	8.00	lobChIP_FOXA1inPBS
chr1	181062233	181062234	MACS_peak_1081	13.00	lobChIP_FOXA1inPBS
chr1	181071230	181071231	MACS_peak_1082	8.00	lobChIP_FOXA1inPBS
chr1	181081808	181081809	MACS_peak_1083	16.00	lobChIP_FOXA1inPBS
chr1	181111602	181111603	MACS_peak_1084	12.00	lobChIP_FOXA1inPBS
chr1	181587942	181587943	MACS_peak_1085	9.00	lobChIP_FOXA1inPBS
chr1	181880292	181880293	MACS_peak_1086	19.00	lobChIP_FOXA1inPBS
chr1	182054096	182054097	MACS_peak_1087	6.00	lobChIP_FOXA1inPBS
chr1	182090138	182090139	MACS_peak_1088	24.00	lobChIP_FOXA1inPBS
chr1	182261134	182261135	MACS_peak_1089	49.00	lobChIP_FOXA1inPBS
chr1	182271091	182271092	MACS_peak_1090	14.00	lobChIP_FOXA1inPBS
chr1	182279024	182279025	MACS_peak_1091	5.00	lobChIP_FOXA1inPBS
chr1	182284538	182284539	MACS_peak_1092	14.00	lobChIP_FOXA1inPBS
chr1	182359031	182359032	MACS_peak_1093	6.00	lobChIP_FOXA1inPBS
chr1	182365976	182365977	MACS_peak_1094	7.00	lobChIP_FOXA1inPBS
chr1	182366581	182366582	MACS_peak_1095	11.00	lobChIP_FOXA1inPBS
chr1	182427158	182427159	MACS_peak_1096	9.00	lobChIP_FOXA1inPBS
chr1	182994948	182994949	MACS_peak_1097	17.00	lobChIP_FOXA1inPBS
chr1	182999367	182999368	MACS_peak_1098	14.00	lobChIP_FOXA1inPBS
chr1	183058469	183058470	MACS_peak_1099	12.00	lobChIP_FOXA1inPBS
chr1	183108948	183108949	MACS_peak_1100	11.00	lobChIP_FOXA1inPBS
chr1	183601464	183601465	MACS_peak_1101	9.00	lobChIP_FOXA1inPBS
chr1	183609556	183609557	MACS_peak_1102	27.00	lobChIP_FOXA1inPBS
chr1	183668912	183668913	MACS_peak_1103	41.00	lobChIP_FOXA1inPBS
chr1	183703817	183703818	MACS_peak_1104	10.00	lobChIP_FOXA1inPBS
chr1	183746641	183746642	MACS_peak_1105	6.00	lobChIP_FOXA1inPBS
chr1	183797505	183797506	MACS_peak_1106	8.00	lobChIP_FOXA1inPBS
chr1	183861554	183861555	MACS_peak_1107	8.00	lobChIP_FOXA1inPBS
chr1	183962491	183962492	MACS_peak_1108	7.00	lobChIP_FOXA1inPBS
chr1	184155541	184155542	MACS_peak_1109	8.00	lobChIP_FOXA1inPBS
chr1	184709683	184709684	MACS_peak_1110	9.00	lobChIP_FOXA1inPBS
chr1	184728444	184728445	MACS_peak_1111	13.00	lobChIP_FOXA1inPBS
chr1	184874905	184874906	MACS_peak_1112	19.00	lobChIP_FOXA1inPBS
chr1	184944475	184944476	MACS_peak_1113	22.00	lobChIP_FOXA1inPBS
chr1	185203572	185203573	MACS_peak_1114	13.00	lobChIP_FOXA1inPBS
chr1	185555207	185555208	MACS_peak_1115	16.00	lobChIP_FOXA1inPBS
chr1	185615579	185615580	MACS_peak_1116	25.00	lobChIP_FOXA1inPBS
chr1	185643545	185643546	MACS_peak_1117	16.00	lobChIP_FOXA1inPBS
chr1	187350673	187350674	MACS_peak_1118	8.00	lobChIP_FOXA1inPBS
chr1	190637169	190637170	MACS_peak_1119	8.00	lobChIP_FOXA1inPBS
chr1	192671482	192671483	MACS_peak_1120	17.00	lobChIP_FOXA1inPBS
chr1	192977083	192977084	MACS_peak_1121	85.00	lobChIP_FOXA1inPBS
chr1	193005687	193005688	MACS_peak_1122	16.00	lobChIP_FOXA1inPBS
chr1	193083938	193083939	MACS_peak_1123	10.00	lobChIP_FOXA1inPBS
chr1	193279867	193279868	MACS_peak_1124	8.00	lobChIP_FOXA1inPBS
chr1	196370782	196370783	MACS_peak_1125	6.00	lobChIP_FOXA1inPBS
chr1	196407774	196407775	MACS_peak_1126	24.00	lobChIP_FOXA1inPBS
chr1	196961210	196961211	MACS_peak_1127	8.00	lobChIP_FOXA1inPBS
chr1	197036731	197036732	MACS_peak_1128	8.00	lobChIP_FOXA1inPBS
chr1	197143430	197143431	MACS_peak_1129	7.00	lobChIP_FOXA1inPBS
chr1	197279294	197279295	MACS_peak_1130	7.00	lobChIP_FOXA1inPBS
chr1	197411345	197411346	MACS_peak_1131	7.00	lobChIP_FOXA1inPBS
chr1	197589975	197589976	MACS_peak_1132	22.00	lobChIP_FOXA1inPBS
chr1	197702489	197702490	MACS_peak_1133	8.00	lobChIP_FOXA1inPBS
chr1	197853392	197853393	MACS_peak_1134	6.00	lobChIP_FOXA1inPBS
chr1	197924098	197924099	MACS_peak_1135	7.00	lobChIP_FOXA1inPBS
chr1	197964393	197964394	MACS_peak_1136	8.00	lobChIP_FOXA1inPBS
chr1	198503885	198503886	MACS_peak_1137	31.00	lobChIP_FOXA1inPBS
chr1	198668531	198668532	MACS_peak_1138	8.00	lobChIP_FOXA1inPBS
chr1	199270766	199270767	MACS_peak_1139	10.00	lobChIP_FOXA1inPBS
chr1	199518764	199518765	MACS_peak_1140	7.00	lobChIP_FOXA1inPBS
chr1	200100962	200100963	MACS_peak_1141	8.00	lobChIP_FOXA1inPBS
chr1	200229867	200229868	MACS_peak_1142	11.00	lobChIP_FOXA1inPBS
chr1	200232421	200232422	MACS_peak_1143	8.00	lobChIP_FOXA1inPBS
chr1	200244762	200244763	MACS_peak_1144	11.00	lobChIP_FOXA1inPBS
chr1	200265950	200265951	MACS_peak_1145	8.00	lobChIP_FOXA1inPBS
chr1	200283602	200283603	MACS_peak_1146	7.00	lobChIP_FOXA1inPBS
chr1	200305736	200305737	MACS_peak_1147	28.00	lobChIP_FOXA1inPBS
chr1	200307524	200307525	MACS_peak_1148	14.00	lobChIP_FOXA1inPBS
chr1	200341301	200341302	MACS_peak_1149	97.00	lobChIP_FOXA1inPBS
chr1	200343129	200343130	MACS_peak_1150	12.00	lobChIP_FOXA1inPBS
chr1	200349591	200349592	MACS_peak_1151	12.00	lobChIP_FOXA1inPBS
chr1	200374577	200374578	MACS_peak_1152	9.00	lobChIP_FOXA1inPBS
chr1	200380208	200380209	MACS_peak_1153	12.00	lobChIP_FOXA1inPBS
chr1	200711995	200711996	MACS_peak_1154	10.00	lobChIP_FOXA1inPBS
chr1	201139857	201139858	MACS_peak_1155	8.00	lobChIP_FOXA1inPBS
chr1	201140885	201140886	MACS_peak_1156	5.00	lobChIP_FOXA1inPBS
chr1	201144506	201144507	MACS_peak_1157	10.00	lobChIP_FOXA1inPBS
chr1	201354657	201354658	MACS_peak_1158	13.00	lobChIP_FOXA1inPBS
chr1	201465197	201465198	MACS_peak_1159	10.00	lobChIP_FOXA1inPBS
chr1	201811810	201811811	MACS_peak_1160	12.00	lobChIP_FOXA1inPBS
chr1	201975373	201975374	MACS_peak_1161	12.00	lobChIP_FOXA1inPBS
chr1	201987978	201987979	MACS_peak_1162	13.00	lobChIP_FOXA1inPBS
chr1	202016367	202016368	MACS_peak_1163	18.00	lobChIP_FOXA1inPBS
chr1	202027600	202027601	MACS_peak_1164	13.00	lobChIP_FOXA1inPBS
chr1	202032456	202032457	MACS_peak_1165	12.00	lobChIP_FOXA1inPBS
chr1	202083248	202083249	MACS_peak_1166	10.00	lobChIP_FOXA1inPBS
chr1	202158703	202158704	MACS_peak_1167	13.00	lobChIP_FOXA1inPBS
chr1	202435173	202435174	MACS_peak_1168	10.00	lobChIP_FOXA1inPBS
chr1	202506516	202506517	MACS_peak_1169	21.00	lobChIP_FOXA1inPBS
chr1	202779927	202779928	MACS_peak_1170	9.00	lobChIP_FOXA1inPBS
chr1	203158043	203158044	MACS_peak_1171	16.00	lobChIP_FOXA1inPBS
chr1	203180268	203180269	MACS_peak_1172	51.00	lobChIP_FOXA1inPBS
chr1	203260491	203260492	MACS_peak_1173	6.00	lobChIP_FOXA1inPBS
chr1	203831210	203831211	MACS_peak_1174	13.00	lobChIP_FOXA1inPBS
chr1	204047666	204047667	MACS_peak_1175	12.00	lobChIP_FOXA1inPBS
chr1	204071357	204071358	MACS_peak_1176	39.00	lobChIP_FOXA1inPBS
chr1	204140954	204140955	MACS_peak_1177	11.00	lobChIP_FOXA1inPBS
chr1	204183577	204183578	MACS_peak_1178	18.00	lobChIP_FOXA1inPBS
chr1	204223410	204223411	MACS_peak_1179	13.00	lobChIP_FOXA1inPBS
chr1	204367756	204367757	MACS_peak_1180	35.00	lobChIP_FOXA1inPBS
chr1	204386408	204386409	MACS_peak_1181	9.00	lobChIP_FOXA1inPBS
chr1	204476126	204476127	MACS_peak_1182	9.00	lobChIP_FOXA1inPBS
chr1	204888064	204888065	MACS_peak_1183	7.00	lobChIP_FOXA1inPBS
chr1	205270294	205270295	MACS_peak_1184	10.00	lobChIP_FOXA1inPBS
chr1	205287927	205287928	MACS_peak_1185	11.00	lobChIP_FOXA1inPBS
chr1	205454712	205454713	MACS_peak_1186	9.00	lobChIP_FOXA1inPBS
chr1	205461511	205461512	MACS_peak_1187	15.00	lobChIP_FOXA1inPBS
chr1	205604724	205604725	MACS_peak_1188	16.00	lobChIP_FOXA1inPBS
chr1	205650452	205650453	MACS_peak_1189	8.00	lobChIP_FOXA1inPBS
chr1	205672367	205672368	MACS_peak_1190	27.00	lobChIP_FOXA1inPBS
chr1	205678296	205678297	MACS_peak_1191	7.00	lobChIP_FOXA1inPBS
chr1	205695807	205695808	MACS_peak_1192	6.00	lobChIP_FOXA1inPBS
chr1	205709111	205709112	MACS_peak_1193	30.00	lobChIP_FOXA1inPBS
chr1	206285446	206285447	MACS_peak_1194	7.00	lobChIP_FOXA1inPBS
chr1	206729736	206729737	MACS_peak_1195	11.00	lobChIP_FOXA1inPBS
chr1	206873245	206873246	MACS_peak_1196	8.00	lobChIP_FOXA1inPBS
chr1	206883606	206883607	MACS_peak_1197	7.00	lobChIP_FOXA1inPBS
chr1	207199115	207199116	MACS_peak_1198	17.00	lobChIP_FOXA1inPBS
chr1	207262459	207262460	MACS_peak_1199	25.00	lobChIP_FOXA1inPBS
chr1	207277472	207277473	MACS_peak_1200	13.00	lobChIP_FOXA1inPBS
chr1	207302018	207302019	MACS_peak_1201	8.00	lobChIP_FOXA1inPBS
chr1	207414752	207414753	MACS_peak_1202	14.00	lobChIP_FOXA1inPBS
chr1	207777307	207777308	MACS_peak_1203	27.00	lobChIP_FOXA1inPBS
chr1	207999939	207999940	MACS_peak_1204	12.00	lobChIP_FOXA1inPBS
chr1	208293438	208293439	MACS_peak_1205	10.00	lobChIP_FOXA1inPBS
chr1	208345572	208345573	MACS_peak_1206	10.00	lobChIP_FOXA1inPBS
chr1	208377020	208377021	MACS_peak_1207	19.00	lobChIP_FOXA1inPBS
chr1	210076120	210076121	MACS_peak_1208	9.00	lobChIP_FOXA1inPBS
chr1	210547397	210547398	MACS_peak_1209	24.00	lobChIP_FOXA1inPBS
chr1	211694943	211694944	MACS_peak_1210	11.00	lobChIP_FOXA1inPBS
chr1	211753344	211753345	MACS_peak_1211	8.00	lobChIP_FOXA1inPBS
chr1	211776896	211776897	MACS_peak_1212	14.00	lobChIP_FOXA1inPBS
chr1	211778086	211778087	MACS_peak_1213	30.00	lobChIP_FOXA1inPBS
chr1	211817285	211817286	MACS_peak_1214	16.00	lobChIP_FOXA1inPBS
chr1	211840654	211840655	MACS_peak_1215	31.00	lobChIP_FOXA1inPBS
chr1	211963589	211963590	MACS_peak_1216	9.00	lobChIP_FOXA1inPBS
chr1	212059664	212059665	MACS_peak_1217	13.00	lobChIP_FOXA1inPBS
chr1	212068896	212068897	MACS_peak_1218	7.00	lobChIP_FOXA1inPBS
chr1	212070573	212070574	MACS_peak_1219	9.00	lobChIP_FOXA1inPBS
chr1	212091347	212091348	MACS_peak_1220	12.00	lobChIP_FOXA1inPBS
chr1	212102549	212102550	MACS_peak_1221	7.00	lobChIP_FOXA1inPBS
chr1	212402297	212402298	MACS_peak_1222	18.00	lobChIP_FOXA1inPBS
chr1	212431610	212431611	MACS_peak_1223	23.00	lobChIP_FOXA1inPBS
chr1	212464970	212464971	MACS_peak_1224	28.00	lobChIP_FOXA1inPBS
chr1	212483296	212483297	MACS_peak_1225	6.00	lobChIP_FOXA1inPBS
chr1	212968275	212968276	MACS_peak_1226	12.00	lobChIP_FOXA1inPBS
chr1	212990257	212990258	MACS_peak_1227	12.00	lobChIP_FOXA1inPBS
chr1	213001194	213001195	MACS_peak_1228	9.00	lobChIP_FOXA1inPBS
chr1	213311301	213311302	MACS_peak_1229	10.00	lobChIP_FOXA1inPBS
chr1	213545745	213545746	MACS_peak_1230	53.00	lobChIP_FOXA1inPBS
chr1	213560890	213560891	MACS_peak_1231	9.00	lobChIP_FOXA1inPBS
chr1	213567532	213567533	MACS_peak_1232	15.00	lobChIP_FOXA1inPBS
chr1	213665740	213665741	MACS_peak_1233	17.00	lobChIP_FOXA1inPBS
chr1	213742766	213742767	MACS_peak_1234	9.00	lobChIP_FOXA1inPBS
chr1	213746476	213746477	MACS_peak_1235	17.00	lobChIP_FOXA1inPBS
chr1	213756511	213756512	MACS_peak_1236	7.00	lobChIP_FOXA1inPBS
chr1	213824153	213824154	MACS_peak_1237	8.00	lobChIP_FOXA1inPBS
chr1	213907239	213907240	MACS_peak_1238	14.00	lobChIP_FOXA1inPBS
chr1	213910931	213910932	MACS_peak_1239	6.00	lobChIP_FOXA1inPBS
chr1	213933359	213933360	MACS_peak_1240	24.00	lobChIP_FOXA1inPBS
chr1	213967098	213967099	MACS_peak_1241	14.00	lobChIP_FOXA1inPBS
chr1	214032263	214032264	MACS_peak_1242	9.00	lobChIP_FOXA1inPBS
chr1	214129061	214129062	MACS_peak_1243	9.00	lobChIP_FOXA1inPBS
chr1	214129934	214129935	MACS_peak_1244	55.00	lobChIP_FOXA1inPBS
chr1	214131911	214131912	MACS_peak_1245	9.00	lobChIP_FOXA1inPBS
chr1	214150215	214150216	MACS_peak_1246	7.00	lobChIP_FOXA1inPBS
chr1	214201058	214201059	MACS_peak_1247	19.00	lobChIP_FOXA1inPBS
chr1	214202431	214202432	MACS_peak_1248	25.00	lobChIP_FOXA1inPBS
chr1	214212882	214212883	MACS_peak_1249	11.00	lobChIP_FOXA1inPBS
chr1	214218094	214218095	MACS_peak_1250	15.00	lobChIP_FOXA1inPBS
chr1	214224465	214224466	MACS_peak_1251	34.00	lobChIP_FOXA1inPBS
chr1	214242127	214242128	MACS_peak_1252	15.00	lobChIP_FOXA1inPBS
chr1	214248783	214248784	MACS_peak_1253	9.00	lobChIP_FOXA1inPBS
chr1	214456737	214456738	MACS_peak_1254	9.00	lobChIP_FOXA1inPBS
chr1	214458362	214458363	MACS_peak_1255	30.00	lobChIP_FOXA1inPBS
chr1	214489978	214489979	MACS_peak_1256	7.00	lobChIP_FOXA1inPBS
chr1	214588198	214588199	MACS_peak_1257	12.00	lobChIP_FOXA1inPBS
chr1	214613168	214613169	MACS_peak_1258	7.00	lobChIP_FOXA1inPBS
chr1	214614285	214614286	MACS_peak_1259	8.00	lobChIP_FOXA1inPBS
chr1	214633798	214633799	MACS_peak_1260	13.00	lobChIP_FOXA1inPBS
chr1	214634343	214634344	MACS_peak_1261	10.00	lobChIP_FOXA1inPBS
chr1	214680454	214680455	MACS_peak_1262	12.00	lobChIP_FOXA1inPBS
chr1	214689941	214689942	MACS_peak_1263	14.00	lobChIP_FOXA1inPBS
chr1	214851884	214851885	MACS_peak_1264	29.00	lobChIP_FOXA1inPBS
chr1	215006897	215006898	MACS_peak_1265	42.00	lobChIP_FOXA1inPBS
chr1	215700033	215700034	MACS_peak_1266	13.00	lobChIP_FOXA1inPBS
chr1	217553356	217553357	MACS_peak_1267	12.00	lobChIP_FOXA1inPBS
chr1	218077385	218077386	MACS_peak_1268	11.00	lobChIP_FOXA1inPBS
chr1	218431474	218431475	MACS_peak_1269	8.00	lobChIP_FOXA1inPBS
chr1	218540193	218540194	MACS_peak_1270	22.00	lobChIP_FOXA1inPBS
chr1	218545781	218545782	MACS_peak_1271	7.00	lobChIP_FOXA1inPBS
chr1	218550286	218550287	MACS_peak_1272	10.00	lobChIP_FOXA1inPBS
chr1	218553904	218553905	MACS_peak_1273	6.00	lobChIP_FOXA1inPBS
chr1	218575727	218575728	MACS_peak_1274	25.00	lobChIP_FOXA1inPBS
chr1	219344665	219344666	MACS_peak_1275	8.00	lobChIP_FOXA1inPBS
chr1	219483854	219483855	MACS_peak_1276	14.00	lobChIP_FOXA1inPBS
chr1	219504226	219504227	MACS_peak_1277	15.00	lobChIP_FOXA1inPBS
chr1	219587754	219587755	MACS_peak_1278	10.00	lobChIP_FOXA1inPBS
chr1	220057336	220057337	MACS_peak_1279	8.00	lobChIP_FOXA1inPBS
chr1	220100266	220100267	MACS_peak_1280	9.00	lobChIP_FOXA1inPBS
chr1	220218990	220218991	MACS_peak_1281	8.00	lobChIP_FOXA1inPBS
chr1	220263169	220263170	MACS_peak_1282	14.00	lobChIP_FOXA1inPBS
chr1	220630423	220630424	MACS_peak_1283	7.00	lobChIP_FOXA1inPBS
chr1	220908018	220908019	MACS_peak_1284	36.00	lobChIP_FOXA1inPBS
chr1	220941664	220941665	MACS_peak_1285	11.00	lobChIP_FOXA1inPBS
chr1	221092126	221092127	MACS_peak_1286	13.00	lobChIP_FOXA1inPBS
chr1	221508761	221508762	MACS_peak_1287	9.00	lobChIP_FOXA1inPBS
chr1	221888989	221888990	MACS_peak_1288	16.00	lobChIP_FOXA1inPBS
chr1	221891989	221891990	MACS_peak_1289	17.00	lobChIP_FOXA1inPBS
chr1	221915726	221915727	MACS_peak_1290	13.00	lobChIP_FOXA1inPBS
chr1	222235444	222235445	MACS_peak_1291	10.00	lobChIP_FOXA1inPBS
chr1	222695621	222695622	MACS_peak_1292	10.00	lobChIP_FOXA1inPBS
chr1	222719551	222719552	MACS_peak_1293	11.00	lobChIP_FOXA1inPBS
chr1	223060150	223060151	MACS_peak_1294	8.00	lobChIP_FOXA1inPBS
chr1	223184517	223184518	MACS_peak_1295	8.00	lobChIP_FOXA1inPBS
chr1	223219618	223219619	MACS_peak_1296	14.00	lobChIP_FOXA1inPBS
chr1	223351224	223351225	MACS_peak_1297	12.00	lobChIP_FOXA1inPBS
chr1	223591034	223591035	MACS_peak_1298	9.00	lobChIP_FOXA1inPBS
chr1	223841921	223841922	MACS_peak_1299	10.00	lobChIP_FOXA1inPBS
chr1	223898391	223898392	MACS_peak_1300	19.00	lobChIP_FOXA1inPBS
chr1	223907333	223907334	MACS_peak_1301	30.00	lobChIP_FOXA1inPBS
chr1	223908781	223908782	MACS_peak_1302	13.00	lobChIP_FOXA1inPBS
chr1	223953784	223953785	MACS_peak_1303	7.00	lobChIP_FOXA1inPBS
chr1	224002916	224002917	MACS_peak_1304	6.00	lobChIP_FOXA1inPBS
chr1	224238519	224238520	MACS_peak_1305	6.00	lobChIP_FOXA1inPBS
chr1	224294903	224294904	MACS_peak_1306	14.00	lobChIP_FOXA1inPBS
chr1	224302676	224302677	MACS_peak_1307	10.00	lobChIP_FOXA1inPBS
chr1	224626874	224626875	MACS_peak_1308	10.00	lobChIP_FOXA1inPBS
chr1	224969632	224969633	MACS_peak_1309	7.00	lobChIP_FOXA1inPBS
chr1	225133907	225133908	MACS_peak_1310	15.00	lobChIP_FOXA1inPBS
chr1	225169872	225169873	MACS_peak_1311	22.00	lobChIP_FOXA1inPBS
chr1	225396967	225396968	MACS_peak_1312	12.00	lobChIP_FOXA1inPBS
chr1	225398112	225398113	MACS_peak_1313	11.00	lobChIP_FOXA1inPBS
chr1	225416740	225416741	MACS_peak_1314	43.00	lobChIP_FOXA1inPBS
chr1	225600707	225600708	MACS_peak_1315	7.00	lobChIP_FOXA1inPBS
chr1	225642020	225642021	MACS_peak_1316	9.00	lobChIP_FOXA1inPBS
chr1	225696301	225696302	MACS_peak_1317	8.00	lobChIP_FOXA1inPBS
chr1	225791323	225791324	MACS_peak_1318	11.00	lobChIP_FOXA1inPBS
chr1	225795324	225795325	MACS_peak_1319	9.00	lobChIP_FOXA1inPBS
chr1	225859306	225859307	MACS_peak_1320	11.00	lobChIP_FOXA1inPBS
chr1	225882064	225882065	MACS_peak_1321	20.00	lobChIP_FOXA1inPBS
chr1	225883796	225883797	MACS_peak_1322	8.00	lobChIP_FOXA1inPBS
chr1	225888121	225888122	MACS_peak_1323	28.00	lobChIP_FOXA1inPBS
chr1	225927478	225927479	MACS_peak_1324	7.00	lobChIP_FOXA1inPBS
chr1	225950360	225950361	MACS_peak_1325	10.00	lobChIP_FOXA1inPBS
chr1	225956527	225956528	MACS_peak_1326	7.00	lobChIP_FOXA1inPBS
chr1	225960081	225960082	MACS_peak_1327	6.00	lobChIP_FOXA1inPBS
chr1	226001272	226001273	MACS_peak_1328	12.00	lobChIP_FOXA1inPBS
chr1	226008796	226008797	MACS_peak_1329	21.00	lobChIP_FOXA1inPBS
chr1	226066194	226066195	MACS_peak_1330	12.00	lobChIP_FOXA1inPBS
chr1	226078952	226078953	MACS_peak_1331	9.00	lobChIP_FOXA1inPBS
chr1	226456979	226456980	MACS_peak_1332	8.00	lobChIP_FOXA1inPBS
chr1	226667286	226667287	MACS_peak_1333	68.00	lobChIP_FOXA1inPBS
chr1	227133168	227133169	MACS_peak_1334	10.00	lobChIP_FOXA1inPBS
chr1	227516274	227516275	MACS_peak_1335	11.00	lobChIP_FOXA1inPBS
chr1	227619835	227619836	MACS_peak_1336	7.00	lobChIP_FOXA1inPBS
chr1	228667723	228667724	MACS_peak_1337	28.00	lobChIP_FOXA1inPBS
chr1	228800307	228800308	MACS_peak_1338	10.00	lobChIP_FOXA1inPBS
chr1	228872395	228872396	MACS_peak_1339	6.00	lobChIP_FOXA1inPBS
chr1	228972435	228972436	MACS_peak_1340	18.00	lobChIP_FOXA1inPBS
chr1	228974391	228974392	MACS_peak_1341	18.00	lobChIP_FOXA1inPBS
chr1	228989688	228989689	MACS_peak_1342	12.00	lobChIP_FOXA1inPBS
chr1	229020813	229020814	MACS_peak_1343	9.00	lobChIP_FOXA1inPBS
chr1	229100463	229100464	MACS_peak_1344	12.00	lobChIP_FOXA1inPBS
chr1	229133713	229133714	MACS_peak_1345	6.00	lobChIP_FOXA1inPBS
chr1	229139497	229139498	MACS_peak_1346	11.00	lobChIP_FOXA1inPBS
chr1	229235045	229235046	MACS_peak_1347	16.00	lobChIP_FOXA1inPBS
chr1	229238875	229238876	MACS_peak_1348	8.00	lobChIP_FOXA1inPBS
chr1	229247855	229247856	MACS_peak_1349	8.00	lobChIP_FOXA1inPBS
chr1	229267282	229267283	MACS_peak_1350	9.00	lobChIP_FOXA1inPBS
chr1	229284165	229284166	MACS_peak_1351	18.00	lobChIP_FOXA1inPBS
chr1	229357457	229357458	MACS_peak_1352	17.00	lobChIP_FOXA1inPBS
chr1	229407499	229407500	MACS_peak_1353	12.00	lobChIP_FOXA1inPBS
chr1	229409706	229409707	MACS_peak_1354	43.00	lobChIP_FOXA1inPBS
chr1	229435144	229435145	MACS_peak_1355	23.00	lobChIP_FOXA1inPBS
chr1	229645485	229645486	MACS_peak_1356	32.00	lobChIP_FOXA1inPBS
chr1	229708179	229708180	MACS_peak_1357	6.00	lobChIP_FOXA1inPBS
chr1	230115872	230115873	MACS_peak_1358	8.00	lobChIP_FOXA1inPBS
chr1	230256838	230256839	MACS_peak_1359	12.00	lobChIP_FOXA1inPBS
chr1	230289846	230289847	MACS_peak_1360	7.00	lobChIP_FOXA1inPBS
chr1	230294882	230294883	MACS_peak_1361	10.00	lobChIP_FOXA1inPBS
chr1	230501663	230501664	MACS_peak_1362	10.00	lobChIP_FOXA1inPBS
chr1	230775613	230775614	MACS_peak_1363	19.00	lobChIP_FOXA1inPBS
chr1	230816565	230816566	MACS_peak_1364	23.00	lobChIP_FOXA1inPBS
chr1	230861208	230861209	MACS_peak_1365	21.00	lobChIP_FOXA1inPBS
chr1	230864866	230864867	MACS_peak_1366	8.00	lobChIP_FOXA1inPBS
chr1	230866997	230866998	MACS_peak_1367	14.00	lobChIP_FOXA1inPBS
chr1	230880360	230880361	MACS_peak_1368	14.00	lobChIP_FOXA1inPBS
chr1	230921583	230921584	MACS_peak_1369	16.00	lobChIP_FOXA1inPBS
chr1	231084789	231084790	MACS_peak_1370	11.00	lobChIP_FOXA1inPBS
chr1	231086328	231086329	MACS_peak_1371	9.00	lobChIP_FOXA1inPBS
chr1	231100828	231100829	MACS_peak_1372	23.00	lobChIP_FOXA1inPBS
chr1	231355521	231355522	MACS_peak_1373	11.00	lobChIP_FOXA1inPBS
chr1	231369053	231369054	MACS_peak_1374	21.00	lobChIP_FOXA1inPBS
chr1	231369887	231369888	MACS_peak_1375	22.00	lobChIP_FOXA1inPBS
chr1	231372007	231372008	MACS_peak_1376	17.00	lobChIP_FOXA1inPBS
chr1	231521302	231521303	MACS_peak_1377	11.00	lobChIP_FOXA1inPBS
chr1	231559628	231559629	MACS_peak_1378	61.00	lobChIP_FOXA1inPBS
chr1	231630254	231630255	MACS_peak_1379	9.00	lobChIP_FOXA1inPBS
chr1	231713024	231713025	MACS_peak_1380	7.00	lobChIP_FOXA1inPBS
chr1	231764080	231764081	MACS_peak_1381	7.00	lobChIP_FOXA1inPBS
chr1	231810485	231810486	MACS_peak_1382	12.00	lobChIP_FOXA1inPBS
chr1	231813958	231813959	MACS_peak_1383	9.00	lobChIP_FOXA1inPBS
chr1	231884724	231884725	MACS_peak_1384	13.00	lobChIP_FOXA1inPBS
chr1	231964102	231964103	MACS_peak_1385	9.00	lobChIP_FOXA1inPBS
chr1	232070690	232070691	MACS_peak_1386	23.00	lobChIP_FOXA1inPBS
chr1	232300287	232300288	MACS_peak_1387	38.00	lobChIP_FOXA1inPBS
chr1	234449871	234449872	MACS_peak_1388	6.00	lobChIP_FOXA1inPBS
chr1	234491769	234491770	MACS_peak_1389	10.00	lobChIP_FOXA1inPBS
chr1	234524872	234524873	MACS_peak_1390	11.00	lobChIP_FOXA1inPBS
chr1	234594303	234594304	MACS_peak_1391	20.00	lobChIP_FOXA1inPBS
chr1	234698131	234698132	MACS_peak_1392	12.00	lobChIP_FOXA1inPBS
chr1	234736466	234736467	MACS_peak_1393	7.00	lobChIP_FOXA1inPBS
chr1	234742056	234742057	MACS_peak_1394	12.00	lobChIP_FOXA1inPBS
chr1	234747194	234747195	MACS_peak_1395	6.00	lobChIP_FOXA1inPBS
chr1	234748365	234748366	MACS_peak_1396	18.00	lobChIP_FOXA1inPBS
chr1	234750207	234750208	MACS_peak_1397	7.00	lobChIP_FOXA1inPBS
chr1	234755714	234755715	MACS_peak_1398	22.00	lobChIP_FOXA1inPBS
chr1	234756983	234756984	MACS_peak_1399	18.00	lobChIP_FOXA1inPBS
chr1	234767661	234767662	MACS_peak_1400	9.00	lobChIP_FOXA1inPBS
chr1	234775405	234775406	MACS_peak_1401	8.00	lobChIP_FOXA1inPBS
chr1	234776852	234776853	MACS_peak_1402	7.00	lobChIP_FOXA1inPBS
chr1	234779150	234779151	MACS_peak_1403	42.00	lobChIP_FOXA1inPBS
chr1	234852220	234852221	MACS_peak_1404	22.00	lobChIP_FOXA1inPBS
chr1	234854048	234854049	MACS_peak_1405	9.00	lobChIP_FOXA1inPBS
chr1	234860213	234860214	MACS_peak_1406	12.00	lobChIP_FOXA1inPBS
chr1	234904060	234904061	MACS_peak_1407	14.00	lobChIP_FOXA1inPBS
chr1	234917261	234917262	MACS_peak_1408	9.00	lobChIP_FOXA1inPBS
chr1	235010469	235010470	MACS_peak_1409	6.00	lobChIP_FOXA1inPBS
chr1	235012432	235012433	MACS_peak_1410	6.00	lobChIP_FOXA1inPBS
chr1	235027341	235027342	MACS_peak_1411	12.00	lobChIP_FOXA1inPBS
chr1	235089549	235089550	MACS_peak_1412	13.00	lobChIP_FOXA1inPBS
chr1	235093282	235093283	MACS_peak_1413	12.00	lobChIP_FOXA1inPBS
chr1	235096582	235096583	MACS_peak_1414	41.00	lobChIP_FOXA1inPBS
chr1	235099390	235099391	MACS_peak_1415	45.00	lobChIP_FOXA1inPBS
chr1	235111048	235111049	MACS_peak_1416	30.00	lobChIP_FOXA1inPBS
chr1	235114555	235114556	MACS_peak_1417	9.00	lobChIP_FOXA1inPBS
chr1	235324902	235324903	MACS_peak_1418	10.00	lobChIP_FOXA1inPBS
chr1	235413879	235413880	MACS_peak_1419	8.00	lobChIP_FOXA1inPBS
chr1	235459488	235459489	MACS_peak_1420	17.00	lobChIP_FOXA1inPBS
chr1	235572299	235572300	MACS_peak_1421	8.00	lobChIP_FOXA1inPBS
chr1	235575334	235575335	MACS_peak_1422	9.00	lobChIP_FOXA1inPBS
chr1	235794131	235794132	MACS_peak_1423	19.00	lobChIP_FOXA1inPBS
chr1	235987256	235987257	MACS_peak_1424	7.00	lobChIP_FOXA1inPBS
chr1	235989601	235989602	MACS_peak_1425	22.00	lobChIP_FOXA1inPBS
chr1	236056570	236056571	MACS_peak_1426	28.00	lobChIP_FOXA1inPBS
chr1	236109456	236109457	MACS_peak_1427	10.00	lobChIP_FOXA1inPBS
chr1	236203198	236203199	MACS_peak_1428	27.00	lobChIP_FOXA1inPBS
chr1	236236915	236236916	MACS_peak_1429	10.00	lobChIP_FOXA1inPBS
chr1	236238144	236238145	MACS_peak_1430	12.00	lobChIP_FOXA1inPBS
chr1	236423033	236423034	MACS_peak_1431	15.00	lobChIP_FOXA1inPBS
chr1	236552770	236552771	MACS_peak_1432	9.00	lobChIP_FOXA1inPBS
chr1	236562991	236562992	MACS_peak_1433	35.00	lobChIP_FOXA1inPBS
chr1	236654207	236654208	MACS_peak_1434	14.00	lobChIP_FOXA1inPBS
chr1	236655093	236655094	MACS_peak_1435	9.00	lobChIP_FOXA1inPBS
chr1	236656801	236656802	MACS_peak_1436	8.00	lobChIP_FOXA1inPBS
chr1	236678621	236678622	MACS_peak_1437	23.00	lobChIP_FOXA1inPBS
chr1	236680695	236680696	MACS_peak_1438	24.00	lobChIP_FOXA1inPBS
chr1	236686386	236686387	MACS_peak_1439	11.00	lobChIP_FOXA1inPBS
chr1	237008103	237008104	MACS_peak_1440	7.00	lobChIP_FOXA1inPBS
chr1	237008488	237008489	MACS_peak_1441	26.00	lobChIP_FOXA1inPBS
chr1	237026133	237026134	MACS_peak_1442	7.00	lobChIP_FOXA1inPBS
chr1	238591598	238591599	MACS_peak_1443	6.00	lobChIP_FOXA1inPBS
chr1	241161922	241161923	MACS_peak_1444	10.00	lobChIP_FOXA1inPBS
chr1	241685165	241685166	MACS_peak_1445	15.00	lobChIP_FOXA1inPBS
chr1	241835859	241835860	MACS_peak_1446	10.00	lobChIP_FOXA1inPBS
chr1	242008065	242008066	MACS_peak_1447	10.00	lobChIP_FOXA1inPBS
chr1	242374671	242374672	MACS_peak_1448	14.00	lobChIP_FOXA1inPBS
chr1	242905883	242905884	MACS_peak_1449	8.00	lobChIP_FOXA1inPBS
chr1	243669438	243669439	MACS_peak_1450	12.00	lobChIP_FOXA1inPBS
chr1	244065660	244065661	MACS_peak_1451	49.00	lobChIP_FOXA1inPBS
chr1	244436294	244436295	MACS_peak_1452	14.00	lobChIP_FOXA1inPBS
chr1	244491025	244491026	MACS_peak_1453	31.00	lobChIP_FOXA1inPBS
chr1	244525383	244525384	MACS_peak_1454	8.00	lobChIP_FOXA1inPBS
chr1	244723026	244723027	MACS_peak_1455	10.00	lobChIP_FOXA1inPBS
chr1	244883220	244883221	MACS_peak_1456	10.00	lobChIP_FOXA1inPBS
chr1	244997078	244997079	MACS_peak_1457	7.00	lobChIP_FOXA1inPBS
chr1	245134874	245134875	MACS_peak_1458	14.00	lobChIP_FOXA1inPBS
chr1	245208989	245208990	MACS_peak_1459	18.00	lobChIP_FOXA1inPBS
chr1	245224191	245224192	MACS_peak_1460	20.00	lobChIP_FOXA1inPBS
chr1	245320713	245320714	MACS_peak_1461	21.00	lobChIP_FOXA1inPBS
chr1	245421768	245421769	MACS_peak_1462	21.00	lobChIP_FOXA1inPBS
chr1	245974531	245974532	MACS_peak_1463	26.00	lobChIP_FOXA1inPBS
chr1	246329896	246329897	MACS_peak_1464	10.00	lobChIP_FOXA1inPBS
chr1	246339861	246339862	MACS_peak_1465	10.00	lobChIP_FOXA1inPBS
chr1	246383259	246383260	MACS_peak_1466	17.00	lobChIP_FOXA1inPBS
chr1	246445153	246445154	MACS_peak_1467	19.00	lobChIP_FOXA1inPBS
chr1	246746944	246746945	MACS_peak_1468	27.00	lobChIP_FOXA1inPBS
chr1	246750905	246750906	MACS_peak_1469	7.00	lobChIP_FOXA1inPBS
chr1	246772209	246772210	MACS_peak_1470	13.00	lobChIP_FOXA1inPBS
chr1	246873345	246873346	MACS_peak_1471	35.00	lobChIP_FOXA1inPBS
chr1	246889968	246889969	MACS_peak_1472	13.00	lobChIP_FOXA1inPBS
chr1	246964883	246964884	MACS_peak_1473	5.00	lobChIP_FOXA1inPBS
chr1	246968796	246968797	MACS_peak_1474	10.00	lobChIP_FOXA1inPBS
chr1	247083495	247083496	MACS_peak_1475	9.00	lobChIP_FOXA1inPBS
chr1	249123342	249123343	MACS_peak_1476	8.00	lobChIP_FOXA1inPBS
chr1	249167426	249167427	MACS_peak_1477	6.00	lobChIP_FOXA1inPBS
chr10	179566	179567	MACS_peak_1478	9.00	lobChIP_FOXA1inPBS
chr10	434452	434453	MACS_peak_1479	10.00	lobChIP_FOXA1inPBS
chr10	560114	560115	MACS_peak_1480	13.00	lobChIP_FOXA1inPBS
chr10	1016524	1016525	MACS_peak_1481	27.00	lobChIP_FOXA1inPBS
chr10	1020180	1020181	MACS_peak_1482	8.00	lobChIP_FOXA1inPBS
chr10	1153798	1153799	MACS_peak_1483	7.00	lobChIP_FOXA1inPBS
chr10	1249113	1249114	MACS_peak_1484	30.00	lobChIP_FOXA1inPBS
chr10	1474558	1474559	MACS_peak_1485	18.00	lobChIP_FOXA1inPBS
chr10	3137719	3137720	MACS_peak_1486	17.00	lobChIP_FOXA1inPBS
chr10	3235261	3235262	MACS_peak_1487	10.00	lobChIP_FOXA1inPBS
chr10	3337425	3337426	MACS_peak_1488	8.00	lobChIP_FOXA1inPBS
chr10	3407864	3407865	MACS_peak_1489	11.00	lobChIP_FOXA1inPBS
chr10	3782723	3782724	MACS_peak_1490	21.00	lobChIP_FOXA1inPBS
chr10	3786981	3786982	MACS_peak_1491	10.00	lobChIP_FOXA1inPBS
chr10	3790424	3790425	MACS_peak_1492	11.00	lobChIP_FOXA1inPBS
chr10	3826254	3826255	MACS_peak_1493	34.00	lobChIP_FOXA1inPBS
chr10	3848845	3848846	MACS_peak_1494	10.00	lobChIP_FOXA1inPBS
chr10	3852619	3852620	MACS_peak_1495	12.00	lobChIP_FOXA1inPBS
chr10	3858987	3858988	MACS_peak_1496	8.00	lobChIP_FOXA1inPBS
chr10	3867842	3867843	MACS_peak_1497	5.00	lobChIP_FOXA1inPBS
chr10	3873318	3873319	MACS_peak_1498	21.00	lobChIP_FOXA1inPBS
chr10	3881314	3881315	MACS_peak_1499	8.00	lobChIP_FOXA1inPBS
chr10	3893958	3893959	MACS_peak_1500	15.00	lobChIP_FOXA1inPBS
chr10	3921329	3921330	MACS_peak_1501	19.00	lobChIP_FOXA1inPBS
chr10	3948847	3948848	MACS_peak_1502	7.00	lobChIP_FOXA1inPBS
chr10	4425286	4425287	MACS_peak_1503	7.00	lobChIP_FOXA1inPBS
chr10	4704283	4704284	MACS_peak_1504	7.00	lobChIP_FOXA1inPBS
chr10	4768486	4768487	MACS_peak_1505	8.00	lobChIP_FOXA1inPBS
chr10	4990016	4990017	MACS_peak_1506	19.00	lobChIP_FOXA1inPBS
chr10	5051481	5051482	MACS_peak_1507	22.00	lobChIP_FOXA1inPBS
chr10	5111097	5111098	MACS_peak_1508	9.00	lobChIP_FOXA1inPBS
chr10	5113293	5113294	MACS_peak_1509	7.00	lobChIP_FOXA1inPBS
chr10	5122215	5122216	MACS_peak_1510	14.00	lobChIP_FOXA1inPBS
chr10	5189669	5189670	MACS_peak_1511	29.00	lobChIP_FOXA1inPBS
chr10	5190779	5190780	MACS_peak_1512	13.00	lobChIP_FOXA1inPBS
chr10	5238169	5238170	MACS_peak_1513	11.00	lobChIP_FOXA1inPBS
chr10	5261783	5261784	MACS_peak_1514	10.00	lobChIP_FOXA1inPBS
chr10	5276260	5276261	MACS_peak_1515	19.00	lobChIP_FOXA1inPBS
chr10	5285636	5285637	MACS_peak_1516	57.00	lobChIP_FOXA1inPBS
chr10	5535831	5535832	MACS_peak_1517	54.00	lobChIP_FOXA1inPBS
chr10	5558270	5558271	MACS_peak_1518	24.00	lobChIP_FOXA1inPBS
chr10	5560303	5560304	MACS_peak_1519	10.00	lobChIP_FOXA1inPBS
chr10	5570563	5570564	MACS_peak_1520	12.00	lobChIP_FOXA1inPBS
chr10	5590236	5590237	MACS_peak_1521	7.00	lobChIP_FOXA1inPBS
chr10	5596470	5596471	MACS_peak_1522	10.00	lobChIP_FOXA1inPBS
chr10	5613446	5613447	MACS_peak_1523	12.00	lobChIP_FOXA1inPBS
chr10	5702076	5702077	MACS_peak_1524	10.00	lobChIP_FOXA1inPBS
chr10	5734736	5734737	MACS_peak_1525	10.00	lobChIP_FOXA1inPBS
chr10	5804691	5804692	MACS_peak_1526	23.00	lobChIP_FOXA1inPBS
chr10	5807674	5807675	MACS_peak_1527	6.00	lobChIP_FOXA1inPBS
chr10	5859774	5859775	MACS_peak_1528	13.00	lobChIP_FOXA1inPBS
chr10	6128522	6128523	MACS_peak_1529	10.00	lobChIP_FOXA1inPBS
chr10	6313901	6313902	MACS_peak_1530	32.00	lobChIP_FOXA1inPBS
chr10	6585213	6585214	MACS_peak_1531	8.00	lobChIP_FOXA1inPBS
chr10	7275455	7275456	MACS_peak_1532	12.00	lobChIP_FOXA1inPBS
chr10	7555857	7555858	MACS_peak_1533	9.00	lobChIP_FOXA1inPBS
chr10	7584404	7584405	MACS_peak_1534	25.00	lobChIP_FOXA1inPBS
chr10	7659001	7659002	MACS_peak_1535	5.00	lobChIP_FOXA1inPBS
chr10	7728983	7728984	MACS_peak_1536	6.00	lobChIP_FOXA1inPBS
chr10	7738805	7738806	MACS_peak_1537	13.00	lobChIP_FOXA1inPBS
chr10	7742798	7742799	MACS_peak_1538	29.00	lobChIP_FOXA1inPBS
chr10	7745181	7745182	MACS_peak_1539	20.00	lobChIP_FOXA1inPBS
chr10	7749273	7749274	MACS_peak_1540	28.00	lobChIP_FOXA1inPBS
chr10	7837813	7837814	MACS_peak_1541	25.00	lobChIP_FOXA1inPBS
chr10	7853851	7853852	MACS_peak_1542	7.00	lobChIP_FOXA1inPBS
chr10	7974483	7974484	MACS_peak_1543	11.00	lobChIP_FOXA1inPBS
chr10	7985009	7985010	MACS_peak_1544	7.00	lobChIP_FOXA1inPBS
chr10	8092793	8092794	MACS_peak_1545	27.00	lobChIP_FOXA1inPBS
chr10	8378723	8378724	MACS_peak_1546	8.00	lobChIP_FOXA1inPBS
chr10	9347212	9347213	MACS_peak_1547	21.00	lobChIP_FOXA1inPBS
chr10	11565098	11565099	MACS_peak_1548	11.00	lobChIP_FOXA1inPBS
chr10	11574452	11574453	MACS_peak_1549	7.00	lobChIP_FOXA1inPBS
chr10	11645103	11645104	MACS_peak_1550	15.00	lobChIP_FOXA1inPBS
chr10	11652648	11652649	MACS_peak_1551	6.00	lobChIP_FOXA1inPBS
chr10	11682364	11682365	MACS_peak_1552	31.00	lobChIP_FOXA1inPBS
chr10	11758143	11758144	MACS_peak_1553	12.00	lobChIP_FOXA1inPBS
chr10	11799648	11799649	MACS_peak_1554	17.00	lobChIP_FOXA1inPBS
chr10	11873335	11873336	MACS_peak_1555	36.00	lobChIP_FOXA1inPBS
chr10	12028729	12028730	MACS_peak_1556	37.00	lobChIP_FOXA1inPBS
chr10	12063030	12063031	MACS_peak_1557	7.00	lobChIP_FOXA1inPBS
chr10	12198870	12198871	MACS_peak_1558	7.00	lobChIP_FOXA1inPBS
chr10	12279912	12279913	MACS_peak_1559	11.00	lobChIP_FOXA1inPBS
chr10	12304915	12304916	MACS_peak_1560	20.00	lobChIP_FOXA1inPBS
chr10	12513305	12513306	MACS_peak_1561	7.00	lobChIP_FOXA1inPBS
chr10	12543421	12543422	MACS_peak_1562	7.00	lobChIP_FOXA1inPBS
chr10	12647877	12647878	MACS_peak_1563	17.00	lobChIP_FOXA1inPBS
chr10	13082376	13082377	MACS_peak_1564	10.00	lobChIP_FOXA1inPBS
chr10	13149761	13149762	MACS_peak_1565	30.00	lobChIP_FOXA1inPBS
chr10	13325442	13325443	MACS_peak_1566	13.00	lobChIP_FOXA1inPBS
chr10	13334037	13334038	MACS_peak_1567	15.00	lobChIP_FOXA1inPBS
chr10	13810041	13810042	MACS_peak_1568	6.00	lobChIP_FOXA1inPBS
chr10	14226997	14226998	MACS_peak_1569	11.00	lobChIP_FOXA1inPBS
chr10	14234959	14234960	MACS_peak_1570	14.00	lobChIP_FOXA1inPBS
chr10	14302387	14302388	MACS_peak_1571	26.00	lobChIP_FOXA1inPBS
chr10	14592653	14592654	MACS_peak_1572	11.00	lobChIP_FOXA1inPBS
chr10	14614233	14614234	MACS_peak_1573	9.00	lobChIP_FOXA1inPBS
chr10	14620829	14620830	MACS_peak_1574	11.00	lobChIP_FOXA1inPBS
chr10	15126696	15126697	MACS_peak_1575	24.00	lobChIP_FOXA1inPBS
chr10	15179263	15179264	MACS_peak_1576	6.00	lobChIP_FOXA1inPBS
chr10	15209589	15209590	MACS_peak_1577	5.00	lobChIP_FOXA1inPBS
chr10	15358376	15358377	MACS_peak_1578	7.00	lobChIP_FOXA1inPBS
chr10	15386977	15386978	MACS_peak_1579	20.00	lobChIP_FOXA1inPBS
chr10	16193177	16193178	MACS_peak_1580	5.00	lobChIP_FOXA1inPBS
chr10	16423226	16423227	MACS_peak_1581	8.00	lobChIP_FOXA1inPBS
chr10	17430301	17430302	MACS_peak_1582	7.00	lobChIP_FOXA1inPBS
chr10	17693225	17693226	MACS_peak_1583	6.00	lobChIP_FOXA1inPBS
chr10	17710656	17710657	MACS_peak_1584	12.00	lobChIP_FOXA1inPBS
chr10	17715857	17715858	MACS_peak_1585	9.00	lobChIP_FOXA1inPBS
chr10	18899639	18899640	MACS_peak_1586	13.00	lobChIP_FOXA1inPBS
chr10	18926588	18926589	MACS_peak_1587	18.00	lobChIP_FOXA1inPBS
chr10	19310257	19310258	MACS_peak_1588	13.00	lobChIP_FOXA1inPBS
chr10	19898178	19898179	MACS_peak_1589	7.00	lobChIP_FOXA1inPBS
chr10	20090698	20090699	MACS_peak_1590	6.00	lobChIP_FOXA1inPBS
chr10	20377453	20377454	MACS_peak_1591	13.00	lobChIP_FOXA1inPBS
chr10	21275246	21275247	MACS_peak_1592	10.00	lobChIP_FOXA1inPBS
chr10	21461574	21461575	MACS_peak_1593	21.00	lobChIP_FOXA1inPBS
chr10	21764390	21764391	MACS_peak_1594	14.00	lobChIP_FOXA1inPBS
chr10	21791124	21791125	MACS_peak_1595	23.00	lobChIP_FOXA1inPBS
chr10	21794989	21794990	MACS_peak_1596	18.00	lobChIP_FOXA1inPBS
chr10	21814525	21814526	MACS_peak_1597	8.00	lobChIP_FOXA1inPBS
chr10	21819270	21819271	MACS_peak_1598	8.00	lobChIP_FOXA1inPBS
chr10	21853367	21853368	MACS_peak_1599	11.00	lobChIP_FOXA1inPBS
chr10	21856216	21856217	MACS_peak_1600	30.00	lobChIP_FOXA1inPBS
chr10	21889746	21889747	MACS_peak_1601	6.00	lobChIP_FOXA1inPBS
chr10	21903886	21903887	MACS_peak_1602	6.00	lobChIP_FOXA1inPBS
chr10	21964202	21964203	MACS_peak_1603	12.00	lobChIP_FOXA1inPBS
chr10	22016864	22016865	MACS_peak_1604	14.00	lobChIP_FOXA1inPBS
chr10	22264476	22264477	MACS_peak_1605	11.00	lobChIP_FOXA1inPBS
chr10	22375827	22375828	MACS_peak_1606	14.00	lobChIP_FOXA1inPBS
chr10	22606021	22606022	MACS_peak_1607	8.00	lobChIP_FOXA1inPBS
chr10	22823991	22823992	MACS_peak_1608	13.00	lobChIP_FOXA1inPBS
chr10	22913330	22913331	MACS_peak_1609	29.00	lobChIP_FOXA1inPBS
chr10	22955472	22955473	MACS_peak_1610	9.00	lobChIP_FOXA1inPBS
chr10	22971499	22971500	MACS_peak_1611	16.00	lobChIP_FOXA1inPBS
chr10	22972434	22972435	MACS_peak_1612	19.00	lobChIP_FOXA1inPBS
chr10	23248390	23248391	MACS_peak_1613	16.00	lobChIP_FOXA1inPBS
chr10	23475746	23475747	MACS_peak_1614	21.00	lobChIP_FOXA1inPBS
chr10	23542829	23542830	MACS_peak_1615	5.00	lobChIP_FOXA1inPBS
chr10	23806299	23806300	MACS_peak_1616	8.00	lobChIP_FOXA1inPBS
chr10	24491255	24491256	MACS_peak_1617	6.00	lobChIP_FOXA1inPBS
chr10	24497221	24497222	MACS_peak_1618	7.00	lobChIP_FOXA1inPBS
chr10	24526914	24526915	MACS_peak_1619	10.00	lobChIP_FOXA1inPBS
chr10	24731314	24731315	MACS_peak_1620	15.00	lobChIP_FOXA1inPBS
chr10	24755233	24755234	MACS_peak_1621	16.00	lobChIP_FOXA1inPBS
chr10	24830430	24830431	MACS_peak_1622	16.00	lobChIP_FOXA1inPBS
chr10	24831542	24831543	MACS_peak_1623	10.00	lobChIP_FOXA1inPBS
chr10	24970274	24970275	MACS_peak_1624	34.00	lobChIP_FOXA1inPBS
chr10	25001895	25001896	MACS_peak_1625	10.00	lobChIP_FOXA1inPBS
chr10	25007651	25007652	MACS_peak_1626	6.00	lobChIP_FOXA1inPBS
chr10	25348808	25348809	MACS_peak_1627	13.00	lobChIP_FOXA1inPBS
chr10	27023300	27023301	MACS_peak_1628	13.00	lobChIP_FOXA1inPBS
chr10	27027491	27027492	MACS_peak_1629	7.00	lobChIP_FOXA1inPBS
chr10	27527364	27527365	MACS_peak_1630	9.00	lobChIP_FOXA1inPBS
chr10	27555091	27555092	MACS_peak_1631	13.00	lobChIP_FOXA1inPBS
chr10	27630141	27630142	MACS_peak_1632	7.00	lobChIP_FOXA1inPBS
chr10	27649653	27649654	MACS_peak_1633	7.00	lobChIP_FOXA1inPBS
chr10	27988880	27988881	MACS_peak_1634	10.00	lobChIP_FOXA1inPBS
chr10	28188625	28188626	MACS_peak_1635	9.00	lobChIP_FOXA1inPBS
chr10	28286852	28286853	MACS_peak_1636	8.00	lobChIP_FOXA1inPBS
chr10	28552249	28552250	MACS_peak_1637	10.00	lobChIP_FOXA1inPBS
chr10	28669420	28669421	MACS_peak_1638	9.00	lobChIP_FOXA1inPBS
chr10	28858808	28858809	MACS_peak_1639	28.00	lobChIP_FOXA1inPBS
chr10	28971544	28971545	MACS_peak_1640	12.00	lobChIP_FOXA1inPBS
chr10	29003603	29003604	MACS_peak_1641	9.00	lobChIP_FOXA1inPBS
chr10	29058412	29058413	MACS_peak_1642	10.00	lobChIP_FOXA1inPBS
chr10	29063247	29063248	MACS_peak_1643	10.00	lobChIP_FOXA1inPBS
chr10	29128095	29128096	MACS_peak_1644	10.00	lobChIP_FOXA1inPBS
chr10	29173541	29173542	MACS_peak_1645	24.00	lobChIP_FOXA1inPBS
chr10	29198707	29198708	MACS_peak_1646	8.00	lobChIP_FOXA1inPBS
chr10	29229030	29229031	MACS_peak_1647	93.00	lobChIP_FOXA1inPBS
chr10	29388107	29388108	MACS_peak_1648	8.00	lobChIP_FOXA1inPBS
chr10	29419303	29419304	MACS_peak_1649	12.00	lobChIP_FOXA1inPBS
chr10	29606461	29606462	MACS_peak_1650	7.00	lobChIP_FOXA1inPBS
chr10	29607285	29607286	MACS_peak_1651	14.00	lobChIP_FOXA1inPBS
chr10	29615959	29615960	MACS_peak_1652	7.00	lobChIP_FOXA1inPBS
chr10	29616578	29616579	MACS_peak_1653	35.00	lobChIP_FOXA1inPBS
chr10	29618538	29618539	MACS_peak_1654	7.00	lobChIP_FOXA1inPBS
chr10	29681793	29681794	MACS_peak_1655	8.00	lobChIP_FOXA1inPBS
chr10	29785539	29785540	MACS_peak_1656	11.00	lobChIP_FOXA1inPBS
chr10	29802938	29802939	MACS_peak_1657	12.00	lobChIP_FOXA1inPBS
chr10	29908327	29908328	MACS_peak_1658	23.00	lobChIP_FOXA1inPBS
chr10	29917000	29917001	MACS_peak_1659	17.00	lobChIP_FOXA1inPBS
chr10	30530243	30530244	MACS_peak_1660	12.00	lobChIP_FOXA1inPBS
chr10	30727655	30727656	MACS_peak_1661	14.00	lobChIP_FOXA1inPBS
chr10	30817028	30817029	MACS_peak_1662	16.00	lobChIP_FOXA1inPBS
chr10	30831370	30831371	MACS_peak_1663	35.00	lobChIP_FOXA1inPBS
chr10	30885616	30885617	MACS_peak_1664	7.00	lobChIP_FOXA1inPBS
chr10	30894943	30894944	MACS_peak_1665	38.00	lobChIP_FOXA1inPBS
chr10	32154091	32154092	MACS_peak_1666	17.00	lobChIP_FOXA1inPBS
chr10	32226144	32226145	MACS_peak_1667	16.00	lobChIP_FOXA1inPBS
chr10	32230011	32230012	MACS_peak_1668	9.00	lobChIP_FOXA1inPBS
chr10	32232037	32232038	MACS_peak_1669	15.00	lobChIP_FOXA1inPBS
chr10	32233979	32233980	MACS_peak_1670	23.00	lobChIP_FOXA1inPBS
chr10	32348663	32348664	MACS_peak_1671	13.00	lobChIP_FOXA1inPBS
chr10	32384249	32384250	MACS_peak_1672	38.00	lobChIP_FOXA1inPBS
chr10	32394189	32394190	MACS_peak_1673	13.00	lobChIP_FOXA1inPBS
chr10	32394951	32394952	MACS_peak_1674	26.00	lobChIP_FOXA1inPBS
chr10	32604977	32604978	MACS_peak_1675	7.00	lobChIP_FOXA1inPBS
chr10	32639860	32639861	MACS_peak_1676	7.00	lobChIP_FOXA1inPBS
chr10	32780324	32780325	MACS_peak_1677	7.00	lobChIP_FOXA1inPBS
chr10	33084720	33084721	MACS_peak_1678	20.00	lobChIP_FOXA1inPBS
chr10	33240083	33240084	MACS_peak_1679	7.00	lobChIP_FOXA1inPBS
chr10	33275144	33275145	MACS_peak_1680	11.00	lobChIP_FOXA1inPBS
chr10	33445138	33445139	MACS_peak_1681	103.00	lobChIP_FOXA1inPBS
chr10	33452458	33452459	MACS_peak_1682	9.00	lobChIP_FOXA1inPBS
chr10	33485026	33485027	MACS_peak_1683	15.00	lobChIP_FOXA1inPBS
chr10	33487742	33487743	MACS_peak_1684	19.00	lobChIP_FOXA1inPBS
chr10	33553346	33553347	MACS_peak_1685	27.00	lobChIP_FOXA1inPBS
chr10	33576208	33576209	MACS_peak_1686	9.00	lobChIP_FOXA1inPBS
chr10	33595850	33595851	MACS_peak_1687	19.00	lobChIP_FOXA1inPBS
chr10	33615982	33615983	MACS_peak_1688	12.00	lobChIP_FOXA1inPBS
chr10	33619559	33619560	MACS_peak_1689	9.00	lobChIP_FOXA1inPBS
chr10	33620473	33620474	MACS_peak_1690	9.00	lobChIP_FOXA1inPBS
chr10	33779756	33779757	MACS_peak_1691	6.00	lobChIP_FOXA1inPBS
chr10	33797108	33797109	MACS_peak_1692	10.00	lobChIP_FOXA1inPBS
chr10	33798318	33798319	MACS_peak_1693	8.00	lobChIP_FOXA1inPBS
chr10	34121245	34121246	MACS_peak_1694	8.00	lobChIP_FOXA1inPBS
chr10	34165667	34165668	MACS_peak_1695	9.00	lobChIP_FOXA1inPBS
chr10	34386821	34386822	MACS_peak_1696	24.00	lobChIP_FOXA1inPBS
chr10	34405377	34405378	MACS_peak_1697	10.00	lobChIP_FOXA1inPBS
chr10	34410369	34410370	MACS_peak_1698	12.00	lobChIP_FOXA1inPBS
chr10	34452996	34452997	MACS_peak_1699	13.00	lobChIP_FOXA1inPBS
chr10	34465510	34465511	MACS_peak_1700	5.00	lobChIP_FOXA1inPBS
chr10	34474117	34474118	MACS_peak_1701	15.00	lobChIP_FOXA1inPBS
chr10	34502532	34502533	MACS_peak_1702	20.00	lobChIP_FOXA1inPBS
chr10	34505723	34505724	MACS_peak_1703	7.00	lobChIP_FOXA1inPBS
chr10	34521523	34521524	MACS_peak_1704	6.00	lobChIP_FOXA1inPBS
chr10	34626018	34626019	MACS_peak_1705	7.00	lobChIP_FOXA1inPBS
chr10	34634487	34634488	MACS_peak_1706	27.00	lobChIP_FOXA1inPBS
chr10	34721185	34721186	MACS_peak_1707	10.00	lobChIP_FOXA1inPBS
chr10	34819989	34819990	MACS_peak_1708	9.00	lobChIP_FOXA1inPBS
chr10	34926812	34926813	MACS_peak_1709	11.00	lobChIP_FOXA1inPBS
chr10	34997489	34997490	MACS_peak_1710	9.00	lobChIP_FOXA1inPBS
chr10	35002969	35002970	MACS_peak_1711	38.00	lobChIP_FOXA1inPBS
chr10	35020923	35020924	MACS_peak_1712	8.00	lobChIP_FOXA1inPBS
chr10	35026143	35026144	MACS_peak_1713	82.00	lobChIP_FOXA1inPBS
chr10	35150633	35150634	MACS_peak_1714	11.00	lobChIP_FOXA1inPBS
chr10	35654024	35654025	MACS_peak_1715	7.00	lobChIP_FOXA1inPBS
chr10	35974563	35974564	MACS_peak_1716	18.00	lobChIP_FOXA1inPBS
chr10	36040422	36040423	MACS_peak_1717	7.00	lobChIP_FOXA1inPBS
chr10	36540286	36540287	MACS_peak_1718	11.00	lobChIP_FOXA1inPBS
chr10	37178049	37178050	MACS_peak_1719	11.00	lobChIP_FOXA1inPBS
chr10	37912630	37912631	MACS_peak_1720	19.00	lobChIP_FOXA1inPBS
chr10	38078721	38078722	MACS_peak_1721	8.00	lobChIP_FOXA1inPBS
chr10	43137225	43137226	MACS_peak_1722	10.00	lobChIP_FOXA1inPBS
chr10	43891641	43891642	MACS_peak_1723	15.00	lobChIP_FOXA1inPBS
chr10	44203885	44203886	MACS_peak_1724	6.00	lobChIP_FOXA1inPBS
chr10	44359513	44359514	MACS_peak_1725	14.00	lobChIP_FOXA1inPBS
chr10	44691165	44691166	MACS_peak_1726	21.00	lobChIP_FOXA1inPBS
chr10	44732528	44732529	MACS_peak_1727	49.00	lobChIP_FOXA1inPBS
chr10	44754685	44754686	MACS_peak_1728	12.00	lobChIP_FOXA1inPBS
chr10	44820640	44820641	MACS_peak_1729	6.00	lobChIP_FOXA1inPBS
chr10	45241525	45241526	MACS_peak_1730	25.00	lobChIP_FOXA1inPBS
chr10	45435666	45435667	MACS_peak_1731	15.00	lobChIP_FOXA1inPBS
chr10	45476377	45476378	MACS_peak_1732	13.00	lobChIP_FOXA1inPBS
chr10	45491474	45491475	MACS_peak_1733	23.00	lobChIP_FOXA1inPBS
chr10	45966231	45966232	MACS_peak_1734	24.00	lobChIP_FOXA1inPBS
chr10	45998173	45998174	MACS_peak_1735	10.00	lobChIP_FOXA1inPBS
chr10	46150401	46150402	MACS_peak_1736	11.00	lobChIP_FOXA1inPBS
chr10	47102056	47102057	MACS_peak_1737	8.00	lobChIP_FOXA1inPBS
chr10	47644969	47644970	MACS_peak_1738	7.00	lobChIP_FOXA1inPBS
chr10	49571814	49571815	MACS_peak_1739	8.00	lobChIP_FOXA1inPBS
chr10	49996777	49996778	MACS_peak_1740	9.00	lobChIP_FOXA1inPBS
chr10	50344300	50344301	MACS_peak_1741	7.00	lobChIP_FOXA1inPBS
chr10	50747620	50747621	MACS_peak_1742	8.00	lobChIP_FOXA1inPBS
chr10	51493373	51493374	MACS_peak_1743	7.00	lobChIP_FOXA1inPBS
chr10	51543234	51543235	MACS_peak_1744	59.00	lobChIP_FOXA1inPBS
chr10	51559839	51559840	MACS_peak_1745	11.00	lobChIP_FOXA1inPBS
chr10	51563679	51563680	MACS_peak_1746	31.00	lobChIP_FOXA1inPBS
chr10	51574723	51574724	MACS_peak_1747	13.00	lobChIP_FOXA1inPBS
chr10	51593248	51593249	MACS_peak_1748	31.00	lobChIP_FOXA1inPBS
chr10	52009973	52009974	MACS_peak_1749	10.00	lobChIP_FOXA1inPBS
chr10	52021026	52021027	MACS_peak_1750	8.00	lobChIP_FOXA1inPBS
chr10	52152450	52152451	MACS_peak_1751	11.00	lobChIP_FOXA1inPBS
chr10	52155273	52155274	MACS_peak_1752	25.00	lobChIP_FOXA1inPBS
chr10	52261814	52261815	MACS_peak_1753	12.00	lobChIP_FOXA1inPBS
chr10	52268740	52268741	MACS_peak_1754	10.00	lobChIP_FOXA1inPBS
chr10	52326142	52326143	MACS_peak_1755	23.00	lobChIP_FOXA1inPBS
chr10	52339920	52339921	MACS_peak_1756	9.00	lobChIP_FOXA1inPBS
chr10	52365290	52365291	MACS_peak_1757	7.00	lobChIP_FOXA1inPBS
chr10	52365772	52365773	MACS_peak_1758	19.00	lobChIP_FOXA1inPBS
chr10	52642497	52642498	MACS_peak_1759	32.00	lobChIP_FOXA1inPBS
chr10	53972782	53972783	MACS_peak_1760	14.00	lobChIP_FOXA1inPBS
chr10	54073931	54073932	MACS_peak_1761	12.00	lobChIP_FOXA1inPBS
chr10	54125674	54125675	MACS_peak_1762	15.00	lobChIP_FOXA1inPBS
chr10	54136645	54136646	MACS_peak_1763	7.00	lobChIP_FOXA1inPBS
chr10	54138192	54138193	MACS_peak_1764	15.00	lobChIP_FOXA1inPBS
chr10	54212612	54212613	MACS_peak_1765	48.00	lobChIP_FOXA1inPBS
chr10	54397490	54397491	MACS_peak_1766	26.00	lobChIP_FOXA1inPBS
chr10	54402304	54402305	MACS_peak_1767	9.00	lobChIP_FOXA1inPBS
chr10	54502090	54502091	MACS_peak_1768	7.00	lobChIP_FOXA1inPBS
chr10	54515375	54515376	MACS_peak_1769	8.00	lobChIP_FOXA1inPBS
chr10	54528227	54528228	MACS_peak_1770	10.00	lobChIP_FOXA1inPBS
chr10	54531489	54531490	MACS_peak_1771	10.00	lobChIP_FOXA1inPBS
chr10	54534483	54534484	MACS_peak_1772	20.00	lobChIP_FOXA1inPBS
chr10	54643869	54643870	MACS_peak_1773	9.00	lobChIP_FOXA1inPBS
chr10	54759627	54759628	MACS_peak_1774	8.00	lobChIP_FOXA1inPBS
chr10	54766990	54766991	MACS_peak_1775	39.00	lobChIP_FOXA1inPBS
chr10	55361176	55361177	MACS_peak_1776	35.00	lobChIP_FOXA1inPBS
chr10	55646598	55646599	MACS_peak_1777	20.00	lobChIP_FOXA1inPBS
chr10	56398969	56398970	MACS_peak_1778	9.00	lobChIP_FOXA1inPBS
chr10	56427062	56427063	MACS_peak_1779	7.00	lobChIP_FOXA1inPBS
chr10	57853604	57853605	MACS_peak_1780	34.00	lobChIP_FOXA1inPBS
chr10	60015887	60015888	MACS_peak_1781	7.00	lobChIP_FOXA1inPBS
chr10	60065589	60065590	MACS_peak_1782	6.00	lobChIP_FOXA1inPBS
chr10	60108568	60108569	MACS_peak_1783	14.00	lobChIP_FOXA1inPBS
chr10	60110594	60110595	MACS_peak_1784	13.00	lobChIP_FOXA1inPBS
chr10	60204485	60204486	MACS_peak_1785	7.00	lobChIP_FOXA1inPBS
chr10	60213077	60213078	MACS_peak_1786	9.00	lobChIP_FOXA1inPBS
chr10	60218217	60218218	MACS_peak_1787	12.00	lobChIP_FOXA1inPBS
chr10	60228526	60228527	MACS_peak_1788	151.00	lobChIP_FOXA1inPBS
chr10	60320858	60320859	MACS_peak_1789	8.00	lobChIP_FOXA1inPBS
chr10	60422272	60422273	MACS_peak_1790	10.00	lobChIP_FOXA1inPBS
chr10	60567823	60567824	MACS_peak_1791	11.00	lobChIP_FOXA1inPBS
chr10	60573510	60573511	MACS_peak_1792	8.00	lobChIP_FOXA1inPBS
chr10	60577493	60577494	MACS_peak_1793	12.00	lobChIP_FOXA1inPBS
chr10	60754184	60754185	MACS_peak_1794	9.00	lobChIP_FOXA1inPBS
chr10	60766614	60766615	MACS_peak_1795	27.00	lobChIP_FOXA1inPBS
chr10	60797445	60797446	MACS_peak_1796	18.00	lobChIP_FOXA1inPBS
chr10	60818049	60818050	MACS_peak_1797	50.00	lobChIP_FOXA1inPBS
chr10	60833531	60833532	MACS_peak_1798	74.00	lobChIP_FOXA1inPBS
chr10	60939623	60939624	MACS_peak_1799	13.00	lobChIP_FOXA1inPBS
chr10	60976052	60976053	MACS_peak_1800	6.00	lobChIP_FOXA1inPBS
chr10	61082924	61082925	MACS_peak_1801	13.00	lobChIP_FOXA1inPBS
chr10	61462362	61462363	MACS_peak_1802	16.00	lobChIP_FOXA1inPBS
chr10	62042838	62042839	MACS_peak_1803	26.00	lobChIP_FOXA1inPBS
chr10	62182734	62182735	MACS_peak_1804	18.00	lobChIP_FOXA1inPBS
chr10	62702515	62702516	MACS_peak_1805	10.00	lobChIP_FOXA1inPBS
chr10	62716124	62716125	MACS_peak_1806	22.00	lobChIP_FOXA1inPBS
chr10	62727273	62727274	MACS_peak_1807	8.00	lobChIP_FOXA1inPBS
chr10	62736356	62736357	MACS_peak_1808	9.00	lobChIP_FOXA1inPBS
chr10	62737592	62737593	MACS_peak_1809	12.00	lobChIP_FOXA1inPBS
chr10	62753975	62753976	MACS_peak_1810	5.00	lobChIP_FOXA1inPBS
chr10	62822961	62822962	MACS_peak_1811	67.00	lobChIP_FOXA1inPBS
chr10	62824989	62824990	MACS_peak_1812	11.00	lobChIP_FOXA1inPBS
chr10	62826863	62826864	MACS_peak_1813	43.00	lobChIP_FOXA1inPBS
chr10	62835399	62835400	MACS_peak_1814	50.00	lobChIP_FOXA1inPBS
chr10	62896137	62896138	MACS_peak_1815	12.00	lobChIP_FOXA1inPBS
chr10	62939702	62939703	MACS_peak_1816	9.00	lobChIP_FOXA1inPBS
chr10	63597170	63597171	MACS_peak_1817	31.00	lobChIP_FOXA1inPBS
chr10	63712291	63712292	MACS_peak_1818	16.00	lobChIP_FOXA1inPBS
chr10	63724411	63724412	MACS_peak_1819	64.00	lobChIP_FOXA1inPBS
chr10	63787973	63787974	MACS_peak_1820	7.00	lobChIP_FOXA1inPBS
chr10	63797436	63797437	MACS_peak_1821	13.00	lobChIP_FOXA1inPBS
chr10	63797970	63797971	MACS_peak_1822	17.00	lobChIP_FOXA1inPBS
chr10	63809256	63809257	MACS_peak_1823	9.00	lobChIP_FOXA1inPBS
chr10	63842248	63842249	MACS_peak_1824	11.00	lobChIP_FOXA1inPBS
chr10	63853893	63853894	MACS_peak_1825	7.00	lobChIP_FOXA1inPBS
chr10	63855510	63855511	MACS_peak_1826	32.00	lobChIP_FOXA1inPBS
chr10	63856909	63856910	MACS_peak_1827	8.00	lobChIP_FOXA1inPBS
chr10	63908234	63908235	MACS_peak_1828	7.00	lobChIP_FOXA1inPBS
chr10	63943829	63943830	MACS_peak_1829	40.00	lobChIP_FOXA1inPBS
chr10	64170882	64170883	MACS_peak_1830	8.00	lobChIP_FOXA1inPBS
chr10	65028140	65028141	MACS_peak_1831	14.00	lobChIP_FOXA1inPBS
chr10	65229339	65229340	MACS_peak_1832	10.00	lobChIP_FOXA1inPBS
chr10	65247989	65247990	MACS_peak_1833	10.00	lobChIP_FOXA1inPBS
chr10	65265481	65265482	MACS_peak_1834	17.00	lobChIP_FOXA1inPBS
chr10	65266505	65266506	MACS_peak_1835	11.00	lobChIP_FOXA1inPBS
chr10	65804105	65804106	MACS_peak_1836	16.00	lobChIP_FOXA1inPBS
chr10	66793356	66793357	MACS_peak_1837	6.00	lobChIP_FOXA1inPBS
chr10	67261153	67261154	MACS_peak_1838	12.00	lobChIP_FOXA1inPBS
chr10	68043869	68043870	MACS_peak_1839	12.00	lobChIP_FOXA1inPBS
chr10	68332640	68332641	MACS_peak_1840	11.00	lobChIP_FOXA1inPBS
chr10	68874891	68874892	MACS_peak_1841	15.00	lobChIP_FOXA1inPBS
chr10	69001370	69001371	MACS_peak_1842	18.00	lobChIP_FOXA1inPBS
chr10	69553254	69553255	MACS_peak_1843	15.00	lobChIP_FOXA1inPBS
chr10	69563551	69563552	MACS_peak_1844	54.00	lobChIP_FOXA1inPBS
chr10	69587584	69587585	MACS_peak_1845	8.00	lobChIP_FOXA1inPBS
chr10	69597968	69597969	MACS_peak_1846	10.00	lobChIP_FOXA1inPBS
chr10	69649590	69649591	MACS_peak_1847	16.00	lobChIP_FOXA1inPBS
chr10	69847189	69847190	MACS_peak_1848	5.00	lobChIP_FOXA1inPBS
chr10	70286598	70286599	MACS_peak_1849	13.00	lobChIP_FOXA1inPBS
chr10	70362376	70362377	MACS_peak_1850	14.00	lobChIP_FOXA1inPBS
chr10	70520074	70520075	MACS_peak_1851	5.00	lobChIP_FOXA1inPBS
chr10	70636393	70636394	MACS_peak_1852	31.00	lobChIP_FOXA1inPBS
chr10	70638376	70638377	MACS_peak_1853	12.00	lobChIP_FOXA1inPBS
chr10	70908045	70908046	MACS_peak_1854	6.00	lobChIP_FOXA1inPBS
chr10	71074911	71074912	MACS_peak_1855	13.00	lobChIP_FOXA1inPBS
chr10	71145969	71145970	MACS_peak_1856	11.00	lobChIP_FOXA1inPBS
chr10	71152555	71152556	MACS_peak_1857	8.00	lobChIP_FOXA1inPBS
chr10	71206523	71206524	MACS_peak_1858	20.00	lobChIP_FOXA1inPBS
chr10	71845985	71845986	MACS_peak_1859	24.00	lobChIP_FOXA1inPBS
chr10	71931182	71931183	MACS_peak_1860	10.00	lobChIP_FOXA1inPBS
chr10	72086072	72086073	MACS_peak_1861	13.00	lobChIP_FOXA1inPBS
chr10	72112597	72112598	MACS_peak_1862	23.00	lobChIP_FOXA1inPBS
chr10	72190515	72190516	MACS_peak_1863	12.00	lobChIP_FOXA1inPBS
chr10	72202309	72202310	MACS_peak_1864	15.00	lobChIP_FOXA1inPBS
chr10	72612128	72612129	MACS_peak_1865	11.00	lobChIP_FOXA1inPBS
chr10	73052543	73052544	MACS_peak_1866	10.00	lobChIP_FOXA1inPBS
chr10	73079442	73079443	MACS_peak_1867	9.00	lobChIP_FOXA1inPBS
chr10	73094414	73094415	MACS_peak_1868	7.00	lobChIP_FOXA1inPBS
chr10	73100041	73100042	MACS_peak_1869	37.00	lobChIP_FOXA1inPBS
chr10	73103171	73103172	MACS_peak_1870	10.00	lobChIP_FOXA1inPBS
chr10	73107915	73107916	MACS_peak_1871	15.00	lobChIP_FOXA1inPBS
chr10	73640382	73640383	MACS_peak_1872	21.00	lobChIP_FOXA1inPBS
chr10	73687844	73687845	MACS_peak_1873	11.00	lobChIP_FOXA1inPBS
chr10	73736774	73736775	MACS_peak_1874	8.00	lobChIP_FOXA1inPBS
chr10	73768641	73768642	MACS_peak_1875	6.00	lobChIP_FOXA1inPBS
chr10	73781922	73781923	MACS_peak_1876	10.00	lobChIP_FOXA1inPBS
chr10	73797129	73797130	MACS_peak_1877	6.00	lobChIP_FOXA1inPBS
chr10	73798283	73798284	MACS_peak_1878	15.00	lobChIP_FOXA1inPBS
chr10	73856182	73856183	MACS_peak_1879	6.00	lobChIP_FOXA1inPBS
chr10	73857802	73857803	MACS_peak_1880	31.00	lobChIP_FOXA1inPBS
chr10	74011603	74011604	MACS_peak_1881	8.00	lobChIP_FOXA1inPBS
chr10	74036744	74036745	MACS_peak_1882	18.00	lobChIP_FOXA1inPBS
chr10	74283803	74283804	MACS_peak_1883	12.00	lobChIP_FOXA1inPBS
chr10	74302184	74302185	MACS_peak_1884	9.00	lobChIP_FOXA1inPBS
chr10	74398811	74398812	MACS_peak_1885	13.00	lobChIP_FOXA1inPBS
chr10	74439749	74439750	MACS_peak_1886	30.00	lobChIP_FOXA1inPBS
chr10	74712773	74712774	MACS_peak_1887	11.00	lobChIP_FOXA1inPBS
chr10	74783701	74783702	MACS_peak_1888	11.00	lobChIP_FOXA1inPBS
chr10	74848269	74848270	MACS_peak_1889	12.00	lobChIP_FOXA1inPBS
chr10	74879350	74879351	MACS_peak_1890	29.00	lobChIP_FOXA1inPBS
chr10	74892237	74892238	MACS_peak_1891	30.00	lobChIP_FOXA1inPBS
chr10	75173171	75173172	MACS_peak_1892	15.00	lobChIP_FOXA1inPBS
chr10	75346538	75346539	MACS_peak_1893	24.00	lobChIP_FOXA1inPBS
chr10	75351843	75351844	MACS_peak_1894	23.00	lobChIP_FOXA1inPBS
chr10	75545069	75545070	MACS_peak_1895	10.00	lobChIP_FOXA1inPBS
chr10	75583288	75583289	MACS_peak_1896	10.00	lobChIP_FOXA1inPBS
chr10	75794304	75794305	MACS_peak_1897	7.00	lobChIP_FOXA1inPBS
chr10	75973568	75973569	MACS_peak_1898	12.00	lobChIP_FOXA1inPBS
chr10	76308591	76308592	MACS_peak_1899	13.00	lobChIP_FOXA1inPBS
chr10	76464631	76464632	MACS_peak_1900	10.00	lobChIP_FOXA1inPBS
chr10	76516216	76516217	MACS_peak_1901	11.00	lobChIP_FOXA1inPBS
chr10	76582084	76582085	MACS_peak_1902	18.00	lobChIP_FOXA1inPBS
chr10	76635952	76635953	MACS_peak_1903	8.00	lobChIP_FOXA1inPBS
chr10	76728287	76728288	MACS_peak_1904	10.00	lobChIP_FOXA1inPBS
chr10	76903413	76903414	MACS_peak_1905	5.00	lobChIP_FOXA1inPBS
chr10	76928248	76928249	MACS_peak_1906	48.00	lobChIP_FOXA1inPBS
chr10	76949531	76949532	MACS_peak_1907	15.00	lobChIP_FOXA1inPBS
chr10	76952019	76952020	MACS_peak_1908	15.00	lobChIP_FOXA1inPBS
chr10	77165014	77165015	MACS_peak_1909	15.00	lobChIP_FOXA1inPBS
chr10	77190245	77190246	MACS_peak_1910	40.00	lobChIP_FOXA1inPBS
chr10	77200097	77200098	MACS_peak_1911	7.00	lobChIP_FOXA1inPBS
chr10	77255901	77255902	MACS_peak_1912	8.00	lobChIP_FOXA1inPBS
chr10	77272458	77272459	MACS_peak_1913	8.00	lobChIP_FOXA1inPBS
chr10	77325291	77325292	MACS_peak_1914	7.00	lobChIP_FOXA1inPBS
chr10	77360256	77360257	MACS_peak_1915	13.00	lobChIP_FOXA1inPBS
chr10	77360774	77360775	MACS_peak_1916	12.00	lobChIP_FOXA1inPBS
chr10	77390715	77390716	MACS_peak_1917	7.00	lobChIP_FOXA1inPBS
chr10	77493217	77493218	MACS_peak_1918	78.00	lobChIP_FOXA1inPBS
chr10	77503115	77503116	MACS_peak_1919	6.00	lobChIP_FOXA1inPBS
chr10	77506272	77506273	MACS_peak_1920	17.00	lobChIP_FOXA1inPBS
chr10	77531908	77531909	MACS_peak_1921	6.00	lobChIP_FOXA1inPBS
chr10	77533423	77533424	MACS_peak_1922	12.00	lobChIP_FOXA1inPBS
chr10	77536512	77536513	MACS_peak_1923	26.00	lobChIP_FOXA1inPBS
chr10	77548691	77548692	MACS_peak_1924	21.00	lobChIP_FOXA1inPBS
chr10	77558917	77558918	MACS_peak_1925	10.00	lobChIP_FOXA1inPBS
chr10	77608248	77608249	MACS_peak_1926	11.00	lobChIP_FOXA1inPBS
chr10	77784783	77784784	MACS_peak_1927	12.00	lobChIP_FOXA1inPBS
chr10	77786132	77786133	MACS_peak_1928	8.00	lobChIP_FOXA1inPBS
chr10	77793477	77793478	MACS_peak_1929	8.00	lobChIP_FOXA1inPBS
chr10	77811291	77811292	MACS_peak_1930	17.00	lobChIP_FOXA1inPBS
chr10	77910911	77910912	MACS_peak_1931	8.00	lobChIP_FOXA1inPBS
chr10	78318556	78318557	MACS_peak_1932	22.00	lobChIP_FOXA1inPBS
chr10	78871791	78871792	MACS_peak_1933	22.00	lobChIP_FOXA1inPBS
chr10	79093874	79093875	MACS_peak_1934	16.00	lobChIP_FOXA1inPBS
chr10	79211790	79211791	MACS_peak_1935	13.00	lobChIP_FOXA1inPBS
chr10	79608994	79608995	MACS_peak_1936	35.00	lobChIP_FOXA1inPBS
chr10	79671647	79671648	MACS_peak_1937	16.00	lobChIP_FOXA1inPBS
chr10	79704472	79704473	MACS_peak_1938	9.00	lobChIP_FOXA1inPBS
chr10	79808688	79808689	MACS_peak_1939	11.00	lobChIP_FOXA1inPBS
chr10	80218959	80218960	MACS_peak_1940	94.00	lobChIP_FOXA1inPBS
chr10	80280930	80280931	MACS_peak_1941	7.00	lobChIP_FOXA1inPBS
chr10	80288768	80288769	MACS_peak_1942	27.00	lobChIP_FOXA1inPBS
chr10	80349541	80349542	MACS_peak_1943	14.00	lobChIP_FOXA1inPBS
chr10	80405003	80405004	MACS_peak_1944	13.00	lobChIP_FOXA1inPBS
chr10	80502676	80502677	MACS_peak_1945	13.00	lobChIP_FOXA1inPBS
chr10	80613790	80613791	MACS_peak_1946	14.00	lobChIP_FOXA1inPBS
chr10	80654729	80654730	MACS_peak_1947	6.00	lobChIP_FOXA1inPBS
chr10	80698018	80698019	MACS_peak_1948	11.00	lobChIP_FOXA1inPBS
chr10	80726966	80726967	MACS_peak_1949	8.00	lobChIP_FOXA1inPBS
chr10	80732985	80732986	MACS_peak_1950	6.00	lobChIP_FOXA1inPBS
chr10	80819154	80819155	MACS_peak_1951	22.00	lobChIP_FOXA1inPBS
chr10	80819796	80819797	MACS_peak_1952	10.00	lobChIP_FOXA1inPBS
chr10	80853818	80853819	MACS_peak_1953	11.00	lobChIP_FOXA1inPBS
chr10	80917503	80917504	MACS_peak_1954	10.00	lobChIP_FOXA1inPBS
chr10	81095472	81095473	MACS_peak_1955	9.00	lobChIP_FOXA1inPBS
chr10	81184526	81184527	MACS_peak_1956	9.00	lobChIP_FOXA1inPBS
chr10	81842329	81842330	MACS_peak_1957	9.00	lobChIP_FOXA1inPBS
chr10	81972745	81972746	MACS_peak_1958	7.00	lobChIP_FOXA1inPBS
chr10	82032749	82032750	MACS_peak_1959	5.00	lobChIP_FOXA1inPBS
chr10	82050825	82050826	MACS_peak_1960	31.00	lobChIP_FOXA1inPBS
chr10	82174180	82174181	MACS_peak_1961	7.00	lobChIP_FOXA1inPBS
chr10	82253424	82253425	MACS_peak_1962	6.00	lobChIP_FOXA1inPBS
chr10	82266321	82266322	MACS_peak_1963	6.00	lobChIP_FOXA1inPBS
chr10	85576885	85576886	MACS_peak_1964	9.00	lobChIP_FOXA1inPBS
chr10	86087076	86087077	MACS_peak_1965	24.00	lobChIP_FOXA1inPBS
chr10	86148132	86148133	MACS_peak_1966	11.00	lobChIP_FOXA1inPBS
chr10	86189906	86189907	MACS_peak_1967	15.00	lobChIP_FOXA1inPBS
chr10	86258016	86258017	MACS_peak_1968	26.00	lobChIP_FOXA1inPBS
chr10	86636098	86636099	MACS_peak_1969	24.00	lobChIP_FOXA1inPBS
chr10	87647921	87647922	MACS_peak_1970	13.00	lobChIP_FOXA1inPBS
chr10	88170333	88170334	MACS_peak_1971	15.00	lobChIP_FOXA1inPBS
chr10	88198404	88198405	MACS_peak_1972	9.00	lobChIP_FOXA1inPBS
chr10	88200981	88200982	MACS_peak_1973	14.00	lobChIP_FOXA1inPBS
chr10	88362220	88362221	MACS_peak_1974	8.00	lobChIP_FOXA1inPBS
chr10	88534913	88534914	MACS_peak_1975	6.00	lobChIP_FOXA1inPBS
chr10	88621490	88621491	MACS_peak_1976	10.00	lobChIP_FOXA1inPBS
chr10	88622688	88622689	MACS_peak_1977	38.00	lobChIP_FOXA1inPBS
chr10	88852630	88852631	MACS_peak_1978	17.00	lobChIP_FOXA1inPBS
chr10	88930583	88930584	MACS_peak_1979	7.00	lobChIP_FOXA1inPBS
chr10	89260612	89260613	MACS_peak_1980	45.00	lobChIP_FOXA1inPBS
chr10	89349528	89349529	MACS_peak_1981	6.00	lobChIP_FOXA1inPBS
chr10	89378788	89378789	MACS_peak_1982	35.00	lobChIP_FOXA1inPBS
chr10	89406140	89406141	MACS_peak_1983	16.00	lobChIP_FOXA1inPBS
chr10	89503069	89503070	MACS_peak_1984	10.00	lobChIP_FOXA1inPBS
chr10	89523597	89523598	MACS_peak_1985	7.00	lobChIP_FOXA1inPBS
chr10	89568052	89568053	MACS_peak_1986	10.00	lobChIP_FOXA1inPBS
chr10	89602205	89602206	MACS_peak_1987	14.00	lobChIP_FOXA1inPBS
chr10	90463173	90463174	MACS_peak_1988	11.00	lobChIP_FOXA1inPBS
chr10	90945613	90945614	MACS_peak_1989	19.00	lobChIP_FOXA1inPBS
chr10	90977608	90977609	MACS_peak_1990	13.00	lobChIP_FOXA1inPBS
chr10	91002289	91002290	MACS_peak_1991	10.00	lobChIP_FOXA1inPBS
chr10	91109819	91109820	MACS_peak_1992	9.00	lobChIP_FOXA1inPBS
chr10	91133258	91133259	MACS_peak_1993	30.00	lobChIP_FOXA1inPBS
chr10	91134111	91134112	MACS_peak_1994	20.00	lobChIP_FOXA1inPBS
chr10	91235542	91235543	MACS_peak_1995	9.00	lobChIP_FOXA1inPBS
chr10	91403677	91403678	MACS_peak_1996	17.00	lobChIP_FOXA1inPBS
chr10	91405681	91405682	MACS_peak_1997	6.00	lobChIP_FOXA1inPBS
chr10	91574351	91574352	MACS_peak_1998	19.00	lobChIP_FOXA1inPBS
chr10	92631138	92631139	MACS_peak_1999	8.00	lobChIP_FOXA1inPBS
chr10	92681123	92681124	MACS_peak_2000	15.00	lobChIP_FOXA1inPBS
chr10	92691046	92691047	MACS_peak_2001	9.00	lobChIP_FOXA1inPBS
chr10	93193590	93193591	MACS_peak_2002	13.00	lobChIP_FOXA1inPBS
chr10	93253170	93253171	MACS_peak_2003	25.00	lobChIP_FOXA1inPBS
chr10	93316658	93316659	MACS_peak_2004	43.00	lobChIP_FOXA1inPBS
chr10	93518451	93518452	MACS_peak_2005	12.00	lobChIP_FOXA1inPBS
chr10	93842906	93842907	MACS_peak_2006	13.00	lobChIP_FOXA1inPBS
chr10	93871575	93871576	MACS_peak_2007	26.00	lobChIP_FOXA1inPBS
chr10	93874196	93874197	MACS_peak_2008	8.00	lobChIP_FOXA1inPBS
chr10	93881973	93881974	MACS_peak_2009	9.00	lobChIP_FOXA1inPBS
chr10	93882930	93882931	MACS_peak_2010	20.00	lobChIP_FOXA1inPBS
chr10	93883938	93883939	MACS_peak_2011	10.00	lobChIP_FOXA1inPBS
chr10	94050199	94050200	MACS_peak_2012	10.00	lobChIP_FOXA1inPBS
chr10	94340830	94340831	MACS_peak_2013	6.00	lobChIP_FOXA1inPBS
chr10	94453121	94453122	MACS_peak_2014	12.00	lobChIP_FOXA1inPBS
chr10	94457574	94457575	MACS_peak_2015	30.00	lobChIP_FOXA1inPBS
chr10	94471976	94471977	MACS_peak_2016	12.00	lobChIP_FOXA1inPBS
chr10	94516264	94516265	MACS_peak_2017	21.00	lobChIP_FOXA1inPBS
chr10	94518512	94518513	MACS_peak_2018	10.00	lobChIP_FOXA1inPBS
chr10	94547733	94547734	MACS_peak_2019	8.00	lobChIP_FOXA1inPBS
chr10	94663589	94663590	MACS_peak_2020	9.00	lobChIP_FOXA1inPBS
chr10	94725194	94725195	MACS_peak_2021	14.00	lobChIP_FOXA1inPBS
chr10	94866550	94866551	MACS_peak_2022	51.00	lobChIP_FOXA1inPBS
chr10	95014185	95014186	MACS_peak_2023	17.00	lobChIP_FOXA1inPBS
chr10	95205567	95205568	MACS_peak_2024	38.00	lobChIP_FOXA1inPBS
chr10	95354990	95354991	MACS_peak_2025	5.00	lobChIP_FOXA1inPBS
chr10	95361847	95361848	MACS_peak_2026	11.00	lobChIP_FOXA1inPBS
chr10	95430993	95430994	MACS_peak_2027	17.00	lobChIP_FOXA1inPBS
chr10	95883287	95883288	MACS_peak_2028	24.00	lobChIP_FOXA1inPBS
chr10	96023976	96023977	MACS_peak_2029	15.00	lobChIP_FOXA1inPBS
chr10	96074932	96074933	MACS_peak_2030	9.00	lobChIP_FOXA1inPBS
chr10	96076026	96076027	MACS_peak_2031	10.00	lobChIP_FOXA1inPBS
chr10	96163839	96163840	MACS_peak_2032	7.00	lobChIP_FOXA1inPBS
chr10	96222083	96222084	MACS_peak_2033	37.00	lobChIP_FOXA1inPBS
chr10	96440476	96440477	MACS_peak_2034	7.00	lobChIP_FOXA1inPBS
chr10	96662564	96662565	MACS_peak_2035	27.00	lobChIP_FOXA1inPBS
chr10	96898270	96898271	MACS_peak_2036	17.00	lobChIP_FOXA1inPBS
chr10	96938689	96938690	MACS_peak_2037	21.00	lobChIP_FOXA1inPBS
chr10	96941745	96941746	MACS_peak_2038	9.00	lobChIP_FOXA1inPBS
chr10	96981084	96981085	MACS_peak_2039	6.00	lobChIP_FOXA1inPBS
chr10	97033939	97033940	MACS_peak_2040	13.00	lobChIP_FOXA1inPBS
chr10	97037990	97037991	MACS_peak_2041	46.00	lobChIP_FOXA1inPBS
chr10	97092088	97092089	MACS_peak_2042	7.00	lobChIP_FOXA1inPBS
chr10	97095949	97095950	MACS_peak_2043	6.00	lobChIP_FOXA1inPBS
chr10	97110144	97110145	MACS_peak_2044	11.00	lobChIP_FOXA1inPBS
chr10	97120166	97120167	MACS_peak_2045	6.00	lobChIP_FOXA1inPBS
chr10	97148472	97148473	MACS_peak_2046	21.00	lobChIP_FOXA1inPBS
chr10	97212129	97212130	MACS_peak_2047	8.00	lobChIP_FOXA1inPBS
chr10	97217286	97217287	MACS_peak_2048	11.00	lobChIP_FOXA1inPBS
chr10	97217866	97217867	MACS_peak_2049	10.00	lobChIP_FOXA1inPBS
chr10	97232777	97232778	MACS_peak_2050	8.00	lobChIP_FOXA1inPBS
chr10	97235714	97235715	MACS_peak_2051	13.00	lobChIP_FOXA1inPBS
chr10	97240930	97240931	MACS_peak_2052	10.00	lobChIP_FOXA1inPBS
chr10	97259557	97259558	MACS_peak_2053	6.00	lobChIP_FOXA1inPBS
chr10	97261534	97261535	MACS_peak_2054	11.00	lobChIP_FOXA1inPBS
chr10	97273277	97273278	MACS_peak_2055	8.00	lobChIP_FOXA1inPBS
chr10	97302403	97302404	MACS_peak_2056	21.00	lobChIP_FOXA1inPBS
chr10	97395083	97395084	MACS_peak_2057	7.00	lobChIP_FOXA1inPBS
chr10	97400896	97400897	MACS_peak_2058	24.00	lobChIP_FOXA1inPBS
chr10	97416752	97416753	MACS_peak_2059	10.00	lobChIP_FOXA1inPBS
chr10	97502456	97502457	MACS_peak_2060	14.00	lobChIP_FOXA1inPBS
chr10	97578604	97578605	MACS_peak_2061	20.00	lobChIP_FOXA1inPBS
chr10	97596714	97596715	MACS_peak_2062	9.00	lobChIP_FOXA1inPBS
chr10	97853138	97853139	MACS_peak_2063	8.00	lobChIP_FOXA1inPBS
chr10	97853948	97853949	MACS_peak_2064	22.00	lobChIP_FOXA1inPBS
chr10	97885012	97885013	MACS_peak_2065	10.00	lobChIP_FOXA1inPBS
chr10	98356607	98356608	MACS_peak_2066	35.00	lobChIP_FOXA1inPBS
chr10	98430861	98430862	MACS_peak_2067	59.00	lobChIP_FOXA1inPBS
chr10	98433622	98433623	MACS_peak_2068	28.00	lobChIP_FOXA1inPBS
chr10	98437391	98437392	MACS_peak_2069	23.00	lobChIP_FOXA1inPBS
chr10	98479132	98479133	MACS_peak_2070	6.00	lobChIP_FOXA1inPBS
chr10	98558370	98558371	MACS_peak_2071	9.00	lobChIP_FOXA1inPBS
chr10	98600886	98600887	MACS_peak_2072	13.00	lobChIP_FOXA1inPBS
chr10	98602968	98602969	MACS_peak_2073	9.00	lobChIP_FOXA1inPBS
chr10	98624111	98624112	MACS_peak_2074	63.00	lobChIP_FOXA1inPBS
chr10	98786505	98786506	MACS_peak_2075	11.00	lobChIP_FOXA1inPBS
chr10	98900225	98900226	MACS_peak_2076	23.00	lobChIP_FOXA1inPBS
chr10	99039207	99039208	MACS_peak_2077	6.00	lobChIP_FOXA1inPBS
chr10	99061438	99061439	MACS_peak_2078	7.00	lobChIP_FOXA1inPBS
chr10	99087792	99087793	MACS_peak_2079	31.00	lobChIP_FOXA1inPBS
chr10	99088676	99088677	MACS_peak_2080	12.00	lobChIP_FOXA1inPBS
chr10	99094480	99094481	MACS_peak_2081	11.00	lobChIP_FOXA1inPBS
chr10	99285575	99285576	MACS_peak_2082	12.00	lobChIP_FOXA1inPBS
chr10	99400378	99400379	MACS_peak_2083	12.00	lobChIP_FOXA1inPBS
chr10	99445024	99445025	MACS_peak_2084	15.00	lobChIP_FOXA1inPBS
chr10	99519311	99519312	MACS_peak_2085	7.00	lobChIP_FOXA1inPBS
chr10	99621920	99621921	MACS_peak_2086	10.00	lobChIP_FOXA1inPBS
chr10	100030260	100030261	MACS_peak_2087	37.00	lobChIP_FOXA1inPBS
chr10	100092844	100092845	MACS_peak_2088	8.00	lobChIP_FOXA1inPBS
chr10	100250000	100250001	MACS_peak_2089	10.00	lobChIP_FOXA1inPBS
chr10	100376772	100376773	MACS_peak_2090	6.00	lobChIP_FOXA1inPBS
chr10	100444273	100444274	MACS_peak_2091	12.00	lobChIP_FOXA1inPBS
chr10	100892865	100892866	MACS_peak_2092	9.00	lobChIP_FOXA1inPBS
chr10	101149671	101149672	MACS_peak_2093	15.00	lobChIP_FOXA1inPBS
chr10	101155792	101155793	MACS_peak_2094	25.00	lobChIP_FOXA1inPBS
chr10	101181214	101181215	MACS_peak_2095	17.00	lobChIP_FOXA1inPBS
chr10	101210934	101210935	MACS_peak_2096	12.00	lobChIP_FOXA1inPBS
chr10	101407661	101407662	MACS_peak_2097	11.00	lobChIP_FOXA1inPBS
chr10	101514573	101514574	MACS_peak_2098	19.00	lobChIP_FOXA1inPBS
chr10	101542408	101542409	MACS_peak_2099	11.00	lobChIP_FOXA1inPBS
chr10	101574481	101574482	MACS_peak_2100	6.00	lobChIP_FOXA1inPBS
chr10	101609444	101609445	MACS_peak_2101	11.00	lobChIP_FOXA1inPBS
chr10	101687648	101687649	MACS_peak_2102	20.00	lobChIP_FOXA1inPBS
chr10	101768649	101768650	MACS_peak_2103	27.00	lobChIP_FOXA1inPBS
chr10	101780079	101780080	MACS_peak_2104	38.00	lobChIP_FOXA1inPBS
chr10	101832478	101832479	MACS_peak_2105	11.00	lobChIP_FOXA1inPBS
chr10	101841699	101841700	MACS_peak_2106	21.00	lobChIP_FOXA1inPBS
chr10	101845562	101845563	MACS_peak_2107	27.00	lobChIP_FOXA1inPBS
chr10	101860558	101860559	MACS_peak_2108	7.00	lobChIP_FOXA1inPBS
chr10	101861509	101861510	MACS_peak_2109	46.00	lobChIP_FOXA1inPBS
chr10	101923907	101923908	MACS_peak_2110	10.00	lobChIP_FOXA1inPBS
chr10	101927800	101927801	MACS_peak_2111	7.00	lobChIP_FOXA1inPBS
chr10	101939634	101939635	MACS_peak_2112	8.00	lobChIP_FOXA1inPBS
chr10	101943411	101943412	MACS_peak_2113	24.00	lobChIP_FOXA1inPBS
chr10	102098410	102098411	MACS_peak_2114	12.00	lobChIP_FOXA1inPBS
chr10	102101852	102101853	MACS_peak_2115	16.00	lobChIP_FOXA1inPBS
chr10	102119718	102119719	MACS_peak_2116	9.00	lobChIP_FOXA1inPBS
chr10	102152292	102152293	MACS_peak_2117	22.00	lobChIP_FOXA1inPBS
chr10	102407916	102407917	MACS_peak_2118	7.00	lobChIP_FOXA1inPBS
chr10	102635707	102635708	MACS_peak_2119	31.00	lobChIP_FOXA1inPBS
chr10	102641229	102641230	MACS_peak_2120	13.00	lobChIP_FOXA1inPBS
chr10	102656887	102656888	MACS_peak_2121	9.00	lobChIP_FOXA1inPBS
chr10	102730314	102730315	MACS_peak_2122	8.00	lobChIP_FOXA1inPBS
chr10	102731031	102731032	MACS_peak_2123	43.00	lobChIP_FOXA1inPBS
chr10	102851802	102851803	MACS_peak_2124	10.00	lobChIP_FOXA1inPBS
chr10	102872979	102872980	MACS_peak_2125	18.00	lobChIP_FOXA1inPBS
chr10	103578444	103578445	MACS_peak_2126	8.00	lobChIP_FOXA1inPBS
chr10	103721862	103721863	MACS_peak_2127	10.00	lobChIP_FOXA1inPBS
chr10	104112106	104112107	MACS_peak_2128	10.00	lobChIP_FOXA1inPBS
chr10	104222834	104222835	MACS_peak_2129	9.00	lobChIP_FOXA1inPBS
chr10	104529218	104529219	MACS_peak_2130	18.00	lobChIP_FOXA1inPBS
chr10	104574875	104574876	MACS_peak_2131	12.00	lobChIP_FOXA1inPBS
chr10	104601779	104601780	MACS_peak_2132	12.00	lobChIP_FOXA1inPBS
chr10	104615420	104615421	MACS_peak_2133	7.00	lobChIP_FOXA1inPBS
chr10	104636579	104636580	MACS_peak_2134	14.00	lobChIP_FOXA1inPBS
chr10	104670017	104670018	MACS_peak_2135	9.00	lobChIP_FOXA1inPBS
chr10	104723234	104723235	MACS_peak_2136	12.00	lobChIP_FOXA1inPBS
chr10	104748864	104748865	MACS_peak_2137	35.00	lobChIP_FOXA1inPBS
chr10	104757929	104757930	MACS_peak_2138	9.00	lobChIP_FOXA1inPBS
chr10	104766944	104766945	MACS_peak_2139	8.00	lobChIP_FOXA1inPBS
chr10	104785669	104785670	MACS_peak_2140	17.00	lobChIP_FOXA1inPBS
chr10	104813631	104813632	MACS_peak_2141	12.00	lobChIP_FOXA1inPBS
chr10	104881242	104881243	MACS_peak_2142	6.00	lobChIP_FOXA1inPBS
chr10	104890849	104890850	MACS_peak_2143	8.00	lobChIP_FOXA1inPBS
chr10	104913431	104913432	MACS_peak_2144	10.00	lobChIP_FOXA1inPBS
chr10	104914853	104914854	MACS_peak_2145	14.00	lobChIP_FOXA1inPBS
chr10	104947417	104947418	MACS_peak_2146	10.00	lobChIP_FOXA1inPBS
chr10	104991127	104991128	MACS_peak_2147	18.00	lobChIP_FOXA1inPBS
chr10	105241861	105241862	MACS_peak_2148	6.00	lobChIP_FOXA1inPBS
chr10	105264293	105264294	MACS_peak_2149	14.00	lobChIP_FOXA1inPBS
chr10	105336779	105336780	MACS_peak_2150	26.00	lobChIP_FOXA1inPBS
chr10	105358788	105358789	MACS_peak_2151	24.00	lobChIP_FOXA1inPBS
chr10	105554142	105554143	MACS_peak_2152	50.00	lobChIP_FOXA1inPBS
chr10	105613946	105613947	MACS_peak_2153	9.00	lobChIP_FOXA1inPBS
chr10	105650161	105650162	MACS_peak_2154	7.00	lobChIP_FOXA1inPBS
chr10	106010814	106010815	MACS_peak_2155	7.00	lobChIP_FOXA1inPBS
chr10	106013102	106013103	MACS_peak_2156	42.00	lobChIP_FOXA1inPBS
chr10	106016224	106016225	MACS_peak_2157	9.00	lobChIP_FOXA1inPBS
chr10	106048341	106048342	MACS_peak_2158	29.00	lobChIP_FOXA1inPBS
chr10	106109472	106109473	MACS_peak_2159	11.00	lobChIP_FOXA1inPBS
chr10	107466527	107466528	MACS_peak_2160	10.00	lobChIP_FOXA1inPBS
chr10	111725389	111725390	MACS_peak_2161	28.00	lobChIP_FOXA1inPBS
chr10	111731611	111731612	MACS_peak_2162	11.00	lobChIP_FOXA1inPBS
chr10	111734668	111734669	MACS_peak_2163	23.00	lobChIP_FOXA1inPBS
chr10	111753805	111753806	MACS_peak_2164	9.00	lobChIP_FOXA1inPBS
chr10	111836809	111836810	MACS_peak_2165	13.00	lobChIP_FOXA1inPBS
chr10	111837730	111837731	MACS_peak_2166	10.00	lobChIP_FOXA1inPBS
chr10	112186107	112186108	MACS_peak_2167	8.00	lobChIP_FOXA1inPBS
chr10	112282615	112282616	MACS_peak_2168	6.00	lobChIP_FOXA1inPBS
chr10	112329677	112329678	MACS_peak_2169	15.00	lobChIP_FOXA1inPBS
chr10	112495676	112495677	MACS_peak_2170	10.00	lobChIP_FOXA1inPBS
chr10	112600258	112600259	MACS_peak_2171	14.00	lobChIP_FOXA1inPBS
chr10	112617443	112617444	MACS_peak_2172	9.00	lobChIP_FOXA1inPBS
chr10	112624568	112624569	MACS_peak_2173	10.00	lobChIP_FOXA1inPBS
chr10	112636805	112636806	MACS_peak_2174	12.00	lobChIP_FOXA1inPBS
chr10	113937297	113937298	MACS_peak_2175	8.00	lobChIP_FOXA1inPBS
chr10	113985814	113985815	MACS_peak_2176	7.00	lobChIP_FOXA1inPBS
chr10	113987447	113987448	MACS_peak_2177	16.00	lobChIP_FOXA1inPBS
chr10	114018328	114018329	MACS_peak_2178	9.00	lobChIP_FOXA1inPBS
chr10	114021289	114021290	MACS_peak_2179	9.00	lobChIP_FOXA1inPBS
chr10	114035676	114035677	MACS_peak_2180	18.00	lobChIP_FOXA1inPBS
chr10	114046995	114046996	MACS_peak_2181	17.00	lobChIP_FOXA1inPBS
chr10	114056404	114056405	MACS_peak_2182	7.00	lobChIP_FOXA1inPBS
chr10	114064481	114064482	MACS_peak_2183	17.00	lobChIP_FOXA1inPBS
chr10	114092022	114092023	MACS_peak_2184	6.00	lobChIP_FOXA1inPBS
chr10	114125245	114125246	MACS_peak_2185	8.00	lobChIP_FOXA1inPBS
chr10	114133803	114133804	MACS_peak_2186	21.00	lobChIP_FOXA1inPBS
chr10	114151361	114151362	MACS_peak_2187	11.00	lobChIP_FOXA1inPBS
chr10	114154517	114154518	MACS_peak_2188	51.00	lobChIP_FOXA1inPBS
chr10	114250494	114250495	MACS_peak_2189	16.00	lobChIP_FOXA1inPBS
chr10	114322024	114322025	MACS_peak_2190	8.00	lobChIP_FOXA1inPBS
chr10	114323321	114323322	MACS_peak_2191	40.00	lobChIP_FOXA1inPBS
chr10	114324324	114324325	MACS_peak_2192	29.00	lobChIP_FOXA1inPBS
chr10	114325342	114325343	MACS_peak_2193	9.00	lobChIP_FOXA1inPBS
chr10	114391616	114391617	MACS_peak_2194	8.00	lobChIP_FOXA1inPBS
chr10	114397950	114397951	MACS_peak_2195	8.00	lobChIP_FOXA1inPBS
chr10	114403538	114403539	MACS_peak_2196	45.00	lobChIP_FOXA1inPBS
chr10	114418940	114418941	MACS_peak_2197	40.00	lobChIP_FOXA1inPBS
chr10	114438446	114438447	MACS_peak_2198	6.00	lobChIP_FOXA1inPBS
chr10	114455362	114455363	MACS_peak_2199	14.00	lobChIP_FOXA1inPBS
chr10	114476926	114476927	MACS_peak_2200	14.00	lobChIP_FOXA1inPBS
chr10	114488287	114488288	MACS_peak_2201	25.00	lobChIP_FOXA1inPBS
chr10	114499562	114499563	MACS_peak_2202	47.00	lobChIP_FOXA1inPBS
chr10	114500755	114500756	MACS_peak_2203	6.00	lobChIP_FOXA1inPBS
chr10	114502095	114502096	MACS_peak_2204	15.00	lobChIP_FOXA1inPBS
chr10	114502769	114502770	MACS_peak_2205	10.00	lobChIP_FOXA1inPBS
chr10	114571782	114571783	MACS_peak_2206	59.00	lobChIP_FOXA1inPBS
chr10	114575718	114575719	MACS_peak_2207	8.00	lobChIP_FOXA1inPBS
chr10	114729593	114729594	MACS_peak_2208	11.00	lobChIP_FOXA1inPBS
chr10	114779577	114779578	MACS_peak_2209	12.00	lobChIP_FOXA1inPBS
chr10	114795698	114795699	MACS_peak_2210	14.00	lobChIP_FOXA1inPBS
chr10	114833779	114833780	MACS_peak_2211	11.00	lobChIP_FOXA1inPBS
chr10	114840748	114840749	MACS_peak_2212	8.00	lobChIP_FOXA1inPBS
chr10	114847316	114847317	MACS_peak_2213	29.00	lobChIP_FOXA1inPBS
chr10	114853409	114853410	MACS_peak_2214	34.00	lobChIP_FOXA1inPBS
chr10	116249244	116249245	MACS_peak_2215	11.00	lobChIP_FOXA1inPBS
chr10	116278454	116278455	MACS_peak_2216	6.00	lobChIP_FOXA1inPBS
chr10	116316538	116316539	MACS_peak_2217	10.00	lobChIP_FOXA1inPBS
chr10	116383824	116383825	MACS_peak_2218	11.00	lobChIP_FOXA1inPBS
chr10	116474388	116474389	MACS_peak_2219	9.00	lobChIP_FOXA1inPBS
chr10	117022057	117022058	MACS_peak_2220	7.00	lobChIP_FOXA1inPBS
chr10	117205007	117205008	MACS_peak_2221	18.00	lobChIP_FOXA1inPBS
chr10	117326602	117326603	MACS_peak_2222	36.00	lobChIP_FOXA1inPBS
chr10	117816530	117816531	MACS_peak_2223	26.00	lobChIP_FOXA1inPBS
chr10	118349625	118349626	MACS_peak_2224	14.00	lobChIP_FOXA1inPBS
chr10	118598480	118598481	MACS_peak_2225	10.00	lobChIP_FOXA1inPBS
chr10	118762932	118762933	MACS_peak_2226	10.00	lobChIP_FOXA1inPBS
chr10	119120972	119120973	MACS_peak_2227	43.00	lobChIP_FOXA1inPBS
chr10	119294119	119294120	MACS_peak_2228	10.00	lobChIP_FOXA1inPBS
chr10	119478634	119478635	MACS_peak_2229	12.00	lobChIP_FOXA1inPBS
chr10	120070471	120070472	MACS_peak_2230	12.00	lobChIP_FOXA1inPBS
chr10	120499786	120499787	MACS_peak_2231	7.00	lobChIP_FOXA1inPBS
chr10	120873504	120873505	MACS_peak_2232	13.00	lobChIP_FOXA1inPBS
chr10	120880351	120880352	MACS_peak_2233	6.00	lobChIP_FOXA1inPBS
chr10	120937341	120937342	MACS_peak_2234	48.00	lobChIP_FOXA1inPBS
chr10	121013460	121013461	MACS_peak_2235	9.00	lobChIP_FOXA1inPBS
chr10	121076796	121076797	MACS_peak_2236	16.00	lobChIP_FOXA1inPBS
chr10	121258530	121258531	MACS_peak_2237	10.00	lobChIP_FOXA1inPBS
chr10	121544370	121544371	MACS_peak_2238	6.00	lobChIP_FOXA1inPBS
chr10	121545790	121545791	MACS_peak_2239	11.00	lobChIP_FOXA1inPBS
chr10	121655840	121655841	MACS_peak_2240	9.00	lobChIP_FOXA1inPBS
chr10	122799177	122799178	MACS_peak_2241	8.00	lobChIP_FOXA1inPBS
chr10	122802748	122802749	MACS_peak_2242	11.00	lobChIP_FOXA1inPBS
chr10	122823448	122823449	MACS_peak_2243	26.00	lobChIP_FOXA1inPBS
chr10	122914082	122914083	MACS_peak_2244	8.00	lobChIP_FOXA1inPBS
chr10	122915469	122915470	MACS_peak_2245	12.00	lobChIP_FOXA1inPBS
chr10	123127854	123127855	MACS_peak_2246	8.00	lobChIP_FOXA1inPBS
chr10	123283613	123283614	MACS_peak_2247	22.00	lobChIP_FOXA1inPBS
chr10	123365269	123365270	MACS_peak_2248	10.00	lobChIP_FOXA1inPBS
chr10	123471506	123471507	MACS_peak_2249	9.00	lobChIP_FOXA1inPBS
chr10	123532776	123532777	MACS_peak_2250	11.00	lobChIP_FOXA1inPBS
chr10	123603044	123603045	MACS_peak_2251	17.00	lobChIP_FOXA1inPBS
chr10	123614440	123614441	MACS_peak_2252	9.00	lobChIP_FOXA1inPBS
chr10	123754983	123754984	MACS_peak_2253	5.00	lobChIP_FOXA1inPBS
chr10	123767152	123767153	MACS_peak_2254	8.00	lobChIP_FOXA1inPBS
chr10	123804348	123804349	MACS_peak_2255	7.00	lobChIP_FOXA1inPBS
chr10	123814990	123814991	MACS_peak_2256	8.00	lobChIP_FOXA1inPBS
chr10	123816404	123816405	MACS_peak_2257	7.00	lobChIP_FOXA1inPBS
chr10	123822901	123822902	MACS_peak_2258	44.00	lobChIP_FOXA1inPBS
chr10	123861512	123861513	MACS_peak_2259	8.00	lobChIP_FOXA1inPBS
chr10	123906605	123906606	MACS_peak_2260	10.00	lobChIP_FOXA1inPBS
chr10	123935872	123935873	MACS_peak_2261	12.00	lobChIP_FOXA1inPBS
chr10	124063388	124063389	MACS_peak_2262	6.00	lobChIP_FOXA1inPBS
chr10	124070339	124070340	MACS_peak_2263	12.00	lobChIP_FOXA1inPBS
chr10	124075916	124075917	MACS_peak_2264	6.00	lobChIP_FOXA1inPBS
chr10	124091403	124091404	MACS_peak_2265	9.00	lobChIP_FOXA1inPBS
chr10	124131438	124131439	MACS_peak_2266	10.00	lobChIP_FOXA1inPBS
chr10	124717294	124717295	MACS_peak_2267	10.00	lobChIP_FOXA1inPBS
chr10	124779714	124779715	MACS_peak_2268	100.00	lobChIP_FOXA1inPBS
chr10	124916122	124916123	MACS_peak_2269	9.00	lobChIP_FOXA1inPBS
chr10	124916848	124916849	MACS_peak_2270	10.00	lobChIP_FOXA1inPBS
chr10	124917524	124917525	MACS_peak_2271	7.00	lobChIP_FOXA1inPBS
chr10	124963498	124963499	MACS_peak_2272	9.00	lobChIP_FOXA1inPBS
chr10	125211306	125211307	MACS_peak_2273	8.00	lobChIP_FOXA1inPBS
chr10	125266986	125266987	MACS_peak_2274	8.00	lobChIP_FOXA1inPBS
chr10	125795694	125795695	MACS_peak_2275	15.00	lobChIP_FOXA1inPBS
chr10	125823490	125823491	MACS_peak_2276	14.00	lobChIP_FOXA1inPBS
chr10	126105429	126105430	MACS_peak_2277	10.00	lobChIP_FOXA1inPBS
chr10	126114297	126114298	MACS_peak_2278	8.00	lobChIP_FOXA1inPBS
chr10	126156577	126156578	MACS_peak_2279	8.00	lobChIP_FOXA1inPBS
chr10	126159424	126159425	MACS_peak_2280	35.00	lobChIP_FOXA1inPBS
chr10	126197611	126197612	MACS_peak_2281	11.00	lobChIP_FOXA1inPBS
chr10	126211678	126211679	MACS_peak_2282	11.00	lobChIP_FOXA1inPBS
chr10	126413189	126413190	MACS_peak_2283	13.00	lobChIP_FOXA1inPBS
chr10	126429114	126429115	MACS_peak_2284	7.00	lobChIP_FOXA1inPBS
chr10	126433469	126433470	MACS_peak_2285	23.00	lobChIP_FOXA1inPBS
chr10	126553603	126553604	MACS_peak_2286	10.00	lobChIP_FOXA1inPBS
chr10	126598285	126598286	MACS_peak_2287	10.00	lobChIP_FOXA1inPBS
chr10	127481280	127481281	MACS_peak_2288	15.00	lobChIP_FOXA1inPBS
chr10	127529721	127529722	MACS_peak_2289	9.00	lobChIP_FOXA1inPBS
chr10	127600761	127600762	MACS_peak_2290	56.00	lobChIP_FOXA1inPBS
chr10	128932103	128932104	MACS_peak_2291	35.00	lobChIP_FOXA1inPBS
chr10	129077811	129077812	MACS_peak_2292	21.00	lobChIP_FOXA1inPBS
chr10	129099761	129099762	MACS_peak_2293	10.00	lobChIP_FOXA1inPBS
chr10	129955418	129955419	MACS_peak_2294	9.00	lobChIP_FOXA1inPBS
chr10	130430217	130430218	MACS_peak_2295	9.00	lobChIP_FOXA1inPBS
chr10	131260662	131260663	MACS_peak_2296	42.00	lobChIP_FOXA1inPBS
chr10	131266305	131266306	MACS_peak_2297	8.00	lobChIP_FOXA1inPBS
chr10	131440847	131440848	MACS_peak_2298	24.00	lobChIP_FOXA1inPBS
chr10	131664433	131664434	MACS_peak_2299	9.00	lobChIP_FOXA1inPBS
chr10	131754377	131754378	MACS_peak_2300	27.00	lobChIP_FOXA1inPBS
chr10	133473764	133473765	MACS_peak_2301	13.00	lobChIP_FOXA1inPBS
chr10	133794310	133794311	MACS_peak_2302	8.00	lobChIP_FOXA1inPBS
chr10	133811443	133811444	MACS_peak_2303	12.00	lobChIP_FOXA1inPBS
chr10	134125035	134125036	MACS_peak_2304	10.00	lobChIP_FOXA1inPBS
chr10	134404791	134404792	MACS_peak_2305	9.00	lobChIP_FOXA1inPBS
chr10	134499264	134499265	MACS_peak_2306	9.00	lobChIP_FOXA1inPBS
chr10	135290282	135290283	MACS_peak_2307	9.00	lobChIP_FOXA1inPBS
chr11	305629	305630	MACS_peak_2308	11.00	lobChIP_FOXA1inPBS
chr11	353731	353732	MACS_peak_2309	6.00	lobChIP_FOXA1inPBS
chr11	655363	655364	MACS_peak_2310	12.00	lobChIP_FOXA1inPBS
chr11	705125	705126	MACS_peak_2311	11.00	lobChIP_FOXA1inPBS
chr11	844351	844352	MACS_peak_2312	11.00	lobChIP_FOXA1inPBS
chr11	952356	952357	MACS_peak_2313	7.00	lobChIP_FOXA1inPBS
chr11	1068242	1068243	MACS_peak_2314	14.00	lobChIP_FOXA1inPBS
chr11	1241248	1241249	MACS_peak_2315	60.00	lobChIP_FOXA1inPBS
chr11	1325912	1325913	MACS_peak_2316	8.00	lobChIP_FOXA1inPBS
chr11	1695232	1695233	MACS_peak_2317	10.00	lobChIP_FOXA1inPBS
chr11	1697404	1697405	MACS_peak_2318	7.00	lobChIP_FOXA1inPBS
chr11	1711261	1711262	MACS_peak_2319	9.00	lobChIP_FOXA1inPBS
chr11	1712636	1712637	MACS_peak_2320	6.00	lobChIP_FOXA1inPBS
chr11	2004434	2004435	MACS_peak_2321	10.00	lobChIP_FOXA1inPBS
chr11	2009542	2009543	MACS_peak_2322	19.00	lobChIP_FOXA1inPBS
chr11	2011259	2011260	MACS_peak_2323	12.00	lobChIP_FOXA1inPBS
chr11	2036407	2036408	MACS_peak_2324	5.00	lobChIP_FOXA1inPBS
chr11	2232448	2232449	MACS_peak_2325	24.00	lobChIP_FOXA1inPBS
chr11	2585036	2585037	MACS_peak_2326	22.00	lobChIP_FOXA1inPBS
chr11	2716786	2716787	MACS_peak_2327	14.00	lobChIP_FOXA1inPBS
chr11	3400608	3400609	MACS_peak_2328	10.00	lobChIP_FOXA1inPBS
chr11	3802794	3802795	MACS_peak_2329	8.00	lobChIP_FOXA1inPBS
chr11	3888509	3888510	MACS_peak_2330	8.00	lobChIP_FOXA1inPBS
chr11	3926843	3926844	MACS_peak_2331	13.00	lobChIP_FOXA1inPBS
chr11	3954666	3954667	MACS_peak_2332	23.00	lobChIP_FOXA1inPBS
chr11	3963900	3963901	MACS_peak_2333	37.00	lobChIP_FOXA1inPBS
chr11	3981076	3981077	MACS_peak_2334	13.00	lobChIP_FOXA1inPBS
chr11	5642632	5642633	MACS_peak_2335	25.00	lobChIP_FOXA1inPBS
chr11	6303430	6303431	MACS_peak_2336	11.00	lobChIP_FOXA1inPBS
chr11	6409411	6409412	MACS_peak_2337	31.00	lobChIP_FOXA1inPBS
chr11	6455417	6455418	MACS_peak_2338	18.00	lobChIP_FOXA1inPBS
chr11	6462335	6462336	MACS_peak_2339	33.00	lobChIP_FOXA1inPBS
chr11	6462987	6462988	MACS_peak_2340	9.00	lobChIP_FOXA1inPBS
chr11	6633946	6633947	MACS_peak_2341	12.00	lobChIP_FOXA1inPBS
chr11	6676029	6676030	MACS_peak_2342	10.00	lobChIP_FOXA1inPBS
chr11	7593717	7593718	MACS_peak_2343	22.00	lobChIP_FOXA1inPBS
chr11	7621550	7621551	MACS_peak_2344	8.00	lobChIP_FOXA1inPBS
chr11	7625090	7625091	MACS_peak_2345	8.00	lobChIP_FOXA1inPBS
chr11	8127653	8127654	MACS_peak_2346	7.00	lobChIP_FOXA1inPBS
chr11	8700784	8700785	MACS_peak_2347	13.00	lobChIP_FOXA1inPBS
chr11	8739852	8739853	MACS_peak_2348	5.00	lobChIP_FOXA1inPBS
chr11	9354371	9354372	MACS_peak_2349	25.00	lobChIP_FOXA1inPBS
chr11	9532426	9532427	MACS_peak_2350	88.00	lobChIP_FOXA1inPBS
chr11	9587557	9587558	MACS_peak_2351	8.00	lobChIP_FOXA1inPBS
chr11	9591000	9591001	MACS_peak_2352	28.00	lobChIP_FOXA1inPBS
chr11	9594387	9594388	MACS_peak_2353	18.00	lobChIP_FOXA1inPBS
chr11	9634827	9634828	MACS_peak_2354	20.00	lobChIP_FOXA1inPBS
chr11	9758103	9758104	MACS_peak_2355	8.00	lobChIP_FOXA1inPBS
chr11	9878893	9878894	MACS_peak_2356	13.00	lobChIP_FOXA1inPBS
chr11	9928210	9928211	MACS_peak_2357	11.00	lobChIP_FOXA1inPBS
chr11	9952648	9952649	MACS_peak_2358	9.00	lobChIP_FOXA1inPBS
chr11	9954547	9954548	MACS_peak_2359	8.00	lobChIP_FOXA1inPBS
chr11	10072291	10072292	MACS_peak_2360	16.00	lobChIP_FOXA1inPBS
chr11	10090157	10090158	MACS_peak_2361	9.00	lobChIP_FOXA1inPBS
chr11	10109020	10109021	MACS_peak_2362	11.00	lobChIP_FOXA1inPBS
chr11	10307122	10307123	MACS_peak_2363	48.00	lobChIP_FOXA1inPBS
chr11	10344708	10344709	MACS_peak_2364	7.00	lobChIP_FOXA1inPBS
chr11	10348169	10348170	MACS_peak_2365	28.00	lobChIP_FOXA1inPBS
chr11	10373524	10373525	MACS_peak_2366	9.00	lobChIP_FOXA1inPBS
chr11	10869844	10869845	MACS_peak_2367	6.00	lobChIP_FOXA1inPBS
chr11	10949263	10949264	MACS_peak_2368	7.00	lobChIP_FOXA1inPBS
chr11	12014208	12014209	MACS_peak_2369	51.00	lobChIP_FOXA1inPBS
chr11	12093301	12093302	MACS_peak_2370	7.00	lobChIP_FOXA1inPBS
chr11	12240636	12240637	MACS_peak_2371	24.00	lobChIP_FOXA1inPBS
chr11	12270136	12270137	MACS_peak_2372	8.00	lobChIP_FOXA1inPBS
chr11	12388045	12388046	MACS_peak_2373	7.00	lobChIP_FOXA1inPBS
chr11	12621092	12621093	MACS_peak_2374	7.00	lobChIP_FOXA1inPBS
chr11	12691742	12691743	MACS_peak_2375	9.00	lobChIP_FOXA1inPBS
chr11	12715815	12715816	MACS_peak_2376	7.00	lobChIP_FOXA1inPBS
chr11	12728853	12728854	MACS_peak_2377	11.00	lobChIP_FOXA1inPBS
chr11	12824519	12824520	MACS_peak_2378	6.00	lobChIP_FOXA1inPBS
chr11	12850126	12850127	MACS_peak_2379	11.00	lobChIP_FOXA1inPBS
chr11	12867643	12867644	MACS_peak_2380	14.00	lobChIP_FOXA1inPBS
chr11	13033193	13033194	MACS_peak_2381	12.00	lobChIP_FOXA1inPBS
chr11	13033715	13033716	MACS_peak_2382	14.00	lobChIP_FOXA1inPBS
chr11	13064754	13064755	MACS_peak_2383	10.00	lobChIP_FOXA1inPBS
chr11	14588303	14588304	MACS_peak_2384	16.00	lobChIP_FOXA1inPBS
chr11	14634370	14634371	MACS_peak_2385	7.00	lobChIP_FOXA1inPBS
chr11	14641750	14641751	MACS_peak_2386	36.00	lobChIP_FOXA1inPBS
chr11	14691393	14691394	MACS_peak_2387	6.00	lobChIP_FOXA1inPBS
chr11	14697808	14697809	MACS_peak_2388	19.00	lobChIP_FOXA1inPBS
chr11	14762429	14762430	MACS_peak_2389	8.00	lobChIP_FOXA1inPBS
chr11	14762919	14762920	MACS_peak_2390	10.00	lobChIP_FOXA1inPBS
chr11	14839481	14839482	MACS_peak_2391	7.00	lobChIP_FOXA1inPBS
chr11	14840448	14840449	MACS_peak_2392	14.00	lobChIP_FOXA1inPBS
chr11	15969727	15969728	MACS_peak_2393	7.00	lobChIP_FOXA1inPBS
chr11	16023686	16023687	MACS_peak_2394	11.00	lobChIP_FOXA1inPBS
chr11	16136663	16136664	MACS_peak_2395	33.00	lobChIP_FOXA1inPBS
chr11	16176331	16176332	MACS_peak_2396	17.00	lobChIP_FOXA1inPBS
chr11	16206321	16206322	MACS_peak_2397	21.00	lobChIP_FOXA1inPBS
chr11	16827683	16827684	MACS_peak_2398	9.00	lobChIP_FOXA1inPBS
chr11	17003484	17003485	MACS_peak_2399	9.00	lobChIP_FOXA1inPBS
chr11	17026492	17026493	MACS_peak_2400	15.00	lobChIP_FOXA1inPBS
chr11	17070236	17070237	MACS_peak_2401	9.00	lobChIP_FOXA1inPBS
chr11	17316923	17316924	MACS_peak_2402	10.00	lobChIP_FOXA1inPBS
chr11	17461181	17461182	MACS_peak_2403	41.00	lobChIP_FOXA1inPBS
chr11	17791728	17791729	MACS_peak_2404	52.00	lobChIP_FOXA1inPBS
chr11	18008105	18008106	MACS_peak_2405	5.00	lobChIP_FOXA1inPBS
chr11	18022548	18022549	MACS_peak_2406	9.00	lobChIP_FOXA1inPBS
chr11	18140317	18140318	MACS_peak_2407	12.00	lobChIP_FOXA1inPBS
chr11	18245707	18245708	MACS_peak_2408	21.00	lobChIP_FOXA1inPBS
chr11	18258746	18258747	MACS_peak_2409	7.00	lobChIP_FOXA1inPBS
chr11	18597034	18597035	MACS_peak_2410	7.00	lobChIP_FOXA1inPBS
chr11	18600790	18600791	MACS_peak_2411	20.00	lobChIP_FOXA1inPBS
chr11	18603601	18603602	MACS_peak_2412	7.00	lobChIP_FOXA1inPBS
chr11	18672998	18672999	MACS_peak_2413	6.00	lobChIP_FOXA1inPBS
chr11	19929032	19929033	MACS_peak_2414	13.00	lobChIP_FOXA1inPBS
chr11	19932658	19932659	MACS_peak_2415	8.00	lobChIP_FOXA1inPBS
chr11	19933862	19933863	MACS_peak_2416	23.00	lobChIP_FOXA1inPBS
chr11	19974005	19974006	MACS_peak_2417	16.00	lobChIP_FOXA1inPBS
chr11	19993839	19993840	MACS_peak_2418	7.00	lobChIP_FOXA1inPBS
chr11	20000466	20000467	MACS_peak_2419	8.00	lobChIP_FOXA1inPBS
chr11	20022239	20022240	MACS_peak_2420	12.00	lobChIP_FOXA1inPBS
chr11	20024516	20024517	MACS_peak_2421	10.00	lobChIP_FOXA1inPBS
chr11	20033067	20033068	MACS_peak_2422	11.00	lobChIP_FOXA1inPBS
chr11	20045237	20045238	MACS_peak_2423	8.00	lobChIP_FOXA1inPBS
chr11	20052743	20052744	MACS_peak_2424	19.00	lobChIP_FOXA1inPBS
chr11	20057962	20057963	MACS_peak_2425	10.00	lobChIP_FOXA1inPBS
chr11	20111872	20111873	MACS_peak_2426	26.00	lobChIP_FOXA1inPBS
chr11	20499116	20499117	MACS_peak_2427	8.00	lobChIP_FOXA1inPBS
chr11	20522935	20522936	MACS_peak_2428	5.00	lobChIP_FOXA1inPBS
chr11	20540077	20540078	MACS_peak_2429	15.00	lobChIP_FOXA1inPBS
chr11	22696198	22696199	MACS_peak_2430	38.00	lobChIP_FOXA1inPBS
chr11	22752109	22752110	MACS_peak_2431	13.00	lobChIP_FOXA1inPBS
chr11	22814134	22814135	MACS_peak_2432	10.00	lobChIP_FOXA1inPBS
chr11	25456838	25456839	MACS_peak_2433	8.00	lobChIP_FOXA1inPBS
chr11	25758862	25758863	MACS_peak_2434	40.00	lobChIP_FOXA1inPBS
chr11	26209400	26209401	MACS_peak_2435	12.00	lobChIP_FOXA1inPBS
chr11	27047068	27047069	MACS_peak_2436	10.00	lobChIP_FOXA1inPBS
chr11	27195490	27195491	MACS_peak_2437	12.00	lobChIP_FOXA1inPBS
chr11	27209051	27209052	MACS_peak_2438	9.00	lobChIP_FOXA1inPBS
chr11	27223701	27223702	MACS_peak_2439	18.00	lobChIP_FOXA1inPBS
chr11	27227129	27227130	MACS_peak_2440	9.00	lobChIP_FOXA1inPBS
chr11	27318282	27318283	MACS_peak_2441	7.00	lobChIP_FOXA1inPBS
chr11	27333916	27333917	MACS_peak_2442	10.00	lobChIP_FOXA1inPBS
chr11	27343135	27343136	MACS_peak_2443	7.00	lobChIP_FOXA1inPBS
chr11	27352297	27352298	MACS_peak_2444	16.00	lobChIP_FOXA1inPBS
chr11	27353207	27353208	MACS_peak_2445	9.00	lobChIP_FOXA1inPBS
chr11	27396018	27396019	MACS_peak_2446	35.00	lobChIP_FOXA1inPBS
chr11	27417215	27417216	MACS_peak_2447	11.00	lobChIP_FOXA1inPBS
chr11	27423715	27423716	MACS_peak_2448	26.00	lobChIP_FOXA1inPBS
chr11	27478739	27478740	MACS_peak_2449	10.00	lobChIP_FOXA1inPBS
chr11	27497370	27497371	MACS_peak_2450	28.00	lobChIP_FOXA1inPBS
chr11	29303882	29303883	MACS_peak_2451	10.00	lobChIP_FOXA1inPBS
chr11	29343027	29343028	MACS_peak_2452	10.00	lobChIP_FOXA1inPBS
chr11	30510425	30510426	MACS_peak_2453	49.00	lobChIP_FOXA1inPBS
chr11	31081295	31081296	MACS_peak_2454	11.00	lobChIP_FOXA1inPBS
chr11	31091180	31091181	MACS_peak_2455	9.00	lobChIP_FOXA1inPBS
chr11	31706111	31706112	MACS_peak_2456	18.00	lobChIP_FOXA1inPBS
chr11	31724637	31724638	MACS_peak_2457	5.00	lobChIP_FOXA1inPBS
chr11	31833613	31833614	MACS_peak_2458	17.00	lobChIP_FOXA1inPBS
chr11	32125131	32125132	MACS_peak_2459	37.00	lobChIP_FOXA1inPBS
chr11	32602590	32602591	MACS_peak_2460	9.00	lobChIP_FOXA1inPBS
chr11	32625058	32625059	MACS_peak_2461	7.00	lobChIP_FOXA1inPBS
chr11	32913236	32913237	MACS_peak_2462	6.00	lobChIP_FOXA1inPBS
chr11	33204285	33204286	MACS_peak_2463	10.00	lobChIP_FOXA1inPBS
chr11	33288253	33288254	MACS_peak_2464	7.00	lobChIP_FOXA1inPBS
chr11	33764247	33764248	MACS_peak_2465	22.00	lobChIP_FOXA1inPBS
chr11	33769770	33769771	MACS_peak_2466	9.00	lobChIP_FOXA1inPBS
chr11	34235699	34235700	MACS_peak_2467	11.00	lobChIP_FOXA1inPBS
chr11	34240434	34240435	MACS_peak_2468	13.00	lobChIP_FOXA1inPBS
chr11	34282830	34282831	MACS_peak_2469	12.00	lobChIP_FOXA1inPBS
chr11	34354258	34354259	MACS_peak_2470	11.00	lobChIP_FOXA1inPBS
chr11	34366999	34367000	MACS_peak_2471	8.00	lobChIP_FOXA1inPBS
chr11	34385053	34385054	MACS_peak_2472	18.00	lobChIP_FOXA1inPBS
chr11	34385968	34385969	MACS_peak_2473	8.00	lobChIP_FOXA1inPBS
chr11	34448906	34448907	MACS_peak_2474	17.00	lobChIP_FOXA1inPBS
chr11	34662086	34662087	MACS_peak_2475	20.00	lobChIP_FOXA1inPBS
chr11	34676273	34676274	MACS_peak_2476	11.00	lobChIP_FOXA1inPBS
chr11	34677687	34677688	MACS_peak_2477	9.00	lobChIP_FOXA1inPBS
chr11	34686814	34686815	MACS_peak_2478	55.00	lobChIP_FOXA1inPBS
chr11	34689887	34689888	MACS_peak_2479	44.00	lobChIP_FOXA1inPBS
chr11	34805709	34805710	MACS_peak_2480	15.00	lobChIP_FOXA1inPBS
chr11	34951544	34951545	MACS_peak_2481	26.00	lobChIP_FOXA1inPBS
chr11	35038640	35038641	MACS_peak_2482	6.00	lobChIP_FOXA1inPBS
chr11	35123005	35123006	MACS_peak_2483	9.00	lobChIP_FOXA1inPBS
chr11	35325661	35325662	MACS_peak_2484	12.00	lobChIP_FOXA1inPBS
chr11	35378934	35378935	MACS_peak_2485	8.00	lobChIP_FOXA1inPBS
chr11	35381045	35381046	MACS_peak_2486	10.00	lobChIP_FOXA1inPBS
chr11	35381831	35381832	MACS_peak_2487	25.00	lobChIP_FOXA1inPBS
chr11	35412698	35412699	MACS_peak_2488	18.00	lobChIP_FOXA1inPBS
chr11	35413662	35413663	MACS_peak_2489	11.00	lobChIP_FOXA1inPBS
chr11	35683856	35683857	MACS_peak_2490	27.00	lobChIP_FOXA1inPBS
chr11	38191011	38191012	MACS_peak_2491	12.00	lobChIP_FOXA1inPBS
chr11	40350866	40350867	MACS_peak_2492	10.00	lobChIP_FOXA1inPBS
chr11	41211877	41211878	MACS_peak_2493	14.00	lobChIP_FOXA1inPBS
chr11	42227255	42227256	MACS_peak_2494	8.00	lobChIP_FOXA1inPBS
chr11	43432114	43432115	MACS_peak_2495	10.00	lobChIP_FOXA1inPBS
chr11	45413141	45413142	MACS_peak_2496	12.00	lobChIP_FOXA1inPBS
chr11	45820867	45820868	MACS_peak_2497	7.00	lobChIP_FOXA1inPBS
chr11	46275901	46275902	MACS_peak_2498	9.00	lobChIP_FOXA1inPBS
chr11	46295582	46295583	MACS_peak_2499	51.00	lobChIP_FOXA1inPBS
chr11	46511816	46511817	MACS_peak_2500	6.00	lobChIP_FOXA1inPBS
chr11	46576000	46576001	MACS_peak_2501	50.00	lobChIP_FOXA1inPBS
chr11	46604293	46604294	MACS_peak_2502	7.00	lobChIP_FOXA1inPBS
chr11	46618887	46618888	MACS_peak_2503	9.00	lobChIP_FOXA1inPBS
chr11	46662496	46662497	MACS_peak_2504	12.00	lobChIP_FOXA1inPBS
chr11	46712393	46712394	MACS_peak_2505	10.00	lobChIP_FOXA1inPBS
chr11	46722163	46722164	MACS_peak_2506	12.00	lobChIP_FOXA1inPBS
chr11	46739895	46739896	MACS_peak_2507	22.00	lobChIP_FOXA1inPBS
chr11	46872673	46872674	MACS_peak_2508	8.00	lobChIP_FOXA1inPBS
chr11	47006718	47006719	MACS_peak_2509	21.00	lobChIP_FOXA1inPBS
chr11	47026464	47026465	MACS_peak_2510	11.00	lobChIP_FOXA1inPBS
chr11	47121281	47121282	MACS_peak_2511	16.00	lobChIP_FOXA1inPBS
chr11	47140710	47140711	MACS_peak_2512	7.00	lobChIP_FOXA1inPBS
chr11	47206454	47206455	MACS_peak_2513	13.00	lobChIP_FOXA1inPBS
chr11	47211014	47211015	MACS_peak_2514	26.00	lobChIP_FOXA1inPBS
chr11	47265119	47265120	MACS_peak_2515	18.00	lobChIP_FOXA1inPBS
chr11	47270790	47270791	MACS_peak_2516	13.00	lobChIP_FOXA1inPBS
chr11	47453049	47453050	MACS_peak_2517	7.00	lobChIP_FOXA1inPBS
chr11	47461838	47461839	MACS_peak_2518	7.00	lobChIP_FOXA1inPBS
chr11	47509736	47509737	MACS_peak_2519	8.00	lobChIP_FOXA1inPBS
chr11	47578760	47578761	MACS_peak_2520	11.00	lobChIP_FOXA1inPBS
chr11	47745769	47745770	MACS_peak_2521	12.00	lobChIP_FOXA1inPBS
chr11	47747758	47747759	MACS_peak_2522	9.00	lobChIP_FOXA1inPBS
chr11	56683962	56683963	MACS_peak_2523	6.00	lobChIP_FOXA1inPBS
chr11	57059267	57059268	MACS_peak_2524	8.00	lobChIP_FOXA1inPBS
chr11	57078292	57078293	MACS_peak_2525	13.00	lobChIP_FOXA1inPBS
chr11	57167592	57167593	MACS_peak_2526	27.00	lobChIP_FOXA1inPBS
chr11	57202269	57202270	MACS_peak_2527	16.00	lobChIP_FOXA1inPBS
chr11	57208696	57208697	MACS_peak_2528	9.00	lobChIP_FOXA1inPBS
chr11	57209527	57209528	MACS_peak_2529	21.00	lobChIP_FOXA1inPBS
chr11	57221250	57221251	MACS_peak_2530	39.00	lobChIP_FOXA1inPBS
chr11	57265452	57265453	MACS_peak_2531	13.00	lobChIP_FOXA1inPBS
chr11	57270531	57270532	MACS_peak_2532	21.00	lobChIP_FOXA1inPBS
chr11	57473139	57473140	MACS_peak_2533	10.00	lobChIP_FOXA1inPBS
chr11	57525848	57525849	MACS_peak_2534	7.00	lobChIP_FOXA1inPBS
chr11	57541495	57541496	MACS_peak_2535	8.00	lobChIP_FOXA1inPBS
chr11	57545669	57545670	MACS_peak_2536	11.00	lobChIP_FOXA1inPBS
chr11	57568223	57568224	MACS_peak_2537	29.00	lobChIP_FOXA1inPBS
chr11	57703620	57703621	MACS_peak_2538	7.00	lobChIP_FOXA1inPBS
chr11	57739491	57739492	MACS_peak_2539	27.00	lobChIP_FOXA1inPBS
chr11	57746354	57746355	MACS_peak_2540	18.00	lobChIP_FOXA1inPBS
chr11	57747247	57747248	MACS_peak_2541	6.00	lobChIP_FOXA1inPBS
chr11	57823596	57823597	MACS_peak_2542	12.00	lobChIP_FOXA1inPBS
chr11	57933416	57933417	MACS_peak_2543	8.00	lobChIP_FOXA1inPBS
chr11	58529950	58529951	MACS_peak_2544	35.00	lobChIP_FOXA1inPBS
chr11	58648267	58648268	MACS_peak_2545	34.00	lobChIP_FOXA1inPBS
chr11	58656237	58656238	MACS_peak_2546	16.00	lobChIP_FOXA1inPBS
chr11	58693938	58693939	MACS_peak_2547	37.00	lobChIP_FOXA1inPBS
chr11	58696352	58696353	MACS_peak_2548	13.00	lobChIP_FOXA1inPBS
chr11	58713347	58713348	MACS_peak_2549	12.00	lobChIP_FOXA1inPBS
chr11	58792757	58792758	MACS_peak_2550	8.00	lobChIP_FOXA1inPBS
chr11	59304728	59304729	MACS_peak_2551	8.00	lobChIP_FOXA1inPBS
chr11	59333954	59333955	MACS_peak_2552	8.00	lobChIP_FOXA1inPBS
chr11	59390889	59390890	MACS_peak_2553	8.00	lobChIP_FOXA1inPBS
chr11	59435458	59435459	MACS_peak_2554	7.00	lobChIP_FOXA1inPBS
chr11	59544088	59544089	MACS_peak_2555	13.00	lobChIP_FOXA1inPBS
chr11	59554635	59554636	MACS_peak_2556	6.00	lobChIP_FOXA1inPBS
chr11	59866278	59866279	MACS_peak_2557	9.00	lobChIP_FOXA1inPBS
chr11	60612444	60612445	MACS_peak_2558	26.00	lobChIP_FOXA1inPBS
chr11	60648597	60648598	MACS_peak_2559	21.00	lobChIP_FOXA1inPBS
chr11	60929283	60929284	MACS_peak_2560	6.00	lobChIP_FOXA1inPBS
chr11	60932594	60932595	MACS_peak_2561	19.00	lobChIP_FOXA1inPBS
chr11	61146336	61146337	MACS_peak_2562	14.00	lobChIP_FOXA1inPBS
chr11	61376994	61376995	MACS_peak_2563	26.00	lobChIP_FOXA1inPBS
chr11	61691258	61691259	MACS_peak_2564	9.00	lobChIP_FOXA1inPBS
chr11	61742780	61742781	MACS_peak_2565	7.00	lobChIP_FOXA1inPBS
chr11	61748326	61748327	MACS_peak_2566	10.00	lobChIP_FOXA1inPBS
chr11	61766637	61766638	MACS_peak_2567	8.00	lobChIP_FOXA1inPBS
chr11	61793201	61793202	MACS_peak_2568	9.00	lobChIP_FOXA1inPBS
chr11	61849714	61849715	MACS_peak_2569	9.00	lobChIP_FOXA1inPBS
chr11	61944489	61944490	MACS_peak_2570	17.00	lobChIP_FOXA1inPBS
chr11	62027977	62027978	MACS_peak_2571	26.00	lobChIP_FOXA1inPBS
chr11	62108283	62108284	MACS_peak_2572	8.00	lobChIP_FOXA1inPBS
chr11	62193137	62193138	MACS_peak_2573	6.00	lobChIP_FOXA1inPBS
chr11	62206208	62206209	MACS_peak_2574	8.00	lobChIP_FOXA1inPBS
chr11	62235458	62235459	MACS_peak_2575	19.00	lobChIP_FOXA1inPBS
chr11	62255381	62255382	MACS_peak_2576	6.00	lobChIP_FOXA1inPBS
chr11	62271825	62271826	MACS_peak_2577	15.00	lobChIP_FOXA1inPBS
chr11	62316536	62316537	MACS_peak_2578	15.00	lobChIP_FOXA1inPBS
chr11	62333157	62333158	MACS_peak_2579	8.00	lobChIP_FOXA1inPBS
chr11	62355400	62355401	MACS_peak_2580	12.00	lobChIP_FOXA1inPBS
chr11	62408389	62408390	MACS_peak_2581	37.00	lobChIP_FOXA1inPBS
chr11	62413551	62413552	MACS_peak_2582	5.00	lobChIP_FOXA1inPBS
chr11	62420992	62420993	MACS_peak_2583	18.00	lobChIP_FOXA1inPBS
chr11	62609086	62609087	MACS_peak_2584	22.00	lobChIP_FOXA1inPBS
chr11	63006959	63006960	MACS_peak_2585	27.00	lobChIP_FOXA1inPBS
chr11	63008355	63008356	MACS_peak_2586	24.00	lobChIP_FOXA1inPBS
chr11	63015562	63015563	MACS_peak_2587	21.00	lobChIP_FOXA1inPBS
chr11	63017779	63017780	MACS_peak_2588	27.00	lobChIP_FOXA1inPBS
chr11	63018956	63018957	MACS_peak_2589	17.00	lobChIP_FOXA1inPBS
chr11	63057320	63057321	MACS_peak_2590	7.00	lobChIP_FOXA1inPBS
chr11	63137159	63137160	MACS_peak_2591	9.00	lobChIP_FOXA1inPBS
chr11	63259296	63259297	MACS_peak_2592	26.00	lobChIP_FOXA1inPBS
chr11	63354146	63354147	MACS_peak_2593	13.00	lobChIP_FOXA1inPBS
chr11	63449717	63449718	MACS_peak_2594	8.00	lobChIP_FOXA1inPBS
chr11	63513160	63513161	MACS_peak_2595	13.00	lobChIP_FOXA1inPBS
chr11	63933544	63933545	MACS_peak_2596	10.00	lobChIP_FOXA1inPBS
chr11	64217311	64217312	MACS_peak_2597	7.00	lobChIP_FOXA1inPBS
chr11	64334156	64334157	MACS_peak_2598	10.00	lobChIP_FOXA1inPBS
chr11	64610765	64610766	MACS_peak_2599	12.00	lobChIP_FOXA1inPBS
chr11	64831968	64831969	MACS_peak_2600	6.00	lobChIP_FOXA1inPBS
chr11	64840254	64840255	MACS_peak_2601	10.00	lobChIP_FOXA1inPBS
chr11	64863621	64863622	MACS_peak_2602	8.00	lobChIP_FOXA1inPBS
chr11	64933056	64933057	MACS_peak_2603	8.00	lobChIP_FOXA1inPBS
chr11	64933550	64933551	MACS_peak_2604	10.00	lobChIP_FOXA1inPBS
chr11	64934442	64934443	MACS_peak_2605	7.00	lobChIP_FOXA1inPBS
chr11	65042863	65042864	MACS_peak_2606	7.00	lobChIP_FOXA1inPBS
chr11	65063604	65063605	MACS_peak_2607	10.00	lobChIP_FOXA1inPBS
chr11	65073608	65073609	MACS_peak_2608	40.00	lobChIP_FOXA1inPBS
chr11	65138859	65138860	MACS_peak_2609	14.00	lobChIP_FOXA1inPBS
chr11	65186304	65186305	MACS_peak_2610	7.00	lobChIP_FOXA1inPBS
chr11	65246790	65246791	MACS_peak_2611	6.00	lobChIP_FOXA1inPBS
chr11	65253183	65253184	MACS_peak_2612	8.00	lobChIP_FOXA1inPBS
chr11	65680163	65680164	MACS_peak_2613	11.00	lobChIP_FOXA1inPBS
chr11	65719626	65719627	MACS_peak_2614	16.00	lobChIP_FOXA1inPBS
chr11	65853487	65853488	MACS_peak_2615	7.00	lobChIP_FOXA1inPBS
chr11	65874887	65874888	MACS_peak_2616	7.00	lobChIP_FOXA1inPBS
chr11	65904538	65904539	MACS_peak_2617	13.00	lobChIP_FOXA1inPBS
chr11	65963421	65963422	MACS_peak_2618	15.00	lobChIP_FOXA1inPBS
chr11	66118856	66118857	MACS_peak_2619	42.00	lobChIP_FOXA1inPBS
chr11	66535580	66535581	MACS_peak_2620	12.00	lobChIP_FOXA1inPBS
chr11	66669613	66669614	MACS_peak_2621	27.00	lobChIP_FOXA1inPBS
chr11	66764511	66764512	MACS_peak_2622	11.00	lobChIP_FOXA1inPBS
chr11	66822670	66822671	MACS_peak_2623	8.00	lobChIP_FOXA1inPBS
chr11	67113579	67113580	MACS_peak_2624	11.00	lobChIP_FOXA1inPBS
chr11	67122009	67122010	MACS_peak_2625	7.00	lobChIP_FOXA1inPBS
chr11	67316217	67316218	MACS_peak_2626	8.00	lobChIP_FOXA1inPBS
chr11	67346705	67346706	MACS_peak_2627	28.00	lobChIP_FOXA1inPBS
chr11	67384854	67384855	MACS_peak_2628	7.00	lobChIP_FOXA1inPBS
chr11	67769644	67769645	MACS_peak_2629	20.00	lobChIP_FOXA1inPBS
chr11	67844082	67844083	MACS_peak_2630	7.00	lobChIP_FOXA1inPBS
chr11	67867021	67867022	MACS_peak_2631	12.00	lobChIP_FOXA1inPBS
chr11	67868941	67868942	MACS_peak_2632	7.00	lobChIP_FOXA1inPBS
chr11	67894426	67894427	MACS_peak_2633	11.00	lobChIP_FOXA1inPBS
chr11	67902691	67902692	MACS_peak_2634	12.00	lobChIP_FOXA1inPBS
chr11	67905423	67905424	MACS_peak_2635	15.00	lobChIP_FOXA1inPBS
chr11	67912630	67912631	MACS_peak_2636	6.00	lobChIP_FOXA1inPBS
chr11	67914698	67914699	MACS_peak_2637	36.00	lobChIP_FOXA1inPBS
chr11	68038730	68038731	MACS_peak_2638	20.00	lobChIP_FOXA1inPBS
chr11	68065802	68065803	MACS_peak_2639	18.00	lobChIP_FOXA1inPBS
chr11	68081549	68081550	MACS_peak_2640	13.00	lobChIP_FOXA1inPBS
chr11	68094389	68094390	MACS_peak_2641	6.00	lobChIP_FOXA1inPBS
chr11	68099261	68099262	MACS_peak_2642	12.00	lobChIP_FOXA1inPBS
chr11	68147995	68147996	MACS_peak_2643	27.00	lobChIP_FOXA1inPBS
chr11	68174581	68174582	MACS_peak_2644	15.00	lobChIP_FOXA1inPBS
chr11	68235829	68235830	MACS_peak_2645	15.00	lobChIP_FOXA1inPBS
chr11	68236765	68236766	MACS_peak_2646	7.00	lobChIP_FOXA1inPBS
chr11	68300702	68300703	MACS_peak_2647	10.00	lobChIP_FOXA1inPBS
chr11	68400477	68400478	MACS_peak_2648	9.00	lobChIP_FOXA1inPBS
chr11	68613074	68613075	MACS_peak_2649	25.00	lobChIP_FOXA1inPBS
chr11	68625255	68625256	MACS_peak_2650	42.00	lobChIP_FOXA1inPBS
chr11	68717313	68717314	MACS_peak_2651	5.00	lobChIP_FOXA1inPBS
chr11	69312252	69312253	MACS_peak_2652	9.00	lobChIP_FOXA1inPBS
chr11	69383186	69383187	MACS_peak_2653	13.00	lobChIP_FOXA1inPBS
chr11	69469773	69469774	MACS_peak_2654	32.00	lobChIP_FOXA1inPBS
chr11	69503391	69503392	MACS_peak_2655	9.00	lobChIP_FOXA1inPBS
chr11	69816630	69816631	MACS_peak_2656	62.00	lobChIP_FOXA1inPBS
chr11	70058535	70058536	MACS_peak_2657	9.00	lobChIP_FOXA1inPBS
chr11	70147160	70147161	MACS_peak_2658	11.00	lobChIP_FOXA1inPBS
chr11	70545621	70545622	MACS_peak_2659	9.00	lobChIP_FOXA1inPBS
chr11	70864718	70864719	MACS_peak_2660	12.00	lobChIP_FOXA1inPBS
chr11	70964719	70964720	MACS_peak_2661	35.00	lobChIP_FOXA1inPBS
chr11	71018122	71018123	MACS_peak_2662	21.00	lobChIP_FOXA1inPBS
chr11	71140255	71140256	MACS_peak_2663	11.00	lobChIP_FOXA1inPBS
chr11	71237320	71237321	MACS_peak_2664	8.00	lobChIP_FOXA1inPBS
chr11	71659614	71659615	MACS_peak_2665	10.00	lobChIP_FOXA1inPBS
chr11	71751886	71751887	MACS_peak_2666	11.00	lobChIP_FOXA1inPBS
chr11	71823306	71823307	MACS_peak_2667	8.00	lobChIP_FOXA1inPBS
chr11	72109224	72109225	MACS_peak_2668	23.00	lobChIP_FOXA1inPBS
chr11	72129233	72129234	MACS_peak_2669	10.00	lobChIP_FOXA1inPBS
chr11	72133734	72133735	MACS_peak_2670	7.00	lobChIP_FOXA1inPBS
chr11	72142257	72142258	MACS_peak_2671	12.00	lobChIP_FOXA1inPBS
chr11	72477563	72477564	MACS_peak_2672	27.00	lobChIP_FOXA1inPBS
chr11	72490154	72490155	MACS_peak_2673	19.00	lobChIP_FOXA1inPBS
chr11	72497545	72497546	MACS_peak_2674	19.00	lobChIP_FOXA1inPBS
chr11	72504826	72504827	MACS_peak_2675	7.00	lobChIP_FOXA1inPBS
chr11	72518577	72518578	MACS_peak_2676	9.00	lobChIP_FOXA1inPBS
chr11	72770262	72770263	MACS_peak_2677	31.00	lobChIP_FOXA1inPBS
chr11	72784033	72784034	MACS_peak_2678	12.00	lobChIP_FOXA1inPBS
chr11	72854922	72854923	MACS_peak_2679	10.00	lobChIP_FOXA1inPBS
chr11	72886867	72886868	MACS_peak_2680	11.00	lobChIP_FOXA1inPBS
chr11	72901188	72901189	MACS_peak_2681	13.00	lobChIP_FOXA1inPBS
chr11	72983188	72983189	MACS_peak_2682	16.00	lobChIP_FOXA1inPBS
chr11	73340183	73340184	MACS_peak_2683	27.00	lobChIP_FOXA1inPBS
chr11	73359955	73359956	MACS_peak_2684	12.00	lobChIP_FOXA1inPBS
chr11	73664495	73664496	MACS_peak_2685	10.00	lobChIP_FOXA1inPBS
chr11	73698038	73698039	MACS_peak_2686	8.00	lobChIP_FOXA1inPBS
chr11	73729592	73729593	MACS_peak_2687	16.00	lobChIP_FOXA1inPBS
chr11	73849694	73849695	MACS_peak_2688	9.00	lobChIP_FOXA1inPBS
chr11	74304674	74304675	MACS_peak_2689	20.00	lobChIP_FOXA1inPBS
chr11	74327206	74327207	MACS_peak_2690	19.00	lobChIP_FOXA1inPBS
chr11	74445078	74445079	MACS_peak_2691	23.00	lobChIP_FOXA1inPBS
chr11	74451115	74451116	MACS_peak_2692	11.00	lobChIP_FOXA1inPBS
chr11	74561116	74561117	MACS_peak_2693	11.00	lobChIP_FOXA1inPBS
chr11	74661229	74661230	MACS_peak_2694	7.00	lobChIP_FOXA1inPBS
chr11	74695294	74695295	MACS_peak_2695	10.00	lobChIP_FOXA1inPBS
chr11	74724387	74724388	MACS_peak_2696	9.00	lobChIP_FOXA1inPBS
chr11	74867250	74867251	MACS_peak_2697	11.00	lobChIP_FOXA1inPBS
chr11	74915100	74915101	MACS_peak_2698	10.00	lobChIP_FOXA1inPBS
chr11	75034923	75034924	MACS_peak_2699	34.00	lobChIP_FOXA1inPBS
chr11	75054174	75054175	MACS_peak_2700	11.00	lobChIP_FOXA1inPBS
chr11	75083500	75083501	MACS_peak_2701	13.00	lobChIP_FOXA1inPBS
chr11	75133455	75133456	MACS_peak_2702	17.00	lobChIP_FOXA1inPBS
chr11	75228953	75228954	MACS_peak_2703	11.00	lobChIP_FOXA1inPBS
chr11	75429944	75429945	MACS_peak_2704	9.00	lobChIP_FOXA1inPBS
chr11	75474101	75474102	MACS_peak_2705	15.00	lobChIP_FOXA1inPBS
chr11	75494167	75494168	MACS_peak_2706	9.00	lobChIP_FOXA1inPBS
chr11	75495264	75495265	MACS_peak_2707	24.00	lobChIP_FOXA1inPBS
chr11	75523664	75523665	MACS_peak_2708	9.00	lobChIP_FOXA1inPBS
chr11	75812525	75812526	MACS_peak_2709	6.00	lobChIP_FOXA1inPBS
chr11	75860301	75860302	MACS_peak_2710	7.00	lobChIP_FOXA1inPBS
chr11	76040591	76040592	MACS_peak_2711	8.00	lobChIP_FOXA1inPBS
chr11	76235255	76235256	MACS_peak_2712	17.00	lobChIP_FOXA1inPBS
chr11	76238076	76238077	MACS_peak_2713	22.00	lobChIP_FOXA1inPBS
chr11	76483622	76483623	MACS_peak_2714	18.00	lobChIP_FOXA1inPBS
chr11	76504621	76504622	MACS_peak_2715	13.00	lobChIP_FOXA1inPBS
chr11	76513019	76513020	MACS_peak_2716	31.00	lobChIP_FOXA1inPBS
chr11	76600081	76600082	MACS_peak_2717	9.00	lobChIP_FOXA1inPBS
chr11	76627966	76627967	MACS_peak_2718	37.00	lobChIP_FOXA1inPBS
chr11	76650332	76650333	MACS_peak_2719	17.00	lobChIP_FOXA1inPBS
chr11	76674223	76674224	MACS_peak_2720	18.00	lobChIP_FOXA1inPBS
chr11	76731449	76731450	MACS_peak_2721	14.00	lobChIP_FOXA1inPBS
chr11	76747629	76747630	MACS_peak_2722	38.00	lobChIP_FOXA1inPBS
chr11	76772375	76772376	MACS_peak_2723	44.00	lobChIP_FOXA1inPBS
chr11	76842477	76842478	MACS_peak_2724	6.00	lobChIP_FOXA1inPBS
chr11	76884639	76884640	MACS_peak_2725	9.00	lobChIP_FOXA1inPBS
chr11	77305160	77305161	MACS_peak_2726	7.00	lobChIP_FOXA1inPBS
chr11	77342278	77342279	MACS_peak_2727	8.00	lobChIP_FOXA1inPBS
chr11	77491099	77491100	MACS_peak_2728	11.00	lobChIP_FOXA1inPBS
chr11	77747568	77747569	MACS_peak_2729	19.00	lobChIP_FOXA1inPBS
chr11	77801804	77801805	MACS_peak_2730	21.00	lobChIP_FOXA1inPBS
chr11	77867722	77867723	MACS_peak_2731	12.00	lobChIP_FOXA1inPBS
chr11	77882144	77882145	MACS_peak_2732	8.00	lobChIP_FOXA1inPBS
chr11	77992704	77992705	MACS_peak_2733	7.00	lobChIP_FOXA1inPBS
chr11	78001477	78001478	MACS_peak_2734	15.00	lobChIP_FOXA1inPBS
chr11	78251503	78251504	MACS_peak_2735	11.00	lobChIP_FOXA1inPBS
chr11	78272405	78272406	MACS_peak_2736	27.00	lobChIP_FOXA1inPBS
chr11	78312280	78312281	MACS_peak_2737	36.00	lobChIP_FOXA1inPBS
chr11	80306314	80306315	MACS_peak_2738	6.00	lobChIP_FOXA1inPBS
chr11	82529602	82529603	MACS_peak_2739	13.00	lobChIP_FOXA1inPBS
chr11	82700142	82700143	MACS_peak_2740	21.00	lobChIP_FOXA1inPBS
chr11	82719154	82719155	MACS_peak_2741	8.00	lobChIP_FOXA1inPBS
chr11	82804188	82804189	MACS_peak_2742	15.00	lobChIP_FOXA1inPBS
chr11	82872841	82872842	MACS_peak_2743	9.00	lobChIP_FOXA1inPBS
chr11	82995656	82995657	MACS_peak_2744	14.00	lobChIP_FOXA1inPBS
chr11	83359836	83359837	MACS_peak_2745	25.00	lobChIP_FOXA1inPBS
chr11	83422231	83422232	MACS_peak_2746	11.00	lobChIP_FOXA1inPBS
chr11	83564129	83564130	MACS_peak_2747	9.00	lobChIP_FOXA1inPBS
chr11	83962064	83962065	MACS_peak_2748	27.00	lobChIP_FOXA1inPBS
chr11	84051021	84051022	MACS_peak_2749	17.00	lobChIP_FOXA1inPBS
chr11	84899240	84899241	MACS_peak_2750	11.00	lobChIP_FOXA1inPBS
chr11	85346009	85346010	MACS_peak_2752	8.00	lobChIP_FOXA1inPBS
chr11	85630684	85630685	MACS_peak_2753	11.00	lobChIP_FOXA1inPBS
chr11	85634703	85634704	MACS_peak_2754	12.00	lobChIP_FOXA1inPBS
chr11	85642189	85642190	MACS_peak_2755	23.00	lobChIP_FOXA1inPBS
chr11	85681915	85681916	MACS_peak_2756	16.00	lobChIP_FOXA1inPBS
chr11	85835458	85835459	MACS_peak_2757	14.00	lobChIP_FOXA1inPBS
chr11	85837987	85837988	MACS_peak_2758	8.00	lobChIP_FOXA1inPBS
chr11	85856798	85856799	MACS_peak_2759	9.00	lobChIP_FOXA1inPBS
chr11	85871679	85871680	MACS_peak_2760	8.00	lobChIP_FOXA1inPBS
chr11	85917064	85917065	MACS_peak_2761	33.00	lobChIP_FOXA1inPBS
chr11	85921122	85921123	MACS_peak_2762	63.00	lobChIP_FOXA1inPBS
chr11	86187731	86187732	MACS_peak_2763	9.00	lobChIP_FOXA1inPBS
chr11	86235332	86235333	MACS_peak_2764	16.00	lobChIP_FOXA1inPBS
chr11	86417585	86417586	MACS_peak_2765	62.00	lobChIP_FOXA1inPBS
chr11	86453092	86453093	MACS_peak_2766	17.00	lobChIP_FOXA1inPBS
chr11	86651340	86651341	MACS_peak_2767	13.00	lobChIP_FOXA1inPBS
chr11	86669506	86669507	MACS_peak_2768	14.00	lobChIP_FOXA1inPBS
chr11	86710635	86710636	MACS_peak_2769	12.00	lobChIP_FOXA1inPBS
chr11	86716489	86716490	MACS_peak_2770	104.00	lobChIP_FOXA1inPBS
chr11	86723322	86723323	MACS_peak_2771	15.00	lobChIP_FOXA1inPBS
chr11	86740586	86740587	MACS_peak_2772	17.00	lobChIP_FOXA1inPBS
chr11	86755176	86755177	MACS_peak_2773	11.00	lobChIP_FOXA1inPBS
chr11	86788073	86788074	MACS_peak_2774	16.00	lobChIP_FOXA1inPBS
chr11	86832253	86832254	MACS_peak_2775	7.00	lobChIP_FOXA1inPBS
chr11	86839708	86839709	MACS_peak_2776	16.00	lobChIP_FOXA1inPBS
chr11	86944776	86944777	MACS_peak_2777	10.00	lobChIP_FOXA1inPBS
chr11	86991277	86991278	MACS_peak_2778	11.00	lobChIP_FOXA1inPBS
chr11	89358598	89358599	MACS_peak_2779	12.00	lobChIP_FOXA1inPBS
chr11	90470978	90470979	MACS_peak_2780	17.00	lobChIP_FOXA1inPBS
chr11	90930368	90930369	MACS_peak_2781	8.00	lobChIP_FOXA1inPBS
chr11	91128324	91128325	MACS_peak_2782	9.00	lobChIP_FOXA1inPBS
chr11	91874393	91874394	MACS_peak_2783	12.00	lobChIP_FOXA1inPBS
chr11	93220494	93220495	MACS_peak_2784	8.00	lobChIP_FOXA1inPBS
chr11	93226608	93226609	MACS_peak_2785	14.00	lobChIP_FOXA1inPBS
chr11	93259471	93259472	MACS_peak_2786	11.00	lobChIP_FOXA1inPBS
chr11	93303981	93303982	MACS_peak_2787	9.00	lobChIP_FOXA1inPBS
chr11	93324372	93324373	MACS_peak_2788	6.00	lobChIP_FOXA1inPBS
chr11	93356663	93356664	MACS_peak_2789	13.00	lobChIP_FOXA1inPBS
chr11	93358158	93358159	MACS_peak_2790	7.00	lobChIP_FOXA1inPBS
chr11	93478664	93478665	MACS_peak_2791	10.00	lobChIP_FOXA1inPBS
chr11	93878865	93878866	MACS_peak_2792	12.00	lobChIP_FOXA1inPBS
chr11	93886563	93886564	MACS_peak_2793	9.00	lobChIP_FOXA1inPBS
chr11	93934381	93934382	MACS_peak_2794	6.00	lobChIP_FOXA1inPBS
chr11	94579030	94579031	MACS_peak_2795	12.00	lobChIP_FOXA1inPBS
chr11	94730799	94730800	MACS_peak_2796	9.00	lobChIP_FOXA1inPBS
chr11	95097985	95097986	MACS_peak_2797	26.00	lobChIP_FOXA1inPBS
chr11	95221223	95221224	MACS_peak_2798	9.00	lobChIP_FOXA1inPBS
chr11	95637330	95637331	MACS_peak_2799	12.00	lobChIP_FOXA1inPBS
chr11	95747533	95747534	MACS_peak_2800	16.00	lobChIP_FOXA1inPBS
chr11	95815219	95815220	MACS_peak_2801	10.00	lobChIP_FOXA1inPBS
chr11	95922094	95922095	MACS_peak_2802	9.00	lobChIP_FOXA1inPBS
chr11	95941017	95941018	MACS_peak_2803	11.00	lobChIP_FOXA1inPBS
chr11	95953645	95953646	MACS_peak_2804	11.00	lobChIP_FOXA1inPBS
chr11	95970974	95970975	MACS_peak_2805	12.00	lobChIP_FOXA1inPBS
chr11	95974780	95974781	MACS_peak_2806	11.00	lobChIP_FOXA1inPBS
chr11	95979920	95979921	MACS_peak_2807	7.00	lobChIP_FOXA1inPBS
chr11	96065776	96065777	MACS_peak_2808	9.00	lobChIP_FOXA1inPBS
chr11	96070651	96070652	MACS_peak_2809	8.00	lobChIP_FOXA1inPBS
chr11	96198076	96198077	MACS_peak_2810	7.00	lobChIP_FOXA1inPBS
chr11	96220664	96220665	MACS_peak_2811	14.00	lobChIP_FOXA1inPBS
chr11	99808272	99808273	MACS_peak_2812	8.00	lobChIP_FOXA1inPBS
chr11	100699179	100699180	MACS_peak_2813	11.00	lobChIP_FOXA1inPBS
chr11	101335281	101335282	MACS_peak_2814	8.00	lobChIP_FOXA1inPBS
chr11	101783722	101783723	MACS_peak_2815	8.00	lobChIP_FOXA1inPBS
chr11	101865117	101865118	MACS_peak_2816	49.00	lobChIP_FOXA1inPBS
chr11	101877771	101877772	MACS_peak_2817	17.00	lobChIP_FOXA1inPBS
chr11	101956744	101956745	MACS_peak_2818	15.00	lobChIP_FOXA1inPBS
chr11	101982813	101982814	MACS_peak_2819	14.00	lobChIP_FOXA1inPBS
chr11	101987387	101987388	MACS_peak_2820	12.00	lobChIP_FOXA1inPBS
chr11	101988458	101988459	MACS_peak_2821	5.00	lobChIP_FOXA1inPBS
chr11	101990994	101990995	MACS_peak_2822	37.00	lobChIP_FOXA1inPBS
chr11	102057945	102057946	MACS_peak_2823	6.00	lobChIP_FOXA1inPBS
chr11	102058589	102058590	MACS_peak_2824	17.00	lobChIP_FOXA1inPBS
chr11	102061024	102061025	MACS_peak_2825	17.00	lobChIP_FOXA1inPBS
chr11	102063625	102063626	MACS_peak_2826	17.00	lobChIP_FOXA1inPBS
chr11	102113066	102113067	MACS_peak_2827	13.00	lobChIP_FOXA1inPBS
chr11	102143447	102143448	MACS_peak_2828	6.00	lobChIP_FOXA1inPBS
chr11	102150403	102150404	MACS_peak_2829	17.00	lobChIP_FOXA1inPBS
chr11	102187826	102187827	MACS_peak_2830	7.00	lobChIP_FOXA1inPBS
chr11	102211181	102211182	MACS_peak_2831	9.00	lobChIP_FOXA1inPBS
chr11	102219981	102219982	MACS_peak_2832	12.00	lobChIP_FOXA1inPBS
chr11	102322394	102322395	MACS_peak_2833	20.00	lobChIP_FOXA1inPBS
chr11	102333539	102333540	MACS_peak_2834	15.00	lobChIP_FOXA1inPBS
chr11	102355800	102355801	MACS_peak_2835	8.00	lobChIP_FOXA1inPBS
chr11	102389710	102389711	MACS_peak_2836	14.00	lobChIP_FOXA1inPBS
chr11	102470811	102470812	MACS_peak_2837	15.00	lobChIP_FOXA1inPBS
chr11	102690450	102690451	MACS_peak_2838	8.00	lobChIP_FOXA1inPBS
chr11	102941882	102941883	MACS_peak_2839	8.00	lobChIP_FOXA1inPBS
chr11	103904017	103904018	MACS_peak_2840	17.00	lobChIP_FOXA1inPBS
chr11	104222968	104222969	MACS_peak_2841	11.00	lobChIP_FOXA1inPBS
chr11	104827530	104827531	MACS_peak_2842	12.00	lobChIP_FOXA1inPBS
chr11	104865972	104865973	MACS_peak_2843	12.00	lobChIP_FOXA1inPBS
chr11	104918183	104918184	MACS_peak_2844	20.00	lobChIP_FOXA1inPBS
chr11	104922577	104922578	MACS_peak_2845	8.00	lobChIP_FOXA1inPBS
chr11	105069831	105069832	MACS_peak_2846	7.00	lobChIP_FOXA1inPBS
chr11	105073698	105073699	MACS_peak_2847	12.00	lobChIP_FOXA1inPBS
chr11	105077549	105077550	MACS_peak_2848	9.00	lobChIP_FOXA1inPBS
chr11	105969829	105969830	MACS_peak_2849	13.00	lobChIP_FOXA1inPBS
chr11	106320593	106320594	MACS_peak_2850	9.00	lobChIP_FOXA1inPBS
chr11	107256491	107256492	MACS_peak_2851	7.00	lobChIP_FOXA1inPBS
chr11	107279651	107279652	MACS_peak_2852	30.00	lobChIP_FOXA1inPBS
chr11	107560465	107560466	MACS_peak_2853	10.00	lobChIP_FOXA1inPBS
chr11	107730926	107730927	MACS_peak_2854	7.00	lobChIP_FOXA1inPBS
chr11	108464687	108464688	MACS_peak_2855	17.00	lobChIP_FOXA1inPBS
chr11	108535471	108535472	MACS_peak_2856	8.00	lobChIP_FOXA1inPBS
chr11	108980044	108980045	MACS_peak_2857	13.00	lobChIP_FOXA1inPBS
chr11	110161563	110161564	MACS_peak_2858	10.00	lobChIP_FOXA1inPBS
chr11	110169219	110169220	MACS_peak_2859	17.00	lobChIP_FOXA1inPBS
chr11	110174246	110174247	MACS_peak_2860	10.00	lobChIP_FOXA1inPBS
chr11	110176622	110176623	MACS_peak_2861	11.00	lobChIP_FOXA1inPBS
chr11	110234097	110234098	MACS_peak_2862	9.00	lobChIP_FOXA1inPBS
chr11	110247969	110247970	MACS_peak_2863	7.00	lobChIP_FOXA1inPBS
chr11	111180387	111180388	MACS_peak_2864	21.00	lobChIP_FOXA1inPBS
chr11	111193935	111193936	MACS_peak_2865	7.00	lobChIP_FOXA1inPBS
chr11	111205578	111205579	MACS_peak_2866	37.00	lobChIP_FOXA1inPBS
chr11	111222431	111222432	MACS_peak_2867	10.00	lobChIP_FOXA1inPBS
chr11	111231071	111231072	MACS_peak_2868	17.00	lobChIP_FOXA1inPBS
chr11	111236678	111236679	MACS_peak_2869	5.00	lobChIP_FOXA1inPBS
chr11	111479763	111479764	MACS_peak_2870	6.00	lobChIP_FOXA1inPBS
chr11	111504203	111504204	MACS_peak_2871	36.00	lobChIP_FOXA1inPBS
chr11	111597285	111597286	MACS_peak_2872	14.00	lobChIP_FOXA1inPBS
chr11	111627459	111627460	MACS_peak_2873	15.00	lobChIP_FOXA1inPBS
chr11	111661149	111661150	MACS_peak_2874	19.00	lobChIP_FOXA1inPBS
chr11	111666506	111666507	MACS_peak_2875	6.00	lobChIP_FOXA1inPBS
chr11	111671414	111671415	MACS_peak_2876	42.00	lobChIP_FOXA1inPBS
chr11	111711188	111711189	MACS_peak_2877	18.00	lobChIP_FOXA1inPBS
chr11	111806207	111806208	MACS_peak_2878	14.00	lobChIP_FOXA1inPBS
chr11	111854182	111854183	MACS_peak_2879	7.00	lobChIP_FOXA1inPBS
chr11	111859722	111859723	MACS_peak_2880	42.00	lobChIP_FOXA1inPBS
chr11	111888435	111888436	MACS_peak_2881	11.00	lobChIP_FOXA1inPBS
chr11	111920520	111920521	MACS_peak_2882	6.00	lobChIP_FOXA1inPBS
chr11	112028202	112028203	MACS_peak_2883	10.00	lobChIP_FOXA1inPBS
chr11	112034074	112034075	MACS_peak_2884	9.00	lobChIP_FOXA1inPBS
chr11	112087341	112087342	MACS_peak_2885	7.00	lobChIP_FOXA1inPBS
chr11	112112278	112112279	MACS_peak_2886	6.00	lobChIP_FOXA1inPBS
chr11	113066662	113066663	MACS_peak_2887	10.00	lobChIP_FOXA1inPBS
chr11	113644879	113644880	MACS_peak_2888	6.00	lobChIP_FOXA1inPBS
chr11	113777436	113777437	MACS_peak_2889	9.00	lobChIP_FOXA1inPBS
chr11	113953145	113953146	MACS_peak_2890	10.00	lobChIP_FOXA1inPBS
chr11	114186840	114186841	MACS_peak_2891	47.00	lobChIP_FOXA1inPBS
chr11	115118885	115118886	MACS_peak_2892	10.00	lobChIP_FOXA1inPBS
chr11	115120091	115120092	MACS_peak_2893	22.00	lobChIP_FOXA1inPBS
chr11	115151050	115151051	MACS_peak_2894	9.00	lobChIP_FOXA1inPBS
chr11	115248704	115248705	MACS_peak_2895	10.00	lobChIP_FOXA1inPBS
chr11	115280600	115280601	MACS_peak_2896	41.00	lobChIP_FOXA1inPBS
chr11	115323293	115323294	MACS_peak_2897	12.00	lobChIP_FOXA1inPBS
chr11	115364846	115364847	MACS_peak_2898	12.00	lobChIP_FOXA1inPBS
chr11	115373570	115373571	MACS_peak_2899	8.00	lobChIP_FOXA1inPBS
chr11	115394353	115394354	MACS_peak_2900	8.00	lobChIP_FOXA1inPBS
chr11	115419774	115419775	MACS_peak_2901	11.00	lobChIP_FOXA1inPBS
chr11	115834958	115834959	MACS_peak_2902	23.00	lobChIP_FOXA1inPBS
chr11	115838071	115838072	MACS_peak_2903	9.00	lobChIP_FOXA1inPBS
chr11	116165698	116165699	MACS_peak_2904	16.00	lobChIP_FOXA1inPBS
chr11	116210243	116210244	MACS_peak_2905	15.00	lobChIP_FOXA1inPBS
chr11	116247424	116247425	MACS_peak_2906	19.00	lobChIP_FOXA1inPBS
chr11	116283406	116283407	MACS_peak_2907	25.00	lobChIP_FOXA1inPBS
chr11	116434274	116434275	MACS_peak_2908	13.00	lobChIP_FOXA1inPBS
chr11	116538267	116538268	MACS_peak_2909	76.00	lobChIP_FOXA1inPBS
chr11	116663206	116663207	MACS_peak_2910	12.00	lobChIP_FOXA1inPBS
chr11	116679151	116679152	MACS_peak_2911	59.00	lobChIP_FOXA1inPBS
chr11	116703179	116703180	MACS_peak_2912	39.00	lobChIP_FOXA1inPBS
chr11	116708480	116708481	MACS_peak_2913	29.00	lobChIP_FOXA1inPBS
chr11	116892031	116892032	MACS_peak_2914	10.00	lobChIP_FOXA1inPBS
chr11	116916951	116916952	MACS_peak_2915	13.00	lobChIP_FOXA1inPBS
chr11	116943372	116943373	MACS_peak_2916	9.00	lobChIP_FOXA1inPBS
chr11	116948257	116948258	MACS_peak_2917	87.00	lobChIP_FOXA1inPBS
chr11	116964108	116964109	MACS_peak_2918	15.00	lobChIP_FOXA1inPBS
chr11	116966736	116966737	MACS_peak_2919	31.00	lobChIP_FOXA1inPBS
chr11	117170717	117170718	MACS_peak_2920	5.00	lobChIP_FOXA1inPBS
chr11	117574142	117574143	MACS_peak_2921	11.00	lobChIP_FOXA1inPBS
chr11	117588692	117588693	MACS_peak_2922	27.00	lobChIP_FOXA1inPBS
chr11	117620112	117620113	MACS_peak_2923	19.00	lobChIP_FOXA1inPBS
chr11	118073593	118073594	MACS_peak_2924	8.00	lobChIP_FOXA1inPBS
chr11	118082968	118082969	MACS_peak_2925	11.00	lobChIP_FOXA1inPBS
chr11	118185905	118185906	MACS_peak_2926	35.00	lobChIP_FOXA1inPBS
chr11	118544648	118544649	MACS_peak_2927	14.00	lobChIP_FOXA1inPBS
chr11	118787728	118787729	MACS_peak_2928	11.00	lobChIP_FOXA1inPBS
chr11	118799287	118799288	MACS_peak_2929	22.00	lobChIP_FOXA1inPBS
chr11	118971095	118971096	MACS_peak_2930	8.00	lobChIP_FOXA1inPBS
chr11	118979675	118979676	MACS_peak_2931	14.00	lobChIP_FOXA1inPBS
chr11	119067661	119067662	MACS_peak_2932	8.00	lobChIP_FOXA1inPBS
chr11	119205167	119205168	MACS_peak_2933	11.00	lobChIP_FOXA1inPBS
chr11	119226029	119226030	MACS_peak_2934	13.00	lobChIP_FOXA1inPBS
chr11	119232099	119232100	MACS_peak_2935	8.00	lobChIP_FOXA1inPBS
chr11	119343117	119343118	MACS_peak_2936	8.00	lobChIP_FOXA1inPBS
chr11	119352142	119352143	MACS_peak_2937	18.00	lobChIP_FOXA1inPBS
chr11	119534842	119534843	MACS_peak_2938	6.00	lobChIP_FOXA1inPBS
chr11	120252242	120252243	MACS_peak_2939	10.00	lobChIP_FOXA1inPBS
chr11	120259157	120259158	MACS_peak_2940	27.00	lobChIP_FOXA1inPBS
chr11	120270691	120270692	MACS_peak_2941	13.00	lobChIP_FOXA1inPBS
chr11	120909571	120909572	MACS_peak_2942	32.00	lobChIP_FOXA1inPBS
chr11	120938301	120938302	MACS_peak_2943	30.00	lobChIP_FOXA1inPBS
chr11	121330478	121330479	MACS_peak_2944	20.00	lobChIP_FOXA1inPBS
chr11	121347237	121347238	MACS_peak_2945	21.00	lobChIP_FOXA1inPBS
chr11	121435913	121435914	MACS_peak_2946	9.00	lobChIP_FOXA1inPBS
chr11	121527358	121527359	MACS_peak_2947	7.00	lobChIP_FOXA1inPBS
chr11	121799495	121799496	MACS_peak_2948	9.00	lobChIP_FOXA1inPBS
chr11	121802991	121802992	MACS_peak_2949	11.00	lobChIP_FOXA1inPBS
chr11	122010384	122010385	MACS_peak_2950	12.00	lobChIP_FOXA1inPBS
chr11	122021568	122021569	MACS_peak_2951	15.00	lobChIP_FOXA1inPBS
chr11	122513234	122513235	MACS_peak_2952	10.00	lobChIP_FOXA1inPBS
chr11	122577077	122577078	MACS_peak_2953	9.00	lobChIP_FOXA1inPBS
chr11	122595175	122595176	MACS_peak_2954	7.00	lobChIP_FOXA1inPBS
chr11	123459452	123459453	MACS_peak_2955	11.00	lobChIP_FOXA1inPBS
chr11	124681629	124681630	MACS_peak_2956	7.00	lobChIP_FOXA1inPBS
chr11	124693615	124693616	MACS_peak_2957	8.00	lobChIP_FOXA1inPBS
chr11	124752557	124752558	MACS_peak_2958	7.00	lobChIP_FOXA1inPBS
chr11	124804940	124804941	MACS_peak_2959	14.00	lobChIP_FOXA1inPBS
chr11	124890425	124890426	MACS_peak_2960	9.00	lobChIP_FOXA1inPBS
chr11	125434496	125434497	MACS_peak_2961	9.00	lobChIP_FOXA1inPBS
chr11	125838504	125838505	MACS_peak_2962	14.00	lobChIP_FOXA1inPBS
chr11	125857417	125857418	MACS_peak_2963	14.00	lobChIP_FOXA1inPBS
chr11	125959260	125959261	MACS_peak_2964	10.00	lobChIP_FOXA1inPBS
chr11	125986015	125986016	MACS_peak_2965	15.00	lobChIP_FOXA1inPBS
chr11	126017819	126017820	MACS_peak_2966	23.00	lobChIP_FOXA1inPBS
chr11	126028555	126028556	MACS_peak_2967	10.00	lobChIP_FOXA1inPBS
chr11	126033553	126033554	MACS_peak_2968	14.00	lobChIP_FOXA1inPBS
chr11	126084770	126084771	MACS_peak_2969	7.00	lobChIP_FOXA1inPBS
chr11	128321723	128321724	MACS_peak_2970	7.00	lobChIP_FOXA1inPBS
chr11	128985690	128985691	MACS_peak_2971	39.00	lobChIP_FOXA1inPBS
chr11	129072665	129072666	MACS_peak_2972	13.00	lobChIP_FOXA1inPBS
chr11	129118724	129118725	MACS_peak_2973	17.00	lobChIP_FOXA1inPBS
chr11	129194640	129194641	MACS_peak_2974	46.00	lobChIP_FOXA1inPBS
chr11	129330834	129330835	MACS_peak_2975	9.00	lobChIP_FOXA1inPBS
chr11	129661671	129661672	MACS_peak_2976	16.00	lobChIP_FOXA1inPBS
chr11	129670280	129670281	MACS_peak_2977	14.00	lobChIP_FOXA1inPBS
chr11	129698564	129698565	MACS_peak_2978	16.00	lobChIP_FOXA1inPBS
chr11	129723906	129723907	MACS_peak_2979	33.00	lobChIP_FOXA1inPBS
chr11	129731908	129731909	MACS_peak_2980	6.00	lobChIP_FOXA1inPBS
chr11	129869488	129869489	MACS_peak_2981	25.00	lobChIP_FOXA1inPBS
chr11	129912064	129912065	MACS_peak_2982	9.00	lobChIP_FOXA1inPBS
chr11	129959468	129959469	MACS_peak_2983	9.00	lobChIP_FOXA1inPBS
chr11	129985263	129985264	MACS_peak_2984	8.00	lobChIP_FOXA1inPBS
chr11	130014119	130014120	MACS_peak_2985	22.00	lobChIP_FOXA1inPBS
chr11	130051351	130051352	MACS_peak_2986	12.00	lobChIP_FOXA1inPBS
chr11	130305003	130305004	MACS_peak_2987	22.00	lobChIP_FOXA1inPBS
chr11	130507050	130507051	MACS_peak_2988	9.00	lobChIP_FOXA1inPBS
chr11	130969137	130969138	MACS_peak_2989	24.00	lobChIP_FOXA1inPBS
chr11	134132116	134132117	MACS_peak_2990	34.00	lobChIP_FOXA1inPBS
chr11	134612964	134612965	MACS_peak_2991	9.00	lobChIP_FOXA1inPBS
chr12	319683	319684	MACS_peak_2992	19.00	lobChIP_FOXA1inPBS
chr12	597298	597299	MACS_peak_2993	7.00	lobChIP_FOXA1inPBS
chr12	663979	663980	MACS_peak_2994	9.00	lobChIP_FOXA1inPBS
chr12	680936	680937	MACS_peak_2995	8.00	lobChIP_FOXA1inPBS
chr12	687189	687190	MACS_peak_2996	12.00	lobChIP_FOXA1inPBS
chr12	694877	694878	MACS_peak_2997	18.00	lobChIP_FOXA1inPBS
chr12	741515	741516	MACS_peak_2998	21.00	lobChIP_FOXA1inPBS
chr12	782313	782314	MACS_peak_2999	7.00	lobChIP_FOXA1inPBS
chr12	1099147	1099148	MACS_peak_3000	8.00	lobChIP_FOXA1inPBS
chr12	1099634	1099635	MACS_peak_3001	10.00	lobChIP_FOXA1inPBS
chr12	1163934	1163935	MACS_peak_3002	22.00	lobChIP_FOXA1inPBS
chr12	1476080	1476081	MACS_peak_3003	11.00	lobChIP_FOXA1inPBS
chr12	1719400	1719401	MACS_peak_3004	9.00	lobChIP_FOXA1inPBS
chr12	1776002	1776003	MACS_peak_3005	14.00	lobChIP_FOXA1inPBS
chr12	1842374	1842375	MACS_peak_3006	24.00	lobChIP_FOXA1inPBS
chr12	1848634	1848635	MACS_peak_3007	5.00	lobChIP_FOXA1inPBS
chr12	1850596	1850597	MACS_peak_3008	12.00	lobChIP_FOXA1inPBS
chr12	1854302	1854303	MACS_peak_3009	22.00	lobChIP_FOXA1inPBS
chr12	1875467	1875468	MACS_peak_3010	7.00	lobChIP_FOXA1inPBS
chr12	1877047	1877048	MACS_peak_3011	15.00	lobChIP_FOXA1inPBS
chr12	1879507	1879508	MACS_peak_3012	7.00	lobChIP_FOXA1inPBS
chr12	1947148	1947149	MACS_peak_3013	19.00	lobChIP_FOXA1inPBS
chr12	2103146	2103147	MACS_peak_3014	34.00	lobChIP_FOXA1inPBS
chr12	2357360	2357361	MACS_peak_3015	31.00	lobChIP_FOXA1inPBS
chr12	2400513	2400514	MACS_peak_3016	9.00	lobChIP_FOXA1inPBS
chr12	2894487	2894488	MACS_peak_3017	20.00	lobChIP_FOXA1inPBS
chr12	2922174	2922175	MACS_peak_3018	6.00	lobChIP_FOXA1inPBS
chr12	3071022	3071023	MACS_peak_3019	8.00	lobChIP_FOXA1inPBS
chr12	3213929	3213930	MACS_peak_3020	7.00	lobChIP_FOXA1inPBS
chr12	3942166	3942167	MACS_peak_3021	7.00	lobChIP_FOXA1inPBS
chr12	4068922	4068923	MACS_peak_3022	7.00	lobChIP_FOXA1inPBS
chr12	4120990	4120991	MACS_peak_3023	21.00	lobChIP_FOXA1inPBS
chr12	4124867	4124868	MACS_peak_3024	9.00	lobChIP_FOXA1inPBS
chr12	4166588	4166589	MACS_peak_3025	10.00	lobChIP_FOXA1inPBS
chr12	4595382	4595383	MACS_peak_3026	39.00	lobChIP_FOXA1inPBS
chr12	4615767	4615768	MACS_peak_3027	29.00	lobChIP_FOXA1inPBS
chr12	4816672	4816673	MACS_peak_3028	11.00	lobChIP_FOXA1inPBS
chr12	5677741	5677742	MACS_peak_3029	20.00	lobChIP_FOXA1inPBS
chr12	5695560	5695561	MACS_peak_3030	11.00	lobChIP_FOXA1inPBS
chr12	6168336	6168337	MACS_peak_3031	16.00	lobChIP_FOXA1inPBS
chr12	6180111	6180112	MACS_peak_3032	9.00	lobChIP_FOXA1inPBS
chr12	6181716	6181717	MACS_peak_3033	6.00	lobChIP_FOXA1inPBS
chr12	6347206	6347207	MACS_peak_3034	6.00	lobChIP_FOXA1inPBS
chr12	6403768	6403769	MACS_peak_3035	17.00	lobChIP_FOXA1inPBS
chr12	6472773	6472774	MACS_peak_3036	7.00	lobChIP_FOXA1inPBS
chr12	6480449	6480450	MACS_peak_3037	13.00	lobChIP_FOXA1inPBS
chr12	6529717	6529718	MACS_peak_3038	9.00	lobChIP_FOXA1inPBS
chr12	6641555	6641556	MACS_peak_3039	12.00	lobChIP_FOXA1inPBS
chr12	6885151	6885152	MACS_peak_3040	29.00	lobChIP_FOXA1inPBS
chr12	7090476	7090477	MACS_peak_3041	12.00	lobChIP_FOXA1inPBS
chr12	7186764	7186765	MACS_peak_3042	28.00	lobChIP_FOXA1inPBS
chr12	8004409	8004410	MACS_peak_3043	50.00	lobChIP_FOXA1inPBS
chr12	8043412	8043413	MACS_peak_3044	6.00	lobChIP_FOXA1inPBS
chr12	8058374	8058375	MACS_peak_3045	32.00	lobChIP_FOXA1inPBS
chr12	8059895	8059896	MACS_peak_3046	36.00	lobChIP_FOXA1inPBS
chr12	8135058	8135059	MACS_peak_3047	22.00	lobChIP_FOXA1inPBS
chr12	8810104	8810105	MACS_peak_3048	12.00	lobChIP_FOXA1inPBS
chr12	9266690	9266691	MACS_peak_3049	33.00	lobChIP_FOXA1inPBS
chr12	9268607	9268608	MACS_peak_3050	11.00	lobChIP_FOXA1inPBS
chr12	9274832	9274833	MACS_peak_3051	73.00	lobChIP_FOXA1inPBS
chr12	9284847	9284848	MACS_peak_3052	46.00	lobChIP_FOXA1inPBS
chr12	9288641	9288642	MACS_peak_3053	43.00	lobChIP_FOXA1inPBS
chr12	9405072	9405073	MACS_peak_3054	22.00	lobChIP_FOXA1inPBS
chr12	9495616	9495617	MACS_peak_3055	14.00	lobChIP_FOXA1inPBS
chr12	9743238	9743239	MACS_peak_3056	7.00	lobChIP_FOXA1inPBS
chr12	10548449	10548450	MACS_peak_3057	27.00	lobChIP_FOXA1inPBS
chr12	10592371	10592372	MACS_peak_3058	12.00	lobChIP_FOXA1inPBS
chr12	10946954	10946955	MACS_peak_3059	6.00	lobChIP_FOXA1inPBS
chr12	11038033	11038034	MACS_peak_3060	8.00	lobChIP_FOXA1inPBS
chr12	11044772	11044773	MACS_peak_3061	47.00	lobChIP_FOXA1inPBS
chr12	11071715	11071716	MACS_peak_3062	10.00	lobChIP_FOXA1inPBS
chr12	11224126	11224127	MACS_peak_3063	31.00	lobChIP_FOXA1inPBS
chr12	11865030	11865031	MACS_peak_3064	9.00	lobChIP_FOXA1inPBS
chr12	12038860	12038861	MACS_peak_3065	37.00	lobChIP_FOXA1inPBS
chr12	12062538	12062539	MACS_peak_3066	8.00	lobChIP_FOXA1inPBS
chr12	12112076	12112077	MACS_peak_3067	7.00	lobChIP_FOXA1inPBS
chr12	12321091	12321092	MACS_peak_3068	7.00	lobChIP_FOXA1inPBS
chr12	12369409	12369410	MACS_peak_3069	14.00	lobChIP_FOXA1inPBS
chr12	12394040	12394041	MACS_peak_3070	8.00	lobChIP_FOXA1inPBS
chr12	12430203	12430204	MACS_peak_3071	5.00	lobChIP_FOXA1inPBS
chr12	12464570	12464571	MACS_peak_3072	21.00	lobChIP_FOXA1inPBS
chr12	12467164	12467165	MACS_peak_3073	7.00	lobChIP_FOXA1inPBS
chr12	12504275	12504276	MACS_peak_3074	43.00	lobChIP_FOXA1inPBS
chr12	12534294	12534295	MACS_peak_3075	16.00	lobChIP_FOXA1inPBS
chr12	12545081	12545082	MACS_peak_3076	10.00	lobChIP_FOXA1inPBS
chr12	12551448	12551449	MACS_peak_3077	32.00	lobChIP_FOXA1inPBS
chr12	12556911	12556912	MACS_peak_3078	25.00	lobChIP_FOXA1inPBS
chr12	12559106	12559107	MACS_peak_3079	11.00	lobChIP_FOXA1inPBS
chr12	12570014	12570015	MACS_peak_3080	7.00	lobChIP_FOXA1inPBS
chr12	12583942	12583943	MACS_peak_3081	13.00	lobChIP_FOXA1inPBS
chr12	12592630	12592631	MACS_peak_3082	7.00	lobChIP_FOXA1inPBS
chr12	12602716	12602717	MACS_peak_3083	14.00	lobChIP_FOXA1inPBS
chr12	12603449	12603450	MACS_peak_3084	9.00	lobChIP_FOXA1inPBS
chr12	12642144	12642145	MACS_peak_3085	10.00	lobChIP_FOXA1inPBS
chr12	12699647	12699648	MACS_peak_3086	35.00	lobChIP_FOXA1inPBS
chr12	12713972	12713973	MACS_peak_3087	11.00	lobChIP_FOXA1inPBS
chr12	12764837	12764838	MACS_peak_3088	8.00	lobChIP_FOXA1inPBS
chr12	12857658	12857659	MACS_peak_3089	13.00	lobChIP_FOXA1inPBS
chr12	12875037	12875038	MACS_peak_3090	10.00	lobChIP_FOXA1inPBS
chr12	12875637	12875638	MACS_peak_3091	13.00	lobChIP_FOXA1inPBS
chr12	12910709	12910710	MACS_peak_3092	9.00	lobChIP_FOXA1inPBS
chr12	12932734	12932735	MACS_peak_3093	12.00	lobChIP_FOXA1inPBS
chr12	13036424	13036425	MACS_peak_3094	14.00	lobChIP_FOXA1inPBS
chr12	13136430	13136431	MACS_peak_3095	5.00	lobChIP_FOXA1inPBS
chr12	13340629	13340630	MACS_peak_3096	9.00	lobChIP_FOXA1inPBS
chr12	13376350	13376351	MACS_peak_3097	9.00	lobChIP_FOXA1inPBS
chr12	14145691	14145692	MACS_peak_3098	7.00	lobChIP_FOXA1inPBS
chr12	14416873	14416874	MACS_peak_3099	9.00	lobChIP_FOXA1inPBS
chr12	14437152	14437153	MACS_peak_3100	7.00	lobChIP_FOXA1inPBS
chr12	14519405	14519406	MACS_peak_3101	10.00	lobChIP_FOXA1inPBS
chr12	14537903	14537904	MACS_peak_3102	6.00	lobChIP_FOXA1inPBS
chr12	14995517	14995518	MACS_peak_3103	7.00	lobChIP_FOXA1inPBS
chr12	15746026	15746027	MACS_peak_3104	13.00	lobChIP_FOXA1inPBS
chr12	15769706	15769707	MACS_peak_3105	49.00	lobChIP_FOXA1inPBS
chr12	15782208	15782209	MACS_peak_3106	7.00	lobChIP_FOXA1inPBS
chr12	15933583	15933584	MACS_peak_3107	10.00	lobChIP_FOXA1inPBS
chr12	15973150	15973151	MACS_peak_3108	39.00	lobChIP_FOXA1inPBS
chr12	15976556	15976557	MACS_peak_3109	30.00	lobChIP_FOXA1inPBS
chr12	16283496	16283497	MACS_peak_3110	12.00	lobChIP_FOXA1inPBS
chr12	16758999	16759000	MACS_peak_3111	8.00	lobChIP_FOXA1inPBS
chr12	17092994	17092995	MACS_peak_3112	10.00	lobChIP_FOXA1inPBS
chr12	18395874	18395875	MACS_peak_3113	16.00	lobChIP_FOXA1inPBS
chr12	19219564	19219565	MACS_peak_3114	48.00	lobChIP_FOXA1inPBS
chr12	19250143	19250144	MACS_peak_3115	8.00	lobChIP_FOXA1inPBS
chr12	19284001	19284002	MACS_peak_3116	10.00	lobChIP_FOXA1inPBS
chr12	19439163	19439164	MACS_peak_3117	8.00	lobChIP_FOXA1inPBS
chr12	19591948	19591949	MACS_peak_3118	12.00	lobChIP_FOXA1inPBS
chr12	19631655	19631656	MACS_peak_3119	6.00	lobChIP_FOXA1inPBS
chr12	20328882	20328883	MACS_peak_3120	14.00	lobChIP_FOXA1inPBS
chr12	20754819	20754820	MACS_peak_3121	10.00	lobChIP_FOXA1inPBS
chr12	20782391	20782392	MACS_peak_3122	10.00	lobChIP_FOXA1inPBS
chr12	21489780	21489781	MACS_peak_3123	12.00	lobChIP_FOXA1inPBS
chr12	21504082	21504083	MACS_peak_3124	20.00	lobChIP_FOXA1inPBS
chr12	21716027	21716028	MACS_peak_3125	17.00	lobChIP_FOXA1inPBS
chr12	22204279	22204280	MACS_peak_3126	10.00	lobChIP_FOXA1inPBS
chr12	22488749	22488750	MACS_peak_3127	8.00	lobChIP_FOXA1inPBS
chr12	22751841	22751842	MACS_peak_3128	9.00	lobChIP_FOXA1inPBS
chr12	23910551	23910552	MACS_peak_3129	14.00	lobChIP_FOXA1inPBS
chr12	23980450	23980451	MACS_peak_3130	14.00	lobChIP_FOXA1inPBS
chr12	24094950	24094951	MACS_peak_3131	18.00	lobChIP_FOXA1inPBS
chr12	24178324	24178325	MACS_peak_3132	16.00	lobChIP_FOXA1inPBS
chr12	24191506	24191507	MACS_peak_3133	26.00	lobChIP_FOXA1inPBS
chr12	24202239	24202240	MACS_peak_3134	15.00	lobChIP_FOXA1inPBS
chr12	24222960	24222961	MACS_peak_3135	22.00	lobChIP_FOXA1inPBS
chr12	24231353	24231354	MACS_peak_3136	12.00	lobChIP_FOXA1inPBS
chr12	24233590	24233591	MACS_peak_3137	30.00	lobChIP_FOXA1inPBS
chr12	24802771	24802772	MACS_peak_3138	9.00	lobChIP_FOXA1inPBS
chr12	24850385	24850386	MACS_peak_3139	11.00	lobChIP_FOXA1inPBS
chr12	24906200	24906201	MACS_peak_3140	11.00	lobChIP_FOXA1inPBS
chr12	24907834	24907835	MACS_peak_3141	62.00	lobChIP_FOXA1inPBS
chr12	24913417	24913418	MACS_peak_3142	10.00	lobChIP_FOXA1inPBS
chr12	25064469	25064470	MACS_peak_3143	9.00	lobChIP_FOXA1inPBS
chr12	25409073	25409074	MACS_peak_3144	23.00	lobChIP_FOXA1inPBS
chr12	25429751	25429752	MACS_peak_3145	11.00	lobChIP_FOXA1inPBS
chr12	26182399	26182400	MACS_peak_3146	13.00	lobChIP_FOXA1inPBS
chr12	26582177	26582178	MACS_peak_3147	45.00	lobChIP_FOXA1inPBS
chr12	26789744	26789745	MACS_peak_3148	8.00	lobChIP_FOXA1inPBS
chr12	26834124	26834125	MACS_peak_3149	9.00	lobChIP_FOXA1inPBS
chr12	26889903	26889904	MACS_peak_3150	53.00	lobChIP_FOXA1inPBS
chr12	26890869	26890870	MACS_peak_3151	7.00	lobChIP_FOXA1inPBS
chr12	26898209	26898210	MACS_peak_3152	21.00	lobChIP_FOXA1inPBS
chr12	26920254	26920255	MACS_peak_3153	7.00	lobChIP_FOXA1inPBS
chr12	26922312	26922313	MACS_peak_3154	162.00	lobChIP_FOXA1inPBS
chr12	26929173	26929174	MACS_peak_3155	8.00	lobChIP_FOXA1inPBS
chr12	26943111	26943112	MACS_peak_3156	10.00	lobChIP_FOXA1inPBS
chr12	26968705	26968706	MACS_peak_3157	15.00	lobChIP_FOXA1inPBS
chr12	26971772	26971773	MACS_peak_3158	6.00	lobChIP_FOXA1inPBS
chr12	27701337	27701338	MACS_peak_3159	8.00	lobChIP_FOXA1inPBS
chr12	27849326	27849327	MACS_peak_3160	15.00	lobChIP_FOXA1inPBS
chr12	27850236	27850237	MACS_peak_3161	6.00	lobChIP_FOXA1inPBS
chr12	28052498	28052499	MACS_peak_3162	13.00	lobChIP_FOXA1inPBS
chr12	28174866	28174867	MACS_peak_3163	9.00	lobChIP_FOXA1inPBS
chr12	28355781	28355782	MACS_peak_3164	12.00	lobChIP_FOXA1inPBS
chr12	29961882	29961883	MACS_peak_3165	10.00	lobChIP_FOXA1inPBS
chr12	30661458	30661459	MACS_peak_3166	21.00	lobChIP_FOXA1inPBS
chr12	30749025	30749026	MACS_peak_3167	7.00	lobChIP_FOXA1inPBS
chr12	30752657	30752658	MACS_peak_3168	65.00	lobChIP_FOXA1inPBS
chr12	30757469	30757470	MACS_peak_3169	14.00	lobChIP_FOXA1inPBS
chr12	30916265	30916266	MACS_peak_3170	55.00	lobChIP_FOXA1inPBS
chr12	31508596	31508597	MACS_peak_3171	17.00	lobChIP_FOXA1inPBS
chr12	31516112	31516113	MACS_peak_3172	6.00	lobChIP_FOXA1inPBS
chr12	31522816	31522817	MACS_peak_3173	9.00	lobChIP_FOXA1inPBS
chr12	31742938	31742939	MACS_peak_3174	12.00	lobChIP_FOXA1inPBS
chr12	31868393	31868394	MACS_peak_3175	8.00	lobChIP_FOXA1inPBS
chr12	31902485	31902486	MACS_peak_3176	52.00	lobChIP_FOXA1inPBS
chr12	31926243	31926244	MACS_peak_3177	33.00	lobChIP_FOXA1inPBS
chr12	31968255	31968256	MACS_peak_3178	10.00	lobChIP_FOXA1inPBS
chr12	31998762	31998763	MACS_peak_3179	27.00	lobChIP_FOXA1inPBS
chr12	32007348	32007349	MACS_peak_3180	32.00	lobChIP_FOXA1inPBS
chr12	32340202	32340203	MACS_peak_3181	53.00	lobChIP_FOXA1inPBS
chr12	32599425	32599426	MACS_peak_3182	8.00	lobChIP_FOXA1inPBS
chr12	32614531	32614532	MACS_peak_3183	21.00	lobChIP_FOXA1inPBS
chr12	32687099	32687100	MACS_peak_3184	27.00	lobChIP_FOXA1inPBS
chr12	32804519	32804520	MACS_peak_3185	7.00	lobChIP_FOXA1inPBS
chr12	32831073	32831074	MACS_peak_3186	10.00	lobChIP_FOXA1inPBS
chr12	34271801	34271802	MACS_peak_3187	6.00	lobChIP_FOXA1inPBS
chr12	38518521	38518522	MACS_peak_3188	11.00	lobChIP_FOXA1inPBS
chr12	39280193	39280194	MACS_peak_3189	10.00	lobChIP_FOXA1inPBS
chr12	39286147	39286148	MACS_peak_3190	51.00	lobChIP_FOXA1inPBS
chr12	39713201	39713202	MACS_peak_3191	9.00	lobChIP_FOXA1inPBS
chr12	39821391	39821392	MACS_peak_3192	13.00	lobChIP_FOXA1inPBS
chr12	39837873	39837874	MACS_peak_3193	5.00	lobChIP_FOXA1inPBS
chr12	39869138	39869139	MACS_peak_3194	8.00	lobChIP_FOXA1inPBS
chr12	41680482	41680483	MACS_peak_3195	10.00	lobChIP_FOXA1inPBS
chr12	44189813	44189814	MACS_peak_3196	5.00	lobChIP_FOXA1inPBS
chr12	44389238	44389239	MACS_peak_3197	30.00	lobChIP_FOXA1inPBS
chr12	44838943	44838944	MACS_peak_3198	7.00	lobChIP_FOXA1inPBS
chr12	44848984	44848985	MACS_peak_3199	6.00	lobChIP_FOXA1inPBS
chr12	44919812	44919813	MACS_peak_3200	22.00	lobChIP_FOXA1inPBS
chr12	45654158	45654159	MACS_peak_3201	10.00	lobChIP_FOXA1inPBS
chr12	45663165	45663166	MACS_peak_3202	15.00	lobChIP_FOXA1inPBS
chr12	46198128	46198129	MACS_peak_3203	12.00	lobChIP_FOXA1inPBS
chr12	46388579	46388580	MACS_peak_3204	8.00	lobChIP_FOXA1inPBS
chr12	46752072	46752073	MACS_peak_3205	15.00	lobChIP_FOXA1inPBS
chr12	46759835	46759836	MACS_peak_3206	20.00	lobChIP_FOXA1inPBS
chr12	46763812	46763813	MACS_peak_3207	17.00	lobChIP_FOXA1inPBS
chr12	46780439	46780440	MACS_peak_3208	10.00	lobChIP_FOXA1inPBS
chr12	46797157	46797158	MACS_peak_3209	7.00	lobChIP_FOXA1inPBS
chr12	46824317	46824318	MACS_peak_3210	9.00	lobChIP_FOXA1inPBS
chr12	46850916	46850917	MACS_peak_3211	10.00	lobChIP_FOXA1inPBS
chr12	46867703	46867704	MACS_peak_3212	15.00	lobChIP_FOXA1inPBS
chr12	46877710	46877711	MACS_peak_3213	13.00	lobChIP_FOXA1inPBS
chr12	46886488	46886489	MACS_peak_3214	103.00	lobChIP_FOXA1inPBS
chr12	46887861	46887862	MACS_peak_3215	30.00	lobChIP_FOXA1inPBS
chr12	46917807	46917808	MACS_peak_3216	7.00	lobChIP_FOXA1inPBS
chr12	46970912	46970913	MACS_peak_3217	18.00	lobChIP_FOXA1inPBS
chr12	46973483	46973484	MACS_peak_3218	25.00	lobChIP_FOXA1inPBS
chr12	46979833	46979834	MACS_peak_3219	8.00	lobChIP_FOXA1inPBS
chr12	47059007	47059008	MACS_peak_3220	12.00	lobChIP_FOXA1inPBS
chr12	47169767	47169768	MACS_peak_3221	9.00	lobChIP_FOXA1inPBS
chr12	47202207	47202208	MACS_peak_3222	8.00	lobChIP_FOXA1inPBS
chr12	47208171	47208172	MACS_peak_3223	25.00	lobChIP_FOXA1inPBS
chr12	47234048	47234049	MACS_peak_3224	24.00	lobChIP_FOXA1inPBS
chr12	47328540	47328541	MACS_peak_3225	7.00	lobChIP_FOXA1inPBS
chr12	47377895	47377896	MACS_peak_3226	10.00	lobChIP_FOXA1inPBS
chr12	47615492	47615493	MACS_peak_3227	24.00	lobChIP_FOXA1inPBS
chr12	47740560	47740561	MACS_peak_3228	13.00	lobChIP_FOXA1inPBS
chr12	48028080	48028081	MACS_peak_3229	11.00	lobChIP_FOXA1inPBS
chr12	48046766	48046767	MACS_peak_3230	18.00	lobChIP_FOXA1inPBS
chr12	48109223	48109224	MACS_peak_3231	13.00	lobChIP_FOXA1inPBS
chr12	48136134	48136135	MACS_peak_3232	11.00	lobChIP_FOXA1inPBS
chr12	48152932	48152933	MACS_peak_3233	8.00	lobChIP_FOXA1inPBS
chr12	48162384	48162385	MACS_peak_3234	10.00	lobChIP_FOXA1inPBS
chr12	48214203	48214204	MACS_peak_3235	8.00	lobChIP_FOXA1inPBS
chr12	48302234	48302235	MACS_peak_3236	8.00	lobChIP_FOXA1inPBS
chr12	49260910	49260911	MACS_peak_3237	28.00	lobChIP_FOXA1inPBS
chr12	49277588	49277589	MACS_peak_3238	13.00	lobChIP_FOXA1inPBS
chr12	49301244	49301245	MACS_peak_3239	6.00	lobChIP_FOXA1inPBS
chr12	49304176	49304177	MACS_peak_3240	43.00	lobChIP_FOXA1inPBS
chr12	49402892	49402893	MACS_peak_3241	8.00	lobChIP_FOXA1inPBS
chr12	49649904	49649905	MACS_peak_3242	8.00	lobChIP_FOXA1inPBS
chr12	49661400	49661401	MACS_peak_3243	8.00	lobChIP_FOXA1inPBS
chr12	49815263	49815264	MACS_peak_3244	13.00	lobChIP_FOXA1inPBS
chr12	50066817	50066818	MACS_peak_3245	8.00	lobChIP_FOXA1inPBS
chr12	50136198	50136199	MACS_peak_3246	6.00	lobChIP_FOXA1inPBS
chr12	50281945	50281946	MACS_peak_3247	11.00	lobChIP_FOXA1inPBS
chr12	50401018	50401019	MACS_peak_3248	9.00	lobChIP_FOXA1inPBS
chr12	50482611	50482612	MACS_peak_3249	9.00	lobChIP_FOXA1inPBS
chr12	50526535	50526536	MACS_peak_3250	8.00	lobChIP_FOXA1inPBS
chr12	50582260	50582261	MACS_peak_3251	10.00	lobChIP_FOXA1inPBS
chr12	50635184	50635185	MACS_peak_3252	133.00	lobChIP_FOXA1inPBS
chr12	50653564	50653565	MACS_peak_3253	15.00	lobChIP_FOXA1inPBS
chr12	50720652	50720653	MACS_peak_3254	19.00	lobChIP_FOXA1inPBS
chr12	50790493	50790494	MACS_peak_3255	34.00	lobChIP_FOXA1inPBS
chr12	50796226	50796227	MACS_peak_3256	15.00	lobChIP_FOXA1inPBS
chr12	50903291	50903292	MACS_peak_3257	13.00	lobChIP_FOXA1inPBS
chr12	50921071	50921072	MACS_peak_3258	35.00	lobChIP_FOXA1inPBS
chr12	50932566	50932567	MACS_peak_3259	12.00	lobChIP_FOXA1inPBS
chr12	50942845	50942846	MACS_peak_3260	9.00	lobChIP_FOXA1inPBS
chr12	50961874	50961875	MACS_peak_3261	8.00	lobChIP_FOXA1inPBS
chr12	51028719	51028720	MACS_peak_3262	9.00	lobChIP_FOXA1inPBS
chr12	51049353	51049354	MACS_peak_3263	10.00	lobChIP_FOXA1inPBS
chr12	51053257	51053258	MACS_peak_3264	16.00	lobChIP_FOXA1inPBS
chr12	51128657	51128658	MACS_peak_3265	20.00	lobChIP_FOXA1inPBS
chr12	51180921	51180922	MACS_peak_3266	7.00	lobChIP_FOXA1inPBS
chr12	51326586	51326587	MACS_peak_3267	7.00	lobChIP_FOXA1inPBS
chr12	51329150	51329151	MACS_peak_3268	17.00	lobChIP_FOXA1inPBS
chr12	51378176	51378177	MACS_peak_3269	8.00	lobChIP_FOXA1inPBS
chr12	51419242	51419243	MACS_peak_3270	5.00	lobChIP_FOXA1inPBS
chr12	51461355	51461356	MACS_peak_3271	6.00	lobChIP_FOXA1inPBS
chr12	51555095	51555096	MACS_peak_3272	18.00	lobChIP_FOXA1inPBS
chr12	51592598	51592599	MACS_peak_3273	18.00	lobChIP_FOXA1inPBS
chr12	51864054	51864055	MACS_peak_3274	11.00	lobChIP_FOXA1inPBS
chr12	52348889	52348890	MACS_peak_3275	8.00	lobChIP_FOXA1inPBS
chr12	52355880	52355881	MACS_peak_3276	7.00	lobChIP_FOXA1inPBS
chr12	52513292	52513293	MACS_peak_3277	15.00	lobChIP_FOXA1inPBS
chr12	52641354	52641355	MACS_peak_3278	19.00	lobChIP_FOXA1inPBS
chr12	53289241	53289242	MACS_peak_3279	16.00	lobChIP_FOXA1inPBS
chr12	53321371	53321372	MACS_peak_3280	8.00	lobChIP_FOXA1inPBS
chr12	53343727	53343728	MACS_peak_3281	11.00	lobChIP_FOXA1inPBS
chr12	53370753	53370754	MACS_peak_3282	8.00	lobChIP_FOXA1inPBS
chr12	53386840	53386841	MACS_peak_3283	8.00	lobChIP_FOXA1inPBS
chr12	53405929	53405930	MACS_peak_3284	6.00	lobChIP_FOXA1inPBS
chr12	53445102	53445103	MACS_peak_3285	9.00	lobChIP_FOXA1inPBS
chr12	53455341	53455342	MACS_peak_3286	12.00	lobChIP_FOXA1inPBS
chr12	53568796	53568797	MACS_peak_3287	23.00	lobChIP_FOXA1inPBS
chr12	53593708	53593709	MACS_peak_3288	11.00	lobChIP_FOXA1inPBS
chr12	53643853	53643854	MACS_peak_3289	8.00	lobChIP_FOXA1inPBS
chr12	53932069	53932070	MACS_peak_3290	33.00	lobChIP_FOXA1inPBS
chr12	54052994	54052995	MACS_peak_3291	13.00	lobChIP_FOXA1inPBS
chr12	54219221	54219222	MACS_peak_3292	29.00	lobChIP_FOXA1inPBS
chr12	54529478	54529479	MACS_peak_3293	20.00	lobChIP_FOXA1inPBS
chr12	54530781	54530782	MACS_peak_3294	21.00	lobChIP_FOXA1inPBS
chr12	54547628	54547629	MACS_peak_3295	35.00	lobChIP_FOXA1inPBS
chr12	54722590	54722591	MACS_peak_3296	7.00	lobChIP_FOXA1inPBS
chr12	54981229	54981230	MACS_peak_3297	23.00	lobChIP_FOXA1inPBS
chr12	55286087	55286088	MACS_peak_3298	61.00	lobChIP_FOXA1inPBS
chr12	55893604	55893605	MACS_peak_3299	28.00	lobChIP_FOXA1inPBS
chr12	56013696	56013697	MACS_peak_3300	10.00	lobChIP_FOXA1inPBS
chr12	56050360	56050361	MACS_peak_3301	11.00	lobChIP_FOXA1inPBS
chr12	56098819	56098820	MACS_peak_3302	14.00	lobChIP_FOXA1inPBS
chr12	56474719	56474720	MACS_peak_3303	9.00	lobChIP_FOXA1inPBS
chr12	56475814	56475815	MACS_peak_3304	10.00	lobChIP_FOXA1inPBS
chr12	56520168	56520169	MACS_peak_3305	13.00	lobChIP_FOXA1inPBS
chr12	56545598	56545599	MACS_peak_3306	9.00	lobChIP_FOXA1inPBS
chr12	56658186	56658187	MACS_peak_3307	8.00	lobChIP_FOXA1inPBS
chr12	56695746	56695747	MACS_peak_3308	9.00	lobChIP_FOXA1inPBS
chr12	56756632	56756633	MACS_peak_3309	22.00	lobChIP_FOXA1inPBS
chr12	56768989	56768990	MACS_peak_3310	13.00	lobChIP_FOXA1inPBS
chr12	56920625	56920626	MACS_peak_3311	6.00	lobChIP_FOXA1inPBS
chr12	57153282	57153283	MACS_peak_3312	28.00	lobChIP_FOXA1inPBS
chr12	57291868	57291869	MACS_peak_3313	6.00	lobChIP_FOXA1inPBS
chr12	57335611	57335612	MACS_peak_3314	27.00	lobChIP_FOXA1inPBS
chr12	57395458	57395459	MACS_peak_3315	24.00	lobChIP_FOXA1inPBS
chr12	57412336	57412337	MACS_peak_3316	14.00	lobChIP_FOXA1inPBS
chr12	57471485	57471486	MACS_peak_3317	9.00	lobChIP_FOXA1inPBS
chr12	57506036	57506037	MACS_peak_3318	9.00	lobChIP_FOXA1inPBS
chr12	57524875	57524876	MACS_peak_3319	15.00	lobChIP_FOXA1inPBS
chr12	57527138	57527139	MACS_peak_3320	6.00	lobChIP_FOXA1inPBS
chr12	57540851	57540852	MACS_peak_3321	8.00	lobChIP_FOXA1inPBS
chr12	57543850	57543851	MACS_peak_3322	32.00	lobChIP_FOXA1inPBS
chr12	57845396	57845397	MACS_peak_3323	27.00	lobChIP_FOXA1inPBS
chr12	57970185	57970186	MACS_peak_3324	5.00	lobChIP_FOXA1inPBS
chr12	57973713	57973714	MACS_peak_3325	11.00	lobChIP_FOXA1inPBS
chr12	58087012	58087013	MACS_peak_3326	10.00	lobChIP_FOXA1inPBS
chr12	58239423	58239424	MACS_peak_3327	17.00	lobChIP_FOXA1inPBS
chr12	58296393	58296394	MACS_peak_3328	13.00	lobChIP_FOXA1inPBS
chr12	58619606	58619607	MACS_peak_3329	16.00	lobChIP_FOXA1inPBS
chr12	58704622	58704623	MACS_peak_3330	11.00	lobChIP_FOXA1inPBS
chr12	59075731	59075732	MACS_peak_3331	9.00	lobChIP_FOXA1inPBS
chr12	59153637	59153638	MACS_peak_3332	7.00	lobChIP_FOXA1inPBS
chr12	59159995	59159996	MACS_peak_3333	59.00	lobChIP_FOXA1inPBS
chr12	59234936	59234937	MACS_peak_3334	10.00	lobChIP_FOXA1inPBS
chr12	59306733	59306734	MACS_peak_3335	9.00	lobChIP_FOXA1inPBS
chr12	60043132	60043133	MACS_peak_3336	10.00	lobChIP_FOXA1inPBS
chr12	60118329	60118330	MACS_peak_3337	12.00	lobChIP_FOXA1inPBS
chr12	60562421	60562422	MACS_peak_3338	20.00	lobChIP_FOXA1inPBS
chr12	60660526	60660527	MACS_peak_3339	34.00	lobChIP_FOXA1inPBS
chr12	60845407	60845408	MACS_peak_3340	10.00	lobChIP_FOXA1inPBS
chr12	62400087	62400088	MACS_peak_3341	19.00	lobChIP_FOXA1inPBS
chr12	62995775	62995776	MACS_peak_3342	8.00	lobChIP_FOXA1inPBS
chr12	63154711	63154712	MACS_peak_3343	32.00	lobChIP_FOXA1inPBS
chr12	63286151	63286152	MACS_peak_3344	10.00	lobChIP_FOXA1inPBS
chr12	63294023	63294024	MACS_peak_3345	6.00	lobChIP_FOXA1inPBS
chr12	63319703	63319704	MACS_peak_3346	16.00	lobChIP_FOXA1inPBS
chr12	63351140	63351141	MACS_peak_3347	13.00	lobChIP_FOXA1inPBS
chr12	63360833	63360834	MACS_peak_3348	41.00	lobChIP_FOXA1inPBS
chr12	64199975	64199976	MACS_peak_3349	18.00	lobChIP_FOXA1inPBS
chr12	64239658	64239659	MACS_peak_3350	11.00	lobChIP_FOXA1inPBS
chr12	64256259	64256260	MACS_peak_3351	10.00	lobChIP_FOXA1inPBS
chr12	64311919	64311920	MACS_peak_3352	11.00	lobChIP_FOXA1inPBS
chr12	64312647	64312648	MACS_peak_3353	14.00	lobChIP_FOXA1inPBS
chr12	64338438	64338439	MACS_peak_3354	19.00	lobChIP_FOXA1inPBS
chr12	64631383	64631384	MACS_peak_3355	12.00	lobChIP_FOXA1inPBS
chr12	64984221	64984222	MACS_peak_3356	8.00	lobChIP_FOXA1inPBS
chr12	65019145	65019146	MACS_peak_3357	7.00	lobChIP_FOXA1inPBS
chr12	65027913	65027914	MACS_peak_3358	9.00	lobChIP_FOXA1inPBS
chr12	65030918	65030919	MACS_peak_3359	7.00	lobChIP_FOXA1inPBS
chr12	65049142	65049143	MACS_peak_3360	8.00	lobChIP_FOXA1inPBS
chr12	65050407	65050408	MACS_peak_3361	32.00	lobChIP_FOXA1inPBS
chr12	65060010	65060011	MACS_peak_3362	15.00	lobChIP_FOXA1inPBS
chr12	65138927	65138928	MACS_peak_3363	8.00	lobChIP_FOXA1inPBS
chr12	65190208	65190209	MACS_peak_3364	10.00	lobChIP_FOXA1inPBS
chr12	65191169	65191170	MACS_peak_3365	9.00	lobChIP_FOXA1inPBS
chr12	65212099	65212100	MACS_peak_3366	6.00	lobChIP_FOXA1inPBS
chr12	65507505	65507506	MACS_peak_3367	12.00	lobChIP_FOXA1inPBS
chr12	65721594	65721595	MACS_peak_3368	12.00	lobChIP_FOXA1inPBS
chr12	66025620	66025621	MACS_peak_3369	7.00	lobChIP_FOXA1inPBS
chr12	66092342	66092343	MACS_peak_3370	7.00	lobChIP_FOXA1inPBS
chr12	66232516	66232517	MACS_peak_3371	18.00	lobChIP_FOXA1inPBS
chr12	66248625	66248626	MACS_peak_3372	7.00	lobChIP_FOXA1inPBS
chr12	66290014	66290015	MACS_peak_3373	11.00	lobChIP_FOXA1inPBS
chr12	66324037	66324038	MACS_peak_3374	21.00	lobChIP_FOXA1inPBS
chr12	66331275	66331276	MACS_peak_3375	6.00	lobChIP_FOXA1inPBS
chr12	66443032	66443033	MACS_peak_3376	40.00	lobChIP_FOXA1inPBS
chr12	66460212	66460213	MACS_peak_3377	12.00	lobChIP_FOXA1inPBS
chr12	66468555	66468556	MACS_peak_3378	7.00	lobChIP_FOXA1inPBS
chr12	68823023	68823024	MACS_peak_3379	10.00	lobChIP_FOXA1inPBS
chr12	69181419	69181420	MACS_peak_3380	13.00	lobChIP_FOXA1inPBS
chr12	69183118	69183119	MACS_peak_3381	18.00	lobChIP_FOXA1inPBS
chr12	69201699	69201700	MACS_peak_3382	10.00	lobChIP_FOXA1inPBS
chr12	69244403	69244404	MACS_peak_3383	7.00	lobChIP_FOXA1inPBS
chr12	69440335	69440336	MACS_peak_3384	11.00	lobChIP_FOXA1inPBS
chr12	69446783	69446784	MACS_peak_3385	6.00	lobChIP_FOXA1inPBS
chr12	69545090	69545091	MACS_peak_3386	7.00	lobChIP_FOXA1inPBS
chr12	69724140	69724141	MACS_peak_3387	19.00	lobChIP_FOXA1inPBS
chr12	69726824	69726825	MACS_peak_3388	21.00	lobChIP_FOXA1inPBS
chr12	69732100	69732101	MACS_peak_3389	17.00	lobChIP_FOXA1inPBS
chr12	69749038	69749039	MACS_peak_3390	7.00	lobChIP_FOXA1inPBS
chr12	69811808	69811809	MACS_peak_3391	36.00	lobChIP_FOXA1inPBS
chr12	69860918	69860919	MACS_peak_3392	10.00	lobChIP_FOXA1inPBS
chr12	70319359	70319360	MACS_peak_3393	16.00	lobChIP_FOXA1inPBS
chr12	70664941	70664942	MACS_peak_3394	10.00	lobChIP_FOXA1inPBS
chr12	71105749	71105750	MACS_peak_3395	10.00	lobChIP_FOXA1inPBS
chr12	71127346	71127347	MACS_peak_3396	21.00	lobChIP_FOXA1inPBS
chr12	71549233	71549234	MACS_peak_3397	16.00	lobChIP_FOXA1inPBS
chr12	71552607	71552608	MACS_peak_3398	23.00	lobChIP_FOXA1inPBS
chr12	71557216	71557217	MACS_peak_3399	44.00	lobChIP_FOXA1inPBS
chr12	71560949	71560950	MACS_peak_3400	8.00	lobChIP_FOXA1inPBS
chr12	71572115	71572116	MACS_peak_3401	10.00	lobChIP_FOXA1inPBS
chr12	71805627	71805628	MACS_peak_3402	16.00	lobChIP_FOXA1inPBS
chr12	71832985	71832986	MACS_peak_3403	27.00	lobChIP_FOXA1inPBS
chr12	71834795	71834796	MACS_peak_3404	9.00	lobChIP_FOXA1inPBS
chr12	71866428	71866429	MACS_peak_3405	9.00	lobChIP_FOXA1inPBS
chr12	71930882	71930883	MACS_peak_3406	23.00	lobChIP_FOXA1inPBS
chr12	72348867	72348868	MACS_peak_3407	17.00	lobChIP_FOXA1inPBS
chr12	72740356	72740357	MACS_peak_3408	7.00	lobChIP_FOXA1inPBS
chr12	73777848	73777849	MACS_peak_3409	12.00	lobChIP_FOXA1inPBS
chr12	74163258	74163259	MACS_peak_3410	29.00	lobChIP_FOXA1inPBS
chr12	75952192	75952193	MACS_peak_3411	11.00	lobChIP_FOXA1inPBS
chr12	75972475	75972476	MACS_peak_3412	12.00	lobChIP_FOXA1inPBS
chr12	76017667	76017668	MACS_peak_3413	11.00	lobChIP_FOXA1inPBS
chr12	76029595	76029596	MACS_peak_3414	10.00	lobChIP_FOXA1inPBS
chr12	76060294	76060295	MACS_peak_3415	71.00	lobChIP_FOXA1inPBS
chr12	76102758	76102759	MACS_peak_3416	15.00	lobChIP_FOXA1inPBS
chr12	76106192	76106193	MACS_peak_3417	7.00	lobChIP_FOXA1inPBS
chr12	76108527	76108528	MACS_peak_3418	8.00	lobChIP_FOXA1inPBS
chr12	76149077	76149078	MACS_peak_3419	23.00	lobChIP_FOXA1inPBS
chr12	76160675	76160676	MACS_peak_3420	23.00	lobChIP_FOXA1inPBS
chr12	76192464	76192465	MACS_peak_3421	33.00	lobChIP_FOXA1inPBS
chr12	76338090	76338091	MACS_peak_3422	72.00	lobChIP_FOXA1inPBS
chr12	76371883	76371884	MACS_peak_3423	8.00	lobChIP_FOXA1inPBS
chr12	76384314	76384315	MACS_peak_3424	75.00	lobChIP_FOXA1inPBS
chr12	76431127	76431128	MACS_peak_3425	6.00	lobChIP_FOXA1inPBS
chr12	76741658	76741659	MACS_peak_3426	8.00	lobChIP_FOXA1inPBS
chr12	76814893	76814894	MACS_peak_3427	10.00	lobChIP_FOXA1inPBS
chr12	77015543	77015544	MACS_peak_3428	39.00	lobChIP_FOXA1inPBS
chr12	79442510	79442511	MACS_peak_3429	9.00	lobChIP_FOXA1inPBS
chr12	79694097	79694098	MACS_peak_3430	8.00	lobChIP_FOXA1inPBS
chr12	79818256	79818257	MACS_peak_3431	14.00	lobChIP_FOXA1inPBS
chr12	79924205	79924206	MACS_peak_3432	14.00	lobChIP_FOXA1inPBS
chr12	79926989	79926990	MACS_peak_3433	6.00	lobChIP_FOXA1inPBS
chr12	79943592	79943593	MACS_peak_3434	28.00	lobChIP_FOXA1inPBS
chr12	79954064	79954065	MACS_peak_3435	12.00	lobChIP_FOXA1inPBS
chr12	79960509	79960510	MACS_peak_3436	10.00	lobChIP_FOXA1inPBS
chr12	80037770	80037771	MACS_peak_3437	36.00	lobChIP_FOXA1inPBS
chr12	80055805	80055806	MACS_peak_3438	17.00	lobChIP_FOXA1inPBS
chr12	80166238	80166239	MACS_peak_3439	11.00	lobChIP_FOXA1inPBS
chr12	81311632	81311633	MACS_peak_3440	9.00	lobChIP_FOXA1inPBS
chr12	81320053	81320054	MACS_peak_3441	30.00	lobChIP_FOXA1inPBS
chr12	81352039	81352040	MACS_peak_3442	7.00	lobChIP_FOXA1inPBS
chr12	81472553	81472554	MACS_peak_3443	7.00	lobChIP_FOXA1inPBS
chr12	81479818	81479819	MACS_peak_3444	20.00	lobChIP_FOXA1inPBS
chr12	81534632	81534633	MACS_peak_3445	10.00	lobChIP_FOXA1inPBS
chr12	81537086	81537087	MACS_peak_3446	52.00	lobChIP_FOXA1inPBS
chr12	81540475	81540476	MACS_peak_3447	16.00	lobChIP_FOXA1inPBS
chr12	81662490	81662491	MACS_peak_3448	14.00	lobChIP_FOXA1inPBS
chr12	82708314	82708315	MACS_peak_3449	9.00	lobChIP_FOXA1inPBS
chr12	83028727	83028728	MACS_peak_3450	8.00	lobChIP_FOXA1inPBS
chr12	86036768	86036769	MACS_peak_3451	24.00	lobChIP_FOXA1inPBS
chr12	86230402	86230403	MACS_peak_3452	6.00	lobChIP_FOXA1inPBS
chr12	88347286	88347287	MACS_peak_3453	7.00	lobChIP_FOXA1inPBS
chr12	88852381	88852382	MACS_peak_3454	9.00	lobChIP_FOXA1inPBS
chr12	88958152	88958153	MACS_peak_3455	8.00	lobChIP_FOXA1inPBS
chr12	89320189	89320190	MACS_peak_3456	27.00	lobChIP_FOXA1inPBS
chr12	89352141	89352142	MACS_peak_3457	20.00	lobChIP_FOXA1inPBS
chr12	89452397	89452398	MACS_peak_3458	6.00	lobChIP_FOXA1inPBS
chr12	89746477	89746478	MACS_peak_3459	16.00	lobChIP_FOXA1inPBS
chr12	89764958	89764959	MACS_peak_3460	6.00	lobChIP_FOXA1inPBS
chr12	89777086	89777087	MACS_peak_3461	23.00	lobChIP_FOXA1inPBS
chr12	89826355	89826356	MACS_peak_3462	10.00	lobChIP_FOXA1inPBS
chr12	89853280	89853281	MACS_peak_3463	10.00	lobChIP_FOXA1inPBS
chr12	89901201	89901202	MACS_peak_3464	5.00	lobChIP_FOXA1inPBS
chr12	89915959	89915960	MACS_peak_3465	13.00	lobChIP_FOXA1inPBS
chr12	90019520	90019521	MACS_peak_3466	14.00	lobChIP_FOXA1inPBS
chr12	90026769	90026770	MACS_peak_3467	90.00	lobChIP_FOXA1inPBS
chr12	90039146	90039147	MACS_peak_3468	7.00	lobChIP_FOXA1inPBS
chr12	91245809	91245810	MACS_peak_3469	10.00	lobChIP_FOXA1inPBS
chr12	92458971	92458972	MACS_peak_3470	17.00	lobChIP_FOXA1inPBS
chr12	92464975	92464976	MACS_peak_3471	28.00	lobChIP_FOXA1inPBS
chr12	92735211	92735212	MACS_peak_3472	10.00	lobChIP_FOXA1inPBS
chr12	92897590	92897591	MACS_peak_3473	16.00	lobChIP_FOXA1inPBS
chr12	93221904	93221905	MACS_peak_3474	25.00	lobChIP_FOXA1inPBS
chr12	93339718	93339719	MACS_peak_3475	31.00	lobChIP_FOXA1inPBS
chr12	93341773	93341774	MACS_peak_3476	10.00	lobChIP_FOXA1inPBS
chr12	93367513	93367514	MACS_peak_3477	7.00	lobChIP_FOXA1inPBS
chr12	93399690	93399691	MACS_peak_3478	10.00	lobChIP_FOXA1inPBS
chr12	93410043	93410044	MACS_peak_3479	23.00	lobChIP_FOXA1inPBS
chr12	93414254	93414255	MACS_peak_3480	44.00	lobChIP_FOXA1inPBS
chr12	93481325	93481326	MACS_peak_3481	50.00	lobChIP_FOXA1inPBS
chr12	93484834	93484835	MACS_peak_3482	12.00	lobChIP_FOXA1inPBS
chr12	93521769	93521770	MACS_peak_3483	7.00	lobChIP_FOXA1inPBS
chr12	93529253	93529254	MACS_peak_3484	41.00	lobChIP_FOXA1inPBS
chr12	93570725	93570726	MACS_peak_3485	7.00	lobChIP_FOXA1inPBS
chr12	93668010	93668011	MACS_peak_3486	17.00	lobChIP_FOXA1inPBS
chr12	93686941	93686942	MACS_peak_3487	7.00	lobChIP_FOXA1inPBS
chr12	93708693	93708694	MACS_peak_3488	6.00	lobChIP_FOXA1inPBS
chr12	93790785	93790786	MACS_peak_3489	10.00	lobChIP_FOXA1inPBS
chr12	93833966	93833967	MACS_peak_3490	19.00	lobChIP_FOXA1inPBS
chr12	93920770	93920771	MACS_peak_3491	11.00	lobChIP_FOXA1inPBS
chr12	93957774	93957775	MACS_peak_3492	23.00	lobChIP_FOXA1inPBS
chr12	94136385	94136386	MACS_peak_3493	12.00	lobChIP_FOXA1inPBS
chr12	94208305	94208306	MACS_peak_3494	7.00	lobChIP_FOXA1inPBS
chr12	94451688	94451689	MACS_peak_3495	8.00	lobChIP_FOXA1inPBS
chr12	94546683	94546684	MACS_peak_3496	30.00	lobChIP_FOXA1inPBS
chr12	94567005	94567006	MACS_peak_3497	15.00	lobChIP_FOXA1inPBS
chr12	94577660	94577661	MACS_peak_3498	16.00	lobChIP_FOXA1inPBS
chr12	94624198	94624199	MACS_peak_3499	12.00	lobChIP_FOXA1inPBS
chr12	94699627	94699628	MACS_peak_3500	14.00	lobChIP_FOXA1inPBS
chr12	94929390	94929391	MACS_peak_3501	11.00	lobChIP_FOXA1inPBS
chr12	95158146	95158147	MACS_peak_3502	15.00	lobChIP_FOXA1inPBS
chr12	95324258	95324259	MACS_peak_3503	22.00	lobChIP_FOXA1inPBS
chr12	95557777	95557778	MACS_peak_3504	10.00	lobChIP_FOXA1inPBS
chr12	95585269	95585270	MACS_peak_3505	8.00	lobChIP_FOXA1inPBS
chr12	95712128	95712129	MACS_peak_3506	57.00	lobChIP_FOXA1inPBS
chr12	95734065	95734066	MACS_peak_3507	14.00	lobChIP_FOXA1inPBS
chr12	95839898	95839899	MACS_peak_3508	12.00	lobChIP_FOXA1inPBS
chr12	96028937	96028938	MACS_peak_3509	15.00	lobChIP_FOXA1inPBS
chr12	96131985	96131986	MACS_peak_3510	26.00	lobChIP_FOXA1inPBS
chr12	96156090	96156091	MACS_peak_3511	21.00	lobChIP_FOXA1inPBS
chr12	96160376	96160377	MACS_peak_3512	18.00	lobChIP_FOXA1inPBS
chr12	96196561	96196562	MACS_peak_3513	11.00	lobChIP_FOXA1inPBS
chr12	96334415	96334416	MACS_peak_3514	10.00	lobChIP_FOXA1inPBS
chr12	96343648	96343649	MACS_peak_3515	30.00	lobChIP_FOXA1inPBS
chr12	96349778	96349779	MACS_peak_3516	8.00	lobChIP_FOXA1inPBS
chr12	96360145	96360146	MACS_peak_3517	29.00	lobChIP_FOXA1inPBS
chr12	96368320	96368321	MACS_peak_3518	19.00	lobChIP_FOXA1inPBS
chr12	96390180	96390181	MACS_peak_3519	26.00	lobChIP_FOXA1inPBS
chr12	96392587	96392588	MACS_peak_3520	9.00	lobChIP_FOXA1inPBS
chr12	96442268	96442269	MACS_peak_3521	20.00	lobChIP_FOXA1inPBS
chr12	96506573	96506574	MACS_peak_3522	7.00	lobChIP_FOXA1inPBS
chr12	96588264	96588265	MACS_peak_3523	10.00	lobChIP_FOXA1inPBS
chr12	96648189	96648190	MACS_peak_3524	7.00	lobChIP_FOXA1inPBS
chr12	96657336	96657337	MACS_peak_3525	20.00	lobChIP_FOXA1inPBS
chr12	96696528	96696529	MACS_peak_3526	5.00	lobChIP_FOXA1inPBS
chr12	97064385	97064386	MACS_peak_3527	6.00	lobChIP_FOXA1inPBS
chr12	97113919	97113920	MACS_peak_3528	7.00	lobChIP_FOXA1inPBS
chr12	97296042	97296043	MACS_peak_3529	7.00	lobChIP_FOXA1inPBS
chr12	97300745	97300746	MACS_peak_3530	11.00	lobChIP_FOXA1inPBS
chr12	97774400	97774401	MACS_peak_3531	8.00	lobChIP_FOXA1inPBS
chr12	97798544	97798545	MACS_peak_3532	7.00	lobChIP_FOXA1inPBS
chr12	97868978	97868979	MACS_peak_3533	7.00	lobChIP_FOXA1inPBS
chr12	97962726	97962727	MACS_peak_3534	42.00	lobChIP_FOXA1inPBS
chr12	97989345	97989346	MACS_peak_3535	9.00	lobChIP_FOXA1inPBS
chr12	98335817	98335818	MACS_peak_3536	7.00	lobChIP_FOXA1inPBS
chr12	99047816	99047817	MACS_peak_3537	18.00	lobChIP_FOXA1inPBS
chr12	100535796	100535797	MACS_peak_3538	37.00	lobChIP_FOXA1inPBS
chr12	100578620	100578621	MACS_peak_3539	22.00	lobChIP_FOXA1inPBS
chr12	100607421	100607422	MACS_peak_3540	8.00	lobChIP_FOXA1inPBS
chr12	100831329	100831330	MACS_peak_3541	20.00	lobChIP_FOXA1inPBS
chr12	100858517	100858518	MACS_peak_3542	19.00	lobChIP_FOXA1inPBS
chr12	101853866	101853867	MACS_peak_3543	9.00	lobChIP_FOXA1inPBS
chr12	101993319	101993320	MACS_peak_3544	10.00	lobChIP_FOXA1inPBS
chr12	101994173	101994174	MACS_peak_3545	10.00	lobChIP_FOXA1inPBS
chr12	101998066	101998067	MACS_peak_3546	10.00	lobChIP_FOXA1inPBS
chr12	102002314	102002315	MACS_peak_3547	86.00	lobChIP_FOXA1inPBS
chr12	102010177	102010178	MACS_peak_3548	10.00	lobChIP_FOXA1inPBS
chr12	102012151	102012152	MACS_peak_3549	10.00	lobChIP_FOXA1inPBS
chr12	102104327	102104328	MACS_peak_3550	13.00	lobChIP_FOXA1inPBS
chr12	102285391	102285392	MACS_peak_3551	17.00	lobChIP_FOXA1inPBS
chr12	102303166	102303167	MACS_peak_3552	30.00	lobChIP_FOXA1inPBS
chr12	102453407	102453408	MACS_peak_3553	24.00	lobChIP_FOXA1inPBS
chr12	102455195	102455196	MACS_peak_3554	7.00	lobChIP_FOXA1inPBS
chr12	103270882	103270883	MACS_peak_3555	6.00	lobChIP_FOXA1inPBS
chr12	103292881	103292882	MACS_peak_3556	23.00	lobChIP_FOXA1inPBS
chr12	103297570	103297571	MACS_peak_3557	9.00	lobChIP_FOXA1inPBS
chr12	103314239	103314240	MACS_peak_3558	61.00	lobChIP_FOXA1inPBS
chr12	103315253	103315254	MACS_peak_3559	10.00	lobChIP_FOXA1inPBS
chr12	104066664	104066665	MACS_peak_3560	14.00	lobChIP_FOXA1inPBS
chr12	104199141	104199142	MACS_peak_3561	10.00	lobChIP_FOXA1inPBS
chr12	104232528	104232529	MACS_peak_3562	39.00	lobChIP_FOXA1inPBS
chr12	104242066	104242067	MACS_peak_3563	9.00	lobChIP_FOXA1inPBS
chr12	104249188	104249189	MACS_peak_3564	11.00	lobChIP_FOXA1inPBS
chr12	104264230	104264231	MACS_peak_3565	6.00	lobChIP_FOXA1inPBS
chr12	104450626	104450627	MACS_peak_3566	7.00	lobChIP_FOXA1inPBS
chr12	105290175	105290176	MACS_peak_3567	19.00	lobChIP_FOXA1inPBS
chr12	105495031	105495032	MACS_peak_3568	9.00	lobChIP_FOXA1inPBS
chr12	105711966	105711967	MACS_peak_3569	15.00	lobChIP_FOXA1inPBS
chr12	106492905	106492906	MACS_peak_3570	28.00	lobChIP_FOXA1inPBS
chr12	106514447	106514448	MACS_peak_3571	18.00	lobChIP_FOXA1inPBS
chr12	106668762	106668763	MACS_peak_3572	11.00	lobChIP_FOXA1inPBS
chr12	106675047	106675048	MACS_peak_3573	31.00	lobChIP_FOXA1inPBS
chr12	106676546	106676547	MACS_peak_3574	36.00	lobChIP_FOXA1inPBS
chr12	106678541	106678542	MACS_peak_3575	14.00	lobChIP_FOXA1inPBS
chr12	106682337	106682338	MACS_peak_3576	18.00	lobChIP_FOXA1inPBS
chr12	106685360	106685361	MACS_peak_3577	8.00	lobChIP_FOXA1inPBS
chr12	106753612	106753613	MACS_peak_3578	23.00	lobChIP_FOXA1inPBS
chr12	107249092	107249093	MACS_peak_3579	28.00	lobChIP_FOXA1inPBS
chr12	107368549	107368550	MACS_peak_3580	25.00	lobChIP_FOXA1inPBS
chr12	107370209	107370210	MACS_peak_3581	7.00	lobChIP_FOXA1inPBS
chr12	107743804	107743805	MACS_peak_3582	7.00	lobChIP_FOXA1inPBS
chr12	107746244	107746245	MACS_peak_3583	22.00	lobChIP_FOXA1inPBS
chr12	107749592	107749593	MACS_peak_3584	11.00	lobChIP_FOXA1inPBS
chr12	107758909	107758910	MACS_peak_3585	8.00	lobChIP_FOXA1inPBS
chr12	107774045	107774046	MACS_peak_3586	11.00	lobChIP_FOXA1inPBS
chr12	107900431	107900432	MACS_peak_3587	13.00	lobChIP_FOXA1inPBS
chr12	107909025	107909026	MACS_peak_3588	25.00	lobChIP_FOXA1inPBS
chr12	107918166	107918167	MACS_peak_3589	16.00	lobChIP_FOXA1inPBS
chr12	108025652	108025653	MACS_peak_3590	8.00	lobChIP_FOXA1inPBS
chr12	108183106	108183107	MACS_peak_3591	13.00	lobChIP_FOXA1inPBS
chr12	108707610	108707611	MACS_peak_3592	20.00	lobChIP_FOXA1inPBS
chr12	109077943	109077944	MACS_peak_3593	11.00	lobChIP_FOXA1inPBS
chr12	109085275	109085276	MACS_peak_3594	12.00	lobChIP_FOXA1inPBS
chr12	109345097	109345098	MACS_peak_3595	7.00	lobChIP_FOXA1inPBS
chr12	109636143	109636144	MACS_peak_3596	10.00	lobChIP_FOXA1inPBS
chr12	109715840	109715841	MACS_peak_3597	20.00	lobChIP_FOXA1inPBS
chr12	109745109	109745110	MACS_peak_3598	12.00	lobChIP_FOXA1inPBS
chr12	109899242	109899243	MACS_peak_3599	10.00	lobChIP_FOXA1inPBS
chr12	109932206	109932207	MACS_peak_3600	7.00	lobChIP_FOXA1inPBS
chr12	109990740	109990741	MACS_peak_3601	8.00	lobChIP_FOXA1inPBS
chr12	110024898	110024899	MACS_peak_3602	25.00	lobChIP_FOXA1inPBS
chr12	110026480	110026481	MACS_peak_3603	13.00	lobChIP_FOXA1inPBS
chr12	110044707	110044708	MACS_peak_3604	8.00	lobChIP_FOXA1inPBS
chr12	110134078	110134079	MACS_peak_3605	11.00	lobChIP_FOXA1inPBS
chr12	110159974	110159975	MACS_peak_3606	11.00	lobChIP_FOXA1inPBS
chr12	110302315	110302316	MACS_peak_3607	14.00	lobChIP_FOXA1inPBS
chr12	110477142	110477143	MACS_peak_3608	15.00	lobChIP_FOXA1inPBS
chr12	110517586	110517587	MACS_peak_3609	12.00	lobChIP_FOXA1inPBS
chr12	110529248	110529249	MACS_peak_3610	10.00	lobChIP_FOXA1inPBS
chr12	110547242	110547243	MACS_peak_3611	11.00	lobChIP_FOXA1inPBS
chr12	110549415	110549416	MACS_peak_3612	19.00	lobChIP_FOXA1inPBS
chr12	110556342	110556343	MACS_peak_3613	15.00	lobChIP_FOXA1inPBS
chr12	110638020	110638021	MACS_peak_3614	8.00	lobChIP_FOXA1inPBS
chr12	110662258	110662259	MACS_peak_3615	9.00	lobChIP_FOXA1inPBS
chr12	110685569	110685570	MACS_peak_3616	8.00	lobChIP_FOXA1inPBS
chr12	111004525	111004526	MACS_peak_3617	14.00	lobChIP_FOXA1inPBS
chr12	111029724	111029725	MACS_peak_3618	7.00	lobChIP_FOXA1inPBS
chr12	111169434	111169435	MACS_peak_3619	19.00	lobChIP_FOXA1inPBS
chr12	111181010	111181011	MACS_peak_3620	7.00	lobChIP_FOXA1inPBS
chr12	111205978	111205979	MACS_peak_3621	5.00	lobChIP_FOXA1inPBS
chr12	112016406	112016407	MACS_peak_3622	29.00	lobChIP_FOXA1inPBS
chr12	112025041	112025042	MACS_peak_3623	9.00	lobChIP_FOXA1inPBS
chr12	112211640	112211641	MACS_peak_3624	20.00	lobChIP_FOXA1inPBS
chr12	112213612	112213613	MACS_peak_3625	16.00	lobChIP_FOXA1inPBS
chr12	112215367	112215368	MACS_peak_3626	7.00	lobChIP_FOXA1inPBS
chr12	112225318	112225319	MACS_peak_3627	11.00	lobChIP_FOXA1inPBS
chr12	112723734	112723735	MACS_peak_3628	80.00	lobChIP_FOXA1inPBS
chr12	112904103	112904104	MACS_peak_3629	26.00	lobChIP_FOXA1inPBS
chr12	113660995	113660996	MACS_peak_3630	14.00	lobChIP_FOXA1inPBS
chr12	113668378	113668379	MACS_peak_3631	5.00	lobChIP_FOXA1inPBS
chr12	114204069	114204070	MACS_peak_3632	15.00	lobChIP_FOXA1inPBS
chr12	114208489	114208490	MACS_peak_3633	10.00	lobChIP_FOXA1inPBS
chr12	114219746	114219747	MACS_peak_3634	5.00	lobChIP_FOXA1inPBS
chr12	115046459	115046460	MACS_peak_3635	16.00	lobChIP_FOXA1inPBS
chr12	115049461	115049462	MACS_peak_3636	34.00	lobChIP_FOXA1inPBS
chr12	115094138	115094139	MACS_peak_3637	43.00	lobChIP_FOXA1inPBS
chr12	115095130	115095131	MACS_peak_3638	22.00	lobChIP_FOXA1inPBS
chr12	115095911	115095912	MACS_peak_3639	10.00	lobChIP_FOXA1inPBS
chr12	115108541	115108542	MACS_peak_3640	8.00	lobChIP_FOXA1inPBS
chr12	115122190	115122191	MACS_peak_3641	13.00	lobChIP_FOXA1inPBS
chr12	115141390	115141391	MACS_peak_3642	7.00	lobChIP_FOXA1inPBS
chr12	115251166	115251167	MACS_peak_3643	29.00	lobChIP_FOXA1inPBS
chr12	115832820	115832821	MACS_peak_3644	7.00	lobChIP_FOXA1inPBS
chr12	115836300	115836301	MACS_peak_3645	7.00	lobChIP_FOXA1inPBS
chr12	115859831	115859832	MACS_peak_3646	55.00	lobChIP_FOXA1inPBS
chr12	115861565	115861566	MACS_peak_3647	34.00	lobChIP_FOXA1inPBS
chr12	115864756	115864757	MACS_peak_3648	22.00	lobChIP_FOXA1inPBS
chr12	115871222	115871223	MACS_peak_3649	11.00	lobChIP_FOXA1inPBS
chr12	116086497	116086498	MACS_peak_3650	8.00	lobChIP_FOXA1inPBS
chr12	116571094	116571095	MACS_peak_3651	30.00	lobChIP_FOXA1inPBS
chr12	116593399	116593400	MACS_peak_3652	8.00	lobChIP_FOXA1inPBS
chr12	116627745	116627746	MACS_peak_3653	10.00	lobChIP_FOXA1inPBS
chr12	116674121	116674122	MACS_peak_3654	8.00	lobChIP_FOXA1inPBS
chr12	116713669	116713670	MACS_peak_3655	9.00	lobChIP_FOXA1inPBS
chr12	116714317	116714318	MACS_peak_3656	9.00	lobChIP_FOXA1inPBS
chr12	116814364	116814365	MACS_peak_3657	7.00	lobChIP_FOXA1inPBS
chr12	116967022	116967023	MACS_peak_3658	34.00	lobChIP_FOXA1inPBS
chr12	117073892	117073893	MACS_peak_3659	16.00	lobChIP_FOXA1inPBS
chr12	117074908	117074909	MACS_peak_3660	95.00	lobChIP_FOXA1inPBS
chr12	117096717	117096718	MACS_peak_3661	10.00	lobChIP_FOXA1inPBS
chr12	117172386	117172387	MACS_peak_3662	26.00	lobChIP_FOXA1inPBS
chr12	117174203	117174204	MACS_peak_3663	8.00	lobChIP_FOXA1inPBS
chr12	117327166	117327167	MACS_peak_3664	8.00	lobChIP_FOXA1inPBS
chr12	117354761	117354762	MACS_peak_3665	8.00	lobChIP_FOXA1inPBS
chr12	117486160	117486161	MACS_peak_3666	15.00	lobChIP_FOXA1inPBS
chr12	117581168	117581169	MACS_peak_3667	9.00	lobChIP_FOXA1inPBS
chr12	117945535	117945536	MACS_peak_3668	6.00	lobChIP_FOXA1inPBS
chr12	117956827	117956828	MACS_peak_3669	9.00	lobChIP_FOXA1inPBS
chr12	118291555	118291556	MACS_peak_3670	8.00	lobChIP_FOXA1inPBS
chr12	118294583	118294584	MACS_peak_3671	9.00	lobChIP_FOXA1inPBS
chr12	118562462	118562463	MACS_peak_3672	15.00	lobChIP_FOXA1inPBS
chr12	119041643	119041644	MACS_peak_3673	20.00	lobChIP_FOXA1inPBS
chr12	119152450	119152451	MACS_peak_3674	41.00	lobChIP_FOXA1inPBS
chr12	119278491	119278492	MACS_peak_3675	8.00	lobChIP_FOXA1inPBS
chr12	120051198	120051199	MACS_peak_3676	7.00	lobChIP_FOXA1inPBS
chr12	120108078	120108079	MACS_peak_3677	9.00	lobChIP_FOXA1inPBS
chr12	120154585	120154586	MACS_peak_3678	13.00	lobChIP_FOXA1inPBS
chr12	120378890	120378891	MACS_peak_3679	17.00	lobChIP_FOXA1inPBS
chr12	120479334	120479335	MACS_peak_3680	10.00	lobChIP_FOXA1inPBS
chr12	120493585	120493586	MACS_peak_3681	7.00	lobChIP_FOXA1inPBS
chr12	120559912	120559913	MACS_peak_3682	9.00	lobChIP_FOXA1inPBS
chr12	120665648	120665649	MACS_peak_3683	16.00	lobChIP_FOXA1inPBS
chr12	120730674	120730675	MACS_peak_3684	17.00	lobChIP_FOXA1inPBS
chr12	120740386	120740387	MACS_peak_3685	12.00	lobChIP_FOXA1inPBS
chr12	120755629	120755630	MACS_peak_3686	20.00	lobChIP_FOXA1inPBS
chr12	120759340	120759341	MACS_peak_3687	7.00	lobChIP_FOXA1inPBS
chr12	120763414	120763415	MACS_peak_3688	21.00	lobChIP_FOXA1inPBS
chr12	120983775	120983776	MACS_peak_3689	9.00	lobChIP_FOXA1inPBS
chr12	121190362	121190363	MACS_peak_3690	5.00	lobChIP_FOXA1inPBS
chr12	121263311	121263312	MACS_peak_3691	35.00	lobChIP_FOXA1inPBS
chr12	121410894	121410895	MACS_peak_3692	11.00	lobChIP_FOXA1inPBS
chr12	121416363	121416364	MACS_peak_3693	14.00	lobChIP_FOXA1inPBS
chr12	121418773	121418774	MACS_peak_3694	11.00	lobChIP_FOXA1inPBS
chr12	121419612	121419613	MACS_peak_3695	13.00	lobChIP_FOXA1inPBS
chr12	121421749	121421750	MACS_peak_3696	9.00	lobChIP_FOXA1inPBS
chr12	121463554	121463555	MACS_peak_3697	11.00	lobChIP_FOXA1inPBS
chr12	121533254	121533255	MACS_peak_3698	12.00	lobChIP_FOXA1inPBS
chr12	121676509	121676510	MACS_peak_3699	8.00	lobChIP_FOXA1inPBS
chr12	121814991	121814992	MACS_peak_3700	13.00	lobChIP_FOXA1inPBS
chr12	121989111	121989112	MACS_peak_3701	61.00	lobChIP_FOXA1inPBS
chr12	121995818	121995819	MACS_peak_3702	17.00	lobChIP_FOXA1inPBS
chr12	122020821	122020822	MACS_peak_3703	9.00	lobChIP_FOXA1inPBS
chr12	122036792	122036793	MACS_peak_3704	16.00	lobChIP_FOXA1inPBS
chr12	122071291	122071292	MACS_peak_3705	15.00	lobChIP_FOXA1inPBS
chr12	122298292	122298293	MACS_peak_3706	16.00	lobChIP_FOXA1inPBS
chr12	122391784	122391785	MACS_peak_3707	13.00	lobChIP_FOXA1inPBS
chr12	122501086	122501087	MACS_peak_3708	25.00	lobChIP_FOXA1inPBS
chr12	122662096	122662097	MACS_peak_3709	5.00	lobChIP_FOXA1inPBS
chr12	122853188	122853189	MACS_peak_3710	14.00	lobChIP_FOXA1inPBS
chr12	122885277	122885278	MACS_peak_3711	31.00	lobChIP_FOXA1inPBS
chr12	122890688	122890689	MACS_peak_3712	18.00	lobChIP_FOXA1inPBS
chr12	122909716	122909717	MACS_peak_3713	6.00	lobChIP_FOXA1inPBS
chr12	122916145	122916146	MACS_peak_3714	10.00	lobChIP_FOXA1inPBS
chr12	122956133	122956134	MACS_peak_3715	20.00	lobChIP_FOXA1inPBS
chr12	122973732	122973733	MACS_peak_3716	27.00	lobChIP_FOXA1inPBS
chr12	122976766	122976767	MACS_peak_3717	12.00	lobChIP_FOXA1inPBS
chr12	123009234	123009235	MACS_peak_3718	8.00	lobChIP_FOXA1inPBS
chr12	123163881	123163882	MACS_peak_3719	13.00	lobChIP_FOXA1inPBS
chr12	123175674	123175675	MACS_peak_3720	7.00	lobChIP_FOXA1inPBS
chr12	123201474	123201475	MACS_peak_3721	10.00	lobChIP_FOXA1inPBS
chr12	123244773	123244774	MACS_peak_3722	6.00	lobChIP_FOXA1inPBS
chr12	123342078	123342079	MACS_peak_3723	6.00	lobChIP_FOXA1inPBS
chr12	123383586	123383587	MACS_peak_3724	14.00	lobChIP_FOXA1inPBS
chr12	123402279	123402280	MACS_peak_3725	10.00	lobChIP_FOXA1inPBS
chr12	123576966	123576967	MACS_peak_3726	13.00	lobChIP_FOXA1inPBS
chr12	123754793	123754794	MACS_peak_3727	27.00	lobChIP_FOXA1inPBS
chr12	123778220	123778221	MACS_peak_3728	6.00	lobChIP_FOXA1inPBS
chr12	123933248	123933249	MACS_peak_3729	19.00	lobChIP_FOXA1inPBS
chr12	123942675	123942676	MACS_peak_3730	15.00	lobChIP_FOXA1inPBS
chr12	124080522	124080523	MACS_peak_3731	9.00	lobChIP_FOXA1inPBS
chr12	124351898	124351899	MACS_peak_3732	17.00	lobChIP_FOXA1inPBS
chr12	124615417	124615418	MACS_peak_3733	43.00	lobChIP_FOXA1inPBS
chr12	124716727	124716728	MACS_peak_3734	10.00	lobChIP_FOXA1inPBS
chr12	124763243	124763244	MACS_peak_3735	30.00	lobChIP_FOXA1inPBS
chr12	124994882	124994883	MACS_peak_3736	9.00	lobChIP_FOXA1inPBS
chr12	125165623	125165624	MACS_peak_3737	9.00	lobChIP_FOXA1inPBS
chr12	125262095	125262096	MACS_peak_3738	17.00	lobChIP_FOXA1inPBS
chr12	125379890	125379891	MACS_peak_3739	9.00	lobChIP_FOXA1inPBS
chr12	125391407	125391408	MACS_peak_3740	28.00	lobChIP_FOXA1inPBS
chr12	125406839	125406840	MACS_peak_3741	7.00	lobChIP_FOXA1inPBS
chr12	126280768	126280769	MACS_peak_3742	12.00	lobChIP_FOXA1inPBS
chr12	126466297	126466298	MACS_peak_3743	11.00	lobChIP_FOXA1inPBS
chr12	129389839	129389840	MACS_peak_3745	8.00	lobChIP_FOXA1inPBS
chr12	130528385	130528386	MACS_peak_3746	20.00	lobChIP_FOXA1inPBS
chr12	130529760	130529761	MACS_peak_3747	10.00	lobChIP_FOXA1inPBS
chr12	131348007	131348008	MACS_peak_3748	12.00	lobChIP_FOXA1inPBS
chr12	131497040	131497041	MACS_peak_3749	18.00	lobChIP_FOXA1inPBS
chr12	132421560	132421561	MACS_peak_3750	7.00	lobChIP_FOXA1inPBS
chr12	133047834	133047835	MACS_peak_3751	10.00	lobChIP_FOXA1inPBS
chr12	133067994	133067995	MACS_peak_3752	11.00	lobChIP_FOXA1inPBS
chr12	133443167	133443168	MACS_peak_3753	7.00	lobChIP_FOXA1inPBS
chr13	20171258	20171259	MACS_peak_3754	47.00	lobChIP_FOXA1inPBS
chr13	20178598	20178599	MACS_peak_3755	36.00	lobChIP_FOXA1inPBS
chr13	20690562	20690563	MACS_peak_3756	17.00	lobChIP_FOXA1inPBS
chr13	20694663	20694664	MACS_peak_3757	7.00	lobChIP_FOXA1inPBS
chr13	20737838	20737839	MACS_peak_3758	18.00	lobChIP_FOXA1inPBS
chr13	20791480	20791481	MACS_peak_3759	7.00	lobChIP_FOXA1inPBS
chr13	20862332	20862333	MACS_peak_3760	36.00	lobChIP_FOXA1inPBS
chr13	21080085	21080086	MACS_peak_3761	16.00	lobChIP_FOXA1inPBS
chr13	21108654	21108655	MACS_peak_3762	43.00	lobChIP_FOXA1inPBS
chr13	21363019	21363020	MACS_peak_3763	7.00	lobChIP_FOXA1inPBS
chr13	21495890	21495891	MACS_peak_3764	9.00	lobChIP_FOXA1inPBS
chr13	21514543	21514544	MACS_peak_3765	17.00	lobChIP_FOXA1inPBS
chr13	21524742	21524743	MACS_peak_3766	11.00	lobChIP_FOXA1inPBS
chr13	21527774	21527775	MACS_peak_3767	49.00	lobChIP_FOXA1inPBS
chr13	21675341	21675342	MACS_peak_3768	12.00	lobChIP_FOXA1inPBS
chr13	21777506	21777507	MACS_peak_3769	17.00	lobChIP_FOXA1inPBS
chr13	21799344	21799345	MACS_peak_3770	8.00	lobChIP_FOXA1inPBS
chr13	21856470	21856471	MACS_peak_3771	12.00	lobChIP_FOXA1inPBS
chr13	21977213	21977214	MACS_peak_3772	10.00	lobChIP_FOXA1inPBS
chr13	22040043	22040044	MACS_peak_3773	6.00	lobChIP_FOXA1inPBS
chr13	22101099	22101100	MACS_peak_3774	36.00	lobChIP_FOXA1inPBS
chr13	22132290	22132291	MACS_peak_3775	11.00	lobChIP_FOXA1inPBS
chr13	22165403	22165404	MACS_peak_3776	10.00	lobChIP_FOXA1inPBS
chr13	22237649	22237650	MACS_peak_3777	11.00	lobChIP_FOXA1inPBS
chr13	22271182	22271183	MACS_peak_3778	43.00	lobChIP_FOXA1inPBS
chr13	22327449	22327450	MACS_peak_3779	7.00	lobChIP_FOXA1inPBS
chr13	22412143	22412144	MACS_peak_3780	21.00	lobChIP_FOXA1inPBS
chr13	23042417	23042418	MACS_peak_3781	13.00	lobChIP_FOXA1inPBS
chr13	23363460	23363461	MACS_peak_3782	8.00	lobChIP_FOXA1inPBS
chr13	23368120	23368121	MACS_peak_3783	8.00	lobChIP_FOXA1inPBS
chr13	23948035	23948036	MACS_peak_3784	16.00	lobChIP_FOXA1inPBS
chr13	24045755	24045756	MACS_peak_3785	9.00	lobChIP_FOXA1inPBS
chr13	24126477	24126478	MACS_peak_3786	26.00	lobChIP_FOXA1inPBS
chr13	24133422	24133423	MACS_peak_3787	15.00	lobChIP_FOXA1inPBS
chr13	24144689	24144690	MACS_peak_3788	11.00	lobChIP_FOXA1inPBS
chr13	24145245	24145246	MACS_peak_3789	8.00	lobChIP_FOXA1inPBS
chr13	24173778	24173779	MACS_peak_3790	31.00	lobChIP_FOXA1inPBS
chr13	24184480	24184481	MACS_peak_3791	8.00	lobChIP_FOXA1inPBS
chr13	24203789	24203790	MACS_peak_3792	47.00	lobChIP_FOXA1inPBS
chr13	24205008	24205009	MACS_peak_3793	27.00	lobChIP_FOXA1inPBS
chr13	24231004	24231005	MACS_peak_3794	17.00	lobChIP_FOXA1inPBS
chr13	24309655	24309656	MACS_peak_3795	8.00	lobChIP_FOXA1inPBS
chr13	24318107	24318108	MACS_peak_3796	14.00	lobChIP_FOXA1inPBS
chr13	24327736	24327737	MACS_peak_3797	8.00	lobChIP_FOXA1inPBS
chr13	24329445	24329446	MACS_peak_3798	12.00	lobChIP_FOXA1inPBS
chr13	24353274	24353275	MACS_peak_3799	13.00	lobChIP_FOXA1inPBS
chr13	24370878	24370879	MACS_peak_3800	20.00	lobChIP_FOXA1inPBS
chr13	24544051	24544052	MACS_peak_3801	41.00	lobChIP_FOXA1inPBS
chr13	24660066	24660067	MACS_peak_3802	9.00	lobChIP_FOXA1inPBS
chr13	24757045	24757046	MACS_peak_3803	12.00	lobChIP_FOXA1inPBS
chr13	24758655	24758656	MACS_peak_3804	6.00	lobChIP_FOXA1inPBS
chr13	24795398	24795399	MACS_peak_3805	9.00	lobChIP_FOXA1inPBS
chr13	24881618	24881619	MACS_peak_3806	12.00	lobChIP_FOXA1inPBS
chr13	24993982	24993983	MACS_peak_3807	16.00	lobChIP_FOXA1inPBS
chr13	25268410	25268411	MACS_peak_3808	14.00	lobChIP_FOXA1inPBS
chr13	25407350	25407351	MACS_peak_3809	6.00	lobChIP_FOXA1inPBS
chr13	25499937	25499938	MACS_peak_3810	13.00	lobChIP_FOXA1inPBS
chr13	25502127	25502128	MACS_peak_3811	11.00	lobChIP_FOXA1inPBS
chr13	25828043	25828044	MACS_peak_3812	11.00	lobChIP_FOXA1inPBS
chr13	25911651	25911652	MACS_peak_3813	8.00	lobChIP_FOXA1inPBS
chr13	26301514	26301515	MACS_peak_3814	7.00	lobChIP_FOXA1inPBS
chr13	27118996	27118997	MACS_peak_3815	6.00	lobChIP_FOXA1inPBS
chr13	27133448	27133449	MACS_peak_3816	9.00	lobChIP_FOXA1inPBS
chr13	27536238	27536239	MACS_peak_3817	10.00	lobChIP_FOXA1inPBS
chr13	27557412	27557413	MACS_peak_3818	9.00	lobChIP_FOXA1inPBS
chr13	27565841	27565842	MACS_peak_3819	12.00	lobChIP_FOXA1inPBS
chr13	27597027	27597028	MACS_peak_3820	9.00	lobChIP_FOXA1inPBS
chr13	27941819	27941820	MACS_peak_3821	16.00	lobChIP_FOXA1inPBS
chr13	27947228	27947229	MACS_peak_3822	12.00	lobChIP_FOXA1inPBS
chr13	27978451	27978452	MACS_peak_3823	16.00	lobChIP_FOXA1inPBS
chr13	28198419	28198420	MACS_peak_3824	13.00	lobChIP_FOXA1inPBS
chr13	28211561	28211562	MACS_peak_3825	13.00	lobChIP_FOXA1inPBS
chr13	28284135	28284136	MACS_peak_3826	12.00	lobChIP_FOXA1inPBS
chr13	28535069	28535070	MACS_peak_3827	56.00	lobChIP_FOXA1inPBS
chr13	28719367	28719368	MACS_peak_3828	70.00	lobChIP_FOXA1inPBS
chr13	29212251	29212252	MACS_peak_3829	14.00	lobChIP_FOXA1inPBS
chr13	29267991	29267992	MACS_peak_3830	14.00	lobChIP_FOXA1inPBS
chr13	30146051	30146052	MACS_peak_3831	9.00	lobChIP_FOXA1inPBS
chr13	30384578	30384579	MACS_peak_3832	7.00	lobChIP_FOXA1inPBS
chr13	30494333	30494334	MACS_peak_3833	8.00	lobChIP_FOXA1inPBS
chr13	30498417	30498418	MACS_peak_3834	9.00	lobChIP_FOXA1inPBS
chr13	30927737	30927738	MACS_peak_3835	7.00	lobChIP_FOXA1inPBS
chr13	30969916	30969917	MACS_peak_3836	21.00	lobChIP_FOXA1inPBS
chr13	31002931	31002932	MACS_peak_3837	8.00	lobChIP_FOXA1inPBS
chr13	31038488	31038489	MACS_peak_3838	7.00	lobChIP_FOXA1inPBS
chr13	31425114	31425115	MACS_peak_3839	11.00	lobChIP_FOXA1inPBS
chr13	31746923	31746924	MACS_peak_3840	7.00	lobChIP_FOXA1inPBS
chr13	32497668	32497669	MACS_peak_3841	14.00	lobChIP_FOXA1inPBS
chr13	32614258	32614259	MACS_peak_3842	9.00	lobChIP_FOXA1inPBS
chr13	32634010	32634011	MACS_peak_3843	9.00	lobChIP_FOXA1inPBS
chr13	32649720	32649721	MACS_peak_3844	10.00	lobChIP_FOXA1inPBS
chr13	32654597	32654598	MACS_peak_3845	24.00	lobChIP_FOXA1inPBS
chr13	32662246	32662247	MACS_peak_3846	24.00	lobChIP_FOXA1inPBS
chr13	32713654	32713655	MACS_peak_3847	15.00	lobChIP_FOXA1inPBS
chr13	32714429	32714430	MACS_peak_3848	8.00	lobChIP_FOXA1inPBS
chr13	32717222	32717223	MACS_peak_3849	8.00	lobChIP_FOXA1inPBS
chr13	32769914	32769915	MACS_peak_3850	12.00	lobChIP_FOXA1inPBS
chr13	32822178	32822179	MACS_peak_3851	8.00	lobChIP_FOXA1inPBS
chr13	32830021	32830022	MACS_peak_3852	6.00	lobChIP_FOXA1inPBS
chr13	32835317	32835318	MACS_peak_3853	9.00	lobChIP_FOXA1inPBS
chr13	33170495	33170496	MACS_peak_3854	7.00	lobChIP_FOXA1inPBS
chr13	33237940	33237941	MACS_peak_3855	19.00	lobChIP_FOXA1inPBS
chr13	33413281	33413282	MACS_peak_3856	7.00	lobChIP_FOXA1inPBS
chr13	33581612	33581613	MACS_peak_3857	8.00	lobChIP_FOXA1inPBS
chr13	33665580	33665581	MACS_peak_3858	8.00	lobChIP_FOXA1inPBS
chr13	33676164	33676165	MACS_peak_3859	18.00	lobChIP_FOXA1inPBS
chr13	33685779	33685780	MACS_peak_3860	9.00	lobChIP_FOXA1inPBS
chr13	33742655	33742656	MACS_peak_3861	18.00	lobChIP_FOXA1inPBS
chr13	33802986	33802987	MACS_peak_3862	9.00	lobChIP_FOXA1inPBS
chr13	33804814	33804815	MACS_peak_3863	8.00	lobChIP_FOXA1inPBS
chr13	33833164	33833165	MACS_peak_3864	7.00	lobChIP_FOXA1inPBS
chr13	33834657	33834658	MACS_peak_3865	27.00	lobChIP_FOXA1inPBS
chr13	33992666	33992667	MACS_peak_3866	6.00	lobChIP_FOXA1inPBS
chr13	34019390	34019391	MACS_peak_3867	23.00	lobChIP_FOXA1inPBS
chr13	34036473	34036474	MACS_peak_3868	11.00	lobChIP_FOXA1inPBS
chr13	34111299	34111300	MACS_peak_3869	11.00	lobChIP_FOXA1inPBS
chr13	34217013	34217014	MACS_peak_3870	52.00	lobChIP_FOXA1inPBS
chr13	34228349	34228350	MACS_peak_3871	10.00	lobChIP_FOXA1inPBS
chr13	34236958	34236959	MACS_peak_3872	6.00	lobChIP_FOXA1inPBS
chr13	34253598	34253599	MACS_peak_3873	6.00	lobChIP_FOXA1inPBS
chr13	34340641	34340642	MACS_peak_3874	8.00	lobChIP_FOXA1inPBS
chr13	34531786	34531787	MACS_peak_3875	9.00	lobChIP_FOXA1inPBS
chr13	34543167	34543168	MACS_peak_3876	22.00	lobChIP_FOXA1inPBS
chr13	34567413	34567414	MACS_peak_3877	31.00	lobChIP_FOXA1inPBS
chr13	34586125	34586126	MACS_peak_3878	31.00	lobChIP_FOXA1inPBS
chr13	34612774	34612775	MACS_peak_3879	40.00	lobChIP_FOXA1inPBS
chr13	35858164	35858165	MACS_peak_3880	27.00	lobChIP_FOXA1inPBS
chr13	35982671	35982672	MACS_peak_3881	16.00	lobChIP_FOXA1inPBS
chr13	36538995	36538996	MACS_peak_3882	19.00	lobChIP_FOXA1inPBS
chr13	36920072	36920073	MACS_peak_3883	10.00	lobChIP_FOXA1inPBS
chr13	36930325	36930326	MACS_peak_3884	15.00	lobChIP_FOXA1inPBS
chr13	37457260	37457261	MACS_peak_3885	26.00	lobChIP_FOXA1inPBS
chr13	37493566	37493567	MACS_peak_3886	11.00	lobChIP_FOXA1inPBS
chr13	37497799	37497800	MACS_peak_3887	7.00	lobChIP_FOXA1inPBS
chr13	40513266	40513267	MACS_peak_3888	6.00	lobChIP_FOXA1inPBS
chr13	41052651	41052652	MACS_peak_3889	8.00	lobChIP_FOXA1inPBS
chr13	41078299	41078300	MACS_peak_3890	23.00	lobChIP_FOXA1inPBS
chr13	41110878	41110879	MACS_peak_3891	9.00	lobChIP_FOXA1inPBS
chr13	41114743	41114744	MACS_peak_3892	21.00	lobChIP_FOXA1inPBS
chr13	41196560	41196561	MACS_peak_3893	9.00	lobChIP_FOXA1inPBS
chr13	41230733	41230734	MACS_peak_3894	11.00	lobChIP_FOXA1inPBS
chr13	41360841	41360842	MACS_peak_3895	16.00	lobChIP_FOXA1inPBS
chr13	41455138	41455139	MACS_peak_3896	16.00	lobChIP_FOXA1inPBS
chr13	41546105	41546106	MACS_peak_3897	8.00	lobChIP_FOXA1inPBS
chr13	41578304	41578305	MACS_peak_3898	9.00	lobChIP_FOXA1inPBS
chr13	41582083	41582084	MACS_peak_3899	13.00	lobChIP_FOXA1inPBS
chr13	41582692	41582693	MACS_peak_3900	20.00	lobChIP_FOXA1inPBS
chr13	41588603	41588604	MACS_peak_3901	8.00	lobChIP_FOXA1inPBS
chr13	41591970	41591971	MACS_peak_3902	22.00	lobChIP_FOXA1inPBS
chr13	41599805	41599806	MACS_peak_3903	11.00	lobChIP_FOXA1inPBS
chr13	41834213	41834214	MACS_peak_3904	24.00	lobChIP_FOXA1inPBS
chr13	41863849	41863850	MACS_peak_3905	8.00	lobChIP_FOXA1inPBS
chr13	42163143	42163144	MACS_peak_3906	7.00	lobChIP_FOXA1inPBS
chr13	42181008	42181009	MACS_peak_3907	6.00	lobChIP_FOXA1inPBS
chr13	42222580	42222581	MACS_peak_3908	76.00	lobChIP_FOXA1inPBS
chr13	42508612	42508613	MACS_peak_3909	14.00	lobChIP_FOXA1inPBS
chr13	42519339	42519340	MACS_peak_3910	44.00	lobChIP_FOXA1inPBS
chr13	42807974	42807975	MACS_peak_3911	7.00	lobChIP_FOXA1inPBS
chr13	42872656	42872657	MACS_peak_3912	7.00	lobChIP_FOXA1inPBS
chr13	43127969	43127970	MACS_peak_3913	12.00	lobChIP_FOXA1inPBS
chr13	43579892	43579893	MACS_peak_3914	8.00	lobChIP_FOXA1inPBS
chr13	44378898	44378899	MACS_peak_3915	7.00	lobChIP_FOXA1inPBS
chr13	44938928	44938929	MACS_peak_3916	9.00	lobChIP_FOXA1inPBS
chr13	44978548	44978549	MACS_peak_3917	31.00	lobChIP_FOXA1inPBS
chr13	45022762	45022763	MACS_peak_3918	23.00	lobChIP_FOXA1inPBS
chr13	45140923	45140924	MACS_peak_3919	9.00	lobChIP_FOXA1inPBS
chr13	45306900	45306901	MACS_peak_3920	12.00	lobChIP_FOXA1inPBS
chr13	45358538	45358539	MACS_peak_3921	9.00	lobChIP_FOXA1inPBS
chr13	45390005	45390006	MACS_peak_3922	11.00	lobChIP_FOXA1inPBS
chr13	45392535	45392536	MACS_peak_3923	11.00	lobChIP_FOXA1inPBS
chr13	45427836	45427837	MACS_peak_3924	15.00	lobChIP_FOXA1inPBS
chr13	45716906	45716907	MACS_peak_3925	6.00	lobChIP_FOXA1inPBS
chr13	45753773	45753774	MACS_peak_3926	17.00	lobChIP_FOXA1inPBS
chr13	45991172	45991173	MACS_peak_3927	6.00	lobChIP_FOXA1inPBS
chr13	46008958	46008959	MACS_peak_3928	26.00	lobChIP_FOXA1inPBS
chr13	46015063	46015064	MACS_peak_3929	16.00	lobChIP_FOXA1inPBS
chr13	46021490	46021491	MACS_peak_3930	7.00	lobChIP_FOXA1inPBS
chr13	46038840	46038841	MACS_peak_3931	12.00	lobChIP_FOXA1inPBS
chr13	46100716	46100717	MACS_peak_3932	12.00	lobChIP_FOXA1inPBS
chr13	46102242	46102243	MACS_peak_3933	19.00	lobChIP_FOXA1inPBS
chr13	46305776	46305777	MACS_peak_3934	10.00	lobChIP_FOXA1inPBS
chr13	46381857	46381858	MACS_peak_3935	14.00	lobChIP_FOXA1inPBS
chr13	46679234	46679235	MACS_peak_3936	18.00	lobChIP_FOXA1inPBS
chr13	46717441	46717442	MACS_peak_3937	46.00	lobChIP_FOXA1inPBS
chr13	47190261	47190262	MACS_peak_3938	9.00	lobChIP_FOXA1inPBS
chr13	47234011	47234012	MACS_peak_3939	10.00	lobChIP_FOXA1inPBS
chr13	47240320	47240321	MACS_peak_3940	38.00	lobChIP_FOXA1inPBS
chr13	47262039	47262040	MACS_peak_3941	6.00	lobChIP_FOXA1inPBS
chr13	47327467	47327468	MACS_peak_3942	7.00	lobChIP_FOXA1inPBS
chr13	47372752	47372753	MACS_peak_3943	5.00	lobChIP_FOXA1inPBS
chr13	47373487	47373488	MACS_peak_3944	7.00	lobChIP_FOXA1inPBS
chr13	48482528	48482529	MACS_peak_3945	12.00	lobChIP_FOXA1inPBS
chr13	48507507	48507508	MACS_peak_3946	9.00	lobChIP_FOXA1inPBS
chr13	48540337	48540338	MACS_peak_3947	9.00	lobChIP_FOXA1inPBS
chr13	48670749	48670750	MACS_peak_3948	12.00	lobChIP_FOXA1inPBS
chr13	48674740	48674741	MACS_peak_3949	10.00	lobChIP_FOXA1inPBS
chr13	48775373	48775374	MACS_peak_3950	14.00	lobChIP_FOXA1inPBS
chr13	48828199	48828200	MACS_peak_3951	7.00	lobChIP_FOXA1inPBS
chr13	48987332	48987333	MACS_peak_3952	8.00	lobChIP_FOXA1inPBS
chr13	49355494	49355495	MACS_peak_3953	16.00	lobChIP_FOXA1inPBS
chr13	50031188	50031189	MACS_peak_3954	7.00	lobChIP_FOXA1inPBS
chr13	50039352	50039353	MACS_peak_3955	29.00	lobChIP_FOXA1inPBS
chr13	50134614	50134615	MACS_peak_3956	19.00	lobChIP_FOXA1inPBS
chr13	50161824	50161825	MACS_peak_3957	22.00	lobChIP_FOXA1inPBS
chr13	50264463	50264464	MACS_peak_3958	10.00	lobChIP_FOXA1inPBS
chr13	50315134	50315135	MACS_peak_3959	8.00	lobChIP_FOXA1inPBS
chr13	50381984	50381985	MACS_peak_3960	11.00	lobChIP_FOXA1inPBS
chr13	50385179	50385180	MACS_peak_3961	20.00	lobChIP_FOXA1inPBS
chr13	50430286	50430287	MACS_peak_3962	14.00	lobChIP_FOXA1inPBS
chr13	50432839	50432840	MACS_peak_3963	72.00	lobChIP_FOXA1inPBS
chr13	50443933	50443934	MACS_peak_3964	10.00	lobChIP_FOXA1inPBS
chr13	50699827	50699828	MACS_peak_3965	9.00	lobChIP_FOXA1inPBS
chr13	50700632	50700633	MACS_peak_3966	15.00	lobChIP_FOXA1inPBS
chr13	50761710	50761711	MACS_peak_3967	15.00	lobChIP_FOXA1inPBS
chr13	50815859	50815860	MACS_peak_3968	15.00	lobChIP_FOXA1inPBS
chr13	50819352	50819353	MACS_peak_3969	21.00	lobChIP_FOXA1inPBS
chr13	50857367	50857368	MACS_peak_3970	17.00	lobChIP_FOXA1inPBS
chr13	50894651	50894652	MACS_peak_3971	9.00	lobChIP_FOXA1inPBS
chr13	50908401	50908402	MACS_peak_3972	7.00	lobChIP_FOXA1inPBS
chr13	50940515	50940516	MACS_peak_3973	15.00	lobChIP_FOXA1inPBS
chr13	50977692	50977693	MACS_peak_3974	10.00	lobChIP_FOXA1inPBS
chr13	51096027	51096028	MACS_peak_3975	16.00	lobChIP_FOXA1inPBS
chr13	51246348	51246349	MACS_peak_3976	12.00	lobChIP_FOXA1inPBS
chr13	51485378	51485379	MACS_peak_3977	10.00	lobChIP_FOXA1inPBS
chr13	51514861	51514862	MACS_peak_3978	18.00	lobChIP_FOXA1inPBS
chr13	51580353	51580354	MACS_peak_3979	10.00	lobChIP_FOXA1inPBS
chr13	51697146	51697147	MACS_peak_3980	11.00	lobChIP_FOXA1inPBS
chr13	51971676	51971677	MACS_peak_3981	8.00	lobChIP_FOXA1inPBS
chr13	52051555	52051556	MACS_peak_3982	11.00	lobChIP_FOXA1inPBS
chr13	52098646	52098647	MACS_peak_3983	14.00	lobChIP_FOXA1inPBS
chr13	52210728	52210729	MACS_peak_3984	20.00	lobChIP_FOXA1inPBS
chr13	52239553	52239554	MACS_peak_3985	6.00	lobChIP_FOXA1inPBS
chr13	52242986	52242987	MACS_peak_3986	8.00	lobChIP_FOXA1inPBS
chr13	52263923	52263924	MACS_peak_3987	16.00	lobChIP_FOXA1inPBS
chr13	52270034	52270035	MACS_peak_3988	9.00	lobChIP_FOXA1inPBS
chr13	52322278	52322279	MACS_peak_3989	8.00	lobChIP_FOXA1inPBS
chr13	52433276	52433277	MACS_peak_3990	14.00	lobChIP_FOXA1inPBS
chr13	52545715	52545716	MACS_peak_3991	8.00	lobChIP_FOXA1inPBS
chr13	52553365	52553366	MACS_peak_3992	11.00	lobChIP_FOXA1inPBS
chr13	52563294	52563295	MACS_peak_3993	12.00	lobChIP_FOXA1inPBS
chr13	52572281	52572282	MACS_peak_3994	25.00	lobChIP_FOXA1inPBS
chr13	52599740	52599741	MACS_peak_3995	42.00	lobChIP_FOXA1inPBS
chr13	52737066	52737067	MACS_peak_3996	13.00	lobChIP_FOXA1inPBS
chr13	53176678	53176679	MACS_peak_3997	8.00	lobChIP_FOXA1inPBS
chr13	53227123	53227124	MACS_peak_3998	10.00	lobChIP_FOXA1inPBS
chr13	55876936	55876937	MACS_peak_3999	8.00	lobChIP_FOXA1inPBS
chr13	57291825	57291826	MACS_peak_4000	7.00	lobChIP_FOXA1inPBS
chr13	58915268	58915269	MACS_peak_4001	12.00	lobChIP_FOXA1inPBS
chr13	59021298	59021299	MACS_peak_4002	11.00	lobChIP_FOXA1inPBS
chr13	59026563	59026564	MACS_peak_4003	17.00	lobChIP_FOXA1inPBS
chr13	60266001	60266002	MACS_peak_4004	10.00	lobChIP_FOXA1inPBS
chr13	62239713	62239714	MACS_peak_4005	27.00	lobChIP_FOXA1inPBS
chr13	65787299	65787300	MACS_peak_4006	9.00	lobChIP_FOXA1inPBS
chr13	67409441	67409442	MACS_peak_4007	9.00	lobChIP_FOXA1inPBS
chr13	68301269	68301270	MACS_peak_4008	11.00	lobChIP_FOXA1inPBS
chr13	68631416	68631417	MACS_peak_4009	9.00	lobChIP_FOXA1inPBS
chr13	68869574	68869575	MACS_peak_4010	8.00	lobChIP_FOXA1inPBS
chr13	68943655	68943656	MACS_peak_4011	6.00	lobChIP_FOXA1inPBS
chr13	68952663	68952664	MACS_peak_4012	12.00	lobChIP_FOXA1inPBS
chr13	68954896	68954897	MACS_peak_4013	33.00	lobChIP_FOXA1inPBS
chr13	70780997	70780998	MACS_peak_4014	25.00	lobChIP_FOXA1inPBS
chr13	70823557	70823558	MACS_peak_4015	6.00	lobChIP_FOXA1inPBS
chr13	71463495	71463496	MACS_peak_4016	22.00	lobChIP_FOXA1inPBS
chr13	71464558	71464559	MACS_peak_4017	39.00	lobChIP_FOXA1inPBS
chr13	71469959	71469960	MACS_peak_4018	10.00	lobChIP_FOXA1inPBS
chr13	71605207	71605208	MACS_peak_4019	17.00	lobChIP_FOXA1inPBS
chr13	72420174	72420175	MACS_peak_4020	7.00	lobChIP_FOXA1inPBS
chr13	72958829	72958830	MACS_peak_4021	9.00	lobChIP_FOXA1inPBS
chr13	73111293	73111294	MACS_peak_4022	9.00	lobChIP_FOXA1inPBS
chr13	73119427	73119428	MACS_peak_4023	12.00	lobChIP_FOXA1inPBS
chr13	73217640	73217641	MACS_peak_4024	7.00	lobChIP_FOXA1inPBS
chr13	73222964	73222965	MACS_peak_4025	25.00	lobChIP_FOXA1inPBS
chr13	73339257	73339258	MACS_peak_4026	6.00	lobChIP_FOXA1inPBS
chr13	73655904	73655905	MACS_peak_4027	5.00	lobChIP_FOXA1inPBS
chr13	74025726	74025727	MACS_peak_4028	10.00	lobChIP_FOXA1inPBS
chr13	74303397	74303398	MACS_peak_4029	10.00	lobChIP_FOXA1inPBS
chr13	74599199	74599200	MACS_peak_4030	25.00	lobChIP_FOXA1inPBS
chr13	74656947	74656948	MACS_peak_4031	13.00	lobChIP_FOXA1inPBS
chr13	74682419	74682420	MACS_peak_4032	9.00	lobChIP_FOXA1inPBS
chr13	74736448	74736449	MACS_peak_4033	8.00	lobChIP_FOXA1inPBS
chr13	74802200	74802201	MACS_peak_4034	7.00	lobChIP_FOXA1inPBS
chr13	74861970	74861971	MACS_peak_4035	16.00	lobChIP_FOXA1inPBS
chr13	75876259	75876260	MACS_peak_4036	19.00	lobChIP_FOXA1inPBS
chr13	75914204	75914205	MACS_peak_4037	62.00	lobChIP_FOXA1inPBS
chr13	75921540	75921541	MACS_peak_4038	5.00	lobChIP_FOXA1inPBS
chr13	75923888	75923889	MACS_peak_4039	9.00	lobChIP_FOXA1inPBS
chr13	75951290	75951291	MACS_peak_4040	9.00	lobChIP_FOXA1inPBS
chr13	75960114	75960115	MACS_peak_4041	9.00	lobChIP_FOXA1inPBS
chr13	75974252	75974253	MACS_peak_4042	9.00	lobChIP_FOXA1inPBS
chr13	75975152	75975153	MACS_peak_4043	13.00	lobChIP_FOXA1inPBS
chr13	75986480	75986481	MACS_peak_4044	11.00	lobChIP_FOXA1inPBS
chr13	76006264	76006265	MACS_peak_4045	10.00	lobChIP_FOXA1inPBS
chr13	76014688	76014689	MACS_peak_4046	9.00	lobChIP_FOXA1inPBS
chr13	76033455	76033456	MACS_peak_4047	28.00	lobChIP_FOXA1inPBS
chr13	76041293	76041294	MACS_peak_4048	25.00	lobChIP_FOXA1inPBS
chr13	76053006	76053007	MACS_peak_4049	9.00	lobChIP_FOXA1inPBS
chr13	76161991	76161992	MACS_peak_4050	13.00	lobChIP_FOXA1inPBS
chr13	76208585	76208586	MACS_peak_4051	11.00	lobChIP_FOXA1inPBS
chr13	76321069	76321070	MACS_peak_4052	13.00	lobChIP_FOXA1inPBS
chr13	76334311	76334312	MACS_peak_4053	13.00	lobChIP_FOXA1inPBS
chr13	76336457	76336458	MACS_peak_4054	14.00	lobChIP_FOXA1inPBS
chr13	76366859	76366860	MACS_peak_4055	9.00	lobChIP_FOXA1inPBS
chr13	77717809	77717810	MACS_peak_4056	12.00	lobChIP_FOXA1inPBS
chr13	77864146	77864147	MACS_peak_4057	19.00	lobChIP_FOXA1inPBS
chr13	77880784	77880785	MACS_peak_4058	18.00	lobChIP_FOXA1inPBS
chr13	77933423	77933424	MACS_peak_4059	8.00	lobChIP_FOXA1inPBS
chr13	78137551	78137552	MACS_peak_4060	11.00	lobChIP_FOXA1inPBS
chr13	79220274	79220275	MACS_peak_4061	7.00	lobChIP_FOXA1inPBS
chr13	79759955	79759956	MACS_peak_4062	16.00	lobChIP_FOXA1inPBS
chr13	79786501	79786502	MACS_peak_4063	9.00	lobChIP_FOXA1inPBS
chr13	79845170	79845171	MACS_peak_4064	6.00	lobChIP_FOXA1inPBS
chr13	79968149	79968150	MACS_peak_4065	29.00	lobChIP_FOXA1inPBS
chr13	80043914	80043915	MACS_peak_4066	7.00	lobChIP_FOXA1inPBS
chr13	80218758	80218759	MACS_peak_4067	10.00	lobChIP_FOXA1inPBS
chr13	80936337	80936338	MACS_peak_4068	6.00	lobChIP_FOXA1inPBS
chr13	81048097	81048098	MACS_peak_4069	10.00	lobChIP_FOXA1inPBS
chr13	81680729	81680730	MACS_peak_4070	13.00	lobChIP_FOXA1inPBS
chr13	82067404	82067405	MACS_peak_4071	12.00	lobChIP_FOXA1inPBS
chr13	82448492	82448493	MACS_peak_4072	229.00	lobChIP_FOXA1inPBS
chr13	85947674	85947675	MACS_peak_4073	10.00	lobChIP_FOXA1inPBS
chr13	89714161	89714162	MACS_peak_4074	17.00	lobChIP_FOXA1inPBS
chr13	90167066	90167067	MACS_peak_4075	10.00	lobChIP_FOXA1inPBS
chr13	90207313	90207314	MACS_peak_4076	13.00	lobChIP_FOXA1inPBS
chr13	90552167	90552168	MACS_peak_4077	12.00	lobChIP_FOXA1inPBS
chr13	91519058	91519059	MACS_peak_4078	28.00	lobChIP_FOXA1inPBS
chr13	92014368	92014369	MACS_peak_4079	28.00	lobChIP_FOXA1inPBS
chr13	92706936	92706937	MACS_peak_4080	17.00	lobChIP_FOXA1inPBS
chr13	93071235	93071236	MACS_peak_4081	6.00	lobChIP_FOXA1inPBS
chr13	93854656	93854657	MACS_peak_4082	41.00	lobChIP_FOXA1inPBS
chr13	93877019	93877020	MACS_peak_4083	14.00	lobChIP_FOXA1inPBS
chr13	93877926	93877927	MACS_peak_4084	6.00	lobChIP_FOXA1inPBS
chr13	93884428	93884429	MACS_peak_4085	10.00	lobChIP_FOXA1inPBS
chr13	93901775	93901776	MACS_peak_4086	10.00	lobChIP_FOXA1inPBS
chr13	93913035	93913036	MACS_peak_4087	11.00	lobChIP_FOXA1inPBS
chr13	93923448	93923449	MACS_peak_4088	8.00	lobChIP_FOXA1inPBS
chr13	94014224	94014225	MACS_peak_4089	10.00	lobChIP_FOXA1inPBS
chr13	94392608	94392609	MACS_peak_4090	8.00	lobChIP_FOXA1inPBS
chr13	94419928	94419929	MACS_peak_4091	6.00	lobChIP_FOXA1inPBS
chr13	94427938	94427939	MACS_peak_4092	8.00	lobChIP_FOXA1inPBS
chr13	94615960	94615961	MACS_peak_4093	17.00	lobChIP_FOXA1inPBS
chr13	94618742	94618743	MACS_peak_4094	21.00	lobChIP_FOXA1inPBS
chr13	94901972	94901973	MACS_peak_4095	8.00	lobChIP_FOXA1inPBS
chr13	94915570	94915571	MACS_peak_4096	11.00	lobChIP_FOXA1inPBS
chr13	94944614	94944615	MACS_peak_4097	18.00	lobChIP_FOXA1inPBS
chr13	95053389	95053390	MACS_peak_4098	17.00	lobChIP_FOXA1inPBS
chr13	95252337	95252338	MACS_peak_4099	7.00	lobChIP_FOXA1inPBS
chr13	95696923	95696924	MACS_peak_4100	12.00	lobChIP_FOXA1inPBS
chr13	95776262	95776263	MACS_peak_4101	8.00	lobChIP_FOXA1inPBS
chr13	95813072	95813073	MACS_peak_4102	40.00	lobChIP_FOXA1inPBS
chr13	95833143	95833144	MACS_peak_4103	13.00	lobChIP_FOXA1inPBS
chr13	95927624	95927625	MACS_peak_4104	18.00	lobChIP_FOXA1inPBS
chr13	95950090	95950091	MACS_peak_4105	29.00	lobChIP_FOXA1inPBS
chr13	96146620	96146621	MACS_peak_4106	19.00	lobChIP_FOXA1inPBS
chr13	96187719	96187720	MACS_peak_4107	11.00	lobChIP_FOXA1inPBS
chr13	96350448	96350449	MACS_peak_4108	12.00	lobChIP_FOXA1inPBS
chr13	97500746	97500747	MACS_peak_4109	8.00	lobChIP_FOXA1inPBS
chr13	97578315	97578316	MACS_peak_4110	43.00	lobChIP_FOXA1inPBS
chr13	97793959	97793960	MACS_peak_4111	11.00	lobChIP_FOXA1inPBS
chr13	97818286	97818287	MACS_peak_4112	15.00	lobChIP_FOXA1inPBS
chr13	97896903	97896904	MACS_peak_4113	18.00	lobChIP_FOXA1inPBS
chr13	97909768	97909769	MACS_peak_4114	17.00	lobChIP_FOXA1inPBS
chr13	97910518	97910519	MACS_peak_4115	15.00	lobChIP_FOXA1inPBS
chr13	97927305	97927306	MACS_peak_4116	36.00	lobChIP_FOXA1inPBS
chr13	97960669	97960670	MACS_peak_4117	29.00	lobChIP_FOXA1inPBS
chr13	97961741	97961742	MACS_peak_4118	8.00	lobChIP_FOXA1inPBS
chr13	98079185	98079186	MACS_peak_4119	24.00	lobChIP_FOXA1inPBS
chr13	98115522	98115523	MACS_peak_4120	8.00	lobChIP_FOXA1inPBS
chr13	98143910	98143911	MACS_peak_4121	13.00	lobChIP_FOXA1inPBS
chr13	98679568	98679569	MACS_peak_4122	8.00	lobChIP_FOXA1inPBS
chr13	98702266	98702267	MACS_peak_4123	6.00	lobChIP_FOXA1inPBS
chr13	98813803	98813804	MACS_peak_4124	40.00	lobChIP_FOXA1inPBS
chr13	98825891	98825892	MACS_peak_4125	14.00	lobChIP_FOXA1inPBS
chr13	98852008	98852009	MACS_peak_4126	33.00	lobChIP_FOXA1inPBS
chr13	98896102	98896103	MACS_peak_4127	14.00	lobChIP_FOXA1inPBS
chr13	98898935	98898936	MACS_peak_4128	16.00	lobChIP_FOXA1inPBS
chr13	98919525	98919526	MACS_peak_4129	16.00	lobChIP_FOXA1inPBS
chr13	98922018	98922019	MACS_peak_4130	12.00	lobChIP_FOXA1inPBS
chr13	98925772	98925773	MACS_peak_4131	10.00	lobChIP_FOXA1inPBS
chr13	98926833	98926834	MACS_peak_4132	14.00	lobChIP_FOXA1inPBS
chr13	98944109	98944110	MACS_peak_4133	8.00	lobChIP_FOXA1inPBS
chr13	99142622	99142623	MACS_peak_4134	20.00	lobChIP_FOXA1inPBS
chr13	99160956	99160957	MACS_peak_4135	5.00	lobChIP_FOXA1inPBS
chr13	99221988	99221989	MACS_peak_4136	27.00	lobChIP_FOXA1inPBS
chr13	99300737	99300738	MACS_peak_4137	9.00	lobChIP_FOXA1inPBS
chr13	99636587	99636588	MACS_peak_4138	15.00	lobChIP_FOXA1inPBS
chr13	100149145	100149146	MACS_peak_4139	9.00	lobChIP_FOXA1inPBS
chr13	100173296	100173297	MACS_peak_4140	10.00	lobChIP_FOXA1inPBS
chr13	100207639	100207640	MACS_peak_4141	11.00	lobChIP_FOXA1inPBS
chr13	100211068	100211069	MACS_peak_4142	20.00	lobChIP_FOXA1inPBS
chr13	100314430	100314431	MACS_peak_4143	9.00	lobChIP_FOXA1inPBS
chr13	100593651	100593652	MACS_peak_4144	20.00	lobChIP_FOXA1inPBS
chr13	100608944	100608945	MACS_peak_4145	24.00	lobChIP_FOXA1inPBS
chr13	100611634	100611635	MACS_peak_4146	8.00	lobChIP_FOXA1inPBS
chr13	100612502	100612503	MACS_peak_4147	6.00	lobChIP_FOXA1inPBS
chr13	100791509	100791510	MACS_peak_4148	7.00	lobChIP_FOXA1inPBS
chr13	100827565	100827566	MACS_peak_4149	9.00	lobChIP_FOXA1inPBS
chr13	100887162	100887163	MACS_peak_4150	7.00	lobChIP_FOXA1inPBS
chr13	100944124	100944125	MACS_peak_4151	11.00	lobChIP_FOXA1inPBS
chr13	100967007	100967008	MACS_peak_4152	23.00	lobChIP_FOXA1inPBS
chr13	100976303	100976304	MACS_peak_4153	7.00	lobChIP_FOXA1inPBS
chr13	101386790	101386791	MACS_peak_4154	91.00	lobChIP_FOXA1inPBS
chr13	101460870	101460871	MACS_peak_4155	8.00	lobChIP_FOXA1inPBS
chr13	102229520	102229521	MACS_peak_4156	8.00	lobChIP_FOXA1inPBS
chr13	102301699	102301700	MACS_peak_4157	9.00	lobChIP_FOXA1inPBS
chr13	102362460	102362461	MACS_peak_4158	9.00	lobChIP_FOXA1inPBS
chr13	102528396	102528397	MACS_peak_4159	7.00	lobChIP_FOXA1inPBS
chr13	103320426	103320427	MACS_peak_4160	7.00	lobChIP_FOXA1inPBS
chr13	103422885	103422886	MACS_peak_4161	18.00	lobChIP_FOXA1inPBS
chr13	103483244	103483245	MACS_peak_4162	21.00	lobChIP_FOXA1inPBS
chr13	103552903	103552904	MACS_peak_4163	12.00	lobChIP_FOXA1inPBS
chr13	107331142	107331143	MACS_peak_4164	7.00	lobChIP_FOXA1inPBS
chr13	107552024	107552025	MACS_peak_4165	13.00	lobChIP_FOXA1inPBS
chr13	107844819	107844820	MACS_peak_4166	21.00	lobChIP_FOXA1inPBS
chr13	108807142	108807143	MACS_peak_4167	20.00	lobChIP_FOXA1inPBS
chr13	108882956	108882957	MACS_peak_4168	67.00	lobChIP_FOXA1inPBS
chr13	109568434	109568435	MACS_peak_4169	7.00	lobChIP_FOXA1inPBS
chr13	109962652	109962653	MACS_peak_4170	6.00	lobChIP_FOXA1inPBS
chr13	110373384	110373385	MACS_peak_4171	8.00	lobChIP_FOXA1inPBS
chr13	110455553	110455554	MACS_peak_4172	7.00	lobChIP_FOXA1inPBS
chr13	111208278	111208279	MACS_peak_4173	11.00	lobChIP_FOXA1inPBS
chr13	111213443	111213444	MACS_peak_4174	7.00	lobChIP_FOXA1inPBS
chr13	111229365	111229366	MACS_peak_4175	16.00	lobChIP_FOXA1inPBS
chr13	111856880	111856881	MACS_peak_4176	9.00	lobChIP_FOXA1inPBS
chr13	112666391	112666392	MACS_peak_4177	7.00	lobChIP_FOXA1inPBS
chr13	113411649	113411650	MACS_peak_4178	36.00	lobChIP_FOXA1inPBS
chr13	113560437	113560438	MACS_peak_4179	9.00	lobChIP_FOXA1inPBS
chr13	113580502	113580503	MACS_peak_4180	11.00	lobChIP_FOXA1inPBS
chr13	113606238	113606239	MACS_peak_4181	11.00	lobChIP_FOXA1inPBS
chr13	113607452	113607453	MACS_peak_4182	26.00	lobChIP_FOXA1inPBS
chr13	113760119	113760120	MACS_peak_4183	15.00	lobChIP_FOXA1inPBS
chr13	113793504	113793505	MACS_peak_4184	17.00	lobChIP_FOXA1inPBS
chr13	113824425	113824426	MACS_peak_4185	10.00	lobChIP_FOXA1inPBS
chr13	113838332	113838333	MACS_peak_4186	12.00	lobChIP_FOXA1inPBS
chr13	113874631	113874632	MACS_peak_4187	10.00	lobChIP_FOXA1inPBS
chr13	113954386	113954387	MACS_peak_4188	10.00	lobChIP_FOXA1inPBS
chr13	114034458	114034459	MACS_peak_4189	16.00	lobChIP_FOXA1inPBS
chr13	114147843	114147844	MACS_peak_4190	15.00	lobChIP_FOXA1inPBS
chr13	114927961	114927962	MACS_peak_4191	10.00	lobChIP_FOXA1inPBS
chr14	20346744	20346745	MACS_peak_4192	16.00	lobChIP_FOXA1inPBS
chr14	20797060	20797061	MACS_peak_4193	6.00	lobChIP_FOXA1inPBS
chr14	20799346	20799347	MACS_peak_4194	13.00	lobChIP_FOXA1inPBS
chr14	20937278	20937279	MACS_peak_4195	11.00	lobChIP_FOXA1inPBS
chr14	20940919	20940920	MACS_peak_4196	9.00	lobChIP_FOXA1inPBS
chr14	20999338	20999339	MACS_peak_4197	10.00	lobChIP_FOXA1inPBS
chr14	21081734	21081735	MACS_peak_4198	34.00	lobChIP_FOXA1inPBS
chr14	21099462	21099463	MACS_peak_4199	14.00	lobChIP_FOXA1inPBS
chr14	21140271	21140272	MACS_peak_4200	13.00	lobChIP_FOXA1inPBS
chr14	21153337	21153338	MACS_peak_4201	10.00	lobChIP_FOXA1inPBS
chr14	21154718	21154719	MACS_peak_4202	21.00	lobChIP_FOXA1inPBS
chr14	21155811	21155812	MACS_peak_4203	58.00	lobChIP_FOXA1inPBS
chr14	21156780	21156781	MACS_peak_4204	7.00	lobChIP_FOXA1inPBS
chr14	21411231	21411232	MACS_peak_4205	8.00	lobChIP_FOXA1inPBS
chr14	21522802	21522803	MACS_peak_4206	28.00	lobChIP_FOXA1inPBS
chr14	21524172	21524173	MACS_peak_4207	21.00	lobChIP_FOXA1inPBS
chr14	21530858	21530859	MACS_peak_4208	12.00	lobChIP_FOXA1inPBS
chr14	21535886	21535887	MACS_peak_4209	11.00	lobChIP_FOXA1inPBS
chr14	21596810	21596811	MACS_peak_4210	16.00	lobChIP_FOXA1inPBS
chr14	21776616	21776617	MACS_peak_4211	14.00	lobChIP_FOXA1inPBS
chr14	21852240	21852241	MACS_peak_4212	9.00	lobChIP_FOXA1inPBS
chr14	21944303	21944304	MACS_peak_4213	8.00	lobChIP_FOXA1inPBS
chr14	21997280	21997281	MACS_peak_4214	11.00	lobChIP_FOXA1inPBS
chr14	22025751	22025752	MACS_peak_4215	39.00	lobChIP_FOXA1inPBS
chr14	22179855	22179856	MACS_peak_4216	21.00	lobChIP_FOXA1inPBS
chr14	22515556	22515557	MACS_peak_4217	25.00	lobChIP_FOXA1inPBS
chr14	22764755	22764756	MACS_peak_4218	62.00	lobChIP_FOXA1inPBS
chr14	22802640	22802641	MACS_peak_4219	27.00	lobChIP_FOXA1inPBS
chr14	23041693	23041694	MACS_peak_4220	43.00	lobChIP_FOXA1inPBS
chr14	23067207	23067208	MACS_peak_4221	15.00	lobChIP_FOXA1inPBS
chr14	23075550	23075551	MACS_peak_4222	17.00	lobChIP_FOXA1inPBS
chr14	23122464	23122465	MACS_peak_4223	17.00	lobChIP_FOXA1inPBS
chr14	23169516	23169517	MACS_peak_4224	11.00	lobChIP_FOXA1inPBS
chr14	23223018	23223019	MACS_peak_4225	17.00	lobChIP_FOXA1inPBS
chr14	23265804	23265805	MACS_peak_4226	21.00	lobChIP_FOXA1inPBS
chr14	23278236	23278237	MACS_peak_4227	10.00	lobChIP_FOXA1inPBS
chr14	23288593	23288594	MACS_peak_4228	16.00	lobChIP_FOXA1inPBS
chr14	23294307	23294308	MACS_peak_4229	10.00	lobChIP_FOXA1inPBS
chr14	23340503	23340504	MACS_peak_4230	22.00	lobChIP_FOXA1inPBS
chr14	23372511	23372512	MACS_peak_4231	93.00	lobChIP_FOXA1inPBS
chr14	23446096	23446097	MACS_peak_4232	54.00	lobChIP_FOXA1inPBS
chr14	23475550	23475551	MACS_peak_4233	18.00	lobChIP_FOXA1inPBS
chr14	23476606	23476607	MACS_peak_4234	9.00	lobChIP_FOXA1inPBS
chr14	23643205	23643206	MACS_peak_4235	13.00	lobChIP_FOXA1inPBS
chr14	23646546	23646547	MACS_peak_4236	11.00	lobChIP_FOXA1inPBS
chr14	23714627	23714628	MACS_peak_4237	23.00	lobChIP_FOXA1inPBS
chr14	24052706	24052707	MACS_peak_4238	14.00	lobChIP_FOXA1inPBS
chr14	24079628	24079629	MACS_peak_4239	12.00	lobChIP_FOXA1inPBS
chr14	24082881	24082882	MACS_peak_4240	35.00	lobChIP_FOXA1inPBS
chr14	24087028	24087029	MACS_peak_4241	11.00	lobChIP_FOXA1inPBS
chr14	24087999	24088000	MACS_peak_4242	35.00	lobChIP_FOXA1inPBS
chr14	24159080	24159081	MACS_peak_4243	28.00	lobChIP_FOXA1inPBS
chr14	24207564	24207565	MACS_peak_4244	6.00	lobChIP_FOXA1inPBS
chr14	24236111	24236112	MACS_peak_4245	13.00	lobChIP_FOXA1inPBS
chr14	24297426	24297427	MACS_peak_4246	19.00	lobChIP_FOXA1inPBS
chr14	24353318	24353319	MACS_peak_4247	98.00	lobChIP_FOXA1inPBS
chr14	24443388	24443389	MACS_peak_4248	18.00	lobChIP_FOXA1inPBS
chr14	24476784	24476785	MACS_peak_4249	10.00	lobChIP_FOXA1inPBS
chr14	24502238	24502239	MACS_peak_4250	8.00	lobChIP_FOXA1inPBS
chr14	24564972	24564973	MACS_peak_4251	10.00	lobChIP_FOXA1inPBS
chr14	24580040	24580041	MACS_peak_4252	14.00	lobChIP_FOXA1inPBS
chr14	24721814	24721815	MACS_peak_4253	15.00	lobChIP_FOXA1inPBS
chr14	24856126	24856127	MACS_peak_4254	38.00	lobChIP_FOXA1inPBS
chr14	24896502	24896503	MACS_peak_4255	8.00	lobChIP_FOXA1inPBS
chr14	24912155	24912156	MACS_peak_4256	16.00	lobChIP_FOXA1inPBS
chr14	25259470	25259471	MACS_peak_4257	12.00	lobChIP_FOXA1inPBS
chr14	25286493	25286494	MACS_peak_4258	21.00	lobChIP_FOXA1inPBS
chr14	25308862	25308863	MACS_peak_4259	88.00	lobChIP_FOXA1inPBS
chr14	25361402	25361403	MACS_peak_4260	21.00	lobChIP_FOXA1inPBS
chr14	25378230	25378231	MACS_peak_4261	33.00	lobChIP_FOXA1inPBS
chr14	25385387	25385388	MACS_peak_4262	22.00	lobChIP_FOXA1inPBS
chr14	25452017	25452018	MACS_peak_4263	64.00	lobChIP_FOXA1inPBS
chr14	25504598	25504599	MACS_peak_4264	16.00	lobChIP_FOXA1inPBS
chr14	26852635	26852636	MACS_peak_4265	14.00	lobChIP_FOXA1inPBS
chr14	28886477	28886478	MACS_peak_4266	8.00	lobChIP_FOXA1inPBS
chr14	29498400	29498401	MACS_peak_4267	56.00	lobChIP_FOXA1inPBS
chr14	30365885	30365886	MACS_peak_4268	17.00	lobChIP_FOXA1inPBS
chr14	30824915	30824916	MACS_peak_4269	9.00	lobChIP_FOXA1inPBS
chr14	30857216	30857217	MACS_peak_4270	16.00	lobChIP_FOXA1inPBS
chr14	31028186	31028187	MACS_peak_4271	29.00	lobChIP_FOXA1inPBS
chr14	31358337	31358338	MACS_peak_4272	24.00	lobChIP_FOXA1inPBS
chr14	31436701	31436702	MACS_peak_4273	48.00	lobChIP_FOXA1inPBS
chr14	31502731	31502732	MACS_peak_4274	41.00	lobChIP_FOXA1inPBS
chr14	31517896	31517897	MACS_peak_4275	10.00	lobChIP_FOXA1inPBS
chr14	31523605	31523606	MACS_peak_4276	6.00	lobChIP_FOXA1inPBS
chr14	31560850	31560851	MACS_peak_4277	34.00	lobChIP_FOXA1inPBS
chr14	31597155	31597156	MACS_peak_4278	28.00	lobChIP_FOXA1inPBS
chr14	31633450	31633451	MACS_peak_4279	17.00	lobChIP_FOXA1inPBS
chr14	31667835	31667836	MACS_peak_4280	13.00	lobChIP_FOXA1inPBS
chr14	31674243	31674244	MACS_peak_4281	7.00	lobChIP_FOXA1inPBS
chr14	31690208	31690209	MACS_peak_4282	8.00	lobChIP_FOXA1inPBS
chr14	31699026	31699027	MACS_peak_4283	106.00	lobChIP_FOXA1inPBS
chr14	31722458	31722459	MACS_peak_4284	10.00	lobChIP_FOXA1inPBS
chr14	31727503	31727504	MACS_peak_4285	77.00	lobChIP_FOXA1inPBS
chr14	31731432	31731433	MACS_peak_4286	25.00	lobChIP_FOXA1inPBS
chr14	31733700	31733701	MACS_peak_4287	31.00	lobChIP_FOXA1inPBS
chr14	31735197	31735198	MACS_peak_4288	12.00	lobChIP_FOXA1inPBS
chr14	31737312	31737313	MACS_peak_4289	14.00	lobChIP_FOXA1inPBS
chr14	31743531	31743532	MACS_peak_4290	13.00	lobChIP_FOXA1inPBS
chr14	31749821	31749822	MACS_peak_4291	33.00	lobChIP_FOXA1inPBS
chr14	31847453	31847454	MACS_peak_4292	17.00	lobChIP_FOXA1inPBS
chr14	31890741	31890742	MACS_peak_4293	20.00	lobChIP_FOXA1inPBS
chr14	31927681	31927682	MACS_peak_4294	42.00	lobChIP_FOXA1inPBS
chr14	31998439	31998440	MACS_peak_4295	39.00	lobChIP_FOXA1inPBS
chr14	32019959	32019960	MACS_peak_4296	15.00	lobChIP_FOXA1inPBS
chr14	32088317	32088318	MACS_peak_4297	14.00	lobChIP_FOXA1inPBS
chr14	32089143	32089144	MACS_peak_4298	15.00	lobChIP_FOXA1inPBS
chr14	32095063	32095064	MACS_peak_4299	20.00	lobChIP_FOXA1inPBS
chr14	32165772	32165773	MACS_peak_4300	12.00	lobChIP_FOXA1inPBS
chr14	32186957	32186958	MACS_peak_4301	62.00	lobChIP_FOXA1inPBS
chr14	32217672	32217673	MACS_peak_4302	19.00	lobChIP_FOXA1inPBS
chr14	32324720	32324721	MACS_peak_4303	26.00	lobChIP_FOXA1inPBS
chr14	32509013	32509014	MACS_peak_4304	13.00	lobChIP_FOXA1inPBS
chr14	32548695	32548696	MACS_peak_4305	10.00	lobChIP_FOXA1inPBS
chr14	32553116	32553117	MACS_peak_4306	23.00	lobChIP_FOXA1inPBS
chr14	32777527	32777528	MACS_peak_4307	25.00	lobChIP_FOXA1inPBS
chr14	34369140	34369141	MACS_peak_4308	54.00	lobChIP_FOXA1inPBS
chr14	34378802	34378803	MACS_peak_4309	17.00	lobChIP_FOXA1inPBS
chr14	34460203	34460204	MACS_peak_4310	13.00	lobChIP_FOXA1inPBS
chr14	34493535	34493536	MACS_peak_4311	59.00	lobChIP_FOXA1inPBS
chr14	34505643	34505644	MACS_peak_4312	10.00	lobChIP_FOXA1inPBS
chr14	34508681	34508682	MACS_peak_4313	30.00	lobChIP_FOXA1inPBS
chr14	34532810	34532811	MACS_peak_4314	10.00	lobChIP_FOXA1inPBS
chr14	34551194	34551195	MACS_peak_4315	25.00	lobChIP_FOXA1inPBS
chr14	34609231	34609232	MACS_peak_4316	25.00	lobChIP_FOXA1inPBS
chr14	34837696	34837697	MACS_peak_4317	32.00	lobChIP_FOXA1inPBS
chr14	34905800	34905801	MACS_peak_4318	14.00	lobChIP_FOXA1inPBS
chr14	34919689	34919690	MACS_peak_4319	22.00	lobChIP_FOXA1inPBS
chr14	34930205	34930206	MACS_peak_4320	11.00	lobChIP_FOXA1inPBS
chr14	34952587	34952588	MACS_peak_4321	6.00	lobChIP_FOXA1inPBS
chr14	34966853	34966854	MACS_peak_4322	17.00	lobChIP_FOXA1inPBS
chr14	35008886	35008887	MACS_peak_4323	33.00	lobChIP_FOXA1inPBS
chr14	35031962	35031963	MACS_peak_4324	9.00	lobChIP_FOXA1inPBS
chr14	35079987	35079988	MACS_peak_4325	7.00	lobChIP_FOXA1inPBS
chr14	35156855	35156856	MACS_peak_4326	20.00	lobChIP_FOXA1inPBS
chr14	35177179	35177180	MACS_peak_4327	20.00	lobChIP_FOXA1inPBS
chr14	35267363	35267364	MACS_peak_4328	21.00	lobChIP_FOXA1inPBS
chr14	35307030	35307031	MACS_peak_4329	15.00	lobChIP_FOXA1inPBS
chr14	35310205	35310206	MACS_peak_4330	10.00	lobChIP_FOXA1inPBS
chr14	35345185	35345186	MACS_peak_4331	8.00	lobChIP_FOXA1inPBS
chr14	35450396	35450397	MACS_peak_4332	23.00	lobChIP_FOXA1inPBS
chr14	35517518	35517519	MACS_peak_4333	13.00	lobChIP_FOXA1inPBS
chr14	35520099	35520100	MACS_peak_4334	22.00	lobChIP_FOXA1inPBS
chr14	35658905	35658906	MACS_peak_4335	15.00	lobChIP_FOXA1inPBS
chr14	35673315	35673316	MACS_peak_4336	13.00	lobChIP_FOXA1inPBS
chr14	35683367	35683368	MACS_peak_4337	11.00	lobChIP_FOXA1inPBS
chr14	35796697	35796698	MACS_peak_4338	14.00	lobChIP_FOXA1inPBS
chr14	35805754	35805755	MACS_peak_4339	11.00	lobChIP_FOXA1inPBS
chr14	35816953	35816954	MACS_peak_4340	26.00	lobChIP_FOXA1inPBS
chr14	35823250	35823251	MACS_peak_4341	21.00	lobChIP_FOXA1inPBS
chr14	35825871	35825872	MACS_peak_4342	11.00	lobChIP_FOXA1inPBS
chr14	35842065	35842066	MACS_peak_4343	11.00	lobChIP_FOXA1inPBS
chr14	35867780	35867781	MACS_peak_4344	21.00	lobChIP_FOXA1inPBS
chr14	35876317	35876318	MACS_peak_4345	20.00	lobChIP_FOXA1inPBS
chr14	36131354	36131355	MACS_peak_4346	41.00	lobChIP_FOXA1inPBS
chr14	36216837	36216838	MACS_peak_4347	13.00	lobChIP_FOXA1inPBS
chr14	36276995	36276996	MACS_peak_4348	104.00	lobChIP_FOXA1inPBS
chr14	36283881	36283882	MACS_peak_4349	28.00	lobChIP_FOXA1inPBS
chr14	36678065	36678066	MACS_peak_4350	218.00	lobChIP_FOXA1inPBS
chr14	36923934	36923935	MACS_peak_4351	16.00	lobChIP_FOXA1inPBS
chr14	37074293	37074294	MACS_peak_4352	21.00	lobChIP_FOXA1inPBS
chr14	37240682	37240683	MACS_peak_4353	23.00	lobChIP_FOXA1inPBS
chr14	37445268	37445269	MACS_peak_4354	12.00	lobChIP_FOXA1inPBS
chr14	37449831	37449832	MACS_peak_4355	35.00	lobChIP_FOXA1inPBS
chr14	37506190	37506191	MACS_peak_4356	19.00	lobChIP_FOXA1inPBS
chr14	37669750	37669751	MACS_peak_4357	16.00	lobChIP_FOXA1inPBS
chr14	37700031	37700032	MACS_peak_4358	13.00	lobChIP_FOXA1inPBS
chr14	37715949	37715950	MACS_peak_4359	24.00	lobChIP_FOXA1inPBS
chr14	37740010	37740011	MACS_peak_4360	32.00	lobChIP_FOXA1inPBS
chr14	37748146	37748147	MACS_peak_4361	34.00	lobChIP_FOXA1inPBS
chr14	37772998	37772999	MACS_peak_4362	14.00	lobChIP_FOXA1inPBS
chr14	37799038	37799039	MACS_peak_4363	54.00	lobChIP_FOXA1inPBS
chr14	37838963	37838964	MACS_peak_4364	13.00	lobChIP_FOXA1inPBS
chr14	37854689	37854690	MACS_peak_4365	37.00	lobChIP_FOXA1inPBS
chr14	37883128	37883129	MACS_peak_4366	21.00	lobChIP_FOXA1inPBS
chr14	37936025	37936026	MACS_peak_4367	110.00	lobChIP_FOXA1inPBS
chr14	37937447	37937448	MACS_peak_4368	35.00	lobChIP_FOXA1inPBS
chr14	37950409	37950410	MACS_peak_4369	16.00	lobChIP_FOXA1inPBS
chr14	38016264	38016265	MACS_peak_4370	177.00	lobChIP_FOXA1inPBS
chr14	38034747	38034748	MACS_peak_4371	10.00	lobChIP_FOXA1inPBS
chr14	38058054	38058055	MACS_peak_4372	34.00	lobChIP_FOXA1inPBS
chr14	38058930	38058931	MACS_peak_4373	10.00	lobChIP_FOXA1inPBS
chr14	38072194	38072195	MACS_peak_4374	24.00	lobChIP_FOXA1inPBS
chr14	38119775	38119776	MACS_peak_4375	12.00	lobChIP_FOXA1inPBS
chr14	38127497	38127498	MACS_peak_4376	11.00	lobChIP_FOXA1inPBS
chr14	38167006	38167007	MACS_peak_4377	27.00	lobChIP_FOXA1inPBS
chr14	38168549	38168550	MACS_peak_4378	15.00	lobChIP_FOXA1inPBS
chr14	38181426	38181427	MACS_peak_4379	11.00	lobChIP_FOXA1inPBS
chr14	38257461	38257462	MACS_peak_4380	35.00	lobChIP_FOXA1inPBS
chr14	38260717	38260718	MACS_peak_4381	32.00	lobChIP_FOXA1inPBS
chr14	38286092	38286093	MACS_peak_4382	10.00	lobChIP_FOXA1inPBS
chr14	38288706	38288707	MACS_peak_4383	10.00	lobChIP_FOXA1inPBS
chr14	38307889	38307890	MACS_peak_4384	14.00	lobChIP_FOXA1inPBS
chr14	38318686	38318687	MACS_peak_4385	16.00	lobChIP_FOXA1inPBS
chr14	38334436	38334437	MACS_peak_4386	50.00	lobChIP_FOXA1inPBS
chr14	38340880	38340881	MACS_peak_4387	12.00	lobChIP_FOXA1inPBS
chr14	39075157	39075158	MACS_peak_4388	39.00	lobChIP_FOXA1inPBS
chr14	39338231	39338232	MACS_peak_4389	28.00	lobChIP_FOXA1inPBS
chr14	39434058	39434059	MACS_peak_4390	19.00	lobChIP_FOXA1inPBS
chr14	39442436	39442437	MACS_peak_4391	10.00	lobChIP_FOXA1inPBS
chr14	39493567	39493568	MACS_peak_4392	135.00	lobChIP_FOXA1inPBS
chr14	39495142	39495143	MACS_peak_4393	45.00	lobChIP_FOXA1inPBS
chr14	39630440	39630441	MACS_peak_4394	12.00	lobChIP_FOXA1inPBS
chr14	39687832	39687833	MACS_peak_4395	9.00	lobChIP_FOXA1inPBS
chr14	39703112	39703113	MACS_peak_4396	20.00	lobChIP_FOXA1inPBS
chr14	39734479	39734480	MACS_peak_4397	10.00	lobChIP_FOXA1inPBS
chr14	39737035	39737036	MACS_peak_4398	13.00	lobChIP_FOXA1inPBS
chr14	39902646	39902647	MACS_peak_4399	10.00	lobChIP_FOXA1inPBS
chr14	39937202	39937203	MACS_peak_4400	16.00	lobChIP_FOXA1inPBS
chr14	42132522	42132523	MACS_peak_4401	15.00	lobChIP_FOXA1inPBS
chr14	42492879	42492880	MACS_peak_4402	14.00	lobChIP_FOXA1inPBS
chr14	42642938	42642939	MACS_peak_4403	10.00	lobChIP_FOXA1inPBS
chr14	44248472	44248473	MACS_peak_4404	38.00	lobChIP_FOXA1inPBS
chr14	44249749	44249750	MACS_peak_4405	30.00	lobChIP_FOXA1inPBS
chr14	44365484	44365485	MACS_peak_4406	8.00	lobChIP_FOXA1inPBS
chr14	44369585	44369586	MACS_peak_4407	10.00	lobChIP_FOXA1inPBS
chr14	45366100	45366101	MACS_peak_4408	7.00	lobChIP_FOXA1inPBS
chr14	45386629	45386630	MACS_peak_4409	8.00	lobChIP_FOXA1inPBS
chr14	45464499	45464500	MACS_peak_4410	8.00	lobChIP_FOXA1inPBS
chr14	45527272	45527273	MACS_peak_4411	7.00	lobChIP_FOXA1inPBS
chr14	45582342	45582343	MACS_peak_4412	7.00	lobChIP_FOXA1inPBS
chr14	45604944	45604945	MACS_peak_4413	7.00	lobChIP_FOXA1inPBS
chr14	48175121	48175122	MACS_peak_4414	10.00	lobChIP_FOXA1inPBS
chr14	49274711	49274712	MACS_peak_4415	24.00	lobChIP_FOXA1inPBS
chr14	49946282	49946283	MACS_peak_4416	9.00	lobChIP_FOXA1inPBS
chr14	50063058	50063059	MACS_peak_4417	5.00	lobChIP_FOXA1inPBS
chr14	50108744	50108745	MACS_peak_4418	42.00	lobChIP_FOXA1inPBS
chr14	50127054	50127055	MACS_peak_4419	34.00	lobChIP_FOXA1inPBS
chr14	50188652	50188653	MACS_peak_4420	16.00	lobChIP_FOXA1inPBS
chr14	50229133	50229134	MACS_peak_4421	20.00	lobChIP_FOXA1inPBS
chr14	50234869	50234870	MACS_peak_4422	8.00	lobChIP_FOXA1inPBS
chr14	50237252	50237253	MACS_peak_4423	23.00	lobChIP_FOXA1inPBS
chr14	50413332	50413333	MACS_peak_4424	11.00	lobChIP_FOXA1inPBS
chr14	50439768	50439769	MACS_peak_4425	8.00	lobChIP_FOXA1inPBS
chr14	50454318	50454319	MACS_peak_4426	8.00	lobChIP_FOXA1inPBS
chr14	50469848	50469849	MACS_peak_4427	13.00	lobChIP_FOXA1inPBS
chr14	50485285	50485286	MACS_peak_4428	11.00	lobChIP_FOXA1inPBS
chr14	50511939	50511940	MACS_peak_4429	10.00	lobChIP_FOXA1inPBS
chr14	50676963	50676964	MACS_peak_4430	8.00	lobChIP_FOXA1inPBS
chr14	50770725	50770726	MACS_peak_4431	23.00	lobChIP_FOXA1inPBS
chr14	50775202	50775203	MACS_peak_4432	6.00	lobChIP_FOXA1inPBS
chr14	50809557	50809558	MACS_peak_4433	6.00	lobChIP_FOXA1inPBS
chr14	50908717	50908718	MACS_peak_4434	19.00	lobChIP_FOXA1inPBS
chr14	50988923	50988924	MACS_peak_4435	12.00	lobChIP_FOXA1inPBS
chr14	51009842	51009843	MACS_peak_4436	8.00	lobChIP_FOXA1inPBS
chr14	51067038	51067039	MACS_peak_4437	13.00	lobChIP_FOXA1inPBS
chr14	51300192	51300193	MACS_peak_4438	12.00	lobChIP_FOXA1inPBS
chr14	51306007	51306008	MACS_peak_4439	6.00	lobChIP_FOXA1inPBS
chr14	51341825	51341826	MACS_peak_4440	11.00	lobChIP_FOXA1inPBS
chr14	51402490	51402491	MACS_peak_4441	33.00	lobChIP_FOXA1inPBS
chr14	51406176	51406177	MACS_peak_4442	21.00	lobChIP_FOXA1inPBS
chr14	51412053	51412054	MACS_peak_4443	13.00	lobChIP_FOXA1inPBS
chr14	51472163	51472164	MACS_peak_4444	13.00	lobChIP_FOXA1inPBS
chr14	51502521	51502522	MACS_peak_4445	19.00	lobChIP_FOXA1inPBS
chr14	51614128	51614129	MACS_peak_4446	9.00	lobChIP_FOXA1inPBS
chr14	52001289	52001290	MACS_peak_4447	8.00	lobChIP_FOXA1inPBS
chr14	52143427	52143428	MACS_peak_4448	12.00	lobChIP_FOXA1inPBS
chr14	52924821	52924822	MACS_peak_4449	22.00	lobChIP_FOXA1inPBS
chr14	53167719	53167720	MACS_peak_4450	29.00	lobChIP_FOXA1inPBS
chr14	53173274	53173275	MACS_peak_4451	8.00	lobChIP_FOXA1inPBS
chr14	53222454	53222455	MACS_peak_4452	12.00	lobChIP_FOXA1inPBS
chr14	53260039	53260040	MACS_peak_4453	11.00	lobChIP_FOXA1inPBS
chr14	53288594	53288595	MACS_peak_4454	32.00	lobChIP_FOXA1inPBS
chr14	53308182	53308183	MACS_peak_4455	10.00	lobChIP_FOXA1inPBS
chr14	53319917	53319918	MACS_peak_4456	10.00	lobChIP_FOXA1inPBS
chr14	53393097	53393098	MACS_peak_4457	9.00	lobChIP_FOXA1inPBS
chr14	53393887	53393888	MACS_peak_4458	12.00	lobChIP_FOXA1inPBS
chr14	53401690	53401691	MACS_peak_4459	32.00	lobChIP_FOXA1inPBS
chr14	53414863	53414864	MACS_peak_4460	9.00	lobChIP_FOXA1inPBS
chr14	53908539	53908540	MACS_peak_4461	9.00	lobChIP_FOXA1inPBS
chr14	53917804	53917805	MACS_peak_4462	26.00	lobChIP_FOXA1inPBS
chr14	53925180	53925181	MACS_peak_4463	25.00	lobChIP_FOXA1inPBS
chr14	53940516	53940517	MACS_peak_4464	6.00	lobChIP_FOXA1inPBS
chr14	53945120	53945121	MACS_peak_4465	13.00	lobChIP_FOXA1inPBS
chr14	54080733	54080734	MACS_peak_4466	20.00	lobChIP_FOXA1inPBS
chr14	54082180	54082181	MACS_peak_4467	10.00	lobChIP_FOXA1inPBS
chr14	54170381	54170382	MACS_peak_4468	9.00	lobChIP_FOXA1inPBS
chr14	54233053	54233054	MACS_peak_4469	14.00	lobChIP_FOXA1inPBS
chr14	54260769	54260770	MACS_peak_4470	17.00	lobChIP_FOXA1inPBS
chr14	54309813	54309814	MACS_peak_4471	23.00	lobChIP_FOXA1inPBS
chr14	54318577	54318578	MACS_peak_4472	15.00	lobChIP_FOXA1inPBS
chr14	54322054	54322055	MACS_peak_4473	9.00	lobChIP_FOXA1inPBS
chr14	54348275	54348276	MACS_peak_4474	9.00	lobChIP_FOXA1inPBS
chr14	54375313	54375314	MACS_peak_4475	10.00	lobChIP_FOXA1inPBS
chr14	54394082	54394083	MACS_peak_4476	14.00	lobChIP_FOXA1inPBS
chr14	54401726	54401727	MACS_peak_4477	9.00	lobChIP_FOXA1inPBS
chr14	54409811	54409812	MACS_peak_4478	19.00	lobChIP_FOXA1inPBS
chr14	54414339	54414340	MACS_peak_4479	7.00	lobChIP_FOXA1inPBS
chr14	54427715	54427716	MACS_peak_4480	7.00	lobChIP_FOXA1inPBS
chr14	54468532	54468533	MACS_peak_4481	7.00	lobChIP_FOXA1inPBS
chr14	54513114	54513115	MACS_peak_4482	9.00	lobChIP_FOXA1inPBS
chr14	54514709	54514710	MACS_peak_4483	17.00	lobChIP_FOXA1inPBS
chr14	54515780	54515781	MACS_peak_4484	10.00	lobChIP_FOXA1inPBS
chr14	54555162	54555163	MACS_peak_4485	12.00	lobChIP_FOXA1inPBS
chr14	54569038	54569039	MACS_peak_4486	8.00	lobChIP_FOXA1inPBS
chr14	54575601	54575602	MACS_peak_4487	15.00	lobChIP_FOXA1inPBS
chr14	54584796	54584797	MACS_peak_4488	5.00	lobChIP_FOXA1inPBS
chr14	54600870	54600871	MACS_peak_4489	8.00	lobChIP_FOXA1inPBS
chr14	54602903	54602904	MACS_peak_4490	9.00	lobChIP_FOXA1inPBS
chr14	54604995	54604996	MACS_peak_4491	9.00	lobChIP_FOXA1inPBS
chr14	54605520	54605521	MACS_peak_4492	23.00	lobChIP_FOXA1inPBS
chr14	54664416	54664417	MACS_peak_4493	10.00	lobChIP_FOXA1inPBS
chr14	54810506	54810507	MACS_peak_4494	11.00	lobChIP_FOXA1inPBS
chr14	54819789	54819790	MACS_peak_4495	12.00	lobChIP_FOXA1inPBS
chr14	54820978	54820979	MACS_peak_4496	24.00	lobChIP_FOXA1inPBS
chr14	54827559	54827560	MACS_peak_4497	7.00	lobChIP_FOXA1inPBS
chr14	54908379	54908380	MACS_peak_4498	9.00	lobChIP_FOXA1inPBS
chr14	54914010	54914011	MACS_peak_4499	9.00	lobChIP_FOXA1inPBS
chr14	54950037	54950038	MACS_peak_4500	13.00	lobChIP_FOXA1inPBS
chr14	54956679	54956680	MACS_peak_4501	9.00	lobChIP_FOXA1inPBS
chr14	55004475	55004476	MACS_peak_4502	23.00	lobChIP_FOXA1inPBS
chr14	55110308	55110309	MACS_peak_4503	12.00	lobChIP_FOXA1inPBS
chr14	55114313	55114314	MACS_peak_4504	12.00	lobChIP_FOXA1inPBS
chr14	55117873	55117874	MACS_peak_4505	8.00	lobChIP_FOXA1inPBS
chr14	55123030	55123031	MACS_peak_4506	35.00	lobChIP_FOXA1inPBS
chr14	55168703	55168704	MACS_peak_4507	7.00	lobChIP_FOXA1inPBS
chr14	55304662	55304663	MACS_peak_4508	7.00	lobChIP_FOXA1inPBS
chr14	55306612	55306613	MACS_peak_4509	45.00	lobChIP_FOXA1inPBS
chr14	55355157	55355158	MACS_peak_4510	7.00	lobChIP_FOXA1inPBS
chr14	55411795	55411796	MACS_peak_4511	5.00	lobChIP_FOXA1inPBS
chr14	55823842	55823843	MACS_peak_4512	12.00	lobChIP_FOXA1inPBS
chr14	56059431	56059432	MACS_peak_4513	38.00	lobChIP_FOXA1inPBS
chr14	56065967	56065968	MACS_peak_4514	14.00	lobChIP_FOXA1inPBS
chr14	56066515	56066516	MACS_peak_4515	44.00	lobChIP_FOXA1inPBS
chr14	56071331	56071332	MACS_peak_4516	9.00	lobChIP_FOXA1inPBS
chr14	56072700	56072701	MACS_peak_4517	34.00	lobChIP_FOXA1inPBS
chr14	56298939	56298940	MACS_peak_4518	6.00	lobChIP_FOXA1inPBS
chr14	56392950	56392951	MACS_peak_4519	11.00	lobChIP_FOXA1inPBS
chr14	56584819	56584820	MACS_peak_4520	8.00	lobChIP_FOXA1inPBS
chr14	56691868	56691869	MACS_peak_4521	11.00	lobChIP_FOXA1inPBS
chr14	57022800	57022801	MACS_peak_4522	11.00	lobChIP_FOXA1inPBS
chr14	57050719	57050720	MACS_peak_4523	7.00	lobChIP_FOXA1inPBS
chr14	57126942	57126943	MACS_peak_4524	12.00	lobChIP_FOXA1inPBS
chr14	57524765	57524766	MACS_peak_4525	11.00	lobChIP_FOXA1inPBS
chr14	57759222	57759223	MACS_peak_4526	8.00	lobChIP_FOXA1inPBS
chr14	57813647	57813648	MACS_peak_4527	16.00	lobChIP_FOXA1inPBS
chr14	57816134	57816135	MACS_peak_4528	9.00	lobChIP_FOXA1inPBS
chr14	57858707	57858708	MACS_peak_4529	9.00	lobChIP_FOXA1inPBS
chr14	58739524	58739525	MACS_peak_4530	12.00	lobChIP_FOXA1inPBS
chr14	58754384	58754385	MACS_peak_4531	30.00	lobChIP_FOXA1inPBS
chr14	59480479	59480480	MACS_peak_4532	25.00	lobChIP_FOXA1inPBS
chr14	59594018	59594019	MACS_peak_4533	9.00	lobChIP_FOXA1inPBS
chr14	59680838	59680839	MACS_peak_4534	6.00	lobChIP_FOXA1inPBS
chr14	59729928	59729929	MACS_peak_4535	28.00	lobChIP_FOXA1inPBS
chr14	59750254	59750255	MACS_peak_4536	52.00	lobChIP_FOXA1inPBS
chr14	59763986	59763987	MACS_peak_4537	22.00	lobChIP_FOXA1inPBS
chr14	59772770	59772771	MACS_peak_4538	9.00	lobChIP_FOXA1inPBS
chr14	59781119	59781120	MACS_peak_4539	7.00	lobChIP_FOXA1inPBS
chr14	60755117	60755118	MACS_peak_4540	9.00	lobChIP_FOXA1inPBS
chr14	60981408	60981409	MACS_peak_4541	8.00	lobChIP_FOXA1inPBS
chr14	61083613	61083614	MACS_peak_4542	6.00	lobChIP_FOXA1inPBS
chr14	61110960	61110961	MACS_peak_4543	18.00	lobChIP_FOXA1inPBS
chr14	61132047	61132048	MACS_peak_4544	18.00	lobChIP_FOXA1inPBS
chr14	61132781	61132782	MACS_peak_4545	8.00	lobChIP_FOXA1inPBS
chr14	61153810	61153811	MACS_peak_4546	19.00	lobChIP_FOXA1inPBS
chr14	61257816	61257817	MACS_peak_4547	9.00	lobChIP_FOXA1inPBS
chr14	61282370	61282371	MACS_peak_4548	5.00	lobChIP_FOXA1inPBS
chr14	61286438	61286439	MACS_peak_4549	6.00	lobChIP_FOXA1inPBS
chr14	61431775	61431776	MACS_peak_4550	9.00	lobChIP_FOXA1inPBS
chr14	61509107	61509108	MACS_peak_4551	37.00	lobChIP_FOXA1inPBS
chr14	61569645	61569646	MACS_peak_4552	65.00	lobChIP_FOXA1inPBS
chr14	61896592	61896593	MACS_peak_4553	5.00	lobChIP_FOXA1inPBS
chr14	61905913	61905914	MACS_peak_4554	23.00	lobChIP_FOXA1inPBS
chr14	61958081	61958082	MACS_peak_4555	15.00	lobChIP_FOXA1inPBS
chr14	62001900	62001901	MACS_peak_4556	8.00	lobChIP_FOXA1inPBS
chr14	62003657	62003658	MACS_peak_4557	8.00	lobChIP_FOXA1inPBS
chr14	62004467	62004468	MACS_peak_4558	31.00	lobChIP_FOXA1inPBS
chr14	62020678	62020679	MACS_peak_4559	30.00	lobChIP_FOXA1inPBS
chr14	62022987	62022988	MACS_peak_4560	11.00	lobChIP_FOXA1inPBS
chr14	62027146	62027147	MACS_peak_4561	9.00	lobChIP_FOXA1inPBS
chr14	62048191	62048192	MACS_peak_4562	23.00	lobChIP_FOXA1inPBS
chr14	62075048	62075049	MACS_peak_4563	8.00	lobChIP_FOXA1inPBS
chr14	62178387	62178388	MACS_peak_4564	13.00	lobChIP_FOXA1inPBS
chr14	62180957	62180958	MACS_peak_4565	15.00	lobChIP_FOXA1inPBS
chr14	62181858	62181859	MACS_peak_4566	51.00	lobChIP_FOXA1inPBS
chr14	63156596	63156597	MACS_peak_4567	13.00	lobChIP_FOXA1inPBS
chr14	63159114	63159115	MACS_peak_4568	23.00	lobChIP_FOXA1inPBS
chr14	63267420	63267421	MACS_peak_4569	10.00	lobChIP_FOXA1inPBS
chr14	63513447	63513448	MACS_peak_4570	7.00	lobChIP_FOXA1inPBS
chr14	63938059	63938060	MACS_peak_4571	19.00	lobChIP_FOXA1inPBS
chr14	63948812	63948813	MACS_peak_4572	23.00	lobChIP_FOXA1inPBS
chr14	64032748	64032749	MACS_peak_4573	11.00	lobChIP_FOXA1inPBS
chr14	64193501	64193502	MACS_peak_4574	21.00	lobChIP_FOXA1inPBS
chr14	64215831	64215832	MACS_peak_4575	13.00	lobChIP_FOXA1inPBS
chr14	64390044	64390045	MACS_peak_4576	40.00	lobChIP_FOXA1inPBS
chr14	65115310	65115311	MACS_peak_4577	11.00	lobChIP_FOXA1inPBS
chr14	65405418	65405419	MACS_peak_4578	24.00	lobChIP_FOXA1inPBS
chr14	65409637	65409638	MACS_peak_4579	18.00	lobChIP_FOXA1inPBS
chr14	65486878	65486879	MACS_peak_4580	16.00	lobChIP_FOXA1inPBS
chr14	65661996	65661997	MACS_peak_4581	12.00	lobChIP_FOXA1inPBS
chr14	65692335	65692336	MACS_peak_4582	6.00	lobChIP_FOXA1inPBS
chr14	65693839	65693840	MACS_peak_4583	88.00	lobChIP_FOXA1inPBS
chr14	65698399	65698400	MACS_peak_4584	9.00	lobChIP_FOXA1inPBS
chr14	65705419	65705420	MACS_peak_4585	20.00	lobChIP_FOXA1inPBS
chr14	65709705	65709706	MACS_peak_4586	8.00	lobChIP_FOXA1inPBS
chr14	65710628	65710629	MACS_peak_4587	13.00	lobChIP_FOXA1inPBS
chr14	65721883	65721884	MACS_peak_4588	8.00	lobChIP_FOXA1inPBS
chr14	65725276	65725277	MACS_peak_4589	9.00	lobChIP_FOXA1inPBS
chr14	65735485	65735486	MACS_peak_4590	7.00	lobChIP_FOXA1inPBS
chr14	65745244	65745245	MACS_peak_4591	8.00	lobChIP_FOXA1inPBS
chr14	65764655	65764656	MACS_peak_4592	20.00	lobChIP_FOXA1inPBS
chr14	66060965	66060966	MACS_peak_4593	14.00	lobChIP_FOXA1inPBS
chr14	66106115	66106116	MACS_peak_4594	10.00	lobChIP_FOXA1inPBS
chr14	66957055	66957056	MACS_peak_4595	11.00	lobChIP_FOXA1inPBS
chr14	66968518	66968519	MACS_peak_4596	9.00	lobChIP_FOXA1inPBS
chr14	67385509	67385510	MACS_peak_4597	6.00	lobChIP_FOXA1inPBS
chr14	67399396	67399397	MACS_peak_4598	8.00	lobChIP_FOXA1inPBS
chr14	67778888	67778889	MACS_peak_4599	20.00	lobChIP_FOXA1inPBS
chr14	67780107	67780108	MACS_peak_4600	15.00	lobChIP_FOXA1inPBS
chr14	68056048	68056049	MACS_peak_4601	8.00	lobChIP_FOXA1inPBS
chr14	68165521	68165522	MACS_peak_4602	14.00	lobChIP_FOXA1inPBS
chr14	68177180	68177181	MACS_peak_4603	7.00	lobChIP_FOXA1inPBS
chr14	68183891	68183892	MACS_peak_4604	7.00	lobChIP_FOXA1inPBS
chr14	68205567	68205568	MACS_peak_4605	12.00	lobChIP_FOXA1inPBS
chr14	68207328	68207329	MACS_peak_4606	7.00	lobChIP_FOXA1inPBS
chr14	68230300	68230301	MACS_peak_4607	26.00	lobChIP_FOXA1inPBS
chr14	68311775	68311776	MACS_peak_4608	7.00	lobChIP_FOXA1inPBS
chr14	68450108	68450109	MACS_peak_4609	12.00	lobChIP_FOXA1inPBS
chr14	68631711	68631712	MACS_peak_4610	19.00	lobChIP_FOXA1inPBS
chr14	68639057	68639058	MACS_peak_4611	43.00	lobChIP_FOXA1inPBS
chr14	68662019	68662020	MACS_peak_4612	14.00	lobChIP_FOXA1inPBS
chr14	68721991	68721992	MACS_peak_4613	61.00	lobChIP_FOXA1inPBS
chr14	68745829	68745830	MACS_peak_4614	15.00	lobChIP_FOXA1inPBS
chr14	68761783	68761784	MACS_peak_4615	17.00	lobChIP_FOXA1inPBS
chr14	68762722	68762723	MACS_peak_4616	6.00	lobChIP_FOXA1inPBS
chr14	68783383	68783384	MACS_peak_4617	27.00	lobChIP_FOXA1inPBS
chr14	68816786	68816787	MACS_peak_4618	50.00	lobChIP_FOXA1inPBS
chr14	68926053	68926054	MACS_peak_4619	11.00	lobChIP_FOXA1inPBS
chr14	68930508	68930509	MACS_peak_4620	10.00	lobChIP_FOXA1inPBS
chr14	68936318	68936319	MACS_peak_4621	83.00	lobChIP_FOXA1inPBS
chr14	68953320	68953321	MACS_peak_4622	14.00	lobChIP_FOXA1inPBS
chr14	69030187	69030188	MACS_peak_4623	14.00	lobChIP_FOXA1inPBS
chr14	69036736	69036737	MACS_peak_4624	14.00	lobChIP_FOXA1inPBS
chr14	69044729	69044730	MACS_peak_4625	15.00	lobChIP_FOXA1inPBS
chr14	69105217	69105218	MACS_peak_4626	8.00	lobChIP_FOXA1inPBS
chr14	69183566	69183567	MACS_peak_4627	13.00	lobChIP_FOXA1inPBS
chr14	69190622	69190623	MACS_peak_4628	8.00	lobChIP_FOXA1inPBS
chr14	69195135	69195136	MACS_peak_4629	13.00	lobChIP_FOXA1inPBS
chr14	69221572	69221573	MACS_peak_4630	7.00	lobChIP_FOXA1inPBS
chr14	69229749	69229750	MACS_peak_4631	12.00	lobChIP_FOXA1inPBS
chr14	69245541	69245542	MACS_peak_4632	9.00	lobChIP_FOXA1inPBS
chr14	69250744	69250745	MACS_peak_4633	13.00	lobChIP_FOXA1inPBS
chr14	69263281	69263282	MACS_peak_4634	9.00	lobChIP_FOXA1inPBS
chr14	69480883	69480884	MACS_peak_4635	8.00	lobChIP_FOXA1inPBS
chr14	69560808	69560809	MACS_peak_4636	10.00	lobChIP_FOXA1inPBS
chr14	69823805	69823806	MACS_peak_4637	11.00	lobChIP_FOXA1inPBS
chr14	69839088	69839089	MACS_peak_4638	18.00	lobChIP_FOXA1inPBS
chr14	69891025	69891026	MACS_peak_4639	15.00	lobChIP_FOXA1inPBS
chr14	69897223	69897224	MACS_peak_4640	12.00	lobChIP_FOXA1inPBS
chr14	70110314	70110315	MACS_peak_4641	10.00	lobChIP_FOXA1inPBS
chr14	70124937	70124938	MACS_peak_4642	13.00	lobChIP_FOXA1inPBS
chr14	70157072	70157073	MACS_peak_4643	22.00	lobChIP_FOXA1inPBS
chr14	70208003	70208004	MACS_peak_4644	10.00	lobChIP_FOXA1inPBS
chr14	70259811	70259812	MACS_peak_4645	15.00	lobChIP_FOXA1inPBS
chr14	70317546	70317547	MACS_peak_4646	19.00	lobChIP_FOXA1inPBS
chr14	70322854	70322855	MACS_peak_4647	7.00	lobChIP_FOXA1inPBS
chr14	70343623	70343624	MACS_peak_4648	12.00	lobChIP_FOXA1inPBS
chr14	70469511	70469512	MACS_peak_4649	7.00	lobChIP_FOXA1inPBS
chr14	70471546	70471547	MACS_peak_4650	6.00	lobChIP_FOXA1inPBS
chr14	70472276	70472277	MACS_peak_4651	9.00	lobChIP_FOXA1inPBS
chr14	70514764	70514765	MACS_peak_4652	21.00	lobChIP_FOXA1inPBS
chr14	70785225	70785226	MACS_peak_4653	22.00	lobChIP_FOXA1inPBS
chr14	70915605	70915606	MACS_peak_4654	25.00	lobChIP_FOXA1inPBS
chr14	71183425	71183426	MACS_peak_4655	11.00	lobChIP_FOXA1inPBS
chr14	71278499	71278500	MACS_peak_4656	8.00	lobChIP_FOXA1inPBS
chr14	71431455	71431456	MACS_peak_4657	18.00	lobChIP_FOXA1inPBS
chr14	71486072	71486073	MACS_peak_4658	11.00	lobChIP_FOXA1inPBS
chr14	71529803	71529804	MACS_peak_4659	9.00	lobChIP_FOXA1inPBS
chr14	71570791	71570792	MACS_peak_4660	16.00	lobChIP_FOXA1inPBS
chr14	71583654	71583655	MACS_peak_4661	11.00	lobChIP_FOXA1inPBS
chr14	73243728	73243729	MACS_peak_4662	35.00	lobChIP_FOXA1inPBS
chr14	73248728	73248729	MACS_peak_4663	7.00	lobChIP_FOXA1inPBS
chr14	73613631	73613632	MACS_peak_4664	13.00	lobChIP_FOXA1inPBS
chr14	73616667	73616668	MACS_peak_4665	9.00	lobChIP_FOXA1inPBS
chr14	73871901	73871902	MACS_peak_4666	6.00	lobChIP_FOXA1inPBS
chr14	73895155	73895156	MACS_peak_4667	8.00	lobChIP_FOXA1inPBS
chr14	73899043	73899044	MACS_peak_4668	20.00	lobChIP_FOXA1inPBS
chr14	74080506	74080507	MACS_peak_4669	23.00	lobChIP_FOXA1inPBS
chr14	74130714	74130715	MACS_peak_4670	13.00	lobChIP_FOXA1inPBS
chr14	74197959	74197960	MACS_peak_4671	8.00	lobChIP_FOXA1inPBS
chr14	74214667	74214668	MACS_peak_4672	15.00	lobChIP_FOXA1inPBS
chr14	74230345	74230346	MACS_peak_4673	25.00	lobChIP_FOXA1inPBS
chr14	74252706	74252707	MACS_peak_4674	13.00	lobChIP_FOXA1inPBS
chr14	74310956	74310957	MACS_peak_4675	15.00	lobChIP_FOXA1inPBS
chr14	74464054	74464055	MACS_peak_4676	11.00	lobChIP_FOXA1inPBS
chr14	74495140	74495141	MACS_peak_4677	17.00	lobChIP_FOXA1inPBS
chr14	74716526	74716527	MACS_peak_4678	8.00	lobChIP_FOXA1inPBS
chr14	74769201	74769202	MACS_peak_4679	10.00	lobChIP_FOXA1inPBS
chr14	75100193	75100194	MACS_peak_4680	18.00	lobChIP_FOXA1inPBS
chr14	75288422	75288423	MACS_peak_4681	8.00	lobChIP_FOXA1inPBS
chr14	75412579	75412580	MACS_peak_4682	12.00	lobChIP_FOXA1inPBS
chr14	75620121	75620122	MACS_peak_4683	13.00	lobChIP_FOXA1inPBS
chr14	75641981	75641982	MACS_peak_4684	7.00	lobChIP_FOXA1inPBS
chr14	75663422	75663423	MACS_peak_4685	46.00	lobChIP_FOXA1inPBS
chr14	75795536	75795537	MACS_peak_4686	10.00	lobChIP_FOXA1inPBS
chr14	75919956	75919957	MACS_peak_4687	12.00	lobChIP_FOXA1inPBS
chr14	75987972	75987973	MACS_peak_4688	13.00	lobChIP_FOXA1inPBS
chr14	76000343	76000344	MACS_peak_4689	12.00	lobChIP_FOXA1inPBS
chr14	76002474	76002475	MACS_peak_4690	9.00	lobChIP_FOXA1inPBS
chr14	76110511	76110512	MACS_peak_4691	9.00	lobChIP_FOXA1inPBS
chr14	76114848	76114849	MACS_peak_4692	20.00	lobChIP_FOXA1inPBS
chr14	76116708	76116709	MACS_peak_4693	14.00	lobChIP_FOXA1inPBS
chr14	76117274	76117275	MACS_peak_4694	11.00	lobChIP_FOXA1inPBS
chr14	76178120	76178121	MACS_peak_4695	13.00	lobChIP_FOXA1inPBS
chr14	76292586	76292587	MACS_peak_4696	12.00	lobChIP_FOXA1inPBS
chr14	76324898	76324899	MACS_peak_4697	26.00	lobChIP_FOXA1inPBS
chr14	77120400	77120401	MACS_peak_4698	16.00	lobChIP_FOXA1inPBS
chr14	77154417	77154418	MACS_peak_4699	20.00	lobChIP_FOXA1inPBS
chr14	77435197	77435198	MACS_peak_4700	18.00	lobChIP_FOXA1inPBS
chr14	77490045	77490046	MACS_peak_4701	7.00	lobChIP_FOXA1inPBS
chr14	77495255	77495256	MACS_peak_4702	11.00	lobChIP_FOXA1inPBS
chr14	77500695	77500696	MACS_peak_4703	63.00	lobChIP_FOXA1inPBS
chr14	77767592	77767593	MACS_peak_4704	8.00	lobChIP_FOXA1inPBS
chr14	77781229	77781230	MACS_peak_4705	7.00	lobChIP_FOXA1inPBS
chr14	77968601	77968602	MACS_peak_4706	39.00	lobChIP_FOXA1inPBS
chr14	80469675	80469676	MACS_peak_4707	57.00	lobChIP_FOXA1inPBS
chr14	80929349	80929350	MACS_peak_4708	5.00	lobChIP_FOXA1inPBS
chr14	80935131	80935132	MACS_peak_4709	148.00	lobChIP_FOXA1inPBS
chr14	81044153	81044154	MACS_peak_4710	9.00	lobChIP_FOXA1inPBS
chr14	81562681	81562682	MACS_peak_4711	12.00	lobChIP_FOXA1inPBS
chr14	81651608	81651609	MACS_peak_4712	12.00	lobChIP_FOXA1inPBS
chr14	81685848	81685849	MACS_peak_4713	9.00	lobChIP_FOXA1inPBS
chr14	81743200	81743201	MACS_peak_4714	8.00	lobChIP_FOXA1inPBS
chr14	81769675	81769676	MACS_peak_4715	15.00	lobChIP_FOXA1inPBS
chr14	81785903	81785904	MACS_peak_4716	7.00	lobChIP_FOXA1inPBS
chr14	81983926	81983927	MACS_peak_4717	32.00	lobChIP_FOXA1inPBS
chr14	82003737	82003738	MACS_peak_4718	14.00	lobChIP_FOXA1inPBS
chr14	82452684	82452685	MACS_peak_4719	8.00	lobChIP_FOXA1inPBS
chr14	85109924	85109925	MACS_peak_4720	7.00	lobChIP_FOXA1inPBS
chr14	85938328	85938329	MACS_peak_4721	15.00	lobChIP_FOXA1inPBS
chr14	85949108	85949109	MACS_peak_4722	11.00	lobChIP_FOXA1inPBS
chr14	85986390	85986391	MACS_peak_4723	17.00	lobChIP_FOXA1inPBS
chr14	86480160	86480161	MACS_peak_4724	13.00	lobChIP_FOXA1inPBS
chr14	87175345	87175346	MACS_peak_4725	11.00	lobChIP_FOXA1inPBS
chr14	87189468	87189469	MACS_peak_4726	13.00	lobChIP_FOXA1inPBS
chr14	87358945	87358946	MACS_peak_4727	15.00	lobChIP_FOXA1inPBS
chr14	87364086	87364087	MACS_peak_4728	12.00	lobChIP_FOXA1inPBS
chr14	87900544	87900545	MACS_peak_4729	16.00	lobChIP_FOXA1inPBS
chr14	88490874	88490875	MACS_peak_4730	21.00	lobChIP_FOXA1inPBS
chr14	88498030	88498031	MACS_peak_4731	5.00	lobChIP_FOXA1inPBS
chr14	88510693	88510694	MACS_peak_4732	59.00	lobChIP_FOXA1inPBS
chr14	88855216	88855217	MACS_peak_4733	7.00	lobChIP_FOXA1inPBS
chr14	88877522	88877523	MACS_peak_4734	12.00	lobChIP_FOXA1inPBS
chr14	88958782	88958783	MACS_peak_4735	16.00	lobChIP_FOXA1inPBS
chr14	89019380	89019381	MACS_peak_4736	14.00	lobChIP_FOXA1inPBS
chr14	89030766	89030767	MACS_peak_4737	10.00	lobChIP_FOXA1inPBS
chr14	89080216	89080217	MACS_peak_4738	24.00	lobChIP_FOXA1inPBS
chr14	89099072	89099073	MACS_peak_4739	9.00	lobChIP_FOXA1inPBS
chr14	89507080	89507081	MACS_peak_4740	5.00	lobChIP_FOXA1inPBS
chr14	89507728	89507729	MACS_peak_4741	17.00	lobChIP_FOXA1inPBS
chr14	89571002	89571003	MACS_peak_4742	13.00	lobChIP_FOXA1inPBS
chr14	89626998	89626999	MACS_peak_4743	8.00	lobChIP_FOXA1inPBS
chr14	89642595	89642596	MACS_peak_4744	14.00	lobChIP_FOXA1inPBS
chr14	89680765	89680766	MACS_peak_4745	18.00	lobChIP_FOXA1inPBS
chr14	89698947	89698948	MACS_peak_4746	28.00	lobChIP_FOXA1inPBS
chr14	89714226	89714227	MACS_peak_4747	29.00	lobChIP_FOXA1inPBS
chr14	89818146	89818147	MACS_peak_4748	101.00	lobChIP_FOXA1inPBS
chr14	89925276	89925277	MACS_peak_4749	12.00	lobChIP_FOXA1inPBS
chr14	89960288	89960289	MACS_peak_4750	11.00	lobChIP_FOXA1inPBS
chr14	90006556	90006557	MACS_peak_4751	15.00	lobChIP_FOXA1inPBS
chr14	90055530	90055531	MACS_peak_4752	20.00	lobChIP_FOXA1inPBS
chr14	90108006	90108007	MACS_peak_4753	6.00	lobChIP_FOXA1inPBS
chr14	90377512	90377513	MACS_peak_4754	23.00	lobChIP_FOXA1inPBS
chr14	90421227	90421228	MACS_peak_4755	6.00	lobChIP_FOXA1inPBS
chr14	90422122	90422123	MACS_peak_4756	22.00	lobChIP_FOXA1inPBS
chr14	90980059	90980060	MACS_peak_4757	53.00	lobChIP_FOXA1inPBS
chr14	91616003	91616004	MACS_peak_4758	8.00	lobChIP_FOXA1inPBS
chr14	91622808	91622809	MACS_peak_4759	39.00	lobChIP_FOXA1inPBS
chr14	91623688	91623689	MACS_peak_4760	9.00	lobChIP_FOXA1inPBS
chr14	91643812	91643813	MACS_peak_4761	8.00	lobChIP_FOXA1inPBS
chr14	91855692	91855693	MACS_peak_4762	9.00	lobChIP_FOXA1inPBS
chr14	92424376	92424377	MACS_peak_4763	12.00	lobChIP_FOXA1inPBS
chr14	92601784	92601785	MACS_peak_4764	8.00	lobChIP_FOXA1inPBS
chr14	92814695	92814696	MACS_peak_4765	16.00	lobChIP_FOXA1inPBS
chr14	93023641	93023642	MACS_peak_4766	16.00	lobChIP_FOXA1inPBS
chr14	93160520	93160521	MACS_peak_4767	12.00	lobChIP_FOXA1inPBS
chr14	93372266	93372267	MACS_peak_4768	8.00	lobChIP_FOXA1inPBS
chr14	93451175	93451176	MACS_peak_4769	16.00	lobChIP_FOXA1inPBS
chr14	93452762	93452763	MACS_peak_4770	7.00	lobChIP_FOXA1inPBS
chr14	93546561	93546562	MACS_peak_4771	10.00	lobChIP_FOXA1inPBS
chr14	93617695	93617696	MACS_peak_4772	10.00	lobChIP_FOXA1inPBS
chr14	93644546	93644547	MACS_peak_4773	31.00	lobChIP_FOXA1inPBS
chr14	93799924	93799925	MACS_peak_4774	6.00	lobChIP_FOXA1inPBS
chr14	94244953	94244954	MACS_peak_4775	36.00	lobChIP_FOXA1inPBS
chr14	94256853	94256854	MACS_peak_4776	6.00	lobChIP_FOXA1inPBS
chr14	94266583	94266584	MACS_peak_4777	6.00	lobChIP_FOXA1inPBS
chr14	94370007	94370008	MACS_peak_4778	9.00	lobChIP_FOXA1inPBS
chr14	94402555	94402556	MACS_peak_4779	8.00	lobChIP_FOXA1inPBS
chr14	94494885	94494886	MACS_peak_4780	9.00	lobChIP_FOXA1inPBS
chr14	94643602	94643603	MACS_peak_4781	8.00	lobChIP_FOXA1inPBS
chr14	94786783	94786784	MACS_peak_4782	18.00	lobChIP_FOXA1inPBS
chr14	94789849	94789850	MACS_peak_4783	21.00	lobChIP_FOXA1inPBS
chr14	94806259	94806260	MACS_peak_4784	12.00	lobChIP_FOXA1inPBS
chr14	94806981	94806982	MACS_peak_4785	21.00	lobChIP_FOXA1inPBS
chr14	94842204	94842205	MACS_peak_4786	9.00	lobChIP_FOXA1inPBS
chr14	94853178	94853179	MACS_peak_4787	10.00	lobChIP_FOXA1inPBS
chr14	94862434	94862435	MACS_peak_4788	48.00	lobChIP_FOXA1inPBS
chr14	94866823	94866824	MACS_peak_4789	9.00	lobChIP_FOXA1inPBS
chr14	94919179	94919180	MACS_peak_4790	27.00	lobChIP_FOXA1inPBS
chr14	94922530	94922531	MACS_peak_4791	15.00	lobChIP_FOXA1inPBS
chr14	94929735	94929736	MACS_peak_4792	23.00	lobChIP_FOXA1inPBS
chr14	94932281	94932282	MACS_peak_4793	9.00	lobChIP_FOXA1inPBS
chr14	94968410	94968411	MACS_peak_4794	38.00	lobChIP_FOXA1inPBS
chr14	94988777	94988778	MACS_peak_4795	21.00	lobChIP_FOXA1inPBS
chr14	95030814	95030815	MACS_peak_4796	20.00	lobChIP_FOXA1inPBS
chr14	95039218	95039219	MACS_peak_4797	26.00	lobChIP_FOXA1inPBS
chr14	95060458	95060459	MACS_peak_4798	29.00	lobChIP_FOXA1inPBS
chr14	95067374	95067375	MACS_peak_4799	42.00	lobChIP_FOXA1inPBS
chr14	95103344	95103345	MACS_peak_4800	32.00	lobChIP_FOXA1inPBS
chr14	95293309	95293310	MACS_peak_4801	11.00	lobChIP_FOXA1inPBS
chr14	95740121	95740122	MACS_peak_4802	6.00	lobChIP_FOXA1inPBS
chr14	95762056	95762057	MACS_peak_4803	14.00	lobChIP_FOXA1inPBS
chr14	95767492	95767493	MACS_peak_4804	8.00	lobChIP_FOXA1inPBS
chr14	95806115	95806116	MACS_peak_4805	8.00	lobChIP_FOXA1inPBS
chr14	95865421	95865422	MACS_peak_4806	13.00	lobChIP_FOXA1inPBS
chr14	95910578	95910579	MACS_peak_4807	18.00	lobChIP_FOXA1inPBS
chr14	95989668	95989669	MACS_peak_4808	22.00	lobChIP_FOXA1inPBS
chr14	95991399	95991400	MACS_peak_4809	30.00	lobChIP_FOXA1inPBS
chr14	96003919	96003920	MACS_peak_4810	23.00	lobChIP_FOXA1inPBS
chr14	96662249	96662250	MACS_peak_4811	14.00	lobChIP_FOXA1inPBS
chr14	96828670	96828671	MACS_peak_4812	10.00	lobChIP_FOXA1inPBS
chr14	96928688	96928689	MACS_peak_4813	26.00	lobChIP_FOXA1inPBS
chr14	96972846	96972847	MACS_peak_4814	6.00	lobChIP_FOXA1inPBS
chr14	96975353	96975354	MACS_peak_4815	8.00	lobChIP_FOXA1inPBS
chr14	97005635	97005636	MACS_peak_4816	7.00	lobChIP_FOXA1inPBS
chr14	99782679	99782680	MACS_peak_4817	24.00	lobChIP_FOXA1inPBS
chr14	99977610	99977611	MACS_peak_4818	6.00	lobChIP_FOXA1inPBS
chr14	100016282	100016283	MACS_peak_4819	11.00	lobChIP_FOXA1inPBS
chr14	100021418	100021419	MACS_peak_4820	7.00	lobChIP_FOXA1inPBS
chr14	100250943	100250944	MACS_peak_4821	22.00	lobChIP_FOXA1inPBS
chr14	100305293	100305294	MACS_peak_4822	7.00	lobChIP_FOXA1inPBS
chr14	100597673	100597674	MACS_peak_4823	7.00	lobChIP_FOXA1inPBS
chr14	100671053	100671054	MACS_peak_4824	12.00	lobChIP_FOXA1inPBS
chr14	100737776	100737777	MACS_peak_4825	18.00	lobChIP_FOXA1inPBS
chr14	100942773	100942774	MACS_peak_4826	7.00	lobChIP_FOXA1inPBS
chr14	101139663	101139664	MACS_peak_4827	18.00	lobChIP_FOXA1inPBS
chr14	101216759	101216760	MACS_peak_4828	21.00	lobChIP_FOXA1inPBS
chr14	101217541	101217542	MACS_peak_4829	11.00	lobChIP_FOXA1inPBS
chr14	101220173	101220174	MACS_peak_4830	10.00	lobChIP_FOXA1inPBS
chr14	101220579	101220580	MACS_peak_4831	8.00	lobChIP_FOXA1inPBS
chr14	101228636	101228637	MACS_peak_4832	20.00	lobChIP_FOXA1inPBS
chr14	101249332	101249333	MACS_peak_4833	6.00	lobChIP_FOXA1inPBS
chr14	101282555	101282556	MACS_peak_4834	7.00	lobChIP_FOXA1inPBS
chr14	101283818	101283819	MACS_peak_4835	46.00	lobChIP_FOXA1inPBS
chr14	101359602	101359603	MACS_peak_4836	8.00	lobChIP_FOXA1inPBS
chr14	101366755	101366756	MACS_peak_4837	10.00	lobChIP_FOXA1inPBS
chr14	101507366	101507367	MACS_peak_4838	46.00	lobChIP_FOXA1inPBS
chr14	101693082	101693083	MACS_peak_4839	10.00	lobChIP_FOXA1inPBS
chr14	101705613	101705614	MACS_peak_4840	11.00	lobChIP_FOXA1inPBS
chr14	101754996	101754997	MACS_peak_4841	12.00	lobChIP_FOXA1inPBS
chr14	101755833	101755834	MACS_peak_4842	26.00	lobChIP_FOXA1inPBS
chr14	101791827	101791828	MACS_peak_4843	7.00	lobChIP_FOXA1inPBS
chr14	101801881	101801882	MACS_peak_4844	33.00	lobChIP_FOXA1inPBS
chr14	101879219	101879220	MACS_peak_4845	17.00	lobChIP_FOXA1inPBS
chr14	102275714	102275715	MACS_peak_4846	7.00	lobChIP_FOXA1inPBS
chr14	102651969	102651970	MACS_peak_4847	19.00	lobChIP_FOXA1inPBS
chr14	102909210	102909211	MACS_peak_4848	13.00	lobChIP_FOXA1inPBS
chr14	102931395	102931396	MACS_peak_4849	10.00	lobChIP_FOXA1inPBS
chr14	102948349	102948350	MACS_peak_4850	25.00	lobChIP_FOXA1inPBS
chr14	103150821	103150822	MACS_peak_4851	19.00	lobChIP_FOXA1inPBS
chr14	103157445	103157446	MACS_peak_4852	37.00	lobChIP_FOXA1inPBS
chr14	103192762	103192763	MACS_peak_4853	14.00	lobChIP_FOXA1inPBS
chr14	103261594	103261595	MACS_peak_4854	15.00	lobChIP_FOXA1inPBS
chr14	103324311	103324312	MACS_peak_4855	7.00	lobChIP_FOXA1inPBS
chr14	103340053	103340054	MACS_peak_4856	44.00	lobChIP_FOXA1inPBS
chr14	103567115	103567116	MACS_peak_4857	7.00	lobChIP_FOXA1inPBS
chr14	103570142	103570143	MACS_peak_4858	11.00	lobChIP_FOXA1inPBS
chr14	103679928	103679929	MACS_peak_4859	9.00	lobChIP_FOXA1inPBS
chr14	103813940	103813941	MACS_peak_4860	7.00	lobChIP_FOXA1inPBS
chr14	104329043	104329044	MACS_peak_4861	9.00	lobChIP_FOXA1inPBS
chr14	105131728	105131729	MACS_peak_4862	6.00	lobChIP_FOXA1inPBS
chr14	105132303	105132304	MACS_peak_4863	7.00	lobChIP_FOXA1inPBS
chr14	105213335	105213336	MACS_peak_4864	13.00	lobChIP_FOXA1inPBS
chr15	23028056	23028057	MACS_peak_4865	11.00	lobChIP_FOXA1inPBS
chr15	23161173	23161174	MACS_peak_4866	7.00	lobChIP_FOXA1inPBS
chr15	23620320	23620321	MACS_peak_4867	6.00	lobChIP_FOXA1inPBS
chr15	24101263	24101264	MACS_peak_4868	11.00	lobChIP_FOXA1inPBS
chr15	25102379	25102380	MACS_peak_4869	8.00	lobChIP_FOXA1inPBS
chr15	25208237	25208238	MACS_peak_4870	13.00	lobChIP_FOXA1inPBS
chr15	25682114	25682115	MACS_peak_4871	8.00	lobChIP_FOXA1inPBS
chr15	28489350	28489351	MACS_peak_4872	9.00	lobChIP_FOXA1inPBS
chr15	29693889	29693890	MACS_peak_4873	7.00	lobChIP_FOXA1inPBS
chr15	29731645	29731646	MACS_peak_4874	12.00	lobChIP_FOXA1inPBS
chr15	29991882	29991883	MACS_peak_4875	16.00	lobChIP_FOXA1inPBS
chr15	30115459	30115460	MACS_peak_4876	12.00	lobChIP_FOXA1inPBS
chr15	31381094	31381095	MACS_peak_4877	15.00	lobChIP_FOXA1inPBS
chr15	31450048	31450049	MACS_peak_4878	31.00	lobChIP_FOXA1inPBS
chr15	31566759	31566760	MACS_peak_4879	8.00	lobChIP_FOXA1inPBS
chr15	31586032	31586033	MACS_peak_4880	25.00	lobChIP_FOXA1inPBS
chr15	31588898	31588899	MACS_peak_4881	18.00	lobChIP_FOXA1inPBS
chr15	31697973	31697974	MACS_peak_4882	19.00	lobChIP_FOXA1inPBS
chr15	31891084	31891085	MACS_peak_4883	10.00	lobChIP_FOXA1inPBS
chr15	32952048	32952049	MACS_peak_4884	6.00	lobChIP_FOXA1inPBS
chr15	32962639	32962640	MACS_peak_4885	9.00	lobChIP_FOXA1inPBS
chr15	32969647	32969648	MACS_peak_4886	11.00	lobChIP_FOXA1inPBS
chr15	32980500	32980501	MACS_peak_4887	12.00	lobChIP_FOXA1inPBS
chr15	32992939	32992940	MACS_peak_4888	20.00	lobChIP_FOXA1inPBS
chr15	33131196	33131197	MACS_peak_4889	28.00	lobChIP_FOXA1inPBS
chr15	33132117	33132118	MACS_peak_4890	10.00	lobChIP_FOXA1inPBS
chr15	33145314	33145315	MACS_peak_4891	9.00	lobChIP_FOXA1inPBS
chr15	34450684	34450685	MACS_peak_4892	6.00	lobChIP_FOXA1inPBS
chr15	34864663	34864664	MACS_peak_4893	9.00	lobChIP_FOXA1inPBS
chr15	35272537	35272538	MACS_peak_4894	7.00	lobChIP_FOXA1inPBS
chr15	35352118	35352119	MACS_peak_4895	17.00	lobChIP_FOXA1inPBS
chr15	35382546	35382547	MACS_peak_4896	10.00	lobChIP_FOXA1inPBS
chr15	35798689	35798690	MACS_peak_4897	13.00	lobChIP_FOXA1inPBS
chr15	36799133	36799134	MACS_peak_4898	126.00	lobChIP_FOXA1inPBS
chr15	37076778	37076779	MACS_peak_4899	10.00	lobChIP_FOXA1inPBS
chr15	37301644	37301645	MACS_peak_4900	11.00	lobChIP_FOXA1inPBS
chr15	37312050	37312051	MACS_peak_4901	16.00	lobChIP_FOXA1inPBS
chr15	37338320	37338321	MACS_peak_4902	5.00	lobChIP_FOXA1inPBS
chr15	37511692	37511693	MACS_peak_4903	11.00	lobChIP_FOXA1inPBS
chr15	38002110	38002111	MACS_peak_4904	23.00	lobChIP_FOXA1inPBS
chr15	38509181	38509182	MACS_peak_4905	15.00	lobChIP_FOXA1inPBS
chr15	38543951	38543952	MACS_peak_4906	6.00	lobChIP_FOXA1inPBS
chr15	38546890	38546891	MACS_peak_4907	7.00	lobChIP_FOXA1inPBS
chr15	38732722	38732723	MACS_peak_4908	10.00	lobChIP_FOXA1inPBS
chr15	38747069	38747070	MACS_peak_4909	9.00	lobChIP_FOXA1inPBS
chr15	39165603	39165604	MACS_peak_4910	14.00	lobChIP_FOXA1inPBS
chr15	39503848	39503849	MACS_peak_4911	10.00	lobChIP_FOXA1inPBS
chr15	39512017	39512018	MACS_peak_4912	12.00	lobChIP_FOXA1inPBS
chr15	39566251	39566252	MACS_peak_4913	57.00	lobChIP_FOXA1inPBS
chr15	39584020	39584021	MACS_peak_4914	7.00	lobChIP_FOXA1inPBS
chr15	39652756	39652757	MACS_peak_4915	9.00	lobChIP_FOXA1inPBS
chr15	39656163	39656164	MACS_peak_4916	19.00	lobChIP_FOXA1inPBS
chr15	39727346	39727347	MACS_peak_4917	92.00	lobChIP_FOXA1inPBS
chr15	39740765	39740766	MACS_peak_4918	10.00	lobChIP_FOXA1inPBS
chr15	39748062	39748063	MACS_peak_4919	35.00	lobChIP_FOXA1inPBS
chr15	40079904	40079905	MACS_peak_4920	14.00	lobChIP_FOXA1inPBS
chr15	40294667	40294668	MACS_peak_4921	9.00	lobChIP_FOXA1inPBS
chr15	40374000	40374001	MACS_peak_4922	8.00	lobChIP_FOXA1inPBS
chr15	40376087	40376088	MACS_peak_4923	11.00	lobChIP_FOXA1inPBS
chr15	40384686	40384687	MACS_peak_4924	11.00	lobChIP_FOXA1inPBS
chr15	40391157	40391158	MACS_peak_4925	27.00	lobChIP_FOXA1inPBS
chr15	40398901	40398902	MACS_peak_4926	12.00	lobChIP_FOXA1inPBS
chr15	40942256	40942257	MACS_peak_4927	9.00	lobChIP_FOXA1inPBS
chr15	41085986	41085987	MACS_peak_4928	6.00	lobChIP_FOXA1inPBS
chr15	41115952	41115953	MACS_peak_4929	33.00	lobChIP_FOXA1inPBS
chr15	41138363	41138364	MACS_peak_4930	12.00	lobChIP_FOXA1inPBS
chr15	41144865	41144866	MACS_peak_4931	13.00	lobChIP_FOXA1inPBS
chr15	41225024	41225025	MACS_peak_4932	9.00	lobChIP_FOXA1inPBS
chr15	41237280	41237281	MACS_peak_4933	33.00	lobChIP_FOXA1inPBS
chr15	41417647	41417648	MACS_peak_4934	17.00	lobChIP_FOXA1inPBS
chr15	41420906	41420907	MACS_peak_4935	15.00	lobChIP_FOXA1inPBS
chr15	41460533	41460534	MACS_peak_4936	6.00	lobChIP_FOXA1inPBS
chr15	41534154	41534155	MACS_peak_4937	10.00	lobChIP_FOXA1inPBS
chr15	41548834	41548835	MACS_peak_4938	18.00	lobChIP_FOXA1inPBS
chr15	41555013	41555014	MACS_peak_4939	8.00	lobChIP_FOXA1inPBS
chr15	41788326	41788327	MACS_peak_4940	15.00	lobChIP_FOXA1inPBS
chr15	41860771	41860772	MACS_peak_4941	20.00	lobChIP_FOXA1inPBS
chr15	41976376	41976377	MACS_peak_4942	9.00	lobChIP_FOXA1inPBS
chr15	42220428	42220429	MACS_peak_4943	25.00	lobChIP_FOXA1inPBS
chr15	42253323	42253324	MACS_peak_4944	12.00	lobChIP_FOXA1inPBS
chr15	42339018	42339019	MACS_peak_4945	10.00	lobChIP_FOXA1inPBS
chr15	42431395	42431396	MACS_peak_4946	8.00	lobChIP_FOXA1inPBS
chr15	42485956	42485957	MACS_peak_4947	8.00	lobChIP_FOXA1inPBS
chr15	42486927	42486928	MACS_peak_4948	9.00	lobChIP_FOXA1inPBS
chr15	42519065	42519066	MACS_peak_4949	6.00	lobChIP_FOXA1inPBS
chr15	42565710	42565711	MACS_peak_4950	9.00	lobChIP_FOXA1inPBS
chr15	42578849	42578850	MACS_peak_4951	15.00	lobChIP_FOXA1inPBS
chr15	42883970	42883971	MACS_peak_4952	10.00	lobChIP_FOXA1inPBS
chr15	42894864	42894865	MACS_peak_4953	9.00	lobChIP_FOXA1inPBS
chr15	43029923	43029924	MACS_peak_4954	8.00	lobChIP_FOXA1inPBS
chr15	43264232	43264233	MACS_peak_4955	10.00	lobChIP_FOXA1inPBS
chr15	44061992	44061993	MACS_peak_4956	29.00	lobChIP_FOXA1inPBS
chr15	44450044	44450045	MACS_peak_4957	10.00	lobChIP_FOXA1inPBS
chr15	44720649	44720650	MACS_peak_4958	10.00	lobChIP_FOXA1inPBS
chr15	44880952	44880953	MACS_peak_4959	13.00	lobChIP_FOXA1inPBS
chr15	44975618	44975619	MACS_peak_4960	10.00	lobChIP_FOXA1inPBS
chr15	45019068	45019069	MACS_peak_4961	6.00	lobChIP_FOXA1inPBS
chr15	45104490	45104491	MACS_peak_4962	14.00	lobChIP_FOXA1inPBS
chr15	45123335	45123336	MACS_peak_4963	8.00	lobChIP_FOXA1inPBS
chr15	45128534	45128535	MACS_peak_4964	10.00	lobChIP_FOXA1inPBS
chr15	45145621	45145622	MACS_peak_4965	8.00	lobChIP_FOXA1inPBS
chr15	45205330	45205331	MACS_peak_4966	39.00	lobChIP_FOXA1inPBS
chr15	45272099	45272100	MACS_peak_4967	21.00	lobChIP_FOXA1inPBS
chr15	45312611	45312612	MACS_peak_4968	9.00	lobChIP_FOXA1inPBS
chr15	45626895	45626896	MACS_peak_4969	10.00	lobChIP_FOXA1inPBS
chr15	45630865	45630866	MACS_peak_4970	12.00	lobChIP_FOXA1inPBS
chr15	45677980	45677981	MACS_peak_4971	8.00	lobChIP_FOXA1inPBS
chr15	45679766	45679767	MACS_peak_4972	74.00	lobChIP_FOXA1inPBS
chr15	45814085	45814086	MACS_peak_4973	20.00	lobChIP_FOXA1inPBS
chr15	45933801	45933802	MACS_peak_4974	20.00	lobChIP_FOXA1inPBS
chr15	46087180	46087181	MACS_peak_4975	15.00	lobChIP_FOXA1inPBS
chr15	47416843	47416844	MACS_peak_4976	28.00	lobChIP_FOXA1inPBS
chr15	49328229	49328230	MACS_peak_4977	21.00	lobChIP_FOXA1inPBS
chr15	50296052	50296053	MACS_peak_4978	17.00	lobChIP_FOXA1inPBS
chr15	50456807	50456808	MACS_peak_4979	10.00	lobChIP_FOXA1inPBS
chr15	50636512	50636513	MACS_peak_4980	29.00	lobChIP_FOXA1inPBS
chr15	50835188	50835189	MACS_peak_4981	14.00	lobChIP_FOXA1inPBS
chr15	51058880	51058881	MACS_peak_4982	17.00	lobChIP_FOXA1inPBS
chr15	51352432	51352433	MACS_peak_4983	14.00	lobChIP_FOXA1inPBS
chr15	51591680	51591681	MACS_peak_4984	7.00	lobChIP_FOXA1inPBS
chr15	51604598	51604599	MACS_peak_4985	16.00	lobChIP_FOXA1inPBS
chr15	51723821	51723822	MACS_peak_4986	6.00	lobChIP_FOXA1inPBS
chr15	51740518	51740519	MACS_peak_4987	7.00	lobChIP_FOXA1inPBS
chr15	51786308	51786309	MACS_peak_4988	17.00	lobChIP_FOXA1inPBS
chr15	51861528	51861529	MACS_peak_4989	8.00	lobChIP_FOXA1inPBS
chr15	52262649	52262650	MACS_peak_4990	33.00	lobChIP_FOXA1inPBS
chr15	52301406	52301407	MACS_peak_4991	13.00	lobChIP_FOXA1inPBS
chr15	52334105	52334106	MACS_peak_4992	9.00	lobChIP_FOXA1inPBS
chr15	52346768	52346769	MACS_peak_4993	11.00	lobChIP_FOXA1inPBS
chr15	52408532	52408533	MACS_peak_4994	9.00	lobChIP_FOXA1inPBS
chr15	52432276	52432277	MACS_peak_4995	12.00	lobChIP_FOXA1inPBS
chr15	52543631	52543632	MACS_peak_4996	10.00	lobChIP_FOXA1inPBS
chr15	52580730	52580731	MACS_peak_4997	49.00	lobChIP_FOXA1inPBS
chr15	52778306	52778307	MACS_peak_4998	5.00	lobChIP_FOXA1inPBS
chr15	53025350	53025351	MACS_peak_4999	8.00	lobChIP_FOXA1inPBS
chr15	53327134	53327135	MACS_peak_5000	18.00	lobChIP_FOXA1inPBS
chr15	53511572	53511573	MACS_peak_5001	14.00	lobChIP_FOXA1inPBS
chr15	53590334	53590335	MACS_peak_5002	8.00	lobChIP_FOXA1inPBS
chr15	53726577	53726578	MACS_peak_5003	26.00	lobChIP_FOXA1inPBS
chr15	53739425	53739426	MACS_peak_5004	19.00	lobChIP_FOXA1inPBS
chr15	53745905	53745906	MACS_peak_5005	34.00	lobChIP_FOXA1inPBS
chr15	53747645	53747646	MACS_peak_5006	38.00	lobChIP_FOXA1inPBS
chr15	53749886	53749887	MACS_peak_5007	7.00	lobChIP_FOXA1inPBS
chr15	53765553	53765554	MACS_peak_5008	7.00	lobChIP_FOXA1inPBS
chr15	54124339	54124340	MACS_peak_5009	64.00	lobChIP_FOXA1inPBS
chr15	54125740	54125741	MACS_peak_5010	29.00	lobChIP_FOXA1inPBS
chr15	54162056	54162057	MACS_peak_5011	14.00	lobChIP_FOXA1inPBS
chr15	54185882	54185883	MACS_peak_5012	8.00	lobChIP_FOXA1inPBS
chr15	55542811	55542812	MACS_peak_5013	19.00	lobChIP_FOXA1inPBS
chr15	56247851	56247852	MACS_peak_5014	11.00	lobChIP_FOXA1inPBS
chr15	56323741	56323742	MACS_peak_5015	10.00	lobChIP_FOXA1inPBS
chr15	56480083	56480084	MACS_peak_5016	11.00	lobChIP_FOXA1inPBS
chr15	57030183	57030184	MACS_peak_5017	12.00	lobChIP_FOXA1inPBS
chr15	57065140	57065141	MACS_peak_5018	9.00	lobChIP_FOXA1inPBS
chr15	57150176	57150177	MACS_peak_5019	6.00	lobChIP_FOXA1inPBS
chr15	57261299	57261300	MACS_peak_5020	19.00	lobChIP_FOXA1inPBS
chr15	57262318	57262319	MACS_peak_5021	9.00	lobChIP_FOXA1inPBS
chr15	57271309	57271310	MACS_peak_5022	12.00	lobChIP_FOXA1inPBS
chr15	57280760	57280761	MACS_peak_5023	9.00	lobChIP_FOXA1inPBS
chr15	57281739	57281740	MACS_peak_5024	13.00	lobChIP_FOXA1inPBS
chr15	57326072	57326073	MACS_peak_5025	15.00	lobChIP_FOXA1inPBS
chr15	57599962	57599963	MACS_peak_5026	19.00	lobChIP_FOXA1inPBS
chr15	57605268	57605269	MACS_peak_5027	8.00	lobChIP_FOXA1inPBS
chr15	57683882	57683883	MACS_peak_5028	19.00	lobChIP_FOXA1inPBS
chr15	57770244	57770245	MACS_peak_5029	7.00	lobChIP_FOXA1inPBS
chr15	57789437	57789438	MACS_peak_5030	18.00	lobChIP_FOXA1inPBS
chr15	57840744	57840745	MACS_peak_5031	11.00	lobChIP_FOXA1inPBS
chr15	58025148	58025149	MACS_peak_5032	13.00	lobChIP_FOXA1inPBS
chr15	58369883	58369884	MACS_peak_5033	12.00	lobChIP_FOXA1inPBS
chr15	58521864	58521865	MACS_peak_5034	13.00	lobChIP_FOXA1inPBS
chr15	58578253	58578254	MACS_peak_5035	13.00	lobChIP_FOXA1inPBS
chr15	58624506	58624507	MACS_peak_5036	9.00	lobChIP_FOXA1inPBS
chr15	58625400	58625401	MACS_peak_5037	12.00	lobChIP_FOXA1inPBS
chr15	58747390	58747391	MACS_peak_5038	15.00	lobChIP_FOXA1inPBS
chr15	58759950	58759951	MACS_peak_5039	13.00	lobChIP_FOXA1inPBS
chr15	58761118	58761119	MACS_peak_5040	20.00	lobChIP_FOXA1inPBS
chr15	58844063	58844064	MACS_peak_5041	10.00	lobChIP_FOXA1inPBS
chr15	58896790	58896791	MACS_peak_5042	6.00	lobChIP_FOXA1inPBS
chr15	58918723	58918724	MACS_peak_5043	10.00	lobChIP_FOXA1inPBS
chr15	59198590	59198591	MACS_peak_5044	7.00	lobChIP_FOXA1inPBS
chr15	59361819	59361820	MACS_peak_5045	10.00	lobChIP_FOXA1inPBS
chr15	59458154	59458155	MACS_peak_5046	6.00	lobChIP_FOXA1inPBS
chr15	59569001	59569002	MACS_peak_5047	14.00	lobChIP_FOXA1inPBS
chr15	59631691	59631692	MACS_peak_5048	14.00	lobChIP_FOXA1inPBS
chr15	59653342	59653343	MACS_peak_5049	6.00	lobChIP_FOXA1inPBS
chr15	59659036	59659037	MACS_peak_5050	14.00	lobChIP_FOXA1inPBS
chr15	59671327	59671328	MACS_peak_5051	6.00	lobChIP_FOXA1inPBS
chr15	59843360	59843361	MACS_peak_5052	10.00	lobChIP_FOXA1inPBS
chr15	59846479	59846480	MACS_peak_5053	86.00	lobChIP_FOXA1inPBS
chr15	59886866	59886867	MACS_peak_5054	6.00	lobChIP_FOXA1inPBS
chr15	59980567	59980568	MACS_peak_5055	7.00	lobChIP_FOXA1inPBS
chr15	60031174	60031175	MACS_peak_5056	11.00	lobChIP_FOXA1inPBS
chr15	60076201	60076202	MACS_peak_5057	13.00	lobChIP_FOXA1inPBS
chr15	60298939	60298940	MACS_peak_5058	14.00	lobChIP_FOXA1inPBS
chr15	60385921	60385922	MACS_peak_5059	8.00	lobChIP_FOXA1inPBS
chr15	60667009	60667010	MACS_peak_5060	21.00	lobChIP_FOXA1inPBS
chr15	60672081	60672082	MACS_peak_5061	11.00	lobChIP_FOXA1inPBS
chr15	60685453	60685454	MACS_peak_5062	10.00	lobChIP_FOXA1inPBS
chr15	60785569	60785570	MACS_peak_5063	25.00	lobChIP_FOXA1inPBS
chr15	60813167	60813168	MACS_peak_5064	8.00	lobChIP_FOXA1inPBS
chr15	60868404	60868405	MACS_peak_5065	9.00	lobChIP_FOXA1inPBS
chr15	60883235	60883236	MACS_peak_5066	9.00	lobChIP_FOXA1inPBS
chr15	60943639	60943640	MACS_peak_5067	26.00	lobChIP_FOXA1inPBS
chr15	61027940	61027941	MACS_peak_5068	14.00	lobChIP_FOXA1inPBS
chr15	61044905	61044906	MACS_peak_5069	17.00	lobChIP_FOXA1inPBS
chr15	62199880	62199881	MACS_peak_5070	15.00	lobChIP_FOXA1inPBS
chr15	62416718	62416719	MACS_peak_5071	9.00	lobChIP_FOXA1inPBS
chr15	62579899	62579900	MACS_peak_5072	11.00	lobChIP_FOXA1inPBS
chr15	62580742	62580743	MACS_peak_5073	7.00	lobChIP_FOXA1inPBS
chr15	62723848	62723849	MACS_peak_5074	20.00	lobChIP_FOXA1inPBS
chr15	62792619	62792620	MACS_peak_5075	7.00	lobChIP_FOXA1inPBS
chr15	62801511	62801512	MACS_peak_5076	11.00	lobChIP_FOXA1inPBS
chr15	62862499	62862500	MACS_peak_5077	112.00	lobChIP_FOXA1inPBS
chr15	63049071	63049072	MACS_peak_5078	12.00	lobChIP_FOXA1inPBS
chr15	63114094	63114095	MACS_peak_5079	7.00	lobChIP_FOXA1inPBS
chr15	63120785	63120786	MACS_peak_5080	6.00	lobChIP_FOXA1inPBS
chr15	63173488	63173489	MACS_peak_5081	10.00	lobChIP_FOXA1inPBS
chr15	63178789	63178790	MACS_peak_5082	15.00	lobChIP_FOXA1inPBS
chr15	63187467	63187468	MACS_peak_5083	21.00	lobChIP_FOXA1inPBS
chr15	63196662	63196663	MACS_peak_5084	8.00	lobChIP_FOXA1inPBS
chr15	63290587	63290588	MACS_peak_5085	13.00	lobChIP_FOXA1inPBS
chr15	63302801	63302802	MACS_peak_5086	11.00	lobChIP_FOXA1inPBS
chr15	63303663	63303664	MACS_peak_5087	14.00	lobChIP_FOXA1inPBS
chr15	63390022	63390023	MACS_peak_5088	17.00	lobChIP_FOXA1inPBS
chr15	63400216	63400217	MACS_peak_5089	24.00	lobChIP_FOXA1inPBS
chr15	63449576	63449577	MACS_peak_5090	7.00	lobChIP_FOXA1inPBS
chr15	63478365	63478366	MACS_peak_5091	13.00	lobChIP_FOXA1inPBS
chr15	63539470	63539471	MACS_peak_5092	10.00	lobChIP_FOXA1inPBS
chr15	63553194	63553195	MACS_peak_5093	19.00	lobChIP_FOXA1inPBS
chr15	63574137	63574138	MACS_peak_5094	12.00	lobChIP_FOXA1inPBS
chr15	63765375	63765376	MACS_peak_5095	26.00	lobChIP_FOXA1inPBS
chr15	63766421	63766422	MACS_peak_5096	35.00	lobChIP_FOXA1inPBS
chr15	63793092	63793093	MACS_peak_5097	7.00	lobChIP_FOXA1inPBS
chr15	63807004	63807005	MACS_peak_5098	31.00	lobChIP_FOXA1inPBS
chr15	64010912	64010913	MACS_peak_5099	12.00	lobChIP_FOXA1inPBS
chr15	64342920	64342921	MACS_peak_5100	8.00	lobChIP_FOXA1inPBS
chr15	64365201	64365202	MACS_peak_5101	19.00	lobChIP_FOXA1inPBS
chr15	64385335	64385336	MACS_peak_5102	8.00	lobChIP_FOXA1inPBS
chr15	64509489	64509490	MACS_peak_5103	12.00	lobChIP_FOXA1inPBS
chr15	64540295	64540296	MACS_peak_5104	20.00	lobChIP_FOXA1inPBS
chr15	64771245	64771246	MACS_peak_5105	6.00	lobChIP_FOXA1inPBS
chr15	64817055	64817056	MACS_peak_5106	9.00	lobChIP_FOXA1inPBS
chr15	64861137	64861138	MACS_peak_5107	7.00	lobChIP_FOXA1inPBS
chr15	64902681	64902682	MACS_peak_5108	14.00	lobChIP_FOXA1inPBS
chr15	64903429	64903430	MACS_peak_5109	6.00	lobChIP_FOXA1inPBS
chr15	65024937	65024938	MACS_peak_5110	10.00	lobChIP_FOXA1inPBS
chr15	65118262	65118263	MACS_peak_5111	9.00	lobChIP_FOXA1inPBS
chr15	65336726	65336727	MACS_peak_5112	7.00	lobChIP_FOXA1inPBS
chr15	65337139	65337140	MACS_peak_5113	9.00	lobChIP_FOXA1inPBS
chr15	65337632	65337633	MACS_peak_5114	9.00	lobChIP_FOXA1inPBS
chr15	65464051	65464052	MACS_peak_5115	11.00	lobChIP_FOXA1inPBS
chr15	65538095	65538096	MACS_peak_5116	12.00	lobChIP_FOXA1inPBS
chr15	65597207	65597208	MACS_peak_5117	9.00	lobChIP_FOXA1inPBS
chr15	65644012	65644013	MACS_peak_5118	7.00	lobChIP_FOXA1inPBS
chr15	65660304	65660305	MACS_peak_5119	13.00	lobChIP_FOXA1inPBS
chr15	65825132	65825133	MACS_peak_5120	9.00	lobChIP_FOXA1inPBS
chr15	66016872	66016873	MACS_peak_5121	11.00	lobChIP_FOXA1inPBS
chr15	66080287	66080288	MACS_peak_5122	11.00	lobChIP_FOXA1inPBS
chr15	66093842	66093843	MACS_peak_5123	11.00	lobChIP_FOXA1inPBS
chr15	66097667	66097668	MACS_peak_5124	9.00	lobChIP_FOXA1inPBS
chr15	66125147	66125148	MACS_peak_5125	51.00	lobChIP_FOXA1inPBS
chr15	66132510	66132511	MACS_peak_5126	10.00	lobChIP_FOXA1inPBS
chr15	66335909	66335910	MACS_peak_5127	10.00	lobChIP_FOXA1inPBS
chr15	66665450	66665451	MACS_peak_5128	13.00	lobChIP_FOXA1inPBS
chr15	66797495	66797496	MACS_peak_5129	19.00	lobChIP_FOXA1inPBS
chr15	66909911	66909912	MACS_peak_5130	13.00	lobChIP_FOXA1inPBS
chr15	66944780	66944781	MACS_peak_5131	6.00	lobChIP_FOXA1inPBS
chr15	67062908	67062909	MACS_peak_5132	8.00	lobChIP_FOXA1inPBS
chr15	67224626	67224627	MACS_peak_5133	9.00	lobChIP_FOXA1inPBS
chr15	67283397	67283398	MACS_peak_5134	12.00	lobChIP_FOXA1inPBS
chr15	67322694	67322695	MACS_peak_5135	9.00	lobChIP_FOXA1inPBS
chr15	67341243	67341244	MACS_peak_5136	15.00	lobChIP_FOXA1inPBS
chr15	67350489	67350490	MACS_peak_5137	10.00	lobChIP_FOXA1inPBS
chr15	67356804	67356805	MACS_peak_5138	16.00	lobChIP_FOXA1inPBS
chr15	67366771	67366772	MACS_peak_5139	20.00	lobChIP_FOXA1inPBS
chr15	67391406	67391407	MACS_peak_5140	12.00	lobChIP_FOXA1inPBS
chr15	67427985	67427986	MACS_peak_5141	30.00	lobChIP_FOXA1inPBS
chr15	67430919	67430920	MACS_peak_5142	5.00	lobChIP_FOXA1inPBS
chr15	67439513	67439514	MACS_peak_5143	22.00	lobChIP_FOXA1inPBS
chr15	67749525	67749526	MACS_peak_5144	11.00	lobChIP_FOXA1inPBS
chr15	67907841	67907842	MACS_peak_5145	18.00	lobChIP_FOXA1inPBS
chr15	68126395	68126396	MACS_peak_5146	10.00	lobChIP_FOXA1inPBS
chr15	68208083	68208084	MACS_peak_5147	81.00	lobChIP_FOXA1inPBS
chr15	68238529	68238530	MACS_peak_5148	7.00	lobChIP_FOXA1inPBS
chr15	68318548	68318549	MACS_peak_5149	10.00	lobChIP_FOXA1inPBS
chr15	68334636	68334637	MACS_peak_5150	10.00	lobChIP_FOXA1inPBS
chr15	68405862	68405863	MACS_peak_5151	9.00	lobChIP_FOXA1inPBS
chr15	68567704	68567705	MACS_peak_5152	20.00	lobChIP_FOXA1inPBS
chr15	68577771	68577772	MACS_peak_5153	25.00	lobChIP_FOXA1inPBS
chr15	68592888	68592889	MACS_peak_5154	11.00	lobChIP_FOXA1inPBS
chr15	68601580	68601581	MACS_peak_5155	31.00	lobChIP_FOXA1inPBS
chr15	69114553	69114554	MACS_peak_5156	7.00	lobChIP_FOXA1inPBS
chr15	69192542	69192543	MACS_peak_5157	12.00	lobChIP_FOXA1inPBS
chr15	69302853	69302854	MACS_peak_5158	9.00	lobChIP_FOXA1inPBS
chr15	69418654	69418655	MACS_peak_5159	16.00	lobChIP_FOXA1inPBS
chr15	69568469	69568470	MACS_peak_5160	13.00	lobChIP_FOXA1inPBS
chr15	69743895	69743896	MACS_peak_5161	7.00	lobChIP_FOXA1inPBS
chr15	69846978	69846979	MACS_peak_5162	8.00	lobChIP_FOXA1inPBS
chr15	69853822	69853823	MACS_peak_5163	138.00	lobChIP_FOXA1inPBS
chr15	69884422	69884423	MACS_peak_5164	164.00	lobChIP_FOXA1inPBS
chr15	69887176	69887177	MACS_peak_5165	118.00	lobChIP_FOXA1inPBS
chr15	69896473	69896474	MACS_peak_5166	14.00	lobChIP_FOXA1inPBS
chr15	70371283	70371284	MACS_peak_5167	17.00	lobChIP_FOXA1inPBS
chr15	70372360	70372361	MACS_peak_5168	14.00	lobChIP_FOXA1inPBS
chr15	70384497	70384498	MACS_peak_5169	20.00	lobChIP_FOXA1inPBS
chr15	70392281	70392282	MACS_peak_5170	8.00	lobChIP_FOXA1inPBS
chr15	70432995	70432996	MACS_peak_5171	182.00	lobChIP_FOXA1inPBS
chr15	70660860	70660861	MACS_peak_5172	32.00	lobChIP_FOXA1inPBS
chr15	70713626	70713627	MACS_peak_5173	11.00	lobChIP_FOXA1inPBS
chr15	70800331	70800332	MACS_peak_5174	26.00	lobChIP_FOXA1inPBS
chr15	70888939	70888940	MACS_peak_5175	9.00	lobChIP_FOXA1inPBS
chr15	70892391	70892392	MACS_peak_5176	6.00	lobChIP_FOXA1inPBS
chr15	70909679	70909680	MACS_peak_5177	103.00	lobChIP_FOXA1inPBS
chr15	70983398	70983399	MACS_peak_5178	25.00	lobChIP_FOXA1inPBS
chr15	71051237	71051238	MACS_peak_5179	12.00	lobChIP_FOXA1inPBS
chr15	71104578	71104579	MACS_peak_5180	20.00	lobChIP_FOXA1inPBS
chr15	71117607	71117608	MACS_peak_5181	16.00	lobChIP_FOXA1inPBS
chr15	71571642	71571643	MACS_peak_5182	8.00	lobChIP_FOXA1inPBS
chr15	71581120	71581121	MACS_peak_5183	8.00	lobChIP_FOXA1inPBS
chr15	71737479	71737480	MACS_peak_5184	6.00	lobChIP_FOXA1inPBS
chr15	71748490	71748491	MACS_peak_5185	34.00	lobChIP_FOXA1inPBS
chr15	71804932	71804933	MACS_peak_5186	9.00	lobChIP_FOXA1inPBS
chr15	71946568	71946569	MACS_peak_5187	40.00	lobChIP_FOXA1inPBS
chr15	71953678	71953679	MACS_peak_5188	36.00	lobChIP_FOXA1inPBS
chr15	72040088	72040089	MACS_peak_5189	15.00	lobChIP_FOXA1inPBS
chr15	72402911	72402912	MACS_peak_5190	22.00	lobChIP_FOXA1inPBS
chr15	72434273	72434274	MACS_peak_5191	19.00	lobChIP_FOXA1inPBS
chr15	72475189	72475190	MACS_peak_5192	10.00	lobChIP_FOXA1inPBS
chr15	72503299	72503300	MACS_peak_5193	16.00	lobChIP_FOXA1inPBS
chr15	72707919	72707920	MACS_peak_5194	5.00	lobChIP_FOXA1inPBS
chr15	72766026	72766027	MACS_peak_5195	20.00	lobChIP_FOXA1inPBS
chr15	73019687	73019688	MACS_peak_5196	8.00	lobChIP_FOXA1inPBS
chr15	73345731	73345732	MACS_peak_5197	11.00	lobChIP_FOXA1inPBS
chr15	73397668	73397669	MACS_peak_5198	16.00	lobChIP_FOXA1inPBS
chr15	73586405	73586406	MACS_peak_5199	9.00	lobChIP_FOXA1inPBS
chr15	73778323	73778324	MACS_peak_5200	9.00	lobChIP_FOXA1inPBS
chr15	73893879	73893880	MACS_peak_5201	7.00	lobChIP_FOXA1inPBS
chr15	73921931	73921932	MACS_peak_5202	11.00	lobChIP_FOXA1inPBS
chr15	74507877	74507878	MACS_peak_5203	14.00	lobChIP_FOXA1inPBS
chr15	74688492	74688493	MACS_peak_5204	9.00	lobChIP_FOXA1inPBS
chr15	74689482	74689483	MACS_peak_5205	14.00	lobChIP_FOXA1inPBS
chr15	74734062	74734063	MACS_peak_5206	12.00	lobChIP_FOXA1inPBS
chr15	74734543	74734544	MACS_peak_5207	12.00	lobChIP_FOXA1inPBS
chr15	74736099	74736100	MACS_peak_5208	29.00	lobChIP_FOXA1inPBS
chr15	75052726	75052727	MACS_peak_5209	10.00	lobChIP_FOXA1inPBS
chr15	75072335	75072336	MACS_peak_5210	7.00	lobChIP_FOXA1inPBS
chr15	75159609	75159610	MACS_peak_5211	8.00	lobChIP_FOXA1inPBS
chr15	75221373	75221374	MACS_peak_5212	26.00	lobChIP_FOXA1inPBS
chr15	75358102	75358103	MACS_peak_5213	7.00	lobChIP_FOXA1inPBS
chr15	75432648	75432649	MACS_peak_5214	9.00	lobChIP_FOXA1inPBS
chr15	75474302	75474303	MACS_peak_5215	15.00	lobChIP_FOXA1inPBS
chr15	75661294	75661295	MACS_peak_5216	13.00	lobChIP_FOXA1inPBS
chr15	75744769	75744770	MACS_peak_5217	8.00	lobChIP_FOXA1inPBS
chr15	75930846	75930847	MACS_peak_5218	12.00	lobChIP_FOXA1inPBS
chr15	76178296	76178297	MACS_peak_5219	12.00	lobChIP_FOXA1inPBS
chr15	76253926	76253927	MACS_peak_5220	38.00	lobChIP_FOXA1inPBS
chr15	76605384	76605385	MACS_peak_5221	9.00	lobChIP_FOXA1inPBS
chr15	76825888	76825889	MACS_peak_5222	14.00	lobChIP_FOXA1inPBS
chr15	76826584	76826585	MACS_peak_5223	16.00	lobChIP_FOXA1inPBS
chr15	76865188	76865189	MACS_peak_5224	19.00	lobChIP_FOXA1inPBS
chr15	77348125	77348126	MACS_peak_5225	7.00	lobChIP_FOXA1inPBS
chr15	77356671	77356672	MACS_peak_5226	8.00	lobChIP_FOXA1inPBS
chr15	77465929	77465930	MACS_peak_5227	11.00	lobChIP_FOXA1inPBS
chr15	77476853	77476854	MACS_peak_5228	9.00	lobChIP_FOXA1inPBS
chr15	77516871	77516872	MACS_peak_5229	11.00	lobChIP_FOXA1inPBS
chr15	77574864	77574865	MACS_peak_5230	12.00	lobChIP_FOXA1inPBS
chr15	78355485	78355486	MACS_peak_5231	7.00	lobChIP_FOXA1inPBS
chr15	78357571	78357572	MACS_peak_5232	8.00	lobChIP_FOXA1inPBS
chr15	78667314	78667315	MACS_peak_5233	14.00	lobChIP_FOXA1inPBS
chr15	79244484	79244485	MACS_peak_5234	23.00	lobChIP_FOXA1inPBS
chr15	79286398	79286399	MACS_peak_5235	17.00	lobChIP_FOXA1inPBS
chr15	79463153	79463154	MACS_peak_5236	11.00	lobChIP_FOXA1inPBS
chr15	80178564	80178565	MACS_peak_5237	18.00	lobChIP_FOXA1inPBS
chr15	80191308	80191309	MACS_peak_5238	12.00	lobChIP_FOXA1inPBS
chr15	80216494	80216495	MACS_peak_5239	7.00	lobChIP_FOXA1inPBS
chr15	80271619	80271620	MACS_peak_5240	7.00	lobChIP_FOXA1inPBS
chr15	80325124	80325125	MACS_peak_5241	6.00	lobChIP_FOXA1inPBS
chr15	80449761	80449762	MACS_peak_5242	9.00	lobChIP_FOXA1inPBS
chr15	80498523	80498524	MACS_peak_5243	12.00	lobChIP_FOXA1inPBS
chr15	80500517	80500518	MACS_peak_5244	15.00	lobChIP_FOXA1inPBS
chr15	80542508	80542509	MACS_peak_5245	9.00	lobChIP_FOXA1inPBS
chr15	80757442	80757443	MACS_peak_5246	20.00	lobChIP_FOXA1inPBS
chr15	80876963	80876964	MACS_peak_5247	11.00	lobChIP_FOXA1inPBS
chr15	81035686	81035687	MACS_peak_5248	17.00	lobChIP_FOXA1inPBS
chr15	81097687	81097688	MACS_peak_5249	39.00	lobChIP_FOXA1inPBS
chr15	81131641	81131642	MACS_peak_5250	28.00	lobChIP_FOXA1inPBS
chr15	81134677	81134678	MACS_peak_5251	17.00	lobChIP_FOXA1inPBS
chr15	81149406	81149407	MACS_peak_5252	11.00	lobChIP_FOXA1inPBS
chr15	81206072	81206073	MACS_peak_5253	8.00	lobChIP_FOXA1inPBS
chr15	81742668	81742669	MACS_peak_5254	9.00	lobChIP_FOXA1inPBS
chr15	82555896	82555897	MACS_peak_5255	138.00	lobChIP_FOXA1inPBS
chr15	83432656	83432657	MACS_peak_5256	10.00	lobChIP_FOXA1inPBS
chr15	83588513	83588514	MACS_peak_5257	24.00	lobChIP_FOXA1inPBS
chr15	84599658	84599659	MACS_peak_5258	20.00	lobChIP_FOXA1inPBS
chr15	85075130	85075131	MACS_peak_5259	8.00	lobChIP_FOXA1inPBS
chr15	85114474	85114475	MACS_peak_5260	6.00	lobChIP_FOXA1inPBS
chr15	85150127	85150128	MACS_peak_5261	18.00	lobChIP_FOXA1inPBS
chr15	85295650	85295651	MACS_peak_5262	14.00	lobChIP_FOXA1inPBS
chr15	85388366	85388367	MACS_peak_5263	14.00	lobChIP_FOXA1inPBS
chr15	85591057	85591058	MACS_peak_5264	41.00	lobChIP_FOXA1inPBS
chr15	85892442	85892443	MACS_peak_5265	14.00	lobChIP_FOXA1inPBS
chr15	85942240	85942241	MACS_peak_5266	12.00	lobChIP_FOXA1inPBS
chr15	85956862	85956863	MACS_peak_5267	11.00	lobChIP_FOXA1inPBS
chr15	85957389	85957390	MACS_peak_5268	8.00	lobChIP_FOXA1inPBS
chr15	85987228	85987229	MACS_peak_5269	9.00	lobChIP_FOXA1inPBS
chr15	86058529	86058530	MACS_peak_5270	13.00	lobChIP_FOXA1inPBS
chr15	86251221	86251222	MACS_peak_5271	32.00	lobChIP_FOXA1inPBS
chr15	86324521	86324522	MACS_peak_5272	11.00	lobChIP_FOXA1inPBS
chr15	86558532	86558533	MACS_peak_5273	12.00	lobChIP_FOXA1inPBS
chr15	89181927	89181928	MACS_peak_5274	7.00	lobChIP_FOXA1inPBS
chr15	89437682	89437683	MACS_peak_5275	9.00	lobChIP_FOXA1inPBS
chr15	89663512	89663513	MACS_peak_5276	14.00	lobChIP_FOXA1inPBS
chr15	89664808	89664809	MACS_peak_5277	36.00	lobChIP_FOXA1inPBS
chr15	89673137	89673138	MACS_peak_5278	8.00	lobChIP_FOXA1inPBS
chr15	89680308	89680309	MACS_peak_5279	8.00	lobChIP_FOXA1inPBS
chr15	89685228	89685229	MACS_peak_5280	8.00	lobChIP_FOXA1inPBS
chr15	89708969	89708970	MACS_peak_5281	13.00	lobChIP_FOXA1inPBS
chr15	90218029	90218030	MACS_peak_5282	11.00	lobChIP_FOXA1inPBS
chr15	90300318	90300319	MACS_peak_5283	21.00	lobChIP_FOXA1inPBS
chr15	90316389	90316390	MACS_peak_5284	12.00	lobChIP_FOXA1inPBS
chr15	90338468	90338469	MACS_peak_5285	5.00	lobChIP_FOXA1inPBS
chr15	90352587	90352588	MACS_peak_5286	21.00	lobChIP_FOXA1inPBS
chr15	90388846	90388847	MACS_peak_5287	12.00	lobChIP_FOXA1inPBS
chr15	90541526	90541527	MACS_peak_5288	8.00	lobChIP_FOXA1inPBS
chr15	90546542	90546543	MACS_peak_5289	11.00	lobChIP_FOXA1inPBS
chr15	90572428	90572429	MACS_peak_5290	10.00	lobChIP_FOXA1inPBS
chr15	90578946	90578947	MACS_peak_5291	19.00	lobChIP_FOXA1inPBS
chr15	90581963	90581964	MACS_peak_5292	10.00	lobChIP_FOXA1inPBS
chr15	90669533	90669534	MACS_peak_5293	16.00	lobChIP_FOXA1inPBS
chr15	90698514	90698515	MACS_peak_5294	7.00	lobChIP_FOXA1inPBS
chr15	90709229	90709230	MACS_peak_5295	9.00	lobChIP_FOXA1inPBS
chr15	90944247	90944248	MACS_peak_5296	25.00	lobChIP_FOXA1inPBS
chr15	90965546	90965547	MACS_peak_5297	12.00	lobChIP_FOXA1inPBS
chr15	91033355	91033356	MACS_peak_5298	74.00	lobChIP_FOXA1inPBS
chr15	91056982	91056983	MACS_peak_5299	16.00	lobChIP_FOXA1inPBS
chr15	91294704	91294705	MACS_peak_5300	6.00	lobChIP_FOXA1inPBS
chr15	91379727	91379728	MACS_peak_5301	25.00	lobChIP_FOXA1inPBS
chr15	91404723	91404724	MACS_peak_5302	28.00	lobChIP_FOXA1inPBS
chr15	91417517	91417518	MACS_peak_5303	27.00	lobChIP_FOXA1inPBS
chr15	91473140	91473141	MACS_peak_5304	9.00	lobChIP_FOXA1inPBS
chr15	91499298	91499299	MACS_peak_5305	8.00	lobChIP_FOXA1inPBS
chr15	93160976	93160977	MACS_peak_5306	15.00	lobChIP_FOXA1inPBS
chr15	93191561	93191562	MACS_peak_5307	10.00	lobChIP_FOXA1inPBS
chr15	93210614	93210615	MACS_peak_5308	14.00	lobChIP_FOXA1inPBS
chr15	93289311	93289312	MACS_peak_5309	8.00	lobChIP_FOXA1inPBS
chr15	93347597	93347598	MACS_peak_5310	37.00	lobChIP_FOXA1inPBS
chr15	93354565	93354566	MACS_peak_5311	10.00	lobChIP_FOXA1inPBS
chr15	93374950	93374951	MACS_peak_5312	9.00	lobChIP_FOXA1inPBS
chr15	93425415	93425416	MACS_peak_5313	7.00	lobChIP_FOXA1inPBS
chr15	93427481	93427482	MACS_peak_5314	8.00	lobChIP_FOXA1inPBS
chr15	93461337	93461338	MACS_peak_5315	9.00	lobChIP_FOXA1inPBS
chr15	93865348	93865349	MACS_peak_5316	11.00	lobChIP_FOXA1inPBS
chr15	93884574	93884575	MACS_peak_5317	8.00	lobChIP_FOXA1inPBS
chr15	94540553	94540554	MACS_peak_5318	19.00	lobChIP_FOXA1inPBS
chr15	94654162	94654163	MACS_peak_5319	11.00	lobChIP_FOXA1inPBS
chr15	94707429	94707430	MACS_peak_5320	6.00	lobChIP_FOXA1inPBS
chr15	94791714	94791715	MACS_peak_5321	11.00	lobChIP_FOXA1inPBS
chr15	94796401	94796402	MACS_peak_5322	12.00	lobChIP_FOXA1inPBS
chr15	96597151	96597152	MACS_peak_5323	12.00	lobChIP_FOXA1inPBS
chr15	96666456	96666457	MACS_peak_5324	11.00	lobChIP_FOXA1inPBS
chr15	96714302	96714303	MACS_peak_5325	13.00	lobChIP_FOXA1inPBS
chr15	96727313	96727314	MACS_peak_5326	9.00	lobChIP_FOXA1inPBS
chr15	96752785	96752786	MACS_peak_5327	10.00	lobChIP_FOXA1inPBS
chr15	96797524	96797525	MACS_peak_5328	14.00	lobChIP_FOXA1inPBS
chr15	96805228	96805229	MACS_peak_5329	17.00	lobChIP_FOXA1inPBS
chr15	96807349	96807350	MACS_peak_5330	11.00	lobChIP_FOXA1inPBS
chr15	96869491	96869492	MACS_peak_5331	14.00	lobChIP_FOXA1inPBS
chr15	96880185	96880186	MACS_peak_5332	9.00	lobChIP_FOXA1inPBS
chr15	96882165	96882166	MACS_peak_5333	18.00	lobChIP_FOXA1inPBS
chr15	99198296	99198297	MACS_peak_5334	24.00	lobChIP_FOXA1inPBS
chr15	99214564	99214565	MACS_peak_5335	20.00	lobChIP_FOXA1inPBS
chr15	99342904	99342905	MACS_peak_5336	11.00	lobChIP_FOXA1inPBS
chr15	99386287	99386288	MACS_peak_5337	11.00	lobChIP_FOXA1inPBS
chr15	99387244	99387245	MACS_peak_5338	50.00	lobChIP_FOXA1inPBS
chr15	99440689	99440690	MACS_peak_5339	16.00	lobChIP_FOXA1inPBS
chr15	99721044	99721045	MACS_peak_5340	26.00	lobChIP_FOXA1inPBS
chr15	99788407	99788408	MACS_peak_5341	10.00	lobChIP_FOXA1inPBS
chr15	99847319	99847320	MACS_peak_5342	7.00	lobChIP_FOXA1inPBS
chr15	99863083	99863084	MACS_peak_5343	6.00	lobChIP_FOXA1inPBS
chr15	99864971	99864972	MACS_peak_5344	9.00	lobChIP_FOXA1inPBS
chr15	99866679	99866680	MACS_peak_5345	16.00	lobChIP_FOXA1inPBS
chr15	99961537	99961538	MACS_peak_5346	11.00	lobChIP_FOXA1inPBS
chr15	100168811	100168812	MACS_peak_5347	11.00	lobChIP_FOXA1inPBS
chr15	100208056	100208057	MACS_peak_5348	12.00	lobChIP_FOXA1inPBS
chr15	100209516	100209517	MACS_peak_5349	9.00	lobChIP_FOXA1inPBS
chr15	100244053	100244054	MACS_peak_5350	7.00	lobChIP_FOXA1inPBS
chr15	100261556	100261557	MACS_peak_5351	6.00	lobChIP_FOXA1inPBS
chr15	100890970	100890971	MACS_peak_5352	12.00	lobChIP_FOXA1inPBS
chr15	101256113	101256114	MACS_peak_5353	14.00	lobChIP_FOXA1inPBS
chr15	101262728	101262729	MACS_peak_5354	11.00	lobChIP_FOXA1inPBS
chr15	101611171	101611172	MACS_peak_5355	64.00	lobChIP_FOXA1inPBS
chr15	101671901	101671902	MACS_peak_5356	8.00	lobChIP_FOXA1inPBS
chr15	101757906	101757907	MACS_peak_5357	14.00	lobChIP_FOXA1inPBS
chr15	101978590	101978591	MACS_peak_5358	6.00	lobChIP_FOXA1inPBS
chr15	101979843	101979844	MACS_peak_5359	20.00	lobChIP_FOXA1inPBS
chr15	101985875	101985876	MACS_peak_5360	21.00	lobChIP_FOXA1inPBS
chr15	102025626	102025627	MACS_peak_5361	6.00	lobChIP_FOXA1inPBS
chr15	102068714	102068715	MACS_peak_5362	8.00	lobChIP_FOXA1inPBS
chr15	102156738	102156739	MACS_peak_5363	7.00	lobChIP_FOXA1inPBS
chr16	95614	95615	MACS_peak_5364	9.00	lobChIP_FOXA1inPBS
chr16	392722	392723	MACS_peak_5365	18.00	lobChIP_FOXA1inPBS
chr16	501834	501835	MACS_peak_5366	16.00	lobChIP_FOXA1inPBS
chr16	559361	559362	MACS_peak_5367	12.00	lobChIP_FOXA1inPBS
chr16	962904	962905	MACS_peak_5368	15.00	lobChIP_FOXA1inPBS
chr16	1126950	1126951	MACS_peak_5369	8.00	lobChIP_FOXA1inPBS
chr16	1128726	1128727	MACS_peak_5370	15.00	lobChIP_FOXA1inPBS
chr16	1205068	1205069	MACS_peak_5371	40.00	lobChIP_FOXA1inPBS
chr16	1247454	1247455	MACS_peak_5372	13.00	lobChIP_FOXA1inPBS
chr16	1341077	1341078	MACS_peak_5373	10.00	lobChIP_FOXA1inPBS
chr16	1480969	1480970	MACS_peak_5374	42.00	lobChIP_FOXA1inPBS
chr16	1739345	1739346	MACS_peak_5375	40.00	lobChIP_FOXA1inPBS
chr16	1770059	1770060	MACS_peak_5376	7.00	lobChIP_FOXA1inPBS
chr16	1878868	1878869	MACS_peak_5377	11.00	lobChIP_FOXA1inPBS
chr16	1883186	1883187	MACS_peak_5378	20.00	lobChIP_FOXA1inPBS
chr16	1920485	1920486	MACS_peak_5379	9.00	lobChIP_FOXA1inPBS
chr16	1928177	1928178	MACS_peak_5380	6.00	lobChIP_FOXA1inPBS
chr16	1990755	1990756	MACS_peak_5381	8.00	lobChIP_FOXA1inPBS
chr16	2044045	2044046	MACS_peak_5382	22.00	lobChIP_FOXA1inPBS
chr16	2065504	2065505	MACS_peak_5383	8.00	lobChIP_FOXA1inPBS
chr16	2089666	2089667	MACS_peak_5384	9.00	lobChIP_FOXA1inPBS
chr16	2276610	2276611	MACS_peak_5385	16.00	lobChIP_FOXA1inPBS
chr16	2298761	2298762	MACS_peak_5386	11.00	lobChIP_FOXA1inPBS
chr16	2439047	2439048	MACS_peak_5387	60.00	lobChIP_FOXA1inPBS
chr16	2535137	2535138	MACS_peak_5388	7.00	lobChIP_FOXA1inPBS
chr16	2574454	2574455	MACS_peak_5389	15.00	lobChIP_FOXA1inPBS
chr16	3184040	3184041	MACS_peak_5390	7.00	lobChIP_FOXA1inPBS
chr16	3202285	3202286	MACS_peak_5391	15.00	lobChIP_FOXA1inPBS
chr16	3646581	3646582	MACS_peak_5392	16.00	lobChIP_FOXA1inPBS
chr16	3660113	3660114	MACS_peak_5393	44.00	lobChIP_FOXA1inPBS
chr16	3696438	3696439	MACS_peak_5394	10.00	lobChIP_FOXA1inPBS
chr16	3745978	3745979	MACS_peak_5395	22.00	lobChIP_FOXA1inPBS
chr16	4159421	4159422	MACS_peak_5396	11.00	lobChIP_FOXA1inPBS
chr16	4207591	4207592	MACS_peak_5397	16.00	lobChIP_FOXA1inPBS
chr16	4323093	4323094	MACS_peak_5398	18.00	lobChIP_FOXA1inPBS
chr16	4395979	4395980	MACS_peak_5399	26.00	lobChIP_FOXA1inPBS
chr16	4423166	4423167	MACS_peak_5400	17.00	lobChIP_FOXA1inPBS
chr16	4509582	4509583	MACS_peak_5401	7.00	lobChIP_FOXA1inPBS
chr16	4838748	4838749	MACS_peak_5402	6.00	lobChIP_FOXA1inPBS
chr16	4993313	4993314	MACS_peak_5403	6.00	lobChIP_FOXA1inPBS
chr16	4994776	4994777	MACS_peak_5404	18.00	lobChIP_FOXA1inPBS
chr16	5007929	5007930	MACS_peak_5405	11.00	lobChIP_FOXA1inPBS
chr16	5082868	5082869	MACS_peak_5406	8.00	lobChIP_FOXA1inPBS
chr16	5085501	5085502	MACS_peak_5407	25.00	lobChIP_FOXA1inPBS
chr16	5115113	5115114	MACS_peak_5408	12.00	lobChIP_FOXA1inPBS
chr16	5194543	5194544	MACS_peak_5409	74.00	lobChIP_FOXA1inPBS
chr16	7188147	7188148	MACS_peak_5410	18.00	lobChIP_FOXA1inPBS
chr16	7236021	7236022	MACS_peak_5411	11.00	lobChIP_FOXA1inPBS
chr16	8601536	8601537	MACS_peak_5412	11.00	lobChIP_FOXA1inPBS
chr16	8621390	8621391	MACS_peak_5413	11.00	lobChIP_FOXA1inPBS
chr16	8769711	8769712	MACS_peak_5414	13.00	lobChIP_FOXA1inPBS
chr16	8772739	8772740	MACS_peak_5415	52.00	lobChIP_FOXA1inPBS
chr16	8775177	8775178	MACS_peak_5416	18.00	lobChIP_FOXA1inPBS
chr16	8834799	8834800	MACS_peak_5417	28.00	lobChIP_FOXA1inPBS
chr16	8954272	8954273	MACS_peak_5418	11.00	lobChIP_FOXA1inPBS
chr16	8957636	8957637	MACS_peak_5419	11.00	lobChIP_FOXA1inPBS
chr16	8966084	8966085	MACS_peak_5420	8.00	lobChIP_FOXA1inPBS
chr16	9054321	9054322	MACS_peak_5421	17.00	lobChIP_FOXA1inPBS
chr16	9073105	9073106	MACS_peak_5422	8.00	lobChIP_FOXA1inPBS
chr16	9081465	9081466	MACS_peak_5423	64.00	lobChIP_FOXA1inPBS
chr16	9089118	9089119	MACS_peak_5424	11.00	lobChIP_FOXA1inPBS
chr16	9111026	9111027	MACS_peak_5425	5.00	lobChIP_FOXA1inPBS
chr16	9211553	9211554	MACS_peak_5426	12.00	lobChIP_FOXA1inPBS
chr16	9245097	9245098	MACS_peak_5427	17.00	lobChIP_FOXA1inPBS
chr16	9459381	9459382	MACS_peak_5428	17.00	lobChIP_FOXA1inPBS
chr16	9639966	9639967	MACS_peak_5429	11.00	lobChIP_FOXA1inPBS
chr16	10257921	10257922	MACS_peak_5430	14.00	lobChIP_FOXA1inPBS
chr16	10351084	10351085	MACS_peak_5431	13.00	lobChIP_FOXA1inPBS
chr16	10356391	10356392	MACS_peak_5432	13.00	lobChIP_FOXA1inPBS
chr16	10359985	10359986	MACS_peak_5433	23.00	lobChIP_FOXA1inPBS
chr16	10363696	10363697	MACS_peak_5434	57.00	lobChIP_FOXA1inPBS
chr16	10412834	10412835	MACS_peak_5435	18.00	lobChIP_FOXA1inPBS
chr16	10417087	10417088	MACS_peak_5436	7.00	lobChIP_FOXA1inPBS
chr16	10429573	10429574	MACS_peak_5437	10.00	lobChIP_FOXA1inPBS
chr16	10456499	10456500	MACS_peak_5438	13.00	lobChIP_FOXA1inPBS
chr16	10614454	10614455	MACS_peak_5439	39.00	lobChIP_FOXA1inPBS
chr16	10695894	10695895	MACS_peak_5440	21.00	lobChIP_FOXA1inPBS
chr16	10719990	10719991	MACS_peak_5441	9.00	lobChIP_FOXA1inPBS
chr16	10740442	10740443	MACS_peak_5442	6.00	lobChIP_FOXA1inPBS
chr16	10741824	10741825	MACS_peak_5443	76.00	lobChIP_FOXA1inPBS
chr16	10746589	10746590	MACS_peak_5444	12.00	lobChIP_FOXA1inPBS
chr16	11110669	11110670	MACS_peak_5445	22.00	lobChIP_FOXA1inPBS
chr16	11233328	11233329	MACS_peak_5446	89.00	lobChIP_FOXA1inPBS
chr16	11348381	11348382	MACS_peak_5447	11.00	lobChIP_FOXA1inPBS
chr16	11373610	11373611	MACS_peak_5448	31.00	lobChIP_FOXA1inPBS
chr16	11390908	11390909	MACS_peak_5449	34.00	lobChIP_FOXA1inPBS
chr16	11439101	11439102	MACS_peak_5450	13.00	lobChIP_FOXA1inPBS
chr16	11439685	11439686	MACS_peak_5451	8.00	lobChIP_FOXA1inPBS
chr16	11441360	11441361	MACS_peak_5452	9.00	lobChIP_FOXA1inPBS
chr16	11458395	11458396	MACS_peak_5453	46.00	lobChIP_FOXA1inPBS
chr16	11668533	11668534	MACS_peak_5454	12.00	lobChIP_FOXA1inPBS
chr16	11669633	11669634	MACS_peak_5455	22.00	lobChIP_FOXA1inPBS
chr16	11670617	11670618	MACS_peak_5456	19.00	lobChIP_FOXA1inPBS
chr16	11678072	11678073	MACS_peak_5457	13.00	lobChIP_FOXA1inPBS
chr16	11699007	11699008	MACS_peak_5458	10.00	lobChIP_FOXA1inPBS
chr16	11794343	11794344	MACS_peak_5459	22.00	lobChIP_FOXA1inPBS
chr16	11837440	11837441	MACS_peak_5460	16.00	lobChIP_FOXA1inPBS
chr16	11838336	11838337	MACS_peak_5461	30.00	lobChIP_FOXA1inPBS
chr16	11849147	11849148	MACS_peak_5462	27.00	lobChIP_FOXA1inPBS
chr16	11851825	11851826	MACS_peak_5463	8.00	lobChIP_FOXA1inPBS
chr16	11880003	11880004	MACS_peak_5464	18.00	lobChIP_FOXA1inPBS
chr16	11884057	11884058	MACS_peak_5465	13.00	lobChIP_FOXA1inPBS
chr16	11904260	11904261	MACS_peak_5466	24.00	lobChIP_FOXA1inPBS
chr16	11916234	11916235	MACS_peak_5467	20.00	lobChIP_FOXA1inPBS
chr16	11928306	11928307	MACS_peak_5468	26.00	lobChIP_FOXA1inPBS
chr16	11946236	11946237	MACS_peak_5469	11.00	lobChIP_FOXA1inPBS
chr16	11961526	11961527	MACS_peak_5470	17.00	lobChIP_FOXA1inPBS
chr16	12010685	12010686	MACS_peak_5471	17.00	lobChIP_FOXA1inPBS
chr16	12118608	12118609	MACS_peak_5472	17.00	lobChIP_FOXA1inPBS
chr16	12191676	12191677	MACS_peak_5473	15.00	lobChIP_FOXA1inPBS
chr16	12305703	12305704	MACS_peak_5474	15.00	lobChIP_FOXA1inPBS
chr16	12324283	12324284	MACS_peak_5475	9.00	lobChIP_FOXA1inPBS
chr16	12351980	12351981	MACS_peak_5476	12.00	lobChIP_FOXA1inPBS
chr16	12354717	12354718	MACS_peak_5477	7.00	lobChIP_FOXA1inPBS
chr16	12355219	12355220	MACS_peak_5478	8.00	lobChIP_FOXA1inPBS
chr16	12409592	12409593	MACS_peak_5479	29.00	lobChIP_FOXA1inPBS
chr16	12667927	12667928	MACS_peak_5480	49.00	lobChIP_FOXA1inPBS
chr16	12678599	12678600	MACS_peak_5481	8.00	lobChIP_FOXA1inPBS
chr16	12706591	12706592	MACS_peak_5482	24.00	lobChIP_FOXA1inPBS
chr16	12707301	12707302	MACS_peak_5483	10.00	lobChIP_FOXA1inPBS
chr16	12737925	12737926	MACS_peak_5484	35.00	lobChIP_FOXA1inPBS
chr16	12773013	12773014	MACS_peak_5485	17.00	lobChIP_FOXA1inPBS
chr16	12789449	12789450	MACS_peak_5486	13.00	lobChIP_FOXA1inPBS
chr16	12848187	12848188	MACS_peak_5487	22.00	lobChIP_FOXA1inPBS
chr16	12913358	12913359	MACS_peak_5488	12.00	lobChIP_FOXA1inPBS
chr16	13204383	13204384	MACS_peak_5489	10.00	lobChIP_FOXA1inPBS
chr16	13339771	13339772	MACS_peak_5490	131.00	lobChIP_FOXA1inPBS
chr16	13364008	13364009	MACS_peak_5491	48.00	lobChIP_FOXA1inPBS
chr16	13979695	13979696	MACS_peak_5492	32.00	lobChIP_FOXA1inPBS
chr16	14051862	14051863	MACS_peak_5493	15.00	lobChIP_FOXA1inPBS
chr16	14114241	14114242	MACS_peak_5494	24.00	lobChIP_FOXA1inPBS
chr16	14182156	14182157	MACS_peak_5495	14.00	lobChIP_FOXA1inPBS
chr16	14191913	14191914	MACS_peak_5496	9.00	lobChIP_FOXA1inPBS
chr16	14200474	14200475	MACS_peak_5497	35.00	lobChIP_FOXA1inPBS
chr16	14296833	14296834	MACS_peak_5498	7.00	lobChIP_FOXA1inPBS
chr16	14383795	14383796	MACS_peak_5499	16.00	lobChIP_FOXA1inPBS
chr16	14412330	14412331	MACS_peak_5500	12.00	lobChIP_FOXA1inPBS
chr16	14458490	14458491	MACS_peak_5501	9.00	lobChIP_FOXA1inPBS
chr16	14479293	14479294	MACS_peak_5502	36.00	lobChIP_FOXA1inPBS
chr16	14493560	14493561	MACS_peak_5503	13.00	lobChIP_FOXA1inPBS
chr16	14569485	14569486	MACS_peak_5504	20.00	lobChIP_FOXA1inPBS
chr16	14591566	14591567	MACS_peak_5505	12.00	lobChIP_FOXA1inPBS
chr16	14595619	14595620	MACS_peak_5506	10.00	lobChIP_FOXA1inPBS
chr16	14605097	14605098	MACS_peak_5507	68.00	lobChIP_FOXA1inPBS
chr16	14639520	14639521	MACS_peak_5508	86.00	lobChIP_FOXA1inPBS
chr16	14643715	14643716	MACS_peak_5509	25.00	lobChIP_FOXA1inPBS
chr16	14656822	14656823	MACS_peak_5510	11.00	lobChIP_FOXA1inPBS
chr16	14750466	14750467	MACS_peak_5511	7.00	lobChIP_FOXA1inPBS
chr16	14968337	14968338	MACS_peak_5512	8.00	lobChIP_FOXA1inPBS
chr16	15146029	15146030	MACS_peak_5513	30.00	lobChIP_FOXA1inPBS
chr16	15537907	15537908	MACS_peak_5514	13.00	lobChIP_FOXA1inPBS
chr16	15669194	15669195	MACS_peak_5515	11.00	lobChIP_FOXA1inPBS
chr16	15670338	15670339	MACS_peak_5516	9.00	lobChIP_FOXA1inPBS
chr16	15685018	15685019	MACS_peak_5517	11.00	lobChIP_FOXA1inPBS
chr16	15702428	15702429	MACS_peak_5518	30.00	lobChIP_FOXA1inPBS
chr16	15703483	15703484	MACS_peak_5519	6.00	lobChIP_FOXA1inPBS
chr16	15704544	15704545	MACS_peak_5520	19.00	lobChIP_FOXA1inPBS
chr16	15723020	15723021	MACS_peak_5521	45.00	lobChIP_FOXA1inPBS
chr16	15766935	15766936	MACS_peak_5522	25.00	lobChIP_FOXA1inPBS
chr16	15969135	15969136	MACS_peak_5523	19.00	lobChIP_FOXA1inPBS
chr16	16026966	16026967	MACS_peak_5524	12.00	lobChIP_FOXA1inPBS
chr16	16040391	16040392	MACS_peak_5525	12.00	lobChIP_FOXA1inPBS
chr16	16049731	16049732	MACS_peak_5526	8.00	lobChIP_FOXA1inPBS
chr16	16098041	16098042	MACS_peak_5527	6.00	lobChIP_FOXA1inPBS
chr16	16108924	16108925	MACS_peak_5528	31.00	lobChIP_FOXA1inPBS
chr16	16181914	16181915	MACS_peak_5529	10.00	lobChIP_FOXA1inPBS
chr16	16242754	16242755	MACS_peak_5530	8.00	lobChIP_FOXA1inPBS
chr16	16243776	16243777	MACS_peak_5531	20.00	lobChIP_FOXA1inPBS
chr16	16288948	16288949	MACS_peak_5532	14.00	lobChIP_FOXA1inPBS
chr16	16307963	16307964	MACS_peak_5533	10.00	lobChIP_FOXA1inPBS
chr16	16345431	16345432	MACS_peak_5534	9.00	lobChIP_FOXA1inPBS
chr16	16868412	16868413	MACS_peak_5535	9.00	lobChIP_FOXA1inPBS
chr16	18627633	18627634	MACS_peak_5536	10.00	lobChIP_FOXA1inPBS
chr16	18943764	18943765	MACS_peak_5537	27.00	lobChIP_FOXA1inPBS
chr16	18948517	18948518	MACS_peak_5538	33.00	lobChIP_FOXA1inPBS
chr16	19000512	19000513	MACS_peak_5539	73.00	lobChIP_FOXA1inPBS
chr16	19010213	19010214	MACS_peak_5540	12.00	lobChIP_FOXA1inPBS
chr16	19020244	19020245	MACS_peak_5541	13.00	lobChIP_FOXA1inPBS
chr16	19104960	19104961	MACS_peak_5542	15.00	lobChIP_FOXA1inPBS
chr16	19122299	19122300	MACS_peak_5543	30.00	lobChIP_FOXA1inPBS
chr16	19393818	19393819	MACS_peak_5544	12.00	lobChIP_FOXA1inPBS
chr16	19490792	19490793	MACS_peak_5545	12.00	lobChIP_FOXA1inPBS
chr16	19553522	19553523	MACS_peak_5546	11.00	lobChIP_FOXA1inPBS
chr16	19673933	19673934	MACS_peak_5547	31.00	lobChIP_FOXA1inPBS
chr16	19711776	19711777	MACS_peak_5548	48.00	lobChIP_FOXA1inPBS
chr16	19754646	19754647	MACS_peak_5549	14.00	lobChIP_FOXA1inPBS
chr16	19756157	19756158	MACS_peak_5550	39.00	lobChIP_FOXA1inPBS
chr16	19796928	19796929	MACS_peak_5551	19.00	lobChIP_FOXA1inPBS
chr16	19931176	19931177	MACS_peak_5552	7.00	lobChIP_FOXA1inPBS
chr16	19938233	19938234	MACS_peak_5553	22.00	lobChIP_FOXA1inPBS
chr16	20025577	20025578	MACS_peak_5554	10.00	lobChIP_FOXA1inPBS
chr16	20339257	20339258	MACS_peak_5555	18.00	lobChIP_FOXA1inPBS
chr16	20388691	20388692	MACS_peak_5556	9.00	lobChIP_FOXA1inPBS
chr16	20438040	20438041	MACS_peak_5557	23.00	lobChIP_FOXA1inPBS
chr16	20580371	20580372	MACS_peak_5558	9.00	lobChIP_FOXA1inPBS
chr16	20608267	20608268	MACS_peak_5559	20.00	lobChIP_FOXA1inPBS
chr16	20689188	20689189	MACS_peak_5560	16.00	lobChIP_FOXA1inPBS
chr16	20749936	20749937	MACS_peak_5561	9.00	lobChIP_FOXA1inPBS
chr16	20815168	20815169	MACS_peak_5562	13.00	lobChIP_FOXA1inPBS
chr16	20886030	20886031	MACS_peak_5563	12.00	lobChIP_FOXA1inPBS
chr16	20890233	20890234	MACS_peak_5564	10.00	lobChIP_FOXA1inPBS
chr16	21345748	21345749	MACS_peak_5565	5.00	lobChIP_FOXA1inPBS
chr16	21348476	21348477	MACS_peak_5566	8.00	lobChIP_FOXA1inPBS
chr16	21350493	21350494	MACS_peak_5567	12.00	lobChIP_FOXA1inPBS
chr16	21516878	21516879	MACS_peak_5568	12.00	lobChIP_FOXA1inPBS
chr16	21535343	21535344	MACS_peak_5569	12.00	lobChIP_FOXA1inPBS
chr16	21536812	21536813	MACS_peak_5570	13.00	lobChIP_FOXA1inPBS
chr16	21538366	21538367	MACS_peak_5571	8.00	lobChIP_FOXA1inPBS
chr16	21545992	21545993	MACS_peak_5572	10.00	lobChIP_FOXA1inPBS
chr16	21577469	21577470	MACS_peak_5573	36.00	lobChIP_FOXA1inPBS
chr16	21639617	21639618	MACS_peak_5574	19.00	lobChIP_FOXA1inPBS
chr16	21650215	21650216	MACS_peak_5575	51.00	lobChIP_FOXA1inPBS
chr16	21946571	21946572	MACS_peak_5576	9.00	lobChIP_FOXA1inPBS
chr16	21948807	21948808	MACS_peak_5577	126.00	lobChIP_FOXA1inPBS
chr16	22017782	22017783	MACS_peak_5578	9.00	lobChIP_FOXA1inPBS
chr16	22038113	22038114	MACS_peak_5579	44.00	lobChIP_FOXA1inPBS
chr16	22217391	22217392	MACS_peak_5580	8.00	lobChIP_FOXA1inPBS
chr16	22309490	22309491	MACS_peak_5581	19.00	lobChIP_FOXA1inPBS
chr16	22383102	22383103	MACS_peak_5582	13.00	lobChIP_FOXA1inPBS
chr16	22405996	22405997	MACS_peak_5583	32.00	lobChIP_FOXA1inPBS
chr16	22900353	22900354	MACS_peak_5584	13.00	lobChIP_FOXA1inPBS
chr16	23104293	23104294	MACS_peak_5585	12.00	lobChIP_FOXA1inPBS
chr16	23115954	23115955	MACS_peak_5586	32.00	lobChIP_FOXA1inPBS
chr16	23161533	23161534	MACS_peak_5587	10.00	lobChIP_FOXA1inPBS
chr16	23252608	23252609	MACS_peak_5588	63.00	lobChIP_FOXA1inPBS
chr16	23336328	23336329	MACS_peak_5589	14.00	lobChIP_FOXA1inPBS
chr16	23446034	23446035	MACS_peak_5590	6.00	lobChIP_FOXA1inPBS
chr16	23463998	23463999	MACS_peak_5591	7.00	lobChIP_FOXA1inPBS
chr16	23551782	23551783	MACS_peak_5592	38.00	lobChIP_FOXA1inPBS
chr16	23685772	23685773	MACS_peak_5593	7.00	lobChIP_FOXA1inPBS
chr16	23734373	23734374	MACS_peak_5594	11.00	lobChIP_FOXA1inPBS
chr16	24053437	24053438	MACS_peak_5595	8.00	lobChIP_FOXA1inPBS
chr16	24748949	24748950	MACS_peak_5596	13.00	lobChIP_FOXA1inPBS
chr16	24853302	24853303	MACS_peak_5597	11.00	lobChIP_FOXA1inPBS
chr16	24998345	24998346	MACS_peak_5598	10.00	lobChIP_FOXA1inPBS
chr16	25011394	25011395	MACS_peak_5599	11.00	lobChIP_FOXA1inPBS
chr16	25189568	25189569	MACS_peak_5600	14.00	lobChIP_FOXA1inPBS
chr16	25310017	25310018	MACS_peak_5601	23.00	lobChIP_FOXA1inPBS
chr16	26541976	26541977	MACS_peak_5602	9.00	lobChIP_FOXA1inPBS
chr16	27180993	27180994	MACS_peak_5603	15.00	lobChIP_FOXA1inPBS
chr16	27254430	27254431	MACS_peak_5604	12.00	lobChIP_FOXA1inPBS
chr16	27269818	27269819	MACS_peak_5605	26.00	lobChIP_FOXA1inPBS
chr16	27275676	27275677	MACS_peak_5606	8.00	lobChIP_FOXA1inPBS
chr16	27281386	27281387	MACS_peak_5607	20.00	lobChIP_FOXA1inPBS
chr16	27285441	27285442	MACS_peak_5608	12.00	lobChIP_FOXA1inPBS
chr16	27376028	27376029	MACS_peak_5609	86.00	lobChIP_FOXA1inPBS
chr16	27590631	27590632	MACS_peak_5610	17.00	lobChIP_FOXA1inPBS
chr16	27607024	27607025	MACS_peak_5611	7.00	lobChIP_FOXA1inPBS
chr16	28194883	28194884	MACS_peak_5612	19.00	lobChIP_FOXA1inPBS
chr16	28336112	28336113	MACS_peak_5613	17.00	lobChIP_FOXA1inPBS
chr16	28537868	28537869	MACS_peak_5614	27.00	lobChIP_FOXA1inPBS
chr16	28538399	28538400	MACS_peak_5615	7.00	lobChIP_FOXA1inPBS
chr16	28552542	28552543	MACS_peak_5616	17.00	lobChIP_FOXA1inPBS
chr16	28934799	28934800	MACS_peak_5617	16.00	lobChIP_FOXA1inPBS
chr16	29161490	29161491	MACS_peak_5618	9.00	lobChIP_FOXA1inPBS
chr16	29168562	29168563	MACS_peak_5619	8.00	lobChIP_FOXA1inPBS
chr16	29172238	29172239	MACS_peak_5620	12.00	lobChIP_FOXA1inPBS
chr16	29272523	29272524	MACS_peak_5621	8.00	lobChIP_FOXA1inPBS
chr16	29273057	29273058	MACS_peak_5622	7.00	lobChIP_FOXA1inPBS
chr16	29609988	29609989	MACS_peak_5623	13.00	lobChIP_FOXA1inPBS
chr16	29671985	29671986	MACS_peak_5624	12.00	lobChIP_FOXA1inPBS
chr16	29770110	29770111	MACS_peak_5625	11.00	lobChIP_FOXA1inPBS
chr16	29776960	29776961	MACS_peak_5626	11.00	lobChIP_FOXA1inPBS
chr16	29816915	29816916	MACS_peak_5627	13.00	lobChIP_FOXA1inPBS
chr16	29836196	29836197	MACS_peak_5628	9.00	lobChIP_FOXA1inPBS
chr16	30114493	30114494	MACS_peak_5629	9.00	lobChIP_FOXA1inPBS
chr16	30151397	30151398	MACS_peak_5630	42.00	lobChIP_FOXA1inPBS
chr16	30413273	30413274	MACS_peak_5631	21.00	lobChIP_FOXA1inPBS
chr16	30449220	30449221	MACS_peak_5632	15.00	lobChIP_FOXA1inPBS
chr16	30453850	30453851	MACS_peak_5633	25.00	lobChIP_FOXA1inPBS
chr16	30459449	30459450	MACS_peak_5634	8.00	lobChIP_FOXA1inPBS
chr16	30500207	30500208	MACS_peak_5635	13.00	lobChIP_FOXA1inPBS
chr16	30552567	30552568	MACS_peak_5636	6.00	lobChIP_FOXA1inPBS
chr16	30583562	30583563	MACS_peak_5637	11.00	lobChIP_FOXA1inPBS
chr16	30609656	30609657	MACS_peak_5638	8.00	lobChIP_FOXA1inPBS
chr16	30820679	30820680	MACS_peak_5639	10.00	lobChIP_FOXA1inPBS
chr16	30825667	30825668	MACS_peak_5640	8.00	lobChIP_FOXA1inPBS
chr16	30834640	30834641	MACS_peak_5641	8.00	lobChIP_FOXA1inPBS
chr16	30835663	30835664	MACS_peak_5642	11.00	lobChIP_FOXA1inPBS
chr16	30850357	30850358	MACS_peak_5643	9.00	lobChIP_FOXA1inPBS
chr16	30912062	30912063	MACS_peak_5644	9.00	lobChIP_FOXA1inPBS
chr16	30943518	30943519	MACS_peak_5645	10.00	lobChIP_FOXA1inPBS
chr16	31112483	31112484	MACS_peak_5646	7.00	lobChIP_FOXA1inPBS
chr16	31293774	31293775	MACS_peak_5647	25.00	lobChIP_FOXA1inPBS
chr16	31708427	31708428	MACS_peak_5648	8.00	lobChIP_FOXA1inPBS
chr16	31712537	31712538	MACS_peak_5649	22.00	lobChIP_FOXA1inPBS
chr16	35032321	35032322	MACS_peak_5651	7.00	lobChIP_FOXA1inPBS
chr16	46664242	46664243	MACS_peak_5652	8.00	lobChIP_FOXA1inPBS
chr16	46804884	46804885	MACS_peak_5653	12.00	lobChIP_FOXA1inPBS
chr16	46823173	46823174	MACS_peak_5654	14.00	lobChIP_FOXA1inPBS
chr16	46898435	46898436	MACS_peak_5655	17.00	lobChIP_FOXA1inPBS
chr16	46910698	46910699	MACS_peak_5656	64.00	lobChIP_FOXA1inPBS
chr16	46917481	46917482	MACS_peak_5657	9.00	lobChIP_FOXA1inPBS
chr16	46930044	46930045	MACS_peak_5658	16.00	lobChIP_FOXA1inPBS
chr16	47474285	47474286	MACS_peak_5659	11.00	lobChIP_FOXA1inPBS
chr16	47509800	47509801	MACS_peak_5660	15.00	lobChIP_FOXA1inPBS
chr16	47533667	47533668	MACS_peak_5661	11.00	lobChIP_FOXA1inPBS
chr16	47572535	47572536	MACS_peak_5662	36.00	lobChIP_FOXA1inPBS
chr16	47615189	47615190	MACS_peak_5663	11.00	lobChIP_FOXA1inPBS
chr16	47668176	47668177	MACS_peak_5664	42.00	lobChIP_FOXA1inPBS
chr16	47691185	47691186	MACS_peak_5665	19.00	lobChIP_FOXA1inPBS
chr16	47808801	47808802	MACS_peak_5666	11.00	lobChIP_FOXA1inPBS
chr16	48110740	48110741	MACS_peak_5667	19.00	lobChIP_FOXA1inPBS
chr16	48199546	48199547	MACS_peak_5668	10.00	lobChIP_FOXA1inPBS
chr16	48238599	48238600	MACS_peak_5669	16.00	lobChIP_FOXA1inPBS
chr16	48263235	48263236	MACS_peak_5670	62.00	lobChIP_FOXA1inPBS
chr16	48269762	48269763	MACS_peak_5671	23.00	lobChIP_FOXA1inPBS
chr16	48270407	48270408	MACS_peak_5672	13.00	lobChIP_FOXA1inPBS
chr16	48326528	48326529	MACS_peak_5673	11.00	lobChIP_FOXA1inPBS
chr16	48475918	48475919	MACS_peak_5674	41.00	lobChIP_FOXA1inPBS
chr16	48482067	48482068	MACS_peak_5675	9.00	lobChIP_FOXA1inPBS
chr16	48514007	48514008	MACS_peak_5676	9.00	lobChIP_FOXA1inPBS
chr16	48558550	48558551	MACS_peak_5677	9.00	lobChIP_FOXA1inPBS
chr16	49590517	49590518	MACS_peak_5678	11.00	lobChIP_FOXA1inPBS
chr16	49883441	49883442	MACS_peak_5679	8.00	lobChIP_FOXA1inPBS
chr16	49968682	49968683	MACS_peak_5680	14.00	lobChIP_FOXA1inPBS
chr16	50058704	50058705	MACS_peak_5681	10.00	lobChIP_FOXA1inPBS
chr16	50069516	50069517	MACS_peak_5682	37.00	lobChIP_FOXA1inPBS
chr16	50157488	50157489	MACS_peak_5683	9.00	lobChIP_FOXA1inPBS
chr16	50189087	50189088	MACS_peak_5684	51.00	lobChIP_FOXA1inPBS
chr16	50232875	50232876	MACS_peak_5685	12.00	lobChIP_FOXA1inPBS
chr16	50295979	50295980	MACS_peak_5686	17.00	lobChIP_FOXA1inPBS
chr16	50298440	50298441	MACS_peak_5687	10.00	lobChIP_FOXA1inPBS
chr16	50338133	50338134	MACS_peak_5688	7.00	lobChIP_FOXA1inPBS
chr16	50371065	50371066	MACS_peak_5689	8.00	lobChIP_FOXA1inPBS
chr16	50472030	50472031	MACS_peak_5690	12.00	lobChIP_FOXA1inPBS
chr16	50497839	50497840	MACS_peak_5691	10.00	lobChIP_FOXA1inPBS
chr16	50502056	50502057	MACS_peak_5692	8.00	lobChIP_FOXA1inPBS
chr16	50554657	50554658	MACS_peak_5693	28.00	lobChIP_FOXA1inPBS
chr16	50573250	50573251	MACS_peak_5694	11.00	lobChIP_FOXA1inPBS
chr16	50581442	50581443	MACS_peak_5695	8.00	lobChIP_FOXA1inPBS
chr16	50651578	50651579	MACS_peak_5696	8.00	lobChIP_FOXA1inPBS
chr16	50840047	50840048	MACS_peak_5697	12.00	lobChIP_FOXA1inPBS
chr16	51092068	51092069	MACS_peak_5698	16.00	lobChIP_FOXA1inPBS
chr16	51154844	51154845	MACS_peak_5699	11.00	lobChIP_FOXA1inPBS
chr16	51272077	51272078	MACS_peak_5700	47.00	lobChIP_FOXA1inPBS
chr16	51278186	51278187	MACS_peak_5701	15.00	lobChIP_FOXA1inPBS
chr16	52086960	52086961	MACS_peak_5702	10.00	lobChIP_FOXA1inPBS
chr16	52133451	52133452	MACS_peak_5703	28.00	lobChIP_FOXA1inPBS
chr16	52299357	52299358	MACS_peak_5704	8.00	lobChIP_FOXA1inPBS
chr16	52417745	52417746	MACS_peak_5705	8.00	lobChIP_FOXA1inPBS
chr16	52432681	52432682	MACS_peak_5706	18.00	lobChIP_FOXA1inPBS
chr16	52442609	52442610	MACS_peak_5707	36.00	lobChIP_FOXA1inPBS
chr16	52486199	52486200	MACS_peak_5708	12.00	lobChIP_FOXA1inPBS
chr16	52490665	52490666	MACS_peak_5709	10.00	lobChIP_FOXA1inPBS
chr16	52522942	52522943	MACS_peak_5710	16.00	lobChIP_FOXA1inPBS
chr16	52526897	52526898	MACS_peak_5711	23.00	lobChIP_FOXA1inPBS
chr16	52530606	52530607	MACS_peak_5712	66.00	lobChIP_FOXA1inPBS
chr16	52596135	52596136	MACS_peak_5713	61.00	lobChIP_FOXA1inPBS
chr16	52596716	52596717	MACS_peak_5714	8.00	lobChIP_FOXA1inPBS
chr16	52599083	52599084	MACS_peak_5715	18.00	lobChIP_FOXA1inPBS
chr16	52607695	52607696	MACS_peak_5716	12.00	lobChIP_FOXA1inPBS
chr16	52612207	52612208	MACS_peak_5717	26.00	lobChIP_FOXA1inPBS
chr16	52631789	52631790	MACS_peak_5718	22.00	lobChIP_FOXA1inPBS
chr16	52646067	52646068	MACS_peak_5719	28.00	lobChIP_FOXA1inPBS
chr16	53082563	53082564	MACS_peak_5720	12.00	lobChIP_FOXA1inPBS
chr16	53256590	53256591	MACS_peak_5721	5.00	lobChIP_FOXA1inPBS
chr16	53355355	53355356	MACS_peak_5722	6.00	lobChIP_FOXA1inPBS
chr16	53545444	53545445	MACS_peak_5723	14.00	lobChIP_FOXA1inPBS
chr16	53706568	53706569	MACS_peak_5724	28.00	lobChIP_FOXA1inPBS
chr16	53778637	53778638	MACS_peak_5725	7.00	lobChIP_FOXA1inPBS
chr16	53790754	53790755	MACS_peak_5726	12.00	lobChIP_FOXA1inPBS
chr16	53809923	53809924	MACS_peak_5727	18.00	lobChIP_FOXA1inPBS
chr16	53815293	53815294	MACS_peak_5728	24.00	lobChIP_FOXA1inPBS
chr16	53915637	53915638	MACS_peak_5729	13.00	lobChIP_FOXA1inPBS
chr16	53927826	53927827	MACS_peak_5730	8.00	lobChIP_FOXA1inPBS
chr16	53933119	53933120	MACS_peak_5731	27.00	lobChIP_FOXA1inPBS
chr16	54025760	54025761	MACS_peak_5732	13.00	lobChIP_FOXA1inPBS
chr16	54032178	54032179	MACS_peak_5733	10.00	lobChIP_FOXA1inPBS
chr16	54034139	54034140	MACS_peak_5734	10.00	lobChIP_FOXA1inPBS
chr16	54074329	54074330	MACS_peak_5735	7.00	lobChIP_FOXA1inPBS
chr16	54101728	54101729	MACS_peak_5736	35.00	lobChIP_FOXA1inPBS
chr16	54114789	54114790	MACS_peak_5737	31.00	lobChIP_FOXA1inPBS
chr16	54159096	54159097	MACS_peak_5738	13.00	lobChIP_FOXA1inPBS
chr16	54195649	54195650	MACS_peak_5739	10.00	lobChIP_FOXA1inPBS
chr16	54199291	54199292	MACS_peak_5740	30.00	lobChIP_FOXA1inPBS
chr16	54205966	54205967	MACS_peak_5741	9.00	lobChIP_FOXA1inPBS
chr16	54206374	54206375	MACS_peak_5742	10.00	lobChIP_FOXA1inPBS
chr16	54210361	54210362	MACS_peak_5743	34.00	lobChIP_FOXA1inPBS
chr16	54215062	54215063	MACS_peak_5744	16.00	lobChIP_FOXA1inPBS
chr16	54300458	54300459	MACS_peak_5745	14.00	lobChIP_FOXA1inPBS
chr16	54464569	54464570	MACS_peak_5746	42.00	lobChIP_FOXA1inPBS
chr16	54662958	54662959	MACS_peak_5747	59.00	lobChIP_FOXA1inPBS
chr16	54881726	54881727	MACS_peak_5748	12.00	lobChIP_FOXA1inPBS
chr16	55513782	55513783	MACS_peak_5749	10.00	lobChIP_FOXA1inPBS
chr16	55540138	55540139	MACS_peak_5750	18.00	lobChIP_FOXA1inPBS
chr16	55547794	55547795	MACS_peak_5751	13.00	lobChIP_FOXA1inPBS
chr16	55655810	55655811	MACS_peak_5752	8.00	lobChIP_FOXA1inPBS
chr16	55657296	55657297	MACS_peak_5753	17.00	lobChIP_FOXA1inPBS
chr16	55671104	55671105	MACS_peak_5754	15.00	lobChIP_FOXA1inPBS
chr16	55786934	55786935	MACS_peak_5755	8.00	lobChIP_FOXA1inPBS
chr16	55867195	55867196	MACS_peak_5756	9.00	lobChIP_FOXA1inPBS
chr16	56404960	56404961	MACS_peak_5757	9.00	lobChIP_FOXA1inPBS
chr16	56640850	56640851	MACS_peak_5758	14.00	lobChIP_FOXA1inPBS
chr16	56847057	56847058	MACS_peak_5759	10.00	lobChIP_FOXA1inPBS
chr16	56852036	56852037	MACS_peak_5760	23.00	lobChIP_FOXA1inPBS
chr16	56873117	56873118	MACS_peak_5761	18.00	lobChIP_FOXA1inPBS
chr16	56953344	56953345	MACS_peak_5762	44.00	lobChIP_FOXA1inPBS
chr16	56956506	56956507	MACS_peak_5763	11.00	lobChIP_FOXA1inPBS
chr16	56967076	56967077	MACS_peak_5764	30.00	lobChIP_FOXA1inPBS
chr16	56983284	56983285	MACS_peak_5765	25.00	lobChIP_FOXA1inPBS
chr16	57001855	57001856	MACS_peak_5766	20.00	lobChIP_FOXA1inPBS
chr16	57038656	57038657	MACS_peak_5767	14.00	lobChIP_FOXA1inPBS
chr16	57212782	57212783	MACS_peak_5768	11.00	lobChIP_FOXA1inPBS
chr16	57228670	57228671	MACS_peak_5769	13.00	lobChIP_FOXA1inPBS
chr16	57281857	57281858	MACS_peak_5770	9.00	lobChIP_FOXA1inPBS
chr16	57314101	57314102	MACS_peak_5771	40.00	lobChIP_FOXA1inPBS
chr16	57407028	57407029	MACS_peak_5772	9.00	lobChIP_FOXA1inPBS
chr16	57506154	57506155	MACS_peak_5773	10.00	lobChIP_FOXA1inPBS
chr16	57641025	57641026	MACS_peak_5774	11.00	lobChIP_FOXA1inPBS
chr16	57658010	57658011	MACS_peak_5775	7.00	lobChIP_FOXA1inPBS
chr16	57711732	57711733	MACS_peak_5776	16.00	lobChIP_FOXA1inPBS
chr16	57728348	57728349	MACS_peak_5777	7.00	lobChIP_FOXA1inPBS
chr16	57737953	57737954	MACS_peak_5778	23.00	lobChIP_FOXA1inPBS
chr16	57743634	57743635	MACS_peak_5779	14.00	lobChIP_FOXA1inPBS
chr16	57770828	57770829	MACS_peak_5780	15.00	lobChIP_FOXA1inPBS
chr16	57815801	57815802	MACS_peak_5781	6.00	lobChIP_FOXA1inPBS
chr16	57844332	57844333	MACS_peak_5782	10.00	lobChIP_FOXA1inPBS
chr16	57860691	57860692	MACS_peak_5783	11.00	lobChIP_FOXA1inPBS
chr16	57928735	57928736	MACS_peak_5784	47.00	lobChIP_FOXA1inPBS
chr16	58112139	58112140	MACS_peak_5785	9.00	lobChIP_FOXA1inPBS
chr16	58161115	58161116	MACS_peak_5786	17.00	lobChIP_FOXA1inPBS
chr16	58340367	58340368	MACS_peak_5787	16.00	lobChIP_FOXA1inPBS
chr16	58370764	58370765	MACS_peak_5788	7.00	lobChIP_FOXA1inPBS
chr16	58668579	58668580	MACS_peak_5789	6.00	lobChIP_FOXA1inPBS
chr16	58755042	58755043	MACS_peak_5790	69.00	lobChIP_FOXA1inPBS
chr16	58807957	58807958	MACS_peak_5791	36.00	lobChIP_FOXA1inPBS
chr16	58882151	58882152	MACS_peak_5792	11.00	lobChIP_FOXA1inPBS
chr16	59214312	59214313	MACS_peak_5793	19.00	lobChIP_FOXA1inPBS
chr16	63194617	63194618	MACS_peak_5794	8.00	lobChIP_FOXA1inPBS
chr16	64195213	64195214	MACS_peak_5795	19.00	lobChIP_FOXA1inPBS
chr16	65675864	65675865	MACS_peak_5796	12.00	lobChIP_FOXA1inPBS
chr16	65678888	65678889	MACS_peak_5797	17.00	lobChIP_FOXA1inPBS
chr16	65700177	65700178	MACS_peak_5798	9.00	lobChIP_FOXA1inPBS
chr16	66361032	66361033	MACS_peak_5799	16.00	lobChIP_FOXA1inPBS
chr16	66467620	66467621	MACS_peak_5800	22.00	lobChIP_FOXA1inPBS
chr16	66547148	66547149	MACS_peak_5801	27.00	lobChIP_FOXA1inPBS
chr16	66550956	66550957	MACS_peak_5802	26.00	lobChIP_FOXA1inPBS
chr16	66939290	66939291	MACS_peak_5803	7.00	lobChIP_FOXA1inPBS
chr16	66971296	66971297	MACS_peak_5804	6.00	lobChIP_FOXA1inPBS
chr16	67030470	67030471	MACS_peak_5805	9.00	lobChIP_FOXA1inPBS
chr16	67145271	67145272	MACS_peak_5806	54.00	lobChIP_FOXA1inPBS
chr16	67148091	67148092	MACS_peak_5807	23.00	lobChIP_FOXA1inPBS
chr16	67194262	67194263	MACS_peak_5808	14.00	lobChIP_FOXA1inPBS
chr16	67512014	67512015	MACS_peak_5809	16.00	lobChIP_FOXA1inPBS
chr16	67597890	67597891	MACS_peak_5810	11.00	lobChIP_FOXA1inPBS
chr16	67926767	67926768	MACS_peak_5811	6.00	lobChIP_FOXA1inPBS
chr16	67977340	67977341	MACS_peak_5812	9.00	lobChIP_FOXA1inPBS
chr16	68113902	68113903	MACS_peak_5813	21.00	lobChIP_FOXA1inPBS
chr16	68118263	68118264	MACS_peak_5814	15.00	lobChIP_FOXA1inPBS
chr16	68147132	68147133	MACS_peak_5815	12.00	lobChIP_FOXA1inPBS
chr16	68268521	68268522	MACS_peak_5816	14.00	lobChIP_FOXA1inPBS
chr16	68271582	68271583	MACS_peak_5817	9.00	lobChIP_FOXA1inPBS
chr16	68299264	68299265	MACS_peak_5818	36.00	lobChIP_FOXA1inPBS
chr16	68315779	68315780	MACS_peak_5819	28.00	lobChIP_FOXA1inPBS
chr16	68348158	68348159	MACS_peak_5820	6.00	lobChIP_FOXA1inPBS
chr16	68774144	68774145	MACS_peak_5821	13.00	lobChIP_FOXA1inPBS
chr16	68778851	68778852	MACS_peak_5822	30.00	lobChIP_FOXA1inPBS
chr16	68780396	68780397	MACS_peak_5823	14.00	lobChIP_FOXA1inPBS
chr16	68783364	68783365	MACS_peak_5824	20.00	lobChIP_FOXA1inPBS
chr16	68824283	68824284	MACS_peak_5825	21.00	lobChIP_FOXA1inPBS
chr16	68826742	68826743	MACS_peak_5826	19.00	lobChIP_FOXA1inPBS
chr16	68895419	68895420	MACS_peak_5827	10.00	lobChIP_FOXA1inPBS
chr16	68941790	68941791	MACS_peak_5828	10.00	lobChIP_FOXA1inPBS
chr16	68993108	68993109	MACS_peak_5829	36.00	lobChIP_FOXA1inPBS
chr16	69026564	69026565	MACS_peak_5830	21.00	lobChIP_FOXA1inPBS
chr16	69224462	69224463	MACS_peak_5831	6.00	lobChIP_FOXA1inPBS
chr16	69255852	69255853	MACS_peak_5832	26.00	lobChIP_FOXA1inPBS
chr16	69300432	69300433	MACS_peak_5833	13.00	lobChIP_FOXA1inPBS
chr16	69384782	69384783	MACS_peak_5834	12.00	lobChIP_FOXA1inPBS
chr16	69430651	69430652	MACS_peak_5835	79.00	lobChIP_FOXA1inPBS
chr16	69470423	69470424	MACS_peak_5836	8.00	lobChIP_FOXA1inPBS
chr16	69555436	69555437	MACS_peak_5837	16.00	lobChIP_FOXA1inPBS
chr16	69579741	69579742	MACS_peak_5838	9.00	lobChIP_FOXA1inPBS
chr16	69596051	69596052	MACS_peak_5839	17.00	lobChIP_FOXA1inPBS
chr16	69649609	69649610	MACS_peak_5840	23.00	lobChIP_FOXA1inPBS
chr16	69702364	69702365	MACS_peak_5841	10.00	lobChIP_FOXA1inPBS
chr16	69706039	69706040	MACS_peak_5842	9.00	lobChIP_FOXA1inPBS
chr16	69714453	69714454	MACS_peak_5843	24.00	lobChIP_FOXA1inPBS
chr16	69757863	69757864	MACS_peak_5844	15.00	lobChIP_FOXA1inPBS
chr16	69805703	69805704	MACS_peak_5845	13.00	lobChIP_FOXA1inPBS
chr16	69848743	69848744	MACS_peak_5846	16.00	lobChIP_FOXA1inPBS
chr16	69855253	69855254	MACS_peak_5847	21.00	lobChIP_FOXA1inPBS
chr16	69856414	69856415	MACS_peak_5848	15.00	lobChIP_FOXA1inPBS
chr16	69857929	69857930	MACS_peak_5849	8.00	lobChIP_FOXA1inPBS
chr16	69861094	69861095	MACS_peak_5850	7.00	lobChIP_FOXA1inPBS
chr16	69864812	69864813	MACS_peak_5851	11.00	lobChIP_FOXA1inPBS
chr16	69892246	69892247	MACS_peak_5852	10.00	lobChIP_FOXA1inPBS
chr16	69944267	69944268	MACS_peak_5853	15.00	lobChIP_FOXA1inPBS
chr16	70170622	70170623	MACS_peak_5854	9.00	lobChIP_FOXA1inPBS
chr16	70191495	70191496	MACS_peak_5855	15.00	lobChIP_FOXA1inPBS
chr16	70248044	70248045	MACS_peak_5856	22.00	lobChIP_FOXA1inPBS
chr16	70333394	70333395	MACS_peak_5857	20.00	lobChIP_FOXA1inPBS
chr16	70462324	70462325	MACS_peak_5858	11.00	lobChIP_FOXA1inPBS
chr16	70474139	70474140	MACS_peak_5859	6.00	lobChIP_FOXA1inPBS
chr16	70616719	70616720	MACS_peak_5860	12.00	lobChIP_FOXA1inPBS
chr16	70716132	70716133	MACS_peak_5861	12.00	lobChIP_FOXA1inPBS
chr16	70759514	70759515	MACS_peak_5862	20.00	lobChIP_FOXA1inPBS
chr16	70779965	70779966	MACS_peak_5863	17.00	lobChIP_FOXA1inPBS
chr16	70780824	70780825	MACS_peak_5864	19.00	lobChIP_FOXA1inPBS
chr16	71589634	71589635	MACS_peak_5865	13.00	lobChIP_FOXA1inPBS
chr16	71592303	71592304	MACS_peak_5866	5.00	lobChIP_FOXA1inPBS
chr16	71606554	71606555	MACS_peak_5867	21.00	lobChIP_FOXA1inPBS
chr16	71619258	71619259	MACS_peak_5868	22.00	lobChIP_FOXA1inPBS
chr16	71627575	71627576	MACS_peak_5869	20.00	lobChIP_FOXA1inPBS
chr16	71629599	71629600	MACS_peak_5870	69.00	lobChIP_FOXA1inPBS
chr16	71632535	71632536	MACS_peak_5871	22.00	lobChIP_FOXA1inPBS
chr16	71704693	71704694	MACS_peak_5872	11.00	lobChIP_FOXA1inPBS
chr16	71929153	71929154	MACS_peak_5873	7.00	lobChIP_FOXA1inPBS
chr16	72013709	72013710	MACS_peak_5874	10.00	lobChIP_FOXA1inPBS
chr16	72016146	72016147	MACS_peak_5875	18.00	lobChIP_FOXA1inPBS
chr16	72051281	72051282	MACS_peak_5876	11.00	lobChIP_FOXA1inPBS
chr16	72097008	72097009	MACS_peak_5877	7.00	lobChIP_FOXA1inPBS
chr16	72106849	72106850	MACS_peak_5878	16.00	lobChIP_FOXA1inPBS
chr16	72258655	72258656	MACS_peak_5879	7.00	lobChIP_FOXA1inPBS
chr16	72343000	72343001	MACS_peak_5880	54.00	lobChIP_FOXA1inPBS
chr16	72449528	72449529	MACS_peak_5881	18.00	lobChIP_FOXA1inPBS
chr16	72602594	72602595	MACS_peak_5882	25.00	lobChIP_FOXA1inPBS
chr16	72711885	72711886	MACS_peak_5883	38.00	lobChIP_FOXA1inPBS
chr16	72911533	72911534	MACS_peak_5884	10.00	lobChIP_FOXA1inPBS
chr16	72948464	72948465	MACS_peak_5885	8.00	lobChIP_FOXA1inPBS
chr16	72959042	72959043	MACS_peak_5886	51.00	lobChIP_FOXA1inPBS
chr16	72961737	72961738	MACS_peak_5887	28.00	lobChIP_FOXA1inPBS
chr16	73050073	73050074	MACS_peak_5888	13.00	lobChIP_FOXA1inPBS
chr16	73054448	73054449	MACS_peak_5889	47.00	lobChIP_FOXA1inPBS
chr16	73057982	73057983	MACS_peak_5890	10.00	lobChIP_FOXA1inPBS
chr16	73084880	73084881	MACS_peak_5891	9.00	lobChIP_FOXA1inPBS
chr16	73089863	73089864	MACS_peak_5892	16.00	lobChIP_FOXA1inPBS
chr16	73183392	73183393	MACS_peak_5893	7.00	lobChIP_FOXA1inPBS
chr16	74330418	74330419	MACS_peak_5894	23.00	lobChIP_FOXA1inPBS
chr16	74669413	74669414	MACS_peak_5895	23.00	lobChIP_FOXA1inPBS
chr16	75022982	75022983	MACS_peak_5896	17.00	lobChIP_FOXA1inPBS
chr16	75025607	75025608	MACS_peak_5897	16.00	lobChIP_FOXA1inPBS
chr16	75032166	75032167	MACS_peak_5898	12.00	lobChIP_FOXA1inPBS
chr16	75088317	75088318	MACS_peak_5899	20.00	lobChIP_FOXA1inPBS
chr16	75098482	75098483	MACS_peak_5900	6.00	lobChIP_FOXA1inPBS
chr16	75104479	75104480	MACS_peak_5901	29.00	lobChIP_FOXA1inPBS
chr16	75106393	75106394	MACS_peak_5902	11.00	lobChIP_FOXA1inPBS
chr16	75121409	75121410	MACS_peak_5903	13.00	lobChIP_FOXA1inPBS
chr16	75339238	75339239	MACS_peak_5904	7.00	lobChIP_FOXA1inPBS
chr16	75340410	75340411	MACS_peak_5905	7.00	lobChIP_FOXA1inPBS
chr16	75344039	75344040	MACS_peak_5906	9.00	lobChIP_FOXA1inPBS
chr16	75344519	75344520	MACS_peak_5907	7.00	lobChIP_FOXA1inPBS
chr16	75467747	75467748	MACS_peak_5908	8.00	lobChIP_FOXA1inPBS
chr16	75480493	75480494	MACS_peak_5909	15.00	lobChIP_FOXA1inPBS
chr16	75561939	75561940	MACS_peak_5910	8.00	lobChIP_FOXA1inPBS
chr16	75628201	75628202	MACS_peak_5911	10.00	lobChIP_FOXA1inPBS
chr16	75682632	75682633	MACS_peak_5912	7.00	lobChIP_FOXA1inPBS
chr16	76608794	76608795	MACS_peak_5913	63.00	lobChIP_FOXA1inPBS
chr16	77338944	77338945	MACS_peak_5914	7.00	lobChIP_FOXA1inPBS
chr16	77393061	77393062	MACS_peak_5915	22.00	lobChIP_FOXA1inPBS
chr16	77758039	77758040	MACS_peak_5919	5.00	lobChIP_FOXA1inPBS
chr16	77766635	77766636	MACS_peak_5920	16.00	lobChIP_FOXA1inPBS
chr16	77771052	77771053	MACS_peak_5921	28.00	lobChIP_FOXA1inPBS
chr16	77783511	77783512	MACS_peak_5922	12.00	lobChIP_FOXA1inPBS
chr16	77810248	77810249	MACS_peak_5923	11.00	lobChIP_FOXA1inPBS
chr16	77847150	77847151	MACS_peak_5924	13.00	lobChIP_FOXA1inPBS
chr16	77939619	77939620	MACS_peak_5925	9.00	lobChIP_FOXA1inPBS
chr16	77952892	77952893	MACS_peak_5926	12.00	lobChIP_FOXA1inPBS
chr16	78168054	78168055	MACS_peak_5927	54.00	lobChIP_FOXA1inPBS
chr16	78169099	78169100	MACS_peak_5928	21.00	lobChIP_FOXA1inPBS
chr16	78234913	78234914	MACS_peak_5929	11.00	lobChIP_FOXA1inPBS
chr16	78286864	78286865	MACS_peak_5930	27.00	lobChIP_FOXA1inPBS
chr16	78324914	78324915	MACS_peak_5931	12.00	lobChIP_FOXA1inPBS
chr16	78331135	78331136	MACS_peak_5932	47.00	lobChIP_FOXA1inPBS
chr16	78514675	78514676	MACS_peak_5933	30.00	lobChIP_FOXA1inPBS
chr16	78517568	78517569	MACS_peak_5934	13.00	lobChIP_FOXA1inPBS
chr16	78519520	78519521	MACS_peak_5935	9.00	lobChIP_FOXA1inPBS
chr16	78556310	78556311	MACS_peak_5936	9.00	lobChIP_FOXA1inPBS
chr16	78621783	78621784	MACS_peak_5937	9.00	lobChIP_FOXA1inPBS
chr16	78626012	78626013	MACS_peak_5938	8.00	lobChIP_FOXA1inPBS
chr16	78631029	78631030	MACS_peak_5939	18.00	lobChIP_FOXA1inPBS
chr16	78739814	78739815	MACS_peak_5940	13.00	lobChIP_FOXA1inPBS
chr16	78763820	78763821	MACS_peak_5941	15.00	lobChIP_FOXA1inPBS
chr16	78768660	78768661	MACS_peak_5942	13.00	lobChIP_FOXA1inPBS
chr16	79635573	79635574	MACS_peak_5943	16.00	lobChIP_FOXA1inPBS
chr16	79931577	79931578	MACS_peak_5944	8.00	lobChIP_FOXA1inPBS
chr16	80048285	80048286	MACS_peak_5945	10.00	lobChIP_FOXA1inPBS
chr16	80219426	80219427	MACS_peak_5946	10.00	lobChIP_FOXA1inPBS
chr16	80297982	80297983	MACS_peak_5947	10.00	lobChIP_FOXA1inPBS
chr16	80441357	80441358	MACS_peak_5948	8.00	lobChIP_FOXA1inPBS
chr16	80917298	80917299	MACS_peak_5949	9.00	lobChIP_FOXA1inPBS
chr16	80944555	80944556	MACS_peak_5950	7.00	lobChIP_FOXA1inPBS
chr16	80948313	80948314	MACS_peak_5951	5.00	lobChIP_FOXA1inPBS
chr16	81025609	81025610	MACS_peak_5952	41.00	lobChIP_FOXA1inPBS
chr16	81156943	81156944	MACS_peak_5953	6.00	lobChIP_FOXA1inPBS
chr16	81170475	81170476	MACS_peak_5954	13.00	lobChIP_FOXA1inPBS
chr16	81464921	81464922	MACS_peak_5955	11.00	lobChIP_FOXA1inPBS
chr16	81480198	81480199	MACS_peak_5956	8.00	lobChIP_FOXA1inPBS
chr16	81485904	81485905	MACS_peak_5957	8.00	lobChIP_FOXA1inPBS
chr16	81583145	81583146	MACS_peak_5958	6.00	lobChIP_FOXA1inPBS
chr16	81586992	81586993	MACS_peak_5959	19.00	lobChIP_FOXA1inPBS
chr16	81592599	81592600	MACS_peak_5960	21.00	lobChIP_FOXA1inPBS
chr16	81802005	81802006	MACS_peak_5961	33.00	lobChIP_FOXA1inPBS
chr16	82084779	82084780	MACS_peak_5962	19.00	lobChIP_FOXA1inPBS
chr16	82089904	82089905	MACS_peak_5963	22.00	lobChIP_FOXA1inPBS
chr16	82093766	82093767	MACS_peak_5964	15.00	lobChIP_FOXA1inPBS
chr16	82096379	82096380	MACS_peak_5965	20.00	lobChIP_FOXA1inPBS
chr16	82116581	82116582	MACS_peak_5966	16.00	lobChIP_FOXA1inPBS
chr16	82365839	82365840	MACS_peak_5967	8.00	lobChIP_FOXA1inPBS
chr16	82558727	82558728	MACS_peak_5968	56.00	lobChIP_FOXA1inPBS
chr16	83847730	83847731	MACS_peak_5969	8.00	lobChIP_FOXA1inPBS
chr16	83933736	83933737	MACS_peak_5970	10.00	lobChIP_FOXA1inPBS
chr16	83976673	83976674	MACS_peak_5971	23.00	lobChIP_FOXA1inPBS
chr16	84077026	84077027	MACS_peak_5972	10.00	lobChIP_FOXA1inPBS
chr16	84082198	84082199	MACS_peak_5973	11.00	lobChIP_FOXA1inPBS
chr16	84118430	84118431	MACS_peak_5974	11.00	lobChIP_FOXA1inPBS
chr16	84130428	84130429	MACS_peak_5975	38.00	lobChIP_FOXA1inPBS
chr16	84349152	84349153	MACS_peak_5976	11.00	lobChIP_FOXA1inPBS
chr16	84448865	84448866	MACS_peak_5977	17.00	lobChIP_FOXA1inPBS
chr16	84569256	84569257	MACS_peak_5978	9.00	lobChIP_FOXA1inPBS
chr16	84574061	84574062	MACS_peak_5979	8.00	lobChIP_FOXA1inPBS
chr16	84633339	84633340	MACS_peak_5980	16.00	lobChIP_FOXA1inPBS
chr16	84747179	84747180	MACS_peak_5981	19.00	lobChIP_FOXA1inPBS
chr16	84885070	84885071	MACS_peak_5982	30.00	lobChIP_FOXA1inPBS
chr16	85010576	85010577	MACS_peak_5983	11.00	lobChIP_FOXA1inPBS
chr16	85028568	85028569	MACS_peak_5984	50.00	lobChIP_FOXA1inPBS
chr16	85032936	85032937	MACS_peak_5985	30.00	lobChIP_FOXA1inPBS
chr16	85034739	85034740	MACS_peak_5986	7.00	lobChIP_FOXA1inPBS
chr16	85253996	85253997	MACS_peak_5987	7.00	lobChIP_FOXA1inPBS
chr16	85341428	85341429	MACS_peak_5988	54.00	lobChIP_FOXA1inPBS
chr16	85456597	85456598	MACS_peak_5989	37.00	lobChIP_FOXA1inPBS
chr16	85482611	85482612	MACS_peak_5990	24.00	lobChIP_FOXA1inPBS
chr16	85494785	85494786	MACS_peak_5991	8.00	lobChIP_FOXA1inPBS
chr16	85548736	85548737	MACS_peak_5992	31.00	lobChIP_FOXA1inPBS
chr16	85558295	85558296	MACS_peak_5993	11.00	lobChIP_FOXA1inPBS
chr16	85569219	85569220	MACS_peak_5994	8.00	lobChIP_FOXA1inPBS
chr16	85585284	85585285	MACS_peak_5995	6.00	lobChIP_FOXA1inPBS
chr16	85592135	85592136	MACS_peak_5996	8.00	lobChIP_FOXA1inPBS
chr16	85655868	85655869	MACS_peak_5997	10.00	lobChIP_FOXA1inPBS
chr16	85833862	85833863	MACS_peak_5998	11.00	lobChIP_FOXA1inPBS
chr16	85878532	85878533	MACS_peak_5999	20.00	lobChIP_FOXA1inPBS
chr16	85879952	85879953	MACS_peak_6000	21.00	lobChIP_FOXA1inPBS
chr16	85881684	85881685	MACS_peak_6001	13.00	lobChIP_FOXA1inPBS
chr16	86134255	86134256	MACS_peak_6002	9.00	lobChIP_FOXA1inPBS
chr16	87033866	87033867	MACS_peak_6003	9.00	lobChIP_FOXA1inPBS
chr16	87203390	87203391	MACS_peak_6004	6.00	lobChIP_FOXA1inPBS
chr16	87275183	87275184	MACS_peak_6005	16.00	lobChIP_FOXA1inPBS
chr16	87280022	87280023	MACS_peak_6006	13.00	lobChIP_FOXA1inPBS
chr16	87413042	87413043	MACS_peak_6007	29.00	lobChIP_FOXA1inPBS
chr16	87416012	87416013	MACS_peak_6008	80.00	lobChIP_FOXA1inPBS
chr16	87418360	87418361	MACS_peak_6009	10.00	lobChIP_FOXA1inPBS
chr16	87421980	87421981	MACS_peak_6010	15.00	lobChIP_FOXA1inPBS
chr16	87562576	87562577	MACS_peak_6011	12.00	lobChIP_FOXA1inPBS
chr16	87739791	87739792	MACS_peak_6012	85.00	lobChIP_FOXA1inPBS
chr16	87822682	87822683	MACS_peak_6013	16.00	lobChIP_FOXA1inPBS
chr16	87823296	87823297	MACS_peak_6014	38.00	lobChIP_FOXA1inPBS
chr16	87824566	87824567	MACS_peak_6015	11.00	lobChIP_FOXA1inPBS
chr16	87881708	87881709	MACS_peak_6016	9.00	lobChIP_FOXA1inPBS
chr16	87887960	87887961	MACS_peak_6017	21.00	lobChIP_FOXA1inPBS
chr16	87891294	87891295	MACS_peak_6018	18.00	lobChIP_FOXA1inPBS
chr16	87966206	87966207	MACS_peak_6019	8.00	lobChIP_FOXA1inPBS
chr16	87981976	87981977	MACS_peak_6020	15.00	lobChIP_FOXA1inPBS
chr16	88149998	88149999	MACS_peak_6021	7.00	lobChIP_FOXA1inPBS
chr16	88152740	88152741	MACS_peak_6022	8.00	lobChIP_FOXA1inPBS
chr16	88267734	88267735	MACS_peak_6023	7.00	lobChIP_FOXA1inPBS
chr16	88268321	88268322	MACS_peak_6024	10.00	lobChIP_FOXA1inPBS
chr16	88555046	88555047	MACS_peak_6025	23.00	lobChIP_FOXA1inPBS
chr16	88570699	88570700	MACS_peak_6026	11.00	lobChIP_FOXA1inPBS
chr16	88575650	88575651	MACS_peak_6027	10.00	lobChIP_FOXA1inPBS
chr16	88664494	88664495	MACS_peak_6028	15.00	lobChIP_FOXA1inPBS
chr16	88684986	88684987	MACS_peak_6029	10.00	lobChIP_FOXA1inPBS
chr16	88830175	88830176	MACS_peak_6030	38.00	lobChIP_FOXA1inPBS
chr16	88994908	88994909	MACS_peak_6031	13.00	lobChIP_FOXA1inPBS
chr16	88995665	88995666	MACS_peak_6032	31.00	lobChIP_FOXA1inPBS
chr16	89253294	89253295	MACS_peak_6033	20.00	lobChIP_FOXA1inPBS
chr16	89264427	89264428	MACS_peak_6034	15.00	lobChIP_FOXA1inPBS
chr16	89312999	89313000	MACS_peak_6035	17.00	lobChIP_FOXA1inPBS
chr16	89489646	89489647	MACS_peak_6036	10.00	lobChIP_FOXA1inPBS
chr16	89511655	89511656	MACS_peak_6037	6.00	lobChIP_FOXA1inPBS
chr16	89523004	89523005	MACS_peak_6038	11.00	lobChIP_FOXA1inPBS
chr16	89575531	89575532	MACS_peak_6039	9.00	lobChIP_FOXA1inPBS
chr16	89676454	89676455	MACS_peak_6040	7.00	lobChIP_FOXA1inPBS
chr16	89890314	89890315	MACS_peak_6041	7.00	lobChIP_FOXA1inPBS
chr16	89894191	89894192	MACS_peak_6042	8.00	lobChIP_FOXA1inPBS
chr16	89904335	89904336	MACS_peak_6043	29.00	lobChIP_FOXA1inPBS
chr16	89980478	89980479	MACS_peak_6044	9.00	lobChIP_FOXA1inPBS
chr16	90076332	90076333	MACS_peak_6045	9.00	lobChIP_FOXA1inPBS
chr17	132523	132524	MACS_peak_6046	12.00	lobChIP_FOXA1inPBS
chr17	198612	198613	MACS_peak_6047	8.00	lobChIP_FOXA1inPBS
chr17	202632	202633	MACS_peak_6048	25.00	lobChIP_FOXA1inPBS
chr17	619680	619681	MACS_peak_6049	12.00	lobChIP_FOXA1inPBS
chr17	833812	833813	MACS_peak_6050	51.00	lobChIP_FOXA1inPBS
chr17	838815	838816	MACS_peak_6051	8.00	lobChIP_FOXA1inPBS
chr17	858094	858095	MACS_peak_6052	14.00	lobChIP_FOXA1inPBS
chr17	1018568	1018569	MACS_peak_6053	7.00	lobChIP_FOXA1inPBS
chr17	1026452	1026453	MACS_peak_6054	13.00	lobChIP_FOXA1inPBS
chr17	1040999	1041000	MACS_peak_6055	11.00	lobChIP_FOXA1inPBS
chr17	1352869	1352870	MACS_peak_6056	12.00	lobChIP_FOXA1inPBS
chr17	1360313	1360314	MACS_peak_6057	16.00	lobChIP_FOXA1inPBS
chr17	1493510	1493511	MACS_peak_6058	9.00	lobChIP_FOXA1inPBS
chr17	1501402	1501403	MACS_peak_6059	7.00	lobChIP_FOXA1inPBS
chr17	1639849	1639850	MACS_peak_6060	30.00	lobChIP_FOXA1inPBS
chr17	1673598	1673599	MACS_peak_6061	45.00	lobChIP_FOXA1inPBS
chr17	1813461	1813462	MACS_peak_6062	9.00	lobChIP_FOXA1inPBS
chr17	1970116	1970117	MACS_peak_6063	33.00	lobChIP_FOXA1inPBS
chr17	2046275	2046276	MACS_peak_6064	12.00	lobChIP_FOXA1inPBS
chr17	2078132	2078133	MACS_peak_6065	11.00	lobChIP_FOXA1inPBS
chr17	2080893	2080894	MACS_peak_6066	12.00	lobChIP_FOXA1inPBS
chr17	2389009	2389010	MACS_peak_6067	11.00	lobChIP_FOXA1inPBS
chr17	2475324	2475325	MACS_peak_6068	7.00	lobChIP_FOXA1inPBS
chr17	2621965	2621966	MACS_peak_6069	7.00	lobChIP_FOXA1inPBS
chr17	2793925	2793926	MACS_peak_6070	6.00	lobChIP_FOXA1inPBS
chr17	2794312	2794313	MACS_peak_6071	11.00	lobChIP_FOXA1inPBS
chr17	2855670	2855671	MACS_peak_6072	10.00	lobChIP_FOXA1inPBS
chr17	2858424	2858425	MACS_peak_6073	9.00	lobChIP_FOXA1inPBS
chr17	3377802	3377803	MACS_peak_6074	7.00	lobChIP_FOXA1inPBS
chr17	3706427	3706428	MACS_peak_6075	15.00	lobChIP_FOXA1inPBS
chr17	4213886	4213887	MACS_peak_6076	6.00	lobChIP_FOXA1inPBS
chr17	4219491	4219492	MACS_peak_6077	10.00	lobChIP_FOXA1inPBS
chr17	4259699	4259700	MACS_peak_6078	7.00	lobChIP_FOXA1inPBS
chr17	4357771	4357772	MACS_peak_6079	25.00	lobChIP_FOXA1inPBS
chr17	4552308	4552309	MACS_peak_6080	14.00	lobChIP_FOXA1inPBS
chr17	4624945	4624946	MACS_peak_6081	10.00	lobChIP_FOXA1inPBS
chr17	4663220	4663221	MACS_peak_6082	21.00	lobChIP_FOXA1inPBS
chr17	4681915	4681916	MACS_peak_6083	19.00	lobChIP_FOXA1inPBS
chr17	4691212	4691213	MACS_peak_6084	42.00	lobChIP_FOXA1inPBS
chr17	4948958	4948959	MACS_peak_6085	9.00	lobChIP_FOXA1inPBS
chr17	5135695	5135696	MACS_peak_6086	135.00	lobChIP_FOXA1inPBS
chr17	5217378	5217379	MACS_peak_6087	14.00	lobChIP_FOXA1inPBS
chr17	5308893	5308894	MACS_peak_6088	13.00	lobChIP_FOXA1inPBS
chr17	5736351	5736352	MACS_peak_6089	10.00	lobChIP_FOXA1inPBS
chr17	6586731	6586732	MACS_peak_6090	23.00	lobChIP_FOXA1inPBS
chr17	6603596	6603597	MACS_peak_6091	17.00	lobChIP_FOXA1inPBS
chr17	6614986	6614987	MACS_peak_6092	7.00	lobChIP_FOXA1inPBS
chr17	6699412	6699413	MACS_peak_6093	10.00	lobChIP_FOXA1inPBS
chr17	6752286	6752287	MACS_peak_6094	10.00	lobChIP_FOXA1inPBS
chr17	6991797	6991798	MACS_peak_6095	20.00	lobChIP_FOXA1inPBS
chr17	7001463	7001464	MACS_peak_6096	11.00	lobChIP_FOXA1inPBS
chr17	7016830	7016831	MACS_peak_6097	8.00	lobChIP_FOXA1inPBS
chr17	7035964	7035965	MACS_peak_6098	7.00	lobChIP_FOXA1inPBS
chr17	7039575	7039576	MACS_peak_6099	6.00	lobChIP_FOXA1inPBS
chr17	7118754	7118755	MACS_peak_6100	5.00	lobChIP_FOXA1inPBS
chr17	7170384	7170385	MACS_peak_6101	11.00	lobChIP_FOXA1inPBS
chr17	7186374	7186375	MACS_peak_6102	13.00	lobChIP_FOXA1inPBS
chr17	7766540	7766541	MACS_peak_6103	16.00	lobChIP_FOXA1inPBS
chr17	7769281	7769282	MACS_peak_6104	9.00	lobChIP_FOXA1inPBS
chr17	7836564	7836565	MACS_peak_6105	9.00	lobChIP_FOXA1inPBS
chr17	7959997	7959998	MACS_peak_6106	64.00	lobChIP_FOXA1inPBS
chr17	8076616	8076617	MACS_peak_6107	19.00	lobChIP_FOXA1inPBS
chr17	8114581	8114582	MACS_peak_6108	59.00	lobChIP_FOXA1inPBS
chr17	8177478	8177479	MACS_peak_6109	6.00	lobChIP_FOXA1inPBS
chr17	8323952	8323953	MACS_peak_6110	23.00	lobChIP_FOXA1inPBS
chr17	8351866	8351867	MACS_peak_6111	6.00	lobChIP_FOXA1inPBS
chr17	8353841	8353842	MACS_peak_6112	11.00	lobChIP_FOXA1inPBS
chr17	8435558	8435559	MACS_peak_6113	25.00	lobChIP_FOXA1inPBS
chr17	8446705	8446706	MACS_peak_6114	17.00	lobChIP_FOXA1inPBS
chr17	8537057	8537058	MACS_peak_6115	7.00	lobChIP_FOXA1inPBS
chr17	8657364	8657365	MACS_peak_6116	39.00	lobChIP_FOXA1inPBS
chr17	8692487	8692488	MACS_peak_6117	41.00	lobChIP_FOXA1inPBS
chr17	9054628	9054629	MACS_peak_6118	55.00	lobChIP_FOXA1inPBS
chr17	9161633	9161634	MACS_peak_6119	29.00	lobChIP_FOXA1inPBS
chr17	9252004	9252005	MACS_peak_6120	10.00	lobChIP_FOXA1inPBS
chr17	9264646	9264647	MACS_peak_6121	9.00	lobChIP_FOXA1inPBS
chr17	9458286	9458287	MACS_peak_6122	6.00	lobChIP_FOXA1inPBS
chr17	9588878	9588879	MACS_peak_6123	9.00	lobChIP_FOXA1inPBS
chr17	9632595	9632596	MACS_peak_6124	11.00	lobChIP_FOXA1inPBS
chr17	9745742	9745743	MACS_peak_6125	7.00	lobChIP_FOXA1inPBS
chr17	10350984	10350985	MACS_peak_6126	9.00	lobChIP_FOXA1inPBS
chr17	10640808	10640809	MACS_peak_6127	16.00	lobChIP_FOXA1inPBS
chr17	10648107	10648108	MACS_peak_6128	11.00	lobChIP_FOXA1inPBS
chr17	10660599	10660600	MACS_peak_6129	9.00	lobChIP_FOXA1inPBS
chr17	11512030	11512031	MACS_peak_6130	18.00	lobChIP_FOXA1inPBS
chr17	11831767	11831768	MACS_peak_6131	10.00	lobChIP_FOXA1inPBS
chr17	11890004	11890005	MACS_peak_6132	9.00	lobChIP_FOXA1inPBS
chr17	13010378	13010379	MACS_peak_6133	16.00	lobChIP_FOXA1inPBS
chr17	13314389	13314390	MACS_peak_6134	11.00	lobChIP_FOXA1inPBS
chr17	13324425	13324426	MACS_peak_6135	21.00	lobChIP_FOXA1inPBS
chr17	13341761	13341762	MACS_peak_6136	6.00	lobChIP_FOXA1inPBS
chr17	13787853	13787854	MACS_peak_6137	11.00	lobChIP_FOXA1inPBS
chr17	13855712	13855713	MACS_peak_6138	20.00	lobChIP_FOXA1inPBS
chr17	14229283	14229284	MACS_peak_6139	33.00	lobChIP_FOXA1inPBS
chr17	14265326	14265327	MACS_peak_6140	11.00	lobChIP_FOXA1inPBS
chr17	14276415	14276416	MACS_peak_6141	26.00	lobChIP_FOXA1inPBS
chr17	14321056	14321057	MACS_peak_6142	13.00	lobChIP_FOXA1inPBS
chr17	14374539	14374540	MACS_peak_6143	11.00	lobChIP_FOXA1inPBS
chr17	14602305	14602306	MACS_peak_6144	9.00	lobChIP_FOXA1inPBS
chr17	14603358	14603359	MACS_peak_6145	40.00	lobChIP_FOXA1inPBS
chr17	14641868	14641869	MACS_peak_6146	13.00	lobChIP_FOXA1inPBS
chr17	15140477	15140478	MACS_peak_6147	15.00	lobChIP_FOXA1inPBS
chr17	15336590	15336591	MACS_peak_6148	9.00	lobChIP_FOXA1inPBS
chr17	15337692	15337693	MACS_peak_6149	9.00	lobChIP_FOXA1inPBS
chr17	15869506	15869507	MACS_peak_6150	13.00	lobChIP_FOXA1inPBS
chr17	15944205	15944206	MACS_peak_6151	13.00	lobChIP_FOXA1inPBS
chr17	16192605	16192606	MACS_peak_6152	24.00	lobChIP_FOXA1inPBS
chr17	16200496	16200497	MACS_peak_6153	10.00	lobChIP_FOXA1inPBS
chr17	16216997	16216998	MACS_peak_6154	12.00	lobChIP_FOXA1inPBS
chr17	16243478	16243479	MACS_peak_6155	11.00	lobChIP_FOXA1inPBS
chr17	16281989	16281990	MACS_peak_6156	7.00	lobChIP_FOXA1inPBS
chr17	16322862	16322863	MACS_peak_6157	8.00	lobChIP_FOXA1inPBS
chr17	16329244	16329245	MACS_peak_6158	9.00	lobChIP_FOXA1inPBS
chr17	16348849	16348850	MACS_peak_6159	9.00	lobChIP_FOXA1inPBS
chr17	16371899	16371900	MACS_peak_6160	11.00	lobChIP_FOXA1inPBS
chr17	16497203	16497204	MACS_peak_6161	15.00	lobChIP_FOXA1inPBS
chr17	17205659	17205660	MACS_peak_6162	13.00	lobChIP_FOXA1inPBS
chr17	17248670	17248671	MACS_peak_6163	8.00	lobChIP_FOXA1inPBS
chr17	17279848	17279849	MACS_peak_6164	14.00	lobChIP_FOXA1inPBS
chr17	17283033	17283034	MACS_peak_6165	11.00	lobChIP_FOXA1inPBS
chr17	17319969	17319970	MACS_peak_6166	6.00	lobChIP_FOXA1inPBS
chr17	17332984	17332985	MACS_peak_6167	9.00	lobChIP_FOXA1inPBS
chr17	17346761	17346762	MACS_peak_6168	40.00	lobChIP_FOXA1inPBS
chr17	17349718	17349719	MACS_peak_6169	63.00	lobChIP_FOXA1inPBS
chr17	17437421	17437422	MACS_peak_6170	60.00	lobChIP_FOXA1inPBS
chr17	17442126	17442127	MACS_peak_6171	26.00	lobChIP_FOXA1inPBS
chr17	17614309	17614310	MACS_peak_6172	6.00	lobChIP_FOXA1inPBS
chr17	17715506	17715507	MACS_peak_6173	7.00	lobChIP_FOXA1inPBS
chr17	17903529	17903530	MACS_peak_6174	6.00	lobChIP_FOXA1inPBS
chr17	17921448	17921449	MACS_peak_6175	18.00	lobChIP_FOXA1inPBS
chr17	18824410	18824411	MACS_peak_6176	7.00	lobChIP_FOXA1inPBS
chr17	18828792	18828793	MACS_peak_6177	15.00	lobChIP_FOXA1inPBS
chr17	18841849	18841850	MACS_peak_6178	10.00	lobChIP_FOXA1inPBS
chr17	18909585	18909586	MACS_peak_6179	25.00	lobChIP_FOXA1inPBS
chr17	18914435	18914436	MACS_peak_6180	7.00	lobChIP_FOXA1inPBS
chr17	19453206	19453207	MACS_peak_6181	13.00	lobChIP_FOXA1inPBS
chr17	19532623	19532624	MACS_peak_6182	7.00	lobChIP_FOXA1inPBS
chr17	19545522	19545523	MACS_peak_6183	6.00	lobChIP_FOXA1inPBS
chr17	19546034	19546035	MACS_peak_6184	8.00	lobChIP_FOXA1inPBS
chr17	19550543	19550544	MACS_peak_6185	10.00	lobChIP_FOXA1inPBS
chr17	19560809	19560810	MACS_peak_6186	7.00	lobChIP_FOXA1inPBS
chr17	19563864	19563865	MACS_peak_6187	33.00	lobChIP_FOXA1inPBS
chr17	19575439	19575440	MACS_peak_6188	8.00	lobChIP_FOXA1inPBS
chr17	19637451	19637452	MACS_peak_6189	19.00	lobChIP_FOXA1inPBS
chr17	19651856	19651857	MACS_peak_6190	25.00	lobChIP_FOXA1inPBS
chr17	19715894	19715895	MACS_peak_6191	8.00	lobChIP_FOXA1inPBS
chr17	19840970	19840971	MACS_peak_6192	14.00	lobChIP_FOXA1inPBS
chr17	20881080	20881081	MACS_peak_6193	8.00	lobChIP_FOXA1inPBS
chr17	20997745	20997746	MACS_peak_6194	11.00	lobChIP_FOXA1inPBS
chr17	25574641	25574642	MACS_peak_6195	13.00	lobChIP_FOXA1inPBS
chr17	25650537	25650538	MACS_peak_6196	10.00	lobChIP_FOXA1inPBS
chr17	25785253	25785254	MACS_peak_6197	9.00	lobChIP_FOXA1inPBS
chr17	25858055	25858056	MACS_peak_6198	11.00	lobChIP_FOXA1inPBS
chr17	25978592	25978593	MACS_peak_6199	8.00	lobChIP_FOXA1inPBS
chr17	26380486	26380487	MACS_peak_6200	16.00	lobChIP_FOXA1inPBS
chr17	26385161	26385162	MACS_peak_6201	19.00	lobChIP_FOXA1inPBS
chr17	26516898	26516899	MACS_peak_6202	11.00	lobChIP_FOXA1inPBS
chr17	26651389	26651390	MACS_peak_6203	11.00	lobChIP_FOXA1inPBS
chr17	26662484	26662485	MACS_peak_6204	13.00	lobChIP_FOXA1inPBS
chr17	26674162	26674163	MACS_peak_6205	6.00	lobChIP_FOXA1inPBS
chr17	26695416	26695417	MACS_peak_6206	12.00	lobChIP_FOXA1inPBS
chr17	26697734	26697735	MACS_peak_6207	37.00	lobChIP_FOXA1inPBS
chr17	26802520	26802521	MACS_peak_6208	17.00	lobChIP_FOXA1inPBS
chr17	27037587	27037588	MACS_peak_6209	14.00	lobChIP_FOXA1inPBS
chr17	27046844	27046845	MACS_peak_6210	7.00	lobChIP_FOXA1inPBS
chr17	27335744	27335745	MACS_peak_6211	10.00	lobChIP_FOXA1inPBS
chr17	27386530	27386531	MACS_peak_6212	9.00	lobChIP_FOXA1inPBS
chr17	27403328	27403329	MACS_peak_6213	9.00	lobChIP_FOXA1inPBS
chr17	27485869	27485870	MACS_peak_6214	25.00	lobChIP_FOXA1inPBS
chr17	27718572	27718573	MACS_peak_6215	9.00	lobChIP_FOXA1inPBS
chr17	27720508	27720509	MACS_peak_6216	7.00	lobChIP_FOXA1inPBS
chr17	27882947	27882948	MACS_peak_6217	17.00	lobChIP_FOXA1inPBS
chr17	28072049	28072050	MACS_peak_6218	25.00	lobChIP_FOXA1inPBS
chr17	28580800	28580801	MACS_peak_6219	6.00	lobChIP_FOXA1inPBS
chr17	28671541	28671542	MACS_peak_6220	13.00	lobChIP_FOXA1inPBS
chr17	28673538	28673539	MACS_peak_6221	6.00	lobChIP_FOXA1inPBS
chr17	28703798	28703799	MACS_peak_6222	13.00	lobChIP_FOXA1inPBS
chr17	28722535	28722536	MACS_peak_6223	15.00	lobChIP_FOXA1inPBS
chr17	28777842	28777843	MACS_peak_6224	14.00	lobChIP_FOXA1inPBS
chr17	29243257	29243258	MACS_peak_6225	24.00	lobChIP_FOXA1inPBS
chr17	29415812	29415813	MACS_peak_6226	11.00	lobChIP_FOXA1inPBS
chr17	29601417	29601418	MACS_peak_6227	13.00	lobChIP_FOXA1inPBS
chr17	29784045	29784046	MACS_peak_6228	10.00	lobChIP_FOXA1inPBS
chr17	29793915	29793916	MACS_peak_6229	6.00	lobChIP_FOXA1inPBS
chr17	29802713	29802714	MACS_peak_6230	28.00	lobChIP_FOXA1inPBS
chr17	29803956	29803957	MACS_peak_6231	9.00	lobChIP_FOXA1inPBS
chr17	29808929	29808930	MACS_peak_6232	7.00	lobChIP_FOXA1inPBS
chr17	29825600	29825601	MACS_peak_6233	10.00	lobChIP_FOXA1inPBS
chr17	30080184	30080185	MACS_peak_6234	9.00	lobChIP_FOXA1inPBS
chr17	30130860	30130861	MACS_peak_6235	32.00	lobChIP_FOXA1inPBS
chr17	30185467	30185468	MACS_peak_6236	8.00	lobChIP_FOXA1inPBS
chr17	30533102	30533103	MACS_peak_6237	9.00	lobChIP_FOXA1inPBS
chr17	30629229	30629230	MACS_peak_6238	7.00	lobChIP_FOXA1inPBS
chr17	30654169	30654170	MACS_peak_6239	10.00	lobChIP_FOXA1inPBS
chr17	30750112	30750113	MACS_peak_6240	7.00	lobChIP_FOXA1inPBS
chr17	31154792	31154793	MACS_peak_6241	8.00	lobChIP_FOXA1inPBS
chr17	31239558	31239559	MACS_peak_6242	11.00	lobChIP_FOXA1inPBS
chr17	31254372	31254373	MACS_peak_6243	27.00	lobChIP_FOXA1inPBS
chr17	31611154	31611155	MACS_peak_6244	14.00	lobChIP_FOXA1inPBS
chr17	31841622	31841623	MACS_peak_6245	7.00	lobChIP_FOXA1inPBS
chr17	33378346	33378347	MACS_peak_6246	7.00	lobChIP_FOXA1inPBS
chr17	33390768	33390769	MACS_peak_6247	12.00	lobChIP_FOXA1inPBS
chr17	33400817	33400818	MACS_peak_6248	13.00	lobChIP_FOXA1inPBS
chr17	33406869	33406870	MACS_peak_6249	9.00	lobChIP_FOXA1inPBS
chr17	33414560	33414561	MACS_peak_6250	20.00	lobChIP_FOXA1inPBS
chr17	33581373	33581374	MACS_peak_6251	6.00	lobChIP_FOXA1inPBS
chr17	33954691	33954692	MACS_peak_6252	14.00	lobChIP_FOXA1inPBS
chr17	34170922	34170923	MACS_peak_6253	7.00	lobChIP_FOXA1inPBS
chr17	34300671	34300672	MACS_peak_6254	64.00	lobChIP_FOXA1inPBS
chr17	34307196	34307197	MACS_peak_6255	22.00	lobChIP_FOXA1inPBS
chr17	34334242	34334243	MACS_peak_6256	10.00	lobChIP_FOXA1inPBS
chr17	34899388	34899389	MACS_peak_6257	11.00	lobChIP_FOXA1inPBS
chr17	35078309	35078310	MACS_peak_6258	9.00	lobChIP_FOXA1inPBS
chr17	35504676	35504677	MACS_peak_6259	20.00	lobChIP_FOXA1inPBS
chr17	35596795	35596796	MACS_peak_6260	7.00	lobChIP_FOXA1inPBS
chr17	35798481	35798482	MACS_peak_6261	9.00	lobChIP_FOXA1inPBS
chr17	35848499	35848500	MACS_peak_6262	11.00	lobChIP_FOXA1inPBS
chr17	35862300	35862301	MACS_peak_6263	25.00	lobChIP_FOXA1inPBS
chr17	36010691	36010692	MACS_peak_6264	6.00	lobChIP_FOXA1inPBS
chr17	36036272	36036273	MACS_peak_6265	10.00	lobChIP_FOXA1inPBS
chr17	36043583	36043584	MACS_peak_6266	24.00	lobChIP_FOXA1inPBS
chr17	36049415	36049416	MACS_peak_6267	28.00	lobChIP_FOXA1inPBS
chr17	36068229	36068230	MACS_peak_6268	7.00	lobChIP_FOXA1inPBS
chr17	36070477	36070478	MACS_peak_6269	10.00	lobChIP_FOXA1inPBS
chr17	36077065	36077066	MACS_peak_6270	23.00	lobChIP_FOXA1inPBS
chr17	36190071	36190072	MACS_peak_6271	14.00	lobChIP_FOXA1inPBS
chr17	36770419	36770420	MACS_peak_6272	22.00	lobChIP_FOXA1inPBS
chr17	36777179	36777180	MACS_peak_6273	19.00	lobChIP_FOXA1inPBS
chr17	36907864	36907865	MACS_peak_6274	9.00	lobChIP_FOXA1inPBS
chr17	36996265	36996266	MACS_peak_6275	11.00	lobChIP_FOXA1inPBS
chr17	37029125	37029126	MACS_peak_6276	14.00	lobChIP_FOXA1inPBS
chr17	37094632	37094633	MACS_peak_6277	6.00	lobChIP_FOXA1inPBS
chr17	37139499	37139500	MACS_peak_6278	44.00	lobChIP_FOXA1inPBS
chr17	37264343	37264344	MACS_peak_6279	12.00	lobChIP_FOXA1inPBS
chr17	37309551	37309552	MACS_peak_6280	8.00	lobChIP_FOXA1inPBS
chr17	37413141	37413142	MACS_peak_6281	9.00	lobChIP_FOXA1inPBS
chr17	37437492	37437493	MACS_peak_6282	8.00	lobChIP_FOXA1inPBS
chr17	37609454	37609455	MACS_peak_6283	10.00	lobChIP_FOXA1inPBS
chr17	37610058	37610059	MACS_peak_6284	40.00	lobChIP_FOXA1inPBS
chr17	37617507	37617508	MACS_peak_6285	77.00	lobChIP_FOXA1inPBS
chr17	37852052	37852053	MACS_peak_6286	33.00	lobChIP_FOXA1inPBS
chr17	37859771	37859772	MACS_peak_6287	24.00	lobChIP_FOXA1inPBS
chr17	37893129	37893130	MACS_peak_6288	31.00	lobChIP_FOXA1inPBS
chr17	37895937	37895938	MACS_peak_6289	9.00	lobChIP_FOXA1inPBS
chr17	38070401	38070402	MACS_peak_6290	13.00	lobChIP_FOXA1inPBS
chr17	38080603	38080604	MACS_peak_6291	17.00	lobChIP_FOXA1inPBS
chr17	38083121	38083122	MACS_peak_6292	7.00	lobChIP_FOXA1inPBS
chr17	38090779	38090780	MACS_peak_6293	11.00	lobChIP_FOXA1inPBS
chr17	38117153	38117154	MACS_peak_6294	18.00	lobChIP_FOXA1inPBS
chr17	38171403	38171404	MACS_peak_6295	17.00	lobChIP_FOXA1inPBS
chr17	38257272	38257273	MACS_peak_6296	46.00	lobChIP_FOXA1inPBS
chr17	38259404	38259405	MACS_peak_6297	19.00	lobChIP_FOXA1inPBS
chr17	38270074	38270075	MACS_peak_6298	19.00	lobChIP_FOXA1inPBS
chr17	38271079	38271080	MACS_peak_6299	56.00	lobChIP_FOXA1inPBS
chr17	38341492	38341493	MACS_peak_6300	5.00	lobChIP_FOXA1inPBS
chr17	38347214	38347215	MACS_peak_6301	9.00	lobChIP_FOXA1inPBS
chr17	38358268	38358269	MACS_peak_6302	8.00	lobChIP_FOXA1inPBS
chr17	38442907	38442908	MACS_peak_6303	9.00	lobChIP_FOXA1inPBS
chr17	38482612	38482613	MACS_peak_6304	16.00	lobChIP_FOXA1inPBS
chr17	38491297	38491298	MACS_peak_6305	36.00	lobChIP_FOXA1inPBS
chr17	38509297	38509298	MACS_peak_6306	35.00	lobChIP_FOXA1inPBS
chr17	38555549	38555550	MACS_peak_6307	9.00	lobChIP_FOXA1inPBS
chr17	38576022	38576023	MACS_peak_6308	8.00	lobChIP_FOXA1inPBS
chr17	38605192	38605193	MACS_peak_6309	22.00	lobChIP_FOXA1inPBS
chr17	38606725	38606726	MACS_peak_6310	6.00	lobChIP_FOXA1inPBS
chr17	38637194	38637195	MACS_peak_6311	7.00	lobChIP_FOXA1inPBS
chr17	38642437	38642438	MACS_peak_6312	9.00	lobChIP_FOXA1inPBS
chr17	38673107	38673108	MACS_peak_6313	15.00	lobChIP_FOXA1inPBS
chr17	38689865	38689866	MACS_peak_6314	12.00	lobChIP_FOXA1inPBS
chr17	38705821	38705822	MACS_peak_6315	18.00	lobChIP_FOXA1inPBS
chr17	38708260	38708261	MACS_peak_6316	34.00	lobChIP_FOXA1inPBS
chr17	38960005	38960006	MACS_peak_6317	10.00	lobChIP_FOXA1inPBS
chr17	39058411	39058412	MACS_peak_6318	41.00	lobChIP_FOXA1inPBS
chr17	39060028	39060029	MACS_peak_6319	7.00	lobChIP_FOXA1inPBS
chr17	39066372	39066373	MACS_peak_6320	11.00	lobChIP_FOXA1inPBS
chr17	39071814	39071815	MACS_peak_6321	11.00	lobChIP_FOXA1inPBS
chr17	39105981	39105982	MACS_peak_6322	30.00	lobChIP_FOXA1inPBS
chr17	39113334	39113335	MACS_peak_6323	21.00	lobChIP_FOXA1inPBS
chr17	39114527	39114528	MACS_peak_6324	107.00	lobChIP_FOXA1inPBS
chr17	39165673	39165674	MACS_peak_6325	10.00	lobChIP_FOXA1inPBS
chr17	39173260	39173261	MACS_peak_6326	14.00	lobChIP_FOXA1inPBS
chr17	39215656	39215657	MACS_peak_6327	11.00	lobChIP_FOXA1inPBS
chr17	39569501	39569502	MACS_peak_6328	22.00	lobChIP_FOXA1inPBS
chr17	39662794	39662795	MACS_peak_6329	9.00	lobChIP_FOXA1inPBS
chr17	39695775	39695776	MACS_peak_6330	14.00	lobChIP_FOXA1inPBS
chr17	39844182	39844183	MACS_peak_6331	11.00	lobChIP_FOXA1inPBS
chr17	39846254	39846255	MACS_peak_6332	10.00	lobChIP_FOXA1inPBS
chr17	39933997	39933998	MACS_peak_6333	20.00	lobChIP_FOXA1inPBS
chr17	40070675	40070676	MACS_peak_6334	8.00	lobChIP_FOXA1inPBS
chr17	40086732	40086733	MACS_peak_6335	7.00	lobChIP_FOXA1inPBS
chr17	40141934	40141935	MACS_peak_6336	30.00	lobChIP_FOXA1inPBS
chr17	40155057	40155058	MACS_peak_6337	15.00	lobChIP_FOXA1inPBS
chr17	40240130	40240131	MACS_peak_6338	59.00	lobChIP_FOXA1inPBS
chr17	40268854	40268855	MACS_peak_6339	16.00	lobChIP_FOXA1inPBS
chr17	40421636	40421637	MACS_peak_6340	19.00	lobChIP_FOXA1inPBS
chr17	40478002	40478003	MACS_peak_6341	12.00	lobChIP_FOXA1inPBS
chr17	40580868	40580869	MACS_peak_6342	15.00	lobChIP_FOXA1inPBS
chr17	40593678	40593679	MACS_peak_6343	13.00	lobChIP_FOXA1inPBS
chr17	40620083	40620084	MACS_peak_6344	9.00	lobChIP_FOXA1inPBS
chr17	40636720	40636721	MACS_peak_6345	14.00	lobChIP_FOXA1inPBS
chr17	40680067	40680068	MACS_peak_6346	12.00	lobChIP_FOXA1inPBS
chr17	40683007	40683008	MACS_peak_6347	53.00	lobChIP_FOXA1inPBS
chr17	40730628	40730629	MACS_peak_6348	8.00	lobChIP_FOXA1inPBS
chr17	40819905	40819906	MACS_peak_6349	8.00	lobChIP_FOXA1inPBS
chr17	40896993	40896994	MACS_peak_6350	26.00	lobChIP_FOXA1inPBS
chr17	40932109	40932110	MACS_peak_6351	6.00	lobChIP_FOXA1inPBS
chr17	40942293	40942294	MACS_peak_6352	9.00	lobChIP_FOXA1inPBS
chr17	40944117	40944118	MACS_peak_6353	21.00	lobChIP_FOXA1inPBS
chr17	40954828	40954829	MACS_peak_6354	7.00	lobChIP_FOXA1inPBS
chr17	40957296	40957297	MACS_peak_6355	9.00	lobChIP_FOXA1inPBS
chr17	41046443	41046444	MACS_peak_6356	5.00	lobChIP_FOXA1inPBS
chr17	41071063	41071064	MACS_peak_6357	64.00	lobChIP_FOXA1inPBS
chr17	41120168	41120169	MACS_peak_6358	28.00	lobChIP_FOXA1inPBS
chr17	41150218	41150219	MACS_peak_6359	8.00	lobChIP_FOXA1inPBS
chr17	41156941	41156942	MACS_peak_6360	8.00	lobChIP_FOXA1inPBS
chr17	41162901	41162902	MACS_peak_6361	7.00	lobChIP_FOXA1inPBS
chr17	41192923	41192924	MACS_peak_6362	13.00	lobChIP_FOXA1inPBS
chr17	41221015	41221016	MACS_peak_6363	32.00	lobChIP_FOXA1inPBS
chr17	41266365	41266366	MACS_peak_6364	10.00	lobChIP_FOXA1inPBS
chr17	41304300	41304301	MACS_peak_6365	21.00	lobChIP_FOXA1inPBS
chr17	41321969	41321970	MACS_peak_6366	39.00	lobChIP_FOXA1inPBS
chr17	41324151	41324152	MACS_peak_6367	11.00	lobChIP_FOXA1inPBS
chr17	41327278	41327279	MACS_peak_6368	8.00	lobChIP_FOXA1inPBS
chr17	41363024	41363025	MACS_peak_6369	5.00	lobChIP_FOXA1inPBS
chr17	41410911	41410912	MACS_peak_6370	7.00	lobChIP_FOXA1inPBS
chr17	41438523	41438524	MACS_peak_6371	15.00	lobChIP_FOXA1inPBS
chr17	41487163	41487164	MACS_peak_6372	10.00	lobChIP_FOXA1inPBS
chr17	41522131	41522132	MACS_peak_6373	7.00	lobChIP_FOXA1inPBS
chr17	41527116	41527117	MACS_peak_6374	21.00	lobChIP_FOXA1inPBS
chr17	41534371	41534372	MACS_peak_6375	18.00	lobChIP_FOXA1inPBS
chr17	41535887	41535888	MACS_peak_6376	9.00	lobChIP_FOXA1inPBS
chr17	41538745	41538746	MACS_peak_6377	6.00	lobChIP_FOXA1inPBS
chr17	41553320	41553321	MACS_peak_6378	7.00	lobChIP_FOXA1inPBS
chr17	41557511	41557512	MACS_peak_6379	21.00	lobChIP_FOXA1inPBS
chr17	41561258	41561259	MACS_peak_6380	11.00	lobChIP_FOXA1inPBS
chr17	41605806	41605807	MACS_peak_6381	9.00	lobChIP_FOXA1inPBS
chr17	41608951	41608952	MACS_peak_6382	6.00	lobChIP_FOXA1inPBS
chr17	41622037	41622038	MACS_peak_6383	47.00	lobChIP_FOXA1inPBS
chr17	41636646	41636647	MACS_peak_6384	22.00	lobChIP_FOXA1inPBS
chr17	41651129	41651130	MACS_peak_6385	14.00	lobChIP_FOXA1inPBS
chr17	41656577	41656578	MACS_peak_6386	17.00	lobChIP_FOXA1inPBS
chr17	41662329	41662330	MACS_peak_6387	10.00	lobChIP_FOXA1inPBS
chr17	41745268	41745269	MACS_peak_6388	7.00	lobChIP_FOXA1inPBS
chr17	41849472	41849473	MACS_peak_6389	9.00	lobChIP_FOXA1inPBS
chr17	41850396	41850397	MACS_peak_6390	17.00	lobChIP_FOXA1inPBS
chr17	41879403	41879404	MACS_peak_6391	43.00	lobChIP_FOXA1inPBS
chr17	42042418	42042419	MACS_peak_6392	29.00	lobChIP_FOXA1inPBS
chr17	42061321	42061322	MACS_peak_6393	28.00	lobChIP_FOXA1inPBS
chr17	42154378	42154379	MACS_peak_6394	12.00	lobChIP_FOXA1inPBS
chr17	42164522	42164523	MACS_peak_6395	8.00	lobChIP_FOXA1inPBS
chr17	42186084	42186085	MACS_peak_6396	23.00	lobChIP_FOXA1inPBS
chr17	42204373	42204374	MACS_peak_6397	12.00	lobChIP_FOXA1inPBS
chr17	42216246	42216247	MACS_peak_6398	10.00	lobChIP_FOXA1inPBS
chr17	43139164	43139165	MACS_peak_6399	9.00	lobChIP_FOXA1inPBS
chr17	43179615	43179616	MACS_peak_6400	11.00	lobChIP_FOXA1inPBS
chr17	43225241	43225242	MACS_peak_6401	32.00	lobChIP_FOXA1inPBS
chr17	43235762	43235763	MACS_peak_6402	7.00	lobChIP_FOXA1inPBS
chr17	43248850	43248851	MACS_peak_6403	18.00	lobChIP_FOXA1inPBS
chr17	43449606	43449607	MACS_peak_6404	18.00	lobChIP_FOXA1inPBS
chr17	43539434	43539435	MACS_peak_6405	11.00	lobChIP_FOXA1inPBS
chr17	43980687	43980688	MACS_peak_6406	7.00	lobChIP_FOXA1inPBS
chr17	44013449	44013450	MACS_peak_6407	15.00	lobChIP_FOXA1inPBS
chr17	44065978	44065979	MACS_peak_6408	9.00	lobChIP_FOXA1inPBS
chr17	44177420	44177421	MACS_peak_6409	11.00	lobChIP_FOXA1inPBS
chr17	44185683	44185684	MACS_peak_6410	6.00	lobChIP_FOXA1inPBS
chr17	44245620	44245621	MACS_peak_6411	18.00	lobChIP_FOXA1inPBS
chr17	44267365	44267366	MACS_peak_6412	11.00	lobChIP_FOXA1inPBS
chr17	44308948	44308949	MACS_peak_6413	9.00	lobChIP_FOXA1inPBS
chr17	44364252	44364253	MACS_peak_6414	8.00	lobChIP_FOXA1inPBS
chr17	44836181	44836182	MACS_peak_6415	13.00	lobChIP_FOXA1inPBS
chr17	44849920	44849921	MACS_peak_6416	11.00	lobChIP_FOXA1inPBS
chr17	44910440	44910441	MACS_peak_6417	9.00	lobChIP_FOXA1inPBS
chr17	45368999	45369000	MACS_peak_6418	48.00	lobChIP_FOXA1inPBS
chr17	45406573	45406574	MACS_peak_6419	15.00	lobChIP_FOXA1inPBS
chr17	45407478	45407479	MACS_peak_6420	14.00	lobChIP_FOXA1inPBS
chr17	45505664	45505665	MACS_peak_6421	14.00	lobChIP_FOXA1inPBS
chr17	45698038	45698039	MACS_peak_6422	39.00	lobChIP_FOXA1inPBS
chr17	45908418	45908419	MACS_peak_6423	9.00	lobChIP_FOXA1inPBS
chr17	45919935	45919936	MACS_peak_6424	10.00	lobChIP_FOXA1inPBS
chr17	45982704	45982705	MACS_peak_6425	9.00	lobChIP_FOXA1inPBS
chr17	46086024	46086025	MACS_peak_6426	10.00	lobChIP_FOXA1inPBS
chr17	46240423	46240424	MACS_peak_6427	8.00	lobChIP_FOXA1inPBS
chr17	46873169	46873170	MACS_peak_6428	9.00	lobChIP_FOXA1inPBS
chr17	46949207	46949208	MACS_peak_6429	52.00	lobChIP_FOXA1inPBS
chr17	46968953	46968954	MACS_peak_6430	14.00	lobChIP_FOXA1inPBS
chr17	47038185	47038186	MACS_peak_6431	5.00	lobChIP_FOXA1inPBS
chr17	47048760	47048761	MACS_peak_6432	9.00	lobChIP_FOXA1inPBS
chr17	47049772	47049773	MACS_peak_6433	7.00	lobChIP_FOXA1inPBS
chr17	47077525	47077526	MACS_peak_6434	10.00	lobChIP_FOXA1inPBS
chr17	47082676	47082677	MACS_peak_6435	15.00	lobChIP_FOXA1inPBS
chr17	47145534	47145535	MACS_peak_6436	8.00	lobChIP_FOXA1inPBS
chr17	47146484	47146485	MACS_peak_6437	13.00	lobChIP_FOXA1inPBS
chr17	47184276	47184277	MACS_peak_6438	47.00	lobChIP_FOXA1inPBS
chr17	47223156	47223157	MACS_peak_6439	7.00	lobChIP_FOXA1inPBS
chr17	47485599	47485600	MACS_peak_6440	24.00	lobChIP_FOXA1inPBS
chr17	47674441	47674442	MACS_peak_6441	17.00	lobChIP_FOXA1inPBS
chr17	47812354	47812355	MACS_peak_6442	11.00	lobChIP_FOXA1inPBS
chr17	48354186	48354187	MACS_peak_6443	11.00	lobChIP_FOXA1inPBS
chr17	48500260	48500261	MACS_peak_6444	13.00	lobChIP_FOXA1inPBS
chr17	48501180	48501181	MACS_peak_6445	29.00	lobChIP_FOXA1inPBS
chr17	48506271	48506272	MACS_peak_6446	8.00	lobChIP_FOXA1inPBS
chr17	48517803	48517804	MACS_peak_6447	9.00	lobChIP_FOXA1inPBS
chr17	48620394	48620395	MACS_peak_6448	13.00	lobChIP_FOXA1inPBS
chr17	48762290	48762291	MACS_peak_6449	17.00	lobChIP_FOXA1inPBS
chr17	48770648	48770649	MACS_peak_6450	29.00	lobChIP_FOXA1inPBS
chr17	48771639	48771640	MACS_peak_6451	9.00	lobChIP_FOXA1inPBS
chr17	48772709	48772710	MACS_peak_6452	9.00	lobChIP_FOXA1inPBS
chr17	48778119	48778120	MACS_peak_6453	7.00	lobChIP_FOXA1inPBS
chr17	48785293	48785294	MACS_peak_6454	8.00	lobChIP_FOXA1inPBS
chr17	48793872	48793873	MACS_peak_6455	8.00	lobChIP_FOXA1inPBS
chr17	48840193	48840194	MACS_peak_6456	8.00	lobChIP_FOXA1inPBS
chr17	48883872	48883873	MACS_peak_6457	16.00	lobChIP_FOXA1inPBS
chr17	48935699	48935700	MACS_peak_6458	12.00	lobChIP_FOXA1inPBS
chr17	48968285	48968286	MACS_peak_6459	52.00	lobChIP_FOXA1inPBS
chr17	48976393	48976394	MACS_peak_6460	8.00	lobChIP_FOXA1inPBS
chr17	48982971	48982972	MACS_peak_6461	12.00	lobChIP_FOXA1inPBS
chr17	49022150	49022151	MACS_peak_6462	9.00	lobChIP_FOXA1inPBS
chr17	49119351	49119352	MACS_peak_6463	8.00	lobChIP_FOXA1inPBS
chr17	49123248	49123249	MACS_peak_6464	10.00	lobChIP_FOXA1inPBS
chr17	49190336	49190337	MACS_peak_6465	11.00	lobChIP_FOXA1inPBS
chr17	49195742	49195743	MACS_peak_6466	10.00	lobChIP_FOXA1inPBS
chr17	49203553	49203554	MACS_peak_6467	7.00	lobChIP_FOXA1inPBS
chr17	49223781	49223782	MACS_peak_6468	17.00	lobChIP_FOXA1inPBS
chr17	49399973	49399974	MACS_peak_6469	29.00	lobChIP_FOXA1inPBS
chr17	49513216	49513217	MACS_peak_6470	13.00	lobChIP_FOXA1inPBS
chr17	49685833	49685834	MACS_peak_6471	24.00	lobChIP_FOXA1inPBS
chr17	50075205	50075206	MACS_peak_6472	17.00	lobChIP_FOXA1inPBS
chr17	50496945	50496946	MACS_peak_6473	18.00	lobChIP_FOXA1inPBS
chr17	52002144	52002145	MACS_peak_6474	15.00	lobChIP_FOXA1inPBS
chr17	53344912	53344913	MACS_peak_6475	23.00	lobChIP_FOXA1inPBS
chr17	53361724	53361725	MACS_peak_6476	8.00	lobChIP_FOXA1inPBS
chr17	53363398	53363399	MACS_peak_6477	34.00	lobChIP_FOXA1inPBS
chr17	53364603	53364604	MACS_peak_6478	63.00	lobChIP_FOXA1inPBS
chr17	53379648	53379649	MACS_peak_6479	17.00	lobChIP_FOXA1inPBS
chr17	53383831	53383832	MACS_peak_6480	31.00	lobChIP_FOXA1inPBS
chr17	53472008	53472009	MACS_peak_6481	41.00	lobChIP_FOXA1inPBS
chr17	53485629	53485630	MACS_peak_6482	42.00	lobChIP_FOXA1inPBS
chr17	53500308	53500309	MACS_peak_6483	7.00	lobChIP_FOXA1inPBS
chr17	53510720	53510721	MACS_peak_6484	23.00	lobChIP_FOXA1inPBS
chr17	53524311	53524312	MACS_peak_6485	48.00	lobChIP_FOXA1inPBS
chr17	53528881	53528882	MACS_peak_6486	9.00	lobChIP_FOXA1inPBS
chr17	53532368	53532369	MACS_peak_6487	15.00	lobChIP_FOXA1inPBS
chr17	54849910	54849911	MACS_peak_6488	24.00	lobChIP_FOXA1inPBS
chr17	54964035	54964036	MACS_peak_6489	11.00	lobChIP_FOXA1inPBS
chr17	54992978	54992979	MACS_peak_6490	7.00	lobChIP_FOXA1inPBS
chr17	55004361	55004362	MACS_peak_6491	6.00	lobChIP_FOXA1inPBS
chr17	55163213	55163214	MACS_peak_6492	52.00	lobChIP_FOXA1inPBS
chr17	55198320	55198321	MACS_peak_6493	12.00	lobChIP_FOXA1inPBS
chr17	55298872	55298873	MACS_peak_6494	8.00	lobChIP_FOXA1inPBS
chr17	55362716	55362717	MACS_peak_6495	7.00	lobChIP_FOXA1inPBS
chr17	55422488	55422489	MACS_peak_6496	29.00	lobChIP_FOXA1inPBS
chr17	55444437	55444438	MACS_peak_6497	7.00	lobChIP_FOXA1inPBS
chr17	55449646	55449647	MACS_peak_6498	32.00	lobChIP_FOXA1inPBS
chr17	55484811	55484812	MACS_peak_6499	12.00	lobChIP_FOXA1inPBS
chr17	55487775	55487776	MACS_peak_6500	11.00	lobChIP_FOXA1inPBS
chr17	55491612	55491613	MACS_peak_6501	55.00	lobChIP_FOXA1inPBS
chr17	55522543	55522544	MACS_peak_6502	17.00	lobChIP_FOXA1inPBS
chr17	55536921	55536922	MACS_peak_6503	18.00	lobChIP_FOXA1inPBS
chr17	55546738	55546739	MACS_peak_6504	16.00	lobChIP_FOXA1inPBS
chr17	55593811	55593812	MACS_peak_6505	14.00	lobChIP_FOXA1inPBS
chr17	55595233	55595234	MACS_peak_6506	10.00	lobChIP_FOXA1inPBS
chr17	55626076	55626077	MACS_peak_6507	6.00	lobChIP_FOXA1inPBS
chr17	55638921	55638922	MACS_peak_6508	39.00	lobChIP_FOXA1inPBS
chr17	55910985	55910986	MACS_peak_6509	21.00	lobChIP_FOXA1inPBS
chr17	55916071	55916072	MACS_peak_6510	11.00	lobChIP_FOXA1inPBS
chr17	55928807	55928808	MACS_peak_6511	14.00	lobChIP_FOXA1inPBS
chr17	55929290	55929291	MACS_peak_6512	9.00	lobChIP_FOXA1inPBS
chr17	55932976	55932977	MACS_peak_6513	10.00	lobChIP_FOXA1inPBS
chr17	55938606	55938607	MACS_peak_6514	22.00	lobChIP_FOXA1inPBS
chr17	55956981	55956982	MACS_peak_6515	12.00	lobChIP_FOXA1inPBS
chr17	56015589	56015590	MACS_peak_6516	7.00	lobChIP_FOXA1inPBS
chr17	56024597	56024598	MACS_peak_6517	11.00	lobChIP_FOXA1inPBS
chr17	56038347	56038348	MACS_peak_6518	10.00	lobChIP_FOXA1inPBS
chr17	56052684	56052685	MACS_peak_6519	53.00	lobChIP_FOXA1inPBS
chr17	56064829	56064830	MACS_peak_6520	8.00	lobChIP_FOXA1inPBS
chr17	56162204	56162205	MACS_peak_6521	9.00	lobChIP_FOXA1inPBS
chr17	56429502	56429503	MACS_peak_6522	10.00	lobChIP_FOXA1inPBS
chr17	56442960	56442961	MACS_peak_6523	9.00	lobChIP_FOXA1inPBS
chr17	56468998	56468999	MACS_peak_6524	40.00	lobChIP_FOXA1inPBS
chr17	56490484	56490485	MACS_peak_6525	19.00	lobChIP_FOXA1inPBS
chr17	56491127	56491128	MACS_peak_6526	11.00	lobChIP_FOXA1inPBS
chr17	56493358	56493359	MACS_peak_6527	19.00	lobChIP_FOXA1inPBS
chr17	56513443	56513444	MACS_peak_6528	10.00	lobChIP_FOXA1inPBS
chr17	56709105	56709106	MACS_peak_6529	16.00	lobChIP_FOXA1inPBS
chr17	56736493	56736494	MACS_peak_6530	15.00	lobChIP_FOXA1inPBS
chr17	57140034	57140035	MACS_peak_6531	11.00	lobChIP_FOXA1inPBS
chr17	57232462	57232463	MACS_peak_6532	6.00	lobChIP_FOXA1inPBS
chr17	57233706	57233707	MACS_peak_6533	18.00	lobChIP_FOXA1inPBS
chr17	57271976	57271977	MACS_peak_6534	6.00	lobChIP_FOXA1inPBS
chr17	57367260	57367261	MACS_peak_6535	6.00	lobChIP_FOXA1inPBS
chr17	57374650	57374651	MACS_peak_6536	13.00	lobChIP_FOXA1inPBS
chr17	57428225	57428226	MACS_peak_6537	7.00	lobChIP_FOXA1inPBS
chr17	57444382	57444383	MACS_peak_6538	31.00	lobChIP_FOXA1inPBS
chr17	57479370	57479371	MACS_peak_6539	10.00	lobChIP_FOXA1inPBS
chr17	57507704	57507705	MACS_peak_6540	13.00	lobChIP_FOXA1inPBS
chr17	57542715	57542716	MACS_peak_6541	71.00	lobChIP_FOXA1inPBS
chr17	57547319	57547320	MACS_peak_6542	15.00	lobChIP_FOXA1inPBS
chr17	57550864	57550865	MACS_peak_6543	14.00	lobChIP_FOXA1inPBS
chr17	57589306	57589307	MACS_peak_6544	11.00	lobChIP_FOXA1inPBS
chr17	57591794	57591795	MACS_peak_6545	20.00	lobChIP_FOXA1inPBS
chr17	57803364	57803365	MACS_peak_6546	16.00	lobChIP_FOXA1inPBS
chr17	57805682	57805683	MACS_peak_6547	9.00	lobChIP_FOXA1inPBS
chr17	57832122	57832123	MACS_peak_6548	10.00	lobChIP_FOXA1inPBS
chr17	57833649	57833650	MACS_peak_6549	21.00	lobChIP_FOXA1inPBS
chr17	57851579	57851580	MACS_peak_6550	8.00	lobChIP_FOXA1inPBS
chr17	57862502	57862503	MACS_peak_6551	14.00	lobChIP_FOXA1inPBS
chr17	57864132	57864133	MACS_peak_6552	11.00	lobChIP_FOXA1inPBS
chr17	57866974	57866975	MACS_peak_6553	7.00	lobChIP_FOXA1inPBS
chr17	57880964	57880965	MACS_peak_6554	24.00	lobChIP_FOXA1inPBS
chr17	57910455	57910456	MACS_peak_6555	8.00	lobChIP_FOXA1inPBS
chr17	57914925	57914926	MACS_peak_6556	6.00	lobChIP_FOXA1inPBS
chr17	57921467	57921468	MACS_peak_6557	9.00	lobChIP_FOXA1inPBS
chr17	57922870	57922871	MACS_peak_6558	17.00	lobChIP_FOXA1inPBS
chr17	57923946	57923947	MACS_peak_6559	31.00	lobChIP_FOXA1inPBS
chr17	57930726	57930727	MACS_peak_6560	47.00	lobChIP_FOXA1inPBS
chr17	57933194	57933195	MACS_peak_6561	45.00	lobChIP_FOXA1inPBS
chr17	57971880	57971881	MACS_peak_6562	21.00	lobChIP_FOXA1inPBS
chr17	58220933	58220934	MACS_peak_6563	11.00	lobChIP_FOXA1inPBS
chr17	58249285	58249286	MACS_peak_6564	11.00	lobChIP_FOXA1inPBS
chr17	58422796	58422797	MACS_peak_6565	9.00	lobChIP_FOXA1inPBS
chr17	58433500	58433501	MACS_peak_6566	29.00	lobChIP_FOXA1inPBS
chr17	58434459	58434460	MACS_peak_6567	12.00	lobChIP_FOXA1inPBS
chr17	58464019	58464020	MACS_peak_6568	14.00	lobChIP_FOXA1inPBS
chr17	58667582	58667583	MACS_peak_6569	11.00	lobChIP_FOXA1inPBS
chr17	58683825	58683826	MACS_peak_6570	6.00	lobChIP_FOXA1inPBS
chr17	58718147	58718148	MACS_peak_6571	13.00	lobChIP_FOXA1inPBS
chr17	58799666	58799667	MACS_peak_6572	9.00	lobChIP_FOXA1inPBS
chr17	58807415	58807416	MACS_peak_6573	8.00	lobChIP_FOXA1inPBS
chr17	58965137	58965138	MACS_peak_6574	13.00	lobChIP_FOXA1inPBS
chr17	58997600	58997601	MACS_peak_6575	18.00	lobChIP_FOXA1inPBS
chr17	59091508	59091509	MACS_peak_6576	9.00	lobChIP_FOXA1inPBS
chr17	59119802	59119803	MACS_peak_6577	10.00	lobChIP_FOXA1inPBS
chr17	59168692	59168693	MACS_peak_6578	11.00	lobChIP_FOXA1inPBS
chr17	59183888	59183889	MACS_peak_6579	11.00	lobChIP_FOXA1inPBS
chr17	59313328	59313329	MACS_peak_6580	11.00	lobChIP_FOXA1inPBS
chr17	59413564	59413565	MACS_peak_6581	100.00	lobChIP_FOXA1inPBS
chr17	59434185	59434186	MACS_peak_6582	64.00	lobChIP_FOXA1inPBS
chr17	59441065	59441066	MACS_peak_6583	53.00	lobChIP_FOXA1inPBS
chr17	59442994	59442995	MACS_peak_6584	9.00	lobChIP_FOXA1inPBS
chr17	59444377	59444378	MACS_peak_6585	9.00	lobChIP_FOXA1inPBS
chr17	59506986	59506987	MACS_peak_6586	8.00	lobChIP_FOXA1inPBS
chr17	59532539	59532540	MACS_peak_6587	50.00	lobChIP_FOXA1inPBS
chr17	59538359	59538360	MACS_peak_6588	29.00	lobChIP_FOXA1inPBS
chr17	59539005	59539006	MACS_peak_6589	14.00	lobChIP_FOXA1inPBS
chr17	59566836	59566837	MACS_peak_6590	20.00	lobChIP_FOXA1inPBS
chr17	59572132	59572133	MACS_peak_6591	9.00	lobChIP_FOXA1inPBS
chr17	59574632	59574633	MACS_peak_6592	15.00	lobChIP_FOXA1inPBS
chr17	59646113	59646114	MACS_peak_6593	50.00	lobChIP_FOXA1inPBS
chr17	60227459	60227460	MACS_peak_6594	7.00	lobChIP_FOXA1inPBS
chr17	60238334	60238335	MACS_peak_6595	7.00	lobChIP_FOXA1inPBS
chr17	60241374	60241375	MACS_peak_6596	10.00	lobChIP_FOXA1inPBS
chr17	60282574	60282575	MACS_peak_6597	8.00	lobChIP_FOXA1inPBS
chr17	60471196	60471197	MACS_peak_6598	9.00	lobChIP_FOXA1inPBS
chr17	60615834	60615835	MACS_peak_6599	10.00	lobChIP_FOXA1inPBS
chr17	61509881	61509882	MACS_peak_6600	10.00	lobChIP_FOXA1inPBS
chr17	61595837	61595838	MACS_peak_6601	5.00	lobChIP_FOXA1inPBS
chr17	61598079	61598080	MACS_peak_6602	14.00	lobChIP_FOXA1inPBS
chr17	61655851	61655852	MACS_peak_6603	37.00	lobChIP_FOXA1inPBS
chr17	62152279	62152280	MACS_peak_6604	29.00	lobChIP_FOXA1inPBS
chr17	62159641	62159642	MACS_peak_6605	30.00	lobChIP_FOXA1inPBS
chr17	62162071	62162072	MACS_peak_6606	29.00	lobChIP_FOXA1inPBS
chr17	62170484	62170485	MACS_peak_6607	7.00	lobChIP_FOXA1inPBS
chr17	62182978	62182979	MACS_peak_6608	10.00	lobChIP_FOXA1inPBS
chr17	62185315	62185316	MACS_peak_6609	9.00	lobChIP_FOXA1inPBS
chr17	62211954	62211955	MACS_peak_6610	14.00	lobChIP_FOXA1inPBS
chr17	62224796	62224797	MACS_peak_6611	13.00	lobChIP_FOXA1inPBS
chr17	62227837	62227838	MACS_peak_6612	21.00	lobChIP_FOXA1inPBS
chr17	62255684	62255685	MACS_peak_6613	17.00	lobChIP_FOXA1inPBS
chr17	62264539	62264540	MACS_peak_6614	8.00	lobChIP_FOXA1inPBS
chr17	62294530	62294531	MACS_peak_6615	10.00	lobChIP_FOXA1inPBS
chr17	62333308	62333309	MACS_peak_6616	11.00	lobChIP_FOXA1inPBS
chr17	62359450	62359451	MACS_peak_6617	24.00	lobChIP_FOXA1inPBS
chr17	62396213	62396214	MACS_peak_6618	18.00	lobChIP_FOXA1inPBS
chr17	62398114	62398115	MACS_peak_6619	6.00	lobChIP_FOXA1inPBS
chr17	62615707	62615708	MACS_peak_6620	9.00	lobChIP_FOXA1inPBS
chr17	62804827	62804828	MACS_peak_6621	11.00	lobChIP_FOXA1inPBS
chr17	62955525	62955526	MACS_peak_6622	27.00	lobChIP_FOXA1inPBS
chr17	62965256	62965257	MACS_peak_6623	7.00	lobChIP_FOXA1inPBS
chr17	62978516	62978517	MACS_peak_6624	8.00	lobChIP_FOXA1inPBS
chr17	63060977	63060978	MACS_peak_6625	13.00	lobChIP_FOXA1inPBS
chr17	63071073	63071074	MACS_peak_6626	23.00	lobChIP_FOXA1inPBS
chr17	63512242	63512243	MACS_peak_6627	8.00	lobChIP_FOXA1inPBS
chr17	63538802	63538803	MACS_peak_6628	6.00	lobChIP_FOXA1inPBS
chr17	63553740	63553741	MACS_peak_6629	25.00	lobChIP_FOXA1inPBS
chr17	63554934	63554935	MACS_peak_6630	13.00	lobChIP_FOXA1inPBS
chr17	63558248	63558249	MACS_peak_6631	5.00	lobChIP_FOXA1inPBS
chr17	63581578	63581579	MACS_peak_6632	20.00	lobChIP_FOXA1inPBS
chr17	63630325	63630326	MACS_peak_6633	23.00	lobChIP_FOXA1inPBS
chr17	63653199	63653200	MACS_peak_6634	17.00	lobChIP_FOXA1inPBS
chr17	63667283	63667284	MACS_peak_6635	15.00	lobChIP_FOXA1inPBS
chr17	63686803	63686804	MACS_peak_6636	9.00	lobChIP_FOXA1inPBS
chr17	63695367	63695368	MACS_peak_6637	14.00	lobChIP_FOXA1inPBS
chr17	63719914	63719915	MACS_peak_6638	11.00	lobChIP_FOXA1inPBS
chr17	63781429	63781430	MACS_peak_6639	30.00	lobChIP_FOXA1inPBS
chr17	63893195	63893196	MACS_peak_6640	14.00	lobChIP_FOXA1inPBS
chr17	64197031	64197032	MACS_peak_6641	11.00	lobChIP_FOXA1inPBS
chr17	64225600	64225601	MACS_peak_6642	12.00	lobChIP_FOXA1inPBS
chr17	64231682	64231683	MACS_peak_6643	69.00	lobChIP_FOXA1inPBS
chr17	64336584	64336585	MACS_peak_6644	8.00	lobChIP_FOXA1inPBS
chr17	64337957	64337958	MACS_peak_6645	6.00	lobChIP_FOXA1inPBS
chr17	64340052	64340053	MACS_peak_6646	7.00	lobChIP_FOXA1inPBS
chr17	64344585	64344586	MACS_peak_6647	6.00	lobChIP_FOXA1inPBS
chr17	64364342	64364343	MACS_peak_6648	9.00	lobChIP_FOXA1inPBS
chr17	64383209	64383210	MACS_peak_6649	7.00	lobChIP_FOXA1inPBS
chr17	64411712	64411713	MACS_peak_6650	16.00	lobChIP_FOXA1inPBS
chr17	64447735	64447736	MACS_peak_6651	12.00	lobChIP_FOXA1inPBS
chr17	64456242	64456243	MACS_peak_6652	10.00	lobChIP_FOXA1inPBS
chr17	64459382	64459383	MACS_peak_6653	6.00	lobChIP_FOXA1inPBS
chr17	64465468	64465469	MACS_peak_6654	16.00	lobChIP_FOXA1inPBS
chr17	64502975	64502976	MACS_peak_6655	10.00	lobChIP_FOXA1inPBS
chr17	64543883	64543884	MACS_peak_6656	13.00	lobChIP_FOXA1inPBS
chr17	64708183	64708184	MACS_peak_6657	17.00	lobChIP_FOXA1inPBS
chr17	64773533	64773534	MACS_peak_6658	15.00	lobChIP_FOXA1inPBS
chr17	64920091	64920092	MACS_peak_6659	12.00	lobChIP_FOXA1inPBS
chr17	65241933	65241934	MACS_peak_6660	21.00	lobChIP_FOXA1inPBS
chr17	65243329	65243330	MACS_peak_6661	13.00	lobChIP_FOXA1inPBS
chr17	65275574	65275575	MACS_peak_6662	7.00	lobChIP_FOXA1inPBS
chr17	65387085	65387086	MACS_peak_6663	9.00	lobChIP_FOXA1inPBS
chr17	65635973	65635974	MACS_peak_6664	17.00	lobChIP_FOXA1inPBS
chr17	65959551	65959552	MACS_peak_6665	9.00	lobChIP_FOXA1inPBS
chr17	65990801	65990802	MACS_peak_6666	29.00	lobChIP_FOXA1inPBS
chr17	66025857	66025858	MACS_peak_6667	15.00	lobChIP_FOXA1inPBS
chr17	66030775	66030776	MACS_peak_6668	7.00	lobChIP_FOXA1inPBS
chr17	66031384	66031385	MACS_peak_6669	10.00	lobChIP_FOXA1inPBS
chr17	66262206	66262207	MACS_peak_6670	44.00	lobChIP_FOXA1inPBS
chr17	66289121	66289122	MACS_peak_6671	6.00	lobChIP_FOXA1inPBS
chr17	66308796	66308797	MACS_peak_6672	6.00	lobChIP_FOXA1inPBS
chr17	66314818	66314819	MACS_peak_6673	11.00	lobChIP_FOXA1inPBS
chr17	66374922	66374923	MACS_peak_6674	23.00	lobChIP_FOXA1inPBS
chr17	66376019	66376020	MACS_peak_6675	7.00	lobChIP_FOXA1inPBS
chr17	66379654	66379655	MACS_peak_6676	12.00	lobChIP_FOXA1inPBS
chr17	66382679	66382680	MACS_peak_6677	8.00	lobChIP_FOXA1inPBS
chr17	66395613	66395614	MACS_peak_6678	5.00	lobChIP_FOXA1inPBS
chr17	66401159	66401160	MACS_peak_6679	8.00	lobChIP_FOXA1inPBS
chr17	66448337	66448338	MACS_peak_6680	17.00	lobChIP_FOXA1inPBS
chr17	66478818	66478819	MACS_peak_6681	22.00	lobChIP_FOXA1inPBS
chr17	66594832	66594833	MACS_peak_6682	9.00	lobChIP_FOXA1inPBS
chr17	66755303	66755304	MACS_peak_6683	16.00	lobChIP_FOXA1inPBS
chr17	66774115	66774116	MACS_peak_6684	11.00	lobChIP_FOXA1inPBS
chr17	66785516	66785517	MACS_peak_6685	39.00	lobChIP_FOXA1inPBS
chr17	66790230	66790231	MACS_peak_6686	77.00	lobChIP_FOXA1inPBS
chr17	66816991	66816992	MACS_peak_6687	52.00	lobChIP_FOXA1inPBS
chr17	67043848	67043849	MACS_peak_6688	14.00	lobChIP_FOXA1inPBS
chr17	67067109	67067110	MACS_peak_6689	13.00	lobChIP_FOXA1inPBS
chr17	67069032	67069033	MACS_peak_6690	30.00	lobChIP_FOXA1inPBS
chr17	67099909	67099910	MACS_peak_6691	16.00	lobChIP_FOXA1inPBS
chr17	67118405	67118406	MACS_peak_6692	14.00	lobChIP_FOXA1inPBS
chr17	67139605	67139606	MACS_peak_6693	8.00	lobChIP_FOXA1inPBS
chr17	67172025	67172026	MACS_peak_6694	10.00	lobChIP_FOXA1inPBS
chr17	67265007	67265008	MACS_peak_6695	12.00	lobChIP_FOXA1inPBS
chr17	67310784	67310785	MACS_peak_6696	19.00	lobChIP_FOXA1inPBS
chr17	67387454	67387455	MACS_peak_6697	12.00	lobChIP_FOXA1inPBS
chr17	67603451	67603452	MACS_peak_6698	8.00	lobChIP_FOXA1inPBS
chr17	69678154	69678155	MACS_peak_6699	38.00	lobChIP_FOXA1inPBS
chr17	70067568	70067569	MACS_peak_6700	8.00	lobChIP_FOXA1inPBS
chr17	70089081	70089082	MACS_peak_6701	17.00	lobChIP_FOXA1inPBS
chr17	70119484	70119485	MACS_peak_6702	9.00	lobChIP_FOXA1inPBS
chr17	70148034	70148035	MACS_peak_6703	12.00	lobChIP_FOXA1inPBS
chr17	70169833	70169834	MACS_peak_6704	12.00	lobChIP_FOXA1inPBS
chr17	70359088	70359089	MACS_peak_6705	8.00	lobChIP_FOXA1inPBS
chr17	70383115	70383116	MACS_peak_6706	18.00	lobChIP_FOXA1inPBS
chr17	70395993	70395994	MACS_peak_6707	74.00	lobChIP_FOXA1inPBS
chr17	70400185	70400186	MACS_peak_6708	15.00	lobChIP_FOXA1inPBS
chr17	70409476	70409477	MACS_peak_6709	36.00	lobChIP_FOXA1inPBS
chr17	70443620	70443621	MACS_peak_6710	19.00	lobChIP_FOXA1inPBS
chr17	70498161	70498162	MACS_peak_6711	45.00	lobChIP_FOXA1inPBS
chr17	70515163	70515164	MACS_peak_6712	79.00	lobChIP_FOXA1inPBS
chr17	70565499	70565500	MACS_peak_6713	10.00	lobChIP_FOXA1inPBS
chr17	70571856	70571857	MACS_peak_6714	14.00	lobChIP_FOXA1inPBS
chr17	70576820	70576821	MACS_peak_6715	18.00	lobChIP_FOXA1inPBS
chr17	70616340	70616341	MACS_peak_6716	11.00	lobChIP_FOXA1inPBS
chr17	70628582	70628583	MACS_peak_6717	14.00	lobChIP_FOXA1inPBS
chr17	70676992	70676993	MACS_peak_6718	7.00	lobChIP_FOXA1inPBS
chr17	70678937	70678938	MACS_peak_6719	14.00	lobChIP_FOXA1inPBS
chr17	70680172	70680173	MACS_peak_6720	11.00	lobChIP_FOXA1inPBS
chr17	70694218	70694219	MACS_peak_6721	9.00	lobChIP_FOXA1inPBS
chr17	70698680	70698681	MACS_peak_6722	11.00	lobChIP_FOXA1inPBS
chr17	70712477	70712478	MACS_peak_6723	16.00	lobChIP_FOXA1inPBS
chr17	70730121	70730122	MACS_peak_6724	8.00	lobChIP_FOXA1inPBS
chr17	70925022	70925023	MACS_peak_6725	8.00	lobChIP_FOXA1inPBS
chr17	70958162	70958163	MACS_peak_6726	7.00	lobChIP_FOXA1inPBS
chr17	70968654	70968655	MACS_peak_6727	44.00	lobChIP_FOXA1inPBS
chr17	71006847	71006848	MACS_peak_6728	10.00	lobChIP_FOXA1inPBS
chr17	71086710	71086711	MACS_peak_6729	11.00	lobChIP_FOXA1inPBS
chr17	71148334	71148335	MACS_peak_6730	18.00	lobChIP_FOXA1inPBS
chr17	71183590	71183591	MACS_peak_6731	136.00	lobChIP_FOXA1inPBS
chr17	71197505	71197506	MACS_peak_6732	17.00	lobChIP_FOXA1inPBS
chr17	71201954	71201955	MACS_peak_6733	13.00	lobChIP_FOXA1inPBS
chr17	71258200	71258201	MACS_peak_6734	24.00	lobChIP_FOXA1inPBS
chr17	71269147	71269148	MACS_peak_6735	18.00	lobChIP_FOXA1inPBS
chr17	71285266	71285267	MACS_peak_6736	17.00	lobChIP_FOXA1inPBS
chr17	71309888	71309889	MACS_peak_6737	10.00	lobChIP_FOXA1inPBS
chr17	71423569	71423570	MACS_peak_6738	15.00	lobChIP_FOXA1inPBS
chr17	71792092	71792093	MACS_peak_6739	21.00	lobChIP_FOXA1inPBS
chr17	71794404	71794405	MACS_peak_6740	44.00	lobChIP_FOXA1inPBS
chr17	71933230	71933231	MACS_peak_6741	12.00	lobChIP_FOXA1inPBS
chr17	72426992	72426993	MACS_peak_6742	7.00	lobChIP_FOXA1inPBS
chr17	72711400	72711401	MACS_peak_6743	15.00	lobChIP_FOXA1inPBS
chr17	72734719	72734720	MACS_peak_6744	27.00	lobChIP_FOXA1inPBS
chr17	72759261	72759262	MACS_peak_6745	16.00	lobChIP_FOXA1inPBS
chr17	72873408	72873409	MACS_peak_6746	12.00	lobChIP_FOXA1inPBS
chr17	72886030	72886031	MACS_peak_6747	11.00	lobChIP_FOXA1inPBS
chr17	72968257	72968258	MACS_peak_6748	11.00	lobChIP_FOXA1inPBS
chr17	73031517	73031518	MACS_peak_6749	6.00	lobChIP_FOXA1inPBS
chr17	73033007	73033008	MACS_peak_6750	8.00	lobChIP_FOXA1inPBS
chr17	73080371	73080372	MACS_peak_6751	14.00	lobChIP_FOXA1inPBS
chr17	73092454	73092455	MACS_peak_6752	10.00	lobChIP_FOXA1inPBS
chr17	73155439	73155440	MACS_peak_6753	16.00	lobChIP_FOXA1inPBS
chr17	73196171	73196172	MACS_peak_6754	16.00	lobChIP_FOXA1inPBS
chr17	73263993	73263994	MACS_peak_6755	12.00	lobChIP_FOXA1inPBS
chr17	73290519	73290520	MACS_peak_6756	16.00	lobChIP_FOXA1inPBS
chr17	73346358	73346359	MACS_peak_6757	12.00	lobChIP_FOXA1inPBS
chr17	73379245	73379246	MACS_peak_6758	80.00	lobChIP_FOXA1inPBS
chr17	73402186	73402187	MACS_peak_6759	8.00	lobChIP_FOXA1inPBS
chr17	73451905	73451906	MACS_peak_6760	7.00	lobChIP_FOXA1inPBS
chr17	73455749	73455750	MACS_peak_6761	13.00	lobChIP_FOXA1inPBS
chr17	73508052	73508053	MACS_peak_6762	14.00	lobChIP_FOXA1inPBS
chr17	73521707	73521708	MACS_peak_6763	12.00	lobChIP_FOXA1inPBS
chr17	73843322	73843323	MACS_peak_6764	8.00	lobChIP_FOXA1inPBS
chr17	73865482	73865483	MACS_peak_6765	31.00	lobChIP_FOXA1inPBS
chr17	73937524	73937525	MACS_peak_6766	8.00	lobChIP_FOXA1inPBS
chr17	73956042	73956043	MACS_peak_6767	7.00	lobChIP_FOXA1inPBS
chr17	73972447	73972448	MACS_peak_6768	11.00	lobChIP_FOXA1inPBS
chr17	73999632	73999633	MACS_peak_6769	17.00	lobChIP_FOXA1inPBS
chr17	74102320	74102321	MACS_peak_6770	22.00	lobChIP_FOXA1inPBS
chr17	74128871	74128872	MACS_peak_6771	7.00	lobChIP_FOXA1inPBS
chr17	74134724	74134725	MACS_peak_6772	12.00	lobChIP_FOXA1inPBS
chr17	74170356	74170357	MACS_peak_6773	30.00	lobChIP_FOXA1inPBS
chr17	74234159	74234160	MACS_peak_6774	15.00	lobChIP_FOXA1inPBS
chr17	74248283	74248284	MACS_peak_6775	12.00	lobChIP_FOXA1inPBS
chr17	74254624	74254625	MACS_peak_6776	6.00	lobChIP_FOXA1inPBS
chr17	74262152	74262153	MACS_peak_6777	16.00	lobChIP_FOXA1inPBS
chr17	74313068	74313069	MACS_peak_6778	7.00	lobChIP_FOXA1inPBS
chr17	74347933	74347934	MACS_peak_6779	18.00	lobChIP_FOXA1inPBS
chr17	74353318	74353319	MACS_peak_6780	24.00	lobChIP_FOXA1inPBS
chr17	74361886	74361887	MACS_peak_6781	8.00	lobChIP_FOXA1inPBS
chr17	74363905	74363906	MACS_peak_6782	20.00	lobChIP_FOXA1inPBS
chr17	74372593	74372594	MACS_peak_6783	18.00	lobChIP_FOXA1inPBS
chr17	74479776	74479777	MACS_peak_6784	16.00	lobChIP_FOXA1inPBS
chr17	74495517	74495518	MACS_peak_6785	28.00	lobChIP_FOXA1inPBS
chr17	74521030	74521031	MACS_peak_6786	7.00	lobChIP_FOXA1inPBS
chr17	74529275	74529276	MACS_peak_6787	26.00	lobChIP_FOXA1inPBS
chr17	74547347	74547348	MACS_peak_6788	13.00	lobChIP_FOXA1inPBS
chr17	74592732	74592733	MACS_peak_6789	16.00	lobChIP_FOXA1inPBS
chr17	74637890	74637891	MACS_peak_6790	15.00	lobChIP_FOXA1inPBS
chr17	74668593	74668594	MACS_peak_6791	11.00	lobChIP_FOXA1inPBS
chr17	74758896	74758897	MACS_peak_6792	118.00	lobChIP_FOXA1inPBS
chr17	74911222	74911223	MACS_peak_6793	105.00	lobChIP_FOXA1inPBS
chr17	75098060	75098061	MACS_peak_6794	76.00	lobChIP_FOXA1inPBS
chr17	75115908	75115909	MACS_peak_6795	8.00	lobChIP_FOXA1inPBS
chr17	75118084	75118085	MACS_peak_6796	68.00	lobChIP_FOXA1inPBS
chr17	75143514	75143515	MACS_peak_6797	7.00	lobChIP_FOXA1inPBS
chr17	75156665	75156666	MACS_peak_6798	36.00	lobChIP_FOXA1inPBS
chr17	75279358	75279359	MACS_peak_6799	11.00	lobChIP_FOXA1inPBS
chr17	75282812	75282813	MACS_peak_6800	31.00	lobChIP_FOXA1inPBS
chr17	75296624	75296625	MACS_peak_6801	12.00	lobChIP_FOXA1inPBS
chr17	75323873	75323874	MACS_peak_6802	7.00	lobChIP_FOXA1inPBS
chr17	75373551	75373552	MACS_peak_6803	24.00	lobChIP_FOXA1inPBS
chr17	75426528	75426529	MACS_peak_6804	22.00	lobChIP_FOXA1inPBS
chr17	75448933	75448934	MACS_peak_6805	5.00	lobChIP_FOXA1inPBS
chr17	75508172	75508173	MACS_peak_6806	29.00	lobChIP_FOXA1inPBS
chr17	75930992	75930993	MACS_peak_6807	5.00	lobChIP_FOXA1inPBS
chr17	75931605	75931606	MACS_peak_6808	12.00	lobChIP_FOXA1inPBS
chr17	76101673	76101674	MACS_peak_6809	10.00	lobChIP_FOXA1inPBS
chr17	76184208	76184209	MACS_peak_6810	50.00	lobChIP_FOXA1inPBS
chr17	76395970	76395971	MACS_peak_6811	15.00	lobChIP_FOXA1inPBS
chr17	76417779	76417780	MACS_peak_6812	96.00	lobChIP_FOXA1inPBS
chr17	76646506	76646507	MACS_peak_6813	69.00	lobChIP_FOXA1inPBS
chr17	76662242	76662243	MACS_peak_6814	12.00	lobChIP_FOXA1inPBS
chr17	76714321	76714322	MACS_peak_6815	10.00	lobChIP_FOXA1inPBS
chr17	76731336	76731337	MACS_peak_6816	11.00	lobChIP_FOXA1inPBS
chr17	76732074	76732075	MACS_peak_6817	8.00	lobChIP_FOXA1inPBS
chr17	76733415	76733416	MACS_peak_6818	7.00	lobChIP_FOXA1inPBS
chr17	76744482	76744483	MACS_peak_6819	23.00	lobChIP_FOXA1inPBS
chr17	76747680	76747681	MACS_peak_6820	12.00	lobChIP_FOXA1inPBS
chr17	76828273	76828274	MACS_peak_6821	17.00	lobChIP_FOXA1inPBS
chr17	76861908	76861909	MACS_peak_6822	8.00	lobChIP_FOXA1inPBS
chr17	77023030	77023031	MACS_peak_6823	9.00	lobChIP_FOXA1inPBS
chr17	77063408	77063409	MACS_peak_6824	12.00	lobChIP_FOXA1inPBS
chr17	77706998	77706999	MACS_peak_6825	11.00	lobChIP_FOXA1inPBS
chr17	77724826	77724827	MACS_peak_6826	6.00	lobChIP_FOXA1inPBS
chr17	77795278	77795279	MACS_peak_6827	36.00	lobChIP_FOXA1inPBS
chr17	77803548	77803549	MACS_peak_6828	8.00	lobChIP_FOXA1inPBS
chr17	77810101	77810102	MACS_peak_6829	16.00	lobChIP_FOXA1inPBS
chr17	77879911	77879912	MACS_peak_6830	10.00	lobChIP_FOXA1inPBS
chr17	77902305	77902306	MACS_peak_6831	5.00	lobChIP_FOXA1inPBS
chr17	77960765	77960766	MACS_peak_6832	9.00	lobChIP_FOXA1inPBS
chr17	77961124	77961125	MACS_peak_6833	5.00	lobChIP_FOXA1inPBS
chr17	77962103	77962104	MACS_peak_6834	27.00	lobChIP_FOXA1inPBS
chr17	77967300	77967301	MACS_peak_6835	10.00	lobChIP_FOXA1inPBS
chr17	77998381	77998382	MACS_peak_6836	11.00	lobChIP_FOXA1inPBS
chr17	78042643	78042644	MACS_peak_6837	8.00	lobChIP_FOXA1inPBS
chr17	78135222	78135223	MACS_peak_6838	10.00	lobChIP_FOXA1inPBS
chr17	78235354	78235355	MACS_peak_6839	70.00	lobChIP_FOXA1inPBS
chr17	78603490	78603491	MACS_peak_6840	17.00	lobChIP_FOXA1inPBS
chr17	78606259	78606260	MACS_peak_6841	17.00	lobChIP_FOXA1inPBS
chr17	78641449	78641450	MACS_peak_6842	32.00	lobChIP_FOXA1inPBS
chr17	78646149	78646150	MACS_peak_6843	39.00	lobChIP_FOXA1inPBS
chr17	78647458	78647459	MACS_peak_6844	13.00	lobChIP_FOXA1inPBS
chr17	78648001	78648002	MACS_peak_6845	10.00	lobChIP_FOXA1inPBS
chr17	78680384	78680385	MACS_peak_6846	8.00	lobChIP_FOXA1inPBS
chr17	79272879	79272880	MACS_peak_6847	19.00	lobChIP_FOXA1inPBS
chr17	79313953	79313954	MACS_peak_6848	18.00	lobChIP_FOXA1inPBS
chr17	79367925	79367926	MACS_peak_6849	58.00	lobChIP_FOXA1inPBS
chr17	79371776	79371777	MACS_peak_6850	10.00	lobChIP_FOXA1inPBS
chr17	79543785	79543786	MACS_peak_6851	17.00	lobChIP_FOXA1inPBS
chr17	79551212	79551213	MACS_peak_6852	15.00	lobChIP_FOXA1inPBS
chr17	79694811	79694812	MACS_peak_6853	31.00	lobChIP_FOXA1inPBS
chr17	79774864	79774865	MACS_peak_6854	13.00	lobChIP_FOXA1inPBS
chr17	79952974	79952975	MACS_peak_6855	15.00	lobChIP_FOXA1inPBS
chr17	80189878	80189879	MACS_peak_6856	9.00	lobChIP_FOXA1inPBS
chr17	80200807	80200808	MACS_peak_6857	6.00	lobChIP_FOXA1inPBS
chr17	80253690	80253691	MACS_peak_6858	26.00	lobChIP_FOXA1inPBS
chr17	80420027	80420028	MACS_peak_6859	10.00	lobChIP_FOXA1inPBS
chr17	80465884	80465885	MACS_peak_6860	10.00	lobChIP_FOXA1inPBS
chr17	80475382	80475383	MACS_peak_6861	8.00	lobChIP_FOXA1inPBS
chr17	80483644	80483645	MACS_peak_6862	7.00	lobChIP_FOXA1inPBS
chr17	80509639	80509640	MACS_peak_6863	5.00	lobChIP_FOXA1inPBS
chr17	80526456	80526457	MACS_peak_6864	70.00	lobChIP_FOXA1inPBS
chr17	80673602	80673603	MACS_peak_6865	7.00	lobChIP_FOXA1inPBS
chr17	80696334	80696335	MACS_peak_6866	24.00	lobChIP_FOXA1inPBS
chr17	80730041	80730042	MACS_peak_6867	18.00	lobChIP_FOXA1inPBS
chr17	80833389	80833390	MACS_peak_6868	9.00	lobChIP_FOXA1inPBS
chr17	80847494	80847495	MACS_peak_6869	38.00	lobChIP_FOXA1inPBS
chr17	81006557	81006558	MACS_peak_6870	14.00	lobChIP_FOXA1inPBS
chr17	81099538	81099539	MACS_peak_6871	38.00	lobChIP_FOXA1inPBS
chr17	81122343	81122344	MACS_peak_6872	79.00	lobChIP_FOXA1inPBS
chr17	81137673	81137674	MACS_peak_6873	21.00	lobChIP_FOXA1inPBS
chr18	189400	189401	MACS_peak_6874	26.00	lobChIP_FOXA1inPBS
chr18	323500	323501	MACS_peak_6875	8.00	lobChIP_FOXA1inPBS
chr18	362486	362487	MACS_peak_6876	11.00	lobChIP_FOXA1inPBS
chr18	499635	499636	MACS_peak_6877	9.00	lobChIP_FOXA1inPBS
chr18	673570	673571	MACS_peak_6878	8.00	lobChIP_FOXA1inPBS
chr18	682974	682975	MACS_peak_6879	19.00	lobChIP_FOXA1inPBS
chr18	750688	750689	MACS_peak_6880	9.00	lobChIP_FOXA1inPBS
chr18	770768	770769	MACS_peak_6881	11.00	lobChIP_FOXA1inPBS
chr18	775057	775058	MACS_peak_6882	6.00	lobChIP_FOXA1inPBS
chr18	1624817	1624818	MACS_peak_6883	23.00	lobChIP_FOXA1inPBS
chr18	1774496	1774497	MACS_peak_6884	7.00	lobChIP_FOXA1inPBS
chr18	2605351	2605352	MACS_peak_6885	10.00	lobChIP_FOXA1inPBS
chr18	2657825	2657826	MACS_peak_6886	7.00	lobChIP_FOXA1inPBS
chr18	2983016	2983017	MACS_peak_6887	7.00	lobChIP_FOXA1inPBS
chr18	2986600	2986601	MACS_peak_6888	24.00	lobChIP_FOXA1inPBS
chr18	3037612	3037613	MACS_peak_6889	20.00	lobChIP_FOXA1inPBS
chr18	3045575	3045576	MACS_peak_6890	7.00	lobChIP_FOXA1inPBS
chr18	3052266	3052267	MACS_peak_6891	22.00	lobChIP_FOXA1inPBS
chr18	3084303	3084304	MACS_peak_6892	7.00	lobChIP_FOXA1inPBS
chr18	3117781	3117782	MACS_peak_6893	26.00	lobChIP_FOXA1inPBS
chr18	3153545	3153546	MACS_peak_6894	10.00	lobChIP_FOXA1inPBS
chr18	3200485	3200486	MACS_peak_6895	18.00	lobChIP_FOXA1inPBS
chr18	3218147	3218148	MACS_peak_6896	12.00	lobChIP_FOXA1inPBS
chr18	3233879	3233880	MACS_peak_6897	10.00	lobChIP_FOXA1inPBS
chr18	3248783	3248784	MACS_peak_6898	14.00	lobChIP_FOXA1inPBS
chr18	3256770	3256771	MACS_peak_6899	15.00	lobChIP_FOXA1inPBS
chr18	3313382	3313383	MACS_peak_6900	12.00	lobChIP_FOXA1inPBS
chr18	3449207	3449208	MACS_peak_6901	22.00	lobChIP_FOXA1inPBS
chr18	3456890	3456891	MACS_peak_6902	28.00	lobChIP_FOXA1inPBS
chr18	3596050	3596051	MACS_peak_6903	21.00	lobChIP_FOXA1inPBS
chr18	3604946	3604947	MACS_peak_6904	9.00	lobChIP_FOXA1inPBS
chr18	3618300	3618301	MACS_peak_6905	58.00	lobChIP_FOXA1inPBS
chr18	3634456	3634457	MACS_peak_6906	18.00	lobChIP_FOXA1inPBS
chr18	3648332	3648333	MACS_peak_6907	10.00	lobChIP_FOXA1inPBS
chr18	3655268	3655269	MACS_peak_6908	7.00	lobChIP_FOXA1inPBS
chr18	3664819	3664820	MACS_peak_6909	9.00	lobChIP_FOXA1inPBS
chr18	3671218	3671219	MACS_peak_6910	9.00	lobChIP_FOXA1inPBS
chr18	4301458	4301459	MACS_peak_6911	8.00	lobChIP_FOXA1inPBS
chr18	4589320	4589321	MACS_peak_6912	11.00	lobChIP_FOXA1inPBS
chr18	5298557	5298558	MACS_peak_6913	7.00	lobChIP_FOXA1inPBS
chr18	5303849	5303850	MACS_peak_6914	15.00	lobChIP_FOXA1inPBS
chr18	5339713	5339714	MACS_peak_6915	9.00	lobChIP_FOXA1inPBS
chr18	5747144	5747145	MACS_peak_6916	16.00	lobChIP_FOXA1inPBS
chr18	5788138	5788139	MACS_peak_6917	10.00	lobChIP_FOXA1inPBS
chr18	5970310	5970311	MACS_peak_6918	9.00	lobChIP_FOXA1inPBS
chr18	5998760	5998761	MACS_peak_6919	9.00	lobChIP_FOXA1inPBS
chr18	6052608	6052609	MACS_peak_6920	29.00	lobChIP_FOXA1inPBS
chr18	6211067	6211068	MACS_peak_6921	7.00	lobChIP_FOXA1inPBS
chr18	6282043	6282044	MACS_peak_6922	9.00	lobChIP_FOXA1inPBS
chr18	6362683	6362684	MACS_peak_6923	6.00	lobChIP_FOXA1inPBS
chr18	6377092	6377093	MACS_peak_6924	7.00	lobChIP_FOXA1inPBS
chr18	6503652	6503653	MACS_peak_6925	9.00	lobChIP_FOXA1inPBS
chr18	6835558	6835559	MACS_peak_6926	15.00	lobChIP_FOXA1inPBS
chr18	6962743	6962744	MACS_peak_6927	7.00	lobChIP_FOXA1inPBS
chr18	7626666	7626667	MACS_peak_6928	12.00	lobChIP_FOXA1inPBS
chr18	7637464	7637465	MACS_peak_6929	14.00	lobChIP_FOXA1inPBS
chr18	7646420	7646421	MACS_peak_6930	23.00	lobChIP_FOXA1inPBS
chr18	7658077	7658078	MACS_peak_6931	6.00	lobChIP_FOXA1inPBS
chr18	7749915	7749916	MACS_peak_6932	15.00	lobChIP_FOXA1inPBS
chr18	7755236	7755237	MACS_peak_6933	12.00	lobChIP_FOXA1inPBS
chr18	7804468	7804469	MACS_peak_6934	16.00	lobChIP_FOXA1inPBS
chr18	7812782	7812783	MACS_peak_6935	8.00	lobChIP_FOXA1inPBS
chr18	7915002	7915003	MACS_peak_6936	6.00	lobChIP_FOXA1inPBS
chr18	8177761	8177762	MACS_peak_6937	7.00	lobChIP_FOXA1inPBS
chr18	8238721	8238722	MACS_peak_6938	15.00	lobChIP_FOXA1inPBS
chr18	8284130	8284131	MACS_peak_6939	14.00	lobChIP_FOXA1inPBS
chr18	8341778	8341779	MACS_peak_6940	37.00	lobChIP_FOXA1inPBS
chr18	8627687	8627688	MACS_peak_6941	9.00	lobChIP_FOXA1inPBS
chr18	8730533	8730534	MACS_peak_6942	18.00	lobChIP_FOXA1inPBS
chr18	8732733	8732734	MACS_peak_6943	7.00	lobChIP_FOXA1inPBS
chr18	8959803	8959804	MACS_peak_6944	15.00	lobChIP_FOXA1inPBS
chr18	9067976	9067977	MACS_peak_6945	6.00	lobChIP_FOXA1inPBS
chr18	9073817	9073818	MACS_peak_6946	15.00	lobChIP_FOXA1inPBS
chr18	9092839	9092840	MACS_peak_6947	17.00	lobChIP_FOXA1inPBS
chr18	9094834	9094835	MACS_peak_6948	26.00	lobChIP_FOXA1inPBS
chr18	9104766	9104767	MACS_peak_6949	13.00	lobChIP_FOXA1inPBS
chr18	9215320	9215321	MACS_peak_6950	11.00	lobChIP_FOXA1inPBS
chr18	9514255	9514256	MACS_peak_6951	8.00	lobChIP_FOXA1inPBS
chr18	9535552	9535553	MACS_peak_6952	14.00	lobChIP_FOXA1inPBS
chr18	9596352	9596353	MACS_peak_6953	8.00	lobChIP_FOXA1inPBS
chr18	9609039	9609040	MACS_peak_6954	23.00	lobChIP_FOXA1inPBS
chr18	9743019	9743020	MACS_peak_6955	9.00	lobChIP_FOXA1inPBS
chr18	9814610	9814611	MACS_peak_6956	17.00	lobChIP_FOXA1inPBS
chr18	9949799	9949800	MACS_peak_6957	6.00	lobChIP_FOXA1inPBS
chr18	10141143	10141144	MACS_peak_6958	11.00	lobChIP_FOXA1inPBS
chr18	10464320	10464321	MACS_peak_6959	13.00	lobChIP_FOXA1inPBS
chr18	10705891	10705892	MACS_peak_6960	12.00	lobChIP_FOXA1inPBS
chr18	10901581	10901582	MACS_peak_6961	7.00	lobChIP_FOXA1inPBS
chr18	10985893	10985894	MACS_peak_6962	11.00	lobChIP_FOXA1inPBS
chr18	11002527	11002528	MACS_peak_6963	11.00	lobChIP_FOXA1inPBS
chr18	11005780	11005781	MACS_peak_6964	20.00	lobChIP_FOXA1inPBS
chr18	11041592	11041593	MACS_peak_6965	11.00	lobChIP_FOXA1inPBS
chr18	11113326	11113327	MACS_peak_6966	11.00	lobChIP_FOXA1inPBS
chr18	11500990	11500991	MACS_peak_6967	33.00	lobChIP_FOXA1inPBS
chr18	11862008	11862009	MACS_peak_6968	5.00	lobChIP_FOXA1inPBS
chr18	11887861	11887862	MACS_peak_6969	7.00	lobChIP_FOXA1inPBS
chr18	11925960	11925961	MACS_peak_6970	22.00	lobChIP_FOXA1inPBS
chr18	11957966	11957967	MACS_peak_6971	18.00	lobChIP_FOXA1inPBS
chr18	11994564	11994565	MACS_peak_6972	22.00	lobChIP_FOXA1inPBS
chr18	12000487	12000488	MACS_peak_6973	16.00	lobChIP_FOXA1inPBS
chr18	12008446	12008447	MACS_peak_6974	29.00	lobChIP_FOXA1inPBS
chr18	12337097	12337098	MACS_peak_6975	15.00	lobChIP_FOXA1inPBS
chr18	12411053	12411054	MACS_peak_6976	15.00	lobChIP_FOXA1inPBS
chr18	12461485	12461486	MACS_peak_6977	15.00	lobChIP_FOXA1inPBS
chr18	12574986	12574987	MACS_peak_6978	10.00	lobChIP_FOXA1inPBS
chr18	12595985	12595986	MACS_peak_6979	9.00	lobChIP_FOXA1inPBS
chr18	12598007	12598008	MACS_peak_6980	6.00	lobChIP_FOXA1inPBS
chr18	12608473	12608474	MACS_peak_6981	16.00	lobChIP_FOXA1inPBS
chr18	12640487	12640488	MACS_peak_6982	8.00	lobChIP_FOXA1inPBS
chr18	12699980	12699981	MACS_peak_6983	14.00	lobChIP_FOXA1inPBS
chr18	12730435	12730436	MACS_peak_6984	24.00	lobChIP_FOXA1inPBS
chr18	12732637	12732638	MACS_peak_6985	7.00	lobChIP_FOXA1inPBS
chr18	12741444	12741445	MACS_peak_6986	14.00	lobChIP_FOXA1inPBS
chr18	12931036	12931037	MACS_peak_6987	6.00	lobChIP_FOXA1inPBS
chr18	13147231	13147232	MACS_peak_6988	14.00	lobChIP_FOXA1inPBS
chr18	13176019	13176020	MACS_peak_6989	26.00	lobChIP_FOXA1inPBS
chr18	13179733	13179734	MACS_peak_6990	40.00	lobChIP_FOXA1inPBS
chr18	13270467	13270468	MACS_peak_6991	11.00	lobChIP_FOXA1inPBS
chr18	13313168	13313169	MACS_peak_6992	7.00	lobChIP_FOXA1inPBS
chr18	13338428	13338429	MACS_peak_6993	15.00	lobChIP_FOXA1inPBS
chr18	13472849	13472850	MACS_peak_6994	13.00	lobChIP_FOXA1inPBS
chr18	13540302	13540303	MACS_peak_6995	11.00	lobChIP_FOXA1inPBS
chr18	13542267	13542268	MACS_peak_6996	10.00	lobChIP_FOXA1inPBS
chr18	18966981	18966982	MACS_peak_6997	11.00	lobChIP_FOXA1inPBS
chr18	19130631	19130632	MACS_peak_6998	25.00	lobChIP_FOXA1inPBS
chr18	19218474	19218475	MACS_peak_6999	21.00	lobChIP_FOXA1inPBS
chr18	19266646	19266647	MACS_peak_7000	8.00	lobChIP_FOXA1inPBS
chr18	19315707	19315708	MACS_peak_7001	11.00	lobChIP_FOXA1inPBS
chr18	19445994	19445995	MACS_peak_7002	9.00	lobChIP_FOXA1inPBS
chr18	19558689	19558690	MACS_peak_7003	10.00	lobChIP_FOXA1inPBS
chr18	19571144	19571145	MACS_peak_7004	24.00	lobChIP_FOXA1inPBS
chr18	19581858	19581859	MACS_peak_7005	25.00	lobChIP_FOXA1inPBS
chr18	19598344	19598345	MACS_peak_7006	9.00	lobChIP_FOXA1inPBS
chr18	19629082	19629083	MACS_peak_7007	10.00	lobChIP_FOXA1inPBS
chr18	19755423	19755424	MACS_peak_7008	51.00	lobChIP_FOXA1inPBS
chr18	20284500	20284501	MACS_peak_7009	25.00	lobChIP_FOXA1inPBS
chr18	20453355	20453356	MACS_peak_7010	9.00	lobChIP_FOXA1inPBS
chr18	20516567	20516568	MACS_peak_7011	10.00	lobChIP_FOXA1inPBS
chr18	20679790	20679791	MACS_peak_7012	13.00	lobChIP_FOXA1inPBS
chr18	20681943	20681944	MACS_peak_7013	13.00	lobChIP_FOXA1inPBS
chr18	20714124	20714125	MACS_peak_7014	9.00	lobChIP_FOXA1inPBS
chr18	20721462	20721463	MACS_peak_7015	8.00	lobChIP_FOXA1inPBS
chr18	20722487	20722488	MACS_peak_7016	5.00	lobChIP_FOXA1inPBS
chr18	20758851	20758852	MACS_peak_7017	10.00	lobChIP_FOXA1inPBS
chr18	20937265	20937266	MACS_peak_7018	6.00	lobChIP_FOXA1inPBS
chr18	20965321	20965322	MACS_peak_7019	21.00	lobChIP_FOXA1inPBS
chr18	20981727	20981728	MACS_peak_7020	8.00	lobChIP_FOXA1inPBS
chr18	21022744	21022745	MACS_peak_7021	7.00	lobChIP_FOXA1inPBS
chr18	21125546	21125547	MACS_peak_7022	17.00	lobChIP_FOXA1inPBS
chr18	21186080	21186081	MACS_peak_7023	10.00	lobChIP_FOXA1inPBS
chr18	21198987	21198988	MACS_peak_7024	7.00	lobChIP_FOXA1inPBS
chr18	21209541	21209542	MACS_peak_7025	27.00	lobChIP_FOXA1inPBS
chr18	21231598	21231599	MACS_peak_7026	7.00	lobChIP_FOXA1inPBS
chr18	21438018	21438019	MACS_peak_7027	8.00	lobChIP_FOXA1inPBS
chr18	21439619	21439620	MACS_peak_7028	7.00	lobChIP_FOXA1inPBS
chr18	21463670	21463671	MACS_peak_7029	8.00	lobChIP_FOXA1inPBS
chr18	21562808	21562809	MACS_peak_7030	9.00	lobChIP_FOXA1inPBS
chr18	21631321	21631322	MACS_peak_7031	32.00	lobChIP_FOXA1inPBS
chr18	21632264	21632265	MACS_peak_7032	17.00	lobChIP_FOXA1inPBS
chr18	21643812	21643813	MACS_peak_7033	12.00	lobChIP_FOXA1inPBS
chr18	21651935	21651936	MACS_peak_7034	18.00	lobChIP_FOXA1inPBS
chr18	21695336	21695337	MACS_peak_7035	11.00	lobChIP_FOXA1inPBS
chr18	21795947	21795948	MACS_peak_7036	17.00	lobChIP_FOXA1inPBS
chr18	22657156	22657157	MACS_peak_7037	8.00	lobChIP_FOXA1inPBS
chr18	23887411	23887412	MACS_peak_7038	8.00	lobChIP_FOXA1inPBS
chr18	24125363	24125364	MACS_peak_7039	14.00	lobChIP_FOXA1inPBS
chr18	24622354	24622355	MACS_peak_7040	13.00	lobChIP_FOXA1inPBS
chr18	24674844	24674845	MACS_peak_7041	10.00	lobChIP_FOXA1inPBS
chr18	24778230	24778231	MACS_peak_7042	16.00	lobChIP_FOXA1inPBS
chr18	25165708	25165709	MACS_peak_7043	15.00	lobChIP_FOXA1inPBS
chr18	25457744	25457745	MACS_peak_7044	10.00	lobChIP_FOXA1inPBS
chr18	25548147	25548148	MACS_peak_7045	9.00	lobChIP_FOXA1inPBS
chr18	25653833	25653834	MACS_peak_7046	9.00	lobChIP_FOXA1inPBS
chr18	25671367	25671368	MACS_peak_7047	9.00	lobChIP_FOXA1inPBS
chr18	25692810	25692811	MACS_peak_7048	18.00	lobChIP_FOXA1inPBS
chr18	25708010	25708011	MACS_peak_7049	11.00	lobChIP_FOXA1inPBS
chr18	25742115	25742116	MACS_peak_7050	9.00	lobChIP_FOXA1inPBS
chr18	25747878	25747879	MACS_peak_7051	42.00	lobChIP_FOXA1inPBS
chr18	27204222	27204223	MACS_peak_7052	5.00	lobChIP_FOXA1inPBS
chr18	27383206	27383207	MACS_peak_7053	7.00	lobChIP_FOXA1inPBS
chr18	27887116	27887117	MACS_peak_7054	9.00	lobChIP_FOXA1inPBS
chr18	28591559	28591560	MACS_peak_7055	8.00	lobChIP_FOXA1inPBS
chr18	28810665	28810666	MACS_peak_7056	14.00	lobChIP_FOXA1inPBS
chr18	28822065	28822066	MACS_peak_7057	13.00	lobChIP_FOXA1inPBS
chr18	28842309	28842310	MACS_peak_7058	13.00	lobChIP_FOXA1inPBS
chr18	28854349	28854350	MACS_peak_7059	12.00	lobChIP_FOXA1inPBS
chr18	28855051	28855052	MACS_peak_7060	14.00	lobChIP_FOXA1inPBS
chr18	28858689	28858690	MACS_peak_7061	10.00	lobChIP_FOXA1inPBS
chr18	28953844	28953845	MACS_peak_7062	12.00	lobChIP_FOXA1inPBS
chr18	28977450	28977451	MACS_peak_7063	13.00	lobChIP_FOXA1inPBS
chr18	28982062	28982063	MACS_peak_7064	12.00	lobChIP_FOXA1inPBS
chr18	29092335	29092336	MACS_peak_7065	57.00	lobChIP_FOXA1inPBS
chr18	29138722	29138723	MACS_peak_7066	9.00	lobChIP_FOXA1inPBS
chr18	29166705	29166706	MACS_peak_7067	13.00	lobChIP_FOXA1inPBS
chr18	29167675	29167676	MACS_peak_7068	24.00	lobChIP_FOXA1inPBS
chr18	29171738	29171739	MACS_peak_7069	23.00	lobChIP_FOXA1inPBS
chr18	29176317	29176318	MACS_peak_7070	8.00	lobChIP_FOXA1inPBS
chr18	29266823	29266824	MACS_peak_7071	11.00	lobChIP_FOXA1inPBS
chr18	29523836	29523837	MACS_peak_7072	9.00	lobChIP_FOXA1inPBS
chr18	29530291	29530292	MACS_peak_7073	8.00	lobChIP_FOXA1inPBS
chr18	29599576	29599577	MACS_peak_7074	7.00	lobChIP_FOXA1inPBS
chr18	29941703	29941704	MACS_peak_7075	9.00	lobChIP_FOXA1inPBS
chr18	29952166	29952167	MACS_peak_7076	10.00	lobChIP_FOXA1inPBS
chr18	30031780	30031781	MACS_peak_7077	7.00	lobChIP_FOXA1inPBS
chr18	30344774	30344775	MACS_peak_7078	11.00	lobChIP_FOXA1inPBS
chr18	30378469	30378470	MACS_peak_7079	11.00	lobChIP_FOXA1inPBS
chr18	30383776	30383777	MACS_peak_7080	52.00	lobChIP_FOXA1inPBS
chr18	30384771	30384772	MACS_peak_7081	25.00	lobChIP_FOXA1inPBS
chr18	30490397	30490398	MACS_peak_7082	13.00	lobChIP_FOXA1inPBS
chr18	30594232	30594233	MACS_peak_7083	26.00	lobChIP_FOXA1inPBS
chr18	31331808	31331809	MACS_peak_7084	26.00	lobChIP_FOXA1inPBS
chr18	31944906	31944907	MACS_peak_7085	15.00	lobChIP_FOXA1inPBS
chr18	32072780	32072781	MACS_peak_7086	14.00	lobChIP_FOXA1inPBS
chr18	32123391	32123392	MACS_peak_7087	9.00	lobChIP_FOXA1inPBS
chr18	32128328	32128329	MACS_peak_7088	13.00	lobChIP_FOXA1inPBS
chr18	32211751	32211752	MACS_peak_7089	21.00	lobChIP_FOXA1inPBS
chr18	32494136	32494137	MACS_peak_7090	14.00	lobChIP_FOXA1inPBS
chr18	32494978	32494979	MACS_peak_7091	9.00	lobChIP_FOXA1inPBS
chr18	32505464	32505465	MACS_peak_7092	6.00	lobChIP_FOXA1inPBS
chr18	32509378	32509379	MACS_peak_7093	28.00	lobChIP_FOXA1inPBS
chr18	32528294	32528295	MACS_peak_7094	8.00	lobChIP_FOXA1inPBS
chr18	32537264	32537265	MACS_peak_7095	10.00	lobChIP_FOXA1inPBS
chr18	32539322	32539323	MACS_peak_7096	18.00	lobChIP_FOXA1inPBS
chr18	32559375	32559376	MACS_peak_7097	8.00	lobChIP_FOXA1inPBS
chr18	32664871	32664872	MACS_peak_7098	10.00	lobChIP_FOXA1inPBS
chr18	32665896	32665897	MACS_peak_7099	10.00	lobChIP_FOXA1inPBS
chr18	32751447	32751448	MACS_peak_7100	17.00	lobChIP_FOXA1inPBS
chr18	32925487	32925488	MACS_peak_7101	11.00	lobChIP_FOXA1inPBS
chr18	33038425	33038426	MACS_peak_7102	9.00	lobChIP_FOXA1inPBS
chr18	33049741	33049742	MACS_peak_7103	18.00	lobChIP_FOXA1inPBS
chr18	33062073	33062074	MACS_peak_7104	15.00	lobChIP_FOXA1inPBS
chr18	33133643	33133644	MACS_peak_7105	7.00	lobChIP_FOXA1inPBS
chr18	33283243	33283244	MACS_peak_7106	24.00	lobChIP_FOXA1inPBS
chr18	33609133	33609134	MACS_peak_7107	14.00	lobChIP_FOXA1inPBS
chr18	33711830	33711831	MACS_peak_7108	12.00	lobChIP_FOXA1inPBS
chr18	33756211	33756212	MACS_peak_7109	8.00	lobChIP_FOXA1inPBS
chr18	34044210	34044211	MACS_peak_7110	7.00	lobChIP_FOXA1inPBS
chr18	34436787	34436788	MACS_peak_7111	9.00	lobChIP_FOXA1inPBS
chr18	36821765	36821766	MACS_peak_7112	28.00	lobChIP_FOXA1inPBS
chr18	36987172	36987173	MACS_peak_7113	9.00	lobChIP_FOXA1inPBS
chr18	37200602	37200603	MACS_peak_7114	13.00	lobChIP_FOXA1inPBS
chr18	37281986	37281987	MACS_peak_7115	8.00	lobChIP_FOXA1inPBS
chr18	39627550	39627551	MACS_peak_7116	10.00	lobChIP_FOXA1inPBS
chr18	39692820	39692821	MACS_peak_7117	14.00	lobChIP_FOXA1inPBS
chr18	39748606	39748607	MACS_peak_7118	18.00	lobChIP_FOXA1inPBS
chr18	39920612	39920613	MACS_peak_7119	9.00	lobChIP_FOXA1inPBS
chr18	40026328	40026329	MACS_peak_7120	18.00	lobChIP_FOXA1inPBS
chr18	43632200	43632201	MACS_peak_7121	34.00	lobChIP_FOXA1inPBS
chr18	43773166	43773167	MACS_peak_7122	7.00	lobChIP_FOXA1inPBS
chr18	43779666	43779667	MACS_peak_7123	11.00	lobChIP_FOXA1inPBS
chr18	44411595	44411596	MACS_peak_7124	7.00	lobChIP_FOXA1inPBS
chr18	44622363	44622364	MACS_peak_7125	28.00	lobChIP_FOXA1inPBS
chr18	45101576	45101577	MACS_peak_7126	46.00	lobChIP_FOXA1inPBS
chr18	45408304	45408305	MACS_peak_7127	8.00	lobChIP_FOXA1inPBS
chr18	46015162	46015163	MACS_peak_7128	7.00	lobChIP_FOXA1inPBS
chr18	46039018	46039019	MACS_peak_7129	7.00	lobChIP_FOXA1inPBS
chr18	46067041	46067042	MACS_peak_7130	6.00	lobChIP_FOXA1inPBS
chr18	46102493	46102494	MACS_peak_7131	14.00	lobChIP_FOXA1inPBS
chr18	46156142	46156143	MACS_peak_7132	9.00	lobChIP_FOXA1inPBS
chr18	46161466	46161467	MACS_peak_7133	20.00	lobChIP_FOXA1inPBS
chr18	46428489	46428490	MACS_peak_7134	14.00	lobChIP_FOXA1inPBS
chr18	46436534	46436535	MACS_peak_7135	6.00	lobChIP_FOXA1inPBS
chr18	46473071	46473072	MACS_peak_7136	29.00	lobChIP_FOXA1inPBS
chr18	46474971	46474972	MACS_peak_7137	7.00	lobChIP_FOXA1inPBS
chr18	46520606	46520607	MACS_peak_7138	5.00	lobChIP_FOXA1inPBS
chr18	46597112	46597113	MACS_peak_7139	12.00	lobChIP_FOXA1inPBS
chr18	46626791	46626792	MACS_peak_7140	8.00	lobChIP_FOXA1inPBS
chr18	46781318	46781319	MACS_peak_7141	14.00	lobChIP_FOXA1inPBS
chr18	46812327	46812328	MACS_peak_7142	8.00	lobChIP_FOXA1inPBS
chr18	46816777	46816778	MACS_peak_7143	13.00	lobChIP_FOXA1inPBS
chr18	46841271	46841272	MACS_peak_7144	8.00	lobChIP_FOXA1inPBS
chr18	47156883	47156884	MACS_peak_7145	7.00	lobChIP_FOXA1inPBS
chr18	47165634	47165635	MACS_peak_7146	16.00	lobChIP_FOXA1inPBS
chr18	47358813	47358814	MACS_peak_7147	16.00	lobChIP_FOXA1inPBS
chr18	47361550	47361551	MACS_peak_7148	34.00	lobChIP_FOXA1inPBS
chr18	47362226	47362227	MACS_peak_7149	9.00	lobChIP_FOXA1inPBS
chr18	47363561	47363562	MACS_peak_7150	5.00	lobChIP_FOXA1inPBS
chr18	47364508	47364509	MACS_peak_7151	35.00	lobChIP_FOXA1inPBS
chr18	47437870	47437871	MACS_peak_7152	25.00	lobChIP_FOXA1inPBS
chr18	47617225	47617226	MACS_peak_7153	26.00	lobChIP_FOXA1inPBS
chr18	47652113	47652114	MACS_peak_7154	6.00	lobChIP_FOXA1inPBS
chr18	47920347	47920348	MACS_peak_7155	23.00	lobChIP_FOXA1inPBS
chr18	47931354	47931355	MACS_peak_7156	10.00	lobChIP_FOXA1inPBS
chr18	48002165	48002166	MACS_peak_7157	15.00	lobChIP_FOXA1inPBS
chr18	48141308	48141309	MACS_peak_7158	13.00	lobChIP_FOXA1inPBS
chr18	48333940	48333941	MACS_peak_7159	24.00	lobChIP_FOXA1inPBS
chr18	48429311	48429312	MACS_peak_7160	48.00	lobChIP_FOXA1inPBS
chr18	48500238	48500239	MACS_peak_7161	13.00	lobChIP_FOXA1inPBS
chr18	48555977	48555978	MACS_peak_7162	7.00	lobChIP_FOXA1inPBS
chr18	48564644	48564645	MACS_peak_7163	21.00	lobChIP_FOXA1inPBS
chr18	50551823	50551824	MACS_peak_7164	19.00	lobChIP_FOXA1inPBS
chr18	51773446	51773447	MACS_peak_7165	14.00	lobChIP_FOXA1inPBS
chr18	51780913	51780914	MACS_peak_7166	11.00	lobChIP_FOXA1inPBS
chr18	51787271	51787272	MACS_peak_7167	11.00	lobChIP_FOXA1inPBS
chr18	52433132	52433133	MACS_peak_7168	8.00	lobChIP_FOXA1inPBS
chr18	52613489	52613490	MACS_peak_7169	5.00	lobChIP_FOXA1inPBS
chr18	52615846	52615847	MACS_peak_7170	27.00	lobChIP_FOXA1inPBS
chr18	52624901	52624902	MACS_peak_7171	10.00	lobChIP_FOXA1inPBS
chr18	54809055	54809056	MACS_peak_7172	10.00	lobChIP_FOXA1inPBS
chr18	54911225	54911226	MACS_peak_7173	20.00	lobChIP_FOXA1inPBS
chr18	55089778	55089779	MACS_peak_7174	11.00	lobChIP_FOXA1inPBS
chr18	55091211	55091212	MACS_peak_7175	46.00	lobChIP_FOXA1inPBS
chr18	55113491	55113492	MACS_peak_7176	8.00	lobChIP_FOXA1inPBS
chr18	55200734	55200735	MACS_peak_7177	9.00	lobChIP_FOXA1inPBS
chr18	55293069	55293070	MACS_peak_7178	9.00	lobChIP_FOXA1inPBS
chr18	55405055	55405056	MACS_peak_7179	16.00	lobChIP_FOXA1inPBS
chr18	55416136	55416137	MACS_peak_7180	29.00	lobChIP_FOXA1inPBS
chr18	55439854	55439855	MACS_peak_7181	13.00	lobChIP_FOXA1inPBS
chr18	55464556	55464557	MACS_peak_7182	23.00	lobChIP_FOXA1inPBS
chr18	55478986	55478987	MACS_peak_7183	7.00	lobChIP_FOXA1inPBS
chr18	55594921	55594922	MACS_peak_7184	19.00	lobChIP_FOXA1inPBS
chr18	55618876	55618877	MACS_peak_7185	11.00	lobChIP_FOXA1inPBS
chr18	55648384	55648385	MACS_peak_7186	26.00	lobChIP_FOXA1inPBS
chr18	55679814	55679815	MACS_peak_7187	7.00	lobChIP_FOXA1inPBS
chr18	55680909	55680910	MACS_peak_7188	57.00	lobChIP_FOXA1inPBS
chr18	55682033	55682034	MACS_peak_7189	8.00	lobChIP_FOXA1inPBS
chr18	55684450	55684451	MACS_peak_7190	7.00	lobChIP_FOXA1inPBS
chr18	55691726	55691727	MACS_peak_7191	7.00	lobChIP_FOXA1inPBS
chr18	55710873	55710874	MACS_peak_7192	15.00	lobChIP_FOXA1inPBS
chr18	55713828	55713829	MACS_peak_7193	12.00	lobChIP_FOXA1inPBS
chr18	55902464	55902465	MACS_peak_7194	7.00	lobChIP_FOXA1inPBS
chr18	55953090	55953091	MACS_peak_7195	9.00	lobChIP_FOXA1inPBS
chr18	56075089	56075090	MACS_peak_7196	86.00	lobChIP_FOXA1inPBS
chr18	56080190	56080191	MACS_peak_7197	11.00	lobChIP_FOXA1inPBS
chr18	56081382	56081383	MACS_peak_7198	80.00	lobChIP_FOXA1inPBS
chr18	56194133	56194134	MACS_peak_7199	8.00	lobChIP_FOXA1inPBS
chr18	56217312	56217313	MACS_peak_7200	51.00	lobChIP_FOXA1inPBS
chr18	56272627	56272628	MACS_peak_7201	21.00	lobChIP_FOXA1inPBS
chr18	56337131	56337132	MACS_peak_7202	22.00	lobChIP_FOXA1inPBS
chr18	56620273	56620274	MACS_peak_7203	21.00	lobChIP_FOXA1inPBS
chr18	56809178	56809179	MACS_peak_7204	13.00	lobChIP_FOXA1inPBS
chr18	56810216	56810217	MACS_peak_7205	13.00	lobChIP_FOXA1inPBS
chr18	57020880	57020881	MACS_peak_7206	5.00	lobChIP_FOXA1inPBS
chr18	57120988	57120989	MACS_peak_7207	44.00	lobChIP_FOXA1inPBS
chr18	57183807	57183808	MACS_peak_7208	12.00	lobChIP_FOXA1inPBS
chr18	57663781	57663782	MACS_peak_7209	14.00	lobChIP_FOXA1inPBS
chr18	58045200	58045201	MACS_peak_7210	13.00	lobChIP_FOXA1inPBS
chr18	60088818	60088819	MACS_peak_7211	6.00	lobChIP_FOXA1inPBS
chr18	60093015	60093016	MACS_peak_7212	10.00	lobChIP_FOXA1inPBS
chr18	60119750	60119751	MACS_peak_7213	43.00	lobChIP_FOXA1inPBS
chr18	60127984	60127985	MACS_peak_7214	8.00	lobChIP_FOXA1inPBS
chr18	60168349	60168350	MACS_peak_7215	11.00	lobChIP_FOXA1inPBS
chr18	60178178	60178179	MACS_peak_7216	12.00	lobChIP_FOXA1inPBS
chr18	60391287	60391288	MACS_peak_7217	16.00	lobChIP_FOXA1inPBS
chr18	60408566	60408567	MACS_peak_7218	11.00	lobChIP_FOXA1inPBS
chr18	60468068	60468069	MACS_peak_7219	18.00	lobChIP_FOXA1inPBS
chr18	60477321	60477322	MACS_peak_7220	13.00	lobChIP_FOXA1inPBS
chr18	61090337	61090338	MACS_peak_7221	6.00	lobChIP_FOXA1inPBS
chr18	62388528	62388529	MACS_peak_7222	10.00	lobChIP_FOXA1inPBS
chr18	62921440	62921441	MACS_peak_7223	9.00	lobChIP_FOXA1inPBS
chr18	64266608	64266609	MACS_peak_7224	8.00	lobChIP_FOXA1inPBS
chr18	64541386	64541387	MACS_peak_7225	10.00	lobChIP_FOXA1inPBS
chr18	64542496	64542497	MACS_peak_7226	50.00	lobChIP_FOXA1inPBS
chr18	64801073	64801074	MACS_peak_7227	8.00	lobChIP_FOXA1inPBS
chr18	64867709	64867710	MACS_peak_7228	9.00	lobChIP_FOXA1inPBS
chr18	65845556	65845557	MACS_peak_7229	15.00	lobChIP_FOXA1inPBS
chr18	65876834	65876835	MACS_peak_7230	6.00	lobChIP_FOXA1inPBS
chr18	66554770	66554771	MACS_peak_7231	14.00	lobChIP_FOXA1inPBS
chr18	67017239	67017240	MACS_peak_7232	38.00	lobChIP_FOXA1inPBS
chr18	67026011	67026012	MACS_peak_7233	13.00	lobChIP_FOXA1inPBS
chr18	67326743	67326744	MACS_peak_7234	6.00	lobChIP_FOXA1inPBS
chr18	67420337	67420338	MACS_peak_7235	29.00	lobChIP_FOXA1inPBS
chr18	67446118	67446119	MACS_peak_7236	9.00	lobChIP_FOXA1inPBS
chr18	67813607	67813608	MACS_peak_7237	15.00	lobChIP_FOXA1inPBS
chr18	67981041	67981042	MACS_peak_7238	10.00	lobChIP_FOXA1inPBS
chr18	68000116	68000117	MACS_peak_7239	19.00	lobChIP_FOXA1inPBS
chr18	68001021	68001022	MACS_peak_7240	12.00	lobChIP_FOXA1inPBS
chr18	68002431	68002432	MACS_peak_7241	37.00	lobChIP_FOXA1inPBS
chr18	68041379	68041380	MACS_peak_7242	6.00	lobChIP_FOXA1inPBS
chr18	68044706	68044707	MACS_peak_7243	8.00	lobChIP_FOXA1inPBS
chr18	68098583	68098584	MACS_peak_7244	22.00	lobChIP_FOXA1inPBS
chr18	68129494	68129495	MACS_peak_7245	11.00	lobChIP_FOXA1inPBS
chr18	70857129	70857130	MACS_peak_7246	20.00	lobChIP_FOXA1inPBS
chr18	71863593	71863594	MACS_peak_7247	19.00	lobChIP_FOXA1inPBS
chr18	71893058	71893059	MACS_peak_7248	14.00	lobChIP_FOXA1inPBS
chr18	71901722	71901723	MACS_peak_7249	17.00	lobChIP_FOXA1inPBS
chr18	71950025	71950026	MACS_peak_7250	7.00	lobChIP_FOXA1inPBS
chr18	71984118	71984119	MACS_peak_7251	35.00	lobChIP_FOXA1inPBS
chr18	72148526	72148527	MACS_peak_7252	9.00	lobChIP_FOXA1inPBS
chr18	72445947	72445948	MACS_peak_7253	8.00	lobChIP_FOXA1inPBS
chr18	72552669	72552670	MACS_peak_7254	22.00	lobChIP_FOXA1inPBS
chr18	73002797	73002798	MACS_peak_7255	20.00	lobChIP_FOXA1inPBS
chr18	73544140	73544141	MACS_peak_7256	10.00	lobChIP_FOXA1inPBS
chr18	74088401	74088402	MACS_peak_7257	28.00	lobChIP_FOXA1inPBS
chr18	74205788	74205789	MACS_peak_7258	11.00	lobChIP_FOXA1inPBS
chr18	74266041	74266042	MACS_peak_7259	9.00	lobChIP_FOXA1inPBS
chr18	75079345	75079346	MACS_peak_7260	13.00	lobChIP_FOXA1inPBS
chr18	77551456	77551457	MACS_peak_7261	9.00	lobChIP_FOXA1inPBS
chr18	77694842	77694843	MACS_peak_7262	41.00	lobChIP_FOXA1inPBS
chr18	77796069	77796070	MACS_peak_7263	6.00	lobChIP_FOXA1inPBS
chr19	459935	459936	MACS_peak_7264	7.00	lobChIP_FOXA1inPBS
chr19	513076	513077	MACS_peak_7265	9.00	lobChIP_FOXA1inPBS
chr19	661010	661011	MACS_peak_7266	11.00	lobChIP_FOXA1inPBS
chr19	752933	752934	MACS_peak_7267	8.00	lobChIP_FOXA1inPBS
chr19	770546	770547	MACS_peak_7268	8.00	lobChIP_FOXA1inPBS
chr19	800692	800693	MACS_peak_7269	8.00	lobChIP_FOXA1inPBS
chr19	909419	909420	MACS_peak_7270	15.00	lobChIP_FOXA1inPBS
chr19	935034	935035	MACS_peak_7271	17.00	lobChIP_FOXA1inPBS
chr19	1075615	1075616	MACS_peak_7272	10.00	lobChIP_FOXA1inPBS
chr19	1304411	1304412	MACS_peak_7273	7.00	lobChIP_FOXA1inPBS
chr19	1400624	1400625	MACS_peak_7274	7.00	lobChIP_FOXA1inPBS
chr19	1871523	1871524	MACS_peak_7275	24.00	lobChIP_FOXA1inPBS
chr19	2263759	2263760	MACS_peak_7276	9.00	lobChIP_FOXA1inPBS
chr19	2264595	2264596	MACS_peak_7277	9.00	lobChIP_FOXA1inPBS
chr19	2428047	2428048	MACS_peak_7278	11.00	lobChIP_FOXA1inPBS
chr19	2507279	2507280	MACS_peak_7279	25.00	lobChIP_FOXA1inPBS
chr19	2576292	2576293	MACS_peak_7280	8.00	lobChIP_FOXA1inPBS
chr19	2588454	2588455	MACS_peak_7281	7.00	lobChIP_FOXA1inPBS
chr19	2592110	2592111	MACS_peak_7282	8.00	lobChIP_FOXA1inPBS
chr19	2619310	2619311	MACS_peak_7283	19.00	lobChIP_FOXA1inPBS
chr19	2685792	2685793	MACS_peak_7284	7.00	lobChIP_FOXA1inPBS
chr19	2839703	2839704	MACS_peak_7285	9.00	lobChIP_FOXA1inPBS
chr19	3065880	3065881	MACS_peak_7286	10.00	lobChIP_FOXA1inPBS
chr19	3343642	3343643	MACS_peak_7287	8.00	lobChIP_FOXA1inPBS
chr19	3404233	3404234	MACS_peak_7288	12.00	lobChIP_FOXA1inPBS
chr19	3414055	3414056	MACS_peak_7289	10.00	lobChIP_FOXA1inPBS
chr19	3444039	3444040	MACS_peak_7290	7.00	lobChIP_FOXA1inPBS
chr19	3686879	3686880	MACS_peak_7291	11.00	lobChIP_FOXA1inPBS
chr19	3710614	3710615	MACS_peak_7292	11.00	lobChIP_FOXA1inPBS
chr19	3968848	3968849	MACS_peak_7293	19.00	lobChIP_FOXA1inPBS
chr19	4147476	4147477	MACS_peak_7294	23.00	lobChIP_FOXA1inPBS
chr19	4149883	4149884	MACS_peak_7295	7.00	lobChIP_FOXA1inPBS
chr19	4624448	4624449	MACS_peak_7296	7.00	lobChIP_FOXA1inPBS
chr19	4715923	4715924	MACS_peak_7297	8.00	lobChIP_FOXA1inPBS
chr19	4813193	4813194	MACS_peak_7298	7.00	lobChIP_FOXA1inPBS
chr19	5002364	5002365	MACS_peak_7299	9.00	lobChIP_FOXA1inPBS
chr19	5542875	5542876	MACS_peak_7300	8.00	lobChIP_FOXA1inPBS
chr19	5577886	5577887	MACS_peak_7301	8.00	lobChIP_FOXA1inPBS
chr19	5870419	5870420	MACS_peak_7302	8.00	lobChIP_FOXA1inPBS
chr19	5871211	5871212	MACS_peak_7303	14.00	lobChIP_FOXA1inPBS
chr19	5909321	5909322	MACS_peak_7304	14.00	lobChIP_FOXA1inPBS
chr19	5910190	5910191	MACS_peak_7305	11.00	lobChIP_FOXA1inPBS
chr19	5954869	5954870	MACS_peak_7306	13.00	lobChIP_FOXA1inPBS
chr19	5962264	5962265	MACS_peak_7307	7.00	lobChIP_FOXA1inPBS
chr19	5968151	5968152	MACS_peak_7308	6.00	lobChIP_FOXA1inPBS
chr19	5975128	5975129	MACS_peak_7309	9.00	lobChIP_FOXA1inPBS
chr19	6056357	6056358	MACS_peak_7310	11.00	lobChIP_FOXA1inPBS
chr19	6066115	6066116	MACS_peak_7311	12.00	lobChIP_FOXA1inPBS
chr19	6199866	6199867	MACS_peak_7312	16.00	lobChIP_FOXA1inPBS
chr19	6202100	6202101	MACS_peak_7313	9.00	lobChIP_FOXA1inPBS
chr19	6273994	6273995	MACS_peak_7314	17.00	lobChIP_FOXA1inPBS
chr19	6505022	6505023	MACS_peak_7315	8.00	lobChIP_FOXA1inPBS
chr19	6568100	6568101	MACS_peak_7316	8.00	lobChIP_FOXA1inPBS
chr19	7135197	7135198	MACS_peak_7317	17.00	lobChIP_FOXA1inPBS
chr19	7214576	7214577	MACS_peak_7318	18.00	lobChIP_FOXA1inPBS
chr19	7224417	7224418	MACS_peak_7319	12.00	lobChIP_FOXA1inPBS
chr19	7296369	7296370	MACS_peak_7320	8.00	lobChIP_FOXA1inPBS
chr19	7463836	7463837	MACS_peak_7321	47.00	lobChIP_FOXA1inPBS
chr19	7480696	7480697	MACS_peak_7322	14.00	lobChIP_FOXA1inPBS
chr19	7694719	7694720	MACS_peak_7323	8.00	lobChIP_FOXA1inPBS
chr19	7717258	7717259	MACS_peak_7324	13.00	lobChIP_FOXA1inPBS
chr19	7957542	7957543	MACS_peak_7325	6.00	lobChIP_FOXA1inPBS
chr19	8151176	8151177	MACS_peak_7326	30.00	lobChIP_FOXA1inPBS
chr19	8253773	8253774	MACS_peak_7327	33.00	lobChIP_FOXA1inPBS
chr19	8305495	8305496	MACS_peak_7328	10.00	lobChIP_FOXA1inPBS
chr19	8311515	8311516	MACS_peak_7329	20.00	lobChIP_FOXA1inPBS
chr19	8323158	8323159	MACS_peak_7330	11.00	lobChIP_FOXA1inPBS
chr19	8422322	8422323	MACS_peak_7331	11.00	lobChIP_FOXA1inPBS
chr19	8439364	8439365	MACS_peak_7332	43.00	lobChIP_FOXA1inPBS
chr19	8453171	8453172	MACS_peak_7333	40.00	lobChIP_FOXA1inPBS
chr19	8487064	8487065	MACS_peak_7334	9.00	lobChIP_FOXA1inPBS
chr19	8488942	8488943	MACS_peak_7335	6.00	lobChIP_FOXA1inPBS
chr19	8500395	8500396	MACS_peak_7336	6.00	lobChIP_FOXA1inPBS
chr19	8524881	8524882	MACS_peak_7337	17.00	lobChIP_FOXA1inPBS
chr19	8741121	8741122	MACS_peak_7338	25.00	lobChIP_FOXA1inPBS
chr19	9556914	9556915	MACS_peak_7339	13.00	lobChIP_FOXA1inPBS
chr19	9895585	9895586	MACS_peak_7340	14.00	lobChIP_FOXA1inPBS
chr19	10059474	10059475	MACS_peak_7341	11.00	lobChIP_FOXA1inPBS
chr19	10302189	10302190	MACS_peak_7342	7.00	lobChIP_FOXA1inPBS
chr19	10349290	10349291	MACS_peak_7343	16.00	lobChIP_FOXA1inPBS
chr19	10371911	10371912	MACS_peak_7344	7.00	lobChIP_FOXA1inPBS
chr19	10426792	10426793	MACS_peak_7345	13.00	lobChIP_FOXA1inPBS
chr19	10620392	10620393	MACS_peak_7346	6.00	lobChIP_FOXA1inPBS
chr19	10688084	10688085	MACS_peak_7347	10.00	lobChIP_FOXA1inPBS
chr19	11199487	11199488	MACS_peak_7348	16.00	lobChIP_FOXA1inPBS
chr19	11205967	11205968	MACS_peak_7349	22.00	lobChIP_FOXA1inPBS
chr19	11240528	11240529	MACS_peak_7350	11.00	lobChIP_FOXA1inPBS
chr19	11376597	11376598	MACS_peak_7351	17.00	lobChIP_FOXA1inPBS
chr19	11428543	11428544	MACS_peak_7352	12.00	lobChIP_FOXA1inPBS
chr19	11455201	11455202	MACS_peak_7353	16.00	lobChIP_FOXA1inPBS
chr19	11485992	11485993	MACS_peak_7354	7.00	lobChIP_FOXA1inPBS
chr19	11498239	11498240	MACS_peak_7355	6.00	lobChIP_FOXA1inPBS
chr19	11601776	11601777	MACS_peak_7356	11.00	lobChIP_FOXA1inPBS
chr19	11670167	11670168	MACS_peak_7357	10.00	lobChIP_FOXA1inPBS
chr19	11976105	11976106	MACS_peak_7358	9.00	lobChIP_FOXA1inPBS
chr19	12034564	12034565	MACS_peak_7359	16.00	lobChIP_FOXA1inPBS
chr19	12036539	12036540	MACS_peak_7360	8.00	lobChIP_FOXA1inPBS
chr19	12158889	12158890	MACS_peak_7361	10.00	lobChIP_FOXA1inPBS
chr19	12206308	12206309	MACS_peak_7362	8.00	lobChIP_FOXA1inPBS
chr19	12223339	12223340	MACS_peak_7363	11.00	lobChIP_FOXA1inPBS
chr19	12436749	12436750	MACS_peak_7364	35.00	lobChIP_FOXA1inPBS
chr19	12442210	12442211	MACS_peak_7365	13.00	lobChIP_FOXA1inPBS
chr19	12512270	12512271	MACS_peak_7366	10.00	lobChIP_FOXA1inPBS
chr19	12608132	12608133	MACS_peak_7367	9.00	lobChIP_FOXA1inPBS
chr19	12656224	12656225	MACS_peak_7368	8.00	lobChIP_FOXA1inPBS
chr19	12895012	12895013	MACS_peak_7369	14.00	lobChIP_FOXA1inPBS
chr19	13023667	13023668	MACS_peak_7370	6.00	lobChIP_FOXA1inPBS
chr19	13137540	13137541	MACS_peak_7371	33.00	lobChIP_FOXA1inPBS
chr19	13162589	13162590	MACS_peak_7372	20.00	lobChIP_FOXA1inPBS
chr19	13300654	13300655	MACS_peak_7373	8.00	lobChIP_FOXA1inPBS
chr19	13905854	13905855	MACS_peak_7374	6.00	lobChIP_FOXA1inPBS
chr19	14128860	14128861	MACS_peak_7375	8.00	lobChIP_FOXA1inPBS
chr19	14623992	14623993	MACS_peak_7376	14.00	lobChIP_FOXA1inPBS
chr19	15395664	15395665	MACS_peak_7377	10.00	lobChIP_FOXA1inPBS
chr19	15408389	15408390	MACS_peak_7378	31.00	lobChIP_FOXA1inPBS
chr19	15428389	15428390	MACS_peak_7379	9.00	lobChIP_FOXA1inPBS
chr19	15752090	15752091	MACS_peak_7380	14.00	lobChIP_FOXA1inPBS
chr19	15784211	15784212	MACS_peak_7381	25.00	lobChIP_FOXA1inPBS
chr19	16045364	16045365	MACS_peak_7382	9.00	lobChIP_FOXA1inPBS
chr19	16204655	16204656	MACS_peak_7383	19.00	lobChIP_FOXA1inPBS
chr19	16278067	16278068	MACS_peak_7384	7.00	lobChIP_FOXA1inPBS
chr19	16373538	16373539	MACS_peak_7385	8.00	lobChIP_FOXA1inPBS
chr19	16528746	16528747	MACS_peak_7386	7.00	lobChIP_FOXA1inPBS
chr19	16597544	16597545	MACS_peak_7387	6.00	lobChIP_FOXA1inPBS
chr19	16603852	16603853	MACS_peak_7388	13.00	lobChIP_FOXA1inPBS
chr19	16939978	16939979	MACS_peak_7389	8.00	lobChIP_FOXA1inPBS
chr19	17862140	17862141	MACS_peak_7390	12.00	lobChIP_FOXA1inPBS
chr19	17876103	17876104	MACS_peak_7391	8.00	lobChIP_FOXA1inPBS
chr19	17877895	17877896	MACS_peak_7392	9.00	lobChIP_FOXA1inPBS
chr19	17907577	17907578	MACS_peak_7393	7.00	lobChIP_FOXA1inPBS
chr19	17908613	17908614	MACS_peak_7394	8.00	lobChIP_FOXA1inPBS
chr19	18111428	18111429	MACS_peak_7395	11.00	lobChIP_FOXA1inPBS
chr19	18140521	18140522	MACS_peak_7396	14.00	lobChIP_FOXA1inPBS
chr19	18302839	18302840	MACS_peak_7397	6.00	lobChIP_FOXA1inPBS
chr19	18430755	18430756	MACS_peak_7398	8.00	lobChIP_FOXA1inPBS
chr19	18491060	18491061	MACS_peak_7399	35.00	lobChIP_FOXA1inPBS
chr19	18496857	18496858	MACS_peak_7400	15.00	lobChIP_FOXA1inPBS
chr19	18586755	18586756	MACS_peak_7401	13.00	lobChIP_FOXA1inPBS
chr19	18625891	18625892	MACS_peak_7402	24.00	lobChIP_FOXA1inPBS
chr19	18852093	18852094	MACS_peak_7403	8.00	lobChIP_FOXA1inPBS
chr19	19132085	19132086	MACS_peak_7404	8.00	lobChIP_FOXA1inPBS
chr19	19151407	19151408	MACS_peak_7405	14.00	lobChIP_FOXA1inPBS
chr19	19209644	19209645	MACS_peak_7406	7.00	lobChIP_FOXA1inPBS
chr19	19283004	19283005	MACS_peak_7407	10.00	lobChIP_FOXA1inPBS
chr19	21393058	21393059	MACS_peak_7408	75.00	lobChIP_FOXA1inPBS
chr19	23586511	23586512	MACS_peak_7410	15.00	lobChIP_FOXA1inPBS
chr19	23593126	23593127	MACS_peak_7411	12.00	lobChIP_FOXA1inPBS
chr19	24174193	24174194	MACS_peak_7412	7.00	lobChIP_FOXA1inPBS
chr19	24212873	24212874	MACS_peak_7413	6.00	lobChIP_FOXA1inPBS
chr19	24323037	24323038	MACS_peak_7415	11.00	lobChIP_FOXA1inPBS
chr19	24324468	24324469	MACS_peak_7416	7.00	lobChIP_FOXA1inPBS
chr19	30296278	30296279	MACS_peak_7417	6.00	lobChIP_FOXA1inPBS
chr19	30332583	30332584	MACS_peak_7418	11.00	lobChIP_FOXA1inPBS
chr19	30639087	30639088	MACS_peak_7419	6.00	lobChIP_FOXA1inPBS
chr19	32503569	32503570	MACS_peak_7420	8.00	lobChIP_FOXA1inPBS
chr19	32827438	32827439	MACS_peak_7421	16.00	lobChIP_FOXA1inPBS
chr19	32841572	32841573	MACS_peak_7422	10.00	lobChIP_FOXA1inPBS
chr19	32854238	32854239	MACS_peak_7423	8.00	lobChIP_FOXA1inPBS
chr19	32864902	32864903	MACS_peak_7424	38.00	lobChIP_FOXA1inPBS
chr19	33030425	33030426	MACS_peak_7425	20.00	lobChIP_FOXA1inPBS
chr19	33161079	33161080	MACS_peak_7426	16.00	lobChIP_FOXA1inPBS
chr19	33193161	33193162	MACS_peak_7427	73.00	lobChIP_FOXA1inPBS
chr19	33278538	33278539	MACS_peak_7428	11.00	lobChIP_FOXA1inPBS
chr19	33516585	33516586	MACS_peak_7429	13.00	lobChIP_FOXA1inPBS
chr19	33532345	33532346	MACS_peak_7430	13.00	lobChIP_FOXA1inPBS
chr19	33592235	33592236	MACS_peak_7431	14.00	lobChIP_FOXA1inPBS
chr19	33649452	33649453	MACS_peak_7432	13.00	lobChIP_FOXA1inPBS
chr19	33686385	33686386	MACS_peak_7433	9.00	lobChIP_FOXA1inPBS
chr19	33767137	33767138	MACS_peak_7434	31.00	lobChIP_FOXA1inPBS
chr19	33786220	33786221	MACS_peak_7435	9.00	lobChIP_FOXA1inPBS
chr19	33888255	33888256	MACS_peak_7436	24.00	lobChIP_FOXA1inPBS
chr19	33894715	33894716	MACS_peak_7437	24.00	lobChIP_FOXA1inPBS
chr19	34438007	34438008	MACS_peak_7438	8.00	lobChIP_FOXA1inPBS
chr19	34711567	34711568	MACS_peak_7439	20.00	lobChIP_FOXA1inPBS
chr19	34893150	34893151	MACS_peak_7440	24.00	lobChIP_FOXA1inPBS
chr19	35164642	35164643	MACS_peak_7441	8.00	lobChIP_FOXA1inPBS
chr19	35225374	35225375	MACS_peak_7442	8.00	lobChIP_FOXA1inPBS
chr19	35486012	35486013	MACS_peak_7443	63.00	lobChIP_FOXA1inPBS
chr19	35497457	35497458	MACS_peak_7444	79.00	lobChIP_FOXA1inPBS
chr19	35531887	35531888	MACS_peak_7445	7.00	lobChIP_FOXA1inPBS
chr19	35628403	35628404	MACS_peak_7446	14.00	lobChIP_FOXA1inPBS
chr19	35739661	35739662	MACS_peak_7447	14.00	lobChIP_FOXA1inPBS
chr19	35744857	35744858	MACS_peak_7448	20.00	lobChIP_FOXA1inPBS
chr19	35797213	35797214	MACS_peak_7449	9.00	lobChIP_FOXA1inPBS
chr19	35807649	35807650	MACS_peak_7450	17.00	lobChIP_FOXA1inPBS
chr19	35871470	35871471	MACS_peak_7451	8.00	lobChIP_FOXA1inPBS
chr19	35880263	35880264	MACS_peak_7452	7.00	lobChIP_FOXA1inPBS
chr19	35918200	35918201	MACS_peak_7453	10.00	lobChIP_FOXA1inPBS
chr19	35977256	35977257	MACS_peak_7454	5.00	lobChIP_FOXA1inPBS
chr19	36152538	36152539	MACS_peak_7455	8.00	lobChIP_FOXA1inPBS
chr19	36310969	36310970	MACS_peak_7456	18.00	lobChIP_FOXA1inPBS
chr19	36543683	36543684	MACS_peak_7457	5.00	lobChIP_FOXA1inPBS
chr19	36611097	36611098	MACS_peak_7458	17.00	lobChIP_FOXA1inPBS
chr19	37062866	37062867	MACS_peak_7459	9.00	lobChIP_FOXA1inPBS
chr19	37328535	37328536	MACS_peak_7460	11.00	lobChIP_FOXA1inPBS
chr19	37663705	37663706	MACS_peak_7461	10.00	lobChIP_FOXA1inPBS
chr19	38147009	38147010	MACS_peak_7462	8.00	lobChIP_FOXA1inPBS
chr19	38365586	38365587	MACS_peak_7463	6.00	lobChIP_FOXA1inPBS
chr19	38432289	38432290	MACS_peak_7464	9.00	lobChIP_FOXA1inPBS
chr19	38432741	38432742	MACS_peak_7465	7.00	lobChIP_FOXA1inPBS
chr19	38548853	38548854	MACS_peak_7466	10.00	lobChIP_FOXA1inPBS
chr19	38570835	38570836	MACS_peak_7467	11.00	lobChIP_FOXA1inPBS
chr19	38741516	38741517	MACS_peak_7468	19.00	lobChIP_FOXA1inPBS
chr19	39142882	39142883	MACS_peak_7469	26.00	lobChIP_FOXA1inPBS
chr19	39144084	39144085	MACS_peak_7470	13.00	lobChIP_FOXA1inPBS
chr19	39149465	39149466	MACS_peak_7471	13.00	lobChIP_FOXA1inPBS
chr19	39150377	39150378	MACS_peak_7472	7.00	lobChIP_FOXA1inPBS
chr19	39322739	39322740	MACS_peak_7473	15.00	lobChIP_FOXA1inPBS
chr19	39328574	39328575	MACS_peak_7474	15.00	lobChIP_FOXA1inPBS
chr19	39391579	39391580	MACS_peak_7475	7.00	lobChIP_FOXA1inPBS
chr19	39425252	39425253	MACS_peak_7476	11.00	lobChIP_FOXA1inPBS
chr19	39455852	39455853	MACS_peak_7477	7.00	lobChIP_FOXA1inPBS
chr19	40168786	40168787	MACS_peak_7478	11.00	lobChIP_FOXA1inPBS
chr19	40186592	40186593	MACS_peak_7479	21.00	lobChIP_FOXA1inPBS
chr19	40333183	40333184	MACS_peak_7480	14.00	lobChIP_FOXA1inPBS
chr19	40341722	40341723	MACS_peak_7481	5.00	lobChIP_FOXA1inPBS
chr19	40450178	40450179	MACS_peak_7482	13.00	lobChIP_FOXA1inPBS
chr19	40591161	40591162	MACS_peak_7483	10.00	lobChIP_FOXA1inPBS
chr19	40616693	40616694	MACS_peak_7484	9.00	lobChIP_FOXA1inPBS
chr19	40625497	40625498	MACS_peak_7485	7.00	lobChIP_FOXA1inPBS
chr19	40732897	40732898	MACS_peak_7486	15.00	lobChIP_FOXA1inPBS
chr19	40759791	40759792	MACS_peak_7487	7.00	lobChIP_FOXA1inPBS
chr19	40789691	40789692	MACS_peak_7488	9.00	lobChIP_FOXA1inPBS
chr19	41109836	41109837	MACS_peak_7489	20.00	lobChIP_FOXA1inPBS
chr19	41427300	41427301	MACS_peak_7490	36.00	lobChIP_FOXA1inPBS
chr19	41532489	41532490	MACS_peak_7491	7.00	lobChIP_FOXA1inPBS
chr19	41632769	41632770	MACS_peak_7492	14.00	lobChIP_FOXA1inPBS
chr19	41673176	41673177	MACS_peak_7493	9.00	lobChIP_FOXA1inPBS
chr19	41829602	41829603	MACS_peak_7494	16.00	lobChIP_FOXA1inPBS
chr19	41842821	41842822	MACS_peak_7495	7.00	lobChIP_FOXA1inPBS
chr19	42101898	42101899	MACS_peak_7496	8.00	lobChIP_FOXA1inPBS
chr19	42303543	42303544	MACS_peak_7497	13.00	lobChIP_FOXA1inPBS
chr19	42392050	42392051	MACS_peak_7498	20.00	lobChIP_FOXA1inPBS
chr19	42408764	42408765	MACS_peak_7499	79.00	lobChIP_FOXA1inPBS
chr19	42800484	42800485	MACS_peak_7500	8.00	lobChIP_FOXA1inPBS
chr19	42937931	42937932	MACS_peak_7501	8.00	lobChIP_FOXA1inPBS
chr19	42948208	42948209	MACS_peak_7502	27.00	lobChIP_FOXA1inPBS
chr19	42985106	42985107	MACS_peak_7503	37.00	lobChIP_FOXA1inPBS
chr19	42993344	42993345	MACS_peak_7504	36.00	lobChIP_FOXA1inPBS
chr19	43002213	43002214	MACS_peak_7505	12.00	lobChIP_FOXA1inPBS
chr19	44462269	44462270	MACS_peak_7506	6.00	lobChIP_FOXA1inPBS
chr19	45115352	45115353	MACS_peak_7507	9.00	lobChIP_FOXA1inPBS
chr19	45119383	45119384	MACS_peak_7508	14.00	lobChIP_FOXA1inPBS
chr19	45140168	45140169	MACS_peak_7509	10.00	lobChIP_FOXA1inPBS
chr19	45198229	45198230	MACS_peak_7510	56.00	lobChIP_FOXA1inPBS
chr19	45198825	45198826	MACS_peak_7511	13.00	lobChIP_FOXA1inPBS
chr19	45222054	45222055	MACS_peak_7512	9.00	lobChIP_FOXA1inPBS
chr19	45247018	45247019	MACS_peak_7513	13.00	lobChIP_FOXA1inPBS
chr19	45300114	45300115	MACS_peak_7514	31.00	lobChIP_FOXA1inPBS
chr19	45360159	45360160	MACS_peak_7515	9.00	lobChIP_FOXA1inPBS
chr19	45362508	45362509	MACS_peak_7516	15.00	lobChIP_FOXA1inPBS
chr19	45427656	45427657	MACS_peak_7517	32.00	lobChIP_FOXA1inPBS
chr19	45439285	45439286	MACS_peak_7518	9.00	lobChIP_FOXA1inPBS
chr19	45443036	45443037	MACS_peak_7519	11.00	lobChIP_FOXA1inPBS
chr19	45549075	45549076	MACS_peak_7520	37.00	lobChIP_FOXA1inPBS
chr19	45657467	45657468	MACS_peak_7521	7.00	lobChIP_FOXA1inPBS
chr19	45776467	45776468	MACS_peak_7522	40.00	lobChIP_FOXA1inPBS
chr19	45779758	45779759	MACS_peak_7523	12.00	lobChIP_FOXA1inPBS
chr19	46045676	46045677	MACS_peak_7524	8.00	lobChIP_FOXA1inPBS
chr19	46087340	46087341	MACS_peak_7525	6.00	lobChIP_FOXA1inPBS
chr19	46143286	46143287	MACS_peak_7526	7.00	lobChIP_FOXA1inPBS
chr19	46161302	46161303	MACS_peak_7527	9.00	lobChIP_FOXA1inPBS
chr19	46385387	46385388	MACS_peak_7528	11.00	lobChIP_FOXA1inPBS
chr19	46571736	46571737	MACS_peak_7529	13.00	lobChIP_FOXA1inPBS
chr19	46696601	46696602	MACS_peak_7530	38.00	lobChIP_FOXA1inPBS
chr19	47251973	47251974	MACS_peak_7531	8.00	lobChIP_FOXA1inPBS
chr19	47274363	47274364	MACS_peak_7532	28.00	lobChIP_FOXA1inPBS
chr19	47394177	47394178	MACS_peak_7533	23.00	lobChIP_FOXA1inPBS
chr19	47457920	47457921	MACS_peak_7534	10.00	lobChIP_FOXA1inPBS
chr19	47487939	47487940	MACS_peak_7535	46.00	lobChIP_FOXA1inPBS
chr19	47539600	47539601	MACS_peak_7536	9.00	lobChIP_FOXA1inPBS
chr19	47599535	47599536	MACS_peak_7537	19.00	lobChIP_FOXA1inPBS
chr19	47625871	47625872	MACS_peak_7538	5.00	lobChIP_FOXA1inPBS
chr19	47628441	47628442	MACS_peak_7539	9.00	lobChIP_FOXA1inPBS
chr19	47689631	47689632	MACS_peak_7540	9.00	lobChIP_FOXA1inPBS
chr19	47693673	47693674	MACS_peak_7541	15.00	lobChIP_FOXA1inPBS
chr19	48151679	48151680	MACS_peak_7542	14.00	lobChIP_FOXA1inPBS
chr19	48161069	48161070	MACS_peak_7543	9.00	lobChIP_FOXA1inPBS
chr19	48166251	48166252	MACS_peak_7544	12.00	lobChIP_FOXA1inPBS
chr19	48232214	48232215	MACS_peak_7545	7.00	lobChIP_FOXA1inPBS
chr19	48395550	48395551	MACS_peak_7546	6.00	lobChIP_FOXA1inPBS
chr19	48706733	48706734	MACS_peak_7547	5.00	lobChIP_FOXA1inPBS
chr19	48760021	48760022	MACS_peak_7548	16.00	lobChIP_FOXA1inPBS
chr19	48857955	48857956	MACS_peak_7549	10.00	lobChIP_FOXA1inPBS
chr19	48990099	48990100	MACS_peak_7550	22.00	lobChIP_FOXA1inPBS
chr19	49061773	49061774	MACS_peak_7551	7.00	lobChIP_FOXA1inPBS
chr19	49068579	49068580	MACS_peak_7552	23.00	lobChIP_FOXA1inPBS
chr19	49074886	49074887	MACS_peak_7553	14.00	lobChIP_FOXA1inPBS
chr19	49078193	49078194	MACS_peak_7554	10.00	lobChIP_FOXA1inPBS
chr19	49135221	49135222	MACS_peak_7555	8.00	lobChIP_FOXA1inPBS
chr19	49272702	49272703	MACS_peak_7556	14.00	lobChIP_FOXA1inPBS
chr19	49275238	49275239	MACS_peak_7557	16.00	lobChIP_FOXA1inPBS
chr19	49376368	49376369	MACS_peak_7558	13.00	lobChIP_FOXA1inPBS
chr19	49673816	49673817	MACS_peak_7559	9.00	lobChIP_FOXA1inPBS
chr19	49688955	49688956	MACS_peak_7560	9.00	lobChIP_FOXA1inPBS
chr19	49955078	49955079	MACS_peak_7561	8.00	lobChIP_FOXA1inPBS
chr19	49996103	49996104	MACS_peak_7562	14.00	lobChIP_FOXA1inPBS
chr19	50073699	50073700	MACS_peak_7563	9.00	lobChIP_FOXA1inPBS
chr19	50737806	50737807	MACS_peak_7564	44.00	lobChIP_FOXA1inPBS
chr19	51858283	51858284	MACS_peak_7565	13.00	lobChIP_FOXA1inPBS
chr19	51866178	51866179	MACS_peak_7566	43.00	lobChIP_FOXA1inPBS
chr19	51934584	51934585	MACS_peak_7567	13.00	lobChIP_FOXA1inPBS
chr19	51978553	51978554	MACS_peak_7568	20.00	lobChIP_FOXA1inPBS
chr19	52464335	52464336	MACS_peak_7569	10.00	lobChIP_FOXA1inPBS
chr19	52514152	52514153	MACS_peak_7570	10.00	lobChIP_FOXA1inPBS
chr19	52527934	52527935	MACS_peak_7571	6.00	lobChIP_FOXA1inPBS
chr19	52529595	52529596	MACS_peak_7572	10.00	lobChIP_FOXA1inPBS
chr19	53161962	53161963	MACS_peak_7573	5.00	lobChIP_FOXA1inPBS
chr19	53206161	53206162	MACS_peak_7574	8.00	lobChIP_FOXA1inPBS
chr19	53448637	53448638	MACS_peak_7575	11.00	lobChIP_FOXA1inPBS
chr19	53923974	53923975	MACS_peak_7576	9.00	lobChIP_FOXA1inPBS
chr19	53934791	53934792	MACS_peak_7577	7.00	lobChIP_FOXA1inPBS
chr19	54000787	54000788	MACS_peak_7578	26.00	lobChIP_FOXA1inPBS
chr19	54038953	54038954	MACS_peak_7579	16.00	lobChIP_FOXA1inPBS
chr19	54123237	54123238	MACS_peak_7580	24.00	lobChIP_FOXA1inPBS
chr19	54164555	54164556	MACS_peak_7581	8.00	lobChIP_FOXA1inPBS
chr19	54279008	54279009	MACS_peak_7582	23.00	lobChIP_FOXA1inPBS
chr19	54312085	54312086	MACS_peak_7583	11.00	lobChIP_FOXA1inPBS
chr19	54367855	54367856	MACS_peak_7584	70.00	lobChIP_FOXA1inPBS
chr19	54523618	54523619	MACS_peak_7585	8.00	lobChIP_FOXA1inPBS
chr19	54713985	54713986	MACS_peak_7586	8.00	lobChIP_FOXA1inPBS
chr19	55185693	55185694	MACS_peak_7587	53.00	lobChIP_FOXA1inPBS
chr19	55721619	55721620	MACS_peak_7588	15.00	lobChIP_FOXA1inPBS
chr19	55803463	55803464	MACS_peak_7589	9.00	lobChIP_FOXA1inPBS
chr19	55850237	55850238	MACS_peak_7590	7.00	lobChIP_FOXA1inPBS
chr19	56060856	56060857	MACS_peak_7591	50.00	lobChIP_FOXA1inPBS
chr19	56064832	56064833	MACS_peak_7592	9.00	lobChIP_FOXA1inPBS
chr19	56093239	56093240	MACS_peak_7593	9.00	lobChIP_FOXA1inPBS
chr19	56467976	56467977	MACS_peak_7594	12.00	lobChIP_FOXA1inPBS
chr19	56649633	56649634	MACS_peak_7595	10.00	lobChIP_FOXA1inPBS
chr19	56653717	56653718	MACS_peak_7596	18.00	lobChIP_FOXA1inPBS
chr19	56867032	56867033	MACS_peak_7597	18.00	lobChIP_FOXA1inPBS
chr19	57412116	57412117	MACS_peak_7598	15.00	lobChIP_FOXA1inPBS
chr19	57430188	57430189	MACS_peak_7599	24.00	lobChIP_FOXA1inPBS
chr19	57438865	57438866	MACS_peak_7600	13.00	lobChIP_FOXA1inPBS
chr19	57442485	57442486	MACS_peak_7601	25.00	lobChIP_FOXA1inPBS
chr19	57446287	57446288	MACS_peak_7602	43.00	lobChIP_FOXA1inPBS
chr19	57467214	57467215	MACS_peak_7603	9.00	lobChIP_FOXA1inPBS
chr19	57487937	57487938	MACS_peak_7604	17.00	lobChIP_FOXA1inPBS
chr19	57538839	57538840	MACS_peak_7605	5.00	lobChIP_FOXA1inPBS
chr19	58290105	58290106	MACS_peak_7606	6.00	lobChIP_FOXA1inPBS
chr19	58335204	58335205	MACS_peak_7607	10.00	lobChIP_FOXA1inPBS
chr19	58385988	58385989	MACS_peak_7608	11.00	lobChIP_FOXA1inPBS
chr19	58662550	58662551	MACS_peak_7609	6.00	lobChIP_FOXA1inPBS
chr19	58665050	58665051	MACS_peak_7610	7.00	lobChIP_FOXA1inPBS
chr2	176200	176201	MACS_peak_7611	21.00	lobChIP_FOXA1inPBS
chr2	246849	246850	MACS_peak_7612	13.00	lobChIP_FOXA1inPBS
chr2	249940	249941	MACS_peak_7613	17.00	lobChIP_FOXA1inPBS
chr2	260867	260868	MACS_peak_7614	84.00	lobChIP_FOXA1inPBS
chr2	378554	378555	MACS_peak_7615	18.00	lobChIP_FOXA1inPBS
chr2	843879	843880	MACS_peak_7616	10.00	lobChIP_FOXA1inPBS
chr2	970920	970921	MACS_peak_7617	12.00	lobChIP_FOXA1inPBS
chr2	1037317	1037318	MACS_peak_7618	9.00	lobChIP_FOXA1inPBS
chr2	3048868	3048869	MACS_peak_7619	14.00	lobChIP_FOXA1inPBS
chr2	3384173	3384174	MACS_peak_7620	20.00	lobChIP_FOXA1inPBS
chr2	3522219	3522220	MACS_peak_7621	8.00	lobChIP_FOXA1inPBS
chr2	3527519	3527520	MACS_peak_7622	10.00	lobChIP_FOXA1inPBS
chr2	3633446	3633447	MACS_peak_7623	19.00	lobChIP_FOXA1inPBS
chr2	3668724	3668725	MACS_peak_7624	20.00	lobChIP_FOXA1inPBS
chr2	3693505	3693506	MACS_peak_7625	12.00	lobChIP_FOXA1inPBS
chr2	3704746	3704747	MACS_peak_7626	19.00	lobChIP_FOXA1inPBS
chr2	3740166	3740167	MACS_peak_7627	6.00	lobChIP_FOXA1inPBS
chr2	4306309	4306310	MACS_peak_7628	15.00	lobChIP_FOXA1inPBS
chr2	7058958	7058959	MACS_peak_7629	12.00	lobChIP_FOXA1inPBS
chr2	7062594	7062595	MACS_peak_7630	11.00	lobChIP_FOXA1inPBS
chr2	7095048	7095049	MACS_peak_7631	6.00	lobChIP_FOXA1inPBS
chr2	7172075	7172076	MACS_peak_7632	20.00	lobChIP_FOXA1inPBS
chr2	8234329	8234330	MACS_peak_7633	10.00	lobChIP_FOXA1inPBS
chr2	8243435	8243436	MACS_peak_7634	44.00	lobChIP_FOXA1inPBS
chr2	8259963	8259964	MACS_peak_7635	12.00	lobChIP_FOXA1inPBS
chr2	8266185	8266186	MACS_peak_7636	12.00	lobChIP_FOXA1inPBS
chr2	8279374	8279375	MACS_peak_7637	56.00	lobChIP_FOXA1inPBS
chr2	8504992	8504993	MACS_peak_7638	11.00	lobChIP_FOXA1inPBS
chr2	8554104	8554105	MACS_peak_7639	14.00	lobChIP_FOXA1inPBS
chr2	8633661	8633662	MACS_peak_7640	11.00	lobChIP_FOXA1inPBS
chr2	8640873	8640874	MACS_peak_7641	23.00	lobChIP_FOXA1inPBS
chr2	8712258	8712259	MACS_peak_7642	12.00	lobChIP_FOXA1inPBS
chr2	8751761	8751762	MACS_peak_7643	8.00	lobChIP_FOXA1inPBS
chr2	8753158	8753159	MACS_peak_7644	14.00	lobChIP_FOXA1inPBS
chr2	8765678	8765679	MACS_peak_7645	29.00	lobChIP_FOXA1inPBS
chr2	8767399	8767400	MACS_peak_7646	10.00	lobChIP_FOXA1inPBS
chr2	8796591	8796592	MACS_peak_7647	43.00	lobChIP_FOXA1inPBS
chr2	8817820	8817821	MACS_peak_7648	26.00	lobChIP_FOXA1inPBS
chr2	8820757	8820758	MACS_peak_7649	23.00	lobChIP_FOXA1inPBS
chr2	8944380	8944381	MACS_peak_7650	9.00	lobChIP_FOXA1inPBS
chr2	8960567	8960568	MACS_peak_7651	12.00	lobChIP_FOXA1inPBS
chr2	8980865	8980866	MACS_peak_7652	27.00	lobChIP_FOXA1inPBS
chr2	9308413	9308414	MACS_peak_7653	20.00	lobChIP_FOXA1inPBS
chr2	9373399	9373400	MACS_peak_7654	7.00	lobChIP_FOXA1inPBS
chr2	9466810	9466811	MACS_peak_7655	8.00	lobChIP_FOXA1inPBS
chr2	9468147	9468148	MACS_peak_7656	16.00	lobChIP_FOXA1inPBS
chr2	9477140	9477141	MACS_peak_7657	17.00	lobChIP_FOXA1inPBS
chr2	9478102	9478103	MACS_peak_7658	8.00	lobChIP_FOXA1inPBS
chr2	9748674	9748675	MACS_peak_7659	9.00	lobChIP_FOXA1inPBS
chr2	9797337	9797338	MACS_peak_7660	10.00	lobChIP_FOXA1inPBS
chr2	10029981	10029982	MACS_peak_7661	11.00	lobChIP_FOXA1inPBS
chr2	10036059	10036060	MACS_peak_7662	8.00	lobChIP_FOXA1inPBS
chr2	10159406	10159407	MACS_peak_7663	10.00	lobChIP_FOXA1inPBS
chr2	10260604	10260605	MACS_peak_7664	12.00	lobChIP_FOXA1inPBS
chr2	10276633	10276634	MACS_peak_7665	18.00	lobChIP_FOXA1inPBS
chr2	10467359	10467360	MACS_peak_7666	13.00	lobChIP_FOXA1inPBS
chr2	10506170	10506171	MACS_peak_7667	10.00	lobChIP_FOXA1inPBS
chr2	10508774	10508775	MACS_peak_7668	13.00	lobChIP_FOXA1inPBS
chr2	10592558	10592559	MACS_peak_7669	12.00	lobChIP_FOXA1inPBS
chr2	10627450	10627451	MACS_peak_7670	55.00	lobChIP_FOXA1inPBS
chr2	10724806	10724807	MACS_peak_7671	149.00	lobChIP_FOXA1inPBS
chr2	10939950	10939951	MACS_peak_7672	7.00	lobChIP_FOXA1inPBS
chr2	11076479	11076480	MACS_peak_7673	18.00	lobChIP_FOXA1inPBS
chr2	11274721	11274722	MACS_peak_7674	8.00	lobChIP_FOXA1inPBS
chr2	11394339	11394340	MACS_peak_7675	31.00	lobChIP_FOXA1inPBS
chr2	11480919	11480920	MACS_peak_7676	8.00	lobChIP_FOXA1inPBS
chr2	11482981	11482982	MACS_peak_7677	11.00	lobChIP_FOXA1inPBS
chr2	11494625	11494626	MACS_peak_7678	101.00	lobChIP_FOXA1inPBS
chr2	11497639	11497640	MACS_peak_7679	10.00	lobChIP_FOXA1inPBS
chr2	11506540	11506541	MACS_peak_7680	31.00	lobChIP_FOXA1inPBS
chr2	11520922	11520923	MACS_peak_7681	10.00	lobChIP_FOXA1inPBS
chr2	11528141	11528142	MACS_peak_7682	75.00	lobChIP_FOXA1inPBS
chr2	11607137	11607138	MACS_peak_7683	14.00	lobChIP_FOXA1inPBS
chr2	11639620	11639621	MACS_peak_7684	20.00	lobChIP_FOXA1inPBS
chr2	11646058	11646059	MACS_peak_7685	17.00	lobChIP_FOXA1inPBS
chr2	11654396	11654397	MACS_peak_7686	16.00	lobChIP_FOXA1inPBS
chr2	11676000	11676001	MACS_peak_7687	11.00	lobChIP_FOXA1inPBS
chr2	11680284	11680285	MACS_peak_7688	11.00	lobChIP_FOXA1inPBS
chr2	11684271	11684272	MACS_peak_7689	7.00	lobChIP_FOXA1inPBS
chr2	11689897	11689898	MACS_peak_7690	13.00	lobChIP_FOXA1inPBS
chr2	11723559	11723560	MACS_peak_7691	10.00	lobChIP_FOXA1inPBS
chr2	11863043	11863044	MACS_peak_7692	20.00	lobChIP_FOXA1inPBS
chr2	11959594	11959595	MACS_peak_7693	13.00	lobChIP_FOXA1inPBS
chr2	11970878	11970879	MACS_peak_7694	20.00	lobChIP_FOXA1inPBS
chr2	12012963	12012964	MACS_peak_7695	11.00	lobChIP_FOXA1inPBS
chr2	12596734	12596735	MACS_peak_7696	11.00	lobChIP_FOXA1inPBS
chr2	12655075	12655076	MACS_peak_7697	16.00	lobChIP_FOXA1inPBS
chr2	13885638	13885639	MACS_peak_7698	8.00	lobChIP_FOXA1inPBS
chr2	15313129	15313130	MACS_peak_7699	20.00	lobChIP_FOXA1inPBS
chr2	15314907	15314908	MACS_peak_7700	11.00	lobChIP_FOXA1inPBS
chr2	15500510	15500511	MACS_peak_7701	26.00	lobChIP_FOXA1inPBS
chr2	15769425	15769426	MACS_peak_7702	11.00	lobChIP_FOXA1inPBS
chr2	15781921	15781922	MACS_peak_7703	12.00	lobChIP_FOXA1inPBS
chr2	15804667	15804668	MACS_peak_7704	7.00	lobChIP_FOXA1inPBS
chr2	15906671	15906672	MACS_peak_7705	64.00	lobChIP_FOXA1inPBS
chr2	15916831	15916832	MACS_peak_7706	30.00	lobChIP_FOXA1inPBS
chr2	15922526	15922527	MACS_peak_7707	25.00	lobChIP_FOXA1inPBS
chr2	16191379	16191380	MACS_peak_7708	9.00	lobChIP_FOXA1inPBS
chr2	16208489	16208490	MACS_peak_7709	30.00	lobChIP_FOXA1inPBS
chr2	16210579	16210580	MACS_peak_7710	40.00	lobChIP_FOXA1inPBS
chr2	16244609	16244610	MACS_peak_7711	30.00	lobChIP_FOXA1inPBS
chr2	16574688	16574689	MACS_peak_7712	13.00	lobChIP_FOXA1inPBS
chr2	16607185	16607186	MACS_peak_7713	48.00	lobChIP_FOXA1inPBS
chr2	16646069	16646070	MACS_peak_7714	7.00	lobChIP_FOXA1inPBS
chr2	16999317	16999318	MACS_peak_7715	13.00	lobChIP_FOXA1inPBS
chr2	17390038	17390039	MACS_peak_7716	13.00	lobChIP_FOXA1inPBS
chr2	17487734	17487735	MACS_peak_7717	9.00	lobChIP_FOXA1inPBS
chr2	17661270	17661271	MACS_peak_7718	28.00	lobChIP_FOXA1inPBS
chr2	17727682	17727683	MACS_peak_7719	27.00	lobChIP_FOXA1inPBS
chr2	17760136	17760137	MACS_peak_7720	10.00	lobChIP_FOXA1inPBS
chr2	17775331	17775332	MACS_peak_7721	28.00	lobChIP_FOXA1inPBS
chr2	17946940	17946941	MACS_peak_7722	11.00	lobChIP_FOXA1inPBS
chr2	17949817	17949818	MACS_peak_7723	8.00	lobChIP_FOXA1inPBS
chr2	18933411	18933412	MACS_peak_7724	6.00	lobChIP_FOXA1inPBS
chr2	18944221	18944222	MACS_peak_7725	14.00	lobChIP_FOXA1inPBS
chr2	18958648	18958649	MACS_peak_7726	13.00	lobChIP_FOXA1inPBS
chr2	18981841	18981842	MACS_peak_7727	182.00	lobChIP_FOXA1inPBS
chr2	19005859	19005860	MACS_peak_7728	6.00	lobChIP_FOXA1inPBS
chr2	19012249	19012250	MACS_peak_7729	6.00	lobChIP_FOXA1inPBS
chr2	19071048	19071049	MACS_peak_7730	48.00	lobChIP_FOXA1inPBS
chr2	19152166	19152167	MACS_peak_7731	17.00	lobChIP_FOXA1inPBS
chr2	19248791	19248792	MACS_peak_7732	24.00	lobChIP_FOXA1inPBS
chr2	19340697	19340698	MACS_peak_7733	24.00	lobChIP_FOXA1inPBS
chr2	19705476	19705477	MACS_peak_7734	48.00	lobChIP_FOXA1inPBS
chr2	19769200	19769201	MACS_peak_7735	16.00	lobChIP_FOXA1inPBS
chr2	19780745	19780746	MACS_peak_7736	10.00	lobChIP_FOXA1inPBS
chr2	20100083	20100084	MACS_peak_7737	21.00	lobChIP_FOXA1inPBS
chr2	20205279	20205280	MACS_peak_7738	16.00	lobChIP_FOXA1inPBS
chr2	20208630	20208631	MACS_peak_7739	29.00	lobChIP_FOXA1inPBS
chr2	20272058	20272059	MACS_peak_7740	7.00	lobChIP_FOXA1inPBS
chr2	20298185	20298186	MACS_peak_7741	7.00	lobChIP_FOXA1inPBS
chr2	20308503	20308504	MACS_peak_7742	9.00	lobChIP_FOXA1inPBS
chr2	20384023	20384024	MACS_peak_7743	11.00	lobChIP_FOXA1inPBS
chr2	20408140	20408141	MACS_peak_7744	14.00	lobChIP_FOXA1inPBS
chr2	20415198	20415199	MACS_peak_7745	12.00	lobChIP_FOXA1inPBS
chr2	20551483	20551484	MACS_peak_7746	15.00	lobChIP_FOXA1inPBS
chr2	20553678	20553679	MACS_peak_7747	17.00	lobChIP_FOXA1inPBS
chr2	20578332	20578333	MACS_peak_7748	20.00	lobChIP_FOXA1inPBS
chr2	20611338	20611339	MACS_peak_7749	17.00	lobChIP_FOXA1inPBS
chr2	20617226	20617227	MACS_peak_7750	10.00	lobChIP_FOXA1inPBS
chr2	20638500	20638501	MACS_peak_7751	18.00	lobChIP_FOXA1inPBS
chr2	20649658	20649659	MACS_peak_7752	12.00	lobChIP_FOXA1inPBS
chr2	20658694	20658695	MACS_peak_7753	12.00	lobChIP_FOXA1inPBS
chr2	20680759	20680760	MACS_peak_7754	9.00	lobChIP_FOXA1inPBS
chr2	20728264	20728265	MACS_peak_7755	11.00	lobChIP_FOXA1inPBS
chr2	20776844	20776845	MACS_peak_7756	5.00	lobChIP_FOXA1inPBS
chr2	20780587	20780588	MACS_peak_7757	31.00	lobChIP_FOXA1inPBS
chr2	20785378	20785379	MACS_peak_7758	11.00	lobChIP_FOXA1inPBS
chr2	20793353	20793354	MACS_peak_7759	51.00	lobChIP_FOXA1inPBS
chr2	20796933	20796934	MACS_peak_7760	17.00	lobChIP_FOXA1inPBS
chr2	20811307	20811308	MACS_peak_7761	18.00	lobChIP_FOXA1inPBS
chr2	20917551	20917552	MACS_peak_7762	7.00	lobChIP_FOXA1inPBS
chr2	20928014	20928015	MACS_peak_7763	16.00	lobChIP_FOXA1inPBS
chr2	21135321	21135322	MACS_peak_7764	13.00	lobChIP_FOXA1inPBS
chr2	21251967	21251968	MACS_peak_7765	14.00	lobChIP_FOXA1inPBS
chr2	21261645	21261646	MACS_peak_7766	27.00	lobChIP_FOXA1inPBS
chr2	21265087	21265088	MACS_peak_7767	25.00	lobChIP_FOXA1inPBS
chr2	21266002	21266003	MACS_peak_7768	33.00	lobChIP_FOXA1inPBS
chr2	21266997	21266998	MACS_peak_7769	12.00	lobChIP_FOXA1inPBS
chr2	21270129	21270130	MACS_peak_7770	35.00	lobChIP_FOXA1inPBS
chr2	21279349	21279350	MACS_peak_7771	59.00	lobChIP_FOXA1inPBS
chr2	21290193	21290194	MACS_peak_7772	8.00	lobChIP_FOXA1inPBS
chr2	21293378	21293379	MACS_peak_7773	62.00	lobChIP_FOXA1inPBS
chr2	21326606	21326607	MACS_peak_7774	25.00	lobChIP_FOXA1inPBS
chr2	21378826	21378827	MACS_peak_7775	13.00	lobChIP_FOXA1inPBS
chr2	21723683	21723684	MACS_peak_7776	6.00	lobChIP_FOXA1inPBS
chr2	22278892	22278893	MACS_peak_7777	41.00	lobChIP_FOXA1inPBS
chr2	23887416	23887417	MACS_peak_7778	12.00	lobChIP_FOXA1inPBS
chr2	24186851	24186852	MACS_peak_7779	8.00	lobChIP_FOXA1inPBS
chr2	24238580	24238581	MACS_peak_7780	8.00	lobChIP_FOXA1inPBS
chr2	24274448	24274449	MACS_peak_7781	18.00	lobChIP_FOXA1inPBS
chr2	24275961	24275962	MACS_peak_7782	9.00	lobChIP_FOXA1inPBS
chr2	24308225	24308226	MACS_peak_7783	7.00	lobChIP_FOXA1inPBS
chr2	24318431	24318432	MACS_peak_7784	11.00	lobChIP_FOXA1inPBS
chr2	24381221	24381222	MACS_peak_7785	18.00	lobChIP_FOXA1inPBS
chr2	24461202	24461203	MACS_peak_7786	10.00	lobChIP_FOXA1inPBS
chr2	25198492	25198493	MACS_peak_7787	7.00	lobChIP_FOXA1inPBS
chr2	25745113	25745114	MACS_peak_7788	29.00	lobChIP_FOXA1inPBS
chr2	25841819	25841820	MACS_peak_7789	17.00	lobChIP_FOXA1inPBS
chr2	25895486	25895487	MACS_peak_7790	7.00	lobChIP_FOXA1inPBS
chr2	26010040	26010041	MACS_peak_7791	34.00	lobChIP_FOXA1inPBS
chr2	26031656	26031657	MACS_peak_7792	47.00	lobChIP_FOXA1inPBS
chr2	26040056	26040057	MACS_peak_7793	6.00	lobChIP_FOXA1inPBS
chr2	26046527	26046528	MACS_peak_7794	8.00	lobChIP_FOXA1inPBS
chr2	26052382	26052383	MACS_peak_7795	14.00	lobChIP_FOXA1inPBS
chr2	26074895	26074896	MACS_peak_7796	18.00	lobChIP_FOXA1inPBS
chr2	26102098	26102099	MACS_peak_7797	21.00	lobChIP_FOXA1inPBS
chr2	26106268	26106269	MACS_peak_7798	7.00	lobChIP_FOXA1inPBS
chr2	26108965	26108966	MACS_peak_7799	10.00	lobChIP_FOXA1inPBS
chr2	26220791	26220792	MACS_peak_7800	7.00	lobChIP_FOXA1inPBS
chr2	26235250	26235251	MACS_peak_7801	17.00	lobChIP_FOXA1inPBS
chr2	26259069	26259070	MACS_peak_7802	5.00	lobChIP_FOXA1inPBS
chr2	26296520	26296521	MACS_peak_7803	9.00	lobChIP_FOXA1inPBS
chr2	26299241	26299242	MACS_peak_7804	13.00	lobChIP_FOXA1inPBS
chr2	26303425	26303426	MACS_peak_7805	39.00	lobChIP_FOXA1inPBS
chr2	26461560	26461561	MACS_peak_7806	23.00	lobChIP_FOXA1inPBS
chr2	26854006	26854007	MACS_peak_7807	19.00	lobChIP_FOXA1inPBS
chr2	26944737	26944738	MACS_peak_7808	33.00	lobChIP_FOXA1inPBS
chr2	26995490	26995491	MACS_peak_7809	8.00	lobChIP_FOXA1inPBS
chr2	27148290	27148291	MACS_peak_7810	7.00	lobChIP_FOXA1inPBS
chr2	27318765	27318766	MACS_peak_7811	8.00	lobChIP_FOXA1inPBS
chr2	27394568	27394569	MACS_peak_7812	15.00	lobChIP_FOXA1inPBS
chr2	27400662	27400663	MACS_peak_7813	23.00	lobChIP_FOXA1inPBS
chr2	27403631	27403632	MACS_peak_7814	8.00	lobChIP_FOXA1inPBS
chr2	27417926	27417927	MACS_peak_7815	8.00	lobChIP_FOXA1inPBS
chr2	27516119	27516120	MACS_peak_7816	22.00	lobChIP_FOXA1inPBS
chr2	27526590	27526591	MACS_peak_7817	10.00	lobChIP_FOXA1inPBS
chr2	27579761	27579762	MACS_peak_7818	16.00	lobChIP_FOXA1inPBS
chr2	27580902	27580903	MACS_peak_7819	9.00	lobChIP_FOXA1inPBS
chr2	27635886	27635887	MACS_peak_7820	9.00	lobChIP_FOXA1inPBS
chr2	27648018	27648019	MACS_peak_7821	8.00	lobChIP_FOXA1inPBS
chr2	27719080	27719081	MACS_peak_7822	13.00	lobChIP_FOXA1inPBS
chr2	27837110	27837111	MACS_peak_7823	11.00	lobChIP_FOXA1inPBS
chr2	27948300	27948301	MACS_peak_7824	10.00	lobChIP_FOXA1inPBS
chr2	27969308	27969309	MACS_peak_7825	30.00	lobChIP_FOXA1inPBS
chr2	27986309	27986310	MACS_peak_7826	27.00	lobChIP_FOXA1inPBS
chr2	28098524	28098525	MACS_peak_7827	16.00	lobChIP_FOXA1inPBS
chr2	28234559	28234560	MACS_peak_7828	10.00	lobChIP_FOXA1inPBS
chr2	28542137	28542138	MACS_peak_7829	11.00	lobChIP_FOXA1inPBS
chr2	28582418	28582419	MACS_peak_7830	7.00	lobChIP_FOXA1inPBS
chr2	28619489	28619490	MACS_peak_7831	10.00	lobChIP_FOXA1inPBS
chr2	28932137	28932138	MACS_peak_7832	17.00	lobChIP_FOXA1inPBS
chr2	28943917	28943918	MACS_peak_7833	10.00	lobChIP_FOXA1inPBS
chr2	28976365	28976366	MACS_peak_7834	20.00	lobChIP_FOXA1inPBS
chr2	28990784	28990785	MACS_peak_7835	13.00	lobChIP_FOXA1inPBS
chr2	29096248	29096249	MACS_peak_7836	7.00	lobChIP_FOXA1inPBS
chr2	29170856	29170857	MACS_peak_7837	6.00	lobChIP_FOXA1inPBS
chr2	29346738	29346739	MACS_peak_7838	14.00	lobChIP_FOXA1inPBS
chr2	30364276	30364277	MACS_peak_7839	9.00	lobChIP_FOXA1inPBS
chr2	30408218	30408219	MACS_peak_7840	7.00	lobChIP_FOXA1inPBS
chr2	30497834	30497835	MACS_peak_7841	11.00	lobChIP_FOXA1inPBS
chr2	30657011	30657012	MACS_peak_7842	9.00	lobChIP_FOXA1inPBS
chr2	30668162	30668163	MACS_peak_7843	6.00	lobChIP_FOXA1inPBS
chr2	30728885	30728886	MACS_peak_7844	12.00	lobChIP_FOXA1inPBS
chr2	30827564	30827565	MACS_peak_7845	7.00	lobChIP_FOXA1inPBS
chr2	30908950	30908951	MACS_peak_7846	13.00	lobChIP_FOXA1inPBS
chr2	31030533	31030534	MACS_peak_7847	8.00	lobChIP_FOXA1inPBS
chr2	31783973	31783974	MACS_peak_7848	12.00	lobChIP_FOXA1inPBS
chr2	32037446	32037447	MACS_peak_7849	23.00	lobChIP_FOXA1inPBS
chr2	32191220	32191221	MACS_peak_7850	7.00	lobChIP_FOXA1inPBS
chr2	32231574	32231575	MACS_peak_7851	15.00	lobChIP_FOXA1inPBS
chr2	32287333	32287334	MACS_peak_7852	6.00	lobChIP_FOXA1inPBS
chr2	32288584	32288585	MACS_peak_7853	9.00	lobChIP_FOXA1inPBS
chr2	32457282	32457283	MACS_peak_7854	13.00	lobChIP_FOXA1inPBS
chr2	32502859	32502860	MACS_peak_7855	25.00	lobChIP_FOXA1inPBS
chr2	32554426	32554427	MACS_peak_7856	22.00	lobChIP_FOXA1inPBS
chr2	32682200	32682201	MACS_peak_7857	14.00	lobChIP_FOXA1inPBS
chr2	33030521	33030522	MACS_peak_7858	13.00	lobChIP_FOXA1inPBS
chr2	33500916	33500917	MACS_peak_7859	12.00	lobChIP_FOXA1inPBS
chr2	33516627	33516628	MACS_peak_7860	20.00	lobChIP_FOXA1inPBS
chr2	33623511	33623512	MACS_peak_7861	7.00	lobChIP_FOXA1inPBS
chr2	33637033	33637034	MACS_peak_7862	11.00	lobChIP_FOXA1inPBS
chr2	34578942	34578943	MACS_peak_7863	26.00	lobChIP_FOXA1inPBS
chr2	34599038	34599039	MACS_peak_7864	9.00	lobChIP_FOXA1inPBS
chr2	36008974	36008975	MACS_peak_7865	9.00	lobChIP_FOXA1inPBS
chr2	36566170	36566171	MACS_peak_7866	11.00	lobChIP_FOXA1inPBS
chr2	36578643	36578644	MACS_peak_7867	16.00	lobChIP_FOXA1inPBS
chr2	36584969	36584970	MACS_peak_7868	6.00	lobChIP_FOXA1inPBS
chr2	36588160	36588161	MACS_peak_7869	26.00	lobChIP_FOXA1inPBS
chr2	36594829	36594830	MACS_peak_7870	10.00	lobChIP_FOXA1inPBS
chr2	36603542	36603543	MACS_peak_7871	9.00	lobChIP_FOXA1inPBS
chr2	36660665	36660666	MACS_peak_7872	46.00	lobChIP_FOXA1inPBS
chr2	36680961	36680962	MACS_peak_7873	13.00	lobChIP_FOXA1inPBS
chr2	36709808	36709809	MACS_peak_7874	19.00	lobChIP_FOXA1inPBS
chr2	36713992	36713993	MACS_peak_7875	27.00	lobChIP_FOXA1inPBS
chr2	36729462	36729463	MACS_peak_7876	36.00	lobChIP_FOXA1inPBS
chr2	36733250	36733251	MACS_peak_7877	20.00	lobChIP_FOXA1inPBS
chr2	36887252	36887253	MACS_peak_7878	9.00	lobChIP_FOXA1inPBS
chr2	36891448	36891449	MACS_peak_7879	16.00	lobChIP_FOXA1inPBS
chr2	36924631	36924632	MACS_peak_7880	9.00	lobChIP_FOXA1inPBS
chr2	36981964	36981965	MACS_peak_7881	14.00	lobChIP_FOXA1inPBS
chr2	37146400	37146401	MACS_peak_7882	19.00	lobChIP_FOXA1inPBS
chr2	37194831	37194832	MACS_peak_7883	11.00	lobChIP_FOXA1inPBS
chr2	37331030	37331031	MACS_peak_7884	22.00	lobChIP_FOXA1inPBS
chr2	37398936	37398937	MACS_peak_7885	27.00	lobChIP_FOXA1inPBS
chr2	38059959	38059960	MACS_peak_7886	9.00	lobChIP_FOXA1inPBS
chr2	38146004	38146005	MACS_peak_7887	29.00	lobChIP_FOXA1inPBS
chr2	38147954	38147955	MACS_peak_7888	26.00	lobChIP_FOXA1inPBS
chr2	38155657	38155658	MACS_peak_7889	34.00	lobChIP_FOXA1inPBS
chr2	38189687	38189688	MACS_peak_7890	17.00	lobChIP_FOXA1inPBS
chr2	38572660	38572661	MACS_peak_7891	12.00	lobChIP_FOXA1inPBS
chr2	38663492	38663493	MACS_peak_7892	25.00	lobChIP_FOXA1inPBS
chr2	38724993	38724994	MACS_peak_7893	16.00	lobChIP_FOXA1inPBS
chr2	38735678	38735679	MACS_peak_7894	11.00	lobChIP_FOXA1inPBS
chr2	38768741	38768742	MACS_peak_7895	10.00	lobChIP_FOXA1inPBS
chr2	38827111	38827112	MACS_peak_7896	17.00	lobChIP_FOXA1inPBS
chr2	38838525	38838526	MACS_peak_7897	46.00	lobChIP_FOXA1inPBS
chr2	38847642	38847643	MACS_peak_7898	17.00	lobChIP_FOXA1inPBS
chr2	38854691	38854692	MACS_peak_7899	16.00	lobChIP_FOXA1inPBS
chr2	38912707	38912708	MACS_peak_7900	29.00	lobChIP_FOXA1inPBS
chr2	38918164	38918165	MACS_peak_7901	9.00	lobChIP_FOXA1inPBS
chr2	39134934	39134935	MACS_peak_7902	19.00	lobChIP_FOXA1inPBS
chr2	39149695	39149696	MACS_peak_7903	7.00	lobChIP_FOXA1inPBS
chr2	39208885	39208886	MACS_peak_7904	38.00	lobChIP_FOXA1inPBS
chr2	39229604	39229605	MACS_peak_7905	12.00	lobChIP_FOXA1inPBS
chr2	39348546	39348547	MACS_peak_7906	8.00	lobChIP_FOXA1inPBS
chr2	39361439	39361440	MACS_peak_7907	12.00	lobChIP_FOXA1inPBS
chr2	39365464	39365465	MACS_peak_7908	41.00	lobChIP_FOXA1inPBS
chr2	39378283	39378284	MACS_peak_7909	7.00	lobChIP_FOXA1inPBS
chr2	39444180	39444181	MACS_peak_7910	14.00	lobChIP_FOXA1inPBS
chr2	39607014	39607015	MACS_peak_7911	45.00	lobChIP_FOXA1inPBS
chr2	39638249	39638250	MACS_peak_7912	9.00	lobChIP_FOXA1inPBS
chr2	39675464	39675465	MACS_peak_7913	8.00	lobChIP_FOXA1inPBS
chr2	39695163	39695164	MACS_peak_7914	16.00	lobChIP_FOXA1inPBS
chr2	39719023	39719024	MACS_peak_7915	14.00	lobChIP_FOXA1inPBS
chr2	39719491	39719492	MACS_peak_7916	18.00	lobChIP_FOXA1inPBS
chr2	39731350	39731351	MACS_peak_7917	9.00	lobChIP_FOXA1inPBS
chr2	39742832	39742833	MACS_peak_7918	14.00	lobChIP_FOXA1inPBS
chr2	39753038	39753039	MACS_peak_7919	10.00	lobChIP_FOXA1inPBS
chr2	39777998	39777999	MACS_peak_7920	39.00	lobChIP_FOXA1inPBS
chr2	40048101	40048102	MACS_peak_7921	7.00	lobChIP_FOXA1inPBS
chr2	40699179	40699180	MACS_peak_7922	18.00	lobChIP_FOXA1inPBS
chr2	40947829	40947830	MACS_peak_7923	8.00	lobChIP_FOXA1inPBS
chr2	42055335	42055336	MACS_peak_7924	40.00	lobChIP_FOXA1inPBS
chr2	42075378	42075379	MACS_peak_7925	69.00	lobChIP_FOXA1inPBS
chr2	42122320	42122321	MACS_peak_7926	7.00	lobChIP_FOXA1inPBS
chr2	42193740	42193741	MACS_peak_7927	10.00	lobChIP_FOXA1inPBS
chr2	42239733	42239734	MACS_peak_7928	7.00	lobChIP_FOXA1inPBS
chr2	42273356	42273357	MACS_peak_7929	36.00	lobChIP_FOXA1inPBS
chr2	42283277	42283278	MACS_peak_7930	15.00	lobChIP_FOXA1inPBS
chr2	42291002	42291003	MACS_peak_7931	19.00	lobChIP_FOXA1inPBS
chr2	42291723	42291724	MACS_peak_7932	9.00	lobChIP_FOXA1inPBS
chr2	42319516	42319517	MACS_peak_7933	13.00	lobChIP_FOXA1inPBS
chr2	42323161	42323162	MACS_peak_7934	29.00	lobChIP_FOXA1inPBS
chr2	42333352	42333353	MACS_peak_7935	19.00	lobChIP_FOXA1inPBS
chr2	42355090	42355091	MACS_peak_7936	15.00	lobChIP_FOXA1inPBS
chr2	42363083	42363084	MACS_peak_7937	49.00	lobChIP_FOXA1inPBS
chr2	42364355	42364356	MACS_peak_7938	10.00	lobChIP_FOXA1inPBS
chr2	42369146	42369147	MACS_peak_7939	40.00	lobChIP_FOXA1inPBS
chr2	42415276	42415277	MACS_peak_7940	10.00	lobChIP_FOXA1inPBS
chr2	42418536	42418537	MACS_peak_7941	11.00	lobChIP_FOXA1inPBS
chr2	42421537	42421538	MACS_peak_7942	5.00	lobChIP_FOXA1inPBS
chr2	42422894	42422895	MACS_peak_7943	34.00	lobChIP_FOXA1inPBS
chr2	42424170	42424171	MACS_peak_7944	13.00	lobChIP_FOXA1inPBS
chr2	42444672	42444673	MACS_peak_7945	14.00	lobChIP_FOXA1inPBS
chr2	42449797	42449798	MACS_peak_7946	29.00	lobChIP_FOXA1inPBS
chr2	42471662	42471663	MACS_peak_7947	10.00	lobChIP_FOXA1inPBS
chr2	42554482	42554483	MACS_peak_7948	19.00	lobChIP_FOXA1inPBS
chr2	42702207	42702208	MACS_peak_7949	7.00	lobChIP_FOXA1inPBS
chr2	42831676	42831677	MACS_peak_7950	7.00	lobChIP_FOXA1inPBS
chr2	42903177	42903178	MACS_peak_7951	22.00	lobChIP_FOXA1inPBS
chr2	43014986	43014987	MACS_peak_7952	9.00	lobChIP_FOXA1inPBS
chr2	43153797	43153798	MACS_peak_7953	6.00	lobChIP_FOXA1inPBS
chr2	43155456	43155457	MACS_peak_7954	13.00	lobChIP_FOXA1inPBS
chr2	43156514	43156515	MACS_peak_7955	6.00	lobChIP_FOXA1inPBS
chr2	43169041	43169042	MACS_peak_7956	16.00	lobChIP_FOXA1inPBS
chr2	43205645	43205646	MACS_peak_7957	11.00	lobChIP_FOXA1inPBS
chr2	43206897	43206898	MACS_peak_7958	51.00	lobChIP_FOXA1inPBS
chr2	43214412	43214413	MACS_peak_7959	19.00	lobChIP_FOXA1inPBS
chr2	43245507	43245508	MACS_peak_7960	8.00	lobChIP_FOXA1inPBS
chr2	43302587	43302588	MACS_peak_7961	16.00	lobChIP_FOXA1inPBS
chr2	43314758	43314759	MACS_peak_7962	9.00	lobChIP_FOXA1inPBS
chr2	43316002	43316003	MACS_peak_7963	72.00	lobChIP_FOXA1inPBS
chr2	43320061	43320062	MACS_peak_7964	15.00	lobChIP_FOXA1inPBS
chr2	43373874	43373875	MACS_peak_7965	10.00	lobChIP_FOXA1inPBS
chr2	43390236	43390237	MACS_peak_7966	12.00	lobChIP_FOXA1inPBS
chr2	43421432	43421433	MACS_peak_7967	11.00	lobChIP_FOXA1inPBS
chr2	43437771	43437772	MACS_peak_7968	11.00	lobChIP_FOXA1inPBS
chr2	43454462	43454463	MACS_peak_7969	16.00	lobChIP_FOXA1inPBS
chr2	43458641	43458642	MACS_peak_7970	10.00	lobChIP_FOXA1inPBS
chr2	43485819	43485820	MACS_peak_7971	43.00	lobChIP_FOXA1inPBS
chr2	43521210	43521211	MACS_peak_7972	30.00	lobChIP_FOXA1inPBS
chr2	43538647	43538648	MACS_peak_7973	36.00	lobChIP_FOXA1inPBS
chr2	43587509	43587510	MACS_peak_7974	17.00	lobChIP_FOXA1inPBS
chr2	43603166	43603167	MACS_peak_7975	55.00	lobChIP_FOXA1inPBS
chr2	43606753	43606754	MACS_peak_7976	8.00	lobChIP_FOXA1inPBS
chr2	43617140	43617141	MACS_peak_7977	14.00	lobChIP_FOXA1inPBS
chr2	43647559	43647560	MACS_peak_7978	23.00	lobChIP_FOXA1inPBS
chr2	43651367	43651368	MACS_peak_7979	12.00	lobChIP_FOXA1inPBS
chr2	43653805	43653806	MACS_peak_7980	8.00	lobChIP_FOXA1inPBS
chr2	43656786	43656787	MACS_peak_7981	7.00	lobChIP_FOXA1inPBS
chr2	43678714	43678715	MACS_peak_7982	16.00	lobChIP_FOXA1inPBS
chr2	43690459	43690460	MACS_peak_7983	47.00	lobChIP_FOXA1inPBS
chr2	43839719	43839720	MACS_peak_7984	9.00	lobChIP_FOXA1inPBS
chr2	43840310	43840311	MACS_peak_7985	8.00	lobChIP_FOXA1inPBS
chr2	43861944	43861945	MACS_peak_7986	57.00	lobChIP_FOXA1inPBS
chr2	43986474	43986475	MACS_peak_7987	29.00	lobChIP_FOXA1inPBS
chr2	44033715	44033716	MACS_peak_7988	7.00	lobChIP_FOXA1inPBS
chr2	44035646	44035647	MACS_peak_7989	15.00	lobChIP_FOXA1inPBS
chr2	44069853	44069854	MACS_peak_7990	33.00	lobChIP_FOXA1inPBS
chr2	44112004	44112005	MACS_peak_7991	14.00	lobChIP_FOXA1inPBS
chr2	44134081	44134082	MACS_peak_7992	23.00	lobChIP_FOXA1inPBS
chr2	44292897	44292898	MACS_peak_7993	19.00	lobChIP_FOXA1inPBS
chr2	44314534	44314535	MACS_peak_7994	16.00	lobChIP_FOXA1inPBS
chr2	44460776	44460777	MACS_peak_7995	10.00	lobChIP_FOXA1inPBS
chr2	44522899	44522900	MACS_peak_7996	9.00	lobChIP_FOXA1inPBS
chr2	44987113	44987114	MACS_peak_7997	12.00	lobChIP_FOXA1inPBS
chr2	45346378	45346379	MACS_peak_7998	9.00	lobChIP_FOXA1inPBS
chr2	45484665	45484666	MACS_peak_7999	36.00	lobChIP_FOXA1inPBS
chr2	45500850	45500851	MACS_peak_8000	72.00	lobChIP_FOXA1inPBS
chr2	45501845	45501846	MACS_peak_8001	45.00	lobChIP_FOXA1inPBS
chr2	45527276	45527277	MACS_peak_8002	8.00	lobChIP_FOXA1inPBS
chr2	45600919	45600920	MACS_peak_8003	10.00	lobChIP_FOXA1inPBS
chr2	45670886	45670887	MACS_peak_8004	7.00	lobChIP_FOXA1inPBS
chr2	45697089	45697090	MACS_peak_8005	36.00	lobChIP_FOXA1inPBS
chr2	46005918	46005919	MACS_peak_8006	30.00	lobChIP_FOXA1inPBS
chr2	46170998	46170999	MACS_peak_8007	14.00	lobChIP_FOXA1inPBS
chr2	46219651	46219652	MACS_peak_8008	18.00	lobChIP_FOXA1inPBS
chr2	46229871	46229872	MACS_peak_8009	30.00	lobChIP_FOXA1inPBS
chr2	46260266	46260267	MACS_peak_8010	20.00	lobChIP_FOXA1inPBS
chr2	46265580	46265581	MACS_peak_8011	28.00	lobChIP_FOXA1inPBS
chr2	46266780	46266781	MACS_peak_8012	6.00	lobChIP_FOXA1inPBS
chr2	46269541	46269542	MACS_peak_8013	12.00	lobChIP_FOXA1inPBS
chr2	46400969	46400970	MACS_peak_8014	76.00	lobChIP_FOXA1inPBS
chr2	46453305	46453306	MACS_peak_8015	8.00	lobChIP_FOXA1inPBS
chr2	46510960	46510961	MACS_peak_8016	15.00	lobChIP_FOXA1inPBS
chr2	46526758	46526759	MACS_peak_8017	12.00	lobChIP_FOXA1inPBS
chr2	46534526	46534527	MACS_peak_8018	20.00	lobChIP_FOXA1inPBS
chr2	46541328	46541329	MACS_peak_8019	13.00	lobChIP_FOXA1inPBS
chr2	46553699	46553700	MACS_peak_8020	9.00	lobChIP_FOXA1inPBS
chr2	46621994	46621995	MACS_peak_8021	25.00	lobChIP_FOXA1inPBS
chr2	46636386	46636387	MACS_peak_8022	18.00	lobChIP_FOXA1inPBS
chr2	46669522	46669523	MACS_peak_8023	17.00	lobChIP_FOXA1inPBS
chr2	46884323	46884324	MACS_peak_8024	9.00	lobChIP_FOXA1inPBS
chr2	46935727	46935728	MACS_peak_8025	5.00	lobChIP_FOXA1inPBS
chr2	46964903	46964904	MACS_peak_8026	22.00	lobChIP_FOXA1inPBS
chr2	46974882	46974883	MACS_peak_8027	13.00	lobChIP_FOXA1inPBS
chr2	46976288	46976289	MACS_peak_8028	10.00	lobChIP_FOXA1inPBS
chr2	47198237	47198238	MACS_peak_8029	9.00	lobChIP_FOXA1inPBS
chr2	47257378	47257379	MACS_peak_8030	27.00	lobChIP_FOXA1inPBS
chr2	47262290	47262291	MACS_peak_8031	10.00	lobChIP_FOXA1inPBS
chr2	47306276	47306277	MACS_peak_8032	38.00	lobChIP_FOXA1inPBS
chr2	47351975	47351976	MACS_peak_8033	12.00	lobChIP_FOXA1inPBS
chr2	47419177	47419178	MACS_peak_8034	19.00	lobChIP_FOXA1inPBS
chr2	47420537	47420538	MACS_peak_8035	12.00	lobChIP_FOXA1inPBS
chr2	47454028	47454029	MACS_peak_8036	13.00	lobChIP_FOXA1inPBS
chr2	47460336	47460337	MACS_peak_8037	24.00	lobChIP_FOXA1inPBS
chr2	47492518	47492519	MACS_peak_8038	11.00	lobChIP_FOXA1inPBS
chr2	47509972	47509973	MACS_peak_8039	13.00	lobChIP_FOXA1inPBS
chr2	47514841	47514842	MACS_peak_8040	8.00	lobChIP_FOXA1inPBS
chr2	47537454	47537455	MACS_peak_8041	12.00	lobChIP_FOXA1inPBS
chr2	47540427	47540428	MACS_peak_8042	21.00	lobChIP_FOXA1inPBS
chr2	47572440	47572441	MACS_peak_8043	13.00	lobChIP_FOXA1inPBS
chr2	47628207	47628208	MACS_peak_8044	7.00	lobChIP_FOXA1inPBS
chr2	47630035	47630036	MACS_peak_8045	26.00	lobChIP_FOXA1inPBS
chr2	47979752	47979753	MACS_peak_8046	11.00	lobChIP_FOXA1inPBS
chr2	48129618	48129619	MACS_peak_8047	11.00	lobChIP_FOXA1inPBS
chr2	48449230	48449231	MACS_peak_8048	92.00	lobChIP_FOXA1inPBS
chr2	48532810	48532811	MACS_peak_8049	11.00	lobChIP_FOXA1inPBS
chr2	48571586	48571587	MACS_peak_8050	10.00	lobChIP_FOXA1inPBS
chr2	48573368	48573369	MACS_peak_8051	10.00	lobChIP_FOXA1inPBS
chr2	48675878	48675879	MACS_peak_8052	7.00	lobChIP_FOXA1inPBS
chr2	48736118	48736119	MACS_peak_8053	13.00	lobChIP_FOXA1inPBS
chr2	48901446	48901447	MACS_peak_8054	7.00	lobChIP_FOXA1inPBS
chr2	49467945	49467946	MACS_peak_8055	11.00	lobChIP_FOXA1inPBS
chr2	53942961	53942962	MACS_peak_8056	10.00	lobChIP_FOXA1inPBS
chr2	54054119	54054120	MACS_peak_8057	49.00	lobChIP_FOXA1inPBS
chr2	54196604	54196605	MACS_peak_8058	27.00	lobChIP_FOXA1inPBS
chr2	54198684	54198685	MACS_peak_8059	23.00	lobChIP_FOXA1inPBS
chr2	54271274	54271275	MACS_peak_8060	55.00	lobChIP_FOXA1inPBS
chr2	54334651	54334652	MACS_peak_8061	7.00	lobChIP_FOXA1inPBS
chr2	54349380	54349381	MACS_peak_8062	12.00	lobChIP_FOXA1inPBS
chr2	54435007	54435008	MACS_peak_8063	32.00	lobChIP_FOXA1inPBS
chr2	54437691	54437692	MACS_peak_8064	11.00	lobChIP_FOXA1inPBS
chr2	54461424	54461425	MACS_peak_8065	41.00	lobChIP_FOXA1inPBS
chr2	54488930	54488931	MACS_peak_8066	15.00	lobChIP_FOXA1inPBS
chr2	54556890	54556891	MACS_peak_8067	12.00	lobChIP_FOXA1inPBS
chr2	54681953	54681954	MACS_peak_8068	74.00	lobChIP_FOXA1inPBS
chr2	54714463	54714464	MACS_peak_8069	7.00	lobChIP_FOXA1inPBS
chr2	54759605	54759606	MACS_peak_8070	10.00	lobChIP_FOXA1inPBS
chr2	54773047	54773048	MACS_peak_8071	20.00	lobChIP_FOXA1inPBS
chr2	54784741	54784742	MACS_peak_8072	11.00	lobChIP_FOXA1inPBS
chr2	54799610	54799611	MACS_peak_8073	64.00	lobChIP_FOXA1inPBS
chr2	54801399	54801400	MACS_peak_8074	13.00	lobChIP_FOXA1inPBS
chr2	54806981	54806982	MACS_peak_8075	38.00	lobChIP_FOXA1inPBS
chr2	54817178	54817179	MACS_peak_8076	38.00	lobChIP_FOXA1inPBS
chr2	54857644	54857645	MACS_peak_8077	13.00	lobChIP_FOXA1inPBS
chr2	54897090	54897091	MACS_peak_8078	22.00	lobChIP_FOXA1inPBS
chr2	54931913	54931914	MACS_peak_8079	11.00	lobChIP_FOXA1inPBS
chr2	54933959	54933960	MACS_peak_8080	19.00	lobChIP_FOXA1inPBS
chr2	55085731	55085732	MACS_peak_8081	16.00	lobChIP_FOXA1inPBS
chr2	55099125	55099126	MACS_peak_8082	19.00	lobChIP_FOXA1inPBS
chr2	55100510	55100511	MACS_peak_8083	10.00	lobChIP_FOXA1inPBS
chr2	55239757	55239758	MACS_peak_8084	8.00	lobChIP_FOXA1inPBS
chr2	55269077	55269078	MACS_peak_8085	7.00	lobChIP_FOXA1inPBS
chr2	55274135	55274136	MACS_peak_8086	8.00	lobChIP_FOXA1inPBS
chr2	55336886	55336887	MACS_peak_8087	52.00	lobChIP_FOXA1inPBS
chr2	55407705	55407706	MACS_peak_8088	19.00	lobChIP_FOXA1inPBS
chr2	55466950	55466951	MACS_peak_8089	22.00	lobChIP_FOXA1inPBS
chr2	55511158	55511159	MACS_peak_8090	6.00	lobChIP_FOXA1inPBS
chr2	55821444	55821445	MACS_peak_8091	8.00	lobChIP_FOXA1inPBS
chr2	55843917	55843918	MACS_peak_8092	16.00	lobChIP_FOXA1inPBS
chr2	56064319	56064320	MACS_peak_8093	8.00	lobChIP_FOXA1inPBS
chr2	56066420	56066421	MACS_peak_8094	21.00	lobChIP_FOXA1inPBS
chr2	56069400	56069401	MACS_peak_8095	9.00	lobChIP_FOXA1inPBS
chr2	58121079	58121080	MACS_peak_8096	15.00	lobChIP_FOXA1inPBS
chr2	58308428	58308429	MACS_peak_8097	12.00	lobChIP_FOXA1inPBS
chr2	58397897	58397898	MACS_peak_8098	28.00	lobChIP_FOXA1inPBS
chr2	58422942	58422943	MACS_peak_8099	7.00	lobChIP_FOXA1inPBS
chr2	58431658	58431659	MACS_peak_8100	10.00	lobChIP_FOXA1inPBS
chr2	59300634	59300635	MACS_peak_8101	13.00	lobChIP_FOXA1inPBS
chr2	60321842	60321843	MACS_peak_8102	8.00	lobChIP_FOXA1inPBS
chr2	60760325	60760326	MACS_peak_8103	10.00	lobChIP_FOXA1inPBS
chr2	60984979	60984980	MACS_peak_8104	7.00	lobChIP_FOXA1inPBS
chr2	61112970	61112971	MACS_peak_8105	46.00	lobChIP_FOXA1inPBS
chr2	61153275	61153276	MACS_peak_8106	18.00	lobChIP_FOXA1inPBS
chr2	61204147	61204148	MACS_peak_8107	11.00	lobChIP_FOXA1inPBS
chr2	61243743	61243744	MACS_peak_8108	11.00	lobChIP_FOXA1inPBS
chr2	61282839	61282840	MACS_peak_8109	6.00	lobChIP_FOXA1inPBS
chr2	61353129	61353130	MACS_peak_8110	9.00	lobChIP_FOXA1inPBS
chr2	61494031	61494032	MACS_peak_8111	11.00	lobChIP_FOXA1inPBS
chr2	61583895	61583896	MACS_peak_8112	23.00	lobChIP_FOXA1inPBS
chr2	61699157	61699158	MACS_peak_8113	10.00	lobChIP_FOXA1inPBS
chr2	62194630	62194631	MACS_peak_8114	16.00	lobChIP_FOXA1inPBS
chr2	62452307	62452308	MACS_peak_8115	14.00	lobChIP_FOXA1inPBS
chr2	62636066	62636067	MACS_peak_8116	9.00	lobChIP_FOXA1inPBS
chr2	62694361	62694362	MACS_peak_8117	25.00	lobChIP_FOXA1inPBS
chr2	62695024	62695025	MACS_peak_8118	8.00	lobChIP_FOXA1inPBS
chr2	62713189	62713190	MACS_peak_8119	19.00	lobChIP_FOXA1inPBS
chr2	62753955	62753956	MACS_peak_8120	16.00	lobChIP_FOXA1inPBS
chr2	62755058	62755059	MACS_peak_8121	14.00	lobChIP_FOXA1inPBS
chr2	62767581	62767582	MACS_peak_8122	17.00	lobChIP_FOXA1inPBS
chr2	62792904	62792905	MACS_peak_8123	14.00	lobChIP_FOXA1inPBS
chr2	62796232	62796233	MACS_peak_8124	12.00	lobChIP_FOXA1inPBS
chr2	62797442	62797443	MACS_peak_8125	37.00	lobChIP_FOXA1inPBS
chr2	62798726	62798727	MACS_peak_8126	10.00	lobChIP_FOXA1inPBS
chr2	62806202	62806203	MACS_peak_8127	14.00	lobChIP_FOXA1inPBS
chr2	62869861	62869862	MACS_peak_8128	9.00	lobChIP_FOXA1inPBS
chr2	62881228	62881229	MACS_peak_8129	24.00	lobChIP_FOXA1inPBS
chr2	62966557	62966558	MACS_peak_8130	8.00	lobChIP_FOXA1inPBS
chr2	63022128	63022129	MACS_peak_8131	16.00	lobChIP_FOXA1inPBS
chr2	63222053	63222054	MACS_peak_8132	9.00	lobChIP_FOXA1inPBS
chr2	63420238	63420239	MACS_peak_8133	161.00	lobChIP_FOXA1inPBS
chr2	63823916	63823917	MACS_peak_8134	14.00	lobChIP_FOXA1inPBS
chr2	63948136	63948137	MACS_peak_8135	8.00	lobChIP_FOXA1inPBS
chr2	63961771	63961772	MACS_peak_8136	60.00	lobChIP_FOXA1inPBS
chr2	64047613	64047614	MACS_peak_8137	12.00	lobChIP_FOXA1inPBS
chr2	64055157	64055158	MACS_peak_8138	18.00	lobChIP_FOXA1inPBS
chr2	64209038	64209039	MACS_peak_8139	40.00	lobChIP_FOXA1inPBS
chr2	64211531	64211532	MACS_peak_8140	101.00	lobChIP_FOXA1inPBS
chr2	64218789	64218790	MACS_peak_8141	57.00	lobChIP_FOXA1inPBS
chr2	64248968	64248969	MACS_peak_8142	14.00	lobChIP_FOXA1inPBS
chr2	64251689	64251690	MACS_peak_8143	28.00	lobChIP_FOXA1inPBS
chr2	64315743	64315744	MACS_peak_8144	6.00	lobChIP_FOXA1inPBS
chr2	64356155	64356156	MACS_peak_8145	9.00	lobChIP_FOXA1inPBS
chr2	64653510	64653511	MACS_peak_8146	19.00	lobChIP_FOXA1inPBS
chr2	64661658	64661659	MACS_peak_8147	10.00	lobChIP_FOXA1inPBS
chr2	64743893	64743894	MACS_peak_8148	11.00	lobChIP_FOXA1inPBS
chr2	64744825	64744826	MACS_peak_8149	14.00	lobChIP_FOXA1inPBS
chr2	64770075	64770076	MACS_peak_8150	13.00	lobChIP_FOXA1inPBS
chr2	64860543	64860544	MACS_peak_8151	12.00	lobChIP_FOXA1inPBS
chr2	64871699	64871700	MACS_peak_8152	9.00	lobChIP_FOXA1inPBS
chr2	64873586	64873587	MACS_peak_8153	32.00	lobChIP_FOXA1inPBS
chr2	64878042	64878043	MACS_peak_8154	12.00	lobChIP_FOXA1inPBS
chr2	64894308	64894309	MACS_peak_8155	9.00	lobChIP_FOXA1inPBS
chr2	64912783	64912784	MACS_peak_8156	25.00	lobChIP_FOXA1inPBS
chr2	64957974	64957975	MACS_peak_8157	17.00	lobChIP_FOXA1inPBS
chr2	65063908	65063909	MACS_peak_8158	14.00	lobChIP_FOXA1inPBS
chr2	65150106	65150107	MACS_peak_8159	10.00	lobChIP_FOXA1inPBS
chr2	65170956	65170957	MACS_peak_8160	24.00	lobChIP_FOXA1inPBS
chr2	65209929	65209930	MACS_peak_8161	27.00	lobChIP_FOXA1inPBS
chr2	65253441	65253442	MACS_peak_8162	25.00	lobChIP_FOXA1inPBS
chr2	65292411	65292412	MACS_peak_8163	8.00	lobChIP_FOXA1inPBS
chr2	65528391	65528392	MACS_peak_8164	38.00	lobChIP_FOXA1inPBS
chr2	65590515	65590516	MACS_peak_8165	15.00	lobChIP_FOXA1inPBS
chr2	65600671	65600672	MACS_peak_8166	118.00	lobChIP_FOXA1inPBS
chr2	65623610	65623611	MACS_peak_8167	10.00	lobChIP_FOXA1inPBS
chr2	65659889	65659890	MACS_peak_8168	8.00	lobChIP_FOXA1inPBS
chr2	65710252	65710253	MACS_peak_8169	13.00	lobChIP_FOXA1inPBS
chr2	65804290	65804291	MACS_peak_8170	9.00	lobChIP_FOXA1inPBS
chr2	66146093	66146094	MACS_peak_8171	9.00	lobChIP_FOXA1inPBS
chr2	66496612	66496613	MACS_peak_8172	9.00	lobChIP_FOXA1inPBS
chr2	66750225	66750226	MACS_peak_8173	7.00	lobChIP_FOXA1inPBS
chr2	67285329	67285330	MACS_peak_8174	8.00	lobChIP_FOXA1inPBS
chr2	67536618	67536619	MACS_peak_8175	9.00	lobChIP_FOXA1inPBS
chr2	68480769	68480770	MACS_peak_8176	10.00	lobChIP_FOXA1inPBS
chr2	68667946	68667947	MACS_peak_8177	18.00	lobChIP_FOXA1inPBS
chr2	68821141	68821142	MACS_peak_8178	9.00	lobChIP_FOXA1inPBS
chr2	68936654	68936655	MACS_peak_8179	22.00	lobChIP_FOXA1inPBS
chr2	69115357	69115358	MACS_peak_8180	21.00	lobChIP_FOXA1inPBS
chr2	69289712	69289713	MACS_peak_8181	16.00	lobChIP_FOXA1inPBS
chr2	69310032	69310033	MACS_peak_8182	27.00	lobChIP_FOXA1inPBS
chr2	69324578	69324579	MACS_peak_8183	10.00	lobChIP_FOXA1inPBS
chr2	69394035	69394036	MACS_peak_8184	22.00	lobChIP_FOXA1inPBS
chr2	69405377	69405378	MACS_peak_8185	9.00	lobChIP_FOXA1inPBS
chr2	69430782	69430783	MACS_peak_8186	17.00	lobChIP_FOXA1inPBS
chr2	69443862	69443863	MACS_peak_8187	14.00	lobChIP_FOXA1inPBS
chr2	69544028	69544029	MACS_peak_8188	9.00	lobChIP_FOXA1inPBS
chr2	69711075	69711076	MACS_peak_8189	21.00	lobChIP_FOXA1inPBS
chr2	69727384	69727385	MACS_peak_8190	8.00	lobChIP_FOXA1inPBS
chr2	69902481	69902482	MACS_peak_8191	8.00	lobChIP_FOXA1inPBS
chr2	69909646	69909647	MACS_peak_8192	7.00	lobChIP_FOXA1inPBS
chr2	69913012	69913013	MACS_peak_8193	10.00	lobChIP_FOXA1inPBS
chr2	69961600	69961601	MACS_peak_8194	10.00	lobChIP_FOXA1inPBS
chr2	69971160	69971161	MACS_peak_8195	7.00	lobChIP_FOXA1inPBS
chr2	70008782	70008783	MACS_peak_8196	47.00	lobChIP_FOXA1inPBS
chr2	70173915	70173916	MACS_peak_8197	12.00	lobChIP_FOXA1inPBS
chr2	70220993	70220994	MACS_peak_8198	18.00	lobChIP_FOXA1inPBS
chr2	70277578	70277579	MACS_peak_8199	6.00	lobChIP_FOXA1inPBS
chr2	70313899	70313900	MACS_peak_8200	29.00	lobChIP_FOXA1inPBS
chr2	70322431	70322432	MACS_peak_8201	9.00	lobChIP_FOXA1inPBS
chr2	70337746	70337747	MACS_peak_8202	10.00	lobChIP_FOXA1inPBS
chr2	70367965	70367966	MACS_peak_8203	8.00	lobChIP_FOXA1inPBS
chr2	70415767	70415768	MACS_peak_8204	26.00	lobChIP_FOXA1inPBS
chr2	70476893	70476894	MACS_peak_8205	16.00	lobChIP_FOXA1inPBS
chr2	70496833	70496834	MACS_peak_8206	7.00	lobChIP_FOXA1inPBS
chr2	70752388	70752389	MACS_peak_8207	13.00	lobChIP_FOXA1inPBS
chr2	70784543	70784544	MACS_peak_8208	30.00	lobChIP_FOXA1inPBS
chr2	70792420	70792421	MACS_peak_8209	30.00	lobChIP_FOXA1inPBS
chr2	71034574	71034575	MACS_peak_8210	40.00	lobChIP_FOXA1inPBS
chr2	71147101	71147102	MACS_peak_8211	38.00	lobChIP_FOXA1inPBS
chr2	71222738	71222739	MACS_peak_8212	6.00	lobChIP_FOXA1inPBS
chr2	71356252	71356253	MACS_peak_8213	10.00	lobChIP_FOXA1inPBS
chr2	72305230	72305231	MACS_peak_8214	71.00	lobChIP_FOXA1inPBS
chr2	72595111	72595112	MACS_peak_8215	8.00	lobChIP_FOXA1inPBS
chr2	72775630	72775631	MACS_peak_8216	11.00	lobChIP_FOXA1inPBS
chr2	72783357	72783358	MACS_peak_8217	14.00	lobChIP_FOXA1inPBS
chr2	72833154	72833155	MACS_peak_8218	14.00	lobChIP_FOXA1inPBS
chr2	72908707	72908708	MACS_peak_8219	17.00	lobChIP_FOXA1inPBS
chr2	72922162	72922163	MACS_peak_8220	22.00	lobChIP_FOXA1inPBS
chr2	72951053	72951054	MACS_peak_8221	11.00	lobChIP_FOXA1inPBS
chr2	72970831	72970832	MACS_peak_8222	17.00	lobChIP_FOXA1inPBS
chr2	72984503	72984504	MACS_peak_8223	51.00	lobChIP_FOXA1inPBS
chr2	73043460	73043461	MACS_peak_8224	9.00	lobChIP_FOXA1inPBS
chr2	73248684	73248685	MACS_peak_8225	19.00	lobChIP_FOXA1inPBS
chr2	73259552	73259553	MACS_peak_8226	26.00	lobChIP_FOXA1inPBS
chr2	73419012	73419013	MACS_peak_8227	20.00	lobChIP_FOXA1inPBS
chr2	73427062	73427063	MACS_peak_8228	22.00	lobChIP_FOXA1inPBS
chr2	73440769	73440770	MACS_peak_8229	9.00	lobChIP_FOXA1inPBS
chr2	73489680	73489681	MACS_peak_8230	37.00	lobChIP_FOXA1inPBS
chr2	74007321	74007322	MACS_peak_8231	26.00	lobChIP_FOXA1inPBS
chr2	74069898	74069899	MACS_peak_8232	15.00	lobChIP_FOXA1inPBS
chr2	74207948	74207949	MACS_peak_8233	13.00	lobChIP_FOXA1inPBS
chr2	74378801	74378802	MACS_peak_8234	6.00	lobChIP_FOXA1inPBS
chr2	74520072	74520073	MACS_peak_8235	27.00	lobChIP_FOXA1inPBS
chr2	74713509	74713510	MACS_peak_8236	34.00	lobChIP_FOXA1inPBS
chr2	74821139	74821140	MACS_peak_8237	13.00	lobChIP_FOXA1inPBS
chr2	74866838	74866839	MACS_peak_8238	12.00	lobChIP_FOXA1inPBS
chr2	74983621	74983622	MACS_peak_8239	12.00	lobChIP_FOXA1inPBS
chr2	74990640	74990641	MACS_peak_8240	9.00	lobChIP_FOXA1inPBS
chr2	74992066	74992067	MACS_peak_8241	72.00	lobChIP_FOXA1inPBS
chr2	75033838	75033839	MACS_peak_8242	17.00	lobChIP_FOXA1inPBS
chr2	75041411	75041412	MACS_peak_8243	14.00	lobChIP_FOXA1inPBS
chr2	75043678	75043679	MACS_peak_8244	6.00	lobChIP_FOXA1inPBS
chr2	75083582	75083583	MACS_peak_8245	38.00	lobChIP_FOXA1inPBS
chr2	75129634	75129635	MACS_peak_8246	11.00	lobChIP_FOXA1inPBS
chr2	75164863	75164864	MACS_peak_8247	19.00	lobChIP_FOXA1inPBS
chr2	75167428	75167429	MACS_peak_8248	62.00	lobChIP_FOXA1inPBS
chr2	75229384	75229385	MACS_peak_8249	10.00	lobChIP_FOXA1inPBS
chr2	75280905	75280906	MACS_peak_8250	11.00	lobChIP_FOXA1inPBS
chr2	75380603	75380604	MACS_peak_8251	68.00	lobChIP_FOXA1inPBS
chr2	75708662	75708663	MACS_peak_8252	74.00	lobChIP_FOXA1inPBS
chr2	75743692	75743693	MACS_peak_8253	40.00	lobChIP_FOXA1inPBS
chr2	75748888	75748889	MACS_peak_8254	48.00	lobChIP_FOXA1inPBS
chr2	75750974	75750975	MACS_peak_8255	12.00	lobChIP_FOXA1inPBS
chr2	75756489	75756490	MACS_peak_8256	11.00	lobChIP_FOXA1inPBS
chr2	75776870	75776871	MACS_peak_8257	9.00	lobChIP_FOXA1inPBS
chr2	75792845	75792846	MACS_peak_8258	9.00	lobChIP_FOXA1inPBS
chr2	75798373	75798374	MACS_peak_8259	22.00	lobChIP_FOXA1inPBS
chr2	75804535	75804536	MACS_peak_8260	9.00	lobChIP_FOXA1inPBS
chr2	75890043	75890044	MACS_peak_8261	9.00	lobChIP_FOXA1inPBS
chr2	76399468	76399469	MACS_peak_8262	28.00	lobChIP_FOXA1inPBS
chr2	76420347	76420348	MACS_peak_8263	15.00	lobChIP_FOXA1inPBS
chr2	77237500	77237501	MACS_peak_8264	11.00	lobChIP_FOXA1inPBS
chr2	77418308	77418309	MACS_peak_8265	22.00	lobChIP_FOXA1inPBS
chr2	77832269	77832270	MACS_peak_8266	11.00	lobChIP_FOXA1inPBS
chr2	78050346	78050347	MACS_peak_8267	6.00	lobChIP_FOXA1inPBS
chr2	78065662	78065663	MACS_peak_8268	15.00	lobChIP_FOXA1inPBS
chr2	78203183	78203184	MACS_peak_8269	5.00	lobChIP_FOXA1inPBS
chr2	78389560	78389561	MACS_peak_8270	83.00	lobChIP_FOXA1inPBS
chr2	78414108	78414109	MACS_peak_8271	6.00	lobChIP_FOXA1inPBS
chr2	78488165	78488166	MACS_peak_8272	35.00	lobChIP_FOXA1inPBS
chr2	78643924	78643925	MACS_peak_8273	11.00	lobChIP_FOXA1inPBS
chr2	78769165	78769166	MACS_peak_8274	8.00	lobChIP_FOXA1inPBS
chr2	80245609	80245610	MACS_peak_8275	14.00	lobChIP_FOXA1inPBS
chr2	81474965	81474966	MACS_peak_8276	12.00	lobChIP_FOXA1inPBS
chr2	83298613	83298614	MACS_peak_8277	14.00	lobChIP_FOXA1inPBS
chr2	83306376	83306377	MACS_peak_8278	8.00	lobChIP_FOXA1inPBS
chr2	84180192	84180193	MACS_peak_8279	15.00	lobChIP_FOXA1inPBS
chr2	84521139	84521140	MACS_peak_8280	26.00	lobChIP_FOXA1inPBS
chr2	84527540	84527541	MACS_peak_8281	113.00	lobChIP_FOXA1inPBS
chr2	84530034	84530035	MACS_peak_8282	13.00	lobChIP_FOXA1inPBS
chr2	84687228	84687229	MACS_peak_8283	10.00	lobChIP_FOXA1inPBS
chr2	84840301	84840302	MACS_peak_8284	8.00	lobChIP_FOXA1inPBS
chr2	85135576	85135577	MACS_peak_8285	14.00	lobChIP_FOXA1inPBS
chr2	85147157	85147158	MACS_peak_8286	11.00	lobChIP_FOXA1inPBS
chr2	85186964	85186965	MACS_peak_8287	13.00	lobChIP_FOXA1inPBS
chr2	85191072	85191073	MACS_peak_8288	10.00	lobChIP_FOXA1inPBS
chr2	85191683	85191684	MACS_peak_8289	10.00	lobChIP_FOXA1inPBS
chr2	85319380	85319381	MACS_peak_8290	8.00	lobChIP_FOXA1inPBS
chr2	85552342	85552343	MACS_peak_8291	23.00	lobChIP_FOXA1inPBS
chr2	85647208	85647209	MACS_peak_8292	19.00	lobChIP_FOXA1inPBS
chr2	85648648	85648649	MACS_peak_8293	57.00	lobChIP_FOXA1inPBS
chr2	85774128	85774129	MACS_peak_8294	25.00	lobChIP_FOXA1inPBS
chr2	85779333	85779334	MACS_peak_8295	17.00	lobChIP_FOXA1inPBS
chr2	85812243	85812244	MACS_peak_8296	19.00	lobChIP_FOXA1inPBS
chr2	85814794	85814795	MACS_peak_8297	29.00	lobChIP_FOXA1inPBS
chr2	85969224	85969225	MACS_peak_8298	12.00	lobChIP_FOXA1inPBS
chr2	86117349	86117350	MACS_peak_8299	7.00	lobChIP_FOXA1inPBS
chr2	86182208	86182209	MACS_peak_8300	18.00	lobChIP_FOXA1inPBS
chr2	86268408	86268409	MACS_peak_8301	6.00	lobChIP_FOXA1inPBS
chr2	86343050	86343051	MACS_peak_8302	6.00	lobChIP_FOXA1inPBS
chr2	86668238	86668239	MACS_peak_8303	18.00	lobChIP_FOXA1inPBS
chr2	86783832	86783833	MACS_peak_8304	33.00	lobChIP_FOXA1inPBS
chr2	86797424	86797425	MACS_peak_8305	9.00	lobChIP_FOXA1inPBS
chr2	86855973	86855974	MACS_peak_8306	8.00	lobChIP_FOXA1inPBS
chr2	86859998	86859999	MACS_peak_8307	39.00	lobChIP_FOXA1inPBS
chr2	86933471	86933472	MACS_peak_8308	11.00	lobChIP_FOXA1inPBS
chr2	87831732	87831733	MACS_peak_8309	19.00	lobChIP_FOXA1inPBS
chr2	87957735	87957736	MACS_peak_8310	10.00	lobChIP_FOXA1inPBS
chr2	88315625	88315626	MACS_peak_8311	18.00	lobChIP_FOXA1inPBS
chr2	88428052	88428053	MACS_peak_8312	95.00	lobChIP_FOXA1inPBS
chr2	88431233	88431234	MACS_peak_8313	11.00	lobChIP_FOXA1inPBS
chr2	88506618	88506619	MACS_peak_8314	33.00	lobChIP_FOXA1inPBS
chr2	88510150	88510151	MACS_peak_8315	12.00	lobChIP_FOXA1inPBS
chr2	88621346	88621347	MACS_peak_8316	18.00	lobChIP_FOXA1inPBS
chr2	88629885	88629886	MACS_peak_8317	18.00	lobChIP_FOXA1inPBS
chr2	88754510	88754511	MACS_peak_8318	7.00	lobChIP_FOXA1inPBS
chr2	88968628	88968629	MACS_peak_8319	6.00	lobChIP_FOXA1inPBS
chr2	88985862	88985863	MACS_peak_8320	11.00	lobChIP_FOXA1inPBS
chr2	89006801	89006802	MACS_peak_8321	14.00	lobChIP_FOXA1inPBS
chr2	90246465	90246466	MACS_peak_8322	14.00	lobChIP_FOXA1inPBS
chr2	91809592	91809593	MACS_peak_8323	70.00	lobChIP_FOXA1inPBS
chr2	91844696	91844697	MACS_peak_8324	8.00	lobChIP_FOXA1inPBS
chr2	95781208	95781209	MACS_peak_8325	12.00	lobChIP_FOXA1inPBS
chr2	96733920	96733921	MACS_peak_8326	8.00	lobChIP_FOXA1inPBS
chr2	96853266	96853267	MACS_peak_8327	21.00	lobChIP_FOXA1inPBS
chr2	96926253	96926254	MACS_peak_8328	25.00	lobChIP_FOXA1inPBS
chr2	97073532	97073533	MACS_peak_8329	12.00	lobChIP_FOXA1inPBS
chr2	97304213	97304214	MACS_peak_8330	10.00	lobChIP_FOXA1inPBS
chr2	97346478	97346479	MACS_peak_8331	16.00	lobChIP_FOXA1inPBS
chr2	97481890	97481891	MACS_peak_8332	9.00	lobChIP_FOXA1inPBS
chr2	97485151	97485152	MACS_peak_8333	10.00	lobChIP_FOXA1inPBS
chr2	97490046	97490047	MACS_peak_8334	6.00	lobChIP_FOXA1inPBS
chr2	97528059	97528060	MACS_peak_8335	16.00	lobChIP_FOXA1inPBS
chr2	98207455	98207456	MACS_peak_8336	9.00	lobChIP_FOXA1inPBS
chr2	98360532	98360533	MACS_peak_8337	8.00	lobChIP_FOXA1inPBS
chr2	98474610	98474611	MACS_peak_8338	8.00	lobChIP_FOXA1inPBS
chr2	98552860	98552861	MACS_peak_8339	16.00	lobChIP_FOXA1inPBS
chr2	98587005	98587006	MACS_peak_8340	7.00	lobChIP_FOXA1inPBS
chr2	98630171	98630172	MACS_peak_8341	11.00	lobChIP_FOXA1inPBS
chr2	98701507	98701508	MACS_peak_8342	18.00	lobChIP_FOXA1inPBS
chr2	98911836	98911837	MACS_peak_8343	9.00	lobChIP_FOXA1inPBS
chr2	99200670	99200671	MACS_peak_8344	15.00	lobChIP_FOXA1inPBS
chr2	99319118	99319119	MACS_peak_8345	7.00	lobChIP_FOXA1inPBS
chr2	99321386	99321387	MACS_peak_8346	48.00	lobChIP_FOXA1inPBS
chr2	99353109	99353110	MACS_peak_8347	25.00	lobChIP_FOXA1inPBS
chr2	99412058	99412059	MACS_peak_8348	12.00	lobChIP_FOXA1inPBS
chr2	99485230	99485231	MACS_peak_8349	13.00	lobChIP_FOXA1inPBS
chr2	99728791	99728792	MACS_peak_8350	11.00	lobChIP_FOXA1inPBS
chr2	99799577	99799578	MACS_peak_8351	10.00	lobChIP_FOXA1inPBS
chr2	99895393	99895394	MACS_peak_8352	69.00	lobChIP_FOXA1inPBS
chr2	100684401	100684402	MACS_peak_8353	104.00	lobChIP_FOXA1inPBS
chr2	100859561	100859562	MACS_peak_8354	14.00	lobChIP_FOXA1inPBS
chr2	101384460	101384461	MACS_peak_8355	13.00	lobChIP_FOXA1inPBS
chr2	101396830	101396831	MACS_peak_8356	7.00	lobChIP_FOXA1inPBS
chr2	101413397	101413398	MACS_peak_8357	10.00	lobChIP_FOXA1inPBS
chr2	101440134	101440135	MACS_peak_8358	9.00	lobChIP_FOXA1inPBS
chr2	101492151	101492152	MACS_peak_8359	11.00	lobChIP_FOXA1inPBS
chr2	101498215	101498216	MACS_peak_8360	11.00	lobChIP_FOXA1inPBS
chr2	101653207	101653208	MACS_peak_8361	24.00	lobChIP_FOXA1inPBS
chr2	101681496	101681497	MACS_peak_8362	11.00	lobChIP_FOXA1inPBS
chr2	101682866	101682867	MACS_peak_8363	19.00	lobChIP_FOXA1inPBS
chr2	101723270	101723271	MACS_peak_8364	30.00	lobChIP_FOXA1inPBS
chr2	101727109	101727110	MACS_peak_8365	59.00	lobChIP_FOXA1inPBS
chr2	101730462	101730463	MACS_peak_8366	13.00	lobChIP_FOXA1inPBS
chr2	101732866	101732867	MACS_peak_8367	7.00	lobChIP_FOXA1inPBS
chr2	101733886	101733887	MACS_peak_8368	103.00	lobChIP_FOXA1inPBS
chr2	101741721	101741722	MACS_peak_8369	18.00	lobChIP_FOXA1inPBS
chr2	101812904	101812905	MACS_peak_8370	20.00	lobChIP_FOXA1inPBS
chr2	101824136	101824137	MACS_peak_8371	20.00	lobChIP_FOXA1inPBS
chr2	101856250	101856251	MACS_peak_8372	9.00	lobChIP_FOXA1inPBS
chr2	101868350	101868351	MACS_peak_8373	7.00	lobChIP_FOXA1inPBS
chr2	101950050	101950051	MACS_peak_8374	21.00	lobChIP_FOXA1inPBS
chr2	102157117	102157118	MACS_peak_8375	8.00	lobChIP_FOXA1inPBS
chr2	102173907	102173908	MACS_peak_8376	22.00	lobChIP_FOXA1inPBS
chr2	102177225	102177226	MACS_peak_8377	9.00	lobChIP_FOXA1inPBS
chr2	102183421	102183422	MACS_peak_8378	199.00	lobChIP_FOXA1inPBS
chr2	102185601	102185602	MACS_peak_8379	43.00	lobChIP_FOXA1inPBS
chr2	102193785	102193786	MACS_peak_8380	17.00	lobChIP_FOXA1inPBS
chr2	102272470	102272471	MACS_peak_8381	46.00	lobChIP_FOXA1inPBS
chr2	102296076	102296077	MACS_peak_8382	11.00	lobChIP_FOXA1inPBS
chr2	102316462	102316463	MACS_peak_8383	15.00	lobChIP_FOXA1inPBS
chr2	102354765	102354766	MACS_peak_8384	8.00	lobChIP_FOXA1inPBS
chr2	102357152	102357153	MACS_peak_8385	49.00	lobChIP_FOXA1inPBS
chr2	102380551	102380552	MACS_peak_8386	40.00	lobChIP_FOXA1inPBS
chr2	102384019	102384020	MACS_peak_8387	14.00	lobChIP_FOXA1inPBS
chr2	102388599	102388600	MACS_peak_8388	11.00	lobChIP_FOXA1inPBS
chr2	102419786	102419787	MACS_peak_8389	17.00	lobChIP_FOXA1inPBS
chr2	102577941	102577942	MACS_peak_8390	12.00	lobChIP_FOXA1inPBS
chr2	102647342	102647343	MACS_peak_8391	43.00	lobChIP_FOXA1inPBS
chr2	102649002	102649003	MACS_peak_8392	17.00	lobChIP_FOXA1inPBS
chr2	102650606	102650607	MACS_peak_8393	12.00	lobChIP_FOXA1inPBS
chr2	102662928	102662929	MACS_peak_8394	26.00	lobChIP_FOXA1inPBS
chr2	102674579	102674580	MACS_peak_8395	18.00	lobChIP_FOXA1inPBS
chr2	102717467	102717468	MACS_peak_8396	21.00	lobChIP_FOXA1inPBS
chr2	102722978	102722979	MACS_peak_8397	9.00	lobChIP_FOXA1inPBS
chr2	102730931	102730932	MACS_peak_8398	8.00	lobChIP_FOXA1inPBS
chr2	102731709	102731710	MACS_peak_8399	18.00	lobChIP_FOXA1inPBS
chr2	102772678	102772679	MACS_peak_8400	97.00	lobChIP_FOXA1inPBS
chr2	102857419	102857420	MACS_peak_8401	29.00	lobChIP_FOXA1inPBS
chr2	102872817	102872818	MACS_peak_8402	10.00	lobChIP_FOXA1inPBS
chr2	103133088	103133089	MACS_peak_8403	9.00	lobChIP_FOXA1inPBS
chr2	103340721	103340722	MACS_peak_8404	20.00	lobChIP_FOXA1inPBS
chr2	104793805	104793806	MACS_peak_8405	13.00	lobChIP_FOXA1inPBS
chr2	105093174	105093175	MACS_peak_8406	19.00	lobChIP_FOXA1inPBS
chr2	105466168	105466169	MACS_peak_8407	10.00	lobChIP_FOXA1inPBS
chr2	105956036	105956037	MACS_peak_8408	12.00	lobChIP_FOXA1inPBS
chr2	106011365	106011366	MACS_peak_8409	9.00	lobChIP_FOXA1inPBS
chr2	106260995	106260996	MACS_peak_8410	15.00	lobChIP_FOXA1inPBS
chr2	106439652	106439653	MACS_peak_8411	16.00	lobChIP_FOXA1inPBS
chr2	106651008	106651009	MACS_peak_8412	28.00	lobChIP_FOXA1inPBS
chr2	106741430	106741431	MACS_peak_8413	10.00	lobChIP_FOXA1inPBS
chr2	106897479	106897480	MACS_peak_8414	7.00	lobChIP_FOXA1inPBS
chr2	108425235	108425236	MACS_peak_8415	14.00	lobChIP_FOXA1inPBS
chr2	108809839	108809840	MACS_peak_8416	13.00	lobChIP_FOXA1inPBS
chr2	108817282	108817283	MACS_peak_8417	8.00	lobChIP_FOXA1inPBS
chr2	108882822	108882823	MACS_peak_8418	11.00	lobChIP_FOXA1inPBS
chr2	108888624	108888625	MACS_peak_8419	17.00	lobChIP_FOXA1inPBS
chr2	108905178	108905179	MACS_peak_8420	13.00	lobChIP_FOXA1inPBS
chr2	108986765	108986766	MACS_peak_8421	11.00	lobChIP_FOXA1inPBS
chr2	109002398	109002399	MACS_peak_8422	15.00	lobChIP_FOXA1inPBS
chr2	109149689	109149690	MACS_peak_8423	28.00	lobChIP_FOXA1inPBS
chr2	109300029	109300030	MACS_peak_8424	9.00	lobChIP_FOXA1inPBS
chr2	109636671	109636672	MACS_peak_8425	8.00	lobChIP_FOXA1inPBS
chr2	110381769	110381770	MACS_peak_8426	12.00	lobChIP_FOXA1inPBS
chr2	110402403	110402404	MACS_peak_8427	51.00	lobChIP_FOXA1inPBS
chr2	110438463	110438464	MACS_peak_8428	15.00	lobChIP_FOXA1inPBS
chr2	110448442	110448443	MACS_peak_8429	7.00	lobChIP_FOXA1inPBS
chr2	110864465	110864466	MACS_peak_8430	11.00	lobChIP_FOXA1inPBS
chr2	110903873	110903874	MACS_peak_8431	20.00	lobChIP_FOXA1inPBS
chr2	111526335	111526336	MACS_peak_8432	27.00	lobChIP_FOXA1inPBS
chr2	111765562	111765563	MACS_peak_8433	16.00	lobChIP_FOXA1inPBS
chr2	111878196	111878197	MACS_peak_8434	6.00	lobChIP_FOXA1inPBS
chr2	112049947	112049948	MACS_peak_8435	9.00	lobChIP_FOXA1inPBS
chr2	112176163	112176164	MACS_peak_8436	20.00	lobChIP_FOXA1inPBS
chr2	112221818	112221819	MACS_peak_8437	8.00	lobChIP_FOXA1inPBS
chr2	112280171	112280172	MACS_peak_8438	22.00	lobChIP_FOXA1inPBS
chr2	112293343	112293344	MACS_peak_8439	7.00	lobChIP_FOXA1inPBS
chr2	112597661	112597662	MACS_peak_8440	13.00	lobChIP_FOXA1inPBS
chr2	112645555	112645556	MACS_peak_8441	8.00	lobChIP_FOXA1inPBS
chr2	112676210	112676211	MACS_peak_8442	13.00	lobChIP_FOXA1inPBS
chr2	112702666	112702667	MACS_peak_8443	14.00	lobChIP_FOXA1inPBS
chr2	112712751	112712752	MACS_peak_8444	13.00	lobChIP_FOXA1inPBS
chr2	112732791	112732792	MACS_peak_8445	9.00	lobChIP_FOXA1inPBS
chr2	112745347	112745348	MACS_peak_8446	16.00	lobChIP_FOXA1inPBS
chr2	112752978	112752979	MACS_peak_8447	7.00	lobChIP_FOXA1inPBS
chr2	112842918	112842919	MACS_peak_8448	13.00	lobChIP_FOXA1inPBS
chr2	112927397	112927398	MACS_peak_8449	18.00	lobChIP_FOXA1inPBS
chr2	113033736	113033737	MACS_peak_8450	9.00	lobChIP_FOXA1inPBS
chr2	113072602	113072603	MACS_peak_8451	39.00	lobChIP_FOXA1inPBS
chr2	113399680	113399681	MACS_peak_8452	7.00	lobChIP_FOXA1inPBS
chr2	113401855	113401856	MACS_peak_8453	5.00	lobChIP_FOXA1inPBS
chr2	113519385	113519386	MACS_peak_8454	18.00	lobChIP_FOXA1inPBS
chr2	113561979	113561980	MACS_peak_8455	26.00	lobChIP_FOXA1inPBS
chr2	113575738	113575739	MACS_peak_8456	20.00	lobChIP_FOXA1inPBS
chr2	113751524	113751525	MACS_peak_8457	6.00	lobChIP_FOXA1inPBS
chr2	113846296	113846297	MACS_peak_8458	27.00	lobChIP_FOXA1inPBS
chr2	113884617	113884618	MACS_peak_8459	16.00	lobChIP_FOXA1inPBS
chr2	113896137	113896138	MACS_peak_8460	11.00	lobChIP_FOXA1inPBS
chr2	113923784	113923785	MACS_peak_8461	6.00	lobChIP_FOXA1inPBS
chr2	113925267	113925268	MACS_peak_8462	7.00	lobChIP_FOXA1inPBS
chr2	114466716	114466717	MACS_peak_8463	7.00	lobChIP_FOXA1inPBS
chr2	114511906	114511907	MACS_peak_8464	14.00	lobChIP_FOXA1inPBS
chr2	114576124	114576125	MACS_peak_8465	10.00	lobChIP_FOXA1inPBS
chr2	114652214	114652215	MACS_peak_8466	12.00	lobChIP_FOXA1inPBS
chr2	114658445	114658446	MACS_peak_8467	11.00	lobChIP_FOXA1inPBS
chr2	114735350	114735351	MACS_peak_8468	36.00	lobChIP_FOXA1inPBS
chr2	114764239	114764240	MACS_peak_8469	10.00	lobChIP_FOXA1inPBS
chr2	115308216	115308217	MACS_peak_8470	8.00	lobChIP_FOXA1inPBS
chr2	118586652	118586653	MACS_peak_8471	10.00	lobChIP_FOXA1inPBS
chr2	118691741	118691742	MACS_peak_8472	18.00	lobChIP_FOXA1inPBS
chr2	118804427	118804428	MACS_peak_8473	25.00	lobChIP_FOXA1inPBS
chr2	118843482	118843483	MACS_peak_8474	36.00	lobChIP_FOXA1inPBS
chr2	118848770	118848771	MACS_peak_8475	10.00	lobChIP_FOXA1inPBS
chr2	118870735	118870736	MACS_peak_8476	10.00	lobChIP_FOXA1inPBS
chr2	119079433	119079434	MACS_peak_8477	18.00	lobChIP_FOXA1inPBS
chr2	119994563	119994564	MACS_peak_8478	10.00	lobChIP_FOXA1inPBS
chr2	120027552	120027553	MACS_peak_8479	10.00	lobChIP_FOXA1inPBS
chr2	120062098	120062099	MACS_peak_8480	11.00	lobChIP_FOXA1inPBS
chr2	120109997	120109998	MACS_peak_8481	16.00	lobChIP_FOXA1inPBS
chr2	120123820	120123821	MACS_peak_8482	14.00	lobChIP_FOXA1inPBS
chr2	120183537	120183538	MACS_peak_8483	10.00	lobChIP_FOXA1inPBS
chr2	120227753	120227754	MACS_peak_8484	12.00	lobChIP_FOXA1inPBS
chr2	120436149	120436150	MACS_peak_8485	7.00	lobChIP_FOXA1inPBS
chr2	120452672	120452673	MACS_peak_8486	10.00	lobChIP_FOXA1inPBS
chr2	120523229	120523230	MACS_peak_8487	19.00	lobChIP_FOXA1inPBS
chr2	120526481	120526482	MACS_peak_8488	18.00	lobChIP_FOXA1inPBS
chr2	120568037	120568038	MACS_peak_8489	17.00	lobChIP_FOXA1inPBS
chr2	120774973	120774974	MACS_peak_8490	9.00	lobChIP_FOXA1inPBS
chr2	120824887	120824888	MACS_peak_8491	70.00	lobChIP_FOXA1inPBS
chr2	120891994	120891995	MACS_peak_8492	14.00	lobChIP_FOXA1inPBS
chr2	121070908	121070909	MACS_peak_8493	12.00	lobChIP_FOXA1inPBS
chr2	121106168	121106169	MACS_peak_8494	38.00	lobChIP_FOXA1inPBS
chr2	121156923	121156924	MACS_peak_8495	18.00	lobChIP_FOXA1inPBS
chr2	121199747	121199748	MACS_peak_8496	10.00	lobChIP_FOXA1inPBS
chr2	121221805	121221806	MACS_peak_8497	5.00	lobChIP_FOXA1inPBS
chr2	121223833	121223834	MACS_peak_8498	57.00	lobChIP_FOXA1inPBS
chr2	121228012	121228013	MACS_peak_8499	41.00	lobChIP_FOXA1inPBS
chr2	121261517	121261518	MACS_peak_8500	6.00	lobChIP_FOXA1inPBS
chr2	121297990	121297991	MACS_peak_8501	10.00	lobChIP_FOXA1inPBS
chr2	121303978	121303979	MACS_peak_8502	7.00	lobChIP_FOXA1inPBS
chr2	121322393	121322394	MACS_peak_8503	85.00	lobChIP_FOXA1inPBS
chr2	121377315	121377316	MACS_peak_8504	16.00	lobChIP_FOXA1inPBS
chr2	121427096	121427097	MACS_peak_8505	7.00	lobChIP_FOXA1inPBS
chr2	121692683	121692684	MACS_peak_8506	10.00	lobChIP_FOXA1inPBS
chr2	121696113	121696114	MACS_peak_8507	9.00	lobChIP_FOXA1inPBS
chr2	121772528	121772529	MACS_peak_8508	12.00	lobChIP_FOXA1inPBS
chr2	121793874	121793875	MACS_peak_8509	57.00	lobChIP_FOXA1inPBS
chr2	121867356	121867357	MACS_peak_8510	29.00	lobChIP_FOXA1inPBS
chr2	122040636	122040637	MACS_peak_8511	15.00	lobChIP_FOXA1inPBS
chr2	122113264	122113265	MACS_peak_8512	15.00	lobChIP_FOXA1inPBS
chr2	122325749	122325750	MACS_peak_8513	9.00	lobChIP_FOXA1inPBS
chr2	122338608	122338609	MACS_peak_8514	18.00	lobChIP_FOXA1inPBS
chr2	122355805	122355806	MACS_peak_8515	16.00	lobChIP_FOXA1inPBS
chr2	122366786	122366787	MACS_peak_8516	17.00	lobChIP_FOXA1inPBS
chr2	122446896	122446897	MACS_peak_8517	7.00	lobChIP_FOXA1inPBS
chr2	122514136	122514137	MACS_peak_8518	16.00	lobChIP_FOXA1inPBS
chr2	122534813	122534814	MACS_peak_8519	7.00	lobChIP_FOXA1inPBS
chr2	123190905	123190906	MACS_peak_8520	34.00	lobChIP_FOXA1inPBS
chr2	123506530	123506531	MACS_peak_8521	15.00	lobChIP_FOXA1inPBS
chr2	123772949	123772950	MACS_peak_8522	8.00	lobChIP_FOXA1inPBS
chr2	125324293	125324294	MACS_peak_8523	20.00	lobChIP_FOXA1inPBS
chr2	125328717	125328718	MACS_peak_8524	10.00	lobChIP_FOXA1inPBS
chr2	125844292	125844293	MACS_peak_8525	19.00	lobChIP_FOXA1inPBS
chr2	126771662	126771663	MACS_peak_8526	8.00	lobChIP_FOXA1inPBS
chr2	127748374	127748375	MACS_peak_8527	15.00	lobChIP_FOXA1inPBS
chr2	127754418	127754419	MACS_peak_8528	56.00	lobChIP_FOXA1inPBS
chr2	127873405	127873406	MACS_peak_8529	21.00	lobChIP_FOXA1inPBS
chr2	127984370	127984371	MACS_peak_8530	22.00	lobChIP_FOXA1inPBS
chr2	128115809	128115810	MACS_peak_8531	13.00	lobChIP_FOXA1inPBS
chr2	128143348	128143349	MACS_peak_8532	6.00	lobChIP_FOXA1inPBS
chr2	128168955	128168956	MACS_peak_8533	16.00	lobChIP_FOXA1inPBS
chr2	128171554	128171555	MACS_peak_8534	40.00	lobChIP_FOXA1inPBS
chr2	128175934	128175935	MACS_peak_8535	14.00	lobChIP_FOXA1inPBS
chr2	128176645	128176646	MACS_peak_8536	10.00	lobChIP_FOXA1inPBS
chr2	128196039	128196040	MACS_peak_8537	11.00	lobChIP_FOXA1inPBS
chr2	128212570	128212571	MACS_peak_8538	27.00	lobChIP_FOXA1inPBS
chr2	128258161	128258162	MACS_peak_8539	8.00	lobChIP_FOXA1inPBS
chr2	128462945	128462946	MACS_peak_8540	7.00	lobChIP_FOXA1inPBS
chr2	128493429	128493430	MACS_peak_8541	15.00	lobChIP_FOXA1inPBS
chr2	128542652	128542653	MACS_peak_8542	21.00	lobChIP_FOXA1inPBS
chr2	128742992	128742993	MACS_peak_8543	15.00	lobChIP_FOXA1inPBS
chr2	128927979	128927980	MACS_peak_8544	20.00	lobChIP_FOXA1inPBS
chr2	128929948	128929949	MACS_peak_8545	10.00	lobChIP_FOXA1inPBS
chr2	128936390	128936391	MACS_peak_8546	30.00	lobChIP_FOXA1inPBS
chr2	129064105	129064106	MACS_peak_8547	17.00	lobChIP_FOXA1inPBS
chr2	129136811	129136812	MACS_peak_8548	12.00	lobChIP_FOXA1inPBS
chr2	129352857	129352858	MACS_peak_8549	23.00	lobChIP_FOXA1inPBS
chr2	129513190	129513191	MACS_peak_8550	18.00	lobChIP_FOXA1inPBS
chr2	129649407	129649408	MACS_peak_8551	8.00	lobChIP_FOXA1inPBS
chr2	130998898	130998899	MACS_peak_8552	7.00	lobChIP_FOXA1inPBS
chr2	131502318	131502319	MACS_peak_8553	12.00	lobChIP_FOXA1inPBS
chr2	131642829	131642830	MACS_peak_8554	13.00	lobChIP_FOXA1inPBS
chr2	132250316	132250317	MACS_peak_8555	7.00	lobChIP_FOXA1inPBS
chr2	132496718	132496719	MACS_peak_8556	13.00	lobChIP_FOXA1inPBS
chr2	133176430	133176431	MACS_peak_8558	5.00	lobChIP_FOXA1inPBS
chr2	133915813	133915814	MACS_peak_8559	13.00	lobChIP_FOXA1inPBS
chr2	134243628	134243629	MACS_peak_8560	11.00	lobChIP_FOXA1inPBS
chr2	134523522	134523523	MACS_peak_8561	22.00	lobChIP_FOXA1inPBS
chr2	134857071	134857072	MACS_peak_8562	14.00	lobChIP_FOXA1inPBS
chr2	134890201	134890202	MACS_peak_8563	8.00	lobChIP_FOXA1inPBS
chr2	135169896	135169897	MACS_peak_8564	52.00	lobChIP_FOXA1inPBS
chr2	135190195	135190196	MACS_peak_8565	21.00	lobChIP_FOXA1inPBS
chr2	135581194	135581195	MACS_peak_8566	28.00	lobChIP_FOXA1inPBS
chr2	135849434	135849435	MACS_peak_8567	33.00	lobChIP_FOXA1inPBS
chr2	135908437	135908438	MACS_peak_8568	13.00	lobChIP_FOXA1inPBS
chr2	136269866	136269867	MACS_peak_8569	11.00	lobChIP_FOXA1inPBS
chr2	136328568	136328569	MACS_peak_8570	13.00	lobChIP_FOXA1inPBS
chr2	136432176	136432177	MACS_peak_8571	8.00	lobChIP_FOXA1inPBS
chr2	136633934	136633935	MACS_peak_8572	9.00	lobChIP_FOXA1inPBS
chr2	136720188	136720189	MACS_peak_8573	9.00	lobChIP_FOXA1inPBS
chr2	136781104	136781105	MACS_peak_8574	10.00	lobChIP_FOXA1inPBS
chr2	136862712	136862713	MACS_peak_8575	8.00	lobChIP_FOXA1inPBS
chr2	137875083	137875084	MACS_peak_8576	16.00	lobChIP_FOXA1inPBS
chr2	138226424	138226425	MACS_peak_8577	7.00	lobChIP_FOXA1inPBS
chr2	138686599	138686600	MACS_peak_8578	65.00	lobChIP_FOXA1inPBS
chr2	138700724	138700725	MACS_peak_8579	45.00	lobChIP_FOXA1inPBS
chr2	138721874	138721875	MACS_peak_8580	49.00	lobChIP_FOXA1inPBS
chr2	138723241	138723242	MACS_peak_8581	7.00	lobChIP_FOXA1inPBS
chr2	138726420	138726421	MACS_peak_8582	7.00	lobChIP_FOXA1inPBS
chr2	138817831	138817832	MACS_peak_8583	9.00	lobChIP_FOXA1inPBS
chr2	138839475	138839476	MACS_peak_8584	15.00	lobChIP_FOXA1inPBS
chr2	138868216	138868217	MACS_peak_8585	24.00	lobChIP_FOXA1inPBS
chr2	138895818	138895819	MACS_peak_8586	10.00	lobChIP_FOXA1inPBS
chr2	138934790	138934791	MACS_peak_8587	11.00	lobChIP_FOXA1inPBS
chr2	139034787	139034788	MACS_peak_8588	9.00	lobChIP_FOXA1inPBS
chr2	139039772	139039773	MACS_peak_8589	24.00	lobChIP_FOXA1inPBS
chr2	139179087	139179088	MACS_peak_8590	6.00	lobChIP_FOXA1inPBS
chr2	139233077	139233078	MACS_peak_8591	14.00	lobChIP_FOXA1inPBS
chr2	139248957	139248958	MACS_peak_8592	31.00	lobChIP_FOXA1inPBS
chr2	139250001	139250002	MACS_peak_8593	10.00	lobChIP_FOXA1inPBS
chr2	139270324	139270325	MACS_peak_8594	10.00	lobChIP_FOXA1inPBS
chr2	139272780	139272781	MACS_peak_8595	14.00	lobChIP_FOXA1inPBS
chr2	139471480	139471481	MACS_peak_8596	10.00	lobChIP_FOXA1inPBS
chr2	139482796	139482797	MACS_peak_8597	20.00	lobChIP_FOXA1inPBS
chr2	139535756	139535757	MACS_peak_8598	18.00	lobChIP_FOXA1inPBS
chr2	139572268	139572269	MACS_peak_8599	14.00	lobChIP_FOXA1inPBS
chr2	139603976	139603977	MACS_peak_8600	9.00	lobChIP_FOXA1inPBS
chr2	139700199	139700200	MACS_peak_8601	12.00	lobChIP_FOXA1inPBS
chr2	140017157	140017158	MACS_peak_8602	12.00	lobChIP_FOXA1inPBS
chr2	142275282	142275283	MACS_peak_8603	12.00	lobChIP_FOXA1inPBS
chr2	143360371	143360372	MACS_peak_8604	12.00	lobChIP_FOXA1inPBS
chr2	143615570	143615571	MACS_peak_8605	9.00	lobChIP_FOXA1inPBS
chr2	143621275	143621276	MACS_peak_8606	53.00	lobChIP_FOXA1inPBS
chr2	143632195	143632196	MACS_peak_8607	21.00	lobChIP_FOXA1inPBS
chr2	143635033	143635034	MACS_peak_8608	9.00	lobChIP_FOXA1inPBS
chr2	143636119	143636120	MACS_peak_8609	11.00	lobChIP_FOXA1inPBS
chr2	143709194	143709195	MACS_peak_8610	20.00	lobChIP_FOXA1inPBS
chr2	143710533	143710534	MACS_peak_8611	8.00	lobChIP_FOXA1inPBS
chr2	143738304	143738305	MACS_peak_8612	14.00	lobChIP_FOXA1inPBS
chr2	143739919	143739920	MACS_peak_8613	7.00	lobChIP_FOXA1inPBS
chr2	144051834	144051835	MACS_peak_8614	48.00	lobChIP_FOXA1inPBS
chr2	144708629	144708630	MACS_peak_8615	12.00	lobChIP_FOXA1inPBS
chr2	146515752	146515753	MACS_peak_8616	10.00	lobChIP_FOXA1inPBS
chr2	146519108	146519109	MACS_peak_8617	40.00	lobChIP_FOXA1inPBS
chr2	146558117	146558118	MACS_peak_8618	46.00	lobChIP_FOXA1inPBS
chr2	146580938	146580939	MACS_peak_8619	66.00	lobChIP_FOXA1inPBS
chr2	146602959	146602960	MACS_peak_8620	24.00	lobChIP_FOXA1inPBS
chr2	146972205	146972206	MACS_peak_8621	12.00	lobChIP_FOXA1inPBS
chr2	147840667	147840668	MACS_peak_8622	17.00	lobChIP_FOXA1inPBS
chr2	148567243	148567244	MACS_peak_8623	12.00	lobChIP_FOXA1inPBS
chr2	148609357	148609358	MACS_peak_8624	9.00	lobChIP_FOXA1inPBS
chr2	148684828	148684829	MACS_peak_8625	22.00	lobChIP_FOXA1inPBS
chr2	149002224	149002225	MACS_peak_8626	10.00	lobChIP_FOXA1inPBS
chr2	149168663	149168664	MACS_peak_8627	21.00	lobChIP_FOXA1inPBS
chr2	149259258	149259259	MACS_peak_8628	13.00	lobChIP_FOXA1inPBS
chr2	149403075	149403076	MACS_peak_8629	15.00	lobChIP_FOXA1inPBS
chr2	149476973	149476974	MACS_peak_8630	11.00	lobChIP_FOXA1inPBS
chr2	149504723	149504724	MACS_peak_8631	12.00	lobChIP_FOXA1inPBS
chr2	149877108	149877109	MACS_peak_8632	22.00	lobChIP_FOXA1inPBS
chr2	149981778	149981779	MACS_peak_8633	23.00	lobChIP_FOXA1inPBS
chr2	150419053	150419054	MACS_peak_8634	18.00	lobChIP_FOXA1inPBS
chr2	150441996	150441997	MACS_peak_8635	9.00	lobChIP_FOXA1inPBS
chr2	150846170	150846171	MACS_peak_8636	17.00	lobChIP_FOXA1inPBS
chr2	151071316	151071317	MACS_peak_8637	12.00	lobChIP_FOXA1inPBS
chr2	152271035	152271036	MACS_peak_8638	11.00	lobChIP_FOXA1inPBS
chr2	152607207	152607208	MACS_peak_8639	8.00	lobChIP_FOXA1inPBS
chr2	152607791	152607792	MACS_peak_8640	7.00	lobChIP_FOXA1inPBS
chr2	152616875	152616876	MACS_peak_8641	13.00	lobChIP_FOXA1inPBS
chr2	152665539	152665540	MACS_peak_8642	8.00	lobChIP_FOXA1inPBS
chr2	152706449	152706450	MACS_peak_8643	15.00	lobChIP_FOXA1inPBS
chr2	152709716	152709717	MACS_peak_8644	54.00	lobChIP_FOXA1inPBS
chr2	152852389	152852390	MACS_peak_8645	41.00	lobChIP_FOXA1inPBS
chr2	152882333	152882334	MACS_peak_8646	6.00	lobChIP_FOXA1inPBS
chr2	153028339	153028340	MACS_peak_8647	14.00	lobChIP_FOXA1inPBS
chr2	153049780	153049781	MACS_peak_8648	8.00	lobChIP_FOXA1inPBS
chr2	153199391	153199392	MACS_peak_8649	18.00	lobChIP_FOXA1inPBS
chr2	153201288	153201289	MACS_peak_8650	33.00	lobChIP_FOXA1inPBS
chr2	153207028	153207029	MACS_peak_8651	8.00	lobChIP_FOXA1inPBS
chr2	153212160	153212161	MACS_peak_8652	13.00	lobChIP_FOXA1inPBS
chr2	153216317	153216318	MACS_peak_8653	19.00	lobChIP_FOXA1inPBS
chr2	153234713	153234714	MACS_peak_8654	28.00	lobChIP_FOXA1inPBS
chr2	153261935	153261936	MACS_peak_8655	19.00	lobChIP_FOXA1inPBS
chr2	153266701	153266702	MACS_peak_8656	9.00	lobChIP_FOXA1inPBS
chr2	153282675	153282676	MACS_peak_8657	13.00	lobChIP_FOXA1inPBS
chr2	153338610	153338611	MACS_peak_8658	13.00	lobChIP_FOXA1inPBS
chr2	153342874	153342875	MACS_peak_8659	64.00	lobChIP_FOXA1inPBS
chr2	153504946	153504947	MACS_peak_8660	11.00	lobChIP_FOXA1inPBS
chr2	153506565	153506566	MACS_peak_8661	13.00	lobChIP_FOXA1inPBS
chr2	153540573	153540574	MACS_peak_8662	7.00	lobChIP_FOXA1inPBS
chr2	153576851	153576852	MACS_peak_8663	7.00	lobChIP_FOXA1inPBS
chr2	153718086	153718087	MACS_peak_8664	17.00	lobChIP_FOXA1inPBS
chr2	153768786	153768787	MACS_peak_8665	11.00	lobChIP_FOXA1inPBS
chr2	153916383	153916384	MACS_peak_8666	12.00	lobChIP_FOXA1inPBS
chr2	154691400	154691401	MACS_peak_8667	14.00	lobChIP_FOXA1inPBS
chr2	155304359	155304360	MACS_peak_8668	18.00	lobChIP_FOXA1inPBS
chr2	155306544	155306545	MACS_peak_8669	51.00	lobChIP_FOXA1inPBS
chr2	155319452	155319453	MACS_peak_8670	14.00	lobChIP_FOXA1inPBS
chr2	156047024	156047025	MACS_peak_8671	8.00	lobChIP_FOXA1inPBS
chr2	156672544	156672545	MACS_peak_8672	15.00	lobChIP_FOXA1inPBS
chr2	156832489	156832490	MACS_peak_8673	14.00	lobChIP_FOXA1inPBS
chr2	157045857	157045858	MACS_peak_8674	34.00	lobChIP_FOXA1inPBS
chr2	157157022	157157023	MACS_peak_8675	19.00	lobChIP_FOXA1inPBS
chr2	157192253	157192254	MACS_peak_8676	13.00	lobChIP_FOXA1inPBS
chr2	157515738	157515739	MACS_peak_8677	8.00	lobChIP_FOXA1inPBS
chr2	157909294	157909295	MACS_peak_8678	7.00	lobChIP_FOXA1inPBS
chr2	157912746	157912747	MACS_peak_8679	6.00	lobChIP_FOXA1inPBS
chr2	158449724	158449725	MACS_peak_8680	7.00	lobChIP_FOXA1inPBS
chr2	158722992	158722993	MACS_peak_8681	11.00	lobChIP_FOXA1inPBS
chr2	158936386	158936387	MACS_peak_8682	8.00	lobChIP_FOXA1inPBS
chr2	158952595	158952596	MACS_peak_8683	14.00	lobChIP_FOXA1inPBS
chr2	158984132	158984133	MACS_peak_8684	20.00	lobChIP_FOXA1inPBS
chr2	158985146	158985147	MACS_peak_8685	9.00	lobChIP_FOXA1inPBS
chr2	158987641	158987642	MACS_peak_8686	34.00	lobChIP_FOXA1inPBS
chr2	158988678	158988679	MACS_peak_8687	18.00	lobChIP_FOXA1inPBS
chr2	159041559	159041560	MACS_peak_8688	10.00	lobChIP_FOXA1inPBS
chr2	159046211	159046212	MACS_peak_8689	25.00	lobChIP_FOXA1inPBS
chr2	159047740	159047741	MACS_peak_8690	34.00	lobChIP_FOXA1inPBS
chr2	159089125	159089126	MACS_peak_8691	24.00	lobChIP_FOXA1inPBS
chr2	159308683	159308684	MACS_peak_8692	7.00	lobChIP_FOXA1inPBS
chr2	159314719	159314720	MACS_peak_8693	10.00	lobChIP_FOXA1inPBS
chr2	159354153	159354154	MACS_peak_8694	8.00	lobChIP_FOXA1inPBS
chr2	159359778	159359779	MACS_peak_8695	13.00	lobChIP_FOXA1inPBS
chr2	159360779	159360780	MACS_peak_8696	16.00	lobChIP_FOXA1inPBS
chr2	159443164	159443165	MACS_peak_8697	11.00	lobChIP_FOXA1inPBS
chr2	159580594	159580595	MACS_peak_8698	18.00	lobChIP_FOXA1inPBS
chr2	159592522	159592523	MACS_peak_8699	13.00	lobChIP_FOXA1inPBS
chr2	159596103	159596104	MACS_peak_8700	10.00	lobChIP_FOXA1inPBS
chr2	159805275	159805276	MACS_peak_8701	8.00	lobChIP_FOXA1inPBS
chr2	159806013	159806014	MACS_peak_8702	17.00	lobChIP_FOXA1inPBS
chr2	159851655	159851656	MACS_peak_8703	76.00	lobChIP_FOXA1inPBS
chr2	159853792	159853793	MACS_peak_8704	9.00	lobChIP_FOXA1inPBS
chr2	159855643	159855644	MACS_peak_8705	46.00	lobChIP_FOXA1inPBS
chr2	159867098	159867099	MACS_peak_8706	10.00	lobChIP_FOXA1inPBS
chr2	159981161	159981162	MACS_peak_8707	9.00	lobChIP_FOXA1inPBS
chr2	159998839	159998840	MACS_peak_8708	9.00	lobChIP_FOXA1inPBS
chr2	160256776	160256777	MACS_peak_8709	11.00	lobChIP_FOXA1inPBS
chr2	160259418	160259419	MACS_peak_8710	7.00	lobChIP_FOXA1inPBS
chr2	160654161	160654162	MACS_peak_8711	20.00	lobChIP_FOXA1inPBS
chr2	160655037	160655038	MACS_peak_8712	12.00	lobChIP_FOXA1inPBS
chr2	160656573	160656574	MACS_peak_8713	16.00	lobChIP_FOXA1inPBS
chr2	160664163	160664164	MACS_peak_8714	15.00	lobChIP_FOXA1inPBS
chr2	160678906	160678907	MACS_peak_8715	23.00	lobChIP_FOXA1inPBS
chr2	161037856	161037857	MACS_peak_8716	8.00	lobChIP_FOXA1inPBS
chr2	161174913	161174914	MACS_peak_8717	8.00	lobChIP_FOXA1inPBS
chr2	161181567	161181568	MACS_peak_8718	12.00	lobChIP_FOXA1inPBS
chr2	161286030	161286031	MACS_peak_8719	8.00	lobChIP_FOXA1inPBS
chr2	161290439	161290440	MACS_peak_8720	17.00	lobChIP_FOXA1inPBS
chr2	161290982	161290983	MACS_peak_8721	19.00	lobChIP_FOXA1inPBS
chr2	161307800	161307801	MACS_peak_8722	8.00	lobChIP_FOXA1inPBS
chr2	161347562	161347563	MACS_peak_8723	7.00	lobChIP_FOXA1inPBS
chr2	161349013	161349014	MACS_peak_8724	15.00	lobChIP_FOXA1inPBS
chr2	161991957	161991958	MACS_peak_8725	29.00	lobChIP_FOXA1inPBS
chr2	162016604	162016605	MACS_peak_8726	61.00	lobChIP_FOXA1inPBS
chr2	162017493	162017494	MACS_peak_8727	11.00	lobChIP_FOXA1inPBS
chr2	162129897	162129898	MACS_peak_8728	11.00	lobChIP_FOXA1inPBS
chr2	162499565	162499566	MACS_peak_8729	9.00	lobChIP_FOXA1inPBS
chr2	162701527	162701528	MACS_peak_8730	44.00	lobChIP_FOXA1inPBS
chr2	162941855	162941856	MACS_peak_8731	17.00	lobChIP_FOXA1inPBS
chr2	162964318	162964319	MACS_peak_8732	17.00	lobChIP_FOXA1inPBS
chr2	162967698	162967699	MACS_peak_8733	9.00	lobChIP_FOXA1inPBS
chr2	162969088	162969089	MACS_peak_8734	9.00	lobChIP_FOXA1inPBS
chr2	163422394	163422395	MACS_peak_8735	9.00	lobChIP_FOXA1inPBS
chr2	163431001	163431002	MACS_peak_8736	13.00	lobChIP_FOXA1inPBS
chr2	163436690	163436691	MACS_peak_8737	19.00	lobChIP_FOXA1inPBS
chr2	163439154	163439155	MACS_peak_8738	68.00	lobChIP_FOXA1inPBS
chr2	163464618	163464619	MACS_peak_8739	10.00	lobChIP_FOXA1inPBS
chr2	163566987	163566988	MACS_peak_8740	23.00	lobChIP_FOXA1inPBS
chr2	163606742	163606743	MACS_peak_8741	16.00	lobChIP_FOXA1inPBS
chr2	164331077	164331078	MACS_peak_8742	17.00	lobChIP_FOXA1inPBS
chr2	164428144	164428145	MACS_peak_8743	19.00	lobChIP_FOXA1inPBS
chr2	164497522	164497523	MACS_peak_8744	7.00	lobChIP_FOXA1inPBS
chr2	164523890	164523891	MACS_peak_8745	12.00	lobChIP_FOXA1inPBS
chr2	164574213	164574214	MACS_peak_8746	7.00	lobChIP_FOXA1inPBS
chr2	164591940	164591941	MACS_peak_8747	45.00	lobChIP_FOXA1inPBS
chr2	165416934	165416935	MACS_peak_8748	28.00	lobChIP_FOXA1inPBS
chr2	165424321	165424322	MACS_peak_8749	34.00	lobChIP_FOXA1inPBS
chr2	165518061	165518062	MACS_peak_8750	9.00	lobChIP_FOXA1inPBS
chr2	165550151	165550152	MACS_peak_8751	51.00	lobChIP_FOXA1inPBS
chr2	165568065	165568066	MACS_peak_8752	26.00	lobChIP_FOXA1inPBS
chr2	165599416	165599417	MACS_peak_8753	9.00	lobChIP_FOXA1inPBS
chr2	165601632	165601633	MACS_peak_8754	10.00	lobChIP_FOXA1inPBS
chr2	165614634	165614635	MACS_peak_8755	15.00	lobChIP_FOXA1inPBS
chr2	165659048	165659049	MACS_peak_8756	15.00	lobChIP_FOXA1inPBS
chr2	165667459	165667460	MACS_peak_8757	73.00	lobChIP_FOXA1inPBS
chr2	165675095	165675096	MACS_peak_8758	11.00	lobChIP_FOXA1inPBS
chr2	165679841	165679842	MACS_peak_8759	7.00	lobChIP_FOXA1inPBS
chr2	165696979	165696980	MACS_peak_8760	9.00	lobChIP_FOXA1inPBS
chr2	165735333	165735334	MACS_peak_8761	17.00	lobChIP_FOXA1inPBS
chr2	165744098	165744099	MACS_peak_8762	18.00	lobChIP_FOXA1inPBS
chr2	165755142	165755143	MACS_peak_8763	9.00	lobChIP_FOXA1inPBS
chr2	165768538	165768539	MACS_peak_8764	19.00	lobChIP_FOXA1inPBS
chr2	165790391	165790392	MACS_peak_8765	16.00	lobChIP_FOXA1inPBS
chr2	165791600	165791601	MACS_peak_8766	7.00	lobChIP_FOXA1inPBS
chr2	165794815	165794816	MACS_peak_8767	9.00	lobChIP_FOXA1inPBS
chr2	165967045	165967046	MACS_peak_8768	48.00	lobChIP_FOXA1inPBS
chr2	166634890	166634891	MACS_peak_8769	15.00	lobChIP_FOXA1inPBS
chr2	166683797	166683798	MACS_peak_8770	13.00	lobChIP_FOXA1inPBS
chr2	166984543	166984544	MACS_peak_8771	10.00	lobChIP_FOXA1inPBS
chr2	166991873	166991874	MACS_peak_8772	59.00	lobChIP_FOXA1inPBS
chr2	167020730	167020731	MACS_peak_8773	8.00	lobChIP_FOXA1inPBS
chr2	167086357	167086358	MACS_peak_8774	28.00	lobChIP_FOXA1inPBS
chr2	167228779	167228780	MACS_peak_8775	17.00	lobChIP_FOXA1inPBS
chr2	167275482	167275483	MACS_peak_8776	6.00	lobChIP_FOXA1inPBS
chr2	167465865	167465866	MACS_peak_8777	25.00	lobChIP_FOXA1inPBS
chr2	167960118	167960119	MACS_peak_8778	12.00	lobChIP_FOXA1inPBS
chr2	168367055	168367056	MACS_peak_8779	56.00	lobChIP_FOXA1inPBS
chr2	168651216	168651217	MACS_peak_8780	39.00	lobChIP_FOXA1inPBS
chr2	168672076	168672077	MACS_peak_8781	37.00	lobChIP_FOXA1inPBS
chr2	168673519	168673520	MACS_peak_8782	23.00	lobChIP_FOXA1inPBS
chr2	168684232	168684233	MACS_peak_8783	19.00	lobChIP_FOXA1inPBS
chr2	169006390	169006391	MACS_peak_8784	9.00	lobChIP_FOXA1inPBS
chr2	169023634	169023635	MACS_peak_8785	8.00	lobChIP_FOXA1inPBS
chr2	169037671	169037672	MACS_peak_8786	13.00	lobChIP_FOXA1inPBS
chr2	169042236	169042237	MACS_peak_8787	7.00	lobChIP_FOXA1inPBS
chr2	169049195	169049196	MACS_peak_8788	10.00	lobChIP_FOXA1inPBS
chr2	169077830	169077831	MACS_peak_8789	29.00	lobChIP_FOXA1inPBS
chr2	169089502	169089503	MACS_peak_8790	8.00	lobChIP_FOXA1inPBS
chr2	169128929	169128930	MACS_peak_8791	19.00	lobChIP_FOXA1inPBS
chr2	169135323	169135324	MACS_peak_8792	11.00	lobChIP_FOXA1inPBS
chr2	169324381	169324382	MACS_peak_8793	27.00	lobChIP_FOXA1inPBS
chr2	169351019	169351020	MACS_peak_8794	20.00	lobChIP_FOXA1inPBS
chr2	169479203	169479204	MACS_peak_8795	11.00	lobChIP_FOXA1inPBS
chr2	170430348	170430349	MACS_peak_8796	10.00	lobChIP_FOXA1inPBS
chr2	170498031	170498032	MACS_peak_8797	8.00	lobChIP_FOXA1inPBS
chr2	170540292	170540293	MACS_peak_8798	27.00	lobChIP_FOXA1inPBS
chr2	170572255	170572256	MACS_peak_8799	40.00	lobChIP_FOXA1inPBS
chr2	170573951	170573952	MACS_peak_8800	18.00	lobChIP_FOXA1inPBS
chr2	170589531	170589532	MACS_peak_8801	9.00	lobChIP_FOXA1inPBS
chr2	170860113	170860114	MACS_peak_8802	9.00	lobChIP_FOXA1inPBS
chr2	171312769	171312770	MACS_peak_8803	59.00	lobChIP_FOXA1inPBS
chr2	171342009	171342010	MACS_peak_8804	12.00	lobChIP_FOXA1inPBS
chr2	171386930	171386931	MACS_peak_8805	42.00	lobChIP_FOXA1inPBS
chr2	171568950	171568951	MACS_peak_8806	15.00	lobChIP_FOXA1inPBS
chr2	171571606	171571607	MACS_peak_8807	46.00	lobChIP_FOXA1inPBS
chr2	171600880	171600881	MACS_peak_8808	6.00	lobChIP_FOXA1inPBS
chr2	171943586	171943587	MACS_peak_8809	7.00	lobChIP_FOXA1inPBS
chr2	171946127	171946128	MACS_peak_8810	11.00	lobChIP_FOXA1inPBS
chr2	172006581	172006582	MACS_peak_8811	20.00	lobChIP_FOXA1inPBS
chr2	172023792	172023793	MACS_peak_8812	20.00	lobChIP_FOXA1inPBS
chr2	172128678	172128679	MACS_peak_8813	16.00	lobChIP_FOXA1inPBS
chr2	172285406	172285407	MACS_peak_8814	16.00	lobChIP_FOXA1inPBS
chr2	172585989	172585990	MACS_peak_8815	67.00	lobChIP_FOXA1inPBS
chr2	172615485	172615486	MACS_peak_8816	12.00	lobChIP_FOXA1inPBS
chr2	172629082	172629083	MACS_peak_8817	28.00	lobChIP_FOXA1inPBS
chr2	172664933	172664934	MACS_peak_8818	29.00	lobChIP_FOXA1inPBS
chr2	172746743	172746744	MACS_peak_8819	12.00	lobChIP_FOXA1inPBS
chr2	172917191	172917192	MACS_peak_8820	35.00	lobChIP_FOXA1inPBS
chr2	172938482	172938483	MACS_peak_8821	23.00	lobChIP_FOXA1inPBS
chr2	172953636	172953637	MACS_peak_8822	16.00	lobChIP_FOXA1inPBS
chr2	173205960	173205961	MACS_peak_8823	25.00	lobChIP_FOXA1inPBS
chr2	173293406	173293407	MACS_peak_8824	12.00	lobChIP_FOXA1inPBS
chr2	173299629	173299630	MACS_peak_8825	8.00	lobChIP_FOXA1inPBS
chr2	173300330	173300331	MACS_peak_8826	7.00	lobChIP_FOXA1inPBS
chr2	173903141	173903142	MACS_peak_8827	16.00	lobChIP_FOXA1inPBS
chr2	173913819	173913820	MACS_peak_8828	15.00	lobChIP_FOXA1inPBS
chr2	173953035	173953036	MACS_peak_8829	37.00	lobChIP_FOXA1inPBS
chr2	173974233	173974234	MACS_peak_8830	13.00	lobChIP_FOXA1inPBS
chr2	174103238	174103239	MACS_peak_8831	9.00	lobChIP_FOXA1inPBS
chr2	174212281	174212282	MACS_peak_8832	16.00	lobChIP_FOXA1inPBS
chr2	174328430	174328431	MACS_peak_8833	11.00	lobChIP_FOXA1inPBS
chr2	174599848	174599849	MACS_peak_8834	7.00	lobChIP_FOXA1inPBS
chr2	174834809	174834810	MACS_peak_8835	9.00	lobChIP_FOXA1inPBS
chr2	174851680	174851681	MACS_peak_8836	14.00	lobChIP_FOXA1inPBS
chr2	175098629	175098630	MACS_peak_8837	36.00	lobChIP_FOXA1inPBS
chr2	175198591	175198592	MACS_peak_8838	7.00	lobChIP_FOXA1inPBS
chr2	175293142	175293143	MACS_peak_8839	24.00	lobChIP_FOXA1inPBS
chr2	175481953	175481954	MACS_peak_8840	21.00	lobChIP_FOXA1inPBS
chr2	175893300	175893301	MACS_peak_8841	28.00	lobChIP_FOXA1inPBS
chr2	175901479	175901480	MACS_peak_8842	32.00	lobChIP_FOXA1inPBS
chr2	175927895	175927896	MACS_peak_8843	83.00	lobChIP_FOXA1inPBS
chr2	175928611	175928612	MACS_peak_8844	10.00	lobChIP_FOXA1inPBS
chr2	176032830	176032831	MACS_peak_8845	11.00	lobChIP_FOXA1inPBS
chr2	176470464	176470465	MACS_peak_8846	30.00	lobChIP_FOXA1inPBS
chr2	176471378	176471379	MACS_peak_8847	58.00	lobChIP_FOXA1inPBS
chr2	176495750	176495751	MACS_peak_8848	12.00	lobChIP_FOXA1inPBS
chr2	176778186	176778187	MACS_peak_8849	7.00	lobChIP_FOXA1inPBS
chr2	176778901	176778902	MACS_peak_8850	12.00	lobChIP_FOXA1inPBS
chr2	176812604	176812605	MACS_peak_8851	11.00	lobChIP_FOXA1inPBS
chr2	177052165	177052166	MACS_peak_8852	25.00	lobChIP_FOXA1inPBS
chr2	177517224	177517225	MACS_peak_8853	9.00	lobChIP_FOXA1inPBS
chr2	178035032	178035033	MACS_peak_8854	15.00	lobChIP_FOXA1inPBS
chr2	178039497	178039498	MACS_peak_8855	10.00	lobChIP_FOXA1inPBS
chr2	178052284	178052285	MACS_peak_8856	10.00	lobChIP_FOXA1inPBS
chr2	178053786	178053787	MACS_peak_8857	12.00	lobChIP_FOXA1inPBS
chr2	178068126	178068127	MACS_peak_8858	10.00	lobChIP_FOXA1inPBS
chr2	178128174	178128175	MACS_peak_8859	6.00	lobChIP_FOXA1inPBS
chr2	178129767	178129768	MACS_peak_8860	23.00	lobChIP_FOXA1inPBS
chr2	178131329	178131330	MACS_peak_8861	12.00	lobChIP_FOXA1inPBS
chr2	178134098	178134099	MACS_peak_8862	11.00	lobChIP_FOXA1inPBS
chr2	178160951	178160952	MACS_peak_8863	13.00	lobChIP_FOXA1inPBS
chr2	178259124	178259125	MACS_peak_8864	18.00	lobChIP_FOXA1inPBS
chr2	178261611	178261612	MACS_peak_8865	49.00	lobChIP_FOXA1inPBS
chr2	178262890	178262891	MACS_peak_8866	37.00	lobChIP_FOXA1inPBS
chr2	178269981	178269982	MACS_peak_8867	18.00	lobChIP_FOXA1inPBS
chr2	178444575	178444576	MACS_peak_8868	17.00	lobChIP_FOXA1inPBS
chr2	178586004	178586005	MACS_peak_8869	14.00	lobChIP_FOXA1inPBS
chr2	178836007	178836008	MACS_peak_8870	24.00	lobChIP_FOXA1inPBS
chr2	179058413	179058414	MACS_peak_8871	61.00	lobChIP_FOXA1inPBS
chr2	179180137	179180138	MACS_peak_8872	6.00	lobChIP_FOXA1inPBS
chr2	179334380	179334381	MACS_peak_8873	13.00	lobChIP_FOXA1inPBS
chr2	180044109	180044110	MACS_peak_8874	9.00	lobChIP_FOXA1inPBS
chr2	180056079	180056080	MACS_peak_8875	22.00	lobChIP_FOXA1inPBS
chr2	180103555	180103556	MACS_peak_8876	7.00	lobChIP_FOXA1inPBS
chr2	180126459	180126460	MACS_peak_8877	7.00	lobChIP_FOXA1inPBS
chr2	180137232	180137233	MACS_peak_8878	16.00	lobChIP_FOXA1inPBS
chr2	180320752	180320753	MACS_peak_8879	10.00	lobChIP_FOXA1inPBS
chr2	180383120	180383121	MACS_peak_8880	7.00	lobChIP_FOXA1inPBS
chr2	180408259	180408260	MACS_peak_8881	46.00	lobChIP_FOXA1inPBS
chr2	180439143	180439144	MACS_peak_8882	17.00	lobChIP_FOXA1inPBS
chr2	180496627	180496628	MACS_peak_8883	10.00	lobChIP_FOXA1inPBS
chr2	180812972	180812973	MACS_peak_8884	10.00	lobChIP_FOXA1inPBS
chr2	181119742	181119743	MACS_peak_8885	12.00	lobChIP_FOXA1inPBS
chr2	181154537	181154538	MACS_peak_8886	18.00	lobChIP_FOXA1inPBS
chr2	181472381	181472382	MACS_peak_8887	20.00	lobChIP_FOXA1inPBS
chr2	181628495	181628496	MACS_peak_8888	88.00	lobChIP_FOXA1inPBS
chr2	182104321	182104322	MACS_peak_8889	8.00	lobChIP_FOXA1inPBS
chr2	182655806	182655807	MACS_peak_8890	14.00	lobChIP_FOXA1inPBS
chr2	182668998	182668999	MACS_peak_8891	12.00	lobChIP_FOXA1inPBS
chr2	182675622	182675623	MACS_peak_8892	11.00	lobChIP_FOXA1inPBS
chr2	182757588	182757589	MACS_peak_8893	12.00	lobChIP_FOXA1inPBS
chr2	182765271	182765272	MACS_peak_8894	18.00	lobChIP_FOXA1inPBS
chr2	182771880	182771881	MACS_peak_8895	9.00	lobChIP_FOXA1inPBS
chr2	182864225	182864226	MACS_peak_8896	13.00	lobChIP_FOXA1inPBS
chr2	183482031	183482032	MACS_peak_8897	16.00	lobChIP_FOXA1inPBS
chr2	183679876	183679877	MACS_peak_8898	7.00	lobChIP_FOXA1inPBS
chr2	183756156	183756157	MACS_peak_8899	13.00	lobChIP_FOXA1inPBS
chr2	184871967	184871968	MACS_peak_8900	15.00	lobChIP_FOXA1inPBS
chr2	186183296	186183297	MACS_peak_8901	22.00	lobChIP_FOXA1inPBS
chr2	186245679	186245680	MACS_peak_8902	8.00	lobChIP_FOXA1inPBS
chr2	186678554	186678555	MACS_peak_8903	47.00	lobChIP_FOXA1inPBS
chr2	186853775	186853776	MACS_peak_8904	13.00	lobChIP_FOXA1inPBS
chr2	186994809	186994810	MACS_peak_8905	19.00	lobChIP_FOXA1inPBS
chr2	187046541	187046542	MACS_peak_8906	33.00	lobChIP_FOXA1inPBS
chr2	187206954	187206955	MACS_peak_8907	17.00	lobChIP_FOXA1inPBS
chr2	187247779	187247780	MACS_peak_8908	63.00	lobChIP_FOXA1inPBS
chr2	187285196	187285197	MACS_peak_8909	9.00	lobChIP_FOXA1inPBS
chr2	187291065	187291066	MACS_peak_8910	39.00	lobChIP_FOXA1inPBS
chr2	187325394	187325395	MACS_peak_8911	11.00	lobChIP_FOXA1inPBS
chr2	187398668	187398669	MACS_peak_8912	18.00	lobChIP_FOXA1inPBS
chr2	187409173	187409174	MACS_peak_8913	21.00	lobChIP_FOXA1inPBS
chr2	187426753	187426754	MACS_peak_8914	17.00	lobChIP_FOXA1inPBS
chr2	187440174	187440175	MACS_peak_8915	5.00	lobChIP_FOXA1inPBS
chr2	187460016	187460017	MACS_peak_8916	16.00	lobChIP_FOXA1inPBS
chr2	187479102	187479103	MACS_peak_8917	8.00	lobChIP_FOXA1inPBS
chr2	188346041	188346042	MACS_peak_8918	9.00	lobChIP_FOXA1inPBS
chr2	188374697	188374698	MACS_peak_8919	48.00	lobChIP_FOXA1inPBS
chr2	188376774	188376775	MACS_peak_8920	10.00	lobChIP_FOXA1inPBS
chr2	188394645	188394646	MACS_peak_8921	7.00	lobChIP_FOXA1inPBS
chr2	188406404	188406405	MACS_peak_8922	40.00	lobChIP_FOXA1inPBS
chr2	188410751	188410752	MACS_peak_8923	11.00	lobChIP_FOXA1inPBS
chr2	188414121	188414122	MACS_peak_8924	8.00	lobChIP_FOXA1inPBS
chr2	188414943	188414944	MACS_peak_8925	10.00	lobChIP_FOXA1inPBS
chr2	188730019	188730020	MACS_peak_8926	9.00	lobChIP_FOXA1inPBS
chr2	189915038	189915039	MACS_peak_8927	130.00	lobChIP_FOXA1inPBS
chr2	190212269	190212270	MACS_peak_8928	10.00	lobChIP_FOXA1inPBS
chr2	190304212	190304213	MACS_peak_8929	10.00	lobChIP_FOXA1inPBS
chr2	190386903	190386904	MACS_peak_8930	35.00	lobChIP_FOXA1inPBS
chr2	190416494	190416495	MACS_peak_8931	8.00	lobChIP_FOXA1inPBS
chr2	190417986	190417987	MACS_peak_8932	26.00	lobChIP_FOXA1inPBS
chr2	190420108	190420109	MACS_peak_8933	6.00	lobChIP_FOXA1inPBS
chr2	190435589	190435590	MACS_peak_8934	10.00	lobChIP_FOXA1inPBS
chr2	190474243	190474244	MACS_peak_8935	17.00	lobChIP_FOXA1inPBS
chr2	190498235	190498236	MACS_peak_8936	9.00	lobChIP_FOXA1inPBS
chr2	190526003	190526004	MACS_peak_8937	8.00	lobChIP_FOXA1inPBS
chr2	190647854	190647855	MACS_peak_8938	11.00	lobChIP_FOXA1inPBS
chr2	191001269	191001270	MACS_peak_8939	15.00	lobChIP_FOXA1inPBS
chr2	191135145	191135146	MACS_peak_8940	12.00	lobChIP_FOXA1inPBS
chr2	191178804	191178805	MACS_peak_8941	7.00	lobChIP_FOXA1inPBS
chr2	191199289	191199290	MACS_peak_8942	18.00	lobChIP_FOXA1inPBS
chr2	191217772	191217773	MACS_peak_8943	20.00	lobChIP_FOXA1inPBS
chr2	191237318	191237319	MACS_peak_8944	16.00	lobChIP_FOXA1inPBS
chr2	191247802	191247803	MACS_peak_8945	16.00	lobChIP_FOXA1inPBS
chr2	191358109	191358110	MACS_peak_8946	10.00	lobChIP_FOXA1inPBS
chr2	191364164	191364165	MACS_peak_8947	19.00	lobChIP_FOXA1inPBS
chr2	191393522	191393523	MACS_peak_8948	18.00	lobChIP_FOXA1inPBS
chr2	191419309	191419310	MACS_peak_8949	11.00	lobChIP_FOXA1inPBS
chr2	191674097	191674098	MACS_peak_8950	19.00	lobChIP_FOXA1inPBS
chr2	191677768	191677769	MACS_peak_8951	15.00	lobChIP_FOXA1inPBS
chr2	191746939	191746940	MACS_peak_8952	9.00	lobChIP_FOXA1inPBS
chr2	191748533	191748534	MACS_peak_8953	7.00	lobChIP_FOXA1inPBS
chr2	191757703	191757704	MACS_peak_8954	10.00	lobChIP_FOXA1inPBS
chr2	191803895	191803896	MACS_peak_8955	8.00	lobChIP_FOXA1inPBS
chr2	191879059	191879060	MACS_peak_8956	10.00	lobChIP_FOXA1inPBS
chr2	191915040	191915041	MACS_peak_8957	6.00	lobChIP_FOXA1inPBS
chr2	192096826	192096827	MACS_peak_8958	12.00	lobChIP_FOXA1inPBS
chr2	192304419	192304420	MACS_peak_8959	10.00	lobChIP_FOXA1inPBS
chr2	192502705	192502706	MACS_peak_8960	24.00	lobChIP_FOXA1inPBS
chr2	192729482	192729483	MACS_peak_8961	16.00	lobChIP_FOXA1inPBS
chr2	192731679	192731680	MACS_peak_8962	20.00	lobChIP_FOXA1inPBS
chr2	192734080	192734081	MACS_peak_8963	11.00	lobChIP_FOXA1inPBS
chr2	192747253	192747254	MACS_peak_8964	19.00	lobChIP_FOXA1inPBS
chr2	192934379	192934380	MACS_peak_8965	9.00	lobChIP_FOXA1inPBS
chr2	193443771	193443772	MACS_peak_8966	9.00	lobChIP_FOXA1inPBS
chr2	193877914	193877915	MACS_peak_8967	8.00	lobChIP_FOXA1inPBS
chr2	194293183	194293184	MACS_peak_8968	26.00	lobChIP_FOXA1inPBS
chr2	194404092	194404093	MACS_peak_8969	14.00	lobChIP_FOXA1inPBS
chr2	194598466	194598467	MACS_peak_8970	8.00	lobChIP_FOXA1inPBS
chr2	196522065	196522066	MACS_peak_8971	7.00	lobChIP_FOXA1inPBS
chr2	196568838	196568839	MACS_peak_8972	9.00	lobChIP_FOXA1inPBS
chr2	197594325	197594326	MACS_peak_8973	8.00	lobChIP_FOXA1inPBS
chr2	197792853	197792854	MACS_peak_8974	8.00	lobChIP_FOXA1inPBS
chr2	198267568	198267569	MACS_peak_8975	10.00	lobChIP_FOXA1inPBS
chr2	198478474	198478475	MACS_peak_8976	105.00	lobChIP_FOXA1inPBS
chr2	198522451	198522452	MACS_peak_8977	16.00	lobChIP_FOXA1inPBS
chr2	200229129	200229130	MACS_peak_8978	9.00	lobChIP_FOXA1inPBS
chr2	200231357	200231358	MACS_peak_8979	11.00	lobChIP_FOXA1inPBS
chr2	200325472	200325473	MACS_peak_8980	12.00	lobChIP_FOXA1inPBS
chr2	200389875	200389876	MACS_peak_8981	23.00	lobChIP_FOXA1inPBS
chr2	200582948	200582949	MACS_peak_8982	12.00	lobChIP_FOXA1inPBS
chr2	200603628	200603629	MACS_peak_8983	24.00	lobChIP_FOXA1inPBS
chr2	200842069	200842070	MACS_peak_8984	8.00	lobChIP_FOXA1inPBS
chr2	200942026	200942027	MACS_peak_8985	60.00	lobChIP_FOXA1inPBS
chr2	201086091	201086092	MACS_peak_8986	36.00	lobChIP_FOXA1inPBS
chr2	201095611	201095612	MACS_peak_8987	16.00	lobChIP_FOXA1inPBS
chr2	201167934	201167935	MACS_peak_8988	18.00	lobChIP_FOXA1inPBS
chr2	201171004	201171005	MACS_peak_8989	11.00	lobChIP_FOXA1inPBS
chr2	201174532	201174533	MACS_peak_8990	10.00	lobChIP_FOXA1inPBS
chr2	201175933	201175934	MACS_peak_8991	15.00	lobChIP_FOXA1inPBS
chr2	201183849	201183850	MACS_peak_8992	38.00	lobChIP_FOXA1inPBS
chr2	201184841	201184842	MACS_peak_8993	55.00	lobChIP_FOXA1inPBS
chr2	201232046	201232047	MACS_peak_8994	26.00	lobChIP_FOXA1inPBS
chr2	201270902	201270903	MACS_peak_8995	36.00	lobChIP_FOXA1inPBS
chr2	201372064	201372065	MACS_peak_8996	11.00	lobChIP_FOXA1inPBS
chr2	201443667	201443668	MACS_peak_8997	14.00	lobChIP_FOXA1inPBS
chr2	201457381	201457382	MACS_peak_8998	16.00	lobChIP_FOXA1inPBS
chr2	201468876	201468877	MACS_peak_8999	11.00	lobChIP_FOXA1inPBS
chr2	201542933	201542934	MACS_peak_9000	21.00	lobChIP_FOXA1inPBS
chr2	201567528	201567529	MACS_peak_9001	25.00	lobChIP_FOXA1inPBS
chr2	201606748	201606749	MACS_peak_9002	30.00	lobChIP_FOXA1inPBS
chr2	201613053	201613054	MACS_peak_9003	38.00	lobChIP_FOXA1inPBS
chr2	201621586	201621587	MACS_peak_9004	32.00	lobChIP_FOXA1inPBS
chr2	201623291	201623292	MACS_peak_9005	33.00	lobChIP_FOXA1inPBS
chr2	201651472	201651473	MACS_peak_9006	13.00	lobChIP_FOXA1inPBS
chr2	201796229	201796230	MACS_peak_9007	9.00	lobChIP_FOXA1inPBS
chr2	201796809	201796810	MACS_peak_9008	10.00	lobChIP_FOXA1inPBS
chr2	201828640	201828641	MACS_peak_9009	17.00	lobChIP_FOXA1inPBS
chr2	201873114	201873115	MACS_peak_9010	14.00	lobChIP_FOXA1inPBS
chr2	201905854	201905855	MACS_peak_9011	11.00	lobChIP_FOXA1inPBS
chr2	201922692	201922693	MACS_peak_9012	18.00	lobChIP_FOXA1inPBS
chr2	201934307	201934308	MACS_peak_9013	12.00	lobChIP_FOXA1inPBS
chr2	201935465	201935466	MACS_peak_9014	8.00	lobChIP_FOXA1inPBS
chr2	201972400	201972401	MACS_peak_9015	12.00	lobChIP_FOXA1inPBS
chr2	201980997	201980998	MACS_peak_9016	12.00	lobChIP_FOXA1inPBS
chr2	201981972	201981973	MACS_peak_9017	5.00	lobChIP_FOXA1inPBS
chr2	201984496	201984497	MACS_peak_9018	9.00	lobChIP_FOXA1inPBS
chr2	201993978	201993979	MACS_peak_9019	18.00	lobChIP_FOXA1inPBS
chr2	202023171	202023172	MACS_peak_9020	10.00	lobChIP_FOXA1inPBS
chr2	202046890	202046891	MACS_peak_9021	6.00	lobChIP_FOXA1inPBS
chr2	202047508	202047509	MACS_peak_9022	24.00	lobChIP_FOXA1inPBS
chr2	202069826	202069827	MACS_peak_9023	10.00	lobChIP_FOXA1inPBS
chr2	202266286	202266287	MACS_peak_9024	10.00	lobChIP_FOXA1inPBS
chr2	202423120	202423121	MACS_peak_9025	13.00	lobChIP_FOXA1inPBS
chr2	202445375	202445376	MACS_peak_9026	23.00	lobChIP_FOXA1inPBS
chr2	202619670	202619671	MACS_peak_9027	25.00	lobChIP_FOXA1inPBS
chr2	203143836	203143837	MACS_peak_9028	15.00	lobChIP_FOXA1inPBS
chr2	203359265	203359266	MACS_peak_9029	28.00	lobChIP_FOXA1inPBS
chr2	203453622	203453623	MACS_peak_9030	11.00	lobChIP_FOXA1inPBS
chr2	203704289	203704290	MACS_peak_9031	9.00	lobChIP_FOXA1inPBS
chr2	203746465	203746466	MACS_peak_9032	19.00	lobChIP_FOXA1inPBS
chr2	203776643	203776644	MACS_peak_9033	6.00	lobChIP_FOXA1inPBS
chr2	204217781	204217782	MACS_peak_9034	18.00	lobChIP_FOXA1inPBS
chr2	204408868	204408869	MACS_peak_9035	11.00	lobChIP_FOXA1inPBS
chr2	204416773	204416774	MACS_peak_9036	9.00	lobChIP_FOXA1inPBS
chr2	204513271	204513272	MACS_peak_9037	26.00	lobChIP_FOXA1inPBS
chr2	204521522	204521523	MACS_peak_9038	17.00	lobChIP_FOXA1inPBS
chr2	204562716	204562717	MACS_peak_9039	10.00	lobChIP_FOXA1inPBS
chr2	204578941	204578942	MACS_peak_9040	20.00	lobChIP_FOXA1inPBS
chr2	204585914	204585915	MACS_peak_9041	16.00	lobChIP_FOXA1inPBS
chr2	204597013	204597014	MACS_peak_9042	8.00	lobChIP_FOXA1inPBS
chr2	204648855	204648856	MACS_peak_9043	32.00	lobChIP_FOXA1inPBS
chr2	204664619	204664620	MACS_peak_9044	11.00	lobChIP_FOXA1inPBS
chr2	204677659	204677660	MACS_peak_9045	11.00	lobChIP_FOXA1inPBS
chr2	204680165	204680166	MACS_peak_9046	13.00	lobChIP_FOXA1inPBS
chr2	205204879	205204880	MACS_peak_9047	7.00	lobChIP_FOXA1inPBS
chr2	205478528	205478529	MACS_peak_9048	20.00	lobChIP_FOXA1inPBS
chr2	205509987	205509988	MACS_peak_9049	9.00	lobChIP_FOXA1inPBS
chr2	205535000	205535001	MACS_peak_9050	9.00	lobChIP_FOXA1inPBS
chr2	206055583	206055584	MACS_peak_9051	8.00	lobChIP_FOXA1inPBS
chr2	206138475	206138476	MACS_peak_9052	14.00	lobChIP_FOXA1inPBS
chr2	206583861	206583862	MACS_peak_9053	16.00	lobChIP_FOXA1inPBS
chr2	206596406	206596407	MACS_peak_9054	9.00	lobChIP_FOXA1inPBS
chr2	206651832	206651833	MACS_peak_9055	13.00	lobChIP_FOXA1inPBS
chr2	206766994	206766995	MACS_peak_9056	9.00	lobChIP_FOXA1inPBS
chr2	206802123	206802124	MACS_peak_9057	7.00	lobChIP_FOXA1inPBS
chr2	206840161	206840162	MACS_peak_9058	18.00	lobChIP_FOXA1inPBS
chr2	206854590	206854591	MACS_peak_9059	31.00	lobChIP_FOXA1inPBS
chr2	206942772	206942773	MACS_peak_9060	14.00	lobChIP_FOXA1inPBS
chr2	206992638	206992639	MACS_peak_9061	9.00	lobChIP_FOXA1inPBS
chr2	207267944	207267945	MACS_peak_9062	15.00	lobChIP_FOXA1inPBS
chr2	208030946	208030947	MACS_peak_9063	10.00	lobChIP_FOXA1inPBS
chr2	208181471	208181472	MACS_peak_9064	9.00	lobChIP_FOXA1inPBS
chr2	208273223	208273224	MACS_peak_9065	21.00	lobChIP_FOXA1inPBS
chr2	208339790	208339791	MACS_peak_9066	9.00	lobChIP_FOXA1inPBS
chr2	208583782	208583783	MACS_peak_9067	7.00	lobChIP_FOXA1inPBS
chr2	208599690	208599691	MACS_peak_9068	26.00	lobChIP_FOXA1inPBS
chr2	208612816	208612817	MACS_peak_9069	13.00	lobChIP_FOXA1inPBS
chr2	208630566	208630567	MACS_peak_9070	8.00	lobChIP_FOXA1inPBS
chr2	208647133	208647134	MACS_peak_9071	26.00	lobChIP_FOXA1inPBS
chr2	208661769	208661770	MACS_peak_9072	18.00	lobChIP_FOXA1inPBS
chr2	208666227	208666228	MACS_peak_9073	18.00	lobChIP_FOXA1inPBS
chr2	208675837	208675838	MACS_peak_9074	6.00	lobChIP_FOXA1inPBS
chr2	208693320	208693321	MACS_peak_9075	7.00	lobChIP_FOXA1inPBS
chr2	208693860	208693861	MACS_peak_9076	7.00	lobChIP_FOXA1inPBS
chr2	208700684	208700685	MACS_peak_9077	11.00	lobChIP_FOXA1inPBS
chr2	208710091	208710092	MACS_peak_9078	71.00	lobChIP_FOXA1inPBS
chr2	208712059	208712060	MACS_peak_9079	21.00	lobChIP_FOXA1inPBS
chr2	208712730	208712731	MACS_peak_9080	36.00	lobChIP_FOXA1inPBS
chr2	208713401	208713402	MACS_peak_9081	16.00	lobChIP_FOXA1inPBS
chr2	208735231	208735232	MACS_peak_9082	27.00	lobChIP_FOXA1inPBS
chr2	208740726	208740727	MACS_peak_9083	15.00	lobChIP_FOXA1inPBS
chr2	208762949	208762950	MACS_peak_9084	15.00	lobChIP_FOXA1inPBS
chr2	208771836	208771837	MACS_peak_9085	14.00	lobChIP_FOXA1inPBS
chr2	208801599	208801600	MACS_peak_9086	13.00	lobChIP_FOXA1inPBS
chr2	208834977	208834978	MACS_peak_9087	8.00	lobChIP_FOXA1inPBS
chr2	208838000	208838001	MACS_peak_9088	6.00	lobChIP_FOXA1inPBS
chr2	208878690	208878691	MACS_peak_9089	18.00	lobChIP_FOXA1inPBS
chr2	208880399	208880400	MACS_peak_9090	29.00	lobChIP_FOXA1inPBS
chr2	208890252	208890253	MACS_peak_9091	17.00	lobChIP_FOXA1inPBS
chr2	208896663	208896664	MACS_peak_9092	24.00	lobChIP_FOXA1inPBS
chr2	209008197	209008198	MACS_peak_9093	8.00	lobChIP_FOXA1inPBS
chr2	209158856	209158857	MACS_peak_9094	9.00	lobChIP_FOXA1inPBS
chr2	209235268	209235269	MACS_peak_9095	14.00	lobChIP_FOXA1inPBS
chr2	209659245	209659246	MACS_peak_9096	16.00	lobChIP_FOXA1inPBS
chr2	210373210	210373211	MACS_peak_9097	9.00	lobChIP_FOXA1inPBS
chr2	210835222	210835223	MACS_peak_9098	20.00	lobChIP_FOXA1inPBS
chr2	210937781	210937782	MACS_peak_9099	8.00	lobChIP_FOXA1inPBS
chr2	210996920	210996921	MACS_peak_9100	13.00	lobChIP_FOXA1inPBS
chr2	211388626	211388627	MACS_peak_9101	10.00	lobChIP_FOXA1inPBS
chr2	211405183	211405184	MACS_peak_9102	7.00	lobChIP_FOXA1inPBS
chr2	211406242	211406243	MACS_peak_9103	13.00	lobChIP_FOXA1inPBS
chr2	211413990	211413991	MACS_peak_9104	63.00	lobChIP_FOXA1inPBS
chr2	211551330	211551331	MACS_peak_9105	12.00	lobChIP_FOXA1inPBS
chr2	211828321	211828322	MACS_peak_9106	33.00	lobChIP_FOXA1inPBS
chr2	212794738	212794739	MACS_peak_9107	9.00	lobChIP_FOXA1inPBS
chr2	213224269	213224270	MACS_peak_9108	8.00	lobChIP_FOXA1inPBS
chr2	213226206	213226207	MACS_peak_9109	9.00	lobChIP_FOXA1inPBS
chr2	213263223	213263224	MACS_peak_9110	7.00	lobChIP_FOXA1inPBS
chr2	213289040	213289041	MACS_peak_9111	9.00	lobChIP_FOXA1inPBS
chr2	213316768	213316769	MACS_peak_9112	9.00	lobChIP_FOXA1inPBS
chr2	214103811	214103812	MACS_peak_9113	40.00	lobChIP_FOXA1inPBS
chr2	216262617	216262618	MACS_peak_9114	15.00	lobChIP_FOXA1inPBS
chr2	216280183	216280184	MACS_peak_9115	24.00	lobChIP_FOXA1inPBS
chr2	216282202	216282203	MACS_peak_9116	85.00	lobChIP_FOXA1inPBS
chr2	216283429	216283430	MACS_peak_9117	17.00	lobChIP_FOXA1inPBS
chr2	216284721	216284722	MACS_peak_9118	20.00	lobChIP_FOXA1inPBS
chr2	216285434	216285435	MACS_peak_9119	9.00	lobChIP_FOXA1inPBS
chr2	216304864	216304865	MACS_peak_9120	16.00	lobChIP_FOXA1inPBS
chr2	216363243	216363244	MACS_peak_9121	13.00	lobChIP_FOXA1inPBS
chr2	216371086	216371087	MACS_peak_9122	7.00	lobChIP_FOXA1inPBS
chr2	216394203	216394204	MACS_peak_9123	12.00	lobChIP_FOXA1inPBS
chr2	216482015	216482016	MACS_peak_9124	90.00	lobChIP_FOXA1inPBS
chr2	216484253	216484254	MACS_peak_9125	11.00	lobChIP_FOXA1inPBS
chr2	216490155	216490156	MACS_peak_9126	18.00	lobChIP_FOXA1inPBS
chr2	216545981	216545982	MACS_peak_9127	27.00	lobChIP_FOXA1inPBS
chr2	216558463	216558464	MACS_peak_9128	24.00	lobChIP_FOXA1inPBS
chr2	216578290	216578291	MACS_peak_9129	9.00	lobChIP_FOXA1inPBS
chr2	216591842	216591843	MACS_peak_9130	7.00	lobChIP_FOXA1inPBS
chr2	216656674	216656675	MACS_peak_9131	9.00	lobChIP_FOXA1inPBS
chr2	216658825	216658826	MACS_peak_9132	70.00	lobChIP_FOXA1inPBS
chr2	216673964	216673965	MACS_peak_9133	21.00	lobChIP_FOXA1inPBS
chr2	216691984	216691985	MACS_peak_9134	11.00	lobChIP_FOXA1inPBS
chr2	216695482	216695483	MACS_peak_9135	7.00	lobChIP_FOXA1inPBS
chr2	216698723	216698724	MACS_peak_9136	31.00	lobChIP_FOXA1inPBS
chr2	216741586	216741587	MACS_peak_9137	13.00	lobChIP_FOXA1inPBS
chr2	216816479	216816480	MACS_peak_9138	8.00	lobChIP_FOXA1inPBS
chr2	216847320	216847321	MACS_peak_9139	20.00	lobChIP_FOXA1inPBS
chr2	216853504	216853505	MACS_peak_9140	10.00	lobChIP_FOXA1inPBS
chr2	216877220	216877221	MACS_peak_9141	11.00	lobChIP_FOXA1inPBS
chr2	216936400	216936401	MACS_peak_9142	10.00	lobChIP_FOXA1inPBS
chr2	216943585	216943586	MACS_peak_9143	30.00	lobChIP_FOXA1inPBS
chr2	217381599	217381600	MACS_peak_9144	12.00	lobChIP_FOXA1inPBS
chr2	217388073	217388074	MACS_peak_9145	12.00	lobChIP_FOXA1inPBS
chr2	217436499	217436500	MACS_peak_9146	26.00	lobChIP_FOXA1inPBS
chr2	217484918	217484919	MACS_peak_9147	15.00	lobChIP_FOXA1inPBS
chr2	217531500	217531501	MACS_peak_9148	8.00	lobChIP_FOXA1inPBS
chr2	218196570	218196571	MACS_peak_9149	22.00	lobChIP_FOXA1inPBS
chr2	219158477	219158478	MACS_peak_9150	13.00	lobChIP_FOXA1inPBS
chr2	219161091	219161092	MACS_peak_9151	10.00	lobChIP_FOXA1inPBS
chr2	219176998	219176999	MACS_peak_9152	15.00	lobChIP_FOXA1inPBS
chr2	219246699	219246700	MACS_peak_9153	5.00	lobChIP_FOXA1inPBS
chr2	219261610	219261611	MACS_peak_9154	19.00	lobChIP_FOXA1inPBS
chr2	219309520	219309521	MACS_peak_9155	19.00	lobChIP_FOXA1inPBS
chr2	219570873	219570874	MACS_peak_9156	7.00	lobChIP_FOXA1inPBS
chr2	219592686	219592687	MACS_peak_9157	17.00	lobChIP_FOXA1inPBS
chr2	219598612	219598613	MACS_peak_9158	16.00	lobChIP_FOXA1inPBS
chr2	219620216	219620217	MACS_peak_9159	11.00	lobChIP_FOXA1inPBS
chr2	219620635	219620636	MACS_peak_9160	54.00	lobChIP_FOXA1inPBS
chr2	219621609	219621610	MACS_peak_9161	10.00	lobChIP_FOXA1inPBS
chr2	219645835	219645836	MACS_peak_9162	16.00	lobChIP_FOXA1inPBS
chr2	219671304	219671305	MACS_peak_9163	12.00	lobChIP_FOXA1inPBS
chr2	219774170	219774171	MACS_peak_9164	14.00	lobChIP_FOXA1inPBS
chr2	219866809	219866810	MACS_peak_9165	18.00	lobChIP_FOXA1inPBS
chr2	219936128	219936129	MACS_peak_9166	24.00	lobChIP_FOXA1inPBS
chr2	219946061	219946062	MACS_peak_9167	18.00	lobChIP_FOXA1inPBS
chr2	219976506	219976507	MACS_peak_9168	30.00	lobChIP_FOXA1inPBS
chr2	219981625	219981626	MACS_peak_9169	20.00	lobChIP_FOXA1inPBS
chr2	219991369	219991370	MACS_peak_9170	22.00	lobChIP_FOXA1inPBS
chr2	219992352	219992353	MACS_peak_9171	12.00	lobChIP_FOXA1inPBS
chr2	219996228	219996229	MACS_peak_9172	29.00	lobChIP_FOXA1inPBS
chr2	220042596	220042597	MACS_peak_9173	12.00	lobChIP_FOXA1inPBS
chr2	220057552	220057553	MACS_peak_9174	25.00	lobChIP_FOXA1inPBS
chr2	220114185	220114186	MACS_peak_9175	37.00	lobChIP_FOXA1inPBS
chr2	220179110	220179111	MACS_peak_9176	8.00	lobChIP_FOXA1inPBS
chr2	220263746	220263747	MACS_peak_9177	28.00	lobChIP_FOXA1inPBS
chr2	220436520	220436521	MACS_peak_9178	5.00	lobChIP_FOXA1inPBS
chr2	220441743	220441744	MACS_peak_9179	28.00	lobChIP_FOXA1inPBS
chr2	220701639	220701640	MACS_peak_9180	20.00	lobChIP_FOXA1inPBS
chr2	223252318	223252319	MACS_peak_9181	6.00	lobChIP_FOXA1inPBS
chr2	223384152	223384153	MACS_peak_9182	9.00	lobChIP_FOXA1inPBS
chr2	223459579	223459580	MACS_peak_9183	10.00	lobChIP_FOXA1inPBS
chr2	223573623	223573624	MACS_peak_9184	7.00	lobChIP_FOXA1inPBS
chr2	223598334	223598335	MACS_peak_9185	23.00	lobChIP_FOXA1inPBS
chr2	223606488	223606489	MACS_peak_9186	8.00	lobChIP_FOXA1inPBS
chr2	223642333	223642334	MACS_peak_9187	16.00	lobChIP_FOXA1inPBS
chr2	223660945	223660946	MACS_peak_9188	53.00	lobChIP_FOXA1inPBS
chr2	223707550	223707551	MACS_peak_9189	18.00	lobChIP_FOXA1inPBS
chr2	223729871	223729872	MACS_peak_9190	10.00	lobChIP_FOXA1inPBS
chr2	223743173	223743174	MACS_peak_9191	26.00	lobChIP_FOXA1inPBS
chr2	223743732	223743733	MACS_peak_9192	6.00	lobChIP_FOXA1inPBS
chr2	223768589	223768590	MACS_peak_9193	15.00	lobChIP_FOXA1inPBS
chr2	223780475	223780476	MACS_peak_9194	15.00	lobChIP_FOXA1inPBS
chr2	223891191	223891192	MACS_peak_9195	13.00	lobChIP_FOXA1inPBS
chr2	224063907	224063908	MACS_peak_9196	7.00	lobChIP_FOXA1inPBS
chr2	224624079	224624080	MACS_peak_9197	16.00	lobChIP_FOXA1inPBS
chr2	224718171	224718172	MACS_peak_9198	13.00	lobChIP_FOXA1inPBS
chr2	224726221	224726222	MACS_peak_9199	22.00	lobChIP_FOXA1inPBS
chr2	224752081	224752082	MACS_peak_9200	12.00	lobChIP_FOXA1inPBS
chr2	224773293	224773294	MACS_peak_9201	27.00	lobChIP_FOXA1inPBS
chr2	224798024	224798025	MACS_peak_9202	10.00	lobChIP_FOXA1inPBS
chr2	224888134	224888135	MACS_peak_9203	12.00	lobChIP_FOXA1inPBS
chr2	225011224	225011225	MACS_peak_9204	12.00	lobChIP_FOXA1inPBS
chr2	225016065	225016066	MACS_peak_9205	11.00	lobChIP_FOXA1inPBS
chr2	225233974	225233975	MACS_peak_9206	24.00	lobChIP_FOXA1inPBS
chr2	225350197	225350198	MACS_peak_9207	26.00	lobChIP_FOXA1inPBS
chr2	225403680	225403681	MACS_peak_9208	10.00	lobChIP_FOXA1inPBS
chr2	226336388	226336389	MACS_peak_9209	25.00	lobChIP_FOXA1inPBS
chr2	226979961	226979962	MACS_peak_9210	13.00	lobChIP_FOXA1inPBS
chr2	226983616	226983617	MACS_peak_9211	19.00	lobChIP_FOXA1inPBS
chr2	226984740	226984741	MACS_peak_9212	12.00	lobChIP_FOXA1inPBS
chr2	226988981	226988982	MACS_peak_9213	10.00	lobChIP_FOXA1inPBS
chr2	227002334	227002335	MACS_peak_9214	7.00	lobChIP_FOXA1inPBS
chr2	227024764	227024765	MACS_peak_9215	51.00	lobChIP_FOXA1inPBS
chr2	227035127	227035128	MACS_peak_9216	10.00	lobChIP_FOXA1inPBS
chr2	227045372	227045373	MACS_peak_9217	8.00	lobChIP_FOXA1inPBS
chr2	227237410	227237411	MACS_peak_9218	7.00	lobChIP_FOXA1inPBS
chr2	227276626	227276627	MACS_peak_9219	11.00	lobChIP_FOXA1inPBS
chr2	227321078	227321079	MACS_peak_9220	16.00	lobChIP_FOXA1inPBS
chr2	227342924	227342925	MACS_peak_9221	17.00	lobChIP_FOXA1inPBS
chr2	227348889	227348890	MACS_peak_9222	19.00	lobChIP_FOXA1inPBS
chr2	227380306	227380307	MACS_peak_9223	22.00	lobChIP_FOXA1inPBS
chr2	227424425	227424426	MACS_peak_9224	65.00	lobChIP_FOXA1inPBS
chr2	227484126	227484127	MACS_peak_9225	24.00	lobChIP_FOXA1inPBS
chr2	227515125	227515126	MACS_peak_9226	10.00	lobChIP_FOXA1inPBS
chr2	227522370	227522371	MACS_peak_9227	17.00	lobChIP_FOXA1inPBS
chr2	227566656	227566657	MACS_peak_9228	10.00	lobChIP_FOXA1inPBS
chr2	227598074	227598075	MACS_peak_9229	41.00	lobChIP_FOXA1inPBS
chr2	227603951	227603952	MACS_peak_9230	9.00	lobChIP_FOXA1inPBS
chr2	227605221	227605222	MACS_peak_9231	11.00	lobChIP_FOXA1inPBS
chr2	227637513	227637514	MACS_peak_9232	9.00	lobChIP_FOXA1inPBS
chr2	227665438	227665439	MACS_peak_9233	49.00	lobChIP_FOXA1inPBS
chr2	227703615	227703616	MACS_peak_9234	17.00	lobChIP_FOXA1inPBS
chr2	227903478	227903479	MACS_peak_9235	58.00	lobChIP_FOXA1inPBS
chr2	228102776	228102777	MACS_peak_9236	13.00	lobChIP_FOXA1inPBS
chr2	228176719	228176720	MACS_peak_9237	9.00	lobChIP_FOXA1inPBS
chr2	228178241	228178242	MACS_peak_9238	11.00	lobChIP_FOXA1inPBS
chr2	228204014	228204015	MACS_peak_9239	20.00	lobChIP_FOXA1inPBS
chr2	228316263	228316264	MACS_peak_9240	49.00	lobChIP_FOXA1inPBS
chr2	228319427	228319428	MACS_peak_9241	45.00	lobChIP_FOXA1inPBS
chr2	228331126	228331127	MACS_peak_9242	17.00	lobChIP_FOXA1inPBS
chr2	228333045	228333046	MACS_peak_9243	11.00	lobChIP_FOXA1inPBS
chr2	228335816	228335817	MACS_peak_9244	5.00	lobChIP_FOXA1inPBS
chr2	228509045	228509046	MACS_peak_9245	30.00	lobChIP_FOXA1inPBS
chr2	228518205	228518206	MACS_peak_9246	10.00	lobChIP_FOXA1inPBS
chr2	228626126	228626127	MACS_peak_9247	8.00	lobChIP_FOXA1inPBS
chr2	228639162	228639163	MACS_peak_9248	17.00	lobChIP_FOXA1inPBS
chr2	228661185	228661186	MACS_peak_9249	12.00	lobChIP_FOXA1inPBS
chr2	228678487	228678488	MACS_peak_9250	47.00	lobChIP_FOXA1inPBS
chr2	228681496	228681497	MACS_peak_9251	7.00	lobChIP_FOXA1inPBS
chr2	228683757	228683758	MACS_peak_9252	10.00	lobChIP_FOXA1inPBS
chr2	228727172	228727173	MACS_peak_9253	9.00	lobChIP_FOXA1inPBS
chr2	228913762	228913763	MACS_peak_9254	7.00	lobChIP_FOXA1inPBS
chr2	230025010	230025011	MACS_peak_9255	17.00	lobChIP_FOXA1inPBS
chr2	230764905	230764906	MACS_peak_9256	27.00	lobChIP_FOXA1inPBS
chr2	230931850	230931851	MACS_peak_9257	12.00	lobChIP_FOXA1inPBS
chr2	231192562	231192563	MACS_peak_9258	5.00	lobChIP_FOXA1inPBS
chr2	231220981	231220982	MACS_peak_9259	39.00	lobChIP_FOXA1inPBS
chr2	231281373	231281374	MACS_peak_9260	10.00	lobChIP_FOXA1inPBS
chr2	231288476	231288477	MACS_peak_9261	9.00	lobChIP_FOXA1inPBS
chr2	231356916	231356917	MACS_peak_9262	12.00	lobChIP_FOXA1inPBS
chr2	231378140	231378141	MACS_peak_9263	33.00	lobChIP_FOXA1inPBS
chr2	231466050	231466051	MACS_peak_9264	8.00	lobChIP_FOXA1inPBS
chr2	231515401	231515402	MACS_peak_9265	10.00	lobChIP_FOXA1inPBS
chr2	231603848	231603849	MACS_peak_9266	136.00	lobChIP_FOXA1inPBS
chr2	231620925	231620926	MACS_peak_9267	12.00	lobChIP_FOXA1inPBS
chr2	231872155	231872156	MACS_peak_9268	9.00	lobChIP_FOXA1inPBS
chr2	231899141	231899142	MACS_peak_9269	41.00	lobChIP_FOXA1inPBS
chr2	231915216	231915217	MACS_peak_9270	53.00	lobChIP_FOXA1inPBS
chr2	231930891	231930892	MACS_peak_9271	15.00	lobChIP_FOXA1inPBS
chr2	231933380	231933381	MACS_peak_9272	12.00	lobChIP_FOXA1inPBS
chr2	231943462	231943463	MACS_peak_9273	9.00	lobChIP_FOXA1inPBS
chr2	231944623	231944624	MACS_peak_9274	9.00	lobChIP_FOXA1inPBS
chr2	232165147	232165148	MACS_peak_9275	8.00	lobChIP_FOXA1inPBS
chr2	232203348	232203349	MACS_peak_9276	17.00	lobChIP_FOXA1inPBS
chr2	232581561	232581562	MACS_peak_9277	7.00	lobChIP_FOXA1inPBS
chr2	232822719	232822720	MACS_peak_9278	19.00	lobChIP_FOXA1inPBS
chr2	232828129	232828130	MACS_peak_9279	12.00	lobChIP_FOXA1inPBS
chr2	232863259	232863260	MACS_peak_9280	8.00	lobChIP_FOXA1inPBS
chr2	233005061	233005062	MACS_peak_9281	12.00	lobChIP_FOXA1inPBS
chr2	233165927	233165928	MACS_peak_9282	47.00	lobChIP_FOXA1inPBS
chr2	233439663	233439664	MACS_peak_9283	14.00	lobChIP_FOXA1inPBS
chr2	233489893	233489894	MACS_peak_9284	7.00	lobChIP_FOXA1inPBS
chr2	233722079	233722080	MACS_peak_9285	7.00	lobChIP_FOXA1inPBS
chr2	233725515	233725516	MACS_peak_9286	6.00	lobChIP_FOXA1inPBS
chr2	233730021	233730022	MACS_peak_9287	8.00	lobChIP_FOXA1inPBS
chr2	233737065	233737066	MACS_peak_9288	8.00	lobChIP_FOXA1inPBS
chr2	233814021	233814022	MACS_peak_9289	8.00	lobChIP_FOXA1inPBS
chr2	233878022	233878023	MACS_peak_9290	28.00	lobChIP_FOXA1inPBS
chr2	234654207	234654208	MACS_peak_9291	41.00	lobChIP_FOXA1inPBS
chr2	234665580	234665581	MACS_peak_9292	99.00	lobChIP_FOXA1inPBS
chr2	234885763	234885764	MACS_peak_9293	10.00	lobChIP_FOXA1inPBS
chr2	234890465	234890466	MACS_peak_9294	6.00	lobChIP_FOXA1inPBS
chr2	235031649	235031650	MACS_peak_9295	8.00	lobChIP_FOXA1inPBS
chr2	235585000	235585001	MACS_peak_9296	16.00	lobChIP_FOXA1inPBS
chr2	235585599	235585600	MACS_peak_9297	10.00	lobChIP_FOXA1inPBS
chr2	235597982	235597983	MACS_peak_9298	14.00	lobChIP_FOXA1inPBS
chr2	235601107	235601108	MACS_peak_9299	28.00	lobChIP_FOXA1inPBS
chr2	235619456	235619457	MACS_peak_9300	24.00	lobChIP_FOXA1inPBS
chr2	235704418	235704419	MACS_peak_9301	45.00	lobChIP_FOXA1inPBS
chr2	235732568	235732569	MACS_peak_9302	15.00	lobChIP_FOXA1inPBS
chr2	235752074	235752075	MACS_peak_9303	6.00	lobChIP_FOXA1inPBS
chr2	235754784	235754785	MACS_peak_9304	45.00	lobChIP_FOXA1inPBS
chr2	235779954	235779955	MACS_peak_9305	29.00	lobChIP_FOXA1inPBS
chr2	235783599	235783600	MACS_peak_9306	7.00	lobChIP_FOXA1inPBS
chr2	235784652	235784653	MACS_peak_9307	12.00	lobChIP_FOXA1inPBS
chr2	235789373	235789374	MACS_peak_9308	22.00	lobChIP_FOXA1inPBS
chr2	235858634	235858635	MACS_peak_9309	8.00	lobChIP_FOXA1inPBS
chr2	235890312	235890313	MACS_peak_9310	15.00	lobChIP_FOXA1inPBS
chr2	235904487	235904488	MACS_peak_9311	18.00	lobChIP_FOXA1inPBS
chr2	235909107	235909108	MACS_peak_9312	10.00	lobChIP_FOXA1inPBS
chr2	235909689	235909690	MACS_peak_9313	14.00	lobChIP_FOXA1inPBS
chr2	235914476	235914477	MACS_peak_9314	43.00	lobChIP_FOXA1inPBS
chr2	236414109	236414110	MACS_peak_9315	13.00	lobChIP_FOXA1inPBS
chr2	236449869	236449870	MACS_peak_9316	8.00	lobChIP_FOXA1inPBS
chr2	236450742	236450743	MACS_peak_9317	37.00	lobChIP_FOXA1inPBS
chr2	236504290	236504291	MACS_peak_9318	9.00	lobChIP_FOXA1inPBS
chr2	236507653	236507654	MACS_peak_9319	20.00	lobChIP_FOXA1inPBS
chr2	236508767	236508768	MACS_peak_9320	16.00	lobChIP_FOXA1inPBS
chr2	236797715	236797716	MACS_peak_9321	12.00	lobChIP_FOXA1inPBS
chr2	236921094	236921095	MACS_peak_9322	8.00	lobChIP_FOXA1inPBS
chr2	236956789	236956790	MACS_peak_9323	12.00	lobChIP_FOXA1inPBS
chr2	237026778	237026779	MACS_peak_9324	84.00	lobChIP_FOXA1inPBS
chr2	237158142	237158143	MACS_peak_9325	7.00	lobChIP_FOXA1inPBS
chr2	237864640	237864641	MACS_peak_9326	9.00	lobChIP_FOXA1inPBS
chr2	237984739	237984740	MACS_peak_9327	13.00	lobChIP_FOXA1inPBS
chr2	238411364	238411365	MACS_peak_9328	17.00	lobChIP_FOXA1inPBS
chr2	238498999	238499000	MACS_peak_9329	12.00	lobChIP_FOXA1inPBS
chr2	238523102	238523103	MACS_peak_9330	20.00	lobChIP_FOXA1inPBS
chr2	238589916	238589917	MACS_peak_9331	10.00	lobChIP_FOXA1inPBS
chr2	238642725	238642726	MACS_peak_9332	20.00	lobChIP_FOXA1inPBS
chr2	238652713	238652714	MACS_peak_9333	15.00	lobChIP_FOXA1inPBS
chr2	238683677	238683678	MACS_peak_9334	26.00	lobChIP_FOXA1inPBS
chr2	238769883	238769884	MACS_peak_9335	23.00	lobChIP_FOXA1inPBS
chr2	238797263	238797264	MACS_peak_9336	7.00	lobChIP_FOXA1inPBS
chr2	238914312	238914313	MACS_peak_9337	10.00	lobChIP_FOXA1inPBS
chr2	238988630	238988631	MACS_peak_9338	76.00	lobChIP_FOXA1inPBS
chr2	239194610	239194611	MACS_peak_9339	41.00	lobChIP_FOXA1inPBS
chr2	239312846	239312847	MACS_peak_9340	22.00	lobChIP_FOXA1inPBS
chr2	239358806	239358807	MACS_peak_9341	7.00	lobChIP_FOXA1inPBS
chr2	239438129	239438130	MACS_peak_9342	23.00	lobChIP_FOXA1inPBS
chr2	239773106	239773107	MACS_peak_9343	19.00	lobChIP_FOXA1inPBS
chr2	240103791	240103792	MACS_peak_9344	8.00	lobChIP_FOXA1inPBS
chr2	240190086	240190087	MACS_peak_9345	10.00	lobChIP_FOXA1inPBS
chr2	240202836	240202837	MACS_peak_9346	14.00	lobChIP_FOXA1inPBS
chr2	240204311	240204312	MACS_peak_9347	35.00	lobChIP_FOXA1inPBS
chr2	240351179	240351180	MACS_peak_9348	8.00	lobChIP_FOXA1inPBS
chr2	240367221	240367222	MACS_peak_9349	27.00	lobChIP_FOXA1inPBS
chr2	240496543	240496544	MACS_peak_9350	18.00	lobChIP_FOXA1inPBS
chr2	240653308	240653309	MACS_peak_9351	10.00	lobChIP_FOXA1inPBS
chr2	240737711	240737712	MACS_peak_9352	16.00	lobChIP_FOXA1inPBS
chr2	241804129	241804130	MACS_peak_9353	10.00	lobChIP_FOXA1inPBS
chr2	241900211	241900212	MACS_peak_9354	28.00	lobChIP_FOXA1inPBS
chr2	242033076	242033077	MACS_peak_9355	90.00	lobChIP_FOXA1inPBS
chr2	242224808	242224809	MACS_peak_9356	40.00	lobChIP_FOXA1inPBS
chr2	242251057	242251058	MACS_peak_9357	6.00	lobChIP_FOXA1inPBS
chr2	242357247	242357248	MACS_peak_9358	12.00	lobChIP_FOXA1inPBS
chr2	242372403	242372404	MACS_peak_9359	54.00	lobChIP_FOXA1inPBS
chr2	242467704	242467705	MACS_peak_9360	11.00	lobChIP_FOXA1inPBS
chr2	242490006	242490007	MACS_peak_9361	24.00	lobChIP_FOXA1inPBS
chr2	243023045	243023046	MACS_peak_9362	14.00	lobChIP_FOXA1inPBS
chr20	118562	118563	MACS_peak_9363	13.00	lobChIP_FOXA1inPBS
chr20	136778	136779	MACS_peak_9364	12.00	lobChIP_FOXA1inPBS
chr20	277929	277930	MACS_peak_9365	7.00	lobChIP_FOXA1inPBS
chr20	306106	306107	MACS_peak_9366	19.00	lobChIP_FOXA1inPBS
chr20	328694	328695	MACS_peak_9367	7.00	lobChIP_FOXA1inPBS
chr20	330072	330073	MACS_peak_9368	17.00	lobChIP_FOXA1inPBS
chr20	351124	351125	MACS_peak_9369	14.00	lobChIP_FOXA1inPBS
chr20	371031	371032	MACS_peak_9370	12.00	lobChIP_FOXA1inPBS
chr20	377715	377716	MACS_peak_9371	9.00	lobChIP_FOXA1inPBS
chr20	382449	382450	MACS_peak_9372	12.00	lobChIP_FOXA1inPBS
chr20	453360	453361	MACS_peak_9373	7.00	lobChIP_FOXA1inPBS
chr20	491353	491354	MACS_peak_9374	25.00	lobChIP_FOXA1inPBS
chr20	627240	627241	MACS_peak_9375	12.00	lobChIP_FOXA1inPBS
chr20	648264	648265	MACS_peak_9376	17.00	lobChIP_FOXA1inPBS
chr20	1103372	1103373	MACS_peak_9377	18.00	lobChIP_FOXA1inPBS
chr20	1364887	1364888	MACS_peak_9378	14.00	lobChIP_FOXA1inPBS
chr20	1373952	1373953	MACS_peak_9379	13.00	lobChIP_FOXA1inPBS
chr20	1418211	1418212	MACS_peak_9380	18.00	lobChIP_FOXA1inPBS
chr20	1617529	1617530	MACS_peak_9381	19.00	lobChIP_FOXA1inPBS
chr20	1808643	1808644	MACS_peak_9382	19.00	lobChIP_FOXA1inPBS
chr20	1835958	1835959	MACS_peak_9383	10.00	lobChIP_FOXA1inPBS
chr20	1986642	1986643	MACS_peak_9384	17.00	lobChIP_FOXA1inPBS
chr20	2047344	2047345	MACS_peak_9385	55.00	lobChIP_FOXA1inPBS
chr20	2084414	2084415	MACS_peak_9386	11.00	lobChIP_FOXA1inPBS
chr20	2241954	2241955	MACS_peak_9387	16.00	lobChIP_FOXA1inPBS
chr20	2245837	2245838	MACS_peak_9388	35.00	lobChIP_FOXA1inPBS
chr20	2256867	2256868	MACS_peak_9389	32.00	lobChIP_FOXA1inPBS
chr20	2286761	2286762	MACS_peak_9390	17.00	lobChIP_FOXA1inPBS
chr20	2456814	2456815	MACS_peak_9391	15.00	lobChIP_FOXA1inPBS
chr20	2732101	2732102	MACS_peak_9392	28.00	lobChIP_FOXA1inPBS
chr20	2928299	2928300	MACS_peak_9393	23.00	lobChIP_FOXA1inPBS
chr20	2963153	2963154	MACS_peak_9394	17.00	lobChIP_FOXA1inPBS
chr20	3023019	3023020	MACS_peak_9395	10.00	lobChIP_FOXA1inPBS
chr20	3084561	3084562	MACS_peak_9396	15.00	lobChIP_FOXA1inPBS
chr20	3203709	3203710	MACS_peak_9397	57.00	lobChIP_FOXA1inPBS
chr20	3226007	3226008	MACS_peak_9398	9.00	lobChIP_FOXA1inPBS
chr20	3291429	3291430	MACS_peak_9399	8.00	lobChIP_FOXA1inPBS
chr20	3386822	3386823	MACS_peak_9400	13.00	lobChIP_FOXA1inPBS
chr20	3458636	3458637	MACS_peak_9401	11.00	lobChIP_FOXA1inPBS
chr20	3473145	3473146	MACS_peak_9402	9.00	lobChIP_FOXA1inPBS
chr20	3522617	3522618	MACS_peak_9403	20.00	lobChIP_FOXA1inPBS
chr20	3528776	3528777	MACS_peak_9404	16.00	lobChIP_FOXA1inPBS
chr20	3534609	3534610	MACS_peak_9405	26.00	lobChIP_FOXA1inPBS
chr20	3613372	3613373	MACS_peak_9406	31.00	lobChIP_FOXA1inPBS
chr20	3719232	3719233	MACS_peak_9407	18.00	lobChIP_FOXA1inPBS
chr20	3776516	3776517	MACS_peak_9408	12.00	lobChIP_FOXA1inPBS
chr20	3778857	3778858	MACS_peak_9409	17.00	lobChIP_FOXA1inPBS
chr20	3799187	3799188	MACS_peak_9410	16.00	lobChIP_FOXA1inPBS
chr20	3874314	3874315	MACS_peak_9411	12.00	lobChIP_FOXA1inPBS
chr20	3885259	3885260	MACS_peak_9412	9.00	lobChIP_FOXA1inPBS
chr20	3907645	3907646	MACS_peak_9413	7.00	lobChIP_FOXA1inPBS
chr20	3998496	3998497	MACS_peak_9414	10.00	lobChIP_FOXA1inPBS
chr20	4116058	4116059	MACS_peak_9415	7.00	lobChIP_FOXA1inPBS
chr20	4152449	4152450	MACS_peak_9416	8.00	lobChIP_FOXA1inPBS
chr20	4203140	4203141	MACS_peak_9417	32.00	lobChIP_FOXA1inPBS
chr20	4230752	4230753	MACS_peak_9418	7.00	lobChIP_FOXA1inPBS
chr20	4605737	4605738	MACS_peak_9419	12.00	lobChIP_FOXA1inPBS
chr20	4672742	4672743	MACS_peak_9420	25.00	lobChIP_FOXA1inPBS
chr20	4886510	4886511	MACS_peak_9421	38.00	lobChIP_FOXA1inPBS
chr20	4918402	4918403	MACS_peak_9422	17.00	lobChIP_FOXA1inPBS
chr20	4942648	4942649	MACS_peak_9423	12.00	lobChIP_FOXA1inPBS
chr20	4948068	4948069	MACS_peak_9424	75.00	lobChIP_FOXA1inPBS
chr20	4975922	4975923	MACS_peak_9425	11.00	lobChIP_FOXA1inPBS
chr20	4991067	4991068	MACS_peak_9426	14.00	lobChIP_FOXA1inPBS
chr20	5009574	5009575	MACS_peak_9427	16.00	lobChIP_FOXA1inPBS
chr20	5133737	5133738	MACS_peak_9428	24.00	lobChIP_FOXA1inPBS
chr20	5153737	5153738	MACS_peak_9429	8.00	lobChIP_FOXA1inPBS
chr20	5183333	5183334	MACS_peak_9430	9.00	lobChIP_FOXA1inPBS
chr20	5485915	5485916	MACS_peak_9431	7.00	lobChIP_FOXA1inPBS
chr20	5495044	5495045	MACS_peak_9432	41.00	lobChIP_FOXA1inPBS
chr20	5588959	5588960	MACS_peak_9433	8.00	lobChIP_FOXA1inPBS
chr20	5635365	5635366	MACS_peak_9434	11.00	lobChIP_FOXA1inPBS
chr20	5705395	5705396	MACS_peak_9435	30.00	lobChIP_FOXA1inPBS
chr20	5711020	5711021	MACS_peak_9436	11.00	lobChIP_FOXA1inPBS
chr20	5730895	5730896	MACS_peak_9437	12.00	lobChIP_FOXA1inPBS
chr20	5760371	5760372	MACS_peak_9438	48.00	lobChIP_FOXA1inPBS
chr20	5763295	5763296	MACS_peak_9439	50.00	lobChIP_FOXA1inPBS
chr20	5785099	5785100	MACS_peak_9440	55.00	lobChIP_FOXA1inPBS
chr20	5812390	5812391	MACS_peak_9441	22.00	lobChIP_FOXA1inPBS
chr20	5825180	5825181	MACS_peak_9442	17.00	lobChIP_FOXA1inPBS
chr20	5988424	5988425	MACS_peak_9443	41.00	lobChIP_FOXA1inPBS
chr20	6039396	6039397	MACS_peak_9444	10.00	lobChIP_FOXA1inPBS
chr20	6076334	6076335	MACS_peak_9445	9.00	lobChIP_FOXA1inPBS
chr20	6104338	6104339	MACS_peak_9446	13.00	lobChIP_FOXA1inPBS
chr20	6447301	6447302	MACS_peak_9447	17.00	lobChIP_FOXA1inPBS
chr20	6470280	6470281	MACS_peak_9448	11.00	lobChIP_FOXA1inPBS
chr20	6646713	6646714	MACS_peak_9449	10.00	lobChIP_FOXA1inPBS
chr20	6684976	6684977	MACS_peak_9450	12.00	lobChIP_FOXA1inPBS
chr20	7094666	7094667	MACS_peak_9451	13.00	lobChIP_FOXA1inPBS
chr20	7128017	7128018	MACS_peak_9452	17.00	lobChIP_FOXA1inPBS
chr20	7565821	7565822	MACS_peak_9453	19.00	lobChIP_FOXA1inPBS
chr20	8075626	8075627	MACS_peak_9454	14.00	lobChIP_FOXA1inPBS
chr20	8110485	8110486	MACS_peak_9455	15.00	lobChIP_FOXA1inPBS
chr20	8125470	8125471	MACS_peak_9456	44.00	lobChIP_FOXA1inPBS
chr20	8136147	8136148	MACS_peak_9457	13.00	lobChIP_FOXA1inPBS
chr20	8137446	8137447	MACS_peak_9458	43.00	lobChIP_FOXA1inPBS
chr20	8186795	8186796	MACS_peak_9459	22.00	lobChIP_FOXA1inPBS
chr20	8189884	8189885	MACS_peak_9460	9.00	lobChIP_FOXA1inPBS
chr20	8206493	8206494	MACS_peak_9461	12.00	lobChIP_FOXA1inPBS
chr20	8231385	8231386	MACS_peak_9462	45.00	lobChIP_FOXA1inPBS
chr20	8236758	8236759	MACS_peak_9463	16.00	lobChIP_FOXA1inPBS
chr20	8250694	8250695	MACS_peak_9464	8.00	lobChIP_FOXA1inPBS
chr20	8311741	8311742	MACS_peak_9465	50.00	lobChIP_FOXA1inPBS
chr20	8312964	8312965	MACS_peak_9466	9.00	lobChIP_FOXA1inPBS
chr20	8345338	8345339	MACS_peak_9467	9.00	lobChIP_FOXA1inPBS
chr20	8347205	8347206	MACS_peak_9468	16.00	lobChIP_FOXA1inPBS
chr20	8445250	8445251	MACS_peak_9469	12.00	lobChIP_FOXA1inPBS
chr20	8472900	8472901	MACS_peak_9470	10.00	lobChIP_FOXA1inPBS
chr20	8500019	8500020	MACS_peak_9471	11.00	lobChIP_FOXA1inPBS
chr20	8771235	8771236	MACS_peak_9472	13.00	lobChIP_FOXA1inPBS
chr20	8824890	8824891	MACS_peak_9473	9.00	lobChIP_FOXA1inPBS
chr20	9266450	9266451	MACS_peak_9474	15.00	lobChIP_FOXA1inPBS
chr20	9282590	9282591	MACS_peak_9475	22.00	lobChIP_FOXA1inPBS
chr20	10166985	10166986	MACS_peak_9476	16.00	lobChIP_FOXA1inPBS
chr20	10231624	10231625	MACS_peak_9477	13.00	lobChIP_FOXA1inPBS
chr20	10244529	10244530	MACS_peak_9478	46.00	lobChIP_FOXA1inPBS
chr20	10329426	10329427	MACS_peak_9479	7.00	lobChIP_FOXA1inPBS
chr20	10348687	10348688	MACS_peak_9480	22.00	lobChIP_FOXA1inPBS
chr20	10351102	10351103	MACS_peak_9481	34.00	lobChIP_FOXA1inPBS
chr20	10444362	10444363	MACS_peak_9482	15.00	lobChIP_FOXA1inPBS
chr20	10446005	10446006	MACS_peak_9483	11.00	lobChIP_FOXA1inPBS
chr20	10492812	10492813	MACS_peak_9484	10.00	lobChIP_FOXA1inPBS
chr20	10521468	10521469	MACS_peak_9485	10.00	lobChIP_FOXA1inPBS
chr20	10522865	10522866	MACS_peak_9486	14.00	lobChIP_FOXA1inPBS
chr20	10547087	10547088	MACS_peak_9487	20.00	lobChIP_FOXA1inPBS
chr20	10577663	10577664	MACS_peak_9488	19.00	lobChIP_FOXA1inPBS
chr20	10579712	10579713	MACS_peak_9489	19.00	lobChIP_FOXA1inPBS
chr20	10585419	10585420	MACS_peak_9490	23.00	lobChIP_FOXA1inPBS
chr20	10635387	10635388	MACS_peak_9491	12.00	lobChIP_FOXA1inPBS
chr20	10686749	10686750	MACS_peak_9492	14.00	lobChIP_FOXA1inPBS
chr20	10689587	10689588	MACS_peak_9493	30.00	lobChIP_FOXA1inPBS
chr20	10691884	10691885	MACS_peak_9494	32.00	lobChIP_FOXA1inPBS
chr20	10695887	10695888	MACS_peak_9495	29.00	lobChIP_FOXA1inPBS
chr20	10697717	10697718	MACS_peak_9496	11.00	lobChIP_FOXA1inPBS
chr20	10781187	10781188	MACS_peak_9497	12.00	lobChIP_FOXA1inPBS
chr20	10829918	10829919	MACS_peak_9498	11.00	lobChIP_FOXA1inPBS
chr20	10837115	10837116	MACS_peak_9499	18.00	lobChIP_FOXA1inPBS
chr20	10930762	10930763	MACS_peak_9500	13.00	lobChIP_FOXA1inPBS
chr20	11963406	11963407	MACS_peak_9501	14.00	lobChIP_FOXA1inPBS
chr20	12895712	12895713	MACS_peak_9502	11.00	lobChIP_FOXA1inPBS
chr20	12909722	12909723	MACS_peak_9503	9.00	lobChIP_FOXA1inPBS
chr20	12950067	12950068	MACS_peak_9504	12.00	lobChIP_FOXA1inPBS
chr20	12958715	12958716	MACS_peak_9505	45.00	lobChIP_FOXA1inPBS
chr20	12959840	12959841	MACS_peak_9506	44.00	lobChIP_FOXA1inPBS
chr20	12979975	12979976	MACS_peak_9507	15.00	lobChIP_FOXA1inPBS
chr20	13039779	13039780	MACS_peak_9508	39.00	lobChIP_FOXA1inPBS
chr20	13070815	13070816	MACS_peak_9509	21.00	lobChIP_FOXA1inPBS
chr20	13102728	13102729	MACS_peak_9510	15.00	lobChIP_FOXA1inPBS
chr20	13224624	13224625	MACS_peak_9511	116.00	lobChIP_FOXA1inPBS
chr20	13303423	13303424	MACS_peak_9512	16.00	lobChIP_FOXA1inPBS
chr20	13319974	13319975	MACS_peak_9513	10.00	lobChIP_FOXA1inPBS
chr20	13399259	13399260	MACS_peak_9514	18.00	lobChIP_FOXA1inPBS
chr20	13404746	13404747	MACS_peak_9515	62.00	lobChIP_FOXA1inPBS
chr20	13433440	13433441	MACS_peak_9516	14.00	lobChIP_FOXA1inPBS
chr20	13527320	13527321	MACS_peak_9517	19.00	lobChIP_FOXA1inPBS
chr20	13662459	13662460	MACS_peak_9518	8.00	lobChIP_FOXA1inPBS
chr20	13766047	13766048	MACS_peak_9519	9.00	lobChIP_FOXA1inPBS
chr20	13769311	13769312	MACS_peak_9520	24.00	lobChIP_FOXA1inPBS
chr20	13896026	13896027	MACS_peak_9521	15.00	lobChIP_FOXA1inPBS
chr20	14859871	14859872	MACS_peak_9522	30.00	lobChIP_FOXA1inPBS
chr20	14960348	14960349	MACS_peak_9523	32.00	lobChIP_FOXA1inPBS
chr20	14965947	14965948	MACS_peak_9524	42.00	lobChIP_FOXA1inPBS
chr20	14966821	14966822	MACS_peak_9525	19.00	lobChIP_FOXA1inPBS
chr20	14971848	14971849	MACS_peak_9526	40.00	lobChIP_FOXA1inPBS
chr20	15041408	15041409	MACS_peak_9527	24.00	lobChIP_FOXA1inPBS
chr20	15042861	15042862	MACS_peak_9528	41.00	lobChIP_FOXA1inPBS
chr20	15635004	15635005	MACS_peak_9529	16.00	lobChIP_FOXA1inPBS
chr20	16046106	16046107	MACS_peak_9530	9.00	lobChIP_FOXA1inPBS
chr20	16081587	16081588	MACS_peak_9531	16.00	lobChIP_FOXA1inPBS
chr20	16274453	16274454	MACS_peak_9532	8.00	lobChIP_FOXA1inPBS
chr20	16284174	16284175	MACS_peak_9533	20.00	lobChIP_FOXA1inPBS
chr20	16349525	16349526	MACS_peak_9534	22.00	lobChIP_FOXA1inPBS
chr20	16388574	16388575	MACS_peak_9535	12.00	lobChIP_FOXA1inPBS
chr20	16452780	16452781	MACS_peak_9536	7.00	lobChIP_FOXA1inPBS
chr20	16456163	16456164	MACS_peak_9537	51.00	lobChIP_FOXA1inPBS
chr20	16460544	16460545	MACS_peak_9538	11.00	lobChIP_FOXA1inPBS
chr20	16508956	16508957	MACS_peak_9539	13.00	lobChIP_FOXA1inPBS
chr20	16615445	16615446	MACS_peak_9540	13.00	lobChIP_FOXA1inPBS
chr20	16742121	16742122	MACS_peak_9541	10.00	lobChIP_FOXA1inPBS
chr20	17123450	17123451	MACS_peak_9542	18.00	lobChIP_FOXA1inPBS
chr20	17424828	17424829	MACS_peak_9543	12.00	lobChIP_FOXA1inPBS
chr20	17512084	17512085	MACS_peak_9544	21.00	lobChIP_FOXA1inPBS
chr20	17586070	17586071	MACS_peak_9545	19.00	lobChIP_FOXA1inPBS
chr20	17657862	17657863	MACS_peak_9546	9.00	lobChIP_FOXA1inPBS
chr20	17659099	17659100	MACS_peak_9547	9.00	lobChIP_FOXA1inPBS
chr20	17665077	17665078	MACS_peak_9548	36.00	lobChIP_FOXA1inPBS
chr20	17689552	17689553	MACS_peak_9549	23.00	lobChIP_FOXA1inPBS
chr20	17702886	17702887	MACS_peak_9550	125.00	lobChIP_FOXA1inPBS
chr20	17752322	17752323	MACS_peak_9551	11.00	lobChIP_FOXA1inPBS
chr20	17836893	17836894	MACS_peak_9552	12.00	lobChIP_FOXA1inPBS
chr20	17839952	17839953	MACS_peak_9553	31.00	lobChIP_FOXA1inPBS
chr20	17873005	17873006	MACS_peak_9554	41.00	lobChIP_FOXA1inPBS
chr20	17877579	17877580	MACS_peak_9555	26.00	lobChIP_FOXA1inPBS
chr20	17899093	17899094	MACS_peak_9556	35.00	lobChIP_FOXA1inPBS
chr20	17938211	17938212	MACS_peak_9557	28.00	lobChIP_FOXA1inPBS
chr20	17939406	17939407	MACS_peak_9558	11.00	lobChIP_FOXA1inPBS
chr20	17948128	17948129	MACS_peak_9559	12.00	lobChIP_FOXA1inPBS
chr20	17966497	17966498	MACS_peak_9560	51.00	lobChIP_FOXA1inPBS
chr20	18010827	18010828	MACS_peak_9561	12.00	lobChIP_FOXA1inPBS
chr20	18084337	18084338	MACS_peak_9562	21.00	lobChIP_FOXA1inPBS
chr20	18119714	18119715	MACS_peak_9563	13.00	lobChIP_FOXA1inPBS
chr20	18125176	18125177	MACS_peak_9564	16.00	lobChIP_FOXA1inPBS
chr20	18554431	18554432	MACS_peak_9565	13.00	lobChIP_FOXA1inPBS
chr20	18601210	18601211	MACS_peak_9566	5.00	lobChIP_FOXA1inPBS
chr20	18629813	18629814	MACS_peak_9567	13.00	lobChIP_FOXA1inPBS
chr20	18964768	18964769	MACS_peak_9568	10.00	lobChIP_FOXA1inPBS
chr20	19443812	19443813	MACS_peak_9569	7.00	lobChIP_FOXA1inPBS
chr20	19590610	19590611	MACS_peak_9570	15.00	lobChIP_FOXA1inPBS
chr20	19601321	19601322	MACS_peak_9571	10.00	lobChIP_FOXA1inPBS
chr20	19765262	19765263	MACS_peak_9572	10.00	lobChIP_FOXA1inPBS
chr20	19797883	19797884	MACS_peak_9573	9.00	lobChIP_FOXA1inPBS
chr20	19865653	19865654	MACS_peak_9574	12.00	lobChIP_FOXA1inPBS
chr20	19884032	19884033	MACS_peak_9575	35.00	lobChIP_FOXA1inPBS
chr20	19899237	19899238	MACS_peak_9576	46.00	lobChIP_FOXA1inPBS
chr20	19901357	19901358	MACS_peak_9577	6.00	lobChIP_FOXA1inPBS
chr20	19945337	19945338	MACS_peak_9578	27.00	lobChIP_FOXA1inPBS
chr20	19947682	19947683	MACS_peak_9579	9.00	lobChIP_FOXA1inPBS
chr20	19957574	19957575	MACS_peak_9580	31.00	lobChIP_FOXA1inPBS
chr20	19965647	19965648	MACS_peak_9581	9.00	lobChIP_FOXA1inPBS
chr20	19982962	19982963	MACS_peak_9582	15.00	lobChIP_FOXA1inPBS
chr20	19991411	19991412	MACS_peak_9583	28.00	lobChIP_FOXA1inPBS
chr20	19996625	19996626	MACS_peak_9584	21.00	lobChIP_FOXA1inPBS
chr20	20177367	20177368	MACS_peak_9585	12.00	lobChIP_FOXA1inPBS
chr20	20529552	20529553	MACS_peak_9586	17.00	lobChIP_FOXA1inPBS
chr20	20554556	20554557	MACS_peak_9587	15.00	lobChIP_FOXA1inPBS
chr20	20592014	20592015	MACS_peak_9588	9.00	lobChIP_FOXA1inPBS
chr20	20679859	20679860	MACS_peak_9589	47.00	lobChIP_FOXA1inPBS
chr20	20724372	20724373	MACS_peak_9590	18.00	lobChIP_FOXA1inPBS
chr20	20901286	20901287	MACS_peak_9591	11.00	lobChIP_FOXA1inPBS
chr20	20922451	20922452	MACS_peak_9592	19.00	lobChIP_FOXA1inPBS
chr20	20952684	20952685	MACS_peak_9593	24.00	lobChIP_FOXA1inPBS
chr20	20960796	20960797	MACS_peak_9594	49.00	lobChIP_FOXA1inPBS
chr20	20998428	20998429	MACS_peak_9595	14.00	lobChIP_FOXA1inPBS
chr20	21000290	21000291	MACS_peak_9596	14.00	lobChIP_FOXA1inPBS
chr20	21002428	21002429	MACS_peak_9597	5.00	lobChIP_FOXA1inPBS
chr20	21033838	21033839	MACS_peak_9598	20.00	lobChIP_FOXA1inPBS
chr20	21058786	21058787	MACS_peak_9599	31.00	lobChIP_FOXA1inPBS
chr20	21066442	21066443	MACS_peak_9600	10.00	lobChIP_FOXA1inPBS
chr20	21115737	21115738	MACS_peak_9601	117.00	lobChIP_FOXA1inPBS
chr20	21216877	21216878	MACS_peak_9602	16.00	lobChIP_FOXA1inPBS
chr20	21239735	21239736	MACS_peak_9603	10.00	lobChIP_FOXA1inPBS
chr20	21261138	21261139	MACS_peak_9604	9.00	lobChIP_FOXA1inPBS
chr20	21284453	21284454	MACS_peak_9605	7.00	lobChIP_FOXA1inPBS
chr20	21317181	21317182	MACS_peak_9606	14.00	lobChIP_FOXA1inPBS
chr20	21340346	21340347	MACS_peak_9607	11.00	lobChIP_FOXA1inPBS
chr20	21510713	21510714	MACS_peak_9608	14.00	lobChIP_FOXA1inPBS
chr20	21962743	21962744	MACS_peak_9609	18.00	lobChIP_FOXA1inPBS
chr20	22230727	22230728	MACS_peak_9610	11.00	lobChIP_FOXA1inPBS
chr20	22327514	22327515	MACS_peak_9611	13.00	lobChIP_FOXA1inPBS
chr20	22384734	22384735	MACS_peak_9612	25.00	lobChIP_FOXA1inPBS
chr20	22411514	22411515	MACS_peak_9613	45.00	lobChIP_FOXA1inPBS
chr20	22433234	22433235	MACS_peak_9614	30.00	lobChIP_FOXA1inPBS
chr20	22446897	22446898	MACS_peak_9615	12.00	lobChIP_FOXA1inPBS
chr20	22451318	22451319	MACS_peak_9616	39.00	lobChIP_FOXA1inPBS
chr20	22471603	22471604	MACS_peak_9617	20.00	lobChIP_FOXA1inPBS
chr20	22472249	22472250	MACS_peak_9618	9.00	lobChIP_FOXA1inPBS
chr20	22488026	22488027	MACS_peak_9619	27.00	lobChIP_FOXA1inPBS
chr20	22490364	22490365	MACS_peak_9620	9.00	lobChIP_FOXA1inPBS
chr20	22493240	22493241	MACS_peak_9621	71.00	lobChIP_FOXA1inPBS
chr20	22504473	22504474	MACS_peak_9622	22.00	lobChIP_FOXA1inPBS
chr20	22505803	22505804	MACS_peak_9623	7.00	lobChIP_FOXA1inPBS
chr20	22551226	22551227	MACS_peak_9624	8.00	lobChIP_FOXA1inPBS
chr20	22559447	22559448	MACS_peak_9625	42.00	lobChIP_FOXA1inPBS
chr20	22566202	22566203	MACS_peak_9626	51.00	lobChIP_FOXA1inPBS
chr20	22661117	22661118	MACS_peak_9627	10.00	lobChIP_FOXA1inPBS
chr20	22832474	22832475	MACS_peak_9628	10.00	lobChIP_FOXA1inPBS
chr20	23168992	23168993	MACS_peak_9629	10.00	lobChIP_FOXA1inPBS
chr20	23213403	23213404	MACS_peak_9630	21.00	lobChIP_FOXA1inPBS
chr20	23249613	23249614	MACS_peak_9631	17.00	lobChIP_FOXA1inPBS
chr20	23251007	23251008	MACS_peak_9632	15.00	lobChIP_FOXA1inPBS
chr20	23267357	23267358	MACS_peak_9633	8.00	lobChIP_FOXA1inPBS
chr20	23339829	23339830	MACS_peak_9634	17.00	lobChIP_FOXA1inPBS
chr20	23349223	23349224	MACS_peak_9635	8.00	lobChIP_FOXA1inPBS
chr20	23367352	23367353	MACS_peak_9636	26.00	lobChIP_FOXA1inPBS
chr20	23400755	23400756	MACS_peak_9637	6.00	lobChIP_FOXA1inPBS
chr20	23417243	23417244	MACS_peak_9638	10.00	lobChIP_FOXA1inPBS
chr20	23610582	23610583	MACS_peak_9639	9.00	lobChIP_FOXA1inPBS
chr20	23639921	23639922	MACS_peak_9640	9.00	lobChIP_FOXA1inPBS
chr20	23642303	23642304	MACS_peak_9641	25.00	lobChIP_FOXA1inPBS
chr20	23976942	23976943	MACS_peak_9642	9.00	lobChIP_FOXA1inPBS
chr20	24015660	24015661	MACS_peak_9643	14.00	lobChIP_FOXA1inPBS
chr20	24736359	24736360	MACS_peak_9644	19.00	lobChIP_FOXA1inPBS
chr20	24968133	24968134	MACS_peak_9645	44.00	lobChIP_FOXA1inPBS
chr20	25036076	25036077	MACS_peak_9646	23.00	lobChIP_FOXA1inPBS
chr20	25096113	25096114	MACS_peak_9647	16.00	lobChIP_FOXA1inPBS
chr20	25154720	25154721	MACS_peak_9648	18.00	lobChIP_FOXA1inPBS
chr20	25160083	25160084	MACS_peak_9649	11.00	lobChIP_FOXA1inPBS
chr20	25208018	25208019	MACS_peak_9650	7.00	lobChIP_FOXA1inPBS
chr20	25214241	25214242	MACS_peak_9651	31.00	lobChIP_FOXA1inPBS
chr20	25319233	25319234	MACS_peak_9652	41.00	lobChIP_FOXA1inPBS
chr20	25359257	25359258	MACS_peak_9653	85.00	lobChIP_FOXA1inPBS
chr20	25488855	25488856	MACS_peak_9654	73.00	lobChIP_FOXA1inPBS
chr20	25519897	25519898	MACS_peak_9655	17.00	lobChIP_FOXA1inPBS
chr20	25523011	25523012	MACS_peak_9656	14.00	lobChIP_FOXA1inPBS
chr20	25552648	25552649	MACS_peak_9657	17.00	lobChIP_FOXA1inPBS
chr20	25560461	25560462	MACS_peak_9658	13.00	lobChIP_FOXA1inPBS
chr20	25565403	25565404	MACS_peak_9659	14.00	lobChIP_FOXA1inPBS
chr20	25583824	25583825	MACS_peak_9660	12.00	lobChIP_FOXA1inPBS
chr20	25700000	25700001	MACS_peak_9661	8.00	lobChIP_FOXA1inPBS
chr20	29521131	29521132	MACS_peak_9662	11.00	lobChIP_FOXA1inPBS
chr20	30162133	30162134	MACS_peak_9663	19.00	lobChIP_FOXA1inPBS
chr20	30196450	30196451	MACS_peak_9664	38.00	lobChIP_FOXA1inPBS
chr20	30252230	30252231	MACS_peak_9665	26.00	lobChIP_FOXA1inPBS
chr20	30284272	30284273	MACS_peak_9666	12.00	lobChIP_FOXA1inPBS
chr20	30292579	30292580	MACS_peak_9667	57.00	lobChIP_FOXA1inPBS
chr20	30294717	30294718	MACS_peak_9668	87.00	lobChIP_FOXA1inPBS
chr20	30299385	30299386	MACS_peak_9669	13.00	lobChIP_FOXA1inPBS
chr20	30300426	30300427	MACS_peak_9670	10.00	lobChIP_FOXA1inPBS
chr20	30307764	30307765	MACS_peak_9671	41.00	lobChIP_FOXA1inPBS
chr20	30334030	30334031	MACS_peak_9672	8.00	lobChIP_FOXA1inPBS
chr20	30467967	30467968	MACS_peak_9673	14.00	lobChIP_FOXA1inPBS
chr20	30844516	30844517	MACS_peak_9674	16.00	lobChIP_FOXA1inPBS
chr20	30851272	30851273	MACS_peak_9675	19.00	lobChIP_FOXA1inPBS
chr20	30877588	30877589	MACS_peak_9676	25.00	lobChIP_FOXA1inPBS
chr20	30913842	30913843	MACS_peak_9677	37.00	lobChIP_FOXA1inPBS
chr20	30918174	30918175	MACS_peak_9678	80.00	lobChIP_FOXA1inPBS
chr20	30930263	30930264	MACS_peak_9679	14.00	lobChIP_FOXA1inPBS
chr20	30943125	30943126	MACS_peak_9680	13.00	lobChIP_FOXA1inPBS
chr20	31031847	31031848	MACS_peak_9681	7.00	lobChIP_FOXA1inPBS
chr20	31061476	31061477	MACS_peak_9682	35.00	lobChIP_FOXA1inPBS
chr20	31073139	31073140	MACS_peak_9683	16.00	lobChIP_FOXA1inPBS
chr20	31131686	31131687	MACS_peak_9684	51.00	lobChIP_FOXA1inPBS
chr20	31133191	31133192	MACS_peak_9685	13.00	lobChIP_FOXA1inPBS
chr20	31272555	31272556	MACS_peak_9686	18.00	lobChIP_FOXA1inPBS
chr20	31273737	31273738	MACS_peak_9687	60.00	lobChIP_FOXA1inPBS
chr20	31297503	31297504	MACS_peak_9688	10.00	lobChIP_FOXA1inPBS
chr20	31346800	31346801	MACS_peak_9689	6.00	lobChIP_FOXA1inPBS
chr20	31411890	31411891	MACS_peak_9690	12.00	lobChIP_FOXA1inPBS
chr20	31595424	31595425	MACS_peak_9691	18.00	lobChIP_FOXA1inPBS
chr20	31597347	31597348	MACS_peak_9692	18.00	lobChIP_FOXA1inPBS
chr20	31694035	31694036	MACS_peak_9693	42.00	lobChIP_FOXA1inPBS
chr20	31715818	31715819	MACS_peak_9694	38.00	lobChIP_FOXA1inPBS
chr20	31763086	31763087	MACS_peak_9695	15.00	lobChIP_FOXA1inPBS
chr20	31766752	31766753	MACS_peak_9696	14.00	lobChIP_FOXA1inPBS
chr20	31961710	31961711	MACS_peak_9697	25.00	lobChIP_FOXA1inPBS
chr20	32058375	32058376	MACS_peak_9698	7.00	lobChIP_FOXA1inPBS
chr20	32070571	32070572	MACS_peak_9699	7.00	lobChIP_FOXA1inPBS
chr20	32125610	32125611	MACS_peak_9700	12.00	lobChIP_FOXA1inPBS
chr20	32127068	32127069	MACS_peak_9701	8.00	lobChIP_FOXA1inPBS
chr20	32143351	32143352	MACS_peak_9702	8.00	lobChIP_FOXA1inPBS
chr20	32144313	32144314	MACS_peak_9703	7.00	lobChIP_FOXA1inPBS
chr20	32146750	32146751	MACS_peak_9704	9.00	lobChIP_FOXA1inPBS
chr20	32150056	32150057	MACS_peak_9705	33.00	lobChIP_FOXA1inPBS
chr20	32151718	32151719	MACS_peak_9706	10.00	lobChIP_FOXA1inPBS
chr20	32277179	32277180	MACS_peak_9707	9.00	lobChIP_FOXA1inPBS
chr20	32309797	32309798	MACS_peak_9708	12.00	lobChIP_FOXA1inPBS
chr20	32401191	32401192	MACS_peak_9709	7.00	lobChIP_FOXA1inPBS
chr20	32414496	32414497	MACS_peak_9710	8.00	lobChIP_FOXA1inPBS
chr20	32497844	32497845	MACS_peak_9711	37.00	lobChIP_FOXA1inPBS
chr20	32585502	32585503	MACS_peak_9712	7.00	lobChIP_FOXA1inPBS
chr20	32591381	32591382	MACS_peak_9713	46.00	lobChIP_FOXA1inPBS
chr20	32592681	32592682	MACS_peak_9714	64.00	lobChIP_FOXA1inPBS
chr20	32603021	32603022	MACS_peak_9715	9.00	lobChIP_FOXA1inPBS
chr20	32723532	32723533	MACS_peak_9716	15.00	lobChIP_FOXA1inPBS
chr20	32769459	32769460	MACS_peak_9717	13.00	lobChIP_FOXA1inPBS
chr20	32842865	32842866	MACS_peak_9718	13.00	lobChIP_FOXA1inPBS
chr20	32888775	32888776	MACS_peak_9719	9.00	lobChIP_FOXA1inPBS
chr20	32893444	32893445	MACS_peak_9720	8.00	lobChIP_FOXA1inPBS
chr20	32898451	32898452	MACS_peak_9721	97.00	lobChIP_FOXA1inPBS
chr20	32919367	32919368	MACS_peak_9722	7.00	lobChIP_FOXA1inPBS
chr20	32931976	32931977	MACS_peak_9723	11.00	lobChIP_FOXA1inPBS
chr20	32978856	32978857	MACS_peak_9724	10.00	lobChIP_FOXA1inPBS
chr20	33110628	33110629	MACS_peak_9725	12.00	lobChIP_FOXA1inPBS
chr20	33113778	33113779	MACS_peak_9726	23.00	lobChIP_FOXA1inPBS
chr20	33209586	33209587	MACS_peak_9727	35.00	lobChIP_FOXA1inPBS
chr20	33212689	33212690	MACS_peak_9728	11.00	lobChIP_FOXA1inPBS
chr20	33215350	33215351	MACS_peak_9729	9.00	lobChIP_FOXA1inPBS
chr20	33225543	33225544	MACS_peak_9730	16.00	lobChIP_FOXA1inPBS
chr20	33289895	33289896	MACS_peak_9731	10.00	lobChIP_FOXA1inPBS
chr20	33291498	33291499	MACS_peak_9732	14.00	lobChIP_FOXA1inPBS
chr20	33295283	33295284	MACS_peak_9733	20.00	lobChIP_FOXA1inPBS
chr20	33305376	33305377	MACS_peak_9734	18.00	lobChIP_FOXA1inPBS
chr20	33461961	33461962	MACS_peak_9735	9.00	lobChIP_FOXA1inPBS
chr20	33590134	33590135	MACS_peak_9736	20.00	lobChIP_FOXA1inPBS
chr20	33717790	33717791	MACS_peak_9737	25.00	lobChIP_FOXA1inPBS
chr20	33749431	33749432	MACS_peak_9738	14.00	lobChIP_FOXA1inPBS
chr20	33796330	33796331	MACS_peak_9739	8.00	lobChIP_FOXA1inPBS
chr20	33844637	33844638	MACS_peak_9740	7.00	lobChIP_FOXA1inPBS
chr20	33915118	33915119	MACS_peak_9741	20.00	lobChIP_FOXA1inPBS
chr20	33957855	33957856	MACS_peak_9742	6.00	lobChIP_FOXA1inPBS
chr20	34055585	34055586	MACS_peak_9743	12.00	lobChIP_FOXA1inPBS
chr20	34056794	34056795	MACS_peak_9744	21.00	lobChIP_FOXA1inPBS
chr20	34071579	34071580	MACS_peak_9745	10.00	lobChIP_FOXA1inPBS
chr20	34216812	34216813	MACS_peak_9746	7.00	lobChIP_FOXA1inPBS
chr20	34224232	34224233	MACS_peak_9747	35.00	lobChIP_FOXA1inPBS
chr20	34328707	34328708	MACS_peak_9748	11.00	lobChIP_FOXA1inPBS
chr20	34332160	34332161	MACS_peak_9749	24.00	lobChIP_FOXA1inPBS
chr20	34394497	34394498	MACS_peak_9750	13.00	lobChIP_FOXA1inPBS
chr20	34442940	34442941	MACS_peak_9751	11.00	lobChIP_FOXA1inPBS
chr20	34634061	34634062	MACS_peak_9752	7.00	lobChIP_FOXA1inPBS
chr20	34658464	34658465	MACS_peak_9753	10.00	lobChIP_FOXA1inPBS
chr20	34672994	34672995	MACS_peak_9754	7.00	lobChIP_FOXA1inPBS
chr20	34846066	34846067	MACS_peak_9755	32.00	lobChIP_FOXA1inPBS
chr20	35251949	35251950	MACS_peak_9756	39.00	lobChIP_FOXA1inPBS
chr20	35271461	35271462	MACS_peak_9757	12.00	lobChIP_FOXA1inPBS
chr20	35582739	35582740	MACS_peak_9758	17.00	lobChIP_FOXA1inPBS
chr20	35583441	35583442	MACS_peak_9759	16.00	lobChIP_FOXA1inPBS
chr20	35724455	35724456	MACS_peak_9760	9.00	lobChIP_FOXA1inPBS
chr20	35748389	35748390	MACS_peak_9761	12.00	lobChIP_FOXA1inPBS
chr20	35912875	35912876	MACS_peak_9762	16.00	lobChIP_FOXA1inPBS
chr20	36001595	36001596	MACS_peak_9763	16.00	lobChIP_FOXA1inPBS
chr20	36008887	36008888	MACS_peak_9764	25.00	lobChIP_FOXA1inPBS
chr20	36352266	36352267	MACS_peak_9765	23.00	lobChIP_FOXA1inPBS
chr20	36514372	36514373	MACS_peak_9766	15.00	lobChIP_FOXA1inPBS
chr20	36574011	36574012	MACS_peak_9767	9.00	lobChIP_FOXA1inPBS
chr20	36615370	36615371	MACS_peak_9768	8.00	lobChIP_FOXA1inPBS
chr20	36652127	36652128	MACS_peak_9769	12.00	lobChIP_FOXA1inPBS
chr20	36688437	36688438	MACS_peak_9770	34.00	lobChIP_FOXA1inPBS
chr20	36694325	36694326	MACS_peak_9771	18.00	lobChIP_FOXA1inPBS
chr20	36703803	36703804	MACS_peak_9772	21.00	lobChIP_FOXA1inPBS
chr20	36711509	36711510	MACS_peak_9773	29.00	lobChIP_FOXA1inPBS
chr20	36753626	36753627	MACS_peak_9774	28.00	lobChIP_FOXA1inPBS
chr20	36919671	36919672	MACS_peak_9775	14.00	lobChIP_FOXA1inPBS
chr20	37077172	37077173	MACS_peak_9776	16.00	lobChIP_FOXA1inPBS
chr20	37130679	37130680	MACS_peak_9777	7.00	lobChIP_FOXA1inPBS
chr20	37233660	37233661	MACS_peak_9778	17.00	lobChIP_FOXA1inPBS
chr20	37296382	37296383	MACS_peak_9779	7.00	lobChIP_FOXA1inPBS
chr20	37542471	37542472	MACS_peak_9780	37.00	lobChIP_FOXA1inPBS
chr20	37780981	37780982	MACS_peak_9781	16.00	lobChIP_FOXA1inPBS
chr20	38880187	38880188	MACS_peak_9782	19.00	lobChIP_FOXA1inPBS
chr20	39631916	39631917	MACS_peak_9783	62.00	lobChIP_FOXA1inPBS
chr20	39950492	39950493	MACS_peak_9784	20.00	lobChIP_FOXA1inPBS
chr20	40057162	40057163	MACS_peak_9785	11.00	lobChIP_FOXA1inPBS
chr20	40126008	40126009	MACS_peak_9786	45.00	lobChIP_FOXA1inPBS
chr20	40162351	40162352	MACS_peak_9787	31.00	lobChIP_FOXA1inPBS
chr20	40223263	40223264	MACS_peak_9788	16.00	lobChIP_FOXA1inPBS
chr20	40279306	40279307	MACS_peak_9789	24.00	lobChIP_FOXA1inPBS
chr20	40322740	40322741	MACS_peak_9790	19.00	lobChIP_FOXA1inPBS
chr20	41530549	41530550	MACS_peak_9791	8.00	lobChIP_FOXA1inPBS
chr20	42093053	42093054	MACS_peak_9792	20.00	lobChIP_FOXA1inPBS
chr20	42185527	42185528	MACS_peak_9793	27.00	lobChIP_FOXA1inPBS
chr20	42356801	42356802	MACS_peak_9794	15.00	lobChIP_FOXA1inPBS
chr20	42754009	42754010	MACS_peak_9795	9.00	lobChIP_FOXA1inPBS
chr20	42903319	42903320	MACS_peak_9796	14.00	lobChIP_FOXA1inPBS
chr20	42931542	42931543	MACS_peak_9797	19.00	lobChIP_FOXA1inPBS
chr20	42984368	42984369	MACS_peak_9798	8.00	lobChIP_FOXA1inPBS
chr20	42999498	42999499	MACS_peak_9799	36.00	lobChIP_FOXA1inPBS
chr20	43023273	43023274	MACS_peak_9800	118.00	lobChIP_FOXA1inPBS
chr20	43031036	43031037	MACS_peak_9801	6.00	lobChIP_FOXA1inPBS
chr20	43033640	43033641	MACS_peak_9802	8.00	lobChIP_FOXA1inPBS
chr20	43057276	43057277	MACS_peak_9803	43.00	lobChIP_FOXA1inPBS
chr20	43133355	43133356	MACS_peak_9804	13.00	lobChIP_FOXA1inPBS
chr20	43706494	43706495	MACS_peak_9805	10.00	lobChIP_FOXA1inPBS
chr20	43969285	43969286	MACS_peak_9806	11.00	lobChIP_FOXA1inPBS
chr20	43972230	43972231	MACS_peak_9807	29.00	lobChIP_FOXA1inPBS
chr20	43973563	43973564	MACS_peak_9808	7.00	lobChIP_FOXA1inPBS
chr20	43988119	43988120	MACS_peak_9809	13.00	lobChIP_FOXA1inPBS
chr20	44250502	44250503	MACS_peak_9810	10.00	lobChIP_FOXA1inPBS
chr20	44280579	44280580	MACS_peak_9811	8.00	lobChIP_FOXA1inPBS
chr20	44353929	44353930	MACS_peak_9812	10.00	lobChIP_FOXA1inPBS
chr20	44400022	44400023	MACS_peak_9813	24.00	lobChIP_FOXA1inPBS
chr20	44486211	44486212	MACS_peak_9814	9.00	lobChIP_FOXA1inPBS
chr20	44553406	44553407	MACS_peak_9815	9.00	lobChIP_FOXA1inPBS
chr20	44556192	44556193	MACS_peak_9816	15.00	lobChIP_FOXA1inPBS
chr20	44560953	44560954	MACS_peak_9817	12.00	lobChIP_FOXA1inPBS
chr20	44643849	44643850	MACS_peak_9818	22.00	lobChIP_FOXA1inPBS
chr20	44939933	44939934	MACS_peak_9819	10.00	lobChIP_FOXA1inPBS
chr20	44992678	44992679	MACS_peak_9820	20.00	lobChIP_FOXA1inPBS
chr20	45173413	45173414	MACS_peak_9821	16.00	lobChIP_FOXA1inPBS
chr20	45191334	45191335	MACS_peak_9822	34.00	lobChIP_FOXA1inPBS
chr20	45213414	45213415	MACS_peak_9823	20.00	lobChIP_FOXA1inPBS
[truncated: 5,789,233 more chars]
